# PacBio full-length transcriptome analysis provides new insights into transcription of chloroplast genomes

**DOI:** 10.1080/15476286.2023.2214435

**Published:** 2023-05-25

**Authors:** Jinsong Shi, Shuangyong Yan, Wenjing Li, Xiurong Yang, Zhongqiu Cui, Junling Li, Guangsheng Li, Yuejiao Li, Yanping Hu, Shan Gao

**Affiliations:** a College of Life Sciences, Nankai University, Tianjin, P.R.China; b National Clinical Research Center of Kidney Disease, Jinling Hospital, Nanjing University School of Medicine, Nanjing, China; c Tianjin Institute of Crop Research, Tianjin Academy of Agricultural Sciences, Tianjin, P.R.China; d Qinghai Provincial Key Laboratory of Qinghai-Tibet Plateau Biological Resources, Northwest Institute of Plateau Biology, Chinese Academy of Sciences, Xining, Qinghai, P.R.China; e Key Laboratory of Adaptation and Evolution of Plateau Biota, Northwest Institute of Plateau Biology, Chinese Academy of Sciences, Xining, Qinghai, P.R.China; f Institute of Plant Protection, Tianjin Academy of Agricultural Sciences, Tianjin, P.R.China

**Keywords:** TIS, TTS, AT-rich region, polyA-like site, *Arabidopsis thaliana*

## Abstract

Chloroplast and mitochondrial DNA (cpDNA and mtDNA) are apart from nuclear DNA (nuDNA) in a eukaryotic cell. The transcription system of chloroplasts differs from those of mitochondria and eukaryotes. In contrast to nuDNA and animal mtDNA, the transcription of cpDNA is still not well understood, primarily due to the unresolved identification of transcription initiation sites (TISs) and transcription termination sites (TTSs) on the genome scale. In the present study, we characterized the transcription of chloroplast (cp) genes with greater accuracy and comprehensive information using PacBio full-length transcriptome data from *Arabidopsis thaliana*. The major findings included the discovery of four types of artifacts, the validation and correction of cp gene annotations, the exact identification of TISs that start with G, and the discovery of polyA-like sites as TTSs. Notably, we proposed a new model to explain cp transcription initiation and termination at the whole-genome level. Four types of artifacts, degraded RNAs and splicing intermediates deserve the attention from researchers working with PacBio full-length transcriptome data, as these contaminant sequences can lead to incorrect downstream analysis. Cp transcription initiates at multiple promoters and terminates at polyA-like sites. Our study provides new insights into cp transcription and new clues to study the evolution of promoters, TISs, TTSs and polyA tails of eukaryotic genes.

## Background

Chloroplasts and mitochondria likely evolved from prokaryotes that once lived as independent organisms and retained their own DNAs – chloroplast and mitochondrial DNA (cpDNA and mtDNA), which are apart from the nuclear DNA (nuDNA) in a eukaryotic cell. Most cpDNA, 70–200 Kbp in size, encodes about 80 protein-coding, 4 rRNA and 30 tRNA genes, whereas animal mtDNA, typically about 16 Kbp in size, encodes 13 protein-coding, 2 rRNA and 22 tRNA genes. Although the sizes of cpDNA and mtDNA are far smaller than nuDNA, the transcription of chloroplast (cp) and mitochondrial (mt) genes at the whole-genome level had long been unclear. Later, the transcription of animal mt genes was characterized with greater accuracy and comprehensive information by the PacBio full-length transcriptome sequencing [1]. In addition, the relevant discoveries included the 3′ polyadenylation and possible 5′ m^7^G caps of rRNAs [2], the non-coding mitochondrial RNA 1 (ncMT1) [3], two long non-coding RNAs (lncRNAs) [4], the uninterrupted transcription of mtDNAs [4], and *etc*. Subsequently, all the transcription initiation sites (TISs), transcription termination sites (TTSs) and primary transcripts were identified in mammal mtDNAs and a new model was proposed to explain how the transcription of mammal mt genes initiates and terminates at the whole-genome level [5]. In comparison with studies on animal mt transcription, no significant progress has been made in the study of cp transcription at the whole-genome level, primarily due to the unresolved identification of TISs and TTSs on the genome scale.

Both chloroplasts and mitochondria possess simple transcription systems like prokaryotes, with their genes transcribed as polycistronic transcripts. However, chloroplasts share some features (e.g. RNA splicing) in their transcription with eukaryotes, which are absent in animal mitochondria. Both cpDNA and animal mtDNA are almost entirely transcribed. A primary transcript, initiated in the control region (CR), covers almost the entire H-strand of mammal mtDNA and terminates in the CR [5]. Unlike animal mtDNA, cpDNA does not contain CRs [5], indicating that there is no specific region responsible for the initiation or termination of cp transcription. Multiple TISs and TTSs spread over cpDNA [6], which accounts for the transcription of all cp genes and prevents impacts on the transcription of involved genes caused by structural variations (SVs), particularly the frequent large inversions [7]. However, the high diversity of cp TISs and TTSs, with only a few identified [8] makes it difficult to identify them on the genome scale.

There are several major obstacles to the assembly of primary cp transcripts, which are indispensable for the identification of cp TISs and TTSs. Multiple contaminant sequences, especially chloroplast-derived sequences in nuDNAs and mtDNAs, are the first major obstacle to the assembly of primary cp transcripts using only RNA-seq data based on next-generation sequencing (NGS). Therefore, the previous study [6] using RNA-seq data only confirmed the existing results but did not provide new insights into cp transcription. Another previous study [8] analysed the high diversity of 17 promoters in *Arabidopsis thaliana* cpDNA using 5′ rapid amplification of cDNA end (RACE) [8], but our later studies showed that more than half of the 17 promoters had resulted from degraded RNAs. Therefore, the quantity of cp TISs [6] and promoters [8] had been overestimated in the previous studies due to contaminant sequences, such as degraded RNAs. A cpDNA typically has a circular structure including a large single-copy region (LSC), a small single-copy region (SSC) and two inverted repeats (denoted as IR1 and IR2) separating the LSC and SSC. The structure of two IRs is another major obstacle that cannot be overcome using RNA-seq data alone. Furthermore, this structure even makes it difficult to assemble cpDNA or detect SVs using PacBio DNA-seq data. Due to this reason, we might have not discovered that large inversion is the most accepted mutation type of SVs in cpDNAs and its frequency is much higher than expected [7]. In the present study, we characterized the transcription of cp genes with greater accuracy and comprehensive information using PacBio full-length transcriptome data of *A. thaliana*. By improving the processing of PacBio cDNA-seq data, we aimed to accurately identify the TISs and TTSs on the genome scale, leading to explain how the transcription of cp genes initiates and terminates at the whole-genome level.

## Results

### Four types of artifacts in PacBio full-length transcriptome

**Figure 1.**
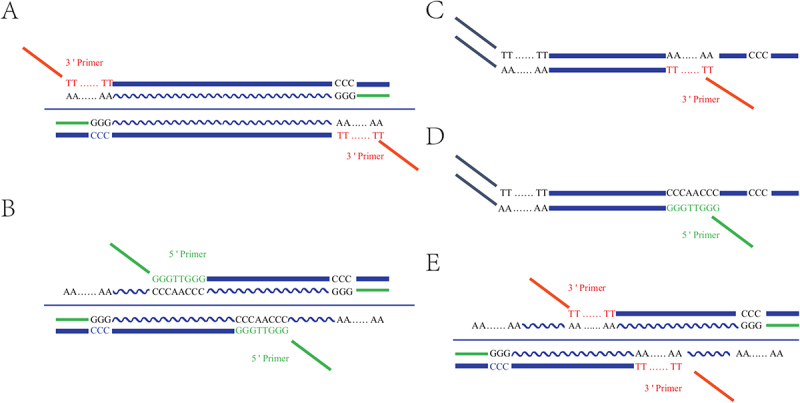
Four types of artifacts in PacBio full-length transcriptome

The PacBio cDNA-seq, Illumina RNA-seq and small RNA-seq (sRNA-seq) data of *A. thaliana* were obtained from the NCBI SRA database (**Methods and Materials**). The PacBio cDNA-seq data include a total of 19,579,559 circular consensus sequencing (CCS) reads, which were mapped to the reference cp genome (RefSeq: NC_000932) of *A. thaliana* with the length of 154,478 bp, resulting in 278,243 aligned CCS reads (**Supplementary file 1**). We defined the strand encoding more genes as the heavy strand and the other one as the light strand (the genome strand). By improving the processing of PacBio cDNA-seq data (**Methods and Materials**), 210,657 of 278,243 aligned CCS reads with 5‘, 3’, or both primers (Figure 1A) were processed into 210,657 cp transcripts. The remaining 24.3% (67,586/278,243) CCS reads were filtered out, including : (1) 20% (55,702/278,243) that contained 5‘ primers at both their 5’ and 3’ ends, defined as double-5 artifacts (Figure 1B) in the present study; (2) 2.67% (7,438/278,243) that contained 3’ primers with polyTs at both their 5‘ and 3’ ends, defined as double-3 artifacts (Figure 1C) in the present study; (3) 0.46% (1,271/278,243) that contained neither 5‘ nor 3’ primers; and (4) 1.14% (3,175/278,243) unclassified artifacts. By manual curation, we excluded that the double-5 and double-3 artifacts were PCR chimeras or artifacts resulting from self-ligation, which are made up of two transcripts. The detected double-5 and double-3 artifacts were made up of single transcripts with a length distribution similar to the 210,657 cp transcripts.

**A**. In the construction of full-length transcriptome library, a 3’ primer typically anneals to the polyA tail of a transcript (indicated by wave lines) and a 5’ primer anneals to the extended CCC of the first strand cDNA (indicated by solid lines). The transcripts may transcribe from the strand of the reference genome (below) or the antisense strand of the genome (above). **B**. if a 5‘ primer anneals to a 5’ primer-match site within a transcript, a double-5 artifact will be generated. **C**. if a 3‘ primer anneals to a 3’ primer-match site within the first strand cDNA, a double-3 artifact will be generated. **D**. if a 5‘ primer anneals to a 5’ primer-match site within the first strand cDNA, a 5’-truncated artifact will be generated. **E**. if a 3‘ primer anneals to a 3’ primer-match site within a transcript, a 3’-truncated artifact will be generated.

Further analysis revealed that the detected double-5 and double-3 artifacts were enriched in a few genomic regions. Most of the double-5 artifacts were enriched in three regions: 54695–56370, 106447–107483, and 131,166–132202 bp, while most of the double-3 artifacts were enriched in more than five regions: 2303–4543, 59387–62664, 81069–81931, 97395–99380, 139269–141315 bp, and *etc*. Notably, the first type of nucleotide (nt) polymers at both ends of the regions [(TTTGG, CCCAACC), (GGGGG, CCCACCCC), and (TTGGGG, CCCCC)] were highly similar to the 3‘ end of 5’ primer GGGTTGGG (Figure 1A) or its reverse complement. Likewise, the second type of nt polymers at both ends of the regions [(T_2_CT_9_, A_3_GA_2_TAGA_6_TA_3_TA_3_TA_6_), (T_11_CAT_4_, A3GA2TAGA6), (T_8_GTATCTCT_2_CT_4_, A_9_), (T_6_CT_5_, A_17_), (T_17_, A_3_CA_3_CGA_4_CGA_3_GA_4_GACGA_3_CAGA_3_) and *etc*, where the subscripts are copy numbers)] were highly similar to the 3‘ polyT of 3’ primer (Figure 1A) or its reverse complement. These two types of nt polymers in the genomes or transcripts were named as 5‘ and 3’ primer-match sites, respectively, in the present study. The only explanation for the generation of double-5 and double-3 artifacts during the process of library construction is that the 5‘ primers or the polyTs of 3’ primers annealed to the primer-match sites within the captured transcripts or their cDNAs, rather than 5‘ or 3’ ends of the transcripts. A double-5 artifact is generated because a cp transcript has a relatively short polyA tail at its 3’ end, causing the annealing of the short polyA tail to the polyT of 3‘ primer to occur less frequently than the annealing of a 5’ primer-match site to the 5’ primer (Figure 1B). A double-3 artifact is generated because of the similar principle (Figure 1C). Furthermore, 5’ primers or the polyTs of 3' primers annealed to primer-match sites in other ways, resulting in two other types of artifacts, which were shorter than the captured transcripts. The two other types of artifacts were named 5’- (Figure 1D) and 3’-truncated artifacts (Figure 1E) in the present study. The fifth type of artifacts, containing 3‘ primers with polyTs and 3’ primers without polyTs at their 5‘ and 3’ ends, respectively, was also detected (Supplementary file 2) but was not analysed further as only several artifacts of the fifth type were detected.

Double-3 artifacts were first reported in the transcripts of 16S rRNA in *Erthesina fullo* mtDNA without further analysis [2], while the other artifacts (double-5, 5’-, and 3’−truncated artifacts) had never been reported in any previous studies using full-length transcriptome sequencing, particularly in high proportions. Although double-5, double-3, 5’- and 3’-truncated artifacts may also be present in PacBio full-length transcriptomes or 5′ RACE PCR products from other organisms, they are unlikely to be as highly generated as they are in chloroplasts, because primer-match sites in cpDNAs are much more abundant than in DNA from other organisms. To improve the processing of PacBio cDNA-seq data (**Methods and Materials**), double-5, double-3, 5’-, and 3’-truncated artifacts need to be removed as contaminant sequences, because they can lead to incorrect downstream analysis, particularly the identification of TISs and TTSs. The worst case scenario is that a cp transcript could be misidentified as transcribed from its antisense strand. Double-5 and double-3 artifacts can be easily detected for removal, but 5’- and 3’-truncated artifacts cannot be easily detected, as they have the same structure as cp transcripts. Therefore, the 210,657 cp transcripts for the downstream analysis still contained 5’− and 3’-truncated artifacts. In addition, these transcripts also contained a large number of other contaminant sequences, particularly degraded RNAs and splicing intermediates (**Described as below**).

### Validation and correction of cp gene annotations

According to the NCBI RefSeq database, 78 and 51 genes are encoded on the two strands of *A. thaliana* cpDNA, respectively. In a cpDNA, the genes encoded in IR1 typically have copies in IR2. However, there are several exceptions in *A. thaliana* cpDNA. For instance, tRNA^His^_GUG_ has only one copy in *A. thaliana* cpDNA. Additionally, *rps*15 in IR2 does not have a copy in IR1, while *rps*19 in IR1 does not have a copy in IR2. In contrast, tRNA^His^_GUG_, *rps*15, and *rps*19 have two copies in *Onobrychis viciifolia* (sainfoin) and *Oryza sativa* (rice) cpDNAs. If 17 genes in IRs with two copies are counted as one, *A. thaliana* cpDNA encodes 78 protein-coding, 4 rRNA, and 30 tRNA genes. As the main result (**Supplementary file 2**), the annotations of most cp genes were validated at the transcription level using the PacBio cDNA-seq, RNA-seq and sRNA-seq data and those of several cp genes (e.g. tRNA^Ser^_GCU_, tRNA^Gly^_UCC_, *rpl*2 and *ycf*3) were corrected. As cp transcripts undergo RNA editing that change specific cytosines (Cs) to uracils (Us), RNA editing sites are used as genomic features to annotate the nt sequence. The previous study [6] identified 50 RNA editing sites in cp transcripts of *A. thaliana*. In the present study, 37 of the 50 editing sites were confirmed using the PacBio cDNA-seq data, while 13 of them were not detected or identified as SNPs of other types. By identifying nine new ones, a total of 46 RNA editing sites were annotated (**Supplementary file 3**).

**Figure 2.**
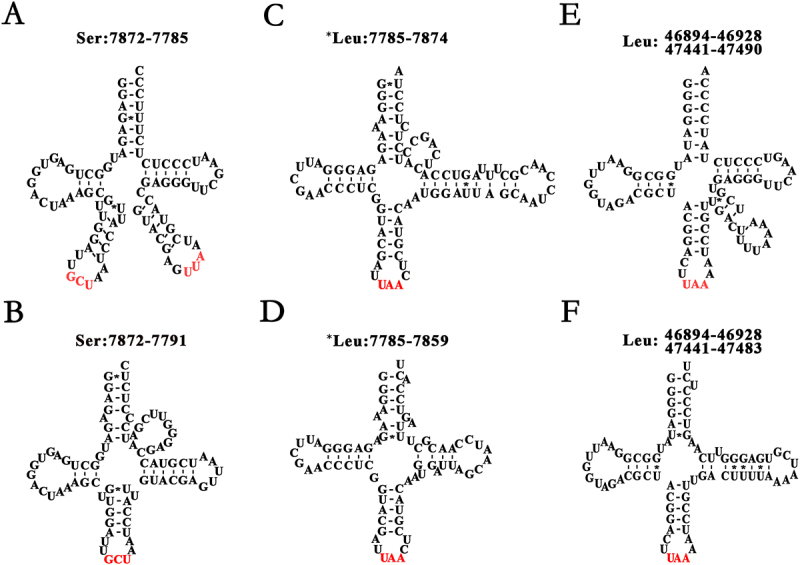
Secondary structures of long cp tRNAs.

Typically, tRNAs are annotated by prediction of their secondary structures or comparative sequence analysis, as they cannot be detected in their mature forms using PacBio cDNA-seq or RNA-seq data. In our previous study [3], we discovered 5‘ and 3’ small RNAs (sRNAs), which can be used to precisely annotate tRNAs. Using 5‘ and 3’ sRNAs, the annotations of 28 tRNAs in *A. thaliana* cpDNA were validated with a few modifications, while those of the other two (tRNA^Ser^_GCU_ and tRNA^Gly^_UCC_) were updated by correcting their strand information (Supplementary file 3). As an unexpected finding, tRNA^Ser^_GCU_ (Figure 2A) putatively encoded on the heavy strand had been incorrectly annotated as *tRNA^Leu^_UAA_ (Figure 2C) putatively encoded on the light strand (the genome strand) in the NCBI RefSeq database. This error is also present in *Menonvillea cuneata* (RefSeq: NC_065153), *Weberbauera imbricatifolia* (RefSeq: NC_065154), *etc*. Although tRNA^Ser^_GCU_ (NC_000932: 7785–7874) and *tRNA^Leu^_UAA_ (NC_000932: 7872–7785) can be predicted from two strands of the *A. thaliana* cpDNA, respectively, only tRNA^Ser^_GCU_ has been accepted for gene annotation. *tRNA^Leu^_UAA_ (Figure 2C,D) does not exist as a mature tRNA, which was validated using sRNA-seq data. In addition, we discovered that tRNA^Ser^_GCU_ forms an irregular secondary structure (Figure 2A), rather than a regular one (Figure 2B). The splicing sites of six multi-exon genes (tRNA^Lys^_UUU_, tRNA^Gly^_UCC_, tRNA^Leu^_UAA_, tRNA^Val^_UAC_, tRNA^Ile^_GAU_, and tRNA^Ala^_UGC_) were validated, and their intron boundaries were determined as GT-AT, GT-AC, TT-AA, GT-AC, TT-AC, and TT-GT, respectively. In a cpDNA, 30 tRNAs can be classified into two groups according to their lengths. The first group includes 22 tRNAs with lengths ranging from 71 to 75 nt, which form regular secondary structures, while the second group includes the other eight tRNAs with lengths greater than 80 nt, which are tRNA^Leu^_CAA_ (81 nt), tRNA^Leu^_UAA_ (85 nt), tRNA^Leu^_UAG_ (80 nt), tRNA^Ser^_GGA_ (87 nt), tRNA^Ser^_UGC_ (92 nt), tRNA^Ser^_GCU_ (88 nt), and tRNA^Tyr^_GUA_ (84 nt). Predictions showed that the second group could form irregular secondary structures in their full-lengths (Figure 2A–E), rather than regular secondary structures in their partial lengths (Figure 2B–F). Using 5‘ and 3’ sRNAs, it was determined that the second group of tRNAs form irregular secondary structures. Notably, tRNA^Ser^_GCU_ was found to contain two anticodon arms that are complementary to the codons of Ser and stop (Figure 2A), respectively. As cp tRNAs are extremely conserved in their nt sequences, the annotations of tRNAs in *A. thaliana* cpDNA can be used to correct annotations of cp genes in other species. For instance, the annotations of tRNA^Lys^_UUU_ and tRNA^Val^_UAC_ were used to correct those of their homologs in sainfoin and rice cpDNAs (**Supplementary file 2**).

The anticodons were indicated by red colours. Eight tRNAs in *Arabidopsis thaliana* cpDNA (RefSeq: NC_000932) have lengths more than 80 nt, and form irregular secondary structures. **A**. tRNA^Ser^_GCU_ (7872-7785) forms an irregular secondary structure and may contain two anticodon arms for Ser and stop codons, respectively. **B**. A regular secondary structure of tRNA^Ser^_GCU_ (NC_000932: 7872-7791) does not exist. *tRNA^Leu^_UAA_ (NC_000932: 7785-7874) can be predicted from the antisense strand of tRNA^Ser^_GCU_ (NC_000932: 7872-7785); however, the irregular secondary structure (**C**) or regular secondary structure (**D)** of *tRNA^Leu^_UAA_ (NC_000932: 7785-7859) does not exist. Another tRNA^Leu^_UAA_ forms an irregular secondary structure in (**E**), not a regular secondary structure in (**F)**.

**Figure 3.**
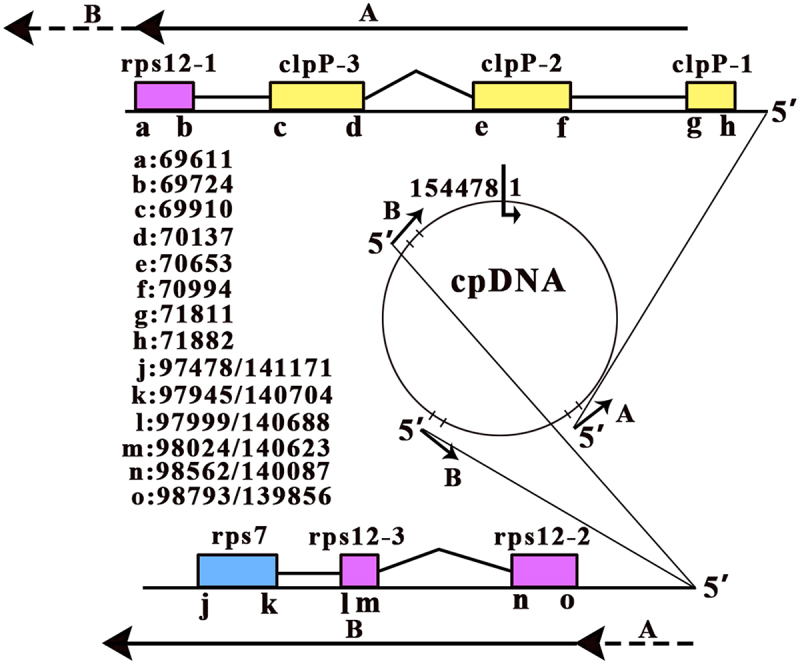
Trans-splicing of the gene rps12.

**Figure 4.**
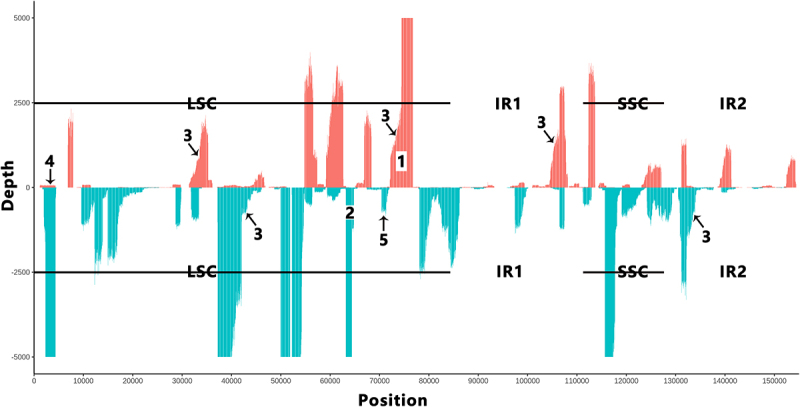
The gene expression profile of Arabidopsis thaliana cpDNA.

**Figure 5.**
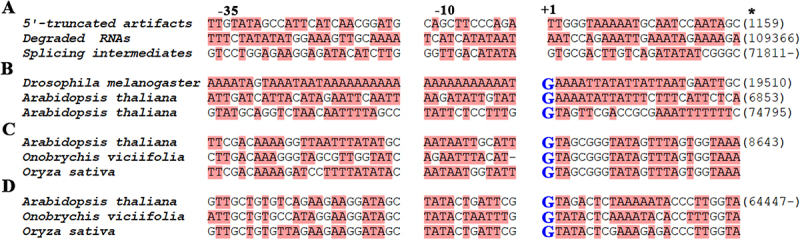
True-positive and false-positive cp promoters.

The 78 protein-coding genes include 76 common genes which are present in both *A. thaliana* and sainfoin cpDNAs, and the other two genes (*rpl*22 and *rps*16) which are present in both *A. thaliana* and rice cpDNAs, but absent in sainfoin cpDNA. Among the 78 protein-coding genes in *A. thaliana*, 12 multi-exon genes are *rpl*2, *rpl*16, *ndh*A, *ndh*B, *pet*B, *pet*D, *atp*F, *rpo*C1, and *rps*16 with two exons, and *clp*P, *rps1*2 and *ycf*3 with three exons. Among the 12 multi-exon genes, *rpo*C1 and *rps1*2 have one and two exons in sainfoin and rice cpDNAs, respectively, while *clp*P has two and one exons in sainfoin and rice cpDNAs, respectively. In the present study, the splicing sites of the 12 multi-exon genes were detected except for that of *rps*16. The annotation of *ycf*3 was corrected, whereas the annotations of 10 other multi-exon genes were validated (**Supplementary file 3**). According to the modified annotations of 78 protein-coding genes, most intron boundaries have the conserved dimers GT and AY (Y = C or T) at their 5‘ and 3’ ends, denoted as the GT-AY motif, while a few have other dimers (e.g. GT-AA and GT-CC). In addition, all the detected RNA splicing events were validated as canonical splicing events, except for those of *rps1*2. Exon 1 of *rps1*2 is located in SSC, while two copies of exons 2 and 3 are located in IR1 and IR2 (Figure 3). A previous study [9] showed that exon 1, 2 and 3 of *rps1*2 are transcribed on separate transcripts and trans-splicing occurs between exon 1 and 2. Our results confirmed these findings and exhibited that exon 1 and the other two exons are transcribed on two separate polycistronic transcripts, respectively (Figure 3). During the validation of gene annotations, we unexpectedly detected a new type of contaminant sequences, which were defined as splicing intermediates in the present study (Figure 4). A typical example was a few transcripts starting with the 5’ boundary GT of intron 1 of the gene *clp*P at the genomic position 71,811 (Figure 5A). The 5’ ends of these transcripts were almost identified as the false-positive TIS_71811-_ (the minus symbol indicates the heavy strand). Obviously, these transcripts had been captured as intermediates after the cleavage of the 5’ exons from their introns.

Polycistronic transcripts A+B (5‘→3’) were detected to start with the 5’ boundary GT of intron 1 of the gene *clp*P and covered h, g, f, ed, c, b, ao, nm, l, k, and j. The lowercase letters (a→o) represent the genomic positions; ed, ao, and nm indicate that intron 2 of the gene *clp*P and intron 1 and 2 of the gene *rps*12 were cleaved from the polycistronic transcripts A+B; *clp*P-1, -2, and -3 represent exon 1, 2, and 3 of the gene *clp*P, respectively; *rps*12-1, -2, and -3 represent exon 1, 2, and 3 of the gene *rps*12, respectively. The *Arabidopsis thaliana* cpDNA (RefSeq: NC_000932) clockwise starts at position 1 and ends at position 154478. Exon 1 of *rps*12 is 28 Kbp upstream of the nearest copy of exons 2 and 3 in IR1 and 70 Kbp away from the distal copy of exons 2 and 3 in IR2.

In total, 210,657 cp transcripts were mapped to *Arabidopsis thaliana* cpDNA (RefSeq: NC_000932) to generate the gene expression profile, which exhibited limited transcriptional units containing clustered genes along the genome. Although the double-5 and double-3 artifacts were removed as contaminant sequences, the cp transcripts still contained other types of contaminant sequences, mainly including 5’- and 3’-truncated artifacts, degraded RNAs and splicing intermediates. The cutoff of depth (Y-axis) was set to 5,000 for proper visualization. It can be seen that: (1) the highest-depth region (NC_000932: 72209-76746) of the light strand spans five genes (*psb*B, *psb*T, *psb*H, *pet*B and *pet*D); (2) the highest-depth region (NC_000932: 63165-64451) on the heavy strand spans four genes (*psb*J, *psb*L, *psb*F and *psa*E); (3) a large number of degraded RNAs are present; (4) 5’-truncated artifacts cover the region from 1159 to 4365 bp; (5) Splicing intermediates start at the position 71811 bp. LSC (NC_000932: 1-84170): large single-copy region; IR1 (NC_000932: 84171-110438): inverted repeat 1; SSC (NC_000932: 110439-128120): small single-copy region; IR2 (NC_000932: 128121-154478): inverted repeat 2.

*indicates the position in the genome and “-” indicates the antisense strand of the reference genome. The 62-nt segments were divided into three regions. including I (-37,-13), II (-12, -1), and III (+1, 25). **A**. A few 5’-truncated artifacts starting at the position of 1159 in *Arabidopsis thaliana* cpDNA (RefSeq: NC_000932) contained TTGGG at their 5’ ends; TIS_109366_ was reported in the previous study [8] but detected at 5‘ends of degraded RNAs in the present study; A few splicing intermediates started with the 5’ boundary GT of intron 1 of the gene *clp*P at the position of 71811. **B**. The transcription of *Drosophila melanogaster* mtDNA (RefSeq: NC_024511) also initiates in an AT-rich region. Almost all TISs start with G (in blue colour) at +1 positions with a few exceptions. Both the low-efficient promoter containing TIS_8643_ (**C**) and the high-efficient promoter containing TIS_64447-_ (**D**) are highly conserved among *A. thaliana*, *Onobrychis viciifolia* (GenBank: MW007721), and *Oryza sativa* (RefSeq: NC_001320) cpDNAs.

### *Gene expression profiling of* Arabidopsis *chloroplast*

The 210,657 cp transcripts covered approximately 82.1% and 85.9% of the light and heavy strands of *A. thaliana* cpDNA respectively, which were lower than the 94% estimated by strand-specific RNA-seq data in the previous study [6]. However, the coverage of the light and heavy strands by cp transcripts without contaminant sequences would have been lower than the current ratios, as the 210,657 cp transcripts still contained a large number of contaminant sequences. Although TISs spread over the whole genome, resulting in overlapping transcripts, the cp gene expression profile exhibited limited transcriptional units containing clustered genes in the genome (Figure 4). However, this profile did not accurately reflect the gene expression of several genomic regions (e.g. 23S rRNA), where double-5 and double-3 artifacts were removed (See above). Generally, the photosynthesis-related genes were expressed at higher levels than the other protein-coding genes. The transcriptional unit (NC_000932: 72209–76754) on the light strand spanned five genes (*psb*B, *psb*T, *psb*H, *pet*B and *pet*D), while the transcriptional unit (NC_000932: 63165–64451) on the heavy strand spanned four genes (*psb*J, *psb*L, *psb*F and *psa*E). These two transcriptional units, with average depths of 5,215 and 8,376, were the highest-expression regions on the light and heavy strands, respectively, while the light and heavy strands were covered by cp transcripts with average depths of 603 and 1,193, respectively. As the most important finding of the present study, almost all the transcriptional units contained A-rich sequences at their 3’ ends or T-rich sequences at their 5’ ends, which were named polyA-like sites (Supplementary file 2). For instance, the transcriptional unit (NC_000932: 72055–76754) contained A_12_GA_4_TA_2_ at its 3 end, and the transcriptional unit (NC_000932: 37184–42045) contained TAT_8_C_2_A_2_T_5_ at its 5’ end. The most common motifs AAUAA and AAGAA or their reverse complements in these polyA-like sites may be polyadenylation signals. Although polyA-like sites are similar to 3’ primer-match sites, it is impossible that all the detected cp transcripts terminating at polyA-like sites are 3’-truncated artifacts resulting from the annealing of polyA-like sites to the polyTs of 3‘ primers as 3’ primer-match sites (Figure 1e). Further analysis showed that a long cp transcript (not longer than 8,153 nt according to the present study) contains many polyA-like sites, while a short cp transcript (not longer than 300 nt) contains not more than one polyA-like site at it is 3’ end. Therefore, we concluded that polyA-like sites function as TTSs. However, the mechanism underlying the read-through of polyA-like sites in long cp transcripts is unknown. In the cp gene expression profile (Figure 4), the short transcripts and parts of the long transcripts formed the transcriptional units, while the remaining parts of the long transcripts filled the “untranscribed” regions with low depths. In the “untranscribed” regions, we detected a certain number of artifacts and degraded RNAs. For instance, in an “untranscribed” region (NC_000932: 154–6852) on the light strand, a few 5’-truncated artifacts from 1159 to 4365 bp contained TTGGG at their 5’ ends (Figure 5A).

Although we improved the processing of PacBio cDNA-seq data (**Methods and Materials**), the 210,657 cp transcripts still contained 5’− and 3’-truncated artifacts and a large number of other contaminant sequences, particularly degraded RNAs and splicing intermediates. Most of the full-length cp transcripts were found to be polycistronic, covering at least two genes. These polycistronic transcripts were composed of mature RNAs or RNA precursors (Figure 3). In general, eukaryotic polycistronic transcripts need to be processed into monocistronic transcripts for protein translation; therefore, monocistronic transcripts containing only single mature RNAs are supposed to be much more abundant than polycistronic transcripts containing RNA precursors, which was observed in mammal and insect mitochondria in our previous studies [2,5]. However, only a few cp transcripts were monocistronic transcripts and the cp RNA processing – how the RNA precursors are processed into mature RNAs remains unclear. As polycistronic transcripts containing only mature RNAs were much more abundant than polycistronic transcripts containing RNA precursors in *A. thaliana* chloroplasts, we proposed that polycistronic transcripts may serve for the translation of at least the first cp genes in them (Figure 3). The widespread distribution of TISs and TTSs accounts for the transcription and translation of all cp genes. Some other mechanisms (e.g. internal ribosome-binding sites) may also play a role. Notably, tRNA^Ser^_GCU_ may contain two anticodon arms for Ser and stop codons (**See above**). This could result in the co-translation of closely arranged genes (e.g. *psb*L, *psb*F and *psb*E) by the read-through of stop codons.

### TISs, TTSs and the transcription model

Based on early studies, all known cp promoters can be classified into three classes, including plastid-encoded plastid RNA polymerase (PEP) promoters for photosynthesis-related genes (class I); nuclear-encoded plastid RNA polymerase (NEP) promoters for few genes (class III); and other promoters of non-photosynthesis-related genes for both PEP and NEP (class II). Later, 17 TISs (**Supplementary file 2**) of 11 genes (*acc*D, *atp*B, *atp*I, *clp*P, *ycf*1, *psa*A, *psb*A, *rpo*B, *rps*4, *rps*15, and 16S rRNA) in *A. thaliana* cpDNA were identified belonging to at least four types (PEP, NEP type-Ia, type-Ib and type-II) of known cp promoters [8], confirming their high diversity. However, only seven of the 17 TISs (TIS_1522-_, TIS_26629-_, TIS_42045-_, TIS_45956-_, TIS_45979-_, TIS_100898_, and TIS_109372_, where the subscripts are +1 positions in promoters and the minus symbol indicates the heavy strand) were validated at low or very low expression levels by analysis of 210,657 cp transcripts, while the others [TIS_14999-_, TIS_54474-_, TIS_54678-_, TIS_71940-_, TIS_123701-_, TIS_56823_, TIS_56903_, TIS_100873_, TIS_109301_, and TIS_109366_ (Figure 5A)] were detected at 5’ ends of degraded RNAs.

In the present study, 181,017 of 210,657 cp transcripts and their CCS reads containing both 5‘ and 3’ primers (Supplementary file 1) were used to study TISs and promoters (TISs/promoters) in *A. thaliana* cpDNA. These sequences were equivalent to sequences from more than thousands of experiments using 5’ RACE. The 5’ ends of full-length cp transcripts were aligned to the cpDNA and the corresponding genomic positions were designated as +1 positions in promoters. Genomic segments spanning the positions from −37 to 25 were used to analyse TISs/promoters (Figure 5). As the 210,657 cp transcripts still contained a large number of other contaminant sequences, we were not able to identify all TISs/promoters in *A. thaliana* cpDNA. Fortunately, we found that the 62-nt segments derived from the alignments of degraded RNAs have lower copy numbers than those containing true-positive TISs/promoters. Then, we extracted the features of TISs/promoters by comparing the selected 62-nt segments with copy numbers of at least 10 to those with copy numbers of lower than 10. The preliminary results not only confirmed the high diversity of cp promoters [8] but also revealed commonalities in this diversity: (1) the true-positive promoters have higher AT-contents than contaminant sequences; (2) almost all TISs start with G at +1 positions with a few exceptions (e.g. TIS_9720_); (3) the transcription of cpDNA is initiated with some degree of randomness; (4) both high-efficient and clustered promoters contribute to high expression of cp genes.

For further analysis, the 62-nt segments were divided into three regions (Figure 5), I (−37,-13), II (−12, −1), and III (+1, 25) with AT-content of 65%, 80% and 65%, respectively. Region II of cp promoters in *A. thaliana* has the highest AT-content, which was consistent with a finding reported in our previous study of TISs in *Drosophila* mtDNAs [10]. Region II of mt promoters in *Drosophila melanogaster* has the AT-content of 100% (Figure 5B). These findings suggested transcription initiation of *A. thaliana* cpDNA and *D. melanogaster* mtDNA is dependent on the local DNA structure of an AT-rich region, rather than conserved sequences. The most common dimer type at +1 positions in promoters (Figure 5B) is GA, and the second most is GT. Among promoters starting with GT, a few with significantly lower AT-content were identified as splicing intermediates (Figure 5A), while the remaining may still include contaminant sequences of unknown types. Both high-efficient and clustered promoters contribute to high expression of cp genes. For example, the promoter containing TIS_51887-_ was identified as a high-efficient promoter with a depth of 9855, which was responsible for the high expression of tRNA^Val^_UAC_, *ndh*C and *ndh*K. Another example was that the three promoters containing TIS_74393-_, TIS_74442-_ and TIS_74795-_ (Figure 5B) clustering in the transcriptional unit (NC_000932: 72055–76754) were responsible for the high expression of *pet*B and *pet*D (Figure 4). Interestingly, the efficiency of cp promoters is not correlated with their conservation. For instance, both the low-efficient promoter containing TIS_8643_ (Figure 5C) and the high-efficient promoters containing TIS_64447-_ (Figure 5D) are highly conserved among *A. thaliana*, sainfoin and rice cpDNAs. TIS_6853_, TIS_8643_, and TIS_64447-_ (Figure 5) were validated using 5’ RNA ligase-mediated RACE (5’ RLM RACE) (**Methods and Materials**).

Finally, we proposed a new model to explain how cp transcription initiates and terminates at the whole-genome level. Cp transcription initiates at multiple promoters and terminates at polyA-like sites. The widespread distribution of TISs and TTSs accounts for the transcription of all cp genes and prevents impacts on the transcription of involved genes caused by SVs, particularly the frequent large inversions [7]. PolyA-like sites function as TTSs, but there is a very low possibility for the transcriptases to read through more than two polyA-like sites, so the median aligned length of cp transcripts is only 1,395 nt. The minimum requirement for a cp promoter is an AT-rich region containing several Gs which are the common first nucleotides for primary RNAs. The most important role of several Gs in promoters is to prevent impacts caused by mutation of G. GAA and TA can be used as elements to generate a simple promoter which does not require any specific sequence motif. Besides, the transcription of cpDNA is initiated with some degree of randomness, so not all primary RNAs start with G. For instance, primary RNAs from TIS_74793_, TIS_74794_ and TIS_74795_ (Figure 5B) start with T, G, and G, respectively. Nascent RNAs without G as the first nucleotide may miss the methylation modifications and undergo rapid degradation. This can explain that a large number of degraded RNAs were detected in cp transcripts. The eukaryotic promoters may have evolved from a site composed of A_n_ and T_n_ (n is the copy number). The polyA-like sites are also composed of A_n_ and T_n_ and their average lengths significantly decrease from *A. thaliana*, sainfoin, rice cpDNAs to *D. melanogaster* and human mtDNAs. A_12_ and T_12_ were detected to be present at 10 and 13 different positions in *A. thaliana* cpDNA, respectively, and 6 and 3 positions in sainfoin cpDNA, respectively. The rice cpDNA and human mtDNAs do not contain A_12_ and T_12_, whereas the *D. melanogaster* mtDNA contains one A_12_ and one T_12_, which are located in its CR – a special genomic region that is responsible for transcription regulation.

## Conclusions

In the present study, we characterized the transcription of cp genes with greater accuracy and comprehensive information using PacBio full-length transcriptome data of *A. thaliana*. During the data processing, we discovered five types of artifacts. Four types (double-5, double-3, 5’-, and 3’−truncated) of artifacts, degraded RNAs and splicing intermediates deserve the attention from researchers working with PacBio full-length transcriptome data, as these contaminant sequences can lead to incorrect downstream analysis. Other main results of the present study included the validation and correction of cp gene annotations, the exact identification of TISs that start with G, and the discovery of polyA-like sites as TTSs. Although we improved the processing of PacBio cDNA-seq data (**Methods and Materials**), the processed cp transcripts still contained a large number of contaminant sequences. Many 5' or 3' end RNA-seq methods can help to obtain the 5' or 3' ends of transcripts; however, none of them has been successfully applied. Even a widely accepted method cap analysis of gene expression (CAGE) produces a large number of reads that align to 3' rather than 5' ends of transcripts [11]. Thus, new protocols or technologies (e.g. using 5’ adapter) need to be designed to improve the full-length transcriptome sequencing.

Based on the identified TISs and TTSs, we proposed a new model to explain how cp transcription initiates and terminates at the whole-genome level. The simplicity of cp transcription system is out of our expectation. Cp transcription initiates at multiple promoters and terminates at polyA-like sites that function as TTSs. Only a few cp transcripts were found to be terminated at IRs which may also function as TTSs. However, a previous study [12] proposed that 3’ IRs act as mRNA processing and stabilizing elements but do not terminate the transcription. According to our model, polycistronic transcripts can serve for the protein translation. Besides, the primary RNAs contain G and polyA-like sequences at their 5’ and 3’ ends, respectively, resembling the mature RNAs. Therefore, our study provides new insights into cp transcription and new clues to study the evolution of promoters, TISs, TTSs and polyA tails of eukaryotic genes. Our results can be used to improve the design of promoters, TISs, and TTSs in synthesis biology.

The multiple TISs and TTSs in *A. thaliana* cpDNA resulted in a certain number of transcripts that do not cover complete ORFs, especially transcripts shorter than an ORF. For instance, transcripts from 55207–56548 or 55207–56565 bp are shorter than the ORF of the gene *rbc*L (NC_000932: 54958–56397). However, if these transcripts function in cells is still unknown. In addition, many detected transcripts (**Supplementary file 2**) are still unidentified. These transcripts do not belong to five types of artifacts and are unlikely to be degraded RNAs or splicing intermediates. For instance, transcripts from 31479 to 32243 bp on the light strand were detected in the non-coding regions, however, if they encode short proteins or peptides is unknown. In the ‘untranscribed’ regions, antisense transcripts from 44234 to 46196 bp on the light strand were detected, as predicted in the previous study [6], however, if they are long non-coding RNAs (lncRNAs) is unknown. Among the unidentified transcripts, there are some transcripts which do not start with G or terminate at polyA-like sites. For instance, transcripts from 9720 to 11499 bp on the light strand start with A (TIS_9720_) and transcripts from 16985 to 18062 bp on the light strand terminate at an IR CTTCCTCAGGAATG. Further analysis of these unidentified cp transcripts helps to reveal the mechanisms underlying cp transcription and its regulation at the whole-genome level.

## Materials and methods

The PacBio cDNA-seq (SRA: SRR17520069), Illumina RNA-seq (SRA: SRR17231442–50) and sRNA-seq data (SRA: SRR17231451–56) of *A. thaliana* were downloaded from the NCBI SRA database. SRR17520069, under the project accession number SRP354186, contains the CCS reads of PacBio full-length transcriptome data. SRR17231442–50 and SRR17231451–56, under the project accession number SRP350800, contain 50-bp and 75-bp single-end reads from the Illumina HiSeq 2500 sequencer, respectively. The reference genomes of *A. thaliana* cpDNA (RefSeq: NC_000932), *O. viciifolia* cpDNA (GenBank: MW007721), *O. sativa* cpDNA (RefSeq: NC_001320), *D. melanogaster* mtDNA (RefSeq: NC_024511), *E. fullo* mtDNA (GenBank: MK374364) and human mtDNA (RefSeq: NC_012920) were downloaded from the NCBI RefSeq or GenBank databases. The 5‘ and 3’ primers ACACTAGATCGCGTGTGCAATGAAGTCGCAGGGTTGGG and AAAAAAAAAAAAAAAAAAAAGTACTCTGCGTTTGATACCACTGCTTACACGCGATCTAGTGT were used in the construction of full-length transcriptome sequencing library in SRP354186 (Figure 1). 5’ RLM RACE was performed using FirstChoice RLM-RACE kits (USA, ThermoFisher), following the procedure provided by the manufacturer.

The cleaning and quality control of Illumina data were performed with the software Fastq_clean [13] v2.0. The alignment of Illumina and PacBio DNA-seq data was performed with the software BWA [14] v0.7.10. From the PacBio cDNA-seq data (SRA: SRR17520069), the 278,243 aligned CCS reads were obtained using the bwa mem algorithm and by filtering CCS reads, using two criteria: (1) CCS reads with aligned regions shorter than 200-bp need to be removed; (2) CCS reads with aligned regions covering <5% of their lengths need to be removed. The identification of double-5, double-3 artifacts, artifacts containing neither 5‘ nor 3’ primers, *etc.*, using Perl scripts (e.g. locateAdapter.pl) in a special folder (named pacbio), which was integrated into Fastq_clean v2.0 (https://github.com/gaoshanT/Fastq_clean). Statistics and plotting were conducted using the software R v2.15.3 with Bioconductor packages [15]. Using the software Tablet [16] v1.17, five types of artifacts, degraded RNAs, and splicing intermediates were confirmed.

## Supplementary Material

Supplemental Material

## Data Availability

278,243 cp CCS read IDs are included in supplementary files.
