# Supplementary material for: PacBio full-length transcriptome analysis provides new insights into transcription of chloroplast genomes
Source: RNA Biol. 2023 May 25;20(1):248–56. doi: 10.1080/15476286.2023.2214435 (PMC10228390; doi:10.1080/15476286.2023.2214435)
Supplement: Supplemental Material [file s1.pdf › s1.pdf]

SRR17520069.6184
SRR17520069.6185
SRR17520069.6186
SRR17520069.6187
SRR17520069.6188
SRR17520069.6189
SRR17520069.6190
SRR17520069.6191
SRR17520069.6192
SRR17520069.6193
SRR17520069.6194
SRR17520069.6195
SRR17520069.6196
SRR17520069.6197
SRR17520069.6198
SRR17520069.6199
SRR17520069.6200
SRR17520069.6201
SRR17520069.6202
SRR17520069.6203
SRR17520069.6204
SRR17520069.6205
SRR17520069.6206
SRR17520069.6207
SRR17520069.6208
SRR17520069.6209
SRR17520069.6210
SRR17520069.16587
SRR17520069.25589
SRR17520069.25590
SRR17520069.25591
SRR17520069.25592
SRR17520069.25593
SRR17520069.25594
SRR17520069.25595
SRR17520069.25596
SRR17520069.25597
SRR17520069.25598
SRR17520069.25599
SRR17520069.25600
SRR17520069.25601
SRR17520069.25602
SRR17520069.25603
SRR17520069.25604
SRR17520069.25605
SRR17520069.25606
SRR17520069.25607

SRR17520069.25608
SRR17520069.25609
SRR17520069.25610
SRR17520069.25611
SRR17520069.25612
SRR17520069.25613
SRR17520069.25614
SRR17520069.25615
SRR17520069.25616
SRR17520069.25617
SRR17520069.25618
SRR17520069.25619
SRR17520069.25620
SRR17520069.25621
SRR17520069.25622
SRR17520069.25623
SRR17520069.25624
SRR17520069.25625
SRR17520069.25626
SRR17520069.25627
SRR17520069.25628
SRR17520069.25629
SRR17520069.25630
SRR17520069.25631
SRR17520069.25632
SRR17520069.25634
SRR17520069.25635
SRR17520069.25636
SRR17520069.25637
SRR17520069.25638
SRR17520069.25639
SRR17520069.25640
SRR17520069.25641
SRR17520069.25642
SRR17520069.25643
SRR17520069.25644
SRR17520069.25645
SRR17520069.25646
SRR17520069.25647
SRR17520069.25648
SRR17520069.25649
SRR17520069.25650
SRR17520069.25651
SRR17520069.25652
SRR17520069.25653
SRR17520069.25654
SRR17520069.25655

SRR17520069.25656
SRR17520069.25657
SRR17520069.25658
SRR17520069.25659
SRR17520069.25660
SRR17520069.25662
SRR17520069.25663
SRR17520069.25664
SRR17520069.25665
SRR17520069.25666
SRR17520069.25667
SRR17520069.25668
SRR17520069.25669
SRR17520069.25670
SRR17520069.25671
SRR17520069.25672
SRR17520069.25673
SRR17520069.25674
SRR17520069.25675
SRR17520069.25676
SRR17520069.25677
SRR17520069.25678
SRR17520069.25679
SRR17520069.25680
SRR17520069.25681
SRR17520069.25682
SRR17520069.25683
SRR17520069.25684
SRR17520069.25685
SRR17520069.25686
SRR17520069.25687
SRR17520069.25688
SRR17520069.25689
SRR17520069.25690
SRR17520069.25691
SRR17520069.25692
SRR17520069.25693
SRR17520069.25694
SRR17520069.25695
SRR17520069.25696
SRR17520069.25697
SRR17520069.25698
SRR17520069.25699
SRR17520069.25700
SRR17520069.25701
SRR17520069.25702
SRR17520069.25703

SRR17520069.25704
SRR17520069.25706
SRR17520069.25707
SRR17520069.25708
SRR17520069.25709
SRR17520069.25710
SRR17520069.25711
SRR17520069.25712
SRR17520069.25713
SRR17520069.25714
SRR17520069.25715
SRR17520069.25716
SRR17520069.25717
SRR17520069.25718
SRR17520069.25719
SRR17520069.25720
SRR17520069.25721
SRR17520069.25722
SRR17520069.25723
SRR17520069.25724
SRR17520069.25725
SRR17520069.25726
SRR17520069.32032
SRR17520069.32033
SRR17520069.32034
SRR17520069.32035
SRR17520069.32036
SRR17520069.32037
SRR17520069.32038
SRR17520069.32039
SRR17520069.32040
SRR17520069.32041
SRR17520069.32042
SRR17520069.32043
SRR17520069.32044
SRR17520069.32045
SRR17520069.32046
SRR17520069.32047
SRR17520069.32048
SRR17520069.32049
SRR17520069.32050
SRR17520069.32051
SRR17520069.32052
SRR17520069.32053
SRR17520069.32054
SRR17520069.32055
SRR17520069.32056

SRR17520069.32057
SRR17520069.32058
SRR17520069.32059
SRR17520069.32060
SRR17520069.32061
SRR17520069.32062
SRR17520069.32063
SRR17520069.32064
SRR17520069.32065
SRR17520069.32066
SRR17520069.32067
SRR17520069.32068
SRR17520069.32069
SRR17520069.32070
SRR17520069.32071
SRR17520069.32073
SRR17520069.32074
SRR17520069.32075
SRR17520069.32076
SRR17520069.32077
SRR17520069.32078
SRR17520069.32079
SRR17520069.32081
SRR17520069.32082
SRR17520069.32083
SRR17520069.32084
SRR17520069.32085
SRR17520069.32086
SRR17520069.32087
SRR17520069.32088
SRR17520069.32089
SRR17520069.32090
SRR17520069.32091
SRR17520069.32092
SRR17520069.32093
SRR17520069.32094
SRR17520069.32095
SRR17520069.32096
SRR17520069.32097
SRR17520069.32098
SRR17520069.32099
SRR17520069.32100
SRR17520069.32101
SRR17520069.32102
SRR17520069.32103
SRR17520069.32104
SRR17520069.32105

SRR17520069.32106
SRR17520069.33387
SRR17520069.33388
SRR17520069.33389
SRR17520069.33390
SRR17520069.33391
SRR17520069.33392
SRR17520069.33393
SRR17520069.33394
SRR17520069.33395
SRR17520069.33396
SRR17520069.33397
SRR17520069.33398
SRR17520069.33399
SRR17520069.33400
SRR17520069.33401
SRR17520069.33402
SRR17520069.33403
SRR17520069.33404
SRR17520069.33405
SRR17520069.33406
SRR17520069.34484
SRR17520069.34485
SRR17520069.34486
SRR17520069.34487
SRR17520069.34488
SRR17520069.34489
SRR17520069.34490
SRR17520069.34491
SRR17520069.34492
SRR17520069.34493
SRR17520069.34494
SRR17520069.34495
SRR17520069.34496
SRR17520069.34497
SRR17520069.34498
SRR17520069.34499
SRR17520069.34500
SRR17520069.34501
SRR17520069.34502
SRR17520069.34503
SRR17520069.34504
SRR17520069.34505
SRR17520069.34506
SRR17520069.34507
SRR17520069.34508
SRR17520069.34509

SRR17520069.34511
SRR17520069.34512
SRR17520069.34513
SRR17520069.34514
SRR17520069.34515
SRR17520069.34517
SRR17520069.34518
SRR17520069.34519
SRR17520069.34520
SRR17520069.34521
SRR17520069.34522
SRR17520069.34523
SRR17520069.34524
SRR17520069.34525
SRR17520069.34526
SRR17520069.34527
SRR17520069.34528
SRR17520069.34529
SRR17520069.34530
SRR17520069.34531
SRR17520069.34532
SRR17520069.34533
SRR17520069.34534
SRR17520069.34535
SRR17520069.34536
SRR17520069.34537
SRR17520069.34538
SRR17520069.34539
SRR17520069.34540
SRR17520069.34541
SRR17520069.34542
SRR17520069.34543
SRR17520069.34544
SRR17520069.34545
SRR17520069.34546
SRR17520069.34547
SRR17520069.34548
SRR17520069.34549
SRR17520069.34550
SRR17520069.34551
SRR17520069.34552
SRR17520069.34553
SRR17520069.34554
SRR17520069.34555
SRR17520069.34556
SRR17520069.34557
SRR17520069.34558

SRR17520069.34559
SRR17520069.34560
SRR17520069.34561
SRR17520069.34562
SRR17520069.34563
SRR17520069.34564
SRR17520069.34565
SRR17520069.34566
SRR17520069.34567
SRR17520069.34568
SRR17520069.34569
SRR17520069.34570
SRR17520069.34571
SRR17520069.34572
SRR17520069.34573
SRR17520069.34575
SRR17520069.34576
SRR17520069.34577
SRR17520069.34578
SRR17520069.34579
SRR17520069.34580
SRR17520069.34581
SRR17520069.34582
SRR17520069.34583
SRR17520069.34584
SRR17520069.34585
SRR17520069.34586
SRR17520069.34587
SRR17520069.34588
SRR17520069.34589
SRR17520069.34590
SRR17520069.34591
SRR17520069.34592
SRR17520069.34593
SRR17520069.34594
SRR17520069.34595
SRR17520069.48274
SRR17520069.48275
SRR17520069.48276
SRR17520069.48277
SRR17520069.48278
SRR17520069.48279
SRR17520069.48280
SRR17520069.48281
SRR17520069.48282
SRR17520069.48283
SRR17520069.48284

SRR17520069.48285
SRR17520069.48286
SRR17520069.48287
SRR17520069.48288
SRR17520069.48289
SRR17520069.48290
SRR17520069.48291
SRR17520069.48293
SRR17520069.48294
SRR17520069.48295
SRR17520069.48296
SRR17520069.48297
SRR17520069.48298
SRR17520069.48299
SRR17520069.48300
SRR17520069.48301
SRR17520069.48302
SRR17520069.48303
SRR17520069.48304
SRR17520069.48305
SRR17520069.48306
SRR17520069.48307
SRR17520069.48308
SRR17520069.48309
SRR17520069.48310
SRR17520069.48311
SRR17520069.48312
SRR17520069.48313
SRR17520069.48314
SRR17520069.48315
SRR17520069.48316
SRR17520069.48317
SRR17520069.48318
SRR17520069.48319
SRR17520069.48320
SRR17520069.48321
SRR17520069.48322
SRR17520069.48323
SRR17520069.48324
SRR17520069.48325
SRR17520069.48326
SRR17520069.48327
SRR17520069.48328
SRR17520069.48329
SRR17520069.48330
SRR17520069.48331
SRR17520069.48332

SRR17520069.48333
SRR17520069.48334
SRR17520069.48335
SRR17520069.48336
SRR17520069.48337
SRR17520069.48338
SRR17520069.48339
SRR17520069.48340
SRR17520069.48341
SRR17520069.48342
SRR17520069.48343
SRR17520069.48344
SRR17520069.48345
SRR17520069.48346
SRR17520069.48347
SRR17520069.48348
SRR17520069.48349
SRR17520069.48350
SRR17520069.48351
SRR17520069.48352
SRR17520069.48353
SRR17520069.48354
SRR17520069.48355
SRR17520069.48356
SRR17520069.48357
SRR17520069.48358
SRR17520069.48359
SRR17520069.48360
SRR17520069.48361
SRR17520069.48362
SRR17520069.48363
SRR17520069.48364
SRR17520069.48365
SRR17520069.48366
SRR17520069.48367
SRR17520069.48368
SRR17520069.48369
SRR17520069.48370
SRR17520069.48371
SRR17520069.48372
SRR17520069.48373
SRR17520069.48374
SRR17520069.48375
SRR17520069.48376
SRR17520069.48377
SRR17520069.48378
SRR17520069.48379

SRR17520069.48380
SRR17520069.48381
SRR17520069.48382
SRR17520069.48384
SRR17520069.48385
SRR17520069.48386
SRR17520069.48387
SRR17520069.48388
SRR17520069.48389
SRR17520069.48390
SRR17520069.48391
SRR17520069.48392
SRR17520069.48393
SRR17520069.48394
SRR17520069.48395
SRR17520069.48396
SRR17520069.48397
SRR17520069.48398
SRR17520069.48399
SRR17520069.48400
SRR17520069.48401
SRR17520069.48402
SRR17520069.48403
SRR17520069.48404
SRR17520069.48406
SRR17520069.48407
SRR17520069.48408
SRR17520069.48409
SRR17520069.48410
SRR17520069.48411
SRR17520069.48412
SRR17520069.48413
SRR17520069.48414
SRR17520069.48415
SRR17520069.48416
SRR17520069.48417
SRR17520069.48418
SRR17520069.48419
SRR17520069.48420
SRR17520069.48421
SRR17520069.48422
SRR17520069.48423
SRR17520069.48424
SRR17520069.48425
SRR17520069.48426
SRR17520069.59872
SRR17520069.59873

SRR17520069.59874
SRR17520069.59875
SRR17520069.59876
SRR17520069.59877
SRR17520069.59878
SRR17520069.59880
SRR17520069.73358
SRR17520069.73359
SRR17520069.73360
SRR17520069.73361
SRR17520069.73362
SRR17520069.82580
SRR17520069.82581
SRR17520069.82582
SRR17520069.82583
SRR17520069.82584
SRR17520069.82585
SRR17520069.82586
SRR17520069.82587
SRR17520069.82588
SRR17520069.82589
SRR17520069.82590
SRR17520069.82592
SRR17520069.82593
SRR17520069.82594
SRR17520069.82595
SRR17520069.82596
SRR17520069.82597
SRR17520069.82598
SRR17520069.82599
SRR17520069.82600
SRR17520069.82601
SRR17520069.82602
SRR17520069.82603
SRR17520069.82604
SRR17520069.82605
SRR17520069.82606
SRR17520069.82607
SRR17520069.82608
SRR17520069.82609
SRR17520069.82610
SRR17520069.82611
SRR17520069.82612
SRR17520069.82613
SRR17520069.82614
SRR17520069.82615
SRR17520069.82616

SRR17520069.82617
SRR17520069.82618
SRR17520069.82619
SRR17520069.82620
SRR17520069.82621
SRR17520069.82622
SRR17520069.82623
SRR17520069.82624
SRR17520069.82625
SRR17520069.82626
SRR17520069.82627
SRR17520069.82628
SRR17520069.82629
SRR17520069.82631
SRR17520069.82632
SRR17520069.82633
SRR17520069.82634
SRR17520069.82635
SRR17520069.82636
SRR17520069.82637
SRR17520069.82638
SRR17520069.82639
SRR17520069.82640
SRR17520069.82641
SRR17520069.82642
SRR17520069.82643
SRR17520069.82644
SRR17520069.82645
SRR17520069.82646
SRR17520069.82647
SRR17520069.82648
SRR17520069.82649
SRR17520069.82650
SRR17520069.82651
SRR17520069.82652
SRR17520069.82653
SRR17520069.82654
SRR17520069.82655
SRR17520069.82656
SRR17520069.82657
SRR17520069.82658
SRR17520069.82659
SRR17520069.82660
SRR17520069.82661
SRR17520069.82662
SRR17520069.82663
SRR17520069.82664

SRR17520069.82665
SRR17520069.82666
SRR17520069.82667
SRR17520069.82668
SRR17520069.82669
SRR17520069.82670
SRR17520069.82671
SRR17520069.82672
SRR17520069.82673
SRR17520069.82674
SRR17520069.82675
SRR17520069.82676
SRR17520069.82677
SRR17520069.82678
SRR17520069.82679
SRR17520069.82680
SRR17520069.82681
SRR17520069.82682
SRR17520069.82683
SRR17520069.82684
SRR17520069.82685
SRR17520069.82686
SRR17520069.82687
SRR17520069.82688
SRR17520069.82689
SRR17520069.82690
SRR17520069.82691
SRR17520069.82692
SRR17520069.82693
SRR17520069.82694
SRR17520069.82695
SRR17520069.82696
SRR17520069.82697
SRR17520069.82698
SRR17520069.82699
SRR17520069.82700
SRR17520069.82701
SRR17520069.82702
SRR17520069.82703
SRR17520069.82704
SRR17520069.82705
SRR17520069.82706
SRR17520069.82707
SRR17520069.82708
SRR17520069.82709
SRR17520069.82710
SRR17520069.82711

SRR17520069.82712
SRR17520069.82713
SRR17520069.82714
SRR17520069.82715
SRR17520069.82716
SRR17520069.82717
SRR17520069.82718
SRR17520069.82719
SRR17520069.82720
SRR17520069.82721
SRR17520069.82722
SRR17520069.82723
SRR17520069.82724
SRR17520069.82725
SRR17520069.82726
SRR17520069.82727
SRR17520069.114790
SRR17520069.123267
SRR17520069.123268
SRR17520069.123269
SRR17520069.123270
SRR17520069.123271
SRR17520069.123272
SRR17520069.123274
SRR17520069.123343
SRR17520069.123349
SRR17520069.123353
SRR17520069.124757
SRR17520069.125877
SRR17520069.125878
SRR17520069.125879
SRR17520069.125880
SRR17520069.125881
SRR17520069.125882
SRR17520069.125883
SRR17520069.125884
SRR17520069.125885
SRR17520069.125886
SRR17520069.125887
SRR17520069.125888
SRR17520069.125889
SRR17520069.125890
SRR17520069.125891
SRR17520069.125892
SRR17520069.125893
SRR17520069.125894
SRR17520069.125895

SRR17520069.125897
SRR17520069.125898
SRR17520069.125899
SRR17520069.125900
SRR17520069.125901
SRR17520069.125902
SRR17520069.125903
SRR17520069.125904
SRR17520069.125905
SRR17520069.125906
SRR17520069.125907
SRR17520069.125908
SRR17520069.125909
SRR17520069.125910
SRR17520069.125911
SRR17520069.125912
SRR17520069.125913
SRR17520069.125914
SRR17520069.125915
SRR17520069.125916
SRR17520069.125917
SRR17520069.125918
SRR17520069.125919
SRR17520069.125920
SRR17520069.125921
SRR17520069.125922
SRR17520069.125923
SRR17520069.125925
SRR17520069.169186
SRR17520069.169188
SRR17520069.169189
SRR17520069.169190
SRR17520069.169191
SRR17520069.169192
SRR17520069.169193
SRR17520069.169194
SRR17520069.169195
SRR17520069.169196
SRR17520069.169197
SRR17520069.169198
SRR17520069.169199
SRR17520069.169200
SRR17520069.169201
SRR17520069.169202
SRR17520069.169203
SRR17520069.169204
SRR17520069.169205

SRR17520069.169206
SRR17520069.169207
SRR17520069.169208
SRR17520069.169209
SRR17520069.169211
SRR17520069.169212
SRR17520069.169213
SRR17520069.169214
SRR17520069.169215
SRR17520069.169216
SRR17520069.169217
SRR17520069.169219
SRR17520069.169220
SRR17520069.169221
SRR17520069.169222
SRR17520069.169223
SRR17520069.169224
SRR17520069.169225
SRR17520069.169227
SRR17520069.169228
SRR17520069.169229
SRR17520069.169231
SRR17520069.169232
SRR17520069.169233
SRR17520069.186039
SRR17520069.187928
SRR17520069.195800
SRR17520069.195801
SRR17520069.195802
SRR17520069.195803
SRR17520069.195804
SRR17520069.195805
SRR17520069.195806
SRR17520069.204634
SRR17520069.207498
SRR17520069.215938
SRR17520069.215939
SRR17520069.215940
SRR17520069.215941
SRR17520069.215942
SRR17520069.215943
SRR17520069.215944
SRR17520069.215945
SRR17520069.215946
SRR17520069.215947
SRR17520069.218115
SRR17520069.224933

SRR17520069.224934
SRR17520069.224935
SRR17520069.224936
SRR17520069.224937
SRR17520069.224938
SRR17520069.224939
SRR17520069.224940
SRR17520069.224941
SRR17520069.224942
SRR17520069.224943
SRR17520069.224944
SRR17520069.224945
SRR17520069.224946
SRR17520069.224947
SRR17520069.224948
SRR17520069.224949
SRR17520069.224950
SRR17520069.224951
SRR17520069.224952
SRR17520069.224953
SRR17520069.224954
SRR17520069.224955
SRR17520069.224956
SRR17520069.224957
SRR17520069.224958
SRR17520069.224959
SRR17520069.224960
SRR17520069.224961
SRR17520069.224962
SRR17520069.224963
SRR17520069.224964
SRR17520069.224965
SRR17520069.224966
SRR17520069.224967
SRR17520069.224968
SRR17520069.224969
SRR17520069.224970
SRR17520069.224971
SRR17520069.224972
SRR17520069.224973
SRR17520069.224974
SRR17520069.224975
SRR17520069.224976
SRR17520069.224977
SRR17520069.234155
SRR17520069.234187
SRR17520069.234197

SRR17520069.243410
SRR17520069.243411
SRR17520069.257210
SRR17520069.265017
SRR17520069.265018
SRR17520069.265019
SRR17520069.265020
SRR17520069.265021
SRR17520069.265022
SRR17520069.265023
SRR17520069.265024
SRR17520069.265025
SRR17520069.265026
SRR17520069.265027
SRR17520069.265028
SRR17520069.265029
SRR17520069.265030
SRR17520069.265031
SRR17520069.265032
SRR17520069.265033
SRR17520069.265034
SRR17520069.265035
SRR17520069.265036
SRR17520069.265037
SRR17520069.265038
SRR17520069.265039
SRR17520069.265040
SRR17520069.265041
SRR17520069.265042
SRR17520069.265043
SRR17520069.265044
SRR17520069.265045
SRR17520069.265046
SRR17520069.265047
SRR17520069.265048
SRR17520069.265049
SRR17520069.265050
SRR17520069.265051
SRR17520069.265052
SRR17520069.265053
SRR17520069.265054
SRR17520069.265055
SRR17520069.265056
SRR17520069.265057
SRR17520069.265058
SRR17520069.265059
SRR17520069.265060

SRR17520069.265061
SRR17520069.265062
SRR17520069.265063
SRR17520069.265064
SRR17520069.265065
SRR17520069.265066
SRR17520069.265068
SRR17520069.265069
SRR17520069.265070
SRR17520069.265071
SRR17520069.265073
SRR17520069.265074
SRR17520069.265075
SRR17520069.265076
SRR17520069.265077
SRR17520069.265078
SRR17520069.265079
SRR17520069.265080
SRR17520069.265081
SRR17520069.265082
SRR17520069.265083
SRR17520069.265084
SRR17520069.265085
SRR17520069.274683
SRR17520069.274684
SRR17520069.278245
SRR17520069.279424
SRR17520069.279429
SRR17520069.279462
SRR17520069.300871
SRR17520069.301036
SRR17520069.338804
SRR17520069.338805
SRR17520069.338806
SRR17520069.338807
SRR17520069.338808
SRR17520069.338809
SRR17520069.338810
SRR17520069.338811
SRR17520069.338813
SRR17520069.338815
SRR17520069.338817
SRR17520069.338818
SRR17520069.338819
SRR17520069.338820
SRR17520069.338821
SRR17520069.338824

SRR17520069.338825
SRR17520069.338827
SRR17520069.338828
SRR17520069.338829
SRR17520069.338830
SRR17520069.338832
SRR17520069.338833
SRR17520069.338835
SRR17520069.341080
SRR17520069.341422
SRR17520069.341423
SRR17520069.341424
SRR17520069.341425
SRR17520069.341426
SRR17520069.341427
SRR17520069.341429
SRR17520069.341430
SRR17520069.341431
SRR17520069.341432
SRR17520069.341433
SRR17520069.341434
SRR17520069.341435
SRR17520069.341436
SRR17520069.341437
SRR17520069.341438
SRR17520069.341439
SRR17520069.341440
SRR17520069.341441
SRR17520069.341442
SRR17520069.341443
SRR17520069.341444
SRR17520069.341445
SRR17520069.341446
SRR17520069.341447
SRR17520069.341448
SRR17520069.341449
SRR17520069.341450
SRR17520069.341451
SRR17520069.341452
SRR17520069.341453
SRR17520069.341454
SRR17520069.341455
SRR17520069.341456
SRR17520069.341457
SRR17520069.341458
SRR17520069.341459
SRR17520069.341460

SRR17520069.341461
SRR17520069.341462
SRR17520069.341463
SRR17520069.341464
SRR17520069.341465
SRR17520069.341466
SRR17520069.341467
SRR17520069.341468
SRR17520069.341469
SRR17520069.341470
SRR17520069.341471
SRR17520069.341472
SRR17520069.341473
SRR17520069.341474
SRR17520069.341475
SRR17520069.341476
SRR17520069.341478
SRR17520069.341479
SRR17520069.341480
SRR17520069.341481
SRR17520069.341482
SRR17520069.341483
SRR17520069.341484
SRR17520069.341485
SRR17520069.341486
SRR17520069.341487
SRR17520069.341488
SRR17520069.341489
SRR17520069.341490
SRR17520069.341491
SRR17520069.341492
SRR17520069.341493
SRR17520069.341494
SRR17520069.341495
SRR17520069.341496
SRR17520069.341497
SRR17520069.341498
SRR17520069.341499
SRR17520069.341500
SRR17520069.341501
SRR17520069.341502
SRR17520069.341503
SRR17520069.341504
SRR17520069.341506
SRR17520069.341507
SRR17520069.341508
SRR17520069.341509

SRR17520069.341510
SRR17520069.341511
SRR17520069.341512
SRR17520069.341513
SRR17520069.341514
SRR17520069.341515
SRR17520069.396358
SRR17520069.396364
SRR17520069.428059
SRR17520069.428753
SRR17520069.442093
SRR17520069.450622
SRR17520069.450679
SRR17520069.450680
SRR17520069.450681
SRR17520069.450682
SRR17520069.450683
SRR17520069.450684
SRR17520069.450685
SRR17520069.450686
SRR17520069.450687
SRR17520069.450688
SRR17520069.450689
SRR17520069.450690
SRR17520069.450691
SRR17520069.450692
SRR17520069.450693
SRR17520069.450694
SRR17520069.450695
SRR17520069.450696
SRR17520069.450697
SRR17520069.450698
SRR17520069.450699
SRR17520069.450700
SRR17520069.450701
SRR17520069.450702
SRR17520069.450703
SRR17520069.450704
SRR17520069.450705
SRR17520069.450706
SRR17520069.450707
SRR17520069.450708
SRR17520069.450710
SRR17520069.450711
SRR17520069.450712
SRR17520069.450713
SRR17520069.450714

SRR17520069.450715
SRR17520069.450716
SRR17520069.450717
SRR17520069.450718
SRR17520069.450719
SRR17520069.450720
SRR17520069.450721
SRR17520069.450722
SRR17520069.450723
SRR17520069.450724
SRR17520069.450725
SRR17520069.450726
SRR17520069.450727
SRR17520069.450728
SRR17520069.450729
SRR17520069.450730
SRR17520069.450731
SRR17520069.450732
SRR17520069.450733
SRR17520069.450734
SRR17520069.450736
SRR17520069.450737
SRR17520069.450738
SRR17520069.450739
SRR17520069.450740
SRR17520069.450741
SRR17520069.450742
SRR17520069.450743
SRR17520069.450744
SRR17520069.450745
SRR17520069.450746
SRR17520069.450747
SRR17520069.450748
SRR17520069.450749
SRR17520069.450750
SRR17520069.450751
SRR17520069.450752
SRR17520069.450753
SRR17520069.450754
SRR17520069.450755
SRR17520069.450756
SRR17520069.450757
SRR17520069.450758
SRR17520069.450759
SRR17520069.450760
SRR17520069.450761
SRR17520069.450762

SRR17520069.450763
SRR17520069.450764
SRR17520069.450765
SRR17520069.450766
SRR17520069.450767
SRR17520069.450768
SRR17520069.450769
SRR17520069.450770
SRR17520069.450771
SRR17520069.450772
SRR17520069.450773
SRR17520069.450774
SRR17520069.450775
SRR17520069.450776
SRR17520069.450777
SRR17520069.450778
SRR17520069.450779
SRR17520069.450780
SRR17520069.450781
SRR17520069.450782
SRR17520069.450783
SRR17520069.450784
SRR17520069.450785
SRR17520069.450786
SRR17520069.450787
SRR17520069.450788
SRR17520069.450789
SRR17520069.450790
SRR17520069.450791
SRR17520069.450792
SRR17520069.450793
SRR17520069.450794
SRR17520069.450795
SRR17520069.450796
SRR17520069.450797
SRR17520069.450798
SRR17520069.450799
SRR17520069.450800
SRR17520069.450801
SRR17520069.450802
SRR17520069.450803
SRR17520069.450804
SRR17520069.450805
SRR17520069.450806
SRR17520069.450807
SRR17520069.450808
SRR17520069.450809

SRR17520069.450810
SRR17520069.450811
SRR17520069.453700
SRR17520069.453701
SRR17520069.453702
SRR17520069.453703
SRR17520069.453704
SRR17520069.453705
SRR17520069.453706
SRR17520069.453707
SRR17520069.453708
SRR17520069.453709
SRR17520069.453710
SRR17520069.453711
SRR17520069.453712
SRR17520069.453713
SRR17520069.453714
SRR17520069.453715
SRR17520069.453716
SRR17520069.453718
SRR17520069.453719
SRR17520069.453720
SRR17520069.453721
SRR17520069.453722
SRR17520069.453723
SRR17520069.453725
SRR17520069.453726
SRR17520069.453727
SRR17520069.453728
SRR17520069.453729
SRR17520069.453730
SRR17520069.453731
SRR17520069.453733
SRR17520069.453734
SRR17520069.453735
SRR17520069.453736
SRR17520069.453737
SRR17520069.453738
SRR17520069.453739
SRR17520069.453740
SRR17520069.453741
SRR17520069.453742
SRR17520069.453743
SRR17520069.453744
SRR17520069.453745
SRR17520069.453746
SRR17520069.453747

SRR17520069.453749
SRR17520069.453750
SRR17520069.453751
SRR17520069.453752
SRR17520069.453753
SRR17520069.453754
SRR17520069.453755
SRR17520069.453756
SRR17520069.453757
SRR17520069.453758
SRR17520069.453759
SRR17520069.453760
SRR17520069.453761
SRR17520069.453762
SRR17520069.453763
SRR17520069.453764
SRR17520069.453765
SRR17520069.453767
SRR17520069.453768
SRR17520069.453769
SRR17520069.453770
SRR17520069.453772
SRR17520069.453773
SRR17520069.453774
SRR17520069.453775
SRR17520069.453776
SRR17520069.453778
SRR17520069.453779
SRR17520069.453780
SRR17520069.453781
SRR17520069.453783
SRR17520069.453784
SRR17520069.453785
SRR17520069.453786
SRR17520069.453787
SRR17520069.453788
SRR17520069.453789
SRR17520069.453790
SRR17520069.453792
SRR17520069.453793
SRR17520069.453794
SRR17520069.453795
SRR17520069.453797
SRR17520069.453798
SRR17520069.453799
SRR17520069.453800
SRR17520069.453801

SRR17520069.453802
SRR17520069.453803
SRR17520069.453804
SRR17520069.453806
SRR17520069.453807
SRR17520069.453808
SRR17520069.453809
SRR17520069.453810
SRR17520069.453811
SRR17520069.453812
SRR17520069.453813
SRR17520069.453814
SRR17520069.453815
SRR17520069.453816
SRR17520069.453817
SRR17520069.453818
SRR17520069.453820
SRR17520069.453821
SRR17520069.453822
SRR17520069.453823
SRR17520069.453824
SRR17520069.453825
SRR17520069.453826
SRR17520069.453827
SRR17520069.453828
SRR17520069.453829
SRR17520069.453830
SRR17520069.453831
SRR17520069.453832
SRR17520069.453834
SRR17520069.453835
SRR17520069.453836
SRR17520069.453837
SRR17520069.453838
SRR17520069.471884
SRR17520069.478824
SRR17520069.498688
SRR17520069.498689
SRR17520069.498690
SRR17520069.498691
SRR17520069.498692
SRR17520069.498693
SRR17520069.498694
SRR17520069.498695
SRR17520069.498696
SRR17520069.498697
SRR17520069.498698

SRR17520069.498699
SRR17520069.498700
SRR17520069.498701
SRR17520069.498702
SRR17520069.498703
SRR17520069.498704
SRR17520069.498705
SRR17520069.498706
SRR17520069.498707
SRR17520069.498708
SRR17520069.498709
SRR17520069.498710
SRR17520069.498711
SRR17520069.498712
SRR17520069.498713
SRR17520069.498714
SRR17520069.498715
SRR17520069.498716
SRR17520069.498717
SRR17520069.498723
SRR17520069.498724
SRR17520069.498725
SRR17520069.498726
SRR17520069.498727
SRR17520069.498728
SRR17520069.498729
SRR17520069.498730
SRR17520069.498731
SRR17520069.498732
SRR17520069.498733
SRR17520069.498734
SRR17520069.498735
SRR17520069.498736
SRR17520069.498737
SRR17520069.498738
SRR17520069.498739
SRR17520069.498740
SRR17520069.498741
SRR17520069.498742
SRR17520069.498743
SRR17520069.498744
SRR17520069.498745
SRR17520069.498746
SRR17520069.498747
SRR17520069.498748
SRR17520069.498749
SRR17520069.498750

SRR17520069.498751
SRR17520069.498752
SRR17520069.498753
SRR17520069.498754
SRR17520069.498755
SRR17520069.498756
SRR17520069.498757
SRR17520069.498758
SRR17520069.498759
SRR17520069.498760
SRR17520069.498761
SRR17520069.498762
SRR17520069.498763
SRR17520069.498764
SRR17520069.498765
SRR17520069.498766
SRR17520069.498767
SRR17520069.498768
SRR17520069.498769
SRR17520069.498770
SRR17520069.498771
SRR17520069.498772
SRR17520069.498773
SRR17520069.498774
SRR17520069.498775
SRR17520069.498776
SRR17520069.498777
SRR17520069.498778
SRR17520069.498779
SRR17520069.498780
SRR17520069.498781
SRR17520069.498782
SRR17520069.498783
SRR17520069.498784
SRR17520069.498785
SRR17520069.498786
SRR17520069.498787
SRR17520069.498788
SRR17520069.498789
SRR17520069.498791
SRR17520069.498792
SRR17520069.498793
SRR17520069.498794
SRR17520069.498795
SRR17520069.498796
SRR17520069.498797
SRR17520069.498798

SRR17520069.498799
SRR17520069.498800
SRR17520069.498801
SRR17520069.498802
SRR17520069.498803
SRR17520069.498805
SRR17520069.498806
SRR17520069.498807
SRR17520069.498808
SRR17520069.498809
SRR17520069.498810
SRR17520069.498811
SRR17520069.498812
SRR17520069.498813
SRR17520069.498814
SRR17520069.498815
SRR17520069.498816
SRR17520069.498817
SRR17520069.498818
SRR17520069.498819
SRR17520069.498820
SRR17520069.498821
SRR17520069.498822
SRR17520069.498823
SRR17520069.498824
SRR17520069.498825
SRR17520069.498826
SRR17520069.498827
SRR17520069.498828
SRR17520069.498829
SRR17520069.498830
SRR17520069.498831
SRR17520069.498832
SRR17520069.498833
SRR17520069.498834
SRR17520069.501532
SRR17520069.516659
SRR17520069.516661
SRR17520069.521179
SRR17520069.521181
SRR17520069.521182
SRR17520069.521183
SRR17520069.521184
SRR17520069.521185
SRR17520069.521186
SRR17520069.521187
SRR17520069.521188

SRR17520069.521189
SRR17520069.521190
SRR17520069.521191
SRR17520069.521192
SRR17520069.521194
SRR17520069.521195
SRR17520069.521196
SRR17520069.521199
SRR17520069.521200
SRR17520069.521203
SRR17520069.521204
SRR17520069.521205
SRR17520069.521206
SRR17520069.521207
SRR17520069.521208
SRR17520069.521209
SRR17520069.521210
SRR17520069.521212
SRR17520069.521213
SRR17520069.521214
SRR17520069.521215
SRR17520069.521216
SRR17520069.521217
SRR17520069.521218
SRR17520069.521220
SRR17520069.521221
SRR17520069.521948
SRR17520069.541356
SRR17520069.541358
SRR17520069.544944
SRR17520069.544949
SRR17520069.545725
SRR17520069.545751
SRR17520069.552353
SRR17520069.556357
SRR17520069.573821
SRR17520069.573822
SRR17520069.573823
SRR17520069.573824
SRR17520069.573825
SRR17520069.573826
SRR17520069.573827
SRR17520069.573828
SRR17520069.573829
SRR17520069.573830
SRR17520069.573831
SRR17520069.573832

SRR17520069.573833
SRR17520069.573834
SRR17520069.573835
SRR17520069.573836
SRR17520069.573837
SRR17520069.573838
SRR17520069.573839
SRR17520069.573840
SRR17520069.573841
SRR17520069.573842
SRR17520069.573843
SRR17520069.573844
SRR17520069.573845
SRR17520069.573846
SRR17520069.573847
SRR17520069.573848
SRR17520069.573849
SRR17520069.573850
SRR17520069.573851
SRR17520069.573852
SRR17520069.573853
SRR17520069.573854
SRR17520069.573855
SRR17520069.573856
SRR17520069.573857
SRR17520069.573858
SRR17520069.573859
SRR17520069.595016
SRR17520069.595017
SRR17520069.595018
SRR17520069.595019
SRR17520069.595021
SRR17520069.595022
SRR17520069.595023
SRR17520069.595024
SRR17520069.595025
SRR17520069.595026
SRR17520069.595027
SRR17520069.595028
SRR17520069.595029
SRR17520069.595030
SRR17520069.595031
SRR17520069.595032
SRR17520069.595033
SRR17520069.595034
SRR17520069.595035
SRR17520069.595036

SRR17520069.595037
SRR17520069.595038
SRR17520069.595039
SRR17520069.595040
SRR17520069.595041
SRR17520069.595042
SRR17520069.595043
SRR17520069.595044
SRR17520069.595045
SRR17520069.595046
SRR17520069.595047
SRR17520069.595048
SRR17520069.595049
SRR17520069.595050
SRR17520069.595051
SRR17520069.595052
SRR17520069.595053
SRR17520069.595054
SRR17520069.595055
SRR17520069.595056
SRR17520069.595057
SRR17520069.595058
SRR17520069.595059
SRR17520069.595060
SRR17520069.595061
SRR17520069.595062
SRR17520069.595063
SRR17520069.595064
SRR17520069.595065
SRR17520069.595066
SRR17520069.595067
SRR17520069.595068
SRR17520069.595069
SRR17520069.595070
SRR17520069.595071
SRR17520069.595072
SRR17520069.595073
SRR17520069.595074
SRR17520069.595075
SRR17520069.595076
SRR17520069.595077
SRR17520069.595078
SRR17520069.595079
SRR17520069.595080
SRR17520069.595081
SRR17520069.595082
SRR17520069.595083

SRR17520069.595084
SRR17520069.595085
SRR17520069.595086
SRR17520069.595087
SRR17520069.595088
SRR17520069.595089
SRR17520069.595090
SRR17520069.595092
SRR17520069.595093
SRR17520069.595094
SRR17520069.595095
SRR17520069.595096
SRR17520069.595097
SRR17520069.595098
SRR17520069.595099
SRR17520069.595100
SRR17520069.595101
SRR17520069.595102
SRR17520069.595103
SRR17520069.598191
SRR17520069.598192
SRR17520069.598193
SRR17520069.598194
SRR17520069.598197
SRR17520069.598198
SRR17520069.598199
SRR17520069.598200
SRR17520069.598201
SRR17520069.598202
SRR17520069.598203
SRR17520069.598204
SRR17520069.598205
SRR17520069.598206
SRR17520069.598207
SRR17520069.598208
SRR17520069.598209
SRR17520069.598210
SRR17520069.598211
SRR17520069.598212
SRR17520069.598213
SRR17520069.598214
SRR17520069.598215
SRR17520069.598216
SRR17520069.598217
SRR17520069.598218
SRR17520069.598219
SRR17520069.598220

SRR17520069.598221
SRR17520069.598222
SRR17520069.598223
SRR17520069.598224
SRR17520069.598225
SRR17520069.598226
SRR17520069.598228
SRR17520069.598229
SRR17520069.598230
SRR17520069.598231
SRR17520069.598232
SRR17520069.598233
SRR17520069.598234
SRR17520069.598235
SRR17520069.598236
SRR17520069.598237
SRR17520069.598238
SRR17520069.598239
SRR17520069.598240
SRR17520069.598241
SRR17520069.598242
SRR17520069.598243
SRR17520069.598244
SRR17520069.598245
SRR17520069.598246
SRR17520069.598247
SRR17520069.598248
SRR17520069.598249
SRR17520069.598250
SRR17520069.598251
SRR17520069.598252
SRR17520069.598253
SRR17520069.598254
SRR17520069.598255
SRR17520069.598256
SRR17520069.598257
SRR17520069.598258
SRR17520069.598259
SRR17520069.598260
SRR17520069.598261
SRR17520069.598262
SRR17520069.598263
SRR17520069.598264
SRR17520069.598265
SRR17520069.598266
SRR17520069.598267
SRR17520069.598268

SRR17520069.598269
SRR17520069.598270
SRR17520069.598271
SRR17520069.598272
SRR17520069.598273
SRR17520069.598274
SRR17520069.598275
SRR17520069.598276
SRR17520069.598277
SRR17520069.598278
SRR17520069.598279
SRR17520069.598280
SRR17520069.598281
SRR17520069.598282
SRR17520069.598283
SRR17520069.598284
SRR17520069.598285
SRR17520069.598286
SRR17520069.598287
SRR17520069.598288
SRR17520069.598289
SRR17520069.598290
SRR17520069.598291
SRR17520069.598292
SRR17520069.598293
SRR17520069.598294
SRR17520069.598295
SRR17520069.598296
SRR17520069.598297
SRR17520069.598298
SRR17520069.598299
SRR17520069.598300
SRR17520069.598301
SRR17520069.598302
SRR17520069.598304
SRR17520069.598305
SRR17520069.598306
SRR17520069.598307
SRR17520069.598308
SRR17520069.598309
SRR17520069.598310
SRR17520069.598311
SRR17520069.598312
SRR17520069.598313
SRR17520069.598314
SRR17520069.598315
SRR17520069.598316

SRR17520069.598317
SRR17520069.598318
SRR17520069.598319
SRR17520069.598320
SRR17520069.598321
SRR17520069.598322
SRR17520069.598323
SRR17520069.598324
SRR17520069.598325
SRR17520069.598326
SRR17520069.598327
SRR17520069.598328
SRR17520069.598329
SRR17520069.598331
SRR17520069.598332
SRR17520069.598333
SRR17520069.598334
SRR17520069.598335
SRR17520069.598336
SRR17520069.598337
SRR17520069.598338
SRR17520069.598339
SRR17520069.598340
SRR17520069.598341
SRR17520069.598342
SRR17520069.598343
SRR17520069.598344
SRR17520069.598345
SRR17520069.598346
SRR17520069.598347
SRR17520069.598348
SRR17520069.598349
SRR17520069.598350
SRR17520069.598351
SRR17520069.598352
SRR17520069.598353
SRR17520069.598354
SRR17520069.598355
SRR17520069.598356
SRR17520069.598357
SRR17520069.604790
SRR17520069.604791
SRR17520069.604792
SRR17520069.604793
SRR17520069.604794
SRR17520069.604795
SRR17520069.604796

SRR17520069.604797
SRR17520069.604798
SRR17520069.604799
SRR17520069.604800
SRR17520069.604801
SRR17520069.604802
SRR17520069.604803
SRR17520069.604804
SRR17520069.604805
SRR17520069.604806
SRR17520069.604807
SRR17520069.604808
SRR17520069.604809
SRR17520069.604810
SRR17520069.604811
SRR17520069.604813
SRR17520069.604814
SRR17520069.604815
SRR17520069.610036
SRR17520069.622212
SRR17520069.622213
SRR17520069.622214
SRR17520069.622215
SRR17520069.622216
SRR17520069.622217
SRR17520069.622218
SRR17520069.622219
SRR17520069.622220
SRR17520069.622221
SRR17520069.622222
SRR17520069.622223
SRR17520069.622225
SRR17520069.622226
SRR17520069.622228
SRR17520069.622229
SRR17520069.622230
SRR17520069.622231
SRR17520069.622232
SRR17520069.622233
SRR17520069.622235
SRR17520069.622236
SRR17520069.622238
SRR17520069.622240
SRR17520069.622241
SRR17520069.622242
SRR17520069.622243
SRR17520069.622244

SRR17520069.622245
SRR17520069.622247
SRR17520069.622248
SRR17520069.622249
SRR17520069.622250
SRR17520069.622251
SRR17520069.622252
SRR17520069.622253
SRR17520069.622254
SRR17520069.622257
SRR17520069.622258
SRR17520069.622259
SRR17520069.622260
SRR17520069.622261
SRR17520069.622262
SRR17520069.622263
SRR17520069.622264
SRR17520069.622265
SRR17520069.622266
SRR17520069.622267
SRR17520069.622268
SRR17520069.622269
SRR17520069.622270
SRR17520069.622271
SRR17520069.622272
SRR17520069.622273
SRR17520069.622274
SRR17520069.622275
SRR17520069.622276
SRR17520069.622277
SRR17520069.622278
SRR17520069.622279
SRR17520069.622280
SRR17520069.622281
SRR17520069.622282
SRR17520069.622283
SRR17520069.622284
SRR17520069.622285
SRR17520069.622286
SRR17520069.622287
SRR17520069.622288
SRR17520069.622289
SRR17520069.622291
SRR17520069.622292
SRR17520069.622293
SRR17520069.622294
SRR17520069.622295

SRR17520069.622296
SRR17520069.622297
SRR17520069.622299
SRR17520069.622300
SRR17520069.622301
SRR17520069.622302
SRR17520069.622303
SRR17520069.622304
SRR17520069.622305
SRR17520069.622306
SRR17520069.622307
SRR17520069.622308
SRR17520069.622309
SRR17520069.622310
SRR17520069.622311
SRR17520069.622312
SRR17520069.622313
SRR17520069.622314
SRR17520069.622315
SRR17520069.622316
SRR17520069.622317
SRR17520069.622319
SRR17520069.622320
SRR17520069.622321
SRR17520069.622323
SRR17520069.622324
SRR17520069.622326
SRR17520069.622327
SRR17520069.624575
SRR17520069.624588
SRR17520069.624617
SRR17520069.641465
SRR17520069.643900
SRR17520069.643901
SRR17520069.643902
SRR17520069.643903
SRR17520069.643904
SRR17520069.643905
SRR17520069.643906
SRR17520069.643907
SRR17520069.643908
SRR17520069.643909
SRR17520069.643910
SRR17520069.643911
SRR17520069.643913
SRR17520069.643914
SRR17520069.643915

SRR17520069.643916
SRR17520069.643917
SRR17520069.643918
SRR17520069.643919
SRR17520069.643920
SRR17520069.643921
SRR17520069.643924
SRR17520069.643925
SRR17520069.643926
SRR17520069.643927
SRR17520069.643929
SRR17520069.643930
SRR17520069.666042
SRR17520069.666044
SRR17520069.666045
SRR17520069.666046
SRR17520069.666047
SRR17520069.666048
SRR17520069.666049
SRR17520069.666050
SRR17520069.666051
SRR17520069.666052
SRR17520069.666053
SRR17520069.666054
SRR17520069.666055
SRR17520069.666056
SRR17520069.666057
SRR17520069.666058
SRR17520069.693242
SRR17520069.693245
SRR17520069.693246
SRR17520069.693247
SRR17520069.693248
SRR17520069.693249
SRR17520069.693250
SRR17520069.693251
SRR17520069.693252
SRR17520069.693253
SRR17520069.693254
SRR17520069.693255
SRR17520069.693256
SRR17520069.693257
SRR17520069.693258
SRR17520069.693259
SRR17520069.693260
SRR17520069.693261
SRR17520069.693262

SRR17520069.693263
SRR17520069.693264
SRR17520069.693265
SRR17520069.693266
SRR17520069.693267
SRR17520069.693268
SRR17520069.693269
SRR17520069.693270
SRR17520069.693271
SRR17520069.693272
SRR17520069.693273
SRR17520069.693274
SRR17520069.693275
SRR17520069.693276
SRR17520069.693277
SRR17520069.693278
SRR17520069.693279
SRR17520069.693280
SRR17520069.693281
SRR17520069.693282
SRR17520069.693283
SRR17520069.693284
SRR17520069.693285
SRR17520069.693286
SRR17520069.693287
SRR17520069.693288
SRR17520069.693289
SRR17520069.693290
SRR17520069.693291
SRR17520069.693292
SRR17520069.693293
SRR17520069.693294
SRR17520069.693295
SRR17520069.693296
SRR17520069.693297
SRR17520069.693298
SRR17520069.693299
SRR17520069.693300
SRR17520069.693301
SRR17520069.693302
SRR17520069.693303
SRR17520069.693304
SRR17520069.693305
SRR17520069.693306
SRR17520069.693307
SRR17520069.693308
SRR17520069.693309

SRR17520069.693310
SRR17520069.693311
SRR17520069.693312
SRR17520069.693313
SRR17520069.693314
SRR17520069.693316
SRR17520069.693317
SRR17520069.693318
SRR17520069.693319
SRR17520069.693320
SRR17520069.693321
SRR17520069.703682
SRR17520069.703683
SRR17520069.703684
SRR17520069.703685
SRR17520069.703686
SRR17520069.703687
SRR17520069.703690
SRR17520069.703691
SRR17520069.703692
SRR17520069.703693
SRR17520069.719551
SRR17520069.719553
SRR17520069.719554
SRR17520069.719555
SRR17520069.719557
SRR17520069.719558
SRR17520069.719559
SRR17520069.719561
SRR17520069.719562
SRR17520069.719563
SRR17520069.719564
SRR17520069.719565
SRR17520069.719566
SRR17520069.719567
SRR17520069.724920
SRR17520069.724921
SRR17520069.724922
SRR17520069.724923
SRR17520069.724924
SRR17520069.724925
SRR17520069.724926
SRR17520069.724927
SRR17520069.724928
SRR17520069.724929
SRR17520069.724930
SRR17520069.724931

SRR17520069.724932
SRR17520069.724933
SRR17520069.724934
SRR17520069.724935
SRR17520069.724936
SRR17520069.724937
SRR17520069.724938
SRR17520069.724939
SRR17520069.724940
SRR17520069.724941
SRR17520069.724942
SRR17520069.724943
SRR17520069.724944
SRR17520069.724945
SRR17520069.724946
SRR17520069.724947
SRR17520069.724948
SRR17520069.724949
SRR17520069.724950
SRR17520069.724951
SRR17520069.724952
SRR17520069.724953
SRR17520069.724954
SRR17520069.724955
SRR17520069.724956
SRR17520069.724957
SRR17520069.724958
SRR17520069.724959
SRR17520069.724960
SRR17520069.724961
SRR17520069.724962
SRR17520069.724963
SRR17520069.724964
SRR17520069.724965
SRR17520069.724967
SRR17520069.724968
SRR17520069.724969
SRR17520069.724970
SRR17520069.724971
SRR17520069.724972
SRR17520069.724973
SRR17520069.724974
SRR17520069.724975
SRR17520069.724976
SRR17520069.724977
SRR17520069.724978
SRR17520069.724979

SRR17520069.724980
SRR17520069.724981
SRR17520069.724982
SRR17520069.724983
SRR17520069.724984
SRR17520069.724985
SRR17520069.724986
SRR17520069.724987
SRR17520069.724988
SRR17520069.724989
SRR17520069.724990
SRR17520069.724991
SRR17520069.724992
SRR17520069.729518
SRR17520069.729519
SRR17520069.729520
SRR17520069.729521
SRR17520069.729523
SRR17520069.729524
SRR17520069.729525
SRR17520069.729526
SRR17520069.729527
SRR17520069.729528
SRR17520069.729529
SRR17520069.729530
SRR17520069.729531
SRR17520069.729532
SRR17520069.729533
SRR17520069.729534
SRR17520069.729535
SRR17520069.729536
SRR17520069.729537
SRR17520069.729538
SRR17520069.729539
SRR17520069.729540
SRR17520069.729541
SRR17520069.729542
SRR17520069.729543
SRR17520069.729544
SRR17520069.729545
SRR17520069.733054
SRR17520069.733056
SRR17520069.733058
SRR17520069.733060
SRR17520069.733061
SRR17520069.733062
SRR17520069.733063

SRR17520069.733064
SRR17520069.733065
SRR17520069.733066
SRR17520069.733067
SRR17520069.733068
SRR17520069.733069
SRR17520069.733070
SRR17520069.733071
SRR17520069.733072
SRR17520069.733073
SRR17520069.733074
SRR17520069.733075
SRR17520069.733077
SRR17520069.733078
SRR17520069.733079
SRR17520069.733080
SRR17520069.733081
SRR17520069.733082
SRR17520069.733083
SRR17520069.733084
SRR17520069.733085
SRR17520069.733086
SRR17520069.733087
SRR17520069.733088
SRR17520069.733089
SRR17520069.733090
SRR17520069.733091
SRR17520069.733092
SRR17520069.733093
SRR17520069.733094
SRR17520069.733095
SRR17520069.733096
SRR17520069.733097
SRR17520069.733098
SRR17520069.733099
SRR17520069.733100
SRR17520069.733101
SRR17520069.733103
SRR17520069.733104
SRR17520069.733105
SRR17520069.733106
SRR17520069.733107
SRR17520069.733108
SRR17520069.733109
SRR17520069.733110
SRR17520069.733112
SRR17520069.733113

SRR17520069.733114
SRR17520069.733115
SRR17520069.733116
SRR17520069.733117
SRR17520069.733118
SRR17520069.733119
SRR17520069.733120
SRR17520069.733121
SRR17520069.733122
SRR17520069.733123
SRR17520069.733124
SRR17520069.733125
SRR17520069.733126
SRR17520069.733127
SRR17520069.733128
SRR17520069.733129
SRR17520069.733130
SRR17520069.733131
SRR17520069.733132
SRR17520069.733133
SRR17520069.733134
SRR17520069.733135
SRR17520069.733136
SRR17520069.733138
SRR17520069.733139
SRR17520069.733140
SRR17520069.733141
SRR17520069.733142
SRR17520069.733143
SRR17520069.733144
SRR17520069.733145
SRR17520069.733146
SRR17520069.733147
SRR17520069.733148
SRR17520069.733149
SRR17520069.733150
SRR17520069.733151
SRR17520069.733152
SRR17520069.733153
SRR17520069.733154
SRR17520069.733155
SRR17520069.733156
SRR17520069.733157
SRR17520069.733158
SRR17520069.733159
SRR17520069.733160
SRR17520069.733161

SRR17520069.733162
SRR17520069.733163
SRR17520069.733164
SRR17520069.733165
SRR17520069.733166
SRR17520069.733167
SRR17520069.733168
SRR17520069.733169
SRR17520069.733170
SRR17520069.733171
SRR17520069.733172
SRR17520069.733173
SRR17520069.733174
SRR17520069.733175
SRR17520069.733176
SRR17520069.733177
SRR17520069.733178
SRR17520069.733179
SRR17520069.733180
SRR17520069.733181
SRR17520069.733182
SRR17520069.733183
SRR17520069.733184
SRR17520069.733185
SRR17520069.733186
SRR17520069.733187
SRR17520069.733188
SRR17520069.733189
SRR17520069.733190
SRR17520069.733191
SRR17520069.733192
SRR17520069.733193
SRR17520069.733194
SRR17520069.733462
SRR17520069.739317
SRR17520069.739424
SRR17520069.739428
SRR17520069.745374
SRR17520069.745375
SRR17520069.750349
SRR17520069.750350
SRR17520069.750351
SRR17520069.750352
SRR17520069.750354
SRR17520069.750356
SRR17520069.750358
SRR17520069.752037

SRR17520069.752365
SRR17520069.789413
SRR17520069.789415
SRR17520069.798128
SRR17520069.803867
SRR17520069.803868
SRR17520069.803869
SRR17520069.803870
SRR17520069.803871
SRR17520069.803872
SRR17520069.803873
SRR17520069.803874
SRR17520069.803875
SRR17520069.803876
SRR17520069.803877
SRR17520069.803878
SRR17520069.803879
SRR17520069.803880
SRR17520069.803881
SRR17520069.803882
SRR17520069.803883
SRR17520069.803884
SRR17520069.803885
SRR17520069.803886
SRR17520069.803887
SRR17520069.803888
SRR17520069.803889
SRR17520069.803890
SRR17520069.803891
SRR17520069.803892
SRR17520069.803893
SRR17520069.803894
SRR17520069.803895
SRR17520069.803896
SRR17520069.803897
SRR17520069.803898
SRR17520069.803899
SRR17520069.803900
SRR17520069.803901
SRR17520069.803902
SRR17520069.803903
SRR17520069.803904
SRR17520069.803905
SRR17520069.803906
SRR17520069.803907
SRR17520069.803908
SRR17520069.803909

SRR17520069.803910
SRR17520069.803911
SRR17520069.803914
SRR17520069.803915
SRR17520069.803916
SRR17520069.803917
SRR17520069.803918
SRR17520069.803919
SRR17520069.803920
SRR17520069.803921
SRR17520069.803922
SRR17520069.803923
SRR17520069.803926
SRR17520069.803927
SRR17520069.803928
SRR17520069.803929
SRR17520069.803930
SRR17520069.803931
SRR17520069.803932
SRR17520069.803933
SRR17520069.803934
SRR17520069.803935
SRR17520069.803936
SRR17520069.803937
SRR17520069.803938
SRR17520069.803939
SRR17520069.803940
SRR17520069.803941
SRR17520069.803942
SRR17520069.803943
SRR17520069.803944
SRR17520069.803945
SRR17520069.803946
SRR17520069.803947
SRR17520069.803948
SRR17520069.803949
SRR17520069.803950
SRR17520069.803951
SRR17520069.803952
SRR17520069.803953
SRR17520069.803954
SRR17520069.803955
SRR17520069.803956
SRR17520069.803957
SRR17520069.803958
SRR17520069.803959
SRR17520069.803960

SRR17520069.803961
SRR17520069.803962
SRR17520069.803963
SRR17520069.803964
SRR17520069.803965
SRR17520069.803966
SRR17520069.803967
SRR17520069.803968
SRR17520069.803969
SRR17520069.803970
SRR17520069.803971
SRR17520069.803972
SRR17520069.803973
SRR17520069.803974
SRR17520069.803975
SRR17520069.803976
SRR17520069.803977
SRR17520069.803978
SRR17520069.803979
SRR17520069.803980
SRR17520069.803981
SRR17520069.803982
SRR17520069.803983
SRR17520069.803984
SRR17520069.803985
SRR17520069.803986
SRR17520069.803987
SRR17520069.803988
SRR17520069.803989
SRR17520069.803990
SRR17520069.803991
SRR17520069.803992
SRR17520069.803993
SRR17520069.803994
SRR17520069.803995
SRR17520069.803996
SRR17520069.803997
SRR17520069.803998
SRR17520069.803999
SRR17520069.804000
SRR17520069.804001
SRR17520069.804002
SRR17520069.804003
SRR17520069.804004
SRR17520069.804006
SRR17520069.804008
SRR17520069.804009

SRR17520069.804010
SRR17520069.804011
SRR17520069.804012
SRR17520069.804013
SRR17520069.804014
SRR17520069.804015
SRR17520069.804016
SRR17520069.804017
SRR17520069.804018
SRR17520069.804019
SRR17520069.804020
SRR17520069.804021
SRR17520069.804022
SRR17520069.804023
SRR17520069.804024
SRR17520069.805874
SRR17520069.835412
SRR17520069.838481
SRR17520069.838482
SRR17520069.838483
SRR17520069.838484
SRR17520069.838485
SRR17520069.838486
SRR17520069.838487
SRR17520069.838488
SRR17520069.838489
SRR17520069.838490
SRR17520069.838492
SRR17520069.838493
SRR17520069.838494
SRR17520069.838495
SRR17520069.838496
SRR17520069.838497
SRR17520069.838498
SRR17520069.838499
SRR17520069.838500
SRR17520069.838501
SRR17520069.838502
SRR17520069.838503
SRR17520069.838504
SRR17520069.838505
SRR17520069.838506
SRR17520069.838507
SRR17520069.838508
SRR17520069.838509
SRR17520069.838510
SRR17520069.838511

SRR17520069.838512
SRR17520069.838513
SRR17520069.838514
SRR17520069.838515
SRR17520069.838516
SRR17520069.838517
SRR17520069.838518
SRR17520069.838519
SRR17520069.838520
SRR17520069.838521
SRR17520069.838522
SRR17520069.838523
SRR17520069.838524
SRR17520069.838525
SRR17520069.838526
SRR17520069.838527
SRR17520069.838528
SRR17520069.838529
SRR17520069.838530
SRR17520069.838531
SRR17520069.838532
SRR17520069.838533
SRR17520069.838534
SRR17520069.838535
SRR17520069.838536
SRR17520069.838537
SRR17520069.838538
SRR17520069.838539
SRR17520069.838540
SRR17520069.838541
SRR17520069.838542
SRR17520069.838543
SRR17520069.838544
SRR17520069.838545
SRR17520069.838546
SRR17520069.838547
SRR17520069.838548
SRR17520069.838549
SRR17520069.838550
SRR17520069.838551
SRR17520069.838552
SRR17520069.838553
SRR17520069.838554
SRR17520069.838555
SRR17520069.838556
SRR17520069.838557
SRR17520069.838558

SRR17520069.838559
SRR17520069.838560
SRR17520069.838561
SRR17520069.838562
SRR17520069.838563
SRR17520069.838564
SRR17520069.838565
SRR17520069.838566
SRR17520069.838567
SRR17520069.838568
SRR17520069.838569
SRR17520069.838570
SRR17520069.838571
SRR17520069.838572
SRR17520069.838573
SRR17520069.838574
SRR17520069.838575
SRR17520069.838576
SRR17520069.838577
SRR17520069.838578
SRR17520069.838579
SRR17520069.838580
SRR17520069.838581
SRR17520069.838582
SRR17520069.838583
SRR17520069.838584
SRR17520069.838585
SRR17520069.838586
SRR17520069.838587
SRR17520069.838588
SRR17520069.838589
SRR17520069.838590
SRR17520069.838591
SRR17520069.838592
SRR17520069.838593
SRR17520069.838594
SRR17520069.838595
SRR17520069.838596
SRR17520069.838597
SRR17520069.838598
SRR17520069.838599
SRR17520069.838600
SRR17520069.838601
SRR17520069.838602
SRR17520069.838603
SRR17520069.838604
SRR17520069.838605

SRR17520069.838606
SRR17520069.838607
SRR17520069.838608
SRR17520069.838609
SRR17520069.838610
SRR17520069.838611
SRR17520069.838612
SRR17520069.838613
SRR17520069.838614
SRR17520069.838615
SRR17520069.838617
SRR17520069.838618
SRR17520069.838619
SRR17520069.838620
SRR17520069.838621
SRR17520069.847139
SRR17520069.867376
SRR17520069.869609
SRR17520069.869610
SRR17520069.869611
SRR17520069.869612
SRR17520069.869613
SRR17520069.869614
SRR17520069.869615
SRR17520069.869616
SRR17520069.869617
SRR17520069.883124
SRR17520069.887598
SRR17520069.893577
SRR17520069.893578
SRR17520069.893579
SRR17520069.893580
SRR17520069.893581
SRR17520069.893582
SRR17520069.893583
SRR17520069.893584
SRR17520069.893585
SRR17520069.893586
SRR17520069.893587
SRR17520069.893588
SRR17520069.893589
SRR17520069.893590
SRR17520069.893591
SRR17520069.893593
SRR17520069.893594
SRR17520069.893595
SRR17520069.893597

SRR17520069.893600
SRR17520069.895683
SRR17520069.895684
SRR17520069.895685
SRR17520069.895686
SRR17520069.895687
SRR17520069.895688
SRR17520069.895689
SRR17520069.911331
SRR17520069.911332
SRR17520069.911334
SRR17520069.911335
SRR17520069.911338
SRR17520069.911339
SRR17520069.911340
SRR17520069.911341
SRR17520069.911342
SRR17520069.911343
SRR17520069.911344
SRR17520069.911345
SRR17520069.911346
SRR17520069.911347
SRR17520069.911348
SRR17520069.911349
SRR17520069.911350
SRR17520069.911351
SRR17520069.911353
SRR17520069.911354
SRR17520069.911355
SRR17520069.911356
SRR17520069.911357
SRR17520069.911359
SRR17520069.911360
SRR17520069.911361
SRR17520069.911362
SRR17520069.911364
SRR17520069.911366
SRR17520069.911367
SRR17520069.911370
SRR17520069.911371
SRR17520069.911372
SRR17520069.911374
SRR17520069.911375
SRR17520069.911376
SRR17520069.911377
SRR17520069.911378
SRR17520069.911379

SRR17520069.911380
SRR17520069.911381
SRR17520069.911382
SRR17520069.911383
SRR17520069.911384
SRR17520069.911385
SRR17520069.911386
SRR17520069.911387
SRR17520069.911388
SRR17520069.911389
SRR17520069.911390
SRR17520069.911392
SRR17520069.911393
SRR17520069.911394
SRR17520069.911395
SRR17520069.911396
SRR17520069.911397
SRR17520069.911398
SRR17520069.911399
SRR17520069.911400
SRR17520069.911401
SRR17520069.911402
SRR17520069.911403
SRR17520069.911404
SRR17520069.911406
SRR17520069.911411
SRR17520069.911412
SRR17520069.911413
SRR17520069.911415
SRR17520069.912567
SRR17520069.912568
SRR17520069.912569
SRR17520069.912570
SRR17520069.912571
SRR17520069.912572
SRR17520069.912573
SRR17520069.912574
SRR17520069.912575
SRR17520069.912576
SRR17520069.912577
SRR17520069.912578
SRR17520069.912579
SRR17520069.912580
SRR17520069.912581
SRR17520069.912582
SRR17520069.912583
SRR17520069.912584

SRR17520069.912585
SRR17520069.912586
SRR17520069.912587
SRR17520069.912588
SRR17520069.924442
SRR17520069.924443
SRR17520069.924444
SRR17520069.924445
SRR17520069.924446
SRR17520069.924447
SRR17520069.924448
SRR17520069.924449
SRR17520069.924450
SRR17520069.924451
SRR17520069.924452
SRR17520069.924453
SRR17520069.924454
SRR17520069.924455
SRR17520069.924456
SRR17520069.924457
SRR17520069.924458
SRR17520069.924459
SRR17520069.924460
SRR17520069.924461
SRR17520069.924462
SRR17520069.924463
SRR17520069.924464
SRR17520069.924465
SRR17520069.924466
SRR17520069.924467
SRR17520069.924468
SRR17520069.924469
SRR17520069.924470
SRR17520069.924471
SRR17520069.924472
SRR17520069.924473
SRR17520069.924474
SRR17520069.924475
SRR17520069.924477
SRR17520069.924478
SRR17520069.924479
SRR17520069.924480
SRR17520069.924481
SRR17520069.924482
SRR17520069.924483
SRR17520069.924484
SRR17520069.924485

SRR17520069.924486
SRR17520069.924487
SRR17520069.924488
SRR17520069.924489
SRR17520069.924490
SRR17520069.924491
SRR17520069.924492
SRR17520069.924493
SRR17520069.924494
SRR17520069.924495
SRR17520069.924496
SRR17520069.924497
SRR17520069.924498
SRR17520069.924499
SRR17520069.924500
SRR17520069.924501
SRR17520069.924502
SRR17520069.924503
SRR17520069.924504
SRR17520069.924505
SRR17520069.924506
SRR17520069.924507
SRR17520069.924508
SRR17520069.924509
SRR17520069.924510
SRR17520069.924511
SRR17520069.924512
SRR17520069.924513
SRR17520069.924514
SRR17520069.924515
SRR17520069.924516
SRR17520069.924517
SRR17520069.924518
SRR17520069.924519
SRR17520069.924520
SRR17520069.924521
SRR17520069.924522
SRR17520069.924523
SRR17520069.924524
SRR17520069.924525
SRR17520069.924526
SRR17520069.924527
SRR17520069.924528
SRR17520069.924529
SRR17520069.924530
SRR17520069.924531
SRR17520069.924532

SRR17520069.924533
SRR17520069.924534
SRR17520069.924535
SRR17520069.924536
SRR17520069.924537
SRR17520069.924538
SRR17520069.924539
SRR17520069.924540
SRR17520069.924541
SRR17520069.924542
SRR17520069.924543
SRR17520069.924544
SRR17520069.924545
SRR17520069.924546
SRR17520069.924547
SRR17520069.924548
SRR17520069.924549
SRR17520069.924550
SRR17520069.924551
SRR17520069.924552
SRR17520069.924553
SRR17520069.924554
SRR17520069.924555
SRR17520069.924556
SRR17520069.924557
SRR17520069.924558
SRR17520069.924559
SRR17520069.924560
SRR17520069.924561
SRR17520069.924562
SRR17520069.924563
SRR17520069.924564
SRR17520069.924565
SRR17520069.924566
SRR17520069.924567
SRR17520069.924568
SRR17520069.924569
SRR17520069.924570
SRR17520069.924571
SRR17520069.924572
SRR17520069.924573
SRR17520069.924574
SRR17520069.924575
SRR17520069.924576
SRR17520069.924577
SRR17520069.924578
SRR17520069.924579

SRR17520069.924580
SRR17520069.924581
SRR17520069.924582
SRR17520069.924583
SRR17520069.924584
SRR17520069.924585
SRR17520069.924586
SRR17520069.924587
SRR17520069.924588
SRR17520069.924589
SRR17520069.924590
SRR17520069.924591
SRR17520069.924592
SRR17520069.924593
SRR17520069.924594
SRR17520069.924595
SRR17520069.924596
SRR17520069.924597
SRR17520069.924598
SRR17520069.924599
SRR17520069.924600
SRR17520069.924601
SRR17520069.924602
SRR17520069.924603
SRR17520069.924604
SRR17520069.924605
SRR17520069.924606
SRR17520069.924607
SRR17520069.924608
SRR17520069.924609
SRR17520069.924610
SRR17520069.924611
SRR17520069.924612
SRR17520069.924613
SRR17520069.924614
SRR17520069.924615
SRR17520069.924617
SRR17520069.924619
SRR17520069.924620
SRR17520069.924621
SRR17520069.924622
SRR17520069.924623
SRR17520069.924624
SRR17520069.924625
SRR17520069.924626
SRR17520069.924627
SRR17520069.924628

SRR17520069.924629
SRR17520069.924630
SRR17520069.924631
SRR17520069.924632
SRR17520069.924633
SRR17520069.924634
SRR17520069.924635
SRR17520069.937642
SRR17520069.941713
SRR17520069.941715
SRR17520069.941717
SRR17520069.941719
SRR17520069.941720
SRR17520069.941721
SRR17520069.941722
SRR17520069.941723
SRR17520069.941724
SRR17520069.941725
SRR17520069.941728
SRR17520069.941729
SRR17520069.941730
SRR17520069.943294
SRR17520069.943295
SRR17520069.943296
SRR17520069.943298
SRR17520069.943299
SRR17520069.943300
SRR17520069.943301
SRR17520069.948063
SRR17520069.949888
SRR17520069.949889
SRR17520069.949890
SRR17520069.949891
SRR17520069.949892
SRR17520069.949893
SRR17520069.949894
SRR17520069.949895
SRR17520069.949897
SRR17520069.949898
SRR17520069.949899
SRR17520069.949900
SRR17520069.949901
SRR17520069.949902
SRR17520069.949903
SRR17520069.949904
SRR17520069.949905
SRR17520069.949906

SRR17520069.949907
SRR17520069.949908
SRR17520069.949909
SRR17520069.949911
SRR17520069.949912
SRR17520069.949914
SRR17520069.949915
SRR17520069.949916
SRR17520069.949917
SRR17520069.949918
SRR17520069.949919
SRR17520069.949920
SRR17520069.949921
SRR17520069.949922
SRR17520069.949923
SRR17520069.949924
SRR17520069.949925
SRR17520069.949926
SRR17520069.949927
SRR17520069.949928
SRR17520069.949929
SRR17520069.949930
SRR17520069.949931
SRR17520069.949932
SRR17520069.949933
SRR17520069.949934
SRR17520069.949935
SRR17520069.949936
SRR17520069.949937
SRR17520069.949938
SRR17520069.949939
SRR17520069.949940
SRR17520069.949942
SRR17520069.949943
SRR17520069.949944
SRR17520069.949945
SRR17520069.949946
SRR17520069.949947
SRR17520069.949948
SRR17520069.949950
SRR17520069.949951
SRR17520069.949952
SRR17520069.949953
SRR17520069.949954
SRR17520069.949955
SRR17520069.949956
SRR17520069.949957

SRR17520069.951321
SRR17520069.957048
SRR17520069.957049
SRR17520069.957050
SRR17520069.957051
SRR17520069.957052
SRR17520069.957053
SRR17520069.957054
SRR17520069.957055
SRR17520069.957056
SRR17520069.957057
SRR17520069.957058
SRR17520069.957059
SRR17520069.957060
SRR17520069.957061
SRR17520069.957062
SRR17520069.964025
SRR17520069.998923
SRR17520069.1010036
SRR17520069.1010332
SRR17520069.1010333
SRR17520069.1010334
SRR17520069.1010335
SRR17520069.1010336
SRR17520069.1010337
SRR17520069.1010338
SRR17520069.1010339
SRR17520069.1010340
SRR17520069.1010341
SRR17520069.1010342
SRR17520069.1010343
SRR17520069.1010344
SRR17520069.1010345
SRR17520069.1010346
SRR17520069.1010347
SRR17520069.1010348
SRR17520069.1010349
SRR17520069.1010351
SRR17520069.1010352
SRR17520069.1010353
SRR17520069.1010354
SRR17520069.1010355
SRR17520069.1010356
SRR17520069.1010357
SRR17520069.1010358
SRR17520069.1010359
SRR17520069.1013727

SRR17520069.1013728
SRR17520069.1013729
SRR17520069.1013730
SRR17520069.1013731
SRR17520069.1022779
SRR17520069.1025295
SRR17520069.1025296
SRR17520069.1025297
SRR17520069.1025298
SRR17520069.1025299
SRR17520069.1025300
SRR17520069.1025301
SRR17520069.1025302
SRR17520069.1025303
SRR17520069.1025304
SRR17520069.1025305
SRR17520069.1025306
SRR17520069.1025307
SRR17520069.1025308
SRR17520069.1025309
SRR17520069.1025310
SRR17520069.1025311
SRR17520069.1025312
SRR17520069.1039207
SRR17520069.1048896
SRR17520069.1048898
SRR17520069.1048899
SRR17520069.1048900
SRR17520069.1048901
SRR17520069.1048902
SRR17520069.1048903
SRR17520069.1048904
SRR17520069.1048905
SRR17520069.1048906
SRR17520069.1048907
SRR17520069.1048908
SRR17520069.1048909
SRR17520069.1048910
SRR17520069.1069198
SRR17520069.1069199
SRR17520069.1069200
SRR17520069.1069201
SRR17520069.1069202
SRR17520069.1069203
SRR17520069.1069204
SRR17520069.1069205
SRR17520069.1069206

SRR17520069.1069207
SRR17520069.1069209
SRR17520069.1069210
SRR17520069.1069211
SRR17520069.1069212
SRR17520069.1069213
SRR17520069.1069214
SRR17520069.1069215
SRR17520069.1069216
SRR17520069.1069217
SRR17520069.1069218
SRR17520069.1069219
SRR17520069.1069220
SRR17520069.1069221
SRR17520069.1069222
SRR17520069.1069223
SRR17520069.1069224
SRR17520069.1069225
SRR17520069.1069226
SRR17520069.1069227
SRR17520069.1069228
SRR17520069.1069229
SRR17520069.1069230
SRR17520069.1069231
SRR17520069.1069232
SRR17520069.1069233
SRR17520069.1069234
SRR17520069.1069235
SRR17520069.1069236
SRR17520069.1069237
SRR17520069.1069238
SRR17520069.1069239
SRR17520069.1069240
SRR17520069.1069241
SRR17520069.1069242
SRR17520069.1069243
SRR17520069.1069244
SRR17520069.1069245
SRR17520069.1069246
SRR17520069.1069247
SRR17520069.1069248
SRR17520069.1069249
SRR17520069.1069250
SRR17520069.1069251
SRR17520069.1069252
SRR17520069.1069253
SRR17520069.1069254

SRR17520069.1069255
SRR17520069.1069256
SRR17520069.1069257
SRR17520069.1069258
SRR17520069.1069259
SRR17520069.1069260
SRR17520069.1069261
SRR17520069.1069262
SRR17520069.1069263
SRR17520069.1069264
SRR17520069.1069265
SRR17520069.1069266
SRR17520069.1069267
SRR17520069.1069268
SRR17520069.1069269
SRR17520069.1069270
SRR17520069.1069272
SRR17520069.1069273
SRR17520069.1069274
SRR17520069.1069275
SRR17520069.1069276
SRR17520069.1069277
SRR17520069.1069278
SRR17520069.1069279
SRR17520069.1069280
SRR17520069.1069281
SRR17520069.1069282
SRR17520069.1069283
SRR17520069.1069284
SRR17520069.1069285
SRR17520069.1069286
SRR17520069.1069287
SRR17520069.1069288
SRR17520069.1069289
SRR17520069.1069290
SRR17520069.1069291
SRR17520069.1069292
SRR17520069.1069293
SRR17520069.1069294
SRR17520069.1069295
SRR17520069.1069296
SRR17520069.1069297
SRR17520069.1069298
SRR17520069.1069299
SRR17520069.1069300
SRR17520069.1069301
SRR17520069.1069302

SRR17520069.1069303
SRR17520069.1069304
SRR17520069.1069305
SRR17520069.1069306
SRR17520069.1069307
SRR17520069.1069308
SRR17520069.1069309
SRR17520069.1069310
SRR17520069.1069311
SRR17520069.1069312
SRR17520069.1069313
SRR17520069.1069314
SRR17520069.1069315
SRR17520069.1069316
SRR17520069.1069317
SRR17520069.1069318
SRR17520069.1069319
SRR17520069.1069320
SRR17520069.1084219
SRR17520069.1084221
SRR17520069.1084222
SRR17520069.1084223
SRR17520069.1084224
SRR17520069.1084226
SRR17520069.1084227
SRR17520069.1084228
SRR17520069.1084229
SRR17520069.1084230
SRR17520069.1084232
SRR17520069.1084233
SRR17520069.1084235
SRR17520069.1084237
SRR17520069.1084238
SRR17520069.1084239
SRR17520069.1084241
SRR17520069.1084242
SRR17520069.1084243
SRR17520069.1084244
SRR17520069.1084245
SRR17520069.1084246
SRR17520069.1084247
SRR17520069.1084248
SRR17520069.1084249
SRR17520069.1084250
SRR17520069.1084251
SRR17520069.1084253
SRR17520069.1084254

SRR17520069.1084255
SRR17520069.1084256
SRR17520069.1084257
SRR17520069.1084258
SRR17520069.1084259
SRR17520069.1084260
SRR17520069.1084261
SRR17520069.1084262
SRR17520069.1084263
SRR17520069.1084264
SRR17520069.1084265
SRR17520069.1084266
SRR17520069.1084267
SRR17520069.1084268
SRR17520069.1084269
SRR17520069.1084270
SRR17520069.1084271
SRR17520069.1084273
SRR17520069.1084274
SRR17520069.1084275
SRR17520069.1084276
SRR17520069.1084277
SRR17520069.1084278
SRR17520069.1084279
SRR17520069.1084280
SRR17520069.1084282
SRR17520069.1084283
SRR17520069.1084285
SRR17520069.1084286
SRR17520069.1084287
SRR17520069.1084288
SRR17520069.1088952
SRR17520069.1088953
SRR17520069.1088955
SRR17520069.1100030
SRR17520069.1100031
SRR17520069.1100032
SRR17520069.1100033
SRR17520069.1100034
SRR17520069.1100035
SRR17520069.1100036
SRR17520069.1100037
SRR17520069.1100038
SRR17520069.1100039
SRR17520069.1100040
SRR17520069.1100041
SRR17520069.1100042

SRR17520069.1100043
SRR17520069.1100044
SRR17520069.1100045
SRR17520069.1100046
SRR17520069.1100047
SRR17520069.1100048
SRR17520069.1100049
SRR17520069.1100050
SRR17520069.1100051
SRR17520069.1100052
SRR17520069.1100053
SRR17520069.1100054
SRR17520069.1100055
SRR17520069.1100056
SRR17520069.1100057
SRR17520069.1100058
SRR17520069.1100059
SRR17520069.1100060
SRR17520069.1100061
SRR17520069.1100062
SRR17520069.1100063
SRR17520069.1100064
SRR17520069.1100065
SRR17520069.1100066
SRR17520069.1100067
SRR17520069.1100068
SRR17520069.1100069
SRR17520069.1100070
SRR17520069.1100071
SRR17520069.1100072
SRR17520069.1100073
SRR17520069.1100074
SRR17520069.1100075
SRR17520069.1100076
SRR17520069.1100077
SRR17520069.1100078
SRR17520069.1100079
SRR17520069.1100080
SRR17520069.1100081
SRR17520069.1100082
SRR17520069.1100083
SRR17520069.1100084
SRR17520069.1100085
SRR17520069.1100086
SRR17520069.1100087
SRR17520069.1100088
SRR17520069.1100089

SRR17520069.1100090
SRR17520069.1100091
SRR17520069.1100092
SRR17520069.1100093
SRR17520069.1100094
SRR17520069.1100095
SRR17520069.1100096
SRR17520069.1100097
SRR17520069.1100098
SRR17520069.1100099
SRR17520069.1100100
SRR17520069.1100101
SRR17520069.1100102
SRR17520069.1100103
SRR17520069.1100104
SRR17520069.1100105
SRR17520069.1100106
SRR17520069.1100107
SRR17520069.1100108
SRR17520069.1100109
SRR17520069.1100110
SRR17520069.1100111
SRR17520069.1100112
SRR17520069.1100113
SRR17520069.1100114
SRR17520069.1100115
SRR17520069.1100116
SRR17520069.1100117
SRR17520069.1100118
SRR17520069.1100119
SRR17520069.1100120
SRR17520069.1100121
SRR17520069.1100122
SRR17520069.1100123
SRR17520069.1100124
SRR17520069.1100125
SRR17520069.1100126
SRR17520069.1100127
SRR17520069.1100128
SRR17520069.1100129
SRR17520069.1100130
SRR17520069.1100131
SRR17520069.1100132
SRR17520069.1100133
SRR17520069.1100134
SRR17520069.1100135
SRR17520069.1100136

SRR17520069.1100137
SRR17520069.1100138
SRR17520069.1100139
SRR17520069.1100140
SRR17520069.1100141
SRR17520069.1100142
SRR17520069.1100143
SRR17520069.1100144
SRR17520069.1100145
SRR17520069.1100146
SRR17520069.1100147
SRR17520069.1100148
SRR17520069.1100150
SRR17520069.1100152
SRR17520069.1100153
SRR17520069.1100154
SRR17520069.1100155
SRR17520069.1100157
SRR17520069.1100158
SRR17520069.1100159
SRR17520069.1100160
SRR17520069.1100161
SRR17520069.1100162
SRR17520069.1100163
SRR17520069.1100165
SRR17520069.1100169
SRR17520069.1100170
SRR17520069.1100172
SRR17520069.1100173
SRR17520069.1100174
SRR17520069.1100175
SRR17520069.1100176
SRR17520069.1100177
SRR17520069.1100179
SRR17520069.1100180
SRR17520069.1100181
SRR17520069.1100182
SRR17520069.1100183
SRR17520069.1100184
SRR17520069.1100185
SRR17520069.1104069
SRR17520069.1121357
SRR17520069.1121358
SRR17520069.1121359
SRR17520069.1121360
SRR17520069.1121361
SRR17520069.1121362

SRR17520069.1121363
SRR17520069.1121364
SRR17520069.1121365
SRR17520069.1121366
SRR17520069.1121367
SRR17520069.1121368
SRR17520069.1121369
SRR17520069.1121370
SRR17520069.1121371
SRR17520069.1121372
SRR17520069.1121373
SRR17520069.1121374
SRR17520069.1121375
SRR17520069.1121376
SRR17520069.1121377
SRR17520069.1121378
SRR17520069.1121379
SRR17520069.1121380
SRR17520069.1121381
SRR17520069.1121382
SRR17520069.1121383
SRR17520069.1121385
SRR17520069.1121386
SRR17520069.1121387
SRR17520069.1121388
SRR17520069.1121389
SRR17520069.1121390
SRR17520069.1121391
SRR17520069.1121392
SRR17520069.1121393
SRR17520069.1121394
SRR17520069.1121395
SRR17520069.1121396
SRR17520069.1121397
SRR17520069.1121398
SRR17520069.1121399
SRR17520069.1121400
SRR17520069.1121401
SRR17520069.1121402
SRR17520069.1121403
SRR17520069.1121404
SRR17520069.1121405
SRR17520069.1121406
SRR17520069.1121407
SRR17520069.1121408
SRR17520069.1121409
SRR17520069.1121410

SRR17520069.1121411
SRR17520069.1121412
SRR17520069.1121413
SRR17520069.1121414
SRR17520069.1121415
SRR17520069.1121416
SRR17520069.1121417
SRR17520069.1128683
SRR17520069.1128684
SRR17520069.1128685
SRR17520069.1128686
SRR17520069.1128687
SRR17520069.1128688
SRR17520069.1128689
SRR17520069.1128690
SRR17520069.1128691
SRR17520069.1128692
SRR17520069.1128693
SRR17520069.1128694
SRR17520069.1128695
SRR17520069.1128696
SRR17520069.1128697
SRR17520069.1128698
SRR17520069.1128699
SRR17520069.1128700
SRR17520069.1128701
SRR17520069.1128702
SRR17520069.1128703
SRR17520069.1128704
SRR17520069.1128705
SRR17520069.1128706
SRR17520069.1128707
SRR17520069.1128708
SRR17520069.1128709
SRR17520069.1128710
SRR17520069.1128711
SRR17520069.1128712
SRR17520069.1128713
SRR17520069.1128714
SRR17520069.1128715
SRR17520069.1128716
SRR17520069.1128717
SRR17520069.1128718
SRR17520069.1128719
SRR17520069.1128720
SRR17520069.1128721
SRR17520069.1128722

SRR17520069.1128723
SRR17520069.1128724
SRR17520069.1128725
SRR17520069.1128726
SRR17520069.1128727
SRR17520069.1128728
SRR17520069.1128730
SRR17520069.1128731
SRR17520069.1128732
SRR17520069.1128733
SRR17520069.1128734
SRR17520069.1128735
SRR17520069.1128736
SRR17520069.1128737
SRR17520069.1128738
SRR17520069.1128740
SRR17520069.1128741
SRR17520069.1128742
SRR17520069.1128743
SRR17520069.1128744
SRR17520069.1128745
SRR17520069.1128746
SRR17520069.1128747
SRR17520069.1128748
SRR17520069.1128749
SRR17520069.1128750
SRR17520069.1128751
SRR17520069.1128752
SRR17520069.1128753
SRR17520069.1128756
SRR17520069.1128757
SRR17520069.1128758
SRR17520069.1128759
SRR17520069.1128760
SRR17520069.1128761
SRR17520069.1128762
SRR17520069.1128763
SRR17520069.1128764
SRR17520069.1128765
SRR17520069.1128766
SRR17520069.1128767
SRR17520069.1128768
SRR17520069.1128769
SRR17520069.1128770
SRR17520069.1128771
SRR17520069.1128772
SRR17520069.1128773

SRR17520069.1128775
SRR17520069.1128776
SRR17520069.1128777
SRR17520069.1128778
SRR17520069.1128779
SRR17520069.1128780
SRR17520069.1128781
SRR17520069.1128782
SRR17520069.1128783
SRR17520069.1128784
SRR17520069.1128785
SRR17520069.1128786
SRR17520069.1128788
SRR17520069.1128789
SRR17520069.1128790
SRR17520069.1128791
SRR17520069.1128792
SRR17520069.1128793
SRR17520069.1128794
SRR17520069.1128795
SRR17520069.1128796
SRR17520069.1128797
SRR17520069.1128798
SRR17520069.1128799
SRR17520069.1128800
SRR17520069.1128801
SRR17520069.1128802
SRR17520069.1128803
SRR17520069.1128804
SRR17520069.1128805
SRR17520069.1128806
SRR17520069.1128807
SRR17520069.1128808
SRR17520069.1128809
SRR17520069.1128810
SRR17520069.1128811
SRR17520069.1128812
SRR17520069.1128813
SRR17520069.1128814
SRR17520069.1128815
SRR17520069.1128816
SRR17520069.1128817
SRR17520069.1128818
SRR17520069.1128819
SRR17520069.1136749
SRR17520069.1141608
SRR17520069.1141637

SRR17520069.1152655
SRR17520069.1152656
SRR17520069.1152657
SRR17520069.1152658
SRR17520069.1152659
SRR17520069.1152660
SRR17520069.1152664
SRR17520069.1152665
SRR17520069.1152670
SRR17520069.1152671
SRR17520069.1152674
SRR17520069.1152676
SRR17520069.1152678
SRR17520069.1152682
SRR17520069.1152683
SRR17520069.1183066
SRR17520069.1189913
SRR17520069.1189914
SRR17520069.1189915
SRR17520069.1189916
SRR17520069.1189917
SRR17520069.1189918
SRR17520069.1189919
SRR17520069.1189920
SRR17520069.1189921
SRR17520069.1189922
SRR17520069.1189923
SRR17520069.1189924
SRR17520069.1189925
SRR17520069.1189926
SRR17520069.1189927
SRR17520069.1189928
SRR17520069.1189929
SRR17520069.1189930
SRR17520069.1189931
SRR17520069.1189932
SRR17520069.1189933
SRR17520069.1189934
SRR17520069.1189935
SRR17520069.1189936
SRR17520069.1189937
SRR17520069.1189938
SRR17520069.1189939
SRR17520069.1189940
SRR17520069.1189941
SRR17520069.1189942
SRR17520069.1189943

SRR17520069.1189944
SRR17520069.1189945
SRR17520069.1189947
SRR17520069.1189948
SRR17520069.1189949
SRR17520069.1189950
SRR17520069.1189951
SRR17520069.1189952
SRR17520069.1189953
SRR17520069.1189954
SRR17520069.1189955
SRR17520069.1189956
SRR17520069.1189957
SRR17520069.1189958
SRR17520069.1189959
SRR17520069.1189960
SRR17520069.1189961
SRR17520069.1189962
SRR17520069.1189963
SRR17520069.1189964
SRR17520069.1189965
SRR17520069.1189966
SRR17520069.1189967
SRR17520069.1189968
SRR17520069.1189969
SRR17520069.1189970
SRR17520069.1189971
SRR17520069.1189972
SRR17520069.1189973
SRR17520069.1189974
SRR17520069.1189975
SRR17520069.1189976
SRR17520069.1189977
SRR17520069.1189978
SRR17520069.1189979
SRR17520069.1189980
SRR17520069.1189981
SRR17520069.1189982
SRR17520069.1189983
SRR17520069.1189984
SRR17520069.1189985
SRR17520069.1189986
SRR17520069.1189987
SRR17520069.1189988
SRR17520069.1189989
SRR17520069.1189990
SRR17520069.1189991

SRR17520069.1189992
SRR17520069.1189994
SRR17520069.1189995
SRR17520069.1189996
SRR17520069.1189997
SRR17520069.1189998
SRR17520069.1189999
SRR17520069.1190000
SRR17520069.1190001
SRR17520069.1190002
SRR17520069.1190003
SRR17520069.1190004
SRR17520069.1190005
SRR17520069.1190006
SRR17520069.1190007
SRR17520069.1190008
SRR17520069.1190009
SRR17520069.1190010
SRR17520069.1190011
SRR17520069.1190012
SRR17520069.1190013
SRR17520069.1190015
SRR17520069.1190016
SRR17520069.1202067
SRR17520069.1202068
SRR17520069.1202069
SRR17520069.1202070
SRR17520069.1202071
SRR17520069.1202072
SRR17520069.1202073
SRR17520069.1202074
SRR17520069.1202075
SRR17520069.1202076
SRR17520069.1202077
SRR17520069.1202078
SRR17520069.1202079
SRR17520069.1202080
SRR17520069.1202081
SRR17520069.1202082
SRR17520069.1202083
SRR17520069.1202084
SRR17520069.1202085
SRR17520069.1202086
SRR17520069.1202087
SRR17520069.1202088
SRR17520069.1202089
SRR17520069.1202090

SRR17520069.1202091
SRR17520069.1202092
SRR17520069.1202094
SRR17520069.1202095
SRR17520069.1202096
SRR17520069.1202097
SRR17520069.1202098
SRR17520069.1202099
SRR17520069.1202100
SRR17520069.1202101
SRR17520069.1202102
SRR17520069.1202103
SRR17520069.1202104
SRR17520069.1202105
SRR17520069.1202106
SRR17520069.1202107
SRR17520069.1202108
SRR17520069.1202109
SRR17520069.1202110
SRR17520069.1202111
SRR17520069.1202112
SRR17520069.1202113
SRR17520069.1202114
SRR17520069.1202115
SRR17520069.1202116
SRR17520069.1202117
SRR17520069.1202118
SRR17520069.1202119
SRR17520069.1202120
SRR17520069.1202121
SRR17520069.1202122
SRR17520069.1202123
SRR17520069.1202124
SRR17520069.1202125
SRR17520069.1202126
SRR17520069.1202127
SRR17520069.1202128
SRR17520069.1202129
SRR17520069.1202130
SRR17520069.1202131
SRR17520069.1202132
SRR17520069.1202133
SRR17520069.1202134
SRR17520069.1202135
SRR17520069.1202136
SRR17520069.1202137
SRR17520069.1202138

SRR17520069.1202139
SRR17520069.1202140
SRR17520069.1202141
SRR17520069.1202142
SRR17520069.1202143
SRR17520069.1202144
SRR17520069.1202145
SRR17520069.1202146
SRR17520069.1202147
SRR17520069.1202148
SRR17520069.1202149
SRR17520069.1202150
SRR17520069.1202151
SRR17520069.1202152
SRR17520069.1202153
SRR17520069.1202154
SRR17520069.1202155
SRR17520069.1202156
SRR17520069.1202157
SRR17520069.1202158
SRR17520069.1202159
SRR17520069.1202160
SRR17520069.1202161
SRR17520069.1202162
SRR17520069.1202163
SRR17520069.1202164
SRR17520069.1211814
SRR17520069.1253748
SRR17520069.1253749
SRR17520069.1253750
SRR17520069.1253751
SRR17520069.1253752
SRR17520069.1264747
SRR17520069.1264748
SRR17520069.1264750
SRR17520069.1264751
SRR17520069.1264752
SRR17520069.1264753
SRR17520069.1264754
SRR17520069.1264755
SRR17520069.1264756
SRR17520069.1264757
SRR17520069.1264758
SRR17520069.1264759
SRR17520069.1264760
SRR17520069.1264761
SRR17520069.1264762

SRR17520069.1264763
SRR17520069.1264766
SRR17520069.1264767
SRR17520069.1264768
SRR17520069.1264769
SRR17520069.1264770
SRR17520069.1264771
SRR17520069.1264772
SRR17520069.1264774
SRR17520069.1264775
SRR17520069.1264776
SRR17520069.1264777
SRR17520069.1264778
SRR17520069.1264779
SRR17520069.1264780
SRR17520069.1266056
SRR17520069.1266057
SRR17520069.1266058
SRR17520069.1266059
SRR17520069.1266060
SRR17520069.1266061
SRR17520069.1266062
SRR17520069.1266063
SRR17520069.1266064
SRR17520069.1266065
SRR17520069.1266066
SRR17520069.1266067
SRR17520069.1266068
SRR17520069.1266069
SRR17520069.1266070
SRR17520069.1266071
SRR17520069.1266072
SRR17520069.1266073
SRR17520069.1266074
SRR17520069.1266075
SRR17520069.1266076
SRR17520069.1266077
SRR17520069.1266078
SRR17520069.1266079
SRR17520069.1266080
SRR17520069.1266081
SRR17520069.1266082
SRR17520069.1266083
SRR17520069.1266084
SRR17520069.1266085
SRR17520069.1266086
SRR17520069.1266087

SRR17520069.1266088
SRR17520069.1266089
SRR17520069.1266090
SRR17520069.1266091
SRR17520069.1266092
SRR17520069.1266093
SRR17520069.1266094
SRR17520069.1266095
SRR17520069.1266096
SRR17520069.1266097
SRR17520069.1266098
SRR17520069.1266099
SRR17520069.1266100
SRR17520069.1266101
SRR17520069.1266102
SRR17520069.1266103
SRR17520069.1266104
SRR17520069.1266105
SRR17520069.1266106
SRR17520069.1266108
SRR17520069.1266109
SRR17520069.1266110
SRR17520069.1266111
SRR17520069.1266112
SRR17520069.1266113
SRR17520069.1266114
SRR17520069.1266115
SRR17520069.1266117
SRR17520069.1266118
SRR17520069.1266119
SRR17520069.1266120
SRR17520069.1266121
SRR17520069.1266122
SRR17520069.1266123
SRR17520069.1266124
SRR17520069.1266125
SRR17520069.1266126
SRR17520069.1266127
SRR17520069.1266128
SRR17520069.1266129
SRR17520069.1266130
SRR17520069.1266132
SRR17520069.1266133
SRR17520069.1266135
SRR17520069.1266136
SRR17520069.1266137
SRR17520069.1266138

SRR17520069.1266140
SRR17520069.1266141
SRR17520069.1266142
SRR17520069.1266143
SRR17520069.1266144
SRR17520069.1266145
SRR17520069.1266146
SRR17520069.1266147
SRR17520069.1266148
SRR17520069.1266149
SRR17520069.1266150
SRR17520069.1266151
SRR17520069.1266152
SRR17520069.1266153
SRR17520069.1266154
SRR17520069.1266155
SRR17520069.1266156
SRR17520069.1266157
SRR17520069.1266158
SRR17520069.1266159
SRR17520069.1266160
SRR17520069.1266161
SRR17520069.1266162
SRR17520069.1266163
SRR17520069.1266164
SRR17520069.1266165
SRR17520069.1266166
SRR17520069.1266167
SRR17520069.1266168
SRR17520069.1266169
SRR17520069.1266170
SRR17520069.1266171
SRR17520069.1266172
SRR17520069.1266173
SRR17520069.1266174
SRR17520069.1266175
SRR17520069.1266176
SRR17520069.1266177
SRR17520069.1266178
SRR17520069.1266179
SRR17520069.1266180
SRR17520069.1266181
SRR17520069.1266182
SRR17520069.1266183
SRR17520069.1266184
SRR17520069.1266185
SRR17520069.1278812

SRR17520069.1278869
SRR17520069.1278909
SRR17520069.1316683
SRR17520069.1316684
SRR17520069.1316686
SRR17520069.1316687
SRR17520069.1316688
SRR17520069.1316689
SRR17520069.1316690
SRR17520069.1316691
SRR17520069.1316692
SRR17520069.1316693
SRR17520069.1316694
SRR17520069.1316695
SRR17520069.1316696
SRR17520069.1316697
SRR17520069.1316698
SRR17520069.1316699
SRR17520069.1316700
SRR17520069.1316701
SRR17520069.1316702
SRR17520069.1316703
SRR17520069.1316704
SRR17520069.1316705
SRR17520069.1316706
SRR17520069.1316707
SRR17520069.1316708
SRR17520069.1316709
SRR17520069.1316710
SRR17520069.1316711
SRR17520069.1316712
SRR17520069.1316713
SRR17520069.1316714
SRR17520069.1316715
SRR17520069.1316716
SRR17520069.1316717
SRR17520069.1316718
SRR17520069.1316719
SRR17520069.1316720
SRR17520069.1316721
SRR17520069.1316722
SRR17520069.1316723
SRR17520069.1316724
SRR17520069.1316725
SRR17520069.1316726
SRR17520069.1316727
SRR17520069.1316728

SRR17520069.1316729
SRR17520069.1316730
SRR17520069.1316731
SRR17520069.1316732
SRR17520069.1316733
SRR17520069.1316734
SRR17520069.1316735
SRR17520069.1316736
SRR17520069.1316737
SRR17520069.1316738
SRR17520069.1316739
SRR17520069.1316740
SRR17520069.1316741
SRR17520069.1316742
SRR17520069.1316743
SRR17520069.1316744
SRR17520069.1316745
SRR17520069.1316746
SRR17520069.1316747
SRR17520069.1316748
SRR17520069.1316749
SRR17520069.1316750
SRR17520069.1316751
SRR17520069.1316752
SRR17520069.1316753
SRR17520069.1316754
SRR17520069.1316755
SRR17520069.1316756
SRR17520069.1316757
SRR17520069.1316758
SRR17520069.1316759
SRR17520069.1316760
SRR17520069.1316763
SRR17520069.1316764
SRR17520069.1316765
SRR17520069.1316766
SRR17520069.1316767
SRR17520069.1316768
SRR17520069.1316769
SRR17520069.1316770
SRR17520069.1316771
SRR17520069.1316772
SRR17520069.1316774
SRR17520069.1316775
SRR17520069.1316776
SRR17520069.1316777
SRR17520069.1316778

SRR17520069.1316779
SRR17520069.1316780
SRR17520069.1316781
SRR17520069.1316782
SRR17520069.1316783
SRR17520069.1316785
SRR17520069.1316786
SRR17520069.1316787
SRR17520069.1316788
SRR17520069.1316790
SRR17520069.1316792
SRR17520069.1316793
SRR17520069.1316794
SRR17520069.1316795
SRR17520069.1316796
SRR17520069.1316798
SRR17520069.1316799
SRR17520069.1316800
SRR17520069.1316801
SRR17520069.1316802
SRR17520069.1316803
SRR17520069.1316804
SRR17520069.1316805
SRR17520069.1316806
SRR17520069.1316807
SRR17520069.1316808
SRR17520069.1316809
SRR17520069.1316810
SRR17520069.1316811
SRR17520069.1316812
SRR17520069.1316813
SRR17520069.1334237
SRR17520069.1334238
SRR17520069.1334239
SRR17520069.1334240
SRR17520069.1334241
SRR17520069.1334242
SRR17520069.1334243
SRR17520069.1334244
SRR17520069.1334245
SRR17520069.1334246
SRR17520069.1334247
SRR17520069.1334248
SRR17520069.1334249
SRR17520069.1334250
SRR17520069.1334251
SRR17520069.1334252

SRR17520069.1334253
SRR17520069.1334254
SRR17520069.1334255
SRR17520069.1334256
SRR17520069.1334257
SRR17520069.1334258
SRR17520069.1334259
SRR17520069.1336037
SRR17520069.1336057
SRR17520069.1342643
SRR17520069.1342644
SRR17520069.1342645
SRR17520069.1342646
SRR17520069.1342647
SRR17520069.1342648
SRR17520069.1342649
SRR17520069.1342650
SRR17520069.1342651
SRR17520069.1342652
SRR17520069.1342653
SRR17520069.1347273
SRR17520069.1347274
SRR17520069.1347275
SRR17520069.1347276
SRR17520069.1347277
SRR17520069.1347278
SRR17520069.1347279
SRR17520069.1347280
SRR17520069.1347281
SRR17520069.1347282
SRR17520069.1347283
SRR17520069.1347284
SRR17520069.1347285
SRR17520069.1347286
SRR17520069.1347287
SRR17520069.1347288
SRR17520069.1347289
SRR17520069.1347290
SRR17520069.1347291
SRR17520069.1347292
SRR17520069.1347293
SRR17520069.1347294
SRR17520069.1347295
SRR17520069.1347296
SRR17520069.1347297
SRR17520069.1347298
SRR17520069.1347299

SRR17520069.1347300
SRR17520069.1347301
SRR17520069.1347302
SRR17520069.1347303
SRR17520069.1347304
SRR17520069.1347305
SRR17520069.1347306
SRR17520069.1347307
SRR17520069.1347308
SRR17520069.1347309
SRR17520069.1347310
SRR17520069.1347311
SRR17520069.1347312
SRR17520069.1347314
SRR17520069.1347315
SRR17520069.1347316
SRR17520069.1347317
SRR17520069.1347318
SRR17520069.1347319
SRR17520069.1347320
SRR17520069.1347321
SRR17520069.1347322
SRR17520069.1347323
SRR17520069.1347324
SRR17520069.1347325
SRR17520069.1347326
SRR17520069.1347327
SRR17520069.1347328
SRR17520069.1347329
SRR17520069.1347330
SRR17520069.1347331
SRR17520069.1347332
SRR17520069.1347333
SRR17520069.1347334
SRR17520069.1347335
SRR17520069.1347337
SRR17520069.1347338
SRR17520069.1347339
SRR17520069.1347340
SRR17520069.1347341
SRR17520069.1347343
SRR17520069.1347345
SRR17520069.1347346
SRR17520069.1347347
SRR17520069.1347348
SRR17520069.1347349
SRR17520069.1347350

SRR17520069.1347351
SRR17520069.1347352
SRR17520069.1347353
SRR17520069.1347354
SRR17520069.1347355
SRR17520069.1347356
SRR17520069.1347357
SRR17520069.1347358
SRR17520069.1347359
SRR17520069.1347360
SRR17520069.1347361
SRR17520069.1347362
SRR17520069.1347363
SRR17520069.1347364
SRR17520069.1347365
SRR17520069.1347366
SRR17520069.1347368
SRR17520069.1347369
SRR17520069.1347370
SRR17520069.1347371
SRR17520069.1347372
SRR17520069.1347373
SRR17520069.1347374
SRR17520069.1347375
SRR17520069.1347376
SRR17520069.1347377
SRR17520069.1347378
SRR17520069.1347379
SRR17520069.1347380
SRR17520069.1347381
SRR17520069.1347382
SRR17520069.1347383
SRR17520069.1347384
SRR17520069.1347385
SRR17520069.1347386
SRR17520069.1347387
SRR17520069.1347388
SRR17520069.1347389
SRR17520069.1347390
SRR17520069.1347391
SRR17520069.1347392
SRR17520069.1347393
SRR17520069.1347394
SRR17520069.1347395
SRR17520069.1347396
SRR17520069.1347397
SRR17520069.1347398

SRR17520069.1347399
SRR17520069.1347400
SRR17520069.1347401
SRR17520069.1347402
SRR17520069.1347403
SRR17520069.1347404
SRR17520069.1347406
SRR17520069.1347407
SRR17520069.1347408
SRR17520069.1347409
SRR17520069.1347410
SRR17520069.1347413
SRR17520069.1347414
SRR17520069.1347415
SRR17520069.1347416
SRR17520069.1347417
SRR17520069.1347418
SRR17520069.1347419
SRR17520069.1347420
SRR17520069.1347421
SRR17520069.1347422
SRR17520069.1347423
SRR17520069.1347424
SRR17520069.1347425
SRR17520069.1347426
SRR17520069.1347427
SRR17520069.1347428
SRR17520069.1347429
SRR17520069.1347430
SRR17520069.1347431
SRR17520069.1347432
SRR17520069.1347433
SRR17520069.1347434
SRR17520069.1347435
SRR17520069.1347436
SRR17520069.1347438
SRR17520069.1347439
SRR17520069.1347440
SRR17520069.1347441
SRR17520069.1347442
SRR17520069.1347443
SRR17520069.1348410
SRR17520069.1348411
SRR17520069.1348412
SRR17520069.1348413
SRR17520069.1348414
SRR17520069.1348415

SRR17520069.1348416
SRR17520069.1348417
SRR17520069.1348418
SRR17520069.1348419
SRR17520069.1348420
SRR17520069.1348421
SRR17520069.1348422
SRR17520069.1348423
SRR17520069.1348424
SRR17520069.1348425
SRR17520069.1348426
SRR17520069.1348427
SRR17520069.1348428
SRR17520069.1396743
SRR17520069.1396815
SRR17520069.1396824
SRR17520069.1412661
SRR17520069.1412663
SRR17520069.1412664
SRR17520069.1412665
SRR17520069.1412666
SRR17520069.1412668
SRR17520069.1412669
SRR17520069.1412670
SRR17520069.1412671
SRR17520069.1412672
SRR17520069.1412673
SRR17520069.1412674
SRR17520069.1412675
SRR17520069.1412676
SRR17520069.1412677
SRR17520069.1412678
SRR17520069.1412679
SRR17520069.1412680
SRR17520069.1412681
SRR17520069.1412682
SRR17520069.1412683
SRR17520069.1412684
SRR17520069.1412685
SRR17520069.1412686
SRR17520069.1412687
SRR17520069.1412688
SRR17520069.1412689
SRR17520069.1412690
SRR17520069.1412691
SRR17520069.1412693
SRR17520069.1412694

SRR17520069.1412695
SRR17520069.1412696
SRR17520069.1412697
SRR17520069.1412698
SRR17520069.1412699
SRR17520069.1412700
SRR17520069.1412701
SRR17520069.1412702
SRR17520069.1412703
SRR17520069.1412704
SRR17520069.1412705
SRR17520069.1412706
SRR17520069.1412707
SRR17520069.1412708
SRR17520069.1412710
SRR17520069.1412711
SRR17520069.1412712
SRR17520069.1412714
SRR17520069.1412715
SRR17520069.1412716
SRR17520069.1412717
SRR17520069.1412718
SRR17520069.1412719
SRR17520069.1412720
SRR17520069.1412721
SRR17520069.1412722
SRR17520069.1412723
SRR17520069.1412725
SRR17520069.1412726
SRR17520069.1412727
SRR17520069.1412728
SRR17520069.1412729
SRR17520069.1412730
SRR17520069.1412731
SRR17520069.1412732
SRR17520069.1412733
SRR17520069.1412734
SRR17520069.1412735
SRR17520069.1412736
SRR17520069.1412737
SRR17520069.1412738
SRR17520069.1412739
SRR17520069.1412740
SRR17520069.1412741
SRR17520069.1412742
SRR17520069.1412743
SRR17520069.1412744

SRR17520069.1412745
SRR17520069.1412746
SRR17520069.1412747
SRR17520069.1412748
SRR17520069.1412749
SRR17520069.1412750
SRR17520069.1412751
SRR17520069.1412752
SRR17520069.1412753
SRR17520069.1412754
SRR17520069.1412756
SRR17520069.1435598
SRR17520069.1435599
SRR17520069.1435600
SRR17520069.1435601
SRR17520069.1435602
SRR17520069.1435603
SRR17520069.1435604
SRR17520069.1435605
SRR17520069.1435606
SRR17520069.1435607
SRR17520069.1435608
SRR17520069.1435609
SRR17520069.1435610
SRR17520069.1435611
SRR17520069.1435612
SRR17520069.1435613
SRR17520069.1435614
SRR17520069.1435615
SRR17520069.1435616
SRR17520069.1435618
SRR17520069.1435619
SRR17520069.1435620
SRR17520069.1435621
SRR17520069.1435622
SRR17520069.1435623
SRR17520069.1435624
SRR17520069.1435625
SRR17520069.1435626
SRR17520069.1435627
SRR17520069.1435629
SRR17520069.1435630
SRR17520069.1435631
SRR17520069.1435632
SRR17520069.1435633
SRR17520069.1435634
SRR17520069.1435635

SRR17520069.1435636
SRR17520069.1435637
SRR17520069.1435638
SRR17520069.1435639
SRR17520069.1435640
SRR17520069.1435641
SRR17520069.1435642
SRR17520069.1435643
SRR17520069.1435644
SRR17520069.1435645
SRR17520069.1435646
SRR17520069.1435647
SRR17520069.1435648
SRR17520069.1435649
SRR17520069.1435650
SRR17520069.1435651
SRR17520069.1435652
SRR17520069.1435653
SRR17520069.1435654
SRR17520069.1435655
SRR17520069.1435656
SRR17520069.1435657
SRR17520069.1435658
SRR17520069.1435659
SRR17520069.1435660
SRR17520069.1435661
SRR17520069.1446773
SRR17520069.1446817
SRR17520069.1462670
SRR17520069.1462671
SRR17520069.1478849
SRR17520069.1486580
SRR17520069.1486581
SRR17520069.1486582
SRR17520069.1486583
SRR17520069.1486584
SRR17520069.1486585
SRR17520069.1486586
SRR17520069.1486587
SRR17520069.1486588
SRR17520069.1486589
SRR17520069.1486590
SRR17520069.1486591
SRR17520069.1486592
SRR17520069.1486593
SRR17520069.1486594
SRR17520069.1486595

SRR17520069.1486596
SRR17520069.1486597
SRR17520069.1486598
SRR17520069.1486599
SRR17520069.1498690
SRR17520069.1498691
SRR17520069.1498692
SRR17520069.1498693
SRR17520069.1498694
SRR17520069.1498695
SRR17520069.1498696
SRR17520069.1498697
SRR17520069.1498698
SRR17520069.1498699
SRR17520069.1498700
SRR17520069.1498701
SRR17520069.1498702
SRR17520069.1498703
SRR17520069.1498704
SRR17520069.1498705
SRR17520069.1498706
SRR17520069.1498707
SRR17520069.1498708
SRR17520069.1498709
SRR17520069.1498710
SRR17520069.1498711
SRR17520069.1498712
SRR17520069.1498713
SRR17520069.1498714
SRR17520069.1498715
SRR17520069.1498716
SRR17520069.1498717
SRR17520069.1498718
SRR17520069.1498719
SRR17520069.1498720
SRR17520069.1498721
SRR17520069.1498722
SRR17520069.1498723
SRR17520069.1498724
SRR17520069.1498725
SRR17520069.1498726
SRR17520069.1498727
SRR17520069.1498728
SRR17520069.1498729
SRR17520069.1498730
SRR17520069.1498731
SRR17520069.1498732

SRR17520069.1498733
SRR17520069.1498735
SRR17520069.1498736
SRR17520069.1498737
SRR17520069.1498738
SRR17520069.1498739
SRR17520069.1498740
SRR17520069.1498741
SRR17520069.1498742
SRR17520069.1498743
SRR17520069.1498744
SRR17520069.1498745
SRR17520069.1498746
SRR17520069.1498747
SRR17520069.1498748
SRR17520069.1498749
SRR17520069.1498750
SRR17520069.1498751
SRR17520069.1498752
SRR17520069.1498753
SRR17520069.1498754
SRR17520069.1498755
SRR17520069.1498756
SRR17520069.1498757
SRR17520069.1498758
SRR17520069.1498759
SRR17520069.1498760
SRR17520069.1498761
SRR17520069.1498762
SRR17520069.1498763
SRR17520069.1498764
SRR17520069.1498765
SRR17520069.1498766
SRR17520069.1498767
SRR17520069.1498768
SRR17520069.1498769
SRR17520069.1498770
SRR17520069.1498771
SRR17520069.1498772
SRR17520069.1498773
SRR17520069.1498774
SRR17520069.1498775
SRR17520069.1498776
SRR17520069.1498777
SRR17520069.1498778
SRR17520069.1498785
SRR17520069.1498787

SRR17520069.1498789
SRR17520069.1498790
SRR17520069.1498794
SRR17520069.1498800
SRR17520069.1498801
SRR17520069.1498805
SRR17520069.1498813
SRR17520069.1500517
SRR17520069.1500518
SRR17520069.1518843
SRR17520069.1534026
SRR17520069.1534027
SRR17520069.1534028
SRR17520069.1534029
SRR17520069.1534030
SRR17520069.1534031
SRR17520069.1534032
SRR17520069.1534033
SRR17520069.1534034
SRR17520069.1534035
SRR17520069.1534036
SRR17520069.1534037
SRR17520069.1534038
SRR17520069.1534039
SRR17520069.1534040
SRR17520069.1534041
SRR17520069.1534042
SRR17520069.1534043
SRR17520069.1534044
SRR17520069.1534045
SRR17520069.1534046
SRR17520069.1534047
SRR17520069.1534048
SRR17520069.1534049
SRR17520069.1534050
SRR17520069.1534051
SRR17520069.1534052
SRR17520069.1534053
SRR17520069.1534054
SRR17520069.1534055
SRR17520069.1534056
SRR17520069.1534057
SRR17520069.1534058
SRR17520069.1534059
SRR17520069.1534060
SRR17520069.1534061
SRR17520069.1534062

SRR17520069.1534063
SRR17520069.1534064
SRR17520069.1534065
SRR17520069.1534066
SRR17520069.1534067
SRR17520069.1534068
SRR17520069.1534069
SRR17520069.1534070
SRR17520069.1534071
SRR17520069.1534072
SRR17520069.1534073
SRR17520069.1534074
SRR17520069.1534075
SRR17520069.1534076
SRR17520069.1534077
SRR17520069.1534078
SRR17520069.1534079
SRR17520069.1534080
SRR17520069.1534081
SRR17520069.1534082
SRR17520069.1534083
SRR17520069.1534084
SRR17520069.1534085
SRR17520069.1534086
SRR17520069.1534087
SRR17520069.1534088
SRR17520069.1534089
SRR17520069.1534090
SRR17520069.1534091
SRR17520069.1534092
SRR17520069.1534093
SRR17520069.1534094
SRR17520069.1534095
SRR17520069.1534096
SRR17520069.1534097
SRR17520069.1534098
SRR17520069.1534099
SRR17520069.1534100
SRR17520069.1534101
SRR17520069.1534102
SRR17520069.1534103
SRR17520069.1534105
SRR17520069.1534106
SRR17520069.1534107
SRR17520069.1534108
SRR17520069.1534109
SRR17520069.1534110

SRR17520069.1534111
SRR17520069.1534112
SRR17520069.1534113
SRR17520069.1534114
SRR17520069.1534115
SRR17520069.1534116
SRR17520069.1534117
SRR17520069.1534118
SRR17520069.1534119
SRR17520069.1534120
SRR17520069.1534121
SRR17520069.1534122
SRR17520069.1534123
SRR17520069.1534124
SRR17520069.1534125
SRR17520069.1534126
SRR17520069.1534127
SRR17520069.1535013
SRR17520069.1535014
SRR17520069.1535015
SRR17520069.1535016
SRR17520069.1535017
SRR17520069.1535018
SRR17520069.1535019
SRR17520069.1535020
SRR17520069.1535021
SRR17520069.1535022
SRR17520069.1535023
SRR17520069.1535024
SRR17520069.1535025
SRR17520069.1535026
SRR17520069.1535027
SRR17520069.1535028
SRR17520069.1535029
SRR17520069.1535030
SRR17520069.1535031
SRR17520069.1535033
SRR17520069.1535034
SRR17520069.1535036
SRR17520069.1535037
SRR17520069.1535038
SRR17520069.1535039
SRR17520069.1535040
SRR17520069.1535041
SRR17520069.1535042
SRR17520069.1535043
SRR17520069.1535044

SRR17520069.1535045
SRR17520069.1535046
SRR17520069.1535047
SRR17520069.1535048
SRR17520069.1535049
SRR17520069.1535050
SRR17520069.1535051
SRR17520069.1535052
SRR17520069.1535053
SRR17520069.1535054
SRR17520069.1535055
SRR17520069.1535056
SRR17520069.1535057
SRR17520069.1535058
SRR17520069.1535059
SRR17520069.1535060
SRR17520069.1535061
SRR17520069.1535062
SRR17520069.1535063
SRR17520069.1535064
SRR17520069.1535065
SRR17520069.1535066
SRR17520069.1535067
SRR17520069.1535068
SRR17520069.1535069
SRR17520069.1535070
SRR17520069.1535071
SRR17520069.1535073
SRR17520069.1535074
SRR17520069.1535075
SRR17520069.1535076
SRR17520069.1535077
SRR17520069.1535078
SRR17520069.1535079
SRR17520069.1535080
SRR17520069.1535081
SRR17520069.1535082
SRR17520069.1535083
SRR17520069.1535085
SRR17520069.1535086
SRR17520069.1535087
SRR17520069.1535088
SRR17520069.1535089
SRR17520069.1535090
SRR17520069.1535091
SRR17520069.1535092
SRR17520069.1535093

SRR17520069.1535094
SRR17520069.1535095
SRR17520069.1535096
SRR17520069.1535097
SRR17520069.1535098
SRR17520069.1535099
SRR17520069.1535100
SRR17520069.1535101
SRR17520069.1535102
SRR17520069.1535103
SRR17520069.1535104
SRR17520069.1535105
SRR17520069.1535106
SRR17520069.1535107
SRR17520069.1535108
SRR17520069.1535109
SRR17520069.1535110
SRR17520069.1535111
SRR17520069.1535112
SRR17520069.1535113
SRR17520069.1535114
SRR17520069.1535115
SRR17520069.1535116
SRR17520069.1535117
SRR17520069.1535118
SRR17520069.1535119
SRR17520069.1535120
SRR17520069.1535121
SRR17520069.1535122
SRR17520069.1535123
SRR17520069.1535773
SRR17520069.1552426
SRR17520069.1552449
SRR17520069.1554053
SRR17520069.1554055
SRR17520069.1554057
SRR17520069.1554059
SRR17520069.1554060
SRR17520069.1554061
SRR17520069.1554062
SRR17520069.1554063
SRR17520069.1554065
SRR17520069.1554066
SRR17520069.1554069
SRR17520069.1554071
SRR17520069.1554072
SRR17520069.1554073

SRR17520069.1554074
SRR17520069.1554075
SRR17520069.1554076
SRR17520069.1554077
SRR17520069.1554078
SRR17520069.1554079
SRR17520069.1554082
SRR17520069.1554085
SRR17520069.1554088
SRR17520069.1554089
SRR17520069.1554091
SRR17520069.1554092
SRR17520069.1554093
SRR17520069.1554094
SRR17520069.1554095
SRR17520069.1554096
SRR17520069.1554098
SRR17520069.1554099
SRR17520069.1554100
SRR17520069.1554101
SRR17520069.1554104
SRR17520069.1554105
SRR17520069.1554106
SRR17520069.1554107
SRR17520069.1554108
SRR17520069.1554110
SRR17520069.1554111
SRR17520069.1554112
SRR17520069.1554113
SRR17520069.1554114
SRR17520069.1554115
SRR17520069.1554116
SRR17520069.1554117
SRR17520069.1554118
SRR17520069.1554119
SRR17520069.1554120
SRR17520069.1554121
SRR17520069.1554122
SRR17520069.1554123
SRR17520069.1554124
SRR17520069.1554125
SRR17520069.1554127
SRR17520069.1554128
SRR17520069.1554129
SRR17520069.1554130
SRR17520069.1554131
SRR17520069.1554132

SRR17520069.1554133
SRR17520069.1554134
SRR17520069.1554135
SRR17520069.1554136
SRR17520069.1554137
SRR17520069.1554138
SRR17520069.1554139
SRR17520069.1554140
SRR17520069.1554141
SRR17520069.1554143
SRR17520069.1554144
SRR17520069.1554145
SRR17520069.1554146
SRR17520069.1554147
SRR17520069.1554149
SRR17520069.1554150
SRR17520069.1554151
SRR17520069.1554152
SRR17520069.1554153
SRR17520069.1554154
SRR17520069.1554155
SRR17520069.1554156
SRR17520069.1554157
SRR17520069.1554158
SRR17520069.1566133
SRR17520069.1585301
SRR17520069.1585302
SRR17520069.1585303
SRR17520069.1585304
SRR17520069.1585305
SRR17520069.1585306
SRR17520069.1585307
SRR17520069.1585308
SRR17520069.1585309
SRR17520069.1585310
SRR17520069.1585311
SRR17520069.1585312
SRR17520069.1585313
SRR17520069.1585314
SRR17520069.1585315
SRR17520069.1585316
SRR17520069.1585317
SRR17520069.1585318
SRR17520069.1585319
SRR17520069.1585320
SRR17520069.1585321
SRR17520069.1585322

SRR17520069.1585323
SRR17520069.1585324
SRR17520069.1585325
SRR17520069.1585326
SRR17520069.1585327
SRR17520069.1585328
SRR17520069.1585329
SRR17520069.1585330
SRR17520069.1585331
SRR17520069.1585332
SRR17520069.1585333
SRR17520069.1585334
SRR17520069.1585335
SRR17520069.1585336
SRR17520069.1585338
SRR17520069.1585339
SRR17520069.1585340
SRR17520069.1585341
SRR17520069.1585342
SRR17520069.1585343
SRR17520069.1585344
SRR17520069.1585345
SRR17520069.1585346
SRR17520069.1585347
SRR17520069.1585348
SRR17520069.1585349
SRR17520069.1585350
SRR17520069.1585351
SRR17520069.1585352
SRR17520069.1585353
SRR17520069.1585354
SRR17520069.1585355
SRR17520069.1585356
SRR17520069.1585357
SRR17520069.1585358
SRR17520069.1585359
SRR17520069.1585360
SRR17520069.1585361
SRR17520069.1585362
SRR17520069.1585365
SRR17520069.1585366
SRR17520069.1585367
SRR17520069.1585368
SRR17520069.1585369
SRR17520069.1585370
SRR17520069.1585371
SRR17520069.1585372

SRR17520069.1585373
SRR17520069.1585374
SRR17520069.1585375
SRR17520069.1585376
SRR17520069.1585377
SRR17520069.1585378
SRR17520069.1585379
SRR17520069.1585380
SRR17520069.1585381
SRR17520069.1585382
SRR17520069.1585383
SRR17520069.1585384
SRR17520069.1585385
SRR17520069.1585386
SRR17520069.1585387
SRR17520069.1585388
SRR17520069.1585389
SRR17520069.1585390
SRR17520069.1585391
SRR17520069.1585392
SRR17520069.1585393
SRR17520069.1585394
SRR17520069.1585395
SRR17520069.1585396
SRR17520069.1585397
SRR17520069.1585399
SRR17520069.1585400
SRR17520069.1585401
SRR17520069.1604986
SRR17520069.1604987
SRR17520069.1604988
SRR17520069.1604989
SRR17520069.1604990
SRR17520069.1604991
SRR17520069.1623653
SRR17520069.1623654
SRR17520069.1623655
SRR17520069.1623658
SRR17520069.1640566
SRR17520069.1640567
SRR17520069.1640568
SRR17520069.1640569
SRR17520069.1640570
SRR17520069.1640571
SRR17520069.1640572
SRR17520069.1640573
SRR17520069.1640574

SRR17520069.1640575
SRR17520069.1640576
SRR17520069.1640577
SRR17520069.1640578
SRR17520069.1640579
SRR17520069.1640580
SRR17520069.1640581
SRR17520069.1640582
SRR17520069.1640583
SRR17520069.1640584
SRR17520069.1640585
SRR17520069.1640726
SRR17520069.1640728
SRR17520069.1640730
SRR17520069.1640731
SRR17520069.1640732
SRR17520069.1640733
SRR17520069.1640734
SRR17520069.1640735
SRR17520069.1640736
SRR17520069.1640737
SRR17520069.1640738
SRR17520069.1640739
SRR17520069.1640740
SRR17520069.1640741
SRR17520069.1640742
SRR17520069.1640743
SRR17520069.1640744
SRR17520069.1640745
SRR17520069.1640746
SRR17520069.1640747
SRR17520069.1640748
SRR17520069.1640749
SRR17520069.1640750
SRR17520069.1640751
SRR17520069.1640752
SRR17520069.1640753
SRR17520069.1640754
SRR17520069.1640755
SRR17520069.1640756
SRR17520069.1640757
SRR17520069.1640758
SRR17520069.1640759
SRR17520069.1640760
SRR17520069.1640761
SRR17520069.1640762
SRR17520069.1640763

SRR17520069.1640765
SRR17520069.1640766
SRR17520069.1640767
SRR17520069.1640768
SRR17520069.1640769
SRR17520069.1640770
SRR17520069.1640771
SRR17520069.1640772
SRR17520069.1640773
SRR17520069.1640774
SRR17520069.1640775
SRR17520069.1640776
SRR17520069.1640777
SRR17520069.1640778
SRR17520069.1640779
SRR17520069.1640780
SRR17520069.1640781
SRR17520069.1640782
SRR17520069.1640783
SRR17520069.1640784
SRR17520069.1640785
SRR17520069.1640786
SRR17520069.1640787
SRR17520069.1640788
SRR17520069.1640789
SRR17520069.1640790
SRR17520069.1640791
SRR17520069.1642992
SRR17520069.1643032
SRR17520069.1650533
SRR17520069.1650545
SRR17520069.1650548
SRR17520069.1650561
SRR17520069.1650607
SRR17520069.1653560
SRR17520069.1653561
SRR17520069.1653562
SRR17520069.1653563
SRR17520069.1653564
SRR17520069.1653565
SRR17520069.1653566
SRR17520069.1653567
SRR17520069.1653568
SRR17520069.1653569
SRR17520069.1653570
SRR17520069.1653571
SRR17520069.1653572

SRR17520069.1653573
SRR17520069.1653574
SRR17520069.1653575
SRR17520069.1653576
SRR17520069.1653577
SRR17520069.1653578
SRR17520069.1653579
SRR17520069.1653580
SRR17520069.1653581
SRR17520069.1653582
SRR17520069.1653583
SRR17520069.1653584
SRR17520069.1653585
SRR17520069.1653586
SRR17520069.1653587
SRR17520069.1653588
SRR17520069.1653589
SRR17520069.1653590
SRR17520069.1653591
SRR17520069.1653592
SRR17520069.1653593
SRR17520069.1653594
SRR17520069.1653595
SRR17520069.1653596
SRR17520069.1653597
SRR17520069.1653598
SRR17520069.1653600
SRR17520069.1653601
SRR17520069.1653602
SRR17520069.1653603
SRR17520069.1653604
SRR17520069.1653605
SRR17520069.1653606
SRR17520069.1653607
SRR17520069.1653608
SRR17520069.1653609
SRR17520069.1653610
SRR17520069.1653611
SRR17520069.1653612
SRR17520069.1653613
SRR17520069.1653614
SRR17520069.1653615
SRR17520069.1653616
SRR17520069.1653617
SRR17520069.1653618
SRR17520069.1653619
SRR17520069.1653620

SRR17520069.1653621
SRR17520069.1653622
SRR17520069.1653623
SRR17520069.1653624
SRR17520069.1653625
SRR17520069.1653626
SRR17520069.1653628
SRR17520069.1653629
SRR17520069.1653630
SRR17520069.1653631
SRR17520069.1653633
SRR17520069.1653634
SRR17520069.1653635
SRR17520069.1653636
SRR17520069.1653637
SRR17520069.1653638
SRR17520069.1653639
SRR17520069.1653640
SRR17520069.1653641
SRR17520069.1653642
SRR17520069.1653643
SRR17520069.1653644
SRR17520069.1653645
SRR17520069.1653646
SRR17520069.1653647
SRR17520069.1653648
SRR17520069.1653649
SRR17520069.1653650
SRR17520069.1653651
SRR17520069.1653652
SRR17520069.1653653
SRR17520069.1653654
SRR17520069.1653655
SRR17520069.1653656
SRR17520069.1653657
SRR17520069.1653658
SRR17520069.1653659
SRR17520069.1653660
SRR17520069.1653662
SRR17520069.1653663
SRR17520069.1653664
SRR17520069.1653666
SRR17520069.1653667
SRR17520069.1653668
SRR17520069.1653669
SRR17520069.1653670
SRR17520069.1653671

SRR17520069.1653672
SRR17520069.1653673
SRR17520069.1653674
SRR17520069.1653675
SRR17520069.1653676
SRR17520069.1653677
SRR17520069.1653678
SRR17520069.1655607
SRR17520069.1688773
SRR17520069.1688774
SRR17520069.1688775
SRR17520069.1688776
SRR17520069.1688777
SRR17520069.1688778
SRR17520069.1688779
SRR17520069.1688780
SRR17520069.1688781
SRR17520069.1691306
SRR17520069.1691307
SRR17520069.1691308
SRR17520069.1691309
SRR17520069.1691310
SRR17520069.1691311
SRR17520069.1691312
SRR17520069.1691313
SRR17520069.1691314
SRR17520069.1691315
SRR17520069.1691316
SRR17520069.1691317
SRR17520069.1691318
SRR17520069.1691319
SRR17520069.1691320
SRR17520069.1691321
SRR17520069.1691322
SRR17520069.1691323
SRR17520069.1691324
SRR17520069.1691325
SRR17520069.1691326
SRR17520069.1691327
SRR17520069.1691328
SRR17520069.1691329
SRR17520069.1691330
SRR17520069.1691331
SRR17520069.1691332
SRR17520069.1691333
SRR17520069.1691334
SRR17520069.1709421

SRR17520069.1724962
SRR17520069.1724963
SRR17520069.1724964
SRR17520069.1724965
SRR17520069.1724966
SRR17520069.1724967
SRR17520069.1724969
SRR17520069.1724970
SRR17520069.1724971
SRR17520069.1724972
SRR17520069.1724973
SRR17520069.1724974
SRR17520069.1724975
SRR17520069.1724976
SRR17520069.1724977
SRR17520069.1724978
SRR17520069.1724979
SRR17520069.1724980
SRR17520069.1724981
SRR17520069.1724982
SRR17520069.1724983
SRR17520069.1724984
SRR17520069.1724985
SRR17520069.1724986
SRR17520069.1724987
SRR17520069.1724988
SRR17520069.1724989
SRR17520069.1724990
SRR17520069.1724991
SRR17520069.1724992
SRR17520069.1724993
SRR17520069.1724994
SRR17520069.1724995
SRR17520069.1724996
SRR17520069.1724997
SRR17520069.1724998
SRR17520069.1724999
SRR17520069.1725000
SRR17520069.1725001
SRR17520069.1725002
SRR17520069.1725003
SRR17520069.1725004
SRR17520069.1725005
SRR17520069.1725006
SRR17520069.1725007
SRR17520069.1725008
SRR17520069.1725009

SRR17520069.1725010
SRR17520069.1725011
SRR17520069.1725012
SRR17520069.1725013
SRR17520069.1725014
SRR17520069.1725015
SRR17520069.1725016
SRR17520069.1725017
SRR17520069.1725019
SRR17520069.1725020
SRR17520069.1725021
SRR17520069.1725024
SRR17520069.1725025
SRR17520069.1725026
SRR17520069.1725027
SRR17520069.1725028
SRR17520069.1725029
SRR17520069.1725030
SRR17520069.1725031
SRR17520069.1725032
SRR17520069.1725033
SRR17520069.1725034
SRR17520069.1725035
SRR17520069.1725036
SRR17520069.1725037
SRR17520069.1725038
SRR17520069.1725039
SRR17520069.1725040
SRR17520069.1729439
SRR17520069.1729440
SRR17520069.1729441
SRR17520069.1729442
SRR17520069.1729444
SRR17520069.1744579
SRR17520069.1744580
SRR17520069.1744581
SRR17520069.1744582
SRR17520069.1744583
SRR17520069.1744584
SRR17520069.1751442
SRR17520069.1751443
SRR17520069.1751444
SRR17520069.1751445
SRR17520069.1751446
SRR17520069.1751447
SRR17520069.1751448
SRR17520069.1751449

SRR17520069.1751450
SRR17520069.1751451
SRR17520069.1751452
SRR17520069.1751453
SRR17520069.1751454
SRR17520069.1751455
SRR17520069.1751456
SRR17520069.1751457
SRR17520069.1751458
SRR17520069.1751459
SRR17520069.1751460
SRR17520069.1751462
SRR17520069.1751463
SRR17520069.1751464
SRR17520069.1751465
SRR17520069.1751466
SRR17520069.1751467
SRR17520069.1751468
SRR17520069.1751469
SRR17520069.1751470
SRR17520069.1751471
SRR17520069.1751472
SRR17520069.1751473
SRR17520069.1751474
SRR17520069.1751475
SRR17520069.1751476
SRR17520069.1751477
SRR17520069.1751478
SRR17520069.1751479
SRR17520069.1751480
SRR17520069.1751481
SRR17520069.1751482
SRR17520069.1751483
SRR17520069.1751484
SRR17520069.1751485
SRR17520069.1751486
SRR17520069.1751487
SRR17520069.1751488
SRR17520069.1751489
SRR17520069.1751490
SRR17520069.1751491
SRR17520069.1751492
SRR17520069.1751493
SRR17520069.1751494
SRR17520069.1751495
SRR17520069.1751496
SRR17520069.1751497

SRR17520069.1751498
SRR17520069.1751499
SRR17520069.1751500
SRR17520069.1751501
SRR17520069.1751502
SRR17520069.1751503
SRR17520069.1751504
SRR17520069.1763952
SRR17520069.1763953
SRR17520069.1763954
SRR17520069.1763955
SRR17520069.1763956
SRR17520069.1763957
SRR17520069.1763958
SRR17520069.1763959
SRR17520069.1763960
SRR17520069.1763961
SRR17520069.1763962
SRR17520069.1763963
SRR17520069.1763964
SRR17520069.1763965
SRR17520069.1763966
SRR17520069.1763967
SRR17520069.1769661
SRR17520069.1771801
SRR17520069.1805330
SRR17520069.1807240
SRR17520069.1807241
SRR17520069.1807242
SRR17520069.1807243
SRR17520069.1807244
SRR17520069.1807245
SRR17520069.1807246
SRR17520069.1807247
SRR17520069.1807248
SRR17520069.1807249
SRR17520069.1807250
SRR17520069.1807251
SRR17520069.1807252
SRR17520069.1807253
SRR17520069.1807254
SRR17520069.1807255
SRR17520069.1807256
SRR17520069.1807257
SRR17520069.1807258
SRR17520069.1807259
SRR17520069.1807260

SRR17520069.1807261
SRR17520069.1807262
SRR17520069.1807263
SRR17520069.1807264
SRR17520069.1807265
SRR17520069.1807266
SRR17520069.1807267
SRR17520069.1807268
SRR17520069.1807269
SRR17520069.1807270
SRR17520069.1807271
SRR17520069.1807272
SRR17520069.1807273
SRR17520069.1807274
SRR17520069.1807276
SRR17520069.1807277
SRR17520069.1807278
SRR17520069.1807279
SRR17520069.1807280
SRR17520069.1807281
SRR17520069.1807282
SRR17520069.1807283
SRR17520069.1807284
SRR17520069.1807285
SRR17520069.1807286
SRR17520069.1807287
SRR17520069.1807288
SRR17520069.1807289
SRR17520069.1807290
SRR17520069.1807291
SRR17520069.1807292
SRR17520069.1807294
SRR17520069.1807295
SRR17520069.1807296
SRR17520069.1807297
SRR17520069.1807298
SRR17520069.1807299
SRR17520069.1807300
SRR17520069.1807301
SRR17520069.1807302
SRR17520069.1807303
SRR17520069.1807304
SRR17520069.1807305
SRR17520069.1807306
SRR17520069.1807307
SRR17520069.1807308
SRR17520069.1807309

SRR17520069.1807310
SRR17520069.1807311
SRR17520069.1807312
SRR17520069.1807313
SRR17520069.1807314
SRR17520069.1807315
SRR17520069.1807316
SRR17520069.1807317
SRR17520069.1807318
SRR17520069.1807319
SRR17520069.1807320
SRR17520069.1807321
SRR17520069.1807322
SRR17520069.1807323
SRR17520069.1807324
SRR17520069.1807325
SRR17520069.1807326
SRR17520069.1807327
SRR17520069.1807328
SRR17520069.1807329
SRR17520069.1807330
SRR17520069.1807331
SRR17520069.1807332
SRR17520069.1818443
SRR17520069.1818444
SRR17520069.1818445
SRR17520069.1818446
SRR17520069.1818447
SRR17520069.1818448
SRR17520069.1818450
SRR17520069.1818451
SRR17520069.1818452
SRR17520069.1818453
SRR17520069.1818454
SRR17520069.1818455
SRR17520069.1818456
SRR17520069.1818457
SRR17520069.1818458
SRR17520069.1818459
SRR17520069.1818460
SRR17520069.1818461
SRR17520069.1818462
SRR17520069.1818463
SRR17520069.1818464
SRR17520069.1818465
SRR17520069.1818466
SRR17520069.1818467

SRR17520069.1848544
SRR17520069.1848546
SRR17520069.1860780
SRR17520069.1860832
SRR17520069.1860833
SRR17520069.1860834
SRR17520069.1860835
SRR17520069.1860836
SRR17520069.1860837
SRR17520069.1860838
SRR17520069.1860839
SRR17520069.1860840
SRR17520069.1866413
SRR17520069.1866414
SRR17520069.1866915
SRR17520069.1866916
SRR17520069.1866918
SRR17520069.1866919
SRR17520069.1866920
SRR17520069.1881310
SRR17520069.1881364
SRR17520069.1892109
SRR17520069.1894007
SRR17520069.1894009
SRR17520069.1894011
SRR17520069.1894012
SRR17520069.1894013
SRR17520069.1894015
SRR17520069.1901870
SRR17520069.1901871
SRR17520069.1901872
SRR17520069.1901873
SRR17520069.1901874
SRR17520069.1901875
SRR17520069.1901876
SRR17520069.1901877
SRR17520069.1904432
SRR17520069.1904433
SRR17520069.1904434
SRR17520069.1904435
SRR17520069.1904436
SRR17520069.1904437
SRR17520069.1904438
SRR17520069.1904439
SRR17520069.1904440
SRR17520069.1904441
SRR17520069.1904442

SRR17520069.1904443
SRR17520069.1904444
SRR17520069.1904445
SRR17520069.1904446
SRR17520069.1904447
SRR17520069.1904448
SRR17520069.1904449
SRR17520069.1904450
SRR17520069.1904451
SRR17520069.1904452
SRR17520069.1904453
SRR17520069.1904454
SRR17520069.1904455
SRR17520069.1904456
SRR17520069.1904457
SRR17520069.1904458
SRR17520069.1904459
SRR17520069.1904460
SRR17520069.1904461
SRR17520069.1904462
SRR17520069.1904463
SRR17520069.1904464
SRR17520069.1904465
SRR17520069.1904466
SRR17520069.1904467
SRR17520069.1904468
SRR17520069.1904469
SRR17520069.1904470
SRR17520069.1904471
SRR17520069.1904472
SRR17520069.1904474
SRR17520069.1904475
SRR17520069.1904476
SRR17520069.1904478
SRR17520069.1904479
SRR17520069.1904480
SRR17520069.1904481
SRR17520069.1904482
SRR17520069.1904483
SRR17520069.1904484
SRR17520069.1904485
SRR17520069.1904486
SRR17520069.1904487
SRR17520069.1904488
SRR17520069.1904489
SRR17520069.1904490
SRR17520069.1904491

SRR17520069.1904492
SRR17520069.1904493
SRR17520069.1904494
SRR17520069.1904495
SRR17520069.1904496
SRR17520069.1904497
SRR17520069.1904498
SRR17520069.1904499
SRR17520069.1904500
SRR17520069.1904501
SRR17520069.1904502
SRR17520069.1904503
SRR17520069.1904504
SRR17520069.1904505
SRR17520069.1904506
SRR17520069.1904507
SRR17520069.1904508
SRR17520069.1904509
SRR17520069.1904510
SRR17520069.1904511
SRR17520069.1904512
SRR17520069.1904513
SRR17520069.1904514
SRR17520069.1904515
SRR17520069.1904516
SRR17520069.1904517
SRR17520069.1904518
SRR17520069.1904519
SRR17520069.1904520
SRR17520069.1904521
SRR17520069.1904522
SRR17520069.1904523
SRR17520069.1904524
SRR17520069.1904525
SRR17520069.1904526
SRR17520069.1904527
SRR17520069.1904528
SRR17520069.1904529
SRR17520069.1904530
SRR17520069.1904531
SRR17520069.1910044
SRR17520069.1921923
SRR17520069.1921924
SRR17520069.1921925
SRR17520069.1921926
SRR17520069.1921928
SRR17520069.1921929

SRR17520069.1921930
SRR17520069.1921931
SRR17520069.1921932
SRR17520069.1921933
SRR17520069.1921934
SRR17520069.1921935
SRR17520069.1921936
SRR17520069.1921937
SRR17520069.1921939
SRR17520069.1921940
SRR17520069.1921941
SRR17520069.1921942
SRR17520069.1921943
SRR17520069.1921944
SRR17520069.1942009
SRR17520069.1969824
SRR17520069.1969825
SRR17520069.1969826
SRR17520069.1969827
SRR17520069.1969828
SRR17520069.1969829
SRR17520069.1969830
SRR17520069.1969831
SRR17520069.1969832
SRR17520069.1969833
SRR17520069.1969834
SRR17520069.1969835
SRR17520069.1969836
SRR17520069.1969837
SRR17520069.1969838
SRR17520069.1969839
SRR17520069.1969840
SRR17520069.1969841
SRR17520069.1969842
SRR17520069.1969843
SRR17520069.1969844
SRR17520069.1969845
SRR17520069.1969846
SRR17520069.1969847
SRR17520069.1969850
SRR17520069.1969851
SRR17520069.1969852
SRR17520069.1969853
SRR17520069.1969854
SRR17520069.1969855
SRR17520069.1969856
SRR17520069.1969857

SRR17520069.1969858
SRR17520069.1969859
SRR17520069.1969860
SRR17520069.1969861
SRR17520069.1969862
SRR17520069.1969863
SRR17520069.1969864
SRR17520069.1969865
SRR17520069.1969866
SRR17520069.1969867
SRR17520069.1969868
SRR17520069.1971243
SRR17520069.1971244
SRR17520069.1971246
SRR17520069.1971248
SRR17520069.1971249
SRR17520069.1971250
SRR17520069.1971251
SRR17520069.1971252
SRR17520069.1971253
SRR17520069.1971254
SRR17520069.1971255
SRR17520069.1971256
SRR17520069.1971257
SRR17520069.1971258
SRR17520069.1971259
SRR17520069.1971260
SRR17520069.1971261
SRR17520069.1971262
SRR17520069.1971264
SRR17520069.1971265
SRR17520069.1971266
SRR17520069.1971267
SRR17520069.1971268
SRR17520069.1971269
SRR17520069.1971270
SRR17520069.1971271
SRR17520069.1971272
SRR17520069.1971273
SRR17520069.1971274
SRR17520069.1971275
SRR17520069.1971276
SRR17520069.1971277
SRR17520069.1971279
SRR17520069.1971281
SRR17520069.1971282
SRR17520069.1971283

SRR17520069.1971284
SRR17520069.1971285
SRR17520069.1971286
SRR17520069.1971287
SRR17520069.1971288
SRR17520069.1971289
SRR17520069.1971290
SRR17520069.1971291
SRR17520069.1971292
SRR17520069.1971293
SRR17520069.1971294
SRR17520069.1971295
SRR17520069.1971296
SRR17520069.1971297
SRR17520069.1971298
SRR17520069.1971299
SRR17520069.1971300
SRR17520069.1971301
SRR17520069.1971302
SRR17520069.1971303
SRR17520069.1971304
SRR17520069.1971305
SRR17520069.1971306
SRR17520069.1971307
SRR17520069.1971308
SRR17520069.1971309
SRR17520069.1971310
SRR17520069.1971311
SRR17520069.1971313
SRR17520069.1971314
SRR17520069.1971315
SRR17520069.1971316
SRR17520069.1971317
SRR17520069.1971318
SRR17520069.1971319
SRR17520069.1971320
SRR17520069.1971321
SRR17520069.1971322
SRR17520069.1971323
SRR17520069.1971324
SRR17520069.1971325
SRR17520069.1971326
SRR17520069.1971327
SRR17520069.1971330
SRR17520069.1971331
SRR17520069.1971332
SRR17520069.1971333

SRR17520069.1971334
SRR17520069.1971335
SRR17520069.1971336
SRR17520069.1971337
SRR17520069.1971338
SRR17520069.1971339
SRR17520069.1971340
SRR17520069.1971341
SRR17520069.1971342
SRR17520069.1971343
SRR17520069.1971344
SRR17520069.1971345
SRR17520069.1971346
SRR17520069.1971347
SRR17520069.1971348
SRR17520069.1971349
SRR17520069.1971350
SRR17520069.1971351
SRR17520069.1971352
SRR17520069.1971353
SRR17520069.1971354
SRR17520069.1971355
SRR17520069.1971356
SRR17520069.1971357
SRR17520069.1971359
SRR17520069.1971360
SRR17520069.1971361
SRR17520069.1971362
SRR17520069.1971363
SRR17520069.1971364
SRR17520069.1971365
SRR17520069.1971366
SRR17520069.1971367
SRR17520069.1971368
SRR17520069.1971369
SRR17520069.1971370
SRR17520069.1971371
SRR17520069.1971372
SRR17520069.1971373
SRR17520069.1971374
SRR17520069.1971375
SRR17520069.1971376
SRR17520069.1971377
SRR17520069.1971378
SRR17520069.1971379
SRR17520069.1971380
SRR17520069.1971381

SRR17520069.1971382
SRR17520069.1971383
SRR17520069.1971384
SRR17520069.1971385
SRR17520069.1971386
SRR17520069.1971387
SRR17520069.1971388
SRR17520069.1971389
SRR17520069.1971390
SRR17520069.1971391
SRR17520069.1971392
SRR17520069.1971393
SRR17520069.1971394
SRR17520069.1971395
SRR17520069.1971396
SRR17520069.1971397
SRR17520069.1971398
SRR17520069.1971399
SRR17520069.1971400
SRR17520069.1971401
SRR17520069.1971402
SRR17520069.1971403
SRR17520069.1971404
SRR17520069.1971405
SRR17520069.1971406
SRR17520069.1971407
SRR17520069.1971408
SRR17520069.1971409
SRR17520069.1971410
SRR17520069.1971411
SRR17520069.1971412
SRR17520069.1971413
SRR17520069.1971414
SRR17520069.1971415
SRR17520069.1971416
SRR17520069.1971417
SRR17520069.1971418
SRR17520069.1971419
SRR17520069.1971420
SRR17520069.1971421
SRR17520069.1971422
SRR17520069.1971423
SRR17520069.1971424
SRR17520069.1971425
SRR17520069.1971426
SRR17520069.1976502
SRR17520069.1976504

SRR17520069.1976508
SRR17520069.1976509
SRR17520069.1976519
SRR17520069.1976520
SRR17520069.1976522
SRR17520069.1976524
SRR17520069.1976526
SRR17520069.1977486
SRR17520069.1977487
SRR17520069.1977488
SRR17520069.1977489
SRR17520069.1977490
SRR17520069.1977491
SRR17520069.1977492
SRR17520069.1977493
SRR17520069.1977494
SRR17520069.1977495
SRR17520069.1977496
SRR17520069.1977497
SRR17520069.1977498
SRR17520069.1977499
SRR17520069.1977500
SRR17520069.1977501
SRR17520069.1977502
SRR17520069.1977503
SRR17520069.1977504
SRR17520069.1977505
SRR17520069.1977506
SRR17520069.1977507
SRR17520069.1977508
SRR17520069.1977509
SRR17520069.1977510
SRR17520069.1977511
SRR17520069.1977512
SRR17520069.1977513
SRR17520069.1977514
SRR17520069.1977515
SRR17520069.1977516
SRR17520069.1977517
SRR17520069.1977518
SRR17520069.1977519
SRR17520069.1977520
SRR17520069.1977521
SRR17520069.1977522
SRR17520069.1977523
SRR17520069.1977524
SRR17520069.1977526

SRR17520069.1977527
SRR17520069.1977528
SRR17520069.1977529
SRR17520069.1977530
SRR17520069.1977531
SRR17520069.1977532
SRR17520069.1977533
SRR17520069.1977534
SRR17520069.1977535
SRR17520069.1977536
SRR17520069.1977537
SRR17520069.1977538
SRR17520069.1977540
SRR17520069.1977541
SRR17520069.1977542
SRR17520069.1977543
SRR17520069.1977544
SRR17520069.1977545
SRR17520069.1977546
SRR17520069.1977547
SRR17520069.1977548
SRR17520069.1977549
SRR17520069.1978655
SRR17520069.1978671
SRR17520069.1978676
SRR17520069.1978686
SRR17520069.1980877
SRR17520069.1980884
SRR17520069.1980995
SRR17520069.1980996
SRR17520069.1983168
SRR17520069.1985078
SRR17520069.1990806
SRR17520069.1990807
SRR17520069.1990808
SRR17520069.1990809
SRR17520069.1990810
SRR17520069.1990811
SRR17520069.1990812
SRR17520069.1990813
SRR17520069.1990814
SRR17520069.1990815
SRR17520069.1990816
SRR17520069.1990817
SRR17520069.1990818
SRR17520069.1990819
SRR17520069.1990820

SRR17520069.1990821
SRR17520069.1990822
SRR17520069.1990823
SRR17520069.1990824
SRR17520069.1990825
SRR17520069.1990826
SRR17520069.1990827
SRR17520069.1990828
SRR17520069.1990829
SRR17520069.1990830
SRR17520069.1990831
SRR17520069.1990832
SRR17520069.1990833
SRR17520069.1990834
SRR17520069.1990835
SRR17520069.1990836
SRR17520069.1990837
SRR17520069.1990838
SRR17520069.1990839
SRR17520069.1990840
SRR17520069.1990841
SRR17520069.2016963
SRR17520069.2034250
SRR17520069.2039054
SRR17520069.2039055
SRR17520069.2039056
SRR17520069.2039057
SRR17520069.2039058
SRR17520069.2039059
SRR17520069.2039060
SRR17520069.2039061
SRR17520069.2039062
SRR17520069.2039063
SRR17520069.2039064
SRR17520069.2039066
SRR17520069.2039068
SRR17520069.2039069
SRR17520069.2039070
SRR17520069.2039071
SRR17520069.2039072
SRR17520069.2039073
SRR17520069.2039074
SRR17520069.2039076
SRR17520069.2039077
SRR17520069.2039078
SRR17520069.2039080
SRR17520069.2039081

SRR17520069.2039082
SRR17520069.2039084
SRR17520069.2039086
SRR17520069.2039087
SRR17520069.2039088
SRR17520069.2039089
SRR17520069.2039090
SRR17520069.2039092
SRR17520069.2039094
SRR17520069.2039095
SRR17520069.2039096
SRR17520069.2039097
SRR17520069.2039098
SRR17520069.2039099
SRR17520069.2039100
SRR17520069.2039101
SRR17520069.2039102
SRR17520069.2039103
SRR17520069.2039104
SRR17520069.2039105
SRR17520069.2039106
SRR17520069.2039107
SRR17520069.2039108
SRR17520069.2039109
SRR17520069.2039110
SRR17520069.2039111
SRR17520069.2039112
SRR17520069.2039113
SRR17520069.2039114
SRR17520069.2039115
SRR17520069.2039116
SRR17520069.2039117
SRR17520069.2039118
SRR17520069.2039119
SRR17520069.2039120
SRR17520069.2039121
SRR17520069.2039122
SRR17520069.2039124
SRR17520069.2039125
SRR17520069.2039126
SRR17520069.2039127
SRR17520069.2039128
SRR17520069.2039130
SRR17520069.2039132
SRR17520069.2039133
SRR17520069.2039134
SRR17520069.2039135

SRR17520069.2039136
SRR17520069.2039137
SRR17520069.2039138
SRR17520069.2039141
SRR17520069.2039142
SRR17520069.2039143
SRR17520069.2039144
SRR17520069.2039145
SRR17520069.2039146
SRR17520069.2039147
SRR17520069.2039148
SRR17520069.2039149
SRR17520069.2039150
SRR17520069.2039151
SRR17520069.2039152
SRR17520069.2039154
SRR17520069.2039156
SRR17520069.2039157
SRR17520069.2039158
SRR17520069.2039159
SRR17520069.2039160
SRR17520069.2039161
SRR17520069.2039162
SRR17520069.2039164
SRR17520069.2039165
SRR17520069.2039166
SRR17520069.2039168
SRR17520069.2039169
SRR17520069.2039170
SRR17520069.2039171
SRR17520069.2039172
SRR17520069.2039173
SRR17520069.2039174
SRR17520069.2039175
SRR17520069.2039176
SRR17520069.2039178
SRR17520069.2039179
SRR17520069.2039180
SRR17520069.2039182
SRR17520069.2039184
SRR17520069.2039185
SRR17520069.2039186
SRR17520069.2039188
SRR17520069.2039190
SRR17520069.2039191
SRR17520069.2039192
SRR17520069.2039193

SRR17520069.2039194
SRR17520069.2039195
SRR17520069.2039196
SRR17520069.2039198
SRR17520069.2039199
SRR17520069.2039200
SRR17520069.2039201
SRR17520069.2039202
SRR17520069.2039203
SRR17520069.2045029
SRR17520069.2050596
SRR17520069.2050597
SRR17520069.2050598
SRR17520069.2050599
SRR17520069.2050600
SRR17520069.2050601
SRR17520069.2050602
SRR17520069.2050603
SRR17520069.2050604
SRR17520069.2050605
SRR17520069.2050606
SRR17520069.2050607
SRR17520069.2050608
SRR17520069.2050609
SRR17520069.2050610
SRR17520069.2050611
SRR17520069.2050612
SRR17520069.2050613
SRR17520069.2050614
SRR17520069.2050615
SRR17520069.2050616
SRR17520069.2050617
SRR17520069.2050618
SRR17520069.2050619
SRR17520069.2050620
SRR17520069.2050621
SRR17520069.2050622
SRR17520069.2050623
SRR17520069.2050624
SRR17520069.2050625
SRR17520069.2050626
SRR17520069.2050627
SRR17520069.2050628
SRR17520069.2050629
SRR17520069.2050630
SRR17520069.2050631
SRR17520069.2050632

SRR17520069.2050633
SRR17520069.2050634
SRR17520069.2050635
SRR17520069.2050636
SRR17520069.2050637
SRR17520069.2050638
SRR17520069.2050639
SRR17520069.2050640
SRR17520069.2050641
SRR17520069.2050642
SRR17520069.2050643
SRR17520069.2050644
SRR17520069.2050645
SRR17520069.2050646
SRR17520069.2050647
SRR17520069.2050648
SRR17520069.2050649
SRR17520069.2050650
SRR17520069.2050651
SRR17520069.2050652
SRR17520069.2050653
SRR17520069.2050654
SRR17520069.2050655
SRR17520069.2050656
SRR17520069.2050657
SRR17520069.2050658
SRR17520069.2050659
SRR17520069.2050660
SRR17520069.2050661
SRR17520069.2050664
SRR17520069.2050665
SRR17520069.2052331
SRR17520069.2076564
SRR17520069.2076566
SRR17520069.2076685
SRR17520069.2106128
SRR17520069.2114388
SRR17520069.2114389
SRR17520069.2114390
SRR17520069.2114391
SRR17520069.2114392
SRR17520069.2114394
SRR17520069.2114395
SRR17520069.2114396
SRR17520069.2114397
SRR17520069.2114398
SRR17520069.2114399

SRR17520069.2114400
SRR17520069.2114401
SRR17520069.2114402
SRR17520069.2114403
SRR17520069.2114404
SRR17520069.2114405
SRR17520069.2114406
SRR17520069.2114407
SRR17520069.2114408
SRR17520069.2114409
SRR17520069.2114410
SRR17520069.2114411
SRR17520069.2114412
SRR17520069.2114413
SRR17520069.2134993
SRR17520069.2134994
SRR17520069.2134995
SRR17520069.2134996
SRR17520069.2134998
SRR17520069.2134999
SRR17520069.2135000
SRR17520069.2135001
SRR17520069.2135002
SRR17520069.2135003
SRR17520069.2135004
SRR17520069.2135005
SRR17520069.2135006
SRR17520069.2135007
SRR17520069.2135008
SRR17520069.2135009
SRR17520069.2135010
SRR17520069.2135011
SRR17520069.2135012
SRR17520069.2135013
SRR17520069.2135014
SRR17520069.2135015
SRR17520069.2135016
SRR17520069.2135017
SRR17520069.2135018
SRR17520069.2135019
SRR17520069.2135020
SRR17520069.2135021
SRR17520069.2135022
SRR17520069.2135023
SRR17520069.2135024
SRR17520069.2135025
SRR17520069.2135026

SRR17520069.2135027
SRR17520069.2135028
SRR17520069.2135029
SRR17520069.2135030
SRR17520069.2135031
SRR17520069.2135032
SRR17520069.2135033
SRR17520069.2135034
SRR17520069.2135035
SRR17520069.2135036
SRR17520069.2135037
SRR17520069.2135038
SRR17520069.2135039
SRR17520069.2135040
SRR17520069.2135041
SRR17520069.2135042
SRR17520069.2135043
SRR17520069.2135044
SRR17520069.2135045
SRR17520069.2135046
SRR17520069.2135047
SRR17520069.2135048
SRR17520069.2135049
SRR17520069.2135050
SRR17520069.2135051
SRR17520069.2135052
SRR17520069.2135053
SRR17520069.2135054
SRR17520069.2135055
SRR17520069.2135056
SRR17520069.2135057
SRR17520069.2135058
SRR17520069.2135059
SRR17520069.2135060
SRR17520069.2135063
SRR17520069.2135064
SRR17520069.2135065
SRR17520069.2135066
SRR17520069.2135067
SRR17520069.2135068
SRR17520069.2135069
SRR17520069.2135070
SRR17520069.2135071
SRR17520069.2135072
SRR17520069.2135073
SRR17520069.2135074
SRR17520069.2135075

SRR17520069.2135076
SRR17520069.2135077
SRR17520069.2135078
SRR17520069.2135080
SRR17520069.2135081
SRR17520069.2138509
SRR17520069.2142413
SRR17520069.2142414
SRR17520069.2142415
SRR17520069.2142416
SRR17520069.2142417
SRR17520069.2142418
SRR17520069.2142419
SRR17520069.2142420
SRR17520069.2142421
SRR17520069.2142422
SRR17520069.2142423
SRR17520069.2142424
SRR17520069.2142425
SRR17520069.2142426
SRR17520069.2142427
SRR17520069.2142428
SRR17520069.2142429
SRR17520069.2142430
SRR17520069.2142431
SRR17520069.2142432
SRR17520069.2142433
SRR17520069.2142434
SRR17520069.2142435
SRR17520069.2142436
SRR17520069.2142437
SRR17520069.2142438
SRR17520069.2142439
SRR17520069.2142440
SRR17520069.2142441
SRR17520069.2142442
SRR17520069.2142443
SRR17520069.2142909
SRR17520069.2147789
SRR17520069.2147790
SRR17520069.2147791
SRR17520069.2147792
SRR17520069.2147793
SRR17520069.2147794
SRR17520069.2147795
SRR17520069.2147796
SRR17520069.2147797

SRR17520069.2147798
SRR17520069.2147799
SRR17520069.2147800
SRR17520069.2147801
SRR17520069.2147802
SRR17520069.2147803
SRR17520069.2148919
SRR17520069.2158264
SRR17520069.2158265
SRR17520069.2158266
SRR17520069.2158267
SRR17520069.2158268
SRR17520069.2158269
SRR17520069.2158270
SRR17520069.2158271
SRR17520069.2158272
SRR17520069.2158273
SRR17520069.2158274
SRR17520069.2158275
SRR17520069.2158276
SRR17520069.2158277
SRR17520069.2158278
SRR17520069.2158279
SRR17520069.2158280
SRR17520069.2158281
SRR17520069.2158282
SRR17520069.2158283
SRR17520069.2158284
SRR17520069.2158285
SRR17520069.2158287
SRR17520069.2158288
SRR17520069.2158289
SRR17520069.2158290
SRR17520069.2158291
SRR17520069.2158292
SRR17520069.2158293
SRR17520069.2158294
SRR17520069.2158295
SRR17520069.2158296
SRR17520069.2158297
SRR17520069.2158299
SRR17520069.2158300
SRR17520069.2158301
SRR17520069.2158302
SRR17520069.2158303
SRR17520069.2158304
SRR17520069.2158305

SRR17520069.2158306
SRR17520069.2158307
SRR17520069.2158308
SRR17520069.2158309
SRR17520069.2158310
SRR17520069.2158311
SRR17520069.2158312
SRR17520069.2158313
SRR17520069.2158314
SRR17520069.2158315
SRR17520069.2158316
SRR17520069.2158317
SRR17520069.2158318
SRR17520069.2158319
SRR17520069.2158320
SRR17520069.2170288
SRR17520069.2170289
SRR17520069.2170290
SRR17520069.2170291
SRR17520069.2170292
SRR17520069.2170293
SRR17520069.2170294
SRR17520069.2170295
SRR17520069.2170296
SRR17520069.2170297
SRR17520069.2170298
SRR17520069.2170299
SRR17520069.2170301
SRR17520069.2170302
SRR17520069.2170303
SRR17520069.2170304
SRR17520069.2170305
SRR17520069.2170306
SRR17520069.2170307
SRR17520069.2170308
SRR17520069.2170309
SRR17520069.2170310
SRR17520069.2170311
SRR17520069.2170312
SRR17520069.2170313
SRR17520069.2170314
SRR17520069.2170315
SRR17520069.2170316
SRR17520069.2170317
SRR17520069.2170318
SRR17520069.2170319
SRR17520069.2170320

SRR17520069.2170321
SRR17520069.2170322
SRR17520069.2170323
SRR17520069.2170324
SRR17520069.2170325
SRR17520069.2170326
SRR17520069.2170327
SRR17520069.2170328
SRR17520069.2170329
SRR17520069.2170330
SRR17520069.2170331
SRR17520069.2170332
SRR17520069.2170334
SRR17520069.2170335
SRR17520069.2170336
SRR17520069.2170337
SRR17520069.2170338
SRR17520069.2170339
SRR17520069.2170340
SRR17520069.2170341
SRR17520069.2170342
SRR17520069.2170343
SRR17520069.2170344
SRR17520069.2170345
SRR17520069.2170346
SRR17520069.2170347
SRR17520069.2170348
SRR17520069.2170349
SRR17520069.2170350
SRR17520069.2170351
SRR17520069.2170352
SRR17520069.2170353
SRR17520069.2170354
SRR17520069.2170355
SRR17520069.2170356
SRR17520069.2170357
SRR17520069.2170358
SRR17520069.2170359
SRR17520069.2170360
SRR17520069.2170361
SRR17520069.2170362
SRR17520069.2170363
SRR17520069.2170364
SRR17520069.2170365
SRR17520069.2170366
SRR17520069.2170367
SRR17520069.2170368

SRR17520069.2170369
SRR17520069.2170370
SRR17520069.2170371
SRR17520069.2170372
SRR17520069.2170373
SRR17520069.2170374
SRR17520069.2170375
SRR17520069.2170376
SRR17520069.2170377
SRR17520069.2170378
SRR17520069.2170379
SRR17520069.2170380
SRR17520069.2170381
SRR17520069.2170382
SRR17520069.2170383
SRR17520069.2170384
SRR17520069.2170385
SRR17520069.2170386
SRR17520069.2170387
SRR17520069.2170388
SRR17520069.2170389
SRR17520069.2170390
SRR17520069.2170391
SRR17520069.2170392
SRR17520069.2170393
SRR17520069.2170394
SRR17520069.2170395
SRR17520069.2170396
SRR17520069.2170397
SRR17520069.2170398
SRR17520069.2170399
SRR17520069.2170400
SRR17520069.2170401
SRR17520069.2170402
SRR17520069.2170403
SRR17520069.2170404
SRR17520069.2170405
SRR17520069.2170406
SRR17520069.2170407
SRR17520069.2170408
SRR17520069.2170409
SRR17520069.2170410
SRR17520069.2170411
SRR17520069.2170412
SRR17520069.2170413
SRR17520069.2170414
SRR17520069.2170415

SRR17520069.2170416
SRR17520069.2170417
SRR17520069.2170418
SRR17520069.2170419
SRR17520069.2170420
SRR17520069.2170421
SRR17520069.2170422
SRR17520069.2170423
SRR17520069.2170424
SRR17520069.2170425
SRR17520069.2170426
SRR17520069.2170427
SRR17520069.2170428
SRR17520069.2170429
SRR17520069.2170430
SRR17520069.2170431
SRR17520069.2170432
SRR17520069.2170433
SRR17520069.2170434
SRR17520069.2170435
SRR17520069.2170436
SRR17520069.2170437
SRR17520069.2170438
SRR17520069.2170439
SRR17520069.2170440
SRR17520069.2170441
SRR17520069.2170442
SRR17520069.2170443
SRR17520069.2170444
SRR17520069.2170445
SRR17520069.2170446
SRR17520069.2170447
SRR17520069.2170448
SRR17520069.2170450
SRR17520069.2170451
SRR17520069.2170452
SRR17520069.2170453
SRR17520069.2170454
SRR17520069.2170455
SRR17520069.2170456
SRR17520069.2170457
SRR17520069.2170458
SRR17520069.2170459
SRR17520069.2170460
SRR17520069.2170461
SRR17520069.2170462
SRR17520069.2170463

SRR17520069.2170464
SRR17520069.2170465
SRR17520069.2170466
SRR17520069.2170467
SRR17520069.2170468
SRR17520069.2170469
SRR17520069.2170470
SRR17520069.2170471
SRR17520069.2170472
SRR17520069.2170473
SRR17520069.2170474
SRR17520069.2170475
SRR17520069.2170476
SRR17520069.2170477
SRR17520069.2170478
SRR17520069.2170479
SRR17520069.2170480
SRR17520069.2170481
SRR17520069.2170482
SRR17520069.2170483
SRR17520069.2170484
SRR17520069.2170485
SRR17520069.2170486
SRR17520069.2170487
SRR17520069.2170488
SRR17520069.2170489
SRR17520069.2170490
SRR17520069.2184610
SRR17520069.2184611
SRR17520069.2184612
SRR17520069.2184613
SRR17520069.2184614
SRR17520069.2184615
SRR17520069.2184616
SRR17520069.2184617
SRR17520069.2184618
SRR17520069.2184619
SRR17520069.2184620
SRR17520069.2184621
SRR17520069.2184622
SRR17520069.2184623
SRR17520069.2184624
SRR17520069.2184625
SRR17520069.2184626
SRR17520069.2184627
SRR17520069.2184628
SRR17520069.2184629

SRR17520069.2184630
SRR17520069.2184631
SRR17520069.2184632
SRR17520069.2184633
SRR17520069.2184634
SRR17520069.2184635
SRR17520069.2184636
SRR17520069.2202463
SRR17520069.2202464
SRR17520069.2202465
SRR17520069.2202466
SRR17520069.2202467
SRR17520069.2202469
SRR17520069.2202470
SRR17520069.2202471
SRR17520069.2202472
SRR17520069.2202473
SRR17520069.2202474
SRR17520069.2202475
SRR17520069.2202476
SRR17520069.2202477
SRR17520069.2202478
SRR17520069.2202480
SRR17520069.2202481
SRR17520069.2202482
SRR17520069.2202483
SRR17520069.2202484
SRR17520069.2202485
SRR17520069.2202486
SRR17520069.2202487
SRR17520069.2202488
SRR17520069.2202489
SRR17520069.2202490
SRR17520069.2202491
SRR17520069.2202492
SRR17520069.2202493
SRR17520069.2202494
SRR17520069.2202495
SRR17520069.2202496
SRR17520069.2202497
SRR17520069.2202498
SRR17520069.2202499
SRR17520069.2202500
SRR17520069.2202501
SRR17520069.2202502
SRR17520069.2202503
SRR17520069.2202504

SRR17520069.2202505
SRR17520069.2202506
SRR17520069.2202507
SRR17520069.2202508
SRR17520069.2202509
SRR17520069.2202510
SRR17520069.2202511
SRR17520069.2202512
SRR17520069.2202513
SRR17520069.2202514
SRR17520069.2202515
SRR17520069.2202516
SRR17520069.2202517
SRR17520069.2202518
SRR17520069.2202519
SRR17520069.2202520
SRR17520069.2202521
SRR17520069.2202522
SRR17520069.2202523
SRR17520069.2202524
SRR17520069.2202525
SRR17520069.2202526
SRR17520069.2202527
SRR17520069.2202528
SRR17520069.2202529
SRR17520069.2202530
SRR17520069.2202531
SRR17520069.2202532
SRR17520069.2202533
SRR17520069.2202534
SRR17520069.2202535
SRR17520069.2202536
SRR17520069.2202537
SRR17520069.2202538
SRR17520069.2202539
SRR17520069.2202540
SRR17520069.2202541
SRR17520069.2202542
SRR17520069.2202543
SRR17520069.2202544
SRR17520069.2202546
SRR17520069.2202547
SRR17520069.2202548
SRR17520069.2202549
SRR17520069.2202550
SRR17520069.2202551
SRR17520069.2202552

SRR17520069.2202553
SRR17520069.2202554
SRR17520069.2202555
SRR17520069.2202556
SRR17520069.2202557
SRR17520069.2202558
SRR17520069.2202559
SRR17520069.2202560
SRR17520069.2202561
SRR17520069.2202562
SRR17520069.2202563
SRR17520069.2202564
SRR17520069.2239844
SRR17520069.2239845
SRR17520069.2239846
SRR17520069.2239847
SRR17520069.2239848
SRR17520069.2239849
SRR17520069.2239850
SRR17520069.2239851
SRR17520069.2239852
SRR17520069.2239853
SRR17520069.2239854
SRR17520069.2239855
SRR17520069.2239856
SRR17520069.2239857
SRR17520069.2239858
SRR17520069.2239859
SRR17520069.2239860
SRR17520069.2239861
SRR17520069.2239862
SRR17520069.2239863
SRR17520069.2239864
SRR17520069.2239865
SRR17520069.2239866
SRR17520069.2239867
SRR17520069.2239868
SRR17520069.2239869
SRR17520069.2239870
SRR17520069.2239871
SRR17520069.2239872
SRR17520069.2239873
SRR17520069.2239874
SRR17520069.2239875
SRR17520069.2239876
SRR17520069.2239877
SRR17520069.2239878

SRR17520069.2239879
SRR17520069.2239880
SRR17520069.2239881
SRR17520069.2239882
SRR17520069.2239883
SRR17520069.2239884
SRR17520069.2239885
SRR17520069.2239886
SRR17520069.2239887
SRR17520069.2239888
SRR17520069.2239889
SRR17520069.2239890
SRR17520069.2239891
SRR17520069.2239892
SRR17520069.2239893
SRR17520069.2239894
SRR17520069.2239895
SRR17520069.2239896
SRR17520069.2239897
SRR17520069.2239898
SRR17520069.2239899
SRR17520069.2239900
SRR17520069.2239901
SRR17520069.2239902
SRR17520069.2239903
SRR17520069.2241563
SRR17520069.2241564
SRR17520069.2241565
SRR17520069.2241566
SRR17520069.2241567
SRR17520069.2241569
SRR17520069.2241570
SRR17520069.2241571
SRR17520069.2241572
SRR17520069.2241573
SRR17520069.2241574
SRR17520069.2241575
SRR17520069.2241576
SRR17520069.2241577
SRR17520069.2241578
SRR17520069.2241579
SRR17520069.2241580
SRR17520069.2241581
SRR17520069.2241582
SRR17520069.2241584
SRR17520069.2241585
SRR17520069.2241586

SRR17520069.2241587
SRR17520069.2241588
SRR17520069.2241589
SRR17520069.2241590
SRR17520069.2241591
SRR17520069.2241592
SRR17520069.2241593
SRR17520069.2241594
SRR17520069.2247029
SRR17520069.2247030
SRR17520069.2279355
SRR17520069.2279356
SRR17520069.2279357
SRR17520069.2279358
SRR17520069.2279359
SRR17520069.2279360
SRR17520069.2279361
SRR17520069.2279362
SRR17520069.2279363
SRR17520069.2279364
SRR17520069.2279365
SRR17520069.2279366
SRR17520069.2279367
SRR17520069.2279368
SRR17520069.2279369
SRR17520069.2279370
SRR17520069.2279371
SRR17520069.2279372
SRR17520069.2279373
SRR17520069.2279374
SRR17520069.2279375
SRR17520069.2279376
SRR17520069.2279377
SRR17520069.2295970
SRR17520069.2295971
SRR17520069.2323263
SRR17520069.2323277
SRR17520069.2351083
SRR17520069.2351084
SRR17520069.2351085
SRR17520069.2351087
SRR17520069.2351088
SRR17520069.2351089
SRR17520069.2363281
SRR17520069.2370154
SRR17520069.2370155
SRR17520069.2370156

SRR17520069.2370157
SRR17520069.2370158
SRR17520069.2370159
SRR17520069.2370160
SRR17520069.2370161
SRR17520069.2370162
SRR17520069.2370163
SRR17520069.2370164
SRR17520069.2370165
SRR17520069.2370166
SRR17520069.2370167
SRR17520069.2370168
SRR17520069.2370169
SRR17520069.2370170
SRR17520069.2370171
SRR17520069.2370172
SRR17520069.2370173
SRR17520069.2370174
SRR17520069.2370359
SRR17520069.2373792
SRR17520069.2373793
SRR17520069.2373796
SRR17520069.2373797
SRR17520069.2373798
SRR17520069.2373799
SRR17520069.2373800
SRR17520069.2373801
SRR17520069.2388484
SRR17520069.2388564
SRR17520069.2388588
SRR17520069.2402402
SRR17520069.2402427
SRR17520069.2402898
SRR17520069.2402899
SRR17520069.2402900
SRR17520069.2402901
SRR17520069.2402902
SRR17520069.2402903
SRR17520069.2402904
SRR17520069.2402905
SRR17520069.2402906
SRR17520069.2402907
SRR17520069.2402908
SRR17520069.2402909
SRR17520069.2402911
SRR17520069.2402912
SRR17520069.2402913

SRR17520069.2402914
SRR17520069.2402915
SRR17520069.2402916
SRR17520069.2402917
SRR17520069.2402918
SRR17520069.2402919
SRR17520069.2402920
SRR17520069.2402921
SRR17520069.2402922
SRR17520069.2402923
SRR17520069.2402924
SRR17520069.2402925
SRR17520069.2402926
SRR17520069.2417756
SRR17520069.2435231
SRR17520069.2435260
SRR17520069.2446175
SRR17520069.2446177
SRR17520069.2446179
SRR17520069.2464949
SRR17520069.2481637
SRR17520069.2495063
SRR17520069.2495089
SRR17520069.2497988
SRR17520069.2497989
SRR17520069.2497990
SRR17520069.2497993
SRR17520069.2497994
SRR17520069.2497995
SRR17520069.2497996
SRR17520069.2497997
SRR17520069.2497998
SRR17520069.2498000
SRR17520069.2498001
SRR17520069.2498002
SRR17520069.2498003
SRR17520069.2498004
SRR17520069.2498005
SRR17520069.2498006
SRR17520069.2498007
SRR17520069.2498008
SRR17520069.2498009
SRR17520069.2498010
SRR17520069.2498011
SRR17520069.2498012
SRR17520069.2498013
SRR17520069.2498014

SRR17520069.2498015
SRR17520069.2498016
SRR17520069.2498017
SRR17520069.2498018
SRR17520069.2498019
SRR17520069.2498020
SRR17520069.2498021
SRR17520069.2498022
SRR17520069.2498023
SRR17520069.2498024
SRR17520069.2498025
SRR17520069.2498026
SRR17520069.2498027
SRR17520069.2498028
SRR17520069.2498029
SRR17520069.2498030
SRR17520069.2498031
SRR17520069.2498032
SRR17520069.2498033
SRR17520069.2498034
SRR17520069.2498035
SRR17520069.2498036
SRR17520069.2498037
SRR17520069.2498038
SRR17520069.2498039
SRR17520069.2498040
SRR17520069.2498041
SRR17520069.2498042
SRR17520069.2498043
SRR17520069.2498044
SRR17520069.2498045
SRR17520069.2498046
SRR17520069.2498047
SRR17520069.2498048
SRR17520069.2498049
SRR17520069.2498050
SRR17520069.2498051
SRR17520069.2498052
SRR17520069.2498053
SRR17520069.2498055
SRR17520069.2498056
SRR17520069.2498057
SRR17520069.2498058
SRR17520069.2498059
SRR17520069.2498060
SRR17520069.2498061
SRR17520069.2498062

SRR17520069.2498063
SRR17520069.2498064
SRR17520069.2498065
SRR17520069.2498066
SRR17520069.2498067
SRR17520069.2498068
SRR17520069.2498069
SRR17520069.2498070
SRR17520069.2498071
SRR17520069.2498072
SRR17520069.2498073
SRR17520069.2498074
SRR17520069.2498075
SRR17520069.2498076
SRR17520069.2498077
SRR17520069.2498078
SRR17520069.2498079
SRR17520069.2498080
SRR17520069.2498081
SRR17520069.2498082
SRR17520069.2498083
SRR17520069.2498084
SRR17520069.2498085
SRR17520069.2500930
SRR17520069.2500931
SRR17520069.2524060
SRR17520069.2532773
SRR17520069.2542109
SRR17520069.2545318
SRR17520069.2577345
SRR17520069.2577346
SRR17520069.2577347
SRR17520069.2577348
SRR17520069.2577349
SRR17520069.2577350
SRR17520069.2577351
SRR17520069.2577352
SRR17520069.2577353
SRR17520069.2577354
SRR17520069.2577355
SRR17520069.2577356
SRR17520069.2577357
SRR17520069.2577358
SRR17520069.2577359
SRR17520069.2577360
SRR17520069.2577361
SRR17520069.2577362

SRR17520069.2577363
SRR17520069.2577364
SRR17520069.2577365
SRR17520069.2577366
SRR17520069.2577367
SRR17520069.2577368
SRR17520069.2577369
SRR17520069.2577370
SRR17520069.2577372
SRR17520069.2577373
SRR17520069.2577374
SRR17520069.2577375
SRR17520069.2577376
SRR17520069.2577378
SRR17520069.2577379
SRR17520069.2577380
SRR17520069.2577381
SRR17520069.2577382
SRR17520069.2577383
SRR17520069.2577384
SRR17520069.2577385
SRR17520069.2577386
SRR17520069.2577387
SRR17520069.2577388
SRR17520069.2577389
SRR17520069.2577390
SRR17520069.2577391
SRR17520069.2577392
SRR17520069.2577393
SRR17520069.2577394
SRR17520069.2577395
SRR17520069.2577396
SRR17520069.2577398
SRR17520069.2587178
SRR17520069.2605281
SRR17520069.2605282
SRR17520069.2605283
SRR17520069.2605284
SRR17520069.2605285
SRR17520069.2605286
SRR17520069.2605287
SRR17520069.2605288
SRR17520069.2605289
SRR17520069.2605290
SRR17520069.2605291
SRR17520069.2605292
SRR17520069.2605293

SRR17520069.2605294
SRR17520069.2605295
SRR17520069.2605296
SRR17520069.2605297
SRR17520069.2605298
SRR17520069.2605299
SRR17520069.2605300
SRR17520069.2605301
SRR17520069.2605302
SRR17520069.2605303
SRR17520069.2605304
SRR17520069.2605305
SRR17520069.2605306
SRR17520069.2605307
SRR17520069.2605308
SRR17520069.2605309
SRR17520069.2605310
SRR17520069.2605311
SRR17520069.2605312
SRR17520069.2605313
SRR17520069.2605314
SRR17520069.2605315
SRR17520069.2605316
SRR17520069.2605317
SRR17520069.2605318
SRR17520069.2605320
SRR17520069.2605321
SRR17520069.2605323
SRR17520069.2605325
SRR17520069.2605326
SRR17520069.2605327
SRR17520069.2605329
SRR17520069.2605330
SRR17520069.2605331
SRR17520069.2605332
SRR17520069.2605333
SRR17520069.2605334
SRR17520069.2605335
SRR17520069.2605336
SRR17520069.2605337
SRR17520069.2605338
SRR17520069.2605339
SRR17520069.2605340
SRR17520069.2605341
SRR17520069.2605342
SRR17520069.2605343
SRR17520069.2605346

SRR17520069.2605347
SRR17520069.2605351
SRR17520069.2605352
SRR17520069.2605353
SRR17520069.2605354
SRR17520069.2605355
SRR17520069.2605356
SRR17520069.2605357
SRR17520069.2605358
SRR17520069.2605359
SRR17520069.2605360
SRR17520069.2605361
SRR17520069.2605362
SRR17520069.2605363
SRR17520069.2605364
SRR17520069.2605365
SRR17520069.2605366
SRR17520069.2605367
SRR17520069.2605368
SRR17520069.2605369
SRR17520069.2605370
SRR17520069.2605371
SRR17520069.2605372
SRR17520069.2605373
SRR17520069.2605374
SRR17520069.2605375
SRR17520069.2605377
SRR17520069.2605378
SRR17520069.2605379
SRR17520069.2605380
SRR17520069.2605381
SRR17520069.2605382
SRR17520069.2605383
SRR17520069.2605384
SRR17520069.2605385
SRR17520069.2605386
SRR17520069.2605387
SRR17520069.2605388
SRR17520069.2605389
SRR17520069.2605390
SRR17520069.2605391
SRR17520069.2605392
SRR17520069.2605393
SRR17520069.2605394
SRR17520069.2605395
SRR17520069.2605396
SRR17520069.2605397

SRR17520069.2605398
SRR17520069.2605399
SRR17520069.2605400
SRR17520069.2605401
SRR17520069.2605402
SRR17520069.2605403
SRR17520069.2605404
SRR17520069.2605405
SRR17520069.2605406
SRR17520069.2605407
SRR17520069.2605408
SRR17520069.2605409
SRR17520069.2605410
SRR17520069.2606271
SRR17520069.2606272
SRR17520069.2606273
SRR17520069.2606274
SRR17520069.2606275
SRR17520069.2606276
SRR17520069.2606277
SRR17520069.2606278
SRR17520069.2606279
SRR17520069.2606280
SRR17520069.2606281
SRR17520069.2606282
SRR17520069.2606283
SRR17520069.2606284
SRR17520069.2606285
SRR17520069.2606286
SRR17520069.2606287
SRR17520069.2606288
SRR17520069.2606289
SRR17520069.2606290
SRR17520069.2606291
SRR17520069.2606292
SRR17520069.2606293
SRR17520069.2606294
SRR17520069.2606295
SRR17520069.2606296
SRR17520069.2606297
SRR17520069.2606298
SRR17520069.2606299
SRR17520069.2606300
SRR17520069.2606301
SRR17520069.2606302
SRR17520069.2606303
SRR17520069.2606304

SRR17520069.2606305
SRR17520069.2606306
SRR17520069.2606307
SRR17520069.2606308
SRR17520069.2606309
SRR17520069.2606310
SRR17520069.2606311
SRR17520069.2606312
SRR17520069.2606313
SRR17520069.2606314
SRR17520069.2606315
SRR17520069.2606316
SRR17520069.2606317
SRR17520069.2606318
SRR17520069.2606319
SRR17520069.2606320
SRR17520069.2606321
SRR17520069.2606322
SRR17520069.2606323
SRR17520069.2606325
SRR17520069.2606326
SRR17520069.2606327
SRR17520069.2606328
SRR17520069.2606329
SRR17520069.2606330
SRR17520069.2606331
SRR17520069.2606332
SRR17520069.2606333
SRR17520069.2606334
SRR17520069.2606335
SRR17520069.2606336
SRR17520069.2606337
SRR17520069.2606338
SRR17520069.2606339
SRR17520069.2606340
SRR17520069.2606341
SRR17520069.2606342
SRR17520069.2606343
SRR17520069.2606345
SRR17520069.2606346
SRR17520069.2606347
SRR17520069.2606348
SRR17520069.2606349
SRR17520069.2606350
SRR17520069.2606351
SRR17520069.2606353
SRR17520069.2606354

SRR17520069.2606355
SRR17520069.2606356
SRR17520069.2606357
SRR17520069.2606358
SRR17520069.2606359
SRR17520069.2606360
SRR17520069.2606361
SRR17520069.2606362
SRR17520069.2606363
SRR17520069.2606364
SRR17520069.2606365
SRR17520069.2606366
SRR17520069.2606367
SRR17520069.2606369
SRR17520069.2606370
SRR17520069.2612565
SRR17520069.2612566
SRR17520069.2612567
SRR17520069.2612568
SRR17520069.2612569
SRR17520069.2612570
SRR17520069.2612571
SRR17520069.2612572
SRR17520069.2612573
SRR17520069.2612574
SRR17520069.2612575
SRR17520069.2612576
SRR17520069.2612577
SRR17520069.2612578
SRR17520069.2612579
SRR17520069.2612580
SRR17520069.2612581
SRR17520069.2612582
SRR17520069.2612583
SRR17520069.2625597
SRR17520069.2625598
SRR17520069.2625599
SRR17520069.2631804
SRR17520069.2631805
SRR17520069.2631806
SRR17520069.2631807
SRR17520069.2631808
SRR17520069.2631809
SRR17520069.2631810
SRR17520069.2631811
SRR17520069.2631812
SRR17520069.2631813

SRR17520069.2631814
SRR17520069.2631815
SRR17520069.2631816
SRR17520069.2631817
SRR17520069.2631818
SRR17520069.2631819
SRR17520069.2631822
SRR17520069.2631823
SRR17520069.2631824
SRR17520069.2631825
SRR17520069.2631826
SRR17520069.2631827
SRR17520069.2631828
SRR17520069.2631829
SRR17520069.2631830
SRR17520069.2631831
SRR17520069.2631832
SRR17520069.2631833
SRR17520069.2631834
SRR17520069.2631835
SRR17520069.2631836
SRR17520069.2631837
SRR17520069.2631838
SRR17520069.2631839
SRR17520069.2631840
SRR17520069.2631841
SRR17520069.2631842
SRR17520069.2631843
SRR17520069.2631844
SRR17520069.2631845
SRR17520069.2631846
SRR17520069.2631847
SRR17520069.2631848
SRR17520069.2631849
SRR17520069.2631850
SRR17520069.2631851
SRR17520069.2631852
SRR17520069.2631853
SRR17520069.2631854
SRR17520069.2631855
SRR17520069.2631856
SRR17520069.2631861
SRR17520069.2631862
SRR17520069.2631864
SRR17520069.2634992
SRR17520069.2636952
SRR17520069.2636958

SRR17520069.2646185
SRR17520069.2646186
SRR17520069.2646187
SRR17520069.2646188
SRR17520069.2646189
SRR17520069.2646190
SRR17520069.2646191
SRR17520069.2646192
SRR17520069.2646193
SRR17520069.2646194
SRR17520069.2646195
SRR17520069.2646196
SRR17520069.2646197
SRR17520069.2646198
SRR17520069.2646199
SRR17520069.2652457
SRR17520069.2652458
SRR17520069.2652459
SRR17520069.2652460
SRR17520069.2652461
SRR17520069.2652462
SRR17520069.2652463
SRR17520069.2652464
SRR17520069.2652465
SRR17520069.2652466
SRR17520069.2652467
SRR17520069.2652468
SRR17520069.2652469
SRR17520069.2652470
SRR17520069.2652471
SRR17520069.2652472
SRR17520069.2652473
SRR17520069.2652474
SRR17520069.2652475
SRR17520069.2652476
SRR17520069.2652477
SRR17520069.2652478
SRR17520069.2652479
SRR17520069.2652480
SRR17520069.2652481
SRR17520069.2652482
SRR17520069.2652483
SRR17520069.2652484
SRR17520069.2693716
SRR17520069.2700278
SRR17520069.2702931
SRR17520069.2705232

SRR17520069.2705233
SRR17520069.2705234
SRR17520069.2705235
SRR17520069.2705236
SRR17520069.2705237
SRR17520069.2705238
SRR17520069.2705239
SRR17520069.2705240
SRR17520069.2705241
SRR17520069.2705242
SRR17520069.2705243
SRR17520069.2705245
SRR17520069.2705246
SRR17520069.2705247
SRR17520069.2705248
SRR17520069.2705249
SRR17520069.2705250
SRR17520069.2705251
SRR17520069.2705252
SRR17520069.2705253
SRR17520069.2705254
SRR17520069.2705255
SRR17520069.2705256
SRR17520069.2705257
SRR17520069.2705258
SRR17520069.2705259
SRR17520069.2705261
SRR17520069.2705262
SRR17520069.2705263
SRR17520069.2705264
SRR17520069.2705265
SRR17520069.2705266
SRR17520069.2705267
SRR17520069.2705268
SRR17520069.2705269
SRR17520069.2705270
SRR17520069.2705271
SRR17520069.2705272
SRR17520069.2705273
SRR17520069.2705274
SRR17520069.2705275
SRR17520069.2705278
SRR17520069.2705279
SRR17520069.2705280
SRR17520069.2705281
SRR17520069.2705283
SRR17520069.2705284

SRR17520069.2705285
SRR17520069.2705287
SRR17520069.2705288
SRR17520069.2705289
SRR17520069.2705290
SRR17520069.2705291
SRR17520069.2705292
SRR17520069.2705293
SRR17520069.2705294
SRR17520069.2705295
SRR17520069.2705296
SRR17520069.2705297
SRR17520069.2705298
SRR17520069.2705299
SRR17520069.2705300
SRR17520069.2705301
SRR17520069.2705302
SRR17520069.2705303
SRR17520069.2705304
SRR17520069.2705305
SRR17520069.2705306
SRR17520069.2705307
SRR17520069.2705308
SRR17520069.2705309
SRR17520069.2705310
SRR17520069.2705311
SRR17520069.2705312
SRR17520069.2705313
SRR17520069.2705315
SRR17520069.2705316
SRR17520069.2705317
SRR17520069.2705318
SRR17520069.2705319
SRR17520069.2705320
SRR17520069.2705322
SRR17520069.2705323
SRR17520069.2705324
SRR17520069.2705325
SRR17520069.2705326
SRR17520069.2715476
SRR17520069.2723511
SRR17520069.2723512
SRR17520069.2723513
SRR17520069.2723515
SRR17520069.2723516
SRR17520069.2723517
SRR17520069.2723518

SRR17520069.2723519
SRR17520069.2723520
SRR17520069.2723521
SRR17520069.2723522
SRR17520069.2723523
SRR17520069.2723524
SRR17520069.2723525
SRR17520069.2723526
SRR17520069.2723527
SRR17520069.2723528
SRR17520069.2723529
SRR17520069.2723530
SRR17520069.2723531
SRR17520069.2723532
SRR17520069.2723533
SRR17520069.2723534
SRR17520069.2733819
SRR17520069.2752748
SRR17520069.2752749
SRR17520069.2752750
SRR17520069.2752751
SRR17520069.2752752
SRR17520069.2752754
SRR17520069.2752755
SRR17520069.2752756
SRR17520069.2752757
SRR17520069.2752758
SRR17520069.2752759
SRR17520069.2752760
SRR17520069.2752761
SRR17520069.2752762
SRR17520069.2752763
SRR17520069.2752764
SRR17520069.2752765
SRR17520069.2752766
SRR17520069.2752767
SRR17520069.2752768
SRR17520069.2752769
SRR17520069.2752770
SRR17520069.2752772
SRR17520069.2752773
SRR17520069.2752774
SRR17520069.2752776
SRR17520069.2752777
SRR17520069.2752778
SRR17520069.2752779
SRR17520069.2752780

SRR17520069.2752781
SRR17520069.2752782
SRR17520069.2752783
SRR17520069.2752784
SRR17520069.2752785
SRR17520069.2752787
SRR17520069.2752788
SRR17520069.2752789
SRR17520069.2752790
SRR17520069.2752791
SRR17520069.2752792
SRR17520069.2752793
SRR17520069.2752794
SRR17520069.2752795
SRR17520069.2752796
SRR17520069.2752797
SRR17520069.2752798
SRR17520069.2752799
SRR17520069.2752800
SRR17520069.2752801
SRR17520069.2752802
SRR17520069.2752803
SRR17520069.2752805
SRR17520069.2752806
SRR17520069.2752807
SRR17520069.2752808
SRR17520069.2752809
SRR17520069.2752810
SRR17520069.2752811
SRR17520069.2752812
SRR17520069.2752813
SRR17520069.2752814
SRR17520069.2752816
SRR17520069.2752817
SRR17520069.2752818
SRR17520069.2752819
SRR17520069.2752820
SRR17520069.2752821
SRR17520069.2752822
SRR17520069.2752823
SRR17520069.2752824
SRR17520069.2752825
SRR17520069.2752826
SRR17520069.2753488
SRR17520069.2753489
SRR17520069.2753490
SRR17520069.2753491

SRR17520069.2753492
SRR17520069.2753493
SRR17520069.2753494
SRR17520069.2753495
SRR17520069.2753496
SRR17520069.2753497
SRR17520069.2753498
SRR17520069.2753499
SRR17520069.2753500
SRR17520069.2753501
SRR17520069.2753502
SRR17520069.2753503
SRR17520069.2753504
SRR17520069.2753505
SRR17520069.2753506
SRR17520069.2753507
SRR17520069.2753508
SRR17520069.2753509
SRR17520069.2753510
SRR17520069.2753511
SRR17520069.2753512
SRR17520069.2753513
SRR17520069.2753514
SRR17520069.2753515
SRR17520069.2753516
SRR17520069.2753517
SRR17520069.2753519
SRR17520069.2753520
SRR17520069.2753521
SRR17520069.2753522
SRR17520069.2753523
SRR17520069.2753524
SRR17520069.2753525
SRR17520069.2753526
SRR17520069.2753527
SRR17520069.2753528
SRR17520069.2753529
SRR17520069.2753530
SRR17520069.2753531
SRR17520069.2753532
SRR17520069.2753533
SRR17520069.2753534
SRR17520069.2753535
SRR17520069.2753536
SRR17520069.2753537
SRR17520069.2753538
SRR17520069.2753539

SRR17520069.2753540
SRR17520069.2753541
SRR17520069.2753542
SRR17520069.2753543
SRR17520069.2753544
SRR17520069.2762966
SRR17520069.2762967
SRR17520069.2762968
SRR17520069.2762969
SRR17520069.2769159
SRR17520069.2769177
SRR17520069.2769178
SRR17520069.2769179
SRR17520069.2769180
SRR17520069.2769181
SRR17520069.2769182
SRR17520069.2769183
SRR17520069.2769184
SRR17520069.2769185
SRR17520069.2769186
SRR17520069.2769187
SRR17520069.2769188
SRR17520069.2769189
SRR17520069.2769190
SRR17520069.2769191
SRR17520069.2769192
SRR17520069.2769193
SRR17520069.2769194
SRR17520069.2769195
SRR17520069.2769196
SRR17520069.2769197
SRR17520069.2769198
SRR17520069.2769199
SRR17520069.2769200
SRR17520069.2769201
SRR17520069.2769202
SRR17520069.2769203
SRR17520069.2769204
SRR17520069.2769205
SRR17520069.2769206
SRR17520069.2769207
SRR17520069.2769208
SRR17520069.2769209
SRR17520069.2769210
SRR17520069.2778853
SRR17520069.2783800
SRR17520069.2783801

SRR17520069.2820597
SRR17520069.2820599
SRR17520069.2820600
SRR17520069.2820601
SRR17520069.2820602
SRR17520069.2820603
SRR17520069.2820605
SRR17520069.2820606
SRR17520069.2820607
SRR17520069.2820608
SRR17520069.2820609
SRR17520069.2820610
SRR17520069.2820611
SRR17520069.2820612
SRR17520069.2820613
SRR17520069.2820614
SRR17520069.2820615
SRR17520069.2820616
SRR17520069.2820617
SRR17520069.2820618
SRR17520069.2820619
SRR17520069.2820621
SRR17520069.2820623
SRR17520069.2820624
SRR17520069.2820627
SRR17520069.2820628
SRR17520069.2820629
SRR17520069.2820630
SRR17520069.2820631
SRR17520069.2820632
SRR17520069.2820635
SRR17520069.2820637
SRR17520069.2820638
SRR17520069.2820639
SRR17520069.2820640
SRR17520069.2820641
SRR17520069.2830956
SRR17520069.2830957
SRR17520069.2830958
SRR17520069.2830959
SRR17520069.2830960
SRR17520069.2830961
SRR17520069.2830962
SRR17520069.2830963
SRR17520069.2830964
SRR17520069.2830965
SRR17520069.2830966

SRR17520069.2847058
SRR17520069.2847077
SRR17520069.2850650
SRR17520069.2850652
SRR17520069.2850653
SRR17520069.2850654
SRR17520069.2850655
SRR17520069.2850656
SRR17520069.2850657
SRR17520069.2850658
SRR17520069.2850659
SRR17520069.2850660
SRR17520069.2850662
SRR17520069.2850663
SRR17520069.2850664
SRR17520069.2850665
SRR17520069.2850666
SRR17520069.2850667
SRR17520069.2850668
SRR17520069.2850669
SRR17520069.2850670
SRR17520069.2850671
SRR17520069.2850672
SRR17520069.2850673
SRR17520069.2850674
SRR17520069.2850675
SRR17520069.2850676
SRR17520069.2850677
SRR17520069.2850678
SRR17520069.2850679
SRR17520069.2850680
SRR17520069.2850681
SRR17520069.2850682
SRR17520069.2850683
SRR17520069.2850684
SRR17520069.2850685
SRR17520069.2850686
SRR17520069.2850687
SRR17520069.2850688
SRR17520069.2850689
SRR17520069.2850690
SRR17520069.2850691
SRR17520069.2850692
SRR17520069.2850693
SRR17520069.2850694
SRR17520069.2850695
SRR17520069.2850696

SRR17520069.2850697
SRR17520069.2850698
SRR17520069.2850699
SRR17520069.2850700
SRR17520069.2850701
SRR17520069.2850702
SRR17520069.2850703
SRR17520069.2850704
SRR17520069.2850705
SRR17520069.2850706
SRR17520069.2850707
SRR17520069.2850708
SRR17520069.2850709
SRR17520069.2850710
SRR17520069.2850711
SRR17520069.2850712
SRR17520069.2850713
SRR17520069.2850714
SRR17520069.2850715
SRR17520069.2850716
SRR17520069.2850717
SRR17520069.2850718
SRR17520069.2850719
SRR17520069.2850720
SRR17520069.2850721
SRR17520069.2850722
SRR17520069.2850723
SRR17520069.2850724
SRR17520069.2850725
SRR17520069.2850726
SRR17520069.2850727
SRR17520069.2850728
SRR17520069.2850729
SRR17520069.2850730
SRR17520069.2850731
SRR17520069.2850732
SRR17520069.2850733
SRR17520069.2850734
SRR17520069.2850735
SRR17520069.2850736
SRR17520069.2850737
SRR17520069.2850738
SRR17520069.2850739
SRR17520069.2850740
SRR17520069.2850741
SRR17520069.2850742
SRR17520069.2850743

SRR17520069.2850744
SRR17520069.2850745
SRR17520069.2851003
SRR17520069.2870014
SRR17520069.2870015
SRR17520069.2870016
SRR17520069.2870017
SRR17520069.2870018
SRR17520069.2870019
SRR17520069.2870020
SRR17520069.2870021
SRR17520069.2870022
SRR17520069.2870023
SRR17520069.2870024
SRR17520069.2870025
SRR17520069.2870026
SRR17520069.2870027
SRR17520069.2870028
SRR17520069.2870029
SRR17520069.2870030
SRR17520069.2870031
SRR17520069.2870032
SRR17520069.2870033
SRR17520069.2870034
SRR17520069.2870035
SRR17520069.2870036
SRR17520069.2870037
SRR17520069.2870038
SRR17520069.2870039
SRR17520069.2870040
SRR17520069.2870041
SRR17520069.2870042
SRR17520069.2870043
SRR17520069.2870044
SRR17520069.2870045
SRR17520069.2870046
SRR17520069.2870047
SRR17520069.2870048
SRR17520069.2870049
SRR17520069.2870050
SRR17520069.2870051
SRR17520069.2870052
SRR17520069.2870053
SRR17520069.2870054
SRR17520069.2870055
SRR17520069.2870056
SRR17520069.2870736

SRR17520069.2870738
SRR17520069.2870739
SRR17520069.2870740
SRR17520069.2870742
SRR17520069.2870743
SRR17520069.2870744
SRR17520069.2870745
SRR17520069.2870746
SRR17520069.2870747
SRR17520069.2870748
SRR17520069.2870750
SRR17520069.2870751
SRR17520069.2870752
SRR17520069.2870753
SRR17520069.2870754
SRR17520069.2870757
SRR17520069.2870758
SRR17520069.2870759
SRR17520069.2870760
SRR17520069.2870762
SRR17520069.2870763
SRR17520069.2870764
SRR17520069.2870765
SRR17520069.2870766
SRR17520069.2870767
SRR17520069.2870768
SRR17520069.2870769
SRR17520069.2870770
SRR17520069.2870771
SRR17520069.2870772
SRR17520069.2870773
SRR17520069.2870774
SRR17520069.2870776
SRR17520069.2870777
SRR17520069.2870778
SRR17520069.2870779
SRR17520069.2870780
SRR17520069.2870781
SRR17520069.2870783
SRR17520069.2870784
SRR17520069.2870785
SRR17520069.2870786
SRR17520069.2870787
SRR17520069.2870788
SRR17520069.2870789
SRR17520069.2870790
SRR17520069.2870791

SRR17520069.2870792
SRR17520069.2870793
SRR17520069.2870794
SRR17520069.2870795
SRR17520069.2870796
SRR17520069.2870797
SRR17520069.2870798
SRR17520069.2870799
SRR17520069.2870800
SRR17520069.2870801
SRR17520069.2870803
SRR17520069.2870805
SRR17520069.2870806
SRR17520069.2870807
SRR17520069.2870808
SRR17520069.2870809
SRR17520069.2870810
SRR17520069.2870811
SRR17520069.2870812
SRR17520069.2874709
SRR17520069.2874710
SRR17520069.2874711
SRR17520069.2874712
SRR17520069.2874713
SRR17520069.2874714
SRR17520069.2874715
SRR17520069.2874716
SRR17520069.2874717
SRR17520069.2874718
SRR17520069.2874719
SRR17520069.2874720
SRR17520069.2874721
SRR17520069.2874722
SRR17520069.2877847
SRR17520069.2879764
SRR17520069.2879765
SRR17520069.2879766
SRR17520069.2879767
SRR17520069.2879768
SRR17520069.2879769
SRR17520069.2879770
SRR17520069.2879771
SRR17520069.2879772
SRR17520069.2879773
SRR17520069.2879774
SRR17520069.2879775
SRR17520069.2879776

SRR17520069.2879777
SRR17520069.2879778
SRR17520069.2879779
SRR17520069.2879780
SRR17520069.2879781
SRR17520069.2879782
SRR17520069.2879783
SRR17520069.2879784
SRR17520069.2879785
SRR17520069.2879786
SRR17520069.2879787
SRR17520069.2879788
SRR17520069.2879789
SRR17520069.2879790
SRR17520069.2879791
SRR17520069.2879792
SRR17520069.2879793
SRR17520069.2879794
SRR17520069.2879795
SRR17520069.2879796
SRR17520069.2879797
SRR17520069.2879798
SRR17520069.2879799
SRR17520069.2879800
SRR17520069.2879801
SRR17520069.2879802
SRR17520069.2879803
SRR17520069.2879804
SRR17520069.2879805
SRR17520069.2879806
SRR17520069.2879807
SRR17520069.2879808
SRR17520069.2879809
SRR17520069.2879810
SRR17520069.2879811
SRR17520069.2879812
SRR17520069.2879813
SRR17520069.2879814
SRR17520069.2879815
SRR17520069.2879817
SRR17520069.2879818
SRR17520069.2879819
SRR17520069.2879820
SRR17520069.2879821
SRR17520069.2879822
SRR17520069.2879823
SRR17520069.2879824

SRR17520069.2879825
SRR17520069.2879826
SRR17520069.2879827
SRR17520069.2879828
SRR17520069.2879829
SRR17520069.2879830
SRR17520069.2879831
SRR17520069.2879832
SRR17520069.2879833
SRR17520069.2879834
SRR17520069.2879835
SRR17520069.2879836
SRR17520069.2879837
SRR17520069.2879838
SRR17520069.2879839
SRR17520069.2879840
SRR17520069.2879841
SRR17520069.2879842
SRR17520069.2879843
SRR17520069.2879844
SRR17520069.2879845
SRR17520069.2879846
SRR17520069.2879847
SRR17520069.2879848
SRR17520069.2879849
SRR17520069.2879850
SRR17520069.2879851
SRR17520069.2879852
SRR17520069.2879853
SRR17520069.2879854
SRR17520069.2879855
SRR17520069.2879856
SRR17520069.2879857
SRR17520069.2879858
SRR17520069.2879859
SRR17520069.2879860
SRR17520069.2879861
SRR17520069.2879862
SRR17520069.2879863
SRR17520069.2879864
SRR17520069.2879865
SRR17520069.2879866
SRR17520069.2879867
SRR17520069.2879868
SRR17520069.2879869
SRR17520069.2886771
SRR17520069.2886772

SRR17520069.2891146
SRR17520069.2891156
SRR17520069.2895447
SRR17520069.2895448
SRR17520069.2895449
SRR17520069.2895450
SRR17520069.2895451
SRR17520069.2895452
SRR17520069.2895453
SRR17520069.2895454
SRR17520069.2895455
SRR17520069.2895456
SRR17520069.2895457
SRR17520069.2895458
SRR17520069.2895459
SRR17520069.2895460
SRR17520069.2895461
SRR17520069.2895462
SRR17520069.2895463
SRR17520069.2895464
SRR17520069.2895465
SRR17520069.2895466
SRR17520069.2895467
SRR17520069.2895468
SRR17520069.2895469
SRR17520069.2895470
SRR17520069.2895471
SRR17520069.2895472
SRR17520069.2895473
SRR17520069.2895474
SRR17520069.2895624
SRR17520069.2898293
SRR17520069.2898295
SRR17520069.2907100
SRR17520069.2907101
SRR17520069.2907102
SRR17520069.2907103
SRR17520069.2907104
SRR17520069.2907105
SRR17520069.2907106
SRR17520069.2907107
SRR17520069.2907108
SRR17520069.2907109
SRR17520069.2907110
SRR17520069.2907111
SRR17520069.2907112
SRR17520069.2907114

SRR17520069.2907116
SRR17520069.2907117
SRR17520069.2907118
SRR17520069.2907119
SRR17520069.2907120
SRR17520069.2907121
SRR17520069.2907122
SRR17520069.2907124
SRR17520069.2907125
SRR17520069.2907126
SRR17520069.2907127
SRR17520069.2907128
SRR17520069.2907129
SRR17520069.2907130
SRR17520069.2907131
SRR17520069.2907132
SRR17520069.2907133
SRR17520069.2907134
SRR17520069.2907135
SRR17520069.2907136
SRR17520069.2907137
SRR17520069.2907138
SRR17520069.2907139
SRR17520069.2907140
SRR17520069.2907141
SRR17520069.2907142
SRR17520069.2907143
SRR17520069.2907144
SRR17520069.2907145
SRR17520069.2907146
SRR17520069.2907147
SRR17520069.2907148
SRR17520069.2907149
SRR17520069.2907150
SRR17520069.2907151
SRR17520069.2907152
SRR17520069.2907153
SRR17520069.2907155
SRR17520069.2907156
SRR17520069.2907157
SRR17520069.2907158
SRR17520069.2907159
SRR17520069.2907161
SRR17520069.2907162
SRR17520069.2907163
SRR17520069.2907164
SRR17520069.2907165

SRR17520069.2907166
SRR17520069.2907168
SRR17520069.2907169
SRR17520069.2907170
SRR17520069.2907171
SRR17520069.2907172
SRR17520069.2907173
SRR17520069.2907174
SRR17520069.2907175
SRR17520069.2907176
SRR17520069.2907177
SRR17520069.2907178
SRR17520069.2907179
SRR17520069.2907180
SRR17520069.2907181
SRR17520069.2907182
SRR17520069.2907183
SRR17520069.2907184
SRR17520069.2923196
SRR17520069.2923197
SRR17520069.2923198
SRR17520069.2923199
SRR17520069.2923200
SRR17520069.2923201
SRR17520069.2923202
SRR17520069.2923203
SRR17520069.2923204
SRR17520069.2923205
SRR17520069.2923206
SRR17520069.2923207
SRR17520069.2923208
SRR17520069.2923209
SRR17520069.2923210
SRR17520069.2923211
SRR17520069.2923212
SRR17520069.2923213
SRR17520069.2923215
SRR17520069.2923216
SRR17520069.2923217
SRR17520069.2923218
SRR17520069.2923219
SRR17520069.2923221
SRR17520069.2923222
SRR17520069.2923223
SRR17520069.2923224
SRR17520069.2923225
SRR17520069.2923228

SRR17520069.2923229
SRR17520069.2923230
SRR17520069.2923231
SRR17520069.2923232
SRR17520069.2923233
SRR17520069.2923234
SRR17520069.2923235
SRR17520069.2923237
SRR17520069.2923238
SRR17520069.2923239
SRR17520069.2923240
SRR17520069.2923241
SRR17520069.2923242
SRR17520069.2923243
SRR17520069.2923244
SRR17520069.2923245
SRR17520069.2923246
SRR17520069.2923247
SRR17520069.2923248
SRR17520069.2923249
SRR17520069.2923250
SRR17520069.2923251
SRR17520069.2923252
SRR17520069.2923253
SRR17520069.2923255
SRR17520069.2923256
SRR17520069.2923257
SRR17520069.2923258
SRR17520069.2923259
SRR17520069.2923260
SRR17520069.2923261
SRR17520069.2923262
SRR17520069.2923263
SRR17520069.2923264
SRR17520069.2923265
SRR17520069.2923266
SRR17520069.2923267
SRR17520069.2923268
SRR17520069.2923269
SRR17520069.2923270
SRR17520069.2923271
SRR17520069.2923272
SRR17520069.2923274
SRR17520069.2923275
SRR17520069.2923276
SRR17520069.2923277
SRR17520069.2923278

SRR17520069.2923279
SRR17520069.2923280
SRR17520069.2923281
SRR17520069.2923283
SRR17520069.2923284
SRR17520069.2923285
SRR17520069.2923286
SRR17520069.2923287
SRR17520069.2923288
SRR17520069.2939517
SRR17520069.2939518
SRR17520069.2939519
SRR17520069.2939520
SRR17520069.2939521
SRR17520069.2939522
SRR17520069.2939523
SRR17520069.2939524
SRR17520069.2939525
SRR17520069.2939526
SRR17520069.2939527
SRR17520069.2939528
SRR17520069.2939529
SRR17520069.2939530
SRR17520069.2939531
SRR17520069.2939532
SRR17520069.2939533
SRR17520069.2939534
SRR17520069.2939535
SRR17520069.2939536
SRR17520069.2939537
SRR17520069.2939538
SRR17520069.2939539
SRR17520069.2939540
SRR17520069.2939541
SRR17520069.2939544
SRR17520069.2939545
SRR17520069.2939547
SRR17520069.2939548
SRR17520069.2939549
SRR17520069.2939550
SRR17520069.2939551
SRR17520069.2939552
SRR17520069.2939553
SRR17520069.2939554
SRR17520069.2939555
SRR17520069.2939556
SRR17520069.2939557

SRR17520069.2939558
SRR17520069.2939559
SRR17520069.2939560
SRR17520069.2939561
SRR17520069.2939562
SRR17520069.2939563
SRR17520069.2939564
SRR17520069.2939565
SRR17520069.2939566
SRR17520069.2939567
SRR17520069.2939568
SRR17520069.2939569
SRR17520069.2939570
SRR17520069.2939571
SRR17520069.2939572
SRR17520069.2939573
SRR17520069.2939574
SRR17520069.2939575
SRR17520069.2939576
SRR17520069.2939577
SRR17520069.2939578
SRR17520069.2939579
SRR17520069.2939580
SRR17520069.2939581
SRR17520069.2939582
SRR17520069.2939583
SRR17520069.2939584
SRR17520069.2939585
SRR17520069.2939586
SRR17520069.2939587
SRR17520069.2939588
SRR17520069.2939589
SRR17520069.2939590
SRR17520069.2939591
SRR17520069.2939592
SRR17520069.2939593
SRR17520069.2939594
SRR17520069.2939595
SRR17520069.2939596
SRR17520069.2939597
SRR17520069.2939598
SRR17520069.2939599
SRR17520069.2939600
SRR17520069.2939601
SRR17520069.2939603
SRR17520069.2939604
SRR17520069.2939605

SRR17520069.2939606
SRR17520069.2939607
SRR17520069.2939608
SRR17520069.2939609
SRR17520069.2939610
SRR17520069.2939611
SRR17520069.2943944
SRR17520069.2943952
SRR17520069.2955366
SRR17520069.2955367
SRR17520069.2955368
SRR17520069.2955369
SRR17520069.2955370
SRR17520069.2955371
SRR17520069.2955372
SRR17520069.2955373
SRR17520069.2955374
SRR17520069.2955375
SRR17520069.2955376
SRR17520069.2955377
SRR17520069.2955378
SRR17520069.2955379
SRR17520069.2955380
SRR17520069.2955381
SRR17520069.2955382
SRR17520069.2955383
SRR17520069.2955384
SRR17520069.2955385
SRR17520069.2955386
SRR17520069.2955387
SRR17520069.2955388
SRR17520069.2955389
SRR17520069.2955390
SRR17520069.2955391
SRR17520069.2955392
SRR17520069.2955393
SRR17520069.2955394
SRR17520069.2955395
SRR17520069.2955396
SRR17520069.2955397
SRR17520069.2955398
SRR17520069.2955399
SRR17520069.2955400
SRR17520069.2955401
SRR17520069.2955402
SRR17520069.2955403
SRR17520069.2955404

SRR17520069.2955405
SRR17520069.2955406
SRR17520069.2955407
SRR17520069.2955408
SRR17520069.2955409
SRR17520069.2955410
SRR17520069.2955411
SRR17520069.2955412
SRR17520069.2955413
SRR17520069.2955414
SRR17520069.2955415
SRR17520069.2955416
SRR17520069.2955417
SRR17520069.2955418
SRR17520069.2955419
SRR17520069.2955420
SRR17520069.2955421
SRR17520069.2955422
SRR17520069.2955423
SRR17520069.2955424
SRR17520069.2955425
SRR17520069.2955426
SRR17520069.2955427
SRR17520069.2955428
SRR17520069.2955429
SRR17520069.2955430
SRR17520069.2955431
SRR17520069.2955432
SRR17520069.2955433
SRR17520069.2955434
SRR17520069.2955435
SRR17520069.2955436
SRR17520069.2955437
SRR17520069.2955438
SRR17520069.2955439
SRR17520069.2965844
SRR17520069.2965904
SRR17520069.2984768
SRR17520069.2984770
SRR17520069.2984771
SRR17520069.2984772
SRR17520069.2984773
SRR17520069.2984774
SRR17520069.2984775
SRR17520069.2984776
SRR17520069.2995373
SRR17520069.2995374

SRR17520069.2995375
SRR17520069.2995376
SRR17520069.2995377
SRR17520069.2995378
SRR17520069.2995379
SRR17520069.2995381
SRR17520069.2995383
SRR17520069.2995385
SRR17520069.2995386
SRR17520069.2995387
SRR17520069.2995388
SRR17520069.2995389
SRR17520069.2995390
SRR17520069.2995391
SRR17520069.2995392
SRR17520069.2995393
SRR17520069.2995394
SRR17520069.2995395
SRR17520069.3010035
SRR17520069.3010036
SRR17520069.3010037
SRR17520069.3010038
SRR17520069.3010039
SRR17520069.3010040
SRR17520069.3010041
SRR17520069.3010042
SRR17520069.3010043
SRR17520069.3010044
SRR17520069.3010045
SRR17520069.3010046
SRR17520069.3010047
SRR17520069.3010048
SRR17520069.3010049
SRR17520069.3010050
SRR17520069.3010051
SRR17520069.3010052
SRR17520069.3010053
SRR17520069.3010054
SRR17520069.3010055
SRR17520069.3010056
SRR17520069.3010057
SRR17520069.3010058
SRR17520069.3010059
SRR17520069.3010060
SRR17520069.3010062
SRR17520069.3010063
SRR17520069.3010064

SRR17520069.3010066
SRR17520069.3010067
SRR17520069.3010068
SRR17520069.3010069
SRR17520069.3010070
SRR17520069.3010071
SRR17520069.3010072
SRR17520069.3010073
SRR17520069.3010074
SRR17520069.3010075
SRR17520069.3010076
SRR17520069.3010077
SRR17520069.3010078
SRR17520069.3010079
SRR17520069.3010080
SRR17520069.3010081
SRR17520069.3010082
SRR17520069.3010083
SRR17520069.3010084
SRR17520069.3010085
SRR17520069.3010086
SRR17520069.3010087
SRR17520069.3010088
SRR17520069.3010089
SRR17520069.3010090
SRR17520069.3010092
SRR17520069.3010093
SRR17520069.3010094
SRR17520069.3010095
SRR17520069.3010096
SRR17520069.3010097
SRR17520069.3010098
SRR17520069.3010099
SRR17520069.3010100
SRR17520069.3010101
SRR17520069.3010102
SRR17520069.3010103
SRR17520069.3010104
SRR17520069.3010105
SRR17520069.3010106
SRR17520069.3010107
SRR17520069.3010108
SRR17520069.3010109
SRR17520069.3010110
SRR17520069.3010111
SRR17520069.3010112
SRR17520069.3010113

SRR17520069.3010114
SRR17520069.3010115
SRR17520069.3010116
SRR17520069.3010117
SRR17520069.3010118
SRR17520069.3010119
SRR17520069.3010120
SRR17520069.3010121
SRR17520069.3010122
SRR17520069.3010123
SRR17520069.3010124
SRR17520069.3010125
SRR17520069.3010126
SRR17520069.3010127
SRR17520069.3010128
SRR17520069.3010129
SRR17520069.3010130
SRR17520069.3010131
SRR17520069.3010132
SRR17520069.3010133
SRR17520069.3010134
SRR17520069.3010135
SRR17520069.3010136
SRR17520069.3010137
SRR17520069.3010138
SRR17520069.3010139
SRR17520069.3010140
SRR17520069.3010141
SRR17520069.3010142
SRR17520069.3010143
SRR17520069.3010144
SRR17520069.3010145
SRR17520069.3010146
SRR17520069.3010147
SRR17520069.3010148
SRR17520069.3010150
SRR17520069.3010151
SRR17520069.3010152
SRR17520069.3010153
SRR17520069.3012081
SRR17520069.3017826
SRR17520069.3017846
SRR17520069.3044052
SRR17520069.3044080
SRR17520069.3044122
SRR17520069.3052339
SRR17520069.3054452

SRR17520069.3054453
SRR17520069.3054454
SRR17520069.3054455
SRR17520069.3054456
SRR17520069.3054457
SRR17520069.3082810
SRR17520069.3082811
SRR17520069.3082812
SRR17520069.3082813
SRR17520069.3082814
SRR17520069.3082815
SRR17520069.3082816
SRR17520069.3082817
SRR17520069.3082818
SRR17520069.3082819
SRR17520069.3090057
SRR17520069.3090058
SRR17520069.3090059
SRR17520069.3090060
SRR17520069.3090061
SRR17520069.3090062
SRR17520069.3090063
SRR17520069.3090065
SRR17520069.3090066
SRR17520069.3090067
SRR17520069.3091522
SRR17520069.3091523
SRR17520069.3091524
SRR17520069.3091525
SRR17520069.3091526
SRR17520069.3091527
SRR17520069.3091528
SRR17520069.3091529
SRR17520069.3091530
SRR17520069.3091531
SRR17520069.3091532
SRR17520069.3091533
SRR17520069.3091534
SRR17520069.3091535
SRR17520069.3091536
SRR17520069.3091537
SRR17520069.3091538
SRR17520069.3091539
SRR17520069.3091540
SRR17520069.3091541
SRR17520069.3091542
SRR17520069.3091543

SRR17520069.3091544
SRR17520069.3091545
SRR17520069.3091546
SRR17520069.3091547
SRR17520069.3091548
SRR17520069.3091549
SRR17520069.3091550
SRR17520069.3091551
SRR17520069.3091552
SRR17520069.3091553
SRR17520069.3091554
SRR17520069.3091555
SRR17520069.3091556
SRR17520069.3091557
SRR17520069.3091558
SRR17520069.3091559
SRR17520069.3091560
SRR17520069.3091561
SRR17520069.3091562
SRR17520069.3091563
SRR17520069.3091564
SRR17520069.3091565
SRR17520069.3091566
SRR17520069.3091567
SRR17520069.3091568
SRR17520069.3091569
SRR17520069.3091570
SRR17520069.3091571
SRR17520069.3091572
SRR17520069.3091573
SRR17520069.3091574
SRR17520069.3091575
SRR17520069.3091576
SRR17520069.3091577
SRR17520069.3091578
SRR17520069.3091579
SRR17520069.3091580
SRR17520069.3091581
SRR17520069.3091582
SRR17520069.3091583
SRR17520069.3091584
SRR17520069.3091585
SRR17520069.3091586
SRR17520069.3091587
SRR17520069.3091588
SRR17520069.3091589
SRR17520069.3091590

SRR17520069.3091591
SRR17520069.3091592
SRR17520069.3091593
SRR17520069.3091594
SRR17520069.3091595
SRR17520069.3091596
SRR17520069.3091597
SRR17520069.3091598
SRR17520069.3091599
SRR17520069.3091600
SRR17520069.3091602
SRR17520069.3091603
SRR17520069.3091604
SRR17520069.3091605
SRR17520069.3091606
SRR17520069.3091607
SRR17520069.3091608
SRR17520069.3091609
SRR17520069.3091610
SRR17520069.3091611
SRR17520069.3091612
SRR17520069.3091613
SRR17520069.3091614
SRR17520069.3091615
SRR17520069.3091616
SRR17520069.3091617
SRR17520069.3091618
SRR17520069.3091619
SRR17520069.3091620
SRR17520069.3091621
SRR17520069.3091622
SRR17520069.3091623
SRR17520069.3091624
SRR17520069.3091625
SRR17520069.3091626
SRR17520069.3091627
SRR17520069.3091628
SRR17520069.3091629
SRR17520069.3091630
SRR17520069.3091631
SRR17520069.3091632
SRR17520069.3091633
SRR17520069.3091634
SRR17520069.3091635
SRR17520069.3091636
SRR17520069.3091637
SRR17520069.3091638

SRR17520069.3091639
SRR17520069.3091640
SRR17520069.3091641
SRR17520069.3091642
SRR17520069.3091644
SRR17520069.3091645
SRR17520069.3091646
SRR17520069.3091647
SRR17520069.3091648
SRR17520069.3091649
SRR17520069.3091650
SRR17520069.3091651
SRR17520069.3091652
SRR17520069.3091653
SRR17520069.3091654
SRR17520069.3091655
SRR17520069.3091656
SRR17520069.3091657
SRR17520069.3091658
SRR17520069.3091659
SRR17520069.3091660
SRR17520069.3091661
SRR17520069.3091662
SRR17520069.3091663
SRR17520069.3091664
SRR17520069.3091665
SRR17520069.3091666
SRR17520069.3091668
SRR17520069.3091669
SRR17520069.3091670
SRR17520069.3091671
SRR17520069.3091672
SRR17520069.3091673
SRR17520069.3091674
SRR17520069.3091675
SRR17520069.3091676
SRR17520069.3091677
SRR17520069.3091678
SRR17520069.3091679
SRR17520069.3091680
SRR17520069.3091681
SRR17520069.3091682
SRR17520069.3104308
SRR17520069.3104309
SRR17520069.3104310
SRR17520069.3104311
SRR17520069.3104312

SRR17520069.3104313
SRR17520069.3104314
SRR17520069.3104315
SRR17520069.3104316
SRR17520069.3104317
SRR17520069.3104318
SRR17520069.3104319
SRR17520069.3104321
SRR17520069.3104322
SRR17520069.3104323
SRR17520069.3104324
SRR17520069.3104325
SRR17520069.3104326
SRR17520069.3104327
SRR17520069.3104328
SRR17520069.3104329
SRR17520069.3104330
SRR17520069.3104331
SRR17520069.3104332
SRR17520069.3104333
SRR17520069.3104334
SRR17520069.3104335
SRR17520069.3104336
SRR17520069.3104337
SRR17520069.3104338
SRR17520069.3104339
SRR17520069.3104340
SRR17520069.3104341
SRR17520069.3104342
SRR17520069.3104343
SRR17520069.3104344
SRR17520069.3104345
SRR17520069.3104346
SRR17520069.3104347
SRR17520069.3104348
SRR17520069.3104349
SRR17520069.3104350
SRR17520069.3104351
SRR17520069.3104352
SRR17520069.3104353
SRR17520069.3131926
SRR17520069.3131927
SRR17520069.3131929
SRR17520069.3131930
SRR17520069.3131931
SRR17520069.3131932
SRR17520069.3131933

SRR17520069.3131934
SRR17520069.3131935
SRR17520069.3132240
SRR17520069.3138076
SRR17520069.3140514
SRR17520069.3140516
SRR17520069.3140517
SRR17520069.3140518
SRR17520069.3140519
SRR17520069.3140520
SRR17520069.3140523
SRR17520069.3140524
SRR17520069.3140526
SRR17520069.3140527
SRR17520069.3140528
SRR17520069.3140530
SRR17520069.3140531
SRR17520069.3152042
SRR17520069.3152043
SRR17520069.3152044
SRR17520069.3152045
SRR17520069.3152046
SRR17520069.3152047
SRR17520069.3152048
SRR17520069.3152049
SRR17520069.3152050
SRR17520069.3152051
SRR17520069.3152052
SRR17520069.3152053
SRR17520069.3152055
SRR17520069.3152056
SRR17520069.3152057
SRR17520069.3152058
SRR17520069.3152059
SRR17520069.3152062
SRR17520069.3152063
SRR17520069.3152064
SRR17520069.3152065
SRR17520069.3152066
SRR17520069.3152067
SRR17520069.3152068
SRR17520069.3152069
SRR17520069.3152070
SRR17520069.3152071
SRR17520069.3152072
SRR17520069.3152073
SRR17520069.3152074

SRR17520069.3152075
SRR17520069.3152076
SRR17520069.3152077
SRR17520069.3152078
SRR17520069.3152080
SRR17520069.3152081
SRR17520069.3152082
SRR17520069.3152085
SRR17520069.3152086
SRR17520069.3152087
SRR17520069.3152088
SRR17520069.3152089
SRR17520069.3152090
SRR17520069.3152092
SRR17520069.3152093
SRR17520069.3152094
SRR17520069.3152095
SRR17520069.3152096
SRR17520069.3152097
SRR17520069.3152098
SRR17520069.3152099
SRR17520069.3152101
SRR17520069.3152102
SRR17520069.3152103
SRR17520069.3152104
SRR17520069.3152105
SRR17520069.3152106
SRR17520069.3152107
SRR17520069.3152108
SRR17520069.3152109
SRR17520069.3152111
SRR17520069.3152112
SRR17520069.3152113
SRR17520069.3152114
SRR17520069.3152115
SRR17520069.3152116
SRR17520069.3152117
SRR17520069.3152118
SRR17520069.3152119
SRR17520069.3152120
SRR17520069.3152121
SRR17520069.3152122
SRR17520069.3152123
SRR17520069.3172844
SRR17520069.3172929
SRR17520069.3172958
SRR17520069.3172960

SRR17520069.3173006
SRR17520069.3180706
SRR17520069.3180707
SRR17520069.3180708
SRR17520069.3180709
SRR17520069.3180710
SRR17520069.3180711
SRR17520069.3180712
SRR17520069.3180713
SRR17520069.3180714
SRR17520069.3180715
SRR17520069.3180716
SRR17520069.3180717
SRR17520069.3180718
SRR17520069.3180719
SRR17520069.3180720
SRR17520069.3180721
SRR17520069.3180722
SRR17520069.3180723
SRR17520069.3180724
SRR17520069.3180725
SRR17520069.3180726
SRR17520069.3180727
SRR17520069.3180728
SRR17520069.3180730
SRR17520069.3180731
SRR17520069.3180732
SRR17520069.3180733
SRR17520069.3180734
SRR17520069.3180735
SRR17520069.3180736
SRR17520069.3180737
SRR17520069.3199490
SRR17520069.3199506
SRR17520069.3204228
SRR17520069.3204229
SRR17520069.3204230
SRR17520069.3204231
SRR17520069.3204232
SRR17520069.3204233
SRR17520069.3204234
SRR17520069.3204235
SRR17520069.3204236
SRR17520069.3204237
SRR17520069.3204238
SRR17520069.3204239
SRR17520069.3204240

SRR17520069.3204241
SRR17520069.3204242
SRR17520069.3204243
SRR17520069.3204244
SRR17520069.3204245
SRR17520069.3204246
SRR17520069.3204247
SRR17520069.3204248
SRR17520069.3204249
SRR17520069.3204250
SRR17520069.3204251
SRR17520069.3204253
SRR17520069.3204254
SRR17520069.3204255
SRR17520069.3204256
SRR17520069.3204257
SRR17520069.3204258
SRR17520069.3204259
SRR17520069.3204260
SRR17520069.3204261
SRR17520069.3204262
SRR17520069.3204263
SRR17520069.3204265
SRR17520069.3204266
SRR17520069.3204267
SRR17520069.3204268
SRR17520069.3204269
SRR17520069.3204270
SRR17520069.3204271
SRR17520069.3204272
SRR17520069.3204273
SRR17520069.3204274
SRR17520069.3204275
SRR17520069.3204276
SRR17520069.3204277
SRR17520069.3204278
SRR17520069.3204279
SRR17520069.3204280
SRR17520069.3204281
SRR17520069.3204282
SRR17520069.3204283
SRR17520069.3204284
SRR17520069.3204285
SRR17520069.3204286
SRR17520069.3204287
SRR17520069.3204288
SRR17520069.3204289

SRR17520069.3204290
SRR17520069.3204291
SRR17520069.3204292
SRR17520069.3204293
SRR17520069.3204294
SRR17520069.3204295
SRR17520069.3204296
SRR17520069.3204297
SRR17520069.3204298
SRR17520069.3204299
SRR17520069.3204300
SRR17520069.3204301
SRR17520069.3204302
SRR17520069.3209958
SRR17520069.3209959
SRR17520069.3209960
SRR17520069.3209961
SRR17520069.3209962
SRR17520069.3209963
SRR17520069.3209964
SRR17520069.3209965
SRR17520069.3209966
SRR17520069.3209967
SRR17520069.3209968
SRR17520069.3209969
SRR17520069.3209970
SRR17520069.3209971
SRR17520069.3209972
SRR17520069.3209973
SRR17520069.3209974
SRR17520069.3209975
SRR17520069.3209976
SRR17520069.3220085
SRR17520069.3220086
SRR17520069.3220087
SRR17520069.3220088
SRR17520069.3220089
SRR17520069.3220091
SRR17520069.3220092
SRR17520069.3220093
SRR17520069.3220094
SRR17520069.3220095
SRR17520069.3220096
SRR17520069.3220097
SRR17520069.3220098
SRR17520069.3220099
SRR17520069.3220100

SRR17520069.3220101
SRR17520069.3220102
SRR17520069.3220103
SRR17520069.3220104
SRR17520069.3220105
SRR17520069.3220106
SRR17520069.3220107
SRR17520069.3220108
SRR17520069.3220109
SRR17520069.3220110
SRR17520069.3220111
SRR17520069.3220112
SRR17520069.3220113
SRR17520069.3220114
SRR17520069.3220115
SRR17520069.3220116
SRR17520069.3220117
SRR17520069.3220118
SRR17520069.3220119
SRR17520069.3220120
SRR17520069.3220121
SRR17520069.3220122
SRR17520069.3220123
SRR17520069.3220124
SRR17520069.3220125
SRR17520069.3220126
SRR17520069.3220127
SRR17520069.3220128
SRR17520069.3220129
SRR17520069.3220130
SRR17520069.3220131
SRR17520069.3220132
SRR17520069.3220133
SRR17520069.3220134
SRR17520069.3220135
SRR17520069.3220136
SRR17520069.3220137
SRR17520069.3220138
SRR17520069.3220139
SRR17520069.3220140
SRR17520069.3220141
SRR17520069.3220143
SRR17520069.3220144
SRR17520069.3220145
SRR17520069.3220146
SRR17520069.3220147
SRR17520069.3220148

SRR17520069.3220149
SRR17520069.3220150
SRR17520069.3220151
SRR17520069.3220152
SRR17520069.3220154
SRR17520069.3220155
SRR17520069.3220156
SRR17520069.3220157
SRR17520069.3220158
SRR17520069.3220159
SRR17520069.3220160
SRR17520069.3220161
SRR17520069.3220162
SRR17520069.3220163
SRR17520069.3220164
SRR17520069.3220165
SRR17520069.3220166
SRR17520069.3220167
SRR17520069.3220168
SRR17520069.3220169
SRR17520069.3220170
SRR17520069.3220171
SRR17520069.3220172
SRR17520069.3220173
SRR17520069.3220174
SRR17520069.3220175
SRR17520069.3220176
SRR17520069.3220177
SRR17520069.3220178
SRR17520069.3220179
SRR17520069.3228483
SRR17520069.3228484
SRR17520069.3228485
SRR17520069.3228486
SRR17520069.3228487
SRR17520069.3228488
SRR17520069.3228489
SRR17520069.3228491
SRR17520069.3228492
SRR17520069.3228494
SRR17520069.3228495
SRR17520069.3228496
SRR17520069.3228497
SRR17520069.3228498
SRR17520069.3228499
SRR17520069.3235305
SRR17520069.3235306

SRR17520069.3235307
SRR17520069.3235308
SRR17520069.3235309
SRR17520069.3235310
SRR17520069.3235311
SRR17520069.3235313
SRR17520069.3235316
SRR17520069.3235319
SRR17520069.3235320
SRR17520069.3235322
SRR17520069.3236277
SRR17520069.3236278
SRR17520069.3236279
SRR17520069.3236280
SRR17520069.3236281
SRR17520069.3236282
SRR17520069.3236283
SRR17520069.3236284
SRR17520069.3236285
SRR17520069.3236286
SRR17520069.3236287
SRR17520069.3236288
SRR17520069.3236291
SRR17520069.3236292
SRR17520069.3236293
SRR17520069.3236294
SRR17520069.3236295
SRR17520069.3236296
SRR17520069.3236297
SRR17520069.3236298
SRR17520069.3236299
SRR17520069.3236300
SRR17520069.3236301
SRR17520069.3236302
SRR17520069.3236303
SRR17520069.3236305
SRR17520069.3236306
SRR17520069.3236307
SRR17520069.3236308
SRR17520069.3236309
SRR17520069.3236310
SRR17520069.3236311
SRR17520069.3236312
SRR17520069.3236313
SRR17520069.3236314
SRR17520069.3236315
SRR17520069.3236316

SRR17520069.3237072
SRR17520069.3249816
SRR17520069.3249818
SRR17520069.3249819
SRR17520069.3255852
SRR17520069.3255854
SRR17520069.3255855
SRR17520069.3255856
SRR17520069.3255858
SRR17520069.3255859
SRR17520069.3255860
SRR17520069.3255861
SRR17520069.3255862
SRR17520069.3255863
SRR17520069.3255864
SRR17520069.3255866
SRR17520069.3255867
SRR17520069.3255868
SRR17520069.3255870
SRR17520069.3255872
SRR17520069.3255874
SRR17520069.3255875
SRR17520069.3258020
SRR17520069.3260532
SRR17520069.3260533
SRR17520069.3260534
SRR17520069.3260535
SRR17520069.3260536
SRR17520069.3260537
SRR17520069.3260538
SRR17520069.3260539
SRR17520069.3260540
SRR17520069.3260541
SRR17520069.3260542
SRR17520069.3260543
SRR17520069.3260544
SRR17520069.3260545
SRR17520069.3260546
SRR17520069.3260547
SRR17520069.3260548
SRR17520069.3260549
SRR17520069.3260550
SRR17520069.3260551
SRR17520069.3260552
SRR17520069.3260553
SRR17520069.3260554
SRR17520069.3260555

SRR17520069.3260556
SRR17520069.3260558
SRR17520069.3260559
SRR17520069.3260560
SRR17520069.3260561
SRR17520069.3260562
SRR17520069.3260563
SRR17520069.3260564
SRR17520069.3260565
SRR17520069.3260566
SRR17520069.3260567
SRR17520069.3260568
SRR17520069.3260569
SRR17520069.3260570
SRR17520069.3260571
SRR17520069.3260572
SRR17520069.3260573
SRR17520069.3260574
SRR17520069.3260575
SRR17520069.3260576
SRR17520069.3260577
SRR17520069.3260578
SRR17520069.3260579
SRR17520069.3260580
SRR17520069.3260581
SRR17520069.3260582
SRR17520069.3260583
SRR17520069.3260584
SRR17520069.3260585
SRR17520069.3260586
SRR17520069.3273009
SRR17520069.3273018
SRR17520069.3273019
SRR17520069.3273023
SRR17520069.3324386
SRR17520069.3324387
SRR17520069.3324388
SRR17520069.3324389
SRR17520069.3324390
SRR17520069.3324391
SRR17520069.3324392
SRR17520069.3324393
SRR17520069.3324394
SRR17520069.3324395
SRR17520069.3324396
SRR17520069.3324397
SRR17520069.3324398

SRR17520069.3324399
SRR17520069.3324400
SRR17520069.3324401
SRR17520069.3324402
SRR17520069.3324403
SRR17520069.3324404
SRR17520069.3324405
SRR17520069.3324406
SRR17520069.3324407
SRR17520069.3332966
SRR17520069.3332967
SRR17520069.3332968
SRR17520069.3332969
SRR17520069.3332970
SRR17520069.3332971
SRR17520069.3332972
SRR17520069.3332973
SRR17520069.3332974
SRR17520069.3332975
SRR17520069.3332976
SRR17520069.3332977
SRR17520069.3332978
SRR17520069.3332979
SRR17520069.3332980
SRR17520069.3332981
SRR17520069.3332982
SRR17520069.3332983
SRR17520069.3332984
SRR17520069.3332985
SRR17520069.3332986
SRR17520069.3332987
SRR17520069.3332988
SRR17520069.3332989
SRR17520069.3332991
SRR17520069.3332992
SRR17520069.3332993
SRR17520069.3332994
SRR17520069.3332995
SRR17520069.3332996
SRR17520069.3332997
SRR17520069.3332998
SRR17520069.3332999
SRR17520069.3333000
SRR17520069.3333001
SRR17520069.3333002
SRR17520069.3333003
SRR17520069.3333004

SRR17520069.3333005
SRR17520069.3333006
SRR17520069.3333007
SRR17520069.3333008
SRR17520069.3333009
SRR17520069.3348865
SRR17520069.3348866
SRR17520069.3348867
SRR17520069.3348868
SRR17520069.3348869
SRR17520069.3348870
SRR17520069.3348871
SRR17520069.3348872
SRR17520069.3348873
SRR17520069.3348874
SRR17520069.3348875
SRR17520069.3348876
SRR17520069.3348877
SRR17520069.3348878
SRR17520069.3348879
SRR17520069.3348880
SRR17520069.3348881
SRR17520069.3348882
SRR17520069.3348883
SRR17520069.3348884
SRR17520069.3348885
SRR17520069.3348886
SRR17520069.3348887
SRR17520069.3348888
SRR17520069.3348889
SRR17520069.3348890
SRR17520069.3348891
SRR17520069.3348892
SRR17520069.3348893
SRR17520069.3348894
SRR17520069.3348895
SRR17520069.3348896
SRR17520069.3348897
SRR17520069.3348898
SRR17520069.3348900
SRR17520069.3348901
SRR17520069.3369648
SRR17520069.3369649
SRR17520069.3369650
SRR17520069.3369651
SRR17520069.3369652
SRR17520069.3369653

SRR17520069.3369654
SRR17520069.3369655
SRR17520069.3369656
SRR17520069.3369657
SRR17520069.3369658
SRR17520069.3369659
SRR17520069.3369660
SRR17520069.3369661
SRR17520069.3369662
SRR17520069.3409415
SRR17520069.3409416
SRR17520069.3409417
SRR17520069.3409418
SRR17520069.3409419
SRR17520069.3409420
SRR17520069.3409421
SRR17520069.3409422
SRR17520069.3409423
SRR17520069.3409424
SRR17520069.3409425
SRR17520069.3409426
SRR17520069.3409427
SRR17520069.3409428
SRR17520069.3409429
SRR17520069.3409430
SRR17520069.3409431
SRR17520069.3409432
SRR17520069.3409433
SRR17520069.3409434
SRR17520069.3409436
SRR17520069.3409437
SRR17520069.3409438
SRR17520069.3409439
SRR17520069.3409440
SRR17520069.3409441
SRR17520069.3409442
SRR17520069.3409443
SRR17520069.3409444
SRR17520069.3409445
SRR17520069.3409446
SRR17520069.3409448
SRR17520069.3409449
SRR17520069.3409450
SRR17520069.3409451
SRR17520069.3409452
SRR17520069.3409453
SRR17520069.3409454

SRR17520069.3409455
SRR17520069.3409456
SRR17520069.3409457
SRR17520069.3409458
SRR17520069.3409459
SRR17520069.3409460
SRR17520069.3409461
SRR17520069.3409462
SRR17520069.3409463
SRR17520069.3409464
SRR17520069.3409465
SRR17520069.3409466
SRR17520069.3409467
SRR17520069.3409468
SRR17520069.3409469
SRR17520069.3409470
SRR17520069.3409471
SRR17520069.3409472
SRR17520069.3409473
SRR17520069.3409474
SRR17520069.3414943
SRR17520069.3414944
SRR17520069.3414945
SRR17520069.3414946
SRR17520069.3414948
SRR17520069.3414949
SRR17520069.3414950
SRR17520069.3414951
SRR17520069.3414952
SRR17520069.3414953
SRR17520069.3414954
SRR17520069.3414955
SRR17520069.3414956
SRR17520069.3414957
SRR17520069.3414958
SRR17520069.3414959
SRR17520069.3414960
SRR17520069.3414961
SRR17520069.3414962
SRR17520069.3414963
SRR17520069.3414964
SRR17520069.3414965
SRR17520069.3414966
SRR17520069.3414967
SRR17520069.3414968
SRR17520069.3414970
SRR17520069.3414971

SRR17520069.3414972
SRR17520069.3414973
SRR17520069.3414974
SRR17520069.3414976
SRR17520069.3414977
SRR17520069.3414980
SRR17520069.3414981
SRR17520069.3414982
SRR17520069.3414983
SRR17520069.3414984
SRR17520069.3414985
SRR17520069.3414986
SRR17520069.3414987
SRR17520069.3414988
SRR17520069.3414989
SRR17520069.3414990
SRR17520069.3414991
SRR17520069.3437701
SRR17520069.3443258
SRR17520069.3443259
SRR17520069.3443260
SRR17520069.3443264
SRR17520069.3443266
SRR17520069.3443268
SRR17520069.3462379
SRR17520069.3465344
SRR17520069.3465345
SRR17520069.3465346
SRR17520069.3465347
SRR17520069.3465348
SRR17520069.3465349
SRR17520069.3465350
SRR17520069.3465351
SRR17520069.3465352
SRR17520069.3465353
SRR17520069.3465354
SRR17520069.3465355
SRR17520069.3465356
SRR17520069.3465357
SRR17520069.3465358
SRR17520069.3465359
SRR17520069.3465360
SRR17520069.3465361
SRR17520069.3465362
SRR17520069.3465363
SRR17520069.3465364
SRR17520069.3465365

SRR17520069.3465366
SRR17520069.3465367
SRR17520069.3465368
SRR17520069.3465369
SRR17520069.3465370
SRR17520069.3465371
SRR17520069.3465372
SRR17520069.3465373
SRR17520069.3465374
SRR17520069.3465375
SRR17520069.3465376
SRR17520069.3465377
SRR17520069.3465378
SRR17520069.3465379
SRR17520069.3465380
SRR17520069.3465381
SRR17520069.3465382
SRR17520069.3465383
SRR17520069.3465384
SRR17520069.3465385
SRR17520069.3465386
SRR17520069.3465387
SRR17520069.3465388
SRR17520069.3465390
SRR17520069.3465391
SRR17520069.3465392
SRR17520069.3465393
SRR17520069.3465394
SRR17520069.3465395
SRR17520069.3465396
SRR17520069.3465397
SRR17520069.3465398
SRR17520069.3465399
SRR17520069.3465400
SRR17520069.3465401
SRR17520069.3465402
SRR17520069.3465403
SRR17520069.3465404
SRR17520069.3465405
SRR17520069.3465406
SRR17520069.3465407
SRR17520069.3465408
SRR17520069.3465409
SRR17520069.3465410
SRR17520069.3465411
SRR17520069.3465412
SRR17520069.3465413

SRR17520069.3465414
SRR17520069.3465415
SRR17520069.3465425
SRR17520069.3465426
SRR17520069.3465427
SRR17520069.3465428
SRR17520069.3465429
SRR17520069.3465430
SRR17520069.3465431
SRR17520069.3465432
SRR17520069.3465433
SRR17520069.3465434
SRR17520069.3465435
SRR17520069.3465436
SRR17520069.3465437
SRR17520069.3465438
SRR17520069.3465439
SRR17520069.3465440
SRR17520069.3465441
SRR17520069.3465442
SRR17520069.3465443
SRR17520069.3465444
SRR17520069.3465445
SRR17520069.3465446
SRR17520069.3465447
SRR17520069.3465448
SRR17520069.3465449
SRR17520069.3465450
SRR17520069.3465451
SRR17520069.3465452
SRR17520069.3465453
SRR17520069.3465454
SRR17520069.3465455
SRR17520069.3465456
SRR17520069.3465457
SRR17520069.3465458
SRR17520069.3465460
SRR17520069.3465461
SRR17520069.3465463
SRR17520069.3465464
SRR17520069.3465465
SRR17520069.3465466
SRR17520069.3465467
SRR17520069.3465468
SRR17520069.3465469
SRR17520069.3465470
SRR17520069.3465471

SRR17520069.3465472
SRR17520069.3465474
SRR17520069.3465476
SRR17520069.3465477
SRR17520069.3471372
SRR17520069.3471373
SRR17520069.3471374
SRR17520069.3471375
SRR17520069.3471377
SRR17520069.3471378
SRR17520069.3471379
SRR17520069.3471381
SRR17520069.3471382
SRR17520069.3476110
SRR17520069.3476111
SRR17520069.3476114
SRR17520069.3476115
SRR17520069.3476116
SRR17520069.3476117
SRR17520069.3476118
SRR17520069.3476119
SRR17520069.3476120
SRR17520069.3476121
SRR17520069.3476122
SRR17520069.3476123
SRR17520069.3476124
SRR17520069.3476125
SRR17520069.3476126
SRR17520069.3476127
SRR17520069.3476128
SRR17520069.3476129
SRR17520069.3476130
SRR17520069.3476131
SRR17520069.3476132
SRR17520069.3476133
SRR17520069.3476134
SRR17520069.3476135
SRR17520069.3476136
SRR17520069.3476137
SRR17520069.3476138
SRR17520069.3476139
SRR17520069.3476140
SRR17520069.3476141
SRR17520069.3476142
SRR17520069.3476143
SRR17520069.3476144
SRR17520069.3476145

SRR17520069.3476147
SRR17520069.3476148
SRR17520069.3476149
SRR17520069.3476150
SRR17520069.3476151
SRR17520069.3476152
SRR17520069.3476153
SRR17520069.3476154
SRR17520069.3476155
SRR17520069.3476156
SRR17520069.3476157
SRR17520069.3476158
SRR17520069.3476159
SRR17520069.3476160
SRR17520069.3476161
SRR17520069.3476162
SRR17520069.3476163
SRR17520069.3476164
SRR17520069.3476165
SRR17520069.3476166
SRR17520069.3476167
SRR17520069.3476168
SRR17520069.3476169
SRR17520069.3476170
SRR17520069.3476171
SRR17520069.3476172
SRR17520069.3476173
SRR17520069.3476174
SRR17520069.3476175
SRR17520069.3476176
SRR17520069.3476177
SRR17520069.3476178
SRR17520069.3476179
SRR17520069.3476180
SRR17520069.3476181
SRR17520069.3476182
SRR17520069.3476184
SRR17520069.3476185
SRR17520069.3476186
SRR17520069.3476187
SRR17520069.3476188
SRR17520069.3476189
SRR17520069.3476190
SRR17520069.3476191
SRR17520069.3476192
SRR17520069.3476193
SRR17520069.3476194

SRR17520069.3476195
SRR17520069.3476196
SRR17520069.3476197
SRR17520069.3476198
SRR17520069.3476199
SRR17520069.3476200
SRR17520069.3476201
SRR17520069.3476202
SRR17520069.3476203
SRR17520069.3476204
SRR17520069.3476205
SRR17520069.3476206
SRR17520069.3476207
SRR17520069.3476209
SRR17520069.3476210
SRR17520069.3476211
SRR17520069.3476212
SRR17520069.3476213
SRR17520069.3476214
SRR17520069.3476215
SRR17520069.3476216
SRR17520069.3476217
SRR17520069.3476218
SRR17520069.3476219
SRR17520069.3476220
SRR17520069.3476221
SRR17520069.3476222
SRR17520069.3476223
SRR17520069.3476224
SRR17520069.3476225
SRR17520069.3476226
SRR17520069.3476227
SRR17520069.3476228
SRR17520069.3476229
SRR17520069.3476230
SRR17520069.3476231
SRR17520069.3476232
SRR17520069.3478088
SRR17520069.3478094
SRR17520069.3488481
SRR17520069.3488482
SRR17520069.3488483
SRR17520069.3488484
SRR17520069.3488485
SRR17520069.3488486
SRR17520069.3488487
SRR17520069.3488488

SRR17520069.3488489
SRR17520069.3488490
SRR17520069.3488492
SRR17520069.3488493
SRR17520069.3488494
SRR17520069.3488495
SRR17520069.3488496
SRR17520069.3488497
SRR17520069.3488498
SRR17520069.3488500
SRR17520069.3488501
SRR17520069.3488502
SRR17520069.3488503
SRR17520069.3488504
SRR17520069.3488505
SRR17520069.3488506
SRR17520069.3491554
SRR17520069.3501685
SRR17520069.3512093
SRR17520069.3512095
SRR17520069.3512096
SRR17520069.3512097
SRR17520069.3512098
SRR17520069.3512099
SRR17520069.3512100
SRR17520069.3512101
SRR17520069.3512102
SRR17520069.3512103
SRR17520069.3512104
SRR17520069.3512105
SRR17520069.3512106
SRR17520069.3512107
SRR17520069.3512108
SRR17520069.3512109
SRR17520069.3512110
SRR17520069.3512111
SRR17520069.3512112
SRR17520069.3512113
SRR17520069.3512114
SRR17520069.3512115
SRR17520069.3512116
SRR17520069.3512117
SRR17520069.3512118
SRR17520069.3512119
SRR17520069.3512120
SRR17520069.3512121
SRR17520069.3512122

SRR17520069.3512123
SRR17520069.3512124
SRR17520069.3512125
SRR17520069.3512126
SRR17520069.3512127
SRR17520069.3512128
SRR17520069.3512129
SRR17520069.3512130
SRR17520069.3512131
SRR17520069.3512132
SRR17520069.3512133
SRR17520069.3512134
SRR17520069.3512135
SRR17520069.3512137
SRR17520069.3512138
SRR17520069.3512139
SRR17520069.3512140
SRR17520069.3512141
SRR17520069.3512142
SRR17520069.3512143
SRR17520069.3512144
SRR17520069.3512145
SRR17520069.3512146
SRR17520069.3512147
SRR17520069.3512148
SRR17520069.3512149
SRR17520069.3512150
SRR17520069.3512151
SRR17520069.3512152
SRR17520069.3512153
SRR17520069.3512154
SRR17520069.3512155
SRR17520069.3512156
SRR17520069.3512157
SRR17520069.3512158
SRR17520069.3512159
SRR17520069.3512160
SRR17520069.3512161
SRR17520069.3512162
SRR17520069.3512164
SRR17520069.3512165
SRR17520069.3512166
SRR17520069.3512167
SRR17520069.3512168
SRR17520069.3512169
SRR17520069.3512170
SRR17520069.3512171

SRR17520069.3512172
SRR17520069.3512173
SRR17520069.3512174
SRR17520069.3512175
SRR17520069.3512176
SRR17520069.3512177
SRR17520069.3512178
SRR17520069.3512179
SRR17520069.3512180
SRR17520069.3512181
SRR17520069.3512182
SRR17520069.3512183
SRR17520069.3512184
SRR17520069.3512185
SRR17520069.3512186
SRR17520069.3512187
SRR17520069.3512188
SRR17520069.3512189
SRR17520069.3512190
SRR17520069.3512191
SRR17520069.3512192
SRR17520069.3512193
SRR17520069.3512194
SRR17520069.3512195
SRR17520069.3512196
SRR17520069.3512197
SRR17520069.3512198
SRR17520069.3512199
SRR17520069.3512200
SRR17520069.3512201
SRR17520069.3512203
SRR17520069.3512204
SRR17520069.3512205
SRR17520069.3512206
SRR17520069.3512207
SRR17520069.3512208
SRR17520069.3512209
SRR17520069.3512210
SRR17520069.3512211
SRR17520069.3512212
SRR17520069.3512213
SRR17520069.3512214
SRR17520069.3512215
SRR17520069.3512216
SRR17520069.3512217
SRR17520069.3512218
SRR17520069.3512219

SRR17520069.3512220
SRR17520069.3512221
SRR17520069.3512222
SRR17520069.3512223
SRR17520069.3512224
SRR17520069.3512225
SRR17520069.3512226
SRR17520069.3512228
SRR17520069.3512229
SRR17520069.3512230
SRR17520069.3512231
SRR17520069.3512232
SRR17520069.3512233
SRR17520069.3512234
SRR17520069.3512235
SRR17520069.3512236
SRR17520069.3512237
SRR17520069.3512238
SRR17520069.3512239
SRR17520069.3512240
SRR17520069.3512241
SRR17520069.3512242
SRR17520069.3512243
SRR17520069.3512244
SRR17520069.3512245
SRR17520069.3512246
SRR17520069.3512247
SRR17520069.3512248
SRR17520069.3512249
SRR17520069.3512250
SRR17520069.3512251
SRR17520069.3512253
SRR17520069.3512254
SRR17520069.3512255
SRR17520069.3512256
SRR17520069.3512257
SRR17520069.3512258
SRR17520069.3512259
SRR17520069.3512260
SRR17520069.3512261
SRR17520069.3512262
SRR17520069.3512263
SRR17520069.3512264
SRR17520069.3512265
SRR17520069.3512266
SRR17520069.3512267
SRR17520069.3512268

SRR17520069.3512269
SRR17520069.3512270
SRR17520069.3512271
SRR17520069.3512272
SRR17520069.3512273
SRR17520069.3512274
SRR17520069.3512275
SRR17520069.3512276
SRR17520069.3512277
SRR17520069.3512278
SRR17520069.3512279
SRR17520069.3512280
SRR17520069.3512281
SRR17520069.3512282
SRR17520069.3512283
SRR17520069.3512284
SRR17520069.3512285
SRR17520069.3512286
SRR17520069.3512287
SRR17520069.3512288
SRR17520069.3512289
SRR17520069.3512290
SRR17520069.3512291
SRR17520069.3512292
SRR17520069.3512293
SRR17520069.3512294
SRR17520069.3512295
SRR17520069.3512296
SRR17520069.3521627
SRR17520069.3539842
SRR17520069.3539934
SRR17520069.3539952
SRR17520069.3544669
SRR17520069.3553516
SRR17520069.3553517
SRR17520069.3553518
SRR17520069.3553519
SRR17520069.3553520
SRR17520069.3553521
SRR17520069.3553522
SRR17520069.3553523
SRR17520069.3553525
SRR17520069.3553526
SRR17520069.3553527
SRR17520069.3553528
SRR17520069.3553530
SRR17520069.3553531

SRR17520069.3568947
SRR17520069.3568955
SRR17520069.3573601
SRR17520069.3573782
SRR17520069.3578753
SRR17520069.3578754
SRR17520069.3578755
SRR17520069.3578756
SRR17520069.3578757
SRR17520069.3578758
SRR17520069.3578759
SRR17520069.3578760
SRR17520069.3578761
SRR17520069.3578762
SRR17520069.3578763
SRR17520069.3578764
SRR17520069.3578765
SRR17520069.3578766
SRR17520069.3578767
SRR17520069.3578768
SRR17520069.3578769
SRR17520069.3578770
SRR17520069.3578771
SRR17520069.3578772
SRR17520069.3578773
SRR17520069.3578774
SRR17520069.3578775
SRR17520069.3578776
SRR17520069.3578777
SRR17520069.3578778
SRR17520069.3578779
SRR17520069.3578780
SRR17520069.3578781
SRR17520069.3578782
SRR17520069.3578783
SRR17520069.3578784
SRR17520069.3578785
SRR17520069.3578786
SRR17520069.3578787
SRR17520069.3578788
SRR17520069.3578789
SRR17520069.3578790
SRR17520069.3578791
SRR17520069.3578792
SRR17520069.3578793
SRR17520069.3578796
SRR17520069.3578797

SRR17520069.3578798
SRR17520069.3578799
SRR17520069.3578800
SRR17520069.3578801
SRR17520069.3578802
SRR17520069.3578803
SRR17520069.3578804
SRR17520069.3578805
SRR17520069.3578806
SRR17520069.3578807
SRR17520069.3578808
SRR17520069.3578810
SRR17520069.3578812
SRR17520069.3578813
SRR17520069.3578814
SRR17520069.3578815
SRR17520069.3578816
SRR17520069.3578817
SRR17520069.3578818
SRR17520069.3578819
SRR17520069.3578820
SRR17520069.3578821
SRR17520069.3578822
SRR17520069.3578823
SRR17520069.3578824
SRR17520069.3578825
SRR17520069.3578826
SRR17520069.3578827
SRR17520069.3578828
SRR17520069.3578829
SRR17520069.3578830
SRR17520069.3578831
SRR17520069.3578833
SRR17520069.3578834
SRR17520069.3578835
SRR17520069.3578836
SRR17520069.3578837
SRR17520069.3578838
SRR17520069.3578839
SRR17520069.3578840
SRR17520069.3578841
SRR17520069.3578842
SRR17520069.3578843
SRR17520069.3578844
SRR17520069.3578845
SRR17520069.3578846
SRR17520069.3586039

SRR17520069.3586040
SRR17520069.3586041
SRR17520069.3586042
SRR17520069.3586043
SRR17520069.3586044
SRR17520069.3586045
SRR17520069.3586046
SRR17520069.3586047
SRR17520069.3586048
SRR17520069.3586049
SRR17520069.3586050
SRR17520069.3586051
SRR17520069.3586052
SRR17520069.3586053
SRR17520069.3586054
SRR17520069.3586055
SRR17520069.3586056
SRR17520069.3586057
SRR17520069.3586058
SRR17520069.3588623
SRR17520069.3588626
SRR17520069.3588627
SRR17520069.3588628
SRR17520069.3588629
SRR17520069.3588630
SRR17520069.3588631
SRR17520069.3588632
SRR17520069.3588633
SRR17520069.3588634
SRR17520069.3588635
SRR17520069.3588636
SRR17520069.3588638
SRR17520069.3588639
SRR17520069.3588640
SRR17520069.3588641
SRR17520069.3588642
SRR17520069.3588643
SRR17520069.3588644
SRR17520069.3588645
SRR17520069.3588646
SRR17520069.3588647
SRR17520069.3588648
SRR17520069.3588649
SRR17520069.3588650
SRR17520069.3588651
SRR17520069.3588652
SRR17520069.3588653

SRR17520069.3588654
SRR17520069.3630078
SRR17520069.3630079
SRR17520069.3630080
SRR17520069.3630081
SRR17520069.3630082
SRR17520069.3630083
SRR17520069.3630084
SRR17520069.3630085
SRR17520069.3630086
SRR17520069.3630087
SRR17520069.3630088
SRR17520069.3630089
SRR17520069.3630090
SRR17520069.3630091
SRR17520069.3630092
SRR17520069.3630093
SRR17520069.3630094
SRR17520069.3630095
SRR17520069.3630096
SRR17520069.3630097
SRR17520069.3630099
SRR17520069.3630101
SRR17520069.3630102
SRR17520069.3630103
SRR17520069.3630107
SRR17520069.3630109
SRR17520069.3630110
SRR17520069.3630111
SRR17520069.3630113
SRR17520069.3630117
SRR17520069.3630120
SRR17520069.3630122
SRR17520069.3630123
SRR17520069.3630128
SRR17520069.3630129
SRR17520069.3630130
SRR17520069.3630131
SRR17520069.3630132
SRR17520069.3630133
SRR17520069.3630134
SRR17520069.3630135
SRR17520069.3630136
SRR17520069.3630137
SRR17520069.3630138
SRR17520069.3630139
SRR17520069.3630140

SRR17520069.3630141
SRR17520069.3630142
SRR17520069.3636801
SRR17520069.3636802
SRR17520069.3636803
SRR17520069.3636804
SRR17520069.3636805
SRR17520069.3636806
SRR17520069.3636807
SRR17520069.3636808
SRR17520069.3636809
SRR17520069.3636810
SRR17520069.3636811
SRR17520069.3636812
SRR17520069.3636813
SRR17520069.3636814
SRR17520069.3636815
SRR17520069.3636816
SRR17520069.3636817
SRR17520069.3636818
SRR17520069.3636819
SRR17520069.3636820
SRR17520069.3636822
SRR17520069.3636823
SRR17520069.3636824
SRR17520069.3636825
SRR17520069.3638453
SRR17520069.3638454
SRR17520069.3638455
SRR17520069.3638456
SRR17520069.3638457
SRR17520069.3638458
SRR17520069.3638459
SRR17520069.3638460
SRR17520069.3638461
SRR17520069.3638462
SRR17520069.3638463
SRR17520069.3638464
SRR17520069.3638465
SRR17520069.3638466
SRR17520069.3638467
SRR17520069.3638468
SRR17520069.3638469
SRR17520069.3638470
SRR17520069.3638471
SRR17520069.3638472
SRR17520069.3638473

SRR17520069.3638474
SRR17520069.3638475
SRR17520069.3638476
SRR17520069.3638477
SRR17520069.3638478
SRR17520069.3638479
SRR17520069.3638480
SRR17520069.3638481
SRR17520069.3638482
SRR17520069.3638483
SRR17520069.3638484
SRR17520069.3638486
SRR17520069.3638487
SRR17520069.3638488
SRR17520069.3638489
SRR17520069.3638490
SRR17520069.3638491
SRR17520069.3638492
SRR17520069.3638493
SRR17520069.3638494
SRR17520069.3638495
SRR17520069.3638496
SRR17520069.3638497
SRR17520069.3638498
SRR17520069.3638499
SRR17520069.3638500
SRR17520069.3638501
SRR17520069.3638502
SRR17520069.3638503
SRR17520069.3638504
SRR17520069.3638505
SRR17520069.3638506
SRR17520069.3638507
SRR17520069.3638508
SRR17520069.3638509
SRR17520069.3638510
SRR17520069.3638511
SRR17520069.3638512
SRR17520069.3638513
SRR17520069.3638514
SRR17520069.3638515
SRR17520069.3638516
SRR17520069.3638517
SRR17520069.3638518
SRR17520069.3638519
SRR17520069.3638520
SRR17520069.3638521

SRR17520069.3638522
SRR17520069.3638523
SRR17520069.3638524
SRR17520069.3638525
SRR17520069.3638526
SRR17520069.3638527
SRR17520069.3638528
SRR17520069.3638529
SRR17520069.3638530
SRR17520069.3638531
SRR17520069.3638532
SRR17520069.3638533
SRR17520069.3638534
SRR17520069.3638535
SRR17520069.3638536
SRR17520069.3638537
SRR17520069.3644448
SRR17520069.3644449
SRR17520069.3644450
SRR17520069.3660895
SRR17520069.3660896
SRR17520069.3660897
SRR17520069.3660898
SRR17520069.3660899
SRR17520069.3660900
SRR17520069.3660901
SRR17520069.3660902
SRR17520069.3660903
SRR17520069.3660904
SRR17520069.3660905
SRR17520069.3660906
SRR17520069.3660907
SRR17520069.3660908
SRR17520069.3660909
SRR17520069.3660910
SRR17520069.3660911
SRR17520069.3660912
SRR17520069.3660913
SRR17520069.3660914
SRR17520069.3660915
SRR17520069.3660916
SRR17520069.3660917
SRR17520069.3660918
SRR17520069.3660919
SRR17520069.3660920
SRR17520069.3660921
SRR17520069.3660922

SRR17520069.3660923
SRR17520069.3660924
SRR17520069.3660925
SRR17520069.3660926
SRR17520069.3660927
SRR17520069.3660928
SRR17520069.3660929
SRR17520069.3660930
SRR17520069.3660931
SRR17520069.3660932
SRR17520069.3662702
SRR17520069.3668032
SRR17520069.3668507
SRR17520069.3669703
SRR17520069.3669704
SRR17520069.3669705
SRR17520069.3669706
SRR17520069.3669707
SRR17520069.3669708
SRR17520069.3669709
SRR17520069.3669710
SRR17520069.3669711
SRR17520069.3688361
SRR17520069.3702757
SRR17520069.3704588
SRR17520069.3704589
SRR17520069.3704590
SRR17520069.3704591
SRR17520069.3704592
SRR17520069.3704593
SRR17520069.3704594
SRR17520069.3706530
SRR17520069.3706531
SRR17520069.3706532
SRR17520069.3706533
SRR17520069.3706534
SRR17520069.3706535
SRR17520069.3710458
SRR17520069.3716033
SRR17520069.3716034
SRR17520069.3716035
SRR17520069.3716036
SRR17520069.3716037
SRR17520069.3716039
SRR17520069.3716040
SRR17520069.3716041
SRR17520069.3716042

SRR17520069.3738609
SRR17520069.3738610
SRR17520069.3738611
SRR17520069.3738612
SRR17520069.3738613
SRR17520069.3738614
SRR17520069.3738615
SRR17520069.3738616
SRR17520069.3738617
SRR17520069.3738618
SRR17520069.3738619
SRR17520069.3738620
SRR17520069.3738621
SRR17520069.3738622
SRR17520069.3738623
SRR17520069.3738624
SRR17520069.3766847
SRR17520069.3772443
SRR17520069.3795334
SRR17520069.3795335
SRR17520069.3795336
SRR17520069.3795337
SRR17520069.3795338
SRR17520069.3795339
SRR17520069.3795340
SRR17520069.3795341
SRR17520069.3795342
SRR17520069.3795346
SRR17520069.3795347
SRR17520069.3795348
SRR17520069.3795349
SRR17520069.3795350
SRR17520069.3795351
SRR17520069.3795352
SRR17520069.3795353
SRR17520069.3795356
SRR17520069.3795357
SRR17520069.3795359
SRR17520069.3795360
SRR17520069.3795361
SRR17520069.3795362
SRR17520069.3795365
SRR17520069.3795366
SRR17520069.3795367
SRR17520069.3809623
SRR17520069.3809624
SRR17520069.3809625

SRR17520069.3809626
SRR17520069.3809627
SRR17520069.3809628
SRR17520069.3809629
SRR17520069.3809630
SRR17520069.3820081
SRR17520069.3820082
SRR17520069.3820083
SRR17520069.3820084
SRR17520069.3820085
SRR17520069.3838510
SRR17520069.3838511
SRR17520069.3838512
SRR17520069.3838513
SRR17520069.3838514
SRR17520069.3838515
SRR17520069.3838516
SRR17520069.3838517
SRR17520069.3838518
SRR17520069.3838519
SRR17520069.3838520
SRR17520069.3838521
SRR17520069.3838522
SRR17520069.3838523
SRR17520069.3838524
SRR17520069.3838525
SRR17520069.3838526
SRR17520069.3838527
SRR17520069.3838528
SRR17520069.3838529
SRR17520069.3838530
SRR17520069.3838531
SRR17520069.3838532
SRR17520069.3838533
SRR17520069.3838534
SRR17520069.3838535
SRR17520069.3838536
SRR17520069.3838537
SRR17520069.3838538
SRR17520069.3838539
SRR17520069.3838540
SRR17520069.3838541
SRR17520069.3838542
SRR17520069.3838543
SRR17520069.3838544
SRR17520069.3838545
SRR17520069.3838546

SRR17520069.3838547
SRR17520069.3838548
SRR17520069.3838549
SRR17520069.3838550
SRR17520069.3838551
SRR17520069.3838552
SRR17520069.3838553
SRR17520069.3838554
SRR17520069.3838555
SRR17520069.3838556
SRR17520069.3838557
SRR17520069.3838558
SRR17520069.3838559
SRR17520069.3838560
SRR17520069.3838561
SRR17520069.3838563
SRR17520069.3838564
SRR17520069.3838565
SRR17520069.3838566
SRR17520069.3838567
SRR17520069.3838568
SRR17520069.3838569
SRR17520069.3838570
SRR17520069.3838571
SRR17520069.3838572
SRR17520069.3838573
SRR17520069.3838574
SRR17520069.3838575
SRR17520069.3838576
SRR17520069.3838577
SRR17520069.3838578
SRR17520069.3838579
SRR17520069.3838580
SRR17520069.3838581
SRR17520069.3838582
SRR17520069.3838583
SRR17520069.3838584
SRR17520069.3838585
SRR17520069.3838586
SRR17520069.3838587
SRR17520069.3838588
SRR17520069.3838589
SRR17520069.3838590
SRR17520069.3838591
SRR17520069.3838592
SRR17520069.3838594
SRR17520069.3838595

SRR17520069.3838596
SRR17520069.3838597
SRR17520069.3838598
SRR17520069.3838599
SRR17520069.3838600
SRR17520069.3838601
SRR17520069.3838602
SRR17520069.3838603
SRR17520069.3838604
SRR17520069.3838605
SRR17520069.3838606
SRR17520069.3838607
SRR17520069.3838608
SRR17520069.3838609
SRR17520069.3838610
SRR17520069.3838611
SRR17520069.3838612
SRR17520069.3838613
SRR17520069.3838614
SRR17520069.3838615
SRR17520069.3838616
SRR17520069.3838617
SRR17520069.3838618
SRR17520069.3838619
SRR17520069.3838620
SRR17520069.3838621
SRR17520069.3838622
SRR17520069.3838623
SRR17520069.3858109
SRR17520069.3858110
SRR17520069.3858111
SRR17520069.3858113
SRR17520069.3858114
SRR17520069.3858115
SRR17520069.3858116
SRR17520069.3858117
SRR17520069.3858118
SRR17520069.3858119
SRR17520069.3858120
SRR17520069.3858121
SRR17520069.3858122
SRR17520069.3858123
SRR17520069.3858124
SRR17520069.3858125
SRR17520069.3858126
SRR17520069.3858127
SRR17520069.3858128

SRR17520069.3858129
SRR17520069.3858130
SRR17520069.3858131
SRR17520069.3858133
SRR17520069.3858134
SRR17520069.3858135
SRR17520069.3858136
SRR17520069.3858137
SRR17520069.3858138
SRR17520069.3858139
SRR17520069.3858140
SRR17520069.3858141
SRR17520069.3858142
SRR17520069.3858144
SRR17520069.3858145
SRR17520069.3858146
SRR17520069.3858147
SRR17520069.3858148
SRR17520069.3858149
SRR17520069.3858150
SRR17520069.3858151
SRR17520069.3858152
SRR17520069.3858154
SRR17520069.3858155
SRR17520069.3858156
SRR17520069.3858157
SRR17520069.3858158
SRR17520069.3858159
SRR17520069.3858160
SRR17520069.3858161
SRR17520069.3858162
SRR17520069.3858163
SRR17520069.3858165
SRR17520069.3858167
SRR17520069.3858168
SRR17520069.3858169
SRR17520069.3858170
SRR17520069.3858171
SRR17520069.3858172
SRR17520069.3858173
SRR17520069.3858174
SRR17520069.3858176
SRR17520069.3858177
SRR17520069.3858178
SRR17520069.3858179
SRR17520069.3858181
SRR17520069.3858183

SRR17520069.3858184
SRR17520069.3858185
SRR17520069.3858186
SRR17520069.3858187
SRR17520069.3863455
SRR17520069.3866257
SRR17520069.3881547
SRR17520069.3888043
SRR17520069.3888044
SRR17520069.3888045
SRR17520069.3911716
SRR17520069.3911717
SRR17520069.3911718
SRR17520069.3911720
SRR17520069.3911721
SRR17520069.3911722
SRR17520069.3911723
SRR17520069.3911724
SRR17520069.3911725
SRR17520069.3911726
SRR17520069.3911730
SRR17520069.3911735
SRR17520069.3911738
SRR17520069.3911742
SRR17520069.3911754
SRR17520069.3913915
SRR17520069.3924493
SRR17520069.3924494
SRR17520069.3924495
SRR17520069.3924496
SRR17520069.3924731
SRR17520069.3924746
SRR17520069.3924819
SRR17520069.3930330
SRR17520069.3932308
SRR17520069.3932309
SRR17520069.3932310
SRR17520069.3932311
SRR17520069.3932312
SRR17520069.3932313
SRR17520069.3932314
SRR17520069.3932315
SRR17520069.3932316
SRR17520069.3932317
SRR17520069.3932319
SRR17520069.3932320
SRR17520069.3932321

SRR17520069.3932322
SRR17520069.3932323
SRR17520069.3932324
SRR17520069.3932325
SRR17520069.3932326
SRR17520069.3932327
SRR17520069.3932328
SRR17520069.3932329
SRR17520069.3932330
SRR17520069.3932331
SRR17520069.3932332
SRR17520069.3932333
SRR17520069.3932334
SRR17520069.3932335
SRR17520069.3932337
SRR17520069.3932338
SRR17520069.3932339
SRR17520069.3932340
SRR17520069.3932341
SRR17520069.3932342
SRR17520069.3932343
SRR17520069.3932344
SRR17520069.3932345
SRR17520069.3932347
SRR17520069.3932348
SRR17520069.3932349
SRR17520069.3932350
SRR17520069.3932351
SRR17520069.3932352
SRR17520069.3932354
SRR17520069.3940198
SRR17520069.3940199
SRR17520069.3940200
SRR17520069.3940201
SRR17520069.3940202
SRR17520069.3940203
SRR17520069.3940204
SRR17520069.3940205
SRR17520069.3940206
SRR17520069.3940207
SRR17520069.3940208
SRR17520069.3940209
SRR17520069.3940210
SRR17520069.3940211
SRR17520069.3940212
SRR17520069.3940213
SRR17520069.3940214

SRR17520069.3940215
SRR17520069.3940216
SRR17520069.3940217
SRR17520069.3940218
SRR17520069.3940219
SRR17520069.3940220
SRR17520069.3940221
SRR17520069.3940222
SRR17520069.3940223
SRR17520069.3940224
SRR17520069.3940225
SRR17520069.3940226
SRR17520069.3940227
SRR17520069.3940228
SRR17520069.3940229
SRR17520069.3940230
SRR17520069.3940231
SRR17520069.3940232
SRR17520069.3940234
SRR17520069.3940235
SRR17520069.3940236
SRR17520069.3940237
SRR17520069.3940238
SRR17520069.3940239
SRR17520069.3940240
SRR17520069.3940241
SRR17520069.3940242
SRR17520069.3940243
SRR17520069.3940244
SRR17520069.3940245
SRR17520069.3940246
SRR17520069.3940247
SRR17520069.3940248
SRR17520069.3940249
SRR17520069.3940250
SRR17520069.3953077
SRR17520069.3953078
SRR17520069.3953079
SRR17520069.3953080
SRR17520069.3953081
SRR17520069.3953082
SRR17520069.3953083
SRR17520069.3953084
SRR17520069.3967023
SRR17520069.3967583
SRR17520069.3967584
SRR17520069.3967585

SRR17520069.3967586
SRR17520069.3967587
SRR17520069.3967588
SRR17520069.3967589
SRR17520069.3967590
SRR17520069.3967591
SRR17520069.3967592
SRR17520069.3967593
SRR17520069.3967594
SRR17520069.3967595
SRR17520069.3967596
SRR17520069.3967597
SRR17520069.3967598
SRR17520069.3967599
SRR17520069.3967600
SRR17520069.3967601
SRR17520069.3967602
SRR17520069.3967603
SRR17520069.3967604
SRR17520069.3967605
SRR17520069.3967606
SRR17520069.3967607
SRR17520069.3967608
SRR17520069.3967610
SRR17520069.3967611
SRR17520069.3967612
SRR17520069.3967613
SRR17520069.3967614
SRR17520069.3967615
SRR17520069.3967616
SRR17520069.3967617
SRR17520069.3967618
SRR17520069.3967619
SRR17520069.3967620
SRR17520069.3967621
SRR17520069.3967622
SRR17520069.3967623
SRR17520069.3967624
SRR17520069.3967625
SRR17520069.3967626
SRR17520069.3967627
SRR17520069.3967628
SRR17520069.3967629
SRR17520069.3967631
SRR17520069.3967632
SRR17520069.3967633
SRR17520069.3967634

SRR17520069.3967635
SRR17520069.3967636
SRR17520069.3967637
SRR17520069.3968309
SRR17520069.3996181
SRR17520069.4000566
SRR17520069.4000567
SRR17520069.4000568
SRR17520069.4000569
SRR17520069.4000570
SRR17520069.4000571
SRR17520069.4000572
SRR17520069.4000573
SRR17520069.4000574
SRR17520069.4000575
SRR17520069.4000576
SRR17520069.4000577
SRR17520069.4000578
SRR17520069.4001604
SRR17520069.4001606
SRR17520069.4001608
SRR17520069.4001609
SRR17520069.4001610
SRR17520069.4001611
SRR17520069.4001613
SRR17520069.4001614
SRR17520069.4001615
SRR17520069.4001617
SRR17520069.4018180
SRR17520069.4018182
SRR17520069.4018183
SRR17520069.4018184
SRR17520069.4018185
SRR17520069.4018186
SRR17520069.4018187
SRR17520069.4018188
SRR17520069.4018189
SRR17520069.4018190
SRR17520069.4018192
SRR17520069.4018193
SRR17520069.4018194
SRR17520069.4018195
SRR17520069.4018196
SRR17520069.4018197
SRR17520069.4018198
SRR17520069.4018199
SRR17520069.4018200

SRR17520069.4018201
SRR17520069.4018202
SRR17520069.4018203
SRR17520069.4018204
SRR17520069.4018205
SRR17520069.4018206
SRR17520069.4018207
SRR17520069.4018208
SRR17520069.4042144
SRR17520069.4042145
SRR17520069.4042146
SRR17520069.4042147
SRR17520069.4042148
SRR17520069.4042149
SRR17520069.4042150
SRR17520069.4042151
SRR17520069.4042152
SRR17520069.4042153
SRR17520069.4042154
SRR17520069.4042155
SRR17520069.4042157
SRR17520069.4042158
SRR17520069.4049605
SRR17520069.4049645
SRR17520069.4064683
SRR17520069.4071457
SRR17520069.4071458
SRR17520069.4072193
SRR17520069.4072194
SRR17520069.4072195
SRR17520069.4072196
SRR17520069.4072197
SRR17520069.4072198
SRR17520069.4072199
SRR17520069.4072200
SRR17520069.4072202
SRR17520069.4072203
SRR17520069.4072204
SRR17520069.4072206
SRR17520069.4072207
SRR17520069.4072208
SRR17520069.4072209
SRR17520069.4072210
SRR17520069.4072211
SRR17520069.4072212
SRR17520069.4072213
SRR17520069.4072214

SRR17520069.4072215
SRR17520069.4072216
SRR17520069.4072217
SRR17520069.4072218
SRR17520069.4072219
SRR17520069.4072220
SRR17520069.4072221
SRR17520069.4072223
SRR17520069.4072224
SRR17520069.4072226
SRR17520069.4072227
SRR17520069.4076997
SRR17520069.4076998
SRR17520069.4076999
SRR17520069.4077000
SRR17520069.4077001
SRR17520069.4077002
SRR17520069.4077003
SRR17520069.4077004
SRR17520069.4077005
SRR17520069.4077006
SRR17520069.4077007
SRR17520069.4077008
SRR17520069.4077010
SRR17520069.4077011
SRR17520069.4077012
SRR17520069.4077013
SRR17520069.4094495
SRR17520069.4094496
SRR17520069.4094497
SRR17520069.4094498
SRR17520069.4094499
SRR17520069.4094500
SRR17520069.4094501
SRR17520069.4094502
SRR17520069.4094503
SRR17520069.4094506
SRR17520069.4094507
SRR17520069.4094508
SRR17520069.4103237
SRR17520069.4103278
SRR17520069.4103283
SRR17520069.4103326
SRR17520069.4121578
SRR17520069.4121579
SRR17520069.4121580
SRR17520069.4121581

SRR17520069.4121582
SRR17520069.4121583
SRR17520069.4121584
SRR17520069.4121585
SRR17520069.4121586
SRR17520069.4121587
SRR17520069.4121588
SRR17520069.4121589
SRR17520069.4121590
SRR17520069.4121591
SRR17520069.4121592
SRR17520069.4121593
SRR17520069.4121594
SRR17520069.4121595
SRR17520069.4121596
SRR17520069.4121597
SRR17520069.4121599
SRR17520069.4121600
SRR17520069.4121601
SRR17520069.4121603
SRR17520069.4124618
SRR17520069.4124619
SRR17520069.4124620
SRR17520069.4124621
SRR17520069.4124622
SRR17520069.4124623
SRR17520069.4124624
SRR17520069.4124625
SRR17520069.4124626
SRR17520069.4133097
SRR17520069.4133098
SRR17520069.4133099
SRR17520069.4133100
SRR17520069.4133101
SRR17520069.4133103
SRR17520069.4133104
SRR17520069.4133105
SRR17520069.4133106
SRR17520069.4133107
SRR17520069.4133108
SRR17520069.4133109
SRR17520069.4133110
SRR17520069.4133111
SRR17520069.4133112
SRR17520069.4133113
SRR17520069.4133114
SRR17520069.4137678

SRR17520069.4137680
SRR17520069.4137681
SRR17520069.4137682
SRR17520069.4137683
SRR17520069.4137684
SRR17520069.4137685
SRR17520069.4137686
SRR17520069.4137687
SRR17520069.4137688
SRR17520069.4137689
SRR17520069.4137690
SRR17520069.4137691
SRR17520069.4137692
SRR17520069.4137693
SRR17520069.4137695
SRR17520069.4137696
SRR17520069.4137697
SRR17520069.4137698
SRR17520069.4137699
SRR17520069.4137700
SRR17520069.4137701
SRR17520069.4137702
SRR17520069.4137703
SRR17520069.4137704
SRR17520069.4137705
SRR17520069.4143077
SRR17520069.4143078
SRR17520069.4162534
SRR17520069.4164884
SRR17520069.4170357
SRR17520069.4173065
SRR17520069.4178809
SRR17520069.4180885
SRR17520069.4180886
SRR17520069.4180887
SRR17520069.4180888
SRR17520069.4180889
SRR17520069.4180890
SRR17520069.4180892
SRR17520069.4180894
SRR17520069.4180895
SRR17520069.4180896
SRR17520069.4180898
SRR17520069.4180900
SRR17520069.4180901
SRR17520069.4180902
SRR17520069.4180903

SRR17520069.4180904
SRR17520069.4180905
SRR17520069.4180906
SRR17520069.4180907
SRR17520069.4180910
SRR17520069.4180912
SRR17520069.4180913
SRR17520069.4180914
SRR17520069.4180915
SRR17520069.4180916
SRR17520069.4180917
SRR17520069.4180918
SRR17520069.4180920
SRR17520069.4180921
SRR17520069.4180922
SRR17520069.4180925
SRR17520069.4180928
SRR17520069.4180929
SRR17520069.4191679
SRR17520069.4205999
SRR17520069.4225249
SRR17520069.4232003
SRR17520069.4232039
SRR17520069.4232096
SRR17520069.4242381
SRR17520069.4245668
SRR17520069.4245669
SRR17520069.4245670
SRR17520069.4245671
SRR17520069.4245672
SRR17520069.4245673
SRR17520069.4245674
SRR17520069.4245675
SRR17520069.4245676
SRR17520069.4245677
SRR17520069.4245678
SRR17520069.4245679
SRR17520069.4245680
SRR17520069.4245681
SRR17520069.4245682
SRR17520069.4245683
SRR17520069.4245684
SRR17520069.4245685
SRR17520069.4245686
SRR17520069.4245687
SRR17520069.4245688
SRR17520069.4245689

SRR17520069.4245690
SRR17520069.4245691
SRR17520069.4245692
SRR17520069.4245693
SRR17520069.4245694
SRR17520069.4245695
SRR17520069.4245696
SRR17520069.4245697
SRR17520069.4245698
SRR17520069.4245699
SRR17520069.4253233
SRR17520069.4253234
SRR17520069.4253235
SRR17520069.4253236
SRR17520069.4253237
SRR17520069.4253238
SRR17520069.4253239
SRR17520069.4253240
SRR17520069.4253241
SRR17520069.4253242
SRR17520069.4253243
SRR17520069.4253244
SRR17520069.4253245
SRR17520069.4253246
SRR17520069.4253247
SRR17520069.4253248
SRR17520069.4253249
SRR17520069.4253250
SRR17520069.4260134
SRR17520069.4260135
SRR17520069.4260136
SRR17520069.4260137
SRR17520069.4260138
SRR17520069.4260139
SRR17520069.4260140
SRR17520069.4260141
SRR17520069.4260142
SRR17520069.4260143
SRR17520069.4260144
SRR17520069.4260145
SRR17520069.4260146
SRR17520069.4260147
SRR17520069.4260148
SRR17520069.4260149
SRR17520069.4260150
SRR17520069.4260152
SRR17520069.4260154

SRR17520069.4260156
SRR17520069.4262987
SRR17520069.4262988
SRR17520069.4262989
SRR17520069.4262990
SRR17520069.4262991
SRR17520069.4262992
SRR17520069.4262993
SRR17520069.4262994
SRR17520069.4262995
SRR17520069.4262996
SRR17520069.4262997
SRR17520069.4262999
SRR17520069.4263000
SRR17520069.4263001
SRR17520069.4263002
SRR17520069.4263003
SRR17520069.4263004
SRR17520069.4263005
SRR17520069.4263006
SRR17520069.4263007
SRR17520069.4263008
SRR17520069.4263009
SRR17520069.4263010
SRR17520069.4263012
SRR17520069.4263013
SRR17520069.4263014
SRR17520069.4263015
SRR17520069.4263016
SRR17520069.4263017
SRR17520069.4263018
SRR17520069.4263019
SRR17520069.4263020
SRR17520069.4263021
SRR17520069.4263022
SRR17520069.4263023
SRR17520069.4263024
SRR17520069.4263025
SRR17520069.4263026
SRR17520069.4263027
SRR17520069.4263028
SRR17520069.4263029
SRR17520069.4263030
SRR17520069.4263031
SRR17520069.4263032
SRR17520069.4263033
SRR17520069.4263034

SRR17520069.4263035
SRR17520069.4263036
SRR17520069.4263037
SRR17520069.4263038
SRR17520069.4263039
SRR17520069.4263040
SRR17520069.4263041
SRR17520069.4263042
SRR17520069.4269070
SRR17520069.4269071
SRR17520069.4269072
SRR17520069.4269073
SRR17520069.4269074
SRR17520069.4269075
SRR17520069.4269076
SRR17520069.4269077
SRR17520069.4269078
SRR17520069.4269079
SRR17520069.4269080
SRR17520069.4269081
SRR17520069.4269084
SRR17520069.4269085
SRR17520069.4269086
SRR17520069.4269088
SRR17520069.4269089
SRR17520069.4269090
SRR17520069.4269091
SRR17520069.4269092
SRR17520069.4269094
SRR17520069.4269095
SRR17520069.4269096
SRR17520069.4269097
SRR17520069.4269098
SRR17520069.4269099
SRR17520069.4269100
SRR17520069.4269101
SRR17520069.4269102
SRR17520069.4269103
SRR17520069.4272766
SRR17520069.4272769
SRR17520069.4272770
SRR17520069.4273067
SRR17520069.4273068
SRR17520069.4273069
SRR17520069.4273070
SRR17520069.4274484
SRR17520069.4274485

SRR17520069.4274486
SRR17520069.4274487
SRR17520069.4274488
SRR17520069.4274490
SRR17520069.4274491
SRR17520069.4274492
SRR17520069.4274494
SRR17520069.4274495
SRR17520069.4274496
SRR17520069.4274497
SRR17520069.4274498
SRR17520069.4274499
SRR17520069.4274500
SRR17520069.4274501
SRR17520069.4274502
SRR17520069.4274503
SRR17520069.4274504
SRR17520069.4274505
SRR17520069.4274506
SRR17520069.4274509
SRR17520069.4274510
SRR17520069.4274511
SRR17520069.4274512
SRR17520069.4274513
SRR17520069.4274514
SRR17520069.4274515
SRR17520069.4274517
SRR17520069.4274518
SRR17520069.4274519
SRR17520069.4274520
SRR17520069.4274522
SRR17520069.4274524
SRR17520069.4274525
SRR17520069.4274526
SRR17520069.4274528
SRR17520069.4274530
SRR17520069.4274531
SRR17520069.4274532
SRR17520069.4274533
SRR17520069.4274534
SRR17520069.4274536
SRR17520069.4274537
SRR17520069.4274538
SRR17520069.4274539
SRR17520069.4274540
SRR17520069.4274541
SRR17520069.4274542

SRR17520069.4274543
SRR17520069.4274545
SRR17520069.4274546
SRR17520069.4274547
SRR17520069.4274548
SRR17520069.4274549
SRR17520069.4274550
SRR17520069.4274551
SRR17520069.4274553
SRR17520069.4274554
SRR17520069.4274555
SRR17520069.4274556
SRR17520069.4274557
SRR17520069.4274558
SRR17520069.4274559
SRR17520069.4274560
SRR17520069.4274561
SRR17520069.4274562
SRR17520069.4274563
SRR17520069.4274564
SRR17520069.4274565
SRR17520069.4274566
SRR17520069.4274567
SRR17520069.4274569
SRR17520069.4274571
SRR17520069.4274572
SRR17520069.4274573
SRR17520069.4274574
SRR17520069.4274575
SRR17520069.4274576
SRR17520069.4274577
SRR17520069.4274578
SRR17520069.4274580
SRR17520069.4274581
SRR17520069.4274582
SRR17520069.4274584
SRR17520069.4274585
SRR17520069.4274586
SRR17520069.4274588
SRR17520069.4274589
SRR17520069.4274591
SRR17520069.4274592
SRR17520069.4274594
SRR17520069.4274596
SRR17520069.4274597
SRR17520069.4274598
SRR17520069.4274599

SRR17520069.4274600
SRR17520069.4274601
SRR17520069.4274604
SRR17520069.4274605
SRR17520069.4274606
SRR17520069.4274607
SRR17520069.4274608
SRR17520069.4274610
SRR17520069.4274614
SRR17520069.4274616
SRR17520069.4274617
SRR17520069.4274618
SRR17520069.4274619
SRR17520069.4274620
SRR17520069.4274621
SRR17520069.4274622
SRR17520069.4274623
SRR17520069.4274624
SRR17520069.4274625
SRR17520069.4274626
SRR17520069.4274627
SRR17520069.4274628
SRR17520069.4274630
SRR17520069.4274631
SRR17520069.4274632
SRR17520069.4274633
SRR17520069.4274634
SRR17520069.4274635
SRR17520069.4274636
SRR17520069.4274637
SRR17520069.4274638
SRR17520069.4274639
SRR17520069.4274640
SRR17520069.4274641
SRR17520069.4274642
SRR17520069.4274643
SRR17520069.4274644
SRR17520069.4274645
SRR17520069.4274646
SRR17520069.4274647
SRR17520069.4274648
SRR17520069.4274649
SRR17520069.4274651
SRR17520069.4274652
SRR17520069.4274653
SRR17520069.4274654
SRR17520069.4274656

SRR17520069.4274657
SRR17520069.4274659
SRR17520069.4274660
SRR17520069.4274661
SRR17520069.4274662
SRR17520069.4274663
SRR17520069.4274664
SRR17520069.4274665
SRR17520069.4274666
SRR17520069.4274667
SRR17520069.4274668
SRR17520069.4274669
SRR17520069.4274670
SRR17520069.4274671
SRR17520069.4274673
SRR17520069.4274674
SRR17520069.4274675
SRR17520069.4274676
SRR17520069.4274677
SRR17520069.4274678
SRR17520069.4274679
SRR17520069.4274681
SRR17520069.4274682
SRR17520069.4274683
SRR17520069.4274684
SRR17520069.4274685
SRR17520069.4274686
SRR17520069.4274687
SRR17520069.4274689
SRR17520069.4274690
SRR17520069.4274691
SRR17520069.4290030
SRR17520069.4316310
SRR17520069.4316311
SRR17520069.4316312
SRR17520069.4316313
SRR17520069.4316314
SRR17520069.4316315
SRR17520069.4323369
SRR17520069.4323370
SRR17520069.4323374
SRR17520069.4324361
SRR17520069.4329444
SRR17520069.4329501
SRR17520069.4329576
SRR17520069.4329585
SRR17520069.4342283

SRR17520069.4355824
SRR17520069.4356388
SRR17520069.4356389
SRR17520069.4356390
SRR17520069.4356391
SRR17520069.4356392
SRR17520069.4356393
SRR17520069.4356394
SRR17520069.4356395
SRR17520069.4356396
SRR17520069.4356397
SRR17520069.4356398
SRR17520069.4356399
SRR17520069.4356400
SRR17520069.4356401
SRR17520069.4356406
SRR17520069.4356408
SRR17520069.4356411
SRR17520069.4356412
SRR17520069.4356414
SRR17520069.4356426
SRR17520069.4369536
SRR17520069.4369537
SRR17520069.4369538
SRR17520069.4369539
SRR17520069.4369540
SRR17520069.4369541
SRR17520069.4369542
SRR17520069.4369543
SRR17520069.4369544
SRR17520069.4369545
SRR17520069.4369546
SRR17520069.4369547
SRR17520069.4369548
SRR17520069.4369549
SRR17520069.4369550
SRR17520069.4369551
SRR17520069.4369552
SRR17520069.4369553
SRR17520069.4369554
SRR17520069.4369555
SRR17520069.4369556
SRR17520069.4369557
SRR17520069.4369558
SRR17520069.4369559
SRR17520069.4369560
SRR17520069.4369561

SRR17520069.4369562
SRR17520069.4369563
SRR17520069.4369564
SRR17520069.4369565
SRR17520069.4369566
SRR17520069.4369567
SRR17520069.4369568
SRR17520069.4369569
SRR17520069.4369570
SRR17520069.4369571
SRR17520069.4369572
SRR17520069.4369573
SRR17520069.4369574
SRR17520069.4369575
SRR17520069.4369576
SRR17520069.4369577
SRR17520069.4369578
SRR17520069.4369579
SRR17520069.4369580
SRR17520069.4369581
SRR17520069.4369582
SRR17520069.4369583
SRR17520069.4369584
SRR17520069.4369585
SRR17520069.4369586
SRR17520069.4369587
SRR17520069.4369588
SRR17520069.4369589
SRR17520069.4369590
SRR17520069.4369591
SRR17520069.4369592
SRR17520069.4369593
SRR17520069.4369594
SRR17520069.4369595
SRR17520069.4369596
SRR17520069.4369597
SRR17520069.4369598
SRR17520069.4369599
SRR17520069.4369600
SRR17520069.4369601
SRR17520069.4369602
SRR17520069.4369603
SRR17520069.4369604
SRR17520069.4369605
SRR17520069.4369606
SRR17520069.4369607
SRR17520069.4369608

SRR17520069.4369609
SRR17520069.4369610
SRR17520069.4369611
SRR17520069.4369612
SRR17520069.4369613
SRR17520069.4369614
SRR17520069.4369615
SRR17520069.4369616
SRR17520069.4369617
SRR17520069.4369618
SRR17520069.4369619
SRR17520069.4369620
SRR17520069.4369621
SRR17520069.4369622
SRR17520069.4369623
SRR17520069.4369624
SRR17520069.4369625
SRR17520069.4369626
SRR17520069.4369627
SRR17520069.4369628
SRR17520069.4369629
SRR17520069.4369630
SRR17520069.4369631
SRR17520069.4369666
SRR17520069.4369667
SRR17520069.4369668
SRR17520069.4369669
SRR17520069.4369670
SRR17520069.4369671
SRR17520069.4369672
SRR17520069.4369673
SRR17520069.4369674
SRR17520069.4369675
SRR17520069.4369676
SRR17520069.4369677
SRR17520069.4369678
SRR17520069.4369679
SRR17520069.4369680
SRR17520069.4369681
SRR17520069.4369682
SRR17520069.4369683
SRR17520069.4369684
SRR17520069.4369685
SRR17520069.4369686
SRR17520069.4369687
SRR17520069.4369688
SRR17520069.4369689

SRR17520069.4369690
SRR17520069.4369691
SRR17520069.4369692
SRR17520069.4369693
SRR17520069.4369694
SRR17520069.4369695
SRR17520069.4369696
SRR17520069.4369697
SRR17520069.4369698
SRR17520069.4369699
SRR17520069.4369700
SRR17520069.4369701
SRR17520069.4369702
SRR17520069.4369703
SRR17520069.4369704
SRR17520069.4369705
SRR17520069.4369706
SRR17520069.4369707
SRR17520069.4369708
SRR17520069.4369709
SRR17520069.4373830
SRR17520069.4373831
SRR17520069.4373832
SRR17520069.4373833
SRR17520069.4373834
SRR17520069.4373835
SRR17520069.4373836
SRR17520069.4373838
SRR17520069.4373839
SRR17520069.4373840
SRR17520069.4373842
SRR17520069.4373843
SRR17520069.4373844
SRR17520069.4373845
SRR17520069.4373846
SRR17520069.4373847
SRR17520069.4373848
SRR17520069.4402759
SRR17520069.4411085
SRR17520069.4411086
SRR17520069.4411088
SRR17520069.4411089
SRR17520069.4411090
SRR17520069.4411091
SRR17520069.4411092
SRR17520069.4411093
SRR17520069.4411094

SRR17520069.4411095
SRR17520069.4411096
SRR17520069.4411097
SRR17520069.4411098
SRR17520069.4411099
SRR17520069.4411100
SRR17520069.4411101
SRR17520069.4411102
SRR17520069.4411103
SRR17520069.4411104
SRR17520069.4411105
SRR17520069.4411106
SRR17520069.4411107
SRR17520069.4411108
SRR17520069.4411109
SRR17520069.4411110
SRR17520069.4411111
SRR17520069.4411112
SRR17520069.4411113
SRR17520069.4411114
SRR17520069.4411115
SRR17520069.4411116
SRR17520069.4411117
SRR17520069.4411118
SRR17520069.4411119
SRR17520069.4416454
SRR17520069.4416455
SRR17520069.4416456
SRR17520069.4416457
SRR17520069.4416458
SRR17520069.4416459
SRR17520069.4416460
SRR17520069.4416461
SRR17520069.4416462
SRR17520069.4416463
SRR17520069.4416464
SRR17520069.4416465
SRR17520069.4416466
SRR17520069.4416467
SRR17520069.4416468
SRR17520069.4416469
SRR17520069.4416470
SRR17520069.4416471
SRR17520069.4416472
SRR17520069.4416474
SRR17520069.4416475
SRR17520069.4416476

SRR17520069.4416477
SRR17520069.4416478
SRR17520069.4416479
SRR17520069.4416480
SRR17520069.4416481
SRR17520069.4416482
SRR17520069.4416483
SRR17520069.4416484
SRR17520069.4416485
SRR17520069.4416486
SRR17520069.4416487
SRR17520069.4416488
SRR17520069.4416489
SRR17520069.4416490
SRR17520069.4416491
SRR17520069.4416492
SRR17520069.4416493
SRR17520069.4416494
SRR17520069.4416495
SRR17520069.4416496
SRR17520069.4416497
SRR17520069.4416498
SRR17520069.4416499
SRR17520069.4416500
SRR17520069.4416501
SRR17520069.4416502
SRR17520069.4416503
SRR17520069.4416504
SRR17520069.4416505
SRR17520069.4416506
SRR17520069.4416507
SRR17520069.4416508
SRR17520069.4416956
SRR17520069.4417055
SRR17520069.4417064
SRR17520069.4417066
SRR17520069.4417067
SRR17520069.4417068
SRR17520069.4417069
SRR17520069.4417070
SRR17520069.4417071
SRR17520069.4417072
SRR17520069.4437453
SRR17520069.4437454
SRR17520069.4437455
SRR17520069.4437456
SRR17520069.4437457

SRR17520069.4437458
SRR17520069.4441083
SRR17520069.4442527
SRR17520069.4442528
SRR17520069.4442529
SRR17520069.4442530
SRR17520069.4442531
SRR17520069.4442532
SRR17520069.4442533
SRR17520069.4442534
SRR17520069.4442536
SRR17520069.4442539
SRR17520069.4442540
SRR17520069.4442542
SRR17520069.4442543
SRR17520069.4442544
SRR17520069.4461257
SRR17520069.4461258
SRR17520069.4461259
SRR17520069.4461260
SRR17520069.4469291
SRR17520069.4469292
SRR17520069.4469293
SRR17520069.4469294
SRR17520069.4469295
SRR17520069.4469296
SRR17520069.4469297
SRR17520069.4469298
SRR17520069.4469299
SRR17520069.4469300
SRR17520069.4469301
SRR17520069.4469302
SRR17520069.4469303
SRR17520069.4469304
SRR17520069.4469305
SRR17520069.4469306
SRR17520069.4469307
SRR17520069.4469308
SRR17520069.4469309
SRR17520069.4469310
SRR17520069.4469311
SRR17520069.4469312
SRR17520069.4474447
SRR17520069.4480766
SRR17520069.4480789
SRR17520069.4482419
SRR17520069.4482420

SRR17520069.4482421
SRR17520069.4482422
SRR17520069.4482423
SRR17520069.4482425
SRR17520069.4482427
SRR17520069.4482428
SRR17520069.4482429
SRR17520069.4482430
SRR17520069.4482431
SRR17520069.4482432
SRR17520069.4482433
SRR17520069.4482435
SRR17520069.4482436
SRR17520069.4482437
SRR17520069.4482438
SRR17520069.4482439
SRR17520069.4482440
SRR17520069.4482441
SRR17520069.4482442
SRR17520069.4482443
SRR17520069.4482444
SRR17520069.4482445
SRR17520069.4482446
SRR17520069.4482447
SRR17520069.4482448
SRR17520069.4482449
SRR17520069.4482450
SRR17520069.4482451
SRR17520069.4482452
SRR17520069.4482453
SRR17520069.4482454
SRR17520069.4482455
SRR17520069.4482456
SRR17520069.4482457
SRR17520069.4485302
SRR17520069.4485303
SRR17520069.4485304
SRR17520069.4485305
SRR17520069.4485306
SRR17520069.4485307
SRR17520069.4485308
SRR17520069.4485309
SRR17520069.4485310
SRR17520069.4485311
SRR17520069.4485312
SRR17520069.4485313
SRR17520069.4485314

SRR17520069.4485315
SRR17520069.4485316
SRR17520069.4485317
SRR17520069.4485318
SRR17520069.4485319
SRR17520069.4485320
SRR17520069.4485321
SRR17520069.4485322
SRR17520069.4485323
SRR17520069.4485324
SRR17520069.4485325
SRR17520069.4485326
SRR17520069.4485327
SRR17520069.4485328
SRR17520069.4485329
SRR17520069.4485330
SRR17520069.4485331
SRR17520069.4485332
SRR17520069.4485333
SRR17520069.4485334
SRR17520069.4485335
SRR17520069.4485336
SRR17520069.4485337
SRR17520069.4485338
SRR17520069.4485339
SRR17520069.4485340
SRR17520069.4485341
SRR17520069.4485342
SRR17520069.4519347
SRR17520069.4526673
SRR17520069.4526674
SRR17520069.4526675
SRR17520069.4526676
SRR17520069.4526677
SRR17520069.4526678
SRR17520069.4526679
SRR17520069.4526680
SRR17520069.4526681
SRR17520069.4526682
SRR17520069.4526683
SRR17520069.4526684
SRR17520069.4526685
SRR17520069.4526686
SRR17520069.4526687
SRR17520069.4526688
SRR17520069.4526689
SRR17520069.4526690

SRR17520069.4526691
SRR17520069.4526692
SRR17520069.4526693
SRR17520069.4526694
SRR17520069.4526695
SRR17520069.4526696
SRR17520069.4526697
SRR17520069.4526698
SRR17520069.4526699
SRR17520069.4526700
SRR17520069.4526701
SRR17520069.4526702
SRR17520069.4526703
SRR17520069.4526704
SRR17520069.4526705
SRR17520069.4526706
SRR17520069.4526707
SRR17520069.4526708
SRR17520069.4526709
SRR17520069.4526710
SRR17520069.4526711
SRR17520069.4526712
SRR17520069.4526713
SRR17520069.4526714
SRR17520069.4526715
SRR17520069.4526716
SRR17520069.4526717
SRR17520069.4526718
SRR17520069.4526719
SRR17520069.4526720
SRR17520069.4526721
SRR17520069.4526722
SRR17520069.4526723
SRR17520069.4526724
SRR17520069.4526725
SRR17520069.4526726
SRR17520069.4526727
SRR17520069.4526728
SRR17520069.4526729
SRR17520069.4526730
SRR17520069.4526731
SRR17520069.4526732
SRR17520069.4526733
SRR17520069.4526734
SRR17520069.4526735
SRR17520069.4530005
SRR17520069.4530204

SRR17520069.4530239
SRR17520069.4537265
SRR17520069.4539757
SRR17520069.4539758
SRR17520069.4539759
SRR17520069.4539760
SRR17520069.4539761
SRR17520069.4539762
SRR17520069.4539764
SRR17520069.4539765
SRR17520069.4539766
SRR17520069.4539767
SRR17520069.4539768
SRR17520069.4539770
SRR17520069.4539773
SRR17520069.4539775
SRR17520069.4539776
SRR17520069.4539783
SRR17520069.4539793
SRR17520069.4539795
SRR17520069.4539800
SRR17520069.4539801
SRR17520069.4539802
SRR17520069.4539804
SRR17520069.4540628
SRR17520069.4540629
SRR17520069.4540630
SRR17520069.4540631
SRR17520069.4540632
SRR17520069.4540633
SRR17520069.4540636
SRR17520069.4540637
SRR17520069.4540638
SRR17520069.4549510
SRR17520069.4554430
SRR17520069.4580155
SRR17520069.4580156
SRR17520069.4580157
SRR17520069.4580158
SRR17520069.4580159
SRR17520069.4580160
SRR17520069.4580161
SRR17520069.4580162
SRR17520069.4580163
SRR17520069.4580164
SRR17520069.4580165
SRR17520069.4580166

SRR17520069.4580168
SRR17520069.4580169
SRR17520069.4580170
SRR17520069.4580171
SRR17520069.4580173
SRR17520069.4580174
SRR17520069.4580175
SRR17520069.4580176
SRR17520069.4580177
SRR17520069.4580178
SRR17520069.4580179
SRR17520069.4580180
SRR17520069.4580181
SRR17520069.4580182
SRR17520069.4580183
SRR17520069.4580184
SRR17520069.4580185
SRR17520069.4580186
SRR17520069.4580187
SRR17520069.4580188
SRR17520069.4580189
SRR17520069.4580190
SRR17520069.4580191
SRR17520069.4580192
SRR17520069.4580193
SRR17520069.4580194
SRR17520069.4580195
SRR17520069.4580196
SRR17520069.4580197
SRR17520069.4580198
SRR17520069.4580199
SRR17520069.4580200
SRR17520069.4580201
SRR17520069.4580202
SRR17520069.4580203
SRR17520069.4580204
SRR17520069.4580205
SRR17520069.4580206
SRR17520069.4580207
SRR17520069.4580208
SRR17520069.4580209
SRR17520069.4580210
SRR17520069.4580212
SRR17520069.4580213
SRR17520069.4580214
SRR17520069.4580215
SRR17520069.4580216

SRR17520069.4580217
SRR17520069.4580218
SRR17520069.4580219
SRR17520069.4580220
SRR17520069.4580221
SRR17520069.4580222
SRR17520069.4580223
SRR17520069.4580224
SRR17520069.4580225
SRR17520069.4580226
SRR17520069.4580227
SRR17520069.4580228
SRR17520069.4580229
SRR17520069.4580230
SRR17520069.4580231
SRR17520069.4580233
SRR17520069.4580235
SRR17520069.4580236
SRR17520069.4580237
SRR17520069.4580238
SRR17520069.4594949
SRR17520069.4594950
SRR17520069.4594951
SRR17520069.4594952
SRR17520069.4594953
SRR17520069.4594954
SRR17520069.4594955
SRR17520069.4594956
SRR17520069.4594957
SRR17520069.4594958
SRR17520069.4594959
SRR17520069.4594960
SRR17520069.4594961
SRR17520069.4594962
SRR17520069.4594963
SRR17520069.4594964
SRR17520069.4594965
SRR17520069.4594966
SRR17520069.4594967
SRR17520069.4594968
SRR17520069.4594970
SRR17520069.4594971
SRR17520069.4594972
SRR17520069.4594973
SRR17520069.4594974
SRR17520069.4594975
SRR17520069.4594976

SRR17520069.4594977
SRR17520069.4594978
SRR17520069.4594979
SRR17520069.4594980
SRR17520069.4594981
SRR17520069.4594982
SRR17520069.4594983
SRR17520069.4594984
SRR17520069.4594985
SRR17520069.4594986
SRR17520069.4594987
SRR17520069.4594988
SRR17520069.4594990
SRR17520069.4594991
SRR17520069.4594992
SRR17520069.4594993
SRR17520069.4594994
SRR17520069.4594995
SRR17520069.4594996
SRR17520069.4594997
SRR17520069.4594998
SRR17520069.4595000
SRR17520069.4595001
SRR17520069.4595002
SRR17520069.4595003
SRR17520069.4595004
SRR17520069.4595005
SRR17520069.4595006
SRR17520069.4595007
SRR17520069.4595008
SRR17520069.4595009
SRR17520069.4595010
SRR17520069.4595012
SRR17520069.4595013
SRR17520069.4595014
SRR17520069.4595015
SRR17520069.4595016
SRR17520069.4595017
SRR17520069.4595018
SRR17520069.4595019
SRR17520069.4595020
SRR17520069.4595021
SRR17520069.4595022
SRR17520069.4595023
SRR17520069.4595024
SRR17520069.4595025
SRR17520069.4595026

SRR17520069.4595027
SRR17520069.4595028
SRR17520069.4595029
SRR17520069.4595030
SRR17520069.4611249
SRR17520069.4611250
SRR17520069.4611251
SRR17520069.4611252
SRR17520069.4611253
SRR17520069.4611254
SRR17520069.4611255
SRR17520069.4611256
SRR17520069.4611257
SRR17520069.4611258
SRR17520069.4611259
SRR17520069.4611260
SRR17520069.4611261
SRR17520069.4611262
SRR17520069.4611263
SRR17520069.4611264
SRR17520069.4611265
SRR17520069.4611266
SRR17520069.4611267
SRR17520069.4611268
SRR17520069.4611269
SRR17520069.4611270
SRR17520069.4611271
SRR17520069.4611272
SRR17520069.4611273
SRR17520069.4611274
SRR17520069.4611275
SRR17520069.4611276
SRR17520069.4611277
SRR17520069.4611278
SRR17520069.4611279
SRR17520069.4611280
SRR17520069.4611281
SRR17520069.4611282
SRR17520069.4611283
SRR17520069.4611284
SRR17520069.4611285
SRR17520069.4611286
SRR17520069.4611287
SRR17520069.4611288
SRR17520069.4611289
SRR17520069.4611290
SRR17520069.4611291

SRR17520069.4611519
SRR17520069.4611520
SRR17520069.4611523
SRR17520069.4611524
SRR17520069.4616187
SRR17520069.4616188
SRR17520069.4616189
SRR17520069.4616190
SRR17520069.4616191
SRR17520069.4616192
SRR17520069.4616193
SRR17520069.4616194
SRR17520069.4616195
SRR17520069.4616196
SRR17520069.4616197
SRR17520069.4616198
SRR17520069.4616199
SRR17520069.4616200
SRR17520069.4616201
SRR17520069.4616202
SRR17520069.4616204
SRR17520069.4616205
SRR17520069.4616206
SRR17520069.4616207
SRR17520069.4616208
SRR17520069.4616209
SRR17520069.4616210
SRR17520069.4616211
SRR17520069.4616212
SRR17520069.4616213
SRR17520069.4616214
SRR17520069.4616215
SRR17520069.4616216
SRR17520069.4616217
SRR17520069.4616218
SRR17520069.4616219
SRR17520069.4616220
SRR17520069.4616221
SRR17520069.4616222
SRR17520069.4616223
SRR17520069.4616224
SRR17520069.4616225
SRR17520069.4616226
SRR17520069.4616227
SRR17520069.4616228
SRR17520069.4616229
SRR17520069.4616230

SRR17520069.4616231
SRR17520069.4616232
SRR17520069.4616233
SRR17520069.4616234
SRR17520069.4616235
SRR17520069.4616236
SRR17520069.4616237
SRR17520069.4616238
SRR17520069.4616239
SRR17520069.4616240
SRR17520069.4616241
SRR17520069.4616242
SRR17520069.4616243
SRR17520069.4616244
SRR17520069.4616245
SRR17520069.4616246
SRR17520069.4616247
SRR17520069.4616248
SRR17520069.4616249
SRR17520069.4616250
SRR17520069.4616251
SRR17520069.4616252
SRR17520069.4616253
SRR17520069.4616255
SRR17520069.4616256
SRR17520069.4616257
SRR17520069.4616258
SRR17520069.4616259
SRR17520069.4616260
SRR17520069.4616261
SRR17520069.4616263
SRR17520069.4616264
SRR17520069.4616265
SRR17520069.4616266
SRR17520069.4616267
SRR17520069.4616268
SRR17520069.4616269
SRR17520069.4616270
SRR17520069.4616271
SRR17520069.4616272
SRR17520069.4616273
SRR17520069.4616274
SRR17520069.4616275
SRR17520069.4616276
SRR17520069.4616277
SRR17520069.4616278
SRR17520069.4616279

SRR17520069.4616280
SRR17520069.4616281
SRR17520069.4616282
SRR17520069.4616283
SRR17520069.4616284
SRR17520069.4616285
SRR17520069.4616286
SRR17520069.4616287
SRR17520069.4616288
SRR17520069.4616289
SRR17520069.4616290
SRR17520069.4616291
SRR17520069.4616292
SRR17520069.4616294
SRR17520069.4616295
SRR17520069.4616296
SRR17520069.4616297
SRR17520069.4616298
SRR17520069.4616299
SRR17520069.4616300
SRR17520069.4616301
SRR17520069.4616302
SRR17520069.4616303
SRR17520069.4616304
SRR17520069.4616305
SRR17520069.4616306
SRR17520069.4616307
SRR17520069.4644890
SRR17520069.4644891
SRR17520069.4644892
SRR17520069.4644893
SRR17520069.4644894
SRR17520069.4644895
SRR17520069.4644899
SRR17520069.4644900
SRR17520069.4644901
SRR17520069.4644902
SRR17520069.4644903
SRR17520069.4644904
SRR17520069.4644905
SRR17520069.4644906
SRR17520069.4644907
SRR17520069.4665800
SRR17520069.4665803
SRR17520069.4674887
SRR17520069.4674899
SRR17520069.4677619

SRR17520069.4679302
SRR17520069.4689826
SRR17520069.4691612
SRR17520069.4691613
SRR17520069.4691614
SRR17520069.4691615
SRR17520069.4691616
SRR17520069.4691617
SRR17520069.4691618
SRR17520069.4691619
SRR17520069.4691620
SRR17520069.4691621
SRR17520069.4691622
SRR17520069.4703111
SRR17520069.4703112
SRR17520069.4703113
SRR17520069.4703114
SRR17520069.4703115
SRR17520069.4703116
SRR17520069.4703117
SRR17520069.4703118
SRR17520069.4703119
SRR17520069.4703120
SRR17520069.4703121
SRR17520069.4703122
SRR17520069.4703123
SRR17520069.4703124
SRR17520069.4703125
SRR17520069.4703126
SRR17520069.4703127
SRR17520069.4703128
SRR17520069.4703129
SRR17520069.4703130
SRR17520069.4703131
SRR17520069.4703132
SRR17520069.4703133
SRR17520069.4703134
SRR17520069.4703135
SRR17520069.4703136
SRR17520069.4703137
SRR17520069.4703138
SRR17520069.4703139
SRR17520069.4703140
SRR17520069.4703141
SRR17520069.4705837
SRR17520069.4711496
SRR17520069.4711497

SRR17520069.4711498
SRR17520069.4711499
SRR17520069.4711500
SRR17520069.4711501
SRR17520069.4711502
SRR17520069.4711503
SRR17520069.4711504
SRR17520069.4711505
SRR17520069.4711506
SRR17520069.4711508
SRR17520069.4711509
SRR17520069.4711510
SRR17520069.4711511
SRR17520069.4711512
SRR17520069.4711513
SRR17520069.4711514
SRR17520069.4711515
SRR17520069.4711516
SRR17520069.4711517
SRR17520069.4711518
SRR17520069.4720474
SRR17520069.4720475
SRR17520069.4720476
SRR17520069.4745741
SRR17520069.4745744
SRR17520069.4745746
SRR17520069.4745747
SRR17520069.4745748
SRR17520069.4745749
SRR17520069.4745750
SRR17520069.4745751
SRR17520069.4745752
SRR17520069.4745753
SRR17520069.4745754
SRR17520069.4745755
SRR17520069.4745756
SRR17520069.4745757
SRR17520069.4745758
SRR17520069.4745759
SRR17520069.4756629
SRR17520069.4756630
SRR17520069.4756631
SRR17520069.4756632
SRR17520069.4756633
SRR17520069.4756634
SRR17520069.4756635
SRR17520069.4756636

SRR17520069.4756637
SRR17520069.4756638
SRR17520069.4756639
SRR17520069.4756640
SRR17520069.4756641
SRR17520069.4756642
SRR17520069.4756643
SRR17520069.4756645
SRR17520069.4756646
SRR17520069.4756647
SRR17520069.4756648
SRR17520069.4756649
SRR17520069.4756650
SRR17520069.4756651
SRR17520069.4756652
SRR17520069.4756653
SRR17520069.4756654
SRR17520069.4756655
SRR17520069.4756656
SRR17520069.4756657
SRR17520069.4756658
SRR17520069.4756659
SRR17520069.4756660
SRR17520069.4756661
SRR17520069.4756662
SRR17520069.4756663
SRR17520069.4756664
SRR17520069.4756665
SRR17520069.4756666
SRR17520069.4756674
SRR17520069.4756676
SRR17520069.4756677
SRR17520069.4756678
SRR17520069.4756679
SRR17520069.4756680
SRR17520069.4756681
SRR17520069.4756684
SRR17520069.4756685
SRR17520069.4756686
SRR17520069.4756687
SRR17520069.4756688
SRR17520069.4756689
SRR17520069.4756690
SRR17520069.4756691
SRR17520069.4756692
SRR17520069.4756693
SRR17520069.4756694

SRR17520069.4756695
SRR17520069.4756696
SRR17520069.4756698
SRR17520069.4756699
SRR17520069.4756700
SRR17520069.4756701
SRR17520069.4756702
SRR17520069.4756703
SRR17520069.4756704
SRR17520069.4756705
SRR17520069.4756706
SRR17520069.4756707
SRR17520069.4756708
SRR17520069.4756709
SRR17520069.4756710
SRR17520069.4756711
SRR17520069.4756712
SRR17520069.4756713
SRR17520069.4756714
SRR17520069.4756715
SRR17520069.4756716
SRR17520069.4756717
SRR17520069.4756718
SRR17520069.4756719
SRR17520069.4756720
SRR17520069.4756721
SRR17520069.4756722
SRR17520069.4756723
SRR17520069.4756724
SRR17520069.4756725
SRR17520069.4756726
SRR17520069.4756727
SRR17520069.4756728
SRR17520069.4756729
SRR17520069.4756730
SRR17520069.4756732
SRR17520069.4756733
SRR17520069.4756734
SRR17520069.4756735
SRR17520069.4756736
SRR17520069.4756737
SRR17520069.4756738
SRR17520069.4756739
SRR17520069.4756740
SRR17520069.4756741
SRR17520069.4756742
SRR17520069.4756743

SRR17520069.4756744
SRR17520069.4756745
SRR17520069.4756746
SRR17520069.4756747
SRR17520069.4756748
SRR17520069.4756749
SRR17520069.4756750
SRR17520069.4756751
SRR17520069.4756752
SRR17520069.4756753
SRR17520069.4756755
SRR17520069.4756756
SRR17520069.4756757
SRR17520069.4756760
SRR17520069.4756761
SRR17520069.4756762
SRR17520069.4756763
SRR17520069.4756764
SRR17520069.4756765
SRR17520069.4756766
SRR17520069.4756767
SRR17520069.4756768
SRR17520069.4756769
SRR17520069.4756770
SRR17520069.4756771
SRR17520069.4763838
SRR17520069.4764497
SRR17520069.4764498
SRR17520069.4764499
SRR17520069.4764504
SRR17520069.4764511
SRR17520069.4764512
SRR17520069.4764513
SRR17520069.4764514
SRR17520069.4764515
SRR17520069.4764516
SRR17520069.4764517
SRR17520069.4764518
SRR17520069.4764519
SRR17520069.4764520
SRR17520069.4764521
SRR17520069.4764522
SRR17520069.4764523
SRR17520069.4764524
SRR17520069.4764525
SRR17520069.4764526
SRR17520069.4764527

SRR17520069.4764528
SRR17520069.4764529
SRR17520069.4764530
SRR17520069.4764531
SRR17520069.4764532
SRR17520069.4764533
SRR17520069.4764534
SRR17520069.4764535
SRR17520069.4764536
SRR17520069.4764537
SRR17520069.4764538
SRR17520069.4764539
SRR17520069.4764540
SRR17520069.4764541
SRR17520069.4764542
SRR17520069.4764543
SRR17520069.4764544
SRR17520069.4764545
SRR17520069.4764546
SRR17520069.4764547
SRR17520069.4764548
SRR17520069.4764549
SRR17520069.4764550
SRR17520069.4764551
SRR17520069.4764552
SRR17520069.4764553
SRR17520069.4764554
SRR17520069.4764555
SRR17520069.4764556
SRR17520069.4764557
SRR17520069.4764558
SRR17520069.4764559
SRR17520069.4764560
SRR17520069.4764561
SRR17520069.4764562
SRR17520069.4764563
SRR17520069.4764564
SRR17520069.4764565
SRR17520069.4764566
SRR17520069.4764567
SRR17520069.4764568
SRR17520069.4764569
SRR17520069.4764570
SRR17520069.4764571
SRR17520069.4764572
SRR17520069.4764573
SRR17520069.4764574

SRR17520069.4764575
SRR17520069.4764576
SRR17520069.4764577
SRR17520069.4764578
SRR17520069.4764579
SRR17520069.4764580
SRR17520069.4764581
SRR17520069.4764582
SRR17520069.4764583
SRR17520069.4764584
SRR17520069.4764585
SRR17520069.4764586
SRR17520069.4764587
SRR17520069.4764588
SRR17520069.4764589
SRR17520069.4764590
SRR17520069.4764591
SRR17520069.4764593
SRR17520069.4764594
SRR17520069.4764595
SRR17520069.4764596
SRR17520069.4764597
SRR17520069.4765197
SRR17520069.4765198
SRR17520069.4765199
SRR17520069.4765200
SRR17520069.4765201
SRR17520069.4765202
SRR17520069.4765203
SRR17520069.4765204
SRR17520069.4765205
SRR17520069.4765206
SRR17520069.4765207
SRR17520069.4765208
SRR17520069.4765209
SRR17520069.4765210
SRR17520069.4765212
SRR17520069.4765213
SRR17520069.4765214
SRR17520069.4765215
SRR17520069.4765216
SRR17520069.4765217
SRR17520069.4765219
SRR17520069.4765220
SRR17520069.4765223
SRR17520069.4765226
SRR17520069.4765227

SRR17520069.4765228
SRR17520069.4765229
SRR17520069.4765230
SRR17520069.4765231
SRR17520069.4765232
SRR17520069.4765233
SRR17520069.4765234
SRR17520069.4765235
SRR17520069.4765237
SRR17520069.4765238
SRR17520069.4765239
SRR17520069.4765242
SRR17520069.4765243
SRR17520069.4765244
SRR17520069.4765247
SRR17520069.4765248
SRR17520069.4771480
SRR17520069.4771512
SRR17520069.4782224
SRR17520069.4782225
SRR17520069.4782226
SRR17520069.4782227
SRR17520069.4782228
SRR17520069.4782229
SRR17520069.4782230
SRR17520069.4782231
SRR17520069.4782232
SRR17520069.4782233
SRR17520069.4782234
SRR17520069.4782235
SRR17520069.4782236
SRR17520069.4782237
SRR17520069.4782238
SRR17520069.4782241
SRR17520069.4782243
SRR17520069.4782244
SRR17520069.4782245
SRR17520069.4782246
SRR17520069.4782247
SRR17520069.4782248
SRR17520069.4782249
SRR17520069.4782250
SRR17520069.4782251
SRR17520069.4792145
SRR17520069.4792146
SRR17520069.4792147
SRR17520069.4792148

SRR17520069.4792149
SRR17520069.4792150
SRR17520069.4792151
SRR17520069.4792152
SRR17520069.4792153
SRR17520069.4792154
SRR17520069.4792155
SRR17520069.4792156
SRR17520069.4792157
SRR17520069.4792158
SRR17520069.4792159
SRR17520069.4792160
SRR17520069.4792161
SRR17520069.4792162
SRR17520069.4792163
SRR17520069.4792164
SRR17520069.4792165
SRR17520069.4792166
SRR17520069.4792167
SRR17520069.4792168
SRR17520069.4792169
SRR17520069.4792170
SRR17520069.4792171
SRR17520069.4792172
SRR17520069.4792173
SRR17520069.4792174
SRR17520069.4792175
SRR17520069.4792176
SRR17520069.4792177
SRR17520069.4792178
SRR17520069.4792179
SRR17520069.4792180
SRR17520069.4792181
SRR17520069.4792182
SRR17520069.4792183
SRR17520069.4792184
SRR17520069.4792185
SRR17520069.4792186
SRR17520069.4792187
SRR17520069.4792188
SRR17520069.4792189
SRR17520069.4792190
SRR17520069.4792191
SRR17520069.4792192
SRR17520069.4792193
SRR17520069.4792194
SRR17520069.4792195

SRR17520069.4792196
SRR17520069.4792197
SRR17520069.4792198
SRR17520069.4792199
SRR17520069.4792200
SRR17520069.4792201
SRR17520069.4792202
SRR17520069.4792203
SRR17520069.4792204
SRR17520069.4792205
SRR17520069.4792206
SRR17520069.4792207
SRR17520069.4792208
SRR17520069.4792209
SRR17520069.4792210
SRR17520069.4792211
SRR17520069.4792212
SRR17520069.4792213
SRR17520069.4792214
SRR17520069.4792215
SRR17520069.4792218
SRR17520069.4792219
SRR17520069.4792220
SRR17520069.4792221
SRR17520069.4792222
SRR17520069.4792223
SRR17520069.4792224
SRR17520069.4792225
SRR17520069.4792226
SRR17520069.4792227
SRR17520069.4792228
SRR17520069.4792229
SRR17520069.4826986
SRR17520069.4827004
SRR17520069.4838895
SRR17520069.4842554
SRR17520069.4842592
SRR17520069.4842668
SRR17520069.4864153
SRR17520069.4864154
SRR17520069.4864155
SRR17520069.4864156
SRR17520069.4864157
SRR17520069.4864158
SRR17520069.4864159
SRR17520069.4864160
SRR17520069.4864161

SRR17520069.4864162
SRR17520069.4864163
SRR17520069.4864164
SRR17520069.4864165
SRR17520069.4864166
SRR17520069.4864167
SRR17520069.4864168
SRR17520069.4864169
SRR17520069.4864170
SRR17520069.4864171
SRR17520069.4864172
SRR17520069.4864173
SRR17520069.4864174
SRR17520069.4864175
SRR17520069.4864176
SRR17520069.4864177
SRR17520069.4864178
SRR17520069.4864179
SRR17520069.4864180
SRR17520069.4864181
SRR17520069.4864182
SRR17520069.4864183
SRR17520069.4864184
SRR17520069.4864185
SRR17520069.4864186
SRR17520069.4864187
SRR17520069.4864188
SRR17520069.4864189
SRR17520069.4864190
SRR17520069.4864191
SRR17520069.4864192
SRR17520069.4864193
SRR17520069.4864194
SRR17520069.4864195
SRR17520069.4864196
SRR17520069.4864197
SRR17520069.4864198
SRR17520069.4864199
SRR17520069.4864200
SRR17520069.4864201
SRR17520069.4864202
SRR17520069.4864204
SRR17520069.4864205
SRR17520069.4864206
SRR17520069.4864207
SRR17520069.4864208
SRR17520069.4864209

SRR17520069.4864210
SRR17520069.4864211
SRR17520069.4864212
SRR17520069.4864213
SRR17520069.4864214
SRR17520069.4864215
SRR17520069.4864216
SRR17520069.4864217
SRR17520069.4864218
SRR17520069.4864219
SRR17520069.4864220
SRR17520069.4864221
SRR17520069.4864222
SRR17520069.4864223
SRR17520069.4864224
SRR17520069.4864225
SRR17520069.4864226
SRR17520069.4864227
SRR17520069.4864228
SRR17520069.4864229
SRR17520069.4864230
SRR17520069.4864231
SRR17520069.4864232
SRR17520069.4864233
SRR17520069.4864234
SRR17520069.4864235
SRR17520069.4864236
SRR17520069.4864237
SRR17520069.4864238
SRR17520069.4864239
SRR17520069.4864240
SRR17520069.4864241
SRR17520069.4864242
SRR17520069.4864243
SRR17520069.4864244
SRR17520069.4864245
SRR17520069.4864246
SRR17520069.4864247
SRR17520069.4864248
SRR17520069.4864249
SRR17520069.4864250
SRR17520069.4864251
SRR17520069.4864253
SRR17520069.4864254
SRR17520069.4864255
SRR17520069.4866785
SRR17520069.4866803

SRR17520069.4896669
SRR17520069.4896670
SRR17520069.4896671
SRR17520069.4896672
SRR17520069.4896673
SRR17520069.4896674
SRR17520069.4896675
SRR17520069.4896676
SRR17520069.4896677
SRR17520069.4896678
SRR17520069.4896679
SRR17520069.4896680
SRR17520069.4896681
SRR17520069.4896682
SRR17520069.4896683
SRR17520069.4896684
SRR17520069.4896685
SRR17520069.4896686
SRR17520069.4896687
SRR17520069.4896688
SRR17520069.4896689
SRR17520069.4896690
SRR17520069.4896691
SRR17520069.4896692
SRR17520069.4896693
SRR17520069.4896694
SRR17520069.4896695
SRR17520069.4896696
SRR17520069.4896697
SRR17520069.4896698
SRR17520069.4896699
SRR17520069.4896700
SRR17520069.4896701
SRR17520069.4896702
SRR17520069.4896703
SRR17520069.4896704
SRR17520069.4896705
SRR17520069.4896706
SRR17520069.4896707
SRR17520069.4896708
SRR17520069.4896709
SRR17520069.4896710
SRR17520069.4896711
SRR17520069.4896712
SRR17520069.4896713
SRR17520069.4896714
SRR17520069.4896715

SRR17520069.4896716
SRR17520069.4896717
SRR17520069.4896718
SRR17520069.4896719
SRR17520069.4896720
SRR17520069.4896721
SRR17520069.4896722
SRR17520069.4896723
SRR17520069.4896724
SRR17520069.4896725
SRR17520069.4896726
SRR17520069.4896727
SRR17520069.4896728
SRR17520069.4896729
SRR17520069.4896730
SRR17520069.4896731
SRR17520069.4896732
SRR17520069.4896733
SRR17520069.4896734
SRR17520069.4896735
SRR17520069.4896736
SRR17520069.4896737
SRR17520069.4896738
SRR17520069.4896739
SRR17520069.4896740
SRR17520069.4896741
SRR17520069.4896742
SRR17520069.4896743
SRR17520069.4896744
SRR17520069.4896745
SRR17520069.4896746
SRR17520069.4896747
SRR17520069.4896748
SRR17520069.4896749
SRR17520069.4896750
SRR17520069.4896751
SRR17520069.4896752
SRR17520069.4896753
SRR17520069.4896754
SRR17520069.4896755
SRR17520069.4896756
SRR17520069.4896757
SRR17520069.4896758
SRR17520069.4896759
SRR17520069.4896760
SRR17520069.4896761
SRR17520069.4896762

SRR17520069.4896763
SRR17520069.4896764
SRR17520069.4896765
SRR17520069.4896766
SRR17520069.4896767
SRR17520069.4896768
SRR17520069.4896769
SRR17520069.4896770
SRR17520069.4896771
SRR17520069.4896772
SRR17520069.4896773
SRR17520069.4896774
SRR17520069.4896775
SRR17520069.4896776
SRR17520069.4896777
SRR17520069.4896778
SRR17520069.4896779
SRR17520069.4896780
SRR17520069.4904589
SRR17520069.4904641
SRR17520069.4926857
SRR17520069.4930309
SRR17520069.4930312
SRR17520069.4930314
SRR17520069.4930315
SRR17520069.4930317
SRR17520069.4930318
SRR17520069.4930319
SRR17520069.4930320
SRR17520069.4930321
SRR17520069.4930322
SRR17520069.4930323
SRR17520069.4930324
SRR17520069.4930325
SRR17520069.4930327
SRR17520069.4930328
SRR17520069.4930329
SRR17520069.4931231
SRR17520069.4931254
SRR17520069.4931273
SRR17520069.4931289
SRR17520069.4931310
SRR17520069.4943378
SRR17520069.4963400
SRR17520069.4963401
SRR17520069.4963402
SRR17520069.4963403

SRR17520069.4963404
SRR17520069.4963405
SRR17520069.4963406
SRR17520069.4963407
SRR17520069.4963408
SRR17520069.4963409
SRR17520069.4963410
SRR17520069.4963411
SRR17520069.4963412
SRR17520069.4963413
SRR17520069.4963414
SRR17520069.4963415
SRR17520069.4963416
SRR17520069.4963417
SRR17520069.4963418
SRR17520069.4963419
SRR17520069.4963420
SRR17520069.4963421
SRR17520069.4963423
SRR17520069.4963428
SRR17520069.4963441
SRR17520069.4963443
SRR17520069.4966076
SRR17520069.4966077
SRR17520069.4966078
SRR17520069.4966080
SRR17520069.4966081
SRR17520069.4966082
SRR17520069.4966083
SRR17520069.4966084
SRR17520069.4966085
SRR17520069.4966086
SRR17520069.4966087
SRR17520069.4966088
SRR17520069.4966089
SRR17520069.4966091
SRR17520069.4966092
SRR17520069.4966093
SRR17520069.4966094
SRR17520069.4966095
SRR17520069.4966096
SRR17520069.4966097
SRR17520069.4966098
SRR17520069.4966099
SRR17520069.4966100
SRR17520069.4966101
SRR17520069.4966102

SRR17520069.4966103
SRR17520069.4966104
SRR17520069.4966105
SRR17520069.4966106
SRR17520069.4966107
SRR17520069.4987257
SRR17520069.4987258
SRR17520069.4987259
SRR17520069.4987260
SRR17520069.4987262
SRR17520069.4987690
SRR17520069.4987691
SRR17520069.4987692
SRR17520069.4987693
SRR17520069.4987694
SRR17520069.4987695
SRR17520069.4987696
SRR17520069.4987697
SRR17520069.4987698
SRR17520069.4987699
SRR17520069.4987700
SRR17520069.4987701
SRR17520069.4987702
SRR17520069.4987703
SRR17520069.4987704
SRR17520069.4987705
SRR17520069.4987706
SRR17520069.4987707
SRR17520069.4987708
SRR17520069.4987709
SRR17520069.4987710
SRR17520069.4987711
SRR17520069.4987712
SRR17520069.4987713
SRR17520069.4987714
SRR17520069.4987715
SRR17520069.4987716
SRR17520069.4987717
SRR17520069.4987718
SRR17520069.4987719
SRR17520069.4987720
SRR17520069.4987721
SRR17520069.4987722
SRR17520069.4987723
SRR17520069.4987724
SRR17520069.4987725
SRR17520069.4987726

SRR17520069.4987727
SRR17520069.4987728
SRR17520069.4987729
SRR17520069.4987730
SRR17520069.4987731
SRR17520069.4987732
SRR17520069.4987733
SRR17520069.4987734
SRR17520069.4987735
SRR17520069.4987736
SRR17520069.4987737
SRR17520069.4987738
SRR17520069.4987739
SRR17520069.4987740
SRR17520069.4987741
SRR17520069.4987742
SRR17520069.4987743
SRR17520069.4987744
SRR17520069.4987745
SRR17520069.4987746
SRR17520069.4987747
SRR17520069.4987748
SRR17520069.4987749
SRR17520069.4987750
SRR17520069.4987751
SRR17520069.4987752
SRR17520069.4987753
SRR17520069.4987754
SRR17520069.4987755
SRR17520069.4987756
SRR17520069.4987757
SRR17520069.4987758
SRR17520069.4987759
SRR17520069.4987760
SRR17520069.4987761
SRR17520069.4987762
SRR17520069.4987763
SRR17520069.4987764
SRR17520069.4987765
SRR17520069.4987766
SRR17520069.4987767
SRR17520069.4987768
SRR17520069.4987769
SRR17520069.4987770
SRR17520069.4987771
SRR17520069.4987772
SRR17520069.4987773

SRR17520069.4987774
SRR17520069.4987775
SRR17520069.4987776
SRR17520069.4987777
SRR17520069.4987778
SRR17520069.4987779
SRR17520069.4987780
SRR17520069.4987781
SRR17520069.4987782
SRR17520069.4987783
SRR17520069.4987784
SRR17520069.4987785
SRR17520069.4987786
SRR17520069.4987787
SRR17520069.4987788
SRR17520069.4987789
SRR17520069.4987791
SRR17520069.4987792
SRR17520069.4987793
SRR17520069.4987794
SRR17520069.4987795
SRR17520069.4987796
SRR17520069.4987797
SRR17520069.4987798
SRR17520069.4987799
SRR17520069.4987800
SRR17520069.4987802
SRR17520069.4987803
SRR17520069.4987804
SRR17520069.4987805
SRR17520069.4987806
SRR17520069.4987807
SRR17520069.4987808
SRR17520069.4987809
SRR17520069.4987810
SRR17520069.4987811
SRR17520069.4987812
SRR17520069.4987813
SRR17520069.4987814
SRR17520069.4987815
SRR17520069.4987816
SRR17520069.4987817
SRR17520069.4987818
SRR17520069.4987819
SRR17520069.4987820
SRR17520069.4987821
SRR17520069.4987822

SRR17520069.4987823
SRR17520069.4987824
SRR17520069.4987825
SRR17520069.4987826
SRR17520069.4987827
SRR17520069.4987828
SRR17520069.4987829
SRR17520069.4987830
SRR17520069.4987831
SRR17520069.4987832
SRR17520069.4989594
SRR17520069.4989595
SRR17520069.4989596
SRR17520069.4989597
SRR17520069.4989598
SRR17520069.4989599
SRR17520069.4989600
SRR17520069.4989601
SRR17520069.4989602
SRR17520069.4989603
SRR17520069.4989604
SRR17520069.4989605
SRR17520069.4989606
SRR17520069.5000291
SRR17520069.5000292
SRR17520069.5000293
SRR17520069.5000294
SRR17520069.5000295
SRR17520069.5000296
SRR17520069.5000297
SRR17520069.5000298
SRR17520069.5000299
SRR17520069.5000300
SRR17520069.5000301
SRR17520069.5000302
SRR17520069.5000303
SRR17520069.5000304
SRR17520069.5000305
SRR17520069.5000306
SRR17520069.5000307
SRR17520069.5000308
SRR17520069.5000309
SRR17520069.5000310
SRR17520069.5000311
SRR17520069.5000312
SRR17520069.5000313
SRR17520069.5000314

SRR17520069.5000315
SRR17520069.5000316
SRR17520069.5000317
SRR17520069.5000318
SRR17520069.5000319
SRR17520069.5000320
SRR17520069.5000321
SRR17520069.5000322
SRR17520069.5000323
SRR17520069.5000324
SRR17520069.5000325
SRR17520069.5000326
SRR17520069.5000327
SRR17520069.5000328
SRR17520069.5000329
SRR17520069.5000330
SRR17520069.5000331
SRR17520069.5000332
SRR17520069.5000333
SRR17520069.5000334
SRR17520069.5000335
SRR17520069.5000336
SRR17520069.5000337
SRR17520069.5000338
SRR17520069.5000339
SRR17520069.5000340
SRR17520069.5000341
SRR17520069.5000342
SRR17520069.5000343
SRR17520069.5000344
SRR17520069.5000345
SRR17520069.5000346
SRR17520069.5000347
SRR17520069.5000348
SRR17520069.5000350
SRR17520069.5000351
SRR17520069.5000352
SRR17520069.5000353
SRR17520069.5000354
SRR17520069.5000355
SRR17520069.5000356
SRR17520069.5000357
SRR17520069.5000359
SRR17520069.5000360
SRR17520069.5000361
SRR17520069.5000362
SRR17520069.5000363

SRR17520069.5000364
SRR17520069.5000365
SRR17520069.5000366
SRR17520069.5000368
SRR17520069.5000369
SRR17520069.5000370
SRR17520069.5000371
SRR17520069.5000372
SRR17520069.5000373
SRR17520069.5000374
SRR17520069.5000375
SRR17520069.5000376
SRR17520069.5000377
SRR17520069.5000378
SRR17520069.5000379
SRR17520069.5000380
SRR17520069.5000381
SRR17520069.5000383
SRR17520069.5000384
SRR17520069.5000385
SRR17520069.5000386
SRR17520069.5000387
SRR17520069.5000388
SRR17520069.5000389
SRR17520069.5000390
SRR17520069.5000391
SRR17520069.5000392
SRR17520069.5000393
SRR17520069.5000394
SRR17520069.5000395
SRR17520069.5000396
SRR17520069.5000397
SRR17520069.5000398
SRR17520069.5000399
SRR17520069.5000400
SRR17520069.5000401
SRR17520069.5000402
SRR17520069.5000403
SRR17520069.5000405
SRR17520069.5000406
SRR17520069.5000407
SRR17520069.5000408
SRR17520069.5000409
SRR17520069.5000410
SRR17520069.5000411
SRR17520069.5000412
SRR17520069.5000413

SRR17520069.5000414
SRR17520069.5000415
SRR17520069.5000417
SRR17520069.5000418
SRR17520069.5000419
SRR17520069.5000420
SRR17520069.5000421
SRR17520069.5000423
SRR17520069.5000424
SRR17520069.5000426
SRR17520069.5000427
SRR17520069.5000428
SRR17520069.5000429
SRR17520069.5000431
SRR17520069.5000432
SRR17520069.5000433
SRR17520069.5000434
SRR17520069.5000435
SRR17520069.5000436
SRR17520069.5000437
SRR17520069.5000438
SRR17520069.5000439
SRR17520069.5000440
SRR17520069.5000441
SRR17520069.5000442
SRR17520069.5000443
SRR17520069.5000444
SRR17520069.5000445
SRR17520069.5000446
SRR17520069.5000447
SRR17520069.5000448
SRR17520069.5000450
SRR17520069.5000451
SRR17520069.5000452
SRR17520069.5000453
SRR17520069.5000454
SRR17520069.5000455
SRR17520069.5000457
SRR17520069.5000458
SRR17520069.5000459
SRR17520069.5000461
SRR17520069.5000462
SRR17520069.5000463
SRR17520069.5000467
SRR17520069.5000468
SRR17520069.5000469
SRR17520069.5000470

SRR17520069.5000471
SRR17520069.5000472
SRR17520069.5006573
SRR17520069.5006574
SRR17520069.5006575
SRR17520069.5006576
SRR17520069.5006577
SRR17520069.5006578
SRR17520069.5006580
SRR17520069.5006581
SRR17520069.5006582
SRR17520069.5006583
SRR17520069.5006584
SRR17520069.5006585
SRR17520069.5006586
SRR17520069.5006587
SRR17520069.5006589
SRR17520069.5006590
SRR17520069.5006591
SRR17520069.5006592
SRR17520069.5006593
SRR17520069.5006594
SRR17520069.5006595
SRR17520069.5006596
SRR17520069.5006597
SRR17520069.5006598
SRR17520069.5006599
SRR17520069.5006600
SRR17520069.5006601
SRR17520069.5006602
SRR17520069.5006603
SRR17520069.5006604
SRR17520069.5006605
SRR17520069.5006606
SRR17520069.5006607
SRR17520069.5006608
SRR17520069.5006611
SRR17520069.5006612
SRR17520069.5006613
SRR17520069.5006614
SRR17520069.5006615
SRR17520069.5006616
SRR17520069.5006617
SRR17520069.5006618
SRR17520069.5006619
SRR17520069.5006620
SRR17520069.5006623

SRR17520069.5006624
SRR17520069.5006625
SRR17520069.5006626
SRR17520069.5006627
SRR17520069.5006628
SRR17520069.5006629
SRR17520069.5006630
SRR17520069.5006631
SRR17520069.5006632
SRR17520069.5006633
SRR17520069.5006634
SRR17520069.5006635
SRR17520069.5006636
SRR17520069.5006637
SRR17520069.5006638
SRR17520069.5006639
SRR17520069.5006640
SRR17520069.5006641
SRR17520069.5006642
SRR17520069.5006643
SRR17520069.5006645
SRR17520069.5006646
SRR17520069.5006647
SRR17520069.5006649
SRR17520069.5033158
SRR17520069.5033159
SRR17520069.5033160
SRR17520069.5033161
SRR17520069.5033162
SRR17520069.5033163
SRR17520069.5033164
SRR17520069.5033165
SRR17520069.5033167
SRR17520069.5033168
SRR17520069.5033169
SRR17520069.5033170
SRR17520069.5033171
SRR17520069.5033173
SRR17520069.5033176
SRR17520069.5033178
SRR17520069.5033179
SRR17520069.5033180
SRR17520069.5033182
SRR17520069.5033186
SRR17520069.5033187
SRR17520069.5033188
SRR17520069.5039427

SRR17520069.5039428
SRR17520069.5039429
SRR17520069.5039431
SRR17520069.5039433
SRR17520069.5039435
SRR17520069.5039436
SRR17520069.5039437
SRR17520069.5039439
SRR17520069.5039440
SRR17520069.5039441
SRR17520069.5039442
SRR17520069.5039445
SRR17520069.5039446
SRR17520069.5039447
SRR17520069.5039448
SRR17520069.5039449
SRR17520069.5039450
SRR17520069.5039455
SRR17520069.5039456
SRR17520069.5039458
SRR17520069.5039459
SRR17520069.5039460
SRR17520069.5039461
SRR17520069.5039462
SRR17520069.5039463
SRR17520069.5039466
SRR17520069.5039467
SRR17520069.5039468
SRR17520069.5039469
SRR17520069.5039470
SRR17520069.5039471
SRR17520069.5039473
SRR17520069.5050059
SRR17520069.5050060
SRR17520069.5050061
SRR17520069.5050062
SRR17520069.5050063
SRR17520069.5050064
SRR17520069.5050065
SRR17520069.5050066
SRR17520069.5050067
SRR17520069.5050068
SRR17520069.5050069
SRR17520069.5050070
SRR17520069.5050071
SRR17520069.5050072
SRR17520069.5050073

SRR17520069.5050074
SRR17520069.5050075
SRR17520069.5050076
SRR17520069.5050077
SRR17520069.5050078
SRR17520069.5050079
SRR17520069.5050080
SRR17520069.5050081
SRR17520069.5050082
SRR17520069.5050084
SRR17520069.5050085
SRR17520069.5050086
SRR17520069.5050087
SRR17520069.5050088
SRR17520069.5050089
SRR17520069.5050090
SRR17520069.5050091
SRR17520069.5050092
SRR17520069.5050093
SRR17520069.5050094
SRR17520069.5050095
SRR17520069.5050096
SRR17520069.5050097
SRR17520069.5050099
SRR17520069.5050100
SRR17520069.5050101
SRR17520069.5050102
SRR17520069.5050103
SRR17520069.5050104
SRR17520069.5050105
SRR17520069.5050106
SRR17520069.5050107
SRR17520069.5050108
SRR17520069.5050109
SRR17520069.5050110
SRR17520069.5050111
SRR17520069.5050112
SRR17520069.5050113
SRR17520069.5050114
SRR17520069.5050115
SRR17520069.5050117
SRR17520069.5050118
SRR17520069.5050119
SRR17520069.5050121
SRR17520069.5050122
SRR17520069.5050123
SRR17520069.5050124

SRR17520069.5050125
SRR17520069.5050126
SRR17520069.5050127
SRR17520069.5051036
SRR17520069.5051037
SRR17520069.5051042
SRR17520069.5051043
SRR17520069.5051045
SRR17520069.5051047
SRR17520069.5051048
SRR17520069.5051049
SRR17520069.5056949
SRR17520069.5056950
SRR17520069.5062050
SRR17520069.5062051
SRR17520069.5062052
SRR17520069.5062053
SRR17520069.5062054
SRR17520069.5062055
SRR17520069.5062056
SRR17520069.5062057
SRR17520069.5062058
SRR17520069.5062059
SRR17520069.5062060
SRR17520069.5062061
SRR17520069.5062062
SRR17520069.5062063
SRR17520069.5062064
SRR17520069.5062065
SRR17520069.5062066
SRR17520069.5062067
SRR17520069.5062068
SRR17520069.5062069
SRR17520069.5062070
SRR17520069.5062071
SRR17520069.5062072
SRR17520069.5062073
SRR17520069.5062074
SRR17520069.5062075
SRR17520069.5062076
SRR17520069.5062077
SRR17520069.5062078
SRR17520069.5062079
SRR17520069.5062080
SRR17520069.5062082
SRR17520069.5062083
SRR17520069.5062084

SRR17520069.5062085
SRR17520069.5062086
SRR17520069.5062087
SRR17520069.5062088
SRR17520069.5062089
SRR17520069.5062090
SRR17520069.5062091
SRR17520069.5062092
SRR17520069.5062093
SRR17520069.5062094
SRR17520069.5062095
SRR17520069.5062096
SRR17520069.5062097
SRR17520069.5062098
SRR17520069.5062099
SRR17520069.5062100
SRR17520069.5062101
SRR17520069.5062102
SRR17520069.5062103
SRR17520069.5087737
SRR17520069.5087738
SRR17520069.5087739
SRR17520069.5087740
SRR17520069.5087741
SRR17520069.5087742
SRR17520069.5087743
SRR17520069.5087744
SRR17520069.5087745
SRR17520069.5087746
SRR17520069.5087747
SRR17520069.5087748
SRR17520069.5087749
SRR17520069.5087750
SRR17520069.5087751
SRR17520069.5087752
SRR17520069.5087753
SRR17520069.5087754
SRR17520069.5087755
SRR17520069.5087756
SRR17520069.5087757
SRR17520069.5087758
SRR17520069.5087759
SRR17520069.5087760
SRR17520069.5087761
SRR17520069.5087762
SRR17520069.5087763
SRR17520069.5087764

SRR17520069.5087765
SRR17520069.5087766
SRR17520069.5087768
SRR17520069.5087769
SRR17520069.5087770
SRR17520069.5087771
SRR17520069.5087772
SRR17520069.5087773
SRR17520069.5087774
SRR17520069.5087775
SRR17520069.5087776
SRR17520069.5087777
SRR17520069.5087778
SRR17520069.5087779
SRR17520069.5087780
SRR17520069.5087781
SRR17520069.5087782
SRR17520069.5087783
SRR17520069.5087784
SRR17520069.5087785
SRR17520069.5087787
SRR17520069.5087788
SRR17520069.5087790
SRR17520069.5087791
SRR17520069.5087792
SRR17520069.5087793
SRR17520069.5087794
SRR17520069.5087795
SRR17520069.5087796
SRR17520069.5087797
SRR17520069.5087798
SRR17520069.5087799
SRR17520069.5087800
SRR17520069.5087801
SRR17520069.5087802
SRR17520069.5087803
SRR17520069.5087804
SRR17520069.5087805
SRR17520069.5087806
SRR17520069.5087807
SRR17520069.5087808
SRR17520069.5087809
SRR17520069.5087810
SRR17520069.5087811
SRR17520069.5087812
SRR17520069.5087813
SRR17520069.5087814

SRR17520069.5087815
SRR17520069.5087816
SRR17520069.5087817
SRR17520069.5087818
SRR17520069.5087819
SRR17520069.5087820
SRR17520069.5087821
SRR17520069.5087822
SRR17520069.5087823
SRR17520069.5087824
SRR17520069.5087825
SRR17520069.5087826
SRR17520069.5087827
SRR17520069.5087828
SRR17520069.5087829
SRR17520069.5087830
SRR17520069.5087831
SRR17520069.5087832
SRR17520069.5087833
SRR17520069.5087834
SRR17520069.5087835
SRR17520069.5087836
SRR17520069.5087837
SRR17520069.5087838
SRR17520069.5087839
SRR17520069.5087840
SRR17520069.5087841
SRR17520069.5087842
SRR17520069.5087843
SRR17520069.5087844
SRR17520069.5087845
SRR17520069.5087846
SRR17520069.5087847
SRR17520069.5087848
SRR17520069.5087849
SRR17520069.5087850
SRR17520069.5087851
SRR17520069.5087852
SRR17520069.5087853
SRR17520069.5087854
SRR17520069.5087855
SRR17520069.5087856
SRR17520069.5087857
SRR17520069.5087858
SRR17520069.5087859
SRR17520069.5087860
SRR17520069.5087861

SRR17520069.5087862
SRR17520069.5087864
SRR17520069.5087865
SRR17520069.5087866
SRR17520069.5087867
SRR17520069.5087868
SRR17520069.5087869
SRR17520069.5087870
SRR17520069.5087871
SRR17520069.5087872
SRR17520069.5087873
SRR17520069.5087874
SRR17520069.5087875
SRR17520069.5087876
SRR17520069.5087877
SRR17520069.5087878
SRR17520069.5087879
SRR17520069.5087880
SRR17520069.5087881
SRR17520069.5087882
SRR17520069.5087883
SRR17520069.5087884
SRR17520069.5087885
SRR17520069.5087886
SRR17520069.5087887
SRR17520069.5087888
SRR17520069.5087889
SRR17520069.5087890
SRR17520069.5087891
SRR17520069.5087892
SRR17520069.5087893
SRR17520069.5087894
SRR17520069.5087895
SRR17520069.5087896
SRR17520069.5087897
SRR17520069.5087898
SRR17520069.5087899
SRR17520069.5087900
SRR17520069.5087901
SRR17520069.5087902
SRR17520069.5087903
SRR17520069.5087904
SRR17520069.5087905
SRR17520069.5087906
SRR17520069.5087907
SRR17520069.5087908
SRR17520069.5087909

SRR17520069.5087911
SRR17520069.5087912
SRR17520069.5087913
SRR17520069.5087914
SRR17520069.5087915
SRR17520069.5087916
SRR17520069.5087917
SRR17520069.5087918
SRR17520069.5087919
SRR17520069.5087920
SRR17520069.5087921
SRR17520069.5087922
SRR17520069.5087923
SRR17520069.5087924
SRR17520069.5087925
SRR17520069.5087926
SRR17520069.5087927
SRR17520069.5087928
SRR17520069.5087929
SRR17520069.5087930
SRR17520069.5087931
SRR17520069.5087932
SRR17520069.5094259
SRR17520069.5095390
SRR17520069.5101506
SRR17520069.5116363
SRR17520069.5125473
SRR17520069.5125479
SRR17520069.5125482
SRR17520069.5125489
SRR17520069.5125490
SRR17520069.5125495
SRR17520069.5125502
SRR17520069.5125512
SRR17520069.5125513
SRR17520069.5125514
SRR17520069.5125516
SRR17520069.5125520
SRR17520069.5125530
SRR17520069.5125533
SRR17520069.5126127
SRR17520069.5131270
SRR17520069.5131271
SRR17520069.5131272
SRR17520069.5131273
SRR17520069.5131274
SRR17520069.5131275

SRR17520069.5131276
SRR17520069.5131277
SRR17520069.5131278
SRR17520069.5131279
SRR17520069.5131280
SRR17520069.5131281
SRR17520069.5131282
SRR17520069.5131283
SRR17520069.5131285
SRR17520069.5131286
SRR17520069.5131287
SRR17520069.5131288
SRR17520069.5131289
SRR17520069.5131290
SRR17520069.5131291
SRR17520069.5131292
SRR17520069.5131293
SRR17520069.5131294
SRR17520069.5131295
SRR17520069.5131296
SRR17520069.5131297
SRR17520069.5131298
SRR17520069.5131299
SRR17520069.5131300
SRR17520069.5131301
SRR17520069.5131302
SRR17520069.5131303
SRR17520069.5131304
SRR17520069.5131305
SRR17520069.5131306
SRR17520069.5131307
SRR17520069.5131308
SRR17520069.5131309
SRR17520069.5131310
SRR17520069.5131311
SRR17520069.5131312
SRR17520069.5131313
SRR17520069.5131314
SRR17520069.5131315
SRR17520069.5131316
SRR17520069.5131317
SRR17520069.5131318
SRR17520069.5131319
SRR17520069.5131320
SRR17520069.5131321
SRR17520069.5131322
SRR17520069.5131323

SRR17520069.5131324
SRR17520069.5131325
SRR17520069.5131326
SRR17520069.5131327
SRR17520069.5131328
SRR17520069.5131329
SRR17520069.5131330
SRR17520069.5131331
SRR17520069.5131332
SRR17520069.5131333
SRR17520069.5131334
SRR17520069.5131335
SRR17520069.5131336
SRR17520069.5131337
SRR17520069.5131338
SRR17520069.5131339
SRR17520069.5131340
SRR17520069.5131341
SRR17520069.5131342
SRR17520069.5131343
SRR17520069.5131344
SRR17520069.5131345
SRR17520069.5131346
SRR17520069.5131347
SRR17520069.5131348
SRR17520069.5131349
SRR17520069.5131350
SRR17520069.5131351
SRR17520069.5131352
SRR17520069.5131353
SRR17520069.5131354
SRR17520069.5131355
SRR17520069.5131356
SRR17520069.5131357
SRR17520069.5131358
SRR17520069.5131359
SRR17520069.5131360
SRR17520069.5131361
SRR17520069.5131362
SRR17520069.5131363
SRR17520069.5131364
SRR17520069.5131365
SRR17520069.5131366
SRR17520069.5131367
SRR17520069.5131368
SRR17520069.5131369
SRR17520069.5131370

SRR17520069.5131371
SRR17520069.5131372
SRR17520069.5131373
SRR17520069.5131374
SRR17520069.5131376
SRR17520069.5131377
SRR17520069.5131378
SRR17520069.5131380
SRR17520069.5131381
SRR17520069.5131382
SRR17520069.5131383
SRR17520069.5131384
SRR17520069.5131385
SRR17520069.5131386
SRR17520069.5131387
SRR17520069.5131388
SRR17520069.5131389
SRR17520069.5131390
SRR17520069.5131391
SRR17520069.5131392
SRR17520069.5131393
SRR17520069.5131395
SRR17520069.5131396
SRR17520069.5131397
SRR17520069.5131398
SRR17520069.5131399
SRR17520069.5131400
SRR17520069.5131401
SRR17520069.5131402
SRR17520069.5131403
SRR17520069.5131404
SRR17520069.5131405
SRR17520069.5131406
SRR17520069.5131408
SRR17520069.5139421
SRR17520069.5139422
SRR17520069.5139423
SRR17520069.5139424
SRR17520069.5139425
SRR17520069.5139426
SRR17520069.5139428
SRR17520069.5139429
SRR17520069.5139430
SRR17520069.5139431
SRR17520069.5139432
SRR17520069.5170875
SRR17520069.5170876

SRR17520069.5170878
SRR17520069.5170880
SRR17520069.5170882
SRR17520069.5175799
SRR17520069.5175809
SRR17520069.5176043
SRR17520069.5176044
SRR17520069.5176045
SRR17520069.5176046
SRR17520069.5176047
SRR17520069.5176048
SRR17520069.5176049
SRR17520069.5176050
SRR17520069.5176051
SRR17520069.5176052
SRR17520069.5176053
SRR17520069.5176054
SRR17520069.5176055
SRR17520069.5176056
SRR17520069.5176057
SRR17520069.5176058
SRR17520069.5176059
SRR17520069.5176060
SRR17520069.5176061
SRR17520069.5176062
SRR17520069.5176063
SRR17520069.5176064
SRR17520069.5176065
SRR17520069.5176066
SRR17520069.5176067
SRR17520069.5176068
SRR17520069.5176073
SRR17520069.5176074
SRR17520069.5176075
SRR17520069.5176076
SRR17520069.5176077
SRR17520069.5176079
SRR17520069.5176080
SRR17520069.5176081
SRR17520069.5176082
SRR17520069.5176085
SRR17520069.5176086
SRR17520069.5176087
SRR17520069.5176088
SRR17520069.5176089
SRR17520069.5176090
SRR17520069.5176092

SRR17520069.5176093
SRR17520069.5176094
SRR17520069.5176095
SRR17520069.5176096
SRR17520069.5176097
SRR17520069.5176099
SRR17520069.5176100
SRR17520069.5176101
SRR17520069.5176102
SRR17520069.5176103
SRR17520069.5176104
SRR17520069.5176105
SRR17520069.5176106
SRR17520069.5176107
SRR17520069.5176108
SRR17520069.5176109
SRR17520069.5176110
SRR17520069.5176112
SRR17520069.5176113
SRR17520069.5176114
SRR17520069.5176115
SRR17520069.5176117
SRR17520069.5176118
SRR17520069.5176119
SRR17520069.5176120
SRR17520069.5176121
SRR17520069.5176122
SRR17520069.5176123
SRR17520069.5176124
SRR17520069.5176126
SRR17520069.5176127
SRR17520069.5176128
SRR17520069.5176129
SRR17520069.5176130
SRR17520069.5176131
SRR17520069.5176132
SRR17520069.5176133
SRR17520069.5176134
SRR17520069.5176136
SRR17520069.5176137
SRR17520069.5176138
SRR17520069.5176139
SRR17520069.5176140
SRR17520069.5176141
SRR17520069.5176142
SRR17520069.5176143
SRR17520069.5176145

SRR17520069.5176146
SRR17520069.5176147
SRR17520069.5176148
SRR17520069.5176149
SRR17520069.5176150
SRR17520069.5176151
SRR17520069.5176152
SRR17520069.5176153
SRR17520069.5176154
SRR17520069.5176155
SRR17520069.5176156
SRR17520069.5176157
SRR17520069.5176158
SRR17520069.5176159
SRR17520069.5176160
SRR17520069.5176161
SRR17520069.5176162
SRR17520069.5184225
SRR17520069.5199448
SRR17520069.5200056
SRR17520069.5200057
SRR17520069.5200058
SRR17520069.5200059
SRR17520069.5200062
SRR17520069.5200063
SRR17520069.5200064
SRR17520069.5200065
SRR17520069.5200066
SRR17520069.5200067
SRR17520069.5200068
SRR17520069.5200069
SRR17520069.5200070
SRR17520069.5200071
SRR17520069.5200072
SRR17520069.5200073
SRR17520069.5200074
SRR17520069.5200075
SRR17520069.5200076
SRR17520069.5200077
SRR17520069.5200078
SRR17520069.5200079
SRR17520069.5200080
SRR17520069.5200081
SRR17520069.5200082
SRR17520069.5200083
SRR17520069.5200084
SRR17520069.5200085

SRR17520069.5200086
SRR17520069.5200087
SRR17520069.5200088
SRR17520069.5200089
SRR17520069.5200090
SRR17520069.5200092
SRR17520069.5200093
SRR17520069.5200094
SRR17520069.5200095
SRR17520069.5200096
SRR17520069.5200097
SRR17520069.5200098
SRR17520069.5200099
SRR17520069.5200100
SRR17520069.5200101
SRR17520069.5200102
SRR17520069.5200103
SRR17520069.5200105
SRR17520069.5200106
SRR17520069.5200107
SRR17520069.5200108
SRR17520069.5200109
SRR17520069.5200110
SRR17520069.5200111
SRR17520069.5200112
SRR17520069.5200113
SRR17520069.5200114
SRR17520069.5200115
SRR17520069.5200117
SRR17520069.5200118
SRR17520069.5200119
SRR17520069.5200120
SRR17520069.5200121
SRR17520069.5200122
SRR17520069.5200124
SRR17520069.5200125
SRR17520069.5200126
SRR17520069.5200127
SRR17520069.5200128
SRR17520069.5200129
SRR17520069.5200130
SRR17520069.5200131
SRR17520069.5200132
SRR17520069.5200133
SRR17520069.5200134
SRR17520069.5200135
SRR17520069.5200136

SRR17520069.5200137
SRR17520069.5200138
SRR17520069.5200166
SRR17520069.5200167
SRR17520069.5200168
SRR17520069.5200169
SRR17520069.5200170
SRR17520069.5200171
SRR17520069.5200172
SRR17520069.5200173
SRR17520069.5200174
SRR17520069.5200177
SRR17520069.5200178
SRR17520069.5200179
SRR17520069.5200180
SRR17520069.5200181
SRR17520069.5200182
SRR17520069.5200183
SRR17520069.5200184
SRR17520069.5200185
SRR17520069.5200186
SRR17520069.5200187
SRR17520069.5200188
SRR17520069.5200189
SRR17520069.5200190
SRR17520069.5200191
SRR17520069.5200192
SRR17520069.5200193
SRR17520069.5200194
SRR17520069.5200195
SRR17520069.5200196
SRR17520069.5200197
SRR17520069.5200198
SRR17520069.5200199
SRR17520069.5200200
SRR17520069.5200201
SRR17520069.5200202
SRR17520069.5200203
SRR17520069.5200204
SRR17520069.5200205
SRR17520069.5200206
SRR17520069.5200207
SRR17520069.5200208
SRR17520069.5200209
SRR17520069.5200210
SRR17520069.5200211
SRR17520069.5200212

SRR17520069.5200213
SRR17520069.5200214
SRR17520069.5200215
SRR17520069.5200216
SRR17520069.5200217
SRR17520069.5200218
SRR17520069.5200219
SRR17520069.5200220
SRR17520069.5200221
SRR17520069.5200222
SRR17520069.5200223
SRR17520069.5207215
SRR17520069.5213932
SRR17520069.5213933
SRR17520069.5213934
SRR17520069.5213935
SRR17520069.5213936
SRR17520069.5213937
SRR17520069.5213938
SRR17520069.5213939
SRR17520069.5213940
SRR17520069.5213941
SRR17520069.5213942
SRR17520069.5213943
SRR17520069.5213944
SRR17520069.5213945
SRR17520069.5213946
SRR17520069.5213947
SRR17520069.5213948
SRR17520069.5213949
SRR17520069.5213950
SRR17520069.5213951
SRR17520069.5213952
SRR17520069.5213953
SRR17520069.5213954
SRR17520069.5213955
SRR17520069.5213956
SRR17520069.5213957
SRR17520069.5213958
SRR17520069.5213959
SRR17520069.5213960
SRR17520069.5213961
SRR17520069.5213962
SRR17520069.5213963
SRR17520069.5213964
SRR17520069.5213965
SRR17520069.5213966

SRR17520069.5213967
SRR17520069.5213968
SRR17520069.5213969
SRR17520069.5213970
SRR17520069.5213971
SRR17520069.5213972
SRR17520069.5213973
SRR17520069.5213974
SRR17520069.5213975
SRR17520069.5213976
SRR17520069.5213977
SRR17520069.5213978
SRR17520069.5213979
SRR17520069.5213980
SRR17520069.5213981
SRR17520069.5213982
SRR17520069.5213983
SRR17520069.5213984
SRR17520069.5213985
SRR17520069.5213986
SRR17520069.5213987
SRR17520069.5213988
SRR17520069.5213989
SRR17520069.5213990
SRR17520069.5213991
SRR17520069.5213992
SRR17520069.5213993
SRR17520069.5213994
SRR17520069.5213995
SRR17520069.5213996
SRR17520069.5213997
SRR17520069.5213998
SRR17520069.5213999
SRR17520069.5214000
SRR17520069.5214001
SRR17520069.5214002
SRR17520069.5214003
SRR17520069.5214004
SRR17520069.5214005
SRR17520069.5214006
SRR17520069.5214007
SRR17520069.5214008
SRR17520069.5214009
SRR17520069.5214010
SRR17520069.5214011
SRR17520069.5214012
SRR17520069.5214013

SRR17520069.5214014
SRR17520069.5214015
SRR17520069.5214016
SRR17520069.5214017
SRR17520069.5214018
SRR17520069.5214019
SRR17520069.5214020
SRR17520069.5214021
SRR17520069.5214022
SRR17520069.5214023
SRR17520069.5214024
SRR17520069.5214025
SRR17520069.5214026
SRR17520069.5214027
SRR17520069.5214028
SRR17520069.5214029
SRR17520069.5214030
SRR17520069.5214031
SRR17520069.5214032
SRR17520069.5214033
SRR17520069.5214034
SRR17520069.5214035
SRR17520069.5214036
SRR17520069.5214037
SRR17520069.5214038
SRR17520069.5214039
SRR17520069.5214040
SRR17520069.5214041
SRR17520069.5214042
SRR17520069.5214043
SRR17520069.5214045
SRR17520069.5214046
SRR17520069.5214047
SRR17520069.5214048
SRR17520069.5214049
SRR17520069.5214050
SRR17520069.5214051
SRR17520069.5214052
SRR17520069.5214053
SRR17520069.5214054
SRR17520069.5214055
SRR17520069.5214056
SRR17520069.5214057
SRR17520069.5214058
SRR17520069.5214059
SRR17520069.5214060
SRR17520069.5214061

SRR17520069.5214062
SRR17520069.5214063
SRR17520069.5214064
SRR17520069.5214065
SRR17520069.5214066
SRR17520069.5261387
SRR17520069.5261388
SRR17520069.5261389
SRR17520069.5261390
SRR17520069.5261391
SRR17520069.5261392
SRR17520069.5261393
SRR17520069.5261394
SRR17520069.5261395
SRR17520069.5261396
SRR17520069.5261397
SRR17520069.5261398
SRR17520069.5261399
SRR17520069.5261400
SRR17520069.5261401
SRR17520069.5261402
SRR17520069.5261404
SRR17520069.5261405
SRR17520069.5261406
SRR17520069.5261407
SRR17520069.5261408
SRR17520069.5261409
SRR17520069.5261410
SRR17520069.5261411
SRR17520069.5261412
SRR17520069.5261414
SRR17520069.5261415
SRR17520069.5261416
SRR17520069.5261417
SRR17520069.5261419
SRR17520069.5261420
SRR17520069.5261422
SRR17520069.5261423
SRR17520069.5261424
SRR17520069.5261425
SRR17520069.5261426
SRR17520069.5261427
SRR17520069.5261428
SRR17520069.5261429
SRR17520069.5261431
SRR17520069.5261432
SRR17520069.5261433

SRR17520069.5261434
SRR17520069.5261435
SRR17520069.5261436
SRR17520069.5261437
SRR17520069.5261438
SRR17520069.5261439
SRR17520069.5261440
SRR17520069.5261442
SRR17520069.5261443
SRR17520069.5261444
SRR17520069.5261445
SRR17520069.5261446
SRR17520069.5261449
SRR17520069.5261450
SRR17520069.5261451
SRR17520069.5261452
SRR17520069.5261453
SRR17520069.5261454
SRR17520069.5261455
SRR17520069.5261457
SRR17520069.5261458
SRR17520069.5316911
SRR17520069.5316913
SRR17520069.5316928
SRR17520069.5321504
SRR17520069.5321541
SRR17520069.5344210
SRR17520069.5344211
SRR17520069.5344212
SRR17520069.5344213
SRR17520069.5344214
SRR17520069.5344215
SRR17520069.5344216
SRR17520069.5344217
SRR17520069.5344218
SRR17520069.5344219
SRR17520069.5344220
SRR17520069.5344221
SRR17520069.5344222
SRR17520069.5344223
SRR17520069.5344224
SRR17520069.5344225
SRR17520069.5344226
SRR17520069.5344227
SRR17520069.5344228
SRR17520069.5344229
SRR17520069.5344230

SRR17520069.5344231
SRR17520069.5344232
SRR17520069.5344233
SRR17520069.5344234
SRR17520069.5344235
SRR17520069.5344236
SRR17520069.5344237
SRR17520069.5344238
SRR17520069.5344239
SRR17520069.5344240
SRR17520069.5344241
SRR17520069.5344242
SRR17520069.5344243
SRR17520069.5344244
SRR17520069.5344245
SRR17520069.5344246
SRR17520069.5344247
SRR17520069.5344248
SRR17520069.5344249
SRR17520069.5344250
SRR17520069.5344251
SRR17520069.5344252
SRR17520069.5344253
SRR17520069.5344254
SRR17520069.5344255
SRR17520069.5344256
SRR17520069.5344257
SRR17520069.5344258
SRR17520069.5344259
SRR17520069.5344260
SRR17520069.5344261
SRR17520069.5344262
SRR17520069.5344263
SRR17520069.5344264
SRR17520069.5344265
SRR17520069.5344266
SRR17520069.5344267
SRR17520069.5344268
SRR17520069.5344269
SRR17520069.5344270
SRR17520069.5344271
SRR17520069.5344272
SRR17520069.5344273
SRR17520069.5344274
SRR17520069.5344275
SRR17520069.5344276
SRR17520069.5344277

SRR17520069.5344278
SRR17520069.5344279
SRR17520069.5344280
SRR17520069.5344281
SRR17520069.5344282
SRR17520069.5344283
SRR17520069.5344284
SRR17520069.5344285
SRR17520069.5344286
SRR17520069.5344287
SRR17520069.5344288
SRR17520069.5344289
SRR17520069.5344290
SRR17520069.5344291
SRR17520069.5344292
SRR17520069.5344293
SRR17520069.5344294
SRR17520069.5344295
SRR17520069.5344296
SRR17520069.5344297
SRR17520069.5344298
SRR17520069.5344299
SRR17520069.5344300
SRR17520069.5344301
SRR17520069.5344302
SRR17520069.5344303
SRR17520069.5344304
SRR17520069.5344305
SRR17520069.5344306
SRR17520069.5344307
SRR17520069.5344308
SRR17520069.5344309
SRR17520069.5344310
SRR17520069.5344311
SRR17520069.5344312
SRR17520069.5344313
SRR17520069.5344314
SRR17520069.5344315
SRR17520069.5344316
SRR17520069.5344317
SRR17520069.5344318
SRR17520069.5344319
SRR17520069.5344320
SRR17520069.5344321
SRR17520069.5344322
SRR17520069.5344323
SRR17520069.5344324

SRR17520069.5344326
SRR17520069.5344327
SRR17520069.5344328
SRR17520069.5344329
SRR17520069.5344330
SRR17520069.5344331
SRR17520069.5344332
SRR17520069.5344333
SRR17520069.5344334
SRR17520069.5344335
SRR17520069.5344336
SRR17520069.5344337
SRR17520069.5344338
SRR17520069.5344339
SRR17520069.5344340
SRR17520069.5344341
SRR17520069.5344342
SRR17520069.5344343
SRR17520069.5344344
SRR17520069.5344345
SRR17520069.5344346
SRR17520069.5344347
SRR17520069.5344348
SRR17520069.5344349
SRR17520069.5344350
SRR17520069.5344351
SRR17520069.5344352
SRR17520069.5344353
SRR17520069.5344354
SRR17520069.5344355
SRR17520069.5344356
SRR17520069.5344358
SRR17520069.5344359
SRR17520069.5344360
SRR17520069.5344361
SRR17520069.5344362
SRR17520069.5344363
SRR17520069.5344364
SRR17520069.5344365
SRR17520069.5344366
SRR17520069.5344367
SRR17520069.5344368
SRR17520069.5344369
SRR17520069.5344370
SRR17520069.5344372
SRR17520069.5344373
SRR17520069.5344374

SRR17520069.5344375
SRR17520069.5344376
SRR17520069.5344377
SRR17520069.5344378
SRR17520069.5344379
SRR17520069.5344380
SRR17520069.5344381
SRR17520069.5344382
SRR17520069.5344383
SRR17520069.5344384
SRR17520069.5344385
SRR17520069.5351555
SRR17520069.5351620
SRR17520069.5356291
SRR17520069.5358482
SRR17520069.5369497
SRR17520069.5369498
SRR17520069.5369500
SRR17520069.5369501
SRR17520069.5369507
SRR17520069.5378973
SRR17520069.5378974
SRR17520069.5378976
SRR17520069.5378977
SRR17520069.5378978
SRR17520069.5378979
SRR17520069.5378980
SRR17520069.5378981
SRR17520069.5378982
SRR17520069.5378983
SRR17520069.5378984
SRR17520069.5378985
SRR17520069.5378986
SRR17520069.5378987
SRR17520069.5378988
SRR17520069.5378989
SRR17520069.5378991
SRR17520069.5378992
SRR17520069.5385266
SRR17520069.5389865
SRR17520069.5389866
SRR17520069.5389867
SRR17520069.5389868
SRR17520069.5389869
SRR17520069.5389870
SRR17520069.5389871
SRR17520069.5389873

SRR17520069.5389874
SRR17520069.5389875
SRR17520069.5389876
SRR17520069.5389877
SRR17520069.5389878
SRR17520069.5389879
SRR17520069.5389880
SRR17520069.5389881
SRR17520069.5389882
SRR17520069.5389883
SRR17520069.5389884
SRR17520069.5389885
SRR17520069.5389886
SRR17520069.5389887
SRR17520069.5389888
SRR17520069.5389889
SRR17520069.5389890
SRR17520069.5389891
SRR17520069.5389892
SRR17520069.5389893
SRR17520069.5389894
SRR17520069.5389895
SRR17520069.5389896
SRR17520069.5389897
SRR17520069.5389898
SRR17520069.5389899
SRR17520069.5389900
SRR17520069.5389901
SRR17520069.5389902
SRR17520069.5389903
SRR17520069.5389904
SRR17520069.5389905
SRR17520069.5389906
SRR17520069.5389907
SRR17520069.5389908
SRR17520069.5389909
SRR17520069.5389910
SRR17520069.5389911
SRR17520069.5389912
SRR17520069.5389913
SRR17520069.5389914
SRR17520069.5389915
SRR17520069.5389916
SRR17520069.5389917
SRR17520069.5389918
SRR17520069.5389919
SRR17520069.5389920

SRR17520069.5389921
SRR17520069.5389922
SRR17520069.5389923
SRR17520069.5389924
SRR17520069.5389925
SRR17520069.5397419
SRR17520069.5397420
SRR17520069.5397421
SRR17520069.5397422
SRR17520069.5397423
SRR17520069.5397424
SRR17520069.5397425
SRR17520069.5397426
SRR17520069.5397427
SRR17520069.5397428
SRR17520069.5397429
SRR17520069.5397430
SRR17520069.5397431
SRR17520069.5397432
SRR17520069.5397433
SRR17520069.5397434
SRR17520069.5397435
SRR17520069.5397436
SRR17520069.5397437
SRR17520069.5397438
SRR17520069.5397439
SRR17520069.5397440
SRR17520069.5397441
SRR17520069.5397442
SRR17520069.5397443
SRR17520069.5397444
SRR17520069.5397445
SRR17520069.5397446
SRR17520069.5397447
SRR17520069.5397448
SRR17520069.5397449
SRR17520069.5397450
SRR17520069.5397451
SRR17520069.5397452
SRR17520069.5397453
SRR17520069.5397454
SRR17520069.5397455
SRR17520069.5397456
SRR17520069.5397457
SRR17520069.5397458
SRR17520069.5397459
SRR17520069.5397460

SRR17520069.5397461
SRR17520069.5397463
SRR17520069.5397464
SRR17520069.5397465
SRR17520069.5397466
SRR17520069.5397467
SRR17520069.5397468
SRR17520069.5397469
SRR17520069.5397470
SRR17520069.5397471
SRR17520069.5397472
SRR17520069.5397473
SRR17520069.5397474
SRR17520069.5397475
SRR17520069.5397476
SRR17520069.5397477
SRR17520069.5397478
SRR17520069.5397479
SRR17520069.5397480
SRR17520069.5397481
SRR17520069.5397482
SRR17520069.5397483
SRR17520069.5397484
SRR17520069.5397485
SRR17520069.5397487
SRR17520069.5397488
SRR17520069.5397489
SRR17520069.5397490
SRR17520069.5397491
SRR17520069.5397492
SRR17520069.5397493
SRR17520069.5397494
SRR17520069.5397495
SRR17520069.5397496
SRR17520069.5397497
SRR17520069.5397498
SRR17520069.5397499
SRR17520069.5397500
SRR17520069.5397501
SRR17520069.5397502
SRR17520069.5397503
SRR17520069.5397505
SRR17520069.5397506
SRR17520069.5397507
SRR17520069.5397508
SRR17520069.5397509
SRR17520069.5397510

SRR17520069.5397511
SRR17520069.5397512
SRR17520069.5397513
SRR17520069.5397514
SRR17520069.5397515
SRR17520069.5397516
SRR17520069.5397517
SRR17520069.5397518
SRR17520069.5397519
SRR17520069.5397520
SRR17520069.5397521
SRR17520069.5397522
SRR17520069.5397523
SRR17520069.5397524
SRR17520069.5397525
SRR17520069.5397526
SRR17520069.5397527
SRR17520069.5397528
SRR17520069.5397529
SRR17520069.5397531
SRR17520069.5397532
SRR17520069.5397533
SRR17520069.5397534
SRR17520069.5397536
SRR17520069.5397537
SRR17520069.5397538
SRR17520069.5397539
SRR17520069.5397540
SRR17520069.5397541
SRR17520069.5397542
SRR17520069.5397543
SRR17520069.5397544
SRR17520069.5397545
SRR17520069.5397546
SRR17520069.5397547
SRR17520069.5397548
SRR17520069.5397549
SRR17520069.5397550
SRR17520069.5397551
SRR17520069.5397552
SRR17520069.5397553
SRR17520069.5397554
SRR17520069.5397555
SRR17520069.5397556
SRR17520069.5397557
SRR17520069.5397558
SRR17520069.5397559

SRR17520069.5397560
SRR17520069.5397562
SRR17520069.5397563
SRR17520069.5397564
SRR17520069.5397565
SRR17520069.5397566
SRR17520069.5397567
SRR17520069.5397568
SRR17520069.5397569
SRR17520069.5397571
SRR17520069.5397572
SRR17520069.5397573
SRR17520069.5397574
SRR17520069.5397575
SRR17520069.5397576
SRR17520069.5397577
SRR17520069.5397578
SRR17520069.5397579
SRR17520069.5397580
SRR17520069.5397581
SRR17520069.5402926
SRR17520069.5416791
SRR17520069.5416792
SRR17520069.5416793
SRR17520069.5416794
SRR17520069.5416795
SRR17520069.5416796
SRR17520069.5416797
SRR17520069.5416798
SRR17520069.5416799
SRR17520069.5416800
SRR17520069.5416801
SRR17520069.5424120
SRR17520069.5428556
SRR17520069.5428558
SRR17520069.5428559
SRR17520069.5428560
SRR17520069.5428561
SRR17520069.5428562
SRR17520069.5428563
SRR17520069.5428564
SRR17520069.5430413
SRR17520069.5430414
SRR17520069.5430415
SRR17520069.5430416
SRR17520069.5430417
SRR17520069.5430418

SRR17520069.5430419
SRR17520069.5430420
SRR17520069.5430421
SRR17520069.5430422
SRR17520069.5430423
SRR17520069.5430424
SRR17520069.5430425
SRR17520069.5430426
SRR17520069.5430427
SRR17520069.5430428
SRR17520069.5430429
SRR17520069.5430430
SRR17520069.5430431
SRR17520069.5430432
SRR17520069.5430433
SRR17520069.5430434
SRR17520069.5430435
SRR17520069.5430436
SRR17520069.5430437
SRR17520069.5430438
SRR17520069.5444369
SRR17520069.5444370
SRR17520069.5444371
SRR17520069.5444372
SRR17520069.5444373
SRR17520069.5444374
SRR17520069.5444375
SRR17520069.5444376
SRR17520069.5444377
SRR17520069.5444378
SRR17520069.5444379
SRR17520069.5444380
SRR17520069.5444381
SRR17520069.5444382
SRR17520069.5444383
SRR17520069.5444384
SRR17520069.5444385
SRR17520069.5444386
SRR17520069.5444387
SRR17520069.5444388
SRR17520069.5444389
SRR17520069.5444390
SRR17520069.5444391
SRR17520069.5444392
SRR17520069.5444393
SRR17520069.5444394
SRR17520069.5457678

SRR17520069.5457679
SRR17520069.5457683
SRR17520069.5457685
SRR17520069.5457686
SRR17520069.5457687
SRR17520069.5457688
SRR17520069.5457689
SRR17520069.5457690
SRR17520069.5457691
SRR17520069.5457693
SRR17520069.5457694
SRR17520069.5457695
SRR17520069.5457696
SRR17520069.5457697
SRR17520069.5457698
SRR17520069.5457699
SRR17520069.5457700
SRR17520069.5457701
SRR17520069.5457702
SRR17520069.5457703
SRR17520069.5457704
SRR17520069.5457705
SRR17520069.5457707
SRR17520069.5457708
SRR17520069.5457709
SRR17520069.5457711
SRR17520069.5462843
SRR17520069.5462844
SRR17520069.5462845
SRR17520069.5462846
SRR17520069.5462847
SRR17520069.5489865
SRR17520069.5489867
SRR17520069.5489868
SRR17520069.5489869
SRR17520069.5489870
SRR17520069.5489871
SRR17520069.5489872
SRR17520069.5489873
SRR17520069.5489874
SRR17520069.5489875
SRR17520069.5489876
SRR17520069.5489877
SRR17520069.5489878
SRR17520069.5489879
SRR17520069.5489880
SRR17520069.5489881

SRR17520069.5489882
SRR17520069.5489883
SRR17520069.5494092
SRR17520069.5494093
SRR17520069.5494094
SRR17520069.5494095
SRR17520069.5494096
SRR17520069.5494097
SRR17520069.5494098
SRR17520069.5494099
SRR17520069.5494100
SRR17520069.5494101
SRR17520069.5494102
SRR17520069.5494103
SRR17520069.5494104
SRR17520069.5494105
SRR17520069.5494106
SRR17520069.5494107
SRR17520069.5494108
SRR17520069.5494109
SRR17520069.5494110
SRR17520069.5494111
SRR17520069.5494112
SRR17520069.5494113
SRR17520069.5494114
SRR17520069.5494115
SRR17520069.5494116
SRR17520069.5494117
SRR17520069.5494118
SRR17520069.5494119
SRR17520069.5494120
SRR17520069.5494121
SRR17520069.5494122
SRR17520069.5494123
SRR17520069.5494124
SRR17520069.5494125
SRR17520069.5494126
SRR17520069.5494127
SRR17520069.5494128
SRR17520069.5494129
SRR17520069.5494130
SRR17520069.5494131
SRR17520069.5494132
SRR17520069.5494133
SRR17520069.5494134
SRR17520069.5494135
SRR17520069.5494136

SRR17520069.5494137
SRR17520069.5494138
SRR17520069.5494139
SRR17520069.5494140
SRR17520069.5494141
SRR17520069.5494142
SRR17520069.5494143
SRR17520069.5494144
SRR17520069.5494145
SRR17520069.5494146
SRR17520069.5494147
SRR17520069.5494148
SRR17520069.5494149
SRR17520069.5494150
SRR17520069.5494151
SRR17520069.5494152
SRR17520069.5494153
SRR17520069.5494154
SRR17520069.5494155
SRR17520069.5494156
SRR17520069.5494157
SRR17520069.5494158
SRR17520069.5494159
SRR17520069.5494160
SRR17520069.5494161
SRR17520069.5494162
SRR17520069.5494163
SRR17520069.5494164
SRR17520069.5494165
SRR17520069.5494166
SRR17520069.5494167
SRR17520069.5494168
SRR17520069.5494169
SRR17520069.5494170
SRR17520069.5494171
SRR17520069.5494172
SRR17520069.5494173
SRR17520069.5494174
SRR17520069.5520667
SRR17520069.5520668
SRR17520069.5520670
SRR17520069.5520672
SRR17520069.5520673
SRR17520069.5520674
SRR17520069.5520675
SRR17520069.5520676
SRR17520069.5520677

SRR17520069.5520678
SRR17520069.5520680
SRR17520069.5520686
SRR17520069.5520692
SRR17520069.5520696
SRR17520069.5520697
SRR17520069.5520698
SRR17520069.5520700
SRR17520069.5520701
SRR17520069.5520702
SRR17520069.5520703
SRR17520069.5520704
SRR17520069.5520705
SRR17520069.5520706
SRR17520069.5520707
SRR17520069.5520708
SRR17520069.5520709
SRR17520069.5520710
SRR17520069.5520711
SRR17520069.5520712
SRR17520069.5520713
SRR17520069.5521412
SRR17520069.5523414
SRR17520069.5529997
SRR17520069.5529998
SRR17520069.5529999
SRR17520069.5530000
SRR17520069.5530001
SRR17520069.5530002
SRR17520069.5530003
SRR17520069.5530004
SRR17520069.5530005
SRR17520069.5530006
SRR17520069.5530007
SRR17520069.5530008
SRR17520069.5530009
SRR17520069.5530010
SRR17520069.5530011
SRR17520069.5530012
SRR17520069.5530013
SRR17520069.5530014
SRR17520069.5530015
SRR17520069.5530016
SRR17520069.5530017
SRR17520069.5530018
SRR17520069.5530019
SRR17520069.5530020

SRR17520069.5530021
SRR17520069.5530022
SRR17520069.5530023
SRR17520069.5530024
SRR17520069.5530025
SRR17520069.5530026
SRR17520069.5530027
SRR17520069.5530028
SRR17520069.5530029
SRR17520069.5530030
SRR17520069.5530031
SRR17520069.5530032
SRR17520069.5530033
SRR17520069.5530034
SRR17520069.5530035
SRR17520069.5530036
SRR17520069.5530037
SRR17520069.5530038
SRR17520069.5530039
SRR17520069.5530040
SRR17520069.5530041
SRR17520069.5530042
SRR17520069.5530043
SRR17520069.5530044
SRR17520069.5530045
SRR17520069.5530046
SRR17520069.5530047
SRR17520069.5530048
SRR17520069.5530049
SRR17520069.5530050
SRR17520069.5530051
SRR17520069.5530052
SRR17520069.5530053
SRR17520069.5530054
SRR17520069.5530055
SRR17520069.5530056
SRR17520069.5530057
SRR17520069.5530058
SRR17520069.5530059
SRR17520069.5530060
SRR17520069.5530061
SRR17520069.5530062
SRR17520069.5530063
SRR17520069.5530064
SRR17520069.5530065
SRR17520069.5530066
SRR17520069.5530067

SRR17520069.5538371
SRR17520069.5538372
SRR17520069.5538373
SRR17520069.5538374
SRR17520069.5538375
SRR17520069.5538376
SRR17520069.5538377
SRR17520069.5538378
SRR17520069.5538379
SRR17520069.5538380
SRR17520069.5538381
SRR17520069.5538382
SRR17520069.5538383
SRR17520069.5538384
SRR17520069.5538385
SRR17520069.5538386
SRR17520069.5538387
SRR17520069.5538388
SRR17520069.5538389
SRR17520069.5538390
SRR17520069.5538391
SRR17520069.5538392
SRR17520069.5538393
SRR17520069.5538395
SRR17520069.5538397
SRR17520069.5538398
SRR17520069.5538399
SRR17520069.5538400
SRR17520069.5538401
SRR17520069.5538402
SRR17520069.5538403
SRR17520069.5538404
SRR17520069.5538405
SRR17520069.5538409
SRR17520069.5538411
SRR17520069.5538412
SRR17520069.5538413
SRR17520069.5538415
SRR17520069.5538417
SRR17520069.5538418
SRR17520069.5538419
SRR17520069.5538420
SRR17520069.5538421
SRR17520069.5538422
SRR17520069.5538423
SRR17520069.5538424
SRR17520069.5538425

SRR17520069.5538427
SRR17520069.5538428
SRR17520069.5544019
SRR17520069.5544020
SRR17520069.5544021
SRR17520069.5544022
SRR17520069.5544023
SRR17520069.5544024
SRR17520069.5544025
SRR17520069.5544026
SRR17520069.5544027
SRR17520069.5544028
SRR17520069.5544029
SRR17520069.5544030
SRR17520069.5544031
SRR17520069.5544032
SRR17520069.5544033
SRR17520069.5544034
SRR17520069.5544035
SRR17520069.5544036
SRR17520069.5544037
SRR17520069.5544038
SRR17520069.5544040
SRR17520069.5544041
SRR17520069.5544042
SRR17520069.5544043
SRR17520069.5544044
SRR17520069.5544045
SRR17520069.5544046
SRR17520069.5544047
SRR17520069.5544048
SRR17520069.5544049
SRR17520069.5544050
SRR17520069.5544051
SRR17520069.5544052
SRR17520069.5544053
SRR17520069.5544054
SRR17520069.5544055
SRR17520069.5544056
SRR17520069.5544057
SRR17520069.5544058
SRR17520069.5544059
SRR17520069.5544060
SRR17520069.5544061
SRR17520069.5544062
SRR17520069.5544063
SRR17520069.5544064

SRR17520069.5544065
SRR17520069.5544066
SRR17520069.5544067
SRR17520069.5544068
SRR17520069.5544069
SRR17520069.5544070
SRR17520069.5544071
SRR17520069.5544072
SRR17520069.5544073
SRR17520069.5544074
SRR17520069.5544075
SRR17520069.5544076
SRR17520069.5544077
SRR17520069.5544078
SRR17520069.5544079
SRR17520069.5544080
SRR17520069.5544081
SRR17520069.5544082
SRR17520069.5544083
SRR17520069.5544084
SRR17520069.5544085
SRR17520069.5544086
SRR17520069.5544087
SRR17520069.5544088
SRR17520069.5544089
SRR17520069.5544090
SRR17520069.5544091
SRR17520069.5544092
SRR17520069.5544093
SRR17520069.5544094
SRR17520069.5544095
SRR17520069.5544096
SRR17520069.5544097
SRR17520069.5544098
SRR17520069.5544099
SRR17520069.5544100
SRR17520069.5544101
SRR17520069.5544102
SRR17520069.5544103
SRR17520069.5544104
SRR17520069.5544105
SRR17520069.5544106
SRR17520069.5544107
SRR17520069.5544108
SRR17520069.5544109
SRR17520069.5544110
SRR17520069.5544111

SRR17520069.5544112
SRR17520069.5544113
SRR17520069.5544114
SRR17520069.5544115
SRR17520069.5544116
SRR17520069.5544117
SRR17520069.5544118
SRR17520069.5544119
SRR17520069.5544120
SRR17520069.5544121
SRR17520069.5544122
SRR17520069.5544123
SRR17520069.5544124
SRR17520069.5544125
SRR17520069.5544126
SRR17520069.5544127
SRR17520069.5544128
SRR17520069.5544129
SRR17520069.5544130
SRR17520069.5544131
SRR17520069.5544132
SRR17520069.5544133
SRR17520069.5544134
SRR17520069.5544135
SRR17520069.5544136
SRR17520069.5544137
SRR17520069.5544138
SRR17520069.5544139
SRR17520069.5544140
SRR17520069.5544141
SRR17520069.5544142
SRR17520069.5544143
SRR17520069.5544144
SRR17520069.5544145
SRR17520069.5544146
SRR17520069.5544147
SRR17520069.5544148
SRR17520069.5544149
SRR17520069.5544150
SRR17520069.5544151
SRR17520069.5544152
SRR17520069.5544153
SRR17520069.5544154
SRR17520069.5544155
SRR17520069.5544156
SRR17520069.5544157
SRR17520069.5544158

SRR17520069.5544159
SRR17520069.5544160
SRR17520069.5544161
SRR17520069.5544162
SRR17520069.5544163
SRR17520069.5544164
SRR17520069.5544165
SRR17520069.5544166
SRR17520069.5544167
SRR17520069.5544168
SRR17520069.5544169
SRR17520069.5544170
SRR17520069.5544171
SRR17520069.5544172
SRR17520069.5544173
SRR17520069.5544174
SRR17520069.5544175
SRR17520069.5544176
SRR17520069.5544177
SRR17520069.5544178
SRR17520069.5544179
SRR17520069.5544180
SRR17520069.5544181
SRR17520069.5544182
SRR17520069.5553825
SRR17520069.5553826
SRR17520069.5553827
SRR17520069.5553829
SRR17520069.5553830
SRR17520069.5553831
SRR17520069.5553832
SRR17520069.5553833
SRR17520069.5553835
SRR17520069.5553836
SRR17520069.5553837
SRR17520069.5553838
SRR17520069.5553839
SRR17520069.5553840
SRR17520069.5553841
SRR17520069.5553842
SRR17520069.5553843
SRR17520069.5553845
SRR17520069.5553846
SRR17520069.5553847
SRR17520069.5553848
SRR17520069.5553849
SRR17520069.5553850

SRR17520069.5553851
SRR17520069.5553852
SRR17520069.5553853
SRR17520069.5553854
SRR17520069.5553855
SRR17520069.5553856
SRR17520069.5553857
SRR17520069.5553858
SRR17520069.5553859
SRR17520069.5553860
SRR17520069.5553861
SRR17520069.5553862
SRR17520069.5553863
SRR17520069.5553864
SRR17520069.5553865
SRR17520069.5553867
SRR17520069.5553868
SRR17520069.5553869
SRR17520069.5553870
SRR17520069.5553871
SRR17520069.5553872
SRR17520069.5553873
SRR17520069.5553874
SRR17520069.5553875
SRR17520069.5553876
SRR17520069.5553877
SRR17520069.5553878
SRR17520069.5553879
SRR17520069.5553880
SRR17520069.5553881
SRR17520069.5553882
SRR17520069.5553883
SRR17520069.5553884
SRR17520069.5553885
SRR17520069.5553886
SRR17520069.5553887
SRR17520069.5553888
SRR17520069.5553889
SRR17520069.5553890
SRR17520069.5553891
SRR17520069.5553892
SRR17520069.5553893
SRR17520069.5553894
SRR17520069.5553895
SRR17520069.5553896
SRR17520069.5553897
SRR17520069.5553898

SRR17520069.5553899
SRR17520069.5553900
SRR17520069.5553901
SRR17520069.5553902
SRR17520069.5553903
SRR17520069.5553904
SRR17520069.5553905
SRR17520069.5553906
SRR17520069.5553907
SRR17520069.5553908
SRR17520069.5553909
SRR17520069.5553910
SRR17520069.5553911
SRR17520069.5553912
SRR17520069.5553913
SRR17520069.5553914
SRR17520069.5553915
SRR17520069.5553916
SRR17520069.5553917
SRR17520069.5553918
SRR17520069.5553919
SRR17520069.5553920
SRR17520069.5553921
SRR17520069.5553922
SRR17520069.5553923
SRR17520069.5553924
SRR17520069.5553925
SRR17520069.5553926
SRR17520069.5553927
SRR17520069.5553928
SRR17520069.5553929
SRR17520069.5553930
SRR17520069.5553931
SRR17520069.5553932
SRR17520069.5553933
SRR17520069.5553934
SRR17520069.5553935
SRR17520069.5553936
SRR17520069.5553937
SRR17520069.5553938
SRR17520069.5553939
SRR17520069.5553940
SRR17520069.5553941
SRR17520069.5553942
SRR17520069.5553943
SRR17520069.5553944
SRR17520069.5553945

SRR17520069.5553946
SRR17520069.5553947
SRR17520069.5553948
SRR17520069.5553950
SRR17520069.5553951
SRR17520069.5553952
SRR17520069.5553953
SRR17520069.5553954
SRR17520069.5553955
SRR17520069.5553956
SRR17520069.5553957
SRR17520069.5553958
SRR17520069.5553959
SRR17520069.5553960
SRR17520069.5553962
SRR17520069.5553963
SRR17520069.5553964
SRR17520069.5553965
SRR17520069.5553966
SRR17520069.5553967
SRR17520069.5553968
SRR17520069.5553969
SRR17520069.5553970
SRR17520069.5553971
SRR17520069.5553972
SRR17520069.5553973
SRR17520069.5553974
SRR17520069.5553975
SRR17520069.5553976
SRR17520069.5553977
SRR17520069.5553978
SRR17520069.5553979
SRR17520069.5553980
SRR17520069.5553981
SRR17520069.5553982
SRR17520069.5553983
SRR17520069.5553984
SRR17520069.5553985
SRR17520069.5553986
SRR17520069.5553987
SRR17520069.5553988
SRR17520069.5553989
SRR17520069.5553990
SRR17520069.5553991
SRR17520069.5553992
SRR17520069.5553993
SRR17520069.5553994

SRR17520069.5553995
SRR17520069.5553996
SRR17520069.5553997
SRR17520069.5553998
SRR17520069.5553999
SRR17520069.5554000
SRR17520069.5554001
SRR17520069.5554002
SRR17520069.5561789
SRR17520069.5590508
SRR17520069.5599629
SRR17520069.5599630
SRR17520069.5599631
SRR17520069.5609230
SRR17520069.5609236
SRR17520069.5626061
SRR17520069.5643547
SRR17520069.5649791
SRR17520069.5649793
SRR17520069.5659374
SRR17520069.5678814
SRR17520069.5678815
SRR17520069.5678816
SRR17520069.5678817
SRR17520069.5678818
SRR17520069.5678819
SRR17520069.5678820
SRR17520069.5678821
SRR17520069.5678822
SRR17520069.5678823
SRR17520069.5678824
SRR17520069.5678825
SRR17520069.5678826
SRR17520069.5678827
SRR17520069.5678828
SRR17520069.5678829
SRR17520069.5678830
SRR17520069.5678831
SRR17520069.5678832
SRR17520069.5678833
SRR17520069.5678834
SRR17520069.5678835
SRR17520069.5678836
SRR17520069.5678837
SRR17520069.5678838
SRR17520069.5678839
SRR17520069.5678840

SRR17520069.5678841
SRR17520069.5678842
SRR17520069.5678843
SRR17520069.5678844
SRR17520069.5678845
SRR17520069.5678846
SRR17520069.5678847
SRR17520069.5678848
SRR17520069.5678849
SRR17520069.5678850
SRR17520069.5678851
SRR17520069.5678852
SRR17520069.5678853
SRR17520069.5678854
SRR17520069.5678855
SRR17520069.5678856
SRR17520069.5678857
SRR17520069.5678858
SRR17520069.5678859
SRR17520069.5678860
SRR17520069.5678861
SRR17520069.5678862
SRR17520069.5678863
SRR17520069.5678864
SRR17520069.5678865
SRR17520069.5678866
SRR17520069.5678867
SRR17520069.5678868
SRR17520069.5678869
SRR17520069.5678870
SRR17520069.5678871
SRR17520069.5678872
SRR17520069.5678873
SRR17520069.5678874
SRR17520069.5678875
SRR17520069.5678876
SRR17520069.5678877
SRR17520069.5678879
SRR17520069.5678880
SRR17520069.5678881
SRR17520069.5679658
SRR17520069.5679662
SRR17520069.5679664
SRR17520069.5686851
SRR17520069.5686852
SRR17520069.5686853
SRR17520069.5686854

SRR17520069.5686855
SRR17520069.5691364
SRR17520069.5691365
SRR17520069.5691366
SRR17520069.5691367
SRR17520069.5691368
SRR17520069.5691369
SRR17520069.5691370
SRR17520069.5691371
SRR17520069.5691372
SRR17520069.5691373
SRR17520069.5691374
SRR17520069.5691375
SRR17520069.5691376
SRR17520069.5691377
SRR17520069.5691378
SRR17520069.5691379
SRR17520069.5691380
SRR17520069.5691381
SRR17520069.5691382
SRR17520069.5691383
SRR17520069.5691384
SRR17520069.5691385
SRR17520069.5691386
SRR17520069.5691387
SRR17520069.5691388
SRR17520069.5691390
SRR17520069.5691391
SRR17520069.5693921
SRR17520069.5704340
SRR17520069.5704358
SRR17520069.5704360
SRR17520069.5711169
SRR17520069.5715470
SRR17520069.5715471
SRR17520069.5715472
SRR17520069.5715473
SRR17520069.5715475
SRR17520069.5715478
SRR17520069.5715479
SRR17520069.5715480
SRR17520069.5715481
SRR17520069.5715482
SRR17520069.5715483
SRR17520069.5715484
SRR17520069.5715485
SRR17520069.5715486

SRR17520069.5715487
SRR17520069.5715488
SRR17520069.5715489
SRR17520069.5715490
SRR17520069.5715491
SRR17520069.5715492
SRR17520069.5715493
SRR17520069.5715494
SRR17520069.5715500
SRR17520069.5715501
SRR17520069.5715502
SRR17520069.5715504
SRR17520069.5715505
SRR17520069.5715506
SRR17520069.5715508
SRR17520069.5715509
SRR17520069.5715510
SRR17520069.5715511
SRR17520069.5715512
SRR17520069.5719048
SRR17520069.5719050
SRR17520069.5719052
SRR17520069.5719053
SRR17520069.5719054
SRR17520069.5719055
SRR17520069.5719056
SRR17520069.5719057
SRR17520069.5719058
SRR17520069.5719059
SRR17520069.5719060
SRR17520069.5719061
SRR17520069.5719062
SRR17520069.5719063
SRR17520069.5719064
SRR17520069.5719065
SRR17520069.5719066
SRR17520069.5719067
SRR17520069.5719068
SRR17520069.5719069
SRR17520069.5719070
SRR17520069.5719071
SRR17520069.5719072
SRR17520069.5719073
SRR17520069.5719074
SRR17520069.5719075
SRR17520069.5719076
SRR17520069.5728983

SRR17520069.5728984
SRR17520069.5728985
SRR17520069.5728986
SRR17520069.5728987
SRR17520069.5728988
SRR17520069.5728989
SRR17520069.5728990
SRR17520069.5728991
SRR17520069.5728992
SRR17520069.5728993
SRR17520069.5728994
SRR17520069.5728995
SRR17520069.5728996
SRR17520069.5728997
SRR17520069.5728999
SRR17520069.5729000
SRR17520069.5729001
SRR17520069.5729002
SRR17520069.5729003
SRR17520069.5729004
SRR17520069.5729005
SRR17520069.5729006
SRR17520069.5729007
SRR17520069.5729008
SRR17520069.5729009
SRR17520069.5729010
SRR17520069.5729011
SRR17520069.5729012
SRR17520069.5729013
SRR17520069.5729014
SRR17520069.5729015
SRR17520069.5729016
SRR17520069.5729017
SRR17520069.5729018
SRR17520069.5729019
SRR17520069.5729020
SRR17520069.5729022
SRR17520069.5729023
SRR17520069.5729024
SRR17520069.5729025
SRR17520069.5729026
SRR17520069.5729027
SRR17520069.5729028
SRR17520069.5738258
SRR17520069.5738259
SRR17520069.5738261
SRR17520069.5738263

SRR17520069.5738264
SRR17520069.5738265
SRR17520069.5738269
SRR17520069.5738270
SRR17520069.5738271
SRR17520069.5738272
SRR17520069.5738274
SRR17520069.5738275
SRR17520069.5738276
SRR17520069.5738277
SRR17520069.5738279
SRR17520069.5748626
SRR17520069.5748627
SRR17520069.5748628
SRR17520069.5748629
SRR17520069.5748630
SRR17520069.5748631
SRR17520069.5748632
SRR17520069.5748633
SRR17520069.5748634
SRR17520069.5748635
SRR17520069.5748636
SRR17520069.5748637
SRR17520069.5748638
SRR17520069.5748639
SRR17520069.5748640
SRR17520069.5748641
SRR17520069.5748642
SRR17520069.5748643
SRR17520069.5748644
SRR17520069.5748645
SRR17520069.5748646
SRR17520069.5748647
SRR17520069.5748648
SRR17520069.5748649
SRR17520069.5748650
SRR17520069.5748651
SRR17520069.5748652
SRR17520069.5748653
SRR17520069.5748654
SRR17520069.5748655
SRR17520069.5748656
SRR17520069.5748657
SRR17520069.5748658
SRR17520069.5748659
SRR17520069.5748660
SRR17520069.5748661

SRR17520069.5748662
SRR17520069.5748663
SRR17520069.5748664
SRR17520069.5748665
SRR17520069.5748666
SRR17520069.5748667
SRR17520069.5748668
SRR17520069.5748669
SRR17520069.5748670
SRR17520069.5748671
SRR17520069.5748672
SRR17520069.5748673
SRR17520069.5748674
SRR17520069.5748675
SRR17520069.5748676
SRR17520069.5748677
SRR17520069.5748678
SRR17520069.5748679
SRR17520069.5748680
SRR17520069.5748681
SRR17520069.5748682
SRR17520069.5748683
SRR17520069.5748684
SRR17520069.5748685
SRR17520069.5748686
SRR17520069.5748688
SRR17520069.5748689
SRR17520069.5748690
SRR17520069.5748691
SRR17520069.5748692
SRR17520069.5748693
SRR17520069.5748694
SRR17520069.5748695
SRR17520069.5748696
SRR17520069.5748697
SRR17520069.5748698
SRR17520069.5748699
SRR17520069.5748700
SRR17520069.5748701
SRR17520069.5748702
SRR17520069.5748703
SRR17520069.5748704
SRR17520069.5748705
SRR17520069.5748706
SRR17520069.5748707
SRR17520069.5748708
SRR17520069.5748709

SRR17520069.5748710
SRR17520069.5748711
SRR17520069.5748712
SRR17520069.5748713
SRR17520069.5748714
SRR17520069.5748715
SRR17520069.5748717
SRR17520069.5748718
SRR17520069.5748719
SRR17520069.5748720
SRR17520069.5748721
SRR17520069.5748722
SRR17520069.5748723
SRR17520069.5748724
SRR17520069.5748725
SRR17520069.5748726
SRR17520069.5748727
SRR17520069.5748728
SRR17520069.5748729
SRR17520069.5748730
SRR17520069.5748731
SRR17520069.5748732
SRR17520069.5748733
SRR17520069.5748734
SRR17520069.5748735
SRR17520069.5748736
SRR17520069.5748737
SRR17520069.5748738
SRR17520069.5748739
SRR17520069.5748740
SRR17520069.5748741
SRR17520069.5748742
SRR17520069.5748743
SRR17520069.5748744
SRR17520069.5748745
SRR17520069.5748746
SRR17520069.5748747
SRR17520069.5748748
SRR17520069.5748749
SRR17520069.5748750
SRR17520069.5748751
SRR17520069.5748752
SRR17520069.5748753
SRR17520069.5748754
SRR17520069.5748755
SRR17520069.5748756
SRR17520069.5748757

SRR17520069.5748758
SRR17520069.5748759
SRR17520069.5748760
SRR17520069.5748761
SRR17520069.5748762
SRR17520069.5748763
SRR17520069.5748764
SRR17520069.5748765
SRR17520069.5748766
SRR17520069.5748767
SRR17520069.5748768
SRR17520069.5748769
SRR17520069.5748770
SRR17520069.5748771
SRR17520069.5748772
SRR17520069.5748773
SRR17520069.5748774
SRR17520069.5748775
SRR17520069.5748776
SRR17520069.5748777
SRR17520069.5753769
SRR17520069.5767325
SRR17520069.5767326
SRR17520069.5767327
SRR17520069.5767329
SRR17520069.5767330
SRR17520069.5767331
SRR17520069.5767332
SRR17520069.5767333
SRR17520069.5767335
SRR17520069.5767336
SRR17520069.5767338
SRR17520069.5767339
SRR17520069.5767340
SRR17520069.5767341
SRR17520069.5767342
SRR17520069.5767343
SRR17520069.5767344
SRR17520069.5767345
SRR17520069.5767346
SRR17520069.5767347
SRR17520069.5767348
SRR17520069.5767349
SRR17520069.5767350
SRR17520069.5767351
SRR17520069.5767352
SRR17520069.5767353

SRR17520069.5767354
SRR17520069.5767355
SRR17520069.5767356
SRR17520069.5767357
SRR17520069.5767358
SRR17520069.5767359
SRR17520069.5767361
SRR17520069.5783993
SRR17520069.5785829
SRR17520069.5786226
SRR17520069.5798754
SRR17520069.5798755
SRR17520069.5798756
SRR17520069.5798757
SRR17520069.5798758
SRR17520069.5798759
SRR17520069.5798760
SRR17520069.5798761
SRR17520069.5798762
SRR17520069.5798763
SRR17520069.5798764
SRR17520069.5798765
SRR17520069.5798766
SRR17520069.5798767
SRR17520069.5798768
SRR17520069.5798769
SRR17520069.5798770
SRR17520069.5798771
SRR17520069.5798772
SRR17520069.5798773
SRR17520069.5798774
SRR17520069.5798775
SRR17520069.5798776
SRR17520069.5798777
SRR17520069.5798778
SRR17520069.5798779
SRR17520069.5798780
SRR17520069.5798781
SRR17520069.5798782
SRR17520069.5798783
SRR17520069.5798784
SRR17520069.5798785
SRR17520069.5798786
SRR17520069.5798787
SRR17520069.5798788
SRR17520069.5798789
SRR17520069.5798790

SRR17520069.5798791
SRR17520069.5798792
SRR17520069.5798793
SRR17520069.5798794
SRR17520069.5798795
SRR17520069.5798796
SRR17520069.5798797
SRR17520069.5798798
SRR17520069.5798799
SRR17520069.5809856
SRR17520069.5809857
SRR17520069.5809859
SRR17520069.5809860
SRR17520069.5809861
SRR17520069.5809862
SRR17520069.5809863
SRR17520069.5809864
SRR17520069.5809865
SRR17520069.5809866
SRR17520069.5809867
SRR17520069.5809868
SRR17520069.5809869
SRR17520069.5809870
SRR17520069.5809871
SRR17520069.5809872
SRR17520069.5809873
SRR17520069.5831467
SRR17520069.5831468
SRR17520069.5831469
SRR17520069.5831470
SRR17520069.5831471
SRR17520069.5831472
SRR17520069.5831473
SRR17520069.5831474
SRR17520069.5831475
SRR17520069.5831476
SRR17520069.5831477
SRR17520069.5831478
SRR17520069.5831479
SRR17520069.5831480
SRR17520069.5831481
SRR17520069.5831482
SRR17520069.5831483
SRR17520069.5831484
SRR17520069.5831487
SRR17520069.5831488
SRR17520069.5831489

SRR17520069.5831490
SRR17520069.5831491
SRR17520069.5831492
SRR17520069.5831493
SRR17520069.5831494
SRR17520069.5831495
SRR17520069.5831496
SRR17520069.5831497
SRR17520069.5831498
SRR17520069.5831499
SRR17520069.5831500
SRR17520069.5831501
SRR17520069.5831502
SRR17520069.5831503
SRR17520069.5831504
SRR17520069.5831505
SRR17520069.5831506
SRR17520069.5831507
SRR17520069.5831509
SRR17520069.5831510
SRR17520069.5831511
SRR17520069.5831512
SRR17520069.5831513
SRR17520069.5831514
SRR17520069.5831515
SRR17520069.5831516
SRR17520069.5831517
SRR17520069.5831518
SRR17520069.5831519
SRR17520069.5831520
SRR17520069.5831521
SRR17520069.5831522
SRR17520069.5831523
SRR17520069.5831524
SRR17520069.5831525
SRR17520069.5831526
SRR17520069.5831527
SRR17520069.5831528
SRR17520069.5831529
SRR17520069.5831530
SRR17520069.5831531
SRR17520069.5831532
SRR17520069.5831533
SRR17520069.5831534
SRR17520069.5831535
SRR17520069.5831536
SRR17520069.5831537

SRR17520069.5831538
SRR17520069.5831539
SRR17520069.5831540
SRR17520069.5831541
SRR17520069.5831542
SRR17520069.5831543
SRR17520069.5831544
SRR17520069.5831545
SRR17520069.5831546
SRR17520069.5831547
SRR17520069.5831548
SRR17520069.5831549
SRR17520069.5831550
SRR17520069.5831551
SRR17520069.5831552
SRR17520069.5831553
SRR17520069.5831554
SRR17520069.5831555
SRR17520069.5831556
SRR17520069.5831557
SRR17520069.5831558
SRR17520069.5831559
SRR17520069.5831560
SRR17520069.5831561
SRR17520069.5831562
SRR17520069.5848528
SRR17520069.5879984
SRR17520069.5892357
SRR17520069.5894977
SRR17520069.5911905
SRR17520069.5911906
SRR17520069.5911907
SRR17520069.5911908
SRR17520069.5911909
SRR17520069.5911910
SRR17520069.5911911
SRR17520069.5911912
SRR17520069.5911913
SRR17520069.5911915
SRR17520069.5911916
SRR17520069.5911917
SRR17520069.5911918
SRR17520069.5911919
SRR17520069.5911920
SRR17520069.5926512
SRR17520069.5926513
SRR17520069.5926514

SRR17520069.5926515
SRR17520069.5926516
SRR17520069.5926517
SRR17520069.5926518
SRR17520069.5926519
SRR17520069.5926520
SRR17520069.5926521
SRR17520069.5926522
SRR17520069.5926523
SRR17520069.5926524
SRR17520069.5926525
SRR17520069.5926526
SRR17520069.5926527
SRR17520069.5926528
SRR17520069.5926529
SRR17520069.5926530
SRR17520069.5926531
SRR17520069.5926532
SRR17520069.5926533
SRR17520069.5926534
SRR17520069.5926535
SRR17520069.5926536
SRR17520069.5926537
SRR17520069.5926538
SRR17520069.5926539
SRR17520069.5926540
SRR17520069.5926541
SRR17520069.5937156
SRR17520069.5937157
SRR17520069.5937158
SRR17520069.5959829
SRR17520069.5959831
SRR17520069.5959833
SRR17520069.5982924
SRR17520069.5985858
SRR17520069.5985859
SRR17520069.5985860
SRR17520069.5985861
SRR17520069.5985862
SRR17520069.5985863
SRR17520069.5985864
SRR17520069.5985865
SRR17520069.5985866
SRR17520069.5985867
SRR17520069.5985868
SRR17520069.5985869
SRR17520069.5985870

SRR17520069.5985871
SRR17520069.5985872
SRR17520069.5985873
SRR17520069.5985874
SRR17520069.5985875
SRR17520069.5985876
SRR17520069.5985877
SRR17520069.5985878
SRR17520069.5985879
SRR17520069.5985880
SRR17520069.5985881
SRR17520069.5985883
SRR17520069.5985884
SRR17520069.5985885
SRR17520069.5985886
SRR17520069.5985887
SRR17520069.5985888
SRR17520069.5985889
SRR17520069.5985890
SRR17520069.5985891
SRR17520069.5985892
SRR17520069.5985893
SRR17520069.5985894
SRR17520069.5985895
SRR17520069.5985896
SRR17520069.5985897
SRR17520069.5985898
SRR17520069.5985899
SRR17520069.5985900
SRR17520069.5985901
SRR17520069.5985902
SRR17520069.5985903
SRR17520069.5985904
SRR17520069.5985905
SRR17520069.5985906
SRR17520069.5985907
SRR17520069.5985908
SRR17520069.5985909
SRR17520069.5985910
SRR17520069.5985911
SRR17520069.5985912
SRR17520069.5985913
SRR17520069.5985914
SRR17520069.5985915
SRR17520069.5985916
SRR17520069.5985918
SRR17520069.5985919

SRR17520069.5985920
SRR17520069.5985921
SRR17520069.5985922
SRR17520069.5985923
SRR17520069.5985924
SRR17520069.5985925
SRR17520069.5985926
SRR17520069.5985928
SRR17520069.5985929
SRR17520069.5985930
SRR17520069.5985931
SRR17520069.5987775
SRR17520069.5987792
SRR17520069.5989974
SRR17520069.5989975
SRR17520069.5989976
SRR17520069.5989977
SRR17520069.5989978
SRR17520069.5989979
SRR17520069.5989980
SRR17520069.5989981
SRR17520069.5989982
SRR17520069.5989983
SRR17520069.5989984
SRR17520069.5989985
SRR17520069.5989986
SRR17520069.5989987
SRR17520069.5989988
SRR17520069.5989989
SRR17520069.5989990
SRR17520069.5989991
SRR17520069.5989992
SRR17520069.5989993
SRR17520069.5989994
SRR17520069.5989995
SRR17520069.5989996
SRR17520069.5989997
SRR17520069.5989998
SRR17520069.5989999
SRR17520069.5990000
SRR17520069.5990001
SRR17520069.5990002
SRR17520069.5990003
SRR17520069.5990004
SRR17520069.5990005
SRR17520069.5990006
SRR17520069.5990007

SRR17520069.5990008
SRR17520069.5990009
SRR17520069.5990010
SRR17520069.5990011
SRR17520069.5990012
SRR17520069.5990013
SRR17520069.5990014
SRR17520069.5990015
SRR17520069.5990016
SRR17520069.5990017
SRR17520069.5990018
SRR17520069.5990019
SRR17520069.5990020
SRR17520069.5990021
SRR17520069.5990022
SRR17520069.5990023
SRR17520069.5990024
SRR17520069.5990025
SRR17520069.5990026
SRR17520069.5990027
SRR17520069.5990028
SRR17520069.5990029
SRR17520069.5990030
SRR17520069.5990031
SRR17520069.5990032
SRR17520069.5990033
SRR17520069.5990034
SRR17520069.5990035
SRR17520069.5990036
SRR17520069.5990037
SRR17520069.5990038
SRR17520069.5990039
SRR17520069.5990040
SRR17520069.5990041
SRR17520069.5990042
SRR17520069.5990043
SRR17520069.5990044
SRR17520069.5990045
SRR17520069.5990046
SRR17520069.5990047
SRR17520069.5990048
SRR17520069.5990049
SRR17520069.5990050
SRR17520069.5990051
SRR17520069.5990052
SRR17520069.5990053
SRR17520069.5990054

SRR17520069.5990055
SRR17520069.5990056
SRR17520069.5990057
SRR17520069.5990058
SRR17520069.5990059
SRR17520069.5990060
SRR17520069.5990061
SRR17520069.5990062
SRR17520069.5990063
SRR17520069.5990064
SRR17520069.5990065
SRR17520069.5990067
SRR17520069.5990068
SRR17520069.5990069
SRR17520069.5990070
SRR17520069.5990071
SRR17520069.5990072
SRR17520069.5990073
SRR17520069.5990074
SRR17520069.5990075
SRR17520069.5990076
SRR17520069.5990077
SRR17520069.5990078
SRR17520069.5990079
SRR17520069.5990080
SRR17520069.5990081
SRR17520069.5990083
SRR17520069.5990084
SRR17520069.5990085
SRR17520069.5990086
SRR17520069.5990087
SRR17520069.5990088
SRR17520069.5990089
SRR17520069.5990090
SRR17520069.5990091
SRR17520069.5990092
SRR17520069.5990093
SRR17520069.5990094
SRR17520069.5990095
SRR17520069.5990096
SRR17520069.5990097
SRR17520069.5990098
SRR17520069.5990099
SRR17520069.5990100
SRR17520069.5990101
SRR17520069.5990102
SRR17520069.5990103

SRR17520069.5990104
SRR17520069.5990105
SRR17520069.5990106
SRR17520069.5990107
SRR17520069.5990108
SRR17520069.5990109
SRR17520069.5990110
SRR17520069.5990111
SRR17520069.5990112
SRR17520069.5990113
SRR17520069.5990114
SRR17520069.5990115
SRR17520069.5990116
SRR17520069.5990117
SRR17520069.5990118
SRR17520069.5990119
SRR17520069.5990120
SRR17520069.5990121
SRR17520069.5990122
SRR17520069.5992600
SRR17520069.5992601
SRR17520069.5992602
SRR17520069.5992603
SRR17520069.5992604
SRR17520069.5992605
SRR17520069.5992606
SRR17520069.5992607
SRR17520069.5992608
SRR17520069.5992609
SRR17520069.5992610
SRR17520069.5992611
SRR17520069.5992612
SRR17520069.5992613
SRR17520069.5992614
SRR17520069.5992615
SRR17520069.5992616
SRR17520069.5992617
SRR17520069.5992618
SRR17520069.5992619
SRR17520069.5992620
SRR17520069.5992621
SRR17520069.5992622
SRR17520069.5992623
SRR17520069.5992624
SRR17520069.5992625
SRR17520069.5992626
SRR17520069.5992627

SRR17520069.5992628
SRR17520069.5992629
SRR17520069.5992630
SRR17520069.5992631
SRR17520069.5992632
SRR17520069.5992633
SRR17520069.5992634
SRR17520069.5992635
SRR17520069.5992636
SRR17520069.5992637
SRR17520069.5992638
SRR17520069.5992639
SRR17520069.5992640
SRR17520069.5992641
SRR17520069.5992642
SRR17520069.5992643
SRR17520069.5992644
SRR17520069.5992645
SRR17520069.5992646
SRR17520069.5992649
SRR17520069.5992651
SRR17520069.5992652
SRR17520069.5992653
SRR17520069.5992654
SRR17520069.5992655
SRR17520069.5992656
SRR17520069.5992657
SRR17520069.5992658
SRR17520069.5992659
SRR17520069.5992667
SRR17520069.5993251
SRR17520069.5993252
SRR17520069.5993253
SRR17520069.5993254
SRR17520069.5993255
SRR17520069.5993256
SRR17520069.5993257
SRR17520069.5993258
SRR17520069.5993259
SRR17520069.5993260
SRR17520069.5993261
SRR17520069.5993262
SRR17520069.5993263
SRR17520069.5993264
SRR17520069.5993265
SRR17520069.5993266
SRR17520069.5993267

SRR17520069.5993268
SRR17520069.5993269
SRR17520069.5993270
SRR17520069.5993271
SRR17520069.5993272
SRR17520069.5993273
SRR17520069.5993274
SRR17520069.5993275
SRR17520069.5993276
SRR17520069.5993277
SRR17520069.5993278
SRR17520069.5993279
SRR17520069.5993280
SRR17520069.5993281
SRR17520069.5993282
SRR17520069.5993283
SRR17520069.5993284
SRR17520069.5993285
SRR17520069.5993286
SRR17520069.5993287
SRR17520069.5993288
SRR17520069.5993289
SRR17520069.5993290
SRR17520069.5993291
SRR17520069.5993292
SRR17520069.5993293
SRR17520069.5993294
SRR17520069.5993295
SRR17520069.5993296
SRR17520069.5993297
SRR17520069.5993298
SRR17520069.5993299
SRR17520069.5993300
SRR17520069.5993301
SRR17520069.5993302
SRR17520069.5993303
SRR17520069.5993304
SRR17520069.5993305
SRR17520069.5993306
SRR17520069.5993307
SRR17520069.5993309
SRR17520069.5993310
SRR17520069.5993311
SRR17520069.5993312
SRR17520069.5993313
SRR17520069.5993314
SRR17520069.5993315

SRR17520069.5993316
SRR17520069.5993317
SRR17520069.5993318
SRR17520069.5993319
SRR17520069.5993320
SRR17520069.5993321
SRR17520069.5993322
SRR17520069.5993324
SRR17520069.5993325
SRR17520069.5993326
SRR17520069.5993327
SRR17520069.5993328
SRR17520069.5993329
SRR17520069.5993330
SRR17520069.5993331
SRR17520069.5993332
SRR17520069.5993333
SRR17520069.5993334
SRR17520069.5993335
SRR17520069.5993336
SRR17520069.5993337
SRR17520069.5993338
SRR17520069.5993339
SRR17520069.5993340
SRR17520069.5993341
SRR17520069.5993342
SRR17520069.5993343
SRR17520069.5993344
SRR17520069.5993345
SRR17520069.5993346
SRR17520069.5993347
SRR17520069.5993348
SRR17520069.5993349
SRR17520069.5993350
SRR17520069.5993351
SRR17520069.5993352
SRR17520069.5993353
SRR17520069.5993354
SRR17520069.5993355
SRR17520069.5993356
SRR17520069.5993357
SRR17520069.5993358
SRR17520069.5993359
SRR17520069.5993360
SRR17520069.5993361
SRR17520069.5993362
SRR17520069.5993363

SRR17520069.5993364
SRR17520069.5993365
SRR17520069.5993366
SRR17520069.5993367
SRR17520069.5993368
SRR17520069.5993369
SRR17520069.5993370
SRR17520069.5993371
SRR17520069.5993372
SRR17520069.5993373
SRR17520069.5993374
SRR17520069.5993375
SRR17520069.5993376
SRR17520069.5993377
SRR17520069.5993378
SRR17520069.5993379
SRR17520069.5993380
SRR17520069.5993381
SRR17520069.5993382
SRR17520069.5993383
SRR17520069.5993384
SRR17520069.5993385
SRR17520069.5993386
SRR17520069.5993387
SRR17520069.5993388
SRR17520069.5993389
SRR17520069.5993390
SRR17520069.5993391
SRR17520069.5993392
SRR17520069.5993393
SRR17520069.5993394
SRR17520069.5993395
SRR17520069.5993396
SRR17520069.5993397
SRR17520069.5993398
SRR17520069.5993399
SRR17520069.5993400
SRR17520069.5993401
SRR17520069.5993402
SRR17520069.5993403
SRR17520069.5993404
SRR17520069.5993405
SRR17520069.5993406
SRR17520069.5993407
SRR17520069.5993408
SRR17520069.5993409
SRR17520069.5993410

SRR17520069.6000439
SRR17520069.6000440
SRR17520069.6000441
SRR17520069.6000442
SRR17520069.6000443
SRR17520069.6000444
SRR17520069.6000445
SRR17520069.6000446
SRR17520069.6000447
SRR17520069.6000448
SRR17520069.6000449
SRR17520069.6000450
SRR17520069.6000451
SRR17520069.6000452
SRR17520069.6000453
SRR17520069.6000454
SRR17520069.6000455
SRR17520069.6000456
SRR17520069.6000457
SRR17520069.6000458
SRR17520069.6000459
SRR17520069.6000460
SRR17520069.6000461
SRR17520069.6000462
SRR17520069.6000463
SRR17520069.6000464
SRR17520069.6000465
SRR17520069.6000466
SRR17520069.6000467
SRR17520069.6000468
SRR17520069.6000469
SRR17520069.6000470
SRR17520069.6000471
SRR17520069.6000472
SRR17520069.6000473
SRR17520069.6000474
SRR17520069.6000475
SRR17520069.6000476
SRR17520069.6000477
SRR17520069.6000478
SRR17520069.6000479
SRR17520069.6000480
SRR17520069.6000481
SRR17520069.6000482
SRR17520069.6000483
SRR17520069.6000484
SRR17520069.6000485

SRR17520069.6000486
SRR17520069.6000487
SRR17520069.6000488
SRR17520069.6000489
SRR17520069.6000490
SRR17520069.6000491
SRR17520069.6000492
SRR17520069.6000493
SRR17520069.6000494
SRR17520069.6000495
SRR17520069.6000496
SRR17520069.6000497
SRR17520069.6000498
SRR17520069.6000499
SRR17520069.6000500
SRR17520069.6000501
SRR17520069.6000502
SRR17520069.6000503
SRR17520069.6000504
SRR17520069.6000505
SRR17520069.6000506
SRR17520069.6000507
SRR17520069.6000508
SRR17520069.6000509
SRR17520069.6000510
SRR17520069.6000511
SRR17520069.6000512
SRR17520069.6000513
SRR17520069.6000514
SRR17520069.6000515
SRR17520069.6000516
SRR17520069.6000517
SRR17520069.6000518
SRR17520069.6000519
SRR17520069.6000520
SRR17520069.6000521
SRR17520069.6000522
SRR17520069.6000523
SRR17520069.6000524
SRR17520069.6000525
SRR17520069.6000526
SRR17520069.6000527
SRR17520069.6000528
SRR17520069.6000529
SRR17520069.6000530
SRR17520069.6000531
SRR17520069.6000532

SRR17520069.6000533
SRR17520069.6000534
SRR17520069.6000535
SRR17520069.6004981
SRR17520069.6004982
SRR17520069.6004983
SRR17520069.6004984
SRR17520069.6004985
SRR17520069.6004986
SRR17520069.6004987
SRR17520069.6004988
SRR17520069.6004989
SRR17520069.6004990
SRR17520069.6004991
SRR17520069.6004992
SRR17520069.6013075
SRR17520069.6013076
SRR17520069.6013077
SRR17520069.6015521
SRR17520069.6015522
SRR17520069.6015523
SRR17520069.6015524
SRR17520069.6015525
SRR17520069.6015526
SRR17520069.6015527
SRR17520069.6015528
SRR17520069.6015529
SRR17520069.6015530
SRR17520069.6015531
SRR17520069.6015533
SRR17520069.6015534
SRR17520069.6015535
SRR17520069.6015536
SRR17520069.6015537
SRR17520069.6015538
SRR17520069.6015539
SRR17520069.6015541
SRR17520069.6015543
SRR17520069.6015544
SRR17520069.6024142
SRR17520069.6024143
SRR17520069.6024144
SRR17520069.6024145
SRR17520069.6024146
SRR17520069.6024147
SRR17520069.6024148
SRR17520069.6024149

SRR17520069.6024150
SRR17520069.6024151
SRR17520069.6024152
SRR17520069.6024153
SRR17520069.6024154
SRR17520069.6024155
SRR17520069.6024156
SRR17520069.6024157
SRR17520069.6024158
SRR17520069.6024159
SRR17520069.6024160
SRR17520069.6024161
SRR17520069.6024162
SRR17520069.6024163
SRR17520069.6024164
SRR17520069.6024165
SRR17520069.6024166
SRR17520069.6024167
SRR17520069.6024168
SRR17520069.6024169
SRR17520069.6024170
SRR17520069.6024171
SRR17520069.6024172
SRR17520069.6024173
SRR17520069.6024174
SRR17520069.6024175
SRR17520069.6024176
SRR17520069.6024177
SRR17520069.6024178
SRR17520069.6024179
SRR17520069.6024180
SRR17520069.6024181
SRR17520069.6024182
SRR17520069.6024183
SRR17520069.6024184
SRR17520069.6024185
SRR17520069.6024186
SRR17520069.6024187
SRR17520069.6024189
SRR17520069.6024190
SRR17520069.6024191
SRR17520069.6024192
SRR17520069.6024193
SRR17520069.6024194
SRR17520069.6024195
SRR17520069.6024196
SRR17520069.6024197

SRR17520069.6024198
SRR17520069.6024199
SRR17520069.6024200
SRR17520069.6024201
SRR17520069.6024202
SRR17520069.6024203
SRR17520069.6024204
SRR17520069.6024205
SRR17520069.6024206
SRR17520069.6024207
SRR17520069.6024208
SRR17520069.6024209
SRR17520069.6024210
SRR17520069.6024211
SRR17520069.6024212
SRR17520069.6024213
SRR17520069.6024214
SRR17520069.6024215
SRR17520069.6024216
SRR17520069.6024217
SRR17520069.6024218
SRR17520069.6024219
SRR17520069.6024220
SRR17520069.6024221
SRR17520069.6024222
SRR17520069.6024223
SRR17520069.6024224
SRR17520069.6024225
SRR17520069.6024226
SRR17520069.6024227
SRR17520069.6024228
SRR17520069.6024229
SRR17520069.6024230
SRR17520069.6024231
SRR17520069.6024232
SRR17520069.6024233
SRR17520069.6024234
SRR17520069.6024235
SRR17520069.6024236
SRR17520069.6024237
SRR17520069.6024238
SRR17520069.6024239
SRR17520069.6024240
SRR17520069.6024241
SRR17520069.6024242
SRR17520069.6024243
SRR17520069.6024245

SRR17520069.6024246
SRR17520069.6024247
SRR17520069.6024248
SRR17520069.6024249
SRR17520069.6024250
SRR17520069.6024251
SRR17520069.6024252
SRR17520069.6024253
SRR17520069.6024254
SRR17520069.6024255
SRR17520069.6024256
SRR17520069.6024257
SRR17520069.6024258
SRR17520069.6024259
SRR17520069.6024260
SRR17520069.6024261
SRR17520069.6024262
SRR17520069.6024263
SRR17520069.6024264
SRR17520069.6024265
SRR17520069.6024266
SRR17520069.6024267
SRR17520069.6024268
SRR17520069.6024269
SRR17520069.6024270
SRR17520069.6024271
SRR17520069.6024272
SRR17520069.6024273
SRR17520069.6024274
SRR17520069.6024275
SRR17520069.6024276
SRR17520069.6024590
SRR17520069.6024591
SRR17520069.6024593
SRR17520069.6024594
SRR17520069.6024595
SRR17520069.6024596
SRR17520069.6024597
SRR17520069.6024598
SRR17520069.6024600
SRR17520069.6024601
SRR17520069.6024602
SRR17520069.6045193
SRR17520069.6045195
SRR17520069.6045198
SRR17520069.6045200
SRR17520069.6045201

SRR17520069.6045202
SRR17520069.6045203
SRR17520069.6045204
SRR17520069.6045205
SRR17520069.6045206
SRR17520069.6045207
SRR17520069.6045208
SRR17520069.6045209
SRR17520069.6045210
SRR17520069.6045211
SRR17520069.6045212
SRR17520069.6045213
SRR17520069.6045214
SRR17520069.6045215
SRR17520069.6045216
SRR17520069.6045217
SRR17520069.6045218
SRR17520069.6045219
SRR17520069.6045220
SRR17520069.6045221
SRR17520069.6045222
SRR17520069.6045223
SRR17520069.6045224
SRR17520069.6045225
SRR17520069.6045226
SRR17520069.6045227
SRR17520069.6045228
SRR17520069.6045229
SRR17520069.6045231
SRR17520069.6045232
SRR17520069.6045233
SRR17520069.6045234
SRR17520069.6045235
SRR17520069.6045236
SRR17520069.6045237
SRR17520069.6045238
SRR17520069.6045239
SRR17520069.6045240
SRR17520069.6045241
SRR17520069.6045242
SRR17520069.6045243
SRR17520069.6045244
SRR17520069.6045245
SRR17520069.6045246
SRR17520069.6045247
SRR17520069.6045248
SRR17520069.6045249

SRR17520069.6045250
SRR17520069.6045251
SRR17520069.6045252
SRR17520069.6045253
SRR17520069.6045254
SRR17520069.6045255
SRR17520069.6045256
SRR17520069.6045257
SRR17520069.6045258
SRR17520069.6045259
SRR17520069.6045260
SRR17520069.6045261
SRR17520069.6045262
SRR17520069.6049139
SRR17520069.6049154
SRR17520069.6052444
SRR17520069.6052445
SRR17520069.6052446
SRR17520069.6052448
SRR17520069.6052450
SRR17520069.6052451
SRR17520069.6052452
SRR17520069.6052453
SRR17520069.6052454
SRR17520069.6052455
SRR17520069.6052456
SRR17520069.6052457
SRR17520069.6052458
SRR17520069.6052459
SRR17520069.6052460
SRR17520069.6052461
SRR17520069.6052462
SRR17520069.6052463
SRR17520069.6052464
SRR17520069.6052465
SRR17520069.6052466
SRR17520069.6052467
SRR17520069.6052468
SRR17520069.6052469
SRR17520069.6052470
SRR17520069.6052471
SRR17520069.6052472
SRR17520069.6052473
SRR17520069.6052474
SRR17520069.6052475
SRR17520069.6052476
SRR17520069.6052477

SRR17520069.6052478
SRR17520069.6052479
SRR17520069.6052480
SRR17520069.6052481
SRR17520069.6052482
SRR17520069.6052483
SRR17520069.6052484
SRR17520069.6052485
SRR17520069.6052486
SRR17520069.6052487
SRR17520069.6052488
SRR17520069.6052489
SRR17520069.6052490
SRR17520069.6052491
SRR17520069.6052492
SRR17520069.6052493
SRR17520069.6052494
SRR17520069.6052495
SRR17520069.6052496
SRR17520069.6052497
SRR17520069.6052498
SRR17520069.6052499
SRR17520069.6052500
SRR17520069.6052501
SRR17520069.6052502
SRR17520069.6052503
SRR17520069.6052504
SRR17520069.6052505
SRR17520069.6052506
SRR17520069.6052507
SRR17520069.6052508
SRR17520069.6052509
SRR17520069.6052510
SRR17520069.6052511
SRR17520069.6052512
SRR17520069.6052513
SRR17520069.6052514
SRR17520069.6052515
SRR17520069.6052516
SRR17520069.6052517
SRR17520069.6052518
SRR17520069.6052519
SRR17520069.6052520
SRR17520069.6052521
SRR17520069.6052522
SRR17520069.6052523
SRR17520069.6052524

SRR17520069.6052525
SRR17520069.6052526
SRR17520069.6052527
SRR17520069.6052528
SRR17520069.6052530
SRR17520069.6052531
SRR17520069.6052532
SRR17520069.6052533
SRR17520069.6052534
SRR17520069.6052535
SRR17520069.6052536
SRR17520069.6052537
SRR17520069.6052538
SRR17520069.6052539
SRR17520069.6052540
SRR17520069.6052541
SRR17520069.6052542
SRR17520069.6052543
SRR17520069.6052544
SRR17520069.6052545
SRR17520069.6052546
SRR17520069.6052547
SRR17520069.6052548
SRR17520069.6052549
SRR17520069.6052550
SRR17520069.6052551
SRR17520069.6052552
SRR17520069.6052553
SRR17520069.6052554
SRR17520069.6052555
SRR17520069.6052556
SRR17520069.6052557
SRR17520069.6052558
SRR17520069.6052559
SRR17520069.6052560
SRR17520069.6052561
SRR17520069.6052562
SRR17520069.6052563
SRR17520069.6052564
SRR17520069.6052565
SRR17520069.6052566
SRR17520069.6052567
SRR17520069.6052568
SRR17520069.6052569
SRR17520069.6052571
SRR17520069.6052572
SRR17520069.6052573

SRR17520069.6052574
SRR17520069.6052575
SRR17520069.6052576
SRR17520069.6052577
SRR17520069.6052578
SRR17520069.6052579
SRR17520069.6052580
SRR17520069.6052581
SRR17520069.6052582
SRR17520069.6052583
SRR17520069.6052584
SRR17520069.6052585
SRR17520069.6052586
SRR17520069.6052587
SRR17520069.6052588
SRR17520069.6052589
SRR17520069.6052590
SRR17520069.6052591
SRR17520069.6052592
SRR17520069.6052593
SRR17520069.6052594
SRR17520069.6052595
SRR17520069.6069620
SRR17520069.6069621
SRR17520069.6069622
SRR17520069.6069623
SRR17520069.6069624
SRR17520069.6069625
SRR17520069.6069626
SRR17520069.6069627
SRR17520069.6069629
SRR17520069.6069630
SRR17520069.6069631
SRR17520069.6069632
SRR17520069.6069633
SRR17520069.6069634
SRR17520069.6069635
SRR17520069.6069636
SRR17520069.6069637
SRR17520069.6069638
SRR17520069.6069639
SRR17520069.6069640
SRR17520069.6069641
SRR17520069.6069642
SRR17520069.6069643
SRR17520069.6069644
SRR17520069.6069645

SRR17520069.6069646
SRR17520069.6069647
SRR17520069.6069648
SRR17520069.6069649
SRR17520069.6069650
SRR17520069.6069651
SRR17520069.6069652
SRR17520069.6069653
SRR17520069.6069654
SRR17520069.6069655
SRR17520069.6069656
SRR17520069.6069657
SRR17520069.6069658
SRR17520069.6069659
SRR17520069.6069660
SRR17520069.6069661
SRR17520069.6069662
SRR17520069.6069663
SRR17520069.6069664
SRR17520069.6069665
SRR17520069.6069666
SRR17520069.6069667
SRR17520069.6069668
SRR17520069.6069669
SRR17520069.6069670
SRR17520069.6069671
SRR17520069.6069672
SRR17520069.6069673
SRR17520069.6069674
SRR17520069.6069675
SRR17520069.6069676
SRR17520069.6069677
SRR17520069.6069678
SRR17520069.6069679
SRR17520069.6069680
SRR17520069.6069681
SRR17520069.6069682
SRR17520069.6069683
SRR17520069.6069684
SRR17520069.6069685
SRR17520069.6069686
SRR17520069.6069687
SRR17520069.6069688
SRR17520069.6069689
SRR17520069.6069690
SRR17520069.6069691
SRR17520069.6069692

SRR17520069.6069693
SRR17520069.6069694
SRR17520069.6069695
SRR17520069.6069696
SRR17520069.6069697
SRR17520069.6069698
SRR17520069.6069699
SRR17520069.6069700
SRR17520069.6069701
SRR17520069.6069702
SRR17520069.6069703
SRR17520069.6069704
SRR17520069.6069705
SRR17520069.6069706
SRR17520069.6069707
SRR17520069.6069708
SRR17520069.6069709
SRR17520069.6069710
SRR17520069.6069711
SRR17520069.6069712
SRR17520069.6069713
SRR17520069.6069714
SRR17520069.6069715
SRR17520069.6069716
SRR17520069.6069717
SRR17520069.6069718
SRR17520069.6069719
SRR17520069.6069720
SRR17520069.6069721
SRR17520069.6069722
SRR17520069.6069723
SRR17520069.6069724
SRR17520069.6069725
SRR17520069.6069726
SRR17520069.6069727
SRR17520069.6069728
SRR17520069.6069729
SRR17520069.6069730
SRR17520069.6069731
SRR17520069.6069732
SRR17520069.6069733
SRR17520069.6069734
SRR17520069.6069735
SRR17520069.6069736
SRR17520069.6069737
SRR17520069.6069738
SRR17520069.6069739

SRR17520069.6069740
SRR17520069.6069741
SRR17520069.6069742
SRR17520069.6069743
SRR17520069.6069744
SRR17520069.6069745
SRR17520069.6069746
SRR17520069.6069747
SRR17520069.6069748
SRR17520069.6069749
SRR17520069.6069750
SRR17520069.6069751
SRR17520069.6069752
SRR17520069.6069753
SRR17520069.6069754
SRR17520069.6069755
SRR17520069.6069756
SRR17520069.6069757
SRR17520069.6069758
SRR17520069.6069759
SRR17520069.6069760
SRR17520069.6069761
SRR17520069.6069763
SRR17520069.6069764
SRR17520069.6069765
SRR17520069.6069766
SRR17520069.6069767
SRR17520069.6069768
SRR17520069.6069769
SRR17520069.6069770
SRR17520069.6069771
SRR17520069.6069772
SRR17520069.6069773
SRR17520069.6069774
SRR17520069.6069775
SRR17520069.6069776
SRR17520069.6069777
SRR17520069.6069778
SRR17520069.6069779
SRR17520069.6069780
SRR17520069.6069781
SRR17520069.6069782
SRR17520069.6069783
SRR17520069.6069784
SRR17520069.6069785
SRR17520069.6069786
SRR17520069.6069787

SRR17520069.6069788
SRR17520069.6069789
SRR17520069.6069790
SRR17520069.6074511
SRR17520069.6074512
SRR17520069.6074513
SRR17520069.6074514
SRR17520069.6074515
SRR17520069.6074516
SRR17520069.6074517
SRR17520069.6074518
SRR17520069.6074519
SRR17520069.6074520
SRR17520069.6074521
SRR17520069.6074522
SRR17520069.6074523
SRR17520069.6074524
SRR17520069.6074525
SRR17520069.6074526
SRR17520069.6074527
SRR17520069.6074528
SRR17520069.6074529
SRR17520069.6074530
SRR17520069.6074531
SRR17520069.6074532
SRR17520069.6074533
SRR17520069.6074534
SRR17520069.6074535
SRR17520069.6074536
SRR17520069.6074537
SRR17520069.6074538
SRR17520069.6074539
SRR17520069.6074540
SRR17520069.6074541
SRR17520069.6074542
SRR17520069.6074543
SRR17520069.6074544
SRR17520069.6074545
SRR17520069.6074546
SRR17520069.6074547
SRR17520069.6074548
SRR17520069.6074549
SRR17520069.6074550
SRR17520069.6074551
SRR17520069.6074552
SRR17520069.6074553
SRR17520069.6074554

SRR17520069.6074555
SRR17520069.6074556
SRR17520069.6074557
SRR17520069.6074558
SRR17520069.6074559
SRR17520069.6074560
SRR17520069.6074561
SRR17520069.6074562
SRR17520069.6074563
SRR17520069.6074564
SRR17520069.6074565
SRR17520069.6074566
SRR17520069.6074567
SRR17520069.6074568
SRR17520069.6074569
SRR17520069.6074570
SRR17520069.6074571
SRR17520069.6074572
SRR17520069.6074573
SRR17520069.6074574
SRR17520069.6074575
SRR17520069.6074576
SRR17520069.6074577
SRR17520069.6074578
SRR17520069.6074579
SRR17520069.6074580
SRR17520069.6074581
SRR17520069.6074582
SRR17520069.6074583
SRR17520069.6074584
SRR17520069.6074585
SRR17520069.6074586
SRR17520069.6074587
SRR17520069.6074588
SRR17520069.6074589
SRR17520069.6074591
SRR17520069.6074592
SRR17520069.6074593
SRR17520069.6076780
SRR17520069.6076781
SRR17520069.6076782
SRR17520069.6076783
SRR17520069.6076784
SRR17520069.6076785
SRR17520069.6076786
SRR17520069.6076787
SRR17520069.6076788

SRR17520069.6076789
SRR17520069.6076790
SRR17520069.6076791
SRR17520069.6076792
SRR17520069.6076793
SRR17520069.6076794
SRR17520069.6076795
SRR17520069.6076796
SRR17520069.6076797
SRR17520069.6076798
SRR17520069.6076812
SRR17520069.6097159
SRR17520069.6097163
SRR17520069.6097164
SRR17520069.6097167
SRR17520069.6100017
SRR17520069.6100037
SRR17520069.6103158
SRR17520069.6103160
SRR17520069.6103161
SRR17520069.6110700
SRR17520069.6110701
SRR17520069.6110702
SRR17520069.6110703
SRR17520069.6110704
SRR17520069.6110705
SRR17520069.6110706
SRR17520069.6110707
SRR17520069.6110708
SRR17520069.6110709
SRR17520069.6110710
SRR17520069.6110711
SRR17520069.6110712
SRR17520069.6110713
SRR17520069.6110714
SRR17520069.6110715
SRR17520069.6110716
SRR17520069.6110717
SRR17520069.6110718
SRR17520069.6110719
SRR17520069.6110720
SRR17520069.6110721
SRR17520069.6110722
SRR17520069.6110723
SRR17520069.6112586
SRR17520069.6112588
SRR17520069.6112589

SRR17520069.6112590
SRR17520069.6112591
SRR17520069.6112592
SRR17520069.6112594
SRR17520069.6112598
SRR17520069.6112599
SRR17520069.6112600
SRR17520069.6112601
SRR17520069.6112602
SRR17520069.6112604
SRR17520069.6112606
SRR17520069.6112608
SRR17520069.6112609
SRR17520069.6112610
SRR17520069.6112612
SRR17520069.6112613
SRR17520069.6112614
SRR17520069.6112615
SRR17520069.6112616
SRR17520069.6112617
SRR17520069.6112618
SRR17520069.6112619
SRR17520069.6112620
SRR17520069.6112621
SRR17520069.6112622
SRR17520069.6112623
SRR17520069.6112625
SRR17520069.6112626
SRR17520069.6112627
SRR17520069.6112628
SRR17520069.6112629
SRR17520069.6112630
SRR17520069.6112631
SRR17520069.6112634
SRR17520069.6112636
SRR17520069.6112637
SRR17520069.6112639
SRR17520069.6112641
SRR17520069.6112642
SRR17520069.6112643
SRR17520069.6112644
SRR17520069.6112645
SRR17520069.6112646
SRR17520069.6112647
SRR17520069.6112648
SRR17520069.6112649
SRR17520069.6112650

SRR17520069.6112651
SRR17520069.6112652
SRR17520069.6112654
SRR17520069.6112655
SRR17520069.6112656
SRR17520069.6112657
SRR17520069.6112658
SRR17520069.6112659
SRR17520069.6112660
SRR17520069.6112661
SRR17520069.6112662
SRR17520069.6112663
SRR17520069.6112664
SRR17520069.6112665
SRR17520069.6112666
SRR17520069.6112667
SRR17520069.6112668
SRR17520069.6112669
SRR17520069.6112670
SRR17520069.6112672
SRR17520069.6112673
SRR17520069.6112674
SRR17520069.6112675
SRR17520069.6112676
SRR17520069.6112677
SRR17520069.6112678
SRR17520069.6112679
SRR17520069.6112680
SRR17520069.6112682
SRR17520069.6112683
SRR17520069.6112684
SRR17520069.6112686
SRR17520069.6112687
SRR17520069.6112688
SRR17520069.6112689
SRR17520069.6112690
SRR17520069.6112691
SRR17520069.6112692
SRR17520069.6112694
SRR17520069.6112695
SRR17520069.6112696
SRR17520069.6112697
SRR17520069.6112699
SRR17520069.6112700
SRR17520069.6112701
SRR17520069.6112702
SRR17520069.6112703

SRR17520069.6112704
SRR17520069.6112705
SRR17520069.6112707
SRR17520069.6112708
SRR17520069.6112709
SRR17520069.6112710
SRR17520069.6112711
SRR17520069.6112712
SRR17520069.6112713
SRR17520069.6112714
SRR17520069.6112715
SRR17520069.6136241
SRR17520069.6136242
SRR17520069.6136243
SRR17520069.6136244
SRR17520069.6136245
SRR17520069.6136246
SRR17520069.6136247
SRR17520069.6136248
SRR17520069.6136249
SRR17520069.6136250
SRR17520069.6136251
SRR17520069.6136252
SRR17520069.6136253
SRR17520069.6136254
SRR17520069.6136255
SRR17520069.6136256
SRR17520069.6136257
SRR17520069.6136258
SRR17520069.6136259
SRR17520069.6136260
SRR17520069.6136261
SRR17520069.6136262
SRR17520069.6136263
SRR17520069.6136264
SRR17520069.6136265
SRR17520069.6136266
SRR17520069.6136267
SRR17520069.6136268
SRR17520069.6136269
SRR17520069.6136270
SRR17520069.6136272
SRR17520069.6136273
SRR17520069.6136274
SRR17520069.6136276
SRR17520069.6136277
SRR17520069.6136278

SRR17520069.6136279
SRR17520069.6136280
SRR17520069.6136281
SRR17520069.6136282
SRR17520069.6136284
SRR17520069.6136285
SRR17520069.6136286
SRR17520069.6136287
SRR17520069.6136288
SRR17520069.6136289
SRR17520069.6136290
SRR17520069.6136291
SRR17520069.6136292
SRR17520069.6136293
SRR17520069.6136294
SRR17520069.6136295
SRR17520069.6136296
SRR17520069.6136298
SRR17520069.6136299
SRR17520069.6136300
SRR17520069.6136301
SRR17520069.6136302
SRR17520069.6136303
SRR17520069.6136304
SRR17520069.6136305
SRR17520069.6136306
SRR17520069.6136307
SRR17520069.6136308
SRR17520069.6136309
SRR17520069.6136310
SRR17520069.6136311
SRR17520069.6136312
SRR17520069.6136313
SRR17520069.6136314
SRR17520069.6136315
SRR17520069.6136316
SRR17520069.6136317
SRR17520069.6142979
SRR17520069.6142980
SRR17520069.6142981
SRR17520069.6142982
SRR17520069.6142983
SRR17520069.6142984
SRR17520069.6142985
SRR17520069.6142986
SRR17520069.6142987
SRR17520069.6142988

SRR17520069.6142989
SRR17520069.6142990
SRR17520069.6142991
SRR17520069.6142992
SRR17520069.6142993
SRR17520069.6142994
SRR17520069.6147716
SRR17520069.6147738
SRR17520069.6170482
SRR17520069.6170483
SRR17520069.6170484
SRR17520069.6170485
SRR17520069.6170486
SRR17520069.6170487
SRR17520069.6170488
SRR17520069.6170489
SRR17520069.6170490
SRR17520069.6170491
SRR17520069.6170492
SRR17520069.6170493
SRR17520069.6170494
SRR17520069.6170495
SRR17520069.6170496
SRR17520069.6170497
SRR17520069.6170498
SRR17520069.6170499
SRR17520069.6170500
SRR17520069.6170501
SRR17520069.6170502
SRR17520069.6170503
SRR17520069.6170504
SRR17520069.6170505
SRR17520069.6170506
SRR17520069.6170507
SRR17520069.6170508
SRR17520069.6170509
SRR17520069.6170510
SRR17520069.6170511
SRR17520069.6170512
SRR17520069.6170513
SRR17520069.6170514
SRR17520069.6170515
SRR17520069.6170516
SRR17520069.6170518
SRR17520069.6170519
SRR17520069.6170520
SRR17520069.6170521

SRR17520069.6170522
SRR17520069.6170524
SRR17520069.6170525
SRR17520069.6170526
SRR17520069.6170527
SRR17520069.6170528
SRR17520069.6170529
SRR17520069.6170530
SRR17520069.6170531
SRR17520069.6170532
SRR17520069.6170533
SRR17520069.6170534
SRR17520069.6170535
SRR17520069.6170536
SRR17520069.6174986
SRR17520069.6174987
SRR17520069.6174988
SRR17520069.6174989
SRR17520069.6174990
SRR17520069.6174991
SRR17520069.6174992
SRR17520069.6174993
SRR17520069.6174994
SRR17520069.6174995
SRR17520069.6174996
SRR17520069.6174997
SRR17520069.6174998
SRR17520069.6174999
SRR17520069.6175000
SRR17520069.6175001
SRR17520069.6175002
SRR17520069.6175003
SRR17520069.6175004
SRR17520069.6209461
SRR17520069.6221817
SRR17520069.6221818
SRR17520069.6221819
SRR17520069.6221820
SRR17520069.6221821
SRR17520069.6221822
SRR17520069.6221823
SRR17520069.6221824
SRR17520069.6221825
SRR17520069.6221826
SRR17520069.6221827
SRR17520069.6221828
SRR17520069.6221830

SRR17520069.6221831
SRR17520069.6221832
SRR17520069.6221833
SRR17520069.6221834
SRR17520069.6221835
SRR17520069.6221836
SRR17520069.6221837
SRR17520069.6221838
SRR17520069.6221839
SRR17520069.6221840
SRR17520069.6221841
SRR17520069.6221842
SRR17520069.6221843
SRR17520069.6221844
SRR17520069.6221845
SRR17520069.6221846
SRR17520069.6221847
SRR17520069.6221848
SRR17520069.6221849
SRR17520069.6221850
SRR17520069.6221851
SRR17520069.6221852
SRR17520069.6221853
SRR17520069.6221854
SRR17520069.6221856
SRR17520069.6221857
SRR17520069.6221858
SRR17520069.6221859
SRR17520069.6221860
SRR17520069.6221861
SRR17520069.6222535
SRR17520069.6222546
SRR17520069.6222606
SRR17520069.6227803
SRR17520069.6227804
SRR17520069.6227805
SRR17520069.6227806
SRR17520069.6233338
SRR17520069.6233339
SRR17520069.6233340
SRR17520069.6233341
SRR17520069.6233342
SRR17520069.6233343
SRR17520069.6233344
SRR17520069.6233345
SRR17520069.6233346
SRR17520069.6233347

SRR17520069.6233348
SRR17520069.6233349
SRR17520069.6233350
SRR17520069.6233351
SRR17520069.6233352
SRR17520069.6233353
SRR17520069.6233355
SRR17520069.6233357
SRR17520069.6233358
SRR17520069.6233359
SRR17520069.6246546
SRR17520069.6246548
SRR17520069.6246549
SRR17520069.6246550
SRR17520069.6246551
SRR17520069.6246552
SRR17520069.6246554
SRR17520069.6246555
SRR17520069.6246556
SRR17520069.6246557
SRR17520069.6246558
SRR17520069.6246560
SRR17520069.6246561
SRR17520069.6246562
SRR17520069.6246563
SRR17520069.6246564
SRR17520069.6246565
SRR17520069.6246566
SRR17520069.6246567
SRR17520069.6246569
SRR17520069.6246570
SRR17520069.6246571
SRR17520069.6246572
SRR17520069.6246573
SRR17520069.6246574
SRR17520069.6246575
SRR17520069.6246576
SRR17520069.6246577
SRR17520069.6246578
SRR17520069.6246579
SRR17520069.6246580
SRR17520069.6246581
SRR17520069.6246582
SRR17520069.6246583
SRR17520069.6246584
SRR17520069.6246585
SRR17520069.6246587

SRR17520069.6246588
SRR17520069.6246589
SRR17520069.6246590
SRR17520069.6246591
SRR17520069.6246592
SRR17520069.6246593
SRR17520069.6246594
SRR17520069.6246595
SRR17520069.6246596
SRR17520069.6273291
SRR17520069.6273292
SRR17520069.6273293
SRR17520069.6273294
SRR17520069.6273295
SRR17520069.6273296
SRR17520069.6273297
SRR17520069.6273298
SRR17520069.6273299
SRR17520069.6273300
SRR17520069.6273301
SRR17520069.6273302
SRR17520069.6273303
SRR17520069.6273304
SRR17520069.6273305
SRR17520069.6273306
SRR17520069.6273307
SRR17520069.6273308
SRR17520069.6273309
SRR17520069.6273310
SRR17520069.6273311
SRR17520069.6273312
SRR17520069.6273313
SRR17520069.6273314
SRR17520069.6273315
SRR17520069.6273316
SRR17520069.6273317
SRR17520069.6273319
SRR17520069.6273320
SRR17520069.6273321
SRR17520069.6273322
SRR17520069.6273323
SRR17520069.6273327
SRR17520069.6273330
SRR17520069.6273332
SRR17520069.6273334
SRR17520069.6273340
SRR17520069.6273342

SRR17520069.6273347
SRR17520069.6273348
SRR17520069.6273353
SRR17520069.6273354
SRR17520069.6273355
SRR17520069.6280894
SRR17520069.6280895
SRR17520069.6280896
SRR17520069.6280897
SRR17520069.6280899
SRR17520069.6280901
SRR17520069.6280902
SRR17520069.6280903
SRR17520069.6280904
SRR17520069.6280906
SRR17520069.6280907
SRR17520069.6280908
SRR17520069.6280909
SRR17520069.6280910
SRR17520069.6280912
SRR17520069.6280913
SRR17520069.6280914
SRR17520069.6280915
SRR17520069.6280917
SRR17520069.6280918
SRR17520069.6280919
SRR17520069.6280920
SRR17520069.6280921
SRR17520069.6280922
SRR17520069.6280924
SRR17520069.6280925
SRR17520069.6280926
SRR17520069.6280927
SRR17520069.6280928
SRR17520069.6280929
SRR17520069.6280930
SRR17520069.6280931
SRR17520069.6280932
SRR17520069.6280933
SRR17520069.6280934
SRR17520069.6280936
SRR17520069.6280937
SRR17520069.6280938
SRR17520069.6280939
SRR17520069.6280940
SRR17520069.6280941
SRR17520069.6280942

SRR17520069.6280944
SRR17520069.6280945
SRR17520069.6280946
SRR17520069.6280947
SRR17520069.6280948
SRR17520069.6280949
SRR17520069.6280950
SRR17520069.6280951
SRR17520069.6280952
SRR17520069.6280953
SRR17520069.6280954
SRR17520069.6280955
SRR17520069.6280956
SRR17520069.6280957
SRR17520069.6280958
SRR17520069.6280959
SRR17520069.6280960
SRR17520069.6286898
SRR17520069.6286902
SRR17520069.6294099
SRR17520069.6294100
SRR17520069.6294101
SRR17520069.6294102
SRR17520069.6294104
SRR17520069.6294105
SRR17520069.6294106
SRR17520069.6310732
SRR17520069.6310733
SRR17520069.6310735
SRR17520069.6310736
SRR17520069.6310737
SRR17520069.6310738
SRR17520069.6310739
SRR17520069.6310740
SRR17520069.6310742
SRR17520069.6310743
SRR17520069.6310746
SRR17520069.6310747
SRR17520069.6310748
SRR17520069.6310749
SRR17520069.6310751
SRR17520069.6310752
SRR17520069.6310753
SRR17520069.6310754
SRR17520069.6310755
SRR17520069.6310756
SRR17520069.6310757

SRR17520069.6310759
SRR17520069.6310760
SRR17520069.6310761
SRR17520069.6323579
SRR17520069.6323600
SRR17520069.6338536
SRR17520069.6338537
SRR17520069.6338538
SRR17520069.6338539
SRR17520069.6338541
SRR17520069.6338543
SRR17520069.6338544
SRR17520069.6338545
SRR17520069.6338546
SRR17520069.6338547
SRR17520069.6338548
SRR17520069.6338549
SRR17520069.6338550
SRR17520069.6338551
SRR17520069.6338552
SRR17520069.6338553
SRR17520069.6338554
SRR17520069.6338555
SRR17520069.6338556
SRR17520069.6338557
SRR17520069.6338558
SRR17520069.6338559
SRR17520069.6338560
SRR17520069.6338561
SRR17520069.6338562
SRR17520069.6338563
SRR17520069.6338564
SRR17520069.6343705
SRR17520069.6418271
SRR17520069.6418272
SRR17520069.6418273
SRR17520069.6418274
SRR17520069.6418275
SRR17520069.6418276
SRR17520069.6418277
SRR17520069.6418278
SRR17520069.6418279
SRR17520069.6418280
SRR17520069.6418281
SRR17520069.6418282
SRR17520069.6418283
SRR17520069.6418284

SRR17520069.6418285
SRR17520069.6418286
SRR17520069.6418287
SRR17520069.6418288
SRR17520069.6418289
SRR17520069.6418290
SRR17520069.6418291
SRR17520069.6418292
SRR17520069.6418293
SRR17520069.6418294
SRR17520069.6418295
SRR17520069.6418296
SRR17520069.6418297
SRR17520069.6418298
SRR17520069.6418299
SRR17520069.6418300
SRR17520069.6418301
SRR17520069.6418302
SRR17520069.6418304
SRR17520069.6418305
SRR17520069.6418306
SRR17520069.6418307
SRR17520069.6418308
SRR17520069.6418309
SRR17520069.6418310
SRR17520069.6418312
SRR17520069.6418313
SRR17520069.6418314
SRR17520069.6418315
SRR17520069.6418316
SRR17520069.6418318
SRR17520069.6418319
SRR17520069.6418320
SRR17520069.6418321
SRR17520069.6418322
SRR17520069.6418323
SRR17520069.6418324
SRR17520069.6418325
SRR17520069.6418326
SRR17520069.6418327
SRR17520069.6418328
SRR17520069.6418329
SRR17520069.6418330
SRR17520069.6418331
SRR17520069.6418332
SRR17520069.6418333
SRR17520069.6418334

SRR17520069.6418335
SRR17520069.6418336
SRR17520069.6418337
SRR17520069.6418338
SRR17520069.6418339
SRR17520069.6419826
SRR17520069.6419827
SRR17520069.6419828
SRR17520069.6419829
SRR17520069.6419830
SRR17520069.6419831
SRR17520069.6419832
SRR17520069.6419833
SRR17520069.6419834
SRR17520069.6419835
SRR17520069.6419836
SRR17520069.6419837
SRR17520069.6419838
SRR17520069.6419839
SRR17520069.6419840
SRR17520069.6419841
SRR17520069.6419842
SRR17520069.6419843
SRR17520069.6419844
SRR17520069.6419845
SRR17520069.6419847
SRR17520069.6419848
SRR17520069.6419849
SRR17520069.6419850
SRR17520069.6419851
SRR17520069.6419852
SRR17520069.6419853
SRR17520069.6419854
SRR17520069.6419855
SRR17520069.6419856
SRR17520069.6419857
SRR17520069.6419858
SRR17520069.6419859
SRR17520069.6419860
SRR17520069.6419861
SRR17520069.6419862
SRR17520069.6419863
SRR17520069.6419864
SRR17520069.6419865
SRR17520069.6419866
SRR17520069.6419867
SRR17520069.6419868

SRR17520069.6419869
SRR17520069.6419870
SRR17520069.6419871
SRR17520069.6419872
SRR17520069.6419873
SRR17520069.6419874
SRR17520069.6419875
SRR17520069.6419876
SRR17520069.6419877
SRR17520069.6419878
SRR17520069.6419879
SRR17520069.6419880
SRR17520069.6419881
SRR17520069.6419882
SRR17520069.6419883
SRR17520069.6419884
SRR17520069.6419885
SRR17520069.6419886
SRR17520069.6419887
SRR17520069.6419888
SRR17520069.6419889
SRR17520069.6419890
SRR17520069.6419891
SRR17520069.6419892
SRR17520069.6419893
SRR17520069.6419894
SRR17520069.6419895
SRR17520069.6419896
SRR17520069.6419897
SRR17520069.6419898
SRR17520069.6419899
SRR17520069.6421188
SRR17520069.6421198
SRR17520069.6431481
SRR17520069.6431482
SRR17520069.6431483
SRR17520069.6431484
SRR17520069.6431485
SRR17520069.6431486
SRR17520069.6431487
SRR17520069.6431488
SRR17520069.6431489
SRR17520069.6431490
SRR17520069.6431491
SRR17520069.6431492
SRR17520069.6431493
SRR17520069.6431494

SRR17520069.6431495
SRR17520069.6431496
SRR17520069.6431497
SRR17520069.6431498
SRR17520069.6431499
SRR17520069.6431500
SRR17520069.6431501
SRR17520069.6431502
SRR17520069.6431503
SRR17520069.6431504
SRR17520069.6431505
SRR17520069.6431506
SRR17520069.6431507
SRR17520069.6431508
SRR17520069.6431509
SRR17520069.6431510
SRR17520069.6431511
SRR17520069.6431512
SRR17520069.6431513
SRR17520069.6431514
SRR17520069.6431515
SRR17520069.6431516
SRR17520069.6431517
SRR17520069.6446182
SRR17520069.6446183
SRR17520069.6446184
SRR17520069.6446185
SRR17520069.6446186
SRR17520069.6446187
SRR17520069.6446188
SRR17520069.6446189
SRR17520069.6446190
SRR17520069.6446191
SRR17520069.6446192
SRR17520069.6446193
SRR17520069.6446194
SRR17520069.6446195
SRR17520069.6446196
SRR17520069.6446197
SRR17520069.6446198
SRR17520069.6446199
SRR17520069.6446200
SRR17520069.6446201
SRR17520069.6446202
SRR17520069.6446204
SRR17520069.6446205
SRR17520069.6446206

SRR17520069.6446207
SRR17520069.6446208
SRR17520069.6446209
SRR17520069.6446210
SRR17520069.6446211
SRR17520069.6446212
SRR17520069.6446213
SRR17520069.6446214
SRR17520069.6446215
SRR17520069.6446216
SRR17520069.6446217
SRR17520069.6446218
SRR17520069.6446219
SRR17520069.6446220
SRR17520069.6446221
SRR17520069.6446223
SRR17520069.6446224
SRR17520069.6448920
SRR17520069.6448921
SRR17520069.6448922
SRR17520069.6448923
SRR17520069.6448924
SRR17520069.6448925
SRR17520069.6448926
SRR17520069.6448927
SRR17520069.6448928
SRR17520069.6448929
SRR17520069.6448930
SRR17520069.6448931
SRR17520069.6448932
SRR17520069.6448933
SRR17520069.6448934
SRR17520069.6448935
SRR17520069.6448936
SRR17520069.6448937
SRR17520069.6448938
SRR17520069.6448939
SRR17520069.6448940
SRR17520069.6448941
SRR17520069.6448942
SRR17520069.6448943
SRR17520069.6448944
SRR17520069.6448945
SRR17520069.6448946
SRR17520069.6448947
SRR17520069.6448948
SRR17520069.6448949

SRR17520069.6448950
SRR17520069.6448951
SRR17520069.6448952
SRR17520069.6448953
SRR17520069.6448954
SRR17520069.6448955
SRR17520069.6448956
SRR17520069.6448957
SRR17520069.6448958
SRR17520069.6448959
SRR17520069.6448960
SRR17520069.6448961
SRR17520069.6448962
SRR17520069.6448963
SRR17520069.6448964
SRR17520069.6448965
SRR17520069.6448966
SRR17520069.6448967
SRR17520069.6448968
SRR17520069.6448969
SRR17520069.6448970
SRR17520069.6448971
SRR17520069.6448972
SRR17520069.6448973
SRR17520069.6448974
SRR17520069.6448975
SRR17520069.6448976
SRR17520069.6448977
SRR17520069.6448978
SRR17520069.6448979
SRR17520069.6448980
SRR17520069.6448981
SRR17520069.6448982
SRR17520069.6448983
SRR17520069.6448985
SRR17520069.6448986
SRR17520069.6448988
SRR17520069.6448989
SRR17520069.6448990
SRR17520069.6448991
SRR17520069.6448992
SRR17520069.6448993
SRR17520069.6448994
SRR17520069.6448995
SRR17520069.6448996
SRR17520069.6448997
SRR17520069.6448998

SRR17520069.6448999
SRR17520069.6449000
SRR17520069.6449001
SRR17520069.6449002
SRR17520069.6449003
SRR17520069.6449004
SRR17520069.6451872
SRR17520069.6451873
SRR17520069.6451874
SRR17520069.6480948
SRR17520069.6482752
SRR17520069.6487148
SRR17520069.6487149
SRR17520069.6487150
SRR17520069.6487151
SRR17520069.6487152
SRR17520069.6487153
SRR17520069.6487154
SRR17520069.6487155
SRR17520069.6487156
SRR17520069.6487157
SRR17520069.6487158
SRR17520069.6487159
SRR17520069.6487160
SRR17520069.6487161
SRR17520069.6487162
SRR17520069.6487163
SRR17520069.6487164
SRR17520069.6487165
SRR17520069.6487166
SRR17520069.6487167
SRR17520069.6487168
SRR17520069.6487169
SRR17520069.6487170
SRR17520069.6487171
SRR17520069.6487172
SRR17520069.6487173
SRR17520069.6487174
SRR17520069.6487175
SRR17520069.6487176
SRR17520069.6487177
SRR17520069.6487178
SRR17520069.6487179
SRR17520069.6487180
SRR17520069.6505602
SRR17520069.6505603
SRR17520069.6505604

SRR17520069.6505605
SRR17520069.6505606
SRR17520069.6505607
SRR17520069.6505608
SRR17520069.6505609
SRR17520069.6505610
SRR17520069.6505611
SRR17520069.6505612
SRR17520069.6505613
SRR17520069.6505614
SRR17520069.6505615
SRR17520069.6505616
SRR17520069.6505617
SRR17520069.6505618
SRR17520069.6505619
SRR17520069.6505620
SRR17520069.6505622
SRR17520069.6505623
SRR17520069.6505625
SRR17520069.6505626
SRR17520069.6505627
SRR17520069.6505628
SRR17520069.6505629
SRR17520069.6505630
SRR17520069.6505631
SRR17520069.6505632
SRR17520069.6505633
SRR17520069.6505634
SRR17520069.6505635
SRR17520069.6505636
SRR17520069.6505637
SRR17520069.6505639
SRR17520069.6505640
SRR17520069.6505642
SRR17520069.6505643
SRR17520069.6505644
SRR17520069.6505645
SRR17520069.6505646
SRR17520069.6505647
SRR17520069.6505650
SRR17520069.6505651
SRR17520069.6505652
SRR17520069.6505653
SRR17520069.6505654
SRR17520069.6505655
SRR17520069.6505656
SRR17520069.6505657

SRR17520069.6505658
SRR17520069.6505659
SRR17520069.6505660
SRR17520069.6505661
SRR17520069.6505662
SRR17520069.6505663
SRR17520069.6505664
SRR17520069.6505665
SRR17520069.6505666
SRR17520069.6505667
SRR17520069.6505668
SRR17520069.6505669
SRR17520069.6505670
SRR17520069.6505671
SRR17520069.6505672
SRR17520069.6505673
SRR17520069.6505674
SRR17520069.6505675
SRR17520069.6505676
SRR17520069.6505677
SRR17520069.6505678
SRR17520069.6505679
SRR17520069.6505680
SRR17520069.6505681
SRR17520069.6505682
SRR17520069.6505683
SRR17520069.6505684
SRR17520069.6505685
SRR17520069.6505686
SRR17520069.6505687
SRR17520069.6505688
SRR17520069.6505689
SRR17520069.6505690
SRR17520069.6505691
SRR17520069.6505692
SRR17520069.6505693
SRR17520069.6508844
SRR17520069.6508845
SRR17520069.6535191
SRR17520069.6535192
SRR17520069.6535193
SRR17520069.6535194
SRR17520069.6535195
SRR17520069.6535196
SRR17520069.6535197
SRR17520069.6535198
SRR17520069.6535199

SRR17520069.6535200
SRR17520069.6535201
SRR17520069.6535202
SRR17520069.6535203
SRR17520069.6535204
SRR17520069.6535205
SRR17520069.6535206
SRR17520069.6535207
SRR17520069.6535208
SRR17520069.6535209
SRR17520069.6535210
SRR17520069.6535211
SRR17520069.6535212
SRR17520069.6535213
SRR17520069.6535214
SRR17520069.6535215
SRR17520069.6535216
SRR17520069.6535217
SRR17520069.6535218
SRR17520069.6535219
SRR17520069.6535220
SRR17520069.6535221
SRR17520069.6535222
SRR17520069.6535223
SRR17520069.6535225
SRR17520069.6538620
SRR17520069.6548561
SRR17520069.6548562
SRR17520069.6548563
SRR17520069.6548564
SRR17520069.6548565
SRR17520069.6548566
SRR17520069.6548567
SRR17520069.6548568
SRR17520069.6548571
SRR17520069.6548572
SRR17520069.6548573
SRR17520069.6548574
SRR17520069.6548575
SRR17520069.6548576
SRR17520069.6548592
SRR17520069.6548593
SRR17520069.6548594
SRR17520069.6548595
SRR17520069.6548596
SRR17520069.6548597
SRR17520069.6548598

SRR17520069.6548599
SRR17520069.6548600
SRR17520069.6548601
SRR17520069.6548602
SRR17520069.6548603
SRR17520069.6548604
SRR17520069.6548605
SRR17520069.6548606
SRR17520069.6548607
SRR17520069.6548608
SRR17520069.6548609
SRR17520069.6548610
SRR17520069.6548611
SRR17520069.6548612
SRR17520069.6548613
SRR17520069.6548614
SRR17520069.6548615
SRR17520069.6548616
SRR17520069.6548617
SRR17520069.6548618
SRR17520069.6548619
SRR17520069.6548620
SRR17520069.6548621
SRR17520069.6548622
SRR17520069.6548623
SRR17520069.6548624
SRR17520069.6548625
SRR17520069.6548626
SRR17520069.6548627
SRR17520069.6548628
SRR17520069.6548629
SRR17520069.6548630
SRR17520069.6548631
SRR17520069.6548632
SRR17520069.6548633
SRR17520069.6548634
SRR17520069.6548635
SRR17520069.6548636
SRR17520069.6548637
SRR17520069.6548638
SRR17520069.6548639
SRR17520069.6548640
SRR17520069.6548641
SRR17520069.6548642
SRR17520069.6548643
SRR17520069.6554075
SRR17520069.6554076

SRR17520069.6554077
SRR17520069.6554078
SRR17520069.6554079
SRR17520069.6554080
SRR17520069.6554081
SRR17520069.6554082
SRR17520069.6554083
SRR17520069.6554084
SRR17520069.6554085
SRR17520069.6554086
SRR17520069.6554087
SRR17520069.6554088
SRR17520069.6554089
SRR17520069.6554090
SRR17520069.6554091
SRR17520069.6554092
SRR17520069.6554093
SRR17520069.6554094
SRR17520069.6554095
SRR17520069.6554096
SRR17520069.6554097
SRR17520069.6554098
SRR17520069.6554099
SRR17520069.6554100
SRR17520069.6554101
SRR17520069.6554102
SRR17520069.6554103
SRR17520069.6554104
SRR17520069.6554105
SRR17520069.6554106
SRR17520069.6554107
SRR17520069.6554109
SRR17520069.6554110
SRR17520069.6554111
SRR17520069.6554112
SRR17520069.6554113
SRR17520069.6554114
SRR17520069.6554115
SRR17520069.6554116
SRR17520069.6554117
SRR17520069.6554118
SRR17520069.6554119
SRR17520069.6554120
SRR17520069.6554121
SRR17520069.6554122
SRR17520069.6554123
SRR17520069.6554124

SRR17520069.6554125
SRR17520069.6554126
SRR17520069.6554127
SRR17520069.6554128
SRR17520069.6554129
SRR17520069.6554130
SRR17520069.6554131
SRR17520069.6554132
SRR17520069.6554133
SRR17520069.6554134
SRR17520069.6554137
SRR17520069.6554148
SRR17520069.6554149
SRR17520069.6554150
SRR17520069.6554151
SRR17520069.6554152
SRR17520069.6554153
SRR17520069.6554154
SRR17520069.6554155
SRR17520069.6554156
SRR17520069.6554157
SRR17520069.6554158
SRR17520069.6554159
SRR17520069.6554160
SRR17520069.6554161
SRR17520069.6554162
SRR17520069.6554163
SRR17520069.6554164
SRR17520069.6554165
SRR17520069.6554167
SRR17520069.6554168
SRR17520069.6554169
SRR17520069.6554170
SRR17520069.6554171
SRR17520069.6554172
SRR17520069.6554173
SRR17520069.6554174
SRR17520069.6554175
SRR17520069.6554176
SRR17520069.6554177
SRR17520069.6554178
SRR17520069.6554179
SRR17520069.6554180
SRR17520069.6554181
SRR17520069.6554182
SRR17520069.6554183
SRR17520069.6554184

SRR17520069.6554185
SRR17520069.6554186
SRR17520069.6554187
SRR17520069.6554188
SRR17520069.6554189
SRR17520069.6554190
SRR17520069.6594551
SRR17520069.6594552
SRR17520069.6594553
SRR17520069.6594554
SRR17520069.6594555
SRR17520069.6594556
SRR17520069.6594557
SRR17520069.6594558
SRR17520069.6594559
SRR17520069.6594561
SRR17520069.6594563
SRR17520069.6594564
SRR17520069.6594565
SRR17520069.6594566
SRR17520069.6594567
SRR17520069.6594568
SRR17520069.6594569
SRR17520069.6594570
SRR17520069.6594571
SRR17520069.6594572
SRR17520069.6594573
SRR17520069.6594574
SRR17520069.6594575
SRR17520069.6594576
SRR17520069.6594577
SRR17520069.6594578
SRR17520069.6594579
SRR17520069.6594580
SRR17520069.6594581
SRR17520069.6594582
SRR17520069.6594583
SRR17520069.6594584
SRR17520069.6594585
SRR17520069.6594586
SRR17520069.6594587
SRR17520069.6594588
SRR17520069.6594589
SRR17520069.6594590
SRR17520069.6594591
SRR17520069.6594592
SRR17520069.6594593

SRR17520069.6594594
SRR17520069.6594595
SRR17520069.6594596
SRR17520069.6594597
SRR17520069.6594598
SRR17520069.6594599
SRR17520069.6594600
SRR17520069.6594601
SRR17520069.6594602
SRR17520069.6594603
SRR17520069.6594604
SRR17520069.6594605
SRR17520069.6594606
SRR17520069.6594607
SRR17520069.6594608
SRR17520069.6594609
SRR17520069.6594610
SRR17520069.6594611
SRR17520069.6594612
SRR17520069.6594613
SRR17520069.6594614
SRR17520069.6594615
SRR17520069.6594616
SRR17520069.6594617
SRR17520069.6594618
SRR17520069.6594619
SRR17520069.6594620
SRR17520069.6594621
SRR17520069.6594622
SRR17520069.6594623
SRR17520069.6594624
SRR17520069.6594625
SRR17520069.6594627
SRR17520069.6594628
SRR17520069.6594629
SRR17520069.6594630
SRR17520069.6594631
SRR17520069.6594632
SRR17520069.6594633
SRR17520069.6594634
SRR17520069.6594635
SRR17520069.6594636
SRR17520069.6594637
SRR17520069.6594638
SRR17520069.6594639
SRR17520069.6594640
SRR17520069.6594641

SRR17520069.6594642
SRR17520069.6594643
SRR17520069.6594644
SRR17520069.6594645
SRR17520069.6594646
SRR17520069.6594647
SRR17520069.6594648
SRR17520069.6594649
SRR17520069.6594650
SRR17520069.6594651
SRR17520069.6594652
SRR17520069.6594653
SRR17520069.6594654
SRR17520069.6594655
SRR17520069.6594656
SRR17520069.6594657
SRR17520069.6594658
SRR17520069.6594659
SRR17520069.6594660
SRR17520069.6594661
SRR17520069.6594662
SRR17520069.6594663
SRR17520069.6594664
SRR17520069.6594665
SRR17520069.6594666
SRR17520069.6594667
SRR17520069.6594668
SRR17520069.6594669
SRR17520069.6596584
SRR17520069.6596585
SRR17520069.6596586
SRR17520069.6596588
SRR17520069.6596590
SRR17520069.6596591
SRR17520069.6596598
SRR17520069.6598065
SRR17520069.6634449
SRR17520069.6636093
SRR17520069.6642563
SRR17520069.6642564
SRR17520069.6642565
SRR17520069.6642566
SRR17520069.6642567
SRR17520069.6642568
SRR17520069.6642569
SRR17520069.6642570
SRR17520069.6642571

SRR17520069.6642572
SRR17520069.6642573
SRR17520069.6642574
SRR17520069.6642575
SRR17520069.6642576
SRR17520069.6642577
SRR17520069.6642578
SRR17520069.6642623
SRR17520069.6642624
SRR17520069.6642625
SRR17520069.6642626
SRR17520069.6642627
SRR17520069.6642628
SRR17520069.6642629
SRR17520069.6642630
SRR17520069.6642631
SRR17520069.6642632
SRR17520069.6642633
SRR17520069.6642634
SRR17520069.6642635
SRR17520069.6642636
SRR17520069.6642637
SRR17520069.6642638
SRR17520069.6642639
SRR17520069.6642640
SRR17520069.6642641
SRR17520069.6642642
SRR17520069.6642643
SRR17520069.6642644
SRR17520069.6642645
SRR17520069.6642646
SRR17520069.6642647
SRR17520069.6642648
SRR17520069.6642649
SRR17520069.6642650
SRR17520069.6642651
SRR17520069.6642652
SRR17520069.6642653
SRR17520069.6642654
SRR17520069.6642655
SRR17520069.6642656
SRR17520069.6642657
SRR17520069.6642658
SRR17520069.6642659
SRR17520069.6642660
SRR17520069.6642661
SRR17520069.6642662

SRR17520069.6642663
SRR17520069.6642664
SRR17520069.6642665
SRR17520069.6642666
SRR17520069.6642667
SRR17520069.6642668
SRR17520069.6642669
SRR17520069.6642670
SRR17520069.6642671
SRR17520069.6642672
SRR17520069.6642673
SRR17520069.6642674
SRR17520069.6642675
SRR17520069.6642676
SRR17520069.6642677
SRR17520069.6642678
SRR17520069.6642679
SRR17520069.6642680
SRR17520069.6642681
SRR17520069.6642682
SRR17520069.6642683
SRR17520069.6642684
SRR17520069.6642685
SRR17520069.6642686
SRR17520069.6642687
SRR17520069.6642688
SRR17520069.6642689
SRR17520069.6642690
SRR17520069.6642691
SRR17520069.6643652
SRR17520069.6643659
SRR17520069.6644968
SRR17520069.6657705
SRR17520069.6657768
SRR17520069.6660939
SRR17520069.6660940
SRR17520069.6660941
SRR17520069.6660942
SRR17520069.6660943
SRR17520069.6660944
SRR17520069.6660945
SRR17520069.6660946
SRR17520069.6660947
SRR17520069.6660948
SRR17520069.6660949
SRR17520069.6660950
SRR17520069.6660951

SRR17520069.6660952
SRR17520069.6660954
SRR17520069.6660955
SRR17520069.6660956
SRR17520069.6660957
SRR17520069.6660958
SRR17520069.6660959
SRR17520069.6660960
SRR17520069.6660961
SRR17520069.6660962
SRR17520069.6660963
SRR17520069.6663786
SRR17520069.6663787
SRR17520069.6663788
SRR17520069.6663789
SRR17520069.6663790
SRR17520069.6663792
SRR17520069.6663793
SRR17520069.6663794
SRR17520069.6663795
SRR17520069.6663796
SRR17520069.6663797
SRR17520069.6663798
SRR17520069.6663799
SRR17520069.6663800
SRR17520069.6663801
SRR17520069.6663802
SRR17520069.6663803
SRR17520069.6663804
SRR17520069.6663805
SRR17520069.6663806
SRR17520069.6663808
SRR17520069.6663809
SRR17520069.6663810
SRR17520069.6663811
SRR17520069.6663812
SRR17520069.6663813
SRR17520069.6663814
SRR17520069.6663815
SRR17520069.6663816
SRR17520069.6663817
SRR17520069.6663818
SRR17520069.6663820
SRR17520069.6666061
SRR17520069.6666081
SRR17520069.6668147
SRR17520069.6668148

SRR17520069.6668149
SRR17520069.6668150
SRR17520069.6668151
SRR17520069.6668152
SRR17520069.6668153
SRR17520069.6668154
SRR17520069.6668155
SRR17520069.6668156
SRR17520069.6668157
SRR17520069.6668159
SRR17520069.6668160
SRR17520069.6668161
SRR17520069.6668162
SRR17520069.6668163
SRR17520069.6668165
SRR17520069.6668166
SRR17520069.6668167
SRR17520069.6668168
SRR17520069.6668169
SRR17520069.6668170
SRR17520069.6668171
SRR17520069.6668173
SRR17520069.6668174
SRR17520069.6668175
SRR17520069.6668176
SRR17520069.6668177
SRR17520069.6668178
SRR17520069.6668179
SRR17520069.6668180
SRR17520069.6668181
SRR17520069.6668182
SRR17520069.6668183
SRR17520069.6668184
SRR17520069.6668185
SRR17520069.6668187
SRR17520069.6668188
SRR17520069.6668189
SRR17520069.6668190
SRR17520069.6668191
SRR17520069.6668192
SRR17520069.6668193
SRR17520069.6668194
SRR17520069.6668195
SRR17520069.6668197
SRR17520069.6668199
SRR17520069.6668201
SRR17520069.6668202

SRR17520069.6668203
SRR17520069.6668204
SRR17520069.6668205
SRR17520069.6668206
SRR17520069.6668207
SRR17520069.6668209
SRR17520069.6668210
SRR17520069.6668211
SRR17520069.6668212
SRR17520069.6668213
SRR17520069.6668214
SRR17520069.6668215
SRR17520069.6668216
SRR17520069.6668217
SRR17520069.6668218
SRR17520069.6668219
SRR17520069.6668220
SRR17520069.6668221
SRR17520069.6668222
SRR17520069.6668223
SRR17520069.6668224
SRR17520069.6668225
SRR17520069.6668226
SRR17520069.6668227
SRR17520069.6668228
SRR17520069.6668229
SRR17520069.6668230
SRR17520069.6668231
SRR17520069.6668232
SRR17520069.6668233
SRR17520069.6678547
SRR17520069.6678548
SRR17520069.6678549
SRR17520069.6678550
SRR17520069.6678551
SRR17520069.6678552
SRR17520069.6678553
SRR17520069.6678554
SRR17520069.6678555
SRR17520069.6678556
SRR17520069.6678557
SRR17520069.6678558
SRR17520069.6678559
SRR17520069.6678560
SRR17520069.6678561
SRR17520069.6678562
SRR17520069.6678563

SRR17520069.6678564
SRR17520069.6678565
SRR17520069.6678567
SRR17520069.6678568
SRR17520069.6678570
SRR17520069.6678571
SRR17520069.6678572
SRR17520069.6678573
SRR17520069.6678575
SRR17520069.6678576
SRR17520069.6678577
SRR17520069.6678578
SRR17520069.6678579
SRR17520069.6678580
SRR17520069.6678581
SRR17520069.6678582
SRR17520069.6678583
SRR17520069.6678584
SRR17520069.6678585
SRR17520069.6678586
SRR17520069.6678587
SRR17520069.6678588
SRR17520069.6678589
SRR17520069.6678590
SRR17520069.6678591
SRR17520069.6678592
SRR17520069.6678595
SRR17520069.6678596
SRR17520069.6678597
SRR17520069.6678598
SRR17520069.6678599
SRR17520069.6678600
SRR17520069.6678601
SRR17520069.6678602
SRR17520069.6678603
SRR17520069.6678604
SRR17520069.6678605
SRR17520069.6678606
SRR17520069.6678607
SRR17520069.6678608
SRR17520069.6678609
SRR17520069.6678610
SRR17520069.6678611
SRR17520069.6678612
SRR17520069.6678613
SRR17520069.6678614
SRR17520069.6678615

SRR17520069.6678616
SRR17520069.6678617
SRR17520069.6678618
SRR17520069.6678619
SRR17520069.6678620
SRR17520069.6678621
SRR17520069.6678622
SRR17520069.6678625
SRR17520069.6678626
SRR17520069.6678627
SRR17520069.6678628
SRR17520069.6678629
SRR17520069.6678630
SRR17520069.6678631
SRR17520069.6678632
SRR17520069.6678633
SRR17520069.6678634
SRR17520069.6678635
SRR17520069.6678636
SRR17520069.6678637
SRR17520069.6678638
SRR17520069.6678639
SRR17520069.6678640
SRR17520069.6678641
SRR17520069.6678642
SRR17520069.6678643
SRR17520069.6678644
SRR17520069.6678645
SRR17520069.6678646
SRR17520069.6678647
SRR17520069.6678648
SRR17520069.6678649
SRR17520069.6678650
SRR17520069.6678652
SRR17520069.6678653
SRR17520069.6678654
SRR17520069.6678655
SRR17520069.6678656
SRR17520069.6678657
SRR17520069.6678658
SRR17520069.6678659
SRR17520069.6678660
SRR17520069.6678661
SRR17520069.6678662
SRR17520069.6678663
SRR17520069.6678664
SRR17520069.6678665

SRR17520069.6689291
SRR17520069.6689292
SRR17520069.6689293
SRR17520069.6689294
SRR17520069.6689295
SRR17520069.6689296
SRR17520069.6689297
SRR17520069.6689298
SRR17520069.6689299
SRR17520069.6689300
SRR17520069.6689301
SRR17520069.6689302
SRR17520069.6689303
SRR17520069.6689304
SRR17520069.6689305
SRR17520069.6689306
SRR17520069.6689307
SRR17520069.6689308
SRR17520069.6689309
SRR17520069.6689310
SRR17520069.6689311
SRR17520069.6689312
SRR17520069.6689313
SRR17520069.6689314
SRR17520069.6689315
SRR17520069.6689316
SRR17520069.6689317
SRR17520069.6689318
SRR17520069.6689319
SRR17520069.6689320
SRR17520069.6689321
SRR17520069.6689322
SRR17520069.6689323
SRR17520069.6689324
SRR17520069.6689325
SRR17520069.6689326
SRR17520069.6689327
SRR17520069.6689328
SRR17520069.6689329
SRR17520069.6689331
SRR17520069.6689332
SRR17520069.6689333
SRR17520069.6689334
SRR17520069.6689335
SRR17520069.6689336
SRR17520069.6689337
SRR17520069.6689338

SRR17520069.6689339
SRR17520069.6689340
SRR17520069.6689341
SRR17520069.6689342
SRR17520069.6689343
SRR17520069.6689344
SRR17520069.6689345
SRR17520069.6689346
SRR17520069.6689347
SRR17520069.6689348
SRR17520069.6689349
SRR17520069.6689350
SRR17520069.6689351
SRR17520069.6689352
SRR17520069.6689353
SRR17520069.6689354
SRR17520069.6689355
SRR17520069.6689356
SRR17520069.6689357
SRR17520069.6689358
SRR17520069.6689359
SRR17520069.6689360
SRR17520069.6689361
SRR17520069.6689362
SRR17520069.6689363
SRR17520069.6689364
SRR17520069.6689365
SRR17520069.6689366
SRR17520069.6689367
SRR17520069.6689368
SRR17520069.6689369
SRR17520069.6689370
SRR17520069.6689371
SRR17520069.6689372
SRR17520069.6689373
SRR17520069.6689374
SRR17520069.6689375
SRR17520069.6689377
SRR17520069.6689378
SRR17520069.6689379
SRR17520069.6689380
SRR17520069.6689381
SRR17520069.6689382
SRR17520069.6689383
SRR17520069.6689384
SRR17520069.6689385
SRR17520069.6689386

SRR17520069.6689387
SRR17520069.6689388
SRR17520069.6689389
SRR17520069.6689390
SRR17520069.6689391
SRR17520069.6689392
SRR17520069.6689393
SRR17520069.6689394
SRR17520069.6689395
SRR17520069.6689396
SRR17520069.6689397
SRR17520069.6689398
SRR17520069.6689399
SRR17520069.6689400
SRR17520069.6689401
SRR17520069.6689402
SRR17520069.6689403
SRR17520069.6689405
SRR17520069.6689406
SRR17520069.6689407
SRR17520069.6689408
SRR17520069.6689409
SRR17520069.6689410
SRR17520069.6689411
SRR17520069.6689412
SRR17520069.6689413
SRR17520069.6689414
SRR17520069.6689415
SRR17520069.6689416
SRR17520069.6689417
SRR17520069.6689418
SRR17520069.6689419
SRR17520069.6689420
SRR17520069.6689421
SRR17520069.6689422
SRR17520069.6689423
SRR17520069.6689424
SRR17520069.6691508
SRR17520069.6719188
SRR17520069.6719189
SRR17520069.6719190
SRR17520069.6719191
SRR17520069.6719192
SRR17520069.6719193
SRR17520069.6719194
SRR17520069.6719195
SRR17520069.6719196

SRR17520069.6719197
SRR17520069.6719198
SRR17520069.6719199
SRR17520069.6719200
SRR17520069.6719201
SRR17520069.6719202
SRR17520069.6719203
SRR17520069.6719204
SRR17520069.6719205
SRR17520069.6719206
SRR17520069.6719207
SRR17520069.6719208
SRR17520069.6719210
SRR17520069.6719211
SRR17520069.6719212
SRR17520069.6719213
SRR17520069.6752853
SRR17520069.6752854
SRR17520069.6752855
SRR17520069.6752856
SRR17520069.6752857
SRR17520069.6752858
SRR17520069.6752859
SRR17520069.6752860
SRR17520069.6752861
SRR17520069.6752862
SRR17520069.6752863
SRR17520069.6752864
SRR17520069.6752865
SRR17520069.6752866
SRR17520069.6752867
SRR17520069.6752868
SRR17520069.6752869
SRR17520069.6766250
SRR17520069.6766251
SRR17520069.6766252
SRR17520069.6766253
SRR17520069.6766254
SRR17520069.6766255
SRR17520069.6766256
SRR17520069.6766257
SRR17520069.6766258
SRR17520069.6766259
SRR17520069.6766260
SRR17520069.6766261
SRR17520069.6766262
SRR17520069.6766263

SRR17520069.6766264
SRR17520069.6766265
SRR17520069.6766266
SRR17520069.6766267
SRR17520069.6766268
SRR17520069.6766269
SRR17520069.6766270
SRR17520069.6766272
SRR17520069.6766273
SRR17520069.6766274
SRR17520069.6766275
SRR17520069.6766276
SRR17520069.6766277
SRR17520069.6766278
SRR17520069.6766279
SRR17520069.6766280
SRR17520069.6766281
SRR17520069.6766282
SRR17520069.6766283
SRR17520069.6766284
SRR17520069.6766285
SRR17520069.6766286
SRR17520069.6766287
SRR17520069.6766288
SRR17520069.6766289
SRR17520069.6766291
SRR17520069.6766292
SRR17520069.6766293
SRR17520069.6766294
SRR17520069.6766295
SRR17520069.6766296
SRR17520069.6766297
SRR17520069.6766298
SRR17520069.6766299
SRR17520069.6766300
SRR17520069.6766302
SRR17520069.6766303
SRR17520069.6766304
SRR17520069.6766305
SRR17520069.6766306
SRR17520069.6766307
SRR17520069.6766308
SRR17520069.6766309
SRR17520069.6766310
SRR17520069.6766311
SRR17520069.6766312
SRR17520069.6766313

SRR17520069.6766314
SRR17520069.6766315
SRR17520069.6766316
SRR17520069.6766317
SRR17520069.6766318
SRR17520069.6766319
SRR17520069.6766320
SRR17520069.6766321
SRR17520069.6766322
SRR17520069.6766323
SRR17520069.6766324
SRR17520069.6766325
SRR17520069.6766326
SRR17520069.6766327
SRR17520069.6766328
SRR17520069.6766329
SRR17520069.6766330
SRR17520069.6766331
SRR17520069.6766332
SRR17520069.6766333
SRR17520069.6766334
SRR17520069.6766335
SRR17520069.6766336
SRR17520069.6766337
SRR17520069.6766338
SRR17520069.6766339
SRR17520069.6766340
SRR17520069.6766341
SRR17520069.6766342
SRR17520069.6768617
SRR17520069.6768717
SRR17520069.6773195
SRR17520069.6773197
SRR17520069.6773198
SRR17520069.6773199
SRR17520069.6773200
SRR17520069.6773201
SRR17520069.6773203
SRR17520069.6773204
SRR17520069.6773206
SRR17520069.6773207
SRR17520069.6783043
SRR17520069.6795708
SRR17520069.6810742
SRR17520069.6812330
SRR17520069.6812363
SRR17520069.6824347

SRR17520069.6824909
SRR17520069.6826073
SRR17520069.6826074
SRR17520069.6826075
SRR17520069.6826076
SRR17520069.6826077
SRR17520069.6826079
SRR17520069.6826080
SRR17520069.6826081
SRR17520069.6826082
SRR17520069.6826083
SRR17520069.6826084
SRR17520069.6826085
SRR17520069.6826086
SRR17520069.6826087
SRR17520069.6826088
SRR17520069.6826089
SRR17520069.6826090
SRR17520069.6826091
SRR17520069.6826092
SRR17520069.6826093
SRR17520069.6826094
SRR17520069.6826095
SRR17520069.6826096
SRR17520069.6826097
SRR17520069.6826098
SRR17520069.6826099
SRR17520069.6826100
SRR17520069.6826101
SRR17520069.6826102
SRR17520069.6826103
SRR17520069.6826104
SRR17520069.6826105
SRR17520069.6826106
SRR17520069.6826107
SRR17520069.6826108
SRR17520069.6826109
SRR17520069.6826110
SRR17520069.6826111
SRR17520069.6826112
SRR17520069.6826113
SRR17520069.6826114
SRR17520069.6826115
SRR17520069.6826116
SRR17520069.6826117
SRR17520069.6826118
SRR17520069.6826119

SRR17520069.6826120
SRR17520069.6826121
SRR17520069.6826122
SRR17520069.6826123
SRR17520069.6826124
SRR17520069.6826125
SRR17520069.6826126
SRR17520069.6826127
SRR17520069.6826128
SRR17520069.6826129
SRR17520069.6826130
SRR17520069.6826131
SRR17520069.6826132
SRR17520069.6826133
SRR17520069.6826134
SRR17520069.6826135
SRR17520069.6826136
SRR17520069.6826137
SRR17520069.6826138
SRR17520069.6826139
SRR17520069.6826140
SRR17520069.6826141
SRR17520069.6826142
SRR17520069.6826143
SRR17520069.6826144
SRR17520069.6826145
SRR17520069.6826146
SRR17520069.6826147
SRR17520069.6826148
SRR17520069.6826149
SRR17520069.6826150
SRR17520069.6826151
SRR17520069.6826152
SRR17520069.6826153
SRR17520069.6826154
SRR17520069.6826155
SRR17520069.6826156
SRR17520069.6826157
SRR17520069.6837581
SRR17520069.6837582
SRR17520069.6837583
SRR17520069.6837584
SRR17520069.6837585
SRR17520069.6837586
SRR17520069.6837587
SRR17520069.6837588
SRR17520069.6837589

SRR17520069.6837593
SRR17520069.6837597
SRR17520069.6837598
SRR17520069.6837599
SRR17520069.6837600
SRR17520069.6837601
SRR17520069.6837602
SRR17520069.6837603
SRR17520069.6837604
SRR17520069.6837605
SRR17520069.6837606
SRR17520069.6837607
SRR17520069.6837608
SRR17520069.6837609
SRR17520069.6837610
SRR17520069.6837611
SRR17520069.6837612
SRR17520069.6837613
SRR17520069.6837614
SRR17520069.6837615
SRR17520069.6837616
SRR17520069.6837617
SRR17520069.6837619
SRR17520069.6837620
SRR17520069.6837621
SRR17520069.6837622
SRR17520069.6837623
SRR17520069.6837624
SRR17520069.6837625
SRR17520069.6837626
SRR17520069.6837627
SRR17520069.6837628
SRR17520069.6837629
SRR17520069.6837630
SRR17520069.6837631
SRR17520069.6837632
SRR17520069.6837633
SRR17520069.6838800
SRR17520069.6844583
SRR17520069.6844607
SRR17520069.6848274
SRR17520069.6848275
SRR17520069.6848276
SRR17520069.6848277
SRR17520069.6848278
SRR17520069.6848279
SRR17520069.6848280

SRR17520069.6848281
SRR17520069.6848282
SRR17520069.6848283
SRR17520069.6848284
SRR17520069.6848285
SRR17520069.6848286
SRR17520069.6848287
SRR17520069.6855661
SRR17520069.6855662
SRR17520069.6855663
SRR17520069.6855664
SRR17520069.6855665
SRR17520069.6855666
SRR17520069.6855667
SRR17520069.6855668
SRR17520069.6855669
SRR17520069.6855670
SRR17520069.6855671
SRR17520069.6855672
SRR17520069.6855673
SRR17520069.6855675
SRR17520069.6855676
SRR17520069.6855677
SRR17520069.6855678
SRR17520069.6855679
SRR17520069.6855680
SRR17520069.6855681
SRR17520069.6855682
SRR17520069.6855683
SRR17520069.6855684
SRR17520069.6855685
SRR17520069.6855686
SRR17520069.6855687
SRR17520069.6855688
SRR17520069.6855689
SRR17520069.6855690
SRR17520069.6855691
SRR17520069.6855692
SRR17520069.6855693
SRR17520069.6855694
SRR17520069.6855695
SRR17520069.6855696
SRR17520069.6855697
SRR17520069.6855698
SRR17520069.6855699
SRR17520069.6855700
SRR17520069.6858116

SRR17520069.6862903
SRR17520069.6863158
SRR17520069.6863159
SRR17520069.6876271
SRR17520069.6876272
SRR17520069.6876273
SRR17520069.6876274
SRR17520069.6876275
SRR17520069.6876276
SRR17520069.6876277
SRR17520069.6876278
SRR17520069.6876279
SRR17520069.6876280
SRR17520069.6876281
SRR17520069.6876282
SRR17520069.6876283
SRR17520069.6876284
SRR17520069.6876285
SRR17520069.6876286
SRR17520069.6876287
SRR17520069.6876288
SRR17520069.6876289
SRR17520069.6876290
SRR17520069.6876291
SRR17520069.6876292
SRR17520069.6876293
SRR17520069.6876294
SRR17520069.6876295
SRR17520069.6876296
SRR17520069.6876297
SRR17520069.6876298
SRR17520069.6876299
SRR17520069.6876300
SRR17520069.6876301
SRR17520069.6876302
SRR17520069.6876303
SRR17520069.6876304
SRR17520069.6876305
SRR17520069.6876306
SRR17520069.6876307
SRR17520069.6876308
SRR17520069.6876309
SRR17520069.6876310
SRR17520069.6876311
SRR17520069.6876312
SRR17520069.6876313
SRR17520069.6876314

SRR17520069.6876315
SRR17520069.6876316
SRR17520069.6876317
SRR17520069.6876318
SRR17520069.6876319
SRR17520069.6876320
SRR17520069.6876321
SRR17520069.6876322
SRR17520069.6876323
SRR17520069.6876324
SRR17520069.6876325
SRR17520069.6876326
SRR17520069.6876327
SRR17520069.6876328
SRR17520069.6876329
SRR17520069.6876330
SRR17520069.6876331
SRR17520069.6876333
SRR17520069.6876334
SRR17520069.6876335
SRR17520069.6876336
SRR17520069.6876337
SRR17520069.6876338
SRR17520069.6876339
SRR17520069.6876340
SRR17520069.6876341
SRR17520069.6879370
SRR17520069.6879371
SRR17520069.6879372
SRR17520069.6879373
SRR17520069.6879374
SRR17520069.6879375
SRR17520069.6879376
SRR17520069.6879377
SRR17520069.6879378
SRR17520069.6879379
SRR17520069.6879380
SRR17520069.6879381
SRR17520069.6879382
SRR17520069.6879383
SRR17520069.6879384
SRR17520069.6879385
SRR17520069.6879386
SRR17520069.6879387
SRR17520069.6879388
SRR17520069.6879389
SRR17520069.6879390

SRR17520069.6879391
SRR17520069.6879392
SRR17520069.6879394
SRR17520069.6879395
SRR17520069.6879396
SRR17520069.6879397
SRR17520069.6879398
SRR17520069.6879399
SRR17520069.6879400
SRR17520069.6879401
SRR17520069.6879402
SRR17520069.6879403
SRR17520069.6879404
SRR17520069.6879405
SRR17520069.6879406
SRR17520069.6879407
SRR17520069.6879408
SRR17520069.6879409
SRR17520069.6879410
SRR17520069.6879411
SRR17520069.6879412
SRR17520069.6879413
SRR17520069.6879414
SRR17520069.6879415
SRR17520069.6879416
SRR17520069.6879417
SRR17520069.6879418
SRR17520069.6879419
SRR17520069.6879420
SRR17520069.6879421
SRR17520069.6911736
SRR17520069.6911745
SRR17520069.6913825
SRR17520069.6914012
SRR17520069.6914013
SRR17520069.6914014
SRR17520069.6914015
SRR17520069.6914016
SRR17520069.6914017
SRR17520069.6914018
SRR17520069.6921171
SRR17520069.6933583
SRR17520069.6943995
SRR17520069.6947659
SRR17520069.6953992
SRR17520069.6953993
SRR17520069.6953994

SRR17520069.6953995
SRR17520069.6953996
SRR17520069.6953997
SRR17520069.6954785
SRR17520069.6954786
SRR17520069.6954787
SRR17520069.6954788
SRR17520069.6954789
SRR17520069.6954790
SRR17520069.6954791
SRR17520069.6954792
SRR17520069.6954793
SRR17520069.6954794
SRR17520069.6954795
SRR17520069.6954796
SRR17520069.6954797
SRR17520069.6954798
SRR17520069.6954799
SRR17520069.6954800
SRR17520069.6954801
SRR17520069.6954802
SRR17520069.6954803
SRR17520069.6954804
SRR17520069.6954805
SRR17520069.6954806
SRR17520069.6954807
SRR17520069.6954808
SRR17520069.6954809
SRR17520069.6954810
SRR17520069.6954811
SRR17520069.6954812
SRR17520069.6954813
SRR17520069.6954814
SRR17520069.6954815
SRR17520069.6954816
SRR17520069.6954817
SRR17520069.6954818
SRR17520069.6954819
SRR17520069.6954820
SRR17520069.6954821
SRR17520069.6954823
SRR17520069.6954824
SRR17520069.6954825
SRR17520069.6954827
SRR17520069.6954828
SRR17520069.6954829
SRR17520069.6954830

SRR17520069.6954832
SRR17520069.6954833
SRR17520069.6954834
SRR17520069.6954835
SRR17520069.6954836
SRR17520069.6954837
SRR17520069.6954838
SRR17520069.6954840
SRR17520069.6954841
SRR17520069.6954842
SRR17520069.6954843
SRR17520069.6954844
SRR17520069.6954845
SRR17520069.6954846
SRR17520069.6954847
SRR17520069.6954848
SRR17520069.6954849
SRR17520069.6954850
SRR17520069.6954851
SRR17520069.6954853
SRR17520069.6954854
SRR17520069.6954855
SRR17520069.6954856
SRR17520069.6954857
SRR17520069.6954859
SRR17520069.6954860
SRR17520069.6954861
SRR17520069.6954862
SRR17520069.6954863
SRR17520069.6954864
SRR17520069.6954865
SRR17520069.6954866
SRR17520069.6954867
SRR17520069.6954868
SRR17520069.6954870
SRR17520069.6954871
SRR17520069.6954872
SRR17520069.6954873
SRR17520069.6954874
SRR17520069.6954875
SRR17520069.6974706
SRR17520069.6974707
SRR17520069.6974708
SRR17520069.6974709
SRR17520069.6974710
SRR17520069.6974711
SRR17520069.6974712

SRR17520069.6974713
SRR17520069.6974715
SRR17520069.6974716
SRR17520069.6974717
SRR17520069.6974718
SRR17520069.6974719
SRR17520069.6974720
SRR17520069.6974721
SRR17520069.6974722
SRR17520069.6974723
SRR17520069.6974724
SRR17520069.6974725
SRR17520069.6974726
SRR17520069.6974727
SRR17520069.6974728
SRR17520069.6974729
SRR17520069.6974730
SRR17520069.6974731
SRR17520069.6974732
SRR17520069.6974733
SRR17520069.6974734
SRR17520069.6974735
SRR17520069.6974736
SRR17520069.6974737
SRR17520069.6974738
SRR17520069.6974739
SRR17520069.6974740
SRR17520069.6974741
SRR17520069.6974742
SRR17520069.6974743
SRR17520069.6974744
SRR17520069.6974745
SRR17520069.6974746
SRR17520069.6974747
SRR17520069.6974748
SRR17520069.6974749
SRR17520069.6974750
SRR17520069.6974751
SRR17520069.6974752
SRR17520069.6974753
SRR17520069.6974754
SRR17520069.6974755
SRR17520069.6974756
SRR17520069.6974757
SRR17520069.6974758
SRR17520069.6974759
SRR17520069.6974760

SRR17520069.6974761
SRR17520069.6974762
SRR17520069.6974763
SRR17520069.6974764
SRR17520069.6974765
SRR17520069.6974766
SRR17520069.6974767
SRR17520069.6974768
SRR17520069.6974769
SRR17520069.6974770
SRR17520069.6974771
SRR17520069.6974772
SRR17520069.6974773
SRR17520069.6974774
SRR17520069.6974775
SRR17520069.6974776
SRR17520069.6974777
SRR17520069.6974778
SRR17520069.6974779
SRR17520069.6974780
SRR17520069.6974781
SRR17520069.6974782
SRR17520069.6974783
SRR17520069.6974785
SRR17520069.6974786
SRR17520069.6974787
SRR17520069.6974788
SRR17520069.6974789
SRR17520069.6974790
SRR17520069.6974791
SRR17520069.6974792
SRR17520069.6974793
SRR17520069.6974794
SRR17520069.6974795
SRR17520069.6974796
SRR17520069.6974797
SRR17520069.6974798
SRR17520069.6974799
SRR17520069.6974800
SRR17520069.6974801
SRR17520069.6974802
SRR17520069.6974803
SRR17520069.6974804
SRR17520069.6976587
SRR17520069.6976588
SRR17520069.6976589
SRR17520069.6976590

SRR17520069.6976591
SRR17520069.6976592
SRR17520069.6976593
SRR17520069.6976594
SRR17520069.6976595
SRR17520069.6976596
SRR17520069.6976597
SRR17520069.6976598
SRR17520069.6976599
SRR17520069.6976600
SRR17520069.6976601
SRR17520069.6976602
SRR17520069.6981766
SRR17520069.6981767
SRR17520069.6981768
SRR17520069.6981769
SRR17520069.6981770
SRR17520069.6981771
SRR17520069.6981772
SRR17520069.6981774
SRR17520069.6981775
SRR17520069.6981776
SRR17520069.6981777
SRR17520069.6981778
SRR17520069.6981779
SRR17520069.6981780
SRR17520069.6981781
SRR17520069.6981782
SRR17520069.6981783
SRR17520069.6981784
SRR17520069.6981785
SRR17520069.6981786
SRR17520069.6981787
SRR17520069.6981788
SRR17520069.6981789
SRR17520069.6981790
SRR17520069.6981791
SRR17520069.6981792
SRR17520069.6981793
SRR17520069.6981794
SRR17520069.6981795
SRR17520069.6981796
SRR17520069.6981797
SRR17520069.6981798
SRR17520069.6981799
SRR17520069.6981800
SRR17520069.6981801

SRR17520069.6981802
SRR17520069.6981803
SRR17520069.6981804
SRR17520069.6981805
SRR17520069.6981806
SRR17520069.6981807
SRR17520069.6981808
SRR17520069.6981809
SRR17520069.6981810
SRR17520069.6981811
SRR17520069.6981812
SRR17520069.6981813
SRR17520069.6981814
SRR17520069.6981815
SRR17520069.6981816
SRR17520069.6981817
SRR17520069.6981818
SRR17520069.6981819
SRR17520069.6981820
SRR17520069.6981821
SRR17520069.6981822
SRR17520069.6981823
SRR17520069.6981824
SRR17520069.6981825
SRR17520069.6981826
SRR17520069.6981828
SRR17520069.6981829
SRR17520069.6981830
SRR17520069.6981831
SRR17520069.6981832
SRR17520069.6981833
SRR17520069.6981834
SRR17520069.6981835
SRR17520069.6981836
SRR17520069.6981837
SRR17520069.6981838
SRR17520069.6981839
SRR17520069.6981840
SRR17520069.6981841
SRR17520069.6981842
SRR17520069.6981843
SRR17520069.6981844
SRR17520069.6981845
SRR17520069.6981846
SRR17520069.6981847
SRR17520069.6981848
SRR17520069.6981849

SRR17520069.6981850
SRR17520069.6981851
SRR17520069.6981852
SRR17520069.6981853
SRR17520069.6981854
SRR17520069.6981855
SRR17520069.6981856
SRR17520069.6981857
SRR17520069.6981858
SRR17520069.6981859
SRR17520069.6981860
SRR17520069.6981861
SRR17520069.6981862
SRR17520069.6981863
SRR17520069.6981864
SRR17520069.6981865
SRR17520069.6981866
SRR17520069.6981867
SRR17520069.6981868
SRR17520069.6981869
SRR17520069.6981870
SRR17520069.6981871
SRR17520069.6981872
SRR17520069.6981873
SRR17520069.6981874
SRR17520069.6981875
SRR17520069.6981876
SRR17520069.6981877
SRR17520069.6981878
SRR17520069.6981879
SRR17520069.6981880
SRR17520069.6981881
SRR17520069.6981882
SRR17520069.6981883
SRR17520069.6981884
SRR17520069.6981885
SRR17520069.6981886
SRR17520069.6981887
SRR17520069.6981888
SRR17520069.6981889
SRR17520069.6981890
SRR17520069.6981891
SRR17520069.6981892
SRR17520069.6981893
SRR17520069.6981894
SRR17520069.6981895
SRR17520069.6981896

SRR17520069.6981897
SRR17520069.6981898
SRR17520069.6981899
SRR17520069.6981900
SRR17520069.6981901
SRR17520069.6981902
SRR17520069.6981903
SRR17520069.6981904
SRR17520069.6981905
SRR17520069.6981906
SRR17520069.6981907
SRR17520069.6981908
SRR17520069.6981909
SRR17520069.6981910
SRR17520069.6981911
SRR17520069.6981912
SRR17520069.6981913
SRR17520069.6981914
SRR17520069.6981915
SRR17520069.6981916
SRR17520069.6981918
SRR17520069.6981919
SRR17520069.6981920
SRR17520069.6981921
SRR17520069.6981922
SRR17520069.6981923
SRR17520069.6981924
SRR17520069.6981927
SRR17520069.6981928
SRR17520069.6981929
SRR17520069.6981930
SRR17520069.6981931
SRR17520069.6981932
SRR17520069.6981933
SRR17520069.6981934
SRR17520069.6981935
SRR17520069.6981936
SRR17520069.6981937
SRR17520069.6981938
SRR17520069.6981939
SRR17520069.6981940
SRR17520069.6981941
SRR17520069.6981942
SRR17520069.6981943
SRR17520069.6981944
SRR17520069.6981945
SRR17520069.6981946

SRR17520069.6981947
SRR17520069.6981948
SRR17520069.6981949
SRR17520069.6981950
SRR17520069.6981951
SRR17520069.6981952
SRR17520069.6981953
SRR17520069.6981954
SRR17520069.6981955
SRR17520069.6981956
SRR17520069.6981957
SRR17520069.6981958
SRR17520069.6981959
SRR17520069.6981960
SRR17520069.6990783
SRR17520069.7013888
SRR17520069.7016067
SRR17520069.7017663
SRR17520069.7017664
SRR17520069.7017665
SRR17520069.7017666
SRR17520069.7017667
SRR17520069.7017668
SRR17520069.7017669
SRR17520069.7017670
SRR17520069.7017671
SRR17520069.7017673
SRR17520069.7017674
SRR17520069.7017675
SRR17520069.7017676
SRR17520069.7017677
SRR17520069.7017678
SRR17520069.7017679
SRR17520069.7017680
SRR17520069.7017681
SRR17520069.7017682
SRR17520069.7017683
SRR17520069.7017684
SRR17520069.7017685
SRR17520069.7017686
SRR17520069.7017687
SRR17520069.7017688
SRR17520069.7017689
SRR17520069.7017690
SRR17520069.7017691
SRR17520069.7017692
SRR17520069.7017693

SRR17520069.7017694
SRR17520069.7017695
SRR17520069.7017696
SRR17520069.7017697
SRR17520069.7017698
SRR17520069.7017699
SRR17520069.7017700
SRR17520069.7017701
SRR17520069.7017702
SRR17520069.7017703
SRR17520069.7017704
SRR17520069.7017705
SRR17520069.7017706
SRR17520069.7017707
SRR17520069.7017708
SRR17520069.7017709
SRR17520069.7017710
SRR17520069.7017711
SRR17520069.7017712
SRR17520069.7019816
SRR17520069.7019817
SRR17520069.7019818
SRR17520069.7019819
SRR17520069.7019820
SRR17520069.7033509
SRR17520069.7033510
SRR17520069.7033511
SRR17520069.7033512
SRR17520069.7033513
SRR17520069.7033514
SRR17520069.7033515
SRR17520069.7033516
SRR17520069.7033517
SRR17520069.7033518
SRR17520069.7033519
SRR17520069.7033520
SRR17520069.7033521
SRR17520069.7033522
SRR17520069.7033523
SRR17520069.7033524
SRR17520069.7033525
SRR17520069.7033526
SRR17520069.7033527
SRR17520069.7033528
SRR17520069.7033529
SRR17520069.7033530
SRR17520069.7033531

SRR17520069.7033532
SRR17520069.7033533
SRR17520069.7033534
SRR17520069.7033535
SRR17520069.7033536
SRR17520069.7050690
SRR17520069.7063747
SRR17520069.7064162
SRR17520069.7089415
SRR17520069.7101958
SRR17520069.7113836
SRR17520069.7113839
SRR17520069.7113841
SRR17520069.7113842
SRR17520069.7113845
SRR17520069.7145714
SRR17520069.7145715
SRR17520069.7145716
SRR17520069.7145717
SRR17520069.7145718
SRR17520069.7145719
SRR17520069.7145720
SRR17520069.7145721
SRR17520069.7145722
SRR17520069.7145723
SRR17520069.7145724
SRR17520069.7145725
SRR17520069.7145726
SRR17520069.7145727
SRR17520069.7145728
SRR17520069.7145729
SRR17520069.7145730
SRR17520069.7145731
SRR17520069.7145732
SRR17520069.7145733
SRR17520069.7145734
SRR17520069.7145735
SRR17520069.7145736
SRR17520069.7145737
SRR17520069.7145738
SRR17520069.7145739
SRR17520069.7145740
SRR17520069.7145741
SRR17520069.7161151
SRR17520069.7161152
SRR17520069.7161153
SRR17520069.7161154

SRR17520069.7161155
SRR17520069.7161156
SRR17520069.7161157
SRR17520069.7161158
SRR17520069.7161159
SRR17520069.7161160
SRR17520069.7161161
SRR17520069.7161162
SRR17520069.7161163
SRR17520069.7161164
SRR17520069.7161165
SRR17520069.7161166
SRR17520069.7161167
SRR17520069.7161168
SRR17520069.7161169
SRR17520069.7161170
SRR17520069.7161171
SRR17520069.7161172
SRR17520069.7161173
SRR17520069.7161174
SRR17520069.7161175
SRR17520069.7161176
SRR17520069.7161177
SRR17520069.7161178
SRR17520069.7161179
SRR17520069.7161180
SRR17520069.7161181
SRR17520069.7161182
SRR17520069.7161183
SRR17520069.7161184
SRR17520069.7161185
SRR17520069.7161186
SRR17520069.7161187
SRR17520069.7161188
SRR17520069.7161189
SRR17520069.7161190
SRR17520069.7161191
SRR17520069.7161192
SRR17520069.7161193
SRR17520069.7161194
SRR17520069.7161195
SRR17520069.7161196
SRR17520069.7161197
SRR17520069.7161198
SRR17520069.7161199
SRR17520069.7161200
SRR17520069.7161202

SRR17520069.7161203
SRR17520069.7161204
SRR17520069.7161205
SRR17520069.7161206
SRR17520069.7161207
SRR17520069.7161208
SRR17520069.7161209
SRR17520069.7161210
SRR17520069.7161211
SRR17520069.7161212
SRR17520069.7161213
SRR17520069.7161214
SRR17520069.7161215
SRR17520069.7161216
SRR17520069.7161217
SRR17520069.7161218
SRR17520069.7161219
SRR17520069.7161220
SRR17520069.7161221
SRR17520069.7166657
SRR17520069.7166658
SRR17520069.7166659
SRR17520069.7166660
SRR17520069.7166661
SRR17520069.7166662
SRR17520069.7166663
SRR17520069.7166664
SRR17520069.7166665
SRR17520069.7166666
SRR17520069.7166667
SRR17520069.7166668
SRR17520069.7166669
SRR17520069.7166670
SRR17520069.7166671
SRR17520069.7166672
SRR17520069.7166673
SRR17520069.7166674
SRR17520069.7166675
SRR17520069.7166676
SRR17520069.7166677
SRR17520069.7166678
SRR17520069.7166679
SRR17520069.7166680
SRR17520069.7166681
SRR17520069.7166682
SRR17520069.7166683
SRR17520069.7166684

SRR17520069.7166685
SRR17520069.7166686
SRR17520069.7166687
SRR17520069.7166688
SRR17520069.7166689
SRR17520069.7166690
SRR17520069.7166691
SRR17520069.7166692
SRR17520069.7166693
SRR17520069.7166694
SRR17520069.7166695
SRR17520069.7166696
SRR17520069.7166697
SRR17520069.7166698
SRR17520069.7166699
SRR17520069.7166702
SRR17520069.7166703
SRR17520069.7166704
SRR17520069.7166705
SRR17520069.7166706
SRR17520069.7166707
SRR17520069.7166708
SRR17520069.7166709
SRR17520069.7166710
SRR17520069.7166711
SRR17520069.7166712
SRR17520069.7166713
SRR17520069.7166714
SRR17520069.7166715
SRR17520069.7166716
SRR17520069.7166717
SRR17520069.7166718
SRR17520069.7166719
SRR17520069.7166720
SRR17520069.7166721
SRR17520069.7166722
SRR17520069.7166723
SRR17520069.7166724
SRR17520069.7166725
SRR17520069.7166726
SRR17520069.7166727
SRR17520069.7166728
SRR17520069.7178915
SRR17520069.7185010
SRR17520069.7191352
SRR17520069.7191353
SRR17520069.7191354

SRR17520069.7191355
SRR17520069.7197044
SRR17520069.7197045
SRR17520069.7197046
SRR17520069.7197047
SRR17520069.7197048
SRR17520069.7197049
SRR17520069.7197050
SRR17520069.7197052
SRR17520069.7197053
SRR17520069.7197054
SRR17520069.7197055
SRR17520069.7197056
SRR17520069.7197057
SRR17520069.7197058
SRR17520069.7197059
SRR17520069.7197060
SRR17520069.7197061
SRR17520069.7197062
SRR17520069.7197063
SRR17520069.7197064
SRR17520069.7197065
SRR17520069.7197066
SRR17520069.7197067
SRR17520069.7197068
SRR17520069.7197069
SRR17520069.7197070
SRR17520069.7197071
SRR17520069.7197072
SRR17520069.7197073
SRR17520069.7197074
SRR17520069.7197075
SRR17520069.7197076
SRR17520069.7197077
SRR17520069.7197078
SRR17520069.7197079
SRR17520069.7197080
SRR17520069.7197081
SRR17520069.7197082
SRR17520069.7197083
SRR17520069.7197084
SRR17520069.7197085
SRR17520069.7197086
SRR17520069.7197087
SRR17520069.7197088
SRR17520069.7197089
SRR17520069.7197090

SRR17520069.7197091
SRR17520069.7197092
SRR17520069.7197093
SRR17520069.7197094
SRR17520069.7197095
SRR17520069.7198580
SRR17520069.7198581
SRR17520069.7198582
SRR17520069.7198583
SRR17520069.7198584
SRR17520069.7198585
SRR17520069.7198586
SRR17520069.7198587
SRR17520069.7198588
SRR17520069.7198589
SRR17520069.7198591
SRR17520069.7198592
SRR17520069.7198593
SRR17520069.7198594
SRR17520069.7198596
SRR17520069.7198597
SRR17520069.7198598
SRR17520069.7198599
SRR17520069.7198600
SRR17520069.7198601
SRR17520069.7198602
SRR17520069.7198603
SRR17520069.7198604
SRR17520069.7198605
SRR17520069.7198606
SRR17520069.7198607
SRR17520069.7198608
SRR17520069.7198609
SRR17520069.7198610
SRR17520069.7198611
SRR17520069.7198612
SRR17520069.7198613
SRR17520069.7198614
SRR17520069.7198615
SRR17520069.7198616
SRR17520069.7198617
SRR17520069.7198619
SRR17520069.7198620
SRR17520069.7198621
SRR17520069.7198622
SRR17520069.7198623
SRR17520069.7198624

SRR17520069.7198625
SRR17520069.7198626
SRR17520069.7198627
SRR17520069.7198628
SRR17520069.7198629
SRR17520069.7198630
SRR17520069.7198631
SRR17520069.7198632
SRR17520069.7198633
SRR17520069.7198634
SRR17520069.7198635
SRR17520069.7198636
SRR17520069.7198637
SRR17520069.7198638
SRR17520069.7198639
SRR17520069.7198640
SRR17520069.7198641
SRR17520069.7198642
SRR17520069.7198643
SRR17520069.7198644
SRR17520069.7198645
SRR17520069.7198646
SRR17520069.7198647
SRR17520069.7198648
SRR17520069.7198649
SRR17520069.7198650
SRR17520069.7198651
SRR17520069.7198652
SRR17520069.7198653
SRR17520069.7198654
SRR17520069.7198655
SRR17520069.7202408
SRR17520069.7224656
SRR17520069.7224657
SRR17520069.7224658
SRR17520069.7224659
SRR17520069.7224660
SRR17520069.7224661
SRR17520069.7224662
SRR17520069.7224663
SRR17520069.7224664
SRR17520069.7224665
SRR17520069.7224666
SRR17520069.7224667
SRR17520069.7224668
SRR17520069.7224669
SRR17520069.7224670

SRR17520069.7224671
SRR17520069.7224672
SRR17520069.7224673
SRR17520069.7224674
SRR17520069.7224675
SRR17520069.7224676
SRR17520069.7224677
SRR17520069.7224678
SRR17520069.7224679
SRR17520069.7224680
SRR17520069.7224681
SRR17520069.7224682
SRR17520069.7224683
SRR17520069.7224684
SRR17520069.7224685
SRR17520069.7224686
SRR17520069.7224687
SRR17520069.7224688
SRR17520069.7224689
SRR17520069.7224690
SRR17520069.7224691
SRR17520069.7224692
SRR17520069.7224693
SRR17520069.7224694
SRR17520069.7224695
SRR17520069.7224696
SRR17520069.7224697
SRR17520069.7224698
SRR17520069.7224699
SRR17520069.7224700
SRR17520069.7224703
SRR17520069.7224704
SRR17520069.7224705
SRR17520069.7224706
SRR17520069.7224707
SRR17520069.7224708
SRR17520069.7224709
SRR17520069.7224710
SRR17520069.7224711
SRR17520069.7224712
SRR17520069.7224713
SRR17520069.7224714
SRR17520069.7224715
SRR17520069.7224716
SRR17520069.7224717
SRR17520069.7224718
SRR17520069.7224719

SRR17520069.7224720
SRR17520069.7224721
SRR17520069.7224722
SRR17520069.7224723
SRR17520069.7224725
SRR17520069.7224726
SRR17520069.7224727
SRR17520069.7224728
SRR17520069.7224729
SRR17520069.7224730
SRR17520069.7224731
SRR17520069.7224732
SRR17520069.7224733
SRR17520069.7224734
SRR17520069.7224735
SRR17520069.7224736
SRR17520069.7224737
SRR17520069.7224738
SRR17520069.7224739
SRR17520069.7224740
SRR17520069.7224741
SRR17520069.7224742
SRR17520069.7224743
SRR17520069.7224744
SRR17520069.7224745
SRR17520069.7224746
SRR17520069.7224748
SRR17520069.7224749
SRR17520069.7224750
SRR17520069.7224753
SRR17520069.7224754
SRR17520069.7224755
SRR17520069.7224756
SRR17520069.7224757
SRR17520069.7224758
SRR17520069.7224759
SRR17520069.7224760
SRR17520069.7224762
SRR17520069.7224763
SRR17520069.7224764
SRR17520069.7224765
SRR17520069.7227996
SRR17520069.7227997
SRR17520069.7227998
SRR17520069.7227999
SRR17520069.7228000
SRR17520069.7228001

SRR17520069.7228002
SRR17520069.7228003
SRR17520069.7228004
SRR17520069.7228005
SRR17520069.7228006
SRR17520069.7228007
SRR17520069.7228008
SRR17520069.7228009
SRR17520069.7228010
SRR17520069.7228011
SRR17520069.7228012
SRR17520069.7228013
SRR17520069.7228014
SRR17520069.7228015
SRR17520069.7228016
SRR17520069.7228018
SRR17520069.7228019
SRR17520069.7228020
SRR17520069.7228021
SRR17520069.7228022
SRR17520069.7228023
SRR17520069.7228024
SRR17520069.7228025
SRR17520069.7228026
SRR17520069.7228027
SRR17520069.7228028
SRR17520069.7228029
SRR17520069.7231843
SRR17520069.7231844
SRR17520069.7231845
SRR17520069.7231846
SRR17520069.7231847
SRR17520069.7249763
SRR17520069.7249764
SRR17520069.7249765
SRR17520069.7249766
SRR17520069.7249767
SRR17520069.7249768
SRR17520069.7249769
SRR17520069.7249770
SRR17520069.7249772
SRR17520069.7251358
SRR17520069.7251359
SRR17520069.7251360
SRR17520069.7251361
SRR17520069.7251362
SRR17520069.7251364

SRR17520069.7251365
SRR17520069.7251366
SRR17520069.7251367
SRR17520069.7251368
SRR17520069.7251370
SRR17520069.7251371
SRR17520069.7251372
SRR17520069.7251373
SRR17520069.7251374
SRR17520069.7251375
SRR17520069.7251378
SRR17520069.7251380
SRR17520069.7251381
SRR17520069.7251383
SRR17520069.7251384
SRR17520069.7251385
SRR17520069.7251386
SRR17520069.7251387
SRR17520069.7251388
SRR17520069.7251389
SRR17520069.7251390
SRR17520069.7251391
SRR17520069.7251393
SRR17520069.7251394
SRR17520069.7251395
SRR17520069.7251396
SRR17520069.7251397
SRR17520069.7251398
SRR17520069.7251399
SRR17520069.7251400
SRR17520069.7251401
SRR17520069.7251402
SRR17520069.7251403
SRR17520069.7251404
SRR17520069.7251405
SRR17520069.7251406
SRR17520069.7251407
SRR17520069.7251408
SRR17520069.7251409
SRR17520069.7251410
SRR17520069.7251411
SRR17520069.7251412
SRR17520069.7251413
SRR17520069.7251414
SRR17520069.7251415
SRR17520069.7251416
SRR17520069.7251417

SRR17520069.7251418
SRR17520069.7251419
SRR17520069.7251420
SRR17520069.7251422
SRR17520069.7251423
SRR17520069.7251424
SRR17520069.7251425
SRR17520069.7251426
SRR17520069.7251427
SRR17520069.7251428
SRR17520069.7251429
SRR17520069.7251430
SRR17520069.7251431
SRR17520069.7251432
SRR17520069.7251433
SRR17520069.7251435
SRR17520069.7251436
SRR17520069.7251437
SRR17520069.7251439
SRR17520069.7251440
SRR17520069.7251441
SRR17520069.7251442
SRR17520069.7251443
SRR17520069.7251444
SRR17520069.7251445
SRR17520069.7251446
SRR17520069.7251447
SRR17520069.7251448
SRR17520069.7253813
SRR17520069.7258230
SRR17520069.7258231
SRR17520069.7258232
SRR17520069.7258233
SRR17520069.7258234
SRR17520069.7258235
SRR17520069.7258236
SRR17520069.7258237
SRR17520069.7258238
SRR17520069.7258239
SRR17520069.7258240
SRR17520069.7258241
SRR17520069.7258242
SRR17520069.7258243
SRR17520069.7258244
SRR17520069.7258245
SRR17520069.7258247
SRR17520069.7258248

SRR17520069.7258249
SRR17520069.7258250
SRR17520069.7258251
SRR17520069.7258252
SRR17520069.7258253
SRR17520069.7258254
SRR17520069.7284366
SRR17520069.7285803
SRR17520069.7285820
SRR17520069.7297131
SRR17520069.7311088
SRR17520069.7327092
SRR17520069.7327093
SRR17520069.7327094
SRR17520069.7327095
SRR17520069.7327096
SRR17520069.7327097
SRR17520069.7327098
SRR17520069.7327099
SRR17520069.7327100
SRR17520069.7327101
SRR17520069.7327102
SRR17520069.7327103
SRR17520069.7327104
SRR17520069.7327105
SRR17520069.7327106
SRR17520069.7327107
SRR17520069.7327108
SRR17520069.7327109
SRR17520069.7327110
SRR17520069.7327111
SRR17520069.7327112
SRR17520069.7327113
SRR17520069.7327114
SRR17520069.7327115
SRR17520069.7327116
SRR17520069.7327117
SRR17520069.7327118
SRR17520069.7327119
SRR17520069.7327120
SRR17520069.7327121
SRR17520069.7327122
SRR17520069.7327123
SRR17520069.7327124
SRR17520069.7327125
SRR17520069.7327127
SRR17520069.7327128

SRR17520069.7327129
SRR17520069.7327130
SRR17520069.7327131
SRR17520069.7327132
SRR17520069.7327133
SRR17520069.7327134
SRR17520069.7327135
SRR17520069.7327136
SRR17520069.7327137
SRR17520069.7327138
SRR17520069.7327139
SRR17520069.7327141
SRR17520069.7327142
SRR17520069.7327143
SRR17520069.7327144
SRR17520069.7327145
SRR17520069.7327146
SRR17520069.7327147
SRR17520069.7327148
SRR17520069.7327149
SRR17520069.7327737
SRR17520069.7329034
SRR17520069.7329035
SRR17520069.7329036
SRR17520069.7329039
SRR17520069.7329040
SRR17520069.7329041
SRR17520069.7329042
SRR17520069.7329043
SRR17520069.7329044
SRR17520069.7329045
SRR17520069.7329046
SRR17520069.7329047
SRR17520069.7329048
SRR17520069.7329049
SRR17520069.7329050
SRR17520069.7329051
SRR17520069.7329052
SRR17520069.7329053
SRR17520069.7329054
SRR17520069.7329055
SRR17520069.7329056
SRR17520069.7329057
SRR17520069.7329058
SRR17520069.7329059
SRR17520069.7329060
SRR17520069.7329061

SRR17520069.7329062
SRR17520069.7329063
SRR17520069.7329065
SRR17520069.7329066
SRR17520069.7329067
SRR17520069.7329068
SRR17520069.7329069
SRR17520069.7329070
SRR17520069.7329071
SRR17520069.7329072
SRR17520069.7329073
SRR17520069.7329074
SRR17520069.7329075
SRR17520069.7329076
SRR17520069.7329077
SRR17520069.7329078
SRR17520069.7329079
SRR17520069.7329080
SRR17520069.7329081
SRR17520069.7329082
SRR17520069.7329083
SRR17520069.7329084
SRR17520069.7329085
SRR17520069.7329086
SRR17520069.7329087
SRR17520069.7329088
SRR17520069.7329089
SRR17520069.7329090
SRR17520069.7329091
SRR17520069.7329092
SRR17520069.7329093
SRR17520069.7329094
SRR17520069.7329095
SRR17520069.7329096
SRR17520069.7329097
SRR17520069.7329098
SRR17520069.7329099
SRR17520069.7329100
SRR17520069.7329101
SRR17520069.7329102
SRR17520069.7329103
SRR17520069.7329104
SRR17520069.7329105
SRR17520069.7329106
SRR17520069.7329107
SRR17520069.7329108
SRR17520069.7345450

SRR17520069.7345466
SRR17520069.7349697
SRR17520069.7349698
SRR17520069.7349699
SRR17520069.7349700
SRR17520069.7349701
SRR17520069.7349702
SRR17520069.7349703
SRR17520069.7349704
SRR17520069.7349705
SRR17520069.7349706
SRR17520069.7349707
SRR17520069.7349708
SRR17520069.7349709
SRR17520069.7349710
SRR17520069.7349711
SRR17520069.7349712
SRR17520069.7349713
SRR17520069.7349714
SRR17520069.7349715
SRR17520069.7349716
SRR17520069.7349717
SRR17520069.7349718
SRR17520069.7349719
SRR17520069.7349720
SRR17520069.7349721
SRR17520069.7349722
SRR17520069.7349723
SRR17520069.7349724
SRR17520069.7349725
SRR17520069.7349726
SRR17520069.7349727
SRR17520069.7349728
SRR17520069.7349729
SRR17520069.7349730
SRR17520069.7349731
SRR17520069.7349732
SRR17520069.7349733
SRR17520069.7349734
SRR17520069.7349735
SRR17520069.7349736
SRR17520069.7349737
SRR17520069.7349738
SRR17520069.7349739
SRR17520069.7349740
SRR17520069.7349741
SRR17520069.7349742

SRR17520069.7349743
SRR17520069.7349744
SRR17520069.7349745
SRR17520069.7349746
SRR17520069.7349747
SRR17520069.7349748
SRR17520069.7349749
SRR17520069.7349750
SRR17520069.7349751
SRR17520069.7349752
SRR17520069.7349753
SRR17520069.7349754
SRR17520069.7349755
SRR17520069.7349756
SRR17520069.7349757
SRR17520069.7349758
SRR17520069.7349759
SRR17520069.7349760
SRR17520069.7349761
SRR17520069.7349762
SRR17520069.7349763
SRR17520069.7349764
SRR17520069.7349765
SRR17520069.7349766
SRR17520069.7349767
SRR17520069.7349768
SRR17520069.7349769
SRR17520069.7349770
SRR17520069.7349771
SRR17520069.7349772
SRR17520069.7349773
SRR17520069.7349774
SRR17520069.7349775
SRR17520069.7349776
SRR17520069.7349777
SRR17520069.7349778
SRR17520069.7349779
SRR17520069.7349780
SRR17520069.7349781
SRR17520069.7349782
SRR17520069.7349783
SRR17520069.7349784
SRR17520069.7349785
SRR17520069.7349786
SRR17520069.7349787
SRR17520069.7349788
SRR17520069.7349789

SRR17520069.7349790
SRR17520069.7349791
SRR17520069.7349792
SRR17520069.7349793
SRR17520069.7349794
SRR17520069.7349795
SRR17520069.7349796
SRR17520069.7349797
SRR17520069.7349798
SRR17520069.7349799
SRR17520069.7349800
SRR17520069.7353543
SRR17520069.7353544
SRR17520069.7353545
SRR17520069.7353546
SRR17520069.7353547
SRR17520069.7353548
SRR17520069.7353549
SRR17520069.7353550
SRR17520069.7353551
SRR17520069.7353552
SRR17520069.7353553
SRR17520069.7353554
SRR17520069.7353555
SRR17520069.7353556
SRR17520069.7353557
SRR17520069.7353558
SRR17520069.7353559
SRR17520069.7353560
SRR17520069.7353561
SRR17520069.7353563
SRR17520069.7353564
SRR17520069.7353565
SRR17520069.7353566
SRR17520069.7353567
SRR17520069.7353568
SRR17520069.7353569
SRR17520069.7353570
SRR17520069.7353571
SRR17520069.7387947
SRR17520069.7387948
SRR17520069.7387949
SRR17520069.7387950
SRR17520069.7387951
SRR17520069.7387954
SRR17520069.7387956
SRR17520069.7387957

SRR17520069.7387958
SRR17520069.7387959
SRR17520069.7387961
SRR17520069.7387962
SRR17520069.7387963
SRR17520069.7387964
SRR17520069.7387966
SRR17520069.7387969
SRR17520069.7387971
SRR17520069.7387972
SRR17520069.7387973
SRR17520069.7387974
SRR17520069.7387975
SRR17520069.7387976
SRR17520069.7387977
SRR17520069.7387978
SRR17520069.7387980
SRR17520069.7387981
SRR17520069.7387982
SRR17520069.7387983
SRR17520069.7387984
SRR17520069.7387985
SRR17520069.7387986
SRR17520069.7387987
SRR17520069.7387988
SRR17520069.7387989
SRR17520069.7387990
SRR17520069.7387991
SRR17520069.7387992
SRR17520069.7387993
SRR17520069.7387994
SRR17520069.7387995
SRR17520069.7387996
SRR17520069.7387997
SRR17520069.7387998
SRR17520069.7387999
SRR17520069.7388000
SRR17520069.7388001
SRR17520069.7388002
SRR17520069.7388003
SRR17520069.7388004
SRR17520069.7388005
SRR17520069.7388006
SRR17520069.7388007
SRR17520069.7388008
SRR17520069.7388009
SRR17520069.7388010

SRR17520069.7388011
SRR17520069.7388013
SRR17520069.7388014
SRR17520069.7388015
SRR17520069.7388016
SRR17520069.7388017
SRR17520069.7388018
SRR17520069.7388019
SRR17520069.7388020
SRR17520069.7388021
SRR17520069.7388022
SRR17520069.7388023
SRR17520069.7388025
SRR17520069.7388026
SRR17520069.7388027
SRR17520069.7388028
SRR17520069.7388029
SRR17520069.7388030
SRR17520069.7388031
SRR17520069.7388032
SRR17520069.7388033
SRR17520069.7388034
SRR17520069.7388036
SRR17520069.7388037
SRR17520069.7388038
SRR17520069.7388040
SRR17520069.7388041
SRR17520069.7388042
SRR17520069.7388044
SRR17520069.7388045
SRR17520069.7388046
SRR17520069.7388047
SRR17520069.7388048
SRR17520069.7388049
SRR17520069.7388050
SRR17520069.7388051
SRR17520069.7388053
SRR17520069.7388055
SRR17520069.7388056
SRR17520069.7388057
SRR17520069.7388058
SRR17520069.7388059
SRR17520069.7388060
SRR17520069.7388061
SRR17520069.7388062
SRR17520069.7388063
SRR17520069.7388064

SRR17520069.7388065
SRR17520069.7388066
SRR17520069.7388067
SRR17520069.7388069
SRR17520069.7388070
SRR17520069.7388071
SRR17520069.7388072
SRR17520069.7388073
SRR17520069.7388074
SRR17520069.7388075
SRR17520069.7388076
SRR17520069.7388077
SRR17520069.7388078
SRR17520069.7388079
SRR17520069.7388080
SRR17520069.7388082
SRR17520069.7388083
SRR17520069.7388085
SRR17520069.7388087
SRR17520069.7388088
SRR17520069.7388091
SRR17520069.7388092
SRR17520069.7388093
SRR17520069.7388094
SRR17520069.7388095
SRR17520069.7389643
SRR17520069.7395441
SRR17520069.7401819
SRR17520069.7452388
SRR17520069.7452389
SRR17520069.7452390
SRR17520069.7452391
SRR17520069.7452392
SRR17520069.7452393
SRR17520069.7452394
SRR17520069.7452395
SRR17520069.7452396
SRR17520069.7452397
SRR17520069.7452398
SRR17520069.7476497
SRR17520069.7479490
SRR17520069.7479694
SRR17520069.7488857
SRR17520069.7488858
SRR17520069.7488859
SRR17520069.7488860
SRR17520069.7488861

SRR17520069.7488862
SRR17520069.7488863
SRR17520069.7488864
SRR17520069.7488865
SRR17520069.7488866
SRR17520069.7488867
SRR17520069.7488868
SRR17520069.7488869
SRR17520069.7488870
SRR17520069.7488872
SRR17520069.7488873
SRR17520069.7488874
SRR17520069.7488875
SRR17520069.7488876
SRR17520069.7488877
SRR17520069.7488878
SRR17520069.7488879
SRR17520069.7488880
SRR17520069.7488881
SRR17520069.7488882
SRR17520069.7488883
SRR17520069.7488884
SRR17520069.7488885
SRR17520069.7488886
SRR17520069.7488887
SRR17520069.7488888
SRR17520069.7488889
SRR17520069.7488890
SRR17520069.7488891
SRR17520069.7488892
SRR17520069.7488893
SRR17520069.7488894
SRR17520069.7488895
SRR17520069.7488896
SRR17520069.7488897
SRR17520069.7488898
SRR17520069.7488899
SRR17520069.7488900
SRR17520069.7488901
SRR17520069.7488902
SRR17520069.7488903
SRR17520069.7488904
SRR17520069.7488905
SRR17520069.7488906
SRR17520069.7488907
SRR17520069.7488908
SRR17520069.7488909

SRR17520069.7488910
SRR17520069.7488911
SRR17520069.7488912
SRR17520069.7488913
SRR17520069.7488914
SRR17520069.7488915
SRR17520069.7488916
SRR17520069.7488917
SRR17520069.7488918
SRR17520069.7488919
SRR17520069.7488920
SRR17520069.7488921
SRR17520069.7488923
SRR17520069.7488924
SRR17520069.7488925
SRR17520069.7488926
SRR17520069.7488927
SRR17520069.7488928
SRR17520069.7488929
SRR17520069.7488930
SRR17520069.7488931
SRR17520069.7488932
SRR17520069.7488933
SRR17520069.7488934
SRR17520069.7488935
SRR17520069.7488936
SRR17520069.7488937
SRR17520069.7488938
SRR17520069.7488939
SRR17520069.7488940
SRR17520069.7488941
SRR17520069.7488942
SRR17520069.7488943
SRR17520069.7488944
SRR17520069.7488945
SRR17520069.7488946
SRR17520069.7488947
SRR17520069.7488948
SRR17520069.7488949
SRR17520069.7488950
SRR17520069.7488951
SRR17520069.7488952
SRR17520069.7488953
SRR17520069.7488954
SRR17520069.7488955
SRR17520069.7488956
SRR17520069.7488957

SRR17520069.7488958
SRR17520069.7488959
SRR17520069.7488960
SRR17520069.7488961
SRR17520069.7488962
SRR17520069.7488963
SRR17520069.7488964
SRR17520069.7488965
SRR17520069.7488966
SRR17520069.7488967
SRR17520069.7488968
SRR17520069.7488969
SRR17520069.7488970
SRR17520069.7488971
SRR17520069.7488973
SRR17520069.7488974
SRR17520069.7488975
SRR17520069.7488976
SRR17520069.7488977
SRR17520069.7488978
SRR17520069.7488979
SRR17520069.7488980
SRR17520069.7488981
SRR17520069.7488982
SRR17520069.7488983
SRR17520069.7488984
SRR17520069.7488985
SRR17520069.7488986
SRR17520069.7488987
SRR17520069.7488988
SRR17520069.7488989
SRR17520069.7488990
SRR17520069.7491636
SRR17520069.7491637
SRR17520069.7491638
SRR17520069.7491639
SRR17520069.7491640
SRR17520069.7491641
SRR17520069.7491642
SRR17520069.7512683
SRR17520069.7515187
SRR17520069.7515188
SRR17520069.7515189
SRR17520069.7515190
SRR17520069.7515191
SRR17520069.7515192
SRR17520069.7515193

SRR17520069.7515194
SRR17520069.7515195
SRR17520069.7515196
SRR17520069.7515197
SRR17520069.7515198
SRR17520069.7515199
SRR17520069.7515200
SRR17520069.7515201
SRR17520069.7515202
SRR17520069.7515203
SRR17520069.7515204
SRR17520069.7515205
SRR17520069.7515206
SRR17520069.7515207
SRR17520069.7515208
SRR17520069.7515209
SRR17520069.7515210
SRR17520069.7515211
SRR17520069.7515212
SRR17520069.7515214
SRR17520069.7515215
SRR17520069.7515216
SRR17520069.7515217
SRR17520069.7515218
SRR17520069.7515219
SRR17520069.7515220
SRR17520069.7515221
SRR17520069.7515222
SRR17520069.7515223
SRR17520069.7515224
SRR17520069.7533314
SRR17520069.7533315
SRR17520069.7533317
SRR17520069.7533318
SRR17520069.7533319
SRR17520069.7533320
SRR17520069.7533321
SRR17520069.7533322
SRR17520069.7533323
SRR17520069.7533324
SRR17520069.7533325
SRR17520069.7533326
SRR17520069.7533327
SRR17520069.7533328
SRR17520069.7533329
SRR17520069.7533330
SRR17520069.7533331

SRR17520069.7533332
SRR17520069.7533333
SRR17520069.7533334
SRR17520069.7533335
SRR17520069.7533336
SRR17520069.7533337
SRR17520069.7545422
SRR17520069.7545434
SRR17520069.7545438
SRR17520069.7545512
SRR17520069.7559738
SRR17520069.7559739
SRR17520069.7559740
SRR17520069.7559741
SRR17520069.7559742
SRR17520069.7559743
SRR17520069.7559744
SRR17520069.7559745
SRR17520069.7559746
SRR17520069.7559747
SRR17520069.7559748
SRR17520069.7559749
SRR17520069.7559750
SRR17520069.7559751
SRR17520069.7559752
SRR17520069.7559753
SRR17520069.7559754
SRR17520069.7559755
SRR17520069.7559756
SRR17520069.7559757
SRR17520069.7559758
SRR17520069.7559759
SRR17520069.7559760
SRR17520069.7559761
SRR17520069.7559762
SRR17520069.7559763
SRR17520069.7559764
SRR17520069.7559765
SRR17520069.7559766
SRR17520069.7559767
SRR17520069.7559769
SRR17520069.7559770
SRR17520069.7559772
SRR17520069.7559773
SRR17520069.7613041
SRR17520069.7613093
SRR17520069.7617227

SRR17520069.7617228
SRR17520069.7617229
SRR17520069.7617230
SRR17520069.7617231
SRR17520069.7617232
SRR17520069.7617233
SRR17520069.7617234
SRR17520069.7617235
SRR17520069.7617236
SRR17520069.7617237
SRR17520069.7617238
SRR17520069.7617239
SRR17520069.7617240
SRR17520069.7617241
SRR17520069.7617242
SRR17520069.7617243
SRR17520069.7617244
SRR17520069.7617245
SRR17520069.7617246
SRR17520069.7617247
SRR17520069.7617248
SRR17520069.7617249
SRR17520069.7617250
SRR17520069.7617251
SRR17520069.7617252
SRR17520069.7617253
SRR17520069.7617254
SRR17520069.7617255
SRR17520069.7617256
SRR17520069.7617257
SRR17520069.7617258
SRR17520069.7617259
SRR17520069.7617260
SRR17520069.7617261
SRR17520069.7617262
SRR17520069.7617263
SRR17520069.7619474
SRR17520069.7637121
SRR17520069.7642590
SRR17520069.7642607
SRR17520069.7644212
SRR17520069.7672181
SRR17520069.7679144
SRR17520069.7681950
SRR17520069.7681951
SRR17520069.7681952
SRR17520069.7681953

SRR17520069.7681954
SRR17520069.7681955
SRR17520069.7681956
SRR17520069.7681957
SRR17520069.7681958
SRR17520069.7681959
SRR17520069.7681960
SRR17520069.7681961
SRR17520069.7681962
SRR17520069.7681963
SRR17520069.7681964
SRR17520069.7681965
SRR17520069.7681966
SRR17520069.7681967
SRR17520069.7681968
SRR17520069.7681969
SRR17520069.7681970
SRR17520069.7681971
SRR17520069.7681972
SRR17520069.7681973
SRR17520069.7681974
SRR17520069.7681975
SRR17520069.7681976
SRR17520069.7681977
SRR17520069.7681978
SRR17520069.7681979
SRR17520069.7681980
SRR17520069.7681981
SRR17520069.7681982
SRR17520069.7681983
SRR17520069.7681984
SRR17520069.7681985
SRR17520069.7681986
SRR17520069.7681987
SRR17520069.7681988
SRR17520069.7681989
SRR17520069.7681990
SRR17520069.7681991
SRR17520069.7681992
SRR17520069.7681993
SRR17520069.7681994
SRR17520069.7681995
SRR17520069.7681996
SRR17520069.7681997
SRR17520069.7681998
SRR17520069.7681999
SRR17520069.7682000

SRR17520069.7682001
SRR17520069.7682002
SRR17520069.7682003
SRR17520069.7682004
SRR17520069.7682005
SRR17520069.7682006
SRR17520069.7682007
SRR17520069.7682008
SRR17520069.7682009
SRR17520069.7682010
SRR17520069.7682011
SRR17520069.7682012
SRR17520069.7682013
SRR17520069.7682014
SRR17520069.7682015
SRR17520069.7682016
SRR17520069.7682017
SRR17520069.7682018
SRR17520069.7682019
SRR17520069.7682020
SRR17520069.7682021
SRR17520069.7682022
SRR17520069.7682023
SRR17520069.7682024
SRR17520069.7682025
SRR17520069.7682026
SRR17520069.7682027
SRR17520069.7682028
SRR17520069.7682029
SRR17520069.7682030
SRR17520069.7682031
SRR17520069.7682032
SRR17520069.7682033
SRR17520069.7682034
SRR17520069.7682035
SRR17520069.7682036
SRR17520069.7682037
SRR17520069.7682038
SRR17520069.7682039
SRR17520069.7682040
SRR17520069.7682041
SRR17520069.7682042
SRR17520069.7682043
SRR17520069.7682044
SRR17520069.7682045
SRR17520069.7682046
SRR17520069.7682047

SRR17520069.7682048
SRR17520069.7682049
SRR17520069.7682050
SRR17520069.7682051
SRR17520069.7682052
SRR17520069.7682054
SRR17520069.7682055
SRR17520069.7682056
SRR17520069.7682057
SRR17520069.7682058
SRR17520069.7682059
SRR17520069.7682060
SRR17520069.7682061
SRR17520069.7682062
SRR17520069.7682063
SRR17520069.7682064
SRR17520069.7682065
SRR17520069.7682066
SRR17520069.7682067
SRR17520069.7682068
SRR17520069.7682069
SRR17520069.7682070
SRR17520069.7682071
SRR17520069.7682072
SRR17520069.7682073
SRR17520069.7682074
SRR17520069.7682075
SRR17520069.7682076
SRR17520069.7682077
SRR17520069.7682078
SRR17520069.7682079
SRR17520069.7682080
SRR17520069.7682081
SRR17520069.7682082
SRR17520069.7682083
SRR17520069.7682084
SRR17520069.7682085
SRR17520069.7682086
SRR17520069.7682087
SRR17520069.7682088
SRR17520069.7682089
SRR17520069.7682090
SRR17520069.7682091
SRR17520069.7682092
SRR17520069.7682093
SRR17520069.7682094
SRR17520069.7682095

SRR17520069.7682096
SRR17520069.7682097
SRR17520069.7682098
SRR17520069.7682099
SRR17520069.7682100
SRR17520069.7682101
SRR17520069.7682102
SRR17520069.7682103
SRR17520069.7682104
SRR17520069.7682105
SRR17520069.7682106
SRR17520069.7682107
SRR17520069.7682108
SRR17520069.7682109
SRR17520069.7682110
SRR17520069.7682111
SRR17520069.7682112
SRR17520069.7682113
SRR17520069.7688304
SRR17520069.7688305
SRR17520069.7688306
SRR17520069.7688308
SRR17520069.7688309
SRR17520069.7688310
SRR17520069.7688311
SRR17520069.7688313
SRR17520069.7688315
SRR17520069.7688316
SRR17520069.7688317
SRR17520069.7688318
SRR17520069.7688320
SRR17520069.7688321
SRR17520069.7688322
SRR17520069.7694506
SRR17520069.7696434
SRR17520069.7696435
SRR17520069.7696436
SRR17520069.7696437
SRR17520069.7696438
SRR17520069.7696439
SRR17520069.7696440
SRR17520069.7696441
SRR17520069.7696442
SRR17520069.7696443
SRR17520069.7696444
SRR17520069.7696445
SRR17520069.7696446

SRR17520069.7696447
SRR17520069.7696448
SRR17520069.7696449
SRR17520069.7696450
SRR17520069.7696451
SRR17520069.7696452
SRR17520069.7696453
SRR17520069.7696454
SRR17520069.7696455
SRR17520069.7696456
SRR17520069.7696457
SRR17520069.7696458
SRR17520069.7696459
SRR17520069.7696460
SRR17520069.7696461
SRR17520069.7696462
SRR17520069.7696463
SRR17520069.7696464
SRR17520069.7696465
SRR17520069.7696466
SRR17520069.7696467
SRR17520069.7696468
SRR17520069.7696469
SRR17520069.7696470
SRR17520069.7696471
SRR17520069.7696472
SRR17520069.7696473
SRR17520069.7696474
SRR17520069.7696475
SRR17520069.7696476
SRR17520069.7696477
SRR17520069.7696478
SRR17520069.7696479
SRR17520069.7696480
SRR17520069.7696481
SRR17520069.7696482
SRR17520069.7696483
SRR17520069.7696484
SRR17520069.7696485
SRR17520069.7696486
SRR17520069.7696487
SRR17520069.7696488
SRR17520069.7696489
SRR17520069.7696490
SRR17520069.7696491
SRR17520069.7696492
SRR17520069.7696493

SRR17520069.7696494
SRR17520069.7696495
SRR17520069.7696496
SRR17520069.7696497
SRR17520069.7696498
SRR17520069.7696499
SRR17520069.7696500
SRR17520069.7696501
SRR17520069.7696502
SRR17520069.7696503
SRR17520069.7696504
SRR17520069.7696505
SRR17520069.7696506
SRR17520069.7696507
SRR17520069.7696508
SRR17520069.7696509
SRR17520069.7696510
SRR17520069.7696511
SRR17520069.7696512
SRR17520069.7696513
SRR17520069.7696514
SRR17520069.7696515
SRR17520069.7696516
SRR17520069.7696517
SRR17520069.7696518
SRR17520069.7696519
SRR17520069.7696520
SRR17520069.7696521
SRR17520069.7696522
SRR17520069.7696523
SRR17520069.7696524
SRR17520069.7696526
SRR17520069.7696527
SRR17520069.7696528
SRR17520069.7696529
SRR17520069.7696530
SRR17520069.7696531
SRR17520069.7696532
SRR17520069.7696533
SRR17520069.7696534
SRR17520069.7696535
SRR17520069.7696536
SRR17520069.7696537
SRR17520069.7696538
SRR17520069.7696539
SRR17520069.7696540
SRR17520069.7696541

SRR17520069.7696542
SRR17520069.7696543
SRR17520069.7696544
SRR17520069.7696545
SRR17520069.7696546
SRR17520069.7697659
SRR17520069.7703363
SRR17520069.7707598
SRR17520069.7707608
SRR17520069.7707618
SRR17520069.7721679
SRR17520069.7721680
SRR17520069.7721681
SRR17520069.7721682
SRR17520069.7721683
SRR17520069.7721684
SRR17520069.7721685
SRR17520069.7721686
SRR17520069.7721687
SRR17520069.7721688
SRR17520069.7721689
SRR17520069.7721690
SRR17520069.7721691
SRR17520069.7721692
SRR17520069.7721693
SRR17520069.7738693
SRR17520069.7738694
SRR17520069.7738695
SRR17520069.7738696
SRR17520069.7738697
SRR17520069.7738698
SRR17520069.7738699
SRR17520069.7738700
SRR17520069.7738701
SRR17520069.7738702
SRR17520069.7738703
SRR17520069.7738705
SRR17520069.7738706
SRR17520069.7738707
SRR17520069.7738708
SRR17520069.7738709
SRR17520069.7738710
SRR17520069.7738711
SRR17520069.7738712
SRR17520069.7738713
SRR17520069.7738714
SRR17520069.7738715

SRR17520069.7738716
SRR17520069.7738717
SRR17520069.7738718
SRR17520069.7738719
SRR17520069.7738720
SRR17520069.7738721
SRR17520069.7738723
SRR17520069.7738725
SRR17520069.7738726
SRR17520069.7738727
SRR17520069.7738728
SRR17520069.7738729
SRR17520069.7738730
SRR17520069.7778450
SRR17520069.7778451
SRR17520069.7778452
SRR17520069.7778453
SRR17520069.7778455
SRR17520069.7778456
SRR17520069.7778457
SRR17520069.7778458
SRR17520069.7778459
SRR17520069.7778460
SRR17520069.7778461
SRR17520069.7778462
SRR17520069.7778463
SRR17520069.7778464
SRR17520069.7778465
SRR17520069.7778466
SRR17520069.7778467
SRR17520069.7778468
SRR17520069.7778469
SRR17520069.7778470
SRR17520069.7778471
SRR17520069.7778472
SRR17520069.7778473
SRR17520069.7778474
SRR17520069.7778475
SRR17520069.7778476
SRR17520069.7778477
SRR17520069.7778478
SRR17520069.7778479
SRR17520069.7778481
SRR17520069.7778482
SRR17520069.7778483
SRR17520069.7778484
SRR17520069.7778485

SRR17520069.7778486
SRR17520069.7778487
SRR17520069.7778488
SRR17520069.7778489
SRR17520069.7778490
SRR17520069.7778491
SRR17520069.7778492
SRR17520069.7778493
SRR17520069.7778494
SRR17520069.7778495
SRR17520069.7778496
SRR17520069.7778497
SRR17520069.7778498
SRR17520069.7778499
SRR17520069.7778500
SRR17520069.7786999
SRR17520069.7790509
SRR17520069.7790511
SRR17520069.7796733
SRR17520069.7796735
SRR17520069.7796736
SRR17520069.7796737
SRR17520069.7807225
SRR17520069.7807226
SRR17520069.7813679
SRR17520069.7823976
SRR17520069.7823977
SRR17520069.7823978
SRR17520069.7823979
SRR17520069.7823980
SRR17520069.7823981
SRR17520069.7823982
SRR17520069.7823983
SRR17520069.7823984
SRR17520069.7823985
SRR17520069.7823986
SRR17520069.7823987
SRR17520069.7823988
SRR17520069.7823989
SRR17520069.7823990
SRR17520069.7823991
SRR17520069.7823992
SRR17520069.7823993
SRR17520069.7823994
SRR17520069.7823995
SRR17520069.7823996
SRR17520069.7823997

SRR17520069.7823998
SRR17520069.7823999
SRR17520069.7824000
SRR17520069.7824001
SRR17520069.7824002
SRR17520069.7824003
SRR17520069.7824004
SRR17520069.7824005
SRR17520069.7824006
SRR17520069.7824007
SRR17520069.7824008
SRR17520069.7824009
SRR17520069.7824010
SRR17520069.7824011
SRR17520069.7824012
SRR17520069.7824013
SRR17520069.7824014
SRR17520069.7824015
SRR17520069.7824016
SRR17520069.7824017
SRR17520069.7824018
SRR17520069.7824019
SRR17520069.7824020
SRR17520069.7824021
SRR17520069.7824022
SRR17520069.7824023
SRR17520069.7824024
SRR17520069.7824025
SRR17520069.7824026
SRR17520069.7824027
SRR17520069.7824028
SRR17520069.7824029
SRR17520069.7824030
SRR17520069.7824031
SRR17520069.7824032
SRR17520069.7824033
SRR17520069.7824034
SRR17520069.7824035
SRR17520069.7824036
SRR17520069.7824037
SRR17520069.7824038
SRR17520069.7824039
SRR17520069.7824040
SRR17520069.7824041
SRR17520069.7824042
SRR17520069.7824043
SRR17520069.7824044

SRR17520069.7824045
SRR17520069.7824046
SRR17520069.7824047
SRR17520069.7824048
SRR17520069.7824049
SRR17520069.7824050
SRR17520069.7824051
SRR17520069.7824052
SRR17520069.7824053
SRR17520069.7829101
SRR17520069.7829102
SRR17520069.7829103
SRR17520069.7829104
SRR17520069.7829105
SRR17520069.7829106
SRR17520069.7829107
SRR17520069.7829108
SRR17520069.7829109
SRR17520069.7829110
SRR17520069.7829111
SRR17520069.7829112
SRR17520069.7829113
SRR17520069.7829114
SRR17520069.7829115
SRR17520069.7829116
SRR17520069.7829117
SRR17520069.7829118
SRR17520069.7829119
SRR17520069.7829120
SRR17520069.7829121
SRR17520069.7829122
SRR17520069.7829123
SRR17520069.7829124
SRR17520069.7829125
SRR17520069.7829126
SRR17520069.7829127
SRR17520069.7829128
SRR17520069.7829129
SRR17520069.7829130
SRR17520069.7829131
SRR17520069.7829132
SRR17520069.7829133
SRR17520069.7829134
SRR17520069.7829135
SRR17520069.7829136
SRR17520069.7829137
SRR17520069.7829138

SRR17520069.7829139
SRR17520069.7829140
SRR17520069.7829142
SRR17520069.7829143
SRR17520069.7829144
SRR17520069.7829145
SRR17520069.7829146
SRR17520069.7829147
SRR17520069.7829148
SRR17520069.7829149
SRR17520069.7829150
SRR17520069.7829151
SRR17520069.7829153
SRR17520069.7829154
SRR17520069.7829155
SRR17520069.7829156
SRR17520069.7829157
SRR17520069.7829158
SRR17520069.7829159
SRR17520069.7829160
SRR17520069.7829161
SRR17520069.7829162
SRR17520069.7829163
SRR17520069.7829164
SRR17520069.7829165
SRR17520069.7829166
SRR17520069.7829167
SRR17520069.7829168
SRR17520069.7829169
SRR17520069.7829170
SRR17520069.7829171
SRR17520069.7829172
SRR17520069.7829173
SRR17520069.7829174
SRR17520069.7829175
SRR17520069.7829176
SRR17520069.7829177
SRR17520069.7829178
SRR17520069.7829179
SRR17520069.7829180
SRR17520069.7829181
SRR17520069.7829182
SRR17520069.7829185
SRR17520069.7829186
SRR17520069.7829187
SRR17520069.7829188
SRR17520069.7829189

SRR17520069.7829190
SRR17520069.7829191
SRR17520069.7829192
SRR17520069.7829193
SRR17520069.7829194
SRR17520069.7829195
SRR17520069.7829196
SRR17520069.7829197
SRR17520069.7829198
SRR17520069.7829199
SRR17520069.7829200
SRR17520069.7829201
SRR17520069.7829202
SRR17520069.7829203
SRR17520069.7829204
SRR17520069.7829205
SRR17520069.7829206
SRR17520069.7829207
SRR17520069.7829208
SRR17520069.7829209
SRR17520069.7829210
SRR17520069.7829211
SRR17520069.7829212
SRR17520069.7829213
SRR17520069.7829214
SRR17520069.7829215
SRR17520069.7829216
SRR17520069.7857424
SRR17520069.7859839
SRR17520069.7859840
SRR17520069.7859841
SRR17520069.7859842
SRR17520069.7859843
SRR17520069.7859844
SRR17520069.7859845
SRR17520069.7859846
SRR17520069.7859847
SRR17520069.7859848
SRR17520069.7859849
SRR17520069.7859850
SRR17520069.7859852
SRR17520069.7859853
SRR17520069.7859854
SRR17520069.7859855
SRR17520069.7859856
SRR17520069.7859857
SRR17520069.7859858

SRR17520069.7859859
SRR17520069.7859860
SRR17520069.7859861
SRR17520069.7859862
SRR17520069.7859863
SRR17520069.7859864
SRR17520069.7859865
SRR17520069.7859866
SRR17520069.7859867
SRR17520069.7870163
SRR17520069.7889178
SRR17520069.7889264
SRR17520069.7898577
SRR17520069.7898578
SRR17520069.7898579
SRR17520069.7898580
SRR17520069.7898581
SRR17520069.7898582
SRR17520069.7898583
SRR17520069.7898584
SRR17520069.7898585
SRR17520069.7898586
SRR17520069.7898587
SRR17520069.7898588
SRR17520069.7898589
SRR17520069.7898590
SRR17520069.7898591
SRR17520069.7898592
SRR17520069.7898593
SRR17520069.7898594
SRR17520069.7898595
SRR17520069.7898596
SRR17520069.7898597
SRR17520069.7898598
SRR17520069.7898599
SRR17520069.7898600
SRR17520069.7898601
SRR17520069.7898602
SRR17520069.7898603
SRR17520069.7898604
SRR17520069.7898605
SRR17520069.7898606
SRR17520069.7898607
SRR17520069.7898608
SRR17520069.7898610
SRR17520069.7898611
SRR17520069.7898612

SRR17520069.7898613
SRR17520069.7898614
SRR17520069.7898615
SRR17520069.7898616
SRR17520069.7898617
SRR17520069.7898618
SRR17520069.7898619
SRR17520069.7898620
SRR17520069.7898621
SRR17520069.7898623
SRR17520069.7898624
SRR17520069.7898625
SRR17520069.7898626
SRR17520069.7898627
SRR17520069.7898628
SRR17520069.7898629
SRR17520069.7898630
SRR17520069.7898632
SRR17520069.7898636
SRR17520069.7898637
SRR17520069.7898638
SRR17520069.7898639
SRR17520069.7898640
SRR17520069.7898641
SRR17520069.7898642
SRR17520069.7898643
SRR17520069.7898645
SRR17520069.7898647
SRR17520069.7898648
SRR17520069.7898651
SRR17520069.7898652
SRR17520069.7898653
SRR17520069.7898654
SRR17520069.7898655
SRR17520069.7898656
SRR17520069.7898657
SRR17520069.7898661
SRR17520069.7898662
SRR17520069.7898664
SRR17520069.7898665
SRR17520069.7898666
SRR17520069.7898667
SRR17520069.7898668
SRR17520069.7898669
SRR17520069.7898670
SRR17520069.7898671
SRR17520069.7898672

SRR17520069.7906223
SRR17520069.7906224
SRR17520069.7906225
SRR17520069.7906226
SRR17520069.7906227
SRR17520069.7906228
SRR17520069.7906229
SRR17520069.7906230
SRR17520069.7906231
SRR17520069.7906232
SRR17520069.7906233
SRR17520069.7906234
SRR17520069.7906235
SRR17520069.7927233
SRR17520069.7927236
SRR17520069.7927237
SRR17520069.7927238
SRR17520069.7927239
SRR17520069.7927240
SRR17520069.7927241
SRR17520069.7927242
SRR17520069.7927243
SRR17520069.7927244
SRR17520069.7927245
SRR17520069.7927246
SRR17520069.7927248
SRR17520069.7927252
SRR17520069.7927255
SRR17520069.7927256
SRR17520069.7927257
SRR17520069.7927259
SRR17520069.7927263
SRR17520069.7927264
SRR17520069.7927266
SRR17520069.7927267
SRR17520069.7962904
SRR17520069.7962905
SRR17520069.7962906
SRR17520069.7962907
SRR17520069.7962908
SRR17520069.7962909
SRR17520069.7962910
SRR17520069.7962911
SRR17520069.7962912
SRR17520069.7962913
SRR17520069.7962914
SRR17520069.7962915

SRR17520069.7962916
SRR17520069.7962917
SRR17520069.7962918
SRR17520069.7962919
SRR17520069.7962920
SRR17520069.7962921
SRR17520069.7962922
SRR17520069.7962923
SRR17520069.7962924
SRR17520069.7962925
SRR17520069.7962926
SRR17520069.7962927
SRR17520069.7962928
SRR17520069.7962929
SRR17520069.7962930
SRR17520069.7962931
SRR17520069.7962932
SRR17520069.7962933
SRR17520069.7962934
SRR17520069.7962935
SRR17520069.7962936
SRR17520069.7962937
SRR17520069.7962938
SRR17520069.7962939
SRR17520069.7962940
SRR17520069.7962941
SRR17520069.7962942
SRR17520069.7962943
SRR17520069.7962944
SRR17520069.7962945
SRR17520069.7962946
SRR17520069.7962947
SRR17520069.7962948
SRR17520069.7962949
SRR17520069.7962950
SRR17520069.7962951
SRR17520069.7962952
SRR17520069.7962953
SRR17520069.7962954
SRR17520069.7962955
SRR17520069.7962956
SRR17520069.7962957
SRR17520069.7962958
SRR17520069.7962959
SRR17520069.7962960
SRR17520069.7962961
SRR17520069.7962962

SRR17520069.7962963
SRR17520069.7962964
SRR17520069.7962965
SRR17520069.7962966
SRR17520069.7962967
SRR17520069.7962968
SRR17520069.7962969
SRR17520069.7962970
SRR17520069.7962971
SRR17520069.7978233
SRR17520069.7978240
SRR17520069.7978255
SRR17520069.7984327
SRR17520069.8014669
SRR17520069.8014677
SRR17520069.8014751
SRR17520069.8034847
SRR17520069.8034848
SRR17520069.8034849
SRR17520069.8034850
SRR17520069.8034851
SRR17520069.8034852
SRR17520069.8034854
SRR17520069.8034855
SRR17520069.8034856
SRR17520069.8034857
SRR17520069.8034859
SRR17520069.8034861
SRR17520069.8034863
SRR17520069.8034864
SRR17520069.8034865
SRR17520069.8034866
SRR17520069.8034867
SRR17520069.8034868
SRR17520069.8034870
SRR17520069.8034872
SRR17520069.8034875
SRR17520069.8034876
SRR17520069.8034877
SRR17520069.8034878
SRR17520069.8034879
SRR17520069.8063067
SRR17520069.8063103
SRR17520069.8080872
SRR17520069.8080876
SRR17520069.8080879
SRR17520069.8086434

SRR17520069.8086435
SRR17520069.8086436
SRR17520069.8086437
SRR17520069.8086438
SRR17520069.8086439
SRR17520069.8086440
SRR17520069.8086441
SRR17520069.8086442
SRR17520069.8086443
SRR17520069.8086444
SRR17520069.8086445
SRR17520069.8086446
SRR17520069.8086447
SRR17520069.8086448
SRR17520069.8086450
SRR17520069.8086451
SRR17520069.8086453
SRR17520069.8086454
SRR17520069.8086455
SRR17520069.8086456
SRR17520069.8086457
SRR17520069.8086458
SRR17520069.8086459
SRR17520069.8086460
SRR17520069.8086461
SRR17520069.8086463
SRR17520069.8086464
SRR17520069.8086465
SRR17520069.8086466
SRR17520069.8086467
SRR17520069.8086468
SRR17520069.8086469
SRR17520069.8086471
SRR17520069.8086472
SRR17520069.8086473
SRR17520069.8086474
SRR17520069.8086475
SRR17520069.8086477
SRR17520069.8086478
SRR17520069.8086479
SRR17520069.8086480
SRR17520069.8086481
SRR17520069.8086482
SRR17520069.8086483
SRR17520069.8086484
SRR17520069.8086485
SRR17520069.8086486

SRR17520069.8086487
SRR17520069.8086488
SRR17520069.8086489
SRR17520069.8086490
SRR17520069.8086491
SRR17520069.8086492
SRR17520069.8086493
SRR17520069.8086494
SRR17520069.8086495
SRR17520069.8086496
SRR17520069.8086497
SRR17520069.8086498
SRR17520069.8086499
SRR17520069.8106609
SRR17520069.8106610
SRR17520069.8106611
SRR17520069.8106612
SRR17520069.8106613
SRR17520069.8106614
SRR17520069.8106615
SRR17520069.8106616
SRR17520069.8106617
SRR17520069.8106618
SRR17520069.8106619
SRR17520069.8106620
SRR17520069.8106621
SRR17520069.8106623
SRR17520069.8106624
SRR17520069.8106625
SRR17520069.8106626
SRR17520069.8106627
SRR17520069.8107039
SRR17520069.8107040
SRR17520069.8107041
SRR17520069.8107042
SRR17520069.8107043
SRR17520069.8107044
SRR17520069.8107045
SRR17520069.8107046
SRR17520069.8107047
SRR17520069.8107048
SRR17520069.8107049
SRR17520069.8107050
SRR17520069.8107051
SRR17520069.8107052
SRR17520069.8107053
SRR17520069.8107054

SRR17520069.8107055
SRR17520069.8107056
SRR17520069.8107057
SRR17520069.8107058
SRR17520069.8107059
SRR17520069.8107060
SRR17520069.8107061
SRR17520069.8107062
SRR17520069.8107065
SRR17520069.8107066
SRR17520069.8107067
SRR17520069.8107068
SRR17520069.8107069
SRR17520069.8107070
SRR17520069.8107071
SRR17520069.8107072
SRR17520069.8107073
SRR17520069.8107074
SRR17520069.8107075
SRR17520069.8107076
SRR17520069.8107078
SRR17520069.8107079
SRR17520069.8107080
SRR17520069.8107081
SRR17520069.8107082
SRR17520069.8107083
SRR17520069.8107084
SRR17520069.8107085
SRR17520069.8107086
SRR17520069.8107087
SRR17520069.8107088
SRR17520069.8107089
SRR17520069.8107090
SRR17520069.8107091
SRR17520069.8107093
SRR17520069.8107094
SRR17520069.8107095
SRR17520069.8107096
SRR17520069.8107097
SRR17520069.8107098
SRR17520069.8107099
SRR17520069.8107100
SRR17520069.8107101
SRR17520069.8107102
SRR17520069.8107103
SRR17520069.8107104
SRR17520069.8107105

SRR17520069.8107106
SRR17520069.8107107
SRR17520069.8107108
SRR17520069.8107109
SRR17520069.8107110
SRR17520069.8107111
SRR17520069.8107112
SRR17520069.8107113
SRR17520069.8107114
SRR17520069.8107115
SRR17520069.8133570
SRR17520069.8133571
SRR17520069.8133572
SRR17520069.8133573
SRR17520069.8133574
SRR17520069.8133575
SRR17520069.8133576
SRR17520069.8133577
SRR17520069.8133578
SRR17520069.8133579
SRR17520069.8133580
SRR17520069.8133581
SRR17520069.8133582
SRR17520069.8133583
SRR17520069.8133584
SRR17520069.8133585
SRR17520069.8133586
SRR17520069.8136293
SRR17520069.8136294
SRR17520069.8136295
SRR17520069.8136296
SRR17520069.8136297
SRR17520069.8136298
SRR17520069.8136299
SRR17520069.8136300
SRR17520069.8136301
SRR17520069.8136303
SRR17520069.8136304
SRR17520069.8136305
SRR17520069.8136307
SRR17520069.8163552
SRR17520069.8163553
SRR17520069.8163554
SRR17520069.8163555
SRR17520069.8163556
SRR17520069.8163557
SRR17520069.8163558

SRR17520069.8163559
SRR17520069.8163560
SRR17520069.8163561
SRR17520069.8163562
SRR17520069.8163564
SRR17520069.8163565
SRR17520069.8163566
SRR17520069.8163567
SRR17520069.8163568
SRR17520069.8163570
SRR17520069.8163571
SRR17520069.8163572
SRR17520069.8163573
SRR17520069.8163574
SRR17520069.8163576
SRR17520069.8163577
SRR17520069.8163578
SRR17520069.8163579
SRR17520069.8163581
SRR17520069.8163582
SRR17520069.8180213
SRR17520069.8180214
SRR17520069.8180215
SRR17520069.8180216
SRR17520069.8180217
SRR17520069.8180218
SRR17520069.8180219
SRR17520069.8180220
SRR17520069.8180221
SRR17520069.8180222
SRR17520069.8180223
SRR17520069.8180224
SRR17520069.8180225
SRR17520069.8180226
SRR17520069.8180227
SRR17520069.8180228
SRR17520069.8180229
SRR17520069.8180230
SRR17520069.8180231
SRR17520069.8180232
SRR17520069.8180233
SRR17520069.8180234
SRR17520069.8180235
SRR17520069.8180236
SRR17520069.8180237
SRR17520069.8180238
SRR17520069.8180239

SRR17520069.8180240
SRR17520069.8180241
SRR17520069.8180242
SRR17520069.8180243
SRR17520069.8180244
SRR17520069.8180245
SRR17520069.8180246
SRR17520069.8180247
SRR17520069.8180248
SRR17520069.8180249
SRR17520069.8180250
SRR17520069.8180251
SRR17520069.8180252
SRR17520069.8180253
SRR17520069.8180254
SRR17520069.8180255
SRR17520069.8180256
SRR17520069.8180257
SRR17520069.8180258
SRR17520069.8180259
SRR17520069.8180260
SRR17520069.8180261
SRR17520069.8180262
SRR17520069.8180263
SRR17520069.8180264
SRR17520069.8180265
SRR17520069.8180266
SRR17520069.8180267
SRR17520069.8180268
SRR17520069.8180269
SRR17520069.8180270
SRR17520069.8180271
SRR17520069.8180272
SRR17520069.8180273
SRR17520069.8180274
SRR17520069.8180275
SRR17520069.8180276
SRR17520069.8180277
SRR17520069.8180278
SRR17520069.8180279
SRR17520069.8180280
SRR17520069.8180281
SRR17520069.8180282
SRR17520069.8180283
SRR17520069.8180284
SRR17520069.8180285
SRR17520069.8180286

SRR17520069.8180287
SRR17520069.8180288
SRR17520069.8190729
SRR17520069.8192356
SRR17520069.8192357
SRR17520069.8192358
SRR17520069.8192359
SRR17520069.8192360
SRR17520069.8192361
SRR17520069.8192362
SRR17520069.8192363
SRR17520069.8192364
SRR17520069.8208066
SRR17520069.8208067
SRR17520069.8208068
SRR17520069.8208070
SRR17520069.8208071
SRR17520069.8208072
SRR17520069.8208073
SRR17520069.8208074
SRR17520069.8208075
SRR17520069.8208076
SRR17520069.8208077
SRR17520069.8208078
SRR17520069.8208079
SRR17520069.8208080
SRR17520069.8208081
SRR17520069.8208083
SRR17520069.8208084
SRR17520069.8208085
SRR17520069.8208086
SRR17520069.8208087
SRR17520069.8208088
SRR17520069.8208089
SRR17520069.8208090
SRR17520069.8208091
SRR17520069.8208092
SRR17520069.8208093
SRR17520069.8208094
SRR17520069.8208095
SRR17520069.8208096
SRR17520069.8208097
SRR17520069.8208098
SRR17520069.8208099
SRR17520069.8212970
SRR17520069.8212971
SRR17520069.8212973

SRR17520069.8212974
SRR17520069.8212975
SRR17520069.8212976
SRR17520069.8212977
SRR17520069.8212978
SRR17520069.8212979
SRR17520069.8212980
SRR17520069.8212981
SRR17520069.8212982
SRR17520069.8212983
SRR17520069.8212984
SRR17520069.8212985
SRR17520069.8212986
SRR17520069.8212987
SRR17520069.8212988
SRR17520069.8212989
SRR17520069.8212990
SRR17520069.8212991
SRR17520069.8212992
SRR17520069.8212993
SRR17520069.8212994
SRR17520069.8212995
SRR17520069.8212996
SRR17520069.8212997
SRR17520069.8212998
SRR17520069.8212999
SRR17520069.8213000
SRR17520069.8213001
SRR17520069.8213002
SRR17520069.8213003
SRR17520069.8213004
SRR17520069.8213005
SRR17520069.8213007
SRR17520069.8213008
SRR17520069.8213009
SRR17520069.8213010
SRR17520069.8213080
SRR17520069.8213084
SRR17520069.8215799
SRR17520069.8223941
SRR17520069.8223942
SRR17520069.8223943
SRR17520069.8223944
SRR17520069.8223945
SRR17520069.8223946
SRR17520069.8223947
SRR17520069.8223948

SRR17520069.8223949
SRR17520069.8223950
SRR17520069.8223951
SRR17520069.8223952
SRR17520069.8223953
SRR17520069.8223954
SRR17520069.8223955
SRR17520069.8223956
SRR17520069.8223957
SRR17520069.8223958
SRR17520069.8223959
SRR17520069.8223960
SRR17520069.8225032
SRR17520069.8234649
SRR17520069.8243454
SRR17520069.8243460
SRR17520069.8243482
SRR17520069.8243496
SRR17520069.8245476
SRR17520069.8245477
SRR17520069.8245478
SRR17520069.8245479
SRR17520069.8245480
SRR17520069.8245481
SRR17520069.8245482
SRR17520069.8245484
SRR17520069.8245485
SRR17520069.8245487
SRR17520069.8245488
SRR17520069.8245489
SRR17520069.8245490
SRR17520069.8245491
SRR17520069.8245492
SRR17520069.8245493
SRR17520069.8245494
SRR17520069.8245495
SRR17520069.8245496
SRR17520069.8245497
SRR17520069.8245498
SRR17520069.8245499
SRR17520069.8245500
SRR17520069.8245501
SRR17520069.8245502
SRR17520069.8245503
SRR17520069.8245504
SRR17520069.8245505
SRR17520069.8245506

SRR17520069.8245507
SRR17520069.8245508
SRR17520069.8245509
SRR17520069.8245510
SRR17520069.8245511
SRR17520069.8245512
SRR17520069.8245513
SRR17520069.8245514
SRR17520069.8245515
SRR17520069.8245516
SRR17520069.8245517
SRR17520069.8245518
SRR17520069.8245519
SRR17520069.8245520
SRR17520069.8245521
SRR17520069.8245522
SRR17520069.8245523
SRR17520069.8245524
SRR17520069.8245525
SRR17520069.8245526
SRR17520069.8245527
SRR17520069.8245528
SRR17520069.8245529
SRR17520069.8245530
SRR17520069.8245532
SRR17520069.8245533
SRR17520069.8245534
SRR17520069.8245535
SRR17520069.8245536
SRR17520069.8245537
SRR17520069.8245538
SRR17520069.8245539
SRR17520069.8245540
SRR17520069.8245541
SRR17520069.8245542
SRR17520069.8245543
SRR17520069.8245544
SRR17520069.8245545
SRR17520069.8245546
SRR17520069.8245547
SRR17520069.8245548
SRR17520069.8245549
SRR17520069.8245550
SRR17520069.8245551
SRR17520069.8245552
SRR17520069.8245553
SRR17520069.8245554

SRR17520069.8245555
SRR17520069.8245556
SRR17520069.8245558
SRR17520069.8245559
SRR17520069.8245560
SRR17520069.8245561
SRR17520069.8245562
SRR17520069.8245563
SRR17520069.8245564
SRR17520069.8245565
SRR17520069.8245566
SRR17520069.8245567
SRR17520069.8245568
SRR17520069.8245569
SRR17520069.8245570
SRR17520069.8245571
SRR17520069.8245572
SRR17520069.8245573
SRR17520069.8245574
SRR17520069.8253325
SRR17520069.8253327
SRR17520069.8260354
SRR17520069.8262926
SRR17520069.8262927
SRR17520069.8262928
SRR17520069.8262929
SRR17520069.8262930
SRR17520069.8262931
SRR17520069.8262932
SRR17520069.8262933
SRR17520069.8262934
SRR17520069.8262935
SRR17520069.8262936
SRR17520069.8262937
SRR17520069.8262938
SRR17520069.8262939
SRR17520069.8262941
SRR17520069.8262942
SRR17520069.8262943
SRR17520069.8262944
SRR17520069.8262945
SRR17520069.8262946
SRR17520069.8266409
SRR17520069.8266410
SRR17520069.8266411
SRR17520069.8266412
SRR17520069.8266413

SRR17520069.8266414
SRR17520069.8266415
SRR17520069.8266416
SRR17520069.8266417
SRR17520069.8266418
SRR17520069.8266419
SRR17520069.8266420
SRR17520069.8266421
SRR17520069.8266422
SRR17520069.8266423
SRR17520069.8266424
SRR17520069.8266425
SRR17520069.8266426
SRR17520069.8275479
SRR17520069.8275480
SRR17520069.8275481
SRR17520069.8275482
SRR17520069.8275483
SRR17520069.8275484
SRR17520069.8275485
SRR17520069.8275486
SRR17520069.8275487
SRR17520069.8275488
SRR17520069.8291649
SRR17520069.8302075
SRR17520069.8306436
SRR17520069.8306437
SRR17520069.8306438
SRR17520069.8306439
SRR17520069.8306440
SRR17520069.8306441
SRR17520069.8306443
SRR17520069.8306445
SRR17520069.8306446
SRR17520069.8306447
SRR17520069.8306448
SRR17520069.8306449
SRR17520069.8306450
SRR17520069.8306451
SRR17520069.8306452
SRR17520069.8306453
SRR17520069.8306454
SRR17520069.8306455
SRR17520069.8306456
SRR17520069.8306457
SRR17520069.8306458
SRR17520069.8306461

SRR17520069.8306462
SRR17520069.8306463
SRR17520069.8306464
SRR17520069.8306467
SRR17520069.8306468
SRR17520069.8306469
SRR17520069.8306470
SRR17520069.8306471
SRR17520069.8306472
SRR17520069.8306473
SRR17520069.8306474
SRR17520069.8306475
SRR17520069.8306476
SRR17520069.8306477
SRR17520069.8306478
SRR17520069.8306479
SRR17520069.8306480
SRR17520069.8306482
SRR17520069.8306483
SRR17520069.8306484
SRR17520069.8306485
SRR17520069.8306486
SRR17520069.8306487
SRR17520069.8306488
SRR17520069.8306489
SRR17520069.8306490
SRR17520069.8306491
SRR17520069.8306492
SRR17520069.8306493
SRR17520069.8306496
SRR17520069.8319478
SRR17520069.8319479
SRR17520069.8319480
SRR17520069.8319481
SRR17520069.8319482
SRR17520069.8319483
SRR17520069.8319484
SRR17520069.8319485
SRR17520069.8319486
SRR17520069.8319487
SRR17520069.8319488
SRR17520069.8319489
SRR17520069.8319490
SRR17520069.8319491
SRR17520069.8319492
SRR17520069.8319493
SRR17520069.8319494

SRR17520069.8319495
SRR17520069.8319496
SRR17520069.8319497
SRR17520069.8319498
SRR17520069.8319499
SRR17520069.8319500
SRR17520069.8319501
SRR17520069.8319502
SRR17520069.8319503
SRR17520069.8319504
SRR17520069.8319505
SRR17520069.8319506
SRR17520069.8319507
SRR17520069.8319508
SRR17520069.8319509
SRR17520069.8319510
SRR17520069.8319511
SRR17520069.8319512
SRR17520069.8319513
SRR17520069.8319514
SRR17520069.8319515
SRR17520069.8319516
SRR17520069.8319517
SRR17520069.8319518
SRR17520069.8319519
SRR17520069.8319520
SRR17520069.8319521
SRR17520069.8319522
SRR17520069.8319523
SRR17520069.8319524
SRR17520069.8319525
SRR17520069.8319526
SRR17520069.8319527
SRR17520069.8319528
SRR17520069.8319529
SRR17520069.8319530
SRR17520069.8319531
SRR17520069.8319532
SRR17520069.8319533
SRR17520069.8319534
SRR17520069.8319535
SRR17520069.8319536
SRR17520069.8319537
SRR17520069.8319538
SRR17520069.8319539
SRR17520069.8319540
SRR17520069.8319541

SRR17520069.8319542
SRR17520069.8319543
SRR17520069.8319544
SRR17520069.8319545
SRR17520069.8319546
SRR17520069.8319547
SRR17520069.8319548
SRR17520069.8319549
SRR17520069.8319550
SRR17520069.8319551
SRR17520069.8319552
SRR17520069.8325950
SRR17520069.8325951
SRR17520069.8325952
SRR17520069.8325953
SRR17520069.8325954
SRR17520069.8325955
SRR17520069.8325956
SRR17520069.8325957
SRR17520069.8325958
SRR17520069.8325959
SRR17520069.8325960
SRR17520069.8325961
SRR17520069.8325962
SRR17520069.8325963
SRR17520069.8325964
SRR17520069.8327959
SRR17520069.8333858
SRR17520069.8362987
SRR17520069.8362988
SRR17520069.8362989
SRR17520069.8362990
SRR17520069.8362991
SRR17520069.8362992
SRR17520069.8362993
SRR17520069.8362994
SRR17520069.8362995
SRR17520069.8362996
SRR17520069.8362997
SRR17520069.8362998
SRR17520069.8362999
SRR17520069.8363000
SRR17520069.8363001
SRR17520069.8363002
SRR17520069.8363003
SRR17520069.8363004
SRR17520069.8363005

SRR17520069.8363006
SRR17520069.8363007
SRR17520069.8363008
SRR17520069.8363009
SRR17520069.8363010
SRR17520069.8363011
SRR17520069.8363012
SRR17520069.8363013
SRR17520069.8363014
SRR17520069.8363015
SRR17520069.8363016
SRR17520069.8363017
SRR17520069.8363018
SRR17520069.8363019
SRR17520069.8363020
SRR17520069.8363021
SRR17520069.8363022
SRR17520069.8363023
SRR17520069.8363024
SRR17520069.8363025
SRR17520069.8363026
SRR17520069.8363027
SRR17520069.8363028
SRR17520069.8363029
SRR17520069.8363030
SRR17520069.8363031
SRR17520069.8363032
SRR17520069.8363033
SRR17520069.8363034
SRR17520069.8363035
SRR17520069.8363036
SRR17520069.8363037
SRR17520069.8363038
SRR17520069.8363039
SRR17520069.8363040
SRR17520069.8363041
SRR17520069.8363042
SRR17520069.8363043
SRR17520069.8363044
SRR17520069.8363045
SRR17520069.8363046
SRR17520069.8363047
SRR17520069.8363048
SRR17520069.8363049
SRR17520069.8363050
SRR17520069.8363051
SRR17520069.8363052

SRR17520069.8363053
SRR17520069.8363054
SRR17520069.8363055
SRR17520069.8363056
SRR17520069.8363057
SRR17520069.8363058
SRR17520069.8363059
SRR17520069.8363060
SRR17520069.8363061
SRR17520069.8363062
SRR17520069.8363063
SRR17520069.8363064
SRR17520069.8363065
SRR17520069.8363066
SRR17520069.8363067
SRR17520069.8363068
SRR17520069.8363069
SRR17520069.8363070
SRR17520069.8363071
SRR17520069.8363072
SRR17520069.8363073
SRR17520069.8363074
SRR17520069.8363075
SRR17520069.8363077
SRR17520069.8363078
SRR17520069.8363079
SRR17520069.8363080
SRR17520069.8363081
SRR17520069.8363082
SRR17520069.8363083
SRR17520069.8363084
SRR17520069.8363085
SRR17520069.8363086
SRR17520069.8363087
SRR17520069.8363088
SRR17520069.8363089
SRR17520069.8363090
SRR17520069.8363091
SRR17520069.8363092
SRR17520069.8363093
SRR17520069.8363094
SRR17520069.8363095
SRR17520069.8363096
SRR17520069.8363097
SRR17520069.8363098
SRR17520069.8363099
SRR17520069.8363100

SRR17520069.8363101
SRR17520069.8363102
SRR17520069.8368394
SRR17520069.8368395
SRR17520069.8368396
SRR17520069.8368397
SRR17520069.8368398
SRR17520069.8368399
SRR17520069.8368400
SRR17520069.8368401
SRR17520069.8368402
SRR17520069.8368403
SRR17520069.8368404
SRR17520069.8368405
SRR17520069.8368406
SRR17520069.8368407
SRR17520069.8368408
SRR17520069.8368409
SRR17520069.8368410
SRR17520069.8368411
SRR17520069.8368412
SRR17520069.8368413
SRR17520069.8368415
SRR17520069.8368416
SRR17520069.8368417
SRR17520069.8379888
SRR17520069.8386844
SRR17520069.8386845
SRR17520069.8386846
SRR17520069.8386847
SRR17520069.8386848
SRR17520069.8386849
SRR17520069.8386850
SRR17520069.8386851
SRR17520069.8386852
SRR17520069.8386853
SRR17520069.8386854
SRR17520069.8386855
SRR17520069.8386857
SRR17520069.8386858
SRR17520069.8386859
SRR17520069.8386860
SRR17520069.8386862
SRR17520069.8397708
SRR17520069.8397709
SRR17520069.8397710
SRR17520069.8397711

SRR17520069.8397712
SRR17520069.8397713
SRR17520069.8397714
SRR17520069.8397715
SRR17520069.8397716
SRR17520069.8397717
SRR17520069.8397718
SRR17520069.8404844
SRR17520069.8404884
SRR17520069.8406552
SRR17520069.8432861
SRR17520069.8447251
SRR17520069.8453260
SRR17520069.8473175
SRR17520069.8481337
SRR17520069.8497954
SRR17520069.8497955
SRR17520069.8497956
SRR17520069.8497957
SRR17520069.8497958
SRR17520069.8497959
SRR17520069.8497960
SRR17520069.8497961
SRR17520069.8497962
SRR17520069.8497963
SRR17520069.8497964
SRR17520069.8497965
SRR17520069.8497966
SRR17520069.8497967
SRR17520069.8497968
SRR17520069.8497969
SRR17520069.8497970
SRR17520069.8497971
SRR17520069.8497972
SRR17520069.8497973
SRR17520069.8497974
SRR17520069.8497975
SRR17520069.8497976
SRR17520069.8497977
SRR17520069.8497978
SRR17520069.8497979
SRR17520069.8497980
SRR17520069.8497981
SRR17520069.8497982
SRR17520069.8497983
SRR17520069.8497984
SRR17520069.8497985

SRR17520069.8497986
SRR17520069.8497987
SRR17520069.8497988
SRR17520069.8497989
SRR17520069.8497990
SRR17520069.8497991
SRR17520069.8497992
SRR17520069.8497993
SRR17520069.8497994
SRR17520069.8497996
SRR17520069.8497997
SRR17520069.8497998
SRR17520069.8497999
SRR17520069.8498000
SRR17520069.8498001
SRR17520069.8498002
SRR17520069.8498003
SRR17520069.8498004
SRR17520069.8498005
SRR17520069.8498006
SRR17520069.8498007
SRR17520069.8498008
SRR17520069.8498009
SRR17520069.8498010
SRR17520069.8498011
SRR17520069.8498012
SRR17520069.8498013
SRR17520069.8498014
SRR17520069.8498017
SRR17520069.8498018
SRR17520069.8498019
SRR17520069.8498020
SRR17520069.8498021
SRR17520069.8498022
SRR17520069.8498023
SRR17520069.8498024
SRR17520069.8498025
SRR17520069.8498026
SRR17520069.8498027
SRR17520069.8498028
SRR17520069.8498029
SRR17520069.8498030
SRR17520069.8498031
SRR17520069.8498032
SRR17520069.8498033
SRR17520069.8498034
SRR17520069.8498035

SRR17520069.8498036
SRR17520069.8498037
SRR17520069.8498038
SRR17520069.8498039
SRR17520069.8498040
SRR17520069.8498041
SRR17520069.8498042
SRR17520069.8498043
SRR17520069.8498044
SRR17520069.8498045
SRR17520069.8498046
SRR17520069.8498047
SRR17520069.8498048
SRR17520069.8498049
SRR17520069.8498050
SRR17520069.8498051
SRR17520069.8498052
SRR17520069.8498053
SRR17520069.8498054
SRR17520069.8498055
SRR17520069.8498056
SRR17520069.8498057
SRR17520069.8498058
SRR17520069.8498059
SRR17520069.8498060
SRR17520069.8498061
SRR17520069.8498062
SRR17520069.8498063
SRR17520069.8498064
SRR17520069.8498065
SRR17520069.8498066
SRR17520069.8498067
SRR17520069.8498068
SRR17520069.8498069
SRR17520069.8498070
SRR17520069.8498071
SRR17520069.8498072
SRR17520069.8498073
SRR17520069.8498074
SRR17520069.8498075
SRR17520069.8498076
SRR17520069.8498077
SRR17520069.8498078
SRR17520069.8498079
SRR17520069.8498080
SRR17520069.8498081
SRR17520069.8498082

SRR17520069.8498083
SRR17520069.8498084
SRR17520069.8498085
SRR17520069.8498086
SRR17520069.8498087
SRR17520069.8498088
SRR17520069.8498089
SRR17520069.8498090
SRR17520069.8498091
SRR17520069.8498093
SRR17520069.8498094
SRR17520069.8498095
SRR17520069.8498096
SRR17520069.8498097
SRR17520069.8498098
SRR17520069.8498099
SRR17520069.8498100
SRR17520069.8498101
SRR17520069.8498103
SRR17520069.8498104
SRR17520069.8498105
SRR17520069.8498106
SRR17520069.8498107
SRR17520069.8498108
SRR17520069.8498109
SRR17520069.8498110
SRR17520069.8519924
SRR17520069.8519925
SRR17520069.8519926
SRR17520069.8519927
SRR17520069.8519928
SRR17520069.8519929
SRR17520069.8519930
SRR17520069.8519931
SRR17520069.8519932
SRR17520069.8519933
SRR17520069.8519934
SRR17520069.8519935
SRR17520069.8519936
SRR17520069.8519937
SRR17520069.8519938
SRR17520069.8519939
SRR17520069.8519940
SRR17520069.8519941
SRR17520069.8519942
SRR17520069.8519943
SRR17520069.8519944

SRR17520069.8519945
SRR17520069.8519946
SRR17520069.8519947
SRR17520069.8519948
SRR17520069.8519950
SRR17520069.8519951
SRR17520069.8519952
SRR17520069.8519953
SRR17520069.8519954
SRR17520069.8519955
SRR17520069.8519956
SRR17520069.8519957
SRR17520069.8519958
SRR17520069.8519959
SRR17520069.8519960
SRR17520069.8519962
SRR17520069.8519963
SRR17520069.8519964
SRR17520069.8519965
SRR17520069.8519966
SRR17520069.8519967
SRR17520069.8519968
SRR17520069.8519969
SRR17520069.8519970
SRR17520069.8519971
SRR17520069.8519972
SRR17520069.8519973
SRR17520069.8519974
SRR17520069.8522671
SRR17520069.8522679
SRR17520069.8524187
SRR17520069.8524188
SRR17520069.8524189
SRR17520069.8524190
SRR17520069.8524191
SRR17520069.8524195
SRR17520069.8524199
SRR17520069.8524200
SRR17520069.8524202
SRR17520069.8524204
SRR17520069.8524206
SRR17520069.8524207
SRR17520069.8524208
SRR17520069.8524209
SRR17520069.8524211
SRR17520069.8524212
SRR17520069.8524213

SRR17520069.8524214
SRR17520069.8524215
SRR17520069.8524216
SRR17520069.8524217
SRR17520069.8524218
SRR17520069.8524219
SRR17520069.8524220
SRR17520069.8524221
SRR17520069.8524222
SRR17520069.8524224
SRR17520069.8524225
SRR17520069.8524226
SRR17520069.8524227
SRR17520069.8524228
SRR17520069.8524229
SRR17520069.8524230
SRR17520069.8524231
SRR17520069.8524232
SRR17520069.8524233
SRR17520069.8524234
SRR17520069.8524235
SRR17520069.8524236
SRR17520069.8524237
SRR17520069.8524239
SRR17520069.8524240
SRR17520069.8524241
SRR17520069.8524242
SRR17520069.8524243
SRR17520069.8524244
SRR17520069.8524245
SRR17520069.8524246
SRR17520069.8524247
SRR17520069.8524249
SRR17520069.8524250
SRR17520069.8524251
SRR17520069.8524252
SRR17520069.8524253
SRR17520069.8524254
SRR17520069.8524256
SRR17520069.8524257
SRR17520069.8524258
SRR17520069.8524259
SRR17520069.8524260
SRR17520069.8524261
SRR17520069.8524262
SRR17520069.8524263
SRR17520069.8524265

SRR17520069.8524266
SRR17520069.8524267
SRR17520069.8524269
SRR17520069.8524270
SRR17520069.8524271
SRR17520069.8524272
SRR17520069.8524273
SRR17520069.8524274
SRR17520069.8524277
SRR17520069.8526557
SRR17520069.8526558
SRR17520069.8526559
SRR17520069.8526560
SRR17520069.8526561
SRR17520069.8526562
SRR17520069.8526563
SRR17520069.8526564
SRR17520069.8526565
SRR17520069.8526567
SRR17520069.8526568
SRR17520069.8526569
SRR17520069.8526570
SRR17520069.8526571
SRR17520069.8526572
SRR17520069.8526573
SRR17520069.8526574
SRR17520069.8526575
SRR17520069.8526576
SRR17520069.8526577
SRR17520069.8526578
SRR17520069.8526579
SRR17520069.8526580
SRR17520069.8526581
SRR17520069.8526582
SRR17520069.8526583
SRR17520069.8526584
SRR17520069.8526585
SRR17520069.8526586
SRR17520069.8526587
SRR17520069.8526588
SRR17520069.8526590
SRR17520069.8526591
SRR17520069.8526592
SRR17520069.8526593
SRR17520069.8526594
SRR17520069.8526596
SRR17520069.8526597

SRR17520069.8526599
SRR17520069.8526600
SRR17520069.8526601
SRR17520069.8526604
SRR17520069.8526605
SRR17520069.8526606
SRR17520069.8526607
SRR17520069.8526608
SRR17520069.8526609
SRR17520069.8526610
SRR17520069.8526611
SRR17520069.8526613
SRR17520069.8526614
SRR17520069.8526615
SRR17520069.8526617
SRR17520069.8526618
SRR17520069.8526619
SRR17520069.8526620
SRR17520069.8526621
SRR17520069.8526622
SRR17520069.8526623
SRR17520069.8526626
SRR17520069.8526627
SRR17520069.8526628
SRR17520069.8526629
SRR17520069.8526630
SRR17520069.8526631
SRR17520069.8526633
SRR17520069.8526634
SRR17520069.8526635
SRR17520069.8526636
SRR17520069.8526637
SRR17520069.8526638
SRR17520069.8526639
SRR17520069.8526640
SRR17520069.8526641
SRR17520069.8526642
SRR17520069.8526643
SRR17520069.8526644
SRR17520069.8526645
SRR17520069.8526646
SRR17520069.8526648
SRR17520069.8526649
SRR17520069.8526652
SRR17520069.8526653
SRR17520069.8526654
SRR17520069.8526655

SRR17520069.8526657
SRR17520069.8526658
SRR17520069.8526659
SRR17520069.8526660
SRR17520069.8526661
SRR17520069.8526662
SRR17520069.8526663
SRR17520069.8526664
SRR17520069.8526665
SRR17520069.8526666
SRR17520069.8526667
SRR17520069.8526668
SRR17520069.8526669
SRR17520069.8526670
SRR17520069.8526671
SRR17520069.8526672
SRR17520069.8526673
SRR17520069.8526674
SRR17520069.8526675
SRR17520069.8526676
SRR17520069.8526677
SRR17520069.8526678
SRR17520069.8526679
SRR17520069.8526680
SRR17520069.8526681
SRR17520069.8526684
SRR17520069.8526685
SRR17520069.8526686
SRR17520069.8526687
SRR17520069.8526689
SRR17520069.8526690
SRR17520069.8526691
SRR17520069.8526692
SRR17520069.8526695
SRR17520069.8526696
SRR17520069.8526697
SRR17520069.8544767
SRR17520069.8554335
SRR17520069.8569641
SRR17520069.8569642
SRR17520069.8569643
SRR17520069.8569644
SRR17520069.8569645
SRR17520069.8569646
SRR17520069.8569647
SRR17520069.8569648
SRR17520069.8569649

SRR17520069.8569650
SRR17520069.8569651
SRR17520069.8569652
SRR17520069.8569653
SRR17520069.8569654
SRR17520069.8569655
SRR17520069.8569656
SRR17520069.8569657
SRR17520069.8569658
SRR17520069.8569659
SRR17520069.8569660
SRR17520069.8569661
SRR17520069.8569662
SRR17520069.8569663
SRR17520069.8569664
SRR17520069.8569665
SRR17520069.8569666
SRR17520069.8569667
SRR17520069.8569668
SRR17520069.8569669
SRR17520069.8569670
SRR17520069.8569671
SRR17520069.8569672
SRR17520069.8569673
SRR17520069.8569674
SRR17520069.8569675
SRR17520069.8569676
SRR17520069.8569677
SRR17520069.8569678
SRR17520069.8569679
SRR17520069.8569680
SRR17520069.8569681
SRR17520069.8569682
SRR17520069.8569683
SRR17520069.8569684
SRR17520069.8569685
SRR17520069.8569686
SRR17520069.8569687
SRR17520069.8569688
SRR17520069.8569689
SRR17520069.8569690
SRR17520069.8569691
SRR17520069.8569692
SRR17520069.8569693
SRR17520069.8569694
SRR17520069.8569695
SRR17520069.8569696

SRR17520069.8575629
SRR17520069.8575630
SRR17520069.8575631
SRR17520069.8575632
SRR17520069.8575633
SRR17520069.8575634
SRR17520069.8575635
SRR17520069.8575636
SRR17520069.8575637
SRR17520069.8575639
SRR17520069.8575640
SRR17520069.8575641
SRR17520069.8575642
SRR17520069.8575643
SRR17520069.8593772
SRR17520069.8593774
SRR17520069.8593775
SRR17520069.8593776
SRR17520069.8593777
SRR17520069.8593778
SRR17520069.8593779
SRR17520069.8593780
SRR17520069.8593781
SRR17520069.8593782
SRR17520069.8593783
SRR17520069.8593784
SRR17520069.8593785
SRR17520069.8593786
SRR17520069.8593787
SRR17520069.8593788
SRR17520069.8593789
SRR17520069.8593790
SRR17520069.8593791
SRR17520069.8593792
SRR17520069.8593793
SRR17520069.8593794
SRR17520069.8593795
SRR17520069.8593796
SRR17520069.8593797
SRR17520069.8593798
SRR17520069.8593799
SRR17520069.8593800
SRR17520069.8593801
SRR17520069.8593802
SRR17520069.8593803
SRR17520069.8593804
SRR17520069.8593805

SRR17520069.8593806
SRR17520069.8593807
SRR17520069.8593808
SRR17520069.8593809
SRR17520069.8593810
SRR17520069.8593811
SRR17520069.8593812
SRR17520069.8593813
SRR17520069.8593814
SRR17520069.8593815
SRR17520069.8593816
SRR17520069.8593817
SRR17520069.8593818
SRR17520069.8593819
SRR17520069.8593820
SRR17520069.8593821
SRR17520069.8599928
SRR17520069.8607416
SRR17520069.8607417
SRR17520069.8607418
SRR17520069.8607419
SRR17520069.8607420
SRR17520069.8607421
SRR17520069.8607422
SRR17520069.8607423
SRR17520069.8607425
SRR17520069.8607426
SRR17520069.8607427
SRR17520069.8607428
SRR17520069.8607429
SRR17520069.8607430
SRR17520069.8611067
SRR17520069.8630207
SRR17520069.8630261
SRR17520069.8630266
SRR17520069.8672329
SRR17520069.8672330
SRR17520069.8672331
SRR17520069.8672332
SRR17520069.8693102
SRR17520069.8693103
SRR17520069.8693104
SRR17520069.8693108
SRR17520069.8693109
SRR17520069.8693110
SRR17520069.8693111
SRR17520069.8693112

SRR17520069.8693113
SRR17520069.8693114
SRR17520069.8693115
SRR17520069.8693116
SRR17520069.8693117
SRR17520069.8693118
SRR17520069.8693119
SRR17520069.8693120
SRR17520069.8693121
SRR17520069.8693122
SRR17520069.8693123
SRR17520069.8693124
SRR17520069.8693126
SRR17520069.8693127
SRR17520069.8693128
SRR17520069.8693129
SRR17520069.8693130
SRR17520069.8693131
SRR17520069.8693132
SRR17520069.8693133
SRR17520069.8693134
SRR17520069.8693135
SRR17520069.8693136
SRR17520069.8693137
SRR17520069.8693138
SRR17520069.8693139
SRR17520069.8693140
SRR17520069.8693141
SRR17520069.8693142
SRR17520069.8693143
SRR17520069.8693144
SRR17520069.8693145
SRR17520069.8693146
SRR17520069.8693147
SRR17520069.8693148
SRR17520069.8693149
SRR17520069.8693150
SRR17520069.8693151
SRR17520069.8693152
SRR17520069.8693153
SRR17520069.8693154
SRR17520069.8693155
SRR17520069.8693156
SRR17520069.8693157
SRR17520069.8693158
SRR17520069.8693159
SRR17520069.8693160

SRR17520069.8693161
SRR17520069.8693162
SRR17520069.8693163
SRR17520069.8693164
SRR17520069.8693165
SRR17520069.8693166
SRR17520069.8693167
SRR17520069.8693168
SRR17520069.8693169
SRR17520069.8693170
SRR17520069.8693171
SRR17520069.8693172
SRR17520069.8693173
SRR17520069.8693174
SRR17520069.8693175
SRR17520069.8693176
SRR17520069.8693177
SRR17520069.8693178
SRR17520069.8693179
SRR17520069.8693180
SRR17520069.8693181
SRR17520069.8693182
SRR17520069.8693183
SRR17520069.8693184
SRR17520069.8693185
SRR17520069.8693186
SRR17520069.8693187
SRR17520069.8693188
SRR17520069.8693189
SRR17520069.8693190
SRR17520069.8700588
SRR17520069.8702689
SRR17520069.8707133
SRR17520069.8726628
SRR17520069.8728567
SRR17520069.8728568
SRR17520069.8728569
SRR17520069.8728570
SRR17520069.8728571
SRR17520069.8728572
SRR17520069.8728573
SRR17520069.8728574
SRR17520069.8728575
SRR17520069.8728576
SRR17520069.8728577
SRR17520069.8728578
SRR17520069.8728579

SRR17520069.8728580
SRR17520069.8730412
SRR17520069.8738815
SRR17520069.8738886
SRR17520069.8744758
SRR17520069.8744759
SRR17520069.8744760
SRR17520069.8744761
SRR17520069.8744762
SRR17520069.8744763
SRR17520069.8744764
SRR17520069.8744765
SRR17520069.8744766
SRR17520069.8744767
SRR17520069.8744768
SRR17520069.8744769
SRR17520069.8744770
SRR17520069.8744771
SRR17520069.8744772
SRR17520069.8744773
SRR17520069.8744774
SRR17520069.8744775
SRR17520069.8767167
SRR17520069.8767168
SRR17520069.8767169
SRR17520069.8767170
SRR17520069.8767171
SRR17520069.8767172
SRR17520069.8767173
SRR17520069.8767174
SRR17520069.8767175
SRR17520069.8767176
SRR17520069.8767177
SRR17520069.8767178
SRR17520069.8767179
SRR17520069.8767180
SRR17520069.8767181
SRR17520069.8767182
SRR17520069.8767183
SRR17520069.8767184
SRR17520069.8767185
SRR17520069.8767186
SRR17520069.8767187
SRR17520069.8767188
SRR17520069.8767189
SRR17520069.8767190
SRR17520069.8767191

SRR17520069.8767192
SRR17520069.8767193
SRR17520069.8767194
SRR17520069.8767195
SRR17520069.8767196
SRR17520069.8767197
SRR17520069.8767198
SRR17520069.8767199
SRR17520069.8767200
SRR17520069.8767201
SRR17520069.8767202
SRR17520069.8767203
SRR17520069.8767204
SRR17520069.8767205
SRR17520069.8767206
SRR17520069.8767207
SRR17520069.8767208
SRR17520069.8767209
SRR17520069.8767210
SRR17520069.8767211
SRR17520069.8767212
SRR17520069.8767213
SRR17520069.8767214
SRR17520069.8767215
SRR17520069.8767216
SRR17520069.8767217
SRR17520069.8767218
SRR17520069.8767219
SRR17520069.8767220
SRR17520069.8767221
SRR17520069.8767224
SRR17520069.8767225
SRR17520069.8767226
SRR17520069.8767227
SRR17520069.8767228
SRR17520069.8767229
SRR17520069.8767230
SRR17520069.8767231
SRR17520069.8767232
SRR17520069.8767233
SRR17520069.8767234
SRR17520069.8767235
SRR17520069.8767236
SRR17520069.8767237
SRR17520069.8767238
SRR17520069.8767239
SRR17520069.8767240

SRR17520069.8767241
SRR17520069.8767242
SRR17520069.8767243
SRR17520069.8767244
SRR17520069.8767245
SRR17520069.8767246
SRR17520069.8767247
SRR17520069.8767248
SRR17520069.8767249
SRR17520069.8767250
SRR17520069.8767251
SRR17520069.8767252
SRR17520069.8767253
SRR17520069.8772122
SRR17520069.8773271
SRR17520069.8788851
SRR17520069.8788852
SRR17520069.8788853
SRR17520069.8788854
SRR17520069.8788855
SRR17520069.8788856
SRR17520069.8788857
SRR17520069.8788858
SRR17520069.8788859
SRR17520069.8788860
SRR17520069.8788861
SRR17520069.8788862
SRR17520069.8788863
SRR17520069.8791160
SRR17520069.8817246
SRR17520069.8817247
SRR17520069.8817248
SRR17520069.8817249
SRR17520069.8817250
SRR17520069.8817251
SRR17520069.8817252
SRR17520069.8817253
SRR17520069.8817254
SRR17520069.8817255
SRR17520069.8817257
SRR17520069.8817259
SRR17520069.8817260
SRR17520069.8817261
SRR17520069.8817262
SRR17520069.8817263
SRR17520069.8817264
SRR17520069.8817265

SRR17520069.8817266
SRR17520069.8817267
SRR17520069.8817268
SRR17520069.8817269
SRR17520069.8817270
SRR17520069.8817271
SRR17520069.8817272
SRR17520069.8817273
SRR17520069.8817274
SRR17520069.8817275
SRR17520069.8817276
SRR17520069.8817277
SRR17520069.8817278
SRR17520069.8817279
SRR17520069.8817280
SRR17520069.8817281
SRR17520069.8817282
SRR17520069.8817283
SRR17520069.8817284
SRR17520069.8817285
SRR17520069.8817286
SRR17520069.8817287
SRR17520069.8817288
SRR17520069.8817289
SRR17520069.8817290
SRR17520069.8817291
SRR17520069.8817292
SRR17520069.8817293
SRR17520069.8817294
SRR17520069.8817295
SRR17520069.8817296
SRR17520069.8817297
SRR17520069.8817298
SRR17520069.8817299
SRR17520069.8817300
SRR17520069.8817301
SRR17520069.8817302
SRR17520069.8817303
SRR17520069.8817304
SRR17520069.8817305
SRR17520069.8817306
SRR17520069.8817307
SRR17520069.8817308
SRR17520069.8817309
SRR17520069.8817310
SRR17520069.8817311
SRR17520069.8817312

SRR17520069.8817313
SRR17520069.8817314
SRR17520069.8817315
SRR17520069.8817316
SRR17520069.8817318
SRR17520069.8817319
SRR17520069.8817320
SRR17520069.8817321
SRR17520069.8817322
SRR17520069.8817323
SRR17520069.8817324
SRR17520069.8817325
SRR17520069.8817326
SRR17520069.8817328
SRR17520069.8817329
SRR17520069.8817330
SRR17520069.8817331
SRR17520069.8817332
SRR17520069.8817333
SRR17520069.8817334
SRR17520069.8817335
SRR17520069.8817336
SRR17520069.8817337
SRR17520069.8817338
SRR17520069.8817339
SRR17520069.8817340
SRR17520069.8817341
SRR17520069.8817342
SRR17520069.8817343
SRR17520069.8817344
SRR17520069.8817345
SRR17520069.8817346
SRR17520069.8817347
SRR17520069.8832370
SRR17520069.8832371
SRR17520069.8832372
SRR17520069.8832373
SRR17520069.8832375
SRR17520069.8832378
SRR17520069.8832379
SRR17520069.8832380
SRR17520069.8832381
SRR17520069.8832382
SRR17520069.8832383
SRR17520069.8832384
SRR17520069.8832386
SRR17520069.8832387

SRR17520069.8832388
SRR17520069.8832389
SRR17520069.8832390
SRR17520069.8832391
SRR17520069.8832392
SRR17520069.8832393
SRR17520069.8832394
SRR17520069.8832395
SRR17520069.8832396
SRR17520069.8832397
SRR17520069.8832398
SRR17520069.8832399
SRR17520069.8832400
SRR17520069.8832401
SRR17520069.8832402
SRR17520069.8832403
SRR17520069.8832404
SRR17520069.8843344
SRR17520069.8849943
SRR17520069.8849944
SRR17520069.8849945
SRR17520069.8849946
SRR17520069.8849947
SRR17520069.8849948
SRR17520069.8849949
SRR17520069.8849950
SRR17520069.8849951
SRR17520069.8849952
SRR17520069.8849953
SRR17520069.8849954
SRR17520069.8849955
SRR17520069.8849956
SRR17520069.8849957
SRR17520069.8849958
SRR17520069.8849959
SRR17520069.8849960
SRR17520069.8849961
SRR17520069.8849962
SRR17520069.8849963
SRR17520069.8849964
SRR17520069.8849965
SRR17520069.8849966
SRR17520069.8849967
SRR17520069.8849968
SRR17520069.8849969
SRR17520069.8849970
SRR17520069.8849971

SRR17520069.8849972
SRR17520069.8849973
SRR17520069.8849974
SRR17520069.8849975
SRR17520069.8849977
SRR17520069.8849978
SRR17520069.8849979
SRR17520069.8849980
SRR17520069.8849981
SRR17520069.8849982
SRR17520069.8849983
SRR17520069.8857302
SRR17520069.8857303
SRR17520069.8857304
SRR17520069.8857305
SRR17520069.8857306
SRR17520069.8857307
SRR17520069.8857308
SRR17520069.8857309
SRR17520069.8857310
SRR17520069.8857311
SRR17520069.8857312
SRR17520069.8857313
SRR17520069.8857314
SRR17520069.8857315
SRR17520069.8857316
SRR17520069.8857317
SRR17520069.8857318
SRR17520069.8857319
SRR17520069.8857320
SRR17520069.8857321
SRR17520069.8857322
SRR17520069.8857323
SRR17520069.8857324
SRR17520069.8857325
SRR17520069.8857326
SRR17520069.8857327
SRR17520069.8857328
SRR17520069.8857329
SRR17520069.8857330
SRR17520069.8857331
SRR17520069.8857332
SRR17520069.8857333
SRR17520069.8857334
SRR17520069.8857335
SRR17520069.8857336
SRR17520069.8857337

SRR17520069.8857338
SRR17520069.8857339
SRR17520069.8857340
SRR17520069.8857341
SRR17520069.8857342
SRR17520069.8857343
SRR17520069.8857344
SRR17520069.8857345
SRR17520069.8857346
SRR17520069.8857347
SRR17520069.8857348
SRR17520069.8857349
SRR17520069.8857350
SRR17520069.8857351
SRR17520069.8857352
SRR17520069.8857353
SRR17520069.8857354
SRR17520069.8857355
SRR17520069.8857356
SRR17520069.8857357
SRR17520069.8857358
SRR17520069.8857359
SRR17520069.8857360
SRR17520069.8857361
SRR17520069.8857362
SRR17520069.8857363
SRR17520069.8857364
SRR17520069.8857366
SRR17520069.8857367
SRR17520069.8857368
SRR17520069.8857369
SRR17520069.8857370
SRR17520069.8857371
SRR17520069.8857372
SRR17520069.8857373
SRR17520069.8857374
SRR17520069.8857375
SRR17520069.8857376
SRR17520069.8857377
SRR17520069.8857378
SRR17520069.8857379
SRR17520069.8857380
SRR17520069.8857382
SRR17520069.8857383
SRR17520069.8857384
SRR17520069.8857385
SRR17520069.8857386

SRR17520069.8857387
SRR17520069.8857388
SRR17520069.8857389
SRR17520069.8857390
SRR17520069.8857391
SRR17520069.8857392
SRR17520069.8857393
SRR17520069.8857394
SRR17520069.8857395
SRR17520069.8857396
SRR17520069.8857397
SRR17520069.8857398
SRR17520069.8857399
SRR17520069.8857400
SRR17520069.8857401
SRR17520069.8857402
SRR17520069.8857403
SRR17520069.8857404
SRR17520069.8857405
SRR17520069.8857406
SRR17520069.8857407
SRR17520069.8857408
SRR17520069.8857409
SRR17520069.8857410
SRR17520069.8857411
SRR17520069.8857412
SRR17520069.8857413
SRR17520069.8857414
SRR17520069.8857415
SRR17520069.8857416
SRR17520069.8857417
SRR17520069.8857418
SRR17520069.8857419
SRR17520069.8857420
SRR17520069.8857421
SRR17520069.8857422
SRR17520069.8857423
SRR17520069.8857424
SRR17520069.8857425
SRR17520069.8857426
SRR17520069.8857427
SRR17520069.8857428
SRR17520069.8857430
SRR17520069.8857431
SRR17520069.8857432
SRR17520069.8857433
SRR17520069.8857434

SRR17520069.8857435
SRR17520069.8857436
SRR17520069.8857437
SRR17520069.8857438
SRR17520069.8857439
SRR17520069.8857440
SRR17520069.8859418
SRR17520069.8863299
SRR17520069.8863300
SRR17520069.8863301
SRR17520069.8863302
SRR17520069.8863303
SRR17520069.8863304
SRR17520069.8863305
SRR17520069.8863306
SRR17520069.8863307
SRR17520069.8863308
SRR17520069.8863309
SRR17520069.8863311
SRR17520069.8863312
SRR17520069.8863313
SRR17520069.8863314
SRR17520069.8863315
SRR17520069.8863316
SRR17520069.8863317
SRR17520069.8863318
SRR17520069.8863319
SRR17520069.8863320
SRR17520069.8863321
SRR17520069.8863322
SRR17520069.8863323
SRR17520069.8863324
SRR17520069.8863325
SRR17520069.8863326
SRR17520069.8863327
SRR17520069.8874648
SRR17520069.8876090
SRR17520069.8879338
SRR17520069.8879339
SRR17520069.8879342
SRR17520069.8879343
SRR17520069.8879344
SRR17520069.8879345
SRR17520069.8879346
SRR17520069.8879347
SRR17520069.8879348
SRR17520069.8879349

SRR17520069.8879350
SRR17520069.8879351
SRR17520069.8879352
SRR17520069.8879353
SRR17520069.8879355
SRR17520069.8879356
SRR17520069.8879357
SRR17520069.8879358
SRR17520069.8879359
SRR17520069.8879360
SRR17520069.8879361
SRR17520069.8879362
SRR17520069.8879363
SRR17520069.8879365
SRR17520069.8879366
SRR17520069.8879367
SRR17520069.8879368
SRR17520069.8879369
SRR17520069.8879370
SRR17520069.8879371
SRR17520069.8879372
SRR17520069.8879373
SRR17520069.8879374
SRR17520069.8879375
SRR17520069.8879376
SRR17520069.8879377
SRR17520069.8879378
SRR17520069.8879379
SRR17520069.8879380
SRR17520069.8879382
SRR17520069.8879383
SRR17520069.8879384
SRR17520069.8879385
SRR17520069.8879386
SRR17520069.8879387
SRR17520069.8879388
SRR17520069.8879389
SRR17520069.8879390
SRR17520069.8879392
SRR17520069.8879394
SRR17520069.8879395
SRR17520069.8879396
SRR17520069.8879398
SRR17520069.8879399
SRR17520069.8879400
SRR17520069.8879401
SRR17520069.8879402

SRR17520069.8879403
SRR17520069.8879404
SRR17520069.8879405
SRR17520069.8879406
SRR17520069.8879407
SRR17520069.8879408
SRR17520069.8879409
SRR17520069.8879410
SRR17520069.8879411
SRR17520069.8879413
SRR17520069.8879414
SRR17520069.8879415
SRR17520069.8879416
SRR17520069.8879417
SRR17520069.8879418
SRR17520069.8879419
SRR17520069.8879420
SRR17520069.8879421
SRR17520069.8879423
SRR17520069.8879424
SRR17520069.8879425
SRR17520069.8879426
SRR17520069.8879427
SRR17520069.8879428
SRR17520069.8879429
SRR17520069.8879430
SRR17520069.8879432
SRR17520069.8879433
SRR17520069.8879434
SRR17520069.8879435
SRR17520069.8879436
SRR17520069.8879437
SRR17520069.8879438
SRR17520069.8879439
SRR17520069.8879440
SRR17520069.8879441
SRR17520069.8879442
SRR17520069.8879443
SRR17520069.8879444
SRR17520069.8879445
SRR17520069.8879446
SRR17520069.8879447
SRR17520069.8879448
SRR17520069.8879449
SRR17520069.8879450
SRR17520069.8879451
SRR17520069.8879452

SRR17520069.8879453
SRR17520069.8879455
SRR17520069.8879456
SRR17520069.8879457
SRR17520069.8879458
SRR17520069.8879459
SRR17520069.8879463
SRR17520069.8879464
SRR17520069.8879465
SRR17520069.8879469
SRR17520069.8879471
SRR17520069.8945345
SRR17520069.8946580
SRR17520069.8946581
SRR17520069.8946582
SRR17520069.8946583
SRR17520069.8946584
SRR17520069.8946585
SRR17520069.8946586
SRR17520069.8946587
SRR17520069.8946588
SRR17520069.8946589
SRR17520069.8946590
SRR17520069.8946591
SRR17520069.8946592
SRR17520069.8946593
SRR17520069.8946594
SRR17520069.8946595
SRR17520069.8946596
SRR17520069.8946597
SRR17520069.8946598
SRR17520069.8946599
SRR17520069.8946600
SRR17520069.8946601
SRR17520069.8946602
SRR17520069.8946603
SRR17520069.8946604
SRR17520069.8946605
SRR17520069.8946606
SRR17520069.8946607
SRR17520069.8946608
SRR17520069.8946609
SRR17520069.8946610
SRR17520069.8946611
SRR17520069.8946612
SRR17520069.8946613
SRR17520069.8946614

SRR17520069.8946615
SRR17520069.8946616
SRR17520069.8946617
SRR17520069.8946618
SRR17520069.8946619
SRR17520069.8946620
SRR17520069.8946621
SRR17520069.8946622
SRR17520069.8946623
SRR17520069.8946624
SRR17520069.8946625
SRR17520069.8946626
SRR17520069.8946627
SRR17520069.8946628
SRR17520069.8946629
SRR17520069.8946630
SRR17520069.8946631
SRR17520069.8946632
SRR17520069.8946633
SRR17520069.8946634
SRR17520069.8946635
SRR17520069.8946636
SRR17520069.8967959
SRR17520069.8987238
SRR17520069.8987240
SRR17520069.8987241
SRR17520069.8987242
SRR17520069.8987244
SRR17520069.8987245
SRR17520069.8987246
SRR17520069.8987247
SRR17520069.8987248
SRR17520069.8987249
SRR17520069.8987250
SRR17520069.8987251
SRR17520069.8987252
SRR17520069.8987253
SRR17520069.8987254
SRR17520069.8987255
SRR17520069.8987256
SRR17520069.8987257
SRR17520069.8987258
SRR17520069.8987259
SRR17520069.8987260
SRR17520069.8987261
SRR17520069.8987262
SRR17520069.8987263

SRR17520069.8987264
SRR17520069.8987265
SRR17520069.8987266
SRR17520069.8987267
SRR17520069.8987269
SRR17520069.9006294
SRR17520069.9008592
SRR17520069.9008666
SRR17520069.9017115
SRR17520069.9017116
SRR17520069.9017117
SRR17520069.9017118
SRR17520069.9017119
SRR17520069.9017120
SRR17520069.9017121
SRR17520069.9017122
SRR17520069.9017123
SRR17520069.9017124
SRR17520069.9017125
SRR17520069.9017126
SRR17520069.9017127
SRR17520069.9017128
SRR17520069.9017129
SRR17520069.9017130
SRR17520069.9017131
SRR17520069.9017133
SRR17520069.9017134
SRR17520069.9017135
SRR17520069.9017136
SRR17520069.9017137
SRR17520069.9017138
SRR17520069.9017139
SRR17520069.9017140
SRR17520069.9017141
SRR17520069.9017142
SRR17520069.9017143
SRR17520069.9017144
SRR17520069.9017145
SRR17520069.9017146
SRR17520069.9017147
SRR17520069.9017148
SRR17520069.9017149
SRR17520069.9017150
SRR17520069.9017153
SRR17520069.9017154
SRR17520069.9017155
SRR17520069.9017156

SRR17520069.9017157
SRR17520069.9017158
SRR17520069.9017159
SRR17520069.9017160
SRR17520069.9017161
SRR17520069.9017162
SRR17520069.9017163
SRR17520069.9017164
SRR17520069.9017165
SRR17520069.9017166
SRR17520069.9017167
SRR17520069.9017168
SRR17520069.9017169
SRR17520069.9017170
SRR17520069.9017171
SRR17520069.9017172
SRR17520069.9017173
SRR17520069.9017174
SRR17520069.9017175
SRR17520069.9017176
SRR17520069.9017177
SRR17520069.9017178
SRR17520069.9017179
SRR17520069.9017180
SRR17520069.9017181
SRR17520069.9017182
SRR17520069.9017183
SRR17520069.9017184
SRR17520069.9017185
SRR17520069.9017186
SRR17520069.9017187
SRR17520069.9017188
SRR17520069.9017189
SRR17520069.9017190
SRR17520069.9017191
SRR17520069.9017192
SRR17520069.9017193
SRR17520069.9017194
SRR17520069.9017195
SRR17520069.9017196
SRR17520069.9017197
SRR17520069.9017198
SRR17520069.9017199
SRR17520069.9017200
SRR17520069.9017201
SRR17520069.9017203
SRR17520069.9017204

SRR17520069.9017205
SRR17520069.9017206
SRR17520069.9017207
SRR17520069.9017208
SRR17520069.9017209
SRR17520069.9017210
SRR17520069.9017211
SRR17520069.9017212
SRR17520069.9017213
SRR17520069.9017214
SRR17520069.9017215
SRR17520069.9017216
SRR17520069.9026795
SRR17520069.9043820
SRR17520069.9043821
SRR17520069.9043822
SRR17520069.9043823
SRR17520069.9043824
SRR17520069.9043825
SRR17520069.9043826
SRR17520069.9043827
SRR17520069.9043828
SRR17520069.9043829
SRR17520069.9043830
SRR17520069.9043831
SRR17520069.9043832
SRR17520069.9043833
SRR17520069.9043834
SRR17520069.9043835
SRR17520069.9043836
SRR17520069.9043837
SRR17520069.9043838
SRR17520069.9043839
SRR17520069.9043840
SRR17520069.9043841
SRR17520069.9043842
SRR17520069.9043843
SRR17520069.9043844
SRR17520069.9043845
SRR17520069.9043846
SRR17520069.9043847
SRR17520069.9043848
SRR17520069.9043850
SRR17520069.9043851
SRR17520069.9043852
SRR17520069.9043853
SRR17520069.9043854

SRR17520069.9043855
SRR17520069.9043856
SRR17520069.9043857
SRR17520069.9043858
SRR17520069.9043859
SRR17520069.9043860
SRR17520069.9043861
SRR17520069.9043862
SRR17520069.9043863
SRR17520069.9043864
SRR17520069.9043865
SRR17520069.9043866
SRR17520069.9043867
SRR17520069.9043868
SRR17520069.9043869
SRR17520069.9043870
SRR17520069.9043871
SRR17520069.9043872
SRR17520069.9043874
SRR17520069.9043875
SRR17520069.9043876
SRR17520069.9043877
SRR17520069.9043878
SRR17520069.9043879
SRR17520069.9043880
SRR17520069.9043881
SRR17520069.9043882
SRR17520069.9043883
SRR17520069.9043884
SRR17520069.9043885
SRR17520069.9043886
SRR17520069.9043887
SRR17520069.9043888
SRR17520069.9043889
SRR17520069.9043890
SRR17520069.9043891
SRR17520069.9043892
SRR17520069.9043893
SRR17520069.9043894
SRR17520069.9043895
SRR17520069.9043896
SRR17520069.9043897
SRR17520069.9043898
SRR17520069.9043899
SRR17520069.9043900
SRR17520069.9043901
SRR17520069.9043902

SRR17520069.9043903
SRR17520069.9043904
SRR17520069.9043905
SRR17520069.9043906
SRR17520069.9043907
SRR17520069.9043908
SRR17520069.9043909
SRR17520069.9043910
SRR17520069.9043912
SRR17520069.9043913
SRR17520069.9043914
SRR17520069.9043915
SRR17520069.9043916
SRR17520069.9043917
SRR17520069.9043918
SRR17520069.9043919
SRR17520069.9043920
SRR17520069.9043921
SRR17520069.9043922
SRR17520069.9043923
SRR17520069.9043927
SRR17520069.9043928
SRR17520069.9048408
SRR17520069.9048409
SRR17520069.9048410
SRR17520069.9048411
SRR17520069.9060569
SRR17520069.9060570
SRR17520069.9060571
SRR17520069.9060572
SRR17520069.9060573
SRR17520069.9060574
SRR17520069.9060575
SRR17520069.9060576
SRR17520069.9060577
SRR17520069.9060578
SRR17520069.9060579
SRR17520069.9060580
SRR17520069.9060581
SRR17520069.9060582
SRR17520069.9060583
SRR17520069.9060584
SRR17520069.9060585
SRR17520069.9060586
SRR17520069.9060587
SRR17520069.9060588
SRR17520069.9060589

SRR17520069.9060590
SRR17520069.9060591
SRR17520069.9060592
SRR17520069.9060593
SRR17520069.9060594
SRR17520069.9060595
SRR17520069.9060596
SRR17520069.9060597
SRR17520069.9060598
SRR17520069.9060599
SRR17520069.9060600
SRR17520069.9060601
SRR17520069.9060602
SRR17520069.9060603
SRR17520069.9060604
SRR17520069.9060605
SRR17520069.9060606
SRR17520069.9060607
SRR17520069.9060608
SRR17520069.9060609
SRR17520069.9060610
SRR17520069.9060611
SRR17520069.9060612
SRR17520069.9060614
SRR17520069.9060615
SRR17520069.9060616
SRR17520069.9060617
SRR17520069.9060618
SRR17520069.9060619
SRR17520069.9060620
SRR17520069.9060621
SRR17520069.9060622
SRR17520069.9060623
SRR17520069.9060624
SRR17520069.9060625
SRR17520069.9060626
SRR17520069.9060627
SRR17520069.9060628
SRR17520069.9060629
SRR17520069.9060630
SRR17520069.9060631
SRR17520069.9060632
SRR17520069.9060633
SRR17520069.9060634
SRR17520069.9060635
SRR17520069.9060636
SRR17520069.9060637

SRR17520069.9060638
SRR17520069.9060639
SRR17520069.9060640
SRR17520069.9060641
SRR17520069.9060642
SRR17520069.9060643
SRR17520069.9060644
SRR17520069.9060645
SRR17520069.9060646
SRR17520069.9060647
SRR17520069.9060649
SRR17520069.9060650
SRR17520069.9060652
SRR17520069.9060653
SRR17520069.9060654
SRR17520069.9060655
SRR17520069.9060656
SRR17520069.9060657
SRR17520069.9060658
SRR17520069.9060659
SRR17520069.9060660
SRR17520069.9060661
SRR17520069.9060662
SRR17520069.9060663
SRR17520069.9060664
SRR17520069.9060665
SRR17520069.9060666
SRR17520069.9060667
SRR17520069.9060668
SRR17520069.9060669
SRR17520069.9060670
SRR17520069.9060671
SRR17520069.9060672
SRR17520069.9060673
SRR17520069.9060674
SRR17520069.9060675
SRR17520069.9060676
SRR17520069.9060677
SRR17520069.9060678
SRR17520069.9060679
SRR17520069.9060680
SRR17520069.9060681
SRR17520069.9060682
SRR17520069.9061593
SRR17520069.9068188
SRR17520069.9081409
SRR17520069.9083596

SRR17520069.9083598
SRR17520069.9083599
SRR17520069.9083600
SRR17520069.9083601
SRR17520069.9083602
SRR17520069.9083603
SRR17520069.9083604
SRR17520069.9083610
SRR17520069.9083626
SRR17520069.9083630
SRR17520069.9083641
SRR17520069.9102608
SRR17520069.9102609
SRR17520069.9102610
SRR17520069.9102611
SRR17520069.9102613
SRR17520069.9102614
SRR17520069.9102615
SRR17520069.9102616
SRR17520069.9102617
SRR17520069.9102618
SRR17520069.9102619
SRR17520069.9102620
SRR17520069.9102621
SRR17520069.9102622
SRR17520069.9102623
SRR17520069.9102624
SRR17520069.9102625
SRR17520069.9102626
SRR17520069.9102627
SRR17520069.9102628
SRR17520069.9102629
SRR17520069.9102630
SRR17520069.9102631
SRR17520069.9102632
SRR17520069.9102633
SRR17520069.9102634
SRR17520069.9102635
SRR17520069.9102636
SRR17520069.9102637
SRR17520069.9102638
SRR17520069.9102639
SRR17520069.9102640
SRR17520069.9102641
SRR17520069.9102642
SRR17520069.9102643
SRR17520069.9102644

SRR17520069.9102645
SRR17520069.9102646
SRR17520069.9102647
SRR17520069.9102648
SRR17520069.9102649
SRR17520069.9102650
SRR17520069.9102651
SRR17520069.9102652
SRR17520069.9102653
SRR17520069.9102654
SRR17520069.9102655
SRR17520069.9102656
SRR17520069.9102657
SRR17520069.9102658
SRR17520069.9102659
SRR17520069.9102660
SRR17520069.9102661
SRR17520069.9102662
SRR17520069.9102663
SRR17520069.9102664
SRR17520069.9102665
SRR17520069.9102666
SRR17520069.9102667
SRR17520069.9102668
SRR17520069.9102669
SRR17520069.9102670
SRR17520069.9102671
SRR17520069.9102672
SRR17520069.9102673
SRR17520069.9102674
SRR17520069.9102675
SRR17520069.9102676
SRR17520069.9102677
SRR17520069.9102678
SRR17520069.9102679
SRR17520069.9102680
SRR17520069.9102681
SRR17520069.9102682
SRR17520069.9102683
SRR17520069.9102684
SRR17520069.9102685
SRR17520069.9102686
SRR17520069.9102687
SRR17520069.9102688
SRR17520069.9102689
SRR17520069.9102690
SRR17520069.9102691

SRR17520069.9102692
SRR17520069.9102693
SRR17520069.9102694
SRR17520069.9102695
SRR17520069.9120219
SRR17520069.9120256
SRR17520069.9134681
SRR17520069.9134682
SRR17520069.9134683
SRR17520069.9134684
SRR17520069.9134685
SRR17520069.9134686
SRR17520069.9134687
SRR17520069.9134688
SRR17520069.9134689
SRR17520069.9134690
SRR17520069.9134691
SRR17520069.9134692
SRR17520069.9134693
SRR17520069.9134694
SRR17520069.9134695
SRR17520069.9134696
SRR17520069.9134697
SRR17520069.9134698
SRR17520069.9134699
SRR17520069.9134700
SRR17520069.9134701
SRR17520069.9134702
SRR17520069.9134703
SRR17520069.9134704
SRR17520069.9134705
SRR17520069.9134706
SRR17520069.9134710
SRR17520069.9134711
SRR17520069.9134712
SRR17520069.9134713
SRR17520069.9134714
SRR17520069.9134715
SRR17520069.9134716
SRR17520069.9134717
SRR17520069.9134718
SRR17520069.9134719
SRR17520069.9157171
SRR17520069.9159659
SRR17520069.9159660
SRR17520069.9159661
SRR17520069.9159662

SRR17520069.9159663
SRR17520069.9159664
SRR17520069.9159665
SRR17520069.9159666
SRR17520069.9159667
SRR17520069.9159668
SRR17520069.9159669
SRR17520069.9159670
SRR17520069.9159671
SRR17520069.9159672
SRR17520069.9159673
SRR17520069.9159674
SRR17520069.9159675
SRR17520069.9159676
SRR17520069.9159677
SRR17520069.9159678
SRR17520069.9159679
SRR17520069.9159680
SRR17520069.9159681
SRR17520069.9159682
SRR17520069.9159683
SRR17520069.9159684
SRR17520069.9159685
SRR17520069.9159686
SRR17520069.9159687
SRR17520069.9159688
SRR17520069.9159689
SRR17520069.9159690
SRR17520069.9159691
SRR17520069.9159692
SRR17520069.9159693
SRR17520069.9159694
SRR17520069.9159696
SRR17520069.9159697
SRR17520069.9159698
SRR17520069.9159699
SRR17520069.9159700
SRR17520069.9159702
SRR17520069.9159704
SRR17520069.9159705
SRR17520069.9159706
SRR17520069.9159707
SRR17520069.9159708
SRR17520069.9159709
SRR17520069.9159710
SRR17520069.9159711
SRR17520069.9159712

SRR17520069.9159713
SRR17520069.9159714
SRR17520069.9159715
SRR17520069.9159716
SRR17520069.9159717
SRR17520069.9159718
SRR17520069.9159720
SRR17520069.9159721
SRR17520069.9159722
SRR17520069.9159723
SRR17520069.9159724
SRR17520069.9159725
SRR17520069.9159726
SRR17520069.9159727
SRR17520069.9159728
SRR17520069.9159729
SRR17520069.9159730
SRR17520069.9159731
SRR17520069.9159732
SRR17520069.9159733
SRR17520069.9159734
SRR17520069.9159735
SRR17520069.9159736
SRR17520069.9159737
SRR17520069.9159738
SRR17520069.9159739
SRR17520069.9159740
SRR17520069.9159741
SRR17520069.9159742
SRR17520069.9159743
SRR17520069.9159744
SRR17520069.9159745
SRR17520069.9159746
SRR17520069.9159747
SRR17520069.9159748
SRR17520069.9159750
SRR17520069.9159751
SRR17520069.9159752
SRR17520069.9159753
SRR17520069.9159754
SRR17520069.9159755
SRR17520069.9159756
SRR17520069.9159757
SRR17520069.9159758
SRR17520069.9159759
SRR17520069.9159760
SRR17520069.9159761

SRR17520069.9159762
SRR17520069.9159763
SRR17520069.9159765
SRR17520069.9159766
SRR17520069.9159767
SRR17520069.9159768
SRR17520069.9159769
SRR17520069.9159770
SRR17520069.9174268
SRR17520069.9178573
SRR17520069.9200732
SRR17520069.9200878
SRR17520069.9210584
SRR17520069.9210587
SRR17520069.9235319
SRR17520069.9237835
SRR17520069.9243513
SRR17520069.9243514
SRR17520069.9243515
SRR17520069.9243516
SRR17520069.9243517
SRR17520069.9243518
SRR17520069.9243520
SRR17520069.9243521
SRR17520069.9243522
SRR17520069.9243523
SRR17520069.9243525
SRR17520069.9243526
SRR17520069.9243527
SRR17520069.9243528
SRR17520069.9243529
SRR17520069.9243530
SRR17520069.9243531
SRR17520069.9243532
SRR17520069.9243533
SRR17520069.9243534
SRR17520069.9243535
SRR17520069.9243536
SRR17520069.9243537
SRR17520069.9243538
SRR17520069.9243539
SRR17520069.9243540
SRR17520069.9243541
SRR17520069.9243542
SRR17520069.9243543
SRR17520069.9243544
SRR17520069.9243545

SRR17520069.9243546
SRR17520069.9243547
SRR17520069.9243548
SRR17520069.9243549
SRR17520069.9243550
SRR17520069.9243551
SRR17520069.9243552
SRR17520069.9243553
SRR17520069.9243554
SRR17520069.9243555
SRR17520069.9243556
SRR17520069.9243557
SRR17520069.9243558
SRR17520069.9243559
SRR17520069.9243560
SRR17520069.9243561
SRR17520069.9243563
SRR17520069.9243564
SRR17520069.9243565
SRR17520069.9243566
SRR17520069.9243567
SRR17520069.9243568
SRR17520069.9243569
SRR17520069.9243570
SRR17520069.9243571
SRR17520069.9243572
SRR17520069.9243573
SRR17520069.9243574
SRR17520069.9243575
SRR17520069.9243576
SRR17520069.9243577
SRR17520069.9243578
SRR17520069.9243579
SRR17520069.9243580
SRR17520069.9243581
SRR17520069.9243582
SRR17520069.9243583
SRR17520069.9243584
SRR17520069.9243585
SRR17520069.9243586
SRR17520069.9243587
SRR17520069.9243588
SRR17520069.9243589
SRR17520069.9243590
SRR17520069.9243591
SRR17520069.9243592
SRR17520069.9243593

SRR17520069.9243594
SRR17520069.9243595
SRR17520069.9243596
SRR17520069.9243597
SRR17520069.9243598
SRR17520069.9243599
SRR17520069.9243600
SRR17520069.9243601
SRR17520069.9243602
SRR17520069.9243603
SRR17520069.9243604
SRR17520069.9243605
SRR17520069.9243606
SRR17520069.9243607
SRR17520069.9243608
SRR17520069.9243609
SRR17520069.9243610
SRR17520069.9243611
SRR17520069.9243612
SRR17520069.9243613
SRR17520069.9243614
SRR17520069.9243615
SRR17520069.9243616
SRR17520069.9243617
SRR17520069.9243618
SRR17520069.9243619
SRR17520069.9243621
SRR17520069.9243622
SRR17520069.9243623
SRR17520069.9243624
SRR17520069.9279910
SRR17520069.9279911
SRR17520069.9279912
SRR17520069.9279913
SRR17520069.9279914
SRR17520069.9279915
SRR17520069.9279916
SRR17520069.9279917
SRR17520069.9279918
SRR17520069.9279919
SRR17520069.9279921
SRR17520069.9279922
SRR17520069.9279923
SRR17520069.9279924
SRR17520069.9279925
SRR17520069.9279926
SRR17520069.9279927

SRR17520069.9279928
SRR17520069.9279929
SRR17520069.9279930
SRR17520069.9279931
SRR17520069.9279932
SRR17520069.9279933
SRR17520069.9279934
SRR17520069.9279935
SRR17520069.9279936
SRR17520069.9279937
SRR17520069.9279939
SRR17520069.9279940
SRR17520069.9279941
SRR17520069.9279942
SRR17520069.9279943
SRR17520069.9279944
SRR17520069.9279945
SRR17520069.9279946
SRR17520069.9279947
SRR17520069.9279948
SRR17520069.9279949
SRR17520069.9279950
SRR17520069.9279951
SRR17520069.9279952
SRR17520069.9279953
SRR17520069.9279954
SRR17520069.9279955
SRR17520069.9279956
SRR17520069.9279958
SRR17520069.9279960
SRR17520069.9279961
SRR17520069.9279962
SRR17520069.9279963
SRR17520069.9279964
SRR17520069.9279965
SRR17520069.9279966
SRR17520069.9279967
SRR17520069.9279968
SRR17520069.9279969
SRR17520069.9279970
SRR17520069.9279971
SRR17520069.9279972
SRR17520069.9279973
SRR17520069.9279974
SRR17520069.9279975
SRR17520069.9279976
SRR17520069.9279977

SRR17520069.9279978
SRR17520069.9279979
SRR17520069.9279980
SRR17520069.9279981
SRR17520069.9279982
SRR17520069.9279983
SRR17520069.9279984
SRR17520069.9279985
SRR17520069.9279986
SRR17520069.9279987
SRR17520069.9279988
SRR17520069.9288381
SRR17520069.9303982
SRR17520069.9303983
SRR17520069.9303984
SRR17520069.9303985
SRR17520069.9303986
SRR17520069.9303987
SRR17520069.9303988
SRR17520069.9303989
SRR17520069.9303990
SRR17520069.9303991
SRR17520069.9303992
SRR17520069.9303993
SRR17520069.9303994
SRR17520069.9303995
SRR17520069.9303996
SRR17520069.9303997
SRR17520069.9303998
SRR17520069.9303999
SRR17520069.9304000
SRR17520069.9304001
SRR17520069.9304002
SRR17520069.9304003
SRR17520069.9304004
SRR17520069.9304005
SRR17520069.9304006
SRR17520069.9304007
SRR17520069.9304008
SRR17520069.9304009
SRR17520069.9304010
SRR17520069.9304011
SRR17520069.9304012
SRR17520069.9304013
SRR17520069.9304014
SRR17520069.9304015
SRR17520069.9304016

SRR17520069.9304017
SRR17520069.9304018
SRR17520069.9304019
SRR17520069.9304020
SRR17520069.9304022
SRR17520069.9304023
SRR17520069.9304024
SRR17520069.9304025
SRR17520069.9304026
SRR17520069.9304027
SRR17520069.9304029
SRR17520069.9304030
SRR17520069.9304031
SRR17520069.9304032
SRR17520069.9304033
SRR17520069.9304034
SRR17520069.9304035
SRR17520069.9304036
SRR17520069.9304037
SRR17520069.9304038
SRR17520069.9304039
SRR17520069.9338641
SRR17520069.9338642
SRR17520069.9338643
SRR17520069.9338644
SRR17520069.9338645
SRR17520069.9338646
SRR17520069.9338647
SRR17520069.9338648
SRR17520069.9338649
SRR17520069.9338650
SRR17520069.9338651
SRR17520069.9338652
SRR17520069.9338653
SRR17520069.9338654
SRR17520069.9338655
SRR17520069.9338656
SRR17520069.9338657
SRR17520069.9338658
SRR17520069.9338659
SRR17520069.9338660
SRR17520069.9338661
SRR17520069.9338662
SRR17520069.9338663
SRR17520069.9338664
SRR17520069.9338665
SRR17520069.9338666

SRR17520069.9338667
SRR17520069.9338668
SRR17520069.9338669
SRR17520069.9338670
SRR17520069.9338672
SRR17520069.9338673
SRR17520069.9338674
SRR17520069.9338675
SRR17520069.9338676
SRR17520069.9338677
SRR17520069.9338678
SRR17520069.9338679
SRR17520069.9338680
SRR17520069.9338681
SRR17520069.9338682
SRR17520069.9338683
SRR17520069.9338684
SRR17520069.9338685
SRR17520069.9351584
SRR17520069.9351585
SRR17520069.9351586
SRR17520069.9351587
SRR17520069.9351588
SRR17520069.9351589
SRR17520069.9351590
SRR17520069.9351591
SRR17520069.9351592
SRR17520069.9351593
SRR17520069.9351594
SRR17520069.9351595
SRR17520069.9351596
SRR17520069.9351597
SRR17520069.9351598
SRR17520069.9351599
SRR17520069.9351600
SRR17520069.9351601
SRR17520069.9351602
SRR17520069.9351603
SRR17520069.9351604
SRR17520069.9351605
SRR17520069.9351606
SRR17520069.9351607
SRR17520069.9351608
SRR17520069.9351609
SRR17520069.9351610
SRR17520069.9351611
SRR17520069.9351612

SRR17520069.9351613
SRR17520069.9351614
SRR17520069.9351615
SRR17520069.9351616
SRR17520069.9351617
SRR17520069.9351618
SRR17520069.9351619
SRR17520069.9351620
SRR17520069.9351621
SRR17520069.9351622
SRR17520069.9351623
SRR17520069.9351624
SRR17520069.9351625
SRR17520069.9351626
SRR17520069.9351627
SRR17520069.9351628
SRR17520069.9351629
SRR17520069.9351630
SRR17520069.9351631
SRR17520069.9351632
SRR17520069.9351633
SRR17520069.9351634
SRR17520069.9351635
SRR17520069.9351636
SRR17520069.9351637
SRR17520069.9351638
SRR17520069.9351639
SRR17520069.9351640
SRR17520069.9351641
SRR17520069.9351642
SRR17520069.9351643
SRR17520069.9351644
SRR17520069.9351645
SRR17520069.9351646
SRR17520069.9351647
SRR17520069.9351648
SRR17520069.9351649
SRR17520069.9351650
SRR17520069.9351651
SRR17520069.9351652
SRR17520069.9351653
SRR17520069.9351654
SRR17520069.9351655
SRR17520069.9351656
SRR17520069.9351657
SRR17520069.9351658
SRR17520069.9351659

SRR17520069.9351660
SRR17520069.9351661
SRR17520069.9351662
SRR17520069.9351663
SRR17520069.9351664
SRR17520069.9351665
SRR17520069.9351666
SRR17520069.9351667
SRR17520069.9351668
SRR17520069.9351669
SRR17520069.9351670
SRR17520069.9351671
SRR17520069.9351672
SRR17520069.9351673
SRR17520069.9351675
SRR17520069.9351676
SRR17520069.9351677
SRR17520069.9351678
SRR17520069.9351679
SRR17520069.9351680
SRR17520069.9351681
SRR17520069.9351682
SRR17520069.9351683
SRR17520069.9351684
SRR17520069.9359048
SRR17520069.9359049
SRR17520069.9359050
SRR17520069.9359051
SRR17520069.9359052
SRR17520069.9359053
SRR17520069.9359054
SRR17520069.9359055
SRR17520069.9359056
SRR17520069.9359057
SRR17520069.9359058
SRR17520069.9359059
SRR17520069.9359060
SRR17520069.9359061
SRR17520069.9359062
SRR17520069.9359921
SRR17520069.9359922
SRR17520069.9359923
SRR17520069.9369995
SRR17520069.9369996
SRR17520069.9369997
SRR17520069.9369998
SRR17520069.9369999

SRR17520069.9370000
SRR17520069.9370001
SRR17520069.9370002
SRR17520069.9370003
SRR17520069.9370004
SRR17520069.9370005
SRR17520069.9370006
SRR17520069.9370007
SRR17520069.9370008
SRR17520069.9370009
SRR17520069.9370010
SRR17520069.9370011
SRR17520069.9370012
SRR17520069.9370013
SRR17520069.9370014
SRR17520069.9370015
SRR17520069.9370016
SRR17520069.9370017
SRR17520069.9370018
SRR17520069.9404815
SRR17520069.9404816
SRR17520069.9404817
SRR17520069.9404818
SRR17520069.9404819
SRR17520069.9404820
SRR17520069.9404821
SRR17520069.9404822
SRR17520069.9404823
SRR17520069.9404824
SRR17520069.9404825
SRR17520069.9404827
SRR17520069.9404828
SRR17520069.9404829
SRR17520069.9404830
SRR17520069.9404831
SRR17520069.9404832
SRR17520069.9404833
SRR17520069.9404834
SRR17520069.9404835
SRR17520069.9404836
SRR17520069.9404837
SRR17520069.9404838
SRR17520069.9404839
SRR17520069.9404840
SRR17520069.9404841
SRR17520069.9404842
SRR17520069.9404843

SRR17520069.9404844
SRR17520069.9404845
SRR17520069.9404846
SRR17520069.9404847
SRR17520069.9404848
SRR17520069.9404849
SRR17520069.9404850
SRR17520069.9404851
SRR17520069.9404852
SRR17520069.9404853
SRR17520069.9404854
SRR17520069.9404855
SRR17520069.9404861
SRR17520069.9404862
SRR17520069.9404863
SRR17520069.9404864
SRR17520069.9404865
SRR17520069.9404866
SRR17520069.9404867
SRR17520069.9404868
SRR17520069.9404869
SRR17520069.9404870
SRR17520069.9404871
SRR17520069.9404872
SRR17520069.9404874
SRR17520069.9404875
SRR17520069.9404876
SRR17520069.9404877
SRR17520069.9404878
SRR17520069.9404879
SRR17520069.9404880
SRR17520069.9404881
SRR17520069.9404882
SRR17520069.9404883
SRR17520069.9404884
SRR17520069.9404885
SRR17520069.9404886
SRR17520069.9404887
SRR17520069.9404888
SRR17520069.9404889
SRR17520069.9404890
SRR17520069.9404891
SRR17520069.9404892
SRR17520069.9404893
SRR17520069.9404894
SRR17520069.9404895
SRR17520069.9404896

SRR17520069.9404897
SRR17520069.9404898
SRR17520069.9404899
SRR17520069.9404900
SRR17520069.9404901
SRR17520069.9404902
SRR17520069.9404903
SRR17520069.9404904
SRR17520069.9404905
SRR17520069.9404906
SRR17520069.9404907
SRR17520069.9404908
SRR17520069.9404909
SRR17520069.9404910
SRR17520069.9404911
SRR17520069.9404912
SRR17520069.9404913
SRR17520069.9404914
SRR17520069.9404915
SRR17520069.9404916
SRR17520069.9404917
SRR17520069.9404918
SRR17520069.9404919
SRR17520069.9404920
SRR17520069.9404921
SRR17520069.9404922
SRR17520069.9404923
SRR17520069.9404924
SRR17520069.9404925
SRR17520069.9404926
SRR17520069.9404927
SRR17520069.9404928
SRR17520069.9404929
SRR17520069.9404930
SRR17520069.9404931
SRR17520069.9404932
SRR17520069.9404933
SRR17520069.9404934
SRR17520069.9404935
SRR17520069.9404936
SRR17520069.9404937
SRR17520069.9404938
SRR17520069.9404939
SRR17520069.9404940
SRR17520069.9404941
SRR17520069.9404942
SRR17520069.9404943

SRR17520069.9404944
SRR17520069.9404945
SRR17520069.9404946
SRR17520069.9404947
SRR17520069.9404948
SRR17520069.9404949
SRR17520069.9404950
SRR17520069.9404951
SRR17520069.9404953
SRR17520069.9404954
SRR17520069.9404955
SRR17520069.9404956
SRR17520069.9404957
SRR17520069.9404958
SRR17520069.9404959
SRR17520069.9404960
SRR17520069.9404961
SRR17520069.9404962
SRR17520069.9404963
SRR17520069.9404964
SRR17520069.9404965
SRR17520069.9404966
SRR17520069.9404967
SRR17520069.9404968
SRR17520069.9404969
SRR17520069.9404970
SRR17520069.9404971
SRR17520069.9404972
SRR17520069.9404973
SRR17520069.9404974
SRR17520069.9404975
SRR17520069.9404976
SRR17520069.9404977
SRR17520069.9404978
SRR17520069.9404979
SRR17520069.9404980
SRR17520069.9404981
SRR17520069.9404983
SRR17520069.9404984
SRR17520069.9404985
SRR17520069.9404986
SRR17520069.9404987
SRR17520069.9404988
SRR17520069.9404989
SRR17520069.9404990
SRR17520069.9404992
SRR17520069.9404993

SRR17520069.9404994
SRR17520069.9404995
SRR17520069.9404996
SRR17520069.9404997
SRR17520069.9404998
SRR17520069.9404999
SRR17520069.9405000
SRR17520069.9405001
SRR17520069.9405002
SRR17520069.9405003
SRR17520069.9405004
SRR17520069.9405005
SRR17520069.9405006
SRR17520069.9405007
SRR17520069.9405008
SRR17520069.9405009
SRR17520069.9405010
SRR17520069.9405011
SRR17520069.9405012
SRR17520069.9405013
SRR17520069.9405014
SRR17520069.9405015
SRR17520069.9405016
SRR17520069.9405017
SRR17520069.9405018
SRR17520069.9405019
SRR17520069.9405020
SRR17520069.9405021
SRR17520069.9405022
SRR17520069.9405023
SRR17520069.9405025
SRR17520069.9405026
SRR17520069.9405027
SRR17520069.9405028
SRR17520069.9405029
SRR17520069.9405030
SRR17520069.9405033
SRR17520069.9405034
SRR17520069.9405035
SRR17520069.9405036
SRR17520069.9405037
SRR17520069.9405038
SRR17520069.9405039
SRR17520069.9405040
SRR17520069.9405041
SRR17520069.9405042
SRR17520069.9405043

SRR17520069.9405044
SRR17520069.9405045
SRR17520069.9405046
SRR17520069.9405047
SRR17520069.9405048
SRR17520069.9405049
SRR17520069.9405050
SRR17520069.9405051
SRR17520069.9405052
SRR17520069.9405053
SRR17520069.9405054
SRR17520069.9405055
SRR17520069.9405056
SRR17520069.9405057
SRR17520069.9405058
SRR17520069.9405059
SRR17520069.9405060
SRR17520069.9405061
SRR17520069.9405063
SRR17520069.9428395
SRR17520069.9428396
SRR17520069.9428398
SRR17520069.9428399
SRR17520069.9428400
SRR17520069.9428401
SRR17520069.9428402
SRR17520069.9428403
SRR17520069.9428404
SRR17520069.9428405
SRR17520069.9428406
SRR17520069.9428407
SRR17520069.9428408
SRR17520069.9428409
SRR17520069.9428410
SRR17520069.9428411
SRR17520069.9428412
SRR17520069.9428413
SRR17520069.9428414
SRR17520069.9428415
SRR17520069.9428416
SRR17520069.9428417
SRR17520069.9428816
SRR17520069.9442271
SRR17520069.9442272
SRR17520069.9442273
SRR17520069.9442274
SRR17520069.9442275

SRR17520069.9442280
SRR17520069.9442281
SRR17520069.9442282
SRR17520069.9442283
SRR17520069.9442284
SRR17520069.9442285
SRR17520069.9451355
SRR17520069.9471367
SRR17520069.9471389
SRR17520069.9471422
SRR17520069.9472209
SRR17520069.9472213
SRR17520069.9479177
SRR17520069.9489301
SRR17520069.9489303
SRR17520069.9489304
SRR17520069.9489305
SRR17520069.9489306
SRR17520069.9489307
SRR17520069.9489308
SRR17520069.9489309
SRR17520069.9489310
SRR17520069.9489311
SRR17520069.9489312
SRR17520069.9489313
SRR17520069.9489314
SRR17520069.9489315
SRR17520069.9489316
SRR17520069.9489317
SRR17520069.9489318
SRR17520069.9489319
SRR17520069.9489320
SRR17520069.9489321
SRR17520069.9489322
SRR17520069.9489323
SRR17520069.9489324
SRR17520069.9489325
SRR17520069.9489326
SRR17520069.9489327
SRR17520069.9489328
SRR17520069.9489329
SRR17520069.9489330
SRR17520069.9489331
SRR17520069.9489332
SRR17520069.9489333
SRR17520069.9489334
SRR17520069.9489335

SRR17520069.9489336
SRR17520069.9489337
SRR17520069.9489338
SRR17520069.9489339
SRR17520069.9489340
SRR17520069.9489341
SRR17520069.9489342
SRR17520069.9489343
SRR17520069.9489344
SRR17520069.9489345
SRR17520069.9489346
SRR17520069.9489347
SRR17520069.9489348
SRR17520069.9489349
SRR17520069.9489350
SRR17520069.9489351
SRR17520069.9489352
SRR17520069.9489353
SRR17520069.9489354
SRR17520069.9489355
SRR17520069.9489356
SRR17520069.9489357
SRR17520069.9489358
SRR17520069.9489359
SRR17520069.9489360
SRR17520069.9489361
SRR17520069.9489362
SRR17520069.9489363
SRR17520069.9489364
SRR17520069.9489365
SRR17520069.9489366
SRR17520069.9489367
SRR17520069.9489368
SRR17520069.9489369
SRR17520069.9489370
SRR17520069.9489371
SRR17520069.9489372
SRR17520069.9489373
SRR17520069.9489374
SRR17520069.9489375
SRR17520069.9489376
SRR17520069.9489377
SRR17520069.9489378
SRR17520069.9489380
SRR17520069.9489381
SRR17520069.9489382
SRR17520069.9489383

SRR17520069.9489384
SRR17520069.9489385
SRR17520069.9489387
SRR17520069.9489388
SRR17520069.9489390
SRR17520069.9489391
SRR17520069.9489392
SRR17520069.9489394
SRR17520069.9489395
SRR17520069.9489396
SRR17520069.9489397
SRR17520069.9489398
SRR17520069.9489399
SRR17520069.9489400
SRR17520069.9489401
SRR17520069.9489402
SRR17520069.9489403
SRR17520069.9489404
SRR17520069.9489405
SRR17520069.9489406
SRR17520069.9489407
SRR17520069.9489408
SRR17520069.9489995
SRR17520069.9489996
SRR17520069.9489999
SRR17520069.9490000
SRR17520069.9490003
SRR17520069.9490004
SRR17520069.9490005
SRR17520069.9490006
SRR17520069.9490007
SRR17520069.9490008
SRR17520069.9490009
SRR17520069.9490010
SRR17520069.9490011
SRR17520069.9490012
SRR17520069.9490013
SRR17520069.9490014
SRR17520069.9490015
SRR17520069.9490016
SRR17520069.9490017
SRR17520069.9490018
SRR17520069.9490019
SRR17520069.9490020
SRR17520069.9490021
SRR17520069.9490022
SRR17520069.9490023

SRR17520069.9490024
SRR17520069.9490025
SRR17520069.9490026
SRR17520069.9490027
SRR17520069.9490028
SRR17520069.9490029
SRR17520069.9490030
SRR17520069.9490031
SRR17520069.9490032
SRR17520069.9490033
SRR17520069.9490034
SRR17520069.9490035
SRR17520069.9490036
SRR17520069.9490037
SRR17520069.9490038
SRR17520069.9490039
SRR17520069.9490040
SRR17520069.9490042
SRR17520069.9490043
SRR17520069.9490044
SRR17520069.9490045
SRR17520069.9490046
SRR17520069.9490047
SRR17520069.9490048
SRR17520069.9490049
SRR17520069.9490050
SRR17520069.9490051
SRR17520069.9490052
SRR17520069.9490053
SRR17520069.9490054
SRR17520069.9490055
SRR17520069.9490056
SRR17520069.9490057
SRR17520069.9490058
SRR17520069.9490059
SRR17520069.9490060
SRR17520069.9490061
SRR17520069.9490062
SRR17520069.9490063
SRR17520069.9490064
SRR17520069.9490065
SRR17520069.9490066
SRR17520069.9490067
SRR17520069.9490068
SRR17520069.9490069
SRR17520069.9490070
SRR17520069.9490071

SRR17520069.9490072
SRR17520069.9490073
SRR17520069.9490075
SRR17520069.9490076
SRR17520069.9490077
SRR17520069.9490078
SRR17520069.9490079
SRR17520069.9490080
SRR17520069.9490081
SRR17520069.9490082
SRR17520069.9490083
SRR17520069.9490084
SRR17520069.9490085
SRR17520069.9490086
SRR17520069.9490087
SRR17520069.9490088
SRR17520069.9490089
SRR17520069.9490090
SRR17520069.9490091
SRR17520069.9490092
SRR17520069.9490093
SRR17520069.9490094
SRR17520069.9490095
SRR17520069.9490096
SRR17520069.9490097
SRR17520069.9490098
SRR17520069.9490099
SRR17520069.9490100
SRR17520069.9490101
SRR17520069.9490102
SRR17520069.9490103
SRR17520069.9490104
SRR17520069.9490105
SRR17520069.9490106
SRR17520069.9490107
SRR17520069.9490108
SRR17520069.9490109
SRR17520069.9490110
SRR17520069.9490111
SRR17520069.9490112
SRR17520069.9490113
SRR17520069.9490114
SRR17520069.9490115
SRR17520069.9490116
SRR17520069.9490117
SRR17520069.9490118
SRR17520069.9490120

SRR17520069.9490121
SRR17520069.9490122
SRR17520069.9490123
SRR17520069.9490124
SRR17520069.9490125
SRR17520069.9490126
SRR17520069.9505204
SRR17520069.9505205
SRR17520069.9505206
SRR17520069.9505207
SRR17520069.9505208
SRR17520069.9505209
SRR17520069.9505210
SRR17520069.9505211
SRR17520069.9505212
SRR17520069.9505213
SRR17520069.9505214
SRR17520069.9505215
SRR17520069.9505216
SRR17520069.9505217
SRR17520069.9505218
SRR17520069.9505219
SRR17520069.9505220
SRR17520069.9505221
SRR17520069.9505222
SRR17520069.9505223
SRR17520069.9505225
SRR17520069.9505226
SRR17520069.9505227
SRR17520069.9505228
SRR17520069.9505229
SRR17520069.9505230
SRR17520069.9505231
SRR17520069.9505232
SRR17520069.9505233
SRR17520069.9505234
SRR17520069.9505235
SRR17520069.9505236
SRR17520069.9505237
SRR17520069.9505238
SRR17520069.9505239
SRR17520069.9505240
SRR17520069.9505241
SRR17520069.9505242
SRR17520069.9505243
SRR17520069.9505244
SRR17520069.9505245

SRR17520069.9505246
SRR17520069.9505247
SRR17520069.9505248
SRR17520069.9505249
SRR17520069.9505250
SRR17520069.9505252
SRR17520069.9505253
SRR17520069.9505254
SRR17520069.9505255
SRR17520069.9505256
SRR17520069.9505257
SRR17520069.9505258
SRR17520069.9505259
SRR17520069.9505260
SRR17520069.9505261
SRR17520069.9505262
SRR17520069.9505263
SRR17520069.9505264
SRR17520069.9505265
SRR17520069.9505266
SRR17520069.9505267
SRR17520069.9505268
SRR17520069.9505269
SRR17520069.9505270
SRR17520069.9505271
SRR17520069.9505272
SRR17520069.9505273
SRR17520069.9505274
SRR17520069.9505275
SRR17520069.9505276
SRR17520069.9505277
SRR17520069.9505278
SRR17520069.9505279
SRR17520069.9505280
SRR17520069.9505281
SRR17520069.9505282
SRR17520069.9505286
SRR17520069.9505289
SRR17520069.9505292
SRR17520069.9505294
SRR17520069.9505309
SRR17520069.9505314
SRR17520069.9505315
SRR17520069.9505318
SRR17520069.9505325
SRR17520069.9517088
SRR17520069.9517089

SRR17520069.9517090
SRR17520069.9517091
SRR17520069.9517092
SRR17520069.9517093
SRR17520069.9517094
SRR17520069.9517095
SRR17520069.9517096
SRR17520069.9517097
SRR17520069.9517098
SRR17520069.9517099
SRR17520069.9517100
SRR17520069.9517101
SRR17520069.9517102
SRR17520069.9517103
SRR17520069.9517104
SRR17520069.9517105
SRR17520069.9517106
SRR17520069.9517107
SRR17520069.9517108
SRR17520069.9517109
SRR17520069.9517110
SRR17520069.9517111
SRR17520069.9517112
SRR17520069.9517113
SRR17520069.9517114
SRR17520069.9517115
SRR17520069.9517116
SRR17520069.9517117
SRR17520069.9517118
SRR17520069.9517119
SRR17520069.9517120
SRR17520069.9517121
SRR17520069.9517122
SRR17520069.9517123
SRR17520069.9517124
SRR17520069.9517125
SRR17520069.9517126
SRR17520069.9517127
SRR17520069.9517128
SRR17520069.9517129
SRR17520069.9517130
SRR17520069.9522555
SRR17520069.9525618
SRR17520069.9531853
SRR17520069.9532791
SRR17520069.9532792
SRR17520069.9532793

SRR17520069.9532794
SRR17520069.9532795
SRR17520069.9532796
SRR17520069.9532797
SRR17520069.9532798
SRR17520069.9532799
SRR17520069.9532800
SRR17520069.9532801
SRR17520069.9532802
SRR17520069.9532803
SRR17520069.9532804
SRR17520069.9532805
SRR17520069.9532806
SRR17520069.9532807
SRR17520069.9532808
SRR17520069.9537270
SRR17520069.9537271
SRR17520069.9537272
SRR17520069.9537273
SRR17520069.9537274
SRR17520069.9537275
SRR17520069.9537276
SRR17520069.9537277
SRR17520069.9537278
SRR17520069.9537279
SRR17520069.9537280
SRR17520069.9537281
SRR17520069.9537284
SRR17520069.9537285
SRR17520069.9537286
SRR17520069.9537287
SRR17520069.9537288
SRR17520069.9537289
SRR17520069.9537290
SRR17520069.9537291
SRR17520069.9537292
SRR17520069.9537293
SRR17520069.9537294
SRR17520069.9537295
SRR17520069.9537296
SRR17520069.9537297
SRR17520069.9537298
SRR17520069.9537299
SRR17520069.9537300
SRR17520069.9537301
SRR17520069.9537302
SRR17520069.9537303

SRR17520069.9537304
SRR17520069.9537305
SRR17520069.9537306
SRR17520069.9537307
SRR17520069.9537308
SRR17520069.9537309
SRR17520069.9537310
SRR17520069.9537311
SRR17520069.9537312
SRR17520069.9537313
SRR17520069.9537314
SRR17520069.9537315
SRR17520069.9537317
SRR17520069.9537318
SRR17520069.9537319
SRR17520069.9537320
SRR17520069.9537321
SRR17520069.9537322
SRR17520069.9537325
SRR17520069.9537326
SRR17520069.9537327
SRR17520069.9537328
SRR17520069.9537329
SRR17520069.9537330
SRR17520069.9537331
SRR17520069.9537332
SRR17520069.9537333
SRR17520069.9537334
SRR17520069.9537335
SRR17520069.9537337
SRR17520069.9537338
SRR17520069.9537339
SRR17520069.9537340
SRR17520069.9537341
SRR17520069.9537342
SRR17520069.9537343
SRR17520069.9537344
SRR17520069.9537345
SRR17520069.9537346
SRR17520069.9537347
SRR17520069.9537348
SRR17520069.9537349
SRR17520069.9537350
SRR17520069.9537351
SRR17520069.9537352
SRR17520069.9537353
SRR17520069.9537354

SRR17520069.9537355
SRR17520069.9537356
SRR17520069.9537357
SRR17520069.9537358
SRR17520069.9537359
SRR17520069.9537360
SRR17520069.9537361
SRR17520069.9537362
SRR17520069.9537363
SRR17520069.9537364
SRR17520069.9537365
SRR17520069.9537366
SRR17520069.9537367
SRR17520069.9537368
SRR17520069.9541771
SRR17520069.9541876
SRR17520069.9541907
SRR17520069.9566019
SRR17520069.9566020
SRR17520069.9566021
SRR17520069.9566022
SRR17520069.9566023
SRR17520069.9566024
SRR17520069.9566025
SRR17520069.9566026
SRR17520069.9566027
SRR17520069.9566028
SRR17520069.9566029
SRR17520069.9566030
SRR17520069.9566031
SRR17520069.9566032
SRR17520069.9566033
SRR17520069.9566034
SRR17520069.9566035
SRR17520069.9566036
SRR17520069.9566037
SRR17520069.9566038
SRR17520069.9566039
SRR17520069.9566040
SRR17520069.9566041
SRR17520069.9566042
SRR17520069.9566043
SRR17520069.9566044
SRR17520069.9566045
SRR17520069.9566046
SRR17520069.9566047
SRR17520069.9566048

SRR17520069.9566049
SRR17520069.9566050
SRR17520069.9566051
SRR17520069.9566052
SRR17520069.9566053
SRR17520069.9566054
SRR17520069.9566055
SRR17520069.9566056
SRR17520069.9566057
SRR17520069.9566058
SRR17520069.9566059
SRR17520069.9566060
SRR17520069.9566061
SRR17520069.9566062
SRR17520069.9566063
SRR17520069.9566065
SRR17520069.9566066
SRR17520069.9566067
SRR17520069.9566068
SRR17520069.9566069
SRR17520069.9566070
SRR17520069.9566071
SRR17520069.9566072
SRR17520069.9566073
SRR17520069.9566074
SRR17520069.9566075
SRR17520069.9566076
SRR17520069.9566077
SRR17520069.9566078
SRR17520069.9566079
SRR17520069.9566080
SRR17520069.9566081
SRR17520069.9566082
SRR17520069.9566083
SRR17520069.9566084
SRR17520069.9566085
SRR17520069.9566086
SRR17520069.9566087
SRR17520069.9566088
SRR17520069.9566089
SRR17520069.9566090
SRR17520069.9566091
SRR17520069.9566092
SRR17520069.9566093
SRR17520069.9566094
SRR17520069.9566095
SRR17520069.9566096

SRR17520069.9566097
SRR17520069.9566098
SRR17520069.9566099
SRR17520069.9566100
SRR17520069.9566101
SRR17520069.9566102
SRR17520069.9566103
SRR17520069.9566104
SRR17520069.9566105
SRR17520069.9566106
SRR17520069.9566107
SRR17520069.9566108
SRR17520069.9566109
SRR17520069.9571717
SRR17520069.9571728
SRR17520069.9588893
SRR17520069.9588897
SRR17520069.9588898
SRR17520069.9588900
SRR17520069.9598567
SRR17520069.9598570
SRR17520069.9598574
SRR17520069.9598578
SRR17520069.9598582
SRR17520069.9598588
SRR17520069.9598594
SRR17520069.9598595
SRR17520069.9598600
SRR17520069.9598603
SRR17520069.9598604
SRR17520069.9598605
SRR17520069.9598607
SRR17520069.9609065
SRR17520069.9623068
SRR17520069.9641129
SRR17520069.9641130
SRR17520069.9641131
SRR17520069.9641132
SRR17520069.9641133
SRR17520069.9641134
SRR17520069.9641136
SRR17520069.9641137
SRR17520069.9641138
SRR17520069.9641139
SRR17520069.9641140
SRR17520069.9641141
SRR17520069.9641142

SRR17520069.9641143
SRR17520069.9641144
SRR17520069.9641145
SRR17520069.9641146
SRR17520069.9641147
SRR17520069.9641148
SRR17520069.9656184
SRR17520069.9657901
SRR17520069.9667837
SRR17520069.9667838
SRR17520069.9667839
SRR17520069.9667840
SRR17520069.9667841
SRR17520069.9667842
SRR17520069.9667843
SRR17520069.9667844
SRR17520069.9667846
SRR17520069.9667847
SRR17520069.9667848
SRR17520069.9667849
SRR17520069.9667850
SRR17520069.9667851
SRR17520069.9667852
SRR17520069.9667853
SRR17520069.9667854
SRR17520069.9667855
SRR17520069.9667856
SRR17520069.9667857
SRR17520069.9667858
SRR17520069.9667859
SRR17520069.9667860
SRR17520069.9667861
SRR17520069.9667862
SRR17520069.9667863
SRR17520069.9667864
SRR17520069.9667865
SRR17520069.9667866
SRR17520069.9667867
SRR17520069.9667868
SRR17520069.9667869
SRR17520069.9667870
SRR17520069.9667871
SRR17520069.9667872
SRR17520069.9667873
SRR17520069.9667874
SRR17520069.9667875
SRR17520069.9667876

SRR17520069.9667877
SRR17520069.9667878
SRR17520069.9667879
SRR17520069.9667881
SRR17520069.9667882
SRR17520069.9667883
SRR17520069.9667884
SRR17520069.9667885
SRR17520069.9667886
SRR17520069.9667887
SRR17520069.9667888
SRR17520069.9667889
SRR17520069.9667890
SRR17520069.9667891
SRR17520069.9667892
SRR17520069.9667893
SRR17520069.9667894
SRR17520069.9667895
SRR17520069.9667896
SRR17520069.9667897
SRR17520069.9667898
SRR17520069.9667899
SRR17520069.9667900
SRR17520069.9667901
SRR17520069.9667902
SRR17520069.9667903
SRR17520069.9667904
SRR17520069.9667905
SRR17520069.9667906
SRR17520069.9667907
SRR17520069.9667908
SRR17520069.9667909
SRR17520069.9667910
SRR17520069.9667911
SRR17520069.9667912
SRR17520069.9667913
SRR17520069.9667914
SRR17520069.9667915
SRR17520069.9667916
SRR17520069.9667917
SRR17520069.9667918
SRR17520069.9667919
SRR17520069.9667920
SRR17520069.9667921
SRR17520069.9667922
SRR17520069.9667923
SRR17520069.9667924

SRR17520069.9667925
SRR17520069.9694815
SRR17520069.9694816
SRR17520069.9694817
SRR17520069.9694818
SRR17520069.9694819
SRR17520069.9694821
SRR17520069.9694822
SRR17520069.9694823
SRR17520069.9694824
SRR17520069.9694825
SRR17520069.9694826
SRR17520069.9694827
SRR17520069.9694828
SRR17520069.9694829
SRR17520069.9694830
SRR17520069.9694831
SRR17520069.9694832
SRR17520069.9694833
SRR17520069.9694834
SRR17520069.9694835
SRR17520069.9694836
SRR17520069.9694837
SRR17520069.9694838
SRR17520069.9694839
SRR17520069.9694840
SRR17520069.9694841
SRR17520069.9694842
SRR17520069.9694843
SRR17520069.9694844
SRR17520069.9694845
SRR17520069.9694846
SRR17520069.9694847
SRR17520069.9694849
SRR17520069.9694850
SRR17520069.9694851
SRR17520069.9694852
SRR17520069.9694853
SRR17520069.9694854
SRR17520069.9694855
SRR17520069.9694856
SRR17520069.9694857
SRR17520069.9694858
SRR17520069.9694859
SRR17520069.9694860
SRR17520069.9694861
SRR17520069.9694862

SRR17520069.9694863
SRR17520069.9694864
SRR17520069.9694865
SRR17520069.9694866
SRR17520069.9694867
SRR17520069.9694868
SRR17520069.9694869
SRR17520069.9694870
SRR17520069.9694871
SRR17520069.9694872
SRR17520069.9694873
SRR17520069.9694874
SRR17520069.9694875
SRR17520069.9694876
SRR17520069.9694877
SRR17520069.9694878
SRR17520069.9694879
SRR17520069.9694880
SRR17520069.9694881
SRR17520069.9694882
SRR17520069.9694883
SRR17520069.9694884
SRR17520069.9694885
SRR17520069.9694886
SRR17520069.9694887
SRR17520069.9694888
SRR17520069.9694889
SRR17520069.9694890
SRR17520069.9694891
SRR17520069.9694892
SRR17520069.9694893
SRR17520069.9694894
SRR17520069.9694895
SRR17520069.9694896
SRR17520069.9694897
SRR17520069.9694898
SRR17520069.9694899
SRR17520069.9694900
SRR17520069.9694901
SRR17520069.9694902
SRR17520069.9694903
SRR17520069.9694904
SRR17520069.9694905
SRR17520069.9694906
SRR17520069.9694907
SRR17520069.9694908
SRR17520069.9694909

SRR17520069.9694910
SRR17520069.9694911
SRR17520069.9694912
SRR17520069.9694913
SRR17520069.9694914
SRR17520069.9694915
SRR17520069.9694916
SRR17520069.9694917
SRR17520069.9694918
SRR17520069.9694919
SRR17520069.9694920
SRR17520069.9694921
SRR17520069.9694922
SRR17520069.9694923
SRR17520069.9694924
SRR17520069.9699138
SRR17520069.9711880
SRR17520069.9711881
SRR17520069.9711882
SRR17520069.9711883
SRR17520069.9711884
SRR17520069.9711885
SRR17520069.9711886
SRR17520069.9711887
SRR17520069.9711888
SRR17520069.9711889
SRR17520069.9711890
SRR17520069.9711891
SRR17520069.9711892
SRR17520069.9711893
SRR17520069.9711894
SRR17520069.9711895
SRR17520069.9711896
SRR17520069.9711897
SRR17520069.9711899
SRR17520069.9711900
SRR17520069.9711901
SRR17520069.9711902
SRR17520069.9711903
SRR17520069.9711904
SRR17520069.9711905
SRR17520069.9711906
SRR17520069.9711908
SRR17520069.9711909
SRR17520069.9711911
SRR17520069.9711912
SRR17520069.9711913

SRR17520069.9711914
SRR17520069.9711915
SRR17520069.9711916
SRR17520069.9711917
SRR17520069.9711918
SRR17520069.9711919
SRR17520069.9711920
SRR17520069.9711921
SRR17520069.9711922
SRR17520069.9711923
SRR17520069.9711924
SRR17520069.9711925
SRR17520069.9711926
SRR17520069.9711927
SRR17520069.9711928
SRR17520069.9711929
SRR17520069.9711930
SRR17520069.9711931
SRR17520069.9711932
SRR17520069.9711933
SRR17520069.9711934
SRR17520069.9711935
SRR17520069.9711936
SRR17520069.9711937
SRR17520069.9711938
SRR17520069.9711939
SRR17520069.9711940
SRR17520069.9711941
SRR17520069.9711942
SRR17520069.9711943
SRR17520069.9711944
SRR17520069.9711945
SRR17520069.9711946
SRR17520069.9711947
SRR17520069.9711948
SRR17520069.9711949
SRR17520069.9711950
SRR17520069.9711951
SRR17520069.9711952
SRR17520069.9711953
SRR17520069.9711954
SRR17520069.9711955
SRR17520069.9711956
SRR17520069.9711957
SRR17520069.9711958
SRR17520069.9711959
SRR17520069.9711960

SRR17520069.9711961
SRR17520069.9711962
SRR17520069.9711963
SRR17520069.9711964
SRR17520069.9711965
SRR17520069.9711966
SRR17520069.9711967
SRR17520069.9711968
SRR17520069.9711969
SRR17520069.9711970
SRR17520069.9711971
SRR17520069.9711972
SRR17520069.9711973
SRR17520069.9711974
SRR17520069.9711975
SRR17520069.9711976
SRR17520069.9711977
SRR17520069.9711978
SRR17520069.9711979
SRR17520069.9711980
SRR17520069.9711981
SRR17520069.9711982
SRR17520069.9711983
SRR17520069.9711984
SRR17520069.9711985
SRR17520069.9711986
SRR17520069.9711987
SRR17520069.9711988
SRR17520069.9711989
SRR17520069.9711990
SRR17520069.9711991
SRR17520069.9711992
SRR17520069.9711993
SRR17520069.9711994
SRR17520069.9711995
SRR17520069.9711996
SRR17520069.9711998
SRR17520069.9713267
SRR17520069.9741208
SRR17520069.9741209
SRR17520069.9741210
SRR17520069.9741211
SRR17520069.9741212
SRR17520069.9741213
SRR17520069.9741214
SRR17520069.9741215
SRR17520069.9741216

SRR17520069.9741217
SRR17520069.9741218
SRR17520069.9741219
SRR17520069.9741220
SRR17520069.9741221
SRR17520069.9741222
SRR17520069.9741223
SRR17520069.9741224
SRR17520069.9741225
SRR17520069.9741226
SRR17520069.9741227
SRR17520069.9741228
SRR17520069.9741229
SRR17520069.9741230
SRR17520069.9741231
SRR17520069.9741232
SRR17520069.9741233
SRR17520069.9741234
SRR17520069.9741235
SRR17520069.9741236
SRR17520069.9741237
SRR17520069.9741238
SRR17520069.9753041
SRR17520069.9753042
SRR17520069.9753043
SRR17520069.9753044
SRR17520069.9753045
SRR17520069.9753046
SRR17520069.9753047
SRR17520069.9753049
SRR17520069.9753050
SRR17520069.9753051
SRR17520069.9753052
SRR17520069.9753053
SRR17520069.9753054
SRR17520069.9764877
SRR17520069.9793244
SRR17520069.9793245
SRR17520069.9793246
SRR17520069.9793247
SRR17520069.9793248
SRR17520069.9793249
SRR17520069.9793250
SRR17520069.9793251
SRR17520069.9793252
SRR17520069.9793253
SRR17520069.9793254

SRR17520069.9793255
SRR17520069.9793256
SRR17520069.9793257
SRR17520069.9793258
SRR17520069.9793259
SRR17520069.9793260
SRR17520069.9793261
SRR17520069.9793262
SRR17520069.9793263
SRR17520069.9793264
SRR17520069.9793265
SRR17520069.9793266
SRR17520069.9793267
SRR17520069.9793268
SRR17520069.9793269
SRR17520069.9793270
SRR17520069.9793272
SRR17520069.9793273
SRR17520069.9793274
SRR17520069.9793275
SRR17520069.9802373
SRR17520069.9813467
SRR17520069.9813468
SRR17520069.9813469
SRR17520069.9813470
SRR17520069.9813471
SRR17520069.9813472
SRR17520069.9813477
SRR17520069.9813479
SRR17520069.9813480
SRR17520069.9813481
SRR17520069.9813482
SRR17520069.9813485
SRR17520069.9813486
SRR17520069.9843005
SRR17520069.9843006
SRR17520069.9843007
SRR17520069.9843008
SRR17520069.9843009
SRR17520069.9843010
SRR17520069.9843011
SRR17520069.9843012
SRR17520069.9843013
SRR17520069.9843014
SRR17520069.9843015
SRR17520069.9843016
SRR17520069.9843017

SRR17520069.9843018
SRR17520069.9843019
SRR17520069.9848901
SRR17520069.9848902
SRR17520069.9848903
SRR17520069.9848904
SRR17520069.9848905
SRR17520069.9848906
SRR17520069.9848907
SRR17520069.9848908
SRR17520069.9848909
SRR17520069.9848910
SRR17520069.9848911
SRR17520069.9848912
SRR17520069.9848913
SRR17520069.9848914
SRR17520069.9848915
SRR17520069.9848916
SRR17520069.9848917
SRR17520069.9848918
SRR17520069.9848919
SRR17520069.9848920
SRR17520069.9848921
SRR17520069.9848922
SRR17520069.9848923
SRR17520069.9848924
SRR17520069.9848925
SRR17520069.9848926
SRR17520069.9848927
SRR17520069.9848928
SRR17520069.9848929
SRR17520069.9848930
SRR17520069.9848931
SRR17520069.9848932
SRR17520069.9848933
SRR17520069.9848934
SRR17520069.9848935
SRR17520069.9848936
SRR17520069.9848937
SRR17520069.9848938
SRR17520069.9848939
SRR17520069.9848940
SRR17520069.9848941
SRR17520069.9848942
SRR17520069.9848944
SRR17520069.9848945
SRR17520069.9848946

SRR17520069.9848947
SRR17520069.9848948
SRR17520069.9848949
SRR17520069.9849170
SRR17520069.9849171
SRR17520069.9849172
SRR17520069.9908546
SRR17520069.9920443
SRR17520069.9920444
SRR17520069.9920445
SRR17520069.9920446
SRR17520069.9920447
SRR17520069.9920448
SRR17520069.9920450
SRR17520069.9920451
SRR17520069.9920452
SRR17520069.9920453
SRR17520069.9920455
SRR17520069.9920456
SRR17520069.9920457
SRR17520069.9920458
SRR17520069.9920459
SRR17520069.9920460
SRR17520069.9920461
SRR17520069.9920462
SRR17520069.9920463
SRR17520069.9920464
SRR17520069.9920465
SRR17520069.9930205
SRR17520069.9934555
SRR17520069.9953048
SRR17520069.9953049
SRR17520069.9953050
SRR17520069.9953051
SRR17520069.9953444
SRR17520069.9953445
SRR17520069.9953446
SRR17520069.9953447
SRR17520069.9953448
SRR17520069.9953449
SRR17520069.9953450
SRR17520069.9953451
SRR17520069.9953452
SRR17520069.9953453
SRR17520069.9953454
SRR17520069.9953455
SRR17520069.9953456

SRR17520069.9953457
SRR17520069.9953458
SRR17520069.9953459
SRR17520069.9953460
SRR17520069.9953461
SRR17520069.9953462
SRR17520069.9953463
SRR17520069.9953464
SRR17520069.9953465
SRR17520069.9953466
SRR17520069.9953467
SRR17520069.9953468
SRR17520069.9953470
SRR17520069.9953471
SRR17520069.9953472
SRR17520069.9953474
SRR17520069.9953475
SRR17520069.9953476
SRR17520069.9953477
SRR17520069.9982563
SRR17520069.9983751
SRR17520069.9983754
SRR17520069.9983755
SRR17520069.9983756
SRR17520069.9983757
SRR17520069.9983758
SRR17520069.9983759
SRR17520069.9983760
SRR17520069.9983761
SRR17520069.9983762
SRR17520069.9983765
SRR17520069.9983766
SRR17520069.9983768
SRR17520069.9983769
SRR17520069.9983770
SRR17520069.9983771
SRR17520069.9983772
SRR17520069.9983773
SRR17520069.9983774
SRR17520069.9983775
SRR17520069.9983776
SRR17520069.9983778
SRR17520069.9983779
SRR17520069.9983780
SRR17520069.9983782
SRR17520069.9983783
SRR17520069.9983787

SRR17520069.9983789
SRR17520069.9983791
SRR17520069.9983792
SRR17520069.9983793
SRR17520069.9983794
SRR17520069.9983795
SRR17520069.9983796
SRR17520069.9983797
SRR17520069.9996777
SRR17520069.9996778
SRR17520069.9996779
SRR17520069.9996780
SRR17520069.9996781
SRR17520069.9996782
SRR17520069.9996783
SRR17520069.9996784
SRR17520069.9996785
SRR17520069.9996786
SRR17520069.9996787
SRR17520069.9996788
SRR17520069.9996789
SRR17520069.9996799
SRR17520069.9996800
SRR17520069.9996802
SRR17520069.9996811
SRR17520069.9996813
SRR17520069.9996816
SRR17520069.9997038
SRR17520069.9997039
SRR17520069.9997040
SRR17520069.9997041
SRR17520069.9997042
SRR17520069.9997043
SRR17520069.9997044
SRR17520069.9997045
SRR17520069.9997046
SRR17520069.9997047
SRR17520069.9997048
SRR17520069.9997049
SRR17520069.9997050
SRR17520069.9997051
SRR17520069.9997052
SRR17520069.9997053
SRR17520069.9997054
SRR17520069.9997055
SRR17520069.9997056
SRR17520069.9997057

SRR17520069.9997058
SRR17520069.9997059
SRR17520069.9997061
SRR17520069.9997062
SRR17520069.9997063
SRR17520069.9997064
SRR17520069.9997065
SRR17520069.9997066
SRR17520069.9997067
SRR17520069.9997068
SRR17520069.9997069
SRR17520069.9997070
SRR17520069.9997071
SRR17520069.9997072
SRR17520069.9997073
SRR17520069.9997074
SRR17520069.9997075
SRR17520069.9997076
SRR17520069.9997077
SRR17520069.9997078
SRR17520069.9997079
SRR17520069.9997080
SRR17520069.9997081
SRR17520069.9997082
SRR17520069.9997083
SRR17520069.9997085
SRR17520069.9997086
SRR17520069.9997087
SRR17520069.9997088
SRR17520069.9997089
SRR17520069.9997090
SRR17520069.9997091
SRR17520069.9997093
SRR17520069.9997094
SRR17520069.9997095
SRR17520069.9997096
SRR17520069.9997097
SRR17520069.9997098
SRR17520069.9997099
SRR17520069.9997100
SRR17520069.9997101
SRR17520069.9997102
SRR17520069.9997103
SRR17520069.9997104
SRR17520069.9997105
SRR17520069.9997106
SRR17520069.9997107

SRR17520069.9997108
SRR17520069.9997109
SRR17520069.9997110
SRR17520069.9997111
SRR17520069.9997112
SRR17520069.9997113
SRR17520069.9997115
SRR17520069.9997116
SRR17520069.9997117
SRR17520069.9997118
SRR17520069.9997119
SRR17520069.9997120
SRR17520069.9997121
SRR17520069.9997122
SRR17520069.9997123
SRR17520069.9997124
SRR17520069.9997125
SRR17520069.9997126
SRR17520069.9997127
SRR17520069.9997128
SRR17520069.9997129
SRR17520069.9997131
SRR17520069.9997132
SRR17520069.9997133
SRR17520069.9997134
SRR17520069.9997135
SRR17520069.9997136
SRR17520069.9997137
SRR17520069.9997138
SRR17520069.9997139
SRR17520069.9997140
SRR17520069.9997141
SRR17520069.10030174
SRR17520069.10030175
SRR17520069.10030176
SRR17520069.10030177
SRR17520069.10030179
SRR17520069.10030180
SRR17520069.10030181
SRR17520069.10030182
SRR17520069.10030183
SRR17520069.10030184
SRR17520069.10030185
SRR17520069.10030186
SRR17520069.10030187
SRR17520069.10030188
SRR17520069.10030189

SRR17520069.10030190
SRR17520069.10030192
SRR17520069.10030193
SRR17520069.10030194
SRR17520069.10030195
SRR17520069.10030196
SRR17520069.10030197
SRR17520069.10030198
SRR17520069.10030199
SRR17520069.10030200
SRR17520069.10030201
SRR17520069.10030202
SRR17520069.10030203
SRR17520069.10030204
SRR17520069.10030205
SRR17520069.10030206
SRR17520069.10030207
SRR17520069.10030208
SRR17520069.10030209
SRR17520069.10030210
SRR17520069.10030211
SRR17520069.10030212
SRR17520069.10030213
SRR17520069.10030214
SRR17520069.10030215
SRR17520069.10030216
SRR17520069.10030217
SRR17520069.10030218
SRR17520069.10030219
SRR17520069.10030220
SRR17520069.10030221
SRR17520069.10030222
SRR17520069.10030223
SRR17520069.10030224
SRR17520069.10030225
SRR17520069.10030226
SRR17520069.10030227
SRR17520069.10030228
SRR17520069.10030229
SRR17520069.10030230
SRR17520069.10030231
SRR17520069.10030232
SRR17520069.10030233
SRR17520069.10030234
SRR17520069.10030235
SRR17520069.10030236
SRR17520069.10030237

SRR17520069.10030238
SRR17520069.10030239
SRR17520069.10030240
SRR17520069.10030241
SRR17520069.10030242
SRR17520069.10030243
SRR17520069.10030244
SRR17520069.10030245
SRR17520069.10030246
SRR17520069.10030247
SRR17520069.10030248
SRR17520069.10030249
SRR17520069.10030250
SRR17520069.10030251
SRR17520069.10030252
SRR17520069.10030253
SRR17520069.10030254
SRR17520069.10030255
SRR17520069.10030256
SRR17520069.10030257
SRR17520069.10030258
SRR17520069.10030259
SRR17520069.10030260
SRR17520069.10030261
SRR17520069.10030262
SRR17520069.10030263
SRR17520069.10030264
SRR17520069.10030265
SRR17520069.10030266
SRR17520069.10030267
SRR17520069.10030268
SRR17520069.10030269
SRR17520069.10030270
SRR17520069.10030271
SRR17520069.10030272
SRR17520069.10030273
SRR17520069.10030274
SRR17520069.10030275
SRR17520069.10030276
SRR17520069.10030277
SRR17520069.10030278
SRR17520069.10030279
SRR17520069.10030280
SRR17520069.10030281
SRR17520069.10030282
SRR17520069.10030283
SRR17520069.10030284

SRR17520069.10030285
SRR17520069.10030288
SRR17520069.10030289
SRR17520069.10030290
SRR17520069.10030291
SRR17520069.10030292
SRR17520069.10030293
SRR17520069.10030294
SRR17520069.10030295
SRR17520069.10030296
SRR17520069.10030297
SRR17520069.10030298
SRR17520069.10030299
SRR17520069.10030300
SRR17520069.10030301
SRR17520069.10030302
SRR17520069.10030303
SRR17520069.10030304
SRR17520069.10030306
SRR17520069.10030307
SRR17520069.10030308
SRR17520069.10030309
SRR17520069.10030310
SRR17520069.10030311
SRR17520069.10030312
SRR17520069.10030313
SRR17520069.10030314
SRR17520069.10030315
SRR17520069.10030316
SRR17520069.10030317
SRR17520069.10030318
SRR17520069.10030319
SRR17520069.10030320
SRR17520069.10030321
SRR17520069.10030322
SRR17520069.10030323
SRR17520069.10030324
SRR17520069.10030325
SRR17520069.10030326
SRR17520069.10030327
SRR17520069.10030328
SRR17520069.10030329
SRR17520069.10030330
SRR17520069.10030331
SRR17520069.10030332
SRR17520069.10030333
SRR17520069.10030334

SRR17520069.10030335
SRR17520069.10030336
SRR17520069.10030337
SRR17520069.10030338
SRR17520069.10030339
SRR17520069.10030340
SRR17520069.10030341
SRR17520069.10030342
SRR17520069.10030343
SRR17520069.10030344
SRR17520069.10030345
SRR17520069.10030346
SRR17520069.10030347
SRR17520069.10030348
SRR17520069.10030349
SRR17520069.10030350
SRR17520069.10030351
SRR17520069.10030352
SRR17520069.10030353
SRR17520069.10030354
SRR17520069.10030355
SRR17520069.10030356
SRR17520069.10030357
SRR17520069.10030358
SRR17520069.10030359
SRR17520069.10030360
SRR17520069.10030361
SRR17520069.10030362
SRR17520069.10030363
SRR17520069.10030364
SRR17520069.10042352
SRR17520069.10042353
SRR17520069.10042354
SRR17520069.10042355
SRR17520069.10042356
SRR17520069.10042357
SRR17520069.10042358
SRR17520069.10042359
SRR17520069.10042360
SRR17520069.10042361
SRR17520069.10042362
SRR17520069.10042363
SRR17520069.10042364
SRR17520069.10042365
SRR17520069.10042366
SRR17520069.10042367
SRR17520069.10042368

SRR17520069.10042369
SRR17520069.10042370
SRR17520069.10042371
SRR17520069.10042372
SRR17520069.10042373
SRR17520069.10042374
SRR17520069.10042375
SRR17520069.10042376
SRR17520069.10042377
SRR17520069.10042378
SRR17520069.10042379
SRR17520069.10042380
SRR17520069.10042381
SRR17520069.10042382
SRR17520069.10042385
SRR17520069.10042386
SRR17520069.10042387
SRR17520069.10042388
SRR17520069.10042390
SRR17520069.10042391
SRR17520069.10042392
SRR17520069.10042393
SRR17520069.10051247
SRR17520069.10051248
SRR17520069.10051249
SRR17520069.10051250
SRR17520069.10051251
SRR17520069.10051252
SRR17520069.10051253
SRR17520069.10051254
SRR17520069.10051255
SRR17520069.10051256
SRR17520069.10051257
SRR17520069.10051258
SRR17520069.10051259
SRR17520069.10051260
SRR17520069.10051261
SRR17520069.10051262
SRR17520069.10051264
SRR17520069.10051265
SRR17520069.10051268
SRR17520069.10051269
SRR17520069.10051270
SRR17520069.10051271
SRR17520069.10051272
SRR17520069.10051274
SRR17520069.10051275

SRR17520069.10051276
SRR17520069.10051277
SRR17520069.10051278
SRR17520069.10051279
SRR17520069.10051280
SRR17520069.10051281
SRR17520069.10051282
SRR17520069.10051283
SRR17520069.10051284
SRR17520069.10051285
SRR17520069.10051286
SRR17520069.10051287
SRR17520069.10051288
SRR17520069.10051289
SRR17520069.10051290
SRR17520069.10051291
SRR17520069.10051292
SRR17520069.10051293
SRR17520069.10051294
SRR17520069.10051295
SRR17520069.10051296
SRR17520069.10051297
SRR17520069.10051298
SRR17520069.10051299
SRR17520069.10051300
SRR17520069.10051301
SRR17520069.10051302
SRR17520069.10051303
SRR17520069.10051304
SRR17520069.10051305
SRR17520069.10051306
SRR17520069.10051307
SRR17520069.10051308
SRR17520069.10051309
SRR17520069.10051310
SRR17520069.10051311
SRR17520069.10051312
SRR17520069.10051313
SRR17520069.10051314
SRR17520069.10051315
SRR17520069.10051316
SRR17520069.10051317
SRR17520069.10051318
SRR17520069.10051319
SRR17520069.10051320
SRR17520069.10051321
SRR17520069.10051322

SRR17520069.10051323
SRR17520069.10051324
SRR17520069.10051325
SRR17520069.10051326
SRR17520069.10051327
SRR17520069.10051328
SRR17520069.10051329
SRR17520069.10051330
SRR17520069.10051331
SRR17520069.10051332
SRR17520069.10051333
SRR17520069.10051334
SRR17520069.10051335
SRR17520069.10051336
SRR17520069.10051337
SRR17520069.10051338
SRR17520069.10051339
SRR17520069.10051340
SRR17520069.10051341
SRR17520069.10051342
SRR17520069.10051343
SRR17520069.10051344
SRR17520069.10051345
SRR17520069.10051346
SRR17520069.10051347
SRR17520069.10051348
SRR17520069.10051349
SRR17520069.10051350
SRR17520069.10051351
SRR17520069.10053107
SRR17520069.10056572
SRR17520069.10061403
SRR17520069.10061404
SRR17520069.10062324
SRR17520069.10062325
SRR17520069.10062327
SRR17520069.10062328
SRR17520069.10062329
SRR17520069.10062330
SRR17520069.10062331
SRR17520069.10062332
SRR17520069.10062333
SRR17520069.10062334
SRR17520069.10062335
SRR17520069.10062336
SRR17520069.10062337
SRR17520069.10062338

SRR17520069.10062339
SRR17520069.10062340
SRR17520069.10062341
SRR17520069.10062342
SRR17520069.10062343
SRR17520069.10062344
SRR17520069.10062345
SRR17520069.10062346
SRR17520069.10062347
SRR17520069.10062348
SRR17520069.10062349
SRR17520069.10062350
SRR17520069.10062351
SRR17520069.10062352
SRR17520069.10062353
SRR17520069.10062354
SRR17520069.10062355
SRR17520069.10062356
SRR17520069.10062357
SRR17520069.10062358
SRR17520069.10062359
SRR17520069.10062360
SRR17520069.10062361
SRR17520069.10062362
SRR17520069.10062363
SRR17520069.10062364
SRR17520069.10062365
SRR17520069.10062366
SRR17520069.10062368
SRR17520069.10062370
SRR17520069.10062371
SRR17520069.10062372
SRR17520069.10062373
SRR17520069.10062374
SRR17520069.10062375
SRR17520069.10062376
SRR17520069.10062377
SRR17520069.10062378
SRR17520069.10062379
SRR17520069.10062380
SRR17520069.10062381
SRR17520069.10062382
SRR17520069.10062383
SRR17520069.10062384
SRR17520069.10062385
SRR17520069.10062386
SRR17520069.10063806

SRR17520069.10063807
SRR17520069.10063809
SRR17520069.10063810
SRR17520069.10063811
SRR17520069.10063812
SRR17520069.10063813
SRR17520069.10063814
SRR17520069.10063815
SRR17520069.10063816
SRR17520069.10063818
SRR17520069.10063819
SRR17520069.10113924
SRR17520069.10113948
SRR17520069.10119409
SRR17520069.10124122
SRR17520069.10126615
SRR17520069.10128982
SRR17520069.10128983
SRR17520069.10140365
SRR17520069.10140366
SRR17520069.10140368
SRR17520069.10140369
SRR17520069.10140370
SRR17520069.10140371
SRR17520069.10140373
SRR17520069.10140374
SRR17520069.10140375
SRR17520069.10140376
SRR17520069.10140377
SRR17520069.10140379
SRR17520069.10140380
SRR17520069.10140381
SRR17520069.10140382
SRR17520069.10140383
SRR17520069.10140384
SRR17520069.10140385
SRR17520069.10140386
SRR17520069.10140387
SRR17520069.10140388
SRR17520069.10140389
SRR17520069.10140390
SRR17520069.10140391
SRR17520069.10140392
SRR17520069.10140394
SRR17520069.10140395
SRR17520069.10140397
SRR17520069.10140398

SRR17520069.10140399
SRR17520069.10140400
SRR17520069.10140401
SRR17520069.10140402
SRR17520069.10140403
SRR17520069.10140404
SRR17520069.10140405
SRR17520069.10140406
SRR17520069.10140407
SRR17520069.10140408
SRR17520069.10140409
SRR17520069.10140410
SRR17520069.10140411
SRR17520069.10140412
SRR17520069.10140413
SRR17520069.10140414
SRR17520069.10140415
SRR17520069.10142811
SRR17520069.10142861
SRR17520069.10146600
SRR17520069.10146601
SRR17520069.10146603
SRR17520069.10146604
SRR17520069.10146606
SRR17520069.10146607
SRR17520069.10146608
SRR17520069.10146609
SRR17520069.10146610
SRR17520069.10146611
SRR17520069.10146612
SRR17520069.10146613
SRR17520069.10146615
SRR17520069.10146616
SRR17520069.10146617
SRR17520069.10146618
SRR17520069.10146620
SRR17520069.10146621
SRR17520069.10146622
SRR17520069.10146623
SRR17520069.10146625
SRR17520069.10146626
SRR17520069.10146627
SRR17520069.10146628
SRR17520069.10146629
SRR17520069.10146630
SRR17520069.10146631
SRR17520069.10146632

SRR17520069.10146633
SRR17520069.10146634
SRR17520069.10146635
SRR17520069.10146636
SRR17520069.10146637
SRR17520069.10146639
SRR17520069.10146640
SRR17520069.10146641
SRR17520069.10146642
SRR17520069.10146643
SRR17520069.10146644
SRR17520069.10146645
SRR17520069.10146646
SRR17520069.10146647
SRR17520069.10146648
SRR17520069.10146649
SRR17520069.10146650
SRR17520069.10146651
SRR17520069.10146652
SRR17520069.10146653
SRR17520069.10146654
SRR17520069.10146655
SRR17520069.10146656
SRR17520069.10146657
SRR17520069.10146658
SRR17520069.10146659
SRR17520069.10146660
SRR17520069.10146661
SRR17520069.10146662
SRR17520069.10146663
SRR17520069.10146664
SRR17520069.10146665
SRR17520069.10146666
SRR17520069.10146667
SRR17520069.10146668
SRR17520069.10146669
SRR17520069.10146670
SRR17520069.10146671
SRR17520069.10146672
SRR17520069.10146673
SRR17520069.10146674
SRR17520069.10148013
SRR17520069.10148014
SRR17520069.10148015
SRR17520069.10148016
SRR17520069.10148017
SRR17520069.10148018

SRR17520069.10148019
SRR17520069.10148020
SRR17520069.10148021
SRR17520069.10148023
SRR17520069.10148024
SRR17520069.10148025
SRR17520069.10148026
SRR17520069.10148027
SRR17520069.10148028
SRR17520069.10148029
SRR17520069.10148030
SRR17520069.10148031
SRR17520069.10148033
SRR17520069.10148034
SRR17520069.10148035
SRR17520069.10148036
SRR17520069.10148037
SRR17520069.10148038
SRR17520069.10148039
SRR17520069.10148040
SRR17520069.10148041
SRR17520069.10148042
SRR17520069.10148043
SRR17520069.10148044
SRR17520069.10148045
SRR17520069.10148046
SRR17520069.10148047
SRR17520069.10148050
SRR17520069.10148051
SRR17520069.10148052
SRR17520069.10148053
SRR17520069.10148054
SRR17520069.10148055
SRR17520069.10148056
SRR17520069.10148057
SRR17520069.10148058
SRR17520069.10148060
SRR17520069.10148061
SRR17520069.10148062
SRR17520069.10148063
SRR17520069.10148064
SRR17520069.10148065
SRR17520069.10148066
SRR17520069.10148067
SRR17520069.10148068
SRR17520069.10148069
SRR17520069.10148070

SRR17520069.10148071
SRR17520069.10148072
SRR17520069.10148073
SRR17520069.10148074
SRR17520069.10148075
SRR17520069.10148076
SRR17520069.10148077
SRR17520069.10148078
SRR17520069.10148079
SRR17520069.10148080
SRR17520069.10148081
SRR17520069.10148082
SRR17520069.10148084
SRR17520069.10148088
SRR17520069.10148089
SRR17520069.10148090
SRR17520069.10148091
SRR17520069.10148092
SRR17520069.10148093
SRR17520069.10148094
SRR17520069.10148095
SRR17520069.10148096
SRR17520069.10148097
SRR17520069.10148098
SRR17520069.10148099
SRR17520069.10148100
SRR17520069.10148103
SRR17520069.10148104
SRR17520069.10148105
SRR17520069.10148106
SRR17520069.10148107
SRR17520069.10148108
SRR17520069.10148109
SRR17520069.10148110
SRR17520069.10148112
SRR17520069.10148113
SRR17520069.10148114
SRR17520069.10148115
SRR17520069.10148116
SRR17520069.10148117
SRR17520069.10148118
SRR17520069.10148119
SRR17520069.10148120
SRR17520069.10148121
SRR17520069.10148122
SRR17520069.10148123
SRR17520069.10148124

SRR17520069.10148125
SRR17520069.10148126
SRR17520069.10148128
SRR17520069.10148129
SRR17520069.10148130
SRR17520069.10148131
SRR17520069.10148132
SRR17520069.10148133
SRR17520069.10148134
SRR17520069.10148135
SRR17520069.10148136
SRR17520069.10148137
SRR17520069.10148138
SRR17520069.10148139
SRR17520069.10148140
SRR17520069.10148141
SRR17520069.10148142
SRR17520069.10148143
SRR17520069.10148144
SRR17520069.10148145
SRR17520069.10148146
SRR17520069.10148147
SRR17520069.10148148
SRR17520069.10148149
SRR17520069.10148150
SRR17520069.10148151
SRR17520069.10148152
SRR17520069.10148153
SRR17520069.10148154
SRR17520069.10148155
SRR17520069.10148156
SRR17520069.10148157
SRR17520069.10148158
SRR17520069.10148159
SRR17520069.10148160
SRR17520069.10148161
SRR17520069.10148162
SRR17520069.10148163
SRR17520069.10148164
SRR17520069.10148165
SRR17520069.10148166
SRR17520069.10148167
SRR17520069.10148168
SRR17520069.10148169
SRR17520069.10148170
SRR17520069.10148171
SRR17520069.10154856

SRR17520069.10155027
SRR17520069.10160503
SRR17520069.10165092
SRR17520069.10165093
SRR17520069.10165094
SRR17520069.10165095
SRR17520069.10165096
SRR17520069.10165097
SRR17520069.10165098
SRR17520069.10165099
SRR17520069.10165100
SRR17520069.10165101
SRR17520069.10165102
SRR17520069.10165103
SRR17520069.10165104
SRR17520069.10165106
SRR17520069.10165107
SRR17520069.10165108
SRR17520069.10165109
SRR17520069.10165110
SRR17520069.10165111
SRR17520069.10165112
SRR17520069.10165113
SRR17520069.10165114
SRR17520069.10165115
SRR17520069.10165116
SRR17520069.10165117
SRR17520069.10165118
SRR17520069.10165119
SRR17520069.10165120
SRR17520069.10165121
SRR17520069.10165122
SRR17520069.10165123
SRR17520069.10165124
SRR17520069.10165125
SRR17520069.10165126
SRR17520069.10165127
SRR17520069.10165128
SRR17520069.10165129
SRR17520069.10165130
SRR17520069.10179705
SRR17520069.10189692
SRR17520069.10189693
SRR17520069.10189694
SRR17520069.10189695
SRR17520069.10189696
SRR17520069.10189697

SRR17520069.10189698
SRR17520069.10189700
SRR17520069.10189701
SRR17520069.10189702
SRR17520069.10191126
SRR17520069.10191127
SRR17520069.10191128
SRR17520069.10191129
SRR17520069.10191130
SRR17520069.10191131
SRR17520069.10191133
SRR17520069.10191134
SRR17520069.10191135
SRR17520069.10191136
SRR17520069.10191137
SRR17520069.10191138
SRR17520069.10191139
SRR17520069.10191140
SRR17520069.10191141
SRR17520069.10191142
SRR17520069.10191143
SRR17520069.10191144
SRR17520069.10191145
SRR17520069.10191146
SRR17520069.10191147
SRR17520069.10191148
SRR17520069.10191149
SRR17520069.10191150
SRR17520069.10191151
SRR17520069.10191152
SRR17520069.10191153
SRR17520069.10191154
SRR17520069.10191155
SRR17520069.10191156
SRR17520069.10191157
SRR17520069.10191158
SRR17520069.10191159
SRR17520069.10191160
SRR17520069.10191161
SRR17520069.10191162
SRR17520069.10191163
SRR17520069.10191164
SRR17520069.10191165
SRR17520069.10191166
SRR17520069.10191167
SRR17520069.10191168
SRR17520069.10191169

SRR17520069.10191170
SRR17520069.10191171
SRR17520069.10208044
SRR17520069.10208763
SRR17520069.10216682
SRR17520069.10216683
SRR17520069.10216684
SRR17520069.10216685
SRR17520069.10216686
SRR17520069.10216687
SRR17520069.10216688
SRR17520069.10220919
SRR17520069.10228819
SRR17520069.10228820
SRR17520069.10228821
SRR17520069.10228822
SRR17520069.10228823
SRR17520069.10228824
SRR17520069.10228825
SRR17520069.10228826
SRR17520069.10228827
SRR17520069.10228829
SRR17520069.10228830
SRR17520069.10228831
SRR17520069.10228832
SRR17520069.10228833
SRR17520069.10228834
SRR17520069.10228835
SRR17520069.10228836
SRR17520069.10228837
SRR17520069.10228838
SRR17520069.10228839
SRR17520069.10228840
SRR17520069.10228841
SRR17520069.10228843
SRR17520069.10228844
SRR17520069.10228845
SRR17520069.10228846
SRR17520069.10228847
SRR17520069.10228848
SRR17520069.10228849
SRR17520069.10228850
SRR17520069.10228851
SRR17520069.10228852
SRR17520069.10228853
SRR17520069.10228854
SRR17520069.10228856

SRR17520069.10228857
SRR17520069.10228858
SRR17520069.10228859
SRR17520069.10228860
SRR17520069.10228861
SRR17520069.10228862
SRR17520069.10228864
SRR17520069.10228865
SRR17520069.10228866
SRR17520069.10228867
SRR17520069.10228868
SRR17520069.10228869
SRR17520069.10228870
SRR17520069.10228871
SRR17520069.10228872
SRR17520069.10228873
SRR17520069.10228874
SRR17520069.10228875
SRR17520069.10228876
SRR17520069.10228877
SRR17520069.10228878
SRR17520069.10228879
SRR17520069.10228880
SRR17520069.10228881
SRR17520069.10228882
SRR17520069.10228883
SRR17520069.10228884
SRR17520069.10228885
SRR17520069.10228886
SRR17520069.10228887
SRR17520069.10228888
SRR17520069.10228889
SRR17520069.10228891
SRR17520069.10228892
SRR17520069.10228893
SRR17520069.10228894
SRR17520069.10228895
SRR17520069.10228896
SRR17520069.10228897
SRR17520069.10228898
SRR17520069.10228899
SRR17520069.10228900
SRR17520069.10228901
SRR17520069.10228902
SRR17520069.10228903
SRR17520069.10228904
SRR17520069.10228905

SRR17520069.10228906
SRR17520069.10228907
SRR17520069.10228908
SRR17520069.10228909
SRR17520069.10228910
SRR17520069.10228911
SRR17520069.10228912
SRR17520069.10228913
SRR17520069.10228914
SRR17520069.10228915
SRR17520069.10228916
SRR17520069.10228917
SRR17520069.10228918
SRR17520069.10228919
SRR17520069.10228920
SRR17520069.10228921
SRR17520069.10228922
SRR17520069.10228923
SRR17520069.10228924
SRR17520069.10228925
SRR17520069.10228926
SRR17520069.10228927
SRR17520069.10228928
SRR17520069.10228929
SRR17520069.10228930
SRR17520069.10228931
SRR17520069.10228932
SRR17520069.10228933
SRR17520069.10228934
SRR17520069.10228935
SRR17520069.10228936
SRR17520069.10228937
SRR17520069.10228938
SRR17520069.10228939
SRR17520069.10228940
SRR17520069.10228941
SRR17520069.10228942
SRR17520069.10228943
SRR17520069.10228944
SRR17520069.10228945
SRR17520069.10228946
SRR17520069.10228947
SRR17520069.10228948
SRR17520069.10228949
SRR17520069.10228950
SRR17520069.10228951
SRR17520069.10228952

SRR17520069.10228953
SRR17520069.10228954
SRR17520069.10228955
SRR17520069.10228956
SRR17520069.10228957
SRR17520069.10228959
SRR17520069.10228960
SRR17520069.10228961
SRR17520069.10228962
SRR17520069.10228963
SRR17520069.10228964
SRR17520069.10228965
SRR17520069.10228966
SRR17520069.10228967
SRR17520069.10228968
SRR17520069.10228969
SRR17520069.10228970
SRR17520069.10231962
SRR17520069.10231963
SRR17520069.10231964
SRR17520069.10231965
SRR17520069.10231966
SRR17520069.10231967
SRR17520069.10231968
SRR17520069.10248423
SRR17520069.10250780
SRR17520069.10250781
SRR17520069.10250782
SRR17520069.10250783
SRR17520069.10250784
SRR17520069.10250785
SRR17520069.10250786
SRR17520069.10250787
SRR17520069.10250789
SRR17520069.10266208
SRR17520069.10269219
SRR17520069.10269220
SRR17520069.10269221
SRR17520069.10269222
SRR17520069.10269223
SRR17520069.10269224
SRR17520069.10269225
SRR17520069.10269226
SRR17520069.10269227
SRR17520069.10269228
SRR17520069.10269229
SRR17520069.10269230

SRR17520069.10269231
SRR17520069.10269232
SRR17520069.10269233
SRR17520069.10269234
SRR17520069.10269235
SRR17520069.10269236
SRR17520069.10269237
SRR17520069.10269238
SRR17520069.10269239
SRR17520069.10269240
SRR17520069.10269241
SRR17520069.10269242
SRR17520069.10269243
SRR17520069.10269244
SRR17520069.10269245
SRR17520069.10269246
SRR17520069.10269247
SRR17520069.10269248
SRR17520069.10269249
SRR17520069.10269250
SRR17520069.10269251
SRR17520069.10269252
SRR17520069.10269253
SRR17520069.10269254
SRR17520069.10269255
SRR17520069.10269256
SRR17520069.10269257
SRR17520069.10269258
SRR17520069.10269259
SRR17520069.10269260
SRR17520069.10269261
SRR17520069.10269262
SRR17520069.10269263
SRR17520069.10269264
SRR17520069.10269265
SRR17520069.10269266
SRR17520069.10269267
SRR17520069.10269269
SRR17520069.10269270
SRR17520069.10269271
SRR17520069.10269272
SRR17520069.10269273
SRR17520069.10269274
SRR17520069.10269275
SRR17520069.10269276
SRR17520069.10269277
SRR17520069.10269278

SRR17520069.10269279
SRR17520069.10269280
SRR17520069.10269281
SRR17520069.10269282
SRR17520069.10269283
SRR17520069.10269284
SRR17520069.10269285
SRR17520069.10269286
SRR17520069.10269287
SRR17520069.10269288
SRR17520069.10269289
SRR17520069.10269290
SRR17520069.10269291
SRR17520069.10269292
SRR17520069.10269293
SRR17520069.10269294
SRR17520069.10269295
SRR17520069.10269296
SRR17520069.10269297
SRR17520069.10269298
SRR17520069.10269299
SRR17520069.10269300
SRR17520069.10269301
SRR17520069.10269302
SRR17520069.10269303
SRR17520069.10269304
SRR17520069.10269305
SRR17520069.10269306
SRR17520069.10269307
SRR17520069.10269308
SRR17520069.10269309
SRR17520069.10269310
SRR17520069.10269311
SRR17520069.10269312
SRR17520069.10269313
SRR17520069.10269314
SRR17520069.10269315
SRR17520069.10269316
SRR17520069.10269317
SRR17520069.10269318
SRR17520069.10269319
SRR17520069.10269320
SRR17520069.10273598
SRR17520069.10273599
SRR17520069.10273600
SRR17520069.10273601
SRR17520069.10273602

SRR17520069.10273603
SRR17520069.10273604
SRR17520069.10273605
SRR17520069.10273606
SRR17520069.10273607
SRR17520069.10273608
SRR17520069.10273609
SRR17520069.10273610
SRR17520069.10273611
SRR17520069.10273612
SRR17520069.10273613
SRR17520069.10273614
SRR17520069.10273615
SRR17520069.10273616
SRR17520069.10273617
SRR17520069.10273618
SRR17520069.10273619
SRR17520069.10273620
SRR17520069.10273621
SRR17520069.10273622
SRR17520069.10273623
SRR17520069.10273624
SRR17520069.10273625
SRR17520069.10273626
SRR17520069.10273627
SRR17520069.10273628
SRR17520069.10273629
SRR17520069.10273630
SRR17520069.10273631
SRR17520069.10273632
SRR17520069.10273633
SRR17520069.10273634
SRR17520069.10273635
SRR17520069.10273636
SRR17520069.10273637
SRR17520069.10273638
SRR17520069.10273639
SRR17520069.10273640
SRR17520069.10273641
SRR17520069.10273642
SRR17520069.10273643
SRR17520069.10273644
SRR17520069.10273645
SRR17520069.10273646
SRR17520069.10273647
SRR17520069.10273648
SRR17520069.10273649

SRR17520069.10273650
SRR17520069.10273651
SRR17520069.10273652
SRR17520069.10273653
SRR17520069.10273654
SRR17520069.10273655
SRR17520069.10273656
SRR17520069.10273657
SRR17520069.10273658
SRR17520069.10273660
SRR17520069.10273661
SRR17520069.10273662
SRR17520069.10273663
SRR17520069.10273664
SRR17520069.10273665
SRR17520069.10273666
SRR17520069.10273667
SRR17520069.10273668
SRR17520069.10273669
SRR17520069.10273670
SRR17520069.10273671
SRR17520069.10273672
SRR17520069.10273673
SRR17520069.10273674
SRR17520069.10273675
SRR17520069.10273676
SRR17520069.10273677
SRR17520069.10273678
SRR17520069.10273679
SRR17520069.10273680
SRR17520069.10273681
SRR17520069.10273682
SRR17520069.10273683
SRR17520069.10273684
SRR17520069.10273686
SRR17520069.10273687
SRR17520069.10273688
SRR17520069.10273689
SRR17520069.10273690
SRR17520069.10273691
SRR17520069.10274074
SRR17520069.10274075
SRR17520069.10274076
SRR17520069.10274077
SRR17520069.10274078
SRR17520069.10274079
SRR17520069.10274080

SRR17520069.10274081
SRR17520069.10274082
SRR17520069.10274083
SRR17520069.10274084
SRR17520069.10274085
SRR17520069.10274086
SRR17520069.10274087
SRR17520069.10274088
SRR17520069.10274089
SRR17520069.10274090
SRR17520069.10274091
SRR17520069.10274092
SRR17520069.10274093
SRR17520069.10274094
SRR17520069.10274095
SRR17520069.10274096
SRR17520069.10274097
SRR17520069.10274098
SRR17520069.10274099
SRR17520069.10274100
SRR17520069.10274101
SRR17520069.10274102
SRR17520069.10274103
SRR17520069.10274104
SRR17520069.10274105
SRR17520069.10274106
SRR17520069.10274107
SRR17520069.10274108
SRR17520069.10274109
SRR17520069.10274110
SRR17520069.10274111
SRR17520069.10274112
SRR17520069.10274113
SRR17520069.10274114
SRR17520069.10274115
SRR17520069.10274116
SRR17520069.10274117
SRR17520069.10274118
SRR17520069.10274119
SRR17520069.10274121
SRR17520069.10274122
SRR17520069.10274123
SRR17520069.10274124
SRR17520069.10274125
SRR17520069.10274126
SRR17520069.10274127
SRR17520069.10274128

SRR17520069.10274129
SRR17520069.10274130
SRR17520069.10274131
SRR17520069.10274132
SRR17520069.10274133
SRR17520069.10274134
SRR17520069.10274135
SRR17520069.10274136
SRR17520069.10274137
SRR17520069.10274138
SRR17520069.10274139
SRR17520069.10274140
SRR17520069.10274141
SRR17520069.10274142
SRR17520069.10274143
SRR17520069.10274144
SRR17520069.10274145
SRR17520069.10274146
SRR17520069.10274147
SRR17520069.10274148
SRR17520069.10274149
SRR17520069.10274150
SRR17520069.10274151
SRR17520069.10274152
SRR17520069.10274153
SRR17520069.10274154
SRR17520069.10274155
SRR17520069.10274156
SRR17520069.10274157
SRR17520069.10274158
SRR17520069.10274159
SRR17520069.10274160
SRR17520069.10274161
SRR17520069.10274162
SRR17520069.10274163
SRR17520069.10274164
SRR17520069.10274165
SRR17520069.10274166
SRR17520069.10274167
SRR17520069.10274168
SRR17520069.10274169
SRR17520069.10274170
SRR17520069.10274171
SRR17520069.10274172
SRR17520069.10274173
SRR17520069.10274174
SRR17520069.10274175

SRR17520069.10274177
SRR17520069.10274178
SRR17520069.10274180
SRR17520069.10274181
SRR17520069.10274182
SRR17520069.10274183
SRR17520069.10274184
SRR17520069.10274185
SRR17520069.10274186
SRR17520069.10274187
SRR17520069.10274188
SRR17520069.10274189
SRR17520069.10274190
SRR17520069.10274191
SRR17520069.10274192
SRR17520069.10274193
SRR17520069.10274194
SRR17520069.10274195
SRR17520069.10274196
SRR17520069.10274197
SRR17520069.10274198
SRR17520069.10274199
SRR17520069.10274200
SRR17520069.10274201
SRR17520069.10274202
SRR17520069.10274203
SRR17520069.10274204
SRR17520069.10274205
SRR17520069.10274206
SRR17520069.10274207
SRR17520069.10274208
SRR17520069.10274209
SRR17520069.10274210
SRR17520069.10274211
SRR17520069.10274213
SRR17520069.10274214
SRR17520069.10274215
SRR17520069.10274216
SRR17520069.10274217
SRR17520069.10274218
SRR17520069.10274219
SRR17520069.10274220
SRR17520069.10274221
SRR17520069.10274222
SRR17520069.10274223
SRR17520069.10274224
SRR17520069.10274225

SRR17520069.10274226
SRR17520069.10274227
SRR17520069.10274228
SRR17520069.10274229
SRR17520069.10274230
SRR17520069.10274231
SRR17520069.10274232
SRR17520069.10274233
SRR17520069.10274234
SRR17520069.10274235
SRR17520069.10274236
SRR17520069.10274237
SRR17520069.10274238
SRR17520069.10274239
SRR17520069.10274240
SRR17520069.10274241
SRR17520069.10274242
SRR17520069.10274243
SRR17520069.10274244
SRR17520069.10274245
SRR17520069.10274246
SRR17520069.10274247
SRR17520069.10274248
SRR17520069.10274249
SRR17520069.10274250
SRR17520069.10274251
SRR17520069.10274252
SRR17520069.10274253
SRR17520069.10274255
SRR17520069.10285750
SRR17520069.10285751
SRR17520069.10285752
SRR17520069.10285753
SRR17520069.10285754
SRR17520069.10285755
SRR17520069.10285756
SRR17520069.10285757
SRR17520069.10285758
SRR17520069.10285759
SRR17520069.10285760
SRR17520069.10285761
SRR17520069.10285762
SRR17520069.10285763
SRR17520069.10285764
SRR17520069.10285765
SRR17520069.10285766
SRR17520069.10285767

SRR17520069.10285768
SRR17520069.10285769
SRR17520069.10285770
SRR17520069.10285771
SRR17520069.10285772
SRR17520069.10285773
SRR17520069.10285774
SRR17520069.10285775
SRR17520069.10285777
SRR17520069.10285778
SRR17520069.10285779
SRR17520069.10285780
SRR17520069.10285781
SRR17520069.10285782
SRR17520069.10285783
SRR17520069.10285784
SRR17520069.10285785
SRR17520069.10285786
SRR17520069.10285787
SRR17520069.10285788
SRR17520069.10285789
SRR17520069.10285790
SRR17520069.10285791
SRR17520069.10285792
SRR17520069.10285793
SRR17520069.10285794
SRR17520069.10285795
SRR17520069.10285796
SRR17520069.10285797
SRR17520069.10285798
SRR17520069.10285799
SRR17520069.10285800
SRR17520069.10285801
SRR17520069.10285802
SRR17520069.10285803
SRR17520069.10285804
SRR17520069.10285805
SRR17520069.10285806
SRR17520069.10285807
SRR17520069.10285809
SRR17520069.10285810
SRR17520069.10285811
SRR17520069.10285813
SRR17520069.10285814
SRR17520069.10285815
SRR17520069.10285816
SRR17520069.10285817

SRR17520069.10285818
SRR17520069.10285819
SRR17520069.10285820
SRR17520069.10285821
SRR17520069.10285822
SRR17520069.10285823
SRR17520069.10285824
SRR17520069.10285825
SRR17520069.10285826
SRR17520069.10285828
SRR17520069.10285829
SRR17520069.10285830
SRR17520069.10285831
SRR17520069.10285832
SRR17520069.10285833
SRR17520069.10285834
SRR17520069.10285835
SRR17520069.10285836
SRR17520069.10285838
SRR17520069.10285839
SRR17520069.10285840
SRR17520069.10285841
SRR17520069.10285842
SRR17520069.10285843
SRR17520069.10285845
SRR17520069.10285847
SRR17520069.10285849
SRR17520069.10285850
SRR17520069.10285851
SRR17520069.10285852
SRR17520069.10285853
SRR17520069.10285854
SRR17520069.10285855
SRR17520069.10285856
SRR17520069.10285857
SRR17520069.10285858
SRR17520069.10293144
SRR17520069.10314413
SRR17520069.10315361
SRR17520069.10315611
SRR17520069.10315612
SRR17520069.10315613
SRR17520069.10315614
SRR17520069.10315615
SRR17520069.10315616
SRR17520069.10315618
SRR17520069.10315619

SRR17520069.10315620
SRR17520069.10315621
SRR17520069.10315622
SRR17520069.10315623
SRR17520069.10315624
SRR17520069.10315625
SRR17520069.10315626
SRR17520069.10315627
SRR17520069.10315628
SRR17520069.10315629
SRR17520069.10315630
SRR17520069.10315631
SRR17520069.10315632
SRR17520069.10315633
SRR17520069.10315634
SRR17520069.10315635
SRR17520069.10315636
SRR17520069.10315637
SRR17520069.10315638
SRR17520069.10315639
SRR17520069.10315640
SRR17520069.10315641
SRR17520069.10315642
SRR17520069.10315643
SRR17520069.10315644
SRR17520069.10315645
SRR17520069.10315646
SRR17520069.10315647
SRR17520069.10315648
SRR17520069.10315649
SRR17520069.10315650
SRR17520069.10315651
SRR17520069.10315652
SRR17520069.10315653
SRR17520069.10315654
SRR17520069.10315655
SRR17520069.10315656
SRR17520069.10315657
SRR17520069.10315658
SRR17520069.10315659
SRR17520069.10315660
SRR17520069.10315661
SRR17520069.10315662
SRR17520069.10315663
SRR17520069.10315664
SRR17520069.10315665
SRR17520069.10315666

SRR17520069.10315667
SRR17520069.10315668
SRR17520069.10315669
SRR17520069.10315670
SRR17520069.10315671
SRR17520069.10315672
SRR17520069.10315673
SRR17520069.10315674
SRR17520069.10315675
SRR17520069.10315676
SRR17520069.10315677
SRR17520069.10315678
SRR17520069.10315679
SRR17520069.10315680
SRR17520069.10315681
SRR17520069.10315682
SRR17520069.10315683
SRR17520069.10315684
SRR17520069.10315685
SRR17520069.10315686
SRR17520069.10315687
SRR17520069.10315688
SRR17520069.10315689
SRR17520069.10315690
SRR17520069.10315691
SRR17520069.10315692
SRR17520069.10315693
SRR17520069.10315694
SRR17520069.10315695
SRR17520069.10315696
SRR17520069.10315697
SRR17520069.10315698
SRR17520069.10315699
SRR17520069.10315700
SRR17520069.10315702
SRR17520069.10315703
SRR17520069.10315704
SRR17520069.10315705
SRR17520069.10315706
SRR17520069.10315707
SRR17520069.10315708
SRR17520069.10315709
SRR17520069.10316731
SRR17520069.10334272
SRR17520069.10348279
SRR17520069.10348280
SRR17520069.10348281

SRR17520069.10348282
SRR17520069.10348283
SRR17520069.10348284
SRR17520069.10348285
SRR17520069.10348286
SRR17520069.10348287
SRR17520069.10348288
SRR17520069.10348289
SRR17520069.10348290
SRR17520069.10348291
SRR17520069.10348292
SRR17520069.10348293
SRR17520069.10348294
SRR17520069.10348295
SRR17520069.10348297
SRR17520069.10348298
SRR17520069.10348299
SRR17520069.10348300
SRR17520069.10348301
SRR17520069.10348303
SRR17520069.10348304
SRR17520069.10348306
SRR17520069.10348307
SRR17520069.10348308
SRR17520069.10348309
SRR17520069.10348310
SRR17520069.10348311
SRR17520069.10348312
SRR17520069.10348313
SRR17520069.10348314
SRR17520069.10348315
SRR17520069.10348316
SRR17520069.10348317
SRR17520069.10348318
SRR17520069.10348319
SRR17520069.10348320
SRR17520069.10348321
SRR17520069.10348322
SRR17520069.10348323
SRR17520069.10348324
SRR17520069.10348325
SRR17520069.10348326
SRR17520069.10348327
SRR17520069.10348328
SRR17520069.10348329
SRR17520069.10348330
SRR17520069.10348331

SRR17520069.10348332
SRR17520069.10348333
SRR17520069.10348334
SRR17520069.10348335
SRR17520069.10348336
SRR17520069.10348337
SRR17520069.10348339
SRR17520069.10348340
SRR17520069.10348341
SRR17520069.10348342
SRR17520069.10348343
SRR17520069.10348344
SRR17520069.10348345
SRR17520069.10348346
SRR17520069.10348347
SRR17520069.10348348
SRR17520069.10348349
SRR17520069.10348350
SRR17520069.10348352
SRR17520069.10348353
SRR17520069.10348354
SRR17520069.10348355
SRR17520069.10348356
SRR17520069.10348357
SRR17520069.10348358
SRR17520069.10348359
SRR17520069.10348360
SRR17520069.10348361
SRR17520069.10348362
SRR17520069.10348363
SRR17520069.10348364
SRR17520069.10348366
SRR17520069.10348367
SRR17520069.10348369
SRR17520069.10348370
SRR17520069.10348371
SRR17520069.10348372
SRR17520069.10348373
SRR17520069.10348374
SRR17520069.10348375
SRR17520069.10348376
SRR17520069.10348377
SRR17520069.10348378
SRR17520069.10348379
SRR17520069.10348380
SRR17520069.10348381
SRR17520069.10348382

SRR17520069.10348383
SRR17520069.10348384
SRR17520069.10348385
SRR17520069.10348386
SRR17520069.10348387
SRR17520069.10348388
SRR17520069.10348389
SRR17520069.10348390
SRR17520069.10348391
SRR17520069.10348392
SRR17520069.10348393
SRR17520069.10348394
SRR17520069.10348395
SRR17520069.10348396
SRR17520069.10348397
SRR17520069.10348398
SRR17520069.10348399
SRR17520069.10348400
SRR17520069.10348401
SRR17520069.10348402
SRR17520069.10348403
SRR17520069.10348404
SRR17520069.10348405
SRR17520069.10348406
SRR17520069.10348407
SRR17520069.10348409
SRR17520069.10348410
SRR17520069.10348411
SRR17520069.10348412
SRR17520069.10348413
SRR17520069.10348414
SRR17520069.10348415
SRR17520069.10348416
SRR17520069.10348417
SRR17520069.10348418
SRR17520069.10348419
SRR17520069.10348420
SRR17520069.10348421
SRR17520069.10348422
SRR17520069.10348423
SRR17520069.10348424
SRR17520069.10348517
SRR17520069.10353664
SRR17520069.10353665
SRR17520069.10353666
SRR17520069.10353667
SRR17520069.10353668

SRR17520069.10353669
SRR17520069.10353670
SRR17520069.10353671
SRR17520069.10353672
SRR17520069.10353673
SRR17520069.10353674
SRR17520069.10353675
SRR17520069.10353676
SRR17520069.10353677
SRR17520069.10353678
SRR17520069.10353679
SRR17520069.10353680
SRR17520069.10353681
SRR17520069.10353682
SRR17520069.10353683
SRR17520069.10353684
SRR17520069.10353685
SRR17520069.10353686
SRR17520069.10353687
SRR17520069.10353688
SRR17520069.10353689
SRR17520069.10353690
SRR17520069.10353691
SRR17520069.10353692
SRR17520069.10353693
SRR17520069.10353694
SRR17520069.10353695
SRR17520069.10353696
SRR17520069.10353697
SRR17520069.10353699
SRR17520069.10353700
SRR17520069.10353701
SRR17520069.10353702
SRR17520069.10353703
SRR17520069.10353704
SRR17520069.10353705
SRR17520069.10353706
SRR17520069.10353707
SRR17520069.10353708
SRR17520069.10353709
SRR17520069.10353710
SRR17520069.10353711
SRR17520069.10353712
SRR17520069.10358709
SRR17520069.10358710
SRR17520069.10358718
SRR17520069.10358719

SRR17520069.10358720
SRR17520069.10358721
SRR17520069.10358722
SRR17520069.10358723
SRR17520069.10358724
SRR17520069.10358725
SRR17520069.10358726
SRR17520069.10358728
SRR17520069.10358729
SRR17520069.10358730
SRR17520069.10358731
SRR17520069.10358732
SRR17520069.10358733
SRR17520069.10358734
SRR17520069.10358735
SRR17520069.10358736
SRR17520069.10358737
SRR17520069.10358738
SRR17520069.10358739
SRR17520069.10358740
SRR17520069.10358741
SRR17520069.10358742
SRR17520069.10358743
SRR17520069.10358744
SRR17520069.10358745
SRR17520069.10358746
SRR17520069.10358747
SRR17520069.10358748
SRR17520069.10358749
SRR17520069.10358750
SRR17520069.10358751
SRR17520069.10358752
SRR17520069.10358753
SRR17520069.10358754
SRR17520069.10358757
SRR17520069.10358758
SRR17520069.10358759
SRR17520069.10358760
SRR17520069.10358761
SRR17520069.10358762
SRR17520069.10358763
SRR17520069.10358764
SRR17520069.10358765
SRR17520069.10358766
SRR17520069.10358767
SRR17520069.10358768
SRR17520069.10358769

SRR17520069.10358770
SRR17520069.10358771
SRR17520069.10358772
SRR17520069.10358773
SRR17520069.10358774
SRR17520069.10358775
SRR17520069.10358776
SRR17520069.10358777
SRR17520069.10358778
SRR17520069.10358779
SRR17520069.10358780
SRR17520069.10358781
SRR17520069.10358782
SRR17520069.10358783
SRR17520069.10358784
SRR17520069.10358785
SRR17520069.10358786
SRR17520069.10358787
SRR17520069.10358788
SRR17520069.10358789
SRR17520069.10358790
SRR17520069.10358791
SRR17520069.10358792
SRR17520069.10358793
SRR17520069.10358794
SRR17520069.10358795
SRR17520069.10358796
SRR17520069.10358797
SRR17520069.10358799
SRR17520069.10358800
SRR17520069.10358801
SRR17520069.10358802
SRR17520069.10358803
SRR17520069.10358804
SRR17520069.10358805
SRR17520069.10358806
SRR17520069.10358807
SRR17520069.10358808
SRR17520069.10358809
SRR17520069.10358810
SRR17520069.10358811
SRR17520069.10358812
SRR17520069.10358813
SRR17520069.10358814
SRR17520069.10358815
SRR17520069.10358816
SRR17520069.10358817

SRR17520069.10358818
SRR17520069.10358819
SRR17520069.10358820
SRR17520069.10358821
SRR17520069.10358822
SRR17520069.10358823
SRR17520069.10358825
SRR17520069.10358826
SRR17520069.10358827
SRR17520069.10358828
SRR17520069.10358829
SRR17520069.10358830
SRR17520069.10358831
SRR17520069.10358833
SRR17520069.10358834
SRR17520069.10358835
SRR17520069.10363167
SRR17520069.10363193
SRR17520069.10365079
SRR17520069.10365203
SRR17520069.10365204
SRR17520069.10365205
SRR17520069.10365206
SRR17520069.10365207
SRR17520069.10365208
SRR17520069.10365209
SRR17520069.10365211
SRR17520069.10365212
SRR17520069.10365213
SRR17520069.10365214
SRR17520069.10365215
SRR17520069.10365216
SRR17520069.10365217
SRR17520069.10365218
SRR17520069.10365219
SRR17520069.10365220
SRR17520069.10365221
SRR17520069.10365222
SRR17520069.10365223
SRR17520069.10365224
SRR17520069.10365225
SRR17520069.10365226
SRR17520069.10365227
SRR17520069.10365228
SRR17520069.10365229
SRR17520069.10365230
SRR17520069.10365231

SRR17520069.10365232
SRR17520069.10365233
SRR17520069.10365234
SRR17520069.10365235
SRR17520069.10365236
SRR17520069.10365237
SRR17520069.10365238
SRR17520069.10365239
SRR17520069.10365240
SRR17520069.10365241
SRR17520069.10365242
SRR17520069.10365243
SRR17520069.10365244
SRR17520069.10365245
SRR17520069.10365246
SRR17520069.10365247
SRR17520069.10365248
SRR17520069.10365250
SRR17520069.10365251
SRR17520069.10365252
SRR17520069.10365253
SRR17520069.10365254
SRR17520069.10365255
SRR17520069.10365256
SRR17520069.10365257
SRR17520069.10365258
SRR17520069.10365259
SRR17520069.10365260
SRR17520069.10365261
SRR17520069.10365262
SRR17520069.10365263
SRR17520069.10365264
SRR17520069.10365265
SRR17520069.10365266
SRR17520069.10365267
SRR17520069.10365268
SRR17520069.10365269
SRR17520069.10365270
SRR17520069.10365271
SRR17520069.10365272
SRR17520069.10365273
SRR17520069.10374852
SRR17520069.10374853
SRR17520069.10374854
SRR17520069.10374855
SRR17520069.10374856
SRR17520069.10374857

SRR17520069.10374858
SRR17520069.10374859
SRR17520069.10374860
SRR17520069.10374861
SRR17520069.10374862
SRR17520069.10374863
SRR17520069.10374864
SRR17520069.10374865
SRR17520069.10374866
SRR17520069.10374867
SRR17520069.10374868
SRR17520069.10374870
SRR17520069.10374871
SRR17520069.10374872
SRR17520069.10374873
SRR17520069.10374874
SRR17520069.10374875
SRR17520069.10374876
SRR17520069.10374877
SRR17520069.10374878
SRR17520069.10374879
SRR17520069.10376943
SRR17520069.10376944
SRR17520069.10376945
SRR17520069.10376946
SRR17520069.10376947
SRR17520069.10376948
SRR17520069.10376949
SRR17520069.10376950
SRR17520069.10376951
SRR17520069.10376952
SRR17520069.10376953
SRR17520069.10376954
SRR17520069.10376955
SRR17520069.10376956
SRR17520069.10376957
SRR17520069.10376958
SRR17520069.10376959
SRR17520069.10376960
SRR17520069.10376961
SRR17520069.10376962
SRR17520069.10376963
SRR17520069.10376964
SRR17520069.10376965
SRR17520069.10376966
SRR17520069.10376967
SRR17520069.10376968

SRR17520069.10376970
SRR17520069.10376971
SRR17520069.10376972
SRR17520069.10376973
SRR17520069.10376974
SRR17520069.10376975
SRR17520069.10376976
SRR17520069.10376977
SRR17520069.10376978
SRR17520069.10376979
SRR17520069.10376980
SRR17520069.10376981
SRR17520069.10376982
SRR17520069.10376983
SRR17520069.10376984
SRR17520069.10376985
SRR17520069.10376986
SRR17520069.10394186
SRR17520069.10394187
SRR17520069.10394188
SRR17520069.10394189
SRR17520069.10394190
SRR17520069.10394191
SRR17520069.10394192
SRR17520069.10394193
SRR17520069.10394194
SRR17520069.10394195
SRR17520069.10394196
SRR17520069.10394197
SRR17520069.10394198
SRR17520069.10394199
SRR17520069.10394200
SRR17520069.10394201
SRR17520069.10394202
SRR17520069.10394203
SRR17520069.10394204
SRR17520069.10394208
SRR17520069.10394209
SRR17520069.10394210
SRR17520069.10394211
SRR17520069.10394212
SRR17520069.10394213
SRR17520069.10394214
SRR17520069.10394215
SRR17520069.10394216
SRR17520069.10394217
SRR17520069.10394218

SRR17520069.10394219
SRR17520069.10394220
SRR17520069.10394221
SRR17520069.10394222
SRR17520069.10394223
SRR17520069.10394224
SRR17520069.10394225
SRR17520069.10394226
SRR17520069.10394227
SRR17520069.10394228
SRR17520069.10394229
SRR17520069.10394230
SRR17520069.10394231
SRR17520069.10394232
SRR17520069.10394233
SRR17520069.10394234
SRR17520069.10394235
SRR17520069.10394236
SRR17520069.10394237
SRR17520069.10394238
SRR17520069.10394239
SRR17520069.10394240
SRR17520069.10394241
SRR17520069.10394242
SRR17520069.10394243
SRR17520069.10394244
SRR17520069.10394245
SRR17520069.10394246
SRR17520069.10394247
SRR17520069.10394248
SRR17520069.10394249
SRR17520069.10394250
SRR17520069.10394251
SRR17520069.10394252
SRR17520069.10394253
SRR17520069.10394254
SRR17520069.10394255
SRR17520069.10394256
SRR17520069.10394257
SRR17520069.10394258
SRR17520069.10394259
SRR17520069.10404721
SRR17520069.10407077
SRR17520069.10407078
SRR17520069.10407079
SRR17520069.10407080
SRR17520069.10407081

SRR17520069.10407082
SRR17520069.10407083
SRR17520069.10407084
SRR17520069.10407085
SRR17520069.10407086
SRR17520069.10407087
SRR17520069.10407088
SRR17520069.10407089
SRR17520069.10407090
SRR17520069.10407091
SRR17520069.10407092
SRR17520069.10407093
SRR17520069.10407094
SRR17520069.10407095
SRR17520069.10407096
SRR17520069.10407097
SRR17520069.10407098
SRR17520069.10407099
SRR17520069.10407100
SRR17520069.10407101
SRR17520069.10407102
SRR17520069.10407103
SRR17520069.10407105
SRR17520069.10407106
SRR17520069.10407107
SRR17520069.10407108
SRR17520069.10407109
SRR17520069.10407110
SRR17520069.10407111
SRR17520069.10407112
SRR17520069.10407113
SRR17520069.10407114
SRR17520069.10407115
SRR17520069.10407116
SRR17520069.10407117
SRR17520069.10407118
SRR17520069.10407119
SRR17520069.10407120
SRR17520069.10407121
SRR17520069.10407122
SRR17520069.10407123
SRR17520069.10407124
SRR17520069.10407125
SRR17520069.10407126
SRR17520069.10407127
SRR17520069.10407129
SRR17520069.10407130

SRR17520069.10407131
SRR17520069.10407132
SRR17520069.10407133
SRR17520069.10407134
SRR17520069.10407135
SRR17520069.10407136
SRR17520069.10407137
SRR17520069.10407138
SRR17520069.10407139
SRR17520069.10407140
SRR17520069.10407141
SRR17520069.10407142
SRR17520069.10407143
SRR17520069.10407144
SRR17520069.10407145
SRR17520069.10407146
SRR17520069.10407147
SRR17520069.10407148
SRR17520069.10407149
SRR17520069.10407150
SRR17520069.10407151
SRR17520069.10407152
SRR17520069.10407153
SRR17520069.10407154
SRR17520069.10407155
SRR17520069.10407156
SRR17520069.10407157
SRR17520069.10407158
SRR17520069.10407159
SRR17520069.10407160
SRR17520069.10407161
SRR17520069.10407162
SRR17520069.10407163
SRR17520069.10407164
SRR17520069.10407165
SRR17520069.10407166
SRR17520069.10407167
SRR17520069.10407168
SRR17520069.10407169
SRR17520069.10407170
SRR17520069.10407171
SRR17520069.10412234
SRR17520069.10412256
SRR17520069.10420633
SRR17520069.10420634
SRR17520069.10420635
SRR17520069.10420636

SRR17520069.10420637
SRR17520069.10420638
SRR17520069.10420639
SRR17520069.10420641
SRR17520069.10420642
SRR17520069.10420643
SRR17520069.10420644
SRR17520069.10420645
SRR17520069.10420646
SRR17520069.10420647
SRR17520069.10420648
SRR17520069.10420649
SRR17520069.10420650
SRR17520069.10420651
SRR17520069.10420652
SRR17520069.10420653
SRR17520069.10420656
SRR17520069.10420657
SRR17520069.10420658
SRR17520069.10420659
SRR17520069.10420660
SRR17520069.10420661
SRR17520069.10420662
SRR17520069.10420663
SRR17520069.10420664
SRR17520069.10420665
SRR17520069.10420666
SRR17520069.10420668
SRR17520069.10420669
SRR17520069.10420670
SRR17520069.10420671
SRR17520069.10420672
SRR17520069.10420673
SRR17520069.10420674
SRR17520069.10420675
SRR17520069.10420676
SRR17520069.10420677
SRR17520069.10420679
SRR17520069.10420680
SRR17520069.10420681
SRR17520069.10420682
SRR17520069.10420683
SRR17520069.10420684
SRR17520069.10420685
SRR17520069.10420686
SRR17520069.10420688
SRR17520069.10420689

SRR17520069.10420690
SRR17520069.10420692
SRR17520069.10420693
SRR17520069.10420694
SRR17520069.10420695
SRR17520069.10420696
SRR17520069.10420697
SRR17520069.10420699
SRR17520069.10420700
SRR17520069.10420701
SRR17520069.10420702
SRR17520069.10420703
SRR17520069.10420704
SRR17520069.10420705
SRR17520069.10420707
SRR17520069.10420708
SRR17520069.10420709
SRR17520069.10420710
SRR17520069.10420711
SRR17520069.10420712
SRR17520069.10420713
SRR17520069.10420714
SRR17520069.10420715
SRR17520069.10420717
SRR17520069.10420718
SRR17520069.10420719
SRR17520069.10420720
SRR17520069.10420721
SRR17520069.10420722
SRR17520069.10420723
SRR17520069.10420724
SRR17520069.10420725
SRR17520069.10420726
SRR17520069.10428257
SRR17520069.10428258
SRR17520069.10428259
SRR17520069.10428260
SRR17520069.10428261
SRR17520069.10428262
SRR17520069.10428263
SRR17520069.10428264
SRR17520069.10428265
SRR17520069.10428266
SRR17520069.10428267
SRR17520069.10428268
SRR17520069.10428269
SRR17520069.10428270

SRR17520069.10428271
SRR17520069.10428272
SRR17520069.10428273
SRR17520069.10428274
SRR17520069.10428275
SRR17520069.10428276
SRR17520069.10428277
SRR17520069.10428278
SRR17520069.10428279
SRR17520069.10428280
SRR17520069.10428281
SRR17520069.10428282
SRR17520069.10428283
SRR17520069.10428284
SRR17520069.10428285
SRR17520069.10428286
SRR17520069.10428287
SRR17520069.10428288
SRR17520069.10428289
SRR17520069.10428290
SRR17520069.10428291
SRR17520069.10428292
SRR17520069.10428293
SRR17520069.10428294
SRR17520069.10428295
SRR17520069.10428296
SRR17520069.10428297
SRR17520069.10428298
SRR17520069.10428299
SRR17520069.10428300
SRR17520069.10428301
SRR17520069.10428302
SRR17520069.10428303
SRR17520069.10428304
SRR17520069.10428305
SRR17520069.10428306
SRR17520069.10428307
SRR17520069.10428308
SRR17520069.10428309
SRR17520069.10428310
SRR17520069.10428311
SRR17520069.10428312
SRR17520069.10428313
SRR17520069.10428314
SRR17520069.10428315
SRR17520069.10428316
SRR17520069.10428317

SRR17520069.10428318
SRR17520069.10428319
SRR17520069.10428320
SRR17520069.10428321
SRR17520069.10428322
SRR17520069.10428323
SRR17520069.10428324
SRR17520069.10428325
SRR17520069.10428326
SRR17520069.10428327
SRR17520069.10428328
SRR17520069.10428329
SRR17520069.10428330
SRR17520069.10428331
SRR17520069.10428332
SRR17520069.10428333
SRR17520069.10428334
SRR17520069.10428335
SRR17520069.10428336
SRR17520069.10428337
SRR17520069.10428338
SRR17520069.10428339
SRR17520069.10428340
SRR17520069.10428341
SRR17520069.10428342
SRR17520069.10428343
SRR17520069.10428344
SRR17520069.10428345
SRR17520069.10428346
SRR17520069.10428347
SRR17520069.10428348
SRR17520069.10428349
SRR17520069.10428351
SRR17520069.10428352
SRR17520069.10428353
SRR17520069.10428354
SRR17520069.10428355
SRR17520069.10428356
SRR17520069.10428357
SRR17520069.10428358
SRR17520069.10428359
SRR17520069.10428360
SRR17520069.10428361
SRR17520069.10428362
SRR17520069.10428363
SRR17520069.10428364
SRR17520069.10428365

SRR17520069.10428366
SRR17520069.10428367
SRR17520069.10428368
SRR17520069.10428369
SRR17520069.10428370
SRR17520069.10428371
SRR17520069.10428372
SRR17520069.10428373
SRR17520069.10428374
SRR17520069.10428375
SRR17520069.10428376
SRR17520069.10428377
SRR17520069.10428378
SRR17520069.10428379
SRR17520069.10428380
SRR17520069.10428381
SRR17520069.10428382
SRR17520069.10428383
SRR17520069.10428384
SRR17520069.10428385
SRR17520069.10428386
SRR17520069.10428387
SRR17520069.10428388
SRR17520069.10428389
SRR17520069.10428390
SRR17520069.10428391
SRR17520069.10428392
SRR17520069.10428393
SRR17520069.10428394
SRR17520069.10428395
SRR17520069.10428396
SRR17520069.10428397
SRR17520069.10428398
SRR17520069.10428399
SRR17520069.10428400
SRR17520069.10428401
SRR17520069.10428402
SRR17520069.10428403
SRR17520069.10428404
SRR17520069.10428405
SRR17520069.10428406
SRR17520069.10428407
SRR17520069.10428408
SRR17520069.10428409
SRR17520069.10428410
SRR17520069.10428411
SRR17520069.10428412

SRR17520069.10428413
SRR17520069.10428414
SRR17520069.10428415
SRR17520069.10428416
SRR17520069.10428417
SRR17520069.10428418
SRR17520069.10428419
SRR17520069.10428420
SRR17520069.10428421
SRR17520069.10428422
SRR17520069.10428423
SRR17520069.10428425
SRR17520069.10428428
SRR17520069.10428429
SRR17520069.10428430
SRR17520069.10428431
SRR17520069.10428432
SRR17520069.10428433
SRR17520069.10428434
SRR17520069.10428435
SRR17520069.10428437
SRR17520069.10428438
SRR17520069.10428439
SRR17520069.10428440
SRR17520069.10428441
SRR17520069.10428442
SRR17520069.10428443
SRR17520069.10428444
SRR17520069.10428445
SRR17520069.10428446
SRR17520069.10428447
SRR17520069.10428448
SRR17520069.10428449
SRR17520069.10428450
SRR17520069.10428451
SRR17520069.10428452
SRR17520069.10428453
SRR17520069.10428454
SRR17520069.10428455
SRR17520069.10428456
SRR17520069.10428457
SRR17520069.10428458
SRR17520069.10428459
SRR17520069.10428460
SRR17520069.10428461
SRR17520069.10428462
SRR17520069.10428463

SRR17520069.10431180
SRR17520069.10432357
SRR17520069.10432363
SRR17520069.10432373
SRR17520069.10448447
SRR17520069.10448449
SRR17520069.10448450
SRR17520069.10448451
SRR17520069.10448452
SRR17520069.10448456
SRR17520069.10448457
SRR17520069.10448458
SRR17520069.10448459
SRR17520069.10448460
SRR17520069.10448461
SRR17520069.10448462
SRR17520069.10448463
SRR17520069.10448464
SRR17520069.10448465
SRR17520069.10448466
SRR17520069.10448467
SRR17520069.10448469
SRR17520069.10448470
SRR17520069.10448472
SRR17520069.10448473
SRR17520069.10448474
SRR17520069.10448475
SRR17520069.10448476
SRR17520069.10448477
SRR17520069.10448479
SRR17520069.10448481
SRR17520069.10448482
SRR17520069.10448484
SRR17520069.10448485
SRR17520069.10448486
SRR17520069.10448487
SRR17520069.10448489
SRR17520069.10448490
SRR17520069.10448491
SRR17520069.10448493
SRR17520069.10448494
SRR17520069.10448496
SRR17520069.10448497
SRR17520069.10448498
SRR17520069.10448499
SRR17520069.10448500
SRR17520069.10448501

SRR17520069.10448502
SRR17520069.10448503
SRR17520069.10448504
SRR17520069.10448506
SRR17520069.10448507
SRR17520069.10448509
SRR17520069.10448510
SRR17520069.10448511
SRR17520069.10448512
SRR17520069.10448513
SRR17520069.10448516
SRR17520069.10448518
SRR17520069.10448519
SRR17520069.10448523
SRR17520069.10448524
SRR17520069.10448525
SRR17520069.10448526
SRR17520069.10448527
SRR17520069.10448528
SRR17520069.10448529
SRR17520069.10448530
SRR17520069.10448531
SRR17520069.10448532
SRR17520069.10448534
SRR17520069.10448535
SRR17520069.10448536
SRR17520069.10449010
SRR17520069.10449011
SRR17520069.10449012
SRR17520069.10449013
SRR17520069.10449014
SRR17520069.10449015
SRR17520069.10449017
SRR17520069.10449018
SRR17520069.10449019
SRR17520069.10449020
SRR17520069.10449021
SRR17520069.10449022
SRR17520069.10449023
SRR17520069.10449024
SRR17520069.10449025
SRR17520069.10449026
SRR17520069.10449027
SRR17520069.10449028
SRR17520069.10449029
SRR17520069.10449030
SRR17520069.10449031

SRR17520069.10449032
SRR17520069.10449033
SRR17520069.10449034
SRR17520069.10449035
SRR17520069.10449036
SRR17520069.10449037
SRR17520069.10449038
SRR17520069.10449039
SRR17520069.10449040
SRR17520069.10449041
SRR17520069.10449042
SRR17520069.10449043
SRR17520069.10449044
SRR17520069.10449045
SRR17520069.10449046
SRR17520069.10449047
SRR17520069.10449048
SRR17520069.10449049
SRR17520069.10449050
SRR17520069.10449051
SRR17520069.10449052
SRR17520069.10449053
SRR17520069.10449054
SRR17520069.10449055
SRR17520069.10449056
SRR17520069.10449057
SRR17520069.10449059
SRR17520069.10449060
SRR17520069.10449061
SRR17520069.10449062
SRR17520069.10449063
SRR17520069.10449064
SRR17520069.10449065
SRR17520069.10449066
SRR17520069.10449067
SRR17520069.10449068
SRR17520069.10449069
SRR17520069.10449071
SRR17520069.10449073
SRR17520069.10449076
SRR17520069.10449080
SRR17520069.10449081
SRR17520069.10449082
SRR17520069.10449083
SRR17520069.10449084
SRR17520069.10456402
SRR17520069.10459366

SRR17520069.10459367
SRR17520069.10459368
SRR17520069.10459369
SRR17520069.10459370
SRR17520069.10459371
SRR17520069.10459372
SRR17520069.10459373
SRR17520069.10459374
SRR17520069.10459375
SRR17520069.10459376
SRR17520069.10459377
SRR17520069.10459378
SRR17520069.10459379
SRR17520069.10459380
SRR17520069.10459381
SRR17520069.10459382
SRR17520069.10459383
SRR17520069.10459384
SRR17520069.10459385
SRR17520069.10459386
SRR17520069.10459387
SRR17520069.10459388
SRR17520069.10459389
SRR17520069.10459390
SRR17520069.10459391
SRR17520069.10459392
SRR17520069.10459393
SRR17520069.10459394
SRR17520069.10459395
SRR17520069.10459396
SRR17520069.10459397
SRR17520069.10459398
SRR17520069.10459399
SRR17520069.10459400
SRR17520069.10459401
SRR17520069.10459402
SRR17520069.10459403
SRR17520069.10459404
SRR17520069.10459405
SRR17520069.10459406
SRR17520069.10459407
SRR17520069.10459408
SRR17520069.10459409
SRR17520069.10459411
SRR17520069.10459412
SRR17520069.10459413
SRR17520069.10459414

SRR17520069.10459415
SRR17520069.10459416
SRR17520069.10459417
SRR17520069.10459418
SRR17520069.10459419
SRR17520069.10459420
SRR17520069.10459421
SRR17520069.10459422
SRR17520069.10459423
SRR17520069.10459424
SRR17520069.10459425
SRR17520069.10459426
SRR17520069.10459427
SRR17520069.10459428
SRR17520069.10459429
SRR17520069.10459430
SRR17520069.10459431
SRR17520069.10459432
SRR17520069.10459433
SRR17520069.10459434
SRR17520069.10459435
SRR17520069.10459436
SRR17520069.10459437
SRR17520069.10459438
SRR17520069.10459439
SRR17520069.10459440
SRR17520069.10459441
SRR17520069.10459442
SRR17520069.10459443
SRR17520069.10459444
SRR17520069.10459445
SRR17520069.10459446
SRR17520069.10459447
SRR17520069.10459448
SRR17520069.10459449
SRR17520069.10459450
SRR17520069.10459451
SRR17520069.10459452
SRR17520069.10459453
SRR17520069.10459454
SRR17520069.10459455
SRR17520069.10459456
SRR17520069.10459457
SRR17520069.10459458
SRR17520069.10459459
SRR17520069.10459460
SRR17520069.10459461

SRR17520069.10459462
SRR17520069.10459463
SRR17520069.10459464
SRR17520069.10459465
SRR17520069.10459466
SRR17520069.10459467
SRR17520069.10459468
SRR17520069.10459469
SRR17520069.10459470
SRR17520069.10459471
SRR17520069.10459472
SRR17520069.10459473
SRR17520069.10459474
SRR17520069.10459475
SRR17520069.10459477
SRR17520069.10459478
SRR17520069.10459479
SRR17520069.10459480
SRR17520069.10459481
SRR17520069.10459482
SRR17520069.10459483
SRR17520069.10459484
SRR17520069.10459485
SRR17520069.10459486
SRR17520069.10459487
SRR17520069.10459488
SRR17520069.10459489
SRR17520069.10459490
SRR17520069.10459491
SRR17520069.10459492
SRR17520069.10459493
SRR17520069.10459494
SRR17520069.10459495
SRR17520069.10459496
SRR17520069.10459497
SRR17520069.10459498
SRR17520069.10459499
SRR17520069.10459500
SRR17520069.10459501
SRR17520069.10459502
SRR17520069.10459503
SRR17520069.10459504
SRR17520069.10459505
SRR17520069.10459506
SRR17520069.10459507
SRR17520069.10459509
SRR17520069.10459510

SRR17520069.10459511
SRR17520069.10459512
SRR17520069.10459513
SRR17520069.10459514
SRR17520069.10459515
SRR17520069.10459516
SRR17520069.10459517
SRR17520069.10459518
SRR17520069.10459519
SRR17520069.10459520
SRR17520069.10459521
SRR17520069.10459522
SRR17520069.10499017
SRR17520069.10505737
SRR17520069.10547921
SRR17520069.10547922
SRR17520069.10547923
SRR17520069.10547924
SRR17520069.10547925
SRR17520069.10547926
SRR17520069.10547927
SRR17520069.10547928
SRR17520069.10547929
SRR17520069.10547930
SRR17520069.10547931
SRR17520069.10547932
SRR17520069.10547933
SRR17520069.10547934
SRR17520069.10547935
SRR17520069.10547936
SRR17520069.10547937
SRR17520069.10547938
SRR17520069.10547939
SRR17520069.10547940
SRR17520069.10547941
SRR17520069.10547942
SRR17520069.10547943
SRR17520069.10547944
SRR17520069.10547945
SRR17520069.10547946
SRR17520069.10547947
SRR17520069.10547948
SRR17520069.10547949
SRR17520069.10547950
SRR17520069.10547951
SRR17520069.10547952
SRR17520069.10547953

SRR17520069.10547954
SRR17520069.10547955
SRR17520069.10547956
SRR17520069.10547957
SRR17520069.10547958
SRR17520069.10547959
SRR17520069.10547960
SRR17520069.10547961
SRR17520069.10547962
SRR17520069.10547963
SRR17520069.10558427
SRR17520069.10569537
SRR17520069.10569538
SRR17520069.10569539
SRR17520069.10569540
SRR17520069.10569541
SRR17520069.10569542
SRR17520069.10569543
SRR17520069.10569544
SRR17520069.10569545
SRR17520069.10569546
SRR17520069.10569547
SRR17520069.10569548
SRR17520069.10569549
SRR17520069.10569550
SRR17520069.10569551
SRR17520069.10569552
SRR17520069.10569553
SRR17520069.10569554
SRR17520069.10569555
SRR17520069.10569556
SRR17520069.10569557
SRR17520069.10569558
SRR17520069.10569559
SRR17520069.10569560
SRR17520069.10569561
SRR17520069.10569562
SRR17520069.10569563
SRR17520069.10569564
SRR17520069.10569565
SRR17520069.10569566
SRR17520069.10569567
SRR17520069.10569568
SRR17520069.10569569
SRR17520069.10569570
SRR17520069.10569571
SRR17520069.10569572

SRR17520069.10569573
SRR17520069.10569574
SRR17520069.10569575
SRR17520069.10569576
SRR17520069.10569577
SRR17520069.10569578
SRR17520069.10569579
SRR17520069.10569580
SRR17520069.10569581
SRR17520069.10569582
SRR17520069.10569583
SRR17520069.10569584
SRR17520069.10569585
SRR17520069.10569586
SRR17520069.10569587
SRR17520069.10569588
SRR17520069.10569589
SRR17520069.10569590
SRR17520069.10569591
SRR17520069.10569592
SRR17520069.10569593
SRR17520069.10569594
SRR17520069.10569595
SRR17520069.10569596
SRR17520069.10569597
SRR17520069.10569598
SRR17520069.10569599
SRR17520069.10569600
SRR17520069.10569601
SRR17520069.10569602
SRR17520069.10569603
SRR17520069.10569604
SRR17520069.10569605
SRR17520069.10569606
SRR17520069.10569607
SRR17520069.10569608
SRR17520069.10569609
SRR17520069.10569610
SRR17520069.10569611
SRR17520069.10569612
SRR17520069.10569613
SRR17520069.10569614
SRR17520069.10569615
SRR17520069.10569616
SRR17520069.10569617
SRR17520069.10569618
SRR17520069.10569619

SRR17520069.10569620
SRR17520069.10569621
SRR17520069.10569622
SRR17520069.10569623
SRR17520069.10569624
SRR17520069.10569625
SRR17520069.10569626
SRR17520069.10569627
SRR17520069.10569628
SRR17520069.10569629
SRR17520069.10569630
SRR17520069.10569631
SRR17520069.10569632
SRR17520069.10569633
SRR17520069.10569634
SRR17520069.10569635
SRR17520069.10569636
SRR17520069.10569637
SRR17520069.10569638
SRR17520069.10569639
SRR17520069.10569640
SRR17520069.10569641
SRR17520069.10569642
SRR17520069.10569643
SRR17520069.10569644
SRR17520069.10569645
SRR17520069.10569646
SRR17520069.10569647
SRR17520069.10569648
SRR17520069.10569649
SRR17520069.10569650
SRR17520069.10569651
SRR17520069.10569652
SRR17520069.10569653
SRR17520069.10569654
SRR17520069.10569655
SRR17520069.10569656
SRR17520069.10569657
SRR17520069.10569658
SRR17520069.10569659
SRR17520069.10569660
SRR17520069.10569661
SRR17520069.10569662
SRR17520069.10569663
SRR17520069.10569664
SRR17520069.10569665
SRR17520069.10569666

SRR17520069.10569667
SRR17520069.10569668
SRR17520069.10569669
SRR17520069.10569670
SRR17520069.10569671
SRR17520069.10569672
SRR17520069.10569673
SRR17520069.10569674
SRR17520069.10569675
SRR17520069.10569676
SRR17520069.10569677
SRR17520069.10569678
SRR17520069.10569679
SRR17520069.10569680
SRR17520069.10569681
SRR17520069.10569682
SRR17520069.10569683
SRR17520069.10569684
SRR17520069.10581026
SRR17520069.10586093
SRR17520069.10586094
SRR17520069.10586095
SRR17520069.10586096
SRR17520069.10586097
SRR17520069.10586098
SRR17520069.10586099
SRR17520069.10586100
SRR17520069.10586101
SRR17520069.10586102
SRR17520069.10586103
SRR17520069.10586104
SRR17520069.10586105
SRR17520069.10586106
SRR17520069.10586107
SRR17520069.10586108
SRR17520069.10586109
SRR17520069.10586110
SRR17520069.10586111
SRR17520069.10586112
SRR17520069.10586113
SRR17520069.10586114
SRR17520069.10586115
SRR17520069.10586116
SRR17520069.10586117
SRR17520069.10586118
SRR17520069.10586119
SRR17520069.10586120

SRR17520069.10586121
SRR17520069.10586122
SRR17520069.10586123
SRR17520069.10586124
SRR17520069.10586125
SRR17520069.10586126
SRR17520069.10586127
SRR17520069.10586128
SRR17520069.10586129
SRR17520069.10586130
SRR17520069.10586132
SRR17520069.10586133
SRR17520069.10586134
SRR17520069.10586135
SRR17520069.10586136
SRR17520069.10586137
SRR17520069.10586138
SRR17520069.10586139
SRR17520069.10586140
SRR17520069.10586141
SRR17520069.10586142
SRR17520069.10586143
SRR17520069.10586144
SRR17520069.10586145
SRR17520069.10586146
SRR17520069.10586147
SRR17520069.10586148
SRR17520069.10586149
SRR17520069.10586150
SRR17520069.10586151
SRR17520069.10586152
SRR17520069.10586153
SRR17520069.10586154
SRR17520069.10586155
SRR17520069.10586156
SRR17520069.10586157
SRR17520069.10586158
SRR17520069.10586159
SRR17520069.10586160
SRR17520069.10586161
SRR17520069.10586162
SRR17520069.10586163
SRR17520069.10586164
SRR17520069.10586165
SRR17520069.10586166
SRR17520069.10586167
SRR17520069.10586168

SRR17520069.10586169
SRR17520069.10586170
SRR17520069.10586171
SRR17520069.10586172
SRR17520069.10586173
SRR17520069.10586174
SRR17520069.10586175
SRR17520069.10586176
SRR17520069.10586177
SRR17520069.10586178
SRR17520069.10586179
SRR17520069.10586180
SRR17520069.10586182
SRR17520069.10586183
SRR17520069.10586184
SRR17520069.10586185
SRR17520069.10588749
SRR17520069.10596476
SRR17520069.10596477
SRR17520069.10596478
SRR17520069.10596479
SRR17520069.10596480
SRR17520069.10596481
SRR17520069.10596482
SRR17520069.10596483
SRR17520069.10596484
SRR17520069.10596485
SRR17520069.10596486
SRR17520069.10596487
SRR17520069.10596488
SRR17520069.10596489
SRR17520069.10596490
SRR17520069.10596491
SRR17520069.10596492
SRR17520069.10596493
SRR17520069.10596494
SRR17520069.10596495
SRR17520069.10596496
SRR17520069.10596497
SRR17520069.10596498
SRR17520069.10596499
SRR17520069.10596500
SRR17520069.10596501
SRR17520069.10596502
SRR17520069.10596503
SRR17520069.10596504
SRR17520069.10596505

SRR17520069.10596506
SRR17520069.10596507
SRR17520069.10596508
SRR17520069.10596509
SRR17520069.10596510
SRR17520069.10596511
SRR17520069.10596512
SRR17520069.10596514
SRR17520069.10596515
SRR17520069.10596516
SRR17520069.10596517
SRR17520069.10596518
SRR17520069.10596519
SRR17520069.10596520
SRR17520069.10596521
SRR17520069.10596522
SRR17520069.10596524
SRR17520069.10596525
SRR17520069.10596526
SRR17520069.10596527
SRR17520069.10596529
SRR17520069.10596530
SRR17520069.10596531
SRR17520069.10596532
SRR17520069.10596533
SRR17520069.10596534
SRR17520069.10596535
SRR17520069.10596536
SRR17520069.10596537
SRR17520069.10596538
SRR17520069.10596539
SRR17520069.10596540
SRR17520069.10596541
SRR17520069.10596542
SRR17520069.10596543
SRR17520069.10596544
SRR17520069.10596545
SRR17520069.10596546
SRR17520069.10596547
SRR17520069.10596548
SRR17520069.10596549
SRR17520069.10596550
SRR17520069.10596551
SRR17520069.10596552
SRR17520069.10596553
SRR17520069.10596554
SRR17520069.10596555

SRR17520069.10596556
SRR17520069.10596557
SRR17520069.10596558
SRR17520069.10596559
SRR17520069.10596560
SRR17520069.10596561
SRR17520069.10596562
SRR17520069.10596563
SRR17520069.10596564
SRR17520069.10596565
SRR17520069.10596566
SRR17520069.10596567
SRR17520069.10596568
SRR17520069.10596569
SRR17520069.10596570
SRR17520069.10596571
SRR17520069.10596572
SRR17520069.10596573
SRR17520069.10596576
SRR17520069.10596577
SRR17520069.10596578
SRR17520069.10596579
SRR17520069.10596581
SRR17520069.10596582
SRR17520069.10596583
SRR17520069.10596584
SRR17520069.10596585
SRR17520069.10596586
SRR17520069.10596587
SRR17520069.10596588
SRR17520069.10596589
SRR17520069.10596590
SRR17520069.10596591
SRR17520069.10596592
SRR17520069.10596593
SRR17520069.10596594
SRR17520069.10596595
SRR17520069.10596596
SRR17520069.10596597
SRR17520069.10596598
SRR17520069.10596599
SRR17520069.10596600
SRR17520069.10596601
SRR17520069.10596602
SRR17520069.10596603
SRR17520069.10596604
SRR17520069.10596605

SRR17520069.10596606
SRR17520069.10596607
SRR17520069.10596608
SRR17520069.10596609
SRR17520069.10596610
SRR17520069.10596611
SRR17520069.10596612
SRR17520069.10596613
SRR17520069.10596614
SRR17520069.10596615
SRR17520069.10596616
SRR17520069.10596617
SRR17520069.10596618
SRR17520069.10596619
SRR17520069.10596620
SRR17520069.10596621
SRR17520069.10596622
SRR17520069.10596623
SRR17520069.10596624
SRR17520069.10596625
SRR17520069.10596626
SRR17520069.10596627
SRR17520069.10596628
SRR17520069.10596629
SRR17520069.10605698
SRR17520069.10605700
SRR17520069.10605702
SRR17520069.10605703
SRR17520069.10605704
SRR17520069.10605705
SRR17520069.10605706
SRR17520069.10605708
SRR17520069.10605710
SRR17520069.10605711
SRR17520069.10605712
SRR17520069.10605714
SRR17520069.10605715
SRR17520069.10605716
SRR17520069.10605717
SRR17520069.10605718
SRR17520069.10605719
SRR17520069.10605720
SRR17520069.10605721
SRR17520069.10605722
SRR17520069.10605723
SRR17520069.10605724
SRR17520069.10605725

SRR17520069.10605726
SRR17520069.10605728
SRR17520069.10605729
SRR17520069.10605730
SRR17520069.10605731
SRR17520069.10605732
SRR17520069.10605733
SRR17520069.10605734
SRR17520069.10605736
SRR17520069.10605737
SRR17520069.10605738
SRR17520069.10605739
SRR17520069.10605740
SRR17520069.10605741
SRR17520069.10605742
SRR17520069.10605743
SRR17520069.10605744
SRR17520069.10605746
SRR17520069.10605747
SRR17520069.10605748
SRR17520069.10605749
SRR17520069.10605750
SRR17520069.10605751
SRR17520069.10605752
SRR17520069.10605753
SRR17520069.10605754
SRR17520069.10605756
SRR17520069.10605757
SRR17520069.10605759
SRR17520069.10605760
SRR17520069.10605761
SRR17520069.10605762
SRR17520069.10605764
SRR17520069.10605765
SRR17520069.10605766
SRR17520069.10605767
SRR17520069.10605768
SRR17520069.10605770
SRR17520069.10605771
SRR17520069.10605772
SRR17520069.10605773
SRR17520069.10605774
SRR17520069.10605775
SRR17520069.10605776
SRR17520069.10605777
SRR17520069.10605778
SRR17520069.10605779

SRR17520069.10605780
SRR17520069.10605781
SRR17520069.10605782
SRR17520069.10605783
SRR17520069.10605784
SRR17520069.10605785
SRR17520069.10605786
SRR17520069.10605787
SRR17520069.10605788
SRR17520069.10605789
SRR17520069.10605790
SRR17520069.10605791
SRR17520069.10605792
SRR17520069.10605793
SRR17520069.10605794
SRR17520069.10605797
SRR17520069.10605798
SRR17520069.10605799
SRR17520069.10605800
SRR17520069.10605802
SRR17520069.10605803
SRR17520069.10605804
SRR17520069.10605805
SRR17520069.10605806
SRR17520069.10605807
SRR17520069.10605808
SRR17520069.10605809
SRR17520069.10605810
SRR17520069.10605811
SRR17520069.10605812
SRR17520069.10605813
SRR17520069.10605814
SRR17520069.10605815
SRR17520069.10605816
SRR17520069.10605817
SRR17520069.10605818
SRR17520069.10605819
SRR17520069.10605820
SRR17520069.10605821
SRR17520069.10605822
SRR17520069.10605823
SRR17520069.10605824
SRR17520069.10605825
SRR17520069.10605826
SRR17520069.10605827
SRR17520069.10605828
SRR17520069.10605829

SRR17520069.10605830
SRR17520069.10605831
SRR17520069.10605832
SRR17520069.10605833
SRR17520069.10605834
SRR17520069.10605835
SRR17520069.10605836
SRR17520069.10605837
SRR17520069.10605838
SRR17520069.10605839
SRR17520069.10605840
SRR17520069.10605841
SRR17520069.10605842
SRR17520069.10605843
SRR17520069.10605844
SRR17520069.10605845
SRR17520069.10605846
SRR17520069.10605847
SRR17520069.10605849
SRR17520069.10605850
SRR17520069.10605851
SRR17520069.10605852
SRR17520069.10605853
SRR17520069.10605854
SRR17520069.10605855
SRR17520069.10605856
SRR17520069.10605857
SRR17520069.10605858
SRR17520069.10605859
SRR17520069.10605860
SRR17520069.10605861
SRR17520069.10605863
SRR17520069.10605864
SRR17520069.10605865
SRR17520069.10605867
SRR17520069.10605868
SRR17520069.10605869
SRR17520069.10609388
SRR17520069.10609389
SRR17520069.10609390
SRR17520069.10609391
SRR17520069.10609392
SRR17520069.10609393
SRR17520069.10609394
SRR17520069.10609395
SRR17520069.10609396
SRR17520069.10609397

SRR17520069.10609398
SRR17520069.10609399
SRR17520069.10609400
SRR17520069.10609401
SRR17520069.10609402
SRR17520069.10609403
SRR17520069.10609404
SRR17520069.10609405
SRR17520069.10609406
SRR17520069.10609407
SRR17520069.10609408
SRR17520069.10609410
SRR17520069.10609411
SRR17520069.10609412
SRR17520069.10609413
SRR17520069.10609414
SRR17520069.10609415
SRR17520069.10609416
SRR17520069.10609417
SRR17520069.10609418
SRR17520069.10609419
SRR17520069.10609420
SRR17520069.10609421
SRR17520069.10609422
SRR17520069.10609423
SRR17520069.10609424
SRR17520069.10609425
SRR17520069.10609426
SRR17520069.10609427
SRR17520069.10609428
SRR17520069.10609429
SRR17520069.10609430
SRR17520069.10609431
SRR17520069.10609432
SRR17520069.10609433
SRR17520069.10609434
SRR17520069.10609435
SRR17520069.10609436
SRR17520069.10609437
SRR17520069.10609438
SRR17520069.10609439
SRR17520069.10609440
SRR17520069.10609441
SRR17520069.10609442
SRR17520069.10609443
SRR17520069.10609444
SRR17520069.10609445

SRR17520069.10609446
SRR17520069.10609447
SRR17520069.10609448
SRR17520069.10609449
SRR17520069.10609450
SRR17520069.10609452
SRR17520069.10609453
SRR17520069.10609454
SRR17520069.10609455
SRR17520069.10609456
SRR17520069.10609457
SRR17520069.10609458
SRR17520069.10609459
SRR17520069.10609460
SRR17520069.10609461
SRR17520069.10609462
SRR17520069.10609463
SRR17520069.10609464
SRR17520069.10609465
SRR17520069.10609466
SRR17520069.10609467
SRR17520069.10609468
SRR17520069.10609469
SRR17520069.10609470
SRR17520069.10609471
SRR17520069.10609472
SRR17520069.10609473
SRR17520069.10609474
SRR17520069.10609475
SRR17520069.10609476
SRR17520069.10609477
SRR17520069.10609478
SRR17520069.10609479
SRR17520069.10609480
SRR17520069.10609481
SRR17520069.10609483
SRR17520069.10609484
SRR17520069.10609485
SRR17520069.10609486
SRR17520069.10609487
SRR17520069.10609488
SRR17520069.10609489
SRR17520069.10609490
SRR17520069.10609491
SRR17520069.10609492
SRR17520069.10609493
SRR17520069.10609494

SRR17520069.10609495
SRR17520069.10609496
SRR17520069.10609497
SRR17520069.10609498
SRR17520069.10609499
SRR17520069.10609500
SRR17520069.10609501
SRR17520069.10609502
SRR17520069.10609503
SRR17520069.10609504
SRR17520069.10609505
SRR17520069.10609506
SRR17520069.10609507
SRR17520069.10609508
SRR17520069.10609509
SRR17520069.10609510
SRR17520069.10609511
SRR17520069.10609512
SRR17520069.10609513
SRR17520069.10609515
SRR17520069.10609516
SRR17520069.10609517
SRR17520069.10609518
SRR17520069.10609519
SRR17520069.10609520
SRR17520069.10615895
SRR17520069.10615896
SRR17520069.10615897
SRR17520069.10615898
SRR17520069.10615900
SRR17520069.10615901
SRR17520069.10615902
SRR17520069.10615903
SRR17520069.10615904
SRR17520069.10615905
SRR17520069.10615906
SRR17520069.10615907
SRR17520069.10615908
SRR17520069.10615909
SRR17520069.10615910
SRR17520069.10615911
SRR17520069.10615912
SRR17520069.10615913
SRR17520069.10615914
SRR17520069.10615915
SRR17520069.10615916
SRR17520069.10615917

SRR17520069.10615918
SRR17520069.10615919
SRR17520069.10615920
SRR17520069.10615921
SRR17520069.10615922
SRR17520069.10615923
SRR17520069.10615924
SRR17520069.10615925
SRR17520069.10615926
SRR17520069.10615927
SRR17520069.10615928
SRR17520069.10615929
SRR17520069.10615930
SRR17520069.10615931
SRR17520069.10615932
SRR17520069.10615933
SRR17520069.10615934
SRR17520069.10615935
SRR17520069.10615936
SRR17520069.10615937
SRR17520069.10615938
SRR17520069.10615939
SRR17520069.10615940
SRR17520069.10615941
SRR17520069.10615942
SRR17520069.10615943
SRR17520069.10615944
SRR17520069.10615945
SRR17520069.10615946
SRR17520069.10615947
SRR17520069.10615948
SRR17520069.10615949
SRR17520069.10615950
SRR17520069.10615951
SRR17520069.10615952
SRR17520069.10615953
SRR17520069.10615954
SRR17520069.10615955
SRR17520069.10615956
SRR17520069.10615957
SRR17520069.10615958
SRR17520069.10615959
SRR17520069.10615960
SRR17520069.10615961
SRR17520069.10615962
SRR17520069.10615963
SRR17520069.10615964

SRR17520069.10615965
SRR17520069.10615966
SRR17520069.10615967
SRR17520069.10615968
SRR17520069.10615969
SRR17520069.10615970
SRR17520069.10615971
SRR17520069.10615972
SRR17520069.10615973
SRR17520069.10615974
SRR17520069.10615975
SRR17520069.10615976
SRR17520069.10615978
SRR17520069.10615979
SRR17520069.10615980
SRR17520069.10615981
SRR17520069.10615982
SRR17520069.10615983
SRR17520069.10615984
SRR17520069.10615985
SRR17520069.10615986
SRR17520069.10615987
SRR17520069.10615988
SRR17520069.10615989
SRR17520069.10615990
SRR17520069.10615991
SRR17520069.10615992
SRR17520069.10615993
SRR17520069.10615994
SRR17520069.10615995
SRR17520069.10615996
SRR17520069.10615997
SRR17520069.10615998
SRR17520069.10615999
SRR17520069.10616000
SRR17520069.10616001
SRR17520069.10616002
SRR17520069.10616003
SRR17520069.10616004
SRR17520069.10616005
SRR17520069.10616006
SRR17520069.10616007
SRR17520069.10616008
SRR17520069.10616009
SRR17520069.10616010
SRR17520069.10616011
SRR17520069.10616012

SRR17520069.10616013
SRR17520069.10616014
SRR17520069.10616015
SRR17520069.10616016
SRR17520069.10616017
SRR17520069.10616018
SRR17520069.10616019
SRR17520069.10616020
SRR17520069.10616021
SRR17520069.10616022
SRR17520069.10616023
SRR17520069.10616024
SRR17520069.10616025
SRR17520069.10616026
SRR17520069.10616027
SRR17520069.10616028
SRR17520069.10616029
SRR17520069.10616030
SRR17520069.10616031
SRR17520069.10616032
SRR17520069.10616033
SRR17520069.10616034
SRR17520069.10616035
SRR17520069.10616036
SRR17520069.10616037
SRR17520069.10616038
SRR17520069.10616039
SRR17520069.10616040
SRR17520069.10616041
SRR17520069.10616042
SRR17520069.10616043
SRR17520069.10616044
SRR17520069.10616045
SRR17520069.10616046
SRR17520069.10616047
SRR17520069.10616048
SRR17520069.10616049
SRR17520069.10616050
SRR17520069.10616051
SRR17520069.10616052
SRR17520069.10616053
SRR17520069.10616054
SRR17520069.10616055
SRR17520069.10616056
SRR17520069.10616057
SRR17520069.10616058
SRR17520069.10616059

SRR17520069.10616060
SRR17520069.10616061
SRR17520069.10616062
SRR17520069.10616063
SRR17520069.10616064
SRR17520069.10616065
SRR17520069.10616066
SRR17520069.10616067
SRR17520069.10616068
SRR17520069.10616069
SRR17520069.10616070
SRR17520069.10616071
SRR17520069.10616072
SRR17520069.10616073
SRR17520069.10616074
SRR17520069.10616075
SRR17520069.10616076
SRR17520069.10616077
SRR17520069.10616078
SRR17520069.10616080
SRR17520069.10616081
SRR17520069.10616082
SRR17520069.10616083
SRR17520069.10616084
SRR17520069.10616085
SRR17520069.10616086
SRR17520069.10616087
SRR17520069.10616088
SRR17520069.10616089
SRR17520069.10616090
SRR17520069.10616091
SRR17520069.10616092
SRR17520069.10636870
SRR17520069.10637703
SRR17520069.10639091
SRR17520069.10639092
SRR17520069.10639093
SRR17520069.10639094
SRR17520069.10639095
SRR17520069.10639096
SRR17520069.10639097
SRR17520069.10639098
SRR17520069.10639099
SRR17520069.10639100
SRR17520069.10639101
SRR17520069.10639102
SRR17520069.10639103

SRR17520069.10639104
SRR17520069.10639105
SRR17520069.10639106
SRR17520069.10639107
SRR17520069.10639108
SRR17520069.10639109
SRR17520069.10639110
SRR17520069.10639111
SRR17520069.10639112
SRR17520069.10639113
SRR17520069.10639114
SRR17520069.10639115
SRR17520069.10639116
SRR17520069.10639118
SRR17520069.10639119
SRR17520069.10639120
SRR17520069.10639121
SRR17520069.10639122
SRR17520069.10639123
SRR17520069.10639124
SRR17520069.10639125
SRR17520069.10639126
SRR17520069.10639127
SRR17520069.10639128
SRR17520069.10639129
SRR17520069.10639130
SRR17520069.10639131
SRR17520069.10639132
SRR17520069.10639133
SRR17520069.10639134
SRR17520069.10639135
SRR17520069.10639136
SRR17520069.10639137
SRR17520069.10639138
SRR17520069.10641035
SRR17520069.10645001
SRR17520069.10645002
SRR17520069.10645003
SRR17520069.10645005
SRR17520069.10645006
SRR17520069.10645007
SRR17520069.10645008
SRR17520069.10645009
SRR17520069.10645010
SRR17520069.10645011
SRR17520069.10645012
SRR17520069.10645013

SRR17520069.10645014
SRR17520069.10645015
SRR17520069.10645016
SRR17520069.10645017
SRR17520069.10645018
SRR17520069.10645019
SRR17520069.10645020
SRR17520069.10645021
SRR17520069.10645022
SRR17520069.10645023
SRR17520069.10645025
SRR17520069.10645026
SRR17520069.10645027
SRR17520069.10645028
SRR17520069.10645029
SRR17520069.10645030
SRR17520069.10645031
SRR17520069.10645032
SRR17520069.10645033
SRR17520069.10645034
SRR17520069.10645036
SRR17520069.10645037
SRR17520069.10645038
SRR17520069.10645039
SRR17520069.10645040
SRR17520069.10645041
SRR17520069.10645042
SRR17520069.10645043
SRR17520069.10645044
SRR17520069.10645045
SRR17520069.10645046
SRR17520069.10645047
SRR17520069.10645048
SRR17520069.10645049
SRR17520069.10645050
SRR17520069.10645051
SRR17520069.10645052
SRR17520069.10645053
SRR17520069.10645054
SRR17520069.10645055
SRR17520069.10645056
SRR17520069.10645057
SRR17520069.10645058
SRR17520069.10645059
SRR17520069.10645060
SRR17520069.10645061
SRR17520069.10645062

SRR17520069.10645064
SRR17520069.10645065
SRR17520069.10645066
SRR17520069.10645067
SRR17520069.10645068
SRR17520069.10645069
SRR17520069.10645070
SRR17520069.10645071
SRR17520069.10645072
SRR17520069.10645073
SRR17520069.10645074
SRR17520069.10645075
SRR17520069.10645076
SRR17520069.10645077
SRR17520069.10645078
SRR17520069.10645079
SRR17520069.10645080
SRR17520069.10645081
SRR17520069.10645083
SRR17520069.10645084
SRR17520069.10645085
SRR17520069.10645086
SRR17520069.10645087
SRR17520069.10645088
SRR17520069.10645089
SRR17520069.10645090
SRR17520069.10645091
SRR17520069.10645092
SRR17520069.10645093
SRR17520069.10645094
SRR17520069.10645095
SRR17520069.10645096
SRR17520069.10645097
SRR17520069.10645098
SRR17520069.10645099
SRR17520069.10645100
SRR17520069.10645101
SRR17520069.10645102
SRR17520069.10645103
SRR17520069.10645104
SRR17520069.10645105
SRR17520069.10645106
SRR17520069.10645107
SRR17520069.10645108
SRR17520069.10645109
SRR17520069.10645110
SRR17520069.10645111

SRR17520069.10645112
SRR17520069.10645113
SRR17520069.10645114
SRR17520069.10645115
SRR17520069.10645116
SRR17520069.10645117
SRR17520069.10645118
SRR17520069.10645119
SRR17520069.10645120
SRR17520069.10645121
SRR17520069.10645122
SRR17520069.10645123
SRR17520069.10645124
SRR17520069.10645125
SRR17520069.10645126
SRR17520069.10645127
SRR17520069.10645128
SRR17520069.10645129
SRR17520069.10645130
SRR17520069.10645131
SRR17520069.10645132
SRR17520069.10645133
SRR17520069.10645134
SRR17520069.10645135
SRR17520069.10645136
SRR17520069.10645137
SRR17520069.10645138
SRR17520069.10645139
SRR17520069.10645140
SRR17520069.10645141
SRR17520069.10655777
SRR17520069.10655778
SRR17520069.10655779
SRR17520069.10655780
SRR17520069.10655781
SRR17520069.10655782
SRR17520069.10655783
SRR17520069.10655784
SRR17520069.10655785
SRR17520069.10655786
SRR17520069.10655787
SRR17520069.10655788
SRR17520069.10655789
SRR17520069.10658834
SRR17520069.10683580
SRR17520069.10683638
SRR17520069.10683678

SRR17520069.10686371
SRR17520069.10686372
SRR17520069.10686373
SRR17520069.10686374
SRR17520069.10686375
SRR17520069.10686376
SRR17520069.10686377
SRR17520069.10686378
SRR17520069.10686379
SRR17520069.10686380
SRR17520069.10686381
SRR17520069.10686382
SRR17520069.10686383
SRR17520069.10686384
SRR17520069.10686385
SRR17520069.10686386
SRR17520069.10686387
SRR17520069.10686388
SRR17520069.10686389
SRR17520069.10686390
SRR17520069.10686391
SRR17520069.10686392
SRR17520069.10686393
SRR17520069.10686394
SRR17520069.10686395
SRR17520069.10686396
SRR17520069.10686397
SRR17520069.10686398
SRR17520069.10686399
SRR17520069.10686400
SRR17520069.10686401
SRR17520069.10686402
SRR17520069.10686403
SRR17520069.10686404
SRR17520069.10686405
SRR17520069.10686406
SRR17520069.10686407
SRR17520069.10686408
SRR17520069.10686409
SRR17520069.10686410
SRR17520069.10686411
SRR17520069.10686412
SRR17520069.10686413
SRR17520069.10686414
SRR17520069.10686415
SRR17520069.10686416
SRR17520069.10686417

SRR17520069.10686418
SRR17520069.10686419
SRR17520069.10686420
SRR17520069.10686421
SRR17520069.10686422
SRR17520069.10686423
SRR17520069.10686424
SRR17520069.10686425
SRR17520069.10686427
SRR17520069.10686428
SRR17520069.10686429
SRR17520069.10686430
SRR17520069.10686431
SRR17520069.10686432
SRR17520069.10686433
SRR17520069.10686434
SRR17520069.10686435
SRR17520069.10686436
SRR17520069.10686437
SRR17520069.10686438
SRR17520069.10686439
SRR17520069.10686440
SRR17520069.10686441
SRR17520069.10686442
SRR17520069.10686443
SRR17520069.10686445
SRR17520069.10686446
SRR17520069.10686447
SRR17520069.10686448
SRR17520069.10686449
SRR17520069.10686450
SRR17520069.10686451
SRR17520069.10686452
SRR17520069.10686453
SRR17520069.10686454
SRR17520069.10686455
SRR17520069.10686456
SRR17520069.10686457
SRR17520069.10686458
SRR17520069.10686459
SRR17520069.10686460
SRR17520069.10686461
SRR17520069.10686462
SRR17520069.10686463
SRR17520069.10686464
SRR17520069.10686465
SRR17520069.10686466

SRR17520069.10686467
SRR17520069.10686468
SRR17520069.10686469
SRR17520069.10686470
SRR17520069.10686471
SRR17520069.10690873
SRR17520069.10704882
SRR17520069.10704883
SRR17520069.10704884
SRR17520069.10704885
SRR17520069.10704886
SRR17520069.10704887
SRR17520069.10704888
SRR17520069.10704889
SRR17520069.10704890
SRR17520069.10704891
SRR17520069.10704892
SRR17520069.10704893
SRR17520069.10704894
SRR17520069.10715397
SRR17520069.10715398
SRR17520069.10715399
SRR17520069.10715400
SRR17520069.10715401
SRR17520069.10715402
SRR17520069.10715404
SRR17520069.10715405
SRR17520069.10715406
SRR17520069.10715407
SRR17520069.10715408
SRR17520069.10716004
SRR17520069.10716005
SRR17520069.10716006
SRR17520069.10716007
SRR17520069.10716008
SRR17520069.10716009
SRR17520069.10716010
SRR17520069.10716011
SRR17520069.10716012
SRR17520069.10716014
SRR17520069.10717865
SRR17520069.10717866
SRR17520069.10717867
SRR17520069.10717868
SRR17520069.10717869
SRR17520069.10717870
SRR17520069.10717871

SRR17520069.10717872
SRR17520069.10717873
SRR17520069.10717874
SRR17520069.10717875
SRR17520069.10717876
SRR17520069.10717877
SRR17520069.10717878
SRR17520069.10717879
SRR17520069.10717880
SRR17520069.10717881
SRR17520069.10717882
SRR17520069.10727937
SRR17520069.10727938
SRR17520069.10727940
SRR17520069.10727941
SRR17520069.10727942
SRR17520069.10727943
SRR17520069.10727945
SRR17520069.10727946
SRR17520069.10727947
SRR17520069.10727948
SRR17520069.10727949
SRR17520069.10727951
SRR17520069.10727952
SRR17520069.10727953
SRR17520069.10727954
SRR17520069.10727955
SRR17520069.10727956
SRR17520069.10727958
SRR17520069.10727959
SRR17520069.10727960
SRR17520069.10727961
SRR17520069.10727962
SRR17520069.10727963
SRR17520069.10727964
SRR17520069.10727966
SRR17520069.10727967
SRR17520069.10727968
SRR17520069.10727969
SRR17520069.10727970
SRR17520069.10727972
SRR17520069.10727973
SRR17520069.10727974
SRR17520069.10727976
SRR17520069.10727977
SRR17520069.10727978
SRR17520069.10727979

SRR17520069.10727980
SRR17520069.10727981
SRR17520069.10727982
SRR17520069.10727983
SRR17520069.10727984
SRR17520069.10727985
SRR17520069.10727986
SRR17520069.10727987
SRR17520069.10727988
SRR17520069.10727989
SRR17520069.10727990
SRR17520069.10727991
SRR17520069.10727992
SRR17520069.10739908
SRR17520069.10744079
SRR17520069.10744109
SRR17520069.10745138
SRR17520069.10747658
SRR17520069.10747659
SRR17520069.10747660
SRR17520069.10747661
SRR17520069.10754087
SRR17520069.10754088
SRR17520069.10754089
SRR17520069.10754090
SRR17520069.10754093
SRR17520069.10754094
SRR17520069.10754095
SRR17520069.10754096
SRR17520069.10754097
SRR17520069.10754098
SRR17520069.10754099
SRR17520069.10754100
SRR17520069.10754101
SRR17520069.10754102
SRR17520069.10754104
SRR17520069.10754105
SRR17520069.10754107
SRR17520069.10754108
SRR17520069.10754109
SRR17520069.10754110
SRR17520069.10754112
SRR17520069.10754113
SRR17520069.10754115
SRR17520069.10754116
SRR17520069.10754117
SRR17520069.10754118

SRR17520069.10754119
SRR17520069.10754120
SRR17520069.10768266
SRR17520069.10768267
SRR17520069.10768268
SRR17520069.10768269
SRR17520069.10768270
SRR17520069.10768271
SRR17520069.10768272
SRR17520069.10768273
SRR17520069.10768274
SRR17520069.10768275
SRR17520069.10768276
SRR17520069.10768277
SRR17520069.10768278
SRR17520069.10768280
SRR17520069.10768281
SRR17520069.10768282
SRR17520069.10768283
SRR17520069.10768284
SRR17520069.10768285
SRR17520069.10768286
SRR17520069.10768287
SRR17520069.10768288
SRR17520069.10768289
SRR17520069.10768290
SRR17520069.10768291
SRR17520069.10768292
SRR17520069.10768293
SRR17520069.10768294
SRR17520069.10768295
SRR17520069.10768296
SRR17520069.10768297
SRR17520069.10768298
SRR17520069.10768299
SRR17520069.10768300
SRR17520069.10768301
SRR17520069.10768302
SRR17520069.10782442
SRR17520069.10782443
SRR17520069.10782444
SRR17520069.10782445
SRR17520069.10782446
SRR17520069.10782447
SRR17520069.10782448
SRR17520069.10782449
SRR17520069.10782450

SRR17520069.10782451
SRR17520069.10782452
SRR17520069.10782453
SRR17520069.10782454
SRR17520069.10782455
SRR17520069.10782456
SRR17520069.10782457
SRR17520069.10782458
SRR17520069.10782459
SRR17520069.10782460
SRR17520069.10782461
SRR17520069.10782463
SRR17520069.10782464
SRR17520069.10782465
SRR17520069.10782466
SRR17520069.10782467
SRR17520069.10782468
SRR17520069.10782469
SRR17520069.10782470
SRR17520069.10782471
SRR17520069.10782472
SRR17520069.10782473
SRR17520069.10782474
SRR17520069.10782475
SRR17520069.10782476
SRR17520069.10782477
SRR17520069.10782479
SRR17520069.10782480
SRR17520069.10782481
SRR17520069.10782482
SRR17520069.10782483
SRR17520069.10782484
SRR17520069.10782485
SRR17520069.10782486
SRR17520069.10782487
SRR17520069.10782488
SRR17520069.10782489
SRR17520069.10782490
SRR17520069.10782491
SRR17520069.10782492
SRR17520069.10782493
SRR17520069.10782494
SRR17520069.10782495
SRR17520069.10782496
SRR17520069.10782497
SRR17520069.10782498
SRR17520069.10782499

SRR17520069.10782500
SRR17520069.10782501
SRR17520069.10782502
SRR17520069.10782503
SRR17520069.10782504
SRR17520069.10782505
SRR17520069.10782506
SRR17520069.10782507
SRR17520069.10782508
SRR17520069.10782509
SRR17520069.10782510
SRR17520069.10782511
SRR17520069.10782512
SRR17520069.10782513
SRR17520069.10782514
SRR17520069.10782515
SRR17520069.10782516
SRR17520069.10782517
SRR17520069.10782518
SRR17520069.10782519
SRR17520069.10782520
SRR17520069.10782521
SRR17520069.10782523
SRR17520069.10782524
SRR17520069.10782525
SRR17520069.10782526
SRR17520069.10782527
SRR17520069.10782528
SRR17520069.10782529
SRR17520069.10782530
SRR17520069.10791256
SRR17520069.10791258
SRR17520069.10811023
SRR17520069.10811024
SRR17520069.10824469
SRR17520069.10852984
SRR17520069.10859065
SRR17520069.10863023
SRR17520069.10863024
SRR17520069.10863025
SRR17520069.10863026
SRR17520069.10863027
SRR17520069.10863028
SRR17520069.10863029
SRR17520069.10885996
SRR17520069.10886002
SRR17520069.10886014

SRR17520069.10898139
SRR17520069.10904092
SRR17520069.10906108
SRR17520069.10906109
SRR17520069.10912152
SRR17520069.10912153
SRR17520069.10912155
SRR17520069.10912156
SRR17520069.10912157
SRR17520069.10912160
SRR17520069.10912161
SRR17520069.10912162
SRR17520069.10935434
SRR17520069.10938570
SRR17520069.10960209
SRR17520069.10971172
SRR17520069.10976391
SRR17520069.10976392
SRR17520069.10976393
SRR17520069.10976394
SRR17520069.10976395
SRR17520069.10976396
SRR17520069.10976397
SRR17520069.10976398
SRR17520069.10976399
SRR17520069.10976401
SRR17520069.10976402
SRR17520069.10976403
SRR17520069.10976404
SRR17520069.10976405
SRR17520069.10976406
SRR17520069.10976407
SRR17520069.10976408
SRR17520069.10976409
SRR17520069.10976410
SRR17520069.10976411
SRR17520069.10976413
SRR17520069.10976414
SRR17520069.10976415
SRR17520069.10976416
SRR17520069.10976417
SRR17520069.10976418
SRR17520069.10976419
SRR17520069.10976420
SRR17520069.10976421
SRR17520069.10976422
SRR17520069.10976423

SRR17520069.10976775
SRR17520069.10986013
SRR17520069.11012451
SRR17520069.11012452
SRR17520069.11012454
SRR17520069.11012455
SRR17520069.11012456
SRR17520069.11012457
SRR17520069.11012458
SRR17520069.11012459
SRR17520069.11012460
SRR17520069.11012461
SRR17520069.11012463
SRR17520069.11012464
SRR17520069.11012465
SRR17520069.11012466
SRR17520069.11012467
SRR17520069.11012468
SRR17520069.11012469
SRR17520069.11012470
SRR17520069.11012471
SRR17520069.11012472
SRR17520069.11012473
SRR17520069.11012474
SRR17520069.11012475
SRR17520069.11012476
SRR17520069.11012477
SRR17520069.11012478
SRR17520069.11012479
SRR17520069.11012480
SRR17520069.11012481
SRR17520069.11012482
SRR17520069.11012483
SRR17520069.11012484
SRR17520069.11012485
SRR17520069.11012486
SRR17520069.11012487
SRR17520069.11012488
SRR17520069.11012489
SRR17520069.11012490
SRR17520069.11012491
SRR17520069.11012492
SRR17520069.11012493
SRR17520069.11012495
SRR17520069.11012496
SRR17520069.11012497
SRR17520069.11012498

SRR17520069.11012499
SRR17520069.11012500
SRR17520069.11012501
SRR17520069.11012502
SRR17520069.11012503
SRR17520069.11012504
SRR17520069.11012505
SRR17520069.11012506
SRR17520069.11012507
SRR17520069.11012508
SRR17520069.11012509
SRR17520069.11012510
SRR17520069.11012511
SRR17520069.11012512
SRR17520069.11012513
SRR17520069.11012515
SRR17520069.11012516
SRR17520069.11012517
SRR17520069.11012518
SRR17520069.11012519
SRR17520069.11012520
SRR17520069.11012521
SRR17520069.11012522
SRR17520069.11012523
SRR17520069.11012524
SRR17520069.11012525
SRR17520069.11012526
SRR17520069.11012527
SRR17520069.11012528
SRR17520069.11012529
SRR17520069.11012530
SRR17520069.11012531
SRR17520069.11012532
SRR17520069.11012533
SRR17520069.11012534
SRR17520069.11012535
SRR17520069.11012536
SRR17520069.11012537
SRR17520069.11012538
SRR17520069.11012539
SRR17520069.11012540
SRR17520069.11012541
SRR17520069.11012542
SRR17520069.11012543
SRR17520069.11012544
SRR17520069.11012545
SRR17520069.11012546

SRR17520069.11012547
SRR17520069.11012548
SRR17520069.11012549
SRR17520069.11012550
SRR17520069.11012551
SRR17520069.11012552
SRR17520069.11012553
SRR17520069.11012554
SRR17520069.11012556
SRR17520069.11012557
SRR17520069.11012558
SRR17520069.11012559
SRR17520069.11012560
SRR17520069.11012561
SRR17520069.11012562
SRR17520069.11012563
SRR17520069.11012564
SRR17520069.11012565
SRR17520069.11012566
SRR17520069.11012567
SRR17520069.11012568
SRR17520069.11012569
SRR17520069.11012570
SRR17520069.11012571
SRR17520069.11012572
SRR17520069.11012573
SRR17520069.11012574
SRR17520069.11012575
SRR17520069.11012576
SRR17520069.11012577
SRR17520069.11012578
SRR17520069.11012579
SRR17520069.11012580
SRR17520069.11012581
SRR17520069.11012582
SRR17520069.11012583
SRR17520069.11012584
SRR17520069.11012585
SRR17520069.11012586
SRR17520069.11012587
SRR17520069.11012588
SRR17520069.11012589
SRR17520069.11012590
SRR17520069.11012591
SRR17520069.11012592
SRR17520069.11012593
SRR17520069.11012594

SRR17520069.11012595
SRR17520069.11012596
SRR17520069.11012597
SRR17520069.11012598
SRR17520069.11017841
SRR17520069.11022621
SRR17520069.11022622
SRR17520069.11022623
SRR17520069.11022624
SRR17520069.11022625
SRR17520069.11022626
SRR17520069.11022627
SRR17520069.11022628
SRR17520069.11036146
SRR17520069.11036150
SRR17520069.11036166
SRR17520069.11070259
SRR17520069.11070260
SRR17520069.11070262
SRR17520069.11070264
SRR17520069.11070265
SRR17520069.11070266
SRR17520069.11070267
SRR17520069.11070269
SRR17520069.11070270
SRR17520069.11070271
SRR17520069.11070272
SRR17520069.11070273
SRR17520069.11070274
SRR17520069.11070275
SRR17520069.11070276
SRR17520069.11070277
SRR17520069.11070278
SRR17520069.11070279
SRR17520069.11070280
SRR17520069.11070281
SRR17520069.11070282
SRR17520069.11070283
SRR17520069.11070284
SRR17520069.11070285
SRR17520069.11070286
SRR17520069.11070287
SRR17520069.11070289
SRR17520069.11070290
SRR17520069.11070291
SRR17520069.11070292
SRR17520069.11070293

SRR17520069.11070294
SRR17520069.11070295
SRR17520069.11070296
SRR17520069.11070297
SRR17520069.11070298
SRR17520069.11070299
SRR17520069.11070300
SRR17520069.11070301
SRR17520069.11070302
SRR17520069.11070303
SRR17520069.11070304
SRR17520069.11070305
SRR17520069.11076267
SRR17520069.11076268
SRR17520069.11076269
SRR17520069.11076270
SRR17520069.11076271
SRR17520069.11076272
SRR17520069.11076273
SRR17520069.11076274
SRR17520069.11076275
SRR17520069.11076276
SRR17520069.11076277
SRR17520069.11076278
SRR17520069.11076279
SRR17520069.11076280
SRR17520069.11076281
SRR17520069.11076282
SRR17520069.11078351
SRR17520069.11078352
SRR17520069.11078353
SRR17520069.11078354
SRR17520069.11078355
SRR17520069.11078356
SRR17520069.11078357
SRR17520069.11078358
SRR17520069.11078359
SRR17520069.11078360
SRR17520069.11078361
SRR17520069.11078362
SRR17520069.11078363
SRR17520069.11078364
SRR17520069.11078365
SRR17520069.11078366
SRR17520069.11078367
SRR17520069.11078369
SRR17520069.11078370

SRR17520069.11078371
SRR17520069.11078372
SRR17520069.11078373
SRR17520069.11078374
SRR17520069.11078375
SRR17520069.11078376
SRR17520069.11078377
SRR17520069.11078378
SRR17520069.11078379
SRR17520069.11078380
SRR17520069.11078381
SRR17520069.11078382
SRR17520069.11078384
SRR17520069.11078385
SRR17520069.11078386
SRR17520069.11078387
SRR17520069.11078388
SRR17520069.11078389
SRR17520069.11078390
SRR17520069.11078391
SRR17520069.11078392
SRR17520069.11079281
SRR17520069.11079282
SRR17520069.11079283
SRR17520069.11079284
SRR17520069.11079285
SRR17520069.11079286
SRR17520069.11079287
SRR17520069.11079288
SRR17520069.11079289
SRR17520069.11079290
SRR17520069.11079291
SRR17520069.11079292
SRR17520069.11079293
SRR17520069.11079294
SRR17520069.11079295
SRR17520069.11079296
SRR17520069.11118974
SRR17520069.11118998
SRR17520069.11119010
SRR17520069.11119073
SRR17520069.11126134
SRR17520069.11126135
SRR17520069.11126136
SRR17520069.11126138
SRR17520069.11126139
SRR17520069.11126140

SRR17520069.11126141
SRR17520069.11126142
SRR17520069.11126143
SRR17520069.11126144
SRR17520069.11126145
SRR17520069.11126146
SRR17520069.11126147
SRR17520069.11126148
SRR17520069.11126149
SRR17520069.11126150
SRR17520069.11126151
SRR17520069.11126152
SRR17520069.11126153
SRR17520069.11126154
SRR17520069.11126155
SRR17520069.11126156
SRR17520069.11126157
SRR17520069.11126158
SRR17520069.11126159
SRR17520069.11126160
SRR17520069.11126162
SRR17520069.11126163
SRR17520069.11126164
SRR17520069.11126165
SRR17520069.11126166
SRR17520069.11126167
SRR17520069.11126168
SRR17520069.11126169
SRR17520069.11126171
SRR17520069.11126172
SRR17520069.11126173
SRR17520069.11126174
SRR17520069.11126175
SRR17520069.11126176
SRR17520069.11126177
SRR17520069.11126178
SRR17520069.11126179
SRR17520069.11126180
SRR17520069.11126181
SRR17520069.11126182
SRR17520069.11126183
SRR17520069.11126184
SRR17520069.11126185
SRR17520069.11126186
SRR17520069.11126187
SRR17520069.11126188
SRR17520069.11126189

SRR17520069.11126190
SRR17520069.11126191
SRR17520069.11126192
SRR17520069.11126193
SRR17520069.11126194
SRR17520069.11126195
SRR17520069.11126196
SRR17520069.11126197
SRR17520069.11126198
SRR17520069.11126199
SRR17520069.11126200
SRR17520069.11126201
SRR17520069.11126202
SRR17520069.11126203
SRR17520069.11126204
SRR17520069.11126205
SRR17520069.11126206
SRR17520069.11126207
SRR17520069.11126208
SRR17520069.11126209
SRR17520069.11126210
SRR17520069.11126211
SRR17520069.11126212
SRR17520069.11126213
SRR17520069.11126214
SRR17520069.11126215
SRR17520069.11126216
SRR17520069.11126217
SRR17520069.11126218
SRR17520069.11126219
SRR17520069.11126220
SRR17520069.11126221
SRR17520069.11126222
SRR17520069.11126223
SRR17520069.11126224
SRR17520069.11126225
SRR17520069.11126226
SRR17520069.11126227
SRR17520069.11126228
SRR17520069.11126229
SRR17520069.11126230
SRR17520069.11126231
SRR17520069.11126232
SRR17520069.11126233
SRR17520069.11126234
SRR17520069.11126235
SRR17520069.11126236

SRR17520069.11126237
SRR17520069.11126238
SRR17520069.11126239
SRR17520069.11126240
SRR17520069.11126241
SRR17520069.11126242
SRR17520069.11126243
SRR17520069.11126244
SRR17520069.11126245
SRR17520069.11126246
SRR17520069.11126247
SRR17520069.11126248
SRR17520069.11126249
SRR17520069.11126250
SRR17520069.11126251
SRR17520069.11126252
SRR17520069.11126253
SRR17520069.11126254
SRR17520069.11126255
SRR17520069.11126256
SRR17520069.11126257
SRR17520069.11126260
SRR17520069.11126261
SRR17520069.11126262
SRR17520069.11126263
SRR17520069.11126264
SRR17520069.11126265
SRR17520069.11126266
SRR17520069.11126267
SRR17520069.11126268
SRR17520069.11126269
SRR17520069.11126270
SRR17520069.11135965
SRR17520069.11135970
SRR17520069.11140086
SRR17520069.11140087
SRR17520069.11140088
SRR17520069.11140089
SRR17520069.11140090
SRR17520069.11140091
SRR17520069.11140092
SRR17520069.11140093
SRR17520069.11140094
SRR17520069.11140095
SRR17520069.11140096
SRR17520069.11140097
SRR17520069.11140098

SRR17520069.11140099
SRR17520069.11140100
SRR17520069.11140101
SRR17520069.11140102
SRR17520069.11140103
SRR17520069.11140104
SRR17520069.11140105
SRR17520069.11140106
SRR17520069.11140107
SRR17520069.11140108
SRR17520069.11140109
SRR17520069.11140110
SRR17520069.11140111
SRR17520069.11140112
SRR17520069.11140113
SRR17520069.11140114
SRR17520069.11140115
SRR17520069.11140116
SRR17520069.11140117
SRR17520069.11140118
SRR17520069.11140119
SRR17520069.11140120
SRR17520069.11140121
SRR17520069.11140122
SRR17520069.11140123
SRR17520069.11140124
SRR17520069.11140125
SRR17520069.11140126
SRR17520069.11140127
SRR17520069.11140128
SRR17520069.11140129
SRR17520069.11140130
SRR17520069.11140131
SRR17520069.11140132
SRR17520069.11140133
SRR17520069.11140134
SRR17520069.11140135
SRR17520069.11140136
SRR17520069.11140137
SRR17520069.11140138
SRR17520069.11140139
SRR17520069.11140140
SRR17520069.11140141
SRR17520069.11140142
SRR17520069.11140143
SRR17520069.11140144
SRR17520069.11140145

SRR17520069.11140146
SRR17520069.11140147
SRR17520069.11140148
SRR17520069.11140149
SRR17520069.11140150
SRR17520069.11140151
SRR17520069.11140152
SRR17520069.11140153
SRR17520069.11140154
SRR17520069.11140155
SRR17520069.11140156
SRR17520069.11140157
SRR17520069.11140158
SRR17520069.11140159
SRR17520069.11140160
SRR17520069.11140161
SRR17520069.11140162
SRR17520069.11140163
SRR17520069.11140164
SRR17520069.11140165
SRR17520069.11140167
SRR17520069.11140168
SRR17520069.11140169
SRR17520069.11140170
SRR17520069.11140171
SRR17520069.11140172
SRR17520069.11140173
SRR17520069.11140174
SRR17520069.11140175
SRR17520069.11140176
SRR17520069.11140177
SRR17520069.11140178
SRR17520069.11140179
SRR17520069.11140180
SRR17520069.11140181
SRR17520069.11140182
SRR17520069.11140183
SRR17520069.11140184
SRR17520069.11140185
SRR17520069.11140186
SRR17520069.11140187
SRR17520069.11140188
SRR17520069.11140189
SRR17520069.11140191
SRR17520069.11140192
SRR17520069.11140193
SRR17520069.11140194

SRR17520069.11140195
SRR17520069.11140196
SRR17520069.11140197
SRR17520069.11140198
SRR17520069.11140199
SRR17520069.11140200
SRR17520069.11140201
SRR17520069.11140202
SRR17520069.11140203
SRR17520069.11140204
SRR17520069.11140205
SRR17520069.11140206
SRR17520069.11140207
SRR17520069.11140208
SRR17520069.11140209
SRR17520069.11140210
SRR17520069.11140211
SRR17520069.11140212
SRR17520069.11140213
SRR17520069.11140214
SRR17520069.11140215
SRR17520069.11140216
SRR17520069.11140217
SRR17520069.11140218
SRR17520069.11140219
SRR17520069.11140220
SRR17520069.11140221
SRR17520069.11140222
SRR17520069.11140223
SRR17520069.11140224
SRR17520069.11140225
SRR17520069.11140226
SRR17520069.11140227
SRR17520069.11140228
SRR17520069.11140229
SRR17520069.11140230
SRR17520069.11140231
SRR17520069.11140232
SRR17520069.11140233
SRR17520069.11140234
SRR17520069.11140235
SRR17520069.11140236
SRR17520069.11140237
SRR17520069.11140238
SRR17520069.11140239
SRR17520069.11140240
SRR17520069.11140241

SRR17520069.11140242
SRR17520069.11140243
SRR17520069.11140244
SRR17520069.11140245
SRR17520069.11140246
SRR17520069.11140247
SRR17520069.11140248
SRR17520069.11140249
SRR17520069.11140250
SRR17520069.11149047
SRR17520069.11149048
SRR17520069.11160556
SRR17520069.11160557
SRR17520069.11160558
SRR17520069.11160559
SRR17520069.11160560
SRR17520069.11160561
SRR17520069.11160562
SRR17520069.11160563
SRR17520069.11160564
SRR17520069.11160565
SRR17520069.11160566
SRR17520069.11160567
SRR17520069.11160569
SRR17520069.11160570
SRR17520069.11160571
SRR17520069.11160572
SRR17520069.11160573
SRR17520069.11160574
SRR17520069.11160575
SRR17520069.11160577
SRR17520069.11160578
SRR17520069.11160580
SRR17520069.11160581
SRR17520069.11160582
SRR17520069.11160583
SRR17520069.11160585
SRR17520069.11160591
SRR17520069.11160595
SRR17520069.11160596
SRR17520069.11160597
SRR17520069.11160598
SRR17520069.11160599
SRR17520069.11160600
SRR17520069.11160601
SRR17520069.11160602
SRR17520069.11160603

SRR17520069.11160604
SRR17520069.11160605
SRR17520069.11160607
SRR17520069.11160608
SRR17520069.11160609
SRR17520069.11160611
SRR17520069.11160612
SRR17520069.11160613
SRR17520069.11160615
SRR17520069.11160618
SRR17520069.11160620
SRR17520069.11160621
SRR17520069.11160622
SRR17520069.11160623
SRR17520069.11160624
SRR17520069.11160625
SRR17520069.11160626
SRR17520069.11160627
SRR17520069.11160628
SRR17520069.11160629
SRR17520069.11160630
SRR17520069.11160632
SRR17520069.11160634
SRR17520069.11160635
SRR17520069.11160636
SRR17520069.11161179
SRR17520069.11161180
SRR17520069.11161181
SRR17520069.11161182
SRR17520069.11161183
SRR17520069.11161184
SRR17520069.11161185
SRR17520069.11161186
SRR17520069.11161187
SRR17520069.11161188
SRR17520069.11161189
SRR17520069.11161190
SRR17520069.11161191
SRR17520069.11161192
SRR17520069.11161193
SRR17520069.11161194
SRR17520069.11161195
SRR17520069.11161196
SRR17520069.11161197
SRR17520069.11161198
SRR17520069.11161199
SRR17520069.11161200

SRR17520069.11161201
SRR17520069.11161202
SRR17520069.11161203
SRR17520069.11161204
SRR17520069.11161205
SRR17520069.11161206
SRR17520069.11161207
SRR17520069.11161208
SRR17520069.11161209
SRR17520069.11161210
SRR17520069.11161211
SRR17520069.11161212
SRR17520069.11161213
SRR17520069.11161214
SRR17520069.11161215
SRR17520069.11161216
SRR17520069.11161217
SRR17520069.11161218
SRR17520069.11161219
SRR17520069.11161221
SRR17520069.11161222
SRR17520069.11161223
SRR17520069.11161224
SRR17520069.11161225
SRR17520069.11161226
SRR17520069.11161227
SRR17520069.11161228
SRR17520069.11161229
SRR17520069.11161230
SRR17520069.11161231
SRR17520069.11161232
SRR17520069.11161233
SRR17520069.11161234
SRR17520069.11161235
SRR17520069.11161236
SRR17520069.11161237
SRR17520069.11161238
SRR17520069.11161239
SRR17520069.11161240
SRR17520069.11161241
SRR17520069.11161242
SRR17520069.11161243
SRR17520069.11161245
SRR17520069.11161246
SRR17520069.11161247
SRR17520069.11161248
SRR17520069.11161249

SRR17520069.11161250
SRR17520069.11161251
SRR17520069.11161252
SRR17520069.11161253
SRR17520069.11161254
SRR17520069.11161255
SRR17520069.11161256
SRR17520069.11161257
SRR17520069.11161258
SRR17520069.11161259
SRR17520069.11161260
SRR17520069.11161261
SRR17520069.11161262
SRR17520069.11161263
SRR17520069.11161264
SRR17520069.11161265
SRR17520069.11161266
SRR17520069.11161267
SRR17520069.11161268
SRR17520069.11161269
SRR17520069.11161270
SRR17520069.11161271
SRR17520069.11161272
SRR17520069.11161273
SRR17520069.11161274
SRR17520069.11161276
SRR17520069.11161277
SRR17520069.11161278
SRR17520069.11161279
SRR17520069.11161280
SRR17520069.11161281
SRR17520069.11161282
SRR17520069.11161283
SRR17520069.11161284
SRR17520069.11161285
SRR17520069.11187327
SRR17520069.11187349
SRR17520069.11187942
SRR17520069.11187943
SRR17520069.11187944
SRR17520069.11187945
SRR17520069.11187946
SRR17520069.11187947
SRR17520069.11187962
SRR17520069.11187963
SRR17520069.11187970
SRR17520069.11187972

SRR17520069.11187980
SRR17520069.11192597
SRR17520069.11192598
SRR17520069.11192599
SRR17520069.11192600
SRR17520069.11192601
SRR17520069.11192602
SRR17520069.11192603
SRR17520069.11192604
SRR17520069.11192605
SRR17520069.11192606
SRR17520069.11192607
SRR17520069.11192609
SRR17520069.11192610
SRR17520069.11192611
SRR17520069.11192612
SRR17520069.11192613
SRR17520069.11192614
SRR17520069.11192617
SRR17520069.11192618
SRR17520069.11192619
SRR17520069.11192620
SRR17520069.11192621
SRR17520069.11194422
SRR17520069.11235550
SRR17520069.11235551
SRR17520069.11235552
SRR17520069.11235553
SRR17520069.11235554
SRR17520069.11235555
SRR17520069.11235556
SRR17520069.11239952
SRR17520069.11239953
SRR17520069.11239954
SRR17520069.11239955
SRR17520069.11239956
SRR17520069.11239957
SRR17520069.11239958
SRR17520069.11239959
SRR17520069.11239960
SRR17520069.11239961
SRR17520069.11239962
SRR17520069.11239963
SRR17520069.11239964
SRR17520069.11239965
SRR17520069.11239966
SRR17520069.11239967

SRR17520069.11239968
SRR17520069.11239969
SRR17520069.11239970
SRR17520069.11239971
SRR17520069.11239972
SRR17520069.11239973
SRR17520069.11239974
SRR17520069.11239975
SRR17520069.11239976
SRR17520069.11239977
SRR17520069.11239978
SRR17520069.11239979
SRR17520069.11239980
SRR17520069.11239981
SRR17520069.11239982
SRR17520069.11239983
SRR17520069.11239984
SRR17520069.11239985
SRR17520069.11239986
SRR17520069.11239987
SRR17520069.11239988
SRR17520069.11239989
SRR17520069.11239990
SRR17520069.11239991
SRR17520069.11239992
SRR17520069.11239993
SRR17520069.11239994
SRR17520069.11239995
SRR17520069.11239996
SRR17520069.11239997
SRR17520069.11239998
SRR17520069.11239999
SRR17520069.11240000
SRR17520069.11240001
SRR17520069.11240002
SRR17520069.11240003
SRR17520069.11240004
SRR17520069.11240005
SRR17520069.11240006
SRR17520069.11240007
SRR17520069.11240008
SRR17520069.11240009
SRR17520069.11240010
SRR17520069.11240011
SRR17520069.11240012
SRR17520069.11240013
SRR17520069.11240014

SRR17520069.11240015
SRR17520069.11240016
SRR17520069.11240017
SRR17520069.11240018
SRR17520069.11240019
SRR17520069.11240020
SRR17520069.11240021
SRR17520069.11240022
SRR17520069.11240023
SRR17520069.11240024
SRR17520069.11240025
SRR17520069.11240026
SRR17520069.11240027
SRR17520069.11240028
SRR17520069.11240029
SRR17520069.11240030
SRR17520069.11240031
SRR17520069.11240032
SRR17520069.11240033
SRR17520069.11240034
SRR17520069.11240035
SRR17520069.11240036
SRR17520069.11240037
SRR17520069.11240038
SRR17520069.11240039
SRR17520069.11240040
SRR17520069.11240041
SRR17520069.11240042
SRR17520069.11240043
SRR17520069.11240044
SRR17520069.11240045
SRR17520069.11240046
SRR17520069.11240047
SRR17520069.11240048
SRR17520069.11240049
SRR17520069.11240050
SRR17520069.11240051
SRR17520069.11240052
SRR17520069.11240053
SRR17520069.11240054
SRR17520069.11240055
SRR17520069.11240056
SRR17520069.11240057
SRR17520069.11240058
SRR17520069.11240059
SRR17520069.11240060
SRR17520069.11240061

SRR17520069.11240062
SRR17520069.11240063
SRR17520069.11240064
SRR17520069.11240065
SRR17520069.11240066
SRR17520069.11240067
SRR17520069.11240068
SRR17520069.11240069
SRR17520069.11240070
SRR17520069.11240071
SRR17520069.11240072
SRR17520069.11240073
SRR17520069.11240074
SRR17520069.11240075
SRR17520069.11240076
SRR17520069.11240077
SRR17520069.11240078
SRR17520069.11240079
SRR17520069.11240080
SRR17520069.11240081
SRR17520069.11240082
SRR17520069.11240083
SRR17520069.11240084
SRR17520069.11240085
SRR17520069.11240086
SRR17520069.11240087
SRR17520069.11240088
SRR17520069.11240089
SRR17520069.11240090
SRR17520069.11244255
SRR17520069.11256638
SRR17520069.11265768
SRR17520069.11272651
SRR17520069.11297583
SRR17520069.11300380
SRR17520069.11301931
SRR17520069.11301932
SRR17520069.11301933
SRR17520069.11301934
SRR17520069.11301935
SRR17520069.11301936
SRR17520069.11301937
SRR17520069.11301938
SRR17520069.11301939
SRR17520069.11301940
SRR17520069.11301941
SRR17520069.11301942

SRR17520069.11301943
SRR17520069.11301944
SRR17520069.11301948
SRR17520069.11301950
SRR17520069.11301951
SRR17520069.11301953
SRR17520069.11301954
SRR17520069.11301955
SRR17520069.11301956
SRR17520069.11301957
SRR17520069.11301958
SRR17520069.11301959
SRR17520069.11301960
SRR17520069.11301961
SRR17520069.11301962
SRR17520069.11301963
SRR17520069.11301964
SRR17520069.11301965
SRR17520069.11301966
SRR17520069.11301967
SRR17520069.11301968
SRR17520069.11301969
SRR17520069.11301970
SRR17520069.11301971
SRR17520069.11301972
SRR17520069.11301973
SRR17520069.11301974
SRR17520069.11301975
SRR17520069.11301976
SRR17520069.11301978
SRR17520069.11301979
SRR17520069.11301980
SRR17520069.11301981
SRR17520069.11301982
SRR17520069.11301983
SRR17520069.11301986
SRR17520069.11301987
SRR17520069.11301988
SRR17520069.11301989
SRR17520069.11301990
SRR17520069.11301992
SRR17520069.11301993
SRR17520069.11301994
SRR17520069.11301995
SRR17520069.11301996
SRR17520069.11301998
SRR17520069.11301999

SRR17520069.11302000
SRR17520069.11302001
SRR17520069.11302002
SRR17520069.11302003
SRR17520069.11302004
SRR17520069.11302005
SRR17520069.11302006
SRR17520069.11302007
SRR17520069.11302008
SRR17520069.11302009
SRR17520069.11302010
SRR17520069.11302011
SRR17520069.11302012
SRR17520069.11302013
SRR17520069.11302014
SRR17520069.11302015
SRR17520069.11302016
SRR17520069.11302017
SRR17520069.11302018
SRR17520069.11302019
SRR17520069.11302020
SRR17520069.11302021
SRR17520069.11302023
SRR17520069.11302024
SRR17520069.11302025
SRR17520069.11302026
SRR17520069.11302028
SRR17520069.11302029
SRR17520069.11302030
SRR17520069.11302031
SRR17520069.11302032
SRR17520069.11302033
SRR17520069.11302034
SRR17520069.11302035
SRR17520069.11302036
SRR17520069.11330729
SRR17520069.11341555
SRR17520069.11341605
SRR17520069.11353107
SRR17520069.11353108
SRR17520069.11353109
SRR17520069.11353110
SRR17520069.11353111
SRR17520069.11353112
SRR17520069.11353114
SRR17520069.11353115
SRR17520069.11353116

SRR17520069.11353117
SRR17520069.11353118
SRR17520069.11353119
SRR17520069.11353120
SRR17520069.11353121
SRR17520069.11353122
SRR17520069.11353123
SRR17520069.11353124
SRR17520069.11353125
SRR17520069.11353126
SRR17520069.11353128
SRR17520069.11353129
SRR17520069.11353130
SRR17520069.11353131
SRR17520069.11353132
SRR17520069.11353133
SRR17520069.11353134
SRR17520069.11353135
SRR17520069.11353136
SRR17520069.11353137
SRR17520069.11361978
SRR17520069.11361979
SRR17520069.11361980
SRR17520069.11361981
SRR17520069.11361982
SRR17520069.11361983
SRR17520069.11361985
SRR17520069.11361986
SRR17520069.11361987
SRR17520069.11361989
SRR17520069.11361991
SRR17520069.11361992
SRR17520069.11361993
SRR17520069.11361994
SRR17520069.11361995
SRR17520069.11361996
SRR17520069.11361997
SRR17520069.11361998
SRR17520069.11361999
SRR17520069.11362000
SRR17520069.11362001
SRR17520069.11362002
SRR17520069.11362003
SRR17520069.11362004
SRR17520069.11362005
SRR17520069.11362006
SRR17520069.11362007

SRR17520069.11362008
SRR17520069.11362009
SRR17520069.11362010
SRR17520069.11362011
SRR17520069.11362012
SRR17520069.11362013
SRR17520069.11362014
SRR17520069.11362015
SRR17520069.11362017
SRR17520069.11362018
SRR17520069.11362019
SRR17520069.11362020
SRR17520069.11362021
SRR17520069.11362022
SRR17520069.11362023
SRR17520069.11362024
SRR17520069.11362025
SRR17520069.11362026
SRR17520069.11362027
SRR17520069.11362028
SRR17520069.11362029
SRR17520069.11362030
SRR17520069.11362031
SRR17520069.11362032
SRR17520069.11362033
SRR17520069.11362034
SRR17520069.11362035
SRR17520069.11362036
SRR17520069.11362037
SRR17520069.11362038
SRR17520069.11362039
SRR17520069.11362040
SRR17520069.11362041
SRR17520069.11362042
SRR17520069.11362043
SRR17520069.11362044
SRR17520069.11362045
SRR17520069.11362046
SRR17520069.11362048
SRR17520069.11362049
SRR17520069.11362050
SRR17520069.11362051
SRR17520069.11362052
SRR17520069.11362053
SRR17520069.11362054
SRR17520069.11362055
SRR17520069.11362056

SRR17520069.11362057
SRR17520069.11362058
SRR17520069.11362059
SRR17520069.11362060
SRR17520069.11362061
SRR17520069.11362062
SRR17520069.11362063
SRR17520069.11362064
SRR17520069.11362065
SRR17520069.11362066
SRR17520069.11362067
SRR17520069.11362068
SRR17520069.11362069
SRR17520069.11362070
SRR17520069.11362071
SRR17520069.11362072
SRR17520069.11362073
SRR17520069.11379093
SRR17520069.11385951
SRR17520069.11385952
SRR17520069.11385953
SRR17520069.11385954
SRR17520069.11385955
SRR17520069.11385956
SRR17520069.11385957
SRR17520069.11385958
SRR17520069.11385959
SRR17520069.11385960
SRR17520069.11385961
SRR17520069.11385962
SRR17520069.11385963
SRR17520069.11385964
SRR17520069.11385965
SRR17520069.11385966
SRR17520069.11385967
SRR17520069.11385968
SRR17520069.11385969
SRR17520069.11385970
SRR17520069.11385971
SRR17520069.11385972
SRR17520069.11385973
SRR17520069.11385974
SRR17520069.11385975
SRR17520069.11457679
SRR17520069.11457680
SRR17520069.11457681
SRR17520069.11457682

SRR17520069.11457683
SRR17520069.11457684
SRR17520069.11457685
SRR17520069.11477891
SRR17520069.11511723
SRR17520069.11511724
SRR17520069.11511725
SRR17520069.11511726
SRR17520069.11511727
SRR17520069.11511728
SRR17520069.11511729
SRR17520069.11511730
SRR17520069.11511731
SRR17520069.11511732
SRR17520069.11511733
SRR17520069.11511734
SRR17520069.11511735
SRR17520069.11511736
SRR17520069.11511737
SRR17520069.11511740
SRR17520069.11511741
SRR17520069.11511743
SRR17520069.11511744
SRR17520069.11511745
SRR17520069.11511746
SRR17520069.11511747
SRR17520069.11511748
SRR17520069.11511749
SRR17520069.11511750
SRR17520069.11511751
SRR17520069.11511752
SRR17520069.11511753
SRR17520069.11511754
SRR17520069.11511755
SRR17520069.11511756
SRR17520069.11511757
SRR17520069.11511758
SRR17520069.11511759
SRR17520069.11511760
SRR17520069.11511761
SRR17520069.11513266
SRR17520069.11521361
SRR17520069.11521362
SRR17520069.11523296
SRR17520069.11523297
SRR17520069.11523298
SRR17520069.11523299

SRR17520069.11523300
SRR17520069.11526241
SRR17520069.11526242
SRR17520069.11526243
SRR17520069.11526244
SRR17520069.11526245
SRR17520069.11526246
SRR17520069.11526249
SRR17520069.11526250
SRR17520069.11526251
SRR17520069.11526252
SRR17520069.11526253
SRR17520069.11526254
SRR17520069.11526255
SRR17520069.11526256
SRR17520069.11526257
SRR17520069.11526258
SRR17520069.11526259
SRR17520069.11526260
SRR17520069.11526261
SRR17520069.11526262
SRR17520069.11526263
SRR17520069.11526266
SRR17520069.11526267
SRR17520069.11526268
SRR17520069.11526269
SRR17520069.11526270
SRR17520069.11526271
SRR17520069.11526272
SRR17520069.11526273
SRR17520069.11526274
SRR17520069.11526275
SRR17520069.11526276
SRR17520069.11526277
SRR17520069.11526278
SRR17520069.11526279
SRR17520069.11526280
SRR17520069.11526281
SRR17520069.11526282
SRR17520069.11526283
SRR17520069.11526284
SRR17520069.11526285
SRR17520069.11526286
SRR17520069.11526287
SRR17520069.11526288
SRR17520069.11526289
SRR17520069.11526290

SRR17520069.11526291
SRR17520069.11526292
SRR17520069.11526293
SRR17520069.11526294
SRR17520069.11526295
SRR17520069.11526296
SRR17520069.11526297
SRR17520069.11526298
SRR17520069.11526300
SRR17520069.11526301
SRR17520069.11526302
SRR17520069.11526303
SRR17520069.11526304
SRR17520069.11526305
SRR17520069.11526306
SRR17520069.11526307
SRR17520069.11526308
SRR17520069.11526309
SRR17520069.11526310
SRR17520069.11526311
SRR17520069.11526312
SRR17520069.11526313
SRR17520069.11526314
SRR17520069.11526315
SRR17520069.11526316
SRR17520069.11526317
SRR17520069.11526318
SRR17520069.11526319
SRR17520069.11526320
SRR17520069.11526321
SRR17520069.11526322
SRR17520069.11526323
SRR17520069.11526324
SRR17520069.11526325
SRR17520069.11526326
SRR17520069.11526329
SRR17520069.11526330
SRR17520069.11526331
SRR17520069.11526332
SRR17520069.11526333
SRR17520069.11526334
SRR17520069.11526335
SRR17520069.11526336
SRR17520069.11526337
SRR17520069.11526338
SRR17520069.11526339
SRR17520069.11526340

SRR17520069.11526341
SRR17520069.11526342
SRR17520069.11526343
SRR17520069.11526344
SRR17520069.11526345
SRR17520069.11526346
SRR17520069.11526347
SRR17520069.11526348
SRR17520069.11526349
SRR17520069.11526350
SRR17520069.11526351
SRR17520069.11526352
SRR17520069.11526354
SRR17520069.11526355
SRR17520069.11526356
SRR17520069.11526357
SRR17520069.11526358
SRR17520069.11526359
SRR17520069.11526360
SRR17520069.11526361
SRR17520069.11526363
SRR17520069.11526364
SRR17520069.11526365
SRR17520069.11526366
SRR17520069.11526367
SRR17520069.11526368
SRR17520069.11526369
SRR17520069.11526370
SRR17520069.11526371
SRR17520069.11526372
SRR17520069.11526373
SRR17520069.11526374
SRR17520069.11526375
SRR17520069.11526376
SRR17520069.11526377
SRR17520069.11526378
SRR17520069.11526379
SRR17520069.11526380
SRR17520069.11526381
SRR17520069.11526382
SRR17520069.11526383
SRR17520069.11526384
SRR17520069.11526385
SRR17520069.11526386
SRR17520069.11526387
SRR17520069.11526388
SRR17520069.11526389

SRR17520069.11526390
SRR17520069.11526391
SRR17520069.11526392
SRR17520069.11529853
SRR17520069.11529854
SRR17520069.11529855
SRR17520069.11529856
SRR17520069.11529857
SRR17520069.11529858
SRR17520069.11529859
SRR17520069.11529860
SRR17520069.11529861
SRR17520069.11529862
SRR17520069.11529865
SRR17520069.11529867
SRR17520069.11529868
SRR17520069.11529869
SRR17520069.11529870
SRR17520069.11529871
SRR17520069.11529872
SRR17520069.11529873
SRR17520069.11529875
SRR17520069.11529876
SRR17520069.11529877
SRR17520069.11529878
SRR17520069.11529879
SRR17520069.11529880
SRR17520069.11529881
SRR17520069.11529882
SRR17520069.11529883
SRR17520069.11529884
SRR17520069.11529885
SRR17520069.11529886
SRR17520069.11529887
SRR17520069.11529888
SRR17520069.11529889
SRR17520069.11529890
SRR17520069.11529891
SRR17520069.11529892
SRR17520069.11529893
SRR17520069.11529894
SRR17520069.11529895
SRR17520069.11529896
SRR17520069.11529897
SRR17520069.11529898
SRR17520069.11529900
SRR17520069.11529901

SRR17520069.11529902
SRR17520069.11529903
SRR17520069.11529904
SRR17520069.11529905
SRR17520069.11529906
SRR17520069.11529907
SRR17520069.11529908
SRR17520069.11529909
SRR17520069.11529910
SRR17520069.11529911
SRR17520069.11529913
SRR17520069.11529914
SRR17520069.11529915
SRR17520069.11529916
SRR17520069.11540158
SRR17520069.11540160
SRR17520069.11540161
SRR17520069.11540162
SRR17520069.11540163
SRR17520069.11540164
SRR17520069.11540165
SRR17520069.11540166
SRR17520069.11540167
SRR17520069.11540168
SRR17520069.11540169
SRR17520069.11540170
SRR17520069.11540171
SRR17520069.11540172
SRR17520069.11540173
SRR17520069.11540174
SRR17520069.11540175
SRR17520069.11540176
SRR17520069.11540177
SRR17520069.11540178
SRR17520069.11540179
SRR17520069.11540180
SRR17520069.11540181
SRR17520069.11540182
SRR17520069.11540183
SRR17520069.11540184
SRR17520069.11540185
SRR17520069.11540186
SRR17520069.11540187
SRR17520069.11540188
SRR17520069.11540189
SRR17520069.11540190
SRR17520069.11540191

SRR17520069.11540192
SRR17520069.11540193
SRR17520069.11540194
SRR17520069.11540195
SRR17520069.11540196
SRR17520069.11540197
SRR17520069.11540198
SRR17520069.11540199
SRR17520069.11540200
SRR17520069.11540201
SRR17520069.11540202
SRR17520069.11540203
SRR17520069.11540204
SRR17520069.11540205
SRR17520069.11540206
SRR17520069.11540207
SRR17520069.11540208
SRR17520069.11540209
SRR17520069.11540210
SRR17520069.11540211
SRR17520069.11540212
SRR17520069.11540213
SRR17520069.11540214
SRR17520069.11540215
SRR17520069.11540216
SRR17520069.11540217
SRR17520069.11540218
SRR17520069.11540219
SRR17520069.11540220
SRR17520069.11540221
SRR17520069.11540222
SRR17520069.11540223
SRR17520069.11540224
SRR17520069.11540225
SRR17520069.11540226
SRR17520069.11540227
SRR17520069.11540228
SRR17520069.11540229
SRR17520069.11540230
SRR17520069.11540231
SRR17520069.11540232
SRR17520069.11540233
SRR17520069.11540234
SRR17520069.11540235
SRR17520069.11540236
SRR17520069.11540237
SRR17520069.11540238

SRR17520069.11540239
SRR17520069.11540240
SRR17520069.11540241
SRR17520069.11540242
SRR17520069.11562935
SRR17520069.11562936
SRR17520069.11562937
SRR17520069.11562938
SRR17520069.11562939
SRR17520069.11562940
SRR17520069.11562941
SRR17520069.11562942
SRR17520069.11562943
SRR17520069.11562944
SRR17520069.11562945
SRR17520069.11562946
SRR17520069.11562947
SRR17520069.11562948
SRR17520069.11562949
SRR17520069.11562950
SRR17520069.11562951
SRR17520069.11562952
SRR17520069.11562953
SRR17520069.11562954
SRR17520069.11562955
SRR17520069.11562956
SRR17520069.11562957
SRR17520069.11562958
SRR17520069.11562959
SRR17520069.11562960
SRR17520069.11562961
SRR17520069.11562962
SRR17520069.11562963
SRR17520069.11562964
SRR17520069.11562965
SRR17520069.11562966
SRR17520069.11562967
SRR17520069.11562968
SRR17520069.11562969
SRR17520069.11562971
SRR17520069.11562972
SRR17520069.11562973
SRR17520069.11562974
SRR17520069.11562975
SRR17520069.11562976
SRR17520069.11562977
SRR17520069.11562978

SRR17520069.11562979
SRR17520069.11562980
SRR17520069.11562981
SRR17520069.11562982
SRR17520069.11562983
SRR17520069.11562984
SRR17520069.11562985
SRR17520069.11562986
SRR17520069.11562987
SRR17520069.11562988
SRR17520069.11562989
SRR17520069.11562990
SRR17520069.11562991
SRR17520069.11562992
SRR17520069.11562993
SRR17520069.11562994
SRR17520069.11562995
SRR17520069.11562996
SRR17520069.11562997
SRR17520069.11562998
SRR17520069.11562999
SRR17520069.11563001
SRR17520069.11563002
SRR17520069.11563003
SRR17520069.11563004
SRR17520069.11563005
SRR17520069.11563006
SRR17520069.11563007
SRR17520069.11563008
SRR17520069.11563009
SRR17520069.11563010
SRR17520069.11563011
SRR17520069.11563012
SRR17520069.11563013
SRR17520069.11563014
SRR17520069.11563015
SRR17520069.11563016
SRR17520069.11563017
SRR17520069.11563018
SRR17520069.11563019
SRR17520069.11563020
SRR17520069.11563021
SRR17520069.11563024
SRR17520069.11563025
SRR17520069.11563026
SRR17520069.11563027
SRR17520069.11563028

SRR17520069.11569408
SRR17520069.11585929
SRR17520069.11585930
SRR17520069.11585932
SRR17520069.11585933
SRR17520069.11585934
SRR17520069.11585935
SRR17520069.11585936
SRR17520069.11585937
SRR17520069.11585938
SRR17520069.11585939
SRR17520069.11585940
SRR17520069.11608150
SRR17520069.11633532
SRR17520069.11633533
SRR17520069.11633534
SRR17520069.11633535
SRR17520069.11633536
SRR17520069.11633537
SRR17520069.11633538
SRR17520069.11633539
SRR17520069.11633540
SRR17520069.11633541
SRR17520069.11633542
SRR17520069.11633543
SRR17520069.11633544
SRR17520069.11633545
SRR17520069.11633546
SRR17520069.11633547
SRR17520069.11633548
SRR17520069.11633549
SRR17520069.11633552
SRR17520069.11633553
SRR17520069.11633554
SRR17520069.11633555
SRR17520069.11633556
SRR17520069.11633557
SRR17520069.11633558
SRR17520069.11633559
SRR17520069.11633560
SRR17520069.11633561
SRR17520069.11633562
SRR17520069.11633563
SRR17520069.11633564
SRR17520069.11633565
SRR17520069.11633566
SRR17520069.11633567

SRR17520069.11633568
SRR17520069.11633569
SRR17520069.11633570
SRR17520069.11633571
SRR17520069.11633572
SRR17520069.11633573
SRR17520069.11633574
SRR17520069.11633575
SRR17520069.11633576
SRR17520069.11633577
SRR17520069.11633578
SRR17520069.11633579
SRR17520069.11633580
SRR17520069.11633581
SRR17520069.11633582
SRR17520069.11633583
SRR17520069.11633584
SRR17520069.11633585
SRR17520069.11633586
SRR17520069.11633587
SRR17520069.11633588
SRR17520069.11633589
SRR17520069.11633590
SRR17520069.11633591
SRR17520069.11633592
SRR17520069.11633593
SRR17520069.11633594
SRR17520069.11633595
SRR17520069.11633596
SRR17520069.11633597
SRR17520069.11633598
SRR17520069.11633599
SRR17520069.11633600
SRR17520069.11633601
SRR17520069.11633602
SRR17520069.11633603
SRR17520069.11633604
SRR17520069.11633605
SRR17520069.11633606
SRR17520069.11633607
SRR17520069.11633608
SRR17520069.11633609
SRR17520069.11633610
SRR17520069.11633611
SRR17520069.11633612
SRR17520069.11633613
SRR17520069.11633614

SRR17520069.11633615
SRR17520069.11633616
SRR17520069.11633617
SRR17520069.11633618
SRR17520069.11633619
SRR17520069.11633620
SRR17520069.11633621
SRR17520069.11633622
SRR17520069.11633623
SRR17520069.11633624
SRR17520069.11633625
SRR17520069.11633626
SRR17520069.11633627
SRR17520069.11633628
SRR17520069.11633630
SRR17520069.11633631
SRR17520069.11633632
SRR17520069.11633633
SRR17520069.11633634
SRR17520069.11633635
SRR17520069.11633636
SRR17520069.11633637
SRR17520069.11633638
SRR17520069.11633639
SRR17520069.11633640
SRR17520069.11633641
SRR17520069.11633642
SRR17520069.11633643
SRR17520069.11633644
SRR17520069.11633645
SRR17520069.11633646
SRR17520069.11633647
SRR17520069.11650990
SRR17520069.11651001
SRR17520069.11651002
SRR17520069.11661230
SRR17520069.11684451
SRR17520069.11684458
SRR17520069.11698817
SRR17520069.11698822
SRR17520069.11717562
SRR17520069.11717563
SRR17520069.11717564
SRR17520069.11717565
SRR17520069.11717566
SRR17520069.11717567
SRR17520069.11717568

SRR17520069.11717569
SRR17520069.11717570
SRR17520069.11717571
SRR17520069.11717572
SRR17520069.11717573
SRR17520069.11717574
SRR17520069.11717575
SRR17520069.11717576
SRR17520069.11717577
SRR17520069.11717578
SRR17520069.11717579
SRR17520069.11717580
SRR17520069.11717581
SRR17520069.11717582
SRR17520069.11717583
SRR17520069.11717584
SRR17520069.11717585
SRR17520069.11717586
SRR17520069.11744232
SRR17520069.11744233
SRR17520069.11744234
SRR17520069.11744235
SRR17520069.11744236
SRR17520069.11744237
SRR17520069.11744239
SRR17520069.11744240
SRR17520069.11744241
SRR17520069.11744242
SRR17520069.11744243
SRR17520069.11744244
SRR17520069.11744245
SRR17520069.11744248
SRR17520069.11744249
SRR17520069.11744250
SRR17520069.11744251
SRR17520069.11744252
SRR17520069.11744253
SRR17520069.11744254
SRR17520069.11744256
SRR17520069.11744258
SRR17520069.11744259
SRR17520069.11766063
SRR17520069.11766064
SRR17520069.11766065
SRR17520069.11766066
SRR17520069.11766067
SRR17520069.11766068

SRR17520069.11766069
SRR17520069.11766070
SRR17520069.11766071
SRR17520069.11766072
SRR17520069.11766073
SRR17520069.11766074
SRR17520069.11766075
SRR17520069.11766076
SRR17520069.11766078
SRR17520069.11766079
SRR17520069.11766080
SRR17520069.11766081
SRR17520069.11766082
SRR17520069.11766083
SRR17520069.11766084
SRR17520069.11766085
SRR17520069.11766086
SRR17520069.11766087
SRR17520069.11766088
SRR17520069.11766089
SRR17520069.11766090
SRR17520069.11766091
SRR17520069.11766092
SRR17520069.11766093
SRR17520069.11766094
SRR17520069.11766095
SRR17520069.11766096
SRR17520069.11766097
SRR17520069.11766098
SRR17520069.11766099
SRR17520069.11766100
SRR17520069.11766101
SRR17520069.11766102
SRR17520069.11766103
SRR17520069.11766104
SRR17520069.11766105
SRR17520069.11766106
SRR17520069.11766107
SRR17520069.11766108
SRR17520069.11766109
SRR17520069.11766110
SRR17520069.11766111
SRR17520069.11766112
SRR17520069.11766113
SRR17520069.11766114
SRR17520069.11766115
SRR17520069.11766116

SRR17520069.11766117
SRR17520069.11766118
SRR17520069.11766119
SRR17520069.11766120
SRR17520069.11766121
SRR17520069.11766122
SRR17520069.11766123
SRR17520069.11766124
SRR17520069.11766125
SRR17520069.11766126
SRR17520069.11766127
SRR17520069.11766128
SRR17520069.11766129
SRR17520069.11766130
SRR17520069.11766131
SRR17520069.11766132
SRR17520069.11766133
SRR17520069.11766134
SRR17520069.11766135
SRR17520069.11766137
SRR17520069.11766138
SRR17520069.11766139
SRR17520069.11766140
SRR17520069.11766141
SRR17520069.11766142
SRR17520069.11766143
SRR17520069.11766144
SRR17520069.11766145
SRR17520069.11766146
SRR17520069.11766147
SRR17520069.11766148
SRR17520069.11766149
SRR17520069.11766150
SRR17520069.11766151
SRR17520069.11766152
SRR17520069.11766153
SRR17520069.11766154
SRR17520069.11766155
SRR17520069.11766156
SRR17520069.11766157
SRR17520069.11766158
SRR17520069.11766159
SRR17520069.11766161
SRR17520069.11766162
SRR17520069.11766163
SRR17520069.11766164
SRR17520069.11766165

SRR17520069.11766166
SRR17520069.11775373
SRR17520069.11790586
SRR17520069.11790587
SRR17520069.11790588
SRR17520069.11790589
SRR17520069.11790590
SRR17520069.11790591
SRR17520069.11790592
SRR17520069.11790593
SRR17520069.11792377
SRR17520069.11832526
SRR17520069.11843503
SRR17520069.11843504
SRR17520069.11843505
SRR17520069.11843506
SRR17520069.11843507
SRR17520069.11843508
SRR17520069.11843509
SRR17520069.11843510
SRR17520069.11843511
SRR17520069.11843512
SRR17520069.11843513
SRR17520069.11843514
SRR17520069.11843515
SRR17520069.11843516
SRR17520069.11843517
SRR17520069.11843518
SRR17520069.11843519
SRR17520069.11843520
SRR17520069.11843521
SRR17520069.11843522
SRR17520069.11843523
SRR17520069.11843524
SRR17520069.11843525
SRR17520069.11843526
SRR17520069.11843527
SRR17520069.11843528
SRR17520069.11843529
SRR17520069.11843530
SRR17520069.11843531
SRR17520069.11843532
SRR17520069.11843533
SRR17520069.11843534
SRR17520069.11843535
SRR17520069.11843536
SRR17520069.11843538

SRR17520069.11843539
SRR17520069.11843540
SRR17520069.11843541
SRR17520069.11843542
SRR17520069.11843543
SRR17520069.11843545
SRR17520069.11843546
SRR17520069.11843547
SRR17520069.11843549
SRR17520069.11843550
SRR17520069.11843551
SRR17520069.11843552
SRR17520069.11843553
SRR17520069.11843555
SRR17520069.11843556
SRR17520069.11843557
SRR17520069.11843558
SRR17520069.11843560
SRR17520069.11843561
SRR17520069.11843563
SRR17520069.11843564
SRR17520069.11843565
SRR17520069.11843566
SRR17520069.11843567
SRR17520069.11843568
SRR17520069.11843569
SRR17520069.11843570
SRR17520069.11843571
SRR17520069.11843572
SRR17520069.11843573
SRR17520069.11843574
SRR17520069.11843575
SRR17520069.11843576
SRR17520069.11843577
SRR17520069.11843578
SRR17520069.11843579
SRR17520069.11843580
SRR17520069.11843581
SRR17520069.11843582
SRR17520069.11843583
SRR17520069.11843584
SRR17520069.11843585
SRR17520069.11843586
SRR17520069.11843587
SRR17520069.11843588
SRR17520069.11843589
SRR17520069.11843591

SRR17520069.11843592
SRR17520069.11843593
SRR17520069.11843594
SRR17520069.11843595
SRR17520069.11843596
SRR17520069.11843597
SRR17520069.11843599
SRR17520069.11843600
SRR17520069.11843601
SRR17520069.11843602
SRR17520069.11843604
SRR17520069.11843605
SRR17520069.11843606
SRR17520069.11843607
SRR17520069.11843608
SRR17520069.11843609
SRR17520069.11843610
SRR17520069.11843612
SRR17520069.11843613
SRR17520069.11843614
SRR17520069.11843615
SRR17520069.11843616
SRR17520069.11843617
SRR17520069.11843618
SRR17520069.11843619
SRR17520069.11843620
SRR17520069.11843621
SRR17520069.11843622
SRR17520069.11843623
SRR17520069.11843624
SRR17520069.11843625
SRR17520069.11843626
SRR17520069.11843627
SRR17520069.11843628
SRR17520069.11843629
SRR17520069.11843630
SRR17520069.11843631
SRR17520069.11843632
SRR17520069.11843633
SRR17520069.11843634
SRR17520069.11843635
SRR17520069.11843636
SRR17520069.11843637
SRR17520069.11843638
SRR17520069.11843639
SRR17520069.11843640
SRR17520069.11843641

SRR17520069.11843642
SRR17520069.11843643
SRR17520069.11843644
SRR17520069.11843645
SRR17520069.11843646
SRR17520069.11843647
SRR17520069.11843648
SRR17520069.11843649
SRR17520069.11843650
SRR17520069.11843651
SRR17520069.11843652
SRR17520069.11843653
SRR17520069.11843654
SRR17520069.11843655
SRR17520069.11843656
SRR17520069.11843657
SRR17520069.11843658
SRR17520069.11843659
SRR17520069.11843660
SRR17520069.11850356
SRR17520069.11850357
SRR17520069.11850358
SRR17520069.11850359
SRR17520069.11850360
SRR17520069.11850361
SRR17520069.11850362
SRR17520069.11850363
SRR17520069.11850364
SRR17520069.11850365
SRR17520069.11850366
SRR17520069.11850367
SRR17520069.11850368
SRR17520069.11850369
SRR17520069.11850370
SRR17520069.11850371
SRR17520069.11850372
SRR17520069.11882482
SRR17520069.11892871
SRR17520069.11892872
SRR17520069.11892873
SRR17520069.11892874
SRR17520069.11892875
SRR17520069.11892876
SRR17520069.11892877
SRR17520069.11892878
SRR17520069.11892879
SRR17520069.11892880

SRR17520069.11892881
SRR17520069.11892882
SRR17520069.11892883
SRR17520069.11892884
SRR17520069.11892885
SRR17520069.11892886
SRR17520069.11892887
SRR17520069.11892888
SRR17520069.11892889
SRR17520069.11892890
SRR17520069.11894789
SRR17520069.11894790
SRR17520069.11894791
SRR17520069.11894792
SRR17520069.11894793
SRR17520069.11894794
SRR17520069.11894796
SRR17520069.11894797
SRR17520069.11894798
SRR17520069.11894799
SRR17520069.11894800
SRR17520069.11894801
SRR17520069.11894802
SRR17520069.11894803
SRR17520069.11894804
SRR17520069.11894805
SRR17520069.11894806
SRR17520069.11894807
SRR17520069.11894808
SRR17520069.11894809
SRR17520069.11894810
SRR17520069.11894811
SRR17520069.11894812
SRR17520069.11894813
SRR17520069.11894814
SRR17520069.11894815
SRR17520069.11894816
SRR17520069.11894817
SRR17520069.11894818
SRR17520069.11894819
SRR17520069.11894820
SRR17520069.11894821
SRR17520069.11894822
SRR17520069.11894823
SRR17520069.11894824
SRR17520069.11894825
SRR17520069.11894826

SRR17520069.11894827
SRR17520069.11894828
SRR17520069.11894829
SRR17520069.11894830
SRR17520069.11894831
SRR17520069.11894832
SRR17520069.11894833
SRR17520069.11894834
SRR17520069.11894835
SRR17520069.11894836
SRR17520069.11894837
SRR17520069.11894839
SRR17520069.11894840
SRR17520069.11894841
SRR17520069.11894842
SRR17520069.11894843
SRR17520069.11894844
SRR17520069.11894845
SRR17520069.11894846
SRR17520069.11894847
SRR17520069.11894848
SRR17520069.11894849
SRR17520069.11894850
SRR17520069.11894851
SRR17520069.11894852
SRR17520069.11894853
SRR17520069.11894854
SRR17520069.11894855
SRR17520069.11894856
SRR17520069.11894857
SRR17520069.11894858
SRR17520069.11894859
SRR17520069.11894860
SRR17520069.11894861
SRR17520069.11894862
SRR17520069.11894863
SRR17520069.11894864
SRR17520069.11894865
SRR17520069.11894866
SRR17520069.11894867
SRR17520069.11894868
SRR17520069.11894869
SRR17520069.11894870
SRR17520069.11894871
SRR17520069.11894872
SRR17520069.11894873
SRR17520069.11894874

SRR17520069.11894875
SRR17520069.11894876
SRR17520069.11894877
SRR17520069.11894878
SRR17520069.11894879
SRR17520069.11894880
SRR17520069.11894881
SRR17520069.11894883
SRR17520069.11894884
SRR17520069.11894885
SRR17520069.11894886
SRR17520069.11894887
SRR17520069.11894888
SRR17520069.11894889
SRR17520069.11894890
SRR17520069.11894891
SRR17520069.11894892
SRR17520069.11894893
SRR17520069.11894894
SRR17520069.11894895
SRR17520069.11894896
SRR17520069.11894897
SRR17520069.11894898
SRR17520069.11894899
SRR17520069.11894900
SRR17520069.11894901
SRR17520069.11894902
SRR17520069.11894903
SRR17520069.11894904
SRR17520069.11894905
SRR17520069.11902352
SRR17520069.11902353
SRR17520069.11902354
SRR17520069.11902355
SRR17520069.11902356
SRR17520069.11902357
SRR17520069.11902358
SRR17520069.11902359
SRR17520069.11902360
SRR17520069.11902361
SRR17520069.11902362
SRR17520069.11902363
SRR17520069.11902364
SRR17520069.11902365
SRR17520069.11902366
SRR17520069.11902367
SRR17520069.11902368

SRR17520069.11902369
SRR17520069.11902370
SRR17520069.11902371
SRR17520069.11902372
SRR17520069.11902373
SRR17520069.11902374
SRR17520069.11902375
SRR17520069.11902376
SRR17520069.11902378
SRR17520069.11902379
SRR17520069.11902380
SRR17520069.11902381
SRR17520069.11902382
SRR17520069.11902383
SRR17520069.11902384
SRR17520069.11902386
SRR17520069.11902387
SRR17520069.11902388
SRR17520069.11902389
SRR17520069.11902390
SRR17520069.11902391
SRR17520069.11902392
SRR17520069.11902394
SRR17520069.11902395
SRR17520069.11902396
SRR17520069.11902397
SRR17520069.11902398
SRR17520069.11902399
SRR17520069.11902400
SRR17520069.11902401
SRR17520069.11902402
SRR17520069.11914008
SRR17520069.11914009
SRR17520069.11914010
SRR17520069.11914012
SRR17520069.11914013
SRR17520069.11914014
SRR17520069.11914015
SRR17520069.11914016
SRR17520069.11914017
SRR17520069.11914018
SRR17520069.11914019
SRR17520069.11914020
SRR17520069.11914022
SRR17520069.11914023
SRR17520069.11914024
SRR17520069.11914025

SRR17520069.11914027
SRR17520069.11914028
SRR17520069.11914029
SRR17520069.11914030
SRR17520069.11914031
SRR17520069.11914032
SRR17520069.11914033
SRR17520069.11914034
SRR17520069.11914036
SRR17520069.11914037
SRR17520069.11914038
SRR17520069.11914039
SRR17520069.11914040
SRR17520069.11914041
SRR17520069.11914042
SRR17520069.11914856
SRR17520069.11914857
SRR17520069.11914858
SRR17520069.11914859
SRR17520069.11914860
SRR17520069.11914861
SRR17520069.11914862
SRR17520069.11914863
SRR17520069.11914864
SRR17520069.11914865
SRR17520069.11914866
SRR17520069.11914867
SRR17520069.11914868
SRR17520069.11914869
SRR17520069.11914870
SRR17520069.11914871
SRR17520069.11914872
SRR17520069.11914873
SRR17520069.11920158
SRR17520069.11929419
SRR17520069.11929420
SRR17520069.11929421
SRR17520069.11929422
SRR17520069.11929423
SRR17520069.11929424
SRR17520069.11929425
SRR17520069.11929426
SRR17520069.11929427
SRR17520069.11929428
SRR17520069.11929429
SRR17520069.11929431
SRR17520069.11929432

SRR17520069.11929433
SRR17520069.11929434
SRR17520069.11929435
SRR17520069.11929436
SRR17520069.11929437
SRR17520069.11929439
SRR17520069.11929440
SRR17520069.11929441
SRR17520069.11929442
SRR17520069.11929443
SRR17520069.11929444
SRR17520069.11929445
SRR17520069.11929446
SRR17520069.11929447
SRR17520069.11929448
SRR17520069.11929449
SRR17520069.11929450
SRR17520069.11929451
SRR17520069.11929452
SRR17520069.11929453
SRR17520069.11929454
SRR17520069.11929455
SRR17520069.11929456
SRR17520069.11929457
SRR17520069.11929458
SRR17520069.11929462
SRR17520069.11929465
SRR17520069.11929466
SRR17520069.11929467
SRR17520069.11929468
SRR17520069.11929469
SRR17520069.11929470
SRR17520069.11929471
SRR17520069.11929472
SRR17520069.11929473
SRR17520069.11929474
SRR17520069.11929475
SRR17520069.11929476
SRR17520069.11929477
SRR17520069.11929478
SRR17520069.11929479
SRR17520069.11929480
SRR17520069.11929481
SRR17520069.11929482
SRR17520069.11929483
SRR17520069.11929484
SRR17520069.11929485

SRR17520069.11929486
SRR17520069.11929487
SRR17520069.11929488
SRR17520069.11929489
SRR17520069.11929490
SRR17520069.11929491
SRR17520069.11929492
SRR17520069.11929493
SRR17520069.11929494
SRR17520069.11929495
SRR17520069.11929496
SRR17520069.11929497
SRR17520069.11929498
SRR17520069.11929499
SRR17520069.11929500
SRR17520069.11929501
SRR17520069.11929502
SRR17520069.11929503
SRR17520069.11929504
SRR17520069.11929505
SRR17520069.11929506
SRR17520069.11929507
SRR17520069.11929508
SRR17520069.11929509
SRR17520069.11929510
SRR17520069.11929511
SRR17520069.11929512
SRR17520069.11929513
SRR17520069.11929514
SRR17520069.11929515
SRR17520069.11929516
SRR17520069.11929517
SRR17520069.11929518
SRR17520069.11929519
SRR17520069.11929520
SRR17520069.11929521
SRR17520069.11929522
SRR17520069.11929523
SRR17520069.11929524
SRR17520069.11929525
SRR17520069.11929526
SRR17520069.11929527
SRR17520069.11929528
SRR17520069.11929529
SRR17520069.11929530
SRR17520069.11929531
SRR17520069.11929532

SRR17520069.11929533
SRR17520069.11929534
SRR17520069.11929535
SRR17520069.11929536
SRR17520069.11929537
SRR17520069.11929538
SRR17520069.11929539
SRR17520069.11929540
SRR17520069.11929542
SRR17520069.11929543
SRR17520069.11929544
SRR17520069.11929545
SRR17520069.11929546
SRR17520069.11929547
SRR17520069.11929548
SRR17520069.11929549
SRR17520069.11929550
SRR17520069.11929551
SRR17520069.11929552
SRR17520069.11929553
SRR17520069.11929554
SRR17520069.11929555
SRR17520069.11935945
SRR17520069.11935946
SRR17520069.11935947
SRR17520069.11935948
SRR17520069.11935949
SRR17520069.11935950
SRR17520069.11935951
SRR17520069.11935952
SRR17520069.11935953
SRR17520069.11935954
SRR17520069.11935955
SRR17520069.11935956
SRR17520069.11935957
SRR17520069.11935958
SRR17520069.11935959
SRR17520069.11935960
SRR17520069.11935961
SRR17520069.11935962
SRR17520069.11935963
SRR17520069.11935964
SRR17520069.11935965
SRR17520069.11935966
SRR17520069.11935967
SRR17520069.11935968
SRR17520069.11935969

SRR17520069.11935970
SRR17520069.11935971
SRR17520069.11935972
SRR17520069.11935973
SRR17520069.11935974
SRR17520069.11935975
SRR17520069.11935976
SRR17520069.11935977
SRR17520069.11935978
SRR17520069.11935979
SRR17520069.11935980
SRR17520069.11935981
SRR17520069.11946384
SRR17520069.11959094
SRR17520069.11959095
SRR17520069.11959096
SRR17520069.11959097
SRR17520069.11959098
SRR17520069.11959099
SRR17520069.11959100
SRR17520069.11959101
SRR17520069.11959102
SRR17520069.11959103
SRR17520069.11959104
SRR17520069.11959105
SRR17520069.11959106
SRR17520069.11959107
SRR17520069.11959109
SRR17520069.11959110
SRR17520069.11959111
SRR17520069.11959112
SRR17520069.11959113
SRR17520069.11959114
SRR17520069.11974877
SRR17520069.11974878
SRR17520069.11974879
SRR17520069.11974882
SRR17520069.11974883
SRR17520069.11974884
SRR17520069.11974885
SRR17520069.11974886
SRR17520069.11974887
SRR17520069.11974891
SRR17520069.11974893
SRR17520069.11974895
SRR17520069.11974896
SRR17520069.11974897

SRR17520069.11974898
SRR17520069.11974899
SRR17520069.11974900
SRR17520069.11974901
SRR17520069.11974903
SRR17520069.11974904
SRR17520069.11974905
SRR17520069.11974907
SRR17520069.11974908
SRR17520069.11974910
SRR17520069.11974911
SRR17520069.11974912
SRR17520069.11974913
SRR17520069.11974915
SRR17520069.11974916
SRR17520069.11974917
SRR17520069.11974920
SRR17520069.11974921
SRR17520069.11974922
SRR17520069.11974923
SRR17520069.11974925
SRR17520069.11974927
SRR17520069.11974928
SRR17520069.11974931
SRR17520069.11974932
SRR17520069.11974934
SRR17520069.11983630
SRR17520069.12000421
SRR17520069.12000422
SRR17520069.12000423
SRR17520069.12000424
SRR17520069.12000425
SRR17520069.12000426
SRR17520069.12000427
SRR17520069.12000428
SRR17520069.12000429
SRR17520069.12000430
SRR17520069.12000431
SRR17520069.12000432
SRR17520069.12000433
SRR17520069.12000434
SRR17520069.12000435
SRR17520069.12000436
SRR17520069.12000437
SRR17520069.12000438
SRR17520069.12000439
SRR17520069.12000440

SRR17520069.12000441
SRR17520069.12000442
SRR17520069.12000443
SRR17520069.12000444
SRR17520069.12000445
SRR17520069.12000446
SRR17520069.12000447
SRR17520069.12000448
SRR17520069.12000449
SRR17520069.12000450
SRR17520069.12000451
SRR17520069.12000453
SRR17520069.12000454
SRR17520069.12000455
SRR17520069.12000456
SRR17520069.12000457
SRR17520069.12000458
SRR17520069.12000459
SRR17520069.12000460
SRR17520069.12000461
SRR17520069.12000462
SRR17520069.12000463
SRR17520069.12000464
SRR17520069.12000465
SRR17520069.12000466
SRR17520069.12000467
SRR17520069.12000468
SRR17520069.12000469
SRR17520069.12000470
SRR17520069.12000471
SRR17520069.12000472
SRR17520069.12000473
SRR17520069.12000474
SRR17520069.12000475
SRR17520069.12000476
SRR17520069.12000477
SRR17520069.12000478
SRR17520069.12000479
SRR17520069.12000480
SRR17520069.12000481
SRR17520069.12000482
SRR17520069.12000483
SRR17520069.12000484
SRR17520069.12000485
SRR17520069.12000486
SRR17520069.12000487
SRR17520069.12000488

SRR17520069.12000489
SRR17520069.12000490
SRR17520069.12000491
SRR17520069.12000492
SRR17520069.12000494
SRR17520069.12000495
SRR17520069.12000496
SRR17520069.12000497
SRR17520069.12000498
SRR17520069.12000499
SRR17520069.12000500
SRR17520069.12000501
SRR17520069.12000502
SRR17520069.12000503
SRR17520069.12000504
SRR17520069.12000505
SRR17520069.12000506
SRR17520069.12000507
SRR17520069.12000508
SRR17520069.12000509
SRR17520069.12000510
SRR17520069.12000511
SRR17520069.12000512
SRR17520069.12000513
SRR17520069.12000514
SRR17520069.12000515
SRR17520069.12000516
SRR17520069.12000517
SRR17520069.12000518
SRR17520069.12000519
SRR17520069.12000520
SRR17520069.12000521
SRR17520069.12000522
SRR17520069.12000523
SRR17520069.12000524
SRR17520069.12000525
SRR17520069.12000526
SRR17520069.12000527
SRR17520069.12000528
SRR17520069.12000529
SRR17520069.12000530
SRR17520069.12000531
SRR17520069.12000532
SRR17520069.12000533
SRR17520069.12000534
SRR17520069.12000535
SRR17520069.12008629

SRR17520069.12008641
SRR17520069.12042485
SRR17520069.12042521
SRR17520069.12043667
SRR17520069.12050269
SRR17520069.12050270
SRR17520069.12050271
SRR17520069.12050272
SRR17520069.12050273
SRR17520069.12050274
SRR17520069.12050275
SRR17520069.12050276
SRR17520069.12050277
SRR17520069.12050278
SRR17520069.12050279
SRR17520069.12050280
SRR17520069.12050281
SRR17520069.12050282
SRR17520069.12050283
SRR17520069.12050284
SRR17520069.12050285
SRR17520069.12050286
SRR17520069.12050287
SRR17520069.12050288
SRR17520069.12050289
SRR17520069.12050290
SRR17520069.12050291
SRR17520069.12050292
SRR17520069.12050293
SRR17520069.12050294
SRR17520069.12050295
SRR17520069.12050296
SRR17520069.12050297
SRR17520069.12050298
SRR17520069.12050299
SRR17520069.12050300
SRR17520069.12050301
SRR17520069.12050302
SRR17520069.12050303
SRR17520069.12050304
SRR17520069.12050305
SRR17520069.12050306
SRR17520069.12050307
SRR17520069.12050308
SRR17520069.12050309
SRR17520069.12050311
SRR17520069.12050312

SRR17520069.12050313
SRR17520069.12050314
SRR17520069.12050315
SRR17520069.12050316
SRR17520069.12050317
SRR17520069.12050318
SRR17520069.12050319
SRR17520069.12050320
SRR17520069.12050321
SRR17520069.12050322
SRR17520069.12050323
SRR17520069.12050324
SRR17520069.12050325
SRR17520069.12050326
SRR17520069.12050327
SRR17520069.12050328
SRR17520069.12050329
SRR17520069.12050330
SRR17520069.12050331
SRR17520069.12050332
SRR17520069.12050333
SRR17520069.12050334
SRR17520069.12050335
SRR17520069.12050336
SRR17520069.12050337
SRR17520069.12050338
SRR17520069.12050339
SRR17520069.12050340
SRR17520069.12050341
SRR17520069.12050342
SRR17520069.12050343
SRR17520069.12050344
SRR17520069.12050345
SRR17520069.12050346
SRR17520069.12050347
SRR17520069.12050348
SRR17520069.12050349
SRR17520069.12050350
SRR17520069.12050351
SRR17520069.12050352
SRR17520069.12050353
SRR17520069.12050354
SRR17520069.12050355
SRR17520069.12050356
SRR17520069.12071720
SRR17520069.12071721
SRR17520069.12071722

SRR17520069.12071723
SRR17520069.12071724
SRR17520069.12071725
SRR17520069.12071726
SRR17520069.12071727
SRR17520069.12071728
SRR17520069.12071729
SRR17520069.12071730
SRR17520069.12071731
SRR17520069.12071732
SRR17520069.12071733
SRR17520069.12071734
SRR17520069.12071735
SRR17520069.12071736
SRR17520069.12071737
SRR17520069.12081902
SRR17520069.12081903
SRR17520069.12081904
SRR17520069.12081905
SRR17520069.12081906
SRR17520069.12081907
SRR17520069.12081908
SRR17520069.12081909
SRR17520069.12081910
SRR17520069.12081911
SRR17520069.12081912
SRR17520069.12081913
SRR17520069.12081914
SRR17520069.12081915
SRR17520069.12081916
SRR17520069.12081917
SRR17520069.12081918
SRR17520069.12081919
SRR17520069.12081920
SRR17520069.12081921
SRR17520069.12081922
SRR17520069.12081923
SRR17520069.12081924
SRR17520069.12081925
SRR17520069.12081926
SRR17520069.12081927
SRR17520069.12081928
SRR17520069.12081929
SRR17520069.12081930
SRR17520069.12081931
SRR17520069.12081933
SRR17520069.12081934

SRR17520069.12081935
SRR17520069.12081936
SRR17520069.12081937
SRR17520069.12081938
SRR17520069.12081939
SRR17520069.12081940
SRR17520069.12081941
SRR17520069.12081942
SRR17520069.12081943
SRR17520069.12081944
SRR17520069.12081945
SRR17520069.12081946
SRR17520069.12081947
SRR17520069.12081948
SRR17520069.12081949
SRR17520069.12081950
SRR17520069.12081951
SRR17520069.12081952
SRR17520069.12081953
SRR17520069.12081954
SRR17520069.12081955
SRR17520069.12081956
SRR17520069.12081957
SRR17520069.12081958
SRR17520069.12081959
SRR17520069.12081960
SRR17520069.12081961
SRR17520069.12081962
SRR17520069.12081963
SRR17520069.12081964
SRR17520069.12081965
SRR17520069.12081966
SRR17520069.12081967
SRR17520069.12081968
SRR17520069.12081969
SRR17520069.12081970
SRR17520069.12081971
SRR17520069.12081972
SRR17520069.12081973
SRR17520069.12081974
SRR17520069.12081975
SRR17520069.12081976
SRR17520069.12081977
SRR17520069.12081978
SRR17520069.12081979
SRR17520069.12081980
SRR17520069.12081981

SRR17520069.12081982
SRR17520069.12081983
SRR17520069.12081984
SRR17520069.12081985
SRR17520069.12081986
SRR17520069.12081987
SRR17520069.12081988
SRR17520069.12081989
SRR17520069.12081990
SRR17520069.12081991
SRR17520069.12081992
SRR17520069.12081993
SRR17520069.12081994
SRR17520069.12081995
SRR17520069.12081996
SRR17520069.12081997
SRR17520069.12081998
SRR17520069.12081999
SRR17520069.12082000
SRR17520069.12082001
SRR17520069.12082002
SRR17520069.12082003
SRR17520069.12082004
SRR17520069.12082005
SRR17520069.12082006
SRR17520069.12083451
SRR17520069.12083452
SRR17520069.12083453
SRR17520069.12083454
SRR17520069.12083455
SRR17520069.12083456
SRR17520069.12083458
SRR17520069.12083459
SRR17520069.12083460
SRR17520069.12083461
SRR17520069.12083462
SRR17520069.12083463
SRR17520069.12083464
SRR17520069.12083465
SRR17520069.12083466
SRR17520069.12083467
SRR17520069.12083468
SRR17520069.12083469
SRR17520069.12083470
SRR17520069.12083471
SRR17520069.12083472
SRR17520069.12083473

SRR17520069.12083474
SRR17520069.12083475
SRR17520069.12083478
SRR17520069.12083479
SRR17520069.12083480
SRR17520069.12083481
SRR17520069.12083482
SRR17520069.12083483
SRR17520069.12083484
SRR17520069.12083485
SRR17520069.12083486
SRR17520069.12083487
SRR17520069.12083488
SRR17520069.12083489
SRR17520069.12083490
SRR17520069.12083491
SRR17520069.12083492
SRR17520069.12083493
SRR17520069.12083494
SRR17520069.12083495
SRR17520069.12083496
SRR17520069.12083497
SRR17520069.12083498
SRR17520069.12083499
SRR17520069.12083500
SRR17520069.12083501
SRR17520069.12083502
SRR17520069.12083503
SRR17520069.12083504
SRR17520069.12083505
SRR17520069.12083506
SRR17520069.12083507
SRR17520069.12083509
SRR17520069.12083510
SRR17520069.12083511
SRR17520069.12083512
SRR17520069.12083513
SRR17520069.12083514
SRR17520069.12083515
SRR17520069.12083516
SRR17520069.12083517
SRR17520069.12083518
SRR17520069.12083519
SRR17520069.12083520
SRR17520069.12083521
SRR17520069.12083522
SRR17520069.12083523

SRR17520069.12083524
SRR17520069.12083525
SRR17520069.12083526
SRR17520069.12083528
SRR17520069.12083529
SRR17520069.12083530
SRR17520069.12083531
SRR17520069.12083532
SRR17520069.12083533
SRR17520069.12083534
SRR17520069.12083535
SRR17520069.12083536
SRR17520069.12083537
SRR17520069.12083538
SRR17520069.12083539
SRR17520069.12083540
SRR17520069.12083541
SRR17520069.12089038
SRR17520069.12091801
SRR17520069.12091802
SRR17520069.12091803
SRR17520069.12091804
SRR17520069.12091805
SRR17520069.12091808
SRR17520069.12091809
SRR17520069.12091810
SRR17520069.12091811
SRR17520069.12091812
SRR17520069.12091813
SRR17520069.12091814
SRR17520069.12091815
SRR17520069.12091816
SRR17520069.12091817
SRR17520069.12091818
SRR17520069.12091819
SRR17520069.12091820
SRR17520069.12091821
SRR17520069.12091822
SRR17520069.12091823
SRR17520069.12091825
SRR17520069.12091826
SRR17520069.12091827
SRR17520069.12091828
SRR17520069.12091829
SRR17520069.12091831
SRR17520069.12091832
SRR17520069.12091833

SRR17520069.12091834
SRR17520069.12091835
SRR17520069.12091836
SRR17520069.12091837
SRR17520069.12091838
SRR17520069.12091839
SRR17520069.12091840
SRR17520069.12091841
SRR17520069.12091842
SRR17520069.12091843
SRR17520069.12091844
SRR17520069.12091845
SRR17520069.12091847
SRR17520069.12091848
SRR17520069.12091849
SRR17520069.12091850
SRR17520069.12091851
SRR17520069.12091852
SRR17520069.12091853
SRR17520069.12091854
SRR17520069.12091855
SRR17520069.12091856
SRR17520069.12091857
SRR17520069.12091858
SRR17520069.12091859
SRR17520069.12091860
SRR17520069.12091861
SRR17520069.12091862
SRR17520069.12091864
SRR17520069.12091865
SRR17520069.12091866
SRR17520069.12091868
SRR17520069.12091869
SRR17520069.12091870
SRR17520069.12091871
SRR17520069.12091872
SRR17520069.12091873
SRR17520069.12091874
SRR17520069.12091875
SRR17520069.12091876
SRR17520069.12091877
SRR17520069.12091878
SRR17520069.12091879
SRR17520069.12091880
SRR17520069.12110869
SRR17520069.12110871
SRR17520069.12110872

SRR17520069.12110873
SRR17520069.12110874
SRR17520069.12110875
SRR17520069.12117963
SRR17520069.12117964
SRR17520069.12133676
SRR17520069.12133677
SRR17520069.12142952
SRR17520069.12157144
SRR17520069.12168966
SRR17520069.12168968
SRR17520069.12168969
SRR17520069.12168970
SRR17520069.12168972
SRR17520069.12168973
SRR17520069.12168974
SRR17520069.12168975
SRR17520069.12168976
SRR17520069.12168977
SRR17520069.12168978
SRR17520069.12168980
SRR17520069.12168981
SRR17520069.12168982
SRR17520069.12168983
SRR17520069.12168984
SRR17520069.12168986
SRR17520069.12168987
SRR17520069.12168988
SRR17520069.12168989
SRR17520069.12168990
SRR17520069.12168991
SRR17520069.12168992
SRR17520069.12173615
SRR17520069.12200867
SRR17520069.12200868
SRR17520069.12200869
SRR17520069.12200870
SRR17520069.12200871
SRR17520069.12200874
SRR17520069.12200875
SRR17520069.12200876
SRR17520069.12200877
SRR17520069.12200878
SRR17520069.12200879
SRR17520069.12200880
SRR17520069.12200881
SRR17520069.12200882

SRR17520069.12200883
SRR17520069.12200884
SRR17520069.12200885
SRR17520069.12200886
SRR17520069.12200887
SRR17520069.12200889
SRR17520069.12201824
SRR17520069.12237681
SRR17520069.12253718
SRR17520069.12262321
SRR17520069.12265506
SRR17520069.12269818
SRR17520069.12279996
SRR17520069.12279997
SRR17520069.12279998
SRR17520069.12279999
SRR17520069.12280000
SRR17520069.12280001
SRR17520069.12280002
SRR17520069.12280003
SRR17520069.12280005
SRR17520069.12280006
SRR17520069.12280007
SRR17520069.12280008
SRR17520069.12280009
SRR17520069.12280010
SRR17520069.12280011
SRR17520069.12280012
SRR17520069.12280013
SRR17520069.12280014
SRR17520069.12280017
SRR17520069.12280018
SRR17520069.12280019
SRR17520069.12280020
SRR17520069.12280021
SRR17520069.12280022
SRR17520069.12280023
SRR17520069.12280024
SRR17520069.12280025
SRR17520069.12280026
SRR17520069.12280027
SRR17520069.12280028
SRR17520069.12280029
SRR17520069.12280030
SRR17520069.12280031
SRR17520069.12280032
SRR17520069.12280033

SRR17520069.12280034
SRR17520069.12280035
SRR17520069.12280036
SRR17520069.12280037
SRR17520069.12280038
SRR17520069.12280039
SRR17520069.12280040
SRR17520069.12280041
SRR17520069.12280042
SRR17520069.12280043
SRR17520069.12280053
SRR17520069.12280055
SRR17520069.12283321
SRR17520069.12300326
SRR17520069.12316385
SRR17520069.12331052
SRR17520069.12331053
SRR17520069.12331054
SRR17520069.12331055
SRR17520069.12331056
SRR17520069.12331057
SRR17520069.12331058
SRR17520069.12331059
SRR17520069.12331060
SRR17520069.12331061
SRR17520069.12331062
SRR17520069.12331063
SRR17520069.12331064
SRR17520069.12355350
SRR17520069.12355351
SRR17520069.12355352
SRR17520069.12355353
SRR17520069.12355354
SRR17520069.12355355
SRR17520069.12355356
SRR17520069.12355357
SRR17520069.12355358
SRR17520069.12355359
SRR17520069.12355360
SRR17520069.12355361
SRR17520069.12355362
SRR17520069.12355364
SRR17520069.12355365
SRR17520069.12355366
SRR17520069.12355367
SRR17520069.12355368
SRR17520069.12355369

SRR17520069.12355370
SRR17520069.12355371
SRR17520069.12355372
SRR17520069.12355373
SRR17520069.12355374
SRR17520069.12355375
SRR17520069.12355376
SRR17520069.12355377
SRR17520069.12355378
SRR17520069.12355379
SRR17520069.12355380
SRR17520069.12355381
SRR17520069.12355382
SRR17520069.12355383
SRR17520069.12355384
SRR17520069.12355385
SRR17520069.12355386
SRR17520069.12355387
SRR17520069.12355388
SRR17520069.12355389
SRR17520069.12355390
SRR17520069.12355391
SRR17520069.12355392
SRR17520069.12355393
SRR17520069.12355394
SRR17520069.12355395
SRR17520069.12355396
SRR17520069.12355398
SRR17520069.12355399
SRR17520069.12355400
SRR17520069.12355401
SRR17520069.12355402
SRR17520069.12355403
SRR17520069.12366004
SRR17520069.12367725
SRR17520069.12367726
SRR17520069.12367727
SRR17520069.12367728
SRR17520069.12367729
SRR17520069.12367730
SRR17520069.12367731
SRR17520069.12367732
SRR17520069.12386349
SRR17520069.12386350
SRR17520069.12386351
SRR17520069.12386352
SRR17520069.12386353

SRR17520069.12386354
SRR17520069.12386355
SRR17520069.12386356
SRR17520069.12386357
SRR17520069.12386358
SRR17520069.12386359
SRR17520069.12386360
SRR17520069.12386361
SRR17520069.12386362
SRR17520069.12386363
SRR17520069.12386364
SRR17520069.12386365
SRR17520069.12386366
SRR17520069.12386367
SRR17520069.12386368
SRR17520069.12386369
SRR17520069.12386370
SRR17520069.12386371
SRR17520069.12386372
SRR17520069.12386373
SRR17520069.12386374
SRR17520069.12386375
SRR17520069.12386376
SRR17520069.12386377
SRR17520069.12386378
SRR17520069.12386379
SRR17520069.12386380
SRR17520069.12393277
SRR17520069.12395332
SRR17520069.12401091
SRR17520069.12401092
SRR17520069.12401093
SRR17520069.12401094
SRR17520069.12401095
SRR17520069.12401096
SRR17520069.12401097
SRR17520069.12401098
SRR17520069.12401099
SRR17520069.12401100
SRR17520069.12401101
SRR17520069.12401102
SRR17520069.12401103
SRR17520069.12401104
SRR17520069.12401105
SRR17520069.12401106
SRR17520069.12401107
SRR17520069.12401108

SRR17520069.12401109
SRR17520069.12401110
SRR17520069.12401111
SRR17520069.12401112
SRR17520069.12401113
SRR17520069.12401114
SRR17520069.12401115
SRR17520069.12401116
SRR17520069.12401117
SRR17520069.12401118
SRR17520069.12410926
SRR17520069.12410927
SRR17520069.12410928
SRR17520069.12410930
SRR17520069.12410931
SRR17520069.12410932
SRR17520069.12410933
SRR17520069.12410934
SRR17520069.12410935
SRR17520069.12410936
SRR17520069.12410937
SRR17520069.12410938
SRR17520069.12410939
SRR17520069.12410940
SRR17520069.12410941
SRR17520069.12410942
SRR17520069.12410943
SRR17520069.12410944
SRR17520069.12410945
SRR17520069.12410946
SRR17520069.12410947
SRR17520069.12410948
SRR17520069.12410949
SRR17520069.12410950
SRR17520069.12410951
SRR17520069.12410952
SRR17520069.12410953
SRR17520069.12410954
SRR17520069.12410955
SRR17520069.12410956
SRR17520069.12410957
SRR17520069.12410958
SRR17520069.12410959
SRR17520069.12410960
SRR17520069.12410961
SRR17520069.12410962
SRR17520069.12410963

SRR17520069.12410964
SRR17520069.12410965
SRR17520069.12410966
SRR17520069.12410967
SRR17520069.12410968
SRR17520069.12410969
SRR17520069.12410970
SRR17520069.12410971
SRR17520069.12410972
SRR17520069.12410973
SRR17520069.12410974
SRR17520069.12410975
SRR17520069.12410976
SRR17520069.12410977
SRR17520069.12410978
SRR17520069.12410979
SRR17520069.12410980
SRR17520069.12410981
SRR17520069.12410982
SRR17520069.12410983
SRR17520069.12410984
SRR17520069.12410985
SRR17520069.12410986
SRR17520069.12410987
SRR17520069.12410988
SRR17520069.12410989
SRR17520069.12410990
SRR17520069.12410991
SRR17520069.12410992
SRR17520069.12410993
SRR17520069.12410994
SRR17520069.12410995
SRR17520069.12410996
SRR17520069.12410997
SRR17520069.12410998
SRR17520069.12410999
SRR17520069.12411000
SRR17520069.12411001
SRR17520069.12411002
SRR17520069.12411003
SRR17520069.12411004
SRR17520069.12411006
SRR17520069.12411007
SRR17520069.12411008
SRR17520069.12411009
SRR17520069.12411010
SRR17520069.12411011

SRR17520069.12411012
SRR17520069.12411014
SRR17520069.12411015
SRR17520069.12411016
SRR17520069.12411017
SRR17520069.12411018
SRR17520069.12411019
SRR17520069.12411020
SRR17520069.12411021
SRR17520069.12411022
SRR17520069.12411023
SRR17520069.12411024
SRR17520069.12411025
SRR17520069.12411026
SRR17520069.12411027
SRR17520069.12411028
SRR17520069.12411029
SRR17520069.12411030
SRR17520069.12411031
SRR17520069.12411032
SRR17520069.12411033
SRR17520069.12411034
SRR17520069.12411035
SRR17520069.12411036
SRR17520069.12411037
SRR17520069.12411038
SRR17520069.12411039
SRR17520069.12411040
SRR17520069.12411041
SRR17520069.12411042
SRR17520069.12411043
SRR17520069.12411044
SRR17520069.12411045
SRR17520069.12411046
SRR17520069.12411047
SRR17520069.12411048
SRR17520069.12411049
SRR17520069.12411050
SRR17520069.12411051
SRR17520069.12411052
SRR17520069.12411053
SRR17520069.12411054
SRR17520069.12411055
SRR17520069.12411056
SRR17520069.12411057
SRR17520069.12411058
SRR17520069.12411059

SRR17520069.12411060
SRR17520069.12411061
SRR17520069.12411062
SRR17520069.12411063
SRR17520069.12411064
SRR17520069.12411065
SRR17520069.12411067
SRR17520069.12411068
SRR17520069.12411069
SRR17520069.12411070
SRR17520069.12411071
SRR17520069.12411072
SRR17520069.12411073
SRR17520069.12411074
SRR17520069.12411075
SRR17520069.12411076
SRR17520069.12411077
SRR17520069.12411078
SRR17520069.12411079
SRR17520069.12411080
SRR17520069.12411081
SRR17520069.12411082
SRR17520069.12411083
SRR17520069.12411084
SRR17520069.12411085
SRR17520069.12411087
SRR17520069.12411088
SRR17520069.12425050
SRR17520069.12453992
SRR17520069.12460184
SRR17520069.12460185
SRR17520069.12460186
SRR17520069.12460187
SRR17520069.12460188
SRR17520069.12460189
SRR17520069.12460190
SRR17520069.12460191
SRR17520069.12460192
SRR17520069.12460193
SRR17520069.12469810
SRR17520069.12469811
SRR17520069.12469812
SRR17520069.12469813
SRR17520069.12469814
SRR17520069.12469815
SRR17520069.12469816
SRR17520069.12469817

SRR17520069.12469818
SRR17520069.12469819
SRR17520069.12469820
SRR17520069.12469821
SRR17520069.12469822
SRR17520069.12469823
SRR17520069.12469824
SRR17520069.12469825
SRR17520069.12469826
SRR17520069.12469827
SRR17520069.12469828
SRR17520069.12469829
SRR17520069.12469830
SRR17520069.12469831
SRR17520069.12469832
SRR17520069.12469833
SRR17520069.12469834
SRR17520069.12469835
SRR17520069.12469836
SRR17520069.12469837
SRR17520069.12469838
SRR17520069.12469839
SRR17520069.12469840
SRR17520069.12469841
SRR17520069.12469842
SRR17520069.12469843
SRR17520069.12469844
SRR17520069.12469845
SRR17520069.12469846
SRR17520069.12469847
SRR17520069.12469848
SRR17520069.12469849
SRR17520069.12469850
SRR17520069.12469851
SRR17520069.12469852
SRR17520069.12469853
SRR17520069.12469854
SRR17520069.12469855
SRR17520069.12469857
SRR17520069.12469858
SRR17520069.12469859
SRR17520069.12469860
SRR17520069.12469861
SRR17520069.12469862
SRR17520069.12469863
SRR17520069.12469864
SRR17520069.12469865

SRR17520069.12469866
SRR17520069.12469867
SRR17520069.12469868
SRR17520069.12469869
SRR17520069.12469870
SRR17520069.12469871
SRR17520069.12469872
SRR17520069.12469873
SRR17520069.12469874
SRR17520069.12469875
SRR17520069.12469876
SRR17520069.12469877
SRR17520069.12469878
SRR17520069.12469879
SRR17520069.12469880
SRR17520069.12469881
SRR17520069.12469882
SRR17520069.12469883
SRR17520069.12469884
SRR17520069.12469885
SRR17520069.12469886
SRR17520069.12469887
SRR17520069.12469888
SRR17520069.12469889
SRR17520069.12469890
SRR17520069.12469891
SRR17520069.12469892
SRR17520069.12469893
SRR17520069.12469894
SRR17520069.12469895
SRR17520069.12469896
SRR17520069.12469897
SRR17520069.12469898
SRR17520069.12469899
SRR17520069.12469900
SRR17520069.12469901
SRR17520069.12469902
SRR17520069.12469903
SRR17520069.12469904
SRR17520069.12469905
SRR17520069.12469906
SRR17520069.12469907
SRR17520069.12469908
SRR17520069.12469909
SRR17520069.12469910
SRR17520069.12469911
SRR17520069.12469912

SRR17520069.12469913
SRR17520069.12469914
SRR17520069.12469915
SRR17520069.12469916
SRR17520069.12469917
SRR17520069.12469918
SRR17520069.12469919
SRR17520069.12469920
SRR17520069.12469921
SRR17520069.12469922
SRR17520069.12469923
SRR17520069.12469924
SRR17520069.12469925
SRR17520069.12469926
SRR17520069.12469927
SRR17520069.12469928
SRR17520069.12469929
SRR17520069.12469930
SRR17520069.12469931
SRR17520069.12469932
SRR17520069.12469933
SRR17520069.12469934
SRR17520069.12469935
SRR17520069.12469937
SRR17520069.12469938
SRR17520069.12469939
SRR17520069.12469940
SRR17520069.12469941
SRR17520069.12469942
SRR17520069.12469943
SRR17520069.12469944
SRR17520069.12469945
SRR17520069.12469947
SRR17520069.12469948
SRR17520069.12469949
SRR17520069.12469950
SRR17520069.12469951
SRR17520069.12469952
SRR17520069.12469953
SRR17520069.12469954
SRR17520069.12469955
SRR17520069.12469956
SRR17520069.12469957
SRR17520069.12469958
SRR17520069.12469959
SRR17520069.12469960
SRR17520069.12469961

SRR17520069.12469962
SRR17520069.12469963
SRR17520069.12469964
SRR17520069.12469967
SRR17520069.12469968
SRR17520069.12508218
SRR17520069.12508219
SRR17520069.12508220
SRR17520069.12508221
SRR17520069.12508222
SRR17520069.12508223
SRR17520069.12508224
SRR17520069.12508225
SRR17520069.12508226
SRR17520069.12508227
SRR17520069.12508228
SRR17520069.12508230
SRR17520069.12510745
SRR17520069.12510746
SRR17520069.12510747
SRR17520069.12510748
SRR17520069.12510749
SRR17520069.12510750
SRR17520069.12510751
SRR17520069.12510752
SRR17520069.12510753
SRR17520069.12510754
SRR17520069.12510755
SRR17520069.12510760
SRR17520069.12510762
SRR17520069.12510765
SRR17520069.12510766
SRR17520069.12510767
SRR17520069.12510769
SRR17520069.12510775
SRR17520069.12510776
SRR17520069.12510778
SRR17520069.12510782
SRR17520069.12510789
SRR17520069.12510791
SRR17520069.12510792
SRR17520069.12510799
SRR17520069.12510803
SRR17520069.12510805
SRR17520069.12520087
SRR17520069.12520673
SRR17520069.12520674

SRR17520069.12520675
SRR17520069.12520676
SRR17520069.12520677
SRR17520069.12520678
SRR17520069.12520679
SRR17520069.12520680
SRR17520069.12520681
SRR17520069.12520682
SRR17520069.12520685
SRR17520069.12520686
SRR17520069.12520687
SRR17520069.12520688
SRR17520069.12520689
SRR17520069.12520690
SRR17520069.12520691
SRR17520069.12520692
SRR17520069.12520693
SRR17520069.12520694
SRR17520069.12520695
SRR17520069.12520696
SRR17520069.12520697
SRR17520069.12520698
SRR17520069.12520699
SRR17520069.12520700
SRR17520069.12520701
SRR17520069.12520702
SRR17520069.12520703
SRR17520069.12520704
SRR17520069.12520705
SRR17520069.12520706
SRR17520069.12520708
SRR17520069.12520709
SRR17520069.12520710
SRR17520069.12520711
SRR17520069.12520712
SRR17520069.12520713
SRR17520069.12520714
SRR17520069.12520715
SRR17520069.12520716
SRR17520069.12520717
SRR17520069.12520718
SRR17520069.12520719
SRR17520069.12520720
SRR17520069.12520721
SRR17520069.12520722
SRR17520069.12520723
SRR17520069.12520724

SRR17520069.12520725
SRR17520069.12520726
SRR17520069.12520727
SRR17520069.12520728
SRR17520069.12520729
SRR17520069.12520730
SRR17520069.12520731
SRR17520069.12520732
SRR17520069.12520733
SRR17520069.12520734
SRR17520069.12520735
SRR17520069.12520736
SRR17520069.12520737
SRR17520069.12520739
SRR17520069.12520740
SRR17520069.12520741
SRR17520069.12520742
SRR17520069.12520743
SRR17520069.12520744
SRR17520069.12520745
SRR17520069.12520746
SRR17520069.12520747
SRR17520069.12520748
SRR17520069.12520749
SRR17520069.12520750
SRR17520069.12520751
SRR17520069.12520752
SRR17520069.12520753
SRR17520069.12520754
SRR17520069.12520755
SRR17520069.12520756
SRR17520069.12520757
SRR17520069.12520758
SRR17520069.12520759
SRR17520069.12520760
SRR17520069.12520761
SRR17520069.12520762
SRR17520069.12520763
SRR17520069.12520764
SRR17520069.12520765
SRR17520069.12520766
SRR17520069.12520767
SRR17520069.12520769
SRR17520069.12520770
SRR17520069.12520771
SRR17520069.12520772
SRR17520069.12520773

SRR17520069.12520774
SRR17520069.12520775
SRR17520069.12520776
SRR17520069.12520777
SRR17520069.12520778
SRR17520069.12520779
SRR17520069.12520780
SRR17520069.12520781
SRR17520069.12520782
SRR17520069.12520783
SRR17520069.12520784
SRR17520069.12520785
SRR17520069.12520786
SRR17520069.12520787
SRR17520069.12520789
SRR17520069.12520790
SRR17520069.12520791
SRR17520069.12520792
SRR17520069.12520793
SRR17520069.12520794
SRR17520069.12520795
SRR17520069.12520796
SRR17520069.12520797
SRR17520069.12523717
SRR17520069.12523718
SRR17520069.12523719
SRR17520069.12523720
SRR17520069.12523721
SRR17520069.12523722
SRR17520069.12523723
SRR17520069.12523724
SRR17520069.12523725
SRR17520069.12523726
SRR17520069.12523727
SRR17520069.12523728
SRR17520069.12523729
SRR17520069.12523730
SRR17520069.12523731
SRR17520069.12523733
SRR17520069.12523734
SRR17520069.12523735
SRR17520069.12523736
SRR17520069.12523737
SRR17520069.12530518
SRR17520069.12530519
SRR17520069.12530521
SRR17520069.12530523

SRR17520069.12530524
SRR17520069.12530525
SRR17520069.12530527
SRR17520069.12530528
SRR17520069.12530529
SRR17520069.12530530
SRR17520069.12530531
SRR17520069.12530532
SRR17520069.12530533
SRR17520069.12530534
SRR17520069.12530535
SRR17520069.12536331
SRR17520069.12542852
SRR17520069.12542853
SRR17520069.12542856
SRR17520069.12542857
SRR17520069.12542858
SRR17520069.12542863
SRR17520069.12542864
SRR17520069.12558939
SRR17520069.12565387
SRR17520069.12588718
SRR17520069.12588721
SRR17520069.12589504
SRR17520069.12590656
SRR17520069.12611809
SRR17520069.12611810
SRR17520069.12611811
SRR17520069.12611812
SRR17520069.12611813
SRR17520069.12611814
SRR17520069.12611815
SRR17520069.12611816
SRR17520069.12611817
SRR17520069.12611818
SRR17520069.12611819
SRR17520069.12611820
SRR17520069.12611821
SRR17520069.12611822
SRR17520069.12611823
SRR17520069.12611824
SRR17520069.12611825
SRR17520069.12611826
SRR17520069.12611827
SRR17520069.12611828
SRR17520069.12611829
SRR17520069.12611830

SRR17520069.12611831
SRR17520069.12611832
SRR17520069.12611833
SRR17520069.12611834
SRR17520069.12611835
SRR17520069.12611836
SRR17520069.12611837
SRR17520069.12611838
SRR17520069.12611839
SRR17520069.12611840
SRR17520069.12611841
SRR17520069.12611842
SRR17520069.12611843
SRR17520069.12611844
SRR17520069.12611845
SRR17520069.12611846
SRR17520069.12611847
SRR17520069.12611848
SRR17520069.12611849
SRR17520069.12611850
SRR17520069.12611851
SRR17520069.12611852
SRR17520069.12611853
SRR17520069.12611854
SRR17520069.12611855
SRR17520069.12611856
SRR17520069.12611857
SRR17520069.12611858
SRR17520069.12611859
SRR17520069.12611860
SRR17520069.12611861
SRR17520069.12611862
SRR17520069.12611863
SRR17520069.12611864
SRR17520069.12611865
SRR17520069.12611866
SRR17520069.12611867
SRR17520069.12611868
SRR17520069.12611869
SRR17520069.12611870
SRR17520069.12611871
SRR17520069.12611872
SRR17520069.12611873
SRR17520069.12611874
SRR17520069.12611875
SRR17520069.12611877
SRR17520069.12611878

SRR17520069.12611879
SRR17520069.12611880
SRR17520069.12611881
SRR17520069.12611882
SRR17520069.12611883
SRR17520069.12611884
SRR17520069.12611885
SRR17520069.12611886
SRR17520069.12611887
SRR17520069.12611888
SRR17520069.12611889
SRR17520069.12611890
SRR17520069.12611891
SRR17520069.12611892
SRR17520069.12611893
SRR17520069.12611894
SRR17520069.12611895
SRR17520069.12611896
SRR17520069.12611897
SRR17520069.12611898
SRR17520069.12611899
SRR17520069.12611900
SRR17520069.12611901
SRR17520069.12611902
SRR17520069.12611903
SRR17520069.12611904
SRR17520069.12611905
SRR17520069.12611906
SRR17520069.12611907
SRR17520069.12611908
SRR17520069.12611909
SRR17520069.12611910
SRR17520069.12611911
SRR17520069.12611912
SRR17520069.12611913
SRR17520069.12611914
SRR17520069.12611915
SRR17520069.12611916
SRR17520069.12611917
SRR17520069.12611918
SRR17520069.12611919
SRR17520069.12611920
SRR17520069.12611921
SRR17520069.12611922
SRR17520069.12611923
SRR17520069.12611924
SRR17520069.12611925

SRR17520069.12611926
SRR17520069.12611927
SRR17520069.12611928
SRR17520069.12611930
SRR17520069.12611931
SRR17520069.12611932
SRR17520069.12611933
SRR17520069.12611934
SRR17520069.12611935
SRR17520069.12611936
SRR17520069.12611937
SRR17520069.12611938
SRR17520069.12611939
SRR17520069.12611940
SRR17520069.12611941
SRR17520069.12611942
SRR17520069.12614063
SRR17520069.12615406
SRR17520069.12615407
SRR17520069.12615408
SRR17520069.12615409
SRR17520069.12615410
SRR17520069.12615411
SRR17520069.12617629
SRR17520069.12617630
SRR17520069.12617631
SRR17520069.12617632
SRR17520069.12617634
SRR17520069.12617635
SRR17520069.12617636
SRR17520069.12617637
SRR17520069.12617638
SRR17520069.12617639
SRR17520069.12617640
SRR17520069.12617641
SRR17520069.12617642
SRR17520069.12619733
SRR17520069.12621099
SRR17520069.12645529
SRR17520069.12645532
SRR17520069.12645533
SRR17520069.12645534
SRR17520069.12645535
SRR17520069.12645536
SRR17520069.12645537
SRR17520069.12645538
SRR17520069.12645539

SRR17520069.12645540
SRR17520069.12645541
SRR17520069.12645542
SRR17520069.12645543
SRR17520069.12645544
SRR17520069.12645545
SRR17520069.12645546
SRR17520069.12645547
SRR17520069.12645548
SRR17520069.12645549
SRR17520069.12645550
SRR17520069.12645551
SRR17520069.12645552
SRR17520069.12645553
SRR17520069.12645554
SRR17520069.12645555
SRR17520069.12645556
SRR17520069.12645557
SRR17520069.12645558
SRR17520069.12645559
SRR17520069.12645560
SRR17520069.12645561
SRR17520069.12645562
SRR17520069.12645563
SRR17520069.12645564
SRR17520069.12645565
SRR17520069.12645566
SRR17520069.12645567
SRR17520069.12645568
SRR17520069.12645569
SRR17520069.12645570
SRR17520069.12645571
SRR17520069.12645572
SRR17520069.12645573
SRR17520069.12645574
SRR17520069.12645575
SRR17520069.12645576
SRR17520069.12645577
SRR17520069.12645578
SRR17520069.12645579
SRR17520069.12645580
SRR17520069.12645581
SRR17520069.12645582
SRR17520069.12645583
SRR17520069.12645584
SRR17520069.12645585
SRR17520069.12645586

SRR17520069.12645587
SRR17520069.12645588
SRR17520069.12645589
SRR17520069.12645590
SRR17520069.12645591
SRR17520069.12645592
SRR17520069.12645593
SRR17520069.12645594
SRR17520069.12645595
SRR17520069.12645596
SRR17520069.12645597
SRR17520069.12645598
SRR17520069.12645599
SRR17520069.12645600
SRR17520069.12645601
SRR17520069.12645602
SRR17520069.12645603
SRR17520069.12645604
SRR17520069.12645607
SRR17520069.12645608
SRR17520069.12646140
SRR17520069.12646141
SRR17520069.12646142
SRR17520069.12646143
SRR17520069.12646144
SRR17520069.12646145
SRR17520069.12646146
SRR17520069.12646148
SRR17520069.12646151
SRR17520069.12646152
SRR17520069.12646153
SRR17520069.12646154
SRR17520069.12646156
SRR17520069.12646157
SRR17520069.12646158
SRR17520069.12646159
SRR17520069.12646160
SRR17520069.12646162
SRR17520069.12646163
SRR17520069.12646164
SRR17520069.12646165
SRR17520069.12646168
SRR17520069.12646170
SRR17520069.12646171
SRR17520069.12646172
SRR17520069.12648731
SRR17520069.12648732

SRR17520069.12648733
SRR17520069.12648734
SRR17520069.12648735
SRR17520069.12648736
SRR17520069.12648737
SRR17520069.12648738
SRR17520069.12648739
SRR17520069.12648740
SRR17520069.12648741
SRR17520069.12648742
SRR17520069.12648743
SRR17520069.12648744
SRR17520069.12648745
SRR17520069.12648747
SRR17520069.12648748
SRR17520069.12648749
SRR17520069.12648750
SRR17520069.12648751
SRR17520069.12648752
SRR17520069.12648753
SRR17520069.12648754
SRR17520069.12648755
SRR17520069.12648756
SRR17520069.12648757
SRR17520069.12648758
SRR17520069.12648759
SRR17520069.12648760
SRR17520069.12648761
SRR17520069.12648762
SRR17520069.12648763
SRR17520069.12648764
SRR17520069.12648765
SRR17520069.12648766
SRR17520069.12648767
SRR17520069.12648768
SRR17520069.12648769
SRR17520069.12648770
SRR17520069.12648771
SRR17520069.12648772
SRR17520069.12648773
SRR17520069.12648774
SRR17520069.12648775
SRR17520069.12648776
SRR17520069.12648777
SRR17520069.12648778
SRR17520069.12648779
SRR17520069.12683788

SRR17520069.12691631
SRR17520069.12694976
SRR17520069.12694978
SRR17520069.12694979
SRR17520069.12694980
SRR17520069.12694981
SRR17520069.12694982
SRR17520069.12694983
SRR17520069.12694984
SRR17520069.12694986
SRR17520069.12694987
SRR17520069.12694988
SRR17520069.12694989
SRR17520069.12694990
SRR17520069.12694991
SRR17520069.12694992
SRR17520069.12694993
SRR17520069.12694994
SRR17520069.12694995
SRR17520069.12694996
SRR17520069.12695000
SRR17520069.12695001
SRR17520069.12695002
SRR17520069.12695003
SRR17520069.12695004
SRR17520069.12695005
SRR17520069.12695006
SRR17520069.12695007
SRR17520069.12695008
SRR17520069.12695009
SRR17520069.12695010
SRR17520069.12695011
SRR17520069.12695012
SRR17520069.12695013
SRR17520069.12695014
SRR17520069.12695015
SRR17520069.12695016
SRR17520069.12695017
SRR17520069.12695018
SRR17520069.12695019
SRR17520069.12695020
SRR17520069.12695021
SRR17520069.12695022
SRR17520069.12695023
SRR17520069.12695024
SRR17520069.12695025
SRR17520069.12695026

SRR17520069.12695027
SRR17520069.12695028
SRR17520069.12695029
SRR17520069.12695030
SRR17520069.12695031
SRR17520069.12695032
SRR17520069.12695033
SRR17520069.12695034
SRR17520069.12695035
SRR17520069.12695036
SRR17520069.12695037
SRR17520069.12695038
SRR17520069.12695039
SRR17520069.12695040
SRR17520069.12695041
SRR17520069.12695042
SRR17520069.12695043
SRR17520069.12695044
SRR17520069.12695045
SRR17520069.12695046
SRR17520069.12695047
SRR17520069.12695048
SRR17520069.12695049
SRR17520069.12695050
SRR17520069.12695052
SRR17520069.12695053
SRR17520069.12695054
SRR17520069.12695055
SRR17520069.12695056
SRR17520069.12695057
SRR17520069.12695058
SRR17520069.12695059
SRR17520069.12695060
SRR17520069.12695061
SRR17520069.12695062
SRR17520069.12695063
SRR17520069.12695064
SRR17520069.12695065
SRR17520069.12695066
SRR17520069.12695067
SRR17520069.12695068
SRR17520069.12695069
SRR17520069.12695070
SRR17520069.12695071
SRR17520069.12695072
SRR17520069.12695073
SRR17520069.12695074

SRR17520069.12695075
SRR17520069.12695076
SRR17520069.12695077
SRR17520069.12695078
SRR17520069.12695079
SRR17520069.12695080
SRR17520069.12695081
SRR17520069.12695082
SRR17520069.12695083
SRR17520069.12695084
SRR17520069.12695085
SRR17520069.12695086
SRR17520069.12695087
SRR17520069.12695088
SRR17520069.12695089
SRR17520069.12695090
SRR17520069.12695091
SRR17520069.12695092
SRR17520069.12695093
SRR17520069.12695094
SRR17520069.12695095
SRR17520069.12695096
SRR17520069.12695097
SRR17520069.12695098
SRR17520069.12695099
SRR17520069.12695100
SRR17520069.12695101
SRR17520069.12695102
SRR17520069.12695103
SRR17520069.12695104
SRR17520069.12695105
SRR17520069.12695106
SRR17520069.12695107
SRR17520069.12695108
SRR17520069.12695109
SRR17520069.12695110
SRR17520069.12695111
SRR17520069.12695112
SRR17520069.12695113
SRR17520069.12695114
SRR17520069.12695115
SRR17520069.12695116
SRR17520069.12695117
SRR17520069.12695118
SRR17520069.12695119
SRR17520069.12695120
SRR17520069.12695121

SRR17520069.12695122
SRR17520069.12695123
SRR17520069.12695124
SRR17520069.12695125
SRR17520069.12695126
SRR17520069.12695127
SRR17520069.12695128
SRR17520069.12695129
SRR17520069.12695130
SRR17520069.12695131
SRR17520069.12695132
SRR17520069.12695133
SRR17520069.12695134
SRR17520069.12695135
SRR17520069.12695136
SRR17520069.12695137
SRR17520069.12695138
SRR17520069.12695139
SRR17520069.12695140
SRR17520069.12695141
SRR17520069.12695143
SRR17520069.12695144
SRR17520069.12695145
SRR17520069.12695146
SRR17520069.12696542
SRR17520069.12698819
SRR17520069.12699662
SRR17520069.12699663
SRR17520069.12699664
SRR17520069.12699665
SRR17520069.12699666
SRR17520069.12699667
SRR17520069.12699668
SRR17520069.12699669
SRR17520069.12699670
SRR17520069.12699671
SRR17520069.12699672
SRR17520069.12699673
SRR17520069.12699674
SRR17520069.12699675
SRR17520069.12699676
SRR17520069.12699677
SRR17520069.12699678
SRR17520069.12699679
SRR17520069.12699680
SRR17520069.12699681
SRR17520069.12699682

SRR17520069.12699683
SRR17520069.12699684
SRR17520069.12699685
SRR17520069.12699686
SRR17520069.12699687
SRR17520069.12699688
SRR17520069.12699689
SRR17520069.12699690
SRR17520069.12699691
SRR17520069.12699692
SRR17520069.12699693
SRR17520069.12699694
SRR17520069.12699695
SRR17520069.12699696
SRR17520069.12699697
SRR17520069.12699699
SRR17520069.12699700
SRR17520069.12699701
SRR17520069.12699702
SRR17520069.12699703
SRR17520069.12699704
SRR17520069.12699705
SRR17520069.12699706
SRR17520069.12699707
SRR17520069.12699708
SRR17520069.12699709
SRR17520069.12699710
SRR17520069.12699711
SRR17520069.12699712
SRR17520069.12699713
SRR17520069.12699714
SRR17520069.12699715
SRR17520069.12699716
SRR17520069.12699717
SRR17520069.12699718
SRR17520069.12699719
SRR17520069.12699720
SRR17520069.12699721
SRR17520069.12699722
SRR17520069.12699723
SRR17520069.12699724
SRR17520069.12699725
SRR17520069.12699726
SRR17520069.12699727
SRR17520069.12699728
SRR17520069.12699729
SRR17520069.12699730

SRR17520069.12699731
SRR17520069.12699732
SRR17520069.12699733
SRR17520069.12699734
SRR17520069.12699735
SRR17520069.12699736
SRR17520069.12699737
SRR17520069.12699738
SRR17520069.12699739
SRR17520069.12699740
SRR17520069.12699741
SRR17520069.12699742
SRR17520069.12699744
SRR17520069.12699745
SRR17520069.12699746
SRR17520069.12699747
SRR17520069.12699748
SRR17520069.12699749
SRR17520069.12699750
SRR17520069.12699751
SRR17520069.12699752
SRR17520069.12699753
SRR17520069.12699754
SRR17520069.12699755
SRR17520069.12699756
SRR17520069.12699757
SRR17520069.12699758
SRR17520069.12699759
SRR17520069.12699760
SRR17520069.12699761
SRR17520069.12699762
SRR17520069.12699763
SRR17520069.12699764
SRR17520069.12699766
SRR17520069.12699767
SRR17520069.12699768
SRR17520069.12699769
SRR17520069.12699770
SRR17520069.12699771
SRR17520069.12699772
SRR17520069.12699773
SRR17520069.12699774
SRR17520069.12699775
SRR17520069.12699776
SRR17520069.12699777
SRR17520069.12699778
SRR17520069.12699779

SRR17520069.12699780
SRR17520069.12699781
SRR17520069.12699782
SRR17520069.12699783
SRR17520069.12699784
SRR17520069.12699785
SRR17520069.12699786
SRR17520069.12699787
SRR17520069.12699788
SRR17520069.12699790
SRR17520069.12712730
SRR17520069.12712731
SRR17520069.12712735
SRR17520069.12712736
SRR17520069.12712737
SRR17520069.12712738
SRR17520069.12712739
SRR17520069.12712740
SRR17520069.12712743
SRR17520069.12712744
SRR17520069.12712747
SRR17520069.12712748
SRR17520069.12731563
SRR17520069.12735757
SRR17520069.12735758
SRR17520069.12735759
SRR17520069.12735760
SRR17520069.12735761
SRR17520069.12735762
SRR17520069.12735763
SRR17520069.12735764
SRR17520069.12735765
SRR17520069.12735766
SRR17520069.12735767
SRR17520069.12735768
SRR17520069.12735769
SRR17520069.12735771
SRR17520069.12735772
SRR17520069.12735773
SRR17520069.12735774
SRR17520069.12735775
SRR17520069.12735776
SRR17520069.12735777
SRR17520069.12735778
SRR17520069.12735779
SRR17520069.12735780
SRR17520069.12735781

SRR17520069.12735782
SRR17520069.12735783
SRR17520069.12735784
SRR17520069.12735785
SRR17520069.12735786
SRR17520069.12735787
SRR17520069.12735788
SRR17520069.12735789
SRR17520069.12735790
SRR17520069.12735791
SRR17520069.12735792
SRR17520069.12756949
SRR17520069.12756950
SRR17520069.12756951
SRR17520069.12756952
SRR17520069.12756953
SRR17520069.12756954
SRR17520069.12756955
SRR17520069.12756956
SRR17520069.12756957
SRR17520069.12756958
SRR17520069.12756959
SRR17520069.12756960
SRR17520069.12756961
SRR17520069.12756962
SRR17520069.12756963
SRR17520069.12756964
SRR17520069.12756965
SRR17520069.12756967
SRR17520069.12756968
SRR17520069.12756969
SRR17520069.12756970
SRR17520069.12756971
SRR17520069.12756972
SRR17520069.12756973
SRR17520069.12756974
SRR17520069.12756975
SRR17520069.12756976
SRR17520069.12756977
SRR17520069.12756978
SRR17520069.12756979
SRR17520069.12756980
SRR17520069.12756981
SRR17520069.12756982
SRR17520069.12756983
SRR17520069.12756984
SRR17520069.12756985

SRR17520069.12756987
SRR17520069.12756988
SRR17520069.12756989
SRR17520069.12756990
SRR17520069.12756991
SRR17520069.12756992
SRR17520069.12756993
SRR17520069.12756994
SRR17520069.12756995
SRR17520069.12756996
SRR17520069.12756997
SRR17520069.12756998
SRR17520069.12756999
SRR17520069.12757000
SRR17520069.12757001
SRR17520069.12757003
SRR17520069.12757004
SRR17520069.12757005
SRR17520069.12757006
SRR17520069.12757007
SRR17520069.12757008
SRR17520069.12757009
SRR17520069.12757010
SRR17520069.12757011
SRR17520069.12757012
SRR17520069.12757013
SRR17520069.12757014
SRR17520069.12757015
SRR17520069.12757016
SRR17520069.12757017
SRR17520069.12757018
SRR17520069.12757019
SRR17520069.12757020
SRR17520069.12757021
SRR17520069.12757022
SRR17520069.12757023
SRR17520069.12757024
SRR17520069.12757025
SRR17520069.12757026
SRR17520069.12757027
SRR17520069.12757028
SRR17520069.12757029
SRR17520069.12757030
SRR17520069.12757031
SRR17520069.12757032
SRR17520069.12757033
SRR17520069.12757034

SRR17520069.12757035
SRR17520069.12757036
SRR17520069.12757037
SRR17520069.12757038
SRR17520069.12757039
SRR17520069.12757040
SRR17520069.12757041
SRR17520069.12757042
SRR17520069.12757043
SRR17520069.12757044
SRR17520069.12757045
SRR17520069.12757046
SRR17520069.12757047
SRR17520069.12757048
SRR17520069.12757049
SRR17520069.12757050
SRR17520069.12757051
SRR17520069.12757052
SRR17520069.12757053
SRR17520069.12757054
SRR17520069.12757055
SRR17520069.12757056
SRR17520069.12757057
SRR17520069.12757058
SRR17520069.12757059
SRR17520069.12757060
SRR17520069.12759115
SRR17520069.12762837
SRR17520069.12762839
SRR17520069.12768388
SRR17520069.12787890
SRR17520069.12791313
SRR17520069.12791314
SRR17520069.12791315
SRR17520069.12791316
SRR17520069.12791317
SRR17520069.12791318
SRR17520069.12791319
SRR17520069.12791320
SRR17520069.12791321
SRR17520069.12791322
SRR17520069.12791323
SRR17520069.12791324
SRR17520069.12791325
SRR17520069.12791327
SRR17520069.12791328
SRR17520069.12791329

SRR17520069.12791330
SRR17520069.12791331
SRR17520069.12791332
SRR17520069.12791334
SRR17520069.12791335
SRR17520069.12791336
SRR17520069.12791338
SRR17520069.12791339
SRR17520069.12791340
SRR17520069.12791341
SRR17520069.12791342
SRR17520069.12791343
SRR17520069.12791344
SRR17520069.12791345
SRR17520069.12791346
SRR17520069.12791347
SRR17520069.12791348
SRR17520069.12791349
SRR17520069.12791350
SRR17520069.12791351
SRR17520069.12791352
SRR17520069.12802794
SRR17520069.12802795
SRR17520069.12802796
SRR17520069.12802797
SRR17520069.12802798
SRR17520069.12802799
SRR17520069.12802800
SRR17520069.12802801
SRR17520069.12802802
SRR17520069.12802804
SRR17520069.12802805
SRR17520069.12802806
SRR17520069.12802807
SRR17520069.12802808
SRR17520069.12802809
SRR17520069.12802810
SRR17520069.12802811
SRR17520069.12802812
SRR17520069.12802813
SRR17520069.12802814
SRR17520069.12802815
SRR17520069.12802816
SRR17520069.12802817
SRR17520069.12802818
SRR17520069.12802819
SRR17520069.12802820

SRR17520069.12802821
SRR17520069.12802822
SRR17520069.12802823
SRR17520069.12802824
SRR17520069.12802825
SRR17520069.12802826
SRR17520069.12802827
SRR17520069.12802828
SRR17520069.12802829
SRR17520069.12802830
SRR17520069.12802831
SRR17520069.12802832
SRR17520069.12802833
SRR17520069.12802834
SRR17520069.12802835
SRR17520069.12802836
SRR17520069.12802837
SRR17520069.12802838
SRR17520069.12802839
SRR17520069.12802840
SRR17520069.12802841
SRR17520069.12802842
SRR17520069.12802843
SRR17520069.12802844
SRR17520069.12802845
SRR17520069.12802846
SRR17520069.12802847
SRR17520069.12802848
SRR17520069.12802849
SRR17520069.12802850
SRR17520069.12802851
SRR17520069.12802852
SRR17520069.12802853
SRR17520069.12802854
SRR17520069.12802855
SRR17520069.12802856
SRR17520069.12802857
SRR17520069.12802858
SRR17520069.12802859
SRR17520069.12802860
SRR17520069.12802861
SRR17520069.12802862
SRR17520069.12802863
SRR17520069.12802864
SRR17520069.12802865
SRR17520069.12802866
SRR17520069.12802867

SRR17520069.12802868
SRR17520069.12802869
SRR17520069.12802870
SRR17520069.12802872
SRR17520069.12802873
SRR17520069.12802874
SRR17520069.12802875
SRR17520069.12802876
SRR17520069.12802877
SRR17520069.12802878
SRR17520069.12802879
SRR17520069.12802880
SRR17520069.12802881
SRR17520069.12802882
SRR17520069.12802883
SRR17520069.12802884
SRR17520069.12802885
SRR17520069.12802886
SRR17520069.12802887
SRR17520069.12802888
SRR17520069.12802889
SRR17520069.12802890
SRR17520069.12802891
SRR17520069.12802892
SRR17520069.12802893
SRR17520069.12802894
SRR17520069.12802895
SRR17520069.12802896
SRR17520069.12802897
SRR17520069.12802899
SRR17520069.12802900
SRR17520069.12802901
SRR17520069.12802902
SRR17520069.12802903
SRR17520069.12802904
SRR17520069.12802905
SRR17520069.12825560
SRR17520069.12825752
SRR17520069.12825753
SRR17520069.12825754
SRR17520069.12825755
SRR17520069.12825756
SRR17520069.12825757
SRR17520069.12825758
SRR17520069.12825759
SRR17520069.12825760
SRR17520069.12825761

SRR17520069.12837172
SRR17520069.12854284
SRR17520069.12885778
SRR17520069.12888694
SRR17520069.12888695
SRR17520069.12888696
SRR17520069.12888697
SRR17520069.12888698
SRR17520069.12888699
SRR17520069.12888700
SRR17520069.12888701
SRR17520069.12888702
SRR17520069.12888703
SRR17520069.12888704
SRR17520069.12888706
SRR17520069.12888707
SRR17520069.12888708
SRR17520069.12888710
SRR17520069.12888711
SRR17520069.12888712
SRR17520069.12888713
SRR17520069.12888714
SRR17520069.12888715
SRR17520069.12888718
SRR17520069.12888719
SRR17520069.12888720
SRR17520069.12888721
SRR17520069.12888722
SRR17520069.12888723
SRR17520069.12888724
SRR17520069.12888725
SRR17520069.12888726
SRR17520069.12888727
SRR17520069.12888728
SRR17520069.12888729
SRR17520069.12888730
SRR17520069.12888731
SRR17520069.12888732
SRR17520069.12888734
SRR17520069.12888735
SRR17520069.12888736
SRR17520069.12888738
SRR17520069.12888739
SRR17520069.12888740
SRR17520069.12888743
SRR17520069.12888744
SRR17520069.12888745

SRR17520069.12888747
SRR17520069.12888749
SRR17520069.12888751
SRR17520069.12888752
SRR17520069.12888753
SRR17520069.12888754
SRR17520069.12888755
SRR17520069.12888757
SRR17520069.12888758
SRR17520069.12888759
SRR17520069.12888760
SRR17520069.12888761
SRR17520069.12888762
SRR17520069.12888763
SRR17520069.12888764
SRR17520069.12888765
SRR17520069.12888766
SRR17520069.12888767
SRR17520069.12888769
SRR17520069.12888770
SRR17520069.12888771
SRR17520069.12888772
SRR17520069.12888774
SRR17520069.12902324
SRR17520069.12902325
SRR17520069.12902326
SRR17520069.12902327
SRR17520069.12902328
SRR17520069.12902329
SRR17520069.12902330
SRR17520069.12902331
SRR17520069.12902332
SRR17520069.12902334
SRR17520069.12902335
SRR17520069.12902336
SRR17520069.12902337
SRR17520069.12902338
SRR17520069.12902339
SRR17520069.12902340
SRR17520069.12902341
SRR17520069.12902342
SRR17520069.12902343
SRR17520069.12902344
SRR17520069.12902345
SRR17520069.12902346
SRR17520069.12902347
SRR17520069.12902348

SRR17520069.12902349
SRR17520069.12902350
SRR17520069.12902351
SRR17520069.12902352
SRR17520069.12902353
SRR17520069.12902354
SRR17520069.12902355
SRR17520069.12902356
SRR17520069.12902357
SRR17520069.12902358
SRR17520069.12902359
SRR17520069.12902360
SRR17520069.12902361
SRR17520069.12902362
SRR17520069.12902363
SRR17520069.12902365
SRR17520069.12902366
SRR17520069.12902367
SRR17520069.12902368
SRR17520069.12902369
SRR17520069.12902370
SRR17520069.12902371
SRR17520069.12902372
SRR17520069.12902373
SRR17520069.12902374
SRR17520069.12902375
SRR17520069.12902376
SRR17520069.12902377
SRR17520069.12902378
SRR17520069.12902379
SRR17520069.12902380
SRR17520069.12902381
SRR17520069.12902382
SRR17520069.12902383
SRR17520069.12902384
SRR17520069.12902385
SRR17520069.12902386
SRR17520069.12902387
SRR17520069.12902388
SRR17520069.12902389
SRR17520069.12902390
SRR17520069.12902391
SRR17520069.12902392
SRR17520069.12902393
SRR17520069.12902394
SRR17520069.12902395
SRR17520069.12902396

SRR17520069.12902397
SRR17520069.12902398
SRR17520069.12902399
SRR17520069.12902400
SRR17520069.12902401
SRR17520069.12902402
SRR17520069.12902403
SRR17520069.12902404
SRR17520069.12902405
SRR17520069.12902406
SRR17520069.12902407
SRR17520069.12902408
SRR17520069.12902409
SRR17520069.12902410
SRR17520069.12902411
SRR17520069.12902412
SRR17520069.12902413
SRR17520069.12902414
SRR17520069.12902415
SRR17520069.12902416
SRR17520069.12902417
SRR17520069.12902418
SRR17520069.12928605
SRR17520069.12928606
SRR17520069.12928607
SRR17520069.12928608
SRR17520069.12928609
SRR17520069.12928610
SRR17520069.12928611
SRR17520069.12928612
SRR17520069.12928613
SRR17520069.12928614
SRR17520069.12928615
SRR17520069.12928616
SRR17520069.12928617
SRR17520069.12928618
SRR17520069.12928619
SRR17520069.12928620
SRR17520069.12928621
SRR17520069.12928622
SRR17520069.12928623
SRR17520069.12928624
SRR17520069.12928625
SRR17520069.12928626
SRR17520069.12928627
SRR17520069.12928628
SRR17520069.12928629

SRR17520069.12928630
SRR17520069.12928631
SRR17520069.12928632
SRR17520069.12928633
SRR17520069.12928634
SRR17520069.12928635
SRR17520069.12928636
SRR17520069.12928637
SRR17520069.12928638
SRR17520069.12928639
SRR17520069.12928640
SRR17520069.12928641
SRR17520069.12928642
SRR17520069.12928643
SRR17520069.12928644
SRR17520069.12928645
SRR17520069.12928646
SRR17520069.12928647
SRR17520069.12928648
SRR17520069.12928649
SRR17520069.12928650
SRR17520069.12928651
SRR17520069.12928653
SRR17520069.12932001
SRR17520069.12932002
SRR17520069.12932003
SRR17520069.12932004
SRR17520069.12932005
SRR17520069.12932006
SRR17520069.12932007
SRR17520069.12932008
SRR17520069.12932009
SRR17520069.12941039
SRR17520069.12941040
SRR17520069.12941042
SRR17520069.12941044
SRR17520069.12944104
SRR17520069.12952626
SRR17520069.12952627
SRR17520069.12952628
SRR17520069.12952629
SRR17520069.12952630
SRR17520069.12952631
SRR17520069.12952632
SRR17520069.12952633
SRR17520069.12952634
SRR17520069.12952635

SRR17520069.12952636
SRR17520069.12952637
SRR17520069.12952638
SRR17520069.12952639
SRR17520069.12952640
SRR17520069.12952641
SRR17520069.12952642
SRR17520069.12952643
SRR17520069.12952644
SRR17520069.12952645
SRR17520069.12952646
SRR17520069.12952647
SRR17520069.12952648
SRR17520069.12952649
SRR17520069.12952650
SRR17520069.12952651
SRR17520069.12952652
SRR17520069.12952653
SRR17520069.12952654
SRR17520069.12952655
SRR17520069.12952656
SRR17520069.12952657
SRR17520069.12952658
SRR17520069.12952659
SRR17520069.12952660
SRR17520069.12952661
SRR17520069.12952662
SRR17520069.12952663
SRR17520069.12952664
SRR17520069.12952665
SRR17520069.12952666
SRR17520069.12952667
SRR17520069.12952668
SRR17520069.12952669
SRR17520069.12952670
SRR17520069.12952671
SRR17520069.12952672
SRR17520069.12952673
SRR17520069.12952680
SRR17520069.12952688
SRR17520069.12952690
SRR17520069.12952702
SRR17520069.12952712
SRR17520069.12955729
SRR17520069.12955730
SRR17520069.12955731
SRR17520069.12955732

SRR17520069.12955733
SRR17520069.12955734
SRR17520069.12955736
SRR17520069.12955737
SRR17520069.12955738
SRR17520069.12955739
SRR17520069.12955740
SRR17520069.12955741
SRR17520069.12955742
SRR17520069.12957744
SRR17520069.12957745
SRR17520069.12957746
SRR17520069.12957747
SRR17520069.12957748
SRR17520069.12957749
SRR17520069.12957750
SRR17520069.12957751
SRR17520069.12957752
SRR17520069.12957753
SRR17520069.12957754
SRR17520069.12957755
SRR17520069.12957756
SRR17520069.12957757
SRR17520069.12959313
SRR17520069.12959369
SRR17520069.12959952
SRR17520069.12959953
SRR17520069.12959954
SRR17520069.12959955
SRR17520069.12959956
SRR17520069.12959957
SRR17520069.12959958
SRR17520069.12959959
SRR17520069.12959960
SRR17520069.12959961
SRR17520069.12959962
SRR17520069.12959963
SRR17520069.12959964
SRR17520069.12959965
SRR17520069.12959966
SRR17520069.12959967
SRR17520069.12959968
SRR17520069.12959969
SRR17520069.12959970
SRR17520069.12959971
SRR17520069.12959972
SRR17520069.12959973

SRR17520069.12959974
SRR17520069.12959975
SRR17520069.12959976
SRR17520069.12959977
SRR17520069.12959980
SRR17520069.12959981
SRR17520069.12959982
SRR17520069.12959983
SRR17520069.12959984
SRR17520069.12959985
SRR17520069.12959986
SRR17520069.12959987
SRR17520069.12959988
SRR17520069.12959989
SRR17520069.12959991
SRR17520069.12959992
SRR17520069.12959993
SRR17520069.12959994
SRR17520069.12959995
SRR17520069.12959996
SRR17520069.12959997
SRR17520069.12959998
SRR17520069.12959999
SRR17520069.12960000
SRR17520069.12960001
SRR17520069.12960002
SRR17520069.12960003
SRR17520069.12960004
SRR17520069.12960005
SRR17520069.12960006
SRR17520069.12960007
SRR17520069.12960009
SRR17520069.12960010
SRR17520069.12960011
SRR17520069.12960012
SRR17520069.12960013
SRR17520069.12960014
SRR17520069.12960015
SRR17520069.12960016
SRR17520069.12960017
SRR17520069.12960018
SRR17520069.12960019
SRR17520069.12960020
SRR17520069.12960021
SRR17520069.12960022
SRR17520069.12960023
SRR17520069.12960024

SRR17520069.12960026
SRR17520069.12960027
SRR17520069.12960028
SRR17520069.12960029
SRR17520069.12960030
SRR17520069.12960031
SRR17520069.12960032
SRR17520069.12960033
SRR17520069.12960034
SRR17520069.12960035
SRR17520069.12960036
SRR17520069.12960037
SRR17520069.12960038
SRR17520069.12960039
SRR17520069.12960040
SRR17520069.12960041
SRR17520069.12960042
SRR17520069.12960043
SRR17520069.12960044
SRR17520069.12960045
SRR17520069.12960046
SRR17520069.12960047
SRR17520069.12960048
SRR17520069.12960049
SRR17520069.12960050
SRR17520069.12960051
SRR17520069.12960052
SRR17520069.12960053
SRR17520069.12960054
SRR17520069.12960055
SRR17520069.12960056
SRR17520069.12960057
SRR17520069.12960058
SRR17520069.12960059
SRR17520069.12960060
SRR17520069.12960061
SRR17520069.12960062
SRR17520069.12960063
SRR17520069.12960064
SRR17520069.12960065
SRR17520069.12960066
SRR17520069.12960067
SRR17520069.12960068
SRR17520069.12960069
SRR17520069.12960070
SRR17520069.12960071
SRR17520069.12960072

SRR17520069.12960073
SRR17520069.12960074
SRR17520069.12960075
SRR17520069.12960076
SRR17520069.12960077
SRR17520069.12960078
SRR17520069.12960079
SRR17520069.12960080
SRR17520069.12960081
SRR17520069.12960082
SRR17520069.12960083
SRR17520069.12960084
SRR17520069.12960085
SRR17520069.12960086
SRR17520069.12973309
SRR17520069.12973310
SRR17520069.12973311
SRR17520069.12973312
SRR17520069.12973313
SRR17520069.12973314
SRR17520069.12973315
SRR17520069.12973316
SRR17520069.12973317
SRR17520069.12973318
SRR17520069.12973319
SRR17520069.12973320
SRR17520069.12973321
SRR17520069.12973322
SRR17520069.12973323
SRR17520069.12973324
SRR17520069.12973325
SRR17520069.12973326
SRR17520069.12973327
SRR17520069.12973328
SRR17520069.12973329
SRR17520069.12973330
SRR17520069.12973331
SRR17520069.12973332
SRR17520069.12973333
SRR17520069.12973334
SRR17520069.12973335
SRR17520069.12973336
SRR17520069.12973337
SRR17520069.12973338
SRR17520069.12973339
SRR17520069.12973340
SRR17520069.12973341

SRR17520069.12973342
SRR17520069.12973343
SRR17520069.12973344
SRR17520069.12973345
SRR17520069.12973346
SRR17520069.12973347
SRR17520069.12973348
SRR17520069.12973349
SRR17520069.12973350
SRR17520069.12973351
SRR17520069.12973352
SRR17520069.12973353
SRR17520069.12973354
SRR17520069.12973355
SRR17520069.12973356
SRR17520069.12973357
SRR17520069.12973358
SRR17520069.12973359
SRR17520069.12973360
SRR17520069.12973361
SRR17520069.12973362
SRR17520069.12973363
SRR17520069.12973364
SRR17520069.12973365
SRR17520069.12973366
SRR17520069.12973367
SRR17520069.12973368
SRR17520069.12973369
SRR17520069.12973370
SRR17520069.12973371
SRR17520069.12973372
SRR17520069.12973373
SRR17520069.12973374
SRR17520069.12973375
SRR17520069.12973376
SRR17520069.12973377
SRR17520069.12973378
SRR17520069.12973379
SRR17520069.12973380
SRR17520069.12973381
SRR17520069.12973382
SRR17520069.12973383
SRR17520069.12973384
SRR17520069.12973385
SRR17520069.12973386
SRR17520069.12973387
SRR17520069.12973388

SRR17520069.12973389
SRR17520069.12973390
SRR17520069.12973391
SRR17520069.12973392
SRR17520069.12973393
SRR17520069.12973394
SRR17520069.12973395
SRR17520069.12973396
SRR17520069.12973397
SRR17520069.12973398
SRR17520069.12973399
SRR17520069.12973400
SRR17520069.12973401
SRR17520069.12973402
SRR17520069.12973403
SRR17520069.12973404
SRR17520069.12973405
SRR17520069.12973406
SRR17520069.12973407
SRR17520069.12973408
SRR17520069.12973409
SRR17520069.12973410
SRR17520069.12973411
SRR17520069.12973412
SRR17520069.12973413
SRR17520069.12973414
SRR17520069.12973415
SRR17520069.12973416
SRR17520069.12973417
SRR17520069.12973418
SRR17520069.12973419
SRR17520069.12973420
SRR17520069.12973421
SRR17520069.12973422
SRR17520069.12973423
SRR17520069.12973424
SRR17520069.12973425
SRR17520069.12973426
SRR17520069.12973427
SRR17520069.12973428
SRR17520069.12973429
SRR17520069.12973430
SRR17520069.12973431
SRR17520069.12974978
SRR17520069.12974980
SRR17520069.12974981
SRR17520069.12987717

SRR17520069.12987734
SRR17520069.12987759
SRR17520069.13006314
SRR17520069.13006315
SRR17520069.13028638
SRR17520069.13028639
SRR17520069.13028640
SRR17520069.13028641
SRR17520069.13028642
SRR17520069.13028643
SRR17520069.13028644
SRR17520069.13028645
SRR17520069.13031044
SRR17520069.13031045
SRR17520069.13031047
SRR17520069.13031048
SRR17520069.13031049
SRR17520069.13031051
SRR17520069.13031052
SRR17520069.13031053
SRR17520069.13031055
SRR17520069.13031056
SRR17520069.13031057
SRR17520069.13031058
SRR17520069.13031059
SRR17520069.13031061
SRR17520069.13031062
SRR17520069.13031063
SRR17520069.13031064
SRR17520069.13031065
SRR17520069.13031066
SRR17520069.13031068
SRR17520069.13031069
SRR17520069.13031070
SRR17520069.13031071
SRR17520069.13031073
SRR17520069.13031075
SRR17520069.13031076
SRR17520069.13031077
SRR17520069.13031078
SRR17520069.13031079
SRR17520069.13031080
SRR17520069.13031081
SRR17520069.13031082
SRR17520069.13031083
SRR17520069.13031084
SRR17520069.13031085

SRR17520069.13031087
SRR17520069.13031088
SRR17520069.13031089
SRR17520069.13031090
SRR17520069.13031093
SRR17520069.13031094
SRR17520069.13031095
SRR17520069.13031097
SRR17520069.13031098
SRR17520069.13031099
SRR17520069.13031100
SRR17520069.13031101
SRR17520069.13031102
SRR17520069.13031103
SRR17520069.13031104
SRR17520069.13031105
SRR17520069.13031106
SRR17520069.13031107
SRR17520069.13031108
SRR17520069.13031109
SRR17520069.13031110
SRR17520069.13031111
SRR17520069.13031112
SRR17520069.13031113
SRR17520069.13031114
SRR17520069.13031115
SRR17520069.13031116
SRR17520069.13031117
SRR17520069.13031119
SRR17520069.13031120
SRR17520069.13031121
SRR17520069.13031122
SRR17520069.13031123
SRR17520069.13031124
SRR17520069.13031125
SRR17520069.13031126
SRR17520069.13031127
SRR17520069.13031128
SRR17520069.13031129
SRR17520069.13031130
SRR17520069.13031131
SRR17520069.13031132
SRR17520069.13031133
SRR17520069.13031134
SRR17520069.13031135
SRR17520069.13031136
SRR17520069.13031137

SRR17520069.13031138
SRR17520069.13031139
SRR17520069.13031140
SRR17520069.13031141
SRR17520069.13039600
SRR17520069.13039601
SRR17520069.13039602
SRR17520069.13039603
SRR17520069.13039604
SRR17520069.13039605
SRR17520069.13039606
SRR17520069.13039607
SRR17520069.13039608
SRR17520069.13039609
SRR17520069.13039610
SRR17520069.13039611
SRR17520069.13039612
SRR17520069.13039613
SRR17520069.13039614
SRR17520069.13039615
SRR17520069.13039616
SRR17520069.13039617
SRR17520069.13039618
SRR17520069.13039619
SRR17520069.13039620
SRR17520069.13039621
SRR17520069.13039622
SRR17520069.13039623
SRR17520069.13039624
SRR17520069.13039625
SRR17520069.13039626
SRR17520069.13039627
SRR17520069.13039628
SRR17520069.13039629
SRR17520069.13039630
SRR17520069.13039631
SRR17520069.13039632
SRR17520069.13039633
SRR17520069.13039634
SRR17520069.13039635
SRR17520069.13039636
SRR17520069.13039637
SRR17520069.13039638
SRR17520069.13039639
SRR17520069.13039640
SRR17520069.13039641
SRR17520069.13039642

SRR17520069.13039643
SRR17520069.13039644
SRR17520069.13039645
SRR17520069.13039646
SRR17520069.13039647
SRR17520069.13039648
SRR17520069.13039649
SRR17520069.13039650
SRR17520069.13039651
SRR17520069.13039652
SRR17520069.13039653
SRR17520069.13039654
SRR17520069.13039655
SRR17520069.13039656
SRR17520069.13039657
SRR17520069.13039658
SRR17520069.13039659
SRR17520069.13039660
SRR17520069.13039661
SRR17520069.13039662
SRR17520069.13039663
SRR17520069.13039664
SRR17520069.13039665
SRR17520069.13039666
SRR17520069.13039667
SRR17520069.13039668
SRR17520069.13039669
SRR17520069.13039670
SRR17520069.13039671
SRR17520069.13039672
SRR17520069.13039673
SRR17520069.13039674
SRR17520069.13039675
SRR17520069.13039676
SRR17520069.13039677
SRR17520069.13039678
SRR17520069.13039679
SRR17520069.13039680
SRR17520069.13039681
SRR17520069.13039682
SRR17520069.13039683
SRR17520069.13039684
SRR17520069.13039685
SRR17520069.13039686
SRR17520069.13039687
SRR17520069.13052374
SRR17520069.13052375

SRR17520069.13052376
SRR17520069.13052377
SRR17520069.13052378
SRR17520069.13052379
SRR17520069.13052380
SRR17520069.13052381
SRR17520069.13052382
SRR17520069.13052383
SRR17520069.13052384
SRR17520069.13052385
SRR17520069.13052386
SRR17520069.13052387
SRR17520069.13052388
SRR17520069.13052389
SRR17520069.13052390
SRR17520069.13052391
SRR17520069.13052392
SRR17520069.13052393
SRR17520069.13052394
SRR17520069.13052395
SRR17520069.13052396
SRR17520069.13052397
SRR17520069.13052398
SRR17520069.13052399
SRR17520069.13052400
SRR17520069.13052401
SRR17520069.13052402
SRR17520069.13052404
SRR17520069.13052405
SRR17520069.13052406
SRR17520069.13052407
SRR17520069.13052408
SRR17520069.13052409
SRR17520069.13052410
SRR17520069.13052411
SRR17520069.13052412
SRR17520069.13052414
SRR17520069.13052415
SRR17520069.13052416
SRR17520069.13052417
SRR17520069.13052418
SRR17520069.13052419
SRR17520069.13052420
SRR17520069.13052421
SRR17520069.13052422
SRR17520069.13052423
SRR17520069.13052424

SRR17520069.13052425
SRR17520069.13052426
SRR17520069.13052427
SRR17520069.13052428
SRR17520069.13052429
SRR17520069.13052430
SRR17520069.13052431
SRR17520069.13052432
SRR17520069.13052433
SRR17520069.13052434
SRR17520069.13052435
SRR17520069.13052436
SRR17520069.13052437
SRR17520069.13052438
SRR17520069.13052439
SRR17520069.13052440
SRR17520069.13052441
SRR17520069.13052442
SRR17520069.13052443
SRR17520069.13052444
SRR17520069.13052445
SRR17520069.13052446
SRR17520069.13052447
SRR17520069.13052448
SRR17520069.13052449
SRR17520069.13052450
SRR17520069.13052451
SRR17520069.13052452
SRR17520069.13052453
SRR17520069.13052454
SRR17520069.13052455
SRR17520069.13052456
SRR17520069.13052457
SRR17520069.13052458
SRR17520069.13052459
SRR17520069.13052460
SRR17520069.13052461
SRR17520069.13052462
SRR17520069.13052463
SRR17520069.13052464
SRR17520069.13052465
SRR17520069.13052466
SRR17520069.13052467
SRR17520069.13052468
SRR17520069.13052469
SRR17520069.13052470
SRR17520069.13052471

SRR17520069.13052472
SRR17520069.13052473
SRR17520069.13052474
SRR17520069.13052475
SRR17520069.13052476
SRR17520069.13052477
SRR17520069.13052478
SRR17520069.13052479
SRR17520069.13052480
SRR17520069.13052481
SRR17520069.13052482
SRR17520069.13052483
SRR17520069.13052484
SRR17520069.13052485
SRR17520069.13052486
SRR17520069.13052487
SRR17520069.13052488
SRR17520069.13052489
SRR17520069.13052490
SRR17520069.13052491
SRR17520069.13052492
SRR17520069.13052493
SRR17520069.13052494
SRR17520069.13052495
SRR17520069.13052496
SRR17520069.13052497
SRR17520069.13052498
SRR17520069.13052499
SRR17520069.13052501
SRR17520069.13052502
SRR17520069.13052503
SRR17520069.13052504
SRR17520069.13052505
SRR17520069.13052506
SRR17520069.13052507
SRR17520069.13052508
SRR17520069.13052509
SRR17520069.13052510
SRR17520069.13052511
SRR17520069.13052512
SRR17520069.13052513
SRR17520069.13052514
SRR17520069.13052515
SRR17520069.13052516
SRR17520069.13052517
SRR17520069.13052518
SRR17520069.13052519

SRR17520069.13052521
SRR17520069.13052522
SRR17520069.13052524
SRR17520069.13052525
SRR17520069.13052526
SRR17520069.13052527
SRR17520069.13052528
SRR17520069.13052529
SRR17520069.13052530
SRR17520069.13052531
SRR17520069.13052532
SRR17520069.13052533
SRR17520069.13052534
SRR17520069.13052535
SRR17520069.13052536
SRR17520069.13052537
SRR17520069.13052538
SRR17520069.13052539
SRR17520069.13052540
SRR17520069.13052541
SRR17520069.13052542
SRR17520069.13052543
SRR17520069.13052544
SRR17520069.13052545
SRR17520069.13052546
SRR17520069.13052547
SRR17520069.13052548
SRR17520069.13052549
SRR17520069.13052550
SRR17520069.13052551
SRR17520069.13052552
SRR17520069.13052553
SRR17520069.13052554
SRR17520069.13052555
SRR17520069.13052556
SRR17520069.13052557
SRR17520069.13056791
SRR17520069.13056792
SRR17520069.13056793
SRR17520069.13056794
SRR17520069.13060901
SRR17520069.13060916
SRR17520069.13065693
SRR17520069.13065694
SRR17520069.13065695
SRR17520069.13065696
SRR17520069.13065698

SRR17520069.13065699
SRR17520069.13065700
SRR17520069.13065701
SRR17520069.13065702
SRR17520069.13065703
SRR17520069.13065704
SRR17520069.13065705
SRR17520069.13065706
SRR17520069.13065707
SRR17520069.13065708
SRR17520069.13065709
SRR17520069.13065710
SRR17520069.13065711
SRR17520069.13065712
SRR17520069.13065713
SRR17520069.13065714
SRR17520069.13065715
SRR17520069.13065716
SRR17520069.13065717
SRR17520069.13065718
SRR17520069.13065719
SRR17520069.13065720
SRR17520069.13065721
SRR17520069.13065722
SRR17520069.13065723
SRR17520069.13065724
SRR17520069.13065725
SRR17520069.13065726
SRR17520069.13065727
SRR17520069.13065728
SRR17520069.13065729
SRR17520069.13065730
SRR17520069.13065731
SRR17520069.13065732
SRR17520069.13065733
SRR17520069.13065734
SRR17520069.13065735
SRR17520069.13065736
SRR17520069.13065737
SRR17520069.13065738
SRR17520069.13065739
SRR17520069.13065740
SRR17520069.13065741
SRR17520069.13065742
SRR17520069.13065743
SRR17520069.13065744
SRR17520069.13065745

SRR17520069.13065746
SRR17520069.13065747
SRR17520069.13065748
SRR17520069.13065749
SRR17520069.13065750
SRR17520069.13065751
SRR17520069.13065752
SRR17520069.13065753
SRR17520069.13065756
SRR17520069.13065757
SRR17520069.13065758
SRR17520069.13065759
SRR17520069.13065760
SRR17520069.13065761
SRR17520069.13065762
SRR17520069.13065763
SRR17520069.13065764
SRR17520069.13065765
SRR17520069.13065767
SRR17520069.13065768
SRR17520069.13065769
SRR17520069.13065770
SRR17520069.13065771
SRR17520069.13065772
SRR17520069.13065773
SRR17520069.13065774
SRR17520069.13065775
SRR17520069.13065776
SRR17520069.13065777
SRR17520069.13065778
SRR17520069.13065779
SRR17520069.13065780
SRR17520069.13065781
SRR17520069.13065782
SRR17520069.13065783
SRR17520069.13065784
SRR17520069.13065785
SRR17520069.13065786
SRR17520069.13065787
SRR17520069.13065788
SRR17520069.13065789
SRR17520069.13065790
SRR17520069.13065791
SRR17520069.13065792
SRR17520069.13065793
SRR17520069.13065794
SRR17520069.13065795

SRR17520069.13065796
SRR17520069.13065797
SRR17520069.13065798
SRR17520069.13065799
SRR17520069.13065800
SRR17520069.13065801
SRR17520069.13065802
SRR17520069.13065803
SRR17520069.13065804
SRR17520069.13065805
SRR17520069.13065806
SRR17520069.13065807
SRR17520069.13065808
SRR17520069.13065809
SRR17520069.13065810
SRR17520069.13065811
SRR17520069.13065812
SRR17520069.13065813
SRR17520069.13065814
SRR17520069.13065815
SRR17520069.13065816
SRR17520069.13065817
SRR17520069.13065818
SRR17520069.13065819
SRR17520069.13065820
SRR17520069.13065821
SRR17520069.13065822
SRR17520069.13065823
SRR17520069.13073754
SRR17520069.13086776
SRR17520069.13086777
SRR17520069.13086778
SRR17520069.13086779
SRR17520069.13086780
SRR17520069.13086781
SRR17520069.13086782
SRR17520069.13086783
SRR17520069.13086784
SRR17520069.13086786
SRR17520069.13086787
SRR17520069.13086788
SRR17520069.13086789
SRR17520069.13086790
SRR17520069.13086791
SRR17520069.13086792
SRR17520069.13086793
SRR17520069.13086794

SRR17520069.13086795
SRR17520069.13086796
SRR17520069.13090273
SRR17520069.13090274
SRR17520069.13092631
SRR17520069.13107052
SRR17520069.13112235
SRR17520069.13112236
SRR17520069.13112237
SRR17520069.13112238
SRR17520069.13112239
SRR17520069.13112240
SRR17520069.13112241
SRR17520069.13112242
SRR17520069.13112243
SRR17520069.13112244
SRR17520069.13112245
SRR17520069.13112246
SRR17520069.13112248
SRR17520069.13112249
SRR17520069.13112250
SRR17520069.13112251
SRR17520069.13112252
SRR17520069.13112253
SRR17520069.13112254
SRR17520069.13112255
SRR17520069.13112256
SRR17520069.13112257
SRR17520069.13112258
SRR17520069.13112259
SRR17520069.13112260
SRR17520069.13112261
SRR17520069.13112262
SRR17520069.13112263
SRR17520069.13112264
SRR17520069.13112265
SRR17520069.13112266
SRR17520069.13112267
SRR17520069.13112268
SRR17520069.13112270
SRR17520069.13112271
SRR17520069.13112272
SRR17520069.13112273
SRR17520069.13112275
SRR17520069.13112276
SRR17520069.13112277
SRR17520069.13112278

SRR17520069.13112279
SRR17520069.13112280
SRR17520069.13112281
SRR17520069.13112282
SRR17520069.13112283
SRR17520069.13112285
SRR17520069.13112286
SRR17520069.13112287
SRR17520069.13112288
SRR17520069.13112289
SRR17520069.13112290
SRR17520069.13112291
SRR17520069.13112292
SRR17520069.13112293
SRR17520069.13112294
SRR17520069.13112295
SRR17520069.13112296
SRR17520069.13112297
SRR17520069.13112298
SRR17520069.13112299
SRR17520069.13112300
SRR17520069.13112301
SRR17520069.13112302
SRR17520069.13112303
SRR17520069.13112304
SRR17520069.13112305
SRR17520069.13112306
SRR17520069.13112307
SRR17520069.13112308
SRR17520069.13128645
SRR17520069.13128647
SRR17520069.13128648
SRR17520069.13128649
SRR17520069.13128650
SRR17520069.13128651
SRR17520069.13128652
SRR17520069.13128653
SRR17520069.13128655
SRR17520069.13128657
SRR17520069.13128658
SRR17520069.13128659
SRR17520069.13128660
SRR17520069.13128661
SRR17520069.13128662
SRR17520069.13128663
SRR17520069.13128664
SRR17520069.13128665

SRR17520069.13128667
SRR17520069.13128668
SRR17520069.13128669
SRR17520069.13141144
SRR17520069.13144473
SRR17520069.13144474
SRR17520069.13144475
SRR17520069.13144476
SRR17520069.13144477
SRR17520069.13144479
SRR17520069.13144480
SRR17520069.13144482
SRR17520069.13144483
SRR17520069.13144484
SRR17520069.13144485
SRR17520069.13144486
SRR17520069.13144487
SRR17520069.13144488
SRR17520069.13173167
SRR17520069.13173168
SRR17520069.13173169
SRR17520069.13173170
SRR17520069.13173171
SRR17520069.13173172
SRR17520069.13173173
SRR17520069.13173174
SRR17520069.13173175
SRR17520069.13173177
SRR17520069.13173178
SRR17520069.13173179
SRR17520069.13173180
SRR17520069.13173181
SRR17520069.13173182
SRR17520069.13173183
SRR17520069.13173184
SRR17520069.13173185
SRR17520069.13173186
SRR17520069.13173187
SRR17520069.13173189
SRR17520069.13173190
SRR17520069.13173191
SRR17520069.13173192
SRR17520069.13173193
SRR17520069.13173194
SRR17520069.13173195
SRR17520069.13173196
SRR17520069.13173197

SRR17520069.13173198
SRR17520069.13173199
SRR17520069.13173200
SRR17520069.13173201
SRR17520069.13173202
SRR17520069.13173203
SRR17520069.13173204
SRR17520069.13173205
SRR17520069.13173206
SRR17520069.13173207
SRR17520069.13173208
SRR17520069.13173209
SRR17520069.13173210
SRR17520069.13173211
SRR17520069.13173212
SRR17520069.13173213
SRR17520069.13173214
SRR17520069.13173215
SRR17520069.13173216
SRR17520069.13173217
SRR17520069.13173218
SRR17520069.13173219
SRR17520069.13173220
SRR17520069.13173221
SRR17520069.13173222
SRR17520069.13173223
SRR17520069.13173224
SRR17520069.13173225
SRR17520069.13173226
SRR17520069.13173227
SRR17520069.13173228
SRR17520069.13173229
SRR17520069.13173230
SRR17520069.13173231
SRR17520069.13173232
SRR17520069.13173233
SRR17520069.13173234
SRR17520069.13173235
SRR17520069.13173236
SRR17520069.13173237
SRR17520069.13173238
SRR17520069.13173239
SRR17520069.13173241
SRR17520069.13173242
SRR17520069.13173243
SRR17520069.13173244
SRR17520069.13173245

SRR17520069.13173246
SRR17520069.13173247
SRR17520069.13173248
SRR17520069.13173249
SRR17520069.13173250
SRR17520069.13173251
SRR17520069.13173252
SRR17520069.13173253
SRR17520069.13173254
SRR17520069.13173255
SRR17520069.13173256
SRR17520069.13173257
SRR17520069.13173259
SRR17520069.13173260
SRR17520069.13173261
SRR17520069.13173262
SRR17520069.13173263
SRR17520069.13173264
SRR17520069.13173265
SRR17520069.13173266
SRR17520069.13173268
SRR17520069.13173269
SRR17520069.13173270
SRR17520069.13173271
SRR17520069.13173272
SRR17520069.13173273
SRR17520069.13173274
SRR17520069.13173275
SRR17520069.13173276
SRR17520069.13173277
SRR17520069.13173278
SRR17520069.13173279
SRR17520069.13173280
SRR17520069.13173281
SRR17520069.13173282
SRR17520069.13173283
SRR17520069.13173284
SRR17520069.13173285
SRR17520069.13173286
SRR17520069.13173287
SRR17520069.13173288
SRR17520069.13173289
SRR17520069.13173290
SRR17520069.13173291
SRR17520069.13173292
SRR17520069.13173293
SRR17520069.13173294

SRR17520069.13177682
SRR17520069.13177683
SRR17520069.13177685
SRR17520069.13177686
SRR17520069.13177687
SRR17520069.13177688
SRR17520069.13177689
SRR17520069.13177691
SRR17520069.13177693
SRR17520069.13177695
SRR17520069.13177697
SRR17520069.13177699
SRR17520069.13177701
SRR17520069.13177703
SRR17520069.13177707
SRR17520069.13179625
SRR17520069.13179626
SRR17520069.13179627
SRR17520069.13179628
SRR17520069.13179629
SRR17520069.13179630
SRR17520069.13179631
SRR17520069.13179632
SRR17520069.13179633
SRR17520069.13179634
SRR17520069.13179635
SRR17520069.13179636
SRR17520069.13179637
SRR17520069.13179638
SRR17520069.13179639
SRR17520069.13179640
SRR17520069.13179641
SRR17520069.13179642
SRR17520069.13179643
SRR17520069.13179644
SRR17520069.13179645
SRR17520069.13179646
SRR17520069.13179647
SRR17520069.13179648
SRR17520069.13179649
SRR17520069.13179650
SRR17520069.13179651
SRR17520069.13179652
SRR17520069.13179653
SRR17520069.13179654
SRR17520069.13179655
SRR17520069.13179656

SRR17520069.13179657
SRR17520069.13179658
SRR17520069.13179659
SRR17520069.13179660
SRR17520069.13179661
SRR17520069.13179662
SRR17520069.13179663
SRR17520069.13179664
SRR17520069.13179665
SRR17520069.13179666
SRR17520069.13179667
SRR17520069.13179668
SRR17520069.13179669
SRR17520069.13179670
SRR17520069.13179671
SRR17520069.13179672
SRR17520069.13179673
SRR17520069.13179674
SRR17520069.13179675
SRR17520069.13179676
SRR17520069.13179677
SRR17520069.13179678
SRR17520069.13179679
SRR17520069.13179680
SRR17520069.13179681
SRR17520069.13179682
SRR17520069.13179683
SRR17520069.13179684
SRR17520069.13179686
SRR17520069.13179687
SRR17520069.13179688
SRR17520069.13179689
SRR17520069.13179690
SRR17520069.13179691
SRR17520069.13179692
SRR17520069.13179693
SRR17520069.13179694
SRR17520069.13179695
SRR17520069.13179696
SRR17520069.13179697
SRR17520069.13179698
SRR17520069.13179699
SRR17520069.13179700
SRR17520069.13179701
SRR17520069.13179702
SRR17520069.13179703
SRR17520069.13179704

SRR17520069.13179707
SRR17520069.13179708
SRR17520069.13179709
SRR17520069.13179710
SRR17520069.13179711
SRR17520069.13179712
SRR17520069.13179713
SRR17520069.13179714
SRR17520069.13179715
SRR17520069.13179716
SRR17520069.13179717
SRR17520069.13179718
SRR17520069.13179719
SRR17520069.13179720
SRR17520069.13179721
SRR17520069.13179722
SRR17520069.13179723
SRR17520069.13179724
SRR17520069.13179725
SRR17520069.13179726
SRR17520069.13179727
SRR17520069.13179728
SRR17520069.13179729
SRR17520069.13179730
SRR17520069.13179731
SRR17520069.13179732
SRR17520069.13179733
SRR17520069.13179734
SRR17520069.13179735
SRR17520069.13179736
SRR17520069.13179737
SRR17520069.13179738
SRR17520069.13179739
SRR17520069.13179740
SRR17520069.13179741
SRR17520069.13179742
SRR17520069.13179743
SRR17520069.13179744
SRR17520069.13179745
SRR17520069.13179746
SRR17520069.13179747
SRR17520069.13179748
SRR17520069.13179749
SRR17520069.13179750
SRR17520069.13179751
SRR17520069.13179752
SRR17520069.13179753

SRR17520069.13179755
SRR17520069.13179756
SRR17520069.13179757
SRR17520069.13179758
SRR17520069.13179759
SRR17520069.13179760
SRR17520069.13179761
SRR17520069.13179762
SRR17520069.13179763
SRR17520069.13179764
SRR17520069.13179765
SRR17520069.13179766
SRR17520069.13179767
SRR17520069.13179768
SRR17520069.13179769
SRR17520069.13179770
SRR17520069.13179771
SRR17520069.13179772
SRR17520069.13179773
SRR17520069.13179774
SRR17520069.13179775
SRR17520069.13179776
SRR17520069.13179777
SRR17520069.13179778
SRR17520069.13179779
SRR17520069.13179780
SRR17520069.13179781
SRR17520069.13179782
SRR17520069.13179783
SRR17520069.13179784
SRR17520069.13179785
SRR17520069.13179786
SRR17520069.13179787
SRR17520069.13179788
SRR17520069.13179789
SRR17520069.13179790
SRR17520069.13179791
SRR17520069.13179792
SRR17520069.13179793
SRR17520069.13189906
SRR17520069.13189907
SRR17520069.13189908
SRR17520069.13189909
SRR17520069.13189910
SRR17520069.13189911
SRR17520069.13189912
SRR17520069.13189913

SRR17520069.13189914
SRR17520069.13189915
SRR17520069.13189916
SRR17520069.13189917
SRR17520069.13189918
SRR17520069.13189919
SRR17520069.13189920
SRR17520069.13189921
SRR17520069.13189922
SRR17520069.13189923
SRR17520069.13189924
SRR17520069.13189925
SRR17520069.13189926
SRR17520069.13189927
SRR17520069.13189928
SRR17520069.13189929
SRR17520069.13189930
SRR17520069.13189931
SRR17520069.13189932
SRR17520069.13189933
SRR17520069.13189934
SRR17520069.13189936
SRR17520069.13189937
SRR17520069.13189938
SRR17520069.13189939
SRR17520069.13189940
SRR17520069.13189941
SRR17520069.13189942
SRR17520069.13189943
SRR17520069.13189944
SRR17520069.13189945
SRR17520069.13189946
SRR17520069.13189947
SRR17520069.13189948
SRR17520069.13189949
SRR17520069.13189950
SRR17520069.13189951
SRR17520069.13189952
SRR17520069.13189953
SRR17520069.13189954
SRR17520069.13189955
SRR17520069.13189956
SRR17520069.13189957
SRR17520069.13189958
SRR17520069.13189959
SRR17520069.13189960
SRR17520069.13189961

SRR17520069.13189962
SRR17520069.13189963
SRR17520069.13189964
SRR17520069.13189965
SRR17520069.13189966
SRR17520069.13189967
SRR17520069.13189968
SRR17520069.13189969
SRR17520069.13189970
SRR17520069.13189971
SRR17520069.13189972
SRR17520069.13189973
SRR17520069.13189974
SRR17520069.13189975
SRR17520069.13189976
SRR17520069.13189977
SRR17520069.13189978
SRR17520069.13189979
SRR17520069.13189980
SRR17520069.13189981
SRR17520069.13189982
SRR17520069.13189983
SRR17520069.13189984
SRR17520069.13189985
SRR17520069.13189986
SRR17520069.13189987
SRR17520069.13189988
SRR17520069.13189989
SRR17520069.13189990
SRR17520069.13189991
SRR17520069.13189992
SRR17520069.13189993
SRR17520069.13189994
SRR17520069.13189995
SRR17520069.13189996
SRR17520069.13189997
SRR17520069.13189998
SRR17520069.13189999
SRR17520069.13190001
SRR17520069.13190002
SRR17520069.13190003
SRR17520069.13190004
SRR17520069.13190005
SRR17520069.13190006
SRR17520069.13190007
SRR17520069.13190008
SRR17520069.13190009

SRR17520069.13190010
SRR17520069.13190011
SRR17520069.13190012
SRR17520069.13190013
SRR17520069.13190014
SRR17520069.13190015
SRR17520069.13190016
SRR17520069.13190018
SRR17520069.13190019
SRR17520069.13190020
SRR17520069.13190021
SRR17520069.13213558
SRR17520069.13213560
SRR17520069.13213561
SRR17520069.13213562
SRR17520069.13213563
SRR17520069.13213564
SRR17520069.13213565
SRR17520069.13213566
SRR17520069.13213567
SRR17520069.13213568
SRR17520069.13213569
SRR17520069.13213571
SRR17520069.13213572
SRR17520069.13213573
SRR17520069.13213574
SRR17520069.13213575
SRR17520069.13213576
SRR17520069.13213577
SRR17520069.13213578
SRR17520069.13213580
SRR17520069.13213581
SRR17520069.13213582
SRR17520069.13213583
SRR17520069.13213584
SRR17520069.13213585
SRR17520069.13213586
SRR17520069.13213587
SRR17520069.13213588
SRR17520069.13213589
SRR17520069.13213590
SRR17520069.13213591
SRR17520069.13213592
SRR17520069.13213593
SRR17520069.13213594
SRR17520069.13213595
SRR17520069.13213596

SRR17520069.13213597
SRR17520069.13213598
SRR17520069.13213599
SRR17520069.13213600
SRR17520069.13213601
SRR17520069.13213602
SRR17520069.13213603
SRR17520069.13213604
SRR17520069.13213605
SRR17520069.13213606
SRR17520069.13213607
SRR17520069.13213608
SRR17520069.13213609
SRR17520069.13213610
SRR17520069.13213611
SRR17520069.13213612
SRR17520069.13213613
SRR17520069.13213614
SRR17520069.13213616
SRR17520069.13213617
SRR17520069.13213618
SRR17520069.13213619
SRR17520069.13213620
SRR17520069.13213621
SRR17520069.13213622
SRR17520069.13213623
SRR17520069.13213624
SRR17520069.13213625
SRR17520069.13213626
SRR17520069.13213627
SRR17520069.13213628
SRR17520069.13213629
SRR17520069.13213630
SRR17520069.13213631
SRR17520069.13213632
SRR17520069.13213633
SRR17520069.13213634
SRR17520069.13213635
SRR17520069.13213636
SRR17520069.13213637
SRR17520069.13213638
SRR17520069.13213639
SRR17520069.13213640
SRR17520069.13213641
SRR17520069.13213642
SRR17520069.13213643
SRR17520069.13213644

SRR17520069.13213645
SRR17520069.13213646
SRR17520069.13213647
SRR17520069.13213648
SRR17520069.13213649
SRR17520069.13213650
SRR17520069.13213651
SRR17520069.13213652
SRR17520069.13213653
SRR17520069.13213654
SRR17520069.13213655
SRR17520069.13213656
SRR17520069.13213657
SRR17520069.13213658
SRR17520069.13213660
SRR17520069.13213661
SRR17520069.13213662
SRR17520069.13213663
SRR17520069.13213664
SRR17520069.13213665
SRR17520069.13213666
SRR17520069.13213667
SRR17520069.13213668
SRR17520069.13213669
SRR17520069.13213670
SRR17520069.13233936
SRR17520069.13233999
SRR17520069.13247063
SRR17520069.13261862
SRR17520069.13263594
SRR17520069.13263595
SRR17520069.13263598
SRR17520069.13263599
SRR17520069.13263600
SRR17520069.13263601
SRR17520069.13263602
SRR17520069.13263604
SRR17520069.13263605
SRR17520069.13263606
SRR17520069.13263607
SRR17520069.13263610
SRR17520069.13263611
SRR17520069.13263613
SRR17520069.13263614
SRR17520069.13263615
SRR17520069.13263616
SRR17520069.13263617

SRR17520069.13263618
SRR17520069.13271606
SRR17520069.13271607
SRR17520069.13271608
SRR17520069.13271609
SRR17520069.13271610
SRR17520069.13271611
SRR17520069.13271612
SRR17520069.13271613
SRR17520069.13271614
SRR17520069.13271615
SRR17520069.13271616
SRR17520069.13271617
SRR17520069.13271618
SRR17520069.13271619
SRR17520069.13271620
SRR17520069.13271621
SRR17520069.13271622
SRR17520069.13271623
SRR17520069.13271624
SRR17520069.13271625
SRR17520069.13271626
SRR17520069.13271627
SRR17520069.13271628
SRR17520069.13271629
SRR17520069.13271630
SRR17520069.13271631
SRR17520069.13271632
SRR17520069.13271633
SRR17520069.13271634
SRR17520069.13271635
SRR17520069.13271636
SRR17520069.13271637
SRR17520069.13271638
SRR17520069.13271639
SRR17520069.13271640
SRR17520069.13271641
SRR17520069.13271642
SRR17520069.13271643
SRR17520069.13271644
SRR17520069.13271645
SRR17520069.13271646
SRR17520069.13271647
SRR17520069.13271648
SRR17520069.13271649
SRR17520069.13271650
SRR17520069.13271651

SRR17520069.13271652
SRR17520069.13271653
SRR17520069.13271654
SRR17520069.13271655
SRR17520069.13271656
SRR17520069.13271657
SRR17520069.13271658
SRR17520069.13271659
SRR17520069.13271660
SRR17520069.13271661
SRR17520069.13271667
SRR17520069.13271668
SRR17520069.13278636
SRR17520069.13278783
SRR17520069.13278784
SRR17520069.13278785
SRR17520069.13278786
SRR17520069.13278787
SRR17520069.13278788
SRR17520069.13278789
SRR17520069.13278790
SRR17520069.13278791
SRR17520069.13278792
SRR17520069.13278793
SRR17520069.13278794
SRR17520069.13278795
SRR17520069.13278796
SRR17520069.13278797
SRR17520069.13278798
SRR17520069.13278799
SRR17520069.13278800
SRR17520069.13278801
SRR17520069.13278802
SRR17520069.13278803
SRR17520069.13281409
SRR17520069.13281410
SRR17520069.13281411
SRR17520069.13281412
SRR17520069.13281413
SRR17520069.13281414
SRR17520069.13281415
SRR17520069.13281416
SRR17520069.13282110
SRR17520069.13286242
SRR17520069.13304282
SRR17520069.13307222
SRR17520069.13307223

SRR17520069.13310138
SRR17520069.13310139
SRR17520069.13310140
SRR17520069.13310141
SRR17520069.13310142
SRR17520069.13310143
SRR17520069.13310144
SRR17520069.13310145
SRR17520069.13310147
SRR17520069.13310148
SRR17520069.13310149
SRR17520069.13310150
SRR17520069.13310151
SRR17520069.13310152
SRR17520069.13310153
SRR17520069.13310154
SRR17520069.13310155
SRR17520069.13310156
SRR17520069.13311429
SRR17520069.13317022
SRR17520069.13319545
SRR17520069.13319546
SRR17520069.13319547
SRR17520069.13319548
SRR17520069.13319549
SRR17520069.13336885
SRR17520069.13336886
SRR17520069.13336887
SRR17520069.13336888
SRR17520069.13336889
SRR17520069.13336890
SRR17520069.13336891
SRR17520069.13336892
SRR17520069.13336893
SRR17520069.13336894
SRR17520069.13336895
SRR17520069.13336896
SRR17520069.13336897
SRR17520069.13336898
SRR17520069.13336899
SRR17520069.13336900
SRR17520069.13336901
SRR17520069.13336902
SRR17520069.13336903
SRR17520069.13336904
SRR17520069.13336905
SRR17520069.13336906

SRR17520069.13336907
SRR17520069.13336908
SRR17520069.13336909
SRR17520069.13336910
SRR17520069.13336911
SRR17520069.13336912
SRR17520069.13336913
SRR17520069.13336914
SRR17520069.13336915
SRR17520069.13336916
SRR17520069.13336917
SRR17520069.13336918
SRR17520069.13336919
SRR17520069.13336920
SRR17520069.13336921
SRR17520069.13336922
SRR17520069.13336923
SRR17520069.13336924
SRR17520069.13336925
SRR17520069.13336926
SRR17520069.13336927
SRR17520069.13336928
SRR17520069.13336929
SRR17520069.13336930
SRR17520069.13336931
SRR17520069.13336932
SRR17520069.13336933
SRR17520069.13336934
SRR17520069.13336935
SRR17520069.13336936
SRR17520069.13336937
SRR17520069.13336938
SRR17520069.13336939
SRR17520069.13336940
SRR17520069.13336941
SRR17520069.13336942
SRR17520069.13336943
SRR17520069.13336944
SRR17520069.13336945
SRR17520069.13336946
SRR17520069.13336947
SRR17520069.13336948
SRR17520069.13336949
SRR17520069.13336950
SRR17520069.13336951
SRR17520069.13336952
SRR17520069.13336953

SRR17520069.13336954
SRR17520069.13336955
SRR17520069.13336956
SRR17520069.13336957
SRR17520069.13336958
SRR17520069.13336959
SRR17520069.13336960
SRR17520069.13336961
SRR17520069.13336962
SRR17520069.13336963
SRR17520069.13336964
SRR17520069.13336965
SRR17520069.13336966
SRR17520069.13336967
SRR17520069.13336968
SRR17520069.13336969
SRR17520069.13336970
SRR17520069.13336971
SRR17520069.13336972
SRR17520069.13336973
SRR17520069.13336974
SRR17520069.13336975
SRR17520069.13336976
SRR17520069.13336977
SRR17520069.13336978
SRR17520069.13336979
SRR17520069.13336980
SRR17520069.13336981
SRR17520069.13336982
SRR17520069.13336983
SRR17520069.13336984
SRR17520069.13336985
SRR17520069.13336986
SRR17520069.13336987
SRR17520069.13336988
SRR17520069.13336989
SRR17520069.13336990
SRR17520069.13336991
SRR17520069.13336992
SRR17520069.13336993
SRR17520069.13336994
SRR17520069.13336995
SRR17520069.13336996
SRR17520069.13336997
SRR17520069.13336998
SRR17520069.13336999
SRR17520069.13337000

SRR17520069.13337001
SRR17520069.13337002
SRR17520069.13337003
SRR17520069.13337004
SRR17520069.13337005
SRR17520069.13337006
SRR17520069.13337007
SRR17520069.13337008
SRR17520069.13337009
SRR17520069.13337010
SRR17520069.13337011
SRR17520069.13337012
SRR17520069.13337013
SRR17520069.13337014
SRR17520069.13337015
SRR17520069.13337016
SRR17520069.13337017
SRR17520069.13337018
SRR17520069.13337019
SRR17520069.13337020
SRR17520069.13337021
SRR17520069.13337022
SRR17520069.13337023
SRR17520069.13337024
SRR17520069.13337025
SRR17520069.13337026
SRR17520069.13337027
SRR17520069.13337028
SRR17520069.13337029
SRR17520069.13337030
SRR17520069.13337031
SRR17520069.13337032
SRR17520069.13337033
SRR17520069.13345430
SRR17520069.13345456
SRR17520069.13348625
SRR17520069.13354742
SRR17520069.13354743
SRR17520069.13354744
SRR17520069.13354745
SRR17520069.13354746
SRR17520069.13354747
SRR17520069.13354748
SRR17520069.13354749
SRR17520069.13354750
SRR17520069.13354752
SRR17520069.13354753

SRR17520069.13354754
SRR17520069.13354755
SRR17520069.13354756
SRR17520069.13354757
SRR17520069.13354758
SRR17520069.13354759
SRR17520069.13354760
SRR17520069.13354762
SRR17520069.13354763
SRR17520069.13354764
SRR17520069.13354765
SRR17520069.13354766
SRR17520069.13354767
SRR17520069.13354768
SRR17520069.13354769
SRR17520069.13354770
SRR17520069.13354771
SRR17520069.13354772
SRR17520069.13354773
SRR17520069.13354774
SRR17520069.13354775
SRR17520069.13354776
SRR17520069.13354777
SRR17520069.13354778
SRR17520069.13354779
SRR17520069.13354780
SRR17520069.13354781
SRR17520069.13354782
SRR17520069.13354783
SRR17520069.13354784
SRR17520069.13354785
SRR17520069.13354786
SRR17520069.13354787
SRR17520069.13354788
SRR17520069.13354789
SRR17520069.13354790
SRR17520069.13354791
SRR17520069.13354792
SRR17520069.13354793
SRR17520069.13354794
SRR17520069.13354795
SRR17520069.13354796
SRR17520069.13354797
SRR17520069.13354798
SRR17520069.13354799
SRR17520069.13354800
SRR17520069.13354801

SRR17520069.13354802
SRR17520069.13354803
SRR17520069.13354804
SRR17520069.13354805
SRR17520069.13354806
SRR17520069.13354807
SRR17520069.13354808
SRR17520069.13354809
SRR17520069.13354810
SRR17520069.13354811
SRR17520069.13354812
SRR17520069.13354813
SRR17520069.13354814
SRR17520069.13354815
SRR17520069.13354816
SRR17520069.13354817
SRR17520069.13354818
SRR17520069.13354819
SRR17520069.13354820
SRR17520069.13354821
SRR17520069.13354822
SRR17520069.13354823
SRR17520069.13354824
SRR17520069.13354825
SRR17520069.13354827
SRR17520069.13354828
SRR17520069.13354829
SRR17520069.13354830
SRR17520069.13354831
SRR17520069.13354832
SRR17520069.13354833
SRR17520069.13354834
SRR17520069.13354835
SRR17520069.13354836
SRR17520069.13354837
SRR17520069.13354838
SRR17520069.13354839
SRR17520069.13354840
SRR17520069.13354841
SRR17520069.13390505
SRR17520069.13390506
SRR17520069.13390507
SRR17520069.13390508
SRR17520069.13390509
SRR17520069.13390510
SRR17520069.13390511
SRR17520069.13390512

SRR17520069.13390513
SRR17520069.13390514
SRR17520069.13390515
SRR17520069.13390516
SRR17520069.13390517
SRR17520069.13390518
SRR17520069.13390519
SRR17520069.13390520
SRR17520069.13390521
SRR17520069.13390522
SRR17520069.13390524
SRR17520069.13390526
SRR17520069.13396558
SRR17520069.13400958
SRR17520069.13400997
SRR17520069.13415300
SRR17520069.13415320
SRR17520069.13415338
SRR17520069.13415348
SRR17520069.13429703
SRR17520069.13460758
SRR17520069.13460759
SRR17520069.13460761
SRR17520069.13460762
SRR17520069.13460763
SRR17520069.13460764
SRR17520069.13460765
SRR17520069.13460766
SRR17520069.13460767
SRR17520069.13460768
SRR17520069.13460769
SRR17520069.13463363
SRR17520069.13470160
SRR17520069.13470161
SRR17520069.13470162
SRR17520069.13470163
SRR17520069.13470164
SRR17520069.13470165
SRR17520069.13470166
SRR17520069.13470167
SRR17520069.13470168
SRR17520069.13470169
SRR17520069.13470170
SRR17520069.13470171
SRR17520069.13470172
SRR17520069.13470173
SRR17520069.13470174

SRR17520069.13470175
SRR17520069.13470176
SRR17520069.13470177
SRR17520069.13470179
SRR17520069.13470180
SRR17520069.13470181
SRR17520069.13470182
SRR17520069.13470183
SRR17520069.13470184
SRR17520069.13470185
SRR17520069.13470186
SRR17520069.13470187
SRR17520069.13470188
SRR17520069.13470189
SRR17520069.13470190
SRR17520069.13470191
SRR17520069.13470192
SRR17520069.13470193
SRR17520069.13470194
SRR17520069.13470195
SRR17520069.13470196
SRR17520069.13470197
SRR17520069.13470198
SRR17520069.13470199
SRR17520069.13470200
SRR17520069.13470201
SRR17520069.13470202
SRR17520069.13470203
SRR17520069.13470204
SRR17520069.13470205
SRR17520069.13470206
SRR17520069.13477072
SRR17520069.13478297
SRR17520069.13484070
SRR17520069.13484071
SRR17520069.13484072
SRR17520069.13484074
SRR17520069.13484077
SRR17520069.13484079
SRR17520069.13484082
SRR17520069.13484083
SRR17520069.13484085
SRR17520069.13484086
SRR17520069.13484087
SRR17520069.13484088
SRR17520069.13484089
SRR17520069.13484090

SRR17520069.13484091
SRR17520069.13484092
SRR17520069.13484093
SRR17520069.13484094
SRR17520069.13484095
SRR17520069.13484096
SRR17520069.13484098
SRR17520069.13484099
SRR17520069.13484100
SRR17520069.13484101
SRR17520069.13484102
SRR17520069.13484103
SRR17520069.13484104
SRR17520069.13484105
SRR17520069.13484106
SRR17520069.13484107
SRR17520069.13484108
SRR17520069.13484109
SRR17520069.13484110
SRR17520069.13484111
SRR17520069.13484112
SRR17520069.13484113
SRR17520069.13484114
SRR17520069.13484115
SRR17520069.13484116
SRR17520069.13484117
SRR17520069.13484118
SRR17520069.13484120
SRR17520069.13484121
SRR17520069.13484122
SRR17520069.13486677
SRR17520069.13486678
SRR17520069.13486679
SRR17520069.13486680
SRR17520069.13486681
SRR17520069.13486682
SRR17520069.13486683
SRR17520069.13486684
SRR17520069.13486685
SRR17520069.13486686
SRR17520069.13490856
SRR17520069.13490860
SRR17520069.13490875
SRR17520069.13513573
SRR17520069.13513574
SRR17520069.13513575
SRR17520069.13513576

SRR17520069.13513577
SRR17520069.13513578
SRR17520069.13513579
SRR17520069.13513580
SRR17520069.13513581
SRR17520069.13542035
SRR17520069.13542036
SRR17520069.13542037
SRR17520069.13542038
SRR17520069.13542039
SRR17520069.13542040
SRR17520069.13542041
SRR17520069.13542042
SRR17520069.13542043
SRR17520069.13542044
SRR17520069.13542045
SRR17520069.13542046
SRR17520069.13542047
SRR17520069.13542048
SRR17520069.13542049
SRR17520069.13542050
SRR17520069.13542051
SRR17520069.13542052
SRR17520069.13542053
SRR17520069.13542054
SRR17520069.13542055
SRR17520069.13542056
SRR17520069.13542057
SRR17520069.13542058
SRR17520069.13542059
SRR17520069.13542060
SRR17520069.13542061
SRR17520069.13542062
SRR17520069.13542063
SRR17520069.13542064
SRR17520069.13542065
SRR17520069.13542066
SRR17520069.13542067
SRR17520069.13542068
SRR17520069.13542069
SRR17520069.13542070
SRR17520069.13542071
SRR17520069.13542072
SRR17520069.13542073
SRR17520069.13542074
SRR17520069.13542075
SRR17520069.13542077

SRR17520069.13542078
SRR17520069.13542079
SRR17520069.13542080
SRR17520069.13542081
SRR17520069.13542082
SRR17520069.13542083
SRR17520069.13542084
SRR17520069.13542085
SRR17520069.13542086
SRR17520069.13542087
SRR17520069.13542088
SRR17520069.13542089
SRR17520069.13542090
SRR17520069.13542091
SRR17520069.13542092
SRR17520069.13542093
SRR17520069.13542094
SRR17520069.13542095
SRR17520069.13542096
SRR17520069.13542097
SRR17520069.13542098
SRR17520069.13542099
SRR17520069.13542100
SRR17520069.13542101
SRR17520069.13542102
SRR17520069.13542103
SRR17520069.13542104
SRR17520069.13542105
SRR17520069.13542106
SRR17520069.13542107
SRR17520069.13542108
SRR17520069.13542109
SRR17520069.13542110
SRR17520069.13542111
SRR17520069.13542112
SRR17520069.13542113
SRR17520069.13542114
SRR17520069.13542115
SRR17520069.13542116
SRR17520069.13542117
SRR17520069.13542118
SRR17520069.13542119
SRR17520069.13542120
SRR17520069.13542121
SRR17520069.13542122
SRR17520069.13542123
SRR17520069.13542124

SRR17520069.13542125
SRR17520069.13542126
SRR17520069.13542127
SRR17520069.13542128
SRR17520069.13542129
SRR17520069.13542130
SRR17520069.13542131
SRR17520069.13542133
SRR17520069.13542134
SRR17520069.13542135
SRR17520069.13542136
SRR17520069.13542137
SRR17520069.13542138
SRR17520069.13542139
SRR17520069.13542140
SRR17520069.13542141
SRR17520069.13542142
SRR17520069.13542143
SRR17520069.13542144
SRR17520069.13542145
SRR17520069.13542146
SRR17520069.13542147
SRR17520069.13542148
SRR17520069.13542149
SRR17520069.13542150
SRR17520069.13542151
SRR17520069.13542152
SRR17520069.13542154
SRR17520069.13542155
SRR17520069.13542156
SRR17520069.13542157
SRR17520069.13542158
SRR17520069.13542159
SRR17520069.13542160
SRR17520069.13542161
SRR17520069.13542162
SRR17520069.13542163
SRR17520069.13542164
SRR17520069.13542165
SRR17520069.13542166
SRR17520069.13542167
SRR17520069.13542168
SRR17520069.13542169
SRR17520069.13542170
SRR17520069.13542171
SRR17520069.13542172
SRR17520069.13542173

SRR17520069.13542174
SRR17520069.13542175
SRR17520069.13542176
SRR17520069.13542177
SRR17520069.13542178
SRR17520069.13542179
SRR17520069.13542180
SRR17520069.13542181
SRR17520069.13542182
SRR17520069.13542183
SRR17520069.13542184
SRR17520069.13542185
SRR17520069.13542186
SRR17520069.13542187
SRR17520069.13542188
SRR17520069.13542189
SRR17520069.13542190
SRR17520069.13542191
SRR17520069.13542192
SRR17520069.13542194
SRR17520069.13542198
SRR17520069.13542199
SRR17520069.13542200
SRR17520069.13542201
SRR17520069.13542202
SRR17520069.13542203
SRR17520069.13542204
SRR17520069.13542205
SRR17520069.13542207
SRR17520069.13542208
SRR17520069.13542209
SRR17520069.13542211
SRR17520069.13542212
SRR17520069.13542213
SRR17520069.13542214
SRR17520069.13542215
SRR17520069.13542216
SRR17520069.13542217
SRR17520069.13542218
SRR17520069.13542219
SRR17520069.13542220
SRR17520069.13547118
SRR17520069.13547119
SRR17520069.13547122
SRR17520069.13547123
SRR17520069.13547124
SRR17520069.13547125

SRR17520069.13547126
SRR17520069.13547127
SRR17520069.13547128
SRR17520069.13547131
SRR17520069.13547132
SRR17520069.13547135
SRR17520069.13547137
SRR17520069.13547138
SRR17520069.13547139
SRR17520069.13547140
SRR17520069.13547141
SRR17520069.13547142
SRR17520069.13547143
SRR17520069.13547145
SRR17520069.13547146
SRR17520069.13547147
SRR17520069.13547148
SRR17520069.13547149
SRR17520069.13547150
SRR17520069.13547151
SRR17520069.13547152
SRR17520069.13547153
SRR17520069.13547154
SRR17520069.13547155
SRR17520069.13547156
SRR17520069.13547157
SRR17520069.13547158
SRR17520069.13547159
SRR17520069.13547160
SRR17520069.13547161
SRR17520069.13547162
SRR17520069.13547163
SRR17520069.13547164
SRR17520069.13547165
SRR17520069.13547166
SRR17520069.13547167
SRR17520069.13547168
SRR17520069.13547169
SRR17520069.13547170
SRR17520069.13547171
SRR17520069.13547172
SRR17520069.13547173
SRR17520069.13547175
SRR17520069.13547176
SRR17520069.13547177
SRR17520069.13547179
SRR17520069.13547180

SRR17520069.13547182
SRR17520069.13547183
SRR17520069.13573297
SRR17520069.13581378
SRR17520069.13581379
SRR17520069.13581380
SRR17520069.13581381
SRR17520069.13581382
SRR17520069.13581383
SRR17520069.13581384
SRR17520069.13581385
SRR17520069.13581386
SRR17520069.13581387
SRR17520069.13581388
SRR17520069.13581389
SRR17520069.13581390
SRR17520069.13581391
SRR17520069.13581392
SRR17520069.13581393
SRR17520069.13581394
SRR17520069.13581395
SRR17520069.13581396
SRR17520069.13581397
SRR17520069.13581398
SRR17520069.13581399
SRR17520069.13581400
SRR17520069.13581401
SRR17520069.13581402
SRR17520069.13581403
SRR17520069.13581404
SRR17520069.13581405
SRR17520069.13581406
SRR17520069.13581407
SRR17520069.13581409
SRR17520069.13581410
SRR17520069.13581411
SRR17520069.13581412
SRR17520069.13581413
SRR17520069.13581414
SRR17520069.13581415
SRR17520069.13581416
SRR17520069.13581417
SRR17520069.13581418
SRR17520069.13581419
SRR17520069.13581420
SRR17520069.13581421
SRR17520069.13581422

SRR17520069.13581423
SRR17520069.13581424
SRR17520069.13581425
SRR17520069.13581426
SRR17520069.13581427
SRR17520069.13581428
SRR17520069.13581429
SRR17520069.13581430
SRR17520069.13581431
SRR17520069.13581432
SRR17520069.13581433
SRR17520069.13581434
SRR17520069.13581435
SRR17520069.13581436
SRR17520069.13581437
SRR17520069.13581438
SRR17520069.13581440
SRR17520069.13581441
SRR17520069.13584083
SRR17520069.13584084
SRR17520069.13584090
SRR17520069.13584091
SRR17520069.13584097
SRR17520069.13584098
SRR17520069.13584100
SRR17520069.13584101
SRR17520069.13584110
SRR17520069.13584111
SRR17520069.13584114
SRR17520069.13584115
SRR17520069.13584120
SRR17520069.13584121
SRR17520069.13584122
SRR17520069.13584123
SRR17520069.13584126
SRR17520069.13584127
SRR17520069.13584129
SRR17520069.13584134
SRR17520069.13584135
SRR17520069.13584136
SRR17520069.13584137
SRR17520069.13584138
SRR17520069.13584140
SRR17520069.13584141
SRR17520069.13584143
SRR17520069.13584148
SRR17520069.13584150

SRR17520069.13584154
SRR17520069.13584155
SRR17520069.13584158
SRR17520069.13584162
SRR17520069.13584163
SRR17520069.13584164
SRR17520069.13584165
SRR17520069.13584167
SRR17520069.13584172
SRR17520069.13597093
SRR17520069.13608953
SRR17520069.13618904
SRR17520069.13618905
SRR17520069.13618906
SRR17520069.13618907
SRR17520069.13618908
SRR17520069.13618909
SRR17520069.13618910
SRR17520069.13618911
SRR17520069.13618912
SRR17520069.13618913
SRR17520069.13618914
SRR17520069.13618915
SRR17520069.13618916
SRR17520069.13618917
SRR17520069.13618918
SRR17520069.13618919
SRR17520069.13618920
SRR17520069.13618921
SRR17520069.13618922
SRR17520069.13618923
SRR17520069.13618924
SRR17520069.13618925
SRR17520069.13618926
SRR17520069.13618927
SRR17520069.13618928
SRR17520069.13618929
SRR17520069.13618930
SRR17520069.13618933
SRR17520069.13618934
SRR17520069.13618936
SRR17520069.13618938
SRR17520069.13618939
SRR17520069.13618940
SRR17520069.13618941
SRR17520069.13618942
SRR17520069.13620973

SRR17520069.13620974
SRR17520069.13620975
SRR17520069.13620976
SRR17520069.13620977
SRR17520069.13620978
SRR17520069.13620979
SRR17520069.13620980
SRR17520069.13620981
SRR17520069.13620982
SRR17520069.13620983
SRR17520069.13620984
SRR17520069.13620985
SRR17520069.13620986
SRR17520069.13620987
SRR17520069.13620988
SRR17520069.13620989
SRR17520069.13620990
SRR17520069.13620991
SRR17520069.13620992
SRR17520069.13620993
SRR17520069.13620994
SRR17520069.13620995
SRR17520069.13620996
SRR17520069.13620997
SRR17520069.13620998
SRR17520069.13620999
SRR17520069.13621000
SRR17520069.13621001
SRR17520069.13621002
SRR17520069.13621003
SRR17520069.13621004
SRR17520069.13621005
SRR17520069.13621006
SRR17520069.13621007
SRR17520069.13621008
SRR17520069.13638544
SRR17520069.13638545
SRR17520069.13638546
SRR17520069.13638547
SRR17520069.13638548
SRR17520069.13638549
SRR17520069.13638550
SRR17520069.13638551
SRR17520069.13638552
SRR17520069.13638553
SRR17520069.13638554
SRR17520069.13638555

SRR17520069.13638556
SRR17520069.13638557
SRR17520069.13638558
SRR17520069.13640321
SRR17520069.13640323
SRR17520069.13644423
SRR17520069.13644424
SRR17520069.13644425
SRR17520069.13644426
SRR17520069.13644427
SRR17520069.13644428
SRR17520069.13644429
SRR17520069.13644430
SRR17520069.13644431
SRR17520069.13644432
SRR17520069.13644433
SRR17520069.13644434
SRR17520069.13644435
SRR17520069.13644436
SRR17520069.13644437
SRR17520069.13644438
SRR17520069.13644439
SRR17520069.13644440
SRR17520069.13644441
SRR17520069.13644442
SRR17520069.13644443
SRR17520069.13644444
SRR17520069.13644445
SRR17520069.13644446
SRR17520069.13644447
SRR17520069.13651356
SRR17520069.13651357
SRR17520069.13651358
SRR17520069.13651359
SRR17520069.13651360
SRR17520069.13651361
SRR17520069.13651362
SRR17520069.13651363
SRR17520069.13651364
SRR17520069.13651365
SRR17520069.13651366
SRR17520069.13676095
SRR17520069.13676107
SRR17520069.13676148
SRR17520069.13684397
SRR17520069.13684398
SRR17520069.13684399

SRR17520069.13684400
SRR17520069.13684401
SRR17520069.13684402
SRR17520069.13684403
SRR17520069.13684404
SRR17520069.13684405
SRR17520069.13684406
SRR17520069.13684407
SRR17520069.13684408
SRR17520069.13684409
SRR17520069.13684410
SRR17520069.13684411
SRR17520069.13684412
SRR17520069.13684413
SRR17520069.13684414
SRR17520069.13684415
SRR17520069.13684416
SRR17520069.13684417
SRR17520069.13684418
SRR17520069.13684419
SRR17520069.13684420
SRR17520069.13684421
SRR17520069.13684422
SRR17520069.13684423
SRR17520069.13684424
SRR17520069.13684425
SRR17520069.13684426
SRR17520069.13684427
SRR17520069.13684428
SRR17520069.13684429
SRR17520069.13684430
SRR17520069.13684431
SRR17520069.13684432
SRR17520069.13684433
SRR17520069.13684434
SRR17520069.13684435
SRR17520069.13684436
SRR17520069.13684437
SRR17520069.13684438
SRR17520069.13684439
SRR17520069.13684440
SRR17520069.13684441
SRR17520069.13684442
SRR17520069.13684443
SRR17520069.13684444
SRR17520069.13684445
SRR17520069.13684446

SRR17520069.13684447
SRR17520069.13684448
SRR17520069.13684449
SRR17520069.13684450
SRR17520069.13684451
SRR17520069.13684452
SRR17520069.13684453
SRR17520069.13684454
SRR17520069.13684455
SRR17520069.13684456
SRR17520069.13684457
SRR17520069.13684458
SRR17520069.13684459
SRR17520069.13684460
SRR17520069.13684461
SRR17520069.13684462
SRR17520069.13684463
SRR17520069.13684465
SRR17520069.13684466
SRR17520069.13684467
SRR17520069.13684468
SRR17520069.13684469
SRR17520069.13684470
SRR17520069.13684471
SRR17520069.13684472
SRR17520069.13684473
SRR17520069.13684474
SRR17520069.13684475
SRR17520069.13684476
SRR17520069.13684477
SRR17520069.13684478
SRR17520069.13684479
SRR17520069.13684480
SRR17520069.13684481
SRR17520069.13684482
SRR17520069.13684483
SRR17520069.13684484
SRR17520069.13684485
SRR17520069.13684486
SRR17520069.13684487
SRR17520069.13684488
SRR17520069.13684489
SRR17520069.13684490
SRR17520069.13684491
SRR17520069.13684492
SRR17520069.13684493
SRR17520069.13684495

SRR17520069.13684496
SRR17520069.13684497
SRR17520069.13684498
SRR17520069.13684499
SRR17520069.13684500
SRR17520069.13684501
SRR17520069.13684502
SRR17520069.13684503
SRR17520069.13684504
SRR17520069.13684505
SRR17520069.13684506
SRR17520069.13684507
SRR17520069.13684508
SRR17520069.13684509
SRR17520069.13684510
SRR17520069.13684511
SRR17520069.13684512
SRR17520069.13684513
SRR17520069.13684514
SRR17520069.13684515
SRR17520069.13684516
SRR17520069.13684517
SRR17520069.13684518
SRR17520069.13684519
SRR17520069.13684520
SRR17520069.13684521
SRR17520069.13684522
SRR17520069.13684523
SRR17520069.13684524
SRR17520069.13684525
SRR17520069.13684526
SRR17520069.13684527
SRR17520069.13684528
SRR17520069.13684529
SRR17520069.13684530
SRR17520069.13684531
SRR17520069.13684532
SRR17520069.13684533
SRR17520069.13684534
SRR17520069.13684535
SRR17520069.13684536
SRR17520069.13684537
SRR17520069.13684539
SRR17520069.13684541
SRR17520069.13684542
SRR17520069.13684543
SRR17520069.13684544

SRR17520069.13684545
SRR17520069.13684546
SRR17520069.13684548
SRR17520069.13684549
SRR17520069.13684550
SRR17520069.13684551
SRR17520069.13684552
SRR17520069.13684553
SRR17520069.13684554
SRR17520069.13684555
SRR17520069.13701666
SRR17520069.13701680
SRR17520069.13701688
SRR17520069.13701718
SRR17520069.13721479
SRR17520069.13721480
SRR17520069.13721481
SRR17520069.13721482
SRR17520069.13721483
SRR17520069.13721484
SRR17520069.13721485
SRR17520069.13721486
SRR17520069.13721487
SRR17520069.13721488
SRR17520069.13721489
SRR17520069.13721490
SRR17520069.13721491
SRR17520069.13721492
SRR17520069.13721493
SRR17520069.13721494
SRR17520069.13721495
SRR17520069.13721496
SRR17520069.13721497
SRR17520069.13721498
SRR17520069.13721499
SRR17520069.13721500
SRR17520069.13721501
SRR17520069.13721502
SRR17520069.13721503
SRR17520069.13721504
SRR17520069.13721505
SRR17520069.13721507
SRR17520069.13721508
SRR17520069.13721509
SRR17520069.13721510
SRR17520069.13721511
SRR17520069.13721512

SRR17520069.13721513
SRR17520069.13721514
SRR17520069.13721515
SRR17520069.13721516
SRR17520069.13721517
SRR17520069.13721518
SRR17520069.13721519
SRR17520069.13721520
SRR17520069.13721521
SRR17520069.13721522
SRR17520069.13721523
SRR17520069.13721524
SRR17520069.13721525
SRR17520069.13721526
SRR17520069.13721527
SRR17520069.13721528
SRR17520069.13721529
SRR17520069.13721530
SRR17520069.13721531
SRR17520069.13721532
SRR17520069.13721533
SRR17520069.13721535
SRR17520069.13721536
SRR17520069.13721537
SRR17520069.13721538
SRR17520069.13721539
SRR17520069.13721540
SRR17520069.13721541
SRR17520069.13721542
SRR17520069.13721544
SRR17520069.13721545
SRR17520069.13721546
SRR17520069.13721547
SRR17520069.13721548
SRR17520069.13721549
SRR17520069.13721550
SRR17520069.13721552
SRR17520069.13721553
SRR17520069.13721554
SRR17520069.13721555
SRR17520069.13721556
SRR17520069.13721557
SRR17520069.13721558
SRR17520069.13721559
SRR17520069.13721560
SRR17520069.13721561
SRR17520069.13721562

SRR17520069.13721563
SRR17520069.13721564
SRR17520069.13721565
SRR17520069.13721566
SRR17520069.13721567
SRR17520069.13721568
SRR17520069.13721569
SRR17520069.13721570
SRR17520069.13721571
SRR17520069.13721572
SRR17520069.13721573
SRR17520069.13721574
SRR17520069.13721575
SRR17520069.13721576
SRR17520069.13721577
SRR17520069.13721578
SRR17520069.13721579
SRR17520069.13721580
SRR17520069.13721581
SRR17520069.13721582
SRR17520069.13721583
SRR17520069.13721584
SRR17520069.13721585
SRR17520069.13721586
SRR17520069.13721587
SRR17520069.13721588
SRR17520069.13721589
SRR17520069.13721590
SRR17520069.13721591
SRR17520069.13721592
SRR17520069.13721593
SRR17520069.13721594
SRR17520069.13721595
SRR17520069.13721596
SRR17520069.13721597
SRR17520069.13721598
SRR17520069.13721599
SRR17520069.13721600
SRR17520069.13721601
SRR17520069.13721602
SRR17520069.13721603
SRR17520069.13721604
SRR17520069.13721605
SRR17520069.13721606
SRR17520069.13721607
SRR17520069.13721608
SRR17520069.13721609

SRR17520069.13721610
SRR17520069.13721611
SRR17520069.13721612
SRR17520069.13721613
SRR17520069.13721614
SRR17520069.13721615
SRR17520069.13721616
SRR17520069.13721617
SRR17520069.13721618
SRR17520069.13721619
SRR17520069.13721620
SRR17520069.13721621
SRR17520069.13721622
SRR17520069.13721623
SRR17520069.13721624
SRR17520069.13721625
SRR17520069.13721626
SRR17520069.13721627
SRR17520069.13721628
SRR17520069.13721629
SRR17520069.13721630
SRR17520069.13721631
SRR17520069.13721632
SRR17520069.13721633
SRR17520069.13721634
SRR17520069.13721635
SRR17520069.13721636
SRR17520069.13721637
SRR17520069.13721638
SRR17520069.13721639
SRR17520069.13721640
SRR17520069.13721641
SRR17520069.13721642
SRR17520069.13721643
SRR17520069.13756531
SRR17520069.13756532
SRR17520069.13756533
SRR17520069.13756534
SRR17520069.13756535
SRR17520069.13756536
SRR17520069.13756537
SRR17520069.13756538
SRR17520069.13756539
SRR17520069.13756540
SRR17520069.13756541
SRR17520069.13756542
SRR17520069.13756543

SRR17520069.13756545
SRR17520069.13756546
SRR17520069.13756547
SRR17520069.13756548
SRR17520069.13756549
SRR17520069.13756550
SRR17520069.13756551
SRR17520069.13756552
SRR17520069.13756553
SRR17520069.13756554
SRR17520069.13756555
SRR17520069.13756557
SRR17520069.13756558
SRR17520069.13756559
SRR17520069.13756560
SRR17520069.13756561
SRR17520069.13756562
SRR17520069.13756563
SRR17520069.13756564
SRR17520069.13756565
SRR17520069.13756566
SRR17520069.13756567
SRR17520069.13756568
SRR17520069.13756569
SRR17520069.13756570
SRR17520069.13756571
SRR17520069.13756572
SRR17520069.13756573
SRR17520069.13756574
SRR17520069.13756575
SRR17520069.13756576
SRR17520069.13756577
SRR17520069.13756578
SRR17520069.13756579
SRR17520069.13756580
SRR17520069.13756581
SRR17520069.13756582
SRR17520069.13756586
SRR17520069.13756587
SRR17520069.13756588
SRR17520069.13756589
SRR17520069.13756590
SRR17520069.13756592
SRR17520069.13756593
SRR17520069.13756594
SRR17520069.13756595
SRR17520069.13756596

SRR17520069.13756597
SRR17520069.13756598
SRR17520069.13756599
SRR17520069.13756601
SRR17520069.13756602
SRR17520069.13756603
SRR17520069.13756604
SRR17520069.13756606
SRR17520069.13756607
SRR17520069.13756608
SRR17520069.13756609
SRR17520069.13756610
SRR17520069.13756612
SRR17520069.13756614
SRR17520069.13756615
SRR17520069.13756616
SRR17520069.13756617
SRR17520069.13756618
SRR17520069.13756620
SRR17520069.13756621
SRR17520069.13756622
SRR17520069.13756623
SRR17520069.13756624
SRR17520069.13756625
SRR17520069.13756626
SRR17520069.13756627
SRR17520069.13756628
SRR17520069.13756629
SRR17520069.13756630
SRR17520069.13756632
SRR17520069.13756633
SRR17520069.13756634
SRR17520069.13756635
SRR17520069.13756638
SRR17520069.13756639
SRR17520069.13756641
SRR17520069.13756642
SRR17520069.13756643
SRR17520069.13756645
SRR17520069.13756646
SRR17520069.13756647
SRR17520069.13756648
SRR17520069.13756649
SRR17520069.13756650
SRR17520069.13756651
SRR17520069.13756652
SRR17520069.13756653

SRR17520069.13756655
SRR17520069.13756656
SRR17520069.13756658
SRR17520069.13756659
SRR17520069.13756661
SRR17520069.13756662
SRR17520069.13756663
SRR17520069.13756664
SRR17520069.13756667
SRR17520069.13756668
SRR17520069.13756669
SRR17520069.13756670
SRR17520069.13756671
SRR17520069.13756672
SRR17520069.13756673
SRR17520069.13756674
SRR17520069.13756675
SRR17520069.13756676
SRR17520069.13756677
SRR17520069.13756678
SRR17520069.13756679
SRR17520069.13756680
SRR17520069.13756681
SRR17520069.13756682
SRR17520069.13756683
SRR17520069.13756684
SRR17520069.13756686
SRR17520069.13756687
SRR17520069.13756688
SRR17520069.13756689
SRR17520069.13756690
SRR17520069.13756691
SRR17520069.13756692
SRR17520069.13756693
SRR17520069.13756694
SRR17520069.13756695
SRR17520069.13756696
SRR17520069.13756697
SRR17520069.13756698
SRR17520069.13756699
SRR17520069.13756700
SRR17520069.13756701
SRR17520069.13756702
SRR17520069.13756703
SRR17520069.13756704
SRR17520069.13756705
SRR17520069.13756710

SRR17520069.13756711
SRR17520069.13756712
SRR17520069.13756713
SRR17520069.13756714
SRR17520069.13756716
SRR17520069.13756717
SRR17520069.13756718
SRR17520069.13756719
SRR17520069.13756720
SRR17520069.13756721
SRR17520069.13756722
SRR17520069.13756723
SRR17520069.13756724
SRR17520069.13756725
SRR17520069.13756726
SRR17520069.13756727
SRR17520069.13756728
SRR17520069.13756729
SRR17520069.13756730
SRR17520069.13756731
SRR17520069.13756732
SRR17520069.13756733
SRR17520069.13756734
SRR17520069.13756735
SRR17520069.13756736
SRR17520069.13756737
SRR17520069.13756738
SRR17520069.13756739
SRR17520069.13756740
SRR17520069.13756741
SRR17520069.13756742
SRR17520069.13756743
SRR17520069.13756744
SRR17520069.13760777
SRR17520069.13760778
SRR17520069.13760779
SRR17520069.13760780
SRR17520069.13760781
SRR17520069.13760782
SRR17520069.13760783
SRR17520069.13760784
SRR17520069.13760785
SRR17520069.13760786
SRR17520069.13760787
SRR17520069.13760788
SRR17520069.13760789
SRR17520069.13760790

SRR17520069.13760791
SRR17520069.13760792
SRR17520069.13760793
SRR17520069.13760794
SRR17520069.13760795
SRR17520069.13760797
SRR17520069.13760798
SRR17520069.13760799
SRR17520069.13760800
SRR17520069.13760801
SRR17520069.13760802
SRR17520069.13760803
SRR17520069.13760804
SRR17520069.13760805
SRR17520069.13760806
SRR17520069.13760807
SRR17520069.13760808
SRR17520069.13760809
SRR17520069.13760810
SRR17520069.13760812
SRR17520069.13760813
SRR17520069.13760814
SRR17520069.13760815
SRR17520069.13760816
SRR17520069.13760817
SRR17520069.13760818
SRR17520069.13760819
SRR17520069.13760820
SRR17520069.13760821
SRR17520069.13760822
SRR17520069.13760823
SRR17520069.13760824
SRR17520069.13760825
SRR17520069.13760826
SRR17520069.13760827
SRR17520069.13760828
SRR17520069.13760829
SRR17520069.13760830
SRR17520069.13760831
SRR17520069.13760832
SRR17520069.13760833
SRR17520069.13760834
SRR17520069.13760835
SRR17520069.13760836
SRR17520069.13760837
SRR17520069.13760838
SRR17520069.13760839

SRR17520069.13760840
SRR17520069.13760841
SRR17520069.13760842
SRR17520069.13760843
SRR17520069.13760844
SRR17520069.13760845
SRR17520069.13760846
SRR17520069.13760847
SRR17520069.13760848
SRR17520069.13760849
SRR17520069.13760850
SRR17520069.13760851
SRR17520069.13760852
SRR17520069.13760853
SRR17520069.13760854
SRR17520069.13760856
SRR17520069.13760857
SRR17520069.13760858
SRR17520069.13760859
SRR17520069.13760860
SRR17520069.13760861
SRR17520069.13760862
SRR17520069.13760863
SRR17520069.13760864
SRR17520069.13760865
SRR17520069.13760866
SRR17520069.13760867
SRR17520069.13760868
SRR17520069.13760869
SRR17520069.13760870
SRR17520069.13760871
SRR17520069.13760872
SRR17520069.13760873
SRR17520069.13760874
SRR17520069.13760875
SRR17520069.13760876
SRR17520069.13760877
SRR17520069.13760878
SRR17520069.13760880
SRR17520069.13760881
SRR17520069.13761491
SRR17520069.13772291
SRR17520069.13772292
SRR17520069.13772293
SRR17520069.13772294
SRR17520069.13772295
SRR17520069.13772296

SRR17520069.13772297
SRR17520069.13772298
SRR17520069.13772299
SRR17520069.13772300
SRR17520069.13772301
SRR17520069.13772302
SRR17520069.13772303
SRR17520069.13772304
SRR17520069.13772305
SRR17520069.13772306
SRR17520069.13772307
SRR17520069.13772308
SRR17520069.13772309
SRR17520069.13772310
SRR17520069.13772311
SRR17520069.13772313
SRR17520069.13772314
SRR17520069.13772315
SRR17520069.13772316
SRR17520069.13772317
SRR17520069.13772318
SRR17520069.13772319
SRR17520069.13772320
SRR17520069.13772321
SRR17520069.13772322
SRR17520069.13772323
SRR17520069.13772325
SRR17520069.13772326
SRR17520069.13772327
SRR17520069.13772328
SRR17520069.13772329
SRR17520069.13772330
SRR17520069.13772331
SRR17520069.13772337
SRR17520069.13772338
SRR17520069.13772339
SRR17520069.13772340
SRR17520069.13772341
SRR17520069.13772344
SRR17520069.13772346
SRR17520069.13772347
SRR17520069.13772348
SRR17520069.13772349
SRR17520069.13772350
SRR17520069.13772351
SRR17520069.13772352
SRR17520069.13772353

SRR17520069.13772354
SRR17520069.13772355
SRR17520069.13772356
SRR17520069.13772357
SRR17520069.13772358
SRR17520069.13772359
SRR17520069.13772360
SRR17520069.13772361
SRR17520069.13772362
SRR17520069.13772363
SRR17520069.13772364
SRR17520069.13772365
SRR17520069.13772366
SRR17520069.13772367
SRR17520069.13772368
SRR17520069.13772369
SRR17520069.13772370
SRR17520069.13772371
SRR17520069.13772372
SRR17520069.13772373
SRR17520069.13772374
SRR17520069.13772375
SRR17520069.13772376
SRR17520069.13772377
SRR17520069.13772378
SRR17520069.13772379
SRR17520069.13772380
SRR17520069.13772381
SRR17520069.13772382
SRR17520069.13772383
SRR17520069.13772384
SRR17520069.13772385
SRR17520069.13772386
SRR17520069.13772387
SRR17520069.13772388
SRR17520069.13772389
SRR17520069.13772390
SRR17520069.13772391
SRR17520069.13772392
SRR17520069.13772393
SRR17520069.13772394
SRR17520069.13772395
SRR17520069.13772396
SRR17520069.13772397
SRR17520069.13772398
SRR17520069.13772399
SRR17520069.13772400

SRR17520069.13772401
SRR17520069.13772402
SRR17520069.13772403
SRR17520069.13772404
SRR17520069.13772405
SRR17520069.13772406
SRR17520069.13772407
SRR17520069.13772408
SRR17520069.13772409
SRR17520069.13772410
SRR17520069.13772411
SRR17520069.13772412
SRR17520069.13772413
SRR17520069.13772414
SRR17520069.13772415
SRR17520069.13772416
SRR17520069.13772417
SRR17520069.13772418
SRR17520069.13772419
SRR17520069.13772420
SRR17520069.13772421
SRR17520069.13772422
SRR17520069.13772423
SRR17520069.13772424
SRR17520069.13772425
SRR17520069.13772426
SRR17520069.13772427
SRR17520069.13772428
SRR17520069.13772429
SRR17520069.13772430
SRR17520069.13772431
SRR17520069.13772432
SRR17520069.13772433
SRR17520069.13772434
SRR17520069.13772435
SRR17520069.13772436
SRR17520069.13772437
SRR17520069.13772438
SRR17520069.13772439
SRR17520069.13772440
SRR17520069.13772441
SRR17520069.13772442
SRR17520069.13772443
SRR17520069.13772444
SRR17520069.13772445
SRR17520069.13772446
SRR17520069.13772447

SRR17520069.13772448
SRR17520069.13772450
SRR17520069.13772451
SRR17520069.13772452
SRR17520069.13772453
SRR17520069.13775506
SRR17520069.13775516
SRR17520069.13777124
SRR17520069.13777125
SRR17520069.13777127
SRR17520069.13777439
SRR17520069.13777440
SRR17520069.13777441
SRR17520069.13777442
SRR17520069.13777443
SRR17520069.13777445
SRR17520069.13777446
SRR17520069.13777447
SRR17520069.13777448
SRR17520069.13777449
SRR17520069.13777450
SRR17520069.13777451
SRR17520069.13777452
SRR17520069.13777454
SRR17520069.13777455
SRR17520069.13777456
SRR17520069.13777457
SRR17520069.13777458
SRR17520069.13777459
SRR17520069.13777460
SRR17520069.13777461
SRR17520069.13777462
SRR17520069.13777463
SRR17520069.13777464
SRR17520069.13777465
SRR17520069.13777466
SRR17520069.13777467
SRR17520069.13777468
SRR17520069.13777469
SRR17520069.13777470
SRR17520069.13777471
SRR17520069.13777472
SRR17520069.13777473
SRR17520069.13777474
SRR17520069.13777475
SRR17520069.13777476
SRR17520069.13777477

SRR17520069.13777478
SRR17520069.13777479
SRR17520069.13777480
SRR17520069.13777481
SRR17520069.13777482
SRR17520069.13777483
SRR17520069.13777484
SRR17520069.13777485
SRR17520069.13777486
SRR17520069.13777487
SRR17520069.13777488
SRR17520069.13777489
SRR17520069.13777490
SRR17520069.13777491
SRR17520069.13777492
SRR17520069.13777493
SRR17520069.13777494
SRR17520069.13777495
SRR17520069.13777496
SRR17520069.13777497
SRR17520069.13777498
SRR17520069.13777499
SRR17520069.13777500
SRR17520069.13777501
SRR17520069.13777502
SRR17520069.13778841
SRR17520069.13778842
SRR17520069.13778843
SRR17520069.13778844
SRR17520069.13778845
SRR17520069.13778846
SRR17520069.13778847
SRR17520069.13778848
SRR17520069.13778849
SRR17520069.13778850
SRR17520069.13778852
SRR17520069.13778853
SRR17520069.13778854
SRR17520069.13778855
SRR17520069.13778856
SRR17520069.13778857
SRR17520069.13778858
SRR17520069.13778859
SRR17520069.13778860
SRR17520069.13778861
SRR17520069.13788390
SRR17520069.13788391

SRR17520069.13788392
SRR17520069.13788393
SRR17520069.13788395
SRR17520069.13788396
SRR17520069.13788398
SRR17520069.13788399
SRR17520069.13788400
SRR17520069.13788401
SRR17520069.13788402
SRR17520069.13788403
SRR17520069.13788404
SRR17520069.13788405
SRR17520069.13788406
SRR17520069.13788407
SRR17520069.13788408
SRR17520069.13788410
SRR17520069.13788411
SRR17520069.13788412
SRR17520069.13788413
SRR17520069.13788414
SRR17520069.13788415
SRR17520069.13788416
SRR17520069.13788417
SRR17520069.13788418
SRR17520069.13788419
SRR17520069.13788420
SRR17520069.13788421
SRR17520069.13788422
SRR17520069.13788423
SRR17520069.13788425
SRR17520069.13788426
SRR17520069.13788427
SRR17520069.13788428
SRR17520069.13788429
SRR17520069.13788430
SRR17520069.13788668
SRR17520069.13788669
SRR17520069.13788670
SRR17520069.13788671
SRR17520069.13788672
SRR17520069.13788673
SRR17520069.13788674
SRR17520069.13788675
SRR17520069.13788676
SRR17520069.13788677
SRR17520069.13788678
SRR17520069.13788679

SRR17520069.13788680
SRR17520069.13788681
SRR17520069.13788682
SRR17520069.13788683
SRR17520069.13788684
SRR17520069.13788685
SRR17520069.13788686
SRR17520069.13788687
SRR17520069.13788688
SRR17520069.13788689
SRR17520069.13788690
SRR17520069.13788691
SRR17520069.13788692
SRR17520069.13788693
SRR17520069.13788694
SRR17520069.13788695
SRR17520069.13788696
SRR17520069.13788697
SRR17520069.13788698
SRR17520069.13788699
SRR17520069.13788700
SRR17520069.13788701
SRR17520069.13788702
SRR17520069.13788703
SRR17520069.13788704
SRR17520069.13788705
SRR17520069.13788706
SRR17520069.13788707
SRR17520069.13788708
SRR17520069.13788709
SRR17520069.13788710
SRR17520069.13788711
SRR17520069.13788712
SRR17520069.13788713
SRR17520069.13788714
SRR17520069.13788715
SRR17520069.13801382
SRR17520069.13801929
SRR17520069.13801931
SRR17520069.13801936
SRR17520069.13801937
SRR17520069.13801938
SRR17520069.13801939
SRR17520069.13801940
SRR17520069.13801941
SRR17520069.13801942
SRR17520069.13801943

SRR17520069.13801944
SRR17520069.13801945
SRR17520069.13801946
SRR17520069.13801947
SRR17520069.13801948
SRR17520069.13801949
SRR17520069.13801950
SRR17520069.13801951
SRR17520069.13801952
SRR17520069.13801953
SRR17520069.13801954
SRR17520069.13801955
SRR17520069.13801956
SRR17520069.13801957
SRR17520069.13801958
SRR17520069.13801959
SRR17520069.13801960
SRR17520069.13801961
SRR17520069.13801962
SRR17520069.13801963
SRR17520069.13801964
SRR17520069.13801965
SRR17520069.13801966
SRR17520069.13801967
SRR17520069.13801968
SRR17520069.13801969
SRR17520069.13801971
SRR17520069.13801972
SRR17520069.13801973
SRR17520069.13801974
SRR17520069.13801975
SRR17520069.13801976
SRR17520069.13801977
SRR17520069.13801978
SRR17520069.13801979
SRR17520069.13801980
SRR17520069.13801981
SRR17520069.13801982
SRR17520069.13801983
SRR17520069.13801984
SRR17520069.13801985
SRR17520069.13801986
SRR17520069.13801987
SRR17520069.13801988
SRR17520069.13801989
SRR17520069.13801990
SRR17520069.13801991

SRR17520069.13801992
SRR17520069.13801993
SRR17520069.13801994
SRR17520069.13801995
SRR17520069.13801996
SRR17520069.13801997
SRR17520069.13801998
SRR17520069.13801999
SRR17520069.13802000
SRR17520069.13802001
SRR17520069.13802002
SRR17520069.13802003
SRR17520069.13802004
SRR17520069.13802005
SRR17520069.13802006
SRR17520069.13802007
SRR17520069.13802008
SRR17520069.13802009
SRR17520069.13802010
SRR17520069.13802011
SRR17520069.13802012
SRR17520069.13802013
SRR17520069.13802014
SRR17520069.13802015
SRR17520069.13802017
SRR17520069.13802018
SRR17520069.13802019
SRR17520069.13802020
SRR17520069.13802021
SRR17520069.13802022
SRR17520069.13802023
SRR17520069.13802024
SRR17520069.13802026
SRR17520069.13802028
SRR17520069.13802041
SRR17520069.13802045
SRR17520069.13802060
SRR17520069.13802062
SRR17520069.13802067
SRR17520069.13802076
SRR17520069.13807820
SRR17520069.13807821
SRR17520069.13807822
SRR17520069.13807823
SRR17520069.13807825
SRR17520069.13807826
SRR17520069.13807827

SRR17520069.13807828
SRR17520069.13807829
SRR17520069.13807830
SRR17520069.13807831
SRR17520069.13807832
SRR17520069.13807833
SRR17520069.13807834
SRR17520069.13807835
SRR17520069.13807836
SRR17520069.13807837
SRR17520069.13807838
SRR17520069.13807839
SRR17520069.13807840
SRR17520069.13807841
SRR17520069.13807843
SRR17520069.13807844
SRR17520069.13807845
SRR17520069.13807846
SRR17520069.13807847
SRR17520069.13807848
SRR17520069.13807849
SRR17520069.13807850
SRR17520069.13807851
SRR17520069.13807852
SRR17520069.13807853
SRR17520069.13807854
SRR17520069.13807855
SRR17520069.13807856
SRR17520069.13807858
SRR17520069.13807864
SRR17520069.13807866
SRR17520069.13807869
SRR17520069.13811638
SRR17520069.13811639
SRR17520069.13811640
SRR17520069.13822939
SRR17520069.13822973
SRR17520069.13841740
SRR17520069.13841743
SRR17520069.13843293
SRR17520069.13843294
SRR17520069.13843295
SRR17520069.13843296
SRR17520069.13843297
SRR17520069.13843298
SRR17520069.13843299
SRR17520069.13843300

SRR17520069.13843301
SRR17520069.13843302
SRR17520069.13869765
SRR17520069.13869766
SRR17520069.13869767
SRR17520069.13869768
SRR17520069.13869769
SRR17520069.13869770
SRR17520069.13869771
SRR17520069.13869772
SRR17520069.13869773
SRR17520069.13869774
SRR17520069.13869775
SRR17520069.13869776
SRR17520069.13869777
SRR17520069.13869778
SRR17520069.13869779
SRR17520069.13869780
SRR17520069.13869781
SRR17520069.13869782
SRR17520069.13869783
SRR17520069.13869784
SRR17520069.13869785
SRR17520069.13869786
SRR17520069.13869787
SRR17520069.13869788
SRR17520069.13869789
SRR17520069.13869790
SRR17520069.13869791
SRR17520069.13869792
SRR17520069.13869793
SRR17520069.13869794
SRR17520069.13869795
SRR17520069.13869796
SRR17520069.13869797
SRR17520069.13869798
SRR17520069.13869799
SRR17520069.13869800
SRR17520069.13869801
SRR17520069.13869802
SRR17520069.13869803
SRR17520069.13869804
SRR17520069.13869805
SRR17520069.13869806
SRR17520069.13869807
SRR17520069.13869808
SRR17520069.13869809

SRR17520069.13869810
SRR17520069.13869811
SRR17520069.13869812
SRR17520069.13869813
SRR17520069.13869814
SRR17520069.13869815
SRR17520069.13869816
SRR17520069.13869817
SRR17520069.13869818
SRR17520069.13871232
SRR17520069.13871233
SRR17520069.13871234
SRR17520069.13871235
SRR17520069.13871236
SRR17520069.13871237
SRR17520069.13871238
SRR17520069.13871239
SRR17520069.13871240
SRR17520069.13871241
SRR17520069.13871242
SRR17520069.13871243
SRR17520069.13871245
SRR17520069.13871246
SRR17520069.13871247
SRR17520069.13871248
SRR17520069.13871249
SRR17520069.13871250
SRR17520069.13871251
SRR17520069.13871252
SRR17520069.13871253
SRR17520069.13871254
SRR17520069.13871255
SRR17520069.13871256
SRR17520069.13871257
SRR17520069.13871258
SRR17520069.13871259
SRR17520069.13871260
SRR17520069.13871261
SRR17520069.13871262
SRR17520069.13874865
SRR17520069.13917194
SRR17520069.13917530
SRR17520069.13917531
SRR17520069.13917532
SRR17520069.13917533
SRR17520069.13917534
SRR17520069.13917535

SRR17520069.13917536
SRR17520069.13917537
SRR17520069.13921316
SRR17520069.13921319
SRR17520069.13921320
SRR17520069.13921321
SRR17520069.13921322
SRR17520069.13921323
SRR17520069.13921324
SRR17520069.13921326
SRR17520069.13921327
SRR17520069.13921330
SRR17520069.13921331
SRR17520069.13921334
SRR17520069.13921335
SRR17520069.13921336
SRR17520069.13921337
SRR17520069.13921338
SRR17520069.13921339
SRR17520069.13921340
SRR17520069.13921341
SRR17520069.13921342
SRR17520069.13921343
SRR17520069.13921344
SRR17520069.13921345
SRR17520069.13921346
SRR17520069.13921347
SRR17520069.13921348
SRR17520069.13921349
SRR17520069.13921350
SRR17520069.13921351
SRR17520069.13921352
SRR17520069.13921353
SRR17520069.13921354
SRR17520069.13921355
SRR17520069.13921356
SRR17520069.13921357
SRR17520069.13921358
SRR17520069.13921359
SRR17520069.13921360
SRR17520069.13921361
SRR17520069.13921362
SRR17520069.13921363
SRR17520069.13921364
SRR17520069.13921365
SRR17520069.13921366
SRR17520069.13921367

SRR17520069.13921368
SRR17520069.13921369
SRR17520069.13921370
SRR17520069.13921371
SRR17520069.13921372
SRR17520069.13921373
SRR17520069.13921374
SRR17520069.13921375
SRR17520069.13921376
SRR17520069.13921377
SRR17520069.13921378
SRR17520069.13921379
SRR17520069.13921381
SRR17520069.13921382
SRR17520069.13921383
SRR17520069.13921385
SRR17520069.13921386
SRR17520069.13921387
SRR17520069.13921388
SRR17520069.13921389
SRR17520069.13921390
SRR17520069.13921391
SRR17520069.13921392
SRR17520069.13921393
SRR17520069.13921394
SRR17520069.13921395
SRR17520069.13921396
SRR17520069.13921397
SRR17520069.13921398
SRR17520069.13921399
SRR17520069.13921401
SRR17520069.13921402
SRR17520069.13921403
SRR17520069.13921404
SRR17520069.13921405
SRR17520069.13921406
SRR17520069.13921407
SRR17520069.13944821
SRR17520069.13944822
SRR17520069.13944823
SRR17520069.13944824
SRR17520069.13944825
SRR17520069.13944826
SRR17520069.13944827
SRR17520069.13944828
SRR17520069.13944829
SRR17520069.13944830

SRR17520069.13944831
SRR17520069.13944832
SRR17520069.13944833
SRR17520069.13944834
SRR17520069.13944835
SRR17520069.13944836
SRR17520069.13944837
SRR17520069.13944838
SRR17520069.13944839
SRR17520069.13944840
SRR17520069.13944841
SRR17520069.13944842
SRR17520069.13944843
SRR17520069.13944844
SRR17520069.13944845
SRR17520069.13944846
SRR17520069.13944847
SRR17520069.13944848
SRR17520069.13944849
SRR17520069.13944850
SRR17520069.13944851
SRR17520069.13944852
SRR17520069.13944853
SRR17520069.13944854
SRR17520069.13944855
SRR17520069.13944856
SRR17520069.13944857
SRR17520069.13944858
SRR17520069.13944859
SRR17520069.13944860
SRR17520069.13944861
SRR17520069.13944862
SRR17520069.13944863
SRR17520069.13944864
SRR17520069.13944865
SRR17520069.13944866
SRR17520069.13944867
SRR17520069.13944868
SRR17520069.13944869
SRR17520069.13944870
SRR17520069.13944871
SRR17520069.13944872
SRR17520069.13944873
SRR17520069.13944874
SRR17520069.13944875
SRR17520069.13944876
SRR17520069.13944877

SRR17520069.13944878
SRR17520069.13944879
SRR17520069.13944880
SRR17520069.13944881
SRR17520069.13944882
SRR17520069.13944883
SRR17520069.13944884
SRR17520069.13944885
SRR17520069.13944886
SRR17520069.13944887
SRR17520069.13944888
SRR17520069.13944889
SRR17520069.13944890
SRR17520069.13944891
SRR17520069.13944892
SRR17520069.13944893
SRR17520069.13944894
SRR17520069.13944895
SRR17520069.13944896
SRR17520069.13944897
SRR17520069.13944898
SRR17520069.13944899
SRR17520069.13944900
SRR17520069.13944901
SRR17520069.13944902
SRR17520069.13944903
SRR17520069.13944904
SRR17520069.13944905
SRR17520069.13944906
SRR17520069.13944907
SRR17520069.13944908
SRR17520069.13944909
SRR17520069.13944910
SRR17520069.13944911
SRR17520069.13944912
SRR17520069.13944913
SRR17520069.13944914
SRR17520069.13944915
SRR17520069.13944916
SRR17520069.13944917
SRR17520069.13944918
SRR17520069.13979661
SRR17520069.13979662
SRR17520069.13979663
SRR17520069.13979664
SRR17520069.13979665
SRR17520069.13979666

SRR17520069.13979667
SRR17520069.13979668
SRR17520069.13979669
SRR17520069.13979670
SRR17520069.13979671
SRR17520069.13979672
SRR17520069.14002175
SRR17520069.14003618
SRR17520069.14003619
SRR17520069.14003620
SRR17520069.14003621
SRR17520069.14003622
SRR17520069.14003623
SRR17520069.14003624
SRR17520069.14003625
SRR17520069.14003626
SRR17520069.14003627
SRR17520069.14003628
SRR17520069.14003629
SRR17520069.14003630
SRR17520069.14003631
SRR17520069.14003632
SRR17520069.14003633
SRR17520069.14003634
SRR17520069.14003635
SRR17520069.14003636
SRR17520069.14003637
SRR17520069.14003638
SRR17520069.14003639
SRR17520069.14003640
SRR17520069.14003641
SRR17520069.14003642
SRR17520069.14003643
SRR17520069.14003644
SRR17520069.14003645
SRR17520069.14003646
SRR17520069.14003647
SRR17520069.14003648
SRR17520069.14003649
SRR17520069.14003650
SRR17520069.14003651
SRR17520069.14003652
SRR17520069.14012778
SRR17520069.14012779
SRR17520069.14012780
SRR17520069.14012781
SRR17520069.14012782

SRR17520069.14012783
SRR17520069.14012785
SRR17520069.14012786
SRR17520069.14012787
SRR17520069.14012788
SRR17520069.14013073
SRR17520069.14034335
SRR17520069.14034336
SRR17520069.14034337
SRR17520069.14034338
SRR17520069.14034340
SRR17520069.14034341
SRR17520069.14034342
SRR17520069.14034343
SRR17520069.14034346
SRR17520069.14034348
SRR17520069.14034349
SRR17520069.14034350
SRR17520069.14041775
SRR17520069.14041776
SRR17520069.14041777
SRR17520069.14041778
SRR17520069.14041779
SRR17520069.14041780
SRR17520069.14041781
SRR17520069.14041782
SRR17520069.14041783
SRR17520069.14041784
SRR17520069.14041785
SRR17520069.14041786
SRR17520069.14041787
SRR17520069.14041788
SRR17520069.14041789
SRR17520069.14041790
SRR17520069.14041791
SRR17520069.14041792
SRR17520069.14041794
SRR17520069.14041795
SRR17520069.14041796
SRR17520069.14041797
SRR17520069.14041798
SRR17520069.14041799
SRR17520069.14041800
SRR17520069.14041801
SRR17520069.14041802
SRR17520069.14041803
SRR17520069.14041804

SRR17520069.14041805
SRR17520069.14041806
SRR17520069.14041807
SRR17520069.14041808
SRR17520069.14041809
SRR17520069.14041810
SRR17520069.14041811
SRR17520069.14041812
SRR17520069.14041813
SRR17520069.14041814
SRR17520069.14041815
SRR17520069.14041816
SRR17520069.14041817
SRR17520069.14041818
SRR17520069.14041819
SRR17520069.14041820
SRR17520069.14041821
SRR17520069.14041822
SRR17520069.14041823
SRR17520069.14041824
SRR17520069.14041826
SRR17520069.14041827
SRR17520069.14041828
SRR17520069.14041829
SRR17520069.14041830
SRR17520069.14041831
SRR17520069.14041832
SRR17520069.14041833
SRR17520069.14041834
SRR17520069.14041835
SRR17520069.14041836
SRR17520069.14041837
SRR17520069.14041838
SRR17520069.14041839
SRR17520069.14041840
SRR17520069.14041841
SRR17520069.14041842
SRR17520069.14041843
SRR17520069.14041844
SRR17520069.14041845
SRR17520069.14041846
SRR17520069.14041847
SRR17520069.14041848
SRR17520069.14041849
SRR17520069.14041850
SRR17520069.14041851
SRR17520069.14041852

SRR17520069.14041853
SRR17520069.14041854
SRR17520069.14041855
SRR17520069.14041856
SRR17520069.14041857
SRR17520069.14041858
SRR17520069.14041859
SRR17520069.14041860
SRR17520069.14041861
SRR17520069.14041862
SRR17520069.14041863
SRR17520069.14041864
SRR17520069.14041865
SRR17520069.14041866
SRR17520069.14041867
SRR17520069.14041868
SRR17520069.14041869
SRR17520069.14041870
SRR17520069.14041871
SRR17520069.14041872
SRR17520069.14041873
SRR17520069.14041874
SRR17520069.14041875
SRR17520069.14041876
SRR17520069.14041877
SRR17520069.14041878
SRR17520069.14041879
SRR17520069.14041880
SRR17520069.14041881
SRR17520069.14041882
SRR17520069.14041883
SRR17520069.14041884
SRR17520069.14041885
SRR17520069.14041886
SRR17520069.14041887
SRR17520069.14041888
SRR17520069.14041889
SRR17520069.14041890
SRR17520069.14041891
SRR17520069.14041892
SRR17520069.14041893
SRR17520069.14041894
SRR17520069.14041895
SRR17520069.14041896
SRR17520069.14041897
SRR17520069.14041898
SRR17520069.14041899

SRR17520069.14041900
SRR17520069.14041901
SRR17520069.14041902
SRR17520069.14041903
SRR17520069.14041904
SRR17520069.14041905
SRR17520069.14041906
SRR17520069.14041907
SRR17520069.14041908
SRR17520069.14041909
SRR17520069.14041910
SRR17520069.14041911
SRR17520069.14041912
SRR17520069.14041913
SRR17520069.14041915
SRR17520069.14041916
SRR17520069.14041917
SRR17520069.14041918
SRR17520069.14041919
SRR17520069.14041920
SRR17520069.14041921
SRR17520069.14041922
SRR17520069.14041923
SRR17520069.14041924
SRR17520069.14041925
SRR17520069.14041926
SRR17520069.14041927
SRR17520069.14041928
SRR17520069.14041929
SRR17520069.14041930
SRR17520069.14041931
SRR17520069.14041932
SRR17520069.14041933
SRR17520069.14041934
SRR17520069.14041935
SRR17520069.14041936
SRR17520069.14041937
SRR17520069.14041938
SRR17520069.14041939
SRR17520069.14041940
SRR17520069.14041941
SRR17520069.14041942
SRR17520069.14041943
SRR17520069.14041944
SRR17520069.14041945
SRR17520069.14041946
SRR17520069.14041947

SRR17520069.14041948
SRR17520069.14041949
SRR17520069.14041950
SRR17520069.14041951
SRR17520069.14041952
SRR17520069.14041954
SRR17520069.14041955
SRR17520069.14041956
SRR17520069.14041957
SRR17520069.14041958
SRR17520069.14041959
SRR17520069.14041960
SRR17520069.14041961
SRR17520069.14041962
SRR17520069.14041963
SRR17520069.14041964
SRR17520069.14041965
SRR17520069.14041966
SRR17520069.14041967
SRR17520069.14041968
SRR17520069.14041969
SRR17520069.14041970
SRR17520069.14041971
SRR17520069.14041972
SRR17520069.14041973
SRR17520069.14041974
SRR17520069.14041975
SRR17520069.14041976
SRR17520069.14041977
SRR17520069.14041978
SRR17520069.14041979
SRR17520069.14041980
SRR17520069.14041981
SRR17520069.14041982
SRR17520069.14041983
SRR17520069.14064674
SRR17520069.14064675
SRR17520069.14064676
SRR17520069.14064677
SRR17520069.14064678
SRR17520069.14064679
SRR17520069.14064680
SRR17520069.14064681
SRR17520069.14064682
SRR17520069.14064683
SRR17520069.14064684
SRR17520069.14064685

SRR17520069.14064686
SRR17520069.14064687
SRR17520069.14064688
SRR17520069.14064689
SRR17520069.14064690
SRR17520069.14064691
SRR17520069.14064692
SRR17520069.14064694
SRR17520069.14064695
SRR17520069.14064696
SRR17520069.14064697
SRR17520069.14064698
SRR17520069.14064700
SRR17520069.14064701
SRR17520069.14064702
SRR17520069.14064703
SRR17520069.14064704
SRR17520069.14064705
SRR17520069.14064706
SRR17520069.14064707
SRR17520069.14064708
SRR17520069.14064709
SRR17520069.14064710
SRR17520069.14064711
SRR17520069.14064712
SRR17520069.14064713
SRR17520069.14064714
SRR17520069.14064715
SRR17520069.14064716
SRR17520069.14064717
SRR17520069.14064718
SRR17520069.14064719
SRR17520069.14064720
SRR17520069.14064721
SRR17520069.14070607
SRR17520069.14070663
SRR17520069.14070680
SRR17520069.14070683
SRR17520069.14070689
SRR17520069.14089018
SRR17520069.14089452
SRR17520069.14089453
SRR17520069.14089454
SRR17520069.14089455
SRR17520069.14089456
SRR17520069.14150342
SRR17520069.14150343

SRR17520069.14150344
SRR17520069.14150345
SRR17520069.14150346
SRR17520069.14150347
SRR17520069.14150348
SRR17520069.14150349
SRR17520069.14150350
SRR17520069.14150352
SRR17520069.14150353
SRR17520069.14150354
SRR17520069.14150355
SRR17520069.14150356
SRR17520069.14150357
SRR17520069.14150358
SRR17520069.14150359
SRR17520069.14150360
SRR17520069.14150361
SRR17520069.14150362
SRR17520069.14150363
SRR17520069.14150364
SRR17520069.14150365
SRR17520069.14150366
SRR17520069.14150367
SRR17520069.14150368
SRR17520069.14150369
SRR17520069.14150370
SRR17520069.14150371
SRR17520069.14150372
SRR17520069.14150373
SRR17520069.14150374
SRR17520069.14150375
SRR17520069.14150376
SRR17520069.14150377
SRR17520069.14150378
SRR17520069.14150379
SRR17520069.14150380
SRR17520069.14150381
SRR17520069.14150382
SRR17520069.14201444
SRR17520069.14201445
SRR17520069.14201446
SRR17520069.14201447
SRR17520069.14201448
SRR17520069.14201449
SRR17520069.14201450
SRR17520069.14201451
SRR17520069.14201452

SRR17520069.14201454
SRR17520069.14201455
SRR17520069.14201456
SRR17520069.14201457
SRR17520069.14201458
SRR17520069.14201459
SRR17520069.14201460
SRR17520069.14201461
SRR17520069.14201462
SRR17520069.14201463
SRR17520069.14201464
SRR17520069.14201465
SRR17520069.14201466
SRR17520069.14201467
SRR17520069.14201468
SRR17520069.14201469
SRR17520069.14201470
SRR17520069.14201471
SRR17520069.14201472
SRR17520069.14201473
SRR17520069.14201474
SRR17520069.14201475
SRR17520069.14201476
SRR17520069.14201478
SRR17520069.14201479
SRR17520069.14201480
SRR17520069.14201481
SRR17520069.14201482
SRR17520069.14201483
SRR17520069.14201484
SRR17520069.14201485
SRR17520069.14201486
SRR17520069.14201487
SRR17520069.14201488
SRR17520069.14201489
SRR17520069.14201490
SRR17520069.14201491
SRR17520069.14201492
SRR17520069.14201493
SRR17520069.14201494
SRR17520069.14201495
SRR17520069.14201496
SRR17520069.14201497
SRR17520069.14201498
SRR17520069.14201499
SRR17520069.14201500
SRR17520069.14201501

SRR17520069.14201503
SRR17520069.14201504
SRR17520069.14201510
SRR17520069.14201514
SRR17520069.14201516
SRR17520069.14201519
SRR17520069.14201524
SRR17520069.14201525
SRR17520069.14201527
SRR17520069.14201529
SRR17520069.14201530
SRR17520069.14201537
SRR17520069.14201538
SRR17520069.14201541
SRR17520069.14201551
SRR17520069.14218991
SRR17520069.14218992
SRR17520069.14218993
SRR17520069.14218994
SRR17520069.14218995
SRR17520069.14218996
SRR17520069.14218997
SRR17520069.14218998
SRR17520069.14218999
SRR17520069.14219000
SRR17520069.14219001
SRR17520069.14219002
SRR17520069.14219003
SRR17520069.14219004
SRR17520069.14219005
SRR17520069.14219006
SRR17520069.14219007
SRR17520069.14219008
SRR17520069.14219009
SRR17520069.14219010
SRR17520069.14219011
SRR17520069.14219012
SRR17520069.14233222
SRR17520069.14234911
SRR17520069.14234912
SRR17520069.14234913
SRR17520069.14234914
SRR17520069.14234915
SRR17520069.14234916
SRR17520069.14234917
SRR17520069.14234918
SRR17520069.14234919

SRR17520069.14234920
SRR17520069.14234921
SRR17520069.14234922
SRR17520069.14234923
SRR17520069.14234924
SRR17520069.14234925
SRR17520069.14234926
SRR17520069.14234927
SRR17520069.14234928
SRR17520069.14234929
SRR17520069.14234930
SRR17520069.14234931
SRR17520069.14234932
SRR17520069.14234933
SRR17520069.14234934
SRR17520069.14234935
SRR17520069.14234937
SRR17520069.14234938
SRR17520069.14234939
SRR17520069.14234940
SRR17520069.14234941
SRR17520069.14234942
SRR17520069.14234943
SRR17520069.14234944
SRR17520069.14234945
SRR17520069.14234946
SRR17520069.14234947
SRR17520069.14234948
SRR17520069.14234949
SRR17520069.14234951
SRR17520069.14234952
SRR17520069.14234953
SRR17520069.14234954
SRR17520069.14234955
SRR17520069.14234956
SRR17520069.14234957
SRR17520069.14234958
SRR17520069.14234959
SRR17520069.14234960
SRR17520069.14234961
SRR17520069.14234962
SRR17520069.14234963
SRR17520069.14234964
SRR17520069.14234965
SRR17520069.14234966
SRR17520069.14234967
SRR17520069.14234968

SRR17520069.14234969
SRR17520069.14234970
SRR17520069.14234971
SRR17520069.14234972
SRR17520069.14234973
SRR17520069.14234974
SRR17520069.14234975
SRR17520069.14234976
SRR17520069.14234977
SRR17520069.14234978
SRR17520069.14234979
SRR17520069.14234980
SRR17520069.14234981
SRR17520069.14234982
SRR17520069.14234983
SRR17520069.14234984
SRR17520069.14234986
SRR17520069.14234987
SRR17520069.14234988
SRR17520069.14234989
SRR17520069.14234990
SRR17520069.14234991
SRR17520069.14234992
SRR17520069.14234993
SRR17520069.14234994
SRR17520069.14234995
SRR17520069.14234996
SRR17520069.14234997
SRR17520069.14234998
SRR17520069.14234999
SRR17520069.14235000
SRR17520069.14235001
SRR17520069.14235002
SRR17520069.14235003
SRR17520069.14235004
SRR17520069.14235005
SRR17520069.14235006
SRR17520069.14235007
SRR17520069.14235008
SRR17520069.14235009
SRR17520069.14235010
SRR17520069.14235011
SRR17520069.14235012
SRR17520069.14235013
SRR17520069.14235014
SRR17520069.14235015
SRR17520069.14235016

SRR17520069.14235017
SRR17520069.14235018
SRR17520069.14235019
SRR17520069.14235020
SRR17520069.14235021
SRR17520069.14235022
SRR17520069.14235023
SRR17520069.14235024
SRR17520069.14235025
SRR17520069.14235026
SRR17520069.14235027
SRR17520069.14235028
SRR17520069.14235029
SRR17520069.14235030
SRR17520069.14235031
SRR17520069.14235032
SRR17520069.14235033
SRR17520069.14235034
SRR17520069.14235035
SRR17520069.14235036
SRR17520069.14235037
SRR17520069.14235038
SRR17520069.14235039
SRR17520069.14235040
SRR17520069.14235041
SRR17520069.14235043
SRR17520069.14235044
SRR17520069.14235045
SRR17520069.14235046
SRR17520069.14235047
SRR17520069.14264576
SRR17520069.14264577
SRR17520069.14264578
SRR17520069.14264579
SRR17520069.14264580
SRR17520069.14264581
SRR17520069.14264582
SRR17520069.14264583
SRR17520069.14264584
SRR17520069.14264585
SRR17520069.14264586
SRR17520069.14264587
SRR17520069.14264588
SRR17520069.14264589
SRR17520069.14264590
SRR17520069.14264591
SRR17520069.14264592

SRR17520069.14264593
SRR17520069.14264594
SRR17520069.14264595
SRR17520069.14264596
SRR17520069.14264597
SRR17520069.14264598
SRR17520069.14264599
SRR17520069.14264600
SRR17520069.14264601
SRR17520069.14264602
SRR17520069.14264603
SRR17520069.14264604
SRR17520069.14264605
SRR17520069.14264606
SRR17520069.14264607
SRR17520069.14264608
SRR17520069.14264609
SRR17520069.14264610
SRR17520069.14264611
SRR17520069.14264612
SRR17520069.14264613
SRR17520069.14264614
SRR17520069.14264615
SRR17520069.14264616
SRR17520069.14264617
SRR17520069.14264618
SRR17520069.14264619
SRR17520069.14264620
SRR17520069.14264621
SRR17520069.14264622
SRR17520069.14264623
SRR17520069.14264624
SRR17520069.14264625
SRR17520069.14264626
SRR17520069.14264627
SRR17520069.14264628
SRR17520069.14264629
SRR17520069.14264630
SRR17520069.14264631
SRR17520069.14264632
SRR17520069.14264633
SRR17520069.14264634
SRR17520069.14264635
SRR17520069.14264636
SRR17520069.14264637
SRR17520069.14264638
SRR17520069.14264639

SRR17520069.14264640
SRR17520069.14264641
SRR17520069.14264642
SRR17520069.14264643
SRR17520069.14264644
SRR17520069.14264645
SRR17520069.14264646
SRR17520069.14264647
SRR17520069.14264648
SRR17520069.14264649
SRR17520069.14264650
SRR17520069.14264651
SRR17520069.14264652
SRR17520069.14264653
SRR17520069.14264654
SRR17520069.14264655
SRR17520069.14264656
SRR17520069.14264657
SRR17520069.14264658
SRR17520069.14264659
SRR17520069.14264660
SRR17520069.14264661
SRR17520069.14264662
SRR17520069.14264663
SRR17520069.14264664
SRR17520069.14264665
SRR17520069.14264666
SRR17520069.14264667
SRR17520069.14264668
SRR17520069.14264669
SRR17520069.14264670
SRR17520069.14264671
SRR17520069.14264672
SRR17520069.14264673
SRR17520069.14264674
SRR17520069.14264675
SRR17520069.14264676
SRR17520069.14264677
SRR17520069.14264678
SRR17520069.14264680
SRR17520069.14264681
SRR17520069.14264682
SRR17520069.14264683
SRR17520069.14264684
SRR17520069.14264685
SRR17520069.14264686
SRR17520069.14264687

SRR17520069.14264688
SRR17520069.14264689
SRR17520069.14264690
SRR17520069.14264691
SRR17520069.14264692
SRR17520069.14264693
SRR17520069.14264694
SRR17520069.14264695
SRR17520069.14264696
SRR17520069.14264697
SRR17520069.14264698
SRR17520069.14264699
SRR17520069.14264700
SRR17520069.14267928
SRR17520069.14267929
SRR17520069.14267930
SRR17520069.14267931
SRR17520069.14267932
SRR17520069.14267933
SRR17520069.14267934
SRR17520069.14267935
SRR17520069.14267936
SRR17520069.14267937
SRR17520069.14267938
SRR17520069.14267939
SRR17520069.14267940
SRR17520069.14267941
SRR17520069.14267942
SRR17520069.14267943
SRR17520069.14267944
SRR17520069.14267945
SRR17520069.14267946
SRR17520069.14267949
SRR17520069.14267950
SRR17520069.14267951
SRR17520069.14267952
SRR17520069.14267953
SRR17520069.14267954
SRR17520069.14267955
SRR17520069.14267956
SRR17520069.14267957
SRR17520069.14267958
SRR17520069.14267959
SRR17520069.14267960
SRR17520069.14267961
SRR17520069.14267962
SRR17520069.14267963

SRR17520069.14267964
SRR17520069.14267965
SRR17520069.14267966
SRR17520069.14267967
SRR17520069.14267968
SRR17520069.14267969
SRR17520069.14267970
SRR17520069.14267971
SRR17520069.14267972
SRR17520069.14267973
SRR17520069.14267974
SRR17520069.14267975
SRR17520069.14267976
SRR17520069.14267977
SRR17520069.14267978
SRR17520069.14267979
SRR17520069.14267980
SRR17520069.14267981
SRR17520069.14267982
SRR17520069.14267983
SRR17520069.14267984
SRR17520069.14267985
SRR17520069.14267986
SRR17520069.14267987
SRR17520069.14267988
SRR17520069.14267989
SRR17520069.14267990
SRR17520069.14267991
SRR17520069.14267992
SRR17520069.14267993
SRR17520069.14267994
SRR17520069.14267995
SRR17520069.14267996
SRR17520069.14267997
SRR17520069.14267998
SRR17520069.14267999
SRR17520069.14268000
SRR17520069.14268001
SRR17520069.14268002
SRR17520069.14268003
SRR17520069.14268004
SRR17520069.14268005
SRR17520069.14268006
SRR17520069.14268007
SRR17520069.14268008
SRR17520069.14268009
SRR17520069.14268010

SRR17520069.14268011
SRR17520069.14268012
SRR17520069.14268013
SRR17520069.14268014
SRR17520069.14268015
SRR17520069.14268016
SRR17520069.14268017
SRR17520069.14268018
SRR17520069.14268019
SRR17520069.14268020
SRR17520069.14268021
SRR17520069.14268022
SRR17520069.14268023
SRR17520069.14268024
SRR17520069.14268025
SRR17520069.14268026
SRR17520069.14268027
SRR17520069.14268028
SRR17520069.14268029
SRR17520069.14268030
SRR17520069.14268031
SRR17520069.14268032
SRR17520069.14268033
SRR17520069.14268034
SRR17520069.14268035
SRR17520069.14268036
SRR17520069.14268037
SRR17520069.14268038
SRR17520069.14268039
SRR17520069.14268040
SRR17520069.14268041
SRR17520069.14268042
SRR17520069.14268044
SRR17520069.14268045
SRR17520069.14268046
SRR17520069.14268047
SRR17520069.14268048
SRR17520069.14268049
SRR17520069.14268050
SRR17520069.14268051
SRR17520069.14268052
SRR17520069.14268053
SRR17520069.14268054
SRR17520069.14268055
SRR17520069.14268056
SRR17520069.14273012
SRR17520069.14273013

SRR17520069.14273014
SRR17520069.14273015
SRR17520069.14273016
SRR17520069.14273017
SRR17520069.14273018
SRR17520069.14273019
SRR17520069.14273020
SRR17520069.14273021
SRR17520069.14273022
SRR17520069.14273023
SRR17520069.14273024
SRR17520069.14273025
SRR17520069.14273026
SRR17520069.14273027
SRR17520069.14273028
SRR17520069.14273029
SRR17520069.14273030
SRR17520069.14273031
SRR17520069.14273032
SRR17520069.14273033
SRR17520069.14273034
SRR17520069.14273035
SRR17520069.14273036
SRR17520069.14273037
SRR17520069.14273038
SRR17520069.14273039
SRR17520069.14273040
SRR17520069.14273041
SRR17520069.14284530
SRR17520069.14293220
SRR17520069.14293221
SRR17520069.14293222
SRR17520069.14293223
SRR17520069.14293224
SRR17520069.14293225
SRR17520069.14293226
SRR17520069.14293227
SRR17520069.14293228
SRR17520069.14293229
SRR17520069.14293230
SRR17520069.14293231
SRR17520069.14293232
SRR17520069.14293233
SRR17520069.14293234
SRR17520069.14293235
SRR17520069.14293236
SRR17520069.14293237

SRR17520069.14293238
SRR17520069.14293240
SRR17520069.14293241
SRR17520069.14293242
SRR17520069.14293243
SRR17520069.14293244
SRR17520069.14293245
SRR17520069.14293246
SRR17520069.14293247
SRR17520069.14293248
SRR17520069.14293249
SRR17520069.14293250
SRR17520069.14293251
SRR17520069.14293252
SRR17520069.14293253
SRR17520069.14293254
SRR17520069.14293255
SRR17520069.14293256
SRR17520069.14293257
SRR17520069.14293258
SRR17520069.14293259
SRR17520069.14293260
SRR17520069.14293261
SRR17520069.14293262
SRR17520069.14293263
SRR17520069.14293264
SRR17520069.14293265
SRR17520069.14293266
SRR17520069.14293267
SRR17520069.14293268
SRR17520069.14293269
SRR17520069.14293270
SRR17520069.14293271
SRR17520069.14293272
SRR17520069.14293273
SRR17520069.14293274
SRR17520069.14293275
SRR17520069.14293276
SRR17520069.14293277
SRR17520069.14293278
SRR17520069.14293279
SRR17520069.14293280
SRR17520069.14293281
SRR17520069.14293283
SRR17520069.14293287
SRR17520069.14293292
SRR17520069.14293293

SRR17520069.14293295
SRR17520069.14293297
SRR17520069.14293299
SRR17520069.14293301
SRR17520069.14293304
SRR17520069.14293306
SRR17520069.14293309
SRR17520069.14293310
SRR17520069.14298029
SRR17520069.14306113
SRR17520069.14306114
SRR17520069.14306115
SRR17520069.14306116
SRR17520069.14306117
SRR17520069.14306118
SRR17520069.14306119
SRR17520069.14306120
SRR17520069.14306121
SRR17520069.14306122
SRR17520069.14306123
SRR17520069.14306124
SRR17520069.14306125
SRR17520069.14306126
SRR17520069.14306128
SRR17520069.14306129
SRR17520069.14306130
SRR17520069.14306132
SRR17520069.14306133
SRR17520069.14306134
SRR17520069.14306135
SRR17520069.14306136
SRR17520069.14306137
SRR17520069.14306138
SRR17520069.14306139
SRR17520069.14306140
SRR17520069.14306141
SRR17520069.14306142
SRR17520069.14306143
SRR17520069.14306144
SRR17520069.14306145
SRR17520069.14306146
SRR17520069.14306148
SRR17520069.14306149
SRR17520069.14306150
SRR17520069.14306151
SRR17520069.14306152
SRR17520069.14306153

SRR17520069.14306154
SRR17520069.14306155
SRR17520069.14306156
SRR17520069.14306157
SRR17520069.14306158
SRR17520069.14306159
SRR17520069.14306160
SRR17520069.14306161
SRR17520069.14306162
SRR17520069.14306163
SRR17520069.14306164
SRR17520069.14306165
SRR17520069.14306166
SRR17520069.14306167
SRR17520069.14306168
SRR17520069.14306169
SRR17520069.14306170
SRR17520069.14306171
SRR17520069.14306172
SRR17520069.14306173
SRR17520069.14306174
SRR17520069.14306175
SRR17520069.14306176
SRR17520069.14306177
SRR17520069.14306178
SRR17520069.14306179
SRR17520069.14306180
SRR17520069.14306181
SRR17520069.14306182
SRR17520069.14306183
SRR17520069.14306185
SRR17520069.14306186
SRR17520069.14306187
SRR17520069.14306188
SRR17520069.14306189
SRR17520069.14306190
SRR17520069.14325168
SRR17520069.14325169
SRR17520069.14325171
SRR17520069.14325172
SRR17520069.14325173
SRR17520069.14325174
SRR17520069.14325175
SRR17520069.14325176
SRR17520069.14325177
SRR17520069.14325178
SRR17520069.14325179

SRR17520069.14325180
SRR17520069.14325181
SRR17520069.14325182
SRR17520069.14325183
SRR17520069.14325184
SRR17520069.14325185
SRR17520069.14325186
SRR17520069.14325187
SRR17520069.14325188
SRR17520069.14325189
SRR17520069.14325190
SRR17520069.14327780
SRR17520069.14339348
SRR17520069.14339360
SRR17520069.14340120
SRR17520069.14344179
SRR17520069.14359717
SRR17520069.14359718
SRR17520069.14359719
SRR17520069.14359720
SRR17520069.14359721
SRR17520069.14359722
SRR17520069.14359723
SRR17520069.14359724
SRR17520069.14359725
SRR17520069.14359726
SRR17520069.14359727
SRR17520069.14359728
SRR17520069.14359729
SRR17520069.14359730
SRR17520069.14359731
SRR17520069.14359732
SRR17520069.14359733
SRR17520069.14359734
SRR17520069.14359735
SRR17520069.14359736
SRR17520069.14359737
SRR17520069.14359738
SRR17520069.14359739
SRR17520069.14359740
SRR17520069.14359741
SRR17520069.14359742
SRR17520069.14359743
SRR17520069.14359745
SRR17520069.14359746
SRR17520069.14359747
SRR17520069.14359748

SRR17520069.14359749
SRR17520069.14359750
SRR17520069.14359751
SRR17520069.14359752
SRR17520069.14359753
SRR17520069.14359754
SRR17520069.14359755
SRR17520069.14359756
SRR17520069.14359757
SRR17520069.14359758
SRR17520069.14359759
SRR17520069.14359760
SRR17520069.14359761
SRR17520069.14359762
SRR17520069.14359763
SRR17520069.14359764
SRR17520069.14359765
SRR17520069.14359766
SRR17520069.14359769
SRR17520069.14359770
SRR17520069.14359771
SRR17520069.14359772
SRR17520069.14359773
SRR17520069.14359774
SRR17520069.14359775
SRR17520069.14359776
SRR17520069.14359777
SRR17520069.14359778
SRR17520069.14359779
SRR17520069.14359780
SRR17520069.14359781
SRR17520069.14359782
SRR17520069.14359783
SRR17520069.14359784
SRR17520069.14359785
SRR17520069.14359786
SRR17520069.14359787
SRR17520069.14359788
SRR17520069.14359789
SRR17520069.14359790
SRR17520069.14359791
SRR17520069.14359792
SRR17520069.14359793
SRR17520069.14359794
SRR17520069.14359795
SRR17520069.14359796
SRR17520069.14359797

SRR17520069.14359798
SRR17520069.14359799
SRR17520069.14359800
SRR17520069.14359801
SRR17520069.14359802
SRR17520069.14359803
SRR17520069.14359804
SRR17520069.14359805
SRR17520069.14359806
SRR17520069.14359807
SRR17520069.14359808
SRR17520069.14359809
SRR17520069.14359810
SRR17520069.14359811
SRR17520069.14359812
SRR17520069.14359813
SRR17520069.14359814
SRR17520069.14359815
SRR17520069.14359816
SRR17520069.14359817
SRR17520069.14359818
SRR17520069.14359819
SRR17520069.14359821
SRR17520069.14359822
SRR17520069.14359823
SRR17520069.14359824
SRR17520069.14359825
SRR17520069.14359826
SRR17520069.14359828
SRR17520069.14359829
SRR17520069.14359830
SRR17520069.14359831
SRR17520069.14359832
SRR17520069.14359833
SRR17520069.14359834
SRR17520069.14359835
SRR17520069.14359836
SRR17520069.14359837
SRR17520069.14359838
SRR17520069.14359839
SRR17520069.14359840
SRR17520069.14359841
SRR17520069.14359842
SRR17520069.14359843
SRR17520069.14359844
SRR17520069.14359845
SRR17520069.14359846

SRR17520069.14359847
SRR17520069.14359849
SRR17520069.14359850
SRR17520069.14359851
SRR17520069.14359852
SRR17520069.14359853
SRR17520069.14359854
SRR17520069.14359855
SRR17520069.14359856
SRR17520069.14359857
SRR17520069.14359858
SRR17520069.14359859
SRR17520069.14359860
SRR17520069.14359861
SRR17520069.14359862
SRR17520069.14359863
SRR17520069.14359864
SRR17520069.14359865
SRR17520069.14359866
SRR17520069.14359867
SRR17520069.14359868
SRR17520069.14359869
SRR17520069.14359870
SRR17520069.14359871
SRR17520069.14359872
SRR17520069.14359873
SRR17520069.14359874
SRR17520069.14359875
SRR17520069.14359876
SRR17520069.14359877
SRR17520069.14359878
SRR17520069.14359879
SRR17520069.14359880
SRR17520069.14363227
SRR17520069.14363229
SRR17520069.14365091
SRR17520069.14365092
SRR17520069.14365093
SRR17520069.14365094
SRR17520069.14365095
SRR17520069.14365096
SRR17520069.14365097
SRR17520069.14365098
SRR17520069.14365099
SRR17520069.14365100
SRR17520069.14365101
SRR17520069.14365102

SRR17520069.14365103
SRR17520069.14365104
SRR17520069.14365105
SRR17520069.14365106
SRR17520069.14365107
SRR17520069.14365108
SRR17520069.14365109
SRR17520069.14365110
SRR17520069.14365111
SRR17520069.14365112
SRR17520069.14365113
SRR17520069.14365114
SRR17520069.14365115
SRR17520069.14365116
SRR17520069.14365117
SRR17520069.14365118
SRR17520069.14365697
SRR17520069.14365698
SRR17520069.14365699
SRR17520069.14365700
SRR17520069.14365701
SRR17520069.14365702
SRR17520069.14365703
SRR17520069.14365704
SRR17520069.14365705
SRR17520069.14365706
SRR17520069.14365707
SRR17520069.14365708
SRR17520069.14365709
SRR17520069.14365710
SRR17520069.14365711
SRR17520069.14381706
SRR17520069.14381708
SRR17520069.14381710
SRR17520069.14381711
SRR17520069.14381712
SRR17520069.14381713
SRR17520069.14381714
SRR17520069.14381715
SRR17520069.14381716
SRR17520069.14381717
SRR17520069.14381718
SRR17520069.14381719
SRR17520069.14381720
SRR17520069.14381721
SRR17520069.14381722
SRR17520069.14381723

SRR17520069.14381724
SRR17520069.14381725
SRR17520069.14381726
SRR17520069.14381727
SRR17520069.14381728
SRR17520069.14381729
SRR17520069.14381730
SRR17520069.14381731
SRR17520069.14381732
SRR17520069.14381733
SRR17520069.14381734
SRR17520069.14381735
SRR17520069.14381736
SRR17520069.14381737
SRR17520069.14381738
SRR17520069.14381739
SRR17520069.14381740
SRR17520069.14381741
SRR17520069.14381742
SRR17520069.14381743
SRR17520069.14381744
SRR17520069.14381745
SRR17520069.14381746
SRR17520069.14381747
SRR17520069.14381748
SRR17520069.14381749
SRR17520069.14381750
SRR17520069.14381751
SRR17520069.14381752
SRR17520069.14381753
SRR17520069.14381754
SRR17520069.14381755
SRR17520069.14381756
SRR17520069.14381757
SRR17520069.14381758
SRR17520069.14381760
SRR17520069.14381761
SRR17520069.14381762
SRR17520069.14381763
SRR17520069.14381764
SRR17520069.14381765
SRR17520069.14381766
SRR17520069.14381767
SRR17520069.14381768
SRR17520069.14381769
SRR17520069.14381770
SRR17520069.14381771

SRR17520069.14381772
SRR17520069.14381773
SRR17520069.14381774
SRR17520069.14381775
SRR17520069.14381776
SRR17520069.14381777
SRR17520069.14381778
SRR17520069.14381779
SRR17520069.14381780
SRR17520069.14381781
SRR17520069.14381782
SRR17520069.14381783
SRR17520069.14381784
SRR17520069.14381785
SRR17520069.14381786
SRR17520069.14381787
SRR17520069.14381788
SRR17520069.14381789
SRR17520069.14381790
SRR17520069.14381791
SRR17520069.14381792
SRR17520069.14381793
SRR17520069.14381794
SRR17520069.14381795
SRR17520069.14381796
SRR17520069.14381797
SRR17520069.14381798
SRR17520069.14381799
SRR17520069.14399818
SRR17520069.14399819
SRR17520069.14399820
SRR17520069.14399821
SRR17520069.14399822
SRR17520069.14399823
SRR17520069.14399824
SRR17520069.14399825
SRR17520069.14399827
SRR17520069.14399828
SRR17520069.14399829
SRR17520069.14399830
SRR17520069.14399831
SRR17520069.14399832
SRR17520069.14399833
SRR17520069.14399834
SRR17520069.14399835
SRR17520069.14399836
SRR17520069.14399837

SRR17520069.14399838
SRR17520069.14399839
SRR17520069.14399840
SRR17520069.14399841
SRR17520069.14399842
SRR17520069.14399843
SRR17520069.14399844
SRR17520069.14399845
SRR17520069.14399846
SRR17520069.14399847
SRR17520069.14399848
SRR17520069.14399849
SRR17520069.14399850
SRR17520069.14399851
SRR17520069.14399852
SRR17520069.14399853
SRR17520069.14399854
SRR17520069.14399855
SRR17520069.14399856
SRR17520069.14399857
SRR17520069.14399858
SRR17520069.14399859
SRR17520069.14399860
SRR17520069.14399861
SRR17520069.14399862
SRR17520069.14399863
SRR17520069.14399864
SRR17520069.14399865
SRR17520069.14399866
SRR17520069.14399867
SRR17520069.14399868
SRR17520069.14399869
SRR17520069.14399870
SRR17520069.14399871
SRR17520069.14399872
SRR17520069.14399873
SRR17520069.14399874
SRR17520069.14399875
SRR17520069.14399876
SRR17520069.14399877
SRR17520069.14399878
SRR17520069.14399879
SRR17520069.14399880
SRR17520069.14399881
SRR17520069.14399882
SRR17520069.14399883
SRR17520069.14399884

SRR17520069.14399885
SRR17520069.14399886
SRR17520069.14399887
SRR17520069.14399888
SRR17520069.14399889
SRR17520069.14399890
SRR17520069.14399891
SRR17520069.14399892
SRR17520069.14399894
SRR17520069.14399895
SRR17520069.14399896
SRR17520069.14399897
SRR17520069.14399898
SRR17520069.14399899
SRR17520069.14399900
SRR17520069.14399901
SRR17520069.14399902
SRR17520069.14399903
SRR17520069.14399904
SRR17520069.14399905
SRR17520069.14399906
SRR17520069.14440734
SRR17520069.14440735
SRR17520069.14440736
SRR17520069.14440737
SRR17520069.14440738
SRR17520069.14440739
SRR17520069.14440740
SRR17520069.14440741
SRR17520069.14440742
SRR17520069.14440743
SRR17520069.14440744
SRR17520069.14448596
SRR17520069.14448597
SRR17520069.14448598
SRR17520069.14448599
SRR17520069.14448600
SRR17520069.14448601
SRR17520069.14448602
SRR17520069.14448603
SRR17520069.14448604
SRR17520069.14448605
SRR17520069.14448606
SRR17520069.14448607
SRR17520069.14448608
SRR17520069.14448609
SRR17520069.14448610

SRR17520069.14448611
SRR17520069.14448612
SRR17520069.14448613
SRR17520069.14448614
SRR17520069.14448616
SRR17520069.14448618
SRR17520069.14448620
SRR17520069.14448622
SRR17520069.14448623
SRR17520069.14448624
SRR17520069.14448625
SRR17520069.14448626
SRR17520069.14448627
SRR17520069.14448628
SRR17520069.14448629
SRR17520069.14448630
SRR17520069.14448631
SRR17520069.14448632
SRR17520069.14448633
SRR17520069.14448634
SRR17520069.14448635
SRR17520069.14448636
SRR17520069.14448637
SRR17520069.14448638
SRR17520069.14448639
SRR17520069.14448640
SRR17520069.14448641
SRR17520069.14448642
SRR17520069.14448643
SRR17520069.14448644
SRR17520069.14448645
SRR17520069.14448646
SRR17520069.14448647
SRR17520069.14448648
SRR17520069.14448649
SRR17520069.14448651
SRR17520069.14448652
SRR17520069.14448653
SRR17520069.14448654
SRR17520069.14448655
SRR17520069.14448656
SRR17520069.14448657
SRR17520069.14448658
SRR17520069.14448659
SRR17520069.14448660
SRR17520069.14448661
SRR17520069.14448662

SRR17520069.14448663
SRR17520069.14448664
SRR17520069.14448665
SRR17520069.14448666
SRR17520069.14448667
SRR17520069.14448668
SRR17520069.14448669
SRR17520069.14448670
SRR17520069.14448671
SRR17520069.14448672
SRR17520069.14448673
SRR17520069.14448674
SRR17520069.14448675
SRR17520069.14448676
SRR17520069.14448677
SRR17520069.14448678
SRR17520069.14448679
SRR17520069.14448680
SRR17520069.14448681
SRR17520069.14448682
SRR17520069.14448683
SRR17520069.14448684
SRR17520069.14448685
SRR17520069.14448686
SRR17520069.14448687
SRR17520069.14448688
SRR17520069.14448689
SRR17520069.14448690
SRR17520069.14448691
SRR17520069.14448692
SRR17520069.14448693
SRR17520069.14448694
SRR17520069.14448695
SRR17520069.14448696
SRR17520069.14448697
SRR17520069.14448698
SRR17520069.14448699
SRR17520069.14448700
SRR17520069.14448701
SRR17520069.14448702
SRR17520069.14448703
SRR17520069.14448704
SRR17520069.14448705
SRR17520069.14448706
SRR17520069.14448707
SRR17520069.14448708
SRR17520069.14448709

SRR17520069.14448710
SRR17520069.14448711
SRR17520069.14448712
SRR17520069.14448713
SRR17520069.14448714
SRR17520069.14448715
SRR17520069.14448716
SRR17520069.14448717
SRR17520069.14448718
SRR17520069.14448719
SRR17520069.14448720
SRR17520069.14448721
SRR17520069.14448722
SRR17520069.14448723
SRR17520069.14448724
SRR17520069.14448725
SRR17520069.14448726
SRR17520069.14448727
SRR17520069.14448728
SRR17520069.14448729
SRR17520069.14448730
SRR17520069.14448731
SRR17520069.14448732
SRR17520069.14448733
SRR17520069.14448734
SRR17520069.14448735
SRR17520069.14448736
SRR17520069.14448737
SRR17520069.14448738
SRR17520069.14448739
SRR17520069.14448740
SRR17520069.14448741
SRR17520069.14448742
SRR17520069.14448743
SRR17520069.14448744
SRR17520069.14448745
SRR17520069.14448746
SRR17520069.14448747
SRR17520069.14448748
SRR17520069.14448749
SRR17520069.14448750
SRR17520069.14448751
SRR17520069.14448752
SRR17520069.14448753
SRR17520069.14448754
SRR17520069.14448755
SRR17520069.14448756

SRR17520069.14448757
SRR17520069.14448758
SRR17520069.14448759
SRR17520069.14448761
SRR17520069.14448762
SRR17520069.14448763
SRR17520069.14448764
SRR17520069.14448765
SRR17520069.14448766
SRR17520069.14448767
SRR17520069.14448768
SRR17520069.14448769
SRR17520069.14448770
SRR17520069.14448771
SRR17520069.14448772
SRR17520069.14448773
SRR17520069.14448774
SRR17520069.14448775
SRR17520069.14448776
SRR17520069.14448777
SRR17520069.14448778
SRR17520069.14449139
SRR17520069.14449140
SRR17520069.14449141
SRR17520069.14449144
SRR17520069.14449145
SRR17520069.14449146
SRR17520069.14449148
SRR17520069.14449149
SRR17520069.14449150
SRR17520069.14449151
SRR17520069.14449152
SRR17520069.14449153
SRR17520069.14449154
SRR17520069.14449155
SRR17520069.14449156
SRR17520069.14449157
SRR17520069.14449158
SRR17520069.14449159
SRR17520069.14449160
SRR17520069.14449161
SRR17520069.14449162
SRR17520069.14449163
SRR17520069.14449164
SRR17520069.14449165
SRR17520069.14449166
SRR17520069.14449167

SRR17520069.14449168
SRR17520069.14449169
SRR17520069.14449170
SRR17520069.14449171
SRR17520069.14449172
SRR17520069.14449173
SRR17520069.14449174
SRR17520069.14449175
SRR17520069.14449176
SRR17520069.14449177
SRR17520069.14449178
SRR17520069.14449179
SRR17520069.14449180
SRR17520069.14449181
SRR17520069.14449182
SRR17520069.14449183
SRR17520069.14449184
SRR17520069.14449185
SRR17520069.14449186
SRR17520069.14449187
SRR17520069.14449188
SRR17520069.14449189
SRR17520069.14449190
SRR17520069.14449191
SRR17520069.14451007
SRR17520069.14451008
SRR17520069.14451010
SRR17520069.14451011
SRR17520069.14460213
SRR17520069.14460214
SRR17520069.14460215
SRR17520069.14460216
SRR17520069.14460217
SRR17520069.14460218
SRR17520069.14460219
SRR17520069.14460220
SRR17520069.14460221
SRR17520069.14460222
SRR17520069.14460223
SRR17520069.14460224
SRR17520069.14460225
SRR17520069.14460226
SRR17520069.14460227
SRR17520069.14460228
SRR17520069.14460229
SRR17520069.14460230
SRR17520069.14460231

SRR17520069.14460232
SRR17520069.14460233
SRR17520069.14460234
SRR17520069.14460235
SRR17520069.14460236
SRR17520069.14460237
SRR17520069.14460239
SRR17520069.14460240
SRR17520069.14460241
SRR17520069.14460242
SRR17520069.14460243
SRR17520069.14460244
SRR17520069.14460245
SRR17520069.14460246
SRR17520069.14460247
SRR17520069.14460248
SRR17520069.14460249
SRR17520069.14460250
SRR17520069.14460251
SRR17520069.14460252
SRR17520069.14460253
SRR17520069.14460254
SRR17520069.14460255
SRR17520069.14460256
SRR17520069.14460257
SRR17520069.14460258
SRR17520069.14460259
SRR17520069.14460260
SRR17520069.14460261
SRR17520069.14460262
SRR17520069.14460263
SRR17520069.14460264
SRR17520069.14460265
SRR17520069.14460266
SRR17520069.14460267
SRR17520069.14460268
SRR17520069.14460269
SRR17520069.14460270
SRR17520069.14460271
SRR17520069.14460272
SRR17520069.14460273
SRR17520069.14460274
SRR17520069.14460276
SRR17520069.14460277
SRR17520069.14460278
SRR17520069.14460279
SRR17520069.14460280

SRR17520069.14460281
SRR17520069.14511414
SRR17520069.14511415
SRR17520069.14511416
SRR17520069.14511417
SRR17520069.14511418
SRR17520069.14511419
SRR17520069.14511420
SRR17520069.14511421
SRR17520069.14511422
SRR17520069.14511423
SRR17520069.14511424
SRR17520069.14511425
SRR17520069.14511426
SRR17520069.14511428
SRR17520069.14532725
SRR17520069.14532726
SRR17520069.14532727
SRR17520069.14532728
SRR17520069.14532729
SRR17520069.14532730
SRR17520069.14532731
SRR17520069.14532732
SRR17520069.14532733
SRR17520069.14532735
SRR17520069.14532736
SRR17520069.14532737
SRR17520069.14532738
SRR17520069.14532739
SRR17520069.14532740
SRR17520069.14532741
SRR17520069.14532742
SRR17520069.14532743
SRR17520069.14532744
SRR17520069.14532745
SRR17520069.14532746
SRR17520069.14532747
SRR17520069.14532749
SRR17520069.14532750
SRR17520069.14532751
SRR17520069.14532752
SRR17520069.14532753
SRR17520069.14532754
SRR17520069.14532755
SRR17520069.14532756
SRR17520069.14532757
SRR17520069.14532758

SRR17520069.14532759
SRR17520069.14532762
SRR17520069.14532763
SRR17520069.14532764
SRR17520069.14532765
SRR17520069.14532766
SRR17520069.14532767
SRR17520069.14532768
SRR17520069.14532769
SRR17520069.14532770
SRR17520069.14532771
SRR17520069.14532772
SRR17520069.14532773
SRR17520069.14532774
SRR17520069.14532775
SRR17520069.14532776
SRR17520069.14532777
SRR17520069.14532778
SRR17520069.14532779
SRR17520069.14532780
SRR17520069.14532781
SRR17520069.14532782
SRR17520069.14532783
SRR17520069.14532784
SRR17520069.14532785
SRR17520069.14532786
SRR17520069.14532787
SRR17520069.14532788
SRR17520069.14532789
SRR17520069.14532790
SRR17520069.14532791
SRR17520069.14532792
SRR17520069.14532793
SRR17520069.14532794
SRR17520069.14532795
SRR17520069.14532796
SRR17520069.14532797
SRR17520069.14532798
SRR17520069.14532799
SRR17520069.14532800
SRR17520069.14532801
SRR17520069.14532802
SRR17520069.14532803
SRR17520069.14532804
SRR17520069.14532805
SRR17520069.14532806
SRR17520069.14532807

SRR17520069.14532808
SRR17520069.14532809
SRR17520069.14532810
SRR17520069.14532811
SRR17520069.14532812
SRR17520069.14532813
SRR17520069.14532814
SRR17520069.14532815
SRR17520069.14532816
SRR17520069.14532817
SRR17520069.14532818
SRR17520069.14532819
SRR17520069.14532820
SRR17520069.14532821
SRR17520069.14532822
SRR17520069.14532823
SRR17520069.14532824
SRR17520069.14532825
SRR17520069.14532826
SRR17520069.14532827
SRR17520069.14532828
SRR17520069.14532829
SRR17520069.14532830
SRR17520069.14532831
SRR17520069.14532832
SRR17520069.14532833
SRR17520069.14532834
SRR17520069.14532835
SRR17520069.14532836
SRR17520069.14532837
SRR17520069.14532838
SRR17520069.14532839
SRR17520069.14532840
SRR17520069.14532841
SRR17520069.14532842
SRR17520069.14532843
SRR17520069.14532844
SRR17520069.14532845
SRR17520069.14532846
SRR17520069.14532847
SRR17520069.14532848
SRR17520069.14532849
SRR17520069.14532850
SRR17520069.14532851
SRR17520069.14532852
SRR17520069.14532853
SRR17520069.14532854

SRR17520069.14532855
SRR17520069.14532856
SRR17520069.14532857
SRR17520069.14532858
SRR17520069.14532859
SRR17520069.14532860
SRR17520069.14532861
SRR17520069.14532862
SRR17520069.14532863
SRR17520069.14532864
SRR17520069.14532865
SRR17520069.14532866
SRR17520069.14532867
SRR17520069.14532868
SRR17520069.14532869
SRR17520069.14532870
SRR17520069.14540140
SRR17520069.14540142
SRR17520069.14540143
SRR17520069.14540144
SRR17520069.14540145
SRR17520069.14540146
SRR17520069.14540147
SRR17520069.14540148
SRR17520069.14540149
SRR17520069.14540150
SRR17520069.14540153
SRR17520069.14540154
SRR17520069.14540155
SRR17520069.14540156
SRR17520069.14540157
SRR17520069.14540158
SRR17520069.14540159
SRR17520069.14540160
SRR17520069.14540161
SRR17520069.14540162
SRR17520069.14540163
SRR17520069.14540164
SRR17520069.14540166
SRR17520069.14540167
SRR17520069.14540168
SRR17520069.14540169
SRR17520069.14540170
SRR17520069.14540171
SRR17520069.14540172
SRR17520069.14540173
SRR17520069.14540174

SRR17520069.14540175
SRR17520069.14540176
SRR17520069.14540177
SRR17520069.14540178
SRR17520069.14540179
SRR17520069.14540180
SRR17520069.14540181
SRR17520069.14540182
SRR17520069.14540183
SRR17520069.14540184
SRR17520069.14540186
SRR17520069.14540187
SRR17520069.14540188
SRR17520069.14540189
SRR17520069.14540190
SRR17520069.14540191
SRR17520069.14540192
SRR17520069.14540194
SRR17520069.14540195
SRR17520069.14540196
SRR17520069.14540197
SRR17520069.14540198
SRR17520069.14540199
SRR17520069.14540200
SRR17520069.14540202
SRR17520069.14540203
SRR17520069.14540204
SRR17520069.14540205
SRR17520069.14540206
SRR17520069.14540207
SRR17520069.14540208
SRR17520069.14540209
SRR17520069.14540210
SRR17520069.14540211
SRR17520069.14540212
SRR17520069.14540213
SRR17520069.14540214
SRR17520069.14540215
SRR17520069.14540216
SRR17520069.14540217
SRR17520069.14540218
SRR17520069.14540219
SRR17520069.14540220
SRR17520069.14540221
SRR17520069.14540222
SRR17520069.14540223
SRR17520069.14540224

SRR17520069.14540225
SRR17520069.14540226
SRR17520069.14540227
SRR17520069.14540228
SRR17520069.14540229
SRR17520069.14540230
SRR17520069.14540231
SRR17520069.14540232
SRR17520069.14540233
SRR17520069.14540234
SRR17520069.14540236
SRR17520069.14540237
SRR17520069.14540238
SRR17520069.14540239
SRR17520069.14540240
SRR17520069.14540241
SRR17520069.14540242
SRR17520069.14540243
SRR17520069.14540244
SRR17520069.14540245
SRR17520069.14540246
SRR17520069.14540248
SRR17520069.14540249
SRR17520069.14540250
SRR17520069.14540251
SRR17520069.14540252
SRR17520069.14540253
SRR17520069.14540254
SRR17520069.14540256
SRR17520069.14540257
SRR17520069.14540258
SRR17520069.14540259
SRR17520069.14540260
SRR17520069.14540261
SRR17520069.14540262
SRR17520069.14540263
SRR17520069.14540264
SRR17520069.14540265
SRR17520069.14540266
SRR17520069.14540267
SRR17520069.14540268
SRR17520069.14540269
SRR17520069.14550020
SRR17520069.14588462
SRR17520069.14588731
SRR17520069.14588732
SRR17520069.14594159

SRR17520069.14594160
SRR17520069.14594161
SRR17520069.14594162
SRR17520069.14594163
SRR17520069.14594164
SRR17520069.14594165
SRR17520069.14599034
SRR17520069.14599035
SRR17520069.14599036
SRR17520069.14599037
SRR17520069.14599038
SRR17520069.14599039
SRR17520069.14599040
SRR17520069.14599041
SRR17520069.14599042
SRR17520069.14599900
SRR17520069.14599910
SRR17520069.14616228
SRR17520069.14622925
SRR17520069.14657900
SRR17520069.14657901
SRR17520069.14657902
SRR17520069.14657903
SRR17520069.14657904
SRR17520069.14657905
SRR17520069.14657906
SRR17520069.14657907
SRR17520069.14657908
SRR17520069.14657909
SRR17520069.14657910
SRR17520069.14657911
SRR17520069.14663912
SRR17520069.14686203
SRR17520069.14735431
SRR17520069.14764115
SRR17520069.14764116
SRR17520069.14764117
SRR17520069.14764118
SRR17520069.14764119
SRR17520069.14764120
SRR17520069.14764121
SRR17520069.14764122
SRR17520069.14764124
SRR17520069.14764125
SRR17520069.14764126
SRR17520069.14764127
SRR17520069.14764128

SRR17520069.14764129
SRR17520069.14764130
SRR17520069.14764131
SRR17520069.14764132
SRR17520069.14764133
SRR17520069.14764134
SRR17520069.14764135
SRR17520069.14764136
SRR17520069.14764137
SRR17520069.14764139
SRR17520069.14764140
SRR17520069.14764141
SRR17520069.14764142
SRR17520069.14764143
SRR17520069.14764144
SRR17520069.14764145
SRR17520069.14764146
SRR17520069.14764147
SRR17520069.14764148
SRR17520069.14764149
SRR17520069.14764150
SRR17520069.14764151
SRR17520069.14764152
SRR17520069.14764153
SRR17520069.14764154
SRR17520069.14764155
SRR17520069.14764156
SRR17520069.14764157
SRR17520069.14764158
SRR17520069.14764159
SRR17520069.14764160
SRR17520069.14764161
SRR17520069.14764162
SRR17520069.14764163
SRR17520069.14764164
SRR17520069.14764165
SRR17520069.14764166
SRR17520069.14764167
SRR17520069.14764168
SRR17520069.14764169
SRR17520069.14764170
SRR17520069.14764171
SRR17520069.14764172
SRR17520069.14764173
SRR17520069.14764174
SRR17520069.14764175
SRR17520069.14764176

SRR17520069.14764177
SRR17520069.14764178
SRR17520069.14764179
SRR17520069.14764180
SRR17520069.14764181
SRR17520069.14764182
SRR17520069.14764183
SRR17520069.14764184
SRR17520069.14764185
SRR17520069.14764186
SRR17520069.14764187
SRR17520069.14764188
SRR17520069.14764189
SRR17520069.14764190
SRR17520069.14764191
SRR17520069.14764192
SRR17520069.14764193
SRR17520069.14764194
SRR17520069.14764195
SRR17520069.14764196
SRR17520069.14764197
SRR17520069.14764198
SRR17520069.14764199
SRR17520069.14764200
SRR17520069.14764201
SRR17520069.14764202
SRR17520069.14764203
SRR17520069.14764204
SRR17520069.14764205
SRR17520069.14764207
SRR17520069.14764208
SRR17520069.14764209
SRR17520069.14764210
SRR17520069.14764211
SRR17520069.14764212
SRR17520069.14764213
SRR17520069.14764214
SRR17520069.14764215
SRR17520069.14764216
SRR17520069.14764217
SRR17520069.14764218
SRR17520069.14764219
SRR17520069.14764220
SRR17520069.14764221
SRR17520069.14764222
SRR17520069.14764223
SRR17520069.14764224

SRR17520069.14764225
SRR17520069.14764226
SRR17520069.14764227
SRR17520069.14764228
SRR17520069.14764229
SRR17520069.14764230
SRR17520069.14764231
SRR17520069.14764232
SRR17520069.14764233
SRR17520069.14764234
SRR17520069.14764235
SRR17520069.14764236
SRR17520069.14764237
SRR17520069.14764238
SRR17520069.14764239
SRR17520069.14764240
SRR17520069.14764241
SRR17520069.14764242
SRR17520069.14764243
SRR17520069.14764245
SRR17520069.14764246
SRR17520069.14764247
SRR17520069.14764248
SRR17520069.14764249
SRR17520069.14764250
SRR17520069.14764251
SRR17520069.14764253
SRR17520069.14764254
SRR17520069.14764255
SRR17520069.14764256
SRR17520069.14764257
SRR17520069.14764258
SRR17520069.14764259
SRR17520069.14764260
SRR17520069.14764261
SRR17520069.14764262
SRR17520069.14764263
SRR17520069.14764264
SRR17520069.14764265
SRR17520069.14764267
SRR17520069.14764268
SRR17520069.14764269
SRR17520069.14764270
SRR17520069.14764271
SRR17520069.14764273
SRR17520069.14764274
SRR17520069.14764275

SRR17520069.14764276
SRR17520069.14764277
SRR17520069.14764278
SRR17520069.14764279
SRR17520069.14764280
SRR17520069.14764281
SRR17520069.14764282
SRR17520069.14764283
SRR17520069.14764284
SRR17520069.14764285
SRR17520069.14764286
SRR17520069.14764287
SRR17520069.14764288
SRR17520069.14764289
SRR17520069.14764290
SRR17520069.14764291
SRR17520069.14764292
SRR17520069.14764293
SRR17520069.14764294
SRR17520069.14764296
SRR17520069.14764297
SRR17520069.14764298
SRR17520069.14767109
SRR17520069.14819163
SRR17520069.14819164
SRR17520069.14819165
SRR17520069.14819166
SRR17520069.14819167
SRR17520069.14819168
SRR17520069.14819169
SRR17520069.14819170
SRR17520069.14819171
SRR17520069.14819172
SRR17520069.14819173
SRR17520069.14819174
SRR17520069.14819175
SRR17520069.14819176
SRR17520069.14819177
SRR17520069.14819178
SRR17520069.14819179
SRR17520069.14824408
SRR17520069.14824409
SRR17520069.14824410
SRR17520069.14824411
SRR17520069.14824412
SRR17520069.14824413
SRR17520069.14824414

SRR17520069.14824417
SRR17520069.14824418
SRR17520069.14824419
SRR17520069.14824420
SRR17520069.14824421
SRR17520069.14824422
SRR17520069.14824423
SRR17520069.14824424
SRR17520069.14824425
SRR17520069.14824426
SRR17520069.14824427
SRR17520069.14824428
SRR17520069.14824429
SRR17520069.14824430
SRR17520069.14824431
SRR17520069.14824432
SRR17520069.14824433
SRR17520069.14824434
SRR17520069.14824435
SRR17520069.14824436
SRR17520069.14824437
SRR17520069.14824438
SRR17520069.14824439
SRR17520069.14824440
SRR17520069.14824441
SRR17520069.14824442
SRR17520069.14824443
SRR17520069.14824444
SRR17520069.14824445
SRR17520069.14824446
SRR17520069.14824447
SRR17520069.14824448
SRR17520069.14824449
SRR17520069.14824450
SRR17520069.14824451
SRR17520069.14824452
SRR17520069.14824453
SRR17520069.14824454
SRR17520069.14824455
SRR17520069.14824456
SRR17520069.14824457
SRR17520069.14824458
SRR17520069.14824459
SRR17520069.14824460
SRR17520069.14827935
SRR17520069.14827936
SRR17520069.14827937

SRR17520069.14827938
SRR17520069.14827939
SRR17520069.14827940
SRR17520069.14834377
SRR17520069.14841956
SRR17520069.14841957
SRR17520069.14841958
SRR17520069.14841959
SRR17520069.14841960
SRR17520069.14841961
SRR17520069.14841962
SRR17520069.14841963
SRR17520069.14841964
SRR17520069.14841965
SRR17520069.14841966
SRR17520069.14841967
SRR17520069.14841968
SRR17520069.14841969
SRR17520069.14841970
SRR17520069.14841971
SRR17520069.14841972
SRR17520069.14841973
SRR17520069.14841974
SRR17520069.14841975
SRR17520069.14841976
SRR17520069.14841977
SRR17520069.14841978
SRR17520069.14841979
SRR17520069.14841980
SRR17520069.14841981
SRR17520069.14841982
SRR17520069.14841983
SRR17520069.14841984
SRR17520069.14841985
SRR17520069.14841986
SRR17520069.14841987
SRR17520069.14841988
SRR17520069.14841989
SRR17520069.14841990
SRR17520069.14841991
SRR17520069.14841992
SRR17520069.14841993
SRR17520069.14841998
SRR17520069.14842000
SRR17520069.14842001
SRR17520069.14850494
SRR17520069.14874170

SRR17520069.14874171
SRR17520069.14874173
SRR17520069.14874174
SRR17520069.14874175
SRR17520069.14874176
SRR17520069.14874177
SRR17520069.14874178
SRR17520069.14874181
SRR17520069.14874182
SRR17520069.14874183
SRR17520069.14874184
SRR17520069.14874185
SRR17520069.14874186
SRR17520069.14874190
SRR17520069.14874192
SRR17520069.14877914
SRR17520069.14877915
SRR17520069.14877916
SRR17520069.14877917
SRR17520069.14877918
SRR17520069.14877919
SRR17520069.14877920
SRR17520069.14877921
SRR17520069.14877922
SRR17520069.14877923
SRR17520069.14877924
SRR17520069.14877925
SRR17520069.14877926
SRR17520069.14877927
SRR17520069.14877928
SRR17520069.14877929
SRR17520069.14877930
SRR17520069.14877931
SRR17520069.14877932
SRR17520069.14877933
SRR17520069.14877934
SRR17520069.14877935
SRR17520069.14877936
SRR17520069.14877937
SRR17520069.14877938
SRR17520069.14877939
SRR17520069.14877940
SRR17520069.14877941
SRR17520069.14877942
SRR17520069.14877943
SRR17520069.14877944
SRR17520069.14877945

SRR17520069.14877946
SRR17520069.14877947
SRR17520069.14877948
SRR17520069.14877949
SRR17520069.14877950
SRR17520069.14877951
SRR17520069.14877952
SRR17520069.14877953
SRR17520069.14877954
SRR17520069.14877955
SRR17520069.14877956
SRR17520069.14877957
SRR17520069.14877958
SRR17520069.14877959
SRR17520069.14877960
SRR17520069.14877961
SRR17520069.14877962
SRR17520069.14877963
SRR17520069.14877964
SRR17520069.14877965
SRR17520069.14877966
SRR17520069.14877967
SRR17520069.14877968
SRR17520069.14877969
SRR17520069.14877970
SRR17520069.14877971
SRR17520069.14877972
SRR17520069.14877973
SRR17520069.14877974
SRR17520069.14877975
SRR17520069.14879725
SRR17520069.14879726
SRR17520069.14884143
SRR17520069.14897094
SRR17520069.14897098
SRR17520069.14897114
SRR17520069.14898474
SRR17520069.14898475
SRR17520069.14898476
SRR17520069.14898477
SRR17520069.14898478
SRR17520069.14898479
SRR17520069.14898480
SRR17520069.14898481
SRR17520069.14898482
SRR17520069.14898483
SRR17520069.14898484

SRR17520069.14898485
SRR17520069.14898486
SRR17520069.14898487
SRR17520069.14898488
SRR17520069.14898489
SRR17520069.14940924
SRR17520069.14940925
SRR17520069.14940926
SRR17520069.14957119
SRR17520069.14957120
SRR17520069.14957121
SRR17520069.14957122
SRR17520069.14957123
SRR17520069.14957124
SRR17520069.14957125
SRR17520069.14957126
SRR17520069.14957127
SRR17520069.14957128
SRR17520069.14957129
SRR17520069.14957130
SRR17520069.14957131
SRR17520069.14957132
SRR17520069.14957133
SRR17520069.14957134
SRR17520069.14957135
SRR17520069.14957136
SRR17520069.14957137
SRR17520069.14957138
SRR17520069.14957139
SRR17520069.14957140
SRR17520069.14957141
SRR17520069.14957142
SRR17520069.14957143
SRR17520069.14957144
SRR17520069.14957145
SRR17520069.14957146
SRR17520069.14957147
SRR17520069.14957148
SRR17520069.14957149
SRR17520069.14957150
SRR17520069.14957151
SRR17520069.14957152
SRR17520069.14957153
SRR17520069.14957154
SRR17520069.14957155
SRR17520069.14957156
SRR17520069.14957157

SRR17520069.14957158
SRR17520069.14957159
SRR17520069.14957160
SRR17520069.14957161
SRR17520069.14957162
SRR17520069.14957163
SRR17520069.14957165
SRR17520069.14957166
SRR17520069.14957167
SRR17520069.14957168
SRR17520069.14957169
SRR17520069.14957170
SRR17520069.14957171
SRR17520069.14957172
SRR17520069.14957173
SRR17520069.14957174
SRR17520069.14957175
SRR17520069.14957176
SRR17520069.14957177
SRR17520069.14957178
SRR17520069.14957179
SRR17520069.14957180
SRR17520069.14957181
SRR17520069.14957182
SRR17520069.14957183
SRR17520069.14957184
SRR17520069.14957185
SRR17520069.14957186
SRR17520069.14957187
SRR17520069.14957188
SRR17520069.14957189
SRR17520069.14957190
SRR17520069.14957191
SRR17520069.14957192
SRR17520069.14957193
SRR17520069.14957194
SRR17520069.14957195
SRR17520069.14957196
SRR17520069.14957197
SRR17520069.14957198
SRR17520069.14957199
SRR17520069.14957200
SRR17520069.14957201
SRR17520069.14957202
SRR17520069.14957203
SRR17520069.14957204
SRR17520069.14957205

SRR17520069.14957206
SRR17520069.14957207
SRR17520069.14957208
SRR17520069.14957209
SRR17520069.14957210
SRR17520069.14957211
SRR17520069.14957212
SRR17520069.14957213
SRR17520069.14957214
SRR17520069.14957215
SRR17520069.14957216
SRR17520069.14957217
SRR17520069.14957218
SRR17520069.14957219
SRR17520069.14957220
SRR17520069.14957221
SRR17520069.14957222
SRR17520069.14957223
SRR17520069.14957224
SRR17520069.14973197
SRR17520069.14973243
SRR17520069.14983259
SRR17520069.14983260
SRR17520069.14998342
SRR17520069.14998343
SRR17520069.14998344
SRR17520069.14998345
SRR17520069.14998346
SRR17520069.14998347
SRR17520069.14998348
SRR17520069.14998349
SRR17520069.14998350
SRR17520069.14998351
SRR17520069.14998352
SRR17520069.14998353
SRR17520069.14998354
SRR17520069.14998355
SRR17520069.14998357
SRR17520069.14998358
SRR17520069.14998359
SRR17520069.14998360
SRR17520069.14998361
SRR17520069.14998362
SRR17520069.14998363
SRR17520069.14998364
SRR17520069.14998365
SRR17520069.14998366

SRR17520069.14998367
SRR17520069.14998368
SRR17520069.14998369
SRR17520069.14998370
SRR17520069.14998371
SRR17520069.14998372
SRR17520069.14998373
SRR17520069.14998374
SRR17520069.14998375
SRR17520069.14998376
SRR17520069.14998377
SRR17520069.14998378
SRR17520069.14998379
SRR17520069.14998380
SRR17520069.14998381
SRR17520069.14998382
SRR17520069.14998383
SRR17520069.14998384
SRR17520069.14998385
SRR17520069.14998386
SRR17520069.14998387
SRR17520069.14998388
SRR17520069.14998389
SRR17520069.14998390
SRR17520069.14998391
SRR17520069.14998392
SRR17520069.14998393
SRR17520069.14998394
SRR17520069.14998395
SRR17520069.14998396
SRR17520069.14998397
SRR17520069.14998398
SRR17520069.14998399
SRR17520069.14998400
SRR17520069.14998401
SRR17520069.14998402
SRR17520069.14998403
SRR17520069.14998404
SRR17520069.14998405
SRR17520069.14998406
SRR17520069.14998407
SRR17520069.14998408
SRR17520069.14998409
SRR17520069.15014839
SRR17520069.15014840
SRR17520069.15041896
SRR17520069.15041897

SRR17520069.15041898
SRR17520069.15041899
SRR17520069.15041900
SRR17520069.15041901
SRR17520069.15041902
SRR17520069.15041903
SRR17520069.15041904
SRR17520069.15041906
SRR17520069.15041907
SRR17520069.15041908
SRR17520069.15041909
SRR17520069.15041910
SRR17520069.15041911
SRR17520069.15041912
SRR17520069.15041913
SRR17520069.15041914
SRR17520069.15041915
SRR17520069.15041916
SRR17520069.15041917
SRR17520069.15041918
SRR17520069.15041919
SRR17520069.15041920
SRR17520069.15041921
SRR17520069.15041922
SRR17520069.15041923
SRR17520069.15041924
SRR17520069.15044465
SRR17520069.15044466
SRR17520069.15044467
SRR17520069.15044468
SRR17520069.15044469
SRR17520069.15044470
SRR17520069.15044471
SRR17520069.15044472
SRR17520069.15044473
SRR17520069.15044474
SRR17520069.15044475
SRR17520069.15044476
SRR17520069.15044477
SRR17520069.15044478
SRR17520069.15044479
SRR17520069.15044480
SRR17520069.15044482
SRR17520069.15044483
SRR17520069.15044484
SRR17520069.15044485
SRR17520069.15044486

SRR17520069.15044487
SRR17520069.15044488
SRR17520069.15044489
SRR17520069.15044490
SRR17520069.15044491
SRR17520069.15044492
SRR17520069.15044493
SRR17520069.15044494
SRR17520069.15044495
SRR17520069.15044496
SRR17520069.15044497
SRR17520069.15044498
SRR17520069.15044499
SRR17520069.15044500
SRR17520069.15044501
SRR17520069.15044502
SRR17520069.15044503
SRR17520069.15044504
SRR17520069.15044505
SRR17520069.15044506
SRR17520069.15044508
SRR17520069.15044509
SRR17520069.15044510
SRR17520069.15044511
SRR17520069.15044512
SRR17520069.15044513
SRR17520069.15044514
SRR17520069.15044515
SRR17520069.15044516
SRR17520069.15044517
SRR17520069.15044518
SRR17520069.15044519
SRR17520069.15044520
SRR17520069.15044521
SRR17520069.15044522
SRR17520069.15044523
SRR17520069.15044524
SRR17520069.15044525
SRR17520069.15044526
SRR17520069.15044527
SRR17520069.15044528
SRR17520069.15044529
SRR17520069.15044530
SRR17520069.15044531
SRR17520069.15044532
SRR17520069.15044533
SRR17520069.15044534

SRR17520069.15044535
SRR17520069.15044536
SRR17520069.15044537
SRR17520069.15044538
SRR17520069.15044539
SRR17520069.15044540
SRR17520069.15044541
SRR17520069.15044542
SRR17520069.15044543
SRR17520069.15044544
SRR17520069.15044545
SRR17520069.15044546
SRR17520069.15044547
SRR17520069.15044548
SRR17520069.15044549
SRR17520069.15044550
SRR17520069.15044551
SRR17520069.15044552
SRR17520069.15044553
SRR17520069.15044554
SRR17520069.15044555
SRR17520069.15044556
SRR17520069.15044557
SRR17520069.15044558
SRR17520069.15044559
SRR17520069.15044560
SRR17520069.15044561
SRR17520069.15044562
SRR17520069.15044563
SRR17520069.15044564
SRR17520069.15044565
SRR17520069.15044567
SRR17520069.15044568
SRR17520069.15044569
SRR17520069.15044570
SRR17520069.15044572
SRR17520069.15044573
SRR17520069.15044574
SRR17520069.15044575
SRR17520069.15044576
SRR17520069.15044578
SRR17520069.15044579
SRR17520069.15044580
SRR17520069.15044581
SRR17520069.15044582
SRR17520069.15044583
SRR17520069.15044584

SRR17520069.15044585
SRR17520069.15044586
SRR17520069.15044587
SRR17520069.15044588
SRR17520069.15044589
SRR17520069.15044590
SRR17520069.15044591
SRR17520069.15044592
SRR17520069.15044593
SRR17520069.15044594
SRR17520069.15044595
SRR17520069.15044596
SRR17520069.15044597
SRR17520069.15044598
SRR17520069.15044599
SRR17520069.15044600
SRR17520069.15044601
SRR17520069.15044602
SRR17520069.15044603
SRR17520069.15044604
SRR17520069.15044605
SRR17520069.15044606
SRR17520069.15044607
SRR17520069.15044608
SRR17520069.15044609
SRR17520069.15044610
SRR17520069.15044611
SRR17520069.15044612
SRR17520069.15044613
SRR17520069.15044614
SRR17520069.15044615
SRR17520069.15044616
SRR17520069.15044617
SRR17520069.15044618
SRR17520069.15044619
SRR17520069.15044620
SRR17520069.15044621
SRR17520069.15044622
SRR17520069.15044623
SRR17520069.15044624
SRR17520069.15044625
SRR17520069.15044626
SRR17520069.15044627
SRR17520069.15044628
SRR17520069.15044629
SRR17520069.15044630
SRR17520069.15044631

SRR17520069.15044632
SRR17520069.15044633
SRR17520069.15044634
SRR17520069.15044635
SRR17520069.15044636
SRR17520069.15044637
SRR17520069.15044638
SRR17520069.15044639
SRR17520069.15044640
SRR17520069.15044641
SRR17520069.15044642
SRR17520069.15044643
SRR17520069.15044644
SRR17520069.15044645
SRR17520069.15044646
SRR17520069.15044647
SRR17520069.15044648
SRR17520069.15044649
SRR17520069.15044651
SRR17520069.15044652
SRR17520069.15044653
SRR17520069.15044654
SRR17520069.15044655
SRR17520069.15044656
SRR17520069.15044657
SRR17520069.15044658
SRR17520069.15044659
SRR17520069.15044660
SRR17520069.15044661
SRR17520069.15051715
SRR17520069.15051716
SRR17520069.15056516
SRR17520069.15056517
SRR17520069.15056518
SRR17520069.15056519
SRR17520069.15056520
SRR17520069.15056521
SRR17520069.15056522
SRR17520069.15056523
SRR17520069.15056524
SRR17520069.15056525
SRR17520069.15056527
SRR17520069.15056528
SRR17520069.15056529
SRR17520069.15056530
SRR17520069.15056531
SRR17520069.15056532

SRR17520069.15056533
SRR17520069.15056534
SRR17520069.15056535
SRR17520069.15056536
SRR17520069.15056537
SRR17520069.15056538
SRR17520069.15056539
SRR17520069.15056541
SRR17520069.15056542
SRR17520069.15056543
SRR17520069.15056544
SRR17520069.15056545
SRR17520069.15056546
SRR17520069.15056547
SRR17520069.15056548
SRR17520069.15056549
SRR17520069.15056550
SRR17520069.15056551
SRR17520069.15056552
SRR17520069.15056553
SRR17520069.15056554
SRR17520069.15056555
SRR17520069.15056556
SRR17520069.15056557
SRR17520069.15056558
SRR17520069.15056559
SRR17520069.15056560
SRR17520069.15056561
SRR17520069.15056562
SRR17520069.15056563
SRR17520069.15056564
SRR17520069.15056565
SRR17520069.15056566
SRR17520069.15056567
SRR17520069.15056568
SRR17520069.15056569
SRR17520069.15056570
SRR17520069.15056571
SRR17520069.15056572
SRR17520069.15056573
SRR17520069.15056574
SRR17520069.15056575
SRR17520069.15056576
SRR17520069.15056577
SRR17520069.15056578
SRR17520069.15056579
SRR17520069.15056580

SRR17520069.15056581
SRR17520069.15056582
SRR17520069.15056583
SRR17520069.15056584
SRR17520069.15056585
SRR17520069.15056586
SRR17520069.15056587
SRR17520069.15056588
SRR17520069.15056589
SRR17520069.15056590
SRR17520069.15056591
SRR17520069.15056592
SRR17520069.15056593
SRR17520069.15056594
SRR17520069.15056595
SRR17520069.15056596
SRR17520069.15061681
SRR17520069.15061682
SRR17520069.15061683
SRR17520069.15061684
SRR17520069.15061685
SRR17520069.15061686
SRR17520069.15061687
SRR17520069.15061688
SRR17520069.15061689
SRR17520069.15061690
SRR17520069.15061691
SRR17520069.15061692
SRR17520069.15061693
SRR17520069.15061694
SRR17520069.15061695
SRR17520069.15061696
SRR17520069.15061697
SRR17520069.15063030
SRR17520069.15074046
SRR17520069.15079316
SRR17520069.15079401
SRR17520069.15112249
SRR17520069.15112250
SRR17520069.15112251
SRR17520069.15112252
SRR17520069.15112253
SRR17520069.15112254
SRR17520069.15112255
SRR17520069.15112256
SRR17520069.15112257
SRR17520069.15112258

SRR17520069.15112259
SRR17520069.15112260
SRR17520069.15112261
SRR17520069.15112262
SRR17520069.15112263
SRR17520069.15112264
SRR17520069.15112265
SRR17520069.15112266
SRR17520069.15112267
SRR17520069.15112268
SRR17520069.15112269
SRR17520069.15112270
SRR17520069.15112271
SRR17520069.15112272
SRR17520069.15112273
SRR17520069.15112274
SRR17520069.15112275
SRR17520069.15112276
SRR17520069.15112277
SRR17520069.15112278
SRR17520069.15112279
SRR17520069.15112280
SRR17520069.15112281
SRR17520069.15112282
SRR17520069.15112283
SRR17520069.15112284
SRR17520069.15112285
SRR17520069.15112286
SRR17520069.15112287
SRR17520069.15112288
SRR17520069.15112289
SRR17520069.15112290
SRR17520069.15112292
SRR17520069.15112293
SRR17520069.15112294
SRR17520069.15112295
SRR17520069.15112296
SRR17520069.15112297
SRR17520069.15112298
SRR17520069.15112299
SRR17520069.15112300
SRR17520069.15112301
SRR17520069.15112302
SRR17520069.15112303
SRR17520069.15112304
SRR17520069.15112305
SRR17520069.15112306

SRR17520069.15112307
SRR17520069.15112309
SRR17520069.15112310
SRR17520069.15112311
SRR17520069.15112312
SRR17520069.15112313
SRR17520069.15112314
SRR17520069.15112315
SRR17520069.15112316
SRR17520069.15112317
SRR17520069.15112318
SRR17520069.15112319
SRR17520069.15112320
SRR17520069.15112321
SRR17520069.15112322
SRR17520069.15112323
SRR17520069.15112324
SRR17520069.15112325
SRR17520069.15112326
SRR17520069.15112327
SRR17520069.15112328
SRR17520069.15112329
SRR17520069.15112332
SRR17520069.15112333
SRR17520069.15112334
SRR17520069.15112335
SRR17520069.15112336
SRR17520069.15112337
SRR17520069.15112338
SRR17520069.15112339
SRR17520069.15112340
SRR17520069.15112341
SRR17520069.15112342
SRR17520069.15112343
SRR17520069.15112344
SRR17520069.15112345
SRR17520069.15112346
SRR17520069.15112348
SRR17520069.15112349
SRR17520069.15112350
SRR17520069.15112351
SRR17520069.15112352
SRR17520069.15112353
SRR17520069.15112354
SRR17520069.15112355
SRR17520069.15112356
SRR17520069.15112357

SRR17520069.15112358
SRR17520069.15112359
SRR17520069.15112360
SRR17520069.15112361
SRR17520069.15112362
SRR17520069.15112363
SRR17520069.15112364
SRR17520069.15112365
SRR17520069.15112366
SRR17520069.15112367
SRR17520069.15112368
SRR17520069.15112369
SRR17520069.15112370
SRR17520069.15112371
SRR17520069.15112372
SRR17520069.15112373
SRR17520069.15112374
SRR17520069.15112375
SRR17520069.15112376
SRR17520069.15112377
SRR17520069.15112378
SRR17520069.15112379
SRR17520069.15112380
SRR17520069.15112381
SRR17520069.15112382
SRR17520069.15112383
SRR17520069.15112384
SRR17520069.15112385
SRR17520069.15112386
SRR17520069.15112387
SRR17520069.15112388
SRR17520069.15112389
SRR17520069.15112390
SRR17520069.15112391
SRR17520069.15112392
SRR17520069.15112393
SRR17520069.15112394
SRR17520069.15112395
SRR17520069.15112396
SRR17520069.15112397
SRR17520069.15112398
SRR17520069.15112399
SRR17520069.15112400
SRR17520069.15112401
SRR17520069.15112402
SRR17520069.15112403
SRR17520069.15112404

SRR17520069.15112405
SRR17520069.15112406
SRR17520069.15112407
SRR17520069.15112408
SRR17520069.15112409
SRR17520069.15112410
SRR17520069.15112411
SRR17520069.15112412
SRR17520069.15112413
SRR17520069.15112414
SRR17520069.15112415
SRR17520069.15112416
SRR17520069.15112417
SRR17520069.15112418
SRR17520069.15112419
SRR17520069.15112420
SRR17520069.15112421
SRR17520069.15112422
SRR17520069.15112423
SRR17520069.15112424
SRR17520069.15112425
SRR17520069.15112426
SRR17520069.15112427
SRR17520069.15112428
SRR17520069.15112429
SRR17520069.15122220
SRR17520069.15122355
SRR17520069.15122356
SRR17520069.15123271
SRR17520069.15123273
SRR17520069.15123274
SRR17520069.15123275
SRR17520069.15123278
SRR17520069.15123279
SRR17520069.15123280
SRR17520069.15123281
SRR17520069.15123282
SRR17520069.15123284
SRR17520069.15123285
SRR17520069.15123286
SRR17520069.15123287
SRR17520069.15123288
SRR17520069.15123289
SRR17520069.15123291
SRR17520069.15123292
SRR17520069.15123293
SRR17520069.15123294

SRR17520069.15123295
SRR17520069.15123296
SRR17520069.15123297
SRR17520069.15123298
SRR17520069.15123299
SRR17520069.15123300
SRR17520069.15123301
SRR17520069.15123302
SRR17520069.15123303
SRR17520069.15123304
SRR17520069.15123305
SRR17520069.15123306
SRR17520069.15123307
SRR17520069.15123308
SRR17520069.15123309
SRR17520069.15123310
SRR17520069.15123312
SRR17520069.15123313
SRR17520069.15123314
SRR17520069.15134373
SRR17520069.15134374
SRR17520069.15134375
SRR17520069.15134377
SRR17520069.15134378
SRR17520069.15134379
SRR17520069.15134380
SRR17520069.15134381
SRR17520069.15134382
SRR17520069.15134383
SRR17520069.15134384
SRR17520069.15134385
SRR17520069.15134386
SRR17520069.15134387
SRR17520069.15134388
SRR17520069.15134389
SRR17520069.15134390
SRR17520069.15134391
SRR17520069.15134392
SRR17520069.15134393
SRR17520069.15134394
SRR17520069.15134395
SRR17520069.15134396
SRR17520069.15134397
SRR17520069.15134398
SRR17520069.15134399
SRR17520069.15134400
SRR17520069.15134401

SRR17520069.15134402
SRR17520069.15134403
SRR17520069.15134404
SRR17520069.15134405
SRR17520069.15134406
SRR17520069.15134407
SRR17520069.15134408
SRR17520069.15134409
SRR17520069.15134410
SRR17520069.15134411
SRR17520069.15134412
SRR17520069.15134413
SRR17520069.15134414
SRR17520069.15134415
SRR17520069.15134416
SRR17520069.15134417
SRR17520069.15145682
SRR17520069.15145685
SRR17520069.15145686
SRR17520069.15145688
SRR17520069.15145689
SRR17520069.15145690
SRR17520069.15145691
SRR17520069.15145692
SRR17520069.15145693
SRR17520069.15145694
SRR17520069.15145695
SRR17520069.15145696
SRR17520069.15145697
SRR17520069.15145698
SRR17520069.15145699
SRR17520069.15145700
SRR17520069.15145701
SRR17520069.15145702
SRR17520069.15145703
SRR17520069.15145704
SRR17520069.15145705
SRR17520069.15145706
SRR17520069.15145707
SRR17520069.15145708
SRR17520069.15145709
SRR17520069.15145710
SRR17520069.15145711
SRR17520069.15145712
SRR17520069.15145713
SRR17520069.15145714
SRR17520069.15145715

SRR17520069.15145716
SRR17520069.15145717
SRR17520069.15145718
SRR17520069.15145720
SRR17520069.15145721
SRR17520069.15145722
SRR17520069.15145723
SRR17520069.15145724
SRR17520069.15145725
SRR17520069.15145726
SRR17520069.15145727
SRR17520069.15145728
SRR17520069.15145729
SRR17520069.15145730
SRR17520069.15145731
SRR17520069.15145732
SRR17520069.15145733
SRR17520069.15145734
SRR17520069.15145735
SRR17520069.15145736
SRR17520069.15145737
SRR17520069.15145738
SRR17520069.15145739
SRR17520069.15145740
SRR17520069.15145742
SRR17520069.15145743
SRR17520069.15145744
SRR17520069.15145746
SRR17520069.15145747
SRR17520069.15145748
SRR17520069.15145749
SRR17520069.15145750
SRR17520069.15145751
SRR17520069.15145752
SRR17520069.15145753
SRR17520069.15145755
SRR17520069.15145756
SRR17520069.15145757
SRR17520069.15145758
SRR17520069.15145759
SRR17520069.15145760
SRR17520069.15145761
SRR17520069.15145762
SRR17520069.15145763
SRR17520069.15145764
SRR17520069.15145765
SRR17520069.15145766

SRR17520069.15145767
SRR17520069.15145768
SRR17520069.15145769
SRR17520069.15145770
SRR17520069.15145771
SRR17520069.15145772
SRR17520069.15145773
SRR17520069.15145774
SRR17520069.15145776
SRR17520069.15145778
SRR17520069.15145779
SRR17520069.15145781
SRR17520069.15145783
SRR17520069.15179553
SRR17520069.15179555
SRR17520069.15179556
SRR17520069.15179557
SRR17520069.15179559
SRR17520069.15179560
SRR17520069.15179561
SRR17520069.15179562
SRR17520069.15179563
SRR17520069.15179564
SRR17520069.15179565
SRR17520069.15179566
SRR17520069.15179567
SRR17520069.15179568
SRR17520069.15179569
SRR17520069.15179570
SRR17520069.15179571
SRR17520069.15179572
SRR17520069.15179573
SRR17520069.15193665
SRR17520069.15201744
SRR17520069.15201745
SRR17520069.15201746
SRR17520069.15201747
SRR17520069.15201748
SRR17520069.15201749
SRR17520069.15201750
SRR17520069.15201751
SRR17520069.15201752
SRR17520069.15201753
SRR17520069.15201754
SRR17520069.15201755
SRR17520069.15201756
SRR17520069.15201757

SRR17520069.15201758
SRR17520069.15201759
SRR17520069.15201760
SRR17520069.15201761
SRR17520069.15201762
SRR17520069.15201763
SRR17520069.15201764
SRR17520069.15201765
SRR17520069.15201766
SRR17520069.15201768
SRR17520069.15201769
SRR17520069.15201770
SRR17520069.15201771
SRR17520069.15201772
SRR17520069.15201773
SRR17520069.15201774
SRR17520069.15201775
SRR17520069.15201776
SRR17520069.15201777
SRR17520069.15201778
SRR17520069.15201779
SRR17520069.15201780
SRR17520069.15201781
SRR17520069.15201782
SRR17520069.15201783
SRR17520069.15201784
SRR17520069.15201785
SRR17520069.15201786
SRR17520069.15201787
SRR17520069.15201788
SRR17520069.15201789
SRR17520069.15201790
SRR17520069.15201791
SRR17520069.15201792
SRR17520069.15201793
SRR17520069.15201794
SRR17520069.15201795
SRR17520069.15201796
SRR17520069.15201797
SRR17520069.15201798
SRR17520069.15201799
SRR17520069.15201800
SRR17520069.15201801
SRR17520069.15201802
SRR17520069.15201803
SRR17520069.15201804
SRR17520069.15201805

SRR17520069.15201806
SRR17520069.15201807
SRR17520069.15201808
SRR17520069.15201809
SRR17520069.15201810
SRR17520069.15201811
SRR17520069.15201812
SRR17520069.15201813
SRR17520069.15201814
SRR17520069.15201815
SRR17520069.15201816
SRR17520069.15201817
SRR17520069.15201818
SRR17520069.15201819
SRR17520069.15201820
SRR17520069.15201821
SRR17520069.15201822
SRR17520069.15201823
SRR17520069.15201824
SRR17520069.15201825
SRR17520069.15201826
SRR17520069.15201827
SRR17520069.15201828
SRR17520069.15201829
SRR17520069.15201830
SRR17520069.15201831
SRR17520069.15201832
SRR17520069.15201833
SRR17520069.15201834
SRR17520069.15201835
SRR17520069.15201836
SRR17520069.15201837
SRR17520069.15201838
SRR17520069.15201839
SRR17520069.15201840
SRR17520069.15201841
SRR17520069.15201842
SRR17520069.15201843
SRR17520069.15201844
SRR17520069.15201845
SRR17520069.15201846
SRR17520069.15201847
SRR17520069.15201848
SRR17520069.15201849
SRR17520069.15201850
SRR17520069.15201851
SRR17520069.15201852

SRR17520069.15201853
SRR17520069.15201854
SRR17520069.15201855
SRR17520069.15201856
SRR17520069.15201857
SRR17520069.15201858
SRR17520069.15201859
SRR17520069.15201860
SRR17520069.15201861
SRR17520069.15233135
SRR17520069.15237148
SRR17520069.15238337
SRR17520069.15239731
SRR17520069.15240089
SRR17520069.15240090
SRR17520069.15240091
SRR17520069.15240092
SRR17520069.15240093
SRR17520069.15240094
SRR17520069.15240095
SRR17520069.15240096
SRR17520069.15240097
SRR17520069.15240098
SRR17520069.15240099
SRR17520069.15240100
SRR17520069.15240101
SRR17520069.15240102
SRR17520069.15240103
SRR17520069.15240104
SRR17520069.15240105
SRR17520069.15240106
SRR17520069.15240107
SRR17520069.15240108
SRR17520069.15249782
SRR17520069.15249783
SRR17520069.15249784
SRR17520069.15249785
SRR17520069.15249786
SRR17520069.15249787
SRR17520069.15249788
SRR17520069.15249789
SRR17520069.15249790
SRR17520069.15249791
SRR17520069.15249792
SRR17520069.15270037
SRR17520069.15270039
SRR17520069.15270040

SRR17520069.15270041
SRR17520069.15270639
SRR17520069.15270640
SRR17520069.15270641
SRR17520069.15270642
SRR17520069.15270643
SRR17520069.15270644
SRR17520069.15270645
SRR17520069.15270646
SRR17520069.15270647
SRR17520069.15270648
SRR17520069.15270650
SRR17520069.15272061
SRR17520069.15293540
SRR17520069.15293542
SRR17520069.15293543
SRR17520069.15293544
SRR17520069.15293545
SRR17520069.15293546
SRR17520069.15293548
SRR17520069.15293549
SRR17520069.15293550
SRR17520069.15293551
SRR17520069.15293552
SRR17520069.15293553
SRR17520069.15293554
SRR17520069.15293555
SRR17520069.15293556
SRR17520069.15293557
SRR17520069.15293558
SRR17520069.15293559
SRR17520069.15293561
SRR17520069.15293562
SRR17520069.15293563
SRR17520069.15293564
SRR17520069.15293565
SRR17520069.15293566
SRR17520069.15293567
SRR17520069.15293568
SRR17520069.15293569
SRR17520069.15293570
SRR17520069.15293571
SRR17520069.15293572
SRR17520069.15300325
SRR17520069.15303433
SRR17520069.15303434
SRR17520069.15303435

SRR17520069.15303436
SRR17520069.15303437
SRR17520069.15303438
SRR17520069.15303439
SRR17520069.15303440
SRR17520069.15303441
SRR17520069.15303442
SRR17520069.15303443
SRR17520069.15303444
SRR17520069.15303445
SRR17520069.15303446
SRR17520069.15303448
SRR17520069.15303449
SRR17520069.15303450
SRR17520069.15326144
SRR17520069.15326293
SRR17520069.15327471
SRR17520069.15327472
SRR17520069.15327473
SRR17520069.15327474
SRR17520069.15327475
SRR17520069.15327476
SRR17520069.15327477
SRR17520069.15327478
SRR17520069.15327479
SRR17520069.15327480
SRR17520069.15327481
SRR17520069.15327482
SRR17520069.15327483
SRR17520069.15327485
SRR17520069.15327486
SRR17520069.15327488
SRR17520069.15327489
SRR17520069.15327490
SRR17520069.15327491
SRR17520069.15327493
SRR17520069.15327494
SRR17520069.15327495
SRR17520069.15327496
SRR17520069.15327497
SRR17520069.15327498
SRR17520069.15327499
SRR17520069.15327500
SRR17520069.15327501
SRR17520069.15327502
SRR17520069.15327503
SRR17520069.15327504

SRR17520069.15327505
SRR17520069.15327506
SRR17520069.15327507
SRR17520069.15327509
SRR17520069.15327510
SRR17520069.15327511
SRR17520069.15327512
SRR17520069.15327513
SRR17520069.15327514
SRR17520069.15327515
SRR17520069.15327516
SRR17520069.15327517
SRR17520069.15327518
SRR17520069.15327519
SRR17520069.15327520
SRR17520069.15327521
SRR17520069.15327522
SRR17520069.15327523
SRR17520069.15327524
SRR17520069.15327525
SRR17520069.15327526
SRR17520069.15327527
SRR17520069.15327528
SRR17520069.15327529
SRR17520069.15327530
SRR17520069.15327531
SRR17520069.15327533
SRR17520069.15327534
SRR17520069.15327535
SRR17520069.15327536
SRR17520069.15327537
SRR17520069.15327538
SRR17520069.15327539
SRR17520069.15327540
SRR17520069.15327541
SRR17520069.15327542
SRR17520069.15327543
SRR17520069.15327545
SRR17520069.15327546
SRR17520069.15327548
SRR17520069.15327549
SRR17520069.15327550
SRR17520069.15327551
SRR17520069.15327552
SRR17520069.15327553
SRR17520069.15327554
SRR17520069.15327555

SRR17520069.15327556
SRR17520069.15327557
SRR17520069.15327558
SRR17520069.15327559
SRR17520069.15327560
SRR17520069.15327561
SRR17520069.15327564
SRR17520069.15327565
SRR17520069.15327566
SRR17520069.15327567
SRR17520069.15327568
SRR17520069.15327569
SRR17520069.15327570
SRR17520069.15327571
SRR17520069.15327572
SRR17520069.15327573
SRR17520069.15327574
SRR17520069.15327575
SRR17520069.15327576
SRR17520069.15327577
SRR17520069.15340054
SRR17520069.15340239
SRR17520069.15340241
SRR17520069.15340242
SRR17520069.15340243
SRR17520069.15340244
SRR17520069.15340245
SRR17520069.15340246
SRR17520069.15340247
SRR17520069.15340248
SRR17520069.15340249
SRR17520069.15340250
SRR17520069.15340251
SRR17520069.15340252
SRR17520069.15340253
SRR17520069.15340254
SRR17520069.15340255
SRR17520069.15340256
SRR17520069.15340257
SRR17520069.15340258
SRR17520069.15340259
SRR17520069.15340260
SRR17520069.15340261
SRR17520069.15340262
SRR17520069.15340263
SRR17520069.15340264
SRR17520069.15340265

SRR17520069.15340267
SRR17520069.15340268
SRR17520069.15340269
SRR17520069.15340270
SRR17520069.15340271
SRR17520069.15340272
SRR17520069.15340273
SRR17520069.15340274
SRR17520069.15340275
SRR17520069.15340276
SRR17520069.15340277
SRR17520069.15340278
SRR17520069.15340280
SRR17520069.15340281
SRR17520069.15340282
SRR17520069.15340283
SRR17520069.15340284
SRR17520069.15340285
SRR17520069.15340286
SRR17520069.15340287
SRR17520069.15340288
SRR17520069.15340289
SRR17520069.15340290
SRR17520069.15340291
SRR17520069.15340292
SRR17520069.15340293
SRR17520069.15340294
SRR17520069.15340295
SRR17520069.15340296
SRR17520069.15340297
SRR17520069.15340298
SRR17520069.15340299
SRR17520069.15340300
SRR17520069.15340301
SRR17520069.15340302
SRR17520069.15340303
SRR17520069.15340304
SRR17520069.15340305
SRR17520069.15340306
SRR17520069.15340307
SRR17520069.15340308
SRR17520069.15340309
SRR17520069.15340310
SRR17520069.15340312
SRR17520069.15340313
SRR17520069.15340314
SRR17520069.15340315

SRR17520069.15340316
SRR17520069.15340317
SRR17520069.15340318
SRR17520069.15340319
SRR17520069.15340320
SRR17520069.15340321
SRR17520069.15340322
SRR17520069.15340323
SRR17520069.15340324
SRR17520069.15340325
SRR17520069.15340326
SRR17520069.15340327
SRR17520069.15340328
SRR17520069.15340329
SRR17520069.15340330
SRR17520069.15340331
SRR17520069.15340332
SRR17520069.15340333
SRR17520069.15340334
SRR17520069.15340335
SRR17520069.15340336
SRR17520069.15340337
SRR17520069.15340338
SRR17520069.15340339
SRR17520069.15340340
SRR17520069.15340341
SRR17520069.15340342
SRR17520069.15340343
SRR17520069.15340344
SRR17520069.15340345
SRR17520069.15340346
SRR17520069.15340347
SRR17520069.15349719
SRR17520069.15349721
SRR17520069.15349722
SRR17520069.15349723
SRR17520069.15349724
SRR17520069.15349726
SRR17520069.15349727
SRR17520069.15349728
SRR17520069.15349729
SRR17520069.15349730
SRR17520069.15349731
SRR17520069.15349732
SRR17520069.15349733
SRR17520069.15349734
SRR17520069.15349735

SRR17520069.15349736
SRR17520069.15349737
SRR17520069.15349738
SRR17520069.15349739
SRR17520069.15349740
SRR17520069.15349741
SRR17520069.15349742
SRR17520069.15349743
SRR17520069.15349744
SRR17520069.15349745
SRR17520069.15349748
SRR17520069.15349749
SRR17520069.15349750
SRR17520069.15349751
SRR17520069.15349757
SRR17520069.15349758
SRR17520069.15349759
SRR17520069.15349760
SRR17520069.15349761
SRR17520069.15349763
SRR17520069.15349764
SRR17520069.15349765
SRR17520069.15349766
SRR17520069.15349768
SRR17520069.15349769
SRR17520069.15349770
SRR17520069.15349771
SRR17520069.15349772
SRR17520069.15349773
SRR17520069.15349774
SRR17520069.15349775
SRR17520069.15349776
SRR17520069.15349777
SRR17520069.15349778
SRR17520069.15349779
SRR17520069.15349780
SRR17520069.15349781
SRR17520069.15349782
SRR17520069.15349783
SRR17520069.15349784
SRR17520069.15349785
SRR17520069.15349786
SRR17520069.15349787
SRR17520069.15349789
SRR17520069.15349790
SRR17520069.15349791
SRR17520069.15349792

SRR17520069.15349793
SRR17520069.15349794
SRR17520069.15349795
SRR17520069.15349796
SRR17520069.15349797
SRR17520069.15349798
SRR17520069.15349799
SRR17520069.15349800
SRR17520069.15349801
SRR17520069.15349803
SRR17520069.15349804
SRR17520069.15349805
SRR17520069.15349806
SRR17520069.15349807
SRR17520069.15349808
SRR17520069.15349809
SRR17520069.15349810
SRR17520069.15349811
SRR17520069.15349812
SRR17520069.15349813
SRR17520069.15349814
SRR17520069.15349815
SRR17520069.15349816
SRR17520069.15349817
SRR17520069.15349818
SRR17520069.15349819
SRR17520069.15349820
SRR17520069.15349821
SRR17520069.15349822
SRR17520069.15349823
SRR17520069.15355828
SRR17520069.15355829
SRR17520069.15355830
SRR17520069.15355831
SRR17520069.15355832
SRR17520069.15355833
SRR17520069.15355834
SRR17520069.15355835
SRR17520069.15355836
SRR17520069.15372123
SRR17520069.15372157
SRR17520069.15380427
SRR17520069.15380501
SRR17520069.15383541
SRR17520069.15383542
SRR17520069.15383543
SRR17520069.15383544

SRR17520069.15383546
SRR17520069.15383547
SRR17520069.15383548
SRR17520069.15383549
SRR17520069.15383550
SRR17520069.15383551
SRR17520069.15383552
SRR17520069.15383553
SRR17520069.15383554
SRR17520069.15383555
SRR17520069.15383557
SRR17520069.15383558
SRR17520069.15383559
SRR17520069.15395508
SRR17520069.15395509
SRR17520069.15395510
SRR17520069.15395511
SRR17520069.15395512
SRR17520069.15395513
SRR17520069.15395514
SRR17520069.15395515
SRR17520069.15395516
SRR17520069.15395517
SRR17520069.15395518
SRR17520069.15395519
SRR17520069.15395520
SRR17520069.15395521
SRR17520069.15395522
SRR17520069.15395523
SRR17520069.15395524
SRR17520069.15395525
SRR17520069.15395526
SRR17520069.15395527
SRR17520069.15395528
SRR17520069.15395529
SRR17520069.15395530
SRR17520069.15395531
SRR17520069.15395532
SRR17520069.15395533
SRR17520069.15395534
SRR17520069.15395535
SRR17520069.15395536
SRR17520069.15395537
SRR17520069.15395538
SRR17520069.15395539
SRR17520069.15395540
SRR17520069.15395541

SRR17520069.15395542
SRR17520069.15395543
SRR17520069.15395544
SRR17520069.15395545
SRR17520069.15395546
SRR17520069.15395547
SRR17520069.15395548
SRR17520069.15395549
SRR17520069.15395550
SRR17520069.15395551
SRR17520069.15395552
SRR17520069.15395554
SRR17520069.15395555
SRR17520069.15395556
SRR17520069.15395557
SRR17520069.15395558
SRR17520069.15395559
SRR17520069.15395562
SRR17520069.15395593
SRR17520069.15395628
SRR17520069.15395629
SRR17520069.15395630
SRR17520069.15395631
SRR17520069.15395632
SRR17520069.15395633
SRR17520069.15395634
SRR17520069.15395635
SRR17520069.15395636
SRR17520069.15395637
SRR17520069.15395638
SRR17520069.15395639
SRR17520069.15395640
SRR17520069.15395641
SRR17520069.15395642
SRR17520069.15395643
SRR17520069.15395644
SRR17520069.15395645
SRR17520069.15395646
SRR17520069.15400913
SRR17520069.15400914
SRR17520069.15400915
SRR17520069.15400916
SRR17520069.15400917
SRR17520069.15400918
SRR17520069.15400919
SRR17520069.15400920
SRR17520069.15400921

SRR17520069.15400922
SRR17520069.15400923
SRR17520069.15400924
SRR17520069.15400925
SRR17520069.15400926
SRR17520069.15400927
SRR17520069.15400928
SRR17520069.15400929
SRR17520069.15400930
SRR17520069.15400931
SRR17520069.15400932
SRR17520069.15400933
SRR17520069.15400934
SRR17520069.15400936
SRR17520069.15400937
SRR17520069.15400938
SRR17520069.15400939
SRR17520069.15400940
SRR17520069.15400941
SRR17520069.15400942
SRR17520069.15400943
SRR17520069.15400944
SRR17520069.15400945
SRR17520069.15400946
SRR17520069.15400947
SRR17520069.15400950
SRR17520069.15400951
SRR17520069.15400952
SRR17520069.15400953
SRR17520069.15400954
SRR17520069.15400955
SRR17520069.15400956
SRR17520069.15400957
SRR17520069.15400958
SRR17520069.15400959
SRR17520069.15400960
SRR17520069.15400961
SRR17520069.15400962
SRR17520069.15400963
SRR17520069.15400964
SRR17520069.15400965
SRR17520069.15400966
SRR17520069.15400967
SRR17520069.15400968
SRR17520069.15400969
SRR17520069.15400970
SRR17520069.15400971

SRR17520069.15400972
SRR17520069.15400973
SRR17520069.15400974
SRR17520069.15400975
SRR17520069.15400976
SRR17520069.15400977
SRR17520069.15400978
SRR17520069.15400979
SRR17520069.15400980
SRR17520069.15400981
SRR17520069.15400982
SRR17520069.15400983
SRR17520069.15400984
SRR17520069.15400985
SRR17520069.15400986
SRR17520069.15400987
SRR17520069.15402144
SRR17520069.15402145
SRR17520069.15402146
SRR17520069.15402147
SRR17520069.15402150
SRR17520069.15402151
SRR17520069.15402152
SRR17520069.15402153
SRR17520069.15402154
SRR17520069.15402155
SRR17520069.15402156
SRR17520069.15402157
SRR17520069.15402158
SRR17520069.15402160
SRR17520069.15402161
SRR17520069.15402163
SRR17520069.15402164
SRR17520069.15402165
SRR17520069.15402166
SRR17520069.15402167
SRR17520069.15402168
SRR17520069.15402169
SRR17520069.15402170
SRR17520069.15402171
SRR17520069.15402172
SRR17520069.15402173
SRR17520069.15402174
SRR17520069.15402175
SRR17520069.15402176
SRR17520069.15402177
SRR17520069.15402178

SRR17520069.15402179
SRR17520069.15402180
SRR17520069.15402181
SRR17520069.15402182
SRR17520069.15402184
SRR17520069.15402185
SRR17520069.15402186
SRR17520069.15402187
SRR17520069.15402188
SRR17520069.15402189
SRR17520069.15402190
SRR17520069.15402191
SRR17520069.15402192
SRR17520069.15402193
SRR17520069.15402194
SRR17520069.15402195
SRR17520069.15402196
SRR17520069.15402197
SRR17520069.15402198
SRR17520069.15402199
SRR17520069.15402200
SRR17520069.15402201
SRR17520069.15402202
SRR17520069.15402203
SRR17520069.15402204
SRR17520069.15402205
SRR17520069.15402207
SRR17520069.15402208
SRR17520069.15402210
SRR17520069.15402212
SRR17520069.15402213
SRR17520069.15402214
SRR17520069.15402215
SRR17520069.15402216
SRR17520069.15402217
SRR17520069.15402220
SRR17520069.15402221
SRR17520069.15402222
SRR17520069.15402223
SRR17520069.15402224
SRR17520069.15402225
SRR17520069.15402226
SRR17520069.15402227
SRR17520069.15402228
SRR17520069.15402229
SRR17520069.15402230
SRR17520069.15402231

SRR17520069.15402232
SRR17520069.15402233
SRR17520069.15402234
SRR17520069.15402235
SRR17520069.15402237
SRR17520069.15402238
SRR17520069.15402240
SRR17520069.15402241
SRR17520069.15402242
SRR17520069.15402243
SRR17520069.15402244
SRR17520069.15402245
SRR17520069.15402246
SRR17520069.15402247
SRR17520069.15402248
SRR17520069.15402249
SRR17520069.15402250
SRR17520069.15402251
SRR17520069.15402252
SRR17520069.15402253
SRR17520069.15402254
SRR17520069.15402256
SRR17520069.15402257
SRR17520069.15402258
SRR17520069.15402259
SRR17520069.15402260
SRR17520069.15402261
SRR17520069.15402262
SRR17520069.15402263
SRR17520069.15402264
SRR17520069.15402265
SRR17520069.15402266
SRR17520069.15402267
SRR17520069.15402269
SRR17520069.15402270
SRR17520069.15402271
SRR17520069.15402272
SRR17520069.15402273
SRR17520069.15402274
SRR17520069.15402275
SRR17520069.15402276
SRR17520069.15402277
SRR17520069.15402278
SRR17520069.15402279
SRR17520069.15402280
SRR17520069.15402281
SRR17520069.15402282

SRR17520069.15402283
SRR17520069.15402284
SRR17520069.15402285
SRR17520069.15402286
SRR17520069.15402287
SRR17520069.15402288
SRR17520069.15402290
SRR17520069.15402292
SRR17520069.15402293
SRR17520069.15402294
SRR17520069.15402295
SRR17520069.15402297
SRR17520069.15402298
SRR17520069.15402300
SRR17520069.15402301
SRR17520069.15402302
SRR17520069.15402303
SRR17520069.15402304
SRR17520069.15402305
SRR17520069.15406283
SRR17520069.15406284
SRR17520069.15415415
SRR17520069.15415416
SRR17520069.15415417
SRR17520069.15415418
SRR17520069.15415419
SRR17520069.15415420
SRR17520069.15415421
SRR17520069.15415422
SRR17520069.15415423
SRR17520069.15415424
SRR17520069.15415425
SRR17520069.15415426
SRR17520069.15415427
SRR17520069.15415428
SRR17520069.15415429
SRR17520069.15415430
SRR17520069.15415431
SRR17520069.15415432
SRR17520069.15415434
SRR17520069.15415435
SRR17520069.15415436
SRR17520069.15415437
SRR17520069.15415438
SRR17520069.15415439
SRR17520069.15415440
SRR17520069.15415441

SRR17520069.15415442
SRR17520069.15415443
SRR17520069.15415444
SRR17520069.15415445
SRR17520069.15415446
SRR17520069.15415447
SRR17520069.15415448
SRR17520069.15415450
SRR17520069.15415452
SRR17520069.15415453
SRR17520069.15415454
SRR17520069.15415455
SRR17520069.15415456
SRR17520069.15415457
SRR17520069.15415458
SRR17520069.15415459
SRR17520069.15415460
SRR17520069.15415461
SRR17520069.15415462
SRR17520069.15415463
SRR17520069.15415464
SRR17520069.15415465
SRR17520069.15415466
SRR17520069.15415467
SRR17520069.15415468
SRR17520069.15415469
SRR17520069.15424555
SRR17520069.15425205
SRR17520069.15425206
SRR17520069.15425207
SRR17520069.15425208
SRR17520069.15425209
SRR17520069.15425210
SRR17520069.15425211
SRR17520069.15425212
SRR17520069.15425213
SRR17520069.15425214
SRR17520069.15425215
SRR17520069.15425216
SRR17520069.15425217
SRR17520069.15425218
SRR17520069.15425219
SRR17520069.15425220
SRR17520069.15425221
SRR17520069.15425222
SRR17520069.15425223
SRR17520069.15425224

SRR17520069.15425225
SRR17520069.15425226
SRR17520069.15425227
SRR17520069.15425228
SRR17520069.15425229
SRR17520069.15425230
SRR17520069.15425231
SRR17520069.15425232
SRR17520069.15425233
SRR17520069.15425234
SRR17520069.15425235
SRR17520069.15425236
SRR17520069.15425237
SRR17520069.15431595
SRR17520069.15431596
SRR17520069.15431597
SRR17520069.15431598
SRR17520069.15431599
SRR17520069.15431600
SRR17520069.15431601
SRR17520069.15431602
SRR17520069.15431603
SRR17520069.15431604
SRR17520069.15431605
SRR17520069.15431606
SRR17520069.15431607
SRR17520069.15431608
SRR17520069.15431609
SRR17520069.15431610
SRR17520069.15431612
SRR17520069.15431613
SRR17520069.15431614
SRR17520069.15431615
SRR17520069.15437041
SRR17520069.15437042
SRR17520069.15437043
SRR17520069.15437044
SRR17520069.15437046
SRR17520069.15437047
SRR17520069.15437048
SRR17520069.15437051
SRR17520069.15437052
SRR17520069.15437053
SRR17520069.15437054
SRR17520069.15437055
SRR17520069.15437056
SRR17520069.15437057

SRR17520069.15437058
SRR17520069.15437059
SRR17520069.15437060
SRR17520069.15437061
SRR17520069.15437062
SRR17520069.15437063
SRR17520069.15437064
SRR17520069.15437065
SRR17520069.15437066
SRR17520069.15437067
SRR17520069.15437068
SRR17520069.15437069
SRR17520069.15437070
SRR17520069.15437071
SRR17520069.15437072
SRR17520069.15437073
SRR17520069.15437074
SRR17520069.15437075
SRR17520069.15437076
SRR17520069.15437077
SRR17520069.15437078
SRR17520069.15437079
SRR17520069.15441475
SRR17520069.15441476
SRR17520069.15441477
SRR17520069.15441478
SRR17520069.15441479
SRR17520069.15441480
SRR17520069.15441481
SRR17520069.15441482
SRR17520069.15441483
SRR17520069.15441484
SRR17520069.15441485
SRR17520069.15441486
SRR17520069.15441487
SRR17520069.15441488
SRR17520069.15441489
SRR17520069.15441490
SRR17520069.15441491
SRR17520069.15441492
SRR17520069.15441493
SRR17520069.15441494
SRR17520069.15441495
SRR17520069.15441496
SRR17520069.15441497
SRR17520069.15441498
SRR17520069.15441499

SRR17520069.15441500
SRR17520069.15441501
SRR17520069.15441502
SRR17520069.15441503
SRR17520069.15441504
SRR17520069.15441505
SRR17520069.15441506
SRR17520069.15441507
SRR17520069.15441508
SRR17520069.15441509
SRR17520069.15441510
SRR17520069.15441511
SRR17520069.15441512
SRR17520069.15441513
SRR17520069.15441514
SRR17520069.15441515
SRR17520069.15441516
SRR17520069.15441517
SRR17520069.15441518
SRR17520069.15441519
SRR17520069.15441520
SRR17520069.15441521
SRR17520069.15441522
SRR17520069.15441523
SRR17520069.15441524
SRR17520069.15441525
SRR17520069.15441526
SRR17520069.15441527
SRR17520069.15441528
SRR17520069.15441529
SRR17520069.15441530
SRR17520069.15441531
SRR17520069.15441532
SRR17520069.15441533
SRR17520069.15441535
SRR17520069.15441536
SRR17520069.15441537
SRR17520069.15441538
SRR17520069.15441539
SRR17520069.15441540
SRR17520069.15441541
SRR17520069.15441542
SRR17520069.15441543
SRR17520069.15441544
SRR17520069.15441545
SRR17520069.15441546
SRR17520069.15441547

SRR17520069.15441548
SRR17520069.15441549
SRR17520069.15441550
SRR17520069.15441551
SRR17520069.15441552
SRR17520069.15441553
SRR17520069.15441554
SRR17520069.15441555
SRR17520069.15441556
SRR17520069.15441557
SRR17520069.15441558
SRR17520069.15441559
SRR17520069.15441560
SRR17520069.15441561
SRR17520069.15441562
SRR17520069.15441563
SRR17520069.15441564
SRR17520069.15441565
SRR17520069.15441566
SRR17520069.15441567
SRR17520069.15441568
SRR17520069.15441569
SRR17520069.15441570
SRR17520069.15441571
SRR17520069.15441572
SRR17520069.15441573
SRR17520069.15441574
SRR17520069.15441575
SRR17520069.15441576
SRR17520069.15441577
SRR17520069.15441578
SRR17520069.15441579
SRR17520069.15441580
SRR17520069.15441581
SRR17520069.15441582
SRR17520069.15441583
SRR17520069.15441584
SRR17520069.15441585
SRR17520069.15441586
SRR17520069.15441587
SRR17520069.15441588
SRR17520069.15441589
SRR17520069.15441590
SRR17520069.15441591
SRR17520069.15441592
SRR17520069.15441593
SRR17520069.15441594

SRR17520069.15441595
SRR17520069.15441596
SRR17520069.15441597
SRR17520069.15441598
SRR17520069.15441599
SRR17520069.15441600
SRR17520069.15441601
SRR17520069.15441602
SRR17520069.15441603
SRR17520069.15441604
SRR17520069.15441605
SRR17520069.15441606
SRR17520069.15441607
SRR17520069.15441608
SRR17520069.15441609
SRR17520069.15441610
SRR17520069.15441611
SRR17520069.15441612
SRR17520069.15441613
SRR17520069.15441614
SRR17520069.15441615
SRR17520069.15441616
SRR17520069.15441617
SRR17520069.15441618
SRR17520069.15441619
SRR17520069.15441620
SRR17520069.15441621
SRR17520069.15441622
SRR17520069.15441623
SRR17520069.15441624
SRR17520069.15441625
SRR17520069.15441626
SRR17520069.15441627
SRR17520069.15441628
SRR17520069.15441629
SRR17520069.15441630
SRR17520069.15441631
SRR17520069.15441632
SRR17520069.15441633
SRR17520069.15441634
SRR17520069.15441635
SRR17520069.15441636
SRR17520069.15441637
SRR17520069.15441638
SRR17520069.15441639
SRR17520069.15441640
SRR17520069.15441642

SRR17520069.15441643
SRR17520069.15441644
SRR17520069.15441645
SRR17520069.15441646
SRR17520069.15441647
SRR17520069.15441648
SRR17520069.15441649
SRR17520069.15441650
SRR17520069.15441651
SRR17520069.15441652
SRR17520069.15441653
SRR17520069.15441654
SRR17520069.15441655
SRR17520069.15441656
SRR17520069.15441657
SRR17520069.15441658
SRR17520069.15441659
SRR17520069.15441660
SRR17520069.15441661
SRR17520069.15441662
SRR17520069.15441663
SRR17520069.15441664
SRR17520069.15441665
SRR17520069.15441666
SRR17520069.15441667
SRR17520069.15441668
SRR17520069.15441669
SRR17520069.15441670
SRR17520069.15441671
SRR17520069.15441672
SRR17520069.15441673
SRR17520069.15441674
SRR17520069.15441675
SRR17520069.15441676
SRR17520069.15441677
SRR17520069.15441678
SRR17520069.15441679
SRR17520069.15441680
SRR17520069.15441681
SRR17520069.15441682
SRR17520069.15441683
SRR17520069.15441685
SRR17520069.15441686
SRR17520069.15447884
SRR17520069.15447885
SRR17520069.15447886
SRR17520069.15447887

SRR17520069.15447888
SRR17520069.15447892
SRR17520069.15447893
SRR17520069.15447894
SRR17520069.15447895
SRR17520069.15447896
SRR17520069.15447897
SRR17520069.15447898
SRR17520069.15447899
SRR17520069.15447900
SRR17520069.15447901
SRR17520069.15447902
SRR17520069.15447903
SRR17520069.15447906
SRR17520069.15447907
SRR17520069.15447908
SRR17520069.15447909
SRR17520069.15447910
SRR17520069.15447911
SRR17520069.15447912
SRR17520069.15447913
SRR17520069.15447914
SRR17520069.15447915
SRR17520069.15447916
SRR17520069.15447917
SRR17520069.15447918
SRR17520069.15447919
SRR17520069.15447920
SRR17520069.15447921
SRR17520069.15447922
SRR17520069.15447923
SRR17520069.15447924
SRR17520069.15447925
SRR17520069.15447926
SRR17520069.15447927
SRR17520069.15447928
SRR17520069.15447929
SRR17520069.15447930
SRR17520069.15447931
SRR17520069.15447932
SRR17520069.15447933
SRR17520069.15447934
SRR17520069.15447935
SRR17520069.15447936
SRR17520069.15447937
SRR17520069.15447938
SRR17520069.15447939

SRR17520069.15447940
SRR17520069.15447941
SRR17520069.15447942
SRR17520069.15447943
SRR17520069.15447944
SRR17520069.15447945
SRR17520069.15447946
SRR17520069.15447947
SRR17520069.15447948
SRR17520069.15447949
SRR17520069.15447950
SRR17520069.15447951
SRR17520069.15447952
SRR17520069.15447953
SRR17520069.15447954
SRR17520069.15447955
SRR17520069.15447956
SRR17520069.15447957
SRR17520069.15447958
SRR17520069.15447959
SRR17520069.15447960
SRR17520069.15447961
SRR17520069.15447962
SRR17520069.15447963
SRR17520069.15447964
SRR17520069.15447965
SRR17520069.15447966
SRR17520069.15447967
SRR17520069.15447968
SRR17520069.15447969
SRR17520069.15447970
SRR17520069.15447971
SRR17520069.15447972
SRR17520069.15447973
SRR17520069.15447974
SRR17520069.15447975
SRR17520069.15447976
SRR17520069.15447977
SRR17520069.15447978
SRR17520069.15447979
SRR17520069.15447980
SRR17520069.15447981
SRR17520069.15447982
SRR17520069.15447983
SRR17520069.15447984
SRR17520069.15447985
SRR17520069.15447986

SRR17520069.15447987
SRR17520069.15447988
SRR17520069.15447989
SRR17520069.15447990
SRR17520069.15447991
SRR17520069.15447992
SRR17520069.15447993
SRR17520069.15447994
SRR17520069.15447995
SRR17520069.15447996
SRR17520069.15447997
SRR17520069.15447998
SRR17520069.15451785
SRR17520069.15452987
SRR17520069.15452988
SRR17520069.15452989
SRR17520069.15452991
SRR17520069.15452992
SRR17520069.15452993
SRR17520069.15452994
SRR17520069.15452995
SRR17520069.15452996
SRR17520069.15452997
SRR17520069.15452998
SRR17520069.15452999
SRR17520069.15453001
SRR17520069.15453003
SRR17520069.15453005
SRR17520069.15453007
SRR17520069.15453009
SRR17520069.15453011
SRR17520069.15453012
SRR17520069.15453013
SRR17520069.15453015
SRR17520069.15453017
SRR17520069.15453019
SRR17520069.15453021
SRR17520069.15454364
SRR17520069.15454365
SRR17520069.15454366
SRR17520069.15454367
SRR17520069.15454368
SRR17520069.15454369
SRR17520069.15454370
SRR17520069.15454371
SRR17520069.15454372
SRR17520069.15454373

SRR17520069.15454374
SRR17520069.15454375
SRR17520069.15454376
SRR17520069.15454377
SRR17520069.15454378
SRR17520069.15454379
SRR17520069.15454380
SRR17520069.15454381
SRR17520069.15454382
SRR17520069.15454383
SRR17520069.15454384
SRR17520069.15454385
SRR17520069.15454386
SRR17520069.15454387
SRR17520069.15454388
SRR17520069.15454389
SRR17520069.15454390
SRR17520069.15454391
SRR17520069.15454392
SRR17520069.15454393
SRR17520069.15454394
SRR17520069.15454395
SRR17520069.15454396
SRR17520069.15454397
SRR17520069.15454398
SRR17520069.15454399
SRR17520069.15454400
SRR17520069.15454401
SRR17520069.15454403
SRR17520069.15454404
SRR17520069.15454405
SRR17520069.15454406
SRR17520069.15454407
SRR17520069.15454408
SRR17520069.15454409
SRR17520069.15454410
SRR17520069.15454411
SRR17520069.15454412
SRR17520069.15454413
SRR17520069.15454414
SRR17520069.15454415
SRR17520069.15454416
SRR17520069.15454417
SRR17520069.15454418
SRR17520069.15454419
SRR17520069.15454420
SRR17520069.15454421

SRR17520069.15454422
SRR17520069.15454423
SRR17520069.15454424
SRR17520069.15454425
SRR17520069.15454426
SRR17520069.15454427
SRR17520069.15454428
SRR17520069.15454429
SRR17520069.15454430
SRR17520069.15454431
SRR17520069.15454432
SRR17520069.15454433
SRR17520069.15454434
SRR17520069.15454435
SRR17520069.15454436
SRR17520069.15454437
SRR17520069.15454438
SRR17520069.15454439
SRR17520069.15454440
SRR17520069.15454441
SRR17520069.15454442
SRR17520069.15454445
SRR17520069.15454446
SRR17520069.15454447
SRR17520069.15454448
SRR17520069.15454449
SRR17520069.15454450
SRR17520069.15454451
SRR17520069.15454452
SRR17520069.15454453
SRR17520069.15454454
SRR17520069.15454455
SRR17520069.15454456
SRR17520069.15454458
SRR17520069.15454459
SRR17520069.15454460
SRR17520069.15454461
SRR17520069.15454462
SRR17520069.15454463
SRR17520069.15454464
SRR17520069.15454465
SRR17520069.15454466
SRR17520069.15454467
SRR17520069.15454468
SRR17520069.15454469
SRR17520069.15454470
SRR17520069.15454471

SRR17520069.15454472
SRR17520069.15454473
SRR17520069.15454474
SRR17520069.15454475
SRR17520069.15454476
SRR17520069.15454477
SRR17520069.15454479
SRR17520069.15454480
SRR17520069.15454481
SRR17520069.15454482
SRR17520069.15454483
SRR17520069.15454484
SRR17520069.15454485
SRR17520069.15454486
SRR17520069.15454487
SRR17520069.15454488
SRR17520069.15454489
SRR17520069.15454490
SRR17520069.15454491
SRR17520069.15454492
SRR17520069.15454493
SRR17520069.15454494
SRR17520069.15454495
SRR17520069.15454496
SRR17520069.15454497
SRR17520069.15454498
SRR17520069.15454499
SRR17520069.15454500
SRR17520069.15454501
SRR17520069.15454502
SRR17520069.15454503
SRR17520069.15454504
SRR17520069.15454505
SRR17520069.15454506
SRR17520069.15454507
SRR17520069.15454508
SRR17520069.15454509
SRR17520069.15454510
SRR17520069.15454511
SRR17520069.15454512
SRR17520069.15454513
SRR17520069.15454514
SRR17520069.15454515
SRR17520069.15454516
SRR17520069.15454517
SRR17520069.15454518
SRR17520069.15454519

SRR17520069.15454520
SRR17520069.15454521
SRR17520069.15454522
SRR17520069.15454523
SRR17520069.15454524
SRR17520069.15454525
SRR17520069.15454526
SRR17520069.15454527
SRR17520069.15454528
SRR17520069.15454529
SRR17520069.15454530
SRR17520069.15454531
SRR17520069.15454532
SRR17520069.15454533
SRR17520069.15454534
SRR17520069.15454535
SRR17520069.15456852
SRR17520069.15456853
SRR17520069.15456854
SRR17520069.15456855
SRR17520069.15456856
SRR17520069.15456857
SRR17520069.15456858
SRR17520069.15456859
SRR17520069.15456860
SRR17520069.15456861
SRR17520069.15456862
SRR17520069.15456863
SRR17520069.15456864
SRR17520069.15456865
SRR17520069.15456866
SRR17520069.15456867
SRR17520069.15456868
SRR17520069.15456869
SRR17520069.15456870
SRR17520069.15456871
SRR17520069.15456872
SRR17520069.15456873
SRR17520069.15456874
SRR17520069.15456875
SRR17520069.15456876
SRR17520069.15456877
SRR17520069.15456878
SRR17520069.15456879
SRR17520069.15456880
SRR17520069.15456881
SRR17520069.15456882

SRR17520069.15534036
SRR17520069.15534037
SRR17520069.15534038
SRR17520069.15534039
SRR17520069.15534040
SRR17520069.15534041
SRR17520069.15534042
SRR17520069.15534043
SRR17520069.15534044
SRR17520069.15534045
SRR17520069.15534046
SRR17520069.15534048
SRR17520069.15534050
SRR17520069.15534051
SRR17520069.15534052
SRR17520069.15534054
SRR17520069.15534055
SRR17520069.15534056
SRR17520069.15544312
SRR17520069.15553468
SRR17520069.15553469
SRR17520069.15553483
SRR17520069.15564416
SRR17520069.15564418
SRR17520069.15564419
SRR17520069.15564420
SRR17520069.15564421
SRR17520069.15564422
SRR17520069.15564423
SRR17520069.15564424
SRR17520069.15564425
SRR17520069.15564426
SRR17520069.15564427
SRR17520069.15564428
SRR17520069.15564429
SRR17520069.15564430
SRR17520069.15564431
SRR17520069.15564432
SRR17520069.15564433
SRR17520069.15564434
SRR17520069.15564435
SRR17520069.15564436
SRR17520069.15564437
SRR17520069.15564438
SRR17520069.15564440
SRR17520069.15564441
SRR17520069.15564442

SRR17520069.15564444
SRR17520069.15564445
SRR17520069.15565539
SRR17520069.15565540
SRR17520069.15565541
SRR17520069.15565542
SRR17520069.15565545
SRR17520069.15565546
SRR17520069.15565547
SRR17520069.15565548
SRR17520069.15565549
SRR17520069.15565550
SRR17520069.15565551
SRR17520069.15565552
SRR17520069.15565553
SRR17520069.15565554
SRR17520069.15565555
SRR17520069.15565556
SRR17520069.15565557
SRR17520069.15565558
SRR17520069.15565559
SRR17520069.15565560
SRR17520069.15565561
SRR17520069.15565562
SRR17520069.15565563
SRR17520069.15565564
SRR17520069.15565565
SRR17520069.15565566
SRR17520069.15565567
SRR17520069.15565568
SRR17520069.15565569
SRR17520069.15565570
SRR17520069.15565571
SRR17520069.15565572
SRR17520069.15565573
SRR17520069.15565574
SRR17520069.15565575
SRR17520069.15565576
SRR17520069.15565577
SRR17520069.15565578
SRR17520069.15565579
SRR17520069.15565580
SRR17520069.15565581
SRR17520069.15565582
SRR17520069.15565583
SRR17520069.15565584
SRR17520069.15565585

SRR17520069.15565586
SRR17520069.15565587
SRR17520069.15565588
SRR17520069.15565589
SRR17520069.15565590
SRR17520069.15565592
SRR17520069.15565593
SRR17520069.15565594
SRR17520069.15565595
SRR17520069.15565596
SRR17520069.15565597
SRR17520069.15565598
SRR17520069.15565599
SRR17520069.15565600
SRR17520069.15565601
SRR17520069.15565602
SRR17520069.15567423
SRR17520069.15574885
SRR17520069.15577641
SRR17520069.15577663
SRR17520069.15577705
SRR17520069.15590184
SRR17520069.15621229
SRR17520069.15621230
SRR17520069.15621231
SRR17520069.15621232
SRR17520069.15621233
SRR17520069.15621234
SRR17520069.15621235
SRR17520069.15621236
SRR17520069.15621237
SRR17520069.15621238
SRR17520069.15621239
SRR17520069.15621240
SRR17520069.15621241
SRR17520069.15621242
SRR17520069.15621243
SRR17520069.15621244
SRR17520069.15621245
SRR17520069.15621246
SRR17520069.15628353
SRR17520069.15628354
SRR17520069.15628355
SRR17520069.15628356
SRR17520069.15628357
SRR17520069.15628358
SRR17520069.15628360

SRR17520069.15628361
SRR17520069.15628362
SRR17520069.15628363
SRR17520069.15628364
SRR17520069.15628365
SRR17520069.15628366
SRR17520069.15628367
SRR17520069.15628369
SRR17520069.15640147
SRR17520069.15645418
SRR17520069.15645419
SRR17520069.15645420
SRR17520069.15645421
SRR17520069.15645422
SRR17520069.15645423
SRR17520069.15645424
SRR17520069.15645425
SRR17520069.15645426
SRR17520069.15645427
SRR17520069.15665104
SRR17520069.15683067
SRR17520069.15683070
SRR17520069.15684176
SRR17520069.15684177
SRR17520069.15684179
SRR17520069.15684180
SRR17520069.15684181
SRR17520069.15684182
SRR17520069.15684183
SRR17520069.15684184
SRR17520069.15684185
SRR17520069.15684187
SRR17520069.15684188
SRR17520069.15684189
SRR17520069.15684190
SRR17520069.15684191
SRR17520069.15684192
SRR17520069.15684193
SRR17520069.15684194
SRR17520069.15684195
SRR17520069.15684196
SRR17520069.15684197
SRR17520069.15684199
SRR17520069.15684200
SRR17520069.15684201
SRR17520069.15684202
SRR17520069.15684203

SRR17520069.15684204
SRR17520069.15684205
SRR17520069.15684206
SRR17520069.15684207
SRR17520069.15684208
SRR17520069.15684209
SRR17520069.15684210
SRR17520069.15684211
SRR17520069.15684212
SRR17520069.15684213
SRR17520069.15684214
SRR17520069.15684215
SRR17520069.15684216
SRR17520069.15684217
SRR17520069.15684218
SRR17520069.15684219
SRR17520069.15684220
SRR17520069.15684221
SRR17520069.15684222
SRR17520069.15684223
SRR17520069.15684224
SRR17520069.15684225
SRR17520069.15684226
SRR17520069.15684228
SRR17520069.15684229
SRR17520069.15684230
SRR17520069.15684231
SRR17520069.15684232
SRR17520069.15684233
SRR17520069.15684234
SRR17520069.15684235
SRR17520069.15684237
SRR17520069.15684238
SRR17520069.15684239
SRR17520069.15684240
SRR17520069.15684954
SRR17520069.15691700
SRR17520069.15691713
SRR17520069.15704561
SRR17520069.15714865
SRR17520069.15714872
SRR17520069.15714881
SRR17520069.15714887
SRR17520069.15737138
SRR17520069.15737139
SRR17520069.15737140
SRR17520069.15737141

SRR17520069.15737142
SRR17520069.15737143
SRR17520069.15737144
SRR17520069.15737145
SRR17520069.15737146
SRR17520069.15737147
SRR17520069.15737148
SRR17520069.15737149
SRR17520069.15737150
SRR17520069.15737151
SRR17520069.15737152
SRR17520069.15737153
SRR17520069.15737154
SRR17520069.15737155
SRR17520069.15737156
SRR17520069.15737157
SRR17520069.15737158
SRR17520069.15737159
SRR17520069.15737160
SRR17520069.15737161
SRR17520069.15737162
SRR17520069.15737163
SRR17520069.15737164
SRR17520069.15737165
SRR17520069.15737166
SRR17520069.15737167
SRR17520069.15737168
SRR17520069.15737169
SRR17520069.15737170
SRR17520069.15737171
SRR17520069.15737172
SRR17520069.15737173
SRR17520069.15737174
SRR17520069.15737175
SRR17520069.15737176
SRR17520069.15737177
SRR17520069.15737178
SRR17520069.15737179
SRR17520069.15737180
SRR17520069.15737181
SRR17520069.15737183
SRR17520069.15737184
SRR17520069.15737185
SRR17520069.15737186
SRR17520069.15737187
SRR17520069.15737188
SRR17520069.15737189

SRR17520069.15737190
SRR17520069.15737191
SRR17520069.15737192
SRR17520069.15737193
SRR17520069.15737194
SRR17520069.15752682
SRR17520069.15761701
SRR17520069.15761702
SRR17520069.15761703
SRR17520069.15761704
SRR17520069.15761705
SRR17520069.15761707
SRR17520069.15761708
SRR17520069.15770869
SRR17520069.15772333
SRR17520069.15772334
SRR17520069.15772335
SRR17520069.15772336
SRR17520069.15772337
SRR17520069.15772338
SRR17520069.15772339
SRR17520069.15772340
SRR17520069.15772341
SRR17520069.15772342
SRR17520069.15772343
SRR17520069.15772344
SRR17520069.15772345
SRR17520069.15772346
SRR17520069.15772347
SRR17520069.15772348
SRR17520069.15772349
SRR17520069.15772350
SRR17520069.15772351
SRR17520069.15772352
SRR17520069.15772353
SRR17520069.15772354
SRR17520069.15772355
SRR17520069.15772356
SRR17520069.15772357
SRR17520069.15772358
SRR17520069.15772359
SRR17520069.15772360
SRR17520069.15772361
SRR17520069.15772362
SRR17520069.15772363
SRR17520069.15772364
SRR17520069.15772365

SRR17520069.15772366
SRR17520069.15772367
SRR17520069.15772368
SRR17520069.15772369
SRR17520069.15772370
SRR17520069.15772371
SRR17520069.15772373
SRR17520069.15772374
SRR17520069.15772375
SRR17520069.15772376
SRR17520069.15772377
SRR17520069.15772378
SRR17520069.15792398
SRR17520069.15808752
SRR17520069.15808803
SRR17520069.15818692
SRR17520069.15833927
SRR17520069.15838037
SRR17520069.15838038
SRR17520069.15838039
SRR17520069.15838040
SRR17520069.15838041
SRR17520069.15838042
SRR17520069.15838043
SRR17520069.15838044
SRR17520069.15838045
SRR17520069.15838046
SRR17520069.15838047
SRR17520069.15838048
SRR17520069.15838049
SRR17520069.15838050
SRR17520069.15838051
SRR17520069.15838052
SRR17520069.15847234
SRR17520069.15847235
SRR17520069.15847236
SRR17520069.15847237
SRR17520069.15847239
SRR17520069.15847240
SRR17520069.15847241
SRR17520069.15848316
SRR17520069.15848317
SRR17520069.15848318
SRR17520069.15848319
SRR17520069.15848320
SRR17520069.15848321
SRR17520069.15848322

SRR17520069.15848323
SRR17520069.15848324
SRR17520069.15848325
SRR17520069.15848326
SRR17520069.15848327
SRR17520069.15848328
SRR17520069.15848329
SRR17520069.15848330
SRR17520069.15848331
SRR17520069.15848332
SRR17520069.15848333
SRR17520069.15848334
SRR17520069.15848335
SRR17520069.15848336
SRR17520069.15848337
SRR17520069.15848338
SRR17520069.15848339
SRR17520069.15848340
SRR17520069.15848341
SRR17520069.15848342
SRR17520069.15848343
SRR17520069.15848344
SRR17520069.15848345
SRR17520069.15848346
SRR17520069.15848347
SRR17520069.15848348
SRR17520069.15848349
SRR17520069.15848351
SRR17520069.15848352
SRR17520069.15848353
SRR17520069.15848354
SRR17520069.15848355
SRR17520069.15848356
SRR17520069.15848357
SRR17520069.15848358
SRR17520069.15848359
SRR17520069.15848360
SRR17520069.15848361
SRR17520069.15848362
SRR17520069.15848363
SRR17520069.15848364
SRR17520069.15848365
SRR17520069.15848366
SRR17520069.15848367
SRR17520069.15848368
SRR17520069.15848369
SRR17520069.15848370

SRR17520069.15848371
SRR17520069.15848372
SRR17520069.15848373
SRR17520069.15848374
SRR17520069.15848375
SRR17520069.15848376
SRR17520069.15848377
SRR17520069.15848378
SRR17520069.15848379
SRR17520069.15848380
SRR17520069.15848381
SRR17520069.15848382
SRR17520069.15848383
SRR17520069.15848384
SRR17520069.15848385
SRR17520069.15848386
SRR17520069.15848387
SRR17520069.15848388
SRR17520069.15848389
SRR17520069.15848390
SRR17520069.15848391
SRR17520069.15848392
SRR17520069.15848393
SRR17520069.15848394
SRR17520069.15848395
SRR17520069.15848396
SRR17520069.15848397
SRR17520069.15848398
SRR17520069.15848399
SRR17520069.15848400
SRR17520069.15848401
SRR17520069.15848402
SRR17520069.15848403
SRR17520069.15848404
SRR17520069.15848405
SRR17520069.15848406
SRR17520069.15848407
SRR17520069.15848408
SRR17520069.15848409
SRR17520069.15848410
SRR17520069.15848411
SRR17520069.15848412
SRR17520069.15848413
SRR17520069.15848414
SRR17520069.15848415
SRR17520069.15848416
SRR17520069.15848417

SRR17520069.15848418
SRR17520069.15848419
SRR17520069.15848420
SRR17520069.15848421
SRR17520069.15848422
SRR17520069.15848423
SRR17520069.15848424
SRR17520069.15848425
SRR17520069.15848426
SRR17520069.15848428
SRR17520069.15848429
SRR17520069.15848430
SRR17520069.15848431
SRR17520069.15848432
SRR17520069.15848433
SRR17520069.15848434
SRR17520069.15848435
SRR17520069.15848436
SRR17520069.15848437
SRR17520069.15848438
SRR17520069.15848439
SRR17520069.15848440
SRR17520069.15848441
SRR17520069.15853788
SRR17520069.15853789
SRR17520069.15856460
SRR17520069.15880169
SRR17520069.15880751
SRR17520069.15880752
SRR17520069.15880753
SRR17520069.15880754
SRR17520069.15880755
SRR17520069.15880756
SRR17520069.15880757
SRR17520069.15880758
SRR17520069.15880759
SRR17520069.15880760
SRR17520069.15880761
SRR17520069.15880762
SRR17520069.15880763
SRR17520069.15880764
SRR17520069.15880765
SRR17520069.15880766
SRR17520069.15880767
SRR17520069.15880768
SRR17520069.15880769
SRR17520069.15880770

SRR17520069.15880771
SRR17520069.15880772
SRR17520069.15880773
SRR17520069.15880774
SRR17520069.15880775
SRR17520069.15880776
SRR17520069.15880777
SRR17520069.15880778
SRR17520069.15880779
SRR17520069.15880780
SRR17520069.15880781
SRR17520069.15880782
SRR17520069.15880783
SRR17520069.15880784
SRR17520069.15892363
SRR17520069.15892364
SRR17520069.15892365
SRR17520069.15892366
SRR17520069.15892367
SRR17520069.15892770
SRR17520069.15892771
SRR17520069.15892772
SRR17520069.15899622
SRR17520069.15914908
SRR17520069.15914918
SRR17520069.15914926
SRR17520069.15920827
SRR17520069.15938405
SRR17520069.15941825
SRR17520069.15941826
SRR17520069.15941827
SRR17520069.15941828
SRR17520069.15941829
SRR17520069.15941830
SRR17520069.15941831
SRR17520069.15946459
SRR17520069.15946460
SRR17520069.15946461
SRR17520069.15946462
SRR17520069.15946463
SRR17520069.15946464
SRR17520069.15946465
SRR17520069.15946466
SRR17520069.15946467
SRR17520069.15946468
SRR17520069.15946469
SRR17520069.15946470

SRR17520069.15946471
SRR17520069.15949921
SRR17520069.15955638
SRR17520069.15955639
SRR17520069.15975343
SRR17520069.15997591
SRR17520069.15997592
SRR17520069.15997593
SRR17520069.15997594
SRR17520069.15997595
SRR17520069.15997597
SRR17520069.15997598
SRR17520069.15997599
SRR17520069.15997600
SRR17520069.15997601
SRR17520069.15997602
SRR17520069.15997603
SRR17520069.15997604
SRR17520069.15997605
SRR17520069.15997606
SRR17520069.15997607
SRR17520069.15997609
SRR17520069.15997610
SRR17520069.15997611
SRR17520069.15997612
SRR17520069.15997613
SRR17520069.15997614
SRR17520069.15997615
SRR17520069.15997616
SRR17520069.15997617
SRR17520069.15997618
SRR17520069.15997619
SRR17520069.15997620
SRR17520069.15997621
SRR17520069.15997622
SRR17520069.15997623
SRR17520069.15997624
SRR17520069.15997625
SRR17520069.15997626
SRR17520069.15997627
SRR17520069.15997628
SRR17520069.15997629
SRR17520069.16001539
SRR17520069.16001574
SRR17520069.16001594
SRR17520069.16002712
SRR17520069.16011254

SRR17520069.16021801
SRR17520069.16021837
SRR17520069.16026474
SRR17520069.16026475
SRR17520069.16026476
SRR17520069.16026478
SRR17520069.16026479
SRR17520069.16026480
SRR17520069.16026481
SRR17520069.16026482
SRR17520069.16026483
SRR17520069.16026484
SRR17520069.16026486
SRR17520069.16026487
SRR17520069.16026488
SRR17520069.16026490
SRR17520069.16026491
SRR17520069.16026492
SRR17520069.16026495
SRR17520069.16026496
SRR17520069.16026497
SRR17520069.16026498
SRR17520069.16026499
SRR17520069.16026500
SRR17520069.16045244
SRR17520069.16054492
SRR17520069.16054493
SRR17520069.16054494
SRR17520069.16054495
SRR17520069.16066861
SRR17520069.16066862
SRR17520069.16066863
SRR17520069.16066864
SRR17520069.16066866
SRR17520069.16066867
SRR17520069.16066868
SRR17520069.16066869
SRR17520069.16066870
SRR17520069.16066871
SRR17520069.16066872
SRR17520069.16066873
SRR17520069.16066874
SRR17520069.16066875
SRR17520069.16066876
SRR17520069.16066877
SRR17520069.16066878
SRR17520069.16066879

SRR17520069.16066880
SRR17520069.16066881
SRR17520069.16066882
SRR17520069.16066883
SRR17520069.16066884
SRR17520069.16066885
SRR17520069.16066886
SRR17520069.16066887
SRR17520069.16066888
SRR17520069.16066889
SRR17520069.16066890
SRR17520069.16066891
SRR17520069.16066892
SRR17520069.16066893
SRR17520069.16066894
SRR17520069.16066896
SRR17520069.16066897
SRR17520069.16066898
SRR17520069.16066899
SRR17520069.16066900
SRR17520069.16066901
SRR17520069.16066902
SRR17520069.16066903
SRR17520069.16066904
SRR17520069.16066905
SRR17520069.16066906
SRR17520069.16066907
SRR17520069.16066908
SRR17520069.16066909
SRR17520069.16066910
SRR17520069.16066911
SRR17520069.16066912
SRR17520069.16066913
SRR17520069.16066914
SRR17520069.16066915
SRR17520069.16066916
SRR17520069.16066917
SRR17520069.16066918
SRR17520069.16066919
SRR17520069.16066920
SRR17520069.16066921
SRR17520069.16066923
SRR17520069.16066924
SRR17520069.16066925
SRR17520069.16066926
SRR17520069.16066927
SRR17520069.16066928

SRR17520069.16066929
SRR17520069.16066930
SRR17520069.16066931
SRR17520069.16066932
SRR17520069.16066933
SRR17520069.16066934
SRR17520069.16066935
SRR17520069.16066936
SRR17520069.16066937
SRR17520069.16066938
SRR17520069.16066939
SRR17520069.16066940
SRR17520069.16066941
SRR17520069.16066942
SRR17520069.16066943
SRR17520069.16066944
SRR17520069.16066945
SRR17520069.16066946
SRR17520069.16066947
SRR17520069.16066948
SRR17520069.16066949
SRR17520069.16066950
SRR17520069.16066951
SRR17520069.16066952
SRR17520069.16066953
SRR17520069.16066954
SRR17520069.16066955
SRR17520069.16066956
SRR17520069.16066957
SRR17520069.16066958
SRR17520069.16066959
SRR17520069.16066961
SRR17520069.16066962
SRR17520069.16066963
SRR17520069.16066964
SRR17520069.16066965
SRR17520069.16066966
SRR17520069.16066967
SRR17520069.16066968
SRR17520069.16066969
SRR17520069.16066970
SRR17520069.16066971
SRR17520069.16066974
SRR17520069.16075722
SRR17520069.16075723
SRR17520069.16075724
SRR17520069.16075725

SRR17520069.16075726
SRR17520069.16075727
SRR17520069.16075728
SRR17520069.16075729
SRR17520069.16075730
SRR17520069.16075731
SRR17520069.16075732
SRR17520069.16075733
SRR17520069.16075734
SRR17520069.16075735
SRR17520069.16075736
SRR17520069.16075737
SRR17520069.16075738
SRR17520069.16075739
SRR17520069.16075740
SRR17520069.16075741
SRR17520069.16075742
SRR17520069.16075743
SRR17520069.16075744
SRR17520069.16075745
SRR17520069.16075746
SRR17520069.16075747
SRR17520069.16075748
SRR17520069.16075750
SRR17520069.16075751
SRR17520069.16075752
SRR17520069.16075753
SRR17520069.16075754
SRR17520069.16075755
SRR17520069.16075756
SRR17520069.16075757
SRR17520069.16075758
SRR17520069.16075759
SRR17520069.16075760
SRR17520069.16075763
SRR17520069.16075764
SRR17520069.16077928
SRR17520069.16082686
SRR17520069.16086467
SRR17520069.16086468
SRR17520069.16086469
SRR17520069.16086470
SRR17520069.16086471
SRR17520069.16086472
SRR17520069.16086473
SRR17520069.16086474
SRR17520069.16086475

SRR17520069.16086476
SRR17520069.16086477
SRR17520069.16086478
SRR17520069.16086479
SRR17520069.16086480
SRR17520069.16086481
SRR17520069.16086482
SRR17520069.16086483
SRR17520069.16086484
SRR17520069.16086485
SRR17520069.16086486
SRR17520069.16086487
SRR17520069.16086488
SRR17520069.16086489
SRR17520069.16086490
SRR17520069.16086491
SRR17520069.16086492
SRR17520069.16086493
SRR17520069.16086494
SRR17520069.16086495
SRR17520069.16086496
SRR17520069.16086497
SRR17520069.16086498
SRR17520069.16086499
SRR17520069.16086500
SRR17520069.16086501
SRR17520069.16086502
SRR17520069.16086503
SRR17520069.16086504
SRR17520069.16086505
SRR17520069.16086506
SRR17520069.16086507
SRR17520069.16086508
SRR17520069.16086509
SRR17520069.16086510
SRR17520069.16086511
SRR17520069.16086512
SRR17520069.16086513
SRR17520069.16086514
SRR17520069.16086515
SRR17520069.16086516
SRR17520069.16086517
SRR17520069.16086518
SRR17520069.16086519
SRR17520069.16086520
SRR17520069.16086521
SRR17520069.16086522

SRR17520069.16086523
SRR17520069.16086524
SRR17520069.16086525
SRR17520069.16086526
SRR17520069.16086527
SRR17520069.16086529
SRR17520069.16086530
SRR17520069.16086531
SRR17520069.16086532
SRR17520069.16086533
SRR17520069.16086534
SRR17520069.16086535
SRR17520069.16086536
SRR17520069.16086537
SRR17520069.16086538
SRR17520069.16086539
SRR17520069.16086540
SRR17520069.16086541
SRR17520069.16086542
SRR17520069.16086543
SRR17520069.16086544
SRR17520069.16086545
SRR17520069.16086546
SRR17520069.16086547
SRR17520069.16086548
SRR17520069.16086549
SRR17520069.16086550
SRR17520069.16086551
SRR17520069.16086552
SRR17520069.16086553
SRR17520069.16086554
SRR17520069.16086555
SRR17520069.16090046
SRR17520069.16090047
SRR17520069.16090048
SRR17520069.16090049
SRR17520069.16090050
SRR17520069.16090051
SRR17520069.16090052
SRR17520069.16090053
SRR17520069.16090054
SRR17520069.16090055
SRR17520069.16090056
SRR17520069.16090057
SRR17520069.16090058
SRR17520069.16090059
SRR17520069.16090060

SRR17520069.16090061
SRR17520069.16090062
SRR17520069.16090063
SRR17520069.16090064
SRR17520069.16090065
SRR17520069.16090066
SRR17520069.16090067
SRR17520069.16090068
SRR17520069.16090069
SRR17520069.16090070
SRR17520069.16090071
SRR17520069.16090072
SRR17520069.16090073
SRR17520069.16090074
SRR17520069.16090075
SRR17520069.16090076
SRR17520069.16090077
SRR17520069.16090078
SRR17520069.16090080
SRR17520069.16090081
SRR17520069.16090082
SRR17520069.16090083
SRR17520069.16090084
SRR17520069.16090085
SRR17520069.16090086
SRR17520069.16090087
SRR17520069.16090088
SRR17520069.16090089
SRR17520069.16090090
SRR17520069.16090091
SRR17520069.16090092
SRR17520069.16090093
SRR17520069.16090094
SRR17520069.16090095
SRR17520069.16090096
SRR17520069.16090097
SRR17520069.16090098
SRR17520069.16090099
SRR17520069.16090100
SRR17520069.16090101
SRR17520069.16090102
SRR17520069.16090103
SRR17520069.16090104
SRR17520069.16090105
SRR17520069.16090106
SRR17520069.16090107
SRR17520069.16090108

SRR17520069.16090109
SRR17520069.16090110
SRR17520069.16090111
SRR17520069.16090112
SRR17520069.16090113
SRR17520069.16090114
SRR17520069.16090115
SRR17520069.16090116
SRR17520069.16090118
SRR17520069.16090119
SRR17520069.16090120
SRR17520069.16090121
SRR17520069.16090122
SRR17520069.16090123
SRR17520069.16090124
SRR17520069.16090125
SRR17520069.16090126
SRR17520069.16090127
SRR17520069.16090128
SRR17520069.16090129
SRR17520069.16090130
SRR17520069.16090131
SRR17520069.16090132
SRR17520069.16090133
SRR17520069.16090134
SRR17520069.16090135
SRR17520069.16090136
SRR17520069.16090137
SRR17520069.16090138
SRR17520069.16090140
SRR17520069.16090141
SRR17520069.16090142
SRR17520069.16090143
SRR17520069.16090144
SRR17520069.16090145
SRR17520069.16090146
SRR17520069.16090147
SRR17520069.16090148
SRR17520069.16090150
SRR17520069.16090151
SRR17520069.16090152
SRR17520069.16090153
SRR17520069.16090154
SRR17520069.16090155
SRR17520069.16090156
SRR17520069.16090157
SRR17520069.16090158

SRR17520069.16090159
SRR17520069.16090160
SRR17520069.16090161
SRR17520069.16090162
SRR17520069.16090163
SRR17520069.16090164
SRR17520069.16090165
SRR17520069.16091607
SRR17520069.16094462
SRR17520069.16105679
SRR17520069.16105680
SRR17520069.16105681
SRR17520069.16105682
SRR17520069.16105683
SRR17520069.16105684
SRR17520069.16105685
SRR17520069.16105686
SRR17520069.16105687
SRR17520069.16105688
SRR17520069.16105689
SRR17520069.16105690
SRR17520069.16105691
SRR17520069.16105692
SRR17520069.16105693
SRR17520069.16105694
SRR17520069.16105695
SRR17520069.16105696
SRR17520069.16105697
SRR17520069.16105715
SRR17520069.16134708
SRR17520069.16134709
SRR17520069.16134710
SRR17520069.16147763
SRR17520069.16151223
SRR17520069.16157772
SRR17520069.16169153
SRR17520069.16190010
SRR17520069.16190011
SRR17520069.16190012
SRR17520069.16190013
SRR17520069.16190014
SRR17520069.16190015
SRR17520069.16190016
SRR17520069.16190017
SRR17520069.16190018
SRR17520069.16190019
SRR17520069.16190020

SRR17520069.16190022
SRR17520069.16190023
SRR17520069.16190024
SRR17520069.16190025
SRR17520069.16190026
SRR17520069.16190027
SRR17520069.16190028
SRR17520069.16190029
SRR17520069.16190031
SRR17520069.16190032
SRR17520069.16190033
SRR17520069.16190035
SRR17520069.16190036
SRR17520069.16190037
SRR17520069.16190038
SRR17520069.16190039
SRR17520069.16190041
SRR17520069.16190044
SRR17520069.16190045
SRR17520069.16190047
SRR17520069.16190048
SRR17520069.16190049
SRR17520069.16190054
SRR17520069.16190059
SRR17520069.16190061
SRR17520069.16190062
SRR17520069.16190064
SRR17520069.16190065
SRR17520069.16190066
SRR17520069.16190067
SRR17520069.16190068
SRR17520069.16190069
SRR17520069.16190070
SRR17520069.16190071
SRR17520069.16190072
SRR17520069.16190073
SRR17520069.16190074
SRR17520069.16190075
SRR17520069.16190076
SRR17520069.16190077
SRR17520069.16190078
SRR17520069.16190079
SRR17520069.16190080
SRR17520069.16190081
SRR17520069.16190082
SRR17520069.16190083
SRR17520069.16190084

SRR17520069.16190085
SRR17520069.16190086
SRR17520069.16190088
SRR17520069.16190089
SRR17520069.16190090
SRR17520069.16190093
SRR17520069.16190094
SRR17520069.16190095
SRR17520069.16190096
SRR17520069.16190097
SRR17520069.16190098
SRR17520069.16190099
SRR17520069.16190101
SRR17520069.16190103
SRR17520069.16190104
SRR17520069.16190105
SRR17520069.16190108
SRR17520069.16190109
SRR17520069.16190110
SRR17520069.16190111
SRR17520069.16190112
SRR17520069.16190113
SRR17520069.16190114
SRR17520069.16190117
SRR17520069.16190121
SRR17520069.16190122
SRR17520069.16190126
SRR17520069.16190128
SRR17520069.16191608
SRR17520069.16191609
SRR17520069.16191611
SRR17520069.16191612
SRR17520069.16191613
SRR17520069.16191614
SRR17520069.16191615
SRR17520069.16191616
SRR17520069.16191617
SRR17520069.16191618
SRR17520069.16191619
SRR17520069.16191620
SRR17520069.16191621
SRR17520069.16191622
SRR17520069.16191623
SRR17520069.16191624
SRR17520069.16191625
SRR17520069.16191626
SRR17520069.16191627

SRR17520069.16191628
SRR17520069.16191629
SRR17520069.16191631
SRR17520069.16193461
SRR17520069.16193462
SRR17520069.16193463
SRR17520069.16193464
SRR17520069.16193465
SRR17520069.16193466
SRR17520069.16193467
SRR17520069.16193468
SRR17520069.16193469
SRR17520069.16193470
SRR17520069.16193471
SRR17520069.16193472
SRR17520069.16193473
SRR17520069.16193474
SRR17520069.16193475
SRR17520069.16193476
SRR17520069.16193477
SRR17520069.16193478
SRR17520069.16193479
SRR17520069.16193480
SRR17520069.16193481
SRR17520069.16193482
SRR17520069.16193483
SRR17520069.16193484
SRR17520069.16193485
SRR17520069.16193486
SRR17520069.16193487
SRR17520069.16193488
SRR17520069.16193489
SRR17520069.16193490
SRR17520069.16193491
SRR17520069.16193492
SRR17520069.16193493
SRR17520069.16193494
SRR17520069.16193495
SRR17520069.16193496
SRR17520069.16193497
SRR17520069.16193498
SRR17520069.16193499
SRR17520069.16193500
SRR17520069.16193501
SRR17520069.16193502
SRR17520069.16193503
SRR17520069.16193504

SRR17520069.16193505
SRR17520069.16193506
SRR17520069.16193507
SRR17520069.16193508
SRR17520069.16193509
SRR17520069.16193510
SRR17520069.16193511
SRR17520069.16193512
SRR17520069.16193513
SRR17520069.16193514
SRR17520069.16193515
SRR17520069.16193516
SRR17520069.16193517
SRR17520069.16193518
SRR17520069.16193519
SRR17520069.16193520
SRR17520069.16193521
SRR17520069.16193522
SRR17520069.16193523
SRR17520069.16193524
SRR17520069.16193525
SRR17520069.16193526
SRR17520069.16193527
SRR17520069.16193528
SRR17520069.16193529
SRR17520069.16193530
SRR17520069.16193531
SRR17520069.16193532
SRR17520069.16193533
SRR17520069.16193534
SRR17520069.16193535
SRR17520069.16193536
SRR17520069.16193537
SRR17520069.16193538
SRR17520069.16193539
SRR17520069.16193540
SRR17520069.16193541
SRR17520069.16193542
SRR17520069.16193543
SRR17520069.16193544
SRR17520069.16193545
SRR17520069.16193546
SRR17520069.16193547
SRR17520069.16193548
SRR17520069.16193549
SRR17520069.16193550
SRR17520069.16193551

SRR17520069.16193552
SRR17520069.16193553
SRR17520069.16193554
SRR17520069.16193556
SRR17520069.16193557
SRR17520069.16193558
SRR17520069.16193559
SRR17520069.16193560
SRR17520069.16193561
SRR17520069.16193562
SRR17520069.16193563
SRR17520069.16193564
SRR17520069.16193565
SRR17520069.16193566
SRR17520069.16193567
SRR17520069.16193568
SRR17520069.16193569
SRR17520069.16193570
SRR17520069.16193571
SRR17520069.16193572
SRR17520069.16193573
SRR17520069.16193574
SRR17520069.16193575
SRR17520069.16193576
SRR17520069.16193577
SRR17520069.16193578
SRR17520069.16193579
SRR17520069.16193580
SRR17520069.16193581
SRR17520069.16193582
SRR17520069.16193583
SRR17520069.16193584
SRR17520069.16193585
SRR17520069.16193586
SRR17520069.16193587
SRR17520069.16193588
SRR17520069.16193589
SRR17520069.16193590
SRR17520069.16193591
SRR17520069.16193592
SRR17520069.16193593
SRR17520069.16193594
SRR17520069.16193595
SRR17520069.16193596
SRR17520069.16193597
SRR17520069.16193598
SRR17520069.16193599

SRR17520069.16193600
SRR17520069.16193601
SRR17520069.16193602
SRR17520069.16193603
SRR17520069.16193604
SRR17520069.16193605
SRR17520069.16193606
SRR17520069.16193607
SRR17520069.16193608
SRR17520069.16193609
SRR17520069.16193610
SRR17520069.16193611
SRR17520069.16193612
SRR17520069.16193613
SRR17520069.16193615
SRR17520069.16193616
SRR17520069.16193617
SRR17520069.16193618
SRR17520069.16193619
SRR17520069.16193620
SRR17520069.16193621
SRR17520069.16193622
SRR17520069.16193623
SRR17520069.16193624
SRR17520069.16193625
SRR17520069.16193626
SRR17520069.16193627
SRR17520069.16193628
SRR17520069.16193629
SRR17520069.16193631
SRR17520069.16193632
SRR17520069.16193633
SRR17520069.16193634
SRR17520069.16193636
SRR17520069.16193637
SRR17520069.16193638
SRR17520069.16193639
SRR17520069.16193640
SRR17520069.16193641
SRR17520069.16193642
SRR17520069.16193644
SRR17520069.16193645
SRR17520069.16193646
SRR17520069.16193647
SRR17520069.16193648
SRR17520069.16193649
SRR17520069.16193650

SRR17520069.16193651
SRR17520069.16193652
SRR17520069.16193653
SRR17520069.16193654
SRR17520069.16193655
SRR17520069.16193656
SRR17520069.16193657
SRR17520069.16193658
SRR17520069.16193659
SRR17520069.16193660
SRR17520069.16193661
SRR17520069.16193662
SRR17520069.16197426
SRR17520069.16197427
SRR17520069.16197428
SRR17520069.16197429
SRR17520069.16197430
SRR17520069.16197431
SRR17520069.16197432
SRR17520069.16197433
SRR17520069.16197434
SRR17520069.16197435
SRR17520069.16197436
SRR17520069.16197437
SRR17520069.16197438
SRR17520069.16197439
SRR17520069.16197440
SRR17520069.16197441
SRR17520069.16197443
SRR17520069.16197444
SRR17520069.16197445
SRR17520069.16197446
SRR17520069.16197447
SRR17520069.16197448
SRR17520069.16197449
SRR17520069.16197450
SRR17520069.16197451
SRR17520069.16197452
SRR17520069.16197453
SRR17520069.16197455
SRR17520069.16197456
SRR17520069.16197457
SRR17520069.16197458
SRR17520069.16197459
SRR17520069.16197460
SRR17520069.16197461
SRR17520069.16197462

SRR17520069.16197463
SRR17520069.16197464
SRR17520069.16197465
SRR17520069.16197466
SRR17520069.16197467
SRR17520069.16197468
SRR17520069.16197469
SRR17520069.16197470
SRR17520069.16197471
SRR17520069.16197472
SRR17520069.16197473
SRR17520069.16197474
SRR17520069.16197475
SRR17520069.16197476
SRR17520069.16197478
SRR17520069.16197479
SRR17520069.16197481
SRR17520069.16197482
SRR17520069.16197484
SRR17520069.16197485
SRR17520069.16197486
SRR17520069.16197487
SRR17520069.16197489
SRR17520069.16197491
SRR17520069.16200058
SRR17520069.16200059
SRR17520069.16200060
SRR17520069.16200061
SRR17520069.16200062
SRR17520069.16200063
SRR17520069.16200064
SRR17520069.16200066
SRR17520069.16200067
SRR17520069.16200068
SRR17520069.16200069
SRR17520069.16200070
SRR17520069.16200071
SRR17520069.16200072
SRR17520069.16200074
SRR17520069.16200075
SRR17520069.16200076
SRR17520069.16200077
SRR17520069.16200079
SRR17520069.16200081
SRR17520069.16200082
SRR17520069.16200083
SRR17520069.16200084

SRR17520069.16200085
SRR17520069.16200086
SRR17520069.16200088
SRR17520069.16200089
SRR17520069.16200090
SRR17520069.16200091
SRR17520069.16200092
SRR17520069.16200094
SRR17520069.16200095
SRR17520069.16200096
SRR17520069.16200097
SRR17520069.16200098
SRR17520069.16200099
SRR17520069.16200100
SRR17520069.16200101
SRR17520069.16200102
SRR17520069.16200103
SRR17520069.16200104
SRR17520069.16200105
SRR17520069.16200107
SRR17520069.16200108
SRR17520069.16200109
SRR17520069.16201728
SRR17520069.16201729
SRR17520069.16201730
SRR17520069.16201732
SRR17520069.16201733
SRR17520069.16201734
SRR17520069.16201735
SRR17520069.16201736
SRR17520069.16201737
SRR17520069.16201738
SRR17520069.16201739
SRR17520069.16201740
SRR17520069.16201741
SRR17520069.16201743
SRR17520069.16201744
SRR17520069.16201745
SRR17520069.16201746
SRR17520069.16201747
SRR17520069.16201748
SRR17520069.16201749
SRR17520069.16201750
SRR17520069.16201751
SRR17520069.16201752
SRR17520069.16201753
SRR17520069.16201754

SRR17520069.16201755
SRR17520069.16201756
SRR17520069.16201757
SRR17520069.16201758
SRR17520069.16201759
SRR17520069.16201760
SRR17520069.16201761
SRR17520069.16201762
SRR17520069.16201763
SRR17520069.16201764
SRR17520069.16201765
SRR17520069.16201766
SRR17520069.16201767
SRR17520069.16201768
SRR17520069.16201769
SRR17520069.16201770
SRR17520069.16201771
SRR17520069.16201772
SRR17520069.16201773
SRR17520069.16201774
SRR17520069.16201775
SRR17520069.16201776
SRR17520069.16201777
SRR17520069.16201778
SRR17520069.16201779
SRR17520069.16201781
SRR17520069.16201784
SRR17520069.16201785
SRR17520069.16201786
SRR17520069.16209567
SRR17520069.16209568
SRR17520069.16209569
SRR17520069.16209570
SRR17520069.16209571
SRR17520069.16209573
SRR17520069.16209576
SRR17520069.16217709
SRR17520069.16217731
SRR17520069.16219455
SRR17520069.16222649
SRR17520069.16222650
SRR17520069.16222651
SRR17520069.16222652
SRR17520069.16222653
SRR17520069.16222654
SRR17520069.16222655
SRR17520069.16222656

SRR17520069.16222657
SRR17520069.16222658
SRR17520069.16222659
SRR17520069.16222660
SRR17520069.16222662
SRR17520069.16222663
SRR17520069.16222664
SRR17520069.16222665
SRR17520069.16222666
SRR17520069.16222667
SRR17520069.16223996
SRR17520069.16225732
SRR17520069.16238585
SRR17520069.16238586
SRR17520069.16238587
SRR17520069.16238589
SRR17520069.16238592
SRR17520069.16238595
SRR17520069.16238596
SRR17520069.16238597
SRR17520069.16238601
SRR17520069.16238602
SRR17520069.16238603
SRR17520069.16238604
SRR17520069.16238605
SRR17520069.16238606
SRR17520069.16238607
SRR17520069.16238608
SRR17520069.16238609
SRR17520069.16238610
SRR17520069.16238611
SRR17520069.16238612
SRR17520069.16238613
SRR17520069.16238614
SRR17520069.16238616
SRR17520069.16242858
SRR17520069.16243476
SRR17520069.16243477
SRR17520069.16243626
SRR17520069.16248394
SRR17520069.16249314
SRR17520069.16249316
SRR17520069.16268459
SRR17520069.16273575
SRR17520069.16273576
SRR17520069.16273577
SRR17520069.16273578

SRR17520069.16273579
SRR17520069.16273580
SRR17520069.16273581
SRR17520069.16273582
SRR17520069.16273583
SRR17520069.16273584
SRR17520069.16273585
SRR17520069.16273586
SRR17520069.16273587
SRR17520069.16273588
SRR17520069.16273589
SRR17520069.16273591
SRR17520069.16273592
SRR17520069.16273593
SRR17520069.16273594
SRR17520069.16273595
SRR17520069.16273596
SRR17520069.16273597
SRR17520069.16273598
SRR17520069.16273599
SRR17520069.16273600
SRR17520069.16273601
SRR17520069.16273602
SRR17520069.16273603
SRR17520069.16273604
SRR17520069.16273605
SRR17520069.16273606
SRR17520069.16273607
SRR17520069.16273608
SRR17520069.16273609
SRR17520069.16273610
SRR17520069.16273611
SRR17520069.16273612
SRR17520069.16273613
SRR17520069.16273614
SRR17520069.16273615
SRR17520069.16273616
SRR17520069.16273617
SRR17520069.16273618
SRR17520069.16273619
SRR17520069.16273620
SRR17520069.16273621
SRR17520069.16273622
SRR17520069.16273623
SRR17520069.16273624
SRR17520069.16273625
SRR17520069.16273626

SRR17520069.16273627
SRR17520069.16273629
SRR17520069.16273630
SRR17520069.16273631
SRR17520069.16273632
SRR17520069.16273633
SRR17520069.16273634
SRR17520069.16273635
SRR17520069.16273636
SRR17520069.16273637
SRR17520069.16273638
SRR17520069.16273639
SRR17520069.16273640
SRR17520069.16273641
SRR17520069.16273642
SRR17520069.16273643
SRR17520069.16273644
SRR17520069.16273645
SRR17520069.16273646
SRR17520069.16273647
SRR17520069.16273648
SRR17520069.16273649
SRR17520069.16273651
SRR17520069.16273652
SRR17520069.16273653
SRR17520069.16273654
SRR17520069.16273655
SRR17520069.16273656
SRR17520069.16273657
SRR17520069.16273658
SRR17520069.16273659
SRR17520069.16273660
SRR17520069.16273661
SRR17520069.16273662
SRR17520069.16273663
SRR17520069.16273664
SRR17520069.16273665
SRR17520069.16273666
SRR17520069.16273667
SRR17520069.16273668
SRR17520069.16273669
SRR17520069.16273670
SRR17520069.16273671
SRR17520069.16282057
SRR17520069.16282058
SRR17520069.16282059
SRR17520069.16282060

SRR17520069.16282061
SRR17520069.16282062
SRR17520069.16282063
SRR17520069.16282064
SRR17520069.16282065
SRR17520069.16282066
SRR17520069.16282067
SRR17520069.16282068
SRR17520069.16282069
SRR17520069.16282070
SRR17520069.16282071
SRR17520069.16282072
SRR17520069.16282073
SRR17520069.16282074
SRR17520069.16282075
SRR17520069.16282076
SRR17520069.16282077
SRR17520069.16282079
SRR17520069.16282080
SRR17520069.16282081
SRR17520069.16282082
SRR17520069.16282084
SRR17520069.16282085
SRR17520069.16282086
SRR17520069.16282087
SRR17520069.16282088
SRR17520069.16282089
SRR17520069.16282090
SRR17520069.16282092
SRR17520069.16282093
SRR17520069.16282094
SRR17520069.16282095
SRR17520069.16282096
SRR17520069.16282097
SRR17520069.16282098
SRR17520069.16282099
SRR17520069.16282100
SRR17520069.16282101
SRR17520069.16282102
SRR17520069.16282103
SRR17520069.16282104
SRR17520069.16282106
SRR17520069.16282107
SRR17520069.16282108
SRR17520069.16282109
SRR17520069.16282110
SRR17520069.16282111

SRR17520069.16282112
SRR17520069.16282113
SRR17520069.16282114
SRR17520069.16282115
SRR17520069.16282116
SRR17520069.16282117
SRR17520069.16282118
SRR17520069.16282119
SRR17520069.16282120
SRR17520069.16282121
SRR17520069.16282122
SRR17520069.16282123
SRR17520069.16282124
SRR17520069.16282126
SRR17520069.16282127
SRR17520069.16282128
SRR17520069.16282129
SRR17520069.16282130
SRR17520069.16282131
SRR17520069.16282132
SRR17520069.16282133
SRR17520069.16282134
SRR17520069.16282135
SRR17520069.16282136
SRR17520069.16282137
SRR17520069.16282138
SRR17520069.16282139
SRR17520069.16282140
SRR17520069.16282141
SRR17520069.16282142
SRR17520069.16282143
SRR17520069.16282144
SRR17520069.16282145
SRR17520069.16282147
SRR17520069.16282148
SRR17520069.16282149
SRR17520069.16282150
SRR17520069.16282152
SRR17520069.16282153
SRR17520069.16282154
SRR17520069.16282155
SRR17520069.16282157
SRR17520069.16282158
SRR17520069.16282159
SRR17520069.16282160
SRR17520069.16282161
SRR17520069.16282162

SRR17520069.16282163
SRR17520069.16282164
SRR17520069.16282166
SRR17520069.16282167
SRR17520069.16282168
SRR17520069.16282169
SRR17520069.16282170
SRR17520069.16282171
SRR17520069.16282172
SRR17520069.16282173
SRR17520069.16282174
SRR17520069.16282175
SRR17520069.16282176
SRR17520069.16282177
SRR17520069.16282178
SRR17520069.16282180
SRR17520069.16282181
SRR17520069.16282182
SRR17520069.16282183
SRR17520069.16282184
SRR17520069.16282185
SRR17520069.16282186
SRR17520069.16282187
SRR17520069.16282188
SRR17520069.16282189
SRR17520069.16282190
SRR17520069.16282191
SRR17520069.16282192
SRR17520069.16282193
SRR17520069.16282194
SRR17520069.16282195
SRR17520069.16282196
SRR17520069.16282197
SRR17520069.16282200
SRR17520069.16282201
SRR17520069.16282202
SRR17520069.16282203
SRR17520069.16282204
SRR17520069.16282205
SRR17520069.16282206
SRR17520069.16282207
SRR17520069.16282208
SRR17520069.16282209
SRR17520069.16282210
SRR17520069.16282212
SRR17520069.16282213
SRR17520069.16282214

SRR17520069.16282215
SRR17520069.16282216
SRR17520069.16282217
SRR17520069.16282219
SRR17520069.16282222
SRR17520069.16282223
SRR17520069.16282224
SRR17520069.16282225
SRR17520069.16282226
SRR17520069.16282227
SRR17520069.16293703
SRR17520069.16293704
SRR17520069.16293705
SRR17520069.16293706
SRR17520069.16293707
SRR17520069.16293708
SRR17520069.16293709
SRR17520069.16293710
SRR17520069.16293711
SRR17520069.16293712
SRR17520069.16293713
SRR17520069.16293714
SRR17520069.16293715
SRR17520069.16293716
SRR17520069.16293717
SRR17520069.16293718
SRR17520069.16293719
SRR17520069.16293721
SRR17520069.16293723
SRR17520069.16293724
SRR17520069.16293725
SRR17520069.16293726
SRR17520069.16293727
SRR17520069.16293728
SRR17520069.16293729
SRR17520069.16293731
SRR17520069.16293732
SRR17520069.16293733
SRR17520069.16293734
SRR17520069.16293735
SRR17520069.16293736
SRR17520069.16293737
SRR17520069.16293738
SRR17520069.16293739
SRR17520069.16293740
SRR17520069.16293741
SRR17520069.16293742

SRR17520069.16293743
SRR17520069.16293744
SRR17520069.16293745
SRR17520069.16293746
SRR17520069.16293747
SRR17520069.16293748
SRR17520069.16293749
SRR17520069.16293750
SRR17520069.16293751
SRR17520069.16293752
SRR17520069.16293753
SRR17520069.16293754
SRR17520069.16293755
SRR17520069.16320228
SRR17520069.16327040
SRR17520069.16342744
SRR17520069.16342745
SRR17520069.16342746
SRR17520069.16342747
SRR17520069.16342748
SRR17520069.16342749
SRR17520069.16342750
SRR17520069.16342751
SRR17520069.16342752
SRR17520069.16342753
SRR17520069.16342754
SRR17520069.16342755
SRR17520069.16342756
SRR17520069.16342757
SRR17520069.16342758
SRR17520069.16342759
SRR17520069.16342761
SRR17520069.16352161
SRR17520069.16352311
SRR17520069.16358455
SRR17520069.16358456
SRR17520069.16358457
SRR17520069.16358458
SRR17520069.16358459
SRR17520069.16358460
SRR17520069.16358461
SRR17520069.16358462
SRR17520069.16361142
SRR17520069.16361143
SRR17520069.16361144
SRR17520069.16361145
SRR17520069.16361146

SRR17520069.16361147
SRR17520069.16361148
SRR17520069.16361149
SRR17520069.16361150
SRR17520069.16361151
SRR17520069.16361152
SRR17520069.16361153
SRR17520069.16361154
SRR17520069.16361155
SRR17520069.16361156
SRR17520069.16361157
SRR17520069.16361158
SRR17520069.16361159
SRR17520069.16361160
SRR17520069.16361161
SRR17520069.16361162
SRR17520069.16361163
SRR17520069.16361164
SRR17520069.16361165
SRR17520069.16361166
SRR17520069.16361167
SRR17520069.16361168
SRR17520069.16361170
SRR17520069.16361171
SRR17520069.16361172
SRR17520069.16361173
SRR17520069.16361174
SRR17520069.16361175
SRR17520069.16361176
SRR17520069.16361177
SRR17520069.16361178
SRR17520069.16361179
SRR17520069.16361180
SRR17520069.16361181
SRR17520069.16361182
SRR17520069.16361183
SRR17520069.16361184
SRR17520069.16361185
SRR17520069.16361187
SRR17520069.16361188
SRR17520069.16361189
SRR17520069.16361190
SRR17520069.16361191
SRR17520069.16361192
SRR17520069.16361193
SRR17520069.16361194
SRR17520069.16361196

SRR17520069.16361197
SRR17520069.16361198
SRR17520069.16361199
SRR17520069.16361200
SRR17520069.16361201
SRR17520069.16361202
SRR17520069.16361203
SRR17520069.16361204
SRR17520069.16361205
SRR17520069.16361206
SRR17520069.16361207
SRR17520069.16361208
SRR17520069.16361209
SRR17520069.16361210
SRR17520069.16361211
SRR17520069.16361212
SRR17520069.16361213
SRR17520069.16361214
SRR17520069.16361215
SRR17520069.16361216
SRR17520069.16361217
SRR17520069.16361218
SRR17520069.16361220
SRR17520069.16361221
SRR17520069.16361222
SRR17520069.16361223
SRR17520069.16361224
SRR17520069.16361226
SRR17520069.16361227
SRR17520069.16361228
SRR17520069.16361229
SRR17520069.16361230
SRR17520069.16361231
SRR17520069.16361232
SRR17520069.16361233
SRR17520069.16361234
SRR17520069.16361235
SRR17520069.16361236
SRR17520069.16361237
SRR17520069.16361238
SRR17520069.16361239
SRR17520069.16361240
SRR17520069.16361241
SRR17520069.16361242
SRR17520069.16361243
SRR17520069.16361244
SRR17520069.16361245

SRR17520069.16361247
SRR17520069.16361248
SRR17520069.16361249
SRR17520069.16361250
SRR17520069.16361251
SRR17520069.16361252
SRR17520069.16361253
SRR17520069.16361254
SRR17520069.16361255
SRR17520069.16382810
SRR17520069.16383939
SRR17520069.16404234
SRR17520069.16404235
SRR17520069.16404236
SRR17520069.16404237
SRR17520069.16404238
SRR17520069.16404240
SRR17520069.16404241
SRR17520069.16405755
SRR17520069.16405756
SRR17520069.16405757
SRR17520069.16405759
SRR17520069.16405760
SRR17520069.16414011
SRR17520069.16433549
SRR17520069.16433550
SRR17520069.16433551
SRR17520069.16433552
SRR17520069.16434065
SRR17520069.16434066
SRR17520069.16434067
SRR17520069.16434070
SRR17520069.16434072
SRR17520069.16434073
SRR17520069.16434075
SRR17520069.16434077
SRR17520069.16434078
SRR17520069.16434080
SRR17520069.16434081
SRR17520069.16434082
SRR17520069.16440286
SRR17520069.16440287
SRR17520069.16440288
SRR17520069.16440289
SRR17520069.16440290
SRR17520069.16440291
SRR17520069.16440292

SRR17520069.16440293
SRR17520069.16440294
SRR17520069.16440295
SRR17520069.16440296
SRR17520069.16440297
SRR17520069.16440298
SRR17520069.16440299
SRR17520069.16440300
SRR17520069.16440301
SRR17520069.16440302
SRR17520069.16440303
SRR17520069.16440304
SRR17520069.16440305
SRR17520069.16440306
SRR17520069.16440307
SRR17520069.16440308
SRR17520069.16440309
SRR17520069.16440310
SRR17520069.16440311
SRR17520069.16440312
SRR17520069.16440313
SRR17520069.16440314
SRR17520069.16440315
SRR17520069.16440316
SRR17520069.16440317
SRR17520069.16440318
SRR17520069.16440320
SRR17520069.16440321
SRR17520069.16440322
SRR17520069.16440324
SRR17520069.16440325
SRR17520069.16440326
SRR17520069.16440327
SRR17520069.16440328
SRR17520069.16440329
SRR17520069.16440330
SRR17520069.16440331
SRR17520069.16440332
SRR17520069.16440333
SRR17520069.16440334
SRR17520069.16440335
SRR17520069.16440336
SRR17520069.16440337
SRR17520069.16440338
SRR17520069.16440339
SRR17520069.16440340
SRR17520069.16440341

SRR17520069.16440342
SRR17520069.16440343
SRR17520069.16440344
SRR17520069.16440345
SRR17520069.16440346
SRR17520069.16440347
SRR17520069.16440348
SRR17520069.16440349
SRR17520069.16440350
SRR17520069.16440351
SRR17520069.16440352
SRR17520069.16440353
SRR17520069.16440354
SRR17520069.16440355
SRR17520069.16440356
SRR17520069.16440357
SRR17520069.16440358
SRR17520069.16440359
SRR17520069.16440360
SRR17520069.16440361
SRR17520069.16440362
SRR17520069.16440363
SRR17520069.16440364
SRR17520069.16440365
SRR17520069.16440366
SRR17520069.16440367
SRR17520069.16440368
SRR17520069.16440369
SRR17520069.16440370
SRR17520069.16440371
SRR17520069.16440372
SRR17520069.16440373
SRR17520069.16440374
SRR17520069.16440375
SRR17520069.16440376
SRR17520069.16440377
SRR17520069.16440378
SRR17520069.16440379
SRR17520069.16440380
SRR17520069.16440381
SRR17520069.16440382
SRR17520069.16440383
SRR17520069.16440384
SRR17520069.16440385
SRR17520069.16440386
SRR17520069.16440387
SRR17520069.16440388

SRR17520069.16440389
SRR17520069.16440390
SRR17520069.16440391
SRR17520069.16440392
SRR17520069.16440393
SRR17520069.16440394
SRR17520069.16440395
SRR17520069.16440396
SRR17520069.16440397
SRR17520069.16441424
SRR17520069.16474642
SRR17520069.16480699
SRR17520069.16480700
SRR17520069.16480701
SRR17520069.16480702
SRR17520069.16480704
SRR17520069.16481171
SRR17520069.16481172
SRR17520069.16481173
SRR17520069.16481174
SRR17520069.16481175
SRR17520069.16481176
SRR17520069.16481177
SRR17520069.16481178
SRR17520069.16481179
SRR17520069.16481180
SRR17520069.16481181
SRR17520069.16481182
SRR17520069.16481183
SRR17520069.16481184
SRR17520069.16481185
SRR17520069.16481186
SRR17520069.16481187
SRR17520069.16481188
SRR17520069.16481189
SRR17520069.16481190
SRR17520069.16481191
SRR17520069.16481192
SRR17520069.16481193
SRR17520069.16481194
SRR17520069.16481195
SRR17520069.16481196
SRR17520069.16481205
SRR17520069.16486482
SRR17520069.16501712
SRR17520069.16501713
SRR17520069.16501714

SRR17520069.16501715
SRR17520069.16501716
SRR17520069.16501717
SRR17520069.16501718
SRR17520069.16501719
SRR17520069.16501720
SRR17520069.16501730
SRR17520069.16501744
SRR17520069.16501768
SRR17520069.16501799
SRR17520069.16510104
SRR17520069.16510105
SRR17520069.16510106
SRR17520069.16510107
SRR17520069.16510108
SRR17520069.16510109
SRR17520069.16510110
SRR17520069.16510111
SRR17520069.16510112
SRR17520069.16510113
SRR17520069.16510114
SRR17520069.16510115
SRR17520069.16510116
SRR17520069.16510117
SRR17520069.16510118
SRR17520069.16510119
SRR17520069.16510120
SRR17520069.16510121
SRR17520069.16510122
SRR17520069.16510123
SRR17520069.16510124
SRR17520069.16510125
SRR17520069.16510126
SRR17520069.16510127
SRR17520069.16510128
SRR17520069.16510129
SRR17520069.16510130
SRR17520069.16510131
SRR17520069.16510132
SRR17520069.16510133
SRR17520069.16510134
SRR17520069.16510135
SRR17520069.16510136
SRR17520069.16510137
SRR17520069.16510138
SRR17520069.16510139
SRR17520069.16510140

SRR17520069.16510141
SRR17520069.16510142
SRR17520069.16510144
SRR17520069.16510145
SRR17520069.16510146
SRR17520069.16510147
SRR17520069.16510148
SRR17520069.16510149
SRR17520069.16510150
SRR17520069.16510151
SRR17520069.16510152
SRR17520069.16510153
SRR17520069.16510154
SRR17520069.16510155
SRR17520069.16510156
SRR17520069.16510157
SRR17520069.16510158
SRR17520069.16510159
SRR17520069.16510160
SRR17520069.16510161
SRR17520069.16510162
SRR17520069.16510163
SRR17520069.16510164
SRR17520069.16510165
SRR17520069.16510166
SRR17520069.16510167
SRR17520069.16510168
SRR17520069.16510169
SRR17520069.16510170
SRR17520069.16510171
SRR17520069.16510172
SRR17520069.16510173
SRR17520069.16510174
SRR17520069.16510175
SRR17520069.16510177
SRR17520069.16510178
SRR17520069.16510179
SRR17520069.16510180
SRR17520069.16510181
SRR17520069.16510182
SRR17520069.16510183
SRR17520069.16510184
SRR17520069.16510185
SRR17520069.16510186
SRR17520069.16510187
SRR17520069.16510189
SRR17520069.16510190

SRR17520069.16510191
SRR17520069.16510192
SRR17520069.16510193
SRR17520069.16510194
SRR17520069.16510195
SRR17520069.16510196
SRR17520069.16510197
SRR17520069.16510198
SRR17520069.16510200
SRR17520069.16510201
SRR17520069.16510202
SRR17520069.16510204
SRR17520069.16510205
SRR17520069.16510206
SRR17520069.16510207
SRR17520069.16510208
SRR17520069.16510209
SRR17520069.16510210
SRR17520069.16510211
SRR17520069.16510212
SRR17520069.16510213
SRR17520069.16510214
SRR17520069.16510215
SRR17520069.16510216
SRR17520069.16510217
SRR17520069.16510219
SRR17520069.16510220
SRR17520069.16510221
SRR17520069.16510222
SRR17520069.16510223
SRR17520069.16510224
SRR17520069.16510225
SRR17520069.16510226
SRR17520069.16510227
SRR17520069.16510228
SRR17520069.16519053
SRR17520069.16519054
SRR17520069.16519055
SRR17520069.16519056
SRR17520069.16519057
SRR17520069.16519058
SRR17520069.16519059
SRR17520069.16519060
SRR17520069.16519061
SRR17520069.16519062
SRR17520069.16519063
SRR17520069.16519064

SRR17520069.16519065
SRR17520069.16519066
SRR17520069.16519067
SRR17520069.16519068
SRR17520069.16519069
SRR17520069.16519070
SRR17520069.16519071
SRR17520069.16519072
SRR17520069.16519073
SRR17520069.16519074
SRR17520069.16519075
SRR17520069.16519076
SRR17520069.16519077
SRR17520069.16519078
SRR17520069.16519079
SRR17520069.16519080
SRR17520069.16519081
SRR17520069.16519082
SRR17520069.16519083
SRR17520069.16519084
SRR17520069.16519085
SRR17520069.16519086
SRR17520069.16519087
SRR17520069.16519088
SRR17520069.16519089
SRR17520069.16519090
SRR17520069.16519091
SRR17520069.16519092
SRR17520069.16519093
SRR17520069.16519094
SRR17520069.16519095
SRR17520069.16519097
SRR17520069.16519098
SRR17520069.16519099
SRR17520069.16519100
SRR17520069.16519101
SRR17520069.16519102
SRR17520069.16519103
SRR17520069.16519105
SRR17520069.16519106
SRR17520069.16519107
SRR17520069.16519108
SRR17520069.16519109
SRR17520069.16519110
SRR17520069.16519111
SRR17520069.16519112
SRR17520069.16519113

SRR17520069.16519114
SRR17520069.16519115
SRR17520069.16519116
SRR17520069.16519117
SRR17520069.16519118
SRR17520069.16519119
SRR17520069.16519120
SRR17520069.16519121
SRR17520069.16519122
SRR17520069.16519123
SRR17520069.16519124
SRR17520069.16519125
SRR17520069.16519127
SRR17520069.16519128
SRR17520069.16519129
SRR17520069.16519130
SRR17520069.16519131
SRR17520069.16521407
SRR17520069.16527281
SRR17520069.16527282
SRR17520069.16527283
SRR17520069.16527284
SRR17520069.16527285
SRR17520069.16527286
SRR17520069.16527287
SRR17520069.16527288
SRR17520069.16527289
SRR17520069.16527290
SRR17520069.16527291
SRR17520069.16527292
SRR17520069.16527294
SRR17520069.16527295
SRR17520069.16527296
SRR17520069.16527297
SRR17520069.16527298
SRR17520069.16527299
SRR17520069.16527300
SRR17520069.16527301
SRR17520069.16527302
SRR17520069.16527303
SRR17520069.16527304
SRR17520069.16527305
SRR17520069.16527306
SRR17520069.16527307
SRR17520069.16527308
SRR17520069.16527309
SRR17520069.16527310

SRR17520069.16527311
SRR17520069.16527312
SRR17520069.16527313
SRR17520069.16527314
SRR17520069.16527315
SRR17520069.16527316
SRR17520069.16527317
SRR17520069.16527318
SRR17520069.16527319
SRR17520069.16527320
SRR17520069.16581324
SRR17520069.16581325
SRR17520069.16581327
SRR17520069.16581328
SRR17520069.16581329
SRR17520069.16588273
SRR17520069.16588274
SRR17520069.16588275
SRR17520069.16588276
SRR17520069.16588277
SRR17520069.16588278
SRR17520069.16588279
SRR17520069.16588280
SRR17520069.16588281
SRR17520069.16588282
SRR17520069.16588283
SRR17520069.16588284
SRR17520069.16588285
SRR17520069.16588286
SRR17520069.16588287
SRR17520069.16588288
SRR17520069.16588289
SRR17520069.16588290
SRR17520069.16588291
SRR17520069.16588292
SRR17520069.16588293
SRR17520069.16588294
SRR17520069.16588295
SRR17520069.16588296
SRR17520069.16588297
SRR17520069.16588298
SRR17520069.16599844
SRR17520069.16600965
SRR17520069.16600966
SRR17520069.16600967
SRR17520069.16600968
SRR17520069.16600969

SRR17520069.16600970
SRR17520069.16600971
SRR17520069.16600972
SRR17520069.16600973
SRR17520069.16600974
SRR17520069.16600976
SRR17520069.16600977
SRR17520069.16600978
SRR17520069.16600979
SRR17520069.16600980
SRR17520069.16600981
SRR17520069.16600982
SRR17520069.16600983
SRR17520069.16600984
SRR17520069.16600985
SRR17520069.16600987
SRR17520069.16600988
SRR17520069.16600989
SRR17520069.16600990
SRR17520069.16600991
SRR17520069.16600992
SRR17520069.16600993
SRR17520069.16600994
SRR17520069.16600995
SRR17520069.16600996
SRR17520069.16600997
SRR17520069.16600998
SRR17520069.16600999
SRR17520069.16601000
SRR17520069.16601001
SRR17520069.16601002
SRR17520069.16610405
SRR17520069.16610406
SRR17520069.16610407
SRR17520069.16610408
SRR17520069.16610409
SRR17520069.16610410
SRR17520069.16610411
SRR17520069.16610412
SRR17520069.16610413
SRR17520069.16610414
SRR17520069.16610415
SRR17520069.16610416
SRR17520069.16610417
SRR17520069.16610418
SRR17520069.16610419
SRR17520069.16610420

SRR17520069.16610421
SRR17520069.16610422
SRR17520069.16610423
SRR17520069.16610424
SRR17520069.16610425
SRR17520069.16610426
SRR17520069.16610427
SRR17520069.16610428
SRR17520069.16610429
SRR17520069.16610431
SRR17520069.16610432
SRR17520069.16610433
SRR17520069.16610434
SRR17520069.16610435
SRR17520069.16610436
SRR17520069.16610437
SRR17520069.16610438
SRR17520069.16610439
SRR17520069.16610440
SRR17520069.16610441
SRR17520069.16610442
SRR17520069.16610443
SRR17520069.16610444
SRR17520069.16610445
SRR17520069.16610446
SRR17520069.16610447
SRR17520069.16610448
SRR17520069.16610449
SRR17520069.16610450
SRR17520069.16610452
SRR17520069.16610453
SRR17520069.16610454
SRR17520069.16623463
SRR17520069.16623464
SRR17520069.16623465
SRR17520069.16623466
SRR17520069.16623467
SRR17520069.16623468
SRR17520069.16623469
SRR17520069.16623470
SRR17520069.16623471
SRR17520069.16623472
SRR17520069.16623473
SRR17520069.16623474
SRR17520069.16623475
SRR17520069.16623476
SRR17520069.16623477

SRR17520069.16623478
SRR17520069.16623479
SRR17520069.16623480
SRR17520069.16623481
SRR17520069.16623482
SRR17520069.16623483
SRR17520069.16623484
SRR17520069.16623485
SRR17520069.16623486
SRR17520069.16623487
SRR17520069.16623488
SRR17520069.16623489
SRR17520069.16623490
SRR17520069.16623491
SRR17520069.16623492
SRR17520069.16623493
SRR17520069.16623494
SRR17520069.16623495
SRR17520069.16623496
SRR17520069.16623497
SRR17520069.16623498
SRR17520069.16623499
SRR17520069.16623500
SRR17520069.16623501
SRR17520069.16623502
SRR17520069.16623503
SRR17520069.16623504
SRR17520069.16623505
SRR17520069.16623506
SRR17520069.16623507
SRR17520069.16623508
SRR17520069.16623509
SRR17520069.16623510
SRR17520069.16623511
SRR17520069.16623512
SRR17520069.16623513
SRR17520069.16623514
SRR17520069.16623515
SRR17520069.16623516
SRR17520069.16623517
SRR17520069.16623518
SRR17520069.16623519
SRR17520069.16623520
SRR17520069.16623521
SRR17520069.16623522
SRR17520069.16623523
SRR17520069.16623524

SRR17520069.16623525
SRR17520069.16623526
SRR17520069.16623527
SRR17520069.16623528
SRR17520069.16623529
SRR17520069.16623530
SRR17520069.16623531
SRR17520069.16623532
SRR17520069.16623533
SRR17520069.16623534
SRR17520069.16623535
SRR17520069.16623536
SRR17520069.16638097
SRR17520069.16638098
SRR17520069.16638099
SRR17520069.16638100
SRR17520069.16638101
SRR17520069.16638102
SRR17520069.16638103
SRR17520069.16638104
SRR17520069.16638105
SRR17520069.16638106
SRR17520069.16638107
SRR17520069.16638108
SRR17520069.16644339
SRR17520069.16647282
SRR17520069.16647283
SRR17520069.16647284
SRR17520069.16647285
SRR17520069.16647286
SRR17520069.16647287
SRR17520069.16647288
SRR17520069.16647289
SRR17520069.16647290
SRR17520069.16647291
SRR17520069.16647292
SRR17520069.16647294
SRR17520069.16647295
SRR17520069.16647296
SRR17520069.16647297
SRR17520069.16647298
SRR17520069.16647299
SRR17520069.16647300
SRR17520069.16647301
SRR17520069.16647302
SRR17520069.16647305
SRR17520069.16647306

SRR17520069.16647307
SRR17520069.16647308
SRR17520069.16647309
SRR17520069.16647310
SRR17520069.16647311
SRR17520069.16647312
SRR17520069.16647313
SRR17520069.16647314
SRR17520069.16647315
SRR17520069.16647316
SRR17520069.16647317
SRR17520069.16647320
SRR17520069.16647321
SRR17520069.16647322
SRR17520069.16647323
SRR17520069.16647324
SRR17520069.16647325
SRR17520069.16647326
SRR17520069.16647327
SRR17520069.16647328
SRR17520069.16647329
SRR17520069.16647330
SRR17520069.16647331
SRR17520069.16647332
SRR17520069.16647333
SRR17520069.16647334
SRR17520069.16647335
SRR17520069.16647336
SRR17520069.16647337
SRR17520069.16647338
SRR17520069.16647340
SRR17520069.16647341
SRR17520069.16647342
SRR17520069.16647343
SRR17520069.16647344
SRR17520069.16647345
SRR17520069.16647346
SRR17520069.16647347
SRR17520069.16647348
SRR17520069.16647349
SRR17520069.16647350
SRR17520069.16647351
SRR17520069.16647352
SRR17520069.16647353
SRR17520069.16647354
SRR17520069.16647355
SRR17520069.16647356

SRR17520069.16647357
SRR17520069.16647358
SRR17520069.16647359
SRR17520069.16647360
SRR17520069.16647361
SRR17520069.16647362
SRR17520069.16647363
SRR17520069.16647364
SRR17520069.16647365
SRR17520069.16647366
SRR17520069.16647367
SRR17520069.16647368
SRR17520069.16647369
SRR17520069.16647370
SRR17520069.16647371
SRR17520069.16647372
SRR17520069.16647373
SRR17520069.16647374
SRR17520069.16647375
SRR17520069.16647376
SRR17520069.16647377
SRR17520069.16647378
SRR17520069.16647379
SRR17520069.16647380
SRR17520069.16647381
SRR17520069.16647382
SRR17520069.16647383
SRR17520069.16647384
SRR17520069.16647385
SRR17520069.16647386
SRR17520069.16647387
SRR17520069.16647388
SRR17520069.16647389
SRR17520069.16647390
SRR17520069.16647391
SRR17520069.16647392
SRR17520069.16647393
SRR17520069.16647394
SRR17520069.16647395
SRR17520069.16647396
SRR17520069.16647397
SRR17520069.16647398
SRR17520069.16647399
SRR17520069.16647400
SRR17520069.16647401
SRR17520069.16647402
SRR17520069.16647403

SRR17520069.16647404
SRR17520069.16647405
SRR17520069.16647406
SRR17520069.16647407
SRR17520069.16647408
SRR17520069.16647409
SRR17520069.16647410
SRR17520069.16647411
SRR17520069.16647412
SRR17520069.16647413
SRR17520069.16647414
SRR17520069.16647415
SRR17520069.16647416
SRR17520069.16647417
SRR17520069.16647418
SRR17520069.16647419
SRR17520069.16647420
SRR17520069.16647421
SRR17520069.16647422
SRR17520069.16647423
SRR17520069.16647424
SRR17520069.16647425
SRR17520069.16647426
SRR17520069.16647427
SRR17520069.16647428
SRR17520069.16647429
SRR17520069.16647430
SRR17520069.16647431
SRR17520069.16647432
SRR17520069.16647433
SRR17520069.16647434
SRR17520069.16647435
SRR17520069.16647436
SRR17520069.16647437
SRR17520069.16647438
SRR17520069.16647439
SRR17520069.16647440
SRR17520069.16647441
SRR17520069.16647442
SRR17520069.16647443
SRR17520069.16647444
SRR17520069.16647446
SRR17520069.16647447
SRR17520069.16647448
SRR17520069.16647449
SRR17520069.16647450
SRR17520069.16647451

SRR17520069.16647452
SRR17520069.16647453
SRR17520069.16647454
SRR17520069.16647455
SRR17520069.16647456
SRR17520069.16647457
SRR17520069.16647458
SRR17520069.16647459
SRR17520069.16647460
SRR17520069.16647461
SRR17520069.16647462
SRR17520069.16647463
SRR17520069.16647464
SRR17520069.16647465
SRR17520069.16647466
SRR17520069.16648354
SRR17520069.16648355
SRR17520069.16648356
SRR17520069.16648357
SRR17520069.16648358
SRR17520069.16648359
SRR17520069.16648360
SRR17520069.16648361
SRR17520069.16648362
SRR17520069.16648363
SRR17520069.16653035
SRR17520069.16653037
SRR17520069.16653038
SRR17520069.16653039
SRR17520069.16655879
SRR17520069.16655880
SRR17520069.16655881
SRR17520069.16655882
SRR17520069.16655883
SRR17520069.16674499
SRR17520069.16674500
SRR17520069.16674501
SRR17520069.16674502
SRR17520069.16674503
SRR17520069.16674504
SRR17520069.16674505
SRR17520069.16674506
SRR17520069.16674507
SRR17520069.16674508
SRR17520069.16674509
SRR17520069.16674510
SRR17520069.16674511

SRR17520069.16674512
SRR17520069.16674513
SRR17520069.16674514
SRR17520069.16674515
SRR17520069.16674516
SRR17520069.16674517
SRR17520069.16674518
SRR17520069.16674519
SRR17520069.16674520
SRR17520069.16674521
SRR17520069.16674522
SRR17520069.16674523
SRR17520069.16674524
SRR17520069.16674525
SRR17520069.16674526
SRR17520069.16674527
SRR17520069.16674528
SRR17520069.16674529
SRR17520069.16697673
SRR17520069.16707124
SRR17520069.16707159
SRR17520069.16710524
SRR17520069.16710525
SRR17520069.16710526
SRR17520069.16710527
SRR17520069.16714821
SRR17520069.16714833
SRR17520069.16714887
SRR17520069.16714904
SRR17520069.16722106
SRR17520069.16748667
SRR17520069.16751333
SRR17520069.16751362
SRR17520069.16753822
SRR17520069.16753828
SRR17520069.16753843
SRR17520069.16753849
SRR17520069.16753882
SRR17520069.16758554
SRR17520069.16758555
SRR17520069.16758556
SRR17520069.16758557
SRR17520069.16758558
SRR17520069.16758559
SRR17520069.16758560
SRR17520069.16758561
SRR17520069.16758562

SRR17520069.16758563
SRR17520069.16758564
SRR17520069.16758565
SRR17520069.16758566
SRR17520069.16758567
SRR17520069.16758568
SRR17520069.16758569
SRR17520069.16758570
SRR17520069.16758571
SRR17520069.16758572
SRR17520069.16758573
SRR17520069.16770320
SRR17520069.16770334
SRR17520069.16795424
SRR17520069.16795425
SRR17520069.16795426
SRR17520069.16795427
SRR17520069.16795428
SRR17520069.16795429
SRR17520069.16795430
SRR17520069.16795431
SRR17520069.16795432
SRR17520069.16795433
SRR17520069.16795434
SRR17520069.16795435
SRR17520069.16795436
SRR17520069.16795437
SRR17520069.16795438
SRR17520069.16795439
SRR17520069.16795440
SRR17520069.16795441
SRR17520069.16795442
SRR17520069.16795443
SRR17520069.16795444
SRR17520069.16795445
SRR17520069.16795446
SRR17520069.16795447
SRR17520069.16795448
SRR17520069.16795449
SRR17520069.16795450
SRR17520069.16795451
SRR17520069.16795452
SRR17520069.16795453
SRR17520069.16795456
SRR17520069.16795457
SRR17520069.16795458
SRR17520069.16795459

SRR17520069.16795460
SRR17520069.16795461
SRR17520069.16795462
SRR17520069.16795463
SRR17520069.16795464
SRR17520069.16795465
SRR17520069.16795466
SRR17520069.16795467
SRR17520069.16795468
SRR17520069.16795477
SRR17520069.16795486
SRR17520069.16795487
SRR17520069.16795489
SRR17520069.16808477
SRR17520069.16808479
SRR17520069.16808480
SRR17520069.16808481
SRR17520069.16808482
SRR17520069.16808483
SRR17520069.16808484
SRR17520069.16808485
SRR17520069.16808486
SRR17520069.16808487
SRR17520069.16808488
SRR17520069.16808489
SRR17520069.16808490
SRR17520069.16808491
SRR17520069.16808492
SRR17520069.16808493
SRR17520069.16808494
SRR17520069.16808495
SRR17520069.16808496
SRR17520069.16808497
SRR17520069.16808498
SRR17520069.16808499
SRR17520069.16808500
SRR17520069.16808501
SRR17520069.16808502
SRR17520069.16808503
SRR17520069.16808504
SRR17520069.16808505
SRR17520069.16808506
SRR17520069.16808507
SRR17520069.16808508
SRR17520069.16808509
SRR17520069.16808510
SRR17520069.16808511

SRR17520069.16808512
SRR17520069.16808513
SRR17520069.16808514
SRR17520069.16808515
SRR17520069.16808516
SRR17520069.16808517
SRR17520069.16808518
SRR17520069.16808519
SRR17520069.16808520
SRR17520069.16808521
SRR17520069.16808522
SRR17520069.16808523
SRR17520069.16808524
SRR17520069.16808525
SRR17520069.16808526
SRR17520069.16808527
SRR17520069.16808528
SRR17520069.16808529
SRR17520069.16808530
SRR17520069.16808531
SRR17520069.16808532
SRR17520069.16808533
SRR17520069.16808534
SRR17520069.16808535
SRR17520069.16808536
SRR17520069.16808537
SRR17520069.16808538
SRR17520069.16808539
SRR17520069.16808540
SRR17520069.16808541
SRR17520069.16808542
SRR17520069.16808543
SRR17520069.16808544
SRR17520069.16808545
SRR17520069.16808546
SRR17520069.16808547
SRR17520069.16808548
SRR17520069.16808549
SRR17520069.16808550
SRR17520069.16808551
SRR17520069.16808552
SRR17520069.16808553
SRR17520069.16808554
SRR17520069.16808555
SRR17520069.16808556
SRR17520069.16808557
SRR17520069.16808558

SRR17520069.16808559
SRR17520069.16808560
SRR17520069.16808561
SRR17520069.16808562
SRR17520069.16808563
SRR17520069.16808564
SRR17520069.16808565
SRR17520069.16808566
SRR17520069.16808567
SRR17520069.16808568
SRR17520069.16808569
SRR17520069.16808570
SRR17520069.16808571
SRR17520069.16808572
SRR17520069.16808573
SRR17520069.16808574
SRR17520069.16808575
SRR17520069.16808576
SRR17520069.16808577
SRR17520069.16808578
SRR17520069.16808579
SRR17520069.16808580
SRR17520069.16808581
SRR17520069.16808582
SRR17520069.16808583
SRR17520069.16808584
SRR17520069.16808585
SRR17520069.16808586
SRR17520069.16808587
SRR17520069.16808588
SRR17520069.16808589
SRR17520069.16808590
SRR17520069.16808591
SRR17520069.16808593
SRR17520069.16808594
SRR17520069.16808595
SRR17520069.16808596
SRR17520069.16808597
SRR17520069.16808598
SRR17520069.16808599
SRR17520069.16808600
SRR17520069.16808601
SRR17520069.16808602
SRR17520069.16808603
SRR17520069.16808604
SRR17520069.16808606
SRR17520069.16808607

SRR17520069.16808608
SRR17520069.16808609
SRR17520069.16808610
SRR17520069.16808611
SRR17520069.16808612
SRR17520069.16808613
SRR17520069.16808614
SRR17520069.16808615
SRR17520069.16808616
SRR17520069.16808617
SRR17520069.16808618
SRR17520069.16808619
SRR17520069.16808620
SRR17520069.16808621
SRR17520069.16808622
SRR17520069.16808623
SRR17520069.16808624
SRR17520069.16808625
SRR17520069.16808626
SRR17520069.16808627
SRR17520069.16808628
SRR17520069.16808629
SRR17520069.16808630
SRR17520069.16808631
SRR17520069.16808632
SRR17520069.16808633
SRR17520069.16808634
SRR17520069.16808635
SRR17520069.16808636
SRR17520069.16808637
SRR17520069.16808638
SRR17520069.16808639
SRR17520069.16808640
SRR17520069.16808641
SRR17520069.16808642
SRR17520069.16808643
SRR17520069.16808644
SRR17520069.16808645
SRR17520069.16808646
SRR17520069.16808647
SRR17520069.16808648
SRR17520069.16808649
SRR17520069.16808650
SRR17520069.16808651
SRR17520069.16808652
SRR17520069.16808653
SRR17520069.16808654

SRR17520069.16808655
SRR17520069.16808656
SRR17520069.16808657
SRR17520069.16808658
SRR17520069.16808659
SRR17520069.16808660
SRR17520069.16808661
SRR17520069.16808662
SRR17520069.16808663
SRR17520069.16808664
SRR17520069.16808665
SRR17520069.16808666
SRR17520069.16808667
SRR17520069.16808668
SRR17520069.16808669
SRR17520069.16808670
SRR17520069.16808671
SRR17520069.16808672
SRR17520069.16808673
SRR17520069.16808674
SRR17520069.16808675
SRR17520069.16808676
SRR17520069.16808677
SRR17520069.16808678
SRR17520069.16808679
SRR17520069.16808680
SRR17520069.16808681
SRR17520069.16808682
SRR17520069.16808683
SRR17520069.16815334
SRR17520069.16815335
SRR17520069.16815336
SRR17520069.16815337
SRR17520069.16815338
SRR17520069.16815339
SRR17520069.16815340
SRR17520069.16815341
SRR17520069.16815342
SRR17520069.16815343
SRR17520069.16816994
SRR17520069.16816995
SRR17520069.16816996
SRR17520069.16816997
SRR17520069.16816998
SRR17520069.16816999
SRR17520069.16817000
SRR17520069.16817001

SRR17520069.16817002
SRR17520069.16817003
SRR17520069.16817004
SRR17520069.16817005
SRR17520069.16817006
SRR17520069.16817007
SRR17520069.16817008
SRR17520069.16817009
SRR17520069.16817010
SRR17520069.16817011
SRR17520069.16817012
SRR17520069.16817013
SRR17520069.16817014
SRR17520069.16817015
SRR17520069.16817016
SRR17520069.16817017
SRR17520069.16817018
SRR17520069.16817019
SRR17520069.16817020
SRR17520069.16817021
SRR17520069.16817022
SRR17520069.16817023
SRR17520069.16817024
SRR17520069.16817025
SRR17520069.16817026
SRR17520069.16817027
SRR17520069.16817028
SRR17520069.16817029
SRR17520069.16817030
SRR17520069.16817031
SRR17520069.16817032
SRR17520069.16817033
SRR17520069.16817034
SRR17520069.16817035
SRR17520069.16817036
SRR17520069.16817037
SRR17520069.16817038
SRR17520069.16817039
SRR17520069.16817040
SRR17520069.16817041
SRR17520069.16817042
SRR17520069.16817043
SRR17520069.16817044
SRR17520069.16817045
SRR17520069.16817046
SRR17520069.16817047
SRR17520069.16817048

SRR17520069.16817049
SRR17520069.16817050
SRR17520069.16817051
SRR17520069.16817052
SRR17520069.16817053
SRR17520069.16817054
SRR17520069.16817055
SRR17520069.16817056
SRR17520069.16817057
SRR17520069.16817058
SRR17520069.16817059
SRR17520069.16817060
SRR17520069.16817061
SRR17520069.16817062
SRR17520069.16817063
SRR17520069.16817064
SRR17520069.16817065
SRR17520069.16817066
SRR17520069.16817067
SRR17520069.16817068
SRR17520069.16817069
SRR17520069.16817070
SRR17520069.16817071
SRR17520069.16817072
SRR17520069.16817073
SRR17520069.16817074
SRR17520069.16817075
SRR17520069.16817076
SRR17520069.16817077
SRR17520069.16817078
SRR17520069.16817079
SRR17520069.16817080
SRR17520069.16817081
SRR17520069.16817082
SRR17520069.16817083
SRR17520069.16817084
SRR17520069.16817085
SRR17520069.16817086
SRR17520069.16817087
SRR17520069.16817088
SRR17520069.16817089
SRR17520069.16817090
SRR17520069.16817092
SRR17520069.16817093
SRR17520069.16817094
SRR17520069.16817095
SRR17520069.16823407

SRR17520069.16823408
SRR17520069.16845032
SRR17520069.16845034
SRR17520069.16845035
SRR17520069.16845036
SRR17520069.16845037
SRR17520069.16845038
SRR17520069.16845039
SRR17520069.16845041
SRR17520069.16845042
SRR17520069.16845043
SRR17520069.16845044
SRR17520069.16845045
SRR17520069.16845046
SRR17520069.16845047
SRR17520069.16845048
SRR17520069.16845049
SRR17520069.16845050
SRR17520069.16845051
SRR17520069.16845052
SRR17520069.16845053
SRR17520069.16845054
SRR17520069.16845055
SRR17520069.16845060
SRR17520069.16845061
SRR17520069.16850974
SRR17520069.16865840
SRR17520069.16865841
SRR17520069.16865842
SRR17520069.16865843
SRR17520069.16865844
SRR17520069.16865845
SRR17520069.16865846
SRR17520069.16865847
SRR17520069.16865848
SRR17520069.16865849
SRR17520069.16865850
SRR17520069.16865851
SRR17520069.16865852
SRR17520069.16865853
SRR17520069.16865854
SRR17520069.16865855
SRR17520069.16865856
SRR17520069.16865857
SRR17520069.16865858
SRR17520069.16865859
SRR17520069.16867224

SRR17520069.16867225
SRR17520069.16867226
SRR17520069.16867227
SRR17520069.16867228
SRR17520069.16867229
SRR17520069.16867230
SRR17520069.16867231
SRR17520069.16867232
SRR17520069.16867233
SRR17520069.16867234
SRR17520069.16867235
SRR17520069.16867236
SRR17520069.16867237
SRR17520069.16867238
SRR17520069.16867239
SRR17520069.16867240
SRR17520069.16867241
SRR17520069.16867242
SRR17520069.16867243
SRR17520069.16867244
SRR17520069.16867245
SRR17520069.16867246
SRR17520069.16867247
SRR17520069.16867248
SRR17520069.16867249
SRR17520069.16867251
SRR17520069.16867252
SRR17520069.16867253
SRR17520069.16867814
SRR17520069.16886557
SRR17520069.16897350
SRR17520069.16897393
SRR17520069.16897433
SRR17520069.16901265
SRR17520069.16901266
SRR17520069.16901267
SRR17520069.16901268
SRR17520069.16914559
SRR17520069.16914561
SRR17520069.16914562
SRR17520069.16914563
SRR17520069.16914564
SRR17520069.16914565
SRR17520069.16914566
SRR17520069.16914567
SRR17520069.16914568
SRR17520069.16914569

SRR17520069.16914570
SRR17520069.16914571
SRR17520069.16914572
SRR17520069.16914573
SRR17520069.16914574
SRR17520069.16914575
SRR17520069.16914576
SRR17520069.16914577
SRR17520069.16914578
SRR17520069.16914579
SRR17520069.16914580
SRR17520069.16914581
SRR17520069.16914582
SRR17520069.16914583
SRR17520069.16914584
SRR17520069.16914585
SRR17520069.16914586
SRR17520069.16914587
SRR17520069.16914588
SRR17520069.16914589
SRR17520069.16914590
SRR17520069.16914591
SRR17520069.16914592
SRR17520069.16914593
SRR17520069.16914594
SRR17520069.16914596
SRR17520069.16914597
SRR17520069.16914598
SRR17520069.16914599
SRR17520069.16914600
SRR17520069.16914601
SRR17520069.16914602
SRR17520069.16914603
SRR17520069.16914604
SRR17520069.16914605
SRR17520069.16914606
SRR17520069.16923421
SRR17520069.16923422
SRR17520069.16923423
SRR17520069.16923424
SRR17520069.16923425
SRR17520069.16923426
SRR17520069.16923427
SRR17520069.16923428
SRR17520069.16923429
SRR17520069.16923430
SRR17520069.16923431

SRR17520069.16923432
SRR17520069.16923433
SRR17520069.16923434
SRR17520069.16923435
SRR17520069.16923436
SRR17520069.16923438
SRR17520069.16923439
SRR17520069.16923440
SRR17520069.16923441
SRR17520069.16923442
SRR17520069.16923443
SRR17520069.16923444
SRR17520069.16923446
SRR17520069.16923447
SRR17520069.16923448
SRR17520069.16923449
SRR17520069.16923450
SRR17520069.16923451
SRR17520069.16925888
SRR17520069.16932727
SRR17520069.16935746
SRR17520069.16935747
SRR17520069.16935748
SRR17520069.16935749
SRR17520069.16935750
SRR17520069.16935751
SRR17520069.16935752
SRR17520069.16935753
SRR17520069.16935754
SRR17520069.16935755
SRR17520069.16935756
SRR17520069.16935757
SRR17520069.16935758
SRR17520069.16935759
SRR17520069.16935760
SRR17520069.16935761
SRR17520069.16935762
SRR17520069.16935763
SRR17520069.16935764
SRR17520069.16935765
SRR17520069.16935766
SRR17520069.16935767
SRR17520069.16935768
SRR17520069.16935769
SRR17520069.16935770
SRR17520069.16935772
SRR17520069.16935773

SRR17520069.16935774
SRR17520069.16935775
SRR17520069.16935776
SRR17520069.16935777
SRR17520069.16935778
SRR17520069.16935779
SRR17520069.16935780
SRR17520069.16935781
SRR17520069.16935782
SRR17520069.16935783
SRR17520069.16935784
SRR17520069.16935785
SRR17520069.16935786
SRR17520069.16935788
SRR17520069.16935789
SRR17520069.16935790
SRR17520069.16935791
SRR17520069.16935792
SRR17520069.16935793
SRR17520069.16935794
SRR17520069.16935795
SRR17520069.16935796
SRR17520069.16935797
SRR17520069.16935798
SRR17520069.16935799
SRR17520069.16935800
SRR17520069.16935801
SRR17520069.16935802
SRR17520069.16935803
SRR17520069.16935804
SRR17520069.16935806
SRR17520069.16935807
SRR17520069.16935808
SRR17520069.16935809
SRR17520069.16935810
SRR17520069.16935811
SRR17520069.16935812
SRR17520069.16935813
SRR17520069.16935814
SRR17520069.16935815
SRR17520069.16935816
SRR17520069.16935817
SRR17520069.16935818
SRR17520069.16935819
SRR17520069.16935820
SRR17520069.16935821
SRR17520069.16935822

SRR17520069.16935823
SRR17520069.16935824
SRR17520069.16935825
SRR17520069.16935826
SRR17520069.16935827
SRR17520069.16935828
SRR17520069.16935829
SRR17520069.16935830
SRR17520069.16935831
SRR17520069.16935832
SRR17520069.16935833
SRR17520069.16935834
SRR17520069.16935835
SRR17520069.16935836
SRR17520069.16935837
SRR17520069.16935838
SRR17520069.16935839
SRR17520069.16935840
SRR17520069.16935841
SRR17520069.16935843
SRR17520069.16935844
SRR17520069.16935845
SRR17520069.16935846
SRR17520069.16935847
SRR17520069.16935848
SRR17520069.16935849
SRR17520069.16935850
SRR17520069.16935851
SRR17520069.16935852
SRR17520069.16935853
SRR17520069.16935854
SRR17520069.16935855
SRR17520069.16935856
SRR17520069.16935857
SRR17520069.16935858
SRR17520069.16935859
SRR17520069.16935860
SRR17520069.16935861
SRR17520069.16935862
SRR17520069.16935863
SRR17520069.16935864
SRR17520069.16935865
SRR17520069.16935866
SRR17520069.16935867
SRR17520069.16935868
SRR17520069.16935869
SRR17520069.16935870

SRR17520069.16935871
SRR17520069.16935872
SRR17520069.16935873
SRR17520069.16935874
SRR17520069.16935875
SRR17520069.16935876
SRR17520069.16935877
SRR17520069.16935878
SRR17520069.16935879
SRR17520069.16935882
SRR17520069.16935883
SRR17520069.16935884
SRR17520069.16935885
SRR17520069.16935886
SRR17520069.16935887
SRR17520069.16935888
SRR17520069.16935889
SRR17520069.16935890
SRR17520069.16935891
SRR17520069.16935892
SRR17520069.16935893
SRR17520069.16935894
SRR17520069.16935895
SRR17520069.16935896
SRR17520069.16935897
SRR17520069.16935898
SRR17520069.16935899
SRR17520069.16935900
SRR17520069.16935901
SRR17520069.16935902
SRR17520069.16935903
SRR17520069.16935904
SRR17520069.16935905
SRR17520069.16948474
SRR17520069.16948475
SRR17520069.16948476
SRR17520069.16948477
SRR17520069.16948478
SRR17520069.16948479
SRR17520069.16948480
SRR17520069.16948481
SRR17520069.16948482
SRR17520069.16948483
SRR17520069.16948484
SRR17520069.16948485
SRR17520069.16948486
SRR17520069.16948487

SRR17520069.16948488
SRR17520069.16948489
SRR17520069.16948490
SRR17520069.16948491
SRR17520069.16948492
SRR17520069.16948493
SRR17520069.16948495
SRR17520069.16948496
SRR17520069.16948497
SRR17520069.16948498
SRR17520069.16948500
SRR17520069.16948501
SRR17520069.16948502
SRR17520069.16948503
SRR17520069.16948504
SRR17520069.16948505
SRR17520069.16948506
SRR17520069.16948508
SRR17520069.16948509
SRR17520069.16948510
SRR17520069.16948511
SRR17520069.16948512
SRR17520069.16948513
SRR17520069.16948514
SRR17520069.16948515
SRR17520069.16948516
SRR17520069.16948517
SRR17520069.16948518
SRR17520069.16948519
SRR17520069.16948520
SRR17520069.16948521
SRR17520069.16948522
SRR17520069.16948523
SRR17520069.16948524
SRR17520069.16948525
SRR17520069.16948526
SRR17520069.16948527
SRR17520069.16948528
SRR17520069.16948529
SRR17520069.16948530
SRR17520069.16948531
SRR17520069.16948532
SRR17520069.16948533
SRR17520069.16948534
SRR17520069.16948535
SRR17520069.16948536
SRR17520069.16948537

SRR17520069.16948542
SRR17520069.16948543
SRR17520069.16948544
SRR17520069.16948545
SRR17520069.16948546
SRR17520069.16948547
SRR17520069.16948548
SRR17520069.16948549
SRR17520069.16948550
SRR17520069.16948551
SRR17520069.16948552
SRR17520069.16948553
SRR17520069.16948554
SRR17520069.16948555
SRR17520069.16948557
SRR17520069.16948558
SRR17520069.16948559
SRR17520069.16948560
SRR17520069.16948561
SRR17520069.16948562
SRR17520069.16948563
SRR17520069.16948564
SRR17520069.16948565
SRR17520069.16948566
SRR17520069.16948567
SRR17520069.16948568
SRR17520069.16948569
SRR17520069.16948570
SRR17520069.16948571
SRR17520069.16948572
SRR17520069.16948573
SRR17520069.16948574
SRR17520069.16948575
SRR17520069.16948576
SRR17520069.16948577
SRR17520069.16948578
SRR17520069.16948579
SRR17520069.16948580
SRR17520069.16948581
SRR17520069.16948582
SRR17520069.16948583
SRR17520069.16948584
SRR17520069.16948585
SRR17520069.16948586
SRR17520069.16948587
SRR17520069.16948588
SRR17520069.16948589

SRR17520069.16948590
SRR17520069.16948591
SRR17520069.16948592
SRR17520069.16948593
SRR17520069.16948594
SRR17520069.16948595
SRR17520069.16948596
SRR17520069.16948597
SRR17520069.16948598
SRR17520069.16948599
SRR17520069.16948600
SRR17520069.16948601
SRR17520069.16948602
SRR17520069.16948603
SRR17520069.16948604
SRR17520069.16948605
SRR17520069.16948606
SRR17520069.16948607
SRR17520069.16948608
SRR17520069.16948609
SRR17520069.16948610
SRR17520069.16948611
SRR17520069.16948612
SRR17520069.16948613
SRR17520069.16948614
SRR17520069.16948615
SRR17520069.16948616
SRR17520069.16948617
SRR17520069.16948618
SRR17520069.16948619
SRR17520069.16948896
SRR17520069.16961215
SRR17520069.16961216
SRR17520069.16961217
SRR17520069.16961218
SRR17520069.16961219
SRR17520069.16961220
SRR17520069.16961221
SRR17520069.16961222
SRR17520069.16961223
SRR17520069.16961224
SRR17520069.16961225
SRR17520069.16961226
SRR17520069.16961227
SRR17520069.16961228
SRR17520069.16961229
SRR17520069.16961230

SRR17520069.16961231
SRR17520069.16961232
SRR17520069.16961233
SRR17520069.16961234
SRR17520069.16961235
SRR17520069.16961236
SRR17520069.16961237
SRR17520069.16961238
SRR17520069.16961239
SRR17520069.16961240
SRR17520069.16961241
SRR17520069.16961242
SRR17520069.16961243
SRR17520069.16961244
SRR17520069.16961245
SRR17520069.16961246
SRR17520069.16961247
SRR17520069.16961248
SRR17520069.16961249
SRR17520069.16961250
SRR17520069.16961251
SRR17520069.16961252
SRR17520069.16961253
SRR17520069.16961254
SRR17520069.16961255
SRR17520069.16961256
SRR17520069.16961257
SRR17520069.16961258
SRR17520069.16961259
SRR17520069.16961260
SRR17520069.16961261
SRR17520069.16961262
SRR17520069.16961263
SRR17520069.16961264
SRR17520069.16961265
SRR17520069.16961266
SRR17520069.16961267
SRR17520069.16961268
SRR17520069.16961269
SRR17520069.16961270
SRR17520069.16961271
SRR17520069.16961272
SRR17520069.16961273
SRR17520069.16961274
SRR17520069.16961275
SRR17520069.16961276
SRR17520069.16961277

SRR17520069.16961278
SRR17520069.16961279
SRR17520069.16961280
SRR17520069.16961281
SRR17520069.16961282
SRR17520069.16961283
SRR17520069.16961284
SRR17520069.16961285
SRR17520069.16961286
SRR17520069.16961287
SRR17520069.16961288
SRR17520069.16961289
SRR17520069.16961290
SRR17520069.16961291
SRR17520069.16961292
SRR17520069.16961293
SRR17520069.16961294
SRR17520069.16961295
SRR17520069.16961296
SRR17520069.16961297
SRR17520069.16961298
SRR17520069.16961299
SRR17520069.16961300
SRR17520069.16961301
SRR17520069.16961302
SRR17520069.16961303
SRR17520069.16961304
SRR17520069.16961305
SRR17520069.16961306
SRR17520069.16961307
SRR17520069.16961308
SRR17520069.16961309
SRR17520069.16961310
SRR17520069.16961311
SRR17520069.16961312
SRR17520069.16961313
SRR17520069.16961314
SRR17520069.16963803
SRR17520069.16963804
SRR17520069.16963805
SRR17520069.16963806
SRR17520069.16963807
SRR17520069.16963808
SRR17520069.16963809
SRR17520069.16963810
SRR17520069.16963811
SRR17520069.16963814

SRR17520069.16963816
SRR17520069.16963819
SRR17520069.16963820
SRR17520069.16963821
SRR17520069.16963822
SRR17520069.16963823
SRR17520069.16963824
SRR17520069.16963826
SRR17520069.16963827
SRR17520069.16963830
SRR17520069.16963831
SRR17520069.16963833
SRR17520069.16963834
SRR17520069.16963835
SRR17520069.16963837
SRR17520069.16963838
SRR17520069.16963841
SRR17520069.16963842
SRR17520069.16963843
SRR17520069.16963844
SRR17520069.16963846
SRR17520069.16963847
SRR17520069.16963849
SRR17520069.16963850
SRR17520069.16963851
SRR17520069.16963852
SRR17520069.16963853
SRR17520069.16963854
SRR17520069.16963856
SRR17520069.16963857
SRR17520069.16963858
SRR17520069.16963860
SRR17520069.16963861
SRR17520069.16963862
SRR17520069.16963863
SRR17520069.16963866
SRR17520069.16963868
SRR17520069.16963869
SRR17520069.16963870
SRR17520069.16963871
SRR17520069.16963873
SRR17520069.16963875
SRR17520069.16963876
SRR17520069.16963877
SRR17520069.16963878
SRR17520069.16963880
SRR17520069.16963881

SRR17520069.16963882
SRR17520069.16975290
SRR17520069.16975291
SRR17520069.16975292
SRR17520069.16975293
SRR17520069.16975294
SRR17520069.16975295
SRR17520069.16975296
SRR17520069.16975297
SRR17520069.16975299
SRR17520069.16975300
SRR17520069.16975301
SRR17520069.16975302
SRR17520069.16975303
SRR17520069.16975304
SRR17520069.16975305
SRR17520069.16975306
SRR17520069.16975307
SRR17520069.16975308
SRR17520069.16975309
SRR17520069.16975310
SRR17520069.16975311
SRR17520069.16975312
SRR17520069.16975313
SRR17520069.16975314
SRR17520069.16975315
SRR17520069.16975316
SRR17520069.16975317
SRR17520069.16975318
SRR17520069.16975319
SRR17520069.16975320
SRR17520069.16975321
SRR17520069.16975322
SRR17520069.16975323
SRR17520069.16975324
SRR17520069.16975325
SRR17520069.16975326
SRR17520069.16975327
SRR17520069.16975328
SRR17520069.16975329
SRR17520069.16975330
SRR17520069.16975331
SRR17520069.16975332
SRR17520069.16975333
SRR17520069.16975334
SRR17520069.16975335
SRR17520069.16975336

SRR17520069.16975337
SRR17520069.16975338
SRR17520069.16975339
SRR17520069.16975340
SRR17520069.16975341
SRR17520069.16975343
SRR17520069.16975344
SRR17520069.16975345
SRR17520069.16975347
SRR17520069.16975349
SRR17520069.16975350
SRR17520069.16975353
SRR17520069.16991338
SRR17520069.17000792
SRR17520069.17000793
SRR17520069.17000794
SRR17520069.17000795
SRR17520069.17000796
SRR17520069.17000797
SRR17520069.17000798
SRR17520069.17000799
SRR17520069.17000800
SRR17520069.17000801
SRR17520069.17000802
SRR17520069.17000803
SRR17520069.17000804
SRR17520069.17000805
SRR17520069.17000806
SRR17520069.17000807
SRR17520069.17000808
SRR17520069.17000809
SRR17520069.17000810
SRR17520069.17000811
SRR17520069.17000812
SRR17520069.17000813
SRR17520069.17000814
SRR17520069.17000815
SRR17520069.17000816
SRR17520069.17000817
SRR17520069.17000818
SRR17520069.17000819
SRR17520069.17000820
SRR17520069.17000821
SRR17520069.17000822
SRR17520069.17000823
SRR17520069.17000824
SRR17520069.17000825

SRR17520069.17000826
SRR17520069.17000827
SRR17520069.17000828
SRR17520069.17000829
SRR17520069.17000831
SRR17520069.17000832
SRR17520069.17000833
SRR17520069.17000834
SRR17520069.17000835
SRR17520069.17000837
SRR17520069.17000839
SRR17520069.17000840
SRR17520069.17000841
SRR17520069.17000842
SRR17520069.17000843
SRR17520069.17000844
SRR17520069.17000845
SRR17520069.17000846
SRR17520069.17000847
SRR17520069.17000848
SRR17520069.17000849
SRR17520069.17000850
SRR17520069.17000851
SRR17520069.17000852
SRR17520069.17000853
SRR17520069.17000854
SRR17520069.17000855
SRR17520069.17000856
SRR17520069.17000857
SRR17520069.17000858
SRR17520069.17000859
SRR17520069.17000860
SRR17520069.17000861
SRR17520069.17000862
SRR17520069.17000863
SRR17520069.17000864
SRR17520069.17000865
SRR17520069.17000866
SRR17520069.17000867
SRR17520069.17000868
SRR17520069.17000869
SRR17520069.17000870
SRR17520069.17000871
SRR17520069.17000872
SRR17520069.17000873
SRR17520069.17000874
SRR17520069.17000875

SRR17520069.17000876
SRR17520069.17000877
SRR17520069.17000878
SRR17520069.17000879
SRR17520069.17000880
SRR17520069.17000881
SRR17520069.17000882
SRR17520069.17000883
SRR17520069.17000884
SRR17520069.17000886
SRR17520069.17000887
SRR17520069.17000888
SRR17520069.17000889
SRR17520069.17000890
SRR17520069.17000891
SRR17520069.17025308
SRR17520069.17027348
SRR17520069.17027349
SRR17520069.17027350
SRR17520069.17027351
SRR17520069.17027352
SRR17520069.17027353
SRR17520069.17027354
SRR17520069.17027356
SRR17520069.17027357
SRR17520069.17027358
SRR17520069.17027361
SRR17520069.17027362
SRR17520069.17043989
SRR17520069.17043990
SRR17520069.17043991
SRR17520069.17043992
SRR17520069.17043993
SRR17520069.17043994
SRR17520069.17043995
SRR17520069.17043996
SRR17520069.17043997
SRR17520069.17043999
SRR17520069.17044000
SRR17520069.17044001
SRR17520069.17044002
SRR17520069.17044003
SRR17520069.17044005
SRR17520069.17044007
SRR17520069.17044008
SRR17520069.17044009
SRR17520069.17044010

SRR17520069.17044011
SRR17520069.17056926
SRR17520069.17056927
SRR17520069.17056928
SRR17520069.17056929
SRR17520069.17056930
SRR17520069.17056933
SRR17520069.17056934
SRR17520069.17056937
SRR17520069.17056938
SRR17520069.17056940
SRR17520069.17056941
SRR17520069.17056942
SRR17520069.17056943
SRR17520069.17056945
SRR17520069.17056946
SRR17520069.17056947
SRR17520069.17056948
SRR17520069.17056949
SRR17520069.17056950
SRR17520069.17056951
SRR17520069.17056952
SRR17520069.17056953
SRR17520069.17056954
SRR17520069.17056955
SRR17520069.17056956
SRR17520069.17056957
SRR17520069.17056958
SRR17520069.17056959
SRR17520069.17056960
SRR17520069.17056961
SRR17520069.17056962
SRR17520069.17056963
SRR17520069.17056964
SRR17520069.17056965
SRR17520069.17056966
SRR17520069.17056967
SRR17520069.17056969
SRR17520069.17056970
SRR17520069.17056971
SRR17520069.17056972
SRR17520069.17056973
SRR17520069.17056974
SRR17520069.17056975
SRR17520069.17056976
SRR17520069.17056977
SRR17520069.17056978

SRR17520069.17056979
SRR17520069.17056980
SRR17520069.17056981
SRR17520069.17056982
SRR17520069.17056983
SRR17520069.17056984
SRR17520069.17056985
SRR17520069.17056986
SRR17520069.17056987
SRR17520069.17056989
SRR17520069.17056990
SRR17520069.17056991
SRR17520069.17056993
SRR17520069.17056994
SRR17520069.17056995
SRR17520069.17056996
SRR17520069.17056997
SRR17520069.17056998
SRR17520069.17056999
SRR17520069.17057001
SRR17520069.17057002
SRR17520069.17061849
SRR17520069.17061850
SRR17520069.17061851
SRR17520069.17061852
SRR17520069.17061853
SRR17520069.17061854
SRR17520069.17061855
SRR17520069.17061856
SRR17520069.17061857
SRR17520069.17061858
SRR17520069.17061859
SRR17520069.17061860
SRR17520069.17061861
SRR17520069.17061862
SRR17520069.17061863
SRR17520069.17061864
SRR17520069.17061865
SRR17520069.17061866
SRR17520069.17061867
SRR17520069.17061868
SRR17520069.17061869
SRR17520069.17061870
SRR17520069.17061871
SRR17520069.17063084
SRR17520069.17063087
SRR17520069.17063088

SRR17520069.17063089
SRR17520069.17063090
SRR17520069.17063091
SRR17520069.17063092
SRR17520069.17063093
SRR17520069.17063094
SRR17520069.17063095
SRR17520069.17063096
SRR17520069.17063097
SRR17520069.17063098
SRR17520069.17063099
SRR17520069.17063100
SRR17520069.17063101
SRR17520069.17063102
SRR17520069.17063103
SRR17520069.17063104
SRR17520069.17063105
SRR17520069.17063106
SRR17520069.17063107
SRR17520069.17063108
SRR17520069.17063109
SRR17520069.17063110
SRR17520069.17063111
SRR17520069.17063112
SRR17520069.17063113
SRR17520069.17063114
SRR17520069.17063115
SRR17520069.17063116
SRR17520069.17063117
SRR17520069.17063118
SRR17520069.17063119
SRR17520069.17063120
SRR17520069.17063121
SRR17520069.17089908
SRR17520069.17089909
SRR17520069.17089910
SRR17520069.17089911
SRR17520069.17089912
SRR17520069.17089914
SRR17520069.17089915
SRR17520069.17089916
SRR17520069.17089917
SRR17520069.17089918
SRR17520069.17089919
SRR17520069.17089920
SRR17520069.17103966
SRR17520069.17103967

SRR17520069.17103968
SRR17520069.17103969
SRR17520069.17103970
SRR17520069.17103971
SRR17520069.17103972
SRR17520069.17103973
SRR17520069.17103974
SRR17520069.17103975
SRR17520069.17103976
SRR17520069.17103977
SRR17520069.17103978
SRR17520069.17103979
SRR17520069.17103980
SRR17520069.17103981
SRR17520069.17103982
SRR17520069.17103983
SRR17520069.17103984
SRR17520069.17103985
SRR17520069.17103986
SRR17520069.17103987
SRR17520069.17103988
SRR17520069.17103989
SRR17520069.17103991
SRR17520069.17103992
SRR17520069.17103993
SRR17520069.17103994
SRR17520069.17103995
SRR17520069.17103996
SRR17520069.17103997
SRR17520069.17103998
SRR17520069.17103999
SRR17520069.17104000
SRR17520069.17104001
SRR17520069.17104002
SRR17520069.17104003
SRR17520069.17104004
SRR17520069.17104005
SRR17520069.17104006
SRR17520069.17104007
SRR17520069.17104008
SRR17520069.17104009
SRR17520069.17104010
SRR17520069.17104012
SRR17520069.17104013
SRR17520069.17104014
SRR17520069.17104015
SRR17520069.17104016

SRR17520069.17104017
SRR17520069.17104018
SRR17520069.17104019
SRR17520069.17104020
SRR17520069.17104022
SRR17520069.17104023
SRR17520069.17104024
SRR17520069.17104025
SRR17520069.17104026
SRR17520069.17104027
SRR17520069.17104028
SRR17520069.17104030
SRR17520069.17104031
SRR17520069.17104032
SRR17520069.17104033
SRR17520069.17104034
SRR17520069.17104035
SRR17520069.17104036
SRR17520069.17104037
SRR17520069.17104038
SRR17520069.17104039
SRR17520069.17104040
SRR17520069.17104041
SRR17520069.17104042
SRR17520069.17104043
SRR17520069.17104044
SRR17520069.17104045
SRR17520069.17104046
SRR17520069.17104047
SRR17520069.17104048
SRR17520069.17104049
SRR17520069.17104050
SRR17520069.17104051
SRR17520069.17104052
SRR17520069.17104053
SRR17520069.17104054
SRR17520069.17104055
SRR17520069.17104056
SRR17520069.17104057
SRR17520069.17104058
SRR17520069.17104059
SRR17520069.17104060
SRR17520069.17104061
SRR17520069.17104062
SRR17520069.17104063
SRR17520069.17104064
SRR17520069.17104065

SRR17520069.17104066
SRR17520069.17104067
SRR17520069.17104068
SRR17520069.17104069
SRR17520069.17104070
SRR17520069.17104071
SRR17520069.17104072
SRR17520069.17104073
SRR17520069.17104074
SRR17520069.17104075
SRR17520069.17104076
SRR17520069.17104077
SRR17520069.17104078
SRR17520069.17104079
SRR17520069.17104080
SRR17520069.17104081
SRR17520069.17131465
SRR17520069.17131466
SRR17520069.17131467
SRR17520069.17131468
SRR17520069.17131469
SRR17520069.17131470
SRR17520069.17131472
SRR17520069.17131473
SRR17520069.17131474
SRR17520069.17131475
SRR17520069.17131476
SRR17520069.17131477
SRR17520069.17131478
SRR17520069.17131479
SRR17520069.17131480
SRR17520069.17131481
SRR17520069.17131482
SRR17520069.17131483
SRR17520069.17131484
SRR17520069.17131485
SRR17520069.17131486
SRR17520069.17131487
SRR17520069.17131489
SRR17520069.17131490
SRR17520069.17131491
SRR17520069.17131492
SRR17520069.17131493
SRR17520069.17131494
SRR17520069.17131495
SRR17520069.17131496
SRR17520069.17131497

SRR17520069.17131498
SRR17520069.17131499
SRR17520069.17131500
SRR17520069.17131501
SRR17520069.17131502
SRR17520069.17131503
SRR17520069.17131504
SRR17520069.17131505
SRR17520069.17131506
SRR17520069.17131507
SRR17520069.17131508
SRR17520069.17131509
SRR17520069.17131510
SRR17520069.17131511
SRR17520069.17131512
SRR17520069.17131513
SRR17520069.17131514
SRR17520069.17131515
SRR17520069.17131516
SRR17520069.17131517
SRR17520069.17131518
SRR17520069.17131519
SRR17520069.17131520
SRR17520069.17131521
SRR17520069.17131522
SRR17520069.17131523
SRR17520069.17131524
SRR17520069.17131525
SRR17520069.17131527
SRR17520069.17131528
SRR17520069.17131529
SRR17520069.17131530
SRR17520069.17131531
SRR17520069.17131532
SRR17520069.17131533
SRR17520069.17131534
SRR17520069.17131535
SRR17520069.17131536
SRR17520069.17131537
SRR17520069.17131538
SRR17520069.17131539
SRR17520069.17131540
SRR17520069.17131542
SRR17520069.17131543
SRR17520069.17131544
SRR17520069.17131545
SRR17520069.17131546

SRR17520069.17131547
SRR17520069.17131548
SRR17520069.17131549
SRR17520069.17131550
SRR17520069.17131551
SRR17520069.17131553
SRR17520069.17131554
SRR17520069.17131555
SRR17520069.17131556
SRR17520069.17131557
SRR17520069.17131558
SRR17520069.17131559
SRR17520069.17131560
SRR17520069.17131561
SRR17520069.17131562
SRR17520069.17131563
SRR17520069.17131564
SRR17520069.17131565
SRR17520069.17131566
SRR17520069.17131567
SRR17520069.17131568
SRR17520069.17131569
SRR17520069.17131571
SRR17520069.17131573
SRR17520069.17131574
SRR17520069.17131575
SRR17520069.17131576
SRR17520069.17131577
SRR17520069.17131578
SRR17520069.17131579
SRR17520069.17131580
SRR17520069.17131581
SRR17520069.17131582
SRR17520069.17131583
SRR17520069.17131586
SRR17520069.17131587
SRR17520069.17131588
SRR17520069.17131589
SRR17520069.17131590
SRR17520069.17131591
SRR17520069.17131592
SRR17520069.17134580
SRR17520069.17134581
SRR17520069.17134582
SRR17520069.17134583
SRR17520069.17134584
SRR17520069.17134585

SRR17520069.17134586
SRR17520069.17134587
SRR17520069.17134588
SRR17520069.17134589
SRR17520069.17134590
SRR17520069.17134591
SRR17520069.17134592
SRR17520069.17134593
SRR17520069.17134595
SRR17520069.17134596
SRR17520069.17134597
SRR17520069.17134598
SRR17520069.17134599
SRR17520069.17134600
SRR17520069.17134601
SRR17520069.17134602
SRR17520069.17134604
SRR17520069.17134605
SRR17520069.17134606
SRR17520069.17134608
SRR17520069.17134609
SRR17520069.17134610
SRR17520069.17134611
SRR17520069.17134612
SRR17520069.17134613
SRR17520069.17134615
SRR17520069.17134616
SRR17520069.17134617
SRR17520069.17134620
SRR17520069.17134622
SRR17520069.17134623
SRR17520069.17134624
SRR17520069.17134625
SRR17520069.17134626
SRR17520069.17134627
SRR17520069.17134628
SRR17520069.17134630
SRR17520069.17134633
SRR17520069.17134636
SRR17520069.17134638
SRR17520069.17134639
SRR17520069.17134640
SRR17520069.17134641
SRR17520069.17134642
SRR17520069.17134643
SRR17520069.17134645
SRR17520069.17134646

SRR17520069.17134647
SRR17520069.17134648
SRR17520069.17134649
SRR17520069.17134650
SRR17520069.17134651
SRR17520069.17134652
SRR17520069.17134653
SRR17520069.17134654
SRR17520069.17134655
SRR17520069.17134656
SRR17520069.17134657
SRR17520069.17134658
SRR17520069.17134659
SRR17520069.17134660
SRR17520069.17134661
SRR17520069.17134662
SRR17520069.17142440
SRR17520069.17142441
SRR17520069.17142442
SRR17520069.17142445
SRR17520069.17142446
SRR17520069.17142447
SRR17520069.17142449
SRR17520069.17142450
SRR17520069.17142451
SRR17520069.17142452
SRR17520069.17142453
SRR17520069.17142454
SRR17520069.17142458
SRR17520069.17142459
SRR17520069.17142461
SRR17520069.17142462
SRR17520069.17142463
SRR17520069.17142464
SRR17520069.17159509
SRR17520069.17159510
SRR17520069.17159511
SRR17520069.17159512
SRR17520069.17159513
SRR17520069.17159514
SRR17520069.17159515
SRR17520069.17159516
SRR17520069.17159517
SRR17520069.17159518
SRR17520069.17159519
SRR17520069.17159520
SRR17520069.17159521

SRR17520069.17159522
SRR17520069.17159523
SRR17520069.17159524
SRR17520069.17159525
SRR17520069.17159526
SRR17520069.17159527
SRR17520069.17159528
SRR17520069.17159529
SRR17520069.17159530
SRR17520069.17159531
SRR17520069.17159532
SRR17520069.17159533
SRR17520069.17159535
SRR17520069.17159536
SRR17520069.17159537
SRR17520069.17159538
SRR17520069.17159539
SRR17520069.17159540
SRR17520069.17159541
SRR17520069.17159542
SRR17520069.17159543
SRR17520069.17159544
SRR17520069.17159545
SRR17520069.17159546
SRR17520069.17159548
SRR17520069.17159549
SRR17520069.17159550
SRR17520069.17159551
SRR17520069.17159552
SRR17520069.17159553
SRR17520069.17159554
SRR17520069.17159555
SRR17520069.17159556
SRR17520069.17159557
SRR17520069.17159558
SRR17520069.17159559
SRR17520069.17159560
SRR17520069.17159561
SRR17520069.17159562
SRR17520069.17159563
SRR17520069.17159564
SRR17520069.17159565
SRR17520069.17159566
SRR17520069.17159567
SRR17520069.17159568
SRR17520069.17159569
SRR17520069.17159570

SRR17520069.17159571
SRR17520069.17159572
SRR17520069.17159573
SRR17520069.17159574
SRR17520069.17159575
SRR17520069.17159576
SRR17520069.17159577
SRR17520069.17159578
SRR17520069.17159579
SRR17520069.17159580
SRR17520069.17159581
SRR17520069.17159582
SRR17520069.17159583
SRR17520069.17159584
SRR17520069.17159585
SRR17520069.17159586
SRR17520069.17159587
SRR17520069.17179011
SRR17520069.17179012
SRR17520069.17179013
SRR17520069.17179014
SRR17520069.17179015
SRR17520069.17179016
SRR17520069.17179017
SRR17520069.17179018
SRR17520069.17179019
SRR17520069.17179020
SRR17520069.17179021
SRR17520069.17179022
SRR17520069.17179023
SRR17520069.17179024
SRR17520069.17179025
SRR17520069.17179026
SRR17520069.17179027
SRR17520069.17179028
SRR17520069.17179029
SRR17520069.17179030
SRR17520069.17179031
SRR17520069.17179032
SRR17520069.17179033
SRR17520069.17179034
SRR17520069.17179035
SRR17520069.17179036
SRR17520069.17179037
SRR17520069.17179038
SRR17520069.17179039
SRR17520069.17179040

SRR17520069.17179041
SRR17520069.17194464
SRR17520069.17204307
SRR17520069.17204308
SRR17520069.17204309
SRR17520069.17204310
SRR17520069.17204311
SRR17520069.17204312
SRR17520069.17204313
SRR17520069.17204314
SRR17520069.17204315
SRR17520069.17204316
SRR17520069.17204317
SRR17520069.17204318
SRR17520069.17204319
SRR17520069.17204320
SRR17520069.17204321
SRR17520069.17204322
SRR17520069.17204323
SRR17520069.17204324
SRR17520069.17204325
SRR17520069.17204326
SRR17520069.17204327
SRR17520069.17204328
SRR17520069.17204329
SRR17520069.17204330
SRR17520069.17204331
SRR17520069.17204332
SRR17520069.17204334
SRR17520069.17204335
SRR17520069.17204336
SRR17520069.17204337
SRR17520069.17204338
SRR17520069.17204339
SRR17520069.17204340
SRR17520069.17204341
SRR17520069.17204342
SRR17520069.17204345
SRR17520069.17204346
SRR17520069.17204347
SRR17520069.17204348
SRR17520069.17204349
SRR17520069.17204350
SRR17520069.17204352
SRR17520069.17204355
SRR17520069.17204356
SRR17520069.17207746

SRR17520069.17235907
SRR17520069.17235908
SRR17520069.17235909
SRR17520069.17235910
SRR17520069.17235911
SRR17520069.17235912
SRR17520069.17235913
SRR17520069.17235914
SRR17520069.17235915
SRR17520069.17235916
SRR17520069.17235917
SRR17520069.17235918
SRR17520069.17235919
SRR17520069.17235920
SRR17520069.17235921
SRR17520069.17235922
SRR17520069.17235923
SRR17520069.17235924
SRR17520069.17235925
SRR17520069.17235926
SRR17520069.17235927
SRR17520069.17235928
SRR17520069.17235929
SRR17520069.17235930
SRR17520069.17235931
SRR17520069.17235932
SRR17520069.17235933
SRR17520069.17235934
SRR17520069.17235935
SRR17520069.17235936
SRR17520069.17235937
SRR17520069.17235938
SRR17520069.17235939
SRR17520069.17235940
SRR17520069.17235941
SRR17520069.17235942
SRR17520069.17235943
SRR17520069.17235944
SRR17520069.17235945
SRR17520069.17235946
SRR17520069.17235947
SRR17520069.17235948
SRR17520069.17235949
SRR17520069.17235950
SRR17520069.17254383
SRR17520069.17274981
SRR17520069.17275324

SRR17520069.17275325
SRR17520069.17275326
SRR17520069.17275327
SRR17520069.17275328
SRR17520069.17275329
SRR17520069.17275330
SRR17520069.17275331
SRR17520069.17275332
SRR17520069.17275334
SRR17520069.17275335
SRR17520069.17275336
SRR17520069.17275337
SRR17520069.17275338
SRR17520069.17275339
SRR17520069.17275340
SRR17520069.17275341
SRR17520069.17275342
SRR17520069.17275343
SRR17520069.17275344
SRR17520069.17275345
SRR17520069.17275346
SRR17520069.17275347
SRR17520069.17275348
SRR17520069.17275349
SRR17520069.17275350
SRR17520069.17275351
SRR17520069.17275352
SRR17520069.17275353
SRR17520069.17275354
SRR17520069.17275355
SRR17520069.17275356
SRR17520069.17275357
SRR17520069.17275358
SRR17520069.17275359
SRR17520069.17275360
SRR17520069.17275361
SRR17520069.17275362
SRR17520069.17275363
SRR17520069.17275364
SRR17520069.17275365
SRR17520069.17275366
SRR17520069.17275367
SRR17520069.17275368
SRR17520069.17278067
SRR17520069.17278074
SRR17520069.17278128
SRR17520069.17285200

SRR17520069.17290839
SRR17520069.17293940
SRR17520069.17293941
SRR17520069.17293942
SRR17520069.17293943
SRR17520069.17293944
SRR17520069.17293945
SRR17520069.17293946
SRR17520069.17293947
SRR17520069.17293948
SRR17520069.17293949
SRR17520069.17306347
SRR17520069.17306348
SRR17520069.17306349
SRR17520069.17306351
SRR17520069.17306352
SRR17520069.17306353
SRR17520069.17306355
SRR17520069.17306356
SRR17520069.17306357
SRR17520069.17306358
SRR17520069.17306576
SRR17520069.17322267
SRR17520069.17337137
SRR17520069.17337138
SRR17520069.17337139
SRR17520069.17337140
SRR17520069.17337141
SRR17520069.17337142
SRR17520069.17337143
SRR17520069.17337144
SRR17520069.17337145
SRR17520069.17337147
SRR17520069.17337148
SRR17520069.17337149
SRR17520069.17337150
SRR17520069.17337151
SRR17520069.17337152
SRR17520069.17337153
SRR17520069.17337155
SRR17520069.17337156
SRR17520069.17337157
SRR17520069.17337158
SRR17520069.17337159
SRR17520069.17337160
SRR17520069.17337161
SRR17520069.17337162

SRR17520069.17337163
SRR17520069.17337164
SRR17520069.17337165
SRR17520069.17337166
SRR17520069.17337167
SRR17520069.17337168
SRR17520069.17337169
SRR17520069.17337170
SRR17520069.17337171
SRR17520069.17337172
SRR17520069.17337173
SRR17520069.17337174
SRR17520069.17337175
SRR17520069.17337176
SRR17520069.17337177
SRR17520069.17337178
SRR17520069.17337179
SRR17520069.17337180
SRR17520069.17337181
SRR17520069.17337182
SRR17520069.17337183
SRR17520069.17337184
SRR17520069.17337185
SRR17520069.17337187
SRR17520069.17337189
SRR17520069.17337190
SRR17520069.17337191
SRR17520069.17337192
SRR17520069.17337193
SRR17520069.17337194
SRR17520069.17337195
SRR17520069.17337196
SRR17520069.17337197
SRR17520069.17337198
SRR17520069.17337199
SRR17520069.17337200
SRR17520069.17337201
SRR17520069.17337202
SRR17520069.17337203
SRR17520069.17337204
SRR17520069.17337205
SRR17520069.17337206
SRR17520069.17369465
SRR17520069.17369466
SRR17520069.17369467
SRR17520069.17369468
SRR17520069.17377594

SRR17520069.17377596
SRR17520069.17386050
SRR17520069.17386051
SRR17520069.17386052
SRR17520069.17386053
SRR17520069.17386054
SRR17520069.17386055
SRR17520069.17386056
SRR17520069.17386057
SRR17520069.17386058
SRR17520069.17386060
SRR17520069.17386061
SRR17520069.17386062
SRR17520069.17386063
SRR17520069.17386064
SRR17520069.17386065
SRR17520069.17386069
SRR17520069.17386071
SRR17520069.17386072
SRR17520069.17386073
SRR17520069.17386074
SRR17520069.17386075
SRR17520069.17386076
SRR17520069.17386077
SRR17520069.17386078
SRR17520069.17386079
SRR17520069.17386080
SRR17520069.17386081
SRR17520069.17386082
SRR17520069.17386083
SRR17520069.17386084
SRR17520069.17386085
SRR17520069.17386086
SRR17520069.17386087
SRR17520069.17386088
SRR17520069.17386089
SRR17520069.17386090
SRR17520069.17386091
SRR17520069.17386092
SRR17520069.17386093
SRR17520069.17386094
SRR17520069.17386095
SRR17520069.17386096
SRR17520069.17386097
SRR17520069.17386099
SRR17520069.17386100
SRR17520069.17403768

SRR17520069.17403784
SRR17520069.17407600
SRR17520069.17407610
SRR17520069.17407629
SRR17520069.17407668
SRR17520069.17407708
SRR17520069.17407715
SRR17520069.17410230
SRR17520069.17410231
SRR17520069.17410232
SRR17520069.17410233
SRR17520069.17410234
SRR17520069.17410235
SRR17520069.17410236
SRR17520069.17410237
SRR17520069.17410238
SRR17520069.17410239
SRR17520069.17410240
SRR17520069.17410241
SRR17520069.17410242
SRR17520069.17410243
SRR17520069.17410244
SRR17520069.17410245
SRR17520069.17410246
SRR17520069.17410247
SRR17520069.17410248
SRR17520069.17410249
SRR17520069.17410250
SRR17520069.17410251
SRR17520069.17410252
SRR17520069.17410253
SRR17520069.17410254
SRR17520069.17410255
SRR17520069.17410256
SRR17520069.17410257
SRR17520069.17410258
SRR17520069.17410259
SRR17520069.17410260
SRR17520069.17410261
SRR17520069.17410262
SRR17520069.17410263
SRR17520069.17410264
SRR17520069.17410265
SRR17520069.17410266
SRR17520069.17410267
SRR17520069.17410268
SRR17520069.17410269

SRR17520069.17410270
SRR17520069.17410271
SRR17520069.17410272
SRR17520069.17410273
SRR17520069.17410274
SRR17520069.17410275
SRR17520069.17410276
SRR17520069.17410277
SRR17520069.17410278
SRR17520069.17410279
SRR17520069.17410280
SRR17520069.17410281
SRR17520069.17410282
SRR17520069.17410283
SRR17520069.17410284
SRR17520069.17410285
SRR17520069.17410286
SRR17520069.17410287
SRR17520069.17410288
SRR17520069.17410289
SRR17520069.17410290
SRR17520069.17410291
SRR17520069.17410292
SRR17520069.17410294
SRR17520069.17410295
SRR17520069.17410296
SRR17520069.17410297
SRR17520069.17410298
SRR17520069.17410299
SRR17520069.17410300
SRR17520069.17410301
SRR17520069.17410302
SRR17520069.17410303
SRR17520069.17410304
SRR17520069.17410305
SRR17520069.17410306
SRR17520069.17410307
SRR17520069.17410308
SRR17520069.17410310
SRR17520069.17410311
SRR17520069.17410312
SRR17520069.17410313
SRR17520069.17410878
SRR17520069.17410879
SRR17520069.17410880
SRR17520069.17410881
SRR17520069.17410882

SRR17520069.17410883
SRR17520069.17410884
SRR17520069.17410885
SRR17520069.17410886
SRR17520069.17410887
SRR17520069.17410888
SRR17520069.17410889
SRR17520069.17410890
SRR17520069.17410891
SRR17520069.17410892
SRR17520069.17410893
SRR17520069.17410894
SRR17520069.17410895
SRR17520069.17410896
SRR17520069.17427372
SRR17520069.17427373
SRR17520069.17427375
SRR17520069.17427377
SRR17520069.17427378
SRR17520069.17427380
SRR17520069.17427381
SRR17520069.17427382
SRR17520069.17427383
SRR17520069.17427384
SRR17520069.17427385
SRR17520069.17427386
SRR17520069.17427387
SRR17520069.17427388
SRR17520069.17427389
SRR17520069.17427391
SRR17520069.17427392
SRR17520069.17427393
SRR17520069.17427394
SRR17520069.17427395
SRR17520069.17427396
SRR17520069.17427397
SRR17520069.17427398
SRR17520069.17427402
SRR17520069.17427404
SRR17520069.17427405
SRR17520069.17427406
SRR17520069.17427407
SRR17520069.17427408
SRR17520069.17427409
SRR17520069.17427410
SRR17520069.17427411
SRR17520069.17427413

SRR17520069.17427414
SRR17520069.17427415
SRR17520069.17427416
SRR17520069.17427417
SRR17520069.17427418
SRR17520069.17427420
SRR17520069.17427421
SRR17520069.17427422
SRR17520069.17427423
SRR17520069.17427424
SRR17520069.17427425
SRR17520069.17427426
SRR17520069.17427427
SRR17520069.17427428
SRR17520069.17427429
SRR17520069.17427430
SRR17520069.17427432
SRR17520069.17427433
SRR17520069.17427434
SRR17520069.17427435
SRR17520069.17427436
SRR17520069.17427438
SRR17520069.17427439
SRR17520069.17427441
SRR17520069.17427442
SRR17520069.17427443
SRR17520069.17427444
SRR17520069.17427445
SRR17520069.17427446
SRR17520069.17427447
SRR17520069.17427448
SRR17520069.17427449
SRR17520069.17427450
SRR17520069.17427451
SRR17520069.17427453
SRR17520069.17427454
SRR17520069.17427455
SRR17520069.17427456
SRR17520069.17427457
SRR17520069.17427460
SRR17520069.17427461
SRR17520069.17427462
SRR17520069.17427463
SRR17520069.17427465
SRR17520069.17427467
SRR17520069.17427468
SRR17520069.17427469

SRR17520069.17427470
SRR17520069.17427471
SRR17520069.17427474
SRR17520069.17427475
SRR17520069.17427476
SRR17520069.17427477
SRR17520069.17427478
SRR17520069.17427479
SRR17520069.17427480
SRR17520069.17427481
SRR17520069.17427482
SRR17520069.17427483
SRR17520069.17427484
SRR17520069.17427486
SRR17520069.17427489
SRR17520069.17427490
SRR17520069.17427492
SRR17520069.17427493
SRR17520069.17427494
SRR17520069.17427495
SRR17520069.17427496
SRR17520069.17427497
SRR17520069.17427498
SRR17520069.17427499
SRR17520069.17427500
SRR17520069.17427501
SRR17520069.17427502
SRR17520069.17427503
SRR17520069.17427504
SRR17520069.17456031
SRR17520069.17456032
SRR17520069.17456033
SRR17520069.17456034
SRR17520069.17456035
SRR17520069.17456036
SRR17520069.17456037
SRR17520069.17456038
SRR17520069.17456040
SRR17520069.17456041
SRR17520069.17456042
SRR17520069.17456043
SRR17520069.17456044
SRR17520069.17456045
SRR17520069.17456046
SRR17520069.17456047
SRR17520069.17456050
SRR17520069.17456051

SRR17520069.17456052
SRR17520069.17456053
SRR17520069.17456054
SRR17520069.17456055
SRR17520069.17456056
SRR17520069.17456057
SRR17520069.17456058
SRR17520069.17456059
SRR17520069.17456060
SRR17520069.17456061
SRR17520069.17456062
SRR17520069.17456063
SRR17520069.17456064
SRR17520069.17456065
SRR17520069.17456066
SRR17520069.17456067
SRR17520069.17456068
SRR17520069.17456069
SRR17520069.17456070
SRR17520069.17456071
SRR17520069.17456072
SRR17520069.17456073
SRR17520069.17456074
SRR17520069.17456075
SRR17520069.17456076
SRR17520069.17456077
SRR17520069.17456078
SRR17520069.17456079
SRR17520069.17456080
SRR17520069.17456081
SRR17520069.17456082
SRR17520069.17456083
SRR17520069.17456084
SRR17520069.17456085
SRR17520069.17456086
SRR17520069.17456087
SRR17520069.17456088
SRR17520069.17456089
SRR17520069.17456090
SRR17520069.17456091
SRR17520069.17456092
SRR17520069.17456093
SRR17520069.17456094
SRR17520069.17456095
SRR17520069.17456096
SRR17520069.17456097
SRR17520069.17456098

SRR17520069.17456099
SRR17520069.17456100
SRR17520069.17456211
SRR17520069.17456212
SRR17520069.17456213
SRR17520069.17456214
SRR17520069.17456215
SRR17520069.17456216
SRR17520069.17456217
SRR17520069.17456218
SRR17520069.17456219
SRR17520069.17456220
SRR17520069.17456221
SRR17520069.17456222
SRR17520069.17456223
SRR17520069.17456224
SRR17520069.17456225
SRR17520069.17456226
SRR17520069.17456227
SRR17520069.17456228
SRR17520069.17456229
SRR17520069.17456230
SRR17520069.17456231
SRR17520069.17462279
SRR17520069.17462280
SRR17520069.17462281
SRR17520069.17462282
SRR17520069.17462283
SRR17520069.17462284
SRR17520069.17462285
SRR17520069.17462286
SRR17520069.17462287
SRR17520069.17462288
SRR17520069.17462289
SRR17520069.17462290
SRR17520069.17462291
SRR17520069.17462292
SRR17520069.17462293
SRR17520069.17462294
SRR17520069.17462295
SRR17520069.17462296
SRR17520069.17462297
SRR17520069.17462298
SRR17520069.17462299
SRR17520069.17462300
SRR17520069.17462301
SRR17520069.17462302

SRR17520069.17462303
SRR17520069.17462304
SRR17520069.17462305
SRR17520069.17462306
SRR17520069.17462307
SRR17520069.17472140
SRR17520069.17484068
SRR17520069.17484069
SRR17520069.17484070
SRR17520069.17484071
SRR17520069.17484072
SRR17520069.17484076
SRR17520069.17492796
SRR17520069.17492797
SRR17520069.17492798
SRR17520069.17492799
SRR17520069.17492800
SRR17520069.17492801
SRR17520069.17492802
SRR17520069.17492803
SRR17520069.17492804
SRR17520069.17492805
SRR17520069.17492806
SRR17520069.17492807
SRR17520069.17492808
SRR17520069.17492809
SRR17520069.17492810
SRR17520069.17492811
SRR17520069.17492812
SRR17520069.17492813
SRR17520069.17492814
SRR17520069.17492815
SRR17520069.17492816
SRR17520069.17492817
SRR17520069.17492818
SRR17520069.17492819
SRR17520069.17492820
SRR17520069.17492821
SRR17520069.17492822
SRR17520069.17492823
SRR17520069.17492824
SRR17520069.17492825
SRR17520069.17492826
SRR17520069.17492827
SRR17520069.17492828
SRR17520069.17492829
SRR17520069.17492830

SRR17520069.17492831
SRR17520069.17492832
SRR17520069.17492833
SRR17520069.17492834
SRR17520069.17492836
SRR17520069.17492837
SRR17520069.17492838
SRR17520069.17492839
SRR17520069.17492840
SRR17520069.17492841
SRR17520069.17492842
SRR17520069.17492843
SRR17520069.17492844
SRR17520069.17492845
SRR17520069.17492846
SRR17520069.17492847
SRR17520069.17492848
SRR17520069.17496142
SRR17520069.17496143
SRR17520069.17496144
SRR17520069.17496145
SRR17520069.17496146
SRR17520069.17496147
SRR17520069.17496148
SRR17520069.17496149
SRR17520069.17496150
SRR17520069.17496151
SRR17520069.17496152
SRR17520069.17496153
SRR17520069.17496154
SRR17520069.17496155
SRR17520069.17496156
SRR17520069.17496157
SRR17520069.17496158
SRR17520069.17496159
SRR17520069.17496160
SRR17520069.17496161
SRR17520069.17496162
SRR17520069.17496163
SRR17520069.17496164
SRR17520069.17496165
SRR17520069.17496166
SRR17520069.17496167
SRR17520069.17496168
SRR17520069.17496169
SRR17520069.17496170
SRR17520069.17496171

SRR17520069.17496172
SRR17520069.17496173
SRR17520069.17496174
SRR17520069.17496175
SRR17520069.17496176
SRR17520069.17496177
SRR17520069.17496178
SRR17520069.17496179
SRR17520069.17496180
SRR17520069.17496181
SRR17520069.17496182
SRR17520069.17496183
SRR17520069.17496184
SRR17520069.17496185
SRR17520069.17496186
SRR17520069.17496187
SRR17520069.17496188
SRR17520069.17496189
SRR17520069.17496190
SRR17520069.17496191
SRR17520069.17496192
SRR17520069.17496193
SRR17520069.17496194
SRR17520069.17496195
SRR17520069.17496196
SRR17520069.17496197
SRR17520069.17496198
SRR17520069.17496199
SRR17520069.17496200
SRR17520069.17496201
SRR17520069.17496202
SRR17520069.17496203
SRR17520069.17496204
SRR17520069.17496205
SRR17520069.17496206
SRR17520069.17496207
SRR17520069.17496208
SRR17520069.17496209
SRR17520069.17496210
SRR17520069.17496211
SRR17520069.17496212
SRR17520069.17496213
SRR17520069.17496214
SRR17520069.17511695
SRR17520069.17511741
SRR17520069.17529963
SRR17520069.17529964

SRR17520069.17529966
SRR17520069.17529967
SRR17520069.17529968
SRR17520069.17529969
SRR17520069.17529970
SRR17520069.17529971
SRR17520069.17529972
SRR17520069.17529973
SRR17520069.17529974
SRR17520069.17529975
SRR17520069.17529976
SRR17520069.17529977
SRR17520069.17529978
SRR17520069.17529979
SRR17520069.17529980
SRR17520069.17529981
SRR17520069.17529982
SRR17520069.17529983
SRR17520069.17529984
SRR17520069.17529985
SRR17520069.17529986
SRR17520069.17529987
SRR17520069.17529988
SRR17520069.17529989
SRR17520069.17529990
SRR17520069.17529991
SRR17520069.17529992
SRR17520069.17529993
SRR17520069.17529994
SRR17520069.17529995
SRR17520069.17529996
SRR17520069.17529997
SRR17520069.17529998
SRR17520069.17529999
SRR17520069.17530000
SRR17520069.17530001
SRR17520069.17530002
SRR17520069.17530003
SRR17520069.17530004
SRR17520069.17530005
SRR17520069.17530006
SRR17520069.17530007
SRR17520069.17530008
SRR17520069.17530009
SRR17520069.17530010
SRR17520069.17530011
SRR17520069.17530012

SRR17520069.17530013
SRR17520069.17530014
SRR17520069.17530015
SRR17520069.17532933
SRR17520069.17541219
SRR17520069.17541220
SRR17520069.17541221
SRR17520069.17541223
SRR17520069.17541224
SRR17520069.17541225
SRR17520069.17541226
SRR17520069.17541227
SRR17520069.17541228
SRR17520069.17541229
SRR17520069.17541230
SRR17520069.17548073
SRR17520069.17550865
SRR17520069.17550866
SRR17520069.17550867
SRR17520069.17550868
SRR17520069.17550869
SRR17520069.17550870
SRR17520069.17550871
SRR17520069.17550872
SRR17520069.17550873
SRR17520069.17550874
SRR17520069.17550875
SRR17520069.17550876
SRR17520069.17550877
SRR17520069.17550878
SRR17520069.17550879
SRR17520069.17550880
SRR17520069.17550881
SRR17520069.17550882
SRR17520069.17551250
SRR17520069.17551251
SRR17520069.17551252
SRR17520069.17551253
SRR17520069.17551254
SRR17520069.17551255
SRR17520069.17551256
SRR17520069.17551257
SRR17520069.17551258
SRR17520069.17551259
SRR17520069.17551260
SRR17520069.17551261
SRR17520069.17551262

SRR17520069.17551263
SRR17520069.17551264
SRR17520069.17551265
SRR17520069.17557156
SRR17520069.17557159
SRR17520069.17557160
SRR17520069.17557161
SRR17520069.17557162
SRR17520069.17557163
SRR17520069.17557164
SRR17520069.17557166
SRR17520069.17557167
SRR17520069.17557168
SRR17520069.17557169
SRR17520069.17557170
SRR17520069.17557171
SRR17520069.17557172
SRR17520069.17557173
SRR17520069.17557174
SRR17520069.17557175
SRR17520069.17557176
SRR17520069.17557177
SRR17520069.17557178
SRR17520069.17557179
SRR17520069.17557180
SRR17520069.17576503
SRR17520069.17576645
SRR17520069.17576646
SRR17520069.17581365
SRR17520069.17583241
SRR17520069.17583242
SRR17520069.17583243
SRR17520069.17583244
SRR17520069.17583245
SRR17520069.17583246
SRR17520069.17583247
SRR17520069.17583248
SRR17520069.17583249
SRR17520069.17583250
SRR17520069.17583251
SRR17520069.17583252
SRR17520069.17583253
SRR17520069.17583254
SRR17520069.17583255
SRR17520069.17583256
SRR17520069.17583258
SRR17520069.17583259

SRR17520069.17583260
SRR17520069.17583261
SRR17520069.17583262
SRR17520069.17583264
SRR17520069.17583265
SRR17520069.17583266
SRR17520069.17583267
SRR17520069.17583268
SRR17520069.17583269
SRR17520069.17584662
SRR17520069.17593855
SRR17520069.17593856
SRR17520069.17593858
SRR17520069.17593859
SRR17520069.17593860
SRR17520069.17593861
SRR17520069.17593862
SRR17520069.17593863
SRR17520069.17593864
SRR17520069.17593865
SRR17520069.17593866
SRR17520069.17593867
SRR17520069.17628536
SRR17520069.17628560
SRR17520069.17628571
SRR17520069.17641311
SRR17520069.17641312
SRR17520069.17641313
SRR17520069.17641314
SRR17520069.17641315
SRR17520069.17641316
SRR17520069.17641317
SRR17520069.17641318
SRR17520069.17641319
SRR17520069.17641320
SRR17520069.17641321
SRR17520069.17641322
SRR17520069.17641323
SRR17520069.17641324
SRR17520069.17641325
SRR17520069.17641326
SRR17520069.17641327
SRR17520069.17641328
SRR17520069.17641329
SRR17520069.17641330
SRR17520069.17641331
SRR17520069.17641332

SRR17520069.17641333
SRR17520069.17641334
SRR17520069.17641335
SRR17520069.17641336
SRR17520069.17641337
SRR17520069.17641338
SRR17520069.17641339
SRR17520069.17641340
SRR17520069.17641341
SRR17520069.17641342
SRR17520069.17641343
SRR17520069.17641344
SRR17520069.17641345
SRR17520069.17641346
SRR17520069.17641347
SRR17520069.17641348
SRR17520069.17641349
SRR17520069.17641350
SRR17520069.17641351
SRR17520069.17641352
SRR17520069.17641353
SRR17520069.17641354
SRR17520069.17641355
SRR17520069.17641356
SRR17520069.17641357
SRR17520069.17641358
SRR17520069.17641359
SRR17520069.17641360
SRR17520069.17641361
SRR17520069.17641362
SRR17520069.17641363
SRR17520069.17641364
SRR17520069.17641365
SRR17520069.17641366
SRR17520069.17641367
SRR17520069.17641368
SRR17520069.17641369
SRR17520069.17641370
SRR17520069.17641371
SRR17520069.17641372
SRR17520069.17641373
SRR17520069.17641374
SRR17520069.17641375
SRR17520069.17641376
SRR17520069.17641378
SRR17520069.17682695
SRR17520069.17692097

SRR17520069.17692098
SRR17520069.17692099
SRR17520069.17692100
SRR17520069.17692101
SRR17520069.17692102
SRR17520069.17692103
SRR17520069.17692104
SRR17520069.17692105
SRR17520069.17692106
SRR17520069.17692107
SRR17520069.17692108
SRR17520069.17692109
SRR17520069.17692110
SRR17520069.17692111
SRR17520069.17692112
SRR17520069.17692113
SRR17520069.17692114
SRR17520069.17692115
SRR17520069.17692116
SRR17520069.17692117
SRR17520069.17692118
SRR17520069.17692119
SRR17520069.17692120
SRR17520069.17692121
SRR17520069.17692122
SRR17520069.17692123
SRR17520069.17692126
SRR17520069.17692128
SRR17520069.17692130
SRR17520069.17692131
SRR17520069.17712558
SRR17520069.17712559
SRR17520069.17712560
SRR17520069.17712561
SRR17520069.17712562
SRR17520069.17712563
SRR17520069.17712564
SRR17520069.17712565
SRR17520069.17712566
SRR17520069.17731065
SRR17520069.17731066
SRR17520069.17731067
SRR17520069.17736206
SRR17520069.17736207
SRR17520069.17736208
SRR17520069.17736209
SRR17520069.17736210

SRR17520069.17736211
SRR17520069.17736212
SRR17520069.17736213
SRR17520069.17736214
SRR17520069.17736215
SRR17520069.17736216
SRR17520069.17750497
SRR17520069.17750498
SRR17520069.17750499
SRR17520069.17750500
SRR17520069.17750501
SRR17520069.17750502
SRR17520069.17750503
SRR17520069.17750504
SRR17520069.17750505
SRR17520069.17750506
SRR17520069.17750507
SRR17520069.17750508
SRR17520069.17750509
SRR17520069.17750510
SRR17520069.17750511
SRR17520069.17750512
SRR17520069.17750513
SRR17520069.17750514
SRR17520069.17750515
SRR17520069.17750516
SRR17520069.17750517
SRR17520069.17750518
SRR17520069.17750519
SRR17520069.17750520
SRR17520069.17750521
SRR17520069.17750522
SRR17520069.17750523
SRR17520069.17750526
SRR17520069.17750527
SRR17520069.17750528
SRR17520069.17750529
SRR17520069.17750531
SRR17520069.17750532
SRR17520069.17750533
SRR17520069.17750534
SRR17520069.17760786
SRR17520069.17768183
SRR17520069.17774212
SRR17520069.17790539
SRR17520069.17790541
SRR17520069.17795588

SRR17520069.17841244
SRR17520069.17850958
SRR17520069.17852456
SRR17520069.17856756
SRR17520069.17856757
SRR17520069.17856758
SRR17520069.17856759
SRR17520069.17856760
SRR17520069.17856761
SRR17520069.17856762
SRR17520069.17856763
SRR17520069.17856765
SRR17520069.17856766
SRR17520069.17856767
SRR17520069.17856768
SRR17520069.17856769
SRR17520069.17856770
SRR17520069.17856771
SRR17520069.17856772
SRR17520069.17856773
SRR17520069.17856774
SRR17520069.17856775
SRR17520069.17856776
SRR17520069.17856777
SRR17520069.17856778
SRR17520069.17856779
SRR17520069.17856780
SRR17520069.17856781
SRR17520069.17856782
SRR17520069.17856783
SRR17520069.17856784
SRR17520069.17856785
SRR17520069.17856786
SRR17520069.17856787
SRR17520069.17856788
SRR17520069.17856789
SRR17520069.17856790
SRR17520069.17856791
SRR17520069.17856792
SRR17520069.17856793
SRR17520069.17856794
SRR17520069.17856795
SRR17520069.17856796
SRR17520069.17856797
SRR17520069.17856798
SRR17520069.17856799
SRR17520069.17856800

SRR17520069.17856801
SRR17520069.17856802
SRR17520069.17856803
SRR17520069.17856804
SRR17520069.17856805
SRR17520069.17856806
SRR17520069.17856807
SRR17520069.17856808
SRR17520069.17856809
SRR17520069.17856810
SRR17520069.17856811
SRR17520069.17856812
SRR17520069.17856813
SRR17520069.17856814
SRR17520069.17856815
SRR17520069.17856816
SRR17520069.17856817
SRR17520069.17856818
SRR17520069.17856819
SRR17520069.17856820
SRR17520069.17856821
SRR17520069.17856822
SRR17520069.17856823
SRR17520069.17856825
SRR17520069.17856826
SRR17520069.17856827
SRR17520069.17856828
SRR17520069.17856829
SRR17520069.17856830
SRR17520069.17856831
SRR17520069.17856832
SRR17520069.17856833
SRR17520069.17856834
SRR17520069.17856835
SRR17520069.17856836
SRR17520069.17856837
SRR17520069.17856838
SRR17520069.17856839
SRR17520069.17856840
SRR17520069.17856841
SRR17520069.17856842
SRR17520069.17856843
SRR17520069.17856844
SRR17520069.17856845
SRR17520069.17856846
SRR17520069.17856847
SRR17520069.17856848

SRR17520069.17856849
SRR17520069.17856850
SRR17520069.17856851
SRR17520069.17856852
SRR17520069.17856853
SRR17520069.17856854
SRR17520069.17856855
SRR17520069.17856856
SRR17520069.17856857
SRR17520069.17856858
SRR17520069.17856860
SRR17520069.17856861
SRR17520069.17856862
SRR17520069.17856863
SRR17520069.17856864
SRR17520069.17906453
SRR17520069.17913691
SRR17520069.17931634
SRR17520069.17931635
SRR17520069.17935406
SRR17520069.17935407
SRR17520069.17935408
SRR17520069.17935409
SRR17520069.17935410
SRR17520069.17935411
SRR17520069.17935412
SRR17520069.17935413
SRR17520069.17935414
SRR17520069.17935415
SRR17520069.17935416
SRR17520069.17935417
SRR17520069.17935418
SRR17520069.17935419
SRR17520069.17935420
SRR17520069.17935422
SRR17520069.17935423
SRR17520069.17935424
SRR17520069.17935425
SRR17520069.17935426
SRR17520069.17935427
SRR17520069.17935429
SRR17520069.17935430
SRR17520069.17935431
SRR17520069.17935432
SRR17520069.17935433
SRR17520069.17935434
SRR17520069.17935435

SRR17520069.17935436
SRR17520069.17935437
SRR17520069.17935438
SRR17520069.17935439
SRR17520069.17935440
SRR17520069.17935441
SRR17520069.17935442
SRR17520069.17935443
SRR17520069.17935444
SRR17520069.17935445
SRR17520069.17935446
SRR17520069.17935447
SRR17520069.17935448
SRR17520069.17935449
SRR17520069.17935450
SRR17520069.17935451
SRR17520069.17935452
SRR17520069.17935453
SRR17520069.17935454
SRR17520069.17935455
SRR17520069.17935456
SRR17520069.17935457
SRR17520069.17935458
SRR17520069.17935459
SRR17520069.17935460
SRR17520069.17935461
SRR17520069.17935462
SRR17520069.17935463
SRR17520069.17935464
SRR17520069.17935465
SRR17520069.17935466
SRR17520069.17935467
SRR17520069.17935468
SRR17520069.17935469
SRR17520069.17935470
SRR17520069.17935471
SRR17520069.17935472
SRR17520069.17935473
SRR17520069.17935474
SRR17520069.17935475
SRR17520069.17935476
SRR17520069.17935477
SRR17520069.17935478
SRR17520069.17935479
SRR17520069.17935480
SRR17520069.17935481
SRR17520069.17935482

SRR17520069.17935483
SRR17520069.17935484
SRR17520069.17935485
SRR17520069.17935486
SRR17520069.17935487
SRR17520069.17935488
SRR17520069.17935489
SRR17520069.17935490
SRR17520069.17935491
SRR17520069.17935492
SRR17520069.17935493
SRR17520069.17935494
SRR17520069.17935495
SRR17520069.17935496
SRR17520069.17935497
SRR17520069.17935498
SRR17520069.17935499
SRR17520069.17935500
SRR17520069.17935501
SRR17520069.17935504
SRR17520069.17935505
SRR17520069.17935506
SRR17520069.17935507
SRR17520069.17935508
SRR17520069.17935509
SRR17520069.17935510
SRR17520069.17935511
SRR17520069.17935512
SRR17520069.17935513
SRR17520069.17935514
SRR17520069.17935515
SRR17520069.17935516
SRR17520069.17935517
SRR17520069.17935518
SRR17520069.17935519
SRR17520069.17935520
SRR17520069.17935521
SRR17520069.17935522
SRR17520069.17935523
SRR17520069.17935524
SRR17520069.17935525
SRR17520069.17935526
SRR17520069.17935527
SRR17520069.17943005
SRR17520069.17949091
SRR17520069.17949101
SRR17520069.17949102

SRR17520069.17949103
SRR17520069.17949104
SRR17520069.17949105
SRR17520069.17949107
SRR17520069.17949109
SRR17520069.17949110
SRR17520069.17949113
SRR17520069.17949114
SRR17520069.17949115
SRR17520069.17949116
SRR17520069.17949117
SRR17520069.17949118
SRR17520069.17949119
SRR17520069.17949120
SRR17520069.17949121
SRR17520069.17949122
SRR17520069.17949123
SRR17520069.17949124
SRR17520069.17949125
SRR17520069.17949126
SRR17520069.17949127
SRR17520069.17949128
SRR17520069.17949129
SRR17520069.17949130
SRR17520069.17949131
SRR17520069.17949132
SRR17520069.17949133
SRR17520069.17949134
SRR17520069.17949135
SRR17520069.17949136
SRR17520069.17949137
SRR17520069.17949138
SRR17520069.17949139
SRR17520069.17949140
SRR17520069.17949141
SRR17520069.17949142
SRR17520069.17949143
SRR17520069.17949144
SRR17520069.17949145
SRR17520069.17949146
SRR17520069.17949147
SRR17520069.17949148
SRR17520069.17949149
SRR17520069.17949150
SRR17520069.17949151
SRR17520069.17949152
SRR17520069.17949153

SRR17520069.17949155
SRR17520069.17949156
SRR17520069.17949157
SRR17520069.17949158
SRR17520069.17949159
SRR17520069.17949160
SRR17520069.17949161
SRR17520069.17949162
SRR17520069.17949163
SRR17520069.17949164
SRR17520069.17949165
SRR17520069.17949166
SRR17520069.17949167
SRR17520069.17949168
SRR17520069.17949169
SRR17520069.17949170
SRR17520069.17949171
SRR17520069.17949172
SRR17520069.17949173
SRR17520069.17949174
SRR17520069.17949175
SRR17520069.17949176
SRR17520069.17949177
SRR17520069.17949178
SRR17520069.17949179
SRR17520069.17949180
SRR17520069.17949181
SRR17520069.17949182
SRR17520069.17949183
SRR17520069.17949184
SRR17520069.17949185
SRR17520069.17949186
SRR17520069.17949187
SRR17520069.17949188
SRR17520069.17949189
SRR17520069.17949190
SRR17520069.17949191
SRR17520069.17949192
SRR17520069.17949193
SRR17520069.17949194
SRR17520069.17949195
SRR17520069.17949196
SRR17520069.17949197
SRR17520069.17949198
SRR17520069.17949199
SRR17520069.17949201
SRR17520069.17949202

SRR17520069.17949203
SRR17520069.17949204
SRR17520069.17949205
SRR17520069.17949206
SRR17520069.17949207
SRR17520069.17949209
SRR17520069.17949210
SRR17520069.17949211
SRR17520069.17949212
SRR17520069.17949213
SRR17520069.17949214
SRR17520069.17949215
SRR17520069.17949216
SRR17520069.17949217
SRR17520069.17949218
SRR17520069.17949219
SRR17520069.17949220
SRR17520069.17949221
SRR17520069.17949222
SRR17520069.17949223
SRR17520069.17949224
SRR17520069.17949225
SRR17520069.17949226
SRR17520069.17949227
SRR17520069.17949228
SRR17520069.17949229
SRR17520069.17949230
SRR17520069.17949231
SRR17520069.17949232
SRR17520069.17949233
SRR17520069.17949234
SRR17520069.17949235
SRR17520069.17949236
SRR17520069.17949237
SRR17520069.17949238
SRR17520069.17949239
SRR17520069.17949240
SRR17520069.17949241
SRR17520069.17949242
SRR17520069.17949243
SRR17520069.17952331
SRR17520069.17965548
SRR17520069.17965549
SRR17520069.17965550
SRR17520069.17965551
SRR17520069.17965552
SRR17520069.17965553

SRR17520069.17965555
SRR17520069.17965557
SRR17520069.17965558
SRR17520069.17965559
SRR17520069.17965560
SRR17520069.17965561
SRR17520069.17965562
SRR17520069.17965563
SRR17520069.17965564
SRR17520069.17965565
SRR17520069.17965566
SRR17520069.17965567
SRR17520069.17965568
SRR17520069.17965569
SRR17520069.17965570
SRR17520069.17965571
SRR17520069.17965572
SRR17520069.17965573
SRR17520069.17965574
SRR17520069.17965575
SRR17520069.17965576
SRR17520069.17965577
SRR17520069.17965578
SRR17520069.17965579
SRR17520069.17965580
SRR17520069.17965581
SRR17520069.17965582
SRR17520069.17965583
SRR17520069.17965584
SRR17520069.17965585
SRR17520069.17965586
SRR17520069.17965587
SRR17520069.17965588
SRR17520069.17965589
SRR17520069.17965590
SRR17520069.17965591
SRR17520069.17965592
SRR17520069.17965593
SRR17520069.17965594
SRR17520069.17965595
SRR17520069.17965596
SRR17520069.17965597
SRR17520069.17965598
SRR17520069.17965599
SRR17520069.17965600
SRR17520069.17965601
SRR17520069.17965602

SRR17520069.17965603
SRR17520069.17965604
SRR17520069.17965605
SRR17520069.17965606
SRR17520069.17965607
SRR17520069.17965608
SRR17520069.17966081
SRR17520069.17966082
SRR17520069.17966083
SRR17520069.17966084
SRR17520069.17966085
SRR17520069.17966086
SRR17520069.17966087
SRR17520069.17966088
SRR17520069.17966089
SRR17520069.17966090
SRR17520069.17966091
SRR17520069.17966092
SRR17520069.17966093
SRR17520069.17966094
SRR17520069.17966095
SRR17520069.17966096
SRR17520069.17966097
SRR17520069.17966098
SRR17520069.17966099
SRR17520069.17966100
SRR17520069.17966101
SRR17520069.17966102
SRR17520069.17966103
SRR17520069.17966105
SRR17520069.17966106
SRR17520069.17966107
SRR17520069.17966108
SRR17520069.17966109
SRR17520069.17966110
SRR17520069.17966111
SRR17520069.17966112
SRR17520069.17966113
SRR17520069.17966114
SRR17520069.17966115
SRR17520069.17966116
SRR17520069.17966117
SRR17520069.17966118
SRR17520069.17966119
SRR17520069.17966120
SRR17520069.17966121
SRR17520069.17966122

SRR17520069.17966123
SRR17520069.17966124
SRR17520069.17966125
SRR17520069.17966126
SRR17520069.17966127
SRR17520069.17966128
SRR17520069.17966129
SRR17520069.17966130
SRR17520069.17966131
SRR17520069.17966133
SRR17520069.17966134
SRR17520069.17966135
SRR17520069.17966136
SRR17520069.17966137
SRR17520069.17972148
SRR17520069.17972149
SRR17520069.17972150
SRR17520069.17972151
SRR17520069.17972152
SRR17520069.17972153
SRR17520069.17972154
SRR17520069.17972155
SRR17520069.17972156
SRR17520069.17972157
SRR17520069.17972158
SRR17520069.17972159
SRR17520069.17972160
SRR17520069.17972161
SRR17520069.17972162
SRR17520069.17972163
SRR17520069.17972164
SRR17520069.17972165
SRR17520069.17972166
SRR17520069.17972167
SRR17520069.17972168
SRR17520069.17972169
SRR17520069.17989811
SRR17520069.18001506
SRR17520069.18001508
SRR17520069.18001509
SRR17520069.18001510
SRR17520069.18001511
SRR17520069.18001512
SRR17520069.18001514
SRR17520069.18001517
SRR17520069.18001518
SRR17520069.18001520

SRR17520069.18001522
SRR17520069.18001523
SRR17520069.18001524
SRR17520069.18001525
SRR17520069.18001526
SRR17520069.18001527
SRR17520069.18001532
SRR17520069.18003141
SRR17520069.18003142
SRR17520069.18004833
SRR17520069.18013330
SRR17520069.18013331
SRR17520069.18013332
SRR17520069.18013333
SRR17520069.18013335
SRR17520069.18013336
SRR17520069.18013337
SRR17520069.18013338
SRR17520069.18013339
SRR17520069.18013340
SRR17520069.18013341
SRR17520069.18013342
SRR17520069.18013343
SRR17520069.18013344
SRR17520069.18013345
SRR17520069.18013346
SRR17520069.18013347
SRR17520069.18013348
SRR17520069.18013352
SRR17520069.18013353
SRR17520069.18034636
SRR17520069.18034658
SRR17520069.18045697
SRR17520069.18045698
SRR17520069.18045699
SRR17520069.18045700
SRR17520069.18045701
SRR17520069.18045702
SRR17520069.18045703
SRR17520069.18045704
SRR17520069.18045705
SRR17520069.18045706
SRR17520069.18045707
SRR17520069.18045708
SRR17520069.18045709
SRR17520069.18045710
SRR17520069.18045711

SRR17520069.18045712
SRR17520069.18045713
SRR17520069.18045714
SRR17520069.18045715
SRR17520069.18045717
SRR17520069.18045718
SRR17520069.18045719
SRR17520069.18045720
SRR17520069.18045721
SRR17520069.18045722
SRR17520069.18045723
SRR17520069.18045724
SRR17520069.18045725
SRR17520069.18045726
SRR17520069.18045727
SRR17520069.18045728
SRR17520069.18045729
SRR17520069.18045730
SRR17520069.18045731
SRR17520069.18045732
SRR17520069.18045733
SRR17520069.18045734
SRR17520069.18045735
SRR17520069.18045736
SRR17520069.18045737
SRR17520069.18045738
SRR17520069.18045739
SRR17520069.18045740
SRR17520069.18045741
SRR17520069.18045742
SRR17520069.18045743
SRR17520069.18045744
SRR17520069.18045745
SRR17520069.18045746
SRR17520069.18045747
SRR17520069.18045748
SRR17520069.18045749
SRR17520069.18045750
SRR17520069.18045751
SRR17520069.18045752
SRR17520069.18045753
SRR17520069.18045754
SRR17520069.18045755
SRR17520069.18045756
SRR17520069.18045757
SRR17520069.18045758
SRR17520069.18045759

SRR17520069.18045760
SRR17520069.18045761
SRR17520069.18045762
SRR17520069.18045763
SRR17520069.18045764
SRR17520069.18045765
SRR17520069.18045766
SRR17520069.18045767
SRR17520069.18045768
SRR17520069.18045769
SRR17520069.18045771
SRR17520069.18045772
SRR17520069.18045773
SRR17520069.18045774
SRR17520069.18045775
SRR17520069.18045776
SRR17520069.18045777
SRR17520069.18045778
SRR17520069.18045779
SRR17520069.18045780
SRR17520069.18045781
SRR17520069.18045782
SRR17520069.18045783
SRR17520069.18045784
SRR17520069.18045785
SRR17520069.18045786
SRR17520069.18045787
SRR17520069.18045788
SRR17520069.18045789
SRR17520069.18045790
SRR17520069.18045791
SRR17520069.18045792
SRR17520069.18045793
SRR17520069.18045794
SRR17520069.18045795
SRR17520069.18045796
SRR17520069.18045797
SRR17520069.18045798
SRR17520069.18045799
SRR17520069.18045800
SRR17520069.18045801
SRR17520069.18045802
SRR17520069.18045803
SRR17520069.18045804
SRR17520069.18045805
SRR17520069.18045806
SRR17520069.18045807

SRR17520069.18045808
SRR17520069.18045809
SRR17520069.18045810
SRR17520069.18045811
SRR17520069.18057513
SRR17520069.18057514
SRR17520069.18057515
SRR17520069.18083065
SRR17520069.18083082
SRR17520069.18087123
SRR17520069.18087124
SRR17520069.18087125
SRR17520069.18087126
SRR17520069.18087127
SRR17520069.18087128
SRR17520069.18087129
SRR17520069.18087130
SRR17520069.18087131
SRR17520069.18087132
SRR17520069.18087133
SRR17520069.18087134
SRR17520069.18087135
SRR17520069.18087136
SRR17520069.18087137
SRR17520069.18087138
SRR17520069.18087139
SRR17520069.18087140
SRR17520069.18087141
SRR17520069.18087142
SRR17520069.18087143
SRR17520069.18087144
SRR17520069.18087145
SRR17520069.18087146
SRR17520069.18087147
SRR17520069.18087148
SRR17520069.18087149
SRR17520069.18087150
SRR17520069.18087151
SRR17520069.18087152
SRR17520069.18087153
SRR17520069.18087154
SRR17520069.18087155
SRR17520069.18087156
SRR17520069.18087157
SRR17520069.18087158
SRR17520069.18087159
SRR17520069.18087160

SRR17520069.18087161
SRR17520069.18087162
SRR17520069.18087163
SRR17520069.18087164
SRR17520069.18087165
SRR17520069.18087166
SRR17520069.18087167
SRR17520069.18087168
SRR17520069.18087169
SRR17520069.18087170
SRR17520069.18087171
SRR17520069.18087172
SRR17520069.18087173
SRR17520069.18087174
SRR17520069.18087175
SRR17520069.18087176
SRR17520069.18087177
SRR17520069.18087178
SRR17520069.18087179
SRR17520069.18087181
SRR17520069.18087182
SRR17520069.18087183
SRR17520069.18087185
SRR17520069.18087186
SRR17520069.18087187
SRR17520069.18087188
SRR17520069.18087189
SRR17520069.18087190
SRR17520069.18087191
SRR17520069.18087192
SRR17520069.18087193
SRR17520069.18087194
SRR17520069.18087195
SRR17520069.18087196
SRR17520069.18087197
SRR17520069.18087198
SRR17520069.18087199
SRR17520069.18087200
SRR17520069.18087201
SRR17520069.18087202
SRR17520069.18087203
SRR17520069.18087204
SRR17520069.18087205
SRR17520069.18087206
SRR17520069.18087208
SRR17520069.18087209
SRR17520069.18087210

SRR17520069.18087211
SRR17520069.18087212
SRR17520069.18087213
SRR17520069.18087214
SRR17520069.18087215
SRR17520069.18087216
SRR17520069.18087217
SRR17520069.18087218
SRR17520069.18087219
SRR17520069.18087220
SRR17520069.18087221
SRR17520069.18087222
SRR17520069.18087223
SRR17520069.18087224
SRR17520069.18087225
SRR17520069.18087226
SRR17520069.18087227
SRR17520069.18087228
SRR17520069.18087229
SRR17520069.18087230
SRR17520069.18087231
SRR17520069.18087232
SRR17520069.18087233
SRR17520069.18087234
SRR17520069.18087235
SRR17520069.18087236
SRR17520069.18087237
SRR17520069.18087238
SRR17520069.18087239
SRR17520069.18087240
SRR17520069.18087241
SRR17520069.18087242
SRR17520069.18087243
SRR17520069.18087244
SRR17520069.18087245
SRR17520069.18087246
SRR17520069.18087247
SRR17520069.18087248
SRR17520069.18087249
SRR17520069.18087250
SRR17520069.18087251
SRR17520069.18087252
SRR17520069.18087253
SRR17520069.18087254
SRR17520069.18087255
SRR17520069.18087257
SRR17520069.18087258

SRR17520069.18087259
SRR17520069.18087260
SRR17520069.18087261
SRR17520069.18087262
SRR17520069.18087263
SRR17520069.18087264
SRR17520069.18087265
SRR17520069.18087266
SRR17520069.18087267
SRR17520069.18087268
SRR17520069.18087269
SRR17520069.18087270
SRR17520069.18087271
SRR17520069.18087272
SRR17520069.18087273
SRR17520069.18087274
SRR17520069.18087275
SRR17520069.18087276
SRR17520069.18087277
SRR17520069.18087278
SRR17520069.18087279
SRR17520069.18087280
SRR17520069.18087281
SRR17520069.18087282
SRR17520069.18087283
SRR17520069.18087284
SRR17520069.18087285
SRR17520069.18087286
SRR17520069.18087287
SRR17520069.18087288
SRR17520069.18087289
SRR17520069.18087290
SRR17520069.18087292
SRR17520069.18087293
SRR17520069.18087294
SRR17520069.18087295
SRR17520069.18087296
SRR17520069.18087297
SRR17520069.18087298
SRR17520069.18087299
SRR17520069.18087300
SRR17520069.18087301
SRR17520069.18087302
SRR17520069.18087303
SRR17520069.18087304
SRR17520069.18087305
SRR17520069.18087306

SRR17520069.18087307
SRR17520069.18087308
SRR17520069.18087309
SRR17520069.18087310
SRR17520069.18087311
SRR17520069.18087312
SRR17520069.18087313
SRR17520069.18087314
SRR17520069.18087315
SRR17520069.18087316
SRR17520069.18087317
SRR17520069.18087318
SRR17520069.18087319
SRR17520069.18087320
SRR17520069.18087321
SRR17520069.18087322
SRR17520069.18087323
SRR17520069.18087324
SRR17520069.18087325
SRR17520069.18088061
SRR17520069.18088062
SRR17520069.18088063
SRR17520069.18088064
SRR17520069.18088065
SRR17520069.18088066
SRR17520069.18088067
SRR17520069.18088068
SRR17520069.18088069
SRR17520069.18088070
SRR17520069.18088071
SRR17520069.18088072
SRR17520069.18088073
SRR17520069.18088074
SRR17520069.18088075
SRR17520069.18088076
SRR17520069.18088077
SRR17520069.18088078
SRR17520069.18088079
SRR17520069.18088080
SRR17520069.18088081
SRR17520069.18088082
SRR17520069.18088083
SRR17520069.18088084
SRR17520069.18088085
SRR17520069.18088086
SRR17520069.18088087
SRR17520069.18088088

SRR17520069.18088089
SRR17520069.18088090
SRR17520069.18088091
SRR17520069.18088092
SRR17520069.18088093
SRR17520069.18088094
SRR17520069.18088095
SRR17520069.18088096
SRR17520069.18088097
SRR17520069.18088098
SRR17520069.18088099
SRR17520069.18088100
SRR17520069.18088101
SRR17520069.18088102
SRR17520069.18088104
SRR17520069.18088105
SRR17520069.18088107
SRR17520069.18088108
SRR17520069.18088109
SRR17520069.18088110
SRR17520069.18088111
SRR17520069.18088112
SRR17520069.18088113
SRR17520069.18088114
SRR17520069.18088115
SRR17520069.18088116
SRR17520069.18088117
SRR17520069.18088118
SRR17520069.18088119
SRR17520069.18088120
SRR17520069.18088121
SRR17520069.18088122
SRR17520069.18088123
SRR17520069.18088124
SRR17520069.18088125
SRR17520069.18088126
SRR17520069.18088127
SRR17520069.18088128
SRR17520069.18088129
SRR17520069.18088130
SRR17520069.18088131
SRR17520069.18088132
SRR17520069.18088133
SRR17520069.18088134
SRR17520069.18088135
SRR17520069.18088136
SRR17520069.18088137

SRR17520069.18088138
SRR17520069.18088139
SRR17520069.18088140
SRR17520069.18088145
SRR17520069.18088147
SRR17520069.18088149
SRR17520069.18088152
SRR17520069.18088156
SRR17520069.18088158
SRR17520069.18088159
SRR17520069.18088161
SRR17520069.18088163
SRR17520069.18088165
SRR17520069.18088166
SRR17520069.18088167
SRR17520069.18088168
SRR17520069.18088169
SRR17520069.18088170
SRR17520069.18088179
SRR17520069.18088180
SRR17520069.18102681
SRR17520069.18102682
SRR17520069.18102683
SRR17520069.18102684
SRR17520069.18102685
SRR17520069.18102686
SRR17520069.18102687
SRR17520069.18102688
SRR17520069.18102689
SRR17520069.18102690
SRR17520069.18102691
SRR17520069.18102692
SRR17520069.18102697
SRR17520069.18102700
SRR17520069.18102702
SRR17520069.18102703
SRR17520069.18102704
SRR17520069.18102705
SRR17520069.18102706
SRR17520069.18102709
SRR17520069.18102711
SRR17520069.18116520
SRR17520069.18116521
SRR17520069.18116522
SRR17520069.18116523
SRR17520069.18116524
SRR17520069.18116525

SRR17520069.18116526
SRR17520069.18116527
SRR17520069.18116528
SRR17520069.18116529
SRR17520069.18116530
SRR17520069.18116531
SRR17520069.18116532
SRR17520069.18116533
SRR17520069.18116534
SRR17520069.18116535
SRR17520069.18116536
SRR17520069.18116537
SRR17520069.18116538
SRR17520069.18116539
SRR17520069.18116540
SRR17520069.18116541
SRR17520069.18116542
SRR17520069.18116543
SRR17520069.18116544
SRR17520069.18116545
SRR17520069.18116546
SRR17520069.18116547
SRR17520069.18116548
SRR17520069.18116549
SRR17520069.18116550
SRR17520069.18116551
SRR17520069.18116552
SRR17520069.18116553
SRR17520069.18116554
SRR17520069.18116555
SRR17520069.18116556
SRR17520069.18116557
SRR17520069.18116558
SRR17520069.18116559
SRR17520069.18116560
SRR17520069.18116561
SRR17520069.18116562
SRR17520069.18116563
SRR17520069.18116564
SRR17520069.18116565
SRR17520069.18116566
SRR17520069.18116567
SRR17520069.18116568
SRR17520069.18116569
SRR17520069.18116570
SRR17520069.18116571
SRR17520069.18116572

SRR17520069.18116573
SRR17520069.18116574
SRR17520069.18116575
SRR17520069.18116576
SRR17520069.18120214
SRR17520069.18122986
SRR17520069.18122989
SRR17520069.18122990
SRR17520069.18122991
SRR17520069.18122992
SRR17520069.18122993
SRR17520069.18122994
SRR17520069.18152772
SRR17520069.18156285
SRR17520069.18156286
SRR17520069.18156287
SRR17520069.18156288
SRR17520069.18156289
SRR17520069.18156290
SRR17520069.18156291
SRR17520069.18156292
SRR17520069.18156293
SRR17520069.18156294
SRR17520069.18156295
SRR17520069.18156296
SRR17520069.18156297
SRR17520069.18156298
SRR17520069.18156301
SRR17520069.18156303
SRR17520069.18156304
SRR17520069.18156305
SRR17520069.18156306
SRR17520069.18156307
SRR17520069.18156308
SRR17520069.18156309
SRR17520069.18156310
SRR17520069.18156311
SRR17520069.18156312
SRR17520069.18156313
SRR17520069.18156314
SRR17520069.18156315
SRR17520069.18156316
SRR17520069.18156317
SRR17520069.18156318
SRR17520069.18156319
SRR17520069.18156320
SRR17520069.18156321

SRR17520069.18156322
SRR17520069.18156323
SRR17520069.18156324
SRR17520069.18156326
SRR17520069.18156327
SRR17520069.18156328
SRR17520069.18156330
SRR17520069.18156332
SRR17520069.18156333
SRR17520069.18156334
SRR17520069.18156335
SRR17520069.18156336
SRR17520069.18156337
SRR17520069.18156338
SRR17520069.18156339
SRR17520069.18156340
SRR17520069.18156341
SRR17520069.18156342
SRR17520069.18156343
SRR17520069.18156344
SRR17520069.18156345
SRR17520069.18156346
SRR17520069.18156347
SRR17520069.18156348
SRR17520069.18156349
SRR17520069.18156350
SRR17520069.18156351
SRR17520069.18156352
SRR17520069.18156353
SRR17520069.18156354
SRR17520069.18156355
SRR17520069.18156356
SRR17520069.18156357
SRR17520069.18156358
SRR17520069.18156359
SRR17520069.18156360
SRR17520069.18156361
SRR17520069.18156362
SRR17520069.18156363
SRR17520069.18156364
SRR17520069.18156365
SRR17520069.18156366
SRR17520069.18156367
SRR17520069.18156368
SRR17520069.18156369
SRR17520069.18156370
SRR17520069.18156371

SRR17520069.18156372
SRR17520069.18156373
SRR17520069.18156374
SRR17520069.18156375
SRR17520069.18156376
SRR17520069.18156377
SRR17520069.18156378
SRR17520069.18156379
SRR17520069.18156380
SRR17520069.18156381
SRR17520069.18156382
SRR17520069.18156383
SRR17520069.18156384
SRR17520069.18156385
SRR17520069.18156386
SRR17520069.18156387
SRR17520069.18156388
SRR17520069.18156389
SRR17520069.18156390
SRR17520069.18156391
SRR17520069.18156392
SRR17520069.18156393
SRR17520069.18156394
SRR17520069.18156395
SRR17520069.18156396
SRR17520069.18156397
SRR17520069.18156398
SRR17520069.18156399
SRR17520069.18156400
SRR17520069.18156401
SRR17520069.18156402
SRR17520069.18156403
SRR17520069.18156404
SRR17520069.18156405
SRR17520069.18156406
SRR17520069.18156407
SRR17520069.18156408
SRR17520069.18166313
SRR17520069.18168956
SRR17520069.18168957
SRR17520069.18168958
SRR17520069.18168959
SRR17520069.18168960
SRR17520069.18168961
SRR17520069.18168962
SRR17520069.18168963
SRR17520069.18168964

SRR17520069.18168965
SRR17520069.18168966
SRR17520069.18168967
SRR17520069.18168968
SRR17520069.18168969
SRR17520069.18168970
SRR17520069.18168971
SRR17520069.18168972
SRR17520069.18168973
SRR17520069.18168974
SRR17520069.18168975
SRR17520069.18168976
SRR17520069.18168977
SRR17520069.18168978
SRR17520069.18168979
SRR17520069.18168980
SRR17520069.18168981
SRR17520069.18168982
SRR17520069.18168983
SRR17520069.18168984
SRR17520069.18168985
SRR17520069.18168986
SRR17520069.18168987
SRR17520069.18168988
SRR17520069.18168989
SRR17520069.18168990
SRR17520069.18168991
SRR17520069.18168992
SRR17520069.18168993
SRR17520069.18168994
SRR17520069.18168995
SRR17520069.18168996
SRR17520069.18168997
SRR17520069.18168998
SRR17520069.18168999
SRR17520069.18169000
SRR17520069.18169001
SRR17520069.18169002
SRR17520069.18169003
SRR17520069.18169004
SRR17520069.18169005
SRR17520069.18169006
SRR17520069.18169007
SRR17520069.18169008
SRR17520069.18169009
SRR17520069.18169011
SRR17520069.18169012

SRR17520069.18169013
SRR17520069.18169014
SRR17520069.18169015
SRR17520069.18169016
SRR17520069.18169017
SRR17520069.18169018
SRR17520069.18169019
SRR17520069.18169020
SRR17520069.18169021
SRR17520069.18169022
SRR17520069.18169023
SRR17520069.18169024
SRR17520069.18169025
SRR17520069.18169026
SRR17520069.18169027
SRR17520069.18169028
SRR17520069.18169029
SRR17520069.18169030
SRR17520069.18169031
SRR17520069.18169032
SRR17520069.18169033
SRR17520069.18169034
SRR17520069.18169035
SRR17520069.18169036
SRR17520069.18169037
SRR17520069.18169038
SRR17520069.18169039
SRR17520069.18169040
SRR17520069.18169041
SRR17520069.18169042
SRR17520069.18169043
SRR17520069.18169044
SRR17520069.18169045
SRR17520069.18169046
SRR17520069.18169047
SRR17520069.18169048
SRR17520069.18169049
SRR17520069.18169050
SRR17520069.18169051
SRR17520069.18169052
SRR17520069.18169053
SRR17520069.18169054
SRR17520069.18169055
SRR17520069.18169056
SRR17520069.18169057
SRR17520069.18169058
SRR17520069.18169059

SRR17520069.18169060
SRR17520069.18169061
SRR17520069.18169062
SRR17520069.18169063
SRR17520069.18169064
SRR17520069.18169065
SRR17520069.18169066
SRR17520069.18169067
SRR17520069.18169068
SRR17520069.18169069
SRR17520069.18169070
SRR17520069.18169071
SRR17520069.18169072
SRR17520069.18169073
SRR17520069.18169074
SRR17520069.18169075
SRR17520069.18169076
SRR17520069.18169077
SRR17520069.18169078
SRR17520069.18169079
SRR17520069.18169080
SRR17520069.18169081
SRR17520069.18169090
SRR17520069.18169092
SRR17520069.18169093
SRR17520069.18169096
SRR17520069.18169105
SRR17520069.18169107
SRR17520069.18169108
SRR17520069.18170044
SRR17520069.18170045
SRR17520069.18170046
SRR17520069.18170047
SRR17520069.18183396
SRR17520069.18183397
SRR17520069.18183398
SRR17520069.18183399
SRR17520069.18183400
SRR17520069.18183401
SRR17520069.18183402
SRR17520069.18183403
SRR17520069.18183404
SRR17520069.18183405
SRR17520069.18183406
SRR17520069.18183407
SRR17520069.18183408
SRR17520069.18183409

SRR17520069.18183410
SRR17520069.18183411
SRR17520069.18183412
SRR17520069.18183413
SRR17520069.18183414
SRR17520069.18183415
SRR17520069.18183416
SRR17520069.18183417
SRR17520069.18183418
SRR17520069.18183419
SRR17520069.18183420
SRR17520069.18183421
SRR17520069.18183422
SRR17520069.18183423
SRR17520069.18183424
SRR17520069.18183425
SRR17520069.18183426
SRR17520069.18183427
SRR17520069.18183428
SRR17520069.18183429
SRR17520069.18183430
SRR17520069.18183431
SRR17520069.18183432
SRR17520069.18183433
SRR17520069.18183434
SRR17520069.18183435
SRR17520069.18183436
SRR17520069.18183437
SRR17520069.18183438
SRR17520069.18183439
SRR17520069.18183440
SRR17520069.18183441
SRR17520069.18183442
SRR17520069.18183443
SRR17520069.18183444
SRR17520069.18183445
SRR17520069.18183446
SRR17520069.18183447
SRR17520069.18183448
SRR17520069.18183449
SRR17520069.18183450
SRR17520069.18183451
SRR17520069.18183452
SRR17520069.18183453
SRR17520069.18183454
SRR17520069.18183455
SRR17520069.18183456

SRR17520069.18183457
SRR17520069.18183458
SRR17520069.18183459
SRR17520069.18183460
SRR17520069.18183461
SRR17520069.18183462
SRR17520069.18183463
SRR17520069.18183464
SRR17520069.18183465
SRR17520069.18183466
SRR17520069.18183467
SRR17520069.18183468
SRR17520069.18183469
SRR17520069.18183470
SRR17520069.18183472
SRR17520069.18183473
SRR17520069.18183474
SRR17520069.18183475
SRR17520069.18183476
SRR17520069.18183477
SRR17520069.18183478
SRR17520069.18183479
SRR17520069.18183480
SRR17520069.18183481
SRR17520069.18183482
SRR17520069.18183483
SRR17520069.18183484
SRR17520069.18183485
SRR17520069.18183486
SRR17520069.18183487
SRR17520069.18183488
SRR17520069.18183489
SRR17520069.18183490
SRR17520069.18183491
SRR17520069.18183492
SRR17520069.18183493
SRR17520069.18183496
SRR17520069.18183497
SRR17520069.18183498
SRR17520069.18183499
SRR17520069.18183500
SRR17520069.18183501
SRR17520069.18183502
SRR17520069.18183503
SRR17520069.18183504
SRR17520069.18183505
SRR17520069.18183506

SRR17520069.18183507
SRR17520069.18183508
SRR17520069.18183509
SRR17520069.18183510
SRR17520069.18183511
SRR17520069.18183512
SRR17520069.18183513
SRR17520069.18183514
SRR17520069.18183515
SRR17520069.18183516
SRR17520069.18183517
SRR17520069.18199250
SRR17520069.18199309
SRR17520069.18211323
SRR17520069.18211324
SRR17520069.18211326
SRR17520069.18211327
SRR17520069.18211328
SRR17520069.18211329
SRR17520069.18211330
SRR17520069.18211331
SRR17520069.18211332
SRR17520069.18211333
SRR17520069.18211335
SRR17520069.18214476
SRR17520069.18214477
SRR17520069.18214478
SRR17520069.18214479
SRR17520069.18214480
SRR17520069.18214481
SRR17520069.18214482
SRR17520069.18214484
SRR17520069.18214485
SRR17520069.18214486
SRR17520069.18214487
SRR17520069.18214488
SRR17520069.18214489
SRR17520069.18214490
SRR17520069.18214491
SRR17520069.18214492
SRR17520069.18214493
SRR17520069.18214494
SRR17520069.18214495
SRR17520069.18214496
SRR17520069.18214497
SRR17520069.18214498
SRR17520069.18214499

SRR17520069.18214500
SRR17520069.18214501
SRR17520069.18214502
SRR17520069.18214503
SRR17520069.18214504
SRR17520069.18214505
SRR17520069.18214506
SRR17520069.18214507
SRR17520069.18214508
SRR17520069.18214509
SRR17520069.18214510
SRR17520069.18214511
SRR17520069.18214512
SRR17520069.18214513
SRR17520069.18214514
SRR17520069.18214515
SRR17520069.18219362
SRR17520069.18219363
SRR17520069.18219364
SRR17520069.18219365
SRR17520069.18219366
SRR17520069.18219367
SRR17520069.18219368
SRR17520069.18219369
SRR17520069.18219370
SRR17520069.18219372
SRR17520069.18219373
SRR17520069.18219374
SRR17520069.18219375
SRR17520069.18219376
SRR17520069.18219377
SRR17520069.18219378
SRR17520069.18219379
SRR17520069.18219380
SRR17520069.18219382
SRR17520069.18219383
SRR17520069.18219384
SRR17520069.18219385
SRR17520069.18219386
SRR17520069.18219387
SRR17520069.18219388
SRR17520069.18219389
SRR17520069.18219390
SRR17520069.18219391
SRR17520069.18219392
SRR17520069.18219393
SRR17520069.18219394

SRR17520069.18219395
SRR17520069.18219397
SRR17520069.18219398
SRR17520069.18219399
SRR17520069.18232391
SRR17520069.18232452
SRR17520069.18236710
SRR17520069.18236711
SRR17520069.18236712
SRR17520069.18236713
SRR17520069.18236714
SRR17520069.18236715
SRR17520069.18236716
SRR17520069.18236717
SRR17520069.18236718
SRR17520069.18236719
SRR17520069.18236720
SRR17520069.18236721
SRR17520069.18236722
SRR17520069.18236723
SRR17520069.18236724
SRR17520069.18236725
SRR17520069.18236726
SRR17520069.18236727
SRR17520069.18236728
SRR17520069.18236729
SRR17520069.18236730
SRR17520069.18236731
SRR17520069.18236732
SRR17520069.18236733
SRR17520069.18236734
SRR17520069.18236735
SRR17520069.18236736
SRR17520069.18236737
SRR17520069.18236738
SRR17520069.18236739
SRR17520069.18236740
SRR17520069.18236741
SRR17520069.18236742
SRR17520069.18236743
SRR17520069.18236744
SRR17520069.18236745
SRR17520069.18236746
SRR17520069.18236747
SRR17520069.18236748
SRR17520069.18236749
SRR17520069.18236750

SRR17520069.18236751
SRR17520069.18236752
SRR17520069.18236753
SRR17520069.18236754
SRR17520069.18236755
SRR17520069.18236756
SRR17520069.18236757
SRR17520069.18236758
SRR17520069.18236759
SRR17520069.18236760
SRR17520069.18236761
SRR17520069.18236762
SRR17520069.18236763
SRR17520069.18236764
SRR17520069.18236765
SRR17520069.18236766
SRR17520069.18236767
SRR17520069.18236768
SRR17520069.18236769
SRR17520069.18236770
SRR17520069.18236774
SRR17520069.18236775
SRR17520069.18236776
SRR17520069.18236809
SRR17520069.18250866
SRR17520069.18250882
SRR17520069.18250895
SRR17520069.18250898
SRR17520069.18250906
SRR17520069.18258371
SRR17520069.18258373
SRR17520069.18258374
SRR17520069.18258375
SRR17520069.18258376
SRR17520069.18258377
SRR17520069.18258378
SRR17520069.18258379
SRR17520069.18258380
SRR17520069.18258381
SRR17520069.18258382
SRR17520069.18258383
SRR17520069.18258384
SRR17520069.18258385
SRR17520069.18258386
SRR17520069.18258387
SRR17520069.18258388
SRR17520069.18258389

SRR17520069.18258390
SRR17520069.18258391
SRR17520069.18258392
SRR17520069.18258393
SRR17520069.18258394
SRR17520069.18258396
SRR17520069.18258397
SRR17520069.18258398
SRR17520069.18258399
SRR17520069.18258400
SRR17520069.18258401
SRR17520069.18258402
SRR17520069.18258403
SRR17520069.18258404
SRR17520069.18258405
SRR17520069.18258406
SRR17520069.18258407
SRR17520069.18258408
SRR17520069.18258409
SRR17520069.18258410
SRR17520069.18258411
SRR17520069.18258412
SRR17520069.18277579
SRR17520069.18277580
SRR17520069.18277581
SRR17520069.18281254
SRR17520069.18283449
SRR17520069.18283450
SRR17520069.18283451
SRR17520069.18283452
SRR17520069.18283453
SRR17520069.18283454
SRR17520069.18283455
SRR17520069.18283456
SRR17520069.18283457
SRR17520069.18283458
SRR17520069.18283459
SRR17520069.18283460
SRR17520069.18283461
SRR17520069.18283462
SRR17520069.18283463
SRR17520069.18283464
SRR17520069.18283465
SRR17520069.18283466
SRR17520069.18283468
SRR17520069.18283469
SRR17520069.18283470

SRR17520069.18283471
SRR17520069.18283472
SRR17520069.18283473
SRR17520069.18283474
SRR17520069.18283475
SRR17520069.18283477
SRR17520069.18283479
SRR17520069.18283480
SRR17520069.18283481
SRR17520069.18283482
SRR17520069.18283483
SRR17520069.18283486
SRR17520069.18283487
SRR17520069.18283488
SRR17520069.18283489
SRR17520069.18283490
SRR17520069.18283491
SRR17520069.18283492
SRR17520069.18283493
SRR17520069.18283496
SRR17520069.18283497
SRR17520069.18283498
SRR17520069.18283499
SRR17520069.18283500
SRR17520069.18283501
SRR17520069.18283502
SRR17520069.18283503
SRR17520069.18284164
SRR17520069.18293806
SRR17520069.18293807
SRR17520069.18293808
SRR17520069.18293809
SRR17520069.18293810
SRR17520069.18293811
SRR17520069.18293812
SRR17520069.18293813
SRR17520069.18293814
SRR17520069.18293815
SRR17520069.18293816
SRR17520069.18293817
SRR17520069.18293818
SRR17520069.18293819
SRR17520069.18293820
SRR17520069.18293822
SRR17520069.18293823
SRR17520069.18293824
SRR17520069.18293825

SRR17520069.18293826
SRR17520069.18293827
SRR17520069.18293828
SRR17520069.18293829
SRR17520069.18293830
SRR17520069.18293831
SRR17520069.18293832
SRR17520069.18293833
SRR17520069.18293834
SRR17520069.18293835
SRR17520069.18293836
SRR17520069.18293837
SRR17520069.18293838
SRR17520069.18293839
SRR17520069.18293840
SRR17520069.18293841
SRR17520069.18293842
SRR17520069.18293844
SRR17520069.18293845
SRR17520069.18293846
SRR17520069.18293847
SRR17520069.18293848
SRR17520069.18293849
SRR17520069.18293850
SRR17520069.18293851
SRR17520069.18293852
SRR17520069.18293853
SRR17520069.18293854
SRR17520069.18293855
SRR17520069.18293856
SRR17520069.18293857
SRR17520069.18293858
SRR17520069.18293859
SRR17520069.18293860
SRR17520069.18293861
SRR17520069.18293862
SRR17520069.18293863
SRR17520069.18293864
SRR17520069.18293865
SRR17520069.18293866
SRR17520069.18293867
SRR17520069.18293868
SRR17520069.18293869
SRR17520069.18293870
SRR17520069.18293871
SRR17520069.18293872
SRR17520069.18293873

SRR17520069.18293874
SRR17520069.18293875
SRR17520069.18293876
SRR17520069.18293877
SRR17520069.18293878
SRR17520069.18293879
SRR17520069.18293880
SRR17520069.18293881
SRR17520069.18293882
SRR17520069.18293883
SRR17520069.18293884
SRR17520069.18293885
SRR17520069.18293886
SRR17520069.18293887
SRR17520069.18293888
SRR17520069.18293889
SRR17520069.18293890
SRR17520069.18293891
SRR17520069.18293892
SRR17520069.18293893
SRR17520069.18293894
SRR17520069.18293895
SRR17520069.18293896
SRR17520069.18293897
SRR17520069.18293898
SRR17520069.18293899
SRR17520069.18293900
SRR17520069.18293901
SRR17520069.18293903
SRR17520069.18293904
SRR17520069.18293905
SRR17520069.18293906
SRR17520069.18293907
SRR17520069.18293908
SRR17520069.18293909
SRR17520069.18293910
SRR17520069.18293911
SRR17520069.18293912
SRR17520069.18293913
SRR17520069.18293914
SRR17520069.18293915
SRR17520069.18293916
SRR17520069.18293917
SRR17520069.18293919
SRR17520069.18293920
SRR17520069.18293921
SRR17520069.18293922

SRR17520069.18293923
SRR17520069.18293924
SRR17520069.18293925
SRR17520069.18293926
SRR17520069.18293927
SRR17520069.18293928
SRR17520069.18293929
SRR17520069.18293930
SRR17520069.18293931
SRR17520069.18293932
SRR17520069.18293933
SRR17520069.18293934
SRR17520069.18293935
SRR17520069.18293936
SRR17520069.18293937
SRR17520069.18293938
SRR17520069.18293939
SRR17520069.18293940
SRR17520069.18293941
SRR17520069.18293942
SRR17520069.18293943
SRR17520069.18293944
SRR17520069.18293945
SRR17520069.18293946
SRR17520069.18293947
SRR17520069.18293948
SRR17520069.18293949
SRR17520069.18293950
SRR17520069.18293951
SRR17520069.18293952
SRR17520069.18293953
SRR17520069.18293954
SRR17520069.18293955
SRR17520069.18293956
SRR17520069.18293957
SRR17520069.18293958
SRR17520069.18293959
SRR17520069.18293960
SRR17520069.18293961
SRR17520069.18293962
SRR17520069.18293963
SRR17520069.18293964
SRR17520069.18293965
SRR17520069.18293966
SRR17520069.18293967
SRR17520069.18293968
SRR17520069.18293969

SRR17520069.18293970
SRR17520069.18293971
SRR17520069.18293972
SRR17520069.18293973
SRR17520069.18293975
SRR17520069.18293976
SRR17520069.18293977
SRR17520069.18293978
SRR17520069.18293979
SRR17520069.18293980
SRR17520069.18293981
SRR17520069.18293982
SRR17520069.18293983
SRR17520069.18293984
SRR17520069.18293985
SRR17520069.18293986
SRR17520069.18293988
SRR17520069.18293989
SRR17520069.18293990
SRR17520069.18293991
SRR17520069.18293992
SRR17520069.18293993
SRR17520069.18293994
SRR17520069.18293995
SRR17520069.18293996
SRR17520069.18293997
SRR17520069.18293998
SRR17520069.18293999
SRR17520069.18294000
SRR17520069.18294001
SRR17520069.18294002
SRR17520069.18294003
SRR17520069.18294004
SRR17520069.18294005
SRR17520069.18294006
SRR17520069.18294008
SRR17520069.18294009
SRR17520069.18294010
SRR17520069.18294011
SRR17520069.18294012
SRR17520069.18294013
SRR17520069.18294014
SRR17520069.18294015
SRR17520069.18294016
SRR17520069.18294017
SRR17520069.18294448
SRR17520069.18294452

SRR17520069.18294453
SRR17520069.18301481
SRR17520069.18301494
SRR17520069.18301496
SRR17520069.18301525
SRR17520069.18301561
SRR17520069.18317964
SRR17520069.18317965
SRR17520069.18317966
SRR17520069.18317967
SRR17520069.18317968
SRR17520069.18317969
SRR17520069.18321478
SRR17520069.18321479
SRR17520069.18321480
SRR17520069.18321481
SRR17520069.18321482
SRR17520069.18321483
SRR17520069.18321484
SRR17520069.18321485
SRR17520069.18321486
SRR17520069.18321487
SRR17520069.18321488
SRR17520069.18321489
SRR17520069.18321490
SRR17520069.18321491
SRR17520069.18321492
SRR17520069.18321494
SRR17520069.18321495
SRR17520069.18321496
SRR17520069.18321497
SRR17520069.18321498
SRR17520069.18321499
SRR17520069.18321500
SRR17520069.18321501
SRR17520069.18321502
SRR17520069.18321503
SRR17520069.18321504
SRR17520069.18321505
SRR17520069.18321506
SRR17520069.18321507
SRR17520069.18321508
SRR17520069.18321509
SRR17520069.18321510
SRR17520069.18321511
SRR17520069.18321512
SRR17520069.18321513

SRR17520069.18321514
SRR17520069.18321515
SRR17520069.18321516
SRR17520069.18321518
SRR17520069.18321519
SRR17520069.18321520
SRR17520069.18321521
SRR17520069.18321522
SRR17520069.18321523
SRR17520069.18321525
SRR17520069.18321526
SRR17520069.18321527
SRR17520069.18321528
SRR17520069.18321529
SRR17520069.18321530
SRR17520069.18321531
SRR17520069.18321533
SRR17520069.18321534
SRR17520069.18321535
SRR17520069.18321536
SRR17520069.18321537
SRR17520069.18321541
SRR17520069.18321542
SRR17520069.18321544
SRR17520069.18321545
SRR17520069.18321546
SRR17520069.18321547
SRR17520069.18321548
SRR17520069.18321549
SRR17520069.18321550
SRR17520069.18321551
SRR17520069.18321552
SRR17520069.18321553
SRR17520069.18324001
SRR17520069.18324624
SRR17520069.18324625
SRR17520069.18324626
SRR17520069.18324627
SRR17520069.18324628
SRR17520069.18324629
SRR17520069.18324630
SRR17520069.18324631
SRR17520069.18324632
SRR17520069.18324633
SRR17520069.18324634
SRR17520069.18324635
SRR17520069.18324636

SRR17520069.18324637
SRR17520069.18324638
SRR17520069.18324639
SRR17520069.18324640
SRR17520069.18328612
SRR17520069.18328613
SRR17520069.18328614
SRR17520069.18328615
SRR17520069.18328616
SRR17520069.18328617
SRR17520069.18328618
SRR17520069.18328619
SRR17520069.18328620
SRR17520069.18328621
SRR17520069.18328622
SRR17520069.18328623
SRR17520069.18328624
SRR17520069.18328625
SRR17520069.18328626
SRR17520069.18328627
SRR17520069.18328628
SRR17520069.18328629
SRR17520069.18328630
SRR17520069.18328631
SRR17520069.18328633
SRR17520069.18328634
SRR17520069.18328636
SRR17520069.18334030
SRR17520069.18341630
SRR17520069.18341631
SRR17520069.18341632
SRR17520069.18341633
SRR17520069.18341634
SRR17520069.18341635
SRR17520069.18341636
SRR17520069.18341637
SRR17520069.18341638
SRR17520069.18341639
SRR17520069.18341640
SRR17520069.18341641
SRR17520069.18341642
SRR17520069.18341643
SRR17520069.18341644
SRR17520069.18341645
SRR17520069.18341646
SRR17520069.18341647
SRR17520069.18341648

SRR17520069.18341649
SRR17520069.18341650
SRR17520069.18341651
SRR17520069.18341652
SRR17520069.18341654
SRR17520069.18341655
SRR17520069.18341656
SRR17520069.18341657
SRR17520069.18341658
SRR17520069.18341659
SRR17520069.18341660
SRR17520069.18341661
SRR17520069.18341662
SRR17520069.18341663
SRR17520069.18341664
SRR17520069.18341665
SRR17520069.18341666
SRR17520069.18341667
SRR17520069.18341668
SRR17520069.18341669
SRR17520069.18341670
SRR17520069.18341671
SRR17520069.18341673
SRR17520069.18341674
SRR17520069.18341675
SRR17520069.18341676
SRR17520069.18341677
SRR17520069.18341678
SRR17520069.18341679
SRR17520069.18341680
SRR17520069.18341681
SRR17520069.18341682
SRR17520069.18341683
SRR17520069.18341684
SRR17520069.18341685
SRR17520069.18341686
SRR17520069.18341687
SRR17520069.18341688
SRR17520069.18341689
SRR17520069.18341690
SRR17520069.18341691
SRR17520069.18341692
SRR17520069.18341693
SRR17520069.18341694
SRR17520069.18341695
SRR17520069.18341696
SRR17520069.18341697

SRR17520069.18341698
SRR17520069.18341699
SRR17520069.18341700
SRR17520069.18341701
SRR17520069.18341702
SRR17520069.18341703
SRR17520069.18341704
SRR17520069.18341705
SRR17520069.18341706
SRR17520069.18341707
SRR17520069.18341708
SRR17520069.18341709
SRR17520069.18341710
SRR17520069.18341711
SRR17520069.18341712
SRR17520069.18341713
SRR17520069.18341714
SRR17520069.18341715
SRR17520069.18341716
SRR17520069.18341717
SRR17520069.18341718
SRR17520069.18341719
SRR17520069.18341720
SRR17520069.18341721
SRR17520069.18341722
SRR17520069.18341723
SRR17520069.18341724
SRR17520069.18341725
SRR17520069.18341726
SRR17520069.18341727
SRR17520069.18341728
SRR17520069.18341729
SRR17520069.18341730
SRR17520069.18341731
SRR17520069.18341732
SRR17520069.18341733
SRR17520069.18341734
SRR17520069.18341735
SRR17520069.18341736
SRR17520069.18341737
SRR17520069.18341738
SRR17520069.18341739
SRR17520069.18341740
SRR17520069.18341741
SRR17520069.18341742
SRR17520069.18341743
SRR17520069.18341744

SRR17520069.18341745
SRR17520069.18341746
SRR17520069.18341747
SRR17520069.18341748
SRR17520069.18341749
SRR17520069.18341750
SRR17520069.18341751
SRR17520069.18341752
SRR17520069.18341753
SRR17520069.18341754
SRR17520069.18341755
SRR17520069.18341756
SRR17520069.18341757
SRR17520069.18341758
SRR17520069.18341760
SRR17520069.18341761
SRR17520069.18341762
SRR17520069.18341763
SRR17520069.18341764
SRR17520069.18341765
SRR17520069.18341766
SRR17520069.18341767
SRR17520069.18341768
SRR17520069.18341769
SRR17520069.18341770
SRR17520069.18341771
SRR17520069.18341772
SRR17520069.18341773
SRR17520069.18341774
SRR17520069.18341775
SRR17520069.18341776
SRR17520069.18341777
SRR17520069.18341778
SRR17520069.18341779
SRR17520069.18341780
SRR17520069.18341781
SRR17520069.18341782
SRR17520069.18341783
SRR17520069.18341784
SRR17520069.18341785
SRR17520069.18341786
SRR17520069.18341787
SRR17520069.18341788
SRR17520069.18341789
SRR17520069.18341790
SRR17520069.18341791
SRR17520069.18341792

SRR17520069.18341793
SRR17520069.18341794
SRR17520069.18341795
SRR17520069.18341796
SRR17520069.18341797
SRR17520069.18341798
SRR17520069.18341799
SRR17520069.18341800
SRR17520069.18341801
SRR17520069.18341802
SRR17520069.18341803
SRR17520069.18341804
SRR17520069.18341805
SRR17520069.18341806
SRR17520069.18341807
SRR17520069.18341808
SRR17520069.18341809
SRR17520069.18341810
SRR17520069.18341811
SRR17520069.18341812
SRR17520069.18341813
SRR17520069.18341814
SRR17520069.18341815
SRR17520069.18341816
SRR17520069.18341817
SRR17520069.18341818
SRR17520069.18341819
SRR17520069.18341820
SRR17520069.18341821
SRR17520069.18341822
SRR17520069.18341823
SRR17520069.18341824
SRR17520069.18341825
SRR17520069.18341826
SRR17520069.18341827
SRR17520069.18341828
SRR17520069.18341829
SRR17520069.18341830
SRR17520069.18341831
SRR17520069.18341832
SRR17520069.18341833
SRR17520069.18341834
SRR17520069.18341835
SRR17520069.18344744
SRR17520069.18348752
SRR17520069.18362560
SRR17520069.18381621

SRR17520069.18381622
SRR17520069.18381623
SRR17520069.18381624
SRR17520069.18381625
SRR17520069.18381626
SRR17520069.18381627
SRR17520069.18381628
SRR17520069.18381629
SRR17520069.18381630
SRR17520069.18381631
SRR17520069.18381632
SRR17520069.18381633
SRR17520069.18381634
SRR17520069.18381635
SRR17520069.18381636
SRR17520069.18381637
SRR17520069.18381638
SRR17520069.18381639
SRR17520069.18381640
SRR17520069.18381641
SRR17520069.18381642
SRR17520069.18381643
SRR17520069.18381644
SRR17520069.18381645
SRR17520069.18381646
SRR17520069.18381647
SRR17520069.18381648
SRR17520069.18381649
SRR17520069.18381650
SRR17520069.18381651
SRR17520069.18381652
SRR17520069.18381653
SRR17520069.18381655
SRR17520069.18381656
SRR17520069.18381657
SRR17520069.18381658
SRR17520069.18381659
SRR17520069.18381660
SRR17520069.18381661
SRR17520069.18381662
SRR17520069.18381663
SRR17520069.18381664
SRR17520069.18381665
SRR17520069.18381666
SRR17520069.18381667
SRR17520069.18381668
SRR17520069.18381669

SRR17520069.18381670
SRR17520069.18381671
SRR17520069.18381672
SRR17520069.18381673
SRR17520069.18381674
SRR17520069.18381675
SRR17520069.18381676
SRR17520069.18381677
SRR17520069.18381678
SRR17520069.18381679
SRR17520069.18381680
SRR17520069.18381681
SRR17520069.18381682
SRR17520069.18381683
SRR17520069.18381684
SRR17520069.18381685
SRR17520069.18381686
SRR17520069.18381687
SRR17520069.18381688
SRR17520069.18381689
SRR17520069.18381690
SRR17520069.18381691
SRR17520069.18381692
SRR17520069.18381693
SRR17520069.18381694
SRR17520069.18381695
SRR17520069.18381696
SRR17520069.18381697
SRR17520069.18381698
SRR17520069.18381699
SRR17520069.18381700
SRR17520069.18381701
SRR17520069.18381702
SRR17520069.18381703
SRR17520069.18381704
SRR17520069.18381705
SRR17520069.18381706
SRR17520069.18381707
SRR17520069.18381708
SRR17520069.18381709
SRR17520069.18381710
SRR17520069.18381711
SRR17520069.18381712
SRR17520069.18381713
SRR17520069.18381714
SRR17520069.18381715
SRR17520069.18381716

SRR17520069.18381717
SRR17520069.18381718
SRR17520069.18381719
SRR17520069.18381720
SRR17520069.18381721
SRR17520069.18381722
SRR17520069.18381723
SRR17520069.18381724
SRR17520069.18381725
SRR17520069.18381726
SRR17520069.18381727
SRR17520069.18381728
SRR17520069.18381729
SRR17520069.18381730
SRR17520069.18381731
SRR17520069.18381732
SRR17520069.18381733
SRR17520069.18381734
SRR17520069.18381735
SRR17520069.18381736
SRR17520069.18381737
SRR17520069.18381738
SRR17520069.18381739
SRR17520069.18381740
SRR17520069.18381741
SRR17520069.18381742
SRR17520069.18381743
SRR17520069.18381744
SRR17520069.18381745
SRR17520069.18381746
SRR17520069.18381747
SRR17520069.18381748
SRR17520069.18381749
SRR17520069.18383521
SRR17520069.18383542
SRR17520069.18383968
SRR17520069.18383969
SRR17520069.18383971
SRR17520069.18383972
SRR17520069.18383973
SRR17520069.18391318
SRR17520069.18391414
SRR17520069.18391431
SRR17520069.18399013
SRR17520069.18399014
SRR17520069.18399015
SRR17520069.18399016

SRR17520069.18399017
SRR17520069.18399018
SRR17520069.18399019
SRR17520069.18399020
SRR17520069.18399021
SRR17520069.18399022
SRR17520069.18399023
SRR17520069.18399024
SRR17520069.18399025
SRR17520069.18399026
SRR17520069.18399027
SRR17520069.18399028
SRR17520069.18399029
SRR17520069.18399030
SRR17520069.18399031
SRR17520069.18399032
SRR17520069.18399033
SRR17520069.18399034
SRR17520069.18399035
SRR17520069.18399036
SRR17520069.18399037
SRR17520069.18399038
SRR17520069.18399039
SRR17520069.18399040
SRR17520069.18399041
SRR17520069.18434320
SRR17520069.18440768
SRR17520069.18440769
SRR17520069.18440770
SRR17520069.18440771
SRR17520069.18440772
SRR17520069.18440773
SRR17520069.18440774
SRR17520069.18440776
SRR17520069.18440777
SRR17520069.18440780
SRR17520069.18440781
SRR17520069.18440782
SRR17520069.18472662
SRR17520069.18472663
SRR17520069.18472664
SRR17520069.18472665
SRR17520069.18472666
SRR17520069.18472667
SRR17520069.18472668
SRR17520069.18472669
SRR17520069.18472670

SRR17520069.18472671
SRR17520069.18472672
SRR17520069.18472673
SRR17520069.18472674
SRR17520069.18472675
SRR17520069.18472676
SRR17520069.18472677
SRR17520069.18472678
SRR17520069.18472679
SRR17520069.18472680
SRR17520069.18472681
SRR17520069.18472682
SRR17520069.18472683
SRR17520069.18472684
SRR17520069.18472685
SRR17520069.18472686
SRR17520069.18472687
SRR17520069.18472688
SRR17520069.18472689
SRR17520069.18472690
SRR17520069.18472691
SRR17520069.18472692
SRR17520069.18472693
SRR17520069.18472694
SRR17520069.18472695
SRR17520069.18472696
SRR17520069.18472697
SRR17520069.18472698
SRR17520069.18472699
SRR17520069.18472700
SRR17520069.18472701
SRR17520069.18472702
SRR17520069.18472703
SRR17520069.18472704
SRR17520069.18472705
SRR17520069.18472706
SRR17520069.18472707
SRR17520069.18472708
SRR17520069.18472709
SRR17520069.18472710
SRR17520069.18472711
SRR17520069.18472712
SRR17520069.18472713
SRR17520069.18472714
SRR17520069.18472715
SRR17520069.18472716
SRR17520069.18472717

SRR17520069.18472718
SRR17520069.18472719
SRR17520069.18472720
SRR17520069.18472721
SRR17520069.18472722
SRR17520069.18472723
SRR17520069.18472725
SRR17520069.18472726
SRR17520069.18472727
SRR17520069.18472728
SRR17520069.18472729
SRR17520069.18472730
SRR17520069.18472731
SRR17520069.18472732
SRR17520069.18472733
SRR17520069.18472734
SRR17520069.18472735
SRR17520069.18472736
SRR17520069.18472737
SRR17520069.18472738
SRR17520069.18472739
SRR17520069.18472740
SRR17520069.18472741
SRR17520069.18472742
SRR17520069.18472743
SRR17520069.18472744
SRR17520069.18472745
SRR17520069.18472746
SRR17520069.18472747
SRR17520069.18472748
SRR17520069.18472749
SRR17520069.18472750
SRR17520069.18472751
SRR17520069.18472752
SRR17520069.18472753
SRR17520069.18472754
SRR17520069.18472755
SRR17520069.18472756
SRR17520069.18472757
SRR17520069.18472758
SRR17520069.18472759
SRR17520069.18472760
SRR17520069.18472761
SRR17520069.18472762
SRR17520069.18472763
SRR17520069.18472764
SRR17520069.18472765

SRR17520069.18472766
SRR17520069.18472767
SRR17520069.18472768
SRR17520069.18472769
SRR17520069.18472770
SRR17520069.18472771
SRR17520069.18472772
SRR17520069.18472773
SRR17520069.18472774
SRR17520069.18472775
SRR17520069.18472776
SRR17520069.18472777
SRR17520069.18472778
SRR17520069.18472779
SRR17520069.18472780
SRR17520069.18472781
SRR17520069.18472782
SRR17520069.18472783
SRR17520069.18472784
SRR17520069.18472785
SRR17520069.18472786
SRR17520069.18472787
SRR17520069.18472788
SRR17520069.18472789
SRR17520069.18490405
SRR17520069.18492626
SRR17520069.18533383
SRR17520069.18533384
SRR17520069.18533385
SRR17520069.18533386
SRR17520069.18533387
SRR17520069.18533388
SRR17520069.18533389
SRR17520069.18533390
SRR17520069.18533391
SRR17520069.18533392
SRR17520069.18533393
SRR17520069.18533394
SRR17520069.18533395
SRR17520069.18533396
SRR17520069.18533397
SRR17520069.18533398
SRR17520069.18533399
SRR17520069.18533400
SRR17520069.18533401
SRR17520069.18533402
SRR17520069.18533403

SRR17520069.18533404
SRR17520069.18533405
SRR17520069.18533406
SRR17520069.18533407
SRR17520069.18533408
SRR17520069.18533409
SRR17520069.18533410
SRR17520069.18533411
SRR17520069.18533412
SRR17520069.18533413
SRR17520069.18533414
SRR17520069.18533415
SRR17520069.18533416
SRR17520069.18533417
SRR17520069.18533419
SRR17520069.18533420
SRR17520069.18533421
SRR17520069.18533423
SRR17520069.18533424
SRR17520069.18533425
SRR17520069.18533426
SRR17520069.18533427
SRR17520069.18533428
SRR17520069.18533429
SRR17520069.18533431
SRR17520069.18533432
SRR17520069.18533433
SRR17520069.18533434
SRR17520069.18533436
SRR17520069.18533437
SRR17520069.18533438
SRR17520069.18533439
SRR17520069.18533441
SRR17520069.18533442
SRR17520069.18533443
SRR17520069.18533444
SRR17520069.18533445
SRR17520069.18533447
SRR17520069.18533448
SRR17520069.18533449
SRR17520069.18533450
SRR17520069.18533451
SRR17520069.18533453
SRR17520069.18533454
SRR17520069.18533455
SRR17520069.18533456
SRR17520069.18533457

SRR17520069.18533458
SRR17520069.18533460
SRR17520069.18533461
SRR17520069.18533462
SRR17520069.18533463
SRR17520069.18533464
SRR17520069.18533465
SRR17520069.18533466
SRR17520069.18533467
SRR17520069.18533468
SRR17520069.18533469
SRR17520069.18533470
SRR17520069.18533471
SRR17520069.18533472
SRR17520069.18533473
SRR17520069.18533474
SRR17520069.18533475
SRR17520069.18533476
SRR17520069.18533478
SRR17520069.18533479
SRR17520069.18533480
SRR17520069.18533481
SRR17520069.18533482
SRR17520069.18533483
SRR17520069.18533485
SRR17520069.18533486
SRR17520069.18533488
SRR17520069.18533489
SRR17520069.18533490
SRR17520069.18533491
SRR17520069.18533492
SRR17520069.18533493
SRR17520069.18533494
SRR17520069.18533495
SRR17520069.18533496
SRR17520069.18616710
SRR17520069.18620264
SRR17520069.18620265
SRR17520069.18620266
SRR17520069.18620267
SRR17520069.18620268
SRR17520069.18620269
SRR17520069.18620270
SRR17520069.18620271
SRR17520069.18620272
SRR17520069.18620273
SRR17520069.18620274

SRR17520069.18620275
SRR17520069.18620276
SRR17520069.18620277
SRR17520069.18620278
SRR17520069.18620279
SRR17520069.18620280
SRR17520069.18620281
SRR17520069.18620282
SRR17520069.18620283
SRR17520069.18620284
SRR17520069.18620285
SRR17520069.18620286
SRR17520069.18620287
SRR17520069.18620288
SRR17520069.18620289
SRR17520069.18620290
SRR17520069.18620291
SRR17520069.18620292
SRR17520069.18620293
SRR17520069.18620294
SRR17520069.18620295
SRR17520069.18620296
SRR17520069.18620297
SRR17520069.18620298
SRR17520069.18620299
SRR17520069.18620300
SRR17520069.18620301
SRR17520069.18620302
SRR17520069.18620303
SRR17520069.18620304
SRR17520069.18620305
SRR17520069.18620306
SRR17520069.18620307
SRR17520069.18620308
SRR17520069.18620309
SRR17520069.18620310
SRR17520069.18620311
SRR17520069.18620312
SRR17520069.18620313
SRR17520069.18620314
SRR17520069.18620315
SRR17520069.18620316
SRR17520069.18620317
SRR17520069.18620318
SRR17520069.18620319
SRR17520069.18620320
SRR17520069.18620321

SRR17520069.18620322
SRR17520069.18620323
SRR17520069.18620324
SRR17520069.18620325
SRR17520069.18620326
SRR17520069.18620327
SRR17520069.18620328
SRR17520069.18620329
SRR17520069.18621500
SRR17520069.18621501
SRR17520069.18621502
SRR17520069.18621503
SRR17520069.18621504
SRR17520069.18621505
SRR17520069.18621506
SRR17520069.18621507
SRR17520069.18621508
SRR17520069.18621509
SRR17520069.18621510
SRR17520069.18621511
SRR17520069.18621512
SRR17520069.18621513
SRR17520069.18621514
SRR17520069.18626610
SRR17520069.18665126
SRR17520069.18665127
SRR17520069.18665128
SRR17520069.18665129
SRR17520069.18665130
SRR17520069.18665131
SRR17520069.18665132
SRR17520069.18665133
SRR17520069.18665134
SRR17520069.18665135
SRR17520069.18665136
SRR17520069.18665137
SRR17520069.18665138
SRR17520069.18665139
SRR17520069.18665140
SRR17520069.18665141
SRR17520069.18665142
SRR17520069.18665143
SRR17520069.18665144
SRR17520069.18665145
SRR17520069.18665146
SRR17520069.18665147
SRR17520069.18665148

SRR17520069.18665149
SRR17520069.18665150
SRR17520069.18665151
SRR17520069.18665152
SRR17520069.18665153
SRR17520069.18665154
SRR17520069.18665155
SRR17520069.18665156
SRR17520069.18665157
SRR17520069.18665158
SRR17520069.18665159
SRR17520069.18665160
SRR17520069.18665161
SRR17520069.18665162
SRR17520069.18665163
SRR17520069.18665164
SRR17520069.18665165
SRR17520069.18665166
SRR17520069.18665167
SRR17520069.18665168
SRR17520069.18665169
SRR17520069.18665170
SRR17520069.18665171
SRR17520069.18665172
SRR17520069.18665173
SRR17520069.18665174
SRR17520069.18665175
SRR17520069.18665296
SRR17520069.18673524
SRR17520069.18682855
SRR17520069.18682856
SRR17520069.18682857
SRR17520069.18682858
SRR17520069.18682859
SRR17520069.18682860
SRR17520069.18682861
SRR17520069.18682862
SRR17520069.18682863
SRR17520069.18682864
SRR17520069.18689236
SRR17520069.18693970
SRR17520069.18710380
SRR17520069.18716210
SRR17520069.18716211
SRR17520069.18716212
SRR17520069.18716213
SRR17520069.18716214

SRR17520069.18716215
SRR17520069.18716216
SRR17520069.18716217
SRR17520069.18716218
SRR17520069.18716219
SRR17520069.18716220
SRR17520069.18716221
SRR17520069.18716222
SRR17520069.18716223
SRR17520069.18720984
SRR17520069.18722617
SRR17520069.18722618
SRR17520069.18722619
SRR17520069.18722620
SRR17520069.18722621
SRR17520069.18722622
SRR17520069.18722623
SRR17520069.18722624
SRR17520069.18722625
SRR17520069.18722626
SRR17520069.18722627
SRR17520069.18722628
SRR17520069.18722629
SRR17520069.18722631
SRR17520069.18722632
SRR17520069.18722633
SRR17520069.18722634
SRR17520069.18722635
SRR17520069.18722636
SRR17520069.18722637
SRR17520069.18722638
SRR17520069.18722639
SRR17520069.18722640
SRR17520069.18722641
SRR17520069.18722642
SRR17520069.18722643
SRR17520069.18722644
SRR17520069.18722645
SRR17520069.18722647
SRR17520069.18722648
SRR17520069.18722649
SRR17520069.18722650
SRR17520069.18722651
SRR17520069.18722652
SRR17520069.18722653
SRR17520069.18722654
SRR17520069.18722655

SRR17520069.18722656
SRR17520069.18722657
SRR17520069.18722658
SRR17520069.18722659
SRR17520069.18722660
SRR17520069.18722661
SRR17520069.18722662
SRR17520069.18722663
SRR17520069.18722664
SRR17520069.18722665
SRR17520069.18722666
SRR17520069.18722667
SRR17520069.18722668
SRR17520069.18722669
SRR17520069.18722670
SRR17520069.18722671
SRR17520069.18722672
SRR17520069.18722673
SRR17520069.18722674
SRR17520069.18722675
SRR17520069.18722676
SRR17520069.18722677
SRR17520069.18722678
SRR17520069.18722679
SRR17520069.18722680
SRR17520069.18722681
SRR17520069.18722682
SRR17520069.18722683
SRR17520069.18722684
SRR17520069.18722685
SRR17520069.18722686
SRR17520069.18722687
SRR17520069.18722688
SRR17520069.18722689
SRR17520069.18722690
SRR17520069.18722691
SRR17520069.18722692
SRR17520069.18722693
SRR17520069.18722694
SRR17520069.18722695
SRR17520069.18722696
SRR17520069.18722697
SRR17520069.18722698
SRR17520069.18722699
SRR17520069.18722700
SRR17520069.18722701
SRR17520069.18722702

SRR17520069.18722703
SRR17520069.18722704
SRR17520069.18722705
SRR17520069.18722706
SRR17520069.18722708
SRR17520069.18722709
SRR17520069.18722710
SRR17520069.18722711
SRR17520069.18722712
SRR17520069.18722713
SRR17520069.18722714
SRR17520069.18722715
SRR17520069.18722716
SRR17520069.18722717
SRR17520069.18722718
SRR17520069.18722719
SRR17520069.18722720
SRR17520069.18722721
SRR17520069.18722722
SRR17520069.18722723
SRR17520069.18722724
SRR17520069.18722725
SRR17520069.18722726
SRR17520069.18722727
SRR17520069.18722728
SRR17520069.18722729
SRR17520069.18722730
SRR17520069.18722731
SRR17520069.18722732
SRR17520069.18722733
SRR17520069.18725114
SRR17520069.18725115
SRR17520069.18725116
SRR17520069.18725117
SRR17520069.18725118
SRR17520069.18725119
SRR17520069.18725120
SRR17520069.18725121
SRR17520069.18725122
SRR17520069.18725123
SRR17520069.18725124
SRR17520069.18725125
SRR17520069.18725126
SRR17520069.18725127
SRR17520069.18725128
SRR17520069.18725129
SRR17520069.18725130

SRR17520069.18725131
SRR17520069.18725132
SRR17520069.18725133
SRR17520069.18725134
SRR17520069.18725135
SRR17520069.18725136
SRR17520069.18725137
SRR17520069.18725138
SRR17520069.18725139
SRR17520069.18725140
SRR17520069.18725141
SRR17520069.18725142
SRR17520069.18725143
SRR17520069.18725144
SRR17520069.18725145
SRR17520069.18725146
SRR17520069.18725147
SRR17520069.18725148
SRR17520069.18725149
SRR17520069.18725150
SRR17520069.18725151
SRR17520069.18725152
SRR17520069.18725153
SRR17520069.18725154
SRR17520069.18725155
SRR17520069.18725156
SRR17520069.18725157
SRR17520069.18725158
SRR17520069.18725159
SRR17520069.18725160
SRR17520069.18725161
SRR17520069.18725162
SRR17520069.18725164
SRR17520069.18725165
SRR17520069.18725166
SRR17520069.18725167
SRR17520069.18725168
SRR17520069.18725169
SRR17520069.18725170
SRR17520069.18725171
SRR17520069.18725172
SRR17520069.18725173
SRR17520069.18725174
SRR17520069.18725175
SRR17520069.18725176
SRR17520069.18725178
SRR17520069.18725179

SRR17520069.18725180
SRR17520069.18725181
SRR17520069.18725182
SRR17520069.18725183
SRR17520069.18725184
SRR17520069.18725185
SRR17520069.18725186
SRR17520069.18725187
SRR17520069.18725188
SRR17520069.18725190
SRR17520069.18725191
SRR17520069.18725192
SRR17520069.18725193
SRR17520069.18725194
SRR17520069.18725195
SRR17520069.18725196
SRR17520069.18725197
SRR17520069.18725198
SRR17520069.18725199
SRR17520069.18725200
SRR17520069.18725201
SRR17520069.18725202
SRR17520069.18725203
SRR17520069.18725204
SRR17520069.18725206
SRR17520069.18725207
SRR17520069.18725208
SRR17520069.18725209
SRR17520069.18725210
SRR17520069.18725211
SRR17520069.18725212
SRR17520069.18725213
SRR17520069.18725214
SRR17520069.18725218
SRR17520069.18725220
SRR17520069.18732257
SRR17520069.18732258
SRR17520069.18732259
SRR17520069.18744954
SRR17520069.18744955
SRR17520069.18744956
SRR17520069.18744958
SRR17520069.18744959
SRR17520069.18744960
SRR17520069.18744961
SRR17520069.18744962
SRR17520069.18744963

SRR17520069.18744964
SRR17520069.18744965
SRR17520069.18744966
SRR17520069.18744967
SRR17520069.18744968
SRR17520069.18744969
SRR17520069.18744970
SRR17520069.18744971
SRR17520069.18744972
SRR17520069.18744973
SRR17520069.18744974
SRR17520069.18744975
SRR17520069.18744976
SRR17520069.18744977
SRR17520069.18744978
SRR17520069.18744979
SRR17520069.18744981
SRR17520069.18744982
SRR17520069.18744983
SRR17520069.18744984
SRR17520069.18744985
SRR17520069.18744986
SRR17520069.18744987
SRR17520069.18744988
SRR17520069.18744989
SRR17520069.18746596
SRR17520069.18746597
SRR17520069.18746598
SRR17520069.18746599
SRR17520069.18746600
SRR17520069.18746601
SRR17520069.18746602
SRR17520069.18746603
SRR17520069.18746604
SRR17520069.18746605
SRR17520069.18746606
SRR17520069.18746607
SRR17520069.18746608
SRR17520069.18746609
SRR17520069.18746610
SRR17520069.18746611
SRR17520069.18746612
SRR17520069.18746613
SRR17520069.18746614
SRR17520069.18746615
SRR17520069.18746616
SRR17520069.18746617

SRR17520069.18746618
SRR17520069.18746619
SRR17520069.18746620
SRR17520069.18746621
SRR17520069.18746622
SRR17520069.18746623
SRR17520069.18746624
SRR17520069.18746625
SRR17520069.18746626
SRR17520069.18746627
SRR17520069.18746628
SRR17520069.18746629
SRR17520069.18746630
SRR17520069.18746631
SRR17520069.18746632
SRR17520069.18746633
SRR17520069.18746634
SRR17520069.18746635
SRR17520069.18746636
SRR17520069.18746637
SRR17520069.18746638
SRR17520069.18746639
SRR17520069.18746640
SRR17520069.18746641
SRR17520069.18746642
SRR17520069.18746643
SRR17520069.18746644
SRR17520069.18746645
SRR17520069.18746646
SRR17520069.18746647
SRR17520069.18746648
SRR17520069.18746649
SRR17520069.18746651
SRR17520069.18746652
SRR17520069.18746653
SRR17520069.18746654
SRR17520069.18746655
SRR17520069.18746656
SRR17520069.18746657
SRR17520069.18746658
SRR17520069.18746659
SRR17520069.18746660
SRR17520069.18746661
SRR17520069.18746662
SRR17520069.18746663
SRR17520069.18746664
SRR17520069.18746665

SRR17520069.18746666
SRR17520069.18746667
SRR17520069.18746668
SRR17520069.18746669
SRR17520069.18746670
SRR17520069.18746671
SRR17520069.18746672
SRR17520069.18746673
SRR17520069.18746674
SRR17520069.18746675
SRR17520069.18746676
SRR17520069.18746677
SRR17520069.18746678
SRR17520069.18746679
SRR17520069.18746680
SRR17520069.18746681
SRR17520069.18746682
SRR17520069.18746683
SRR17520069.18746684
SRR17520069.18746685
SRR17520069.18746687
SRR17520069.18746688
SRR17520069.18746689
SRR17520069.18746690
SRR17520069.18746691
SRR17520069.18746692
SRR17520069.18746693
SRR17520069.18746694
SRR17520069.18746695
SRR17520069.18746696
SRR17520069.18746697
SRR17520069.18746698
SRR17520069.18746699
SRR17520069.18746700
SRR17520069.18746701
SRR17520069.18746702
SRR17520069.18746703
SRR17520069.18746704
SRR17520069.18746705
SRR17520069.18746707
SRR17520069.18746708
SRR17520069.18746709
SRR17520069.18746710
SRR17520069.18746711
SRR17520069.18746712
SRR17520069.18746713
SRR17520069.18746714

SRR17520069.18746715
SRR17520069.18746716
SRR17520069.18746717
SRR17520069.18746718
SRR17520069.18746719
SRR17520069.18746720
SRR17520069.18746721
SRR17520069.18746722
SRR17520069.18746723
SRR17520069.18746724
SRR17520069.18746725
SRR17520069.18746726
SRR17520069.18746727
SRR17520069.18746728
SRR17520069.18746729
SRR17520069.18746730
SRR17520069.18746731
SRR17520069.18746732
SRR17520069.18746733
SRR17520069.18746734
SRR17520069.18746735
SRR17520069.18746736
SRR17520069.18746737
SRR17520069.18746738
SRR17520069.18746739
SRR17520069.18746740
SRR17520069.18746741
SRR17520069.18746742
SRR17520069.18746743
SRR17520069.18746744
SRR17520069.18746745
SRR17520069.18746746
SRR17520069.18757558
SRR17520069.18757559
SRR17520069.18757560
SRR17520069.18757561
SRR17520069.18757562
SRR17520069.18757563
SRR17520069.18764100
SRR17520069.18764108
SRR17520069.18772539
SRR17520069.18772540
SRR17520069.18772541
SRR17520069.18772543
SRR17520069.18772544
SRR17520069.18772545
SRR17520069.18772546

SRR17520069.18772547
SRR17520069.18778520
SRR17520069.18778521
SRR17520069.18807233
SRR17520069.18810718
SRR17520069.18810763
SRR17520069.18810784
SRR17520069.18810812
SRR17520069.18826398
SRR17520069.18826400
SRR17520069.18826401
SRR17520069.18826402
SRR17520069.18842195
SRR17520069.18842196
SRR17520069.18842197
SRR17520069.18842198
SRR17520069.18842199
SRR17520069.18842200
SRR17520069.18842201
SRR17520069.18842202
SRR17520069.18842203
SRR17520069.18842204
SRR17520069.18842205
SRR17520069.18842206
SRR17520069.18842207
SRR17520069.18842208
SRR17520069.18842209
SRR17520069.18842210
SRR17520069.18842211
SRR17520069.18842212
SRR17520069.18842213
SRR17520069.18842214
SRR17520069.18842215
SRR17520069.18842217
SRR17520069.18842218
SRR17520069.18842219
SRR17520069.18842220
SRR17520069.18842222
SRR17520069.18842223
SRR17520069.18842224
SRR17520069.18842225
SRR17520069.18842226
SRR17520069.18842227
SRR17520069.18842228
SRR17520069.18842229
SRR17520069.18842230
SRR17520069.18842231

SRR17520069.18842232
SRR17520069.18842233
SRR17520069.18842234
SRR17520069.18842235
SRR17520069.18842236
SRR17520069.18842237
SRR17520069.18842238
SRR17520069.18842239
SRR17520069.18842240
SRR17520069.18842241
SRR17520069.18842242
SRR17520069.18842243
SRR17520069.18842244
SRR17520069.18842245
SRR17520069.18842246
SRR17520069.18842247
SRR17520069.18842248
SRR17520069.18842249
SRR17520069.18842250
SRR17520069.18842251
SRR17520069.18842252
SRR17520069.18842253
SRR17520069.18842254
SRR17520069.18842255
SRR17520069.18842256
SRR17520069.18842257
SRR17520069.18842258
SRR17520069.18842259
SRR17520069.18842260
SRR17520069.18842261
SRR17520069.18842262
SRR17520069.18842263
SRR17520069.18842264
SRR17520069.18842265
SRR17520069.18842266
SRR17520069.18842267
SRR17520069.18842268
SRR17520069.18842269
SRR17520069.18842270
SRR17520069.18842271
SRR17520069.18842273
SRR17520069.18842274
SRR17520069.18842275
SRR17520069.18842276
SRR17520069.18842277
SRR17520069.18842278
SRR17520069.18842279

SRR17520069.18842280
SRR17520069.18842281
SRR17520069.18842282
SRR17520069.18842283
SRR17520069.18842284
SRR17520069.18842285
SRR17520069.18842286
SRR17520069.18842287
SRR17520069.18842288
SRR17520069.18842289
SRR17520069.18842290
SRR17520069.18842291
SRR17520069.18842292
SRR17520069.18842293
SRR17520069.18842294
SRR17520069.18842295
SRR17520069.18842296
SRR17520069.18842297
SRR17520069.18842298
SRR17520069.18842299
SRR17520069.18842300
SRR17520069.18842301
SRR17520069.18842302
SRR17520069.18842303
SRR17520069.18842304
SRR17520069.18842305
SRR17520069.18842306
SRR17520069.18842307
SRR17520069.18842308
SRR17520069.18842309
SRR17520069.18842310
SRR17520069.18842311
SRR17520069.18842312
SRR17520069.18842313
SRR17520069.18842314
SRR17520069.18842315
SRR17520069.18842316
SRR17520069.18842317
SRR17520069.18842318
SRR17520069.18842319
SRR17520069.18842320
SRR17520069.18842321
SRR17520069.18842322
SRR17520069.18842323
SRR17520069.18842324
SRR17520069.18842325
SRR17520069.18842326

SRR17520069.18842327
SRR17520069.18842328
SRR17520069.18842329
SRR17520069.18842330
SRR17520069.18842331
SRR17520069.18842332
SRR17520069.18842333
SRR17520069.18842334
SRR17520069.18842335
SRR17520069.18842336
SRR17520069.18842337
SRR17520069.18842338
SRR17520069.18842339
SRR17520069.18842340
SRR17520069.18842341
SRR17520069.18842342
SRR17520069.18842343
SRR17520069.18842344
SRR17520069.18842345
SRR17520069.18842346
SRR17520069.18842347
SRR17520069.18842348
SRR17520069.18842349
SRR17520069.18842350
SRR17520069.18842351
SRR17520069.18842352
SRR17520069.18842353
SRR17520069.18842354
SRR17520069.18842355
SRR17520069.18842356
SRR17520069.18842357
SRR17520069.18842358
SRR17520069.18842359
SRR17520069.18842360
SRR17520069.18842361
SRR17520069.18842362
SRR17520069.18842363
SRR17520069.18842364
SRR17520069.18842365
SRR17520069.18842366
SRR17520069.18842367
SRR17520069.18842368
SRR17520069.18842369
SRR17520069.18848459
SRR17520069.18848827
SRR17520069.18848828
SRR17520069.18848829

SRR17520069.18848830
SRR17520069.18848831
SRR17520069.18848833
SRR17520069.18848834
SRR17520069.18848839
SRR17520069.18848840
SRR17520069.18848841
SRR17520069.18848842
SRR17520069.18848843
SRR17520069.18848844
SRR17520069.18848845
SRR17520069.18848846
SRR17520069.18848847
SRR17520069.18848848
SRR17520069.18848849
SRR17520069.18848850
SRR17520069.18848851
SRR17520069.18848852
SRR17520069.18848853
SRR17520069.18848854
SRR17520069.18848855
SRR17520069.18848856
SRR17520069.18848857
SRR17520069.18848858
SRR17520069.18848859
SRR17520069.18848860
SRR17520069.18848861
SRR17520069.18848862
SRR17520069.18848863
SRR17520069.18848864
SRR17520069.18848865
SRR17520069.18848866
SRR17520069.18848867
SRR17520069.18848868
SRR17520069.18848869
SRR17520069.18848870
SRR17520069.18848871
SRR17520069.18848872
SRR17520069.18848873
SRR17520069.18848874
SRR17520069.18848875
SRR17520069.18848876
SRR17520069.18848877
SRR17520069.18848878
SRR17520069.18848879
SRR17520069.18848880
SRR17520069.18848881

SRR17520069.18848882
SRR17520069.18848883
SRR17520069.18848884
SRR17520069.18848885
SRR17520069.18848886
SRR17520069.18848887
SRR17520069.18848888
SRR17520069.18848889
SRR17520069.18848890
SRR17520069.18868914
SRR17520069.18868915
SRR17520069.18868916
SRR17520069.18868917
SRR17520069.18868918
SRR17520069.18868919
SRR17520069.18868920
SRR17520069.18868921
SRR17520069.18868922
SRR17520069.18868923
SRR17520069.18868924
SRR17520069.18868925
SRR17520069.18868929
SRR17520069.18868930
SRR17520069.18868931
SRR17520069.18868932
SRR17520069.18868933
SRR17520069.18868934
SRR17520069.18868935
SRR17520069.18868936
SRR17520069.18868937
SRR17520069.18868938
SRR17520069.18868939
SRR17520069.18868940
SRR17520069.18868941
SRR17520069.18868942
SRR17520069.18868943
SRR17520069.18868944
SRR17520069.18868945
SRR17520069.18868946
SRR17520069.18868948
SRR17520069.18868949
SRR17520069.18868950
SRR17520069.18868951
SRR17520069.18868952
SRR17520069.18868953
SRR17520069.18868954
SRR17520069.18868955

SRR17520069.18868956
SRR17520069.18868957
SRR17520069.18868958
SRR17520069.18868959
SRR17520069.18868960
SRR17520069.18868961
SRR17520069.18868962
SRR17520069.18868963
SRR17520069.18868964
SRR17520069.18868965
SRR17520069.18868966
SRR17520069.18868967
SRR17520069.18868968
SRR17520069.18868969
SRR17520069.18868970
SRR17520069.18868971
SRR17520069.18868972
SRR17520069.18868973
SRR17520069.18868974
SRR17520069.18868975
SRR17520069.18868976
SRR17520069.18868977
SRR17520069.18868978
SRR17520069.18868979
SRR17520069.18868980
SRR17520069.18868981
SRR17520069.18868982
SRR17520069.18868983
SRR17520069.18868984
SRR17520069.18868985
SRR17520069.18868986
SRR17520069.18868987
SRR17520069.18868988
SRR17520069.18868989
SRR17520069.18868990
SRR17520069.18868992
SRR17520069.18868993
SRR17520069.18868994
SRR17520069.18868995
SRR17520069.18868996
SRR17520069.18868997
SRR17520069.18868998
SRR17520069.18868999
SRR17520069.18869000
SRR17520069.18869001
SRR17520069.18869002
SRR17520069.18869003

SRR17520069.18869004
SRR17520069.18869005
SRR17520069.18869006
SRR17520069.18869007
SRR17520069.18869008
SRR17520069.18869009
SRR17520069.18869010
SRR17520069.18869011
SRR17520069.18869012
SRR17520069.18869013
SRR17520069.18869014
SRR17520069.18869015
SRR17520069.18869016
SRR17520069.18869017
SRR17520069.18869018
SRR17520069.18869019
SRR17520069.18869020
SRR17520069.18869021
SRR17520069.18869022
SRR17520069.18869023
SRR17520069.18869024
SRR17520069.18869025
SRR17520069.18869026
SRR17520069.18869027
SRR17520069.18869028
SRR17520069.18869029
SRR17520069.18869030
SRR17520069.18869031
SRR17520069.18869032
SRR17520069.18869033
SRR17520069.18869034
SRR17520069.18869035
SRR17520069.18869036
SRR17520069.18869037
SRR17520069.18869038
SRR17520069.18869039
SRR17520069.18869040
SRR17520069.18869041
SRR17520069.18869042
SRR17520069.18869043
SRR17520069.18869044
SRR17520069.18869045
SRR17520069.18869046
SRR17520069.18869047
SRR17520069.18869048
SRR17520069.18869049
SRR17520069.18869050

SRR17520069.18869051
SRR17520069.18869052
SRR17520069.18869053
SRR17520069.18869054
SRR17520069.18869055
SRR17520069.18869056
SRR17520069.18894607
SRR17520069.18894608
SRR17520069.18894610
SRR17520069.18894611
SRR17520069.18894612
SRR17520069.18894613
SRR17520069.18894614
SRR17520069.18894615
SRR17520069.18894616
SRR17520069.18894617
SRR17520069.18894618
SRR17520069.18894620
SRR17520069.18894621
SRR17520069.18894622
SRR17520069.18894623
SRR17520069.18894624
SRR17520069.18894625
SRR17520069.18894626
SRR17520069.18894627
SRR17520069.18894628
SRR17520069.18894629
SRR17520069.18894630
SRR17520069.18894631
SRR17520069.18894633
SRR17520069.18894634
SRR17520069.18894635
SRR17520069.18894636
SRR17520069.18894637
SRR17520069.18894638
SRR17520069.18894640
SRR17520069.18894641
SRR17520069.18894642
SRR17520069.18894643
SRR17520069.18894644
SRR17520069.18894645
SRR17520069.18894646
SRR17520069.18894647
SRR17520069.18894649
SRR17520069.18894651
SRR17520069.18894652
SRR17520069.18894654

SRR17520069.18894655
SRR17520069.18894656
SRR17520069.18894657
SRR17520069.18894659
SRR17520069.18894660
SRR17520069.18894661
SRR17520069.18894662
SRR17520069.18894663
SRR17520069.18894664
SRR17520069.18894667
SRR17520069.18894668
SRR17520069.18894669
SRR17520069.18894670
SRR17520069.18894671
SRR17520069.18894672
SRR17520069.18894673
SRR17520069.18894674
SRR17520069.18894675
SRR17520069.18894677
SRR17520069.18894678
SRR17520069.18894679
SRR17520069.18894680
SRR17520069.18894681
SRR17520069.18894682
SRR17520069.18894683
SRR17520069.18894684
SRR17520069.18894685
SRR17520069.18894686
SRR17520069.18894687
SRR17520069.18894688
SRR17520069.18894689
SRR17520069.18894690
SRR17520069.18894691
SRR17520069.18894692
SRR17520069.18894693
SRR17520069.18894694
SRR17520069.18894695
SRR17520069.18894696
SRR17520069.18894697
SRR17520069.18894698
SRR17520069.18894699
SRR17520069.18894700
SRR17520069.18894702
SRR17520069.18894703
SRR17520069.18894704
SRR17520069.18894705
SRR17520069.18894706

SRR17520069.18894707
SRR17520069.18894708
SRR17520069.18894709
SRR17520069.18894710
SRR17520069.18894711
SRR17520069.18894712
SRR17520069.18894713
SRR17520069.18894714
SRR17520069.18894716
SRR17520069.18894718
SRR17520069.18894719
SRR17520069.18894720
SRR17520069.18894721
SRR17520069.18894722
SRR17520069.18894723
SRR17520069.18894725
SRR17520069.18894726
SRR17520069.18894727
SRR17520069.18894728
SRR17520069.18894729
SRR17520069.18894730
SRR17520069.18894731
SRR17520069.18894732
SRR17520069.18894733
SRR17520069.18894735
SRR17520069.18894736
SRR17520069.18894737
SRR17520069.18894738
SRR17520069.18894739
SRR17520069.18894740
SRR17520069.18894741
SRR17520069.18894742
SRR17520069.18894744
SRR17520069.18894745
SRR17520069.18894746
SRR17520069.18894747
SRR17520069.18894749
SRR17520069.18894750
SRR17520069.18894751
SRR17520069.18894752
SRR17520069.18894754
SRR17520069.18894755
SRR17520069.18894756
SRR17520069.18894757
SRR17520069.18894758
SRR17520069.18894759
SRR17520069.18894760

SRR17520069.18894761
SRR17520069.18894762
SRR17520069.18894763
SRR17520069.18913971
SRR17520069.18913972
SRR17520069.18915117
SRR17520069.18915126
SRR17520069.18919357
SRR17520069.18920024
SRR17520069.18920025
SRR17520069.18920026
SRR17520069.18920027
SRR17520069.18920028
SRR17520069.18920029
SRR17520069.18920030
SRR17520069.18920031
SRR17520069.18923951
SRR17520069.18923952
SRR17520069.18923953
SRR17520069.18923954
SRR17520069.18923955
SRR17520069.18923956
SRR17520069.18923958
SRR17520069.18923959
SRR17520069.18923960
SRR17520069.18923961
SRR17520069.18923962
SRR17520069.18923963
SRR17520069.18923964
SRR17520069.18923965
SRR17520069.18923966
SRR17520069.18923967
SRR17520069.18923968
SRR17520069.18923969
SRR17520069.18923970
SRR17520069.18923971
SRR17520069.18923972
SRR17520069.18923973
SRR17520069.18923974
SRR17520069.18923975
SRR17520069.18923976
SRR17520069.18923977
SRR17520069.18923978
SRR17520069.18923979
SRR17520069.18923980
SRR17520069.18923981
SRR17520069.18923982

SRR17520069.18923983
SRR17520069.18923984
SRR17520069.18923985
SRR17520069.18923986
SRR17520069.18923987
SRR17520069.18923988
SRR17520069.18923989
SRR17520069.18923990
SRR17520069.18923991
SRR17520069.18923992
SRR17520069.18923993
SRR17520069.18923995
SRR17520069.18923997
SRR17520069.18923998
SRR17520069.18923999
SRR17520069.18924000
SRR17520069.18924001
SRR17520069.18924002
SRR17520069.18924003
SRR17520069.18924004
SRR17520069.18924005
SRR17520069.18924006
SRR17520069.18924007
SRR17520069.18924008
SRR17520069.18924009
SRR17520069.18924509
SRR17520069.18924514
SRR17520069.18924515
SRR17520069.18924516
SRR17520069.18924528
SRR17520069.18924531
SRR17520069.18924535
SRR17520069.18924536
SRR17520069.18925778
SRR17520069.18925779
SRR17520069.18925780
SRR17520069.18925781
SRR17520069.18925782
SRR17520069.18948051
SRR17520069.18962316
SRR17520069.18962354
SRR17520069.18969058
SRR17520069.18969059
SRR17520069.18969060
SRR17520069.18976740
SRR17520069.18976741
SRR17520069.18976742

SRR17520069.18976743
SRR17520069.18976744
SRR17520069.18976745
SRR17520069.18976746
SRR17520069.18976747
SRR17520069.18986000
SRR17520069.18998578
SRR17520069.18999863
SRR17520069.18999879
SRR17520069.19012375
SRR17520069.19012376
SRR17520069.19012377
SRR17520069.19012378
SRR17520069.19012379
SRR17520069.19012380
SRR17520069.19012381
SRR17520069.19012382
SRR17520069.19012383
SRR17520069.19012385
SRR17520069.19012386
SRR17520069.19012387
SRR17520069.19012388
SRR17520069.19012389
SRR17520069.19012390
SRR17520069.19012391
SRR17520069.19012393
SRR17520069.19012394
SRR17520069.19012396
SRR17520069.19012397
SRR17520069.19012398
SRR17520069.19012399
SRR17520069.19012400
SRR17520069.19012402
SRR17520069.19012403
SRR17520069.19012404
SRR17520069.19012405
SRR17520069.19012406
SRR17520069.19012407
SRR17520069.19012409
SRR17520069.19012410
SRR17520069.19056209
SRR17520069.19056212
SRR17520069.19060867
SRR17520069.19060868
SRR17520069.19060869
SRR17520069.19060870
SRR17520069.19060871

SRR17520069.19060872
SRR17520069.19060874
SRR17520069.19060876
SRR17520069.19060877
SRR17520069.19060878
SRR17520069.19060879
SRR17520069.19060880
SRR17520069.19060882
SRR17520069.19060883
SRR17520069.19060884
SRR17520069.19060885
SRR17520069.19060886
SRR17520069.19060887
SRR17520069.19060888
SRR17520069.19060889
SRR17520069.19060890
SRR17520069.19060891
SRR17520069.19060892
SRR17520069.19060893
SRR17520069.19060894
SRR17520069.19060895
SRR17520069.19060896
SRR17520069.19060897
SRR17520069.19060898
SRR17520069.19060899
SRR17520069.19060900
SRR17520069.19060901
SRR17520069.19060902
SRR17520069.19060903
SRR17520069.19060904
SRR17520069.19060905
SRR17520069.19060907
SRR17520069.19060908
SRR17520069.19060909
SRR17520069.19066002
SRR17520069.19075382
SRR17520069.19075383
SRR17520069.19075384
SRR17520069.19075385
SRR17520069.19075386
SRR17520069.19075387
SRR17520069.19075389
SRR17520069.19075390
SRR17520069.19075391
SRR17520069.19075393
SRR17520069.19075394
SRR17520069.19075395

SRR17520069.19075396
SRR17520069.19075397
SRR17520069.19075398
SRR17520069.19075399
SRR17520069.19075400
SRR17520069.19075401
SRR17520069.19075402
SRR17520069.19075403
SRR17520069.19075404
SRR17520069.19075405
SRR17520069.19075406
SRR17520069.19075407
SRR17520069.19075408
SRR17520069.19075409
SRR17520069.19075410
SRR17520069.19075411
SRR17520069.19075412
SRR17520069.19075413
SRR17520069.19075414
SRR17520069.19075415
SRR17520069.19075416
SRR17520069.19075417
SRR17520069.19075418
SRR17520069.19075419
SRR17520069.19075420
SRR17520069.19075421
SRR17520069.19080927
SRR17520069.19092624
SRR17520069.19092646
SRR17520069.19100242
SRR17520069.19100243
SRR17520069.19100244
SRR17520069.19100245
SRR17520069.19100246
SRR17520069.19100247
SRR17520069.19100248
SRR17520069.19100249
SRR17520069.19100250
SRR17520069.19100251
SRR17520069.19100252
SRR17520069.19100253
SRR17520069.19100254
SRR17520069.19100255
SRR17520069.19100256
SRR17520069.19100257
SRR17520069.19100258
SRR17520069.19100259

SRR17520069.19100260
SRR17520069.19100261
SRR17520069.19100262
SRR17520069.19100263
SRR17520069.19100264
SRR17520069.19100265
SRR17520069.19100267
SRR17520069.19100268
SRR17520069.19100269
SRR17520069.19100270
SRR17520069.19103737
SRR17520069.19103762
SRR17520069.19103765
SRR17520069.19105557
SRR17520069.19105558
SRR17520069.19105559
SRR17520069.19105560
SRR17520069.19105561
SRR17520069.19105562
SRR17520069.19105563
SRR17520069.19105564
SRR17520069.19105565
SRR17520069.19105566
SRR17520069.19105567
SRR17520069.19105568
SRR17520069.19105569
SRR17520069.19105570
SRR17520069.19105571
SRR17520069.19105572
SRR17520069.19105573
SRR17520069.19105574
SRR17520069.19105575
SRR17520069.19105576
SRR17520069.19105577
SRR17520069.19105578
SRR17520069.19105579
SRR17520069.19105580
SRR17520069.19105581
SRR17520069.19105582
SRR17520069.19105583
SRR17520069.19105584
SRR17520069.19105585
SRR17520069.19105586
SRR17520069.19105587
SRR17520069.19105588
SRR17520069.19105589
SRR17520069.19105590

SRR17520069.19105591
SRR17520069.19105592
SRR17520069.19105593
SRR17520069.19105594
SRR17520069.19105595
SRR17520069.19105596
SRR17520069.19105597
SRR17520069.19105598
SRR17520069.19105599
SRR17520069.19105600
SRR17520069.19105601
SRR17520069.19105602
SRR17520069.19105603
SRR17520069.19105604
SRR17520069.19105605
SRR17520069.19105606
SRR17520069.19105607
SRR17520069.19105608
SRR17520069.19105609
SRR17520069.19105610
SRR17520069.19105611
SRR17520069.19105612
SRR17520069.19105613
SRR17520069.19105614
SRR17520069.19105615
SRR17520069.19105616
SRR17520069.19105617
SRR17520069.19105618
SRR17520069.19105619
SRR17520069.19105620
SRR17520069.19105621
SRR17520069.19105622
SRR17520069.19105623
SRR17520069.19105624
SRR17520069.19105625
SRR17520069.19105626
SRR17520069.19105627
SRR17520069.19105628
SRR17520069.19105629
SRR17520069.19105630
SRR17520069.19105631
SRR17520069.19105632
SRR17520069.19105633
SRR17520069.19105634
SRR17520069.19105635
SRR17520069.19105636
SRR17520069.19105637

SRR17520069.19105638
SRR17520069.19105639
SRR17520069.19105640
SRR17520069.19105641
SRR17520069.19105642
SRR17520069.19105643
SRR17520069.19105644
SRR17520069.19105645
SRR17520069.19105646
SRR17520069.19105647
SRR17520069.19105648
SRR17520069.19105649
SRR17520069.19105650
SRR17520069.19105651
SRR17520069.19105652
SRR17520069.19105653
SRR17520069.19105654
SRR17520069.19105655
SRR17520069.19105656
SRR17520069.19105657
SRR17520069.19105658
SRR17520069.19105659
SRR17520069.19105660
SRR17520069.19105661
SRR17520069.19105662
SRR17520069.19105663
SRR17520069.19105664
SRR17520069.19105665
SRR17520069.19105666
SRR17520069.19105667
SRR17520069.19105668
SRR17520069.19105669
SRR17520069.19105670
SRR17520069.19105671
SRR17520069.19105672
SRR17520069.19105673
SRR17520069.19105674
SRR17520069.19105675
SRR17520069.19105676
SRR17520069.19105677
SRR17520069.19105678
SRR17520069.19105679
SRR17520069.19105680
SRR17520069.19105681
SRR17520069.19105682
SRR17520069.19105683
SRR17520069.19105684

SRR17520069.19105685
SRR17520069.19105686
SRR17520069.19105687
SRR17520069.19105688
SRR17520069.19105689
SRR17520069.19105690
SRR17520069.19105691
SRR17520069.19105692
SRR17520069.19105693
SRR17520069.19105694
SRR17520069.19105695
SRR17520069.19105696
SRR17520069.19105697
SRR17520069.19105698
SRR17520069.19105699
SRR17520069.19105700
SRR17520069.19105701
SRR17520069.19105702
SRR17520069.19105703
SRR17520069.19105704
SRR17520069.19105705
SRR17520069.19105706
SRR17520069.19105707
SRR17520069.19132864
SRR17520069.19132865
SRR17520069.19132866
SRR17520069.19132867
SRR17520069.19132868
SRR17520069.19132870
SRR17520069.19132872
SRR17520069.19132873
SRR17520069.19132874
SRR17520069.19132875
SRR17520069.19132876
SRR17520069.19132877
SRR17520069.19132878
SRR17520069.19132879
SRR17520069.19132880
SRR17520069.19132881
SRR17520069.19132882
SRR17520069.19144357
SRR17520069.19144358
SRR17520069.19144359
SRR17520069.19144360
SRR17520069.19144361
SRR17520069.19144363
SRR17520069.19144364

SRR17520069.19144365
SRR17520069.19144367
SRR17520069.19144369
SRR17520069.19144371
SRR17520069.19152507
SRR17520069.19152508
SRR17520069.19152509
SRR17520069.19152510
SRR17520069.19152511
SRR17520069.19152512
SRR17520069.19152513
SRR17520069.19152514
SRR17520069.19152515
SRR17520069.19152516
SRR17520069.19152517
SRR17520069.19152518
SRR17520069.19152519
SRR17520069.19152520
SRR17520069.19152521
SRR17520069.19152522
SRR17520069.19152523
SRR17520069.19152524
SRR17520069.19152525
SRR17520069.19152526
SRR17520069.19152527
SRR17520069.19152528
SRR17520069.19152529
SRR17520069.19152530
SRR17520069.19152531
SRR17520069.19166209
SRR17520069.19166219
SRR17520069.19184854
SRR17520069.19195323
SRR17520069.19195324
SRR17520069.19195325
SRR17520069.19195326
SRR17520069.19195327
SRR17520069.19195328
SRR17520069.19195329
SRR17520069.19195330
SRR17520069.19195331
SRR17520069.19195332
SRR17520069.19195334
SRR17520069.19195335
SRR17520069.19195336
SRR17520069.19195337
SRR17520069.19195338

SRR17520069.19195339
SRR17520069.19195340
SRR17520069.19195341
SRR17520069.19195343
SRR17520069.19195344
SRR17520069.19195345
SRR17520069.19195346
SRR17520069.19195347
SRR17520069.19195348
SRR17520069.19195349
SRR17520069.19195350
SRR17520069.19195351
SRR17520069.19195352
SRR17520069.19195353
SRR17520069.19195354
SRR17520069.19195355
SRR17520069.19195356
SRR17520069.19195357
SRR17520069.19195358
SRR17520069.19195359
SRR17520069.19195360
SRR17520069.19195361
SRR17520069.19195362
SRR17520069.19195363
SRR17520069.19195365
SRR17520069.19195366
SRR17520069.19195367
SRR17520069.19195368
SRR17520069.19195369
SRR17520069.19195370
SRR17520069.19195371
SRR17520069.19195372
SRR17520069.19195373
SRR17520069.19195374
SRR17520069.19195375
SRR17520069.19195376
SRR17520069.19195377
SRR17520069.19195378
SRR17520069.19195379
SRR17520069.19195380
SRR17520069.19195381
SRR17520069.19195382
SRR17520069.19195383
SRR17520069.19195384
SRR17520069.19203916
SRR17520069.19203917
SRR17520069.19203918

SRR17520069.19203919
SRR17520069.19203920
SRR17520069.19203921
SRR17520069.19203922
SRR17520069.19203923
SRR17520069.19203924
SRR17520069.19203925
SRR17520069.19203926
SRR17520069.19203927
SRR17520069.19203928
SRR17520069.19203929
SRR17520069.19203930
SRR17520069.19203932
SRR17520069.19203933
SRR17520069.19203935
SRR17520069.19203936
SRR17520069.19203937
SRR17520069.19203938
SRR17520069.19203939
SRR17520069.19203940
SRR17520069.19203941
SRR17520069.19203942
SRR17520069.19203943
SRR17520069.19203946
SRR17520069.19203947
SRR17520069.19203948
SRR17520069.19203949
SRR17520069.19203950
SRR17520069.19203951
SRR17520069.19203952
SRR17520069.19203953
SRR17520069.19203955
SRR17520069.19203956
SRR17520069.19203957
SRR17520069.19203958
SRR17520069.19208713
SRR17520069.19208714
SRR17520069.19208715
SRR17520069.19208716
SRR17520069.19208717
SRR17520069.19208718
SRR17520069.19208719
SRR17520069.19208720
SRR17520069.19208721
SRR17520069.19208722
SRR17520069.19208723
SRR17520069.19208724

SRR17520069.19208725
SRR17520069.19208726
SRR17520069.19208727
SRR17520069.19208728
SRR17520069.19208729
SRR17520069.19208730
SRR17520069.19208731
SRR17520069.19208732
SRR17520069.19208733
SRR17520069.19208734
SRR17520069.19208735
SRR17520069.19208736
SRR17520069.19208737
SRR17520069.19208738
SRR17520069.19208739
SRR17520069.19208740
SRR17520069.19208741
SRR17520069.19208742
SRR17520069.19208743
SRR17520069.19208744
SRR17520069.19208745
SRR17520069.19208746
SRR17520069.19208747
SRR17520069.19208748
SRR17520069.19208749
SRR17520069.19208750
SRR17520069.19208751
SRR17520069.19208752
SRR17520069.19208753
SRR17520069.19208754
SRR17520069.19208755
SRR17520069.19208756
SRR17520069.19208757
SRR17520069.19240420
SRR17520069.19240421
SRR17520069.19240422
SRR17520069.19240423
SRR17520069.19240424
SRR17520069.19240425
SRR17520069.19240426
SRR17520069.19240427
SRR17520069.19240428
SRR17520069.19240429
SRR17520069.19240430
SRR17520069.19240431
SRR17520069.19240432
SRR17520069.19240433

SRR17520069.19240434
SRR17520069.19240435
SRR17520069.19240436
SRR17520069.19240437
SRR17520069.19240438
SRR17520069.19240439
SRR17520069.19240440
SRR17520069.19240441
SRR17520069.19240442
SRR17520069.19240443
SRR17520069.19240444
SRR17520069.19240445
SRR17520069.19240446
SRR17520069.19240447
SRR17520069.19240448
SRR17520069.19240449
SRR17520069.19240450
SRR17520069.19240451
SRR17520069.19240452
SRR17520069.19240453
SRR17520069.19240454
SRR17520069.19240455
SRR17520069.19240456
SRR17520069.19240457
SRR17520069.19240458
SRR17520069.19240459
SRR17520069.19240460
SRR17520069.19240461
SRR17520069.19240462
SRR17520069.19240463
SRR17520069.19240464
SRR17520069.19240465
SRR17520069.19240466
SRR17520069.19240467
SRR17520069.19240468
SRR17520069.19240469
SRR17520069.19240470
SRR17520069.19240471
SRR17520069.19240472
SRR17520069.19240473
SRR17520069.19240474
SRR17520069.19240475
SRR17520069.19240476
SRR17520069.19240477
SRR17520069.19240478
SRR17520069.19240479
SRR17520069.19240480

SRR17520069.19240481
SRR17520069.19247080
SRR17520069.19252339
SRR17520069.19252340
SRR17520069.19252341
SRR17520069.19252342
SRR17520069.19252343
SRR17520069.19252344
SRR17520069.19252345
SRR17520069.19252346
SRR17520069.19252347
SRR17520069.19252348
SRR17520069.19252349
SRR17520069.19252350
SRR17520069.19252351
SRR17520069.19252352
SRR17520069.19252353
SRR17520069.19252354
SRR17520069.19252355
SRR17520069.19252356
SRR17520069.19252357
SRR17520069.19252358
SRR17520069.19252359
SRR17520069.19252360
SRR17520069.19252361
SRR17520069.19252362
SRR17520069.19252363
SRR17520069.19252364
SRR17520069.19252365
SRR17520069.19252366
SRR17520069.19252367
SRR17520069.19264471
SRR17520069.19298111
SRR17520069.19298112
SRR17520069.19298113
SRR17520069.19298114
SRR17520069.19298115
SRR17520069.19298116
SRR17520069.19298117
SRR17520069.19298118
SRR17520069.19298119
SRR17520069.19300463
SRR17520069.19300464
SRR17520069.19300465
SRR17520069.19300466
SRR17520069.19300467
SRR17520069.19300468

SRR17520069.19300469
SRR17520069.19300470
SRR17520069.19300471
SRR17520069.19300472
SRR17520069.19300473
SRR17520069.19300474
SRR17520069.19300475
SRR17520069.19300476
SRR17520069.19300477
SRR17520069.19300478
SRR17520069.19300479
SRR17520069.19300480
SRR17520069.19300481
SRR17520069.19300482
SRR17520069.19300483
SRR17520069.19300484
SRR17520069.19300485
SRR17520069.19300486
SRR17520069.19300487
SRR17520069.19300488
SRR17520069.19300489
SRR17520069.19300490
SRR17520069.19300491
SRR17520069.19300492
SRR17520069.19300494
SRR17520069.19300495
SRR17520069.19300496
SRR17520069.19300497
SRR17520069.19300498
SRR17520069.19300499
SRR17520069.19300500
SRR17520069.19300501
SRR17520069.19300502
SRR17520069.19300504
SRR17520069.19300505
SRR17520069.19300506
SRR17520069.19300507
SRR17520069.19300508
SRR17520069.19300509
SRR17520069.19300510
SRR17520069.19300511
SRR17520069.19300512
SRR17520069.19300513
SRR17520069.19300514
SRR17520069.19300515
SRR17520069.19300516
SRR17520069.19300517

SRR17520069.19300518
SRR17520069.19300519
SRR17520069.19300520
SRR17520069.19300521
SRR17520069.19300522
SRR17520069.19300523
SRR17520069.19300524
SRR17520069.19300525
SRR17520069.19300526
SRR17520069.19300527
SRR17520069.19300528
SRR17520069.19300529
SRR17520069.19300530
SRR17520069.19300531
SRR17520069.19300532
SRR17520069.19300533
SRR17520069.19300534
SRR17520069.19308821
SRR17520069.19308822
SRR17520069.19308823
SRR17520069.19308824
SRR17520069.19308825
SRR17520069.19308826
SRR17520069.19308827
SRR17520069.19308828
SRR17520069.19308829
SRR17520069.19308830
SRR17520069.19308831
SRR17520069.19308832
SRR17520069.19308834
SRR17520069.19308835
SRR17520069.19308836
SRR17520069.19308837
SRR17520069.19308838
SRR17520069.19308839
SRR17520069.19308840
SRR17520069.19308841
SRR17520069.19311461
SRR17520069.19311462
SRR17520069.19311463
SRR17520069.19311464
SRR17520069.19311465
SRR17520069.19311466
SRR17520069.19311467
SRR17520069.19311468
SRR17520069.19311469
SRR17520069.19311470

SRR17520069.19311471
SRR17520069.19311472
SRR17520069.19311473
SRR17520069.19311474
SRR17520069.19311475
SRR17520069.19311476
SRR17520069.19311477
SRR17520069.19311478
SRR17520069.19311479
SRR17520069.19311480
SRR17520069.19311481
SRR17520069.19311482
SRR17520069.19311483
SRR17520069.19311484
SRR17520069.19311485
SRR17520069.19311486
SRR17520069.19311488
SRR17520069.19311489
SRR17520069.19311491
SRR17520069.19311492
SRR17520069.19311493
SRR17520069.19311494
SRR17520069.19311495
SRR17520069.19311496
SRR17520069.19312877
SRR17520069.19312878
SRR17520069.19312879
SRR17520069.19312880
SRR17520069.19312881
SRR17520069.19312882
SRR17520069.19312883
SRR17520069.19312884
SRR17520069.19312885
SRR17520069.19312886
SRR17520069.19312887
SRR17520069.19317078
SRR17520069.19317079
SRR17520069.19317080
SRR17520069.19317081
SRR17520069.19317082
SRR17520069.19317083
SRR17520069.19317084
SRR17520069.19317085
SRR17520069.19317086
SRR17520069.19317087
SRR17520069.19317088
SRR17520069.19317089

SRR17520069.19317090
SRR17520069.19317091
SRR17520069.19317092
SRR17520069.19317093
SRR17520069.19317094
SRR17520069.19317095
SRR17520069.19317096
SRR17520069.19317097
SRR17520069.19317098
SRR17520069.19317099
SRR17520069.19317100
SRR17520069.19317101
SRR17520069.19317102
SRR17520069.19317103
SRR17520069.19317104
SRR17520069.19317105
SRR17520069.19317106
SRR17520069.19317107
SRR17520069.19317108
SRR17520069.19317109
SRR17520069.19317110
SRR17520069.19317111
SRR17520069.19317112
SRR17520069.19317113
SRR17520069.19317114
SRR17520069.19317115
SRR17520069.19317116
SRR17520069.19317117
SRR17520069.19317118
SRR17520069.19317119
SRR17520069.19317120
SRR17520069.19317121
SRR17520069.19317122
SRR17520069.19317123
SRR17520069.19317124
SRR17520069.19317125
SRR17520069.19317126
SRR17520069.19317127
SRR17520069.19317128
SRR17520069.19317129
SRR17520069.19317130
SRR17520069.19317131
SRR17520069.19317132
SRR17520069.19317133
SRR17520069.19317135
SRR17520069.19317136
SRR17520069.19317137

SRR17520069.19317138
SRR17520069.19317139
SRR17520069.19319607
SRR17520069.19319608
SRR17520069.19319609
SRR17520069.19319610
SRR17520069.19319611
SRR17520069.19319612
SRR17520069.19319613
SRR17520069.19319614
SRR17520069.19319615
SRR17520069.19319616
SRR17520069.19319617
SRR17520069.19319618
SRR17520069.19319619
SRR17520069.19319620
SRR17520069.19319621
SRR17520069.19319622
SRR17520069.19319623
SRR17520069.19325896
SRR17520069.19325897
SRR17520069.19325898
SRR17520069.19325899
SRR17520069.19325900
SRR17520069.19325901
SRR17520069.19325902
SRR17520069.19325903
SRR17520069.19325904
SRR17520069.19325905
SRR17520069.19325906
SRR17520069.19325907
SRR17520069.19325908
SRR17520069.19325909
SRR17520069.19325910
SRR17520069.19325911
SRR17520069.19325912
SRR17520069.19325913
SRR17520069.19325914
SRR17520069.19325915
SRR17520069.19325916
SRR17520069.19325917
SRR17520069.19325918
SRR17520069.19325920
SRR17520069.19325921
SRR17520069.19325922
SRR17520069.19325923
SRR17520069.19325924

SRR17520069.19325925
SRR17520069.19325926
SRR17520069.19325927
SRR17520069.19325928
SRR17520069.19325929
SRR17520069.19325930
SRR17520069.19325931
SRR17520069.19325932
SRR17520069.19325933
SRR17520069.19325934
SRR17520069.19325935
SRR17520069.19325936
SRR17520069.19325937
SRR17520069.19325938
SRR17520069.19325939
SRR17520069.19325940
SRR17520069.19325941
SRR17520069.19325942
SRR17520069.19325943
SRR17520069.19325944
SRR17520069.19325945
SRR17520069.19325946
SRR17520069.19325947
SRR17520069.19325948
SRR17520069.19325949
SRR17520069.19325950
SRR17520069.19325951
SRR17520069.19325953
SRR17520069.19325954
SRR17520069.19325955
SRR17520069.19325956
SRR17520069.19325957
SRR17520069.19325958
SRR17520069.19325959
SRR17520069.19325960
SRR17520069.19325961
SRR17520069.19325962
SRR17520069.19325963
SRR17520069.19325964
SRR17520069.19325965
SRR17520069.19325966
SRR17520069.19325967
SRR17520069.19325968
SRR17520069.19325969
SRR17520069.19325970
SRR17520069.19325971
SRR17520069.19329410

SRR17520069.19348758
SRR17520069.19348759
SRR17520069.19348760
SRR17520069.19397872
SRR17520069.19397873
SRR17520069.19397874
SRR17520069.19409292
SRR17520069.19409293
SRR17520069.19409294
SRR17520069.19409295
SRR17520069.19409296
SRR17520069.19409297
SRR17520069.19409298
SRR17520069.19409299
SRR17520069.19409300
SRR17520069.19409301
SRR17520069.19409302
SRR17520069.19409303
SRR17520069.19409304
SRR17520069.19409305
SRR17520069.19409306
SRR17520069.19409307
SRR17520069.19409308
SRR17520069.19409309
SRR17520069.19409310
SRR17520069.19409311
SRR17520069.19409312
SRR17520069.19409313
SRR17520069.19409314
SRR17520069.19409315
SRR17520069.19409316
SRR17520069.19409317
SRR17520069.19409318
SRR17520069.19409320
SRR17520069.19409321
SRR17520069.19409323
SRR17520069.19409324
SRR17520069.19409325
SRR17520069.19409326
SRR17520069.19409327
SRR17520069.19409328
SRR17520069.19409329
SRR17520069.19409330
SRR17520069.19409331
SRR17520069.19409332
SRR17520069.19412768
SRR17520069.19412769

SRR17520069.19412770
SRR17520069.19412771
SRR17520069.19412772
SRR17520069.19412773
SRR17520069.19412774
SRR17520069.19412775
SRR17520069.19412776
SRR17520069.19412777
SRR17520069.19412778
SRR17520069.19412779
SRR17520069.19412780
SRR17520069.19412781
SRR17520069.19412782
SRR17520069.19412783
SRR17520069.19412784
SRR17520069.19412785
SRR17520069.19412786
SRR17520069.19412787
SRR17520069.19412788
SRR17520069.19412789
SRR17520069.19412790
SRR17520069.19412791
SRR17520069.19412792
SRR17520069.19412793
SRR17520069.19412794
SRR17520069.19412795
SRR17520069.19412796
SRR17520069.19412797
SRR17520069.19412798
SRR17520069.19412799
SRR17520069.19412800
SRR17520069.19412801
SRR17520069.19412802
SRR17520069.19412803
SRR17520069.19412804
SRR17520069.19412805
SRR17520069.19412806
SRR17520069.19412807
SRR17520069.19412808
SRR17520069.19412809
SRR17520069.19412810
SRR17520069.19412811
SRR17520069.19412812
SRR17520069.19412813
SRR17520069.19412814
SRR17520069.19412815
SRR17520069.19412816

SRR17520069.19412817
SRR17520069.19412818
SRR17520069.19412819
SRR17520069.19412820
SRR17520069.19412821
SRR17520069.19412822
SRR17520069.19412823
SRR17520069.19412824
SRR17520069.19412825
SRR17520069.19412826
SRR17520069.19412827
SRR17520069.19412828
SRR17520069.19412829
SRR17520069.19412830
SRR17520069.19412831
SRR17520069.19412833
SRR17520069.19429311
SRR17520069.19469901
SRR17520069.19469902
SRR17520069.19469903
SRR17520069.19469904
SRR17520069.19469905
SRR17520069.19469906
SRR17520069.19469907
SRR17520069.19469908
SRR17520069.19469909
SRR17520069.19469910
SRR17520069.19469911
SRR17520069.19469912
SRR17520069.19469913
SRR17520069.19469914
SRR17520069.19475541
SRR17520069.19475542
SRR17520069.19475543
SRR17520069.19475544
SRR17520069.19475545
SRR17520069.19475546
SRR17520069.19475547
SRR17520069.19475548
SRR17520069.19475549
SRR17520069.19475550
SRR17520069.19475551
SRR17520069.19475552
SRR17520069.19475553
SRR17520069.19475554
SRR17520069.19475555
SRR17520069.19475556

SRR17520069.19475557
SRR17520069.19475558
SRR17520069.19475559
SRR17520069.19475560
SRR17520069.19475561
SRR17520069.19475562
SRR17520069.19475563
SRR17520069.19475564
SRR17520069.19475565
SRR17520069.19475566
SRR17520069.19475567
SRR17520069.19475568
SRR17520069.19475569
SRR17520069.19475570
SRR17520069.19475571
SRR17520069.19475572
SRR17520069.19475573
SRR17520069.19475574
SRR17520069.19475575
SRR17520069.19475576
SRR17520069.19475577
SRR17520069.19475578
SRR17520069.19475579
SRR17520069.19475580
SRR17520069.19475581
SRR17520069.19475582
SRR17520069.19475583
SRR17520069.19475584
SRR17520069.19486128
SRR17520069.19486129
SRR17520069.19486131
SRR17520069.19503478
SRR17520069.19503479
SRR17520069.19503480
SRR17520069.19503481
SRR17520069.19503483
SRR17520069.19503484
SRR17520069.19503485
SRR17520069.19503486
SRR17520069.19503487
SRR17520069.19503488
SRR17520069.19503489
SRR17520069.19503490
SRR17520069.19503491
SRR17520069.19503492
SRR17520069.19503493
SRR17520069.19503494

SRR17520069.19503495
SRR17520069.19503496
SRR17520069.19503497
SRR17520069.19503498
SRR17520069.19503499
SRR17520069.19503500
SRR17520069.19503501
SRR17520069.19503502
SRR17520069.19503503
SRR17520069.19503504
SRR17520069.19503505
SRR17520069.19503506
SRR17520069.19503507
SRR17520069.19503508
SRR17520069.19503509
SRR17520069.19503510
SRR17520069.19503511
SRR17520069.19503512
SRR17520069.19503513
SRR17520069.19503514
SRR17520069.19503515
SRR17520069.19503516
SRR17520069.19503518
SRR17520069.19503519
SRR17520069.19503520
SRR17520069.19503521
SRR17520069.19503522
SRR17520069.19503523
SRR17520069.19503524
SRR17520069.19503525
SRR17520069.19503526
SRR17520069.19503527
SRR17520069.19503528
SRR17520069.19503529
SRR17520069.19503530
SRR17520069.19503531
SRR17520069.19503532
SRR17520069.19503533
SRR17520069.19503534
SRR17520069.19503535
SRR17520069.19503536
SRR17520069.19503537
SRR17520069.19503538
SRR17520069.19503539
SRR17520069.19503540
SRR17520069.19503541
SRR17520069.19503542

SRR17520069.19503543
SRR17520069.19503544
SRR17520069.19503545
SRR17520069.19503546
SRR17520069.19503547
SRR17520069.19503548
SRR17520069.19503549
SRR17520069.19503550
SRR17520069.19503551
SRR17520069.19503552
SRR17520069.19503553
SRR17520069.19503554
SRR17520069.19503555
SRR17520069.19503556
SRR17520069.19503557
SRR17520069.19503559
SRR17520069.19503560
SRR17520069.19503561
SRR17520069.19503562
SRR17520069.19503563
SRR17520069.19503564
SRR17520069.19503565
SRR17520069.19503566
SRR17520069.19506904
SRR17520069.19506905
SRR17520069.19506906
SRR17520069.19506907
SRR17520069.19506908
SRR17520069.19506910
SRR17520069.19506911
SRR17520069.19506912
SRR17520069.19506913
SRR17520069.19506914
SRR17520069.19506915
SRR17520069.19506916
SRR17520069.19506917
SRR17520069.19506918
SRR17520069.19506919
SRR17520069.19506920
SRR17520069.19506921
SRR17520069.19506922
SRR17520069.19506923
SRR17520069.19506924
SRR17520069.19506925
SRR17520069.19506926
SRR17520069.19506927
SRR17520069.19506928

SRR17520069.19506929
SRR17520069.19506930
SRR17520069.19506931
SRR17520069.19506932
SRR17520069.19506933
SRR17520069.19506934
SRR17520069.19506935
SRR17520069.19506937
SRR17520069.19506938
SRR17520069.19506939
SRR17520069.19506940
SRR17520069.19506941
SRR17520069.19506942
SRR17520069.19506943
SRR17520069.19506944
SRR17520069.19506945
SRR17520069.19506946
SRR17520069.19506947
SRR17520069.19506948
SRR17520069.19506949
SRR17520069.19506950
SRR17520069.19506951
SRR17520069.19510737
SRR17520069.19510738
SRR17520069.19510739
SRR17520069.19510740
SRR17520069.19510741
SRR17520069.19510742
SRR17520069.19510743
SRR17520069.19510744
SRR17520069.19510745
SRR17520069.19510746
SRR17520069.19510747
SRR17520069.19510748
SRR17520069.19510749
SRR17520069.19510750
SRR17520069.19510751
SRR17520069.19510752
SRR17520069.19510753
SRR17520069.19510754
SRR17520069.19510755
SRR17520069.19510756
SRR17520069.19510757
SRR17520069.19510758
SRR17520069.19510759
SRR17520069.19510760
SRR17520069.19510761

SRR17520069.19510762
SRR17520069.19510763
SRR17520069.19510764
SRR17520069.19510765
SRR17520069.19510766
SRR17520069.19510767
SRR17520069.19510768
SRR17520069.19510769
SRR17520069.19510770
SRR17520069.19510771
SRR17520069.19510772
SRR17520069.19510773
SRR17520069.19510774
SRR17520069.19510775
SRR17520069.19510776
SRR17520069.19510777
SRR17520069.19510778
SRR17520069.19510779
SRR17520069.19510780
SRR17520069.19510781
SRR17520069.19510782
SRR17520069.19517107
SRR17520069.19517108
SRR17520069.19517109
SRR17520069.19517110
SRR17520069.19517111
SRR17520069.19517112
SRR17520069.19517113
SRR17520069.19517114
SRR17520069.19517115
SRR17520069.19517116
SRR17520069.19517117
SRR17520069.19517118
SRR17520069.19528914
SRR17520069.19548965
SRR17520069.19557903
SRR17520069.19557904
SRR17520069.19557905
SRR17520069.19557906
SRR17520069.19557907
SRR17520069.19557908
SRR17520069.19557909
SRR17520069.19557910
SRR17520069.19557911
SRR17520069.19557912
SRR17520069.19557913
SRR17520069.19557914

SRR17520069.19557915
SRR17520069.19557916
SRR17520069.19557917
SRR17520069.19557918
SRR17520069.19557919
SRR17520069.19557920
SRR17520069.19557921
SRR17520069.19557922
SRR17520069.19557923
SRR17520069.19557924
SRR17520069.19557925
SRR17520069.19557926
SRR17520069.19557927
SRR17520069.19557928
SRR17520069.19557929
SRR17520069.19557930
SRR17520069.19557931
SRR17520069.19557932
SRR17520069.19557933
SRR17520069.19557934
SRR17520069.19557935
SRR17520069.19557936
SRR17520069.19557937
SRR17520069.19557938
SRR17520069.19557939
SRR17520069.19557940
SRR17520069.19557941
SRR17520069.19557942
SRR17520069.19557943
SRR17520069.19557944
SRR17520069.19557945
SRR17520069.19557946
SRR17520069.19557947
SRR17520069.19557948
SRR17520069.19557949
SRR17520069.19557950
SRR17520069.19557951
SRR17520069.19557952
SRR17520069.19557953
SRR17520069.19557954
SRR17520069.19557955
SRR17520069.19557956
SRR17520069.19557957
SRR17520069.19557958
SRR17520069.19557959
SRR17520069.19557960
SRR17520069.19557961

SRR17520069.19557962
SRR17520069.19557963
SRR17520069.19557964
SRR17520069.19557965
SRR17520069.19557966
SRR17520069.19557967
SRR17520069.19557968
SRR17520069.7652
SRR17520069.23562
SRR17520069.29604
SRR17520069.29606
SRR17520069.53776
SRR17520069.65024
SRR17520069.65025
SRR17520069.65026
SRR17520069.65027
SRR17520069.65028
SRR17520069.120847
SRR17520069.120848
SRR17520069.120849
SRR17520069.120850
SRR17520069.120851
SRR17520069.120852
SRR17520069.120853
SRR17520069.120854
SRR17520069.120855
SRR17520069.120856
SRR17520069.123341
SRR17520069.131677
SRR17520069.145018
SRR17520069.145453
SRR17520069.145598
SRR17520069.155813
SRR17520069.192497
SRR17520069.192506
SRR17520069.194010
SRR17520069.202090
SRR17520069.214407
SRR17520069.214408
SRR17520069.214409
SRR17520069.214410
SRR17520069.214411
SRR17520069.214412
SRR17520069.214413
SRR17520069.214414
SRR17520069.214415
SRR17520069.214416

SRR17520069.214417
SRR17520069.214418
SRR17520069.214419
SRR17520069.214420
SRR17520069.214421
SRR17520069.214422
SRR17520069.214423
SRR17520069.214424
SRR17520069.214425
SRR17520069.214426
SRR17520069.214427
SRR17520069.214428
SRR17520069.214429
SRR17520069.214430
SRR17520069.214431
SRR17520069.214432
SRR17520069.214433
SRR17520069.214434
SRR17520069.214435
SRR17520069.214436
SRR17520069.214437
SRR17520069.214438
SRR17520069.214439
SRR17520069.214440
SRR17520069.214441
SRR17520069.214442
SRR17520069.214443
SRR17520069.214444
SRR17520069.214445
SRR17520069.214446
SRR17520069.214447
SRR17520069.214448
SRR17520069.214449
SRR17520069.214450
SRR17520069.214451
SRR17520069.214452
SRR17520069.214453
SRR17520069.214454
SRR17520069.214455
SRR17520069.214456
SRR17520069.214457
SRR17520069.214458
SRR17520069.214459
SRR17520069.214460
SRR17520069.214461
SRR17520069.214463
SRR17520069.224657

SRR17520069.224663
SRR17520069.263630
SRR17520069.265016
SRR17520069.274694
SRR17520069.293510
SRR17520069.299517
SRR17520069.311496
SRR17520069.311607
SRR17520069.366694
SRR17520069.366859
SRR17520069.366940
SRR17520069.433591
SRR17520069.450414
SRR17520069.491007
SRR17520069.491008
SRR17520069.491009
SRR17520069.491010
SRR17520069.491011
SRR17520069.491012
SRR17520069.491014
SRR17520069.491015
SRR17520069.491016
SRR17520069.491017
SRR17520069.491018
SRR17520069.491019
SRR17520069.491020
SRR17520069.491021
SRR17520069.491022
SRR17520069.491023
SRR17520069.491024
SRR17520069.491025
SRR17520069.491027
SRR17520069.491028
SRR17520069.491029
SRR17520069.491030
SRR17520069.491031
SRR17520069.491032
SRR17520069.491033
SRR17520069.491034
SRR17520069.491035
SRR17520069.491036
SRR17520069.491038
SRR17520069.491039
SRR17520069.491040
SRR17520069.491041
SRR17520069.491042
SRR17520069.491044

SRR17520069.491045
SRR17520069.491046
SRR17520069.491047
SRR17520069.491048
SRR17520069.491049
SRR17520069.491050
SRR17520069.491051
SRR17520069.491052
SRR17520069.491054
SRR17520069.491055
SRR17520069.491056
SRR17520069.491057
SRR17520069.491058
SRR17520069.501544
SRR17520069.540249
SRR17520069.544208
SRR17520069.572425
SRR17520069.610118
SRR17520069.624556
SRR17520069.624634
SRR17520069.630651
SRR17520069.644582
SRR17520069.649609
SRR17520069.649610
SRR17520069.663467
SRR17520069.666791
SRR17520069.688744
SRR17520069.688745
SRR17520069.688746
SRR17520069.688750
SRR17520069.688751
SRR17520069.688752
SRR17520069.688753
SRR17520069.688754
SRR17520069.688755
SRR17520069.688756
SRR17520069.688757
SRR17520069.688758
SRR17520069.688759
SRR17520069.688760
SRR17520069.688761
SRR17520069.688762
SRR17520069.688763
SRR17520069.688764
SRR17520069.688765
SRR17520069.688766
SRR17520069.688767

SRR17520069.688768
SRR17520069.688769
SRR17520069.688770
SRR17520069.688771
SRR17520069.688772
SRR17520069.688773
SRR17520069.688774
SRR17520069.688775
SRR17520069.688776
SRR17520069.688777
SRR17520069.688778
SRR17520069.688779
SRR17520069.688780
SRR17520069.688781
SRR17520069.688782
SRR17520069.688783
SRR17520069.688784
SRR17520069.688785
SRR17520069.688786
SRR17520069.688787
SRR17520069.688788
SRR17520069.688789
SRR17520069.731660
SRR17520069.733451
SRR17520069.764123
SRR17520069.769577
SRR17520069.772295
SRR17520069.772303
SRR17520069.772310
SRR17520069.772311
SRR17520069.772315
SRR17520069.772319
SRR17520069.772323
SRR17520069.772329
SRR17520069.772343
SRR17520069.772344
SRR17520069.772346
SRR17520069.772353
SRR17520069.772355
SRR17520069.772379
SRR17520069.772406
SRR17520069.772407
SRR17520069.772411
SRR17520069.773992
SRR17520069.798831
SRR17520069.798832
SRR17520069.798833

SRR17520069.816148
SRR17520069.882602
SRR17520069.885870
SRR17520069.885880
SRR17520069.896960
SRR17520069.901945
SRR17520069.902050
SRR17520069.902063
SRR17520069.930437
SRR17520069.931756
SRR17520069.951316
SRR17520069.961085
SRR17520069.963866
SRR17520069.963929
SRR17520069.981112
SRR17520069.1000237
SRR17520069.1022720
SRR17520069.1033156
SRR17520069.1033157
SRR17520069.1033159
SRR17520069.1033160
SRR17520069.1033161
SRR17520069.1033162
SRR17520069.1033163
SRR17520069.1033164
SRR17520069.1033165
SRR17520069.1033166
SRR17520069.1033167
SRR17520069.1033168
SRR17520069.1033169
SRR17520069.1033170
SRR17520069.1033171
SRR17520069.1033172
SRR17520069.1033173
SRR17520069.1033174
SRR17520069.1033175
SRR17520069.1033176
SRR17520069.1033177
SRR17520069.1033178
SRR17520069.1033179
SRR17520069.1033180
SRR17520069.1033181
SRR17520069.1033182
SRR17520069.1033183
SRR17520069.1033184
SRR17520069.1033185
SRR17520069.1033186

SRR17520069.1033187
SRR17520069.1033188
SRR17520069.1033189
SRR17520069.1033190
SRR17520069.1033191
SRR17520069.1033192
SRR17520069.1033193
SRR17520069.1033194
SRR17520069.1033195
SRR17520069.1033196
SRR17520069.1033197
SRR17520069.1033198
SRR17520069.1033199
SRR17520069.1033200
SRR17520069.1033201
SRR17520069.1033202
SRR17520069.1033203
SRR17520069.1033204
SRR17520069.1033205
SRR17520069.1033206
SRR17520069.1033207
SRR17520069.1033208
SRR17520069.1033209
SRR17520069.1033210
SRR17520069.1033211
SRR17520069.1033212
SRR17520069.1033213
SRR17520069.1033214
SRR17520069.1033215
SRR17520069.1033216
SRR17520069.1038239
SRR17520069.1038240
SRR17520069.1038241
SRR17520069.1038242
SRR17520069.1038243
SRR17520069.1038244
SRR17520069.1038245
SRR17520069.1038246
SRR17520069.1038247
SRR17520069.1038248
SRR17520069.1038249
SRR17520069.1038250
SRR17520069.1038251
SRR17520069.1038252
SRR17520069.1038253
SRR17520069.1038254
SRR17520069.1038255

SRR17520069.1038256
SRR17520069.1038257
SRR17520069.1038258
SRR17520069.1038259
SRR17520069.1038260
SRR17520069.1038261
SRR17520069.1038262
SRR17520069.1038263
SRR17520069.1038264
SRR17520069.1038265
SRR17520069.1038266
SRR17520069.1038267
SRR17520069.1038268
SRR17520069.1038269
SRR17520069.1038270
SRR17520069.1038271
SRR17520069.1038272
SRR17520069.1038273
SRR17520069.1038274
SRR17520069.1038275
SRR17520069.1038276
SRR17520069.1038277
SRR17520069.1038278
SRR17520069.1038279
SRR17520069.1039115
SRR17520069.1044929
SRR17520069.1056827
SRR17520069.1056828
SRR17520069.1056829
SRR17520069.1056830
SRR17520069.1056831
SRR17520069.1056832
SRR17520069.1056833
SRR17520069.1056834
SRR17520069.1056835
SRR17520069.1056836
SRR17520069.1056837
SRR17520069.1056838
SRR17520069.1056839
SRR17520069.1056840
SRR17520069.1056841
SRR17520069.1056842
SRR17520069.1056843
SRR17520069.1056844
SRR17520069.1056845
SRR17520069.1056846
SRR17520069.1056847

SRR17520069.1056849
SRR17520069.1056850
SRR17520069.1056851
SRR17520069.1056852
SRR17520069.1056853
SRR17520069.1056854
SRR17520069.1056855
SRR17520069.1056856
SRR17520069.1056857
SRR17520069.1056858
SRR17520069.1056859
SRR17520069.1056860
SRR17520069.1056861
SRR17520069.1071943
SRR17520069.1078112
SRR17520069.1110981
SRR17520069.1115975
SRR17520069.1124712
SRR17520069.1185833
SRR17520069.1214758
SRR17520069.1249548
SRR17520069.1259956
SRR17520069.1287593
SRR17520069.1287594
SRR17520069.1287596
SRR17520069.1287597
SRR17520069.1287598
SRR17520069.1287599
SRR17520069.1287600
SRR17520069.1287601
SRR17520069.1287602
SRR17520069.1287603
SRR17520069.1287604
SRR17520069.1287605
SRR17520069.1287606
SRR17520069.1287607
SRR17520069.1287608
SRR17520069.1287609
SRR17520069.1287610
SRR17520069.1287611
SRR17520069.1287612
SRR17520069.1287613
SRR17520069.1287614
SRR17520069.1287615
SRR17520069.1287616
SRR17520069.1287617
SRR17520069.1287618

SRR17520069.1287619
SRR17520069.1287620
SRR17520069.1287621
SRR17520069.1287622
SRR17520069.1287623
SRR17520069.1287624
SRR17520069.1287625
SRR17520069.1287626
SRR17520069.1287627
SRR17520069.1287628
SRR17520069.1287629
SRR17520069.1287630
SRR17520069.1287631
SRR17520069.1287632
SRR17520069.1287633
SRR17520069.1287634
SRR17520069.1287635
SRR17520069.1287636
SRR17520069.1287637
SRR17520069.1287638
SRR17520069.1287639
SRR17520069.1287640
SRR17520069.1287641
SRR17520069.1287642
SRR17520069.1287643
SRR17520069.1287644
SRR17520069.1287645
SRR17520069.1287646
SRR17520069.1287647
SRR17520069.1287648
SRR17520069.1287649
SRR17520069.1287650
SRR17520069.1287651
SRR17520069.1287652
SRR17520069.1287653
SRR17520069.1287654
SRR17520069.1287655
SRR17520069.1287656
SRR17520069.1287657
SRR17520069.1287658
SRR17520069.1287659
SRR17520069.1288080
SRR17520069.1288094
SRR17520069.1295158
SRR17520069.1295159
SRR17520069.1295160
SRR17520069.1295161

SRR17520069.1295162
SRR17520069.1295163
SRR17520069.1295164
SRR17520069.1295165
SRR17520069.1295166
SRR17520069.1295167
SRR17520069.1295168
SRR17520069.1295169
SRR17520069.1295170
SRR17520069.1295171
SRR17520069.1295172
SRR17520069.1295173
SRR17520069.1295174
SRR17520069.1295175
SRR17520069.1295176
SRR17520069.1295177
SRR17520069.1295178
SRR17520069.1295179
SRR17520069.1295180
SRR17520069.1295181
SRR17520069.1295182
SRR17520069.1295183
SRR17520069.1295184
SRR17520069.1295185
SRR17520069.1295186
SRR17520069.1295187
SRR17520069.1295188
SRR17520069.1296452
SRR17520069.1299062
SRR17520069.1319114
SRR17520069.1319115
SRR17520069.1319116
SRR17520069.1319117
SRR17520069.1319118
SRR17520069.1319119
SRR17520069.1319120
SRR17520069.1319121
SRR17520069.1319122
SRR17520069.1319123
SRR17520069.1319124
SRR17520069.1319125
SRR17520069.1319126
SRR17520069.1319127
SRR17520069.1319128
SRR17520069.1319129
SRR17520069.1319130
SRR17520069.1319131

SRR17520069.1319132
SRR17520069.1319133
SRR17520069.1319134
SRR17520069.1319135
SRR17520069.1319136
SRR17520069.1319137
SRR17520069.1319138
SRR17520069.1319139
SRR17520069.1319140
SRR17520069.1319141
SRR17520069.1319142
SRR17520069.1319143
SRR17520069.1319144
SRR17520069.1319145
SRR17520069.1319146
SRR17520069.1319147
SRR17520069.1319148
SRR17520069.1319149
SRR17520069.1319150
SRR17520069.1319151
SRR17520069.1319152
SRR17520069.1319153
SRR17520069.1319154
SRR17520069.1319155
SRR17520069.1319156
SRR17520069.1319157
SRR17520069.1319158
SRR17520069.1319159
SRR17520069.1319160
SRR17520069.1319161
SRR17520069.1319162
SRR17520069.1319163
SRR17520069.1319165
SRR17520069.1319166
SRR17520069.1319167
SRR17520069.1319168
SRR17520069.1319169
SRR17520069.1319170
SRR17520069.1319171
SRR17520069.1319172
SRR17520069.1319173
SRR17520069.1319174
SRR17520069.1319175
SRR17520069.1319176
SRR17520069.1319177
SRR17520069.1319178
SRR17520069.1319179

SRR17520069.1319180
SRR17520069.1319181
SRR17520069.1319182
SRR17520069.1319183
SRR17520069.1319184
SRR17520069.1319185
SRR17520069.1319186
SRR17520069.1319187
SRR17520069.1319188
SRR17520069.1319189
SRR17520069.1319190
SRR17520069.1319191
SRR17520069.1319192
SRR17520069.1319193
SRR17520069.1319194
SRR17520069.1319195
SRR17520069.1319196
SRR17520069.1319197
SRR17520069.1319198
SRR17520069.1319199
SRR17520069.1319200
SRR17520069.1319201
SRR17520069.1319202
SRR17520069.1319203
SRR17520069.1319204
SRR17520069.1319205
SRR17520069.1319206
SRR17520069.1319207
SRR17520069.1319208
SRR17520069.1319209
SRR17520069.1319210
SRR17520069.1319211
SRR17520069.1319212
SRR17520069.1319213
SRR17520069.1319214
SRR17520069.1319215
SRR17520069.1319216
SRR17520069.1319217
SRR17520069.1319218
SRR17520069.1319219
SRR17520069.1319220
SRR17520069.1319221
SRR17520069.1319222
SRR17520069.1319223
SRR17520069.1319224
SRR17520069.1319225
SRR17520069.1319226

SRR17520069.1319227
SRR17520069.1319228
SRR17520069.1319229
SRR17520069.1319230
SRR17520069.1319231
SRR17520069.1319232
SRR17520069.1319233
SRR17520069.1319234
SRR17520069.1319235
SRR17520069.1319236
SRR17520069.1319237
SRR17520069.1319238
SRR17520069.1319239
SRR17520069.1319240
SRR17520069.1319241
SRR17520069.1319242
SRR17520069.1319243
SRR17520069.1319244
SRR17520069.1319245
SRR17520069.1319246
SRR17520069.1319247
SRR17520069.1319248
SRR17520069.1319249
SRR17520069.1319250
SRR17520069.1319251
SRR17520069.1319252
SRR17520069.1319253
SRR17520069.1319254
SRR17520069.1319255
SRR17520069.1319256
SRR17520069.1319257
SRR17520069.1319258
SRR17520069.1319259
SRR17520069.1319260
SRR17520069.1319261
SRR17520069.1319262
SRR17520069.1319263
SRR17520069.1319264
SRR17520069.1319265
SRR17520069.1319266
SRR17520069.1319267
SRR17520069.1319268
SRR17520069.1319269
SRR17520069.1319270
SRR17520069.1319271
SRR17520069.1319272
SRR17520069.1319273

SRR17520069.1319274
SRR17520069.1319275
SRR17520069.1319276
SRR17520069.1319277
SRR17520069.1319278
SRR17520069.1319279
SRR17520069.1319280
SRR17520069.1319281
SRR17520069.1319282
SRR17520069.1319283
SRR17520069.1319284
SRR17520069.1319285
SRR17520069.1334572
SRR17520069.1352101
SRR17520069.1367242
SRR17520069.1406562
SRR17520069.1406563
SRR17520069.1406564
SRR17520069.1406565
SRR17520069.1406566
SRR17520069.1406567
SRR17520069.1406568
SRR17520069.1406569
SRR17520069.1406570
SRR17520069.1406571
SRR17520069.1406572
SRR17520069.1406573
SRR17520069.1406574
SRR17520069.1406575
SRR17520069.1406576
SRR17520069.1406577
SRR17520069.1406578
SRR17520069.1406579
SRR17520069.1406580
SRR17520069.1406581
SRR17520069.1406582
SRR17520069.1406583
SRR17520069.1406584
SRR17520069.1406585
SRR17520069.1434977
SRR17520069.1434978
SRR17520069.1434979
SRR17520069.1434980
SRR17520069.1434981
SRR17520069.1434982
SRR17520069.1434983
SRR17520069.1434984

SRR17520069.1434986
SRR17520069.1434987
SRR17520069.1434988
SRR17520069.1434989
SRR17520069.1434990
SRR17520069.1434991
SRR17520069.1468537
SRR17520069.1468538
SRR17520069.1468539
SRR17520069.1468540
SRR17520069.1468541
SRR17520069.1468542
SRR17520069.1468543
SRR17520069.1497629
SRR17520069.1497630
SRR17520069.1497631
SRR17520069.1497632
SRR17520069.1497633
SRR17520069.1497634
SRR17520069.1497635
SRR17520069.1497636
SRR17520069.1497637
SRR17520069.1497638
SRR17520069.1497639
SRR17520069.1497640
SRR17520069.1497641
SRR17520069.1497642
SRR17520069.1497643
SRR17520069.1497644
SRR17520069.1497645
SRR17520069.1497646
SRR17520069.1497647
SRR17520069.1497648
SRR17520069.1497649
SRR17520069.1500504
SRR17520069.1506020
SRR17520069.1518860
SRR17520069.1521107
SRR17520069.1521142
SRR17520069.1521147
SRR17520069.1631653
SRR17520069.1631664
SRR17520069.1650537
SRR17520069.1656125
SRR17520069.1658596
SRR17520069.1667955
SRR17520069.1718533

SRR17520069.1766123
SRR17520069.1796514
SRR17520069.1810033
SRR17520069.1831543
SRR17520069.1833455
SRR17520069.1841481
SRR17520069.1847373
SRR17520069.1848588
SRR17520069.1870940
SRR17520069.1879964
SRR17520069.1890047
SRR17520069.1917944
SRR17520069.1921239
SRR17520069.1921241
SRR17520069.1951292
SRR17520069.1951293
SRR17520069.1978397
SRR17520069.1993953
SRR17520069.2015947
SRR17520069.2016957
SRR17520069.2017968
SRR17520069.2018004
SRR17520069.2035423
SRR17520069.2047454
SRR17520069.2047455
SRR17520069.2047456
SRR17520069.2047457
SRR17520069.2047458
SRR17520069.2047459
SRR17520069.2047461
SRR17520069.2047463
SRR17520069.2047464
SRR17520069.2047465
SRR17520069.2047466
SRR17520069.2047467
SRR17520069.2047469
SRR17520069.2047472
SRR17520069.2047473
SRR17520069.2047474
SRR17520069.2047475
SRR17520069.2047477
SRR17520069.2047478
SRR17520069.2073972
SRR17520069.2076562
SRR17520069.2101561
SRR17520069.2106130
SRR17520069.2113409

SRR17520069.2128826
SRR17520069.2148933
SRR17520069.2188680
SRR17520069.2209524
SRR17520069.2209525
SRR17520069.2209526
SRR17520069.2209527
SRR17520069.2209528
SRR17520069.2209529
SRR17520069.2209530
SRR17520069.2209531
SRR17520069.2209532
SRR17520069.2255347
SRR17520069.2255348
SRR17520069.2255349
SRR17520069.2255350
SRR17520069.2255351
SRR17520069.2255352
SRR17520069.2255353
SRR17520069.2255354
SRR17520069.2255355
SRR17520069.2255356
SRR17520069.2255357
SRR17520069.2255358
SRR17520069.2255359
SRR17520069.2255361
SRR17520069.2255362
SRR17520069.2262168
SRR17520069.2305255
SRR17520069.2363327
SRR17520069.2366370
SRR17520069.2402430
SRR17520069.2424518
SRR17520069.2435280
SRR17520069.2438444
SRR17520069.2463282
SRR17520069.2464943
SRR17520069.2478027
SRR17520069.2478028
SRR17520069.2478029
SRR17520069.2478030
SRR17520069.2478031
SRR17520069.2478032
SRR17520069.2478033
SRR17520069.2478034
SRR17520069.2478035
SRR17520069.2478036

SRR17520069.2478037
SRR17520069.2489180
SRR17520069.2489282
SRR17520069.2494986
SRR17520069.2495091
SRR17520069.2495093
SRR17520069.2514583
SRR17520069.2545385
SRR17520069.2545386
SRR17520069.2545387
SRR17520069.2545388
SRR17520069.2545389
SRR17520069.2545390
SRR17520069.2545391
SRR17520069.2545392
SRR17520069.2545393
SRR17520069.2545394
SRR17520069.2545395
SRR17520069.2545396
SRR17520069.2545397
SRR17520069.2545398
SRR17520069.2545399
SRR17520069.2545400
SRR17520069.2545401
SRR17520069.2545402
SRR17520069.2545403
SRR17520069.2545404
SRR17520069.2545405
SRR17520069.2545406
SRR17520069.2545407
SRR17520069.2545408
SRR17520069.2545410
SRR17520069.2545411
SRR17520069.2545412
SRR17520069.2545413
SRR17520069.2545414
SRR17520069.2545415
SRR17520069.2545416
SRR17520069.2545417
SRR17520069.2545418
SRR17520069.2545419
SRR17520069.2545420
SRR17520069.2564234
SRR17520069.2573171
SRR17520069.2573174
SRR17520069.2573208
SRR17520069.2573215

SRR17520069.2597347
SRR17520069.2600638
SRR17520069.2626878
SRR17520069.2636940
SRR17520069.2638930
SRR17520069.2693755
SRR17520069.2719111
SRR17520069.2719113
SRR17520069.2742435
SRR17520069.2748907
SRR17520069.2778839
SRR17520069.2798796
SRR17520069.2804901
SRR17520069.2804902
SRR17520069.2804903
SRR17520069.2804904
SRR17520069.2804905
SRR17520069.2804906
SRR17520069.2804907
SRR17520069.2804908
SRR17520069.2804909
SRR17520069.2804910
SRR17520069.2804911
SRR17520069.2804912
SRR17520069.2804913
SRR17520069.2804914
SRR17520069.2804915
SRR17520069.2804916
SRR17520069.2804917
SRR17520069.2804919
SRR17520069.2804920
SRR17520069.2804921
SRR17520069.2804922
SRR17520069.2804923
SRR17520069.2804924
SRR17520069.2804925
SRR17520069.2804926
SRR17520069.2804927
SRR17520069.2804928
SRR17520069.2804930
SRR17520069.2804931
SRR17520069.2804932
SRR17520069.2804933
SRR17520069.2804934
SRR17520069.2804935
SRR17520069.2804936
SRR17520069.2804937

SRR17520069.2804938
SRR17520069.2804939
SRR17520069.2804940
SRR17520069.2804941
SRR17520069.2804943
SRR17520069.2804945
SRR17520069.2804946
SRR17520069.2804947
SRR17520069.2804948
SRR17520069.2804949
SRR17520069.2908942
SRR17520069.2908954
SRR17520069.2908994
SRR17520069.2914032
SRR17520069.2916847
SRR17520069.2932276
SRR17520069.2953591
SRR17520069.2957133
SRR17520069.2957146
SRR17520069.2965902
SRR17520069.2999865
SRR17520069.3006802
SRR17520069.3006826
SRR17520069.3044151
SRR17520069.3048494
SRR17520069.3048495
SRR17520069.3048496
SRR17520069.3048498
SRR17520069.3048499
SRR17520069.3048500
SRR17520069.3048501
SRR17520069.3048502
SRR17520069.3048503
SRR17520069.3048504
SRR17520069.3048505
SRR17520069.3048506
SRR17520069.3048507
SRR17520069.3048508
SRR17520069.3048509
SRR17520069.3048510
SRR17520069.3048511
SRR17520069.3048512
SRR17520069.3048513
SRR17520069.3082424
SRR17520069.3082426
SRR17520069.3082427
SRR17520069.3082428

SRR17520069.3082429
SRR17520069.3082430
SRR17520069.3082431
SRR17520069.3082432
SRR17520069.3082433
SRR17520069.3082434
SRR17520069.3161361
SRR17520069.3169902
SRR17520069.3172876
SRR17520069.3172965
SRR17520069.3173010
SRR17520069.3222676
SRR17520069.3222677
SRR17520069.3222678
SRR17520069.3222679
SRR17520069.3222680
SRR17520069.3222681
SRR17520069.3222682
SRR17520069.3222683
SRR17520069.3222684
SRR17520069.3222685
SRR17520069.3222686
SRR17520069.3222687
SRR17520069.3222688
SRR17520069.3222689
SRR17520069.3222690
SRR17520069.3222691
SRR17520069.3222692
SRR17520069.3222693
SRR17520069.3222694
SRR17520069.3222695
SRR17520069.3222696
SRR17520069.3222697
SRR17520069.3222701
SRR17520069.3222704
SRR17520069.3222705
SRR17520069.3222706
SRR17520069.3222708
SRR17520069.3222709
SRR17520069.3222710
SRR17520069.3222711
SRR17520069.3222712
SRR17520069.3222713
SRR17520069.3222714
SRR17520069.3222715
SRR17520069.3222716
SRR17520069.3222717

SRR17520069.3222718
SRR17520069.3222719
SRR17520069.3222720
SRR17520069.3222721
SRR17520069.3222722
SRR17520069.3222723
SRR17520069.3222724
SRR17520069.3222725
SRR17520069.3231608
SRR17520069.3237093
SRR17520069.3252789
SRR17520069.3264950
SRR17520069.3264951
SRR17520069.3264952
SRR17520069.3264953
SRR17520069.3264954
SRR17520069.3264955
SRR17520069.3264956
SRR17520069.3264957
SRR17520069.3264958
SRR17520069.3264959
SRR17520069.3264960
SRR17520069.3264961
SRR17520069.3264962
SRR17520069.3264963
SRR17520069.3264964
SRR17520069.3264965
SRR17520069.3264966
SRR17520069.3264967
SRR17520069.3264968
SRR17520069.3264969
SRR17520069.3264970
SRR17520069.3264971
SRR17520069.3274005
SRR17520069.3274006
SRR17520069.3274007
SRR17520069.3274008
SRR17520069.3274009
SRR17520069.3274010
SRR17520069.3274011
SRR17520069.3274012
SRR17520069.3274013
SRR17520069.3274014
SRR17520069.3274015
SRR17520069.3274016
SRR17520069.3274017
SRR17520069.3274018

SRR17520069.3274019
SRR17520069.3274020
SRR17520069.3274021
SRR17520069.3274022
SRR17520069.3274023
SRR17520069.3274024
SRR17520069.3292219
SRR17520069.3309996
SRR17520069.3309997
SRR17520069.3309998
SRR17520069.3309999
SRR17520069.3310000
SRR17520069.3310001
SRR17520069.3310002
SRR17520069.3310003
SRR17520069.3310004
SRR17520069.3310005
SRR17520069.3310006
SRR17520069.3310007
SRR17520069.3310008
SRR17520069.3310009
SRR17520069.3310010
SRR17520069.3310011
SRR17520069.3310012
SRR17520069.3310013
SRR17520069.3310014
SRR17520069.3310015
SRR17520069.3310016
SRR17520069.3310017
SRR17520069.3310018
SRR17520069.3310019
SRR17520069.3310020
SRR17520069.3310021
SRR17520069.3310022
SRR17520069.3310023
SRR17520069.3310024
SRR17520069.3310025
SRR17520069.3310026
SRR17520069.3310027
SRR17520069.3310028
SRR17520069.3310029
SRR17520069.3310030
SRR17520069.3310031
SRR17520069.3310032
SRR17520069.3310033
SRR17520069.3310034
SRR17520069.3310036

SRR17520069.3310037
SRR17520069.3328787
SRR17520069.3328797
SRR17520069.3339686
SRR17520069.3342622
SRR17520069.3342623
SRR17520069.3342624
SRR17520069.3342625
SRR17520069.3342626
SRR17520069.3342627
SRR17520069.3342628
SRR17520069.3342629
SRR17520069.3342630
SRR17520069.3342631
SRR17520069.3342632
SRR17520069.3342633
SRR17520069.3342634
SRR17520069.3342635
SRR17520069.3342636
SRR17520069.3342637
SRR17520069.3342638
SRR17520069.3342639
SRR17520069.3342640
SRR17520069.3342641
SRR17520069.3342642
SRR17520069.3342643
SRR17520069.3342644
SRR17520069.3342645
SRR17520069.3342646
SRR17520069.3342647
SRR17520069.3342648
SRR17520069.3342649
SRR17520069.3342650
SRR17520069.3342651
SRR17520069.3342652
SRR17520069.3342653
SRR17520069.3342654
SRR17520069.3342655
SRR17520069.3342656
SRR17520069.3342657
SRR17520069.3342658
SRR17520069.3342659
SRR17520069.3342660
SRR17520069.3342662
SRR17520069.3342663
SRR17520069.3342664
SRR17520069.3342665

SRR17520069.3342666
SRR17520069.3342667
SRR17520069.3342668
SRR17520069.3342669
SRR17520069.3342670
SRR17520069.3342671
SRR17520069.3342672
SRR17520069.3342673
SRR17520069.3342674
SRR17520069.3342675
SRR17520069.3342676
SRR17520069.3342677
SRR17520069.3342678
SRR17520069.3342679
SRR17520069.3342680
SRR17520069.3342681
SRR17520069.3342682
SRR17520069.3342683
SRR17520069.3342684
SRR17520069.3342685
SRR17520069.3342686
SRR17520069.3342687
SRR17520069.3342688
SRR17520069.3345265
SRR17520069.3345276
SRR17520069.3345322
SRR17520069.3346376
SRR17520069.3375926
SRR17520069.3375927
SRR17520069.3375928
SRR17520069.3375929
SRR17520069.3375931
SRR17520069.3375932
SRR17520069.3375933
SRR17520069.3375934
SRR17520069.3375935
SRR17520069.3375937
SRR17520069.3375938
SRR17520069.3375939
SRR17520069.3375940
SRR17520069.3375941
SRR17520069.3375943
SRR17520069.3375944
SRR17520069.3375945
SRR17520069.3375946
SRR17520069.3375947
SRR17520069.3375948

SRR17520069.3375949
SRR17520069.3375951
SRR17520069.3375952
SRR17520069.3375953
SRR17520069.3375954
SRR17520069.3375955
SRR17520069.3375956
SRR17520069.3375957
SRR17520069.3375958
SRR17520069.3375959
SRR17520069.3375960
SRR17520069.3375962
SRR17520069.3375963
SRR17520069.3375964
SRR17520069.3375966
SRR17520069.3375967
SRR17520069.3375968
SRR17520069.3375970
SRR17520069.3375971
SRR17520069.3375972
SRR17520069.3375973
SRR17520069.3375974
SRR17520069.3375975
SRR17520069.3375977
SRR17520069.3375978
SRR17520069.3375979
SRR17520069.3375980
SRR17520069.3375981
SRR17520069.3375982
SRR17520069.3375983
SRR17520069.3375984
SRR17520069.3375985
SRR17520069.3375986
SRR17520069.3375987
SRR17520069.3375989
SRR17520069.3375990
SRR17520069.3375991
SRR17520069.3375992
SRR17520069.3375993
SRR17520069.3375994
SRR17520069.3375995
SRR17520069.3375996
SRR17520069.3375997
SRR17520069.3375998
SRR17520069.3375999
SRR17520069.3376000
SRR17520069.3376002

SRR17520069.3376003
SRR17520069.3376004
SRR17520069.3376005
SRR17520069.3376006
SRR17520069.3376007
SRR17520069.3376008
SRR17520069.3376009
SRR17520069.3376010
SRR17520069.3376011
SRR17520069.3376014
SRR17520069.3376015
SRR17520069.3376016
SRR17520069.3376017
SRR17520069.3376018
SRR17520069.3376021
SRR17520069.3376022
SRR17520069.3376023
SRR17520069.3376024
SRR17520069.3376025
SRR17520069.3376026
SRR17520069.3376027
SRR17520069.3376028
SRR17520069.3376029
SRR17520069.3376030
SRR17520069.3376031
SRR17520069.3376032
SRR17520069.3376033
SRR17520069.3376034
SRR17520069.3376035
SRR17520069.3376036
SRR17520069.3376037
SRR17520069.3376038
SRR17520069.3376039
SRR17520069.3376040
SRR17520069.3376041
SRR17520069.3376042
SRR17520069.3376044
SRR17520069.3376045
SRR17520069.3376046
SRR17520069.3376047
SRR17520069.3376048
SRR17520069.3376049
SRR17520069.3376050
SRR17520069.3376051
SRR17520069.3376052
SRR17520069.3376053
SRR17520069.3376054

SRR17520069.3376055
SRR17520069.3376056
SRR17520069.3376057
SRR17520069.3376059
SRR17520069.3376060
SRR17520069.3376061
SRR17520069.3376063
SRR17520069.3376064
SRR17520069.3376065
SRR17520069.3376066
SRR17520069.3376068
SRR17520069.3376069
SRR17520069.3376070
SRR17520069.3376072
SRR17520069.3376073
SRR17520069.3376074
SRR17520069.3376075
SRR17520069.3376076
SRR17520069.3376077
SRR17520069.3376078
SRR17520069.3376079
SRR17520069.3376080
SRR17520069.3376081
SRR17520069.3376082
SRR17520069.3376083
SRR17520069.3376084
SRR17520069.3376085
SRR17520069.3376086
SRR17520069.3376087
SRR17520069.3376088
SRR17520069.3376089
SRR17520069.3376090
SRR17520069.3376092
SRR17520069.3376093
SRR17520069.3376094
SRR17520069.3376095
SRR17520069.3376096
SRR17520069.3376097
SRR17520069.3376098
SRR17520069.3376099
SRR17520069.3376100
SRR17520069.3376101
SRR17520069.3376102
SRR17520069.3376103
SRR17520069.3376104
SRR17520069.3376105
SRR17520069.3376106

SRR17520069.3376107
SRR17520069.3376108
SRR17520069.3376109
SRR17520069.3376110
SRR17520069.3376111
SRR17520069.3376112
SRR17520069.3376114
SRR17520069.3376115
SRR17520069.3376116
SRR17520069.3376117
SRR17520069.3376118
SRR17520069.3376119
SRR17520069.3376121
SRR17520069.3376122
SRR17520069.3376123
SRR17520069.3376125
SRR17520069.3376126
SRR17520069.3376127
SRR17520069.3376128
SRR17520069.3376130
SRR17520069.3376131
SRR17520069.3376132
SRR17520069.3376133
SRR17520069.3376135
SRR17520069.3376137
SRR17520069.3376138
SRR17520069.3376139
SRR17520069.3376140
SRR17520069.3376141
SRR17520069.3376142
SRR17520069.3376143
SRR17520069.3376144
SRR17520069.3376147
SRR17520069.3376148
SRR17520069.3376149
SRR17520069.3376150
SRR17520069.3376151
SRR17520069.3376152
SRR17520069.3376153
SRR17520069.3376154
SRR17520069.3376155
SRR17520069.3376156
SRR17520069.3391609
SRR17520069.3407531
SRR17520069.3407533
SRR17520069.3407534
SRR17520069.3407535

SRR17520069.3407536
SRR17520069.3407537
SRR17520069.3407538
SRR17520069.3407539
SRR17520069.3407540
SRR17520069.3407541
SRR17520069.3407542
SRR17520069.3407543
SRR17520069.3407544
SRR17520069.3407545
SRR17520069.3407546
SRR17520069.3407547
SRR17520069.3407548
SRR17520069.3407549
SRR17520069.3407550
SRR17520069.3407551
SRR17520069.3407553
SRR17520069.3407554
SRR17520069.3407555
SRR17520069.3407556
SRR17520069.3407557
SRR17520069.3407558
SRR17520069.3407559
SRR17520069.3407560
SRR17520069.3407561
SRR17520069.3407563
SRR17520069.3407564
SRR17520069.3407565
SRR17520069.3407566
SRR17520069.3407567
SRR17520069.3407569
SRR17520069.3428104
SRR17520069.3430894
SRR17520069.3430904
SRR17520069.3434679
SRR17520069.3474191
SRR17520069.3480298
SRR17520069.3485892
SRR17520069.3527741
SRR17520069.3527743
SRR17520069.3527745
SRR17520069.3527746
SRR17520069.3527747
SRR17520069.3527748
SRR17520069.3527749
SRR17520069.3527751
SRR17520069.3527752

SRR17520069.3527753
SRR17520069.3527754
SRR17520069.3527755
SRR17520069.3527756
SRR17520069.3527757
SRR17520069.3527759
SRR17520069.3527762
SRR17520069.3527763
SRR17520069.3527765
SRR17520069.3527766
SRR17520069.3527767
SRR17520069.3527768
SRR17520069.3527769
SRR17520069.3527770
SRR17520069.3527771
SRR17520069.3527772
SRR17520069.3527773
SRR17520069.3527774
SRR17520069.3527775
SRR17520069.3527776
SRR17520069.3527777
SRR17520069.3527779
SRR17520069.3527782
SRR17520069.3527783
SRR17520069.3527786
SRR17520069.3527787
SRR17520069.3527788
SRR17520069.3527790
SRR17520069.3527791
SRR17520069.3527792
SRR17520069.3527795
SRR17520069.3527796
SRR17520069.3527797
SRR17520069.3527799
SRR17520069.3527800
SRR17520069.3527801
SRR17520069.3527804
SRR17520069.3527805
SRR17520069.3527809
SRR17520069.3527811
SRR17520069.3527812
SRR17520069.3527813
SRR17520069.3527815
SRR17520069.3527816
SRR17520069.3527817
SRR17520069.3527819
SRR17520069.3527820

SRR17520069.3527822
SRR17520069.3527823
SRR17520069.3527824
SRR17520069.3527826
SRR17520069.3527827
SRR17520069.3527828
SRR17520069.3527829
SRR17520069.3527830
SRR17520069.3527831
SRR17520069.3527832
SRR17520069.3527833
SRR17520069.3527834
SRR17520069.3527835
SRR17520069.3527836
SRR17520069.3527838
SRR17520069.3527839
SRR17520069.3527840
SRR17520069.3527841
SRR17520069.3527843
SRR17520069.3527844
SRR17520069.3527845
SRR17520069.3527847
SRR17520069.3527849
SRR17520069.3527850
SRR17520069.3527851
SRR17520069.3527853
SRR17520069.3527854
SRR17520069.3527855
SRR17520069.3527856
SRR17520069.3527859
SRR17520069.3527861
SRR17520069.3527862
SRR17520069.3527863
SRR17520069.3527864
SRR17520069.3527865
SRR17520069.3527866
SRR17520069.3527867
SRR17520069.3527868
SRR17520069.3527869
SRR17520069.3527870
SRR17520069.3527871
SRR17520069.3540088
SRR17520069.3608319
SRR17520069.3633915
SRR17520069.3633946
SRR17520069.3644271
SRR17520069.3644272

SRR17520069.3644273
SRR17520069.3644274
SRR17520069.3644275
SRR17520069.3644276
SRR17520069.3644277
SRR17520069.3644278
SRR17520069.3644279
SRR17520069.3644280
SRR17520069.3644281
SRR17520069.3644282
SRR17520069.3644283
SRR17520069.3644284
SRR17520069.3644285
SRR17520069.3644286
SRR17520069.3644287
SRR17520069.3644289
SRR17520069.3644290
SRR17520069.3644291
SRR17520069.3644292
SRR17520069.3644293
SRR17520069.3644294
SRR17520069.3644295
SRR17520069.3644296
SRR17520069.3644297
SRR17520069.3644298
SRR17520069.3644299
SRR17520069.3644300
SRR17520069.3644301
SRR17520069.3644302
SRR17520069.3644303
SRR17520069.3644304
SRR17520069.3644305
SRR17520069.3657344
SRR17520069.3657345
SRR17520069.3657346
SRR17520069.3657347
SRR17520069.3657348
SRR17520069.3657349
SRR17520069.3657350
SRR17520069.3657351
SRR17520069.3657352
SRR17520069.3657353
SRR17520069.3657354
SRR17520069.3657356
SRR17520069.3657357
SRR17520069.3657358
SRR17520069.3657359

SRR17520069.3657360
SRR17520069.3657361
SRR17520069.3657362
SRR17520069.3657363
SRR17520069.3657364
SRR17520069.3657365
SRR17520069.3657366
SRR17520069.3657367
SRR17520069.3657368
SRR17520069.3657369
SRR17520069.3657371
SRR17520069.3657372
SRR17520069.3657373
SRR17520069.3657374
SRR17520069.3657375
SRR17520069.3657376
SRR17520069.3657377
SRR17520069.3657378
SRR17520069.3657379
SRR17520069.3657380
SRR17520069.3657381
SRR17520069.3657382
SRR17520069.3657383
SRR17520069.3657384
SRR17520069.3657385
SRR17520069.3657386
SRR17520069.3657387
SRR17520069.3657388
SRR17520069.3657389
SRR17520069.3657390
SRR17520069.3657391
SRR17520069.3657392
SRR17520069.3657393
SRR17520069.3657394
SRR17520069.3657395
SRR17520069.3657396
SRR17520069.3657397
SRR17520069.3657398
SRR17520069.3657399
SRR17520069.3657400
SRR17520069.3657401
SRR17520069.3657402
SRR17520069.3657403
SRR17520069.3657404
SRR17520069.3657405
SRR17520069.3657406
SRR17520069.3657407

SRR17520069.3657408
SRR17520069.3657409
SRR17520069.3657410
SRR17520069.3657411
SRR17520069.3657412
SRR17520069.3657413
SRR17520069.3657414
SRR17520069.3657415
SRR17520069.3657416
SRR17520069.3657417
SRR17520069.3657418
SRR17520069.3657419
SRR17520069.3657420
SRR17520069.3657421
SRR17520069.3657422
SRR17520069.3657423
SRR17520069.3657424
SRR17520069.3657425
SRR17520069.3657426
SRR17520069.3657427
SRR17520069.3657428
SRR17520069.3657429
SRR17520069.3657430
SRR17520069.3657431
SRR17520069.3657432
SRR17520069.3657433
SRR17520069.3657434
SRR17520069.3657435
SRR17520069.3657436
SRR17520069.3657437
SRR17520069.3657438
SRR17520069.3657439
SRR17520069.3657440
SRR17520069.3657441
SRR17520069.3657442
SRR17520069.3657443
SRR17520069.3657444
SRR17520069.3657445
SRR17520069.3657446
SRR17520069.3657447
SRR17520069.3657448
SRR17520069.3657449
SRR17520069.3657450
SRR17520069.3657451
SRR17520069.3657452
SRR17520069.3657454
SRR17520069.3657455

SRR17520069.3657456
SRR17520069.3657457
SRR17520069.3657458
SRR17520069.3657459
SRR17520069.3657460
SRR17520069.3657461
SRR17520069.3657463
SRR17520069.3657464
SRR17520069.3657465
SRR17520069.3657466
SRR17520069.3657467
SRR17520069.3657468
SRR17520069.3657469
SRR17520069.3657470
SRR17520069.3657471
SRR17520069.3657472
SRR17520069.3657473
SRR17520069.3657474
SRR17520069.3657475
SRR17520069.3662829
SRR17520069.3668491
SRR17520069.3668582
SRR17520069.3695955
SRR17520069.3695956
SRR17520069.3695957
SRR17520069.3695958
SRR17520069.3695959
SRR17520069.3695960
SRR17520069.3715784
SRR17520069.3729245
SRR17520069.3730155
SRR17520069.3770176
SRR17520069.3829843
SRR17520069.3839135
SRR17520069.3839199
SRR17520069.3863476
SRR17520069.3891862
SRR17520069.3902636
SRR17520069.3914528
SRR17520069.3914553
SRR17520069.3914559
SRR17520069.3930334
SRR17520069.3954140
SRR17520069.3960619
SRR17520069.3962174
SRR17520069.3971238
SRR17520069.3971239

SRR17520069.3997585
SRR17520069.4004036
SRR17520069.4039357
SRR17520069.4046605
SRR17520069.4046606
SRR17520069.4046607
SRR17520069.4046608
SRR17520069.4046609
SRR17520069.4046610
SRR17520069.4065745
SRR17520069.4069883
SRR17520069.4093484
SRR17520069.4093485
SRR17520069.4093489
SRR17520069.4093490
SRR17520069.4093491
SRR17520069.4093492
SRR17520069.4093493
SRR17520069.4093494
SRR17520069.4093495
SRR17520069.4093496
SRR17520069.4093497
SRR17520069.4093498
SRR17520069.4093499
SRR17520069.4093500
SRR17520069.4093501
SRR17520069.4093502
SRR17520069.4093503
SRR17520069.4093504
SRR17520069.4093505
SRR17520069.4093506
SRR17520069.4111749
SRR17520069.4154367
SRR17520069.4155083
SRR17520069.4155113
SRR17520069.4158221
SRR17520069.4170382
SRR17520069.4173085
SRR17520069.4189992
SRR17520069.4216177
SRR17520069.4216178
SRR17520069.4216179
SRR17520069.4216180
SRR17520069.4216181
SRR17520069.4216182
SRR17520069.4216183
SRR17520069.4216184

SRR17520069.4216185
SRR17520069.4216186
SRR17520069.4216187
SRR17520069.4216188
SRR17520069.4217547
SRR17520069.4223226
SRR17520069.4231937
SRR17520069.4232101
SRR17520069.4234711
SRR17520069.4240845
SRR17520069.4314258
SRR17520069.4314303
SRR17520069.4329468
SRR17520069.4329552
SRR17520069.4342405
SRR17520069.4342406
SRR17520069.4342407
SRR17520069.4342408
SRR17520069.4342409
SRR17520069.4342410
SRR17520069.4342411
SRR17520069.4342412
SRR17520069.4342413
SRR17520069.4473425
SRR17520069.4474442
SRR17520069.4475602
SRR17520069.4480751
SRR17520069.4486052
SRR17520069.4486054
SRR17520069.4486056
SRR17520069.4486057
SRR17520069.4486058
SRR17520069.4486059
SRR17520069.4486060
SRR17520069.4486061
SRR17520069.4486062
SRR17520069.4486063
SRR17520069.4486064
SRR17520069.4486065
SRR17520069.4486066
SRR17520069.4486067
SRR17520069.4486068
SRR17520069.4486069
SRR17520069.4486070
SRR17520069.4486071
SRR17520069.4486072
SRR17520069.4486073

SRR17520069.4486074
SRR17520069.4486075
SRR17520069.4486076
SRR17520069.4486077
SRR17520069.4486078
SRR17520069.4486079
SRR17520069.4486080
SRR17520069.4486081
SRR17520069.4487451
SRR17520069.4492995
SRR17520069.4493070
SRR17520069.4511605
SRR17520069.4511607
SRR17520069.4511612
SRR17520069.4511614
SRR17520069.4511615
SRR17520069.4511616
SRR17520069.4511617
SRR17520069.4520537
SRR17520069.4520558
SRR17520069.4530014
SRR17520069.4531077
SRR17520069.4584111
SRR17520069.4584112
SRR17520069.4584113
SRR17520069.4584114
SRR17520069.4584115
SRR17520069.4584116
SRR17520069.4584117
SRR17520069.4584118
SRR17520069.4584119
SRR17520069.4584120
SRR17520069.4584121
SRR17520069.4584122
SRR17520069.4584123
SRR17520069.4584124
SRR17520069.4584125
SRR17520069.4584126
SRR17520069.4584127
SRR17520069.4584128
SRR17520069.4584129
SRR17520069.4584130
SRR17520069.4584131
SRR17520069.4584132
SRR17520069.4584133
SRR17520069.4584134
SRR17520069.4584135

SRR17520069.4584136
SRR17520069.4584137
SRR17520069.4584138
SRR17520069.4584139
SRR17520069.4584140
SRR17520069.4584141
SRR17520069.4584142
SRR17520069.4584143
SRR17520069.4584144
SRR17520069.4584145
SRR17520069.4584146
SRR17520069.4584147
SRR17520069.4584148
SRR17520069.4584149
SRR17520069.4584150
SRR17520069.4584151
SRR17520069.4584152
SRR17520069.4584153
SRR17520069.4584154
SRR17520069.4584155
SRR17520069.4584156
SRR17520069.4584157
SRR17520069.4584158
SRR17520069.4584159
SRR17520069.4584160
SRR17520069.4584161
SRR17520069.4584898
SRR17520069.4626188
SRR17520069.4655726
SRR17520069.4655727
SRR17520069.4655728
SRR17520069.4655729
SRR17520069.4655730
SRR17520069.4655731
SRR17520069.4655734
SRR17520069.4655735
SRR17520069.4655736
SRR17520069.4655737
SRR17520069.4655738
SRR17520069.4655740
SRR17520069.4703320
SRR17520069.4719662
SRR17520069.4749487
SRR17520069.4770535
SRR17520069.4794806
SRR17520069.4794807
SRR17520069.4794808

SRR17520069.4794809
SRR17520069.4794810
SRR17520069.4838841
SRR17520069.4838861
SRR17520069.4838866
SRR17520069.4843893
SRR17520069.4843894
SRR17520069.4843895
SRR17520069.4843896
SRR17520069.4843897
SRR17520069.4843898
SRR17520069.4843899
SRR17520069.4843900
SRR17520069.4843901
SRR17520069.4843902
SRR17520069.4843903
SRR17520069.4843904
SRR17520069.4843905
SRR17520069.4843907
SRR17520069.4843908
SRR17520069.4843909
SRR17520069.4843910
SRR17520069.4843911
SRR17520069.4843912
SRR17520069.4843913
SRR17520069.4843914
SRR17520069.4843915
SRR17520069.4843916
SRR17520069.4843917
SRR17520069.4843918
SRR17520069.4843919
SRR17520069.4843920
SRR17520069.4843921
SRR17520069.4843922
SRR17520069.4843923
SRR17520069.4843924
SRR17520069.4843925
SRR17520069.4843926
SRR17520069.4843927
SRR17520069.4843928
SRR17520069.4843929
SRR17520069.4843930
SRR17520069.4843931
SRR17520069.4843932
SRR17520069.4843933
SRR17520069.4843934
SRR17520069.4843935

SRR17520069.4843936
SRR17520069.4843937
SRR17520069.4843938
SRR17520069.4843940
SRR17520069.4843941
SRR17520069.4849344
SRR17520069.4964600
SRR17520069.4964601
SRR17520069.4964602
SRR17520069.4964603
SRR17520069.4964604
SRR17520069.4964605
SRR17520069.4964606
SRR17520069.4964607
SRR17520069.4964608
SRR17520069.4964609
SRR17520069.4964610
SRR17520069.4964611
SRR17520069.4964612
SRR17520069.4964613
SRR17520069.4964614
SRR17520069.4964615
SRR17520069.4964616
SRR17520069.4964617
SRR17520069.4964618
SRR17520069.4964619
SRR17520069.4964620
SRR17520069.4964621
SRR17520069.4964622
SRR17520069.4964623
SRR17520069.4964624
SRR17520069.4964625
SRR17520069.4964626
SRR17520069.4964627
SRR17520069.4964628
SRR17520069.4964629
SRR17520069.4964630
SRR17520069.4964631
SRR17520069.4964632
SRR17520069.4964633
SRR17520069.4964634
SRR17520069.4964635
SRR17520069.4964636
SRR17520069.4964637
SRR17520069.4964638
SRR17520069.4964639
SRR17520069.4964640

SRR17520069.4964641
SRR17520069.4964642
SRR17520069.4964643
SRR17520069.4964644
SRR17520069.4964645
SRR17520069.4964646
SRR17520069.4964647
SRR17520069.4964648
SRR17520069.4964649
SRR17520069.4964650
SRR17520069.4964651
SRR17520069.4964652
SRR17520069.4964653
SRR17520069.4964654
SRR17520069.4964655
SRR17520069.4964656
SRR17520069.4964657
SRR17520069.4964658
SRR17520069.4964660
SRR17520069.4964661
SRR17520069.4964662
SRR17520069.4964663
SRR17520069.4964664
SRR17520069.4964665
SRR17520069.4964666
SRR17520069.4964667
SRR17520069.4964668
SRR17520069.4964669
SRR17520069.4964670
SRR17520069.4964672
SRR17520069.4964673
SRR17520069.4964674
SRR17520069.4964675
SRR17520069.4964676
SRR17520069.4964677
SRR17520069.4964678
SRR17520069.4964679
SRR17520069.4964680
SRR17520069.5075063
SRR17520069.5083461
SRR17520069.5095446
SRR17520069.5100669
SRR17520069.5104977
SRR17520069.5112923
SRR17520069.5126118
SRR17520069.5126122
SRR17520069.5126123

SRR17520069.5126125
SRR17520069.5126126
SRR17520069.5126134
SRR17520069.5126135
SRR17520069.5126136
SRR17520069.5126139
SRR17520069.5126140
SRR17520069.5126141
SRR17520069.5154496
SRR17520069.5154546
SRR17520069.5177619
SRR17520069.5210020
SRR17520069.5210021
SRR17520069.5210022
SRR17520069.5219576
SRR17520069.5225579
SRR17520069.5248075
SRR17520069.5316910
SRR17520069.5321523
SRR17520069.5321555
SRR17520069.5351524
SRR17520069.5351575
SRR17520069.5373424
SRR17520069.5373425
SRR17520069.5373426
SRR17520069.5373427
SRR17520069.5373428
SRR17520069.5373429
SRR17520069.5373430
SRR17520069.5373432
SRR17520069.5373433
SRR17520069.5373434
SRR17520069.5373435
SRR17520069.5373436
SRR17520069.5373437
SRR17520069.5373438
SRR17520069.5373439
SRR17520069.5373440
SRR17520069.5373441
SRR17520069.5373442
SRR17520069.5373443
SRR17520069.5373444
SRR17520069.5373446
SRR17520069.5373447
SRR17520069.5373448
SRR17520069.5373450
SRR17520069.5373451

SRR17520069.5373452
SRR17520069.5373453
SRR17520069.5373454
SRR17520069.5373455
SRR17520069.5373456
SRR17520069.5373457
SRR17520069.5373458
SRR17520069.5373459
SRR17520069.5373460
SRR17520069.5373461
SRR17520069.5373462
SRR17520069.5373463
SRR17520069.5373464
SRR17520069.5373465
SRR17520069.5373466
SRR17520069.5373467
SRR17520069.5373468
SRR17520069.5373469
SRR17520069.5373470
SRR17520069.5373471
SRR17520069.5398564
SRR17520069.5424107
SRR17520069.5424213
SRR17520069.5429533
SRR17520069.5439445
SRR17520069.5439446
SRR17520069.5439447
SRR17520069.5439448
SRR17520069.5439449
SRR17520069.5439450
SRR17520069.5439451
SRR17520069.5439452
SRR17520069.5439453
SRR17520069.5439454
SRR17520069.5439455
SRR17520069.5439456
SRR17520069.5439457
SRR17520069.5439458
SRR17520069.5439459
SRR17520069.5439460
SRR17520069.5439461
SRR17520069.5439462
SRR17520069.5439463
SRR17520069.5439464
SRR17520069.5439465
SRR17520069.5439466
SRR17520069.5439467

SRR17520069.5439468
SRR17520069.5439469
SRR17520069.5439470
SRR17520069.5439471
SRR17520069.5439472
SRR17520069.5439473
SRR17520069.5439474
SRR17520069.5439475
SRR17520069.5439476
SRR17520069.5439477
SRR17520069.5439478
SRR17520069.5439479
SRR17520069.5439480
SRR17520069.5439481
SRR17520069.5439482
SRR17520069.5439483
SRR17520069.5439484
SRR17520069.5439485
SRR17520069.5439486
SRR17520069.5439487
SRR17520069.5439488
SRR17520069.5439489
SRR17520069.5439490
SRR17520069.5439491
SRR17520069.5439492
SRR17520069.5439493
SRR17520069.5439494
SRR17520069.5439495
SRR17520069.5439496
SRR17520069.5439497
SRR17520069.5439498
SRR17520069.5439499
SRR17520069.5460115
SRR17520069.5460116
SRR17520069.5460117
SRR17520069.5460118
SRR17520069.5460119
SRR17520069.5460120
SRR17520069.5460122
SRR17520069.5460123
SRR17520069.5460124
SRR17520069.5460125
SRR17520069.5460126
SRR17520069.5460127
SRR17520069.5460128
SRR17520069.5460129
SRR17520069.5460130

SRR17520069.5460131
SRR17520069.5460132
SRR17520069.5460133
SRR17520069.5460134
SRR17520069.5460135
SRR17520069.5460136
SRR17520069.5460137
SRR17520069.5460138
SRR17520069.5460139
SRR17520069.5460140
SRR17520069.5460141
SRR17520069.5460142
SRR17520069.5460144
SRR17520069.5460145
SRR17520069.5460146
SRR17520069.5460147
SRR17520069.5460148
SRR17520069.5460149
SRR17520069.5460151
SRR17520069.5460152
SRR17520069.5460153
SRR17520069.5460154
SRR17520069.5460155
SRR17520069.5460156
SRR17520069.5460157
SRR17520069.5460158
SRR17520069.5460159
SRR17520069.5460160
SRR17520069.5460161
SRR17520069.5460162
SRR17520069.5460163
SRR17520069.5460164
SRR17520069.5460165
SRR17520069.5460167
SRR17520069.5460168
SRR17520069.5460169
SRR17520069.5460170
SRR17520069.5460171
SRR17520069.5460172
SRR17520069.5460216
SRR17520069.5496376
SRR17520069.5496445
SRR17520069.5496450
SRR17520069.5509897
SRR17520069.5521184
SRR17520069.5521185
SRR17520069.5521186

SRR17520069.5521187
SRR17520069.5521188
SRR17520069.5521189
SRR17520069.5521190
SRR17520069.5521191
SRR17520069.5521192
SRR17520069.5521193
SRR17520069.5521194
SRR17520069.5521195
SRR17520069.5521196
SRR17520069.5521197
SRR17520069.5521198
SRR17520069.5521199
SRR17520069.5521200
SRR17520069.5521201
SRR17520069.5521202
SRR17520069.5521203
SRR17520069.5521204
SRR17520069.5521205
SRR17520069.5521206
SRR17520069.5521207
SRR17520069.5521208
SRR17520069.5521209
SRR17520069.5521210
SRR17520069.5521211
SRR17520069.5521212
SRR17520069.5521213
SRR17520069.5521214
SRR17520069.5521215
SRR17520069.5521216
SRR17520069.5521217
SRR17520069.5521218
SRR17520069.5521219
SRR17520069.5521220
SRR17520069.5521221
SRR17520069.5521222
SRR17520069.5521223
SRR17520069.5521224
SRR17520069.5521225
SRR17520069.5521226
SRR17520069.5521227
SRR17520069.5521228
SRR17520069.5521229
SRR17520069.5521230
SRR17520069.5521231
SRR17520069.5550542
SRR17520069.5573146

SRR17520069.5573245
SRR17520069.5574005
SRR17520069.5601796
SRR17520069.5601818
SRR17520069.5601840
SRR17520069.5615830
SRR17520069.5637282
SRR17520069.5637283
SRR17520069.5639441
SRR17520069.5643545
SRR17520069.5693922
SRR17520069.5701802
SRR17520069.5701805
SRR17520069.5701810
SRR17520069.5701817
SRR17520069.5704814
SRR17520069.5704828
SRR17520069.5704829
SRR17520069.5704838
SRR17520069.5704842
SRR17520069.5704844
SRR17520069.5704847
SRR17520069.5711164
SRR17520069.5762172
SRR17520069.5762187
SRR17520069.5784030
SRR17520069.5784064
SRR17520069.5800605
SRR17520069.5822092
SRR17520069.5822094
SRR17520069.5822098
SRR17520069.5822101
SRR17520069.5822106
SRR17520069.5822107
SRR17520069.5822112
SRR17520069.5822116
SRR17520069.5822120
SRR17520069.5822122
SRR17520069.5822123
SRR17520069.5839942
SRR17520069.5843661
SRR17520069.5848963
SRR17520069.5848975
SRR17520069.5885263
SRR17520069.5898471
SRR17520069.5901803
SRR17520069.5956286

SRR17520069.5975718
SRR17520069.5975720
SRR17520069.5975721
SRR17520069.5975722
SRR17520069.5975723
SRR17520069.5975724
SRR17520069.5982847
SRR17520069.5984443
SRR17520069.5987765
SRR17520069.6034089
SRR17520069.6076845
SRR17520069.6076846
SRR17520069.6076847
SRR17520069.6076849
SRR17520069.6076850
SRR17520069.6076851
SRR17520069.6076852
SRR17520069.6076853
SRR17520069.6076854
SRR17520069.6093324
SRR17520069.6093325
SRR17520069.6093326
SRR17520069.6093327
SRR17520069.6093328
SRR17520069.6093329
SRR17520069.6093330
SRR17520069.6093331
SRR17520069.6093332
SRR17520069.6093333
SRR17520069.6093334
SRR17520069.6093335
SRR17520069.6093337
SRR17520069.6093338
SRR17520069.6093339
SRR17520069.6093340
SRR17520069.6093341
SRR17520069.6093342
SRR17520069.6093343
SRR17520069.6093344
SRR17520069.6093345
SRR17520069.6093346
SRR17520069.6093347
SRR17520069.6093348
SRR17520069.6093349
SRR17520069.6093350
SRR17520069.6093351
SRR17520069.6093352

SRR17520069.6093353
SRR17520069.6093354
SRR17520069.6093355
SRR17520069.6093356
SRR17520069.6093357
SRR17520069.6093358
SRR17520069.6093359
SRR17520069.6093360
SRR17520069.6093361
SRR17520069.6093363
SRR17520069.6093364
SRR17520069.6093365
SRR17520069.6093366
SRR17520069.6093367
SRR17520069.6093368
SRR17520069.6093369
SRR17520069.6093370
SRR17520069.6093371
SRR17520069.6093372
SRR17520069.6093373
SRR17520069.6093374
SRR17520069.6093375
SRR17520069.6093376
SRR17520069.6093377
SRR17520069.6093378
SRR17520069.6093379
SRR17520069.6093380
SRR17520069.6093381
SRR17520069.6093382
SRR17520069.6093383
SRR17520069.6093384
SRR17520069.6093385
SRR17520069.6093386
SRR17520069.6093594
SRR17520069.6097161
SRR17520069.6097172
SRR17520069.6108750
SRR17520069.6127117
SRR17520069.6148571
SRR17520069.6155186
SRR17520069.6169724
SRR17520069.6169725
SRR17520069.6169726
SRR17520069.6169727
SRR17520069.6169728
SRR17520069.6169729
SRR17520069.6169730

SRR17520069.6169731
SRR17520069.6169732
SRR17520069.6169734
SRR17520069.6169735
SRR17520069.6169736
SRR17520069.6169737
SRR17520069.6169738
SRR17520069.6169739
SRR17520069.6169740
SRR17520069.6169741
SRR17520069.6169742
SRR17520069.6169743
SRR17520069.6169744
SRR17520069.6169745
SRR17520069.6169746
SRR17520069.6169748
SRR17520069.6169749
SRR17520069.6169750
SRR17520069.6169751
SRR17520069.6169752
SRR17520069.6169753
SRR17520069.6188045
SRR17520069.6191610
SRR17520069.6198434
SRR17520069.6237045
SRR17520069.6237057
SRR17520069.6237064
SRR17520069.6246952
SRR17520069.6246953
SRR17520069.6246954
SRR17520069.6246955
SRR17520069.6246956
SRR17520069.6246957
SRR17520069.6246958
SRR17520069.6246959
SRR17520069.6246961
SRR17520069.6246962
SRR17520069.6246963
SRR17520069.6246964
SRR17520069.6246966
SRR17520069.6246967
SRR17520069.6246968
SRR17520069.6271904
SRR17520069.6289468
SRR17520069.6289514
SRR17520069.6291752
SRR17520069.6291772

SRR17520069.6293723
SRR17520069.6293724
SRR17520069.6293725
SRR17520069.6293726
SRR17520069.6293727
SRR17520069.6293728
SRR17520069.6293729
SRR17520069.6293730
SRR17520069.6293731
SRR17520069.6293732
SRR17520069.6293733
SRR17520069.6293734
SRR17520069.6293735
SRR17520069.6293736
SRR17520069.6293737
SRR17520069.6293738
SRR17520069.6293739
SRR17520069.6293740
SRR17520069.6293741
SRR17520069.6293742
SRR17520069.6293743
SRR17520069.6293744
SRR17520069.6293745
SRR17520069.6293746
SRR17520069.6293747
SRR17520069.6293748
SRR17520069.6341518
SRR17520069.6349863
SRR17520069.6368300
SRR17520069.6378057
SRR17520069.6401979
SRR17520069.6408753
SRR17520069.6421200
SRR17520069.6457925
SRR17520069.6511916
SRR17520069.6574121
SRR17520069.6589322
SRR17520069.6598055
SRR17520069.6632192
SRR17520069.6648885
SRR17520069.6657680
SRR17520069.6657729
SRR17520069.6657746
SRR17520069.6666063
SRR17520069.6666076
SRR17520069.6691510
SRR17520069.6715079

SRR17520069.6738643
SRR17520069.6762427
SRR17520069.6795703
SRR17520069.6835664
SRR17520069.6835665
SRR17520069.6835666
SRR17520069.6835668
SRR17520069.6835669
SRR17520069.6835670
SRR17520069.6835671
SRR17520069.6835672
SRR17520069.6835673
SRR17520069.6835674
SRR17520069.6835675
SRR17520069.6835676
SRR17520069.6835677
SRR17520069.6835678
SRR17520069.6835679
SRR17520069.6835680
SRR17520069.6835681
SRR17520069.6835683
SRR17520069.6835684
SRR17520069.6857971
SRR17520069.6927886
SRR17520069.6936141
SRR17520069.6947693
SRR17520069.6949980
SRR17520069.6949981
SRR17520069.6949982
SRR17520069.6949983
SRR17520069.6949984
SRR17520069.6949985
SRR17520069.6949986
SRR17520069.6949987
SRR17520069.6949988
SRR17520069.6949989
SRR17520069.6949990
SRR17520069.6949991
SRR17520069.6949992
SRR17520069.6949993
SRR17520069.6949994
SRR17520069.6949995
SRR17520069.6949996
SRR17520069.6949997
SRR17520069.6949998
SRR17520069.6949999
SRR17520069.6950000

SRR17520069.6950001
SRR17520069.6950002
SRR17520069.6950003
SRR17520069.6950004
SRR17520069.6950005
SRR17520069.6950006
SRR17520069.6950007
SRR17520069.6950008
SRR17520069.6950009
SRR17520069.6950010
SRR17520069.6950011
SRR17520069.6950012
SRR17520069.6950013
SRR17520069.6950014
SRR17520069.6950015
SRR17520069.6950016
SRR17520069.6950017
SRR17520069.6950018
SRR17520069.6950019
SRR17520069.6950020
SRR17520069.6950021
SRR17520069.6950022
SRR17520069.6950023
SRR17520069.6950024
SRR17520069.6950025
SRR17520069.6950026
SRR17520069.6950027
SRR17520069.6950028
SRR17520069.6950029
SRR17520069.6950030
SRR17520069.6950031
SRR17520069.6950032
SRR17520069.6950033
SRR17520069.6950034
SRR17520069.6950035
SRR17520069.6950036
SRR17520069.6950037
SRR17520069.6950038
SRR17520069.6950039
SRR17520069.6950040
SRR17520069.6950041
SRR17520069.6950042
SRR17520069.6950043
SRR17520069.6950044
SRR17520069.6950045
SRR17520069.6950046
SRR17520069.6950047

SRR17520069.6950048
SRR17520069.6950049
SRR17520069.6950050
SRR17520069.6950051
SRR17520069.6950052
SRR17520069.6950053
SRR17520069.6950054
SRR17520069.6950055
SRR17520069.6950056
SRR17520069.6950057
SRR17520069.6950058
SRR17520069.6950059
SRR17520069.6950060
SRR17520069.6950061
SRR17520069.6950062
SRR17520069.6950063
SRR17520069.6950064
SRR17520069.6950065
SRR17520069.6950066
SRR17520069.6950067
SRR17520069.6950068
SRR17520069.6950069
SRR17520069.6950070
SRR17520069.6950071
SRR17520069.6950072
SRR17520069.6950073
SRR17520069.6950074
SRR17520069.6950075
SRR17520069.6950076
SRR17520069.6950077
SRR17520069.6950078
SRR17520069.6950079
SRR17520069.6950080
SRR17520069.6950081
SRR17520069.6950082
SRR17520069.6950083
SRR17520069.6950084
SRR17520069.6950085
SRR17520069.6950086
SRR17520069.6950087
SRR17520069.6950088
SRR17520069.6950089
SRR17520069.6950090
SRR17520069.6950091
SRR17520069.6950092
SRR17520069.6950093
SRR17520069.6950094

SRR17520069.6950095
SRR17520069.6950096
SRR17520069.6950097
SRR17520069.6950098
SRR17520069.6950099
SRR17520069.6950100
SRR17520069.6950101
SRR17520069.6950102
SRR17520069.6950103
SRR17520069.6950104
SRR17520069.6950105
SRR17520069.6950106
SRR17520069.6950107
SRR17520069.6950108
SRR17520069.6950109
SRR17520069.6950110
SRR17520069.6950111
SRR17520069.6950113
SRR17520069.6950114
SRR17520069.6950115
SRR17520069.6950116
SRR17520069.6950117
SRR17520069.6950118
SRR17520069.6950119
SRR17520069.6950120
SRR17520069.6950121
SRR17520069.6950122
SRR17520069.6950123
SRR17520069.6950124
SRR17520069.6950125
SRR17520069.6950126
SRR17520069.6950127
SRR17520069.6950128
SRR17520069.6950129
SRR17520069.6950130
SRR17520069.6950131
SRR17520069.6950132
SRR17520069.6990785
SRR17520069.6990817
SRR17520069.7021450
SRR17520069.7040884
SRR17520069.7063744
SRR17520069.7101960
SRR17520069.7169076
SRR17520069.7169077
SRR17520069.7169078
SRR17520069.7169079

SRR17520069.7169080
SRR17520069.7169081
SRR17520069.7169082
SRR17520069.7169083
SRR17520069.7169084
SRR17520069.7169085
SRR17520069.7169086
SRR17520069.7169087
SRR17520069.7169088
SRR17520069.7169089
SRR17520069.7169090
SRR17520069.7169091
SRR17520069.7169092
SRR17520069.7192084
SRR17520069.7192091
SRR17520069.7231854
SRR17520069.7231988
SRR17520069.7231993
SRR17520069.7237151
SRR17520069.7254121
SRR17520069.7274565
SRR17520069.7281841
SRR17520069.7281842
SRR17520069.7281843
SRR17520069.7281844
SRR17520069.7281845
SRR17520069.7281846
SRR17520069.7281847
SRR17520069.7281848
SRR17520069.7281849
SRR17520069.7281850
SRR17520069.7281851
SRR17520069.7281852
SRR17520069.7281853
SRR17520069.7281854
SRR17520069.7281855
SRR17520069.7281856
SRR17520069.7281857
SRR17520069.7281858
SRR17520069.7281859
SRR17520069.7281860
SRR17520069.7281861
SRR17520069.7281862
SRR17520069.7281863
SRR17520069.7281864
SRR17520069.7281865
SRR17520069.7284391

SRR17520069.7299631
SRR17520069.7311090
SRR17520069.7318869
SRR17520069.7345390
SRR17520069.7370996
SRR17520069.7371053
SRR17520069.7376257
SRR17520069.7389678
SRR17520069.7394917
SRR17520069.7420613
SRR17520069.7435015
SRR17520069.7435016
SRR17520069.7435017
SRR17520069.7435018
SRR17520069.7435019
SRR17520069.7435020
SRR17520069.7435021
SRR17520069.7435022
SRR17520069.7435023
SRR17520069.7435024
SRR17520069.7435025
SRR17520069.7435026
SRR17520069.7435027
SRR17520069.7435028
SRR17520069.7435029
SRR17520069.7435030
SRR17520069.7456622
SRR17520069.7470059
SRR17520069.7484314
SRR17520069.7484315
SRR17520069.7484316
SRR17520069.7484317
SRR17520069.7484318
SRR17520069.7484319
SRR17520069.7490658
SRR17520069.7491942
SRR17520069.7491953
SRR17520069.7524835
SRR17520069.7529736
SRR17520069.7552695
SRR17520069.7579494
SRR17520069.7605670
SRR17520069.7631215
SRR17520069.7644208
SRR17520069.7679442
SRR17520069.7689268
SRR17520069.7689269

SRR17520069.7689270
SRR17520069.7689271
SRR17520069.7689272
SRR17520069.7689273
SRR17520069.7689275
SRR17520069.7689276
SRR17520069.7689277
SRR17520069.7689278
SRR17520069.7689279
SRR17520069.7689280
SRR17520069.7689281
SRR17520069.7689282
SRR17520069.7689285
SRR17520069.7689286
SRR17520069.7689287
SRR17520069.7689288
SRR17520069.7689289
SRR17520069.7689292
SRR17520069.7689293
SRR17520069.7689295
SRR17520069.7689296
SRR17520069.7689297
SRR17520069.7689298
SRR17520069.7689299
SRR17520069.7689300
SRR17520069.7689302
SRR17520069.7689303
SRR17520069.7689304
SRR17520069.7689305
SRR17520069.7689306
SRR17520069.7689307
SRR17520069.7689309
SRR17520069.7689311
SRR17520069.7689312
SRR17520069.7689313
SRR17520069.7689315
SRR17520069.7689317
SRR17520069.7689318
SRR17520069.7689319
SRR17520069.7689322
SRR17520069.7689323
SRR17520069.7689324
SRR17520069.7689326
SRR17520069.7689327
SRR17520069.7689328
SRR17520069.7689329
SRR17520069.7689330

SRR17520069.7689331
SRR17520069.7689332
SRR17520069.7689333
SRR17520069.7689334
SRR17520069.7689335
SRR17520069.7689336
SRR17520069.7689337
SRR17520069.7689338
SRR17520069.7689339
SRR17520069.7689340
SRR17520069.7689341
SRR17520069.7689342
SRR17520069.7689343
SRR17520069.7689344
SRR17520069.7689345
SRR17520069.7689346
SRR17520069.7689347
SRR17520069.7689348
SRR17520069.7689349
SRR17520069.7689351
SRR17520069.7689353
SRR17520069.7689354
SRR17520069.7689356
SRR17520069.7689357
SRR17520069.7689359
SRR17520069.7689360
SRR17520069.7689362
SRR17520069.7689363
SRR17520069.7689365
SRR17520069.7689366
SRR17520069.7703331
SRR17520069.7703368
SRR17520069.7707620
SRR17520069.7714986
SRR17520069.7716406
SRR17520069.7716407
SRR17520069.7716408
SRR17520069.7716409
SRR17520069.7716410
SRR17520069.7750478
SRR17520069.7754766
SRR17520069.7754857
SRR17520069.7764940
SRR17520069.7806358
SRR17520069.7806375
SRR17520069.7813707
SRR17520069.7817808

SRR17520069.7848310
SRR17520069.7850448
SRR17520069.7857329
SRR17520069.7892868
SRR17520069.7897231
SRR17520069.7905293
SRR17520069.7906128
SRR17520069.7960939
SRR17520069.7960940
SRR17520069.7960941
SRR17520069.7960942
SRR17520069.7960943
SRR17520069.7960944
SRR17520069.7960947
SRR17520069.7960948
SRR17520069.7960949
SRR17520069.7960950
SRR17520069.7964740
SRR17520069.7964748
SRR17520069.8004061
SRR17520069.8004062
SRR17520069.8004063
SRR17520069.8004064
SRR17520069.8004065
SRR17520069.8004066
SRR17520069.8004067
SRR17520069.8004068
SRR17520069.8004069
SRR17520069.8004070
SRR17520069.8004071
SRR17520069.8004072
SRR17520069.8004073
SRR17520069.8004074
SRR17520069.8004075
SRR17520069.8004076
SRR17520069.8004078
SRR17520069.8004079
SRR17520069.8004080
SRR17520069.8004081
SRR17520069.8004082
SRR17520069.8004083
SRR17520069.8004084
SRR17520069.8004085
SRR17520069.8004086
SRR17520069.8004087
SRR17520069.8004088
SRR17520069.8004089

SRR17520069.8004090
SRR17520069.8004091
SRR17520069.8004093
SRR17520069.8007361
SRR17520069.8023015
SRR17520069.8023025
SRR17520069.8033455
SRR17520069.8033457
SRR17520069.8033458
SRR17520069.8033459
SRR17520069.8033460
SRR17520069.8033461
SRR17520069.8033462
SRR17520069.8033463
SRR17520069.8033464
SRR17520069.8033465
SRR17520069.8033466
SRR17520069.8033468
SRR17520069.8033469
SRR17520069.8033470
SRR17520069.8033471
SRR17520069.8033472
SRR17520069.8033473
SRR17520069.8033474
SRR17520069.8033475
SRR17520069.8033476
SRR17520069.8033477
SRR17520069.8033478
SRR17520069.8033479
SRR17520069.8033480
SRR17520069.8033481
SRR17520069.8033482
SRR17520069.8033483
SRR17520069.8033484
SRR17520069.8033485
SRR17520069.8033486
SRR17520069.8033487
SRR17520069.8033488
SRR17520069.8033489
SRR17520069.8033490
SRR17520069.8033491
SRR17520069.8033492
SRR17520069.8033493
SRR17520069.8033494
SRR17520069.8033495
SRR17520069.8033496
SRR17520069.8033497

SRR17520069.8033498
SRR17520069.8033499
SRR17520069.8033500
SRR17520069.8033501
SRR17520069.8033502
SRR17520069.8033503
SRR17520069.8033504
SRR17520069.8033505
SRR17520069.8033506
SRR17520069.8033507
SRR17520069.8033508
SRR17520069.8033509
SRR17520069.8033510
SRR17520069.8033511
SRR17520069.8033512
SRR17520069.8063042
SRR17520069.8077672
SRR17520069.8087944
SRR17520069.8093601
SRR17520069.8147629
SRR17520069.8158000
SRR17520069.8158001
SRR17520069.8158002
SRR17520069.8174494
SRR17520069.8184933
SRR17520069.8193572
SRR17520069.8236162
SRR17520069.8267507
SRR17520069.8282290
SRR17520069.8282291
SRR17520069.8282292
SRR17520069.8282293
SRR17520069.8282294
SRR17520069.8282295
SRR17520069.8282296
SRR17520069.8282297
SRR17520069.8282298
SRR17520069.8282299
SRR17520069.8282300
SRR17520069.8282301
SRR17520069.8282302
SRR17520069.8282303
SRR17520069.8282304
SRR17520069.8282305
SRR17520069.8282306
SRR17520069.8282307
SRR17520069.8282308

SRR17520069.8282309
SRR17520069.8282310
SRR17520069.8282311
SRR17520069.8282312
SRR17520069.8282313
SRR17520069.8282314
SRR17520069.8282315
SRR17520069.8282316
SRR17520069.8282317
SRR17520069.8282318
SRR17520069.8282319
SRR17520069.8282320
SRR17520069.8282321
SRR17520069.8282322
SRR17520069.8282323
SRR17520069.8282324
SRR17520069.8282325
SRR17520069.8282326
SRR17520069.8282327
SRR17520069.8282328
SRR17520069.8282329
SRR17520069.8282330
SRR17520069.8282331
SRR17520069.8282332
SRR17520069.8282333
SRR17520069.8282334
SRR17520069.8282335
SRR17520069.8282336
SRR17520069.8282337
SRR17520069.8288276
SRR17520069.8288278
SRR17520069.8302031
SRR17520069.8315768
SRR17520069.8315771
SRR17520069.8315776
SRR17520069.8315797
SRR17520069.8315799
SRR17520069.8315807
SRR17520069.8315809
SRR17520069.8315811
SRR17520069.8315829
SRR17520069.8315841
SRR17520069.8315843
SRR17520069.8315845
SRR17520069.8315847
SRR17520069.8315849
SRR17520069.8315855

SRR17520069.8315859
SRR17520069.8315861
SRR17520069.8332395
SRR17520069.8335270
SRR17520069.8335340
SRR17520069.8335341
SRR17520069.8335342
SRR17520069.8335343
SRR17520069.8335344
SRR17520069.8335345
SRR17520069.8335346
SRR17520069.8335347
SRR17520069.8335348
SRR17520069.8335349
SRR17520069.8335350
SRR17520069.8335351
SRR17520069.8335352
SRR17520069.8335353
SRR17520069.8335354
SRR17520069.8335355
SRR17520069.8335356
SRR17520069.8335357
SRR17520069.8335358
SRR17520069.8335359
SRR17520069.8335360
SRR17520069.8335361
SRR17520069.8335362
SRR17520069.8335363
SRR17520069.8335364
SRR17520069.8335365
SRR17520069.8335366
SRR17520069.8354555
SRR17520069.8360409
SRR17520069.8363958
SRR17520069.8384140
SRR17520069.8432833
SRR17520069.8448012
SRR17520069.8448013
SRR17520069.8448014
SRR17520069.8448015
SRR17520069.8448016
SRR17520069.8448017
SRR17520069.8448018
SRR17520069.8448019
SRR17520069.8448020
SRR17520069.8467196
SRR17520069.8467221

SRR17520069.8467224
SRR17520069.8481327
SRR17520069.8486624
SRR17520069.8486625
SRR17520069.8486626
SRR17520069.8486627
SRR17520069.8489450
SRR17520069.8500959
SRR17520069.8500960
SRR17520069.8522653
SRR17520069.8552646
SRR17520069.8564353
SRR17520069.8565976
SRR17520069.8585900
SRR17520069.8603842
SRR17520069.8608052
SRR17520069.8608053
SRR17520069.8608054
SRR17520069.8608055
SRR17520069.8622244
SRR17520069.8622245
SRR17520069.8622246
SRR17520069.8622247
SRR17520069.8622248
SRR17520069.8622249
SRR17520069.8622250
SRR17520069.8622251
SRR17520069.8622252
SRR17520069.8622253
SRR17520069.8622254
SRR17520069.8622255
SRR17520069.8622256
SRR17520069.8622257
SRR17520069.8622258
SRR17520069.8622259
SRR17520069.8622260
SRR17520069.8622261
SRR17520069.8630198
SRR17520069.8648482
SRR17520069.8648489
SRR17520069.8648492
SRR17520069.8648509
SRR17520069.8648513
SRR17520069.8648518
SRR17520069.8648521
SRR17520069.8648532
SRR17520069.8648537

SRR17520069.8648552
SRR17520069.8648560
SRR17520069.8648568
SRR17520069.8648572
SRR17520069.8662454
SRR17520069.8698650
SRR17520069.8698651
SRR17520069.8698652
SRR17520069.8698655
SRR17520069.8698656
SRR17520069.8698657
SRR17520069.8698659
SRR17520069.8698660
SRR17520069.8698661
SRR17520069.8698662
SRR17520069.8698663
SRR17520069.8698664
SRR17520069.8698665
SRR17520069.8698666
SRR17520069.8698667
SRR17520069.8698668
SRR17520069.8698669
SRR17520069.8698670
SRR17520069.8698671
SRR17520069.8698673
SRR17520069.8698674
SRR17520069.8698675
SRR17520069.8698677
SRR17520069.8698679
SRR17520069.8698680
SRR17520069.8698681
SRR17520069.8698682
SRR17520069.8698683
SRR17520069.8698685
SRR17520069.8698686
SRR17520069.8698687
SRR17520069.8698688
SRR17520069.8698689
SRR17520069.8698690
SRR17520069.8698691
SRR17520069.8698692
SRR17520069.8698693
SRR17520069.8698695
SRR17520069.8698696
SRR17520069.8698697
SRR17520069.8698698
SRR17520069.8698699

SRR17520069.8698700
SRR17520069.8698701
SRR17520069.8698702
SRR17520069.8698703
SRR17520069.8698704
SRR17520069.8698705
SRR17520069.8698706
SRR17520069.8698707
SRR17520069.8698708
SRR17520069.8700589
SRR17520069.8716172
SRR17520069.8716173
SRR17520069.8716174
SRR17520069.8716175
SRR17520069.8716176
SRR17520069.8716177
SRR17520069.8716178
SRR17520069.8716179
SRR17520069.8716180
SRR17520069.8716181
SRR17520069.8716182
SRR17520069.8716183
SRR17520069.8716184
SRR17520069.8716185
SRR17520069.8716186
SRR17520069.8716187
SRR17520069.8716188
SRR17520069.8716189
SRR17520069.8716190
SRR17520069.8716191
SRR17520069.8716192
SRR17520069.8716193
SRR17520069.8716194
SRR17520069.8716195
SRR17520069.8716196
SRR17520069.8716197
SRR17520069.8716198
SRR17520069.8716199
SRR17520069.8716200
SRR17520069.8716201
SRR17520069.8716202
SRR17520069.8716203
SRR17520069.8716204
SRR17520069.8716205
SRR17520069.8716206
SRR17520069.8720353
SRR17520069.8723273

SRR17520069.8723280
SRR17520069.8723281
SRR17520069.8723289
SRR17520069.8723293
SRR17520069.8723295
SRR17520069.8723303
SRR17520069.8723304
SRR17520069.8723307
SRR17520069.8723309
SRR17520069.8723311
SRR17520069.8723313
SRR17520069.8723315
SRR17520069.8723316
SRR17520069.8723321
SRR17520069.8723329
SRR17520069.8723333
SRR17520069.8723334
SRR17520069.8723339
SRR17520069.8723347
SRR17520069.8723353
SRR17520069.8723356
SRR17520069.8723359
SRR17520069.8723361
SRR17520069.8723363
SRR17520069.8723366
SRR17520069.8723371
SRR17520069.8723377
SRR17520069.8723379
SRR17520069.8723393
SRR17520069.8723394
SRR17520069.8723397
SRR17520069.8723407
SRR17520069.8723409
SRR17520069.8723412
SRR17520069.8723417
SRR17520069.8723418
SRR17520069.8731477
SRR17520069.8758708
SRR17520069.8798213
SRR17520069.8861510
SRR17520069.8861522
SRR17520069.8910636
SRR17520069.8922573
SRR17520069.8933422
SRR17520069.8945337
SRR17520069.8946677
SRR17520069.8974379

SRR17520069.8974383
SRR17520069.8974397
SRR17520069.8975867
SRR17520069.9006238
SRR17520069.9008658
SRR17520069.9026812
SRR17520069.9068189
SRR17520069.9081341
SRR17520069.9081394
SRR17520069.9083297
SRR17520069.9084276
SRR17520069.9108941
SRR17520069.9140605
SRR17520069.9165062
SRR17520069.9165063
SRR17520069.9165923
SRR17520069.9165924
SRR17520069.9165925
SRR17520069.9165926
SRR17520069.9166985
SRR17520069.9166999
SRR17520069.9170568
SRR17520069.9174243
SRR17520069.9198294
SRR17520069.9198331
SRR17520069.9202214
SRR17520069.9202218
SRR17520069.9205571
SRR17520069.9205705
SRR17520069.9248646
SRR17520069.9248654
SRR17520069.9250878
SRR17520069.9250879
SRR17520069.9250880
SRR17520069.9250881
SRR17520069.9251427
SRR17520069.9274531
SRR17520069.9279483
SRR17520069.9279489
SRR17520069.9284868
SRR17520069.9288341
SRR17520069.9305432
SRR17520069.9314095
SRR17520069.9340559
SRR17520069.9342985
SRR17520069.9342986
SRR17520069.9342987

SRR17520069.9342988
SRR17520069.9342989
SRR17520069.9342991
SRR17520069.9342992
SRR17520069.9342993
SRR17520069.9342994
SRR17520069.9342995
SRR17520069.9342996
SRR17520069.9342997
SRR17520069.9342998
SRR17520069.9342999
SRR17520069.9354497
SRR17520069.9371761
SRR17520069.9390979
SRR17520069.9442867
SRR17520069.9442868
SRR17520069.9442869
SRR17520069.9442870
SRR17520069.9442871
SRR17520069.9442872
SRR17520069.9442873
SRR17520069.9442874
SRR17520069.9466561
SRR17520069.9471268
SRR17520069.9479132
SRR17520069.9491423
SRR17520069.9492325
SRR17520069.9498837
SRR17520069.9498849
SRR17520069.9500232
SRR17520069.9500233
SRR17520069.9500234
SRR17520069.9500235
SRR17520069.9500236
SRR17520069.9500237
SRR17520069.9500238
SRR17520069.9500239
SRR17520069.9500240
SRR17520069.9500241
SRR17520069.9500242
SRR17520069.9500243
SRR17520069.9500244
SRR17520069.9500245
SRR17520069.9500246
SRR17520069.9500247
SRR17520069.9500248
SRR17520069.9500249

SRR17520069.9500250
SRR17520069.9500251
SRR17520069.9500252
SRR17520069.9500253
SRR17520069.9500254
SRR17520069.9500255
SRR17520069.9500256
SRR17520069.9500257
SRR17520069.9500258
SRR17520069.9500259
SRR17520069.9500260
SRR17520069.9500261
SRR17520069.9500262
SRR17520069.9500263
SRR17520069.9500264
SRR17520069.9500265
SRR17520069.9500266
SRR17520069.9500267
SRR17520069.9500268
SRR17520069.9500269
SRR17520069.9500270
SRR17520069.9500271
SRR17520069.9500272
SRR17520069.9500273
SRR17520069.9500274
SRR17520069.9500275
SRR17520069.9541783
SRR17520069.9587304
SRR17520069.9587306
SRR17520069.9587307
SRR17520069.9602451
SRR17520069.9602452
SRR17520069.9602453
SRR17520069.9602454
SRR17520069.9602455
SRR17520069.9602456
SRR17520069.9602457
SRR17520069.9602458
SRR17520069.9602459
SRR17520069.9602460
SRR17520069.9602461
SRR17520069.9602462
SRR17520069.9602463
SRR17520069.9602464
SRR17520069.9602465
SRR17520069.9602466
SRR17520069.9602467

SRR17520069.9602468
SRR17520069.9602469
SRR17520069.9602470
SRR17520069.9602471
SRR17520069.9602472
SRR17520069.9602473
SRR17520069.9602474
SRR17520069.9602475
SRR17520069.9602476
SRR17520069.9602477
SRR17520069.9602478
SRR17520069.9602479
SRR17520069.9602480
SRR17520069.9602481
SRR17520069.9602482
SRR17520069.9603974
SRR17520069.9648596
SRR17520069.9648600
SRR17520069.9648604
SRR17520069.9648612
SRR17520069.9648622
SRR17520069.9650856
SRR17520069.9657916
SRR17520069.9663051
SRR17520069.9677008
SRR17520069.9699149
SRR17520069.9731257
SRR17520069.9742322
SRR17520069.9742324
SRR17520069.9742357
SRR17520069.9742363
SRR17520069.9742372
SRR17520069.9742374
SRR17520069.9742417
SRR17520069.9742490
SRR17520069.9742922
SRR17520069.9755629
SRR17520069.9760733
SRR17520069.9776800
SRR17520069.9776801
SRR17520069.9776802
SRR17520069.9776803
SRR17520069.9776804
SRR17520069.9776805
SRR17520069.9776806
SRR17520069.9776807
SRR17520069.9776808

SRR17520069.9776809
SRR17520069.9776810
SRR17520069.9776811
SRR17520069.9776812
SRR17520069.9776813
SRR17520069.9776814
SRR17520069.9776815
SRR17520069.9776816
SRR17520069.9776817
SRR17520069.9776818
SRR17520069.9776819
SRR17520069.9776820
SRR17520069.9776821
SRR17520069.9776822
SRR17520069.9776823
SRR17520069.9783885
SRR17520069.9802345
SRR17520069.9802365
SRR17520069.9854890
SRR17520069.9854904
SRR17520069.9888889
SRR17520069.9892970
SRR17520069.9905584
SRR17520069.9934559
SRR17520069.9937600
SRR17520069.9956200
SRR17520069.9956201
SRR17520069.9956202
SRR17520069.9956203
SRR17520069.9956204
SRR17520069.9956205
SRR17520069.9956206
SRR17520069.9956207
SRR17520069.9956208
SRR17520069.9956209
SRR17520069.9956210
SRR17520069.9956211
SRR17520069.9956212
SRR17520069.9956213
SRR17520069.9956214
SRR17520069.9956215
SRR17520069.9956216
SRR17520069.9956217
SRR17520069.9956218
SRR17520069.9956219
SRR17520069.9956220
SRR17520069.9956221

SRR17520069.9956222
SRR17520069.9956223
SRR17520069.9956224
SRR17520069.9956225
SRR17520069.9956226
SRR17520069.9956227
SRR17520069.9956228
SRR17520069.9956229
SRR17520069.9956230
SRR17520069.9956231
SRR17520069.9956232
SRR17520069.9956233
SRR17520069.9956234
SRR17520069.9956235
SRR17520069.9956236
SRR17520069.9956237
SRR17520069.9956238
SRR17520069.9956239
SRR17520069.9956240
SRR17520069.9956241
SRR17520069.9956242
SRR17520069.9956243
SRR17520069.9956244
SRR17520069.9956245
SRR17520069.9956246
SRR17520069.9956247
SRR17520069.9956249
SRR17520069.9956250
SRR17520069.9956251
SRR17520069.9956252
SRR17520069.9956253
SRR17520069.9956254
SRR17520069.9956255
SRR17520069.9956256
SRR17520069.9956257
SRR17520069.9956258
SRR17520069.9956259
SRR17520069.9956260
SRR17520069.9956261
SRR17520069.9956262
SRR17520069.9956263
SRR17520069.9956264
SRR17520069.9956265
SRR17520069.9956266
SRR17520069.9956267
SRR17520069.9956268
SRR17520069.9956269

SRR17520069.9956270
SRR17520069.9956271
SRR17520069.9956272
SRR17520069.9956273
SRR17520069.9956274
SRR17520069.9956275
SRR17520069.9956276
SRR17520069.9956277
SRR17520069.9956278
SRR17520069.9956279
SRR17520069.9956280
SRR17520069.9956281
SRR17520069.9956282
SRR17520069.9956283
SRR17520069.9956284
SRR17520069.9956285
SRR17520069.9956286
SRR17520069.9956287
SRR17520069.9956288
SRR17520069.9956289
SRR17520069.9956290
SRR17520069.9956292
SRR17520069.9956293
SRR17520069.9956294
SRR17520069.9956295
SRR17520069.9956297
SRR17520069.9956298
SRR17520069.9956299
SRR17520069.9956300
SRR17520069.9956301
SRR17520069.9956302
SRR17520069.9956303
SRR17520069.9956304
SRR17520069.9956305
SRR17520069.9956306
SRR17520069.9956307
SRR17520069.9956308
SRR17520069.9956310
SRR17520069.9956311
SRR17520069.9956312
SRR17520069.9956313
SRR17520069.9956314
SRR17520069.9956315
SRR17520069.9956316
SRR17520069.9956317
SRR17520069.9956318
SRR17520069.9956319

SRR17520069.9956320
SRR17520069.9956321
SRR17520069.9956322
SRR17520069.9956323
SRR17520069.9956324
SRR17520069.9956325
SRR17520069.9971761
SRR17520069.9980593
SRR17520069.10014143
SRR17520069.10017982
SRR17520069.10033017
SRR17520069.10039973
SRR17520069.10051009
SRR17520069.10053117
SRR17520069.10057245
SRR17520069.10081829
SRR17520069.10088858
SRR17520069.10120431
SRR17520069.10120433
SRR17520069.10120434
SRR17520069.10120435
SRR17520069.10120436
SRR17520069.10120437
SRR17520069.10120438
SRR17520069.10120439
SRR17520069.10120440
SRR17520069.10120441
SRR17520069.10120442
SRR17520069.10120443
SRR17520069.10120444
SRR17520069.10128971
SRR17520069.10146119
SRR17520069.10146120
SRR17520069.10146121
SRR17520069.10146122
SRR17520069.10146124
SRR17520069.10146125
SRR17520069.10146126
SRR17520069.10146127
SRR17520069.10146128
SRR17520069.10146129
SRR17520069.10146130
SRR17520069.10146131
SRR17520069.10146132
SRR17520069.10146133
SRR17520069.10146134
SRR17520069.10146136

SRR17520069.10146137
SRR17520069.10146139
SRR17520069.10146140
SRR17520069.10146141
SRR17520069.10146142
SRR17520069.10146143
SRR17520069.10146144
SRR17520069.10146145
SRR17520069.10146146
SRR17520069.10146147
SRR17520069.10146148
SRR17520069.10146149
SRR17520069.10146150
SRR17520069.10147602
SRR17520069.10147611
SRR17520069.10147622
SRR17520069.10154871
SRR17520069.10247605
SRR17520069.10247606
SRR17520069.10247607
SRR17520069.10247608
SRR17520069.10247609
SRR17520069.10247610
SRR17520069.10247611
SRR17520069.10247612
SRR17520069.10247613
SRR17520069.10247614
SRR17520069.10247615
SRR17520069.10247616
SRR17520069.10299860
SRR17520069.10305978
SRR17520069.10305985
SRR17520069.10315375
SRR17520069.10316729
SRR17520069.10317665
SRR17520069.10317676
SRR17520069.10327150
SRR17520069.10336223
SRR17520069.10336224
SRR17520069.10336225
SRR17520069.10336226
SRR17520069.10336227
SRR17520069.10336228
SRR17520069.10336229
SRR17520069.10336230
SRR17520069.10336231
SRR17520069.10336232

SRR17520069.10336233
SRR17520069.10336234
SRR17520069.10336235
SRR17520069.10336236
SRR17520069.10336237
SRR17520069.10336238
SRR17520069.10336239
SRR17520069.10336240
SRR17520069.10336241
SRR17520069.10336242
SRR17520069.10336243
SRR17520069.10336244
SRR17520069.10336245
SRR17520069.10336246
SRR17520069.10336247
SRR17520069.10336248
SRR17520069.10336249
SRR17520069.10336250
SRR17520069.10336251
SRR17520069.10336252
SRR17520069.10336253
SRR17520069.10336254
SRR17520069.10336255
SRR17520069.10336256
SRR17520069.10336258
SRR17520069.10336259
SRR17520069.10336260
SRR17520069.10336262
SRR17520069.10336263
SRR17520069.10336264
SRR17520069.10336265
SRR17520069.10336266
SRR17520069.10336267
SRR17520069.10336268
SRR17520069.10336269
SRR17520069.10336270
SRR17520069.10336271
SRR17520069.10336273
SRR17520069.10336274
SRR17520069.10336275
SRR17520069.10336276
SRR17520069.10336277
SRR17520069.10339159
SRR17520069.10378588
SRR17520069.10378602
SRR17520069.10382222
SRR17520069.10394678

SRR17520069.10394679
SRR17520069.10394680
SRR17520069.10394681
SRR17520069.10394682
SRR17520069.10394683
SRR17520069.10394684
SRR17520069.10394685
SRR17520069.10394686
SRR17520069.10394687
SRR17520069.10394688
SRR17520069.10394689
SRR17520069.10394690
SRR17520069.10394691
SRR17520069.10394692
SRR17520069.10394693
SRR17520069.10394694
SRR17520069.10394695
SRR17520069.10394696
SRR17520069.10394697
SRR17520069.10394698
SRR17520069.10394699
SRR17520069.10394700
SRR17520069.10394701
SRR17520069.10394702
SRR17520069.10394703
SRR17520069.10394704
SRR17520069.10394705
SRR17520069.10394706
SRR17520069.10394707
SRR17520069.10394708
SRR17520069.10394709
SRR17520069.10394710
SRR17520069.10394711
SRR17520069.10394712
SRR17520069.10394713
SRR17520069.10394714
SRR17520069.10394715
SRR17520069.10422271
SRR17520069.10422283
SRR17520069.10431196
SRR17520069.10456441
SRR17520069.10476839
SRR17520069.10476860
SRR17520069.10487884
SRR17520069.10487885
SRR17520069.10487886
SRR17520069.10487887

SRR17520069.10513296
SRR17520069.10518614
SRR17520069.10551083
SRR17520069.10560064
SRR17520069.10562404
SRR17520069.10568322
SRR17520069.10593951
SRR17520069.10593952
SRR17520069.10593957
SRR17520069.10593958
SRR17520069.10593971
SRR17520069.10593988
SRR17520069.10593991
SRR17520069.10593992
SRR17520069.10594013
SRR17520069.10594018
SRR17520069.10599961
SRR17520069.10602410
SRR17520069.10602411
SRR17520069.10602412
SRR17520069.10602413
SRR17520069.10602414
SRR17520069.10602415
SRR17520069.10602416
SRR17520069.10602417
SRR17520069.10602418
SRR17520069.10602419
SRR17520069.10602420
SRR17520069.10602421
SRR17520069.10602422
SRR17520069.10602423
SRR17520069.10602424
SRR17520069.10602425
SRR17520069.10602426
SRR17520069.10602427
SRR17520069.10602428
SRR17520069.10602429
SRR17520069.10602430
SRR17520069.10602431
SRR17520069.10602432
SRR17520069.10602433
SRR17520069.10602434
SRR17520069.10602435
SRR17520069.10602436
SRR17520069.10602437
SRR17520069.10602438
SRR17520069.10602439

SRR17520069.10602441
SRR17520069.10602442
SRR17520069.10602443
SRR17520069.10602444
SRR17520069.10602445
SRR17520069.10602446
SRR17520069.10602448
SRR17520069.10611032
SRR17520069.10611033
SRR17520069.10611034
SRR17520069.10611035
SRR17520069.10611036
SRR17520069.10611037
SRR17520069.10611038
SRR17520069.10611039
SRR17520069.10611040
SRR17520069.10611041
SRR17520069.10611042
SRR17520069.10611043
SRR17520069.10611044
SRR17520069.10611045
SRR17520069.10611046
SRR17520069.10611047
SRR17520069.10611048
SRR17520069.10611049
SRR17520069.10611050
SRR17520069.10611051
SRR17520069.10611052
SRR17520069.10611053
SRR17520069.10611054
SRR17520069.10611055
SRR17520069.10611056
SRR17520069.10611057
SRR17520069.10611058
SRR17520069.10611059
SRR17520069.10611060
SRR17520069.10611061
SRR17520069.10611062
SRR17520069.10611063
SRR17520069.10611064
SRR17520069.10611065
SRR17520069.10611066
SRR17520069.10611067
SRR17520069.10611068
SRR17520069.10611069
SRR17520069.10611070
SRR17520069.10611071

SRR17520069.10611072
SRR17520069.10611073
SRR17520069.10611074
SRR17520069.10611075
SRR17520069.10611076
SRR17520069.10611077
SRR17520069.10611078
SRR17520069.10611079
SRR17520069.10611080
SRR17520069.10611082
SRR17520069.10611083
SRR17520069.10611084
SRR17520069.10611085
SRR17520069.10611086
SRR17520069.10611087
SRR17520069.10611088
SRR17520069.10611089
SRR17520069.10611090
SRR17520069.10611091
SRR17520069.10611092
SRR17520069.10611093
SRR17520069.10611094
SRR17520069.10611095
SRR17520069.10611096
SRR17520069.10611097
SRR17520069.10611098
SRR17520069.10611099
SRR17520069.10611100
SRR17520069.10611101
SRR17520069.10611102
SRR17520069.10611103
SRR17520069.10611104
SRR17520069.10614385
SRR17520069.10633050
SRR17520069.10633051
SRR17520069.10633052
SRR17520069.10633053
SRR17520069.10633054
SRR17520069.10633055
SRR17520069.10633056
SRR17520069.10633057
SRR17520069.10633058
SRR17520069.10633059
SRR17520069.10633060
SRR17520069.10633061
SRR17520069.10633062
SRR17520069.10633063

SRR17520069.10633064
SRR17520069.10633065
SRR17520069.10633066
SRR17520069.10633067
SRR17520069.10633068
SRR17520069.10633069
SRR17520069.10633070
SRR17520069.10633071
SRR17520069.10633072
SRR17520069.10633073
SRR17520069.10637731
SRR17520069.10657797
SRR17520069.10657798
SRR17520069.10657799
SRR17520069.10657800
SRR17520069.10657802
SRR17520069.10657803
SRR17520069.10657804
SRR17520069.10657805
SRR17520069.10657806
SRR17520069.10657807
SRR17520069.10657808
SRR17520069.10657809
SRR17520069.10657810
SRR17520069.10657811
SRR17520069.10657812
SRR17520069.10657813
SRR17520069.10657814
SRR17520069.10657815
SRR17520069.10657816
SRR17520069.10657817
SRR17520069.10657818
SRR17520069.10657819
SRR17520069.10657820
SRR17520069.10657821
SRR17520069.10657822
SRR17520069.10657823
SRR17520069.10657824
SRR17520069.10657825
SRR17520069.10657826
SRR17520069.10657827
SRR17520069.10657828
SRR17520069.10657829
SRR17520069.10662567
SRR17520069.10663615
SRR17520069.10663616
SRR17520069.10678461

SRR17520069.10678462
SRR17520069.10678463
SRR17520069.10678464
SRR17520069.10678465
SRR17520069.10678467
SRR17520069.10678468
SRR17520069.10678469
SRR17520069.10678470
SRR17520069.10678471
SRR17520069.10678472
SRR17520069.10678473
SRR17520069.10678474
SRR17520069.10678475
SRR17520069.10678476
SRR17520069.10678477
SRR17520069.10678478
SRR17520069.10678479
SRR17520069.10678480
SRR17520069.10678481
SRR17520069.10678482
SRR17520069.10678484
SRR17520069.10678485
SRR17520069.10678486
SRR17520069.10678487
SRR17520069.10678488
SRR17520069.10678489
SRR17520069.10678490
SRR17520069.10678491
SRR17520069.10678492
SRR17520069.10678493
SRR17520069.10678494
SRR17520069.10678495
SRR17520069.10678498
SRR17520069.10678499
SRR17520069.10678500
SRR17520069.10678501
SRR17520069.10678502
SRR17520069.10678503
SRR17520069.10678504
SRR17520069.10678505
SRR17520069.10678506
SRR17520069.10678507
SRR17520069.10678508
SRR17520069.10678509
SRR17520069.10678510
SRR17520069.10678511
SRR17520069.10678512

SRR17520069.10678513
SRR17520069.10678514
SRR17520069.10678515
SRR17520069.10678516
SRR17520069.10678517
SRR17520069.10678518
SRR17520069.10678519
SRR17520069.10678520
SRR17520069.10678521
SRR17520069.10678522
SRR17520069.10678523
SRR17520069.10678524
SRR17520069.10678525
SRR17520069.10678526
SRR17520069.10678527
SRR17520069.10678528
SRR17520069.10678529
SRR17520069.10678530
SRR17520069.10678531
SRR17520069.10678532
SRR17520069.10678533
SRR17520069.10678534
SRR17520069.10678535
SRR17520069.10678536
SRR17520069.10678537
SRR17520069.10678538
SRR17520069.10678539
SRR17520069.10678540
SRR17520069.10678541
SRR17520069.10678542
SRR17520069.10678543
SRR17520069.10678544
SRR17520069.10678545
SRR17520069.10678546
SRR17520069.10678547
SRR17520069.10678548
SRR17520069.10678549
SRR17520069.10678550
SRR17520069.10678551
SRR17520069.10678552
SRR17520069.10678553
SRR17520069.10678554
SRR17520069.10678555
SRR17520069.10678556
SRR17520069.10678557
SRR17520069.10678558
SRR17520069.10678559

SRR17520069.10678560
SRR17520069.10678561
SRR17520069.10678562
SRR17520069.10678563
SRR17520069.10678564
SRR17520069.10678565
SRR17520069.10678566
SRR17520069.10678567
SRR17520069.10678568
SRR17520069.10678569
SRR17520069.10678570
SRR17520069.10678571
SRR17520069.10678572
SRR17520069.10678573
SRR17520069.10678574
SRR17520069.10678575
SRR17520069.10678576
SRR17520069.10678578
SRR17520069.10678579
SRR17520069.10678580
SRR17520069.10678581
SRR17520069.10678582
SRR17520069.10678583
SRR17520069.10678584
SRR17520069.10678585
SRR17520069.10678586
SRR17520069.10678587
SRR17520069.10678588
SRR17520069.10690050
SRR17520069.10717160
SRR17520069.10717161
SRR17520069.10717162
SRR17520069.10717163
SRR17520069.10717164
SRR17520069.10717165
SRR17520069.10717166
SRR17520069.10717167
SRR17520069.10717168
SRR17520069.10717169
SRR17520069.10717170
SRR17520069.10717171
SRR17520069.10717172
SRR17520069.10717173
SRR17520069.10717174
SRR17520069.10717175
SRR17520069.10717176
SRR17520069.10717177

SRR17520069.10717178
SRR17520069.10717179
SRR17520069.10717180
SRR17520069.10717181
SRR17520069.10717182
SRR17520069.10717183
SRR17520069.10717186
SRR17520069.10717187
SRR17520069.10717188
SRR17520069.10717189
SRR17520069.10717190
SRR17520069.10717191
SRR17520069.10717192
SRR17520069.10717193
SRR17520069.10717194
SRR17520069.10717195
SRR17520069.10717196
SRR17520069.10717197
SRR17520069.10717198
SRR17520069.10717199
SRR17520069.10717200
SRR17520069.10717201
SRR17520069.10717202
SRR17520069.10717203
SRR17520069.10717204
SRR17520069.10717205
SRR17520069.10717206
SRR17520069.10717207
SRR17520069.10717208
SRR17520069.10721283
SRR17520069.10744089
SRR17520069.10747969
SRR17520069.10768641
SRR17520069.10774734
SRR17520069.10809808
SRR17520069.10813511
SRR17520069.10813512
SRR17520069.10813514
SRR17520069.10813515
SRR17520069.10813517
SRR17520069.10813518
SRR17520069.10813519
SRR17520069.10813520
SRR17520069.10813521
SRR17520069.10813522
SRR17520069.10813523
SRR17520069.10813524

SRR17520069.10813525
SRR17520069.10813526
SRR17520069.10813527
SRR17520069.10813529
SRR17520069.10813530
SRR17520069.10813531
SRR17520069.10813532
SRR17520069.10813533
SRR17520069.10813534
SRR17520069.10813535
SRR17520069.10813536
SRR17520069.10813537
SRR17520069.10813538
SRR17520069.10813539
SRR17520069.10813540
SRR17520069.10813613
SRR17520069.10823064
SRR17520069.10823065
SRR17520069.10823066
SRR17520069.10823067
SRR17520069.10823068
SRR17520069.10823070
SRR17520069.10823071
SRR17520069.10823072
SRR17520069.10823073
SRR17520069.10823074
SRR17520069.10823075
SRR17520069.10823076
SRR17520069.10823077
SRR17520069.10823078
SRR17520069.10823079
SRR17520069.10823080
SRR17520069.10823081
SRR17520069.10823082
SRR17520069.10823083
SRR17520069.10823084
SRR17520069.10823085
SRR17520069.10823086
SRR17520069.10823087
SRR17520069.10823088
SRR17520069.10823089
SRR17520069.10823090
SRR17520069.10823091
SRR17520069.10823092
SRR17520069.10823093
SRR17520069.10823094
SRR17520069.10823095

SRR17520069.10823096
SRR17520069.10823097
SRR17520069.10823098
SRR17520069.10841600
SRR17520069.10841604
SRR17520069.10852306
SRR17520069.10853023
SRR17520069.10859994
SRR17520069.10859995
SRR17520069.10859997
SRR17520069.10859998
SRR17520069.10859999
SRR17520069.10860000
SRR17520069.10860002
SRR17520069.10860003
SRR17520069.10860004
SRR17520069.10860005
SRR17520069.10860006
SRR17520069.10860007
SRR17520069.10860008
SRR17520069.10860009
SRR17520069.10860010
SRR17520069.10860011
SRR17520069.10860012
SRR17520069.10860013
SRR17520069.10860014
SRR17520069.10860015
SRR17520069.10860016
SRR17520069.10860017
SRR17520069.10860018
SRR17520069.10860019
SRR17520069.10860020
SRR17520069.10860022
SRR17520069.10860023
SRR17520069.10860024
SRR17520069.10860025
SRR17520069.10860026
SRR17520069.10860027
SRR17520069.10860028
SRR17520069.10860030
SRR17520069.10860031
SRR17520069.10860032
SRR17520069.10861853
SRR17520069.10877026
SRR17520069.10884905
SRR17520069.10921425
SRR17520069.10967381

SRR17520069.10971801
SRR17520069.10980443
SRR17520069.10988107
SRR17520069.11003520
SRR17520069.11003521
SRR17520069.11003522
SRR17520069.11009249
SRR17520069.11011758
SRR17520069.11015019
SRR17520069.11017349
SRR17520069.11017350
SRR17520069.11017351
SRR17520069.11017352
SRR17520069.11017354
SRR17520069.11017355
SRR17520069.11017356
SRR17520069.11017357
SRR17520069.11017358
SRR17520069.11017360
SRR17520069.11017361
SRR17520069.11017362
SRR17520069.11017363
SRR17520069.11017364
SRR17520069.11017365
SRR17520069.11017366
SRR17520069.11017368
SRR17520069.11017369
SRR17520069.11018136
SRR17520069.11037224
SRR17520069.11037225
SRR17520069.11037226
SRR17520069.11046021
SRR17520069.11046025
SRR17520069.11076057
SRR17520069.11091938
SRR17520069.11103989
SRR17520069.11115915
SRR17520069.11115916
SRR17520069.11115917
SRR17520069.11115918
SRR17520069.11115919
SRR17520069.11115920
SRR17520069.11115921
SRR17520069.11115922
SRR17520069.11115923
SRR17520069.11115924
SRR17520069.11115925

SRR17520069.11115926
SRR17520069.11115927
SRR17520069.11115928
SRR17520069.11115929
SRR17520069.11115930
SRR17520069.11115931
SRR17520069.11115932
SRR17520069.11115933
SRR17520069.11115934
SRR17520069.11115935
SRR17520069.11115936
SRR17520069.11115937
SRR17520069.11115938
SRR17520069.11115939
SRR17520069.11115940
SRR17520069.11115941
SRR17520069.11115942
SRR17520069.11115943
SRR17520069.11115944
SRR17520069.11115945
SRR17520069.11115946
SRR17520069.11115947
SRR17520069.11115948
SRR17520069.11115949
SRR17520069.11118373
SRR17520069.11118956
SRR17520069.11122278
SRR17520069.11144491
SRR17520069.11155512
SRR17520069.11155513
SRR17520069.11155514
SRR17520069.11155515
SRR17520069.11155516
SRR17520069.11155517
SRR17520069.11155518
SRR17520069.11155519
SRR17520069.11155520
SRR17520069.11155521
SRR17520069.11155522
SRR17520069.11155523
SRR17520069.11155524
SRR17520069.11155525
SRR17520069.11155526
SRR17520069.11155527
SRR17520069.11155528
SRR17520069.11155529
SRR17520069.11155530

SRR17520069.11155531
SRR17520069.11155532
SRR17520069.11155533
SRR17520069.11155534
SRR17520069.11155535
SRR17520069.11155536
SRR17520069.11155537
SRR17520069.11155538
SRR17520069.11155539
SRR17520069.11155540
SRR17520069.11188048
SRR17520069.11216324
SRR17520069.11241824
SRR17520069.11300379
SRR17520069.11300400
SRR17520069.11300408
SRR17520069.11331339
SRR17520069.11331404
SRR17520069.11349339
SRR17520069.11349452
SRR17520069.11379106
SRR17520069.11383461
SRR17520069.11415099
SRR17520069.11415174
SRR17520069.11425710
SRR17520069.11433268
SRR17520069.11433295
SRR17520069.11434280
SRR17520069.11458274
SRR17520069.11458277
SRR17520069.11458278
SRR17520069.11458279
SRR17520069.11458280
SRR17520069.11458281
SRR17520069.11458282
SRR17520069.11458283
SRR17520069.11458284
SRR17520069.11458285
SRR17520069.11458286
SRR17520069.11458287
SRR17520069.11458288
SRR17520069.11458289
SRR17520069.11458290
SRR17520069.11458291
SRR17520069.11458292
SRR17520069.11458293
SRR17520069.11458294

SRR17520069.11458295
SRR17520069.11458296
SRR17520069.11458297
SRR17520069.11458298
SRR17520069.11458299
SRR17520069.11458300
SRR17520069.11458301
SRR17520069.11458302
SRR17520069.11458303
SRR17520069.11458304
SRR17520069.11458305
SRR17520069.11458306
SRR17520069.11458307
SRR17520069.11458308
SRR17520069.11458309
SRR17520069.11458310
SRR17520069.11458311
SRR17520069.11458312
SRR17520069.11458313
SRR17520069.11458314
SRR17520069.11458315
SRR17520069.11458316
SRR17520069.11458317
SRR17520069.11458318
SRR17520069.11458319
SRR17520069.11458320
SRR17520069.11458321
SRR17520069.11458322
SRR17520069.11458323
SRR17520069.11458324
SRR17520069.11458325
SRR17520069.11458326
SRR17520069.11458327
SRR17520069.11458328
SRR17520069.11473791
SRR17520069.11473792
SRR17520069.11473793
SRR17520069.11473794
SRR17520069.11473795
SRR17520069.11473796
SRR17520069.11473797
SRR17520069.11473798
SRR17520069.11473799
SRR17520069.11509194
SRR17520069.11509259
SRR17520069.11569395
SRR17520069.11587693

SRR17520069.11603566
SRR17520069.11608166
SRR17520069.11624721
SRR17520069.11638979
SRR17520069.11661271
SRR17520069.11672039
SRR17520069.11708043
SRR17520069.11736312
SRR17520069.11789946
SRR17520069.11790252
SRR17520069.11790254
SRR17520069.11823684
SRR17520069.11865001
SRR17520069.11882468
SRR17520069.11883711
SRR17520069.11924585
SRR17520069.11924598
SRR17520069.11945268
SRR17520069.11946388
SRR17520069.11982609
SRR17520069.12029217
SRR17520069.12042549
SRR17520069.12051763
SRR17520069.12051771
SRR17520069.12051775
SRR17520069.12051777
SRR17520069.12051783
SRR17520069.12051785
SRR17520069.12051787
SRR17520069.12051789
SRR17520069.12051792
SRR17520069.12051795
SRR17520069.12051805
SRR17520069.12051813
SRR17520069.12051816
SRR17520069.12051823
SRR17520069.12051829
SRR17520069.12051835
SRR17520069.12051838
SRR17520069.12051839
SRR17520069.12051849
SRR17520069.12051853
SRR17520069.12051857
SRR17520069.12051859
SRR17520069.12068051
SRR17520069.12079242
SRR17520069.12102348

SRR17520069.12110454
SRR17520069.12117639
SRR17520069.12129086
SRR17520069.12155209
SRR17520069.12155212
SRR17520069.12155213
SRR17520069.12155214
SRR17520069.12155215
SRR17520069.12155216
SRR17520069.12155217
SRR17520069.12155218
SRR17520069.12155219
SRR17520069.12155220
SRR17520069.12155221
SRR17520069.12155222
SRR17520069.12155223
SRR17520069.12155224
SRR17520069.12155225
SRR17520069.12155228
SRR17520069.12155229
SRR17520069.12155231
SRR17520069.12155232
SRR17520069.12155233
SRR17520069.12155234
SRR17520069.12155235
SRR17520069.12155236
SRR17520069.12155237
SRR17520069.12155238
SRR17520069.12155239
SRR17520069.12155240
SRR17520069.12155242
SRR17520069.12155243
SRR17520069.12155244
SRR17520069.12155246
SRR17520069.12155247
SRR17520069.12155248
SRR17520069.12155249
SRR17520069.12173636
SRR17520069.12185065
SRR17520069.12185310
SRR17520069.12199849
SRR17520069.12201834
SRR17520069.12235055
SRR17520069.12245691
SRR17520069.12249097
SRR17520069.12249104
SRR17520069.12260606

SRR17520069.12260607
SRR17520069.12260608
SRR17520069.12260609
SRR17520069.12260610
SRR17520069.12260612
SRR17520069.12260614
SRR17520069.12260615
SRR17520069.12260617
SRR17520069.12260618
SRR17520069.12260619
SRR17520069.12260621
SRR17520069.12260622
SRR17520069.12260624
SRR17520069.12260625
SRR17520069.12260626
SRR17520069.12260627
SRR17520069.12260628
SRR17520069.12260629
SRR17520069.12260630
SRR17520069.12260631
SRR17520069.12260632
SRR17520069.12260633
SRR17520069.12260634
SRR17520069.12260635
SRR17520069.12260636
SRR17520069.12260637
SRR17520069.12260638
SRR17520069.12260639
SRR17520069.12260640
SRR17520069.12260641
SRR17520069.12269740
SRR17520069.12289503
SRR17520069.12297497
SRR17520069.12303460
SRR17520069.12303464
SRR17520069.12316397
SRR17520069.12323276
SRR17520069.12326650
SRR17520069.12326651
SRR17520069.12326652
SRR17520069.12326653
SRR17520069.12326654
SRR17520069.12366029
SRR17520069.12448509
SRR17520069.12490007
SRR17520069.12507144
SRR17520069.12520025

SRR17520069.12520034
SRR17520069.12531043
SRR17520069.12535688
SRR17520069.12536820
SRR17520069.12565930
SRR17520069.12584595
SRR17520069.12665794
SRR17520069.12683610
SRR17520069.12683611
SRR17520069.12683612
SRR17520069.12683613
SRR17520069.12683614
SRR17520069.12683615
SRR17520069.12683616
SRR17520069.12683617
SRR17520069.12683618
SRR17520069.12683619
SRR17520069.12683620
SRR17520069.12683621
SRR17520069.12683622
SRR17520069.12683623
SRR17520069.12683624
SRR17520069.12683625
SRR17520069.12683626
SRR17520069.12683627
SRR17520069.12683628
SRR17520069.12683629
SRR17520069.12683630
SRR17520069.12683631
SRR17520069.12683632
SRR17520069.12683633
SRR17520069.12683634
SRR17520069.12683635
SRR17520069.12683636
SRR17520069.12683637
SRR17520069.12683638
SRR17520069.12683639
SRR17520069.12683640
SRR17520069.12683641
SRR17520069.12683642
SRR17520069.12683643
SRR17520069.12683644
SRR17520069.12683645
SRR17520069.12683646
SRR17520069.12683647
SRR17520069.12683648
SRR17520069.12683649

SRR17520069.12721559
SRR17520069.12731614
SRR17520069.12735510
SRR17520069.12767099
SRR17520069.12767107
SRR17520069.12785051
SRR17520069.12787909
SRR17520069.12820017
SRR17520069.12820018
SRR17520069.12820019
SRR17520069.12820020
SRR17520069.12820021
SRR17520069.12820022
SRR17520069.12820023
SRR17520069.12820025
SRR17520069.12820029
SRR17520069.12820030
SRR17520069.12820031
SRR17520069.12820032
SRR17520069.12820033
SRR17520069.12820034
SRR17520069.12820035
SRR17520069.12820036
SRR17520069.12820037
SRR17520069.12820038
SRR17520069.12820039
SRR17520069.12820040
SRR17520069.12820041
SRR17520069.12820042
SRR17520069.12820043
SRR17520069.12820044
SRR17520069.12820046
SRR17520069.12820047
SRR17520069.12820048
SRR17520069.12820049
SRR17520069.12820050
SRR17520069.12820051
SRR17520069.12820052
SRR17520069.12820053
SRR17520069.12820054
SRR17520069.12832898
SRR17520069.12832899
SRR17520069.12832900
SRR17520069.12832901
SRR17520069.12832902
SRR17520069.12832903
SRR17520069.12832904

SRR17520069.12832905
SRR17520069.12832906
SRR17520069.12832907
SRR17520069.12832908
SRR17520069.12832909
SRR17520069.12832910
SRR17520069.12832911
SRR17520069.12832912
SRR17520069.12832913
SRR17520069.12832914
SRR17520069.12832915
SRR17520069.12832916
SRR17520069.12832917
SRR17520069.12832918
SRR17520069.12832920
SRR17520069.12832921
SRR17520069.12832922
SRR17520069.12832923
SRR17520069.12832924
SRR17520069.12832925
SRR17520069.12832926
SRR17520069.12832927
SRR17520069.12832928
SRR17520069.12832929
SRR17520069.12832930
SRR17520069.12832931
SRR17520069.12832932
SRR17520069.12832933
SRR17520069.12832934
SRR17520069.12832935
SRR17520069.12832936
SRR17520069.12832937
SRR17520069.12832938
SRR17520069.12832939
SRR17520069.12832940
SRR17520069.12832941
SRR17520069.12832942
SRR17520069.12832943
SRR17520069.12832944
SRR17520069.12832945
SRR17520069.12832946
SRR17520069.12841050
SRR17520069.12854936
SRR17520069.12896345
SRR17520069.12897142
SRR17520069.12925413
SRR17520069.12925414

SRR17520069.12925415
SRR17520069.12925417
SRR17520069.12925419
SRR17520069.12925422
SRR17520069.12925423
SRR17520069.12925424
SRR17520069.12925425
SRR17520069.12925426
SRR17520069.12927564
SRR17520069.12932345
SRR17520069.12959823
SRR17520069.12965414
SRR17520069.12969490
SRR17520069.12985330
SRR17520069.13042829
SRR17520069.13042840
SRR17520069.13042850
SRR17520069.13042868
SRR17520069.13058177
SRR17520069.13058189
SRR17520069.13093109
SRR17520069.13093110
SRR17520069.13093111
SRR17520069.13093112
SRR17520069.13098028
SRR17520069.13126063
SRR17520069.13133177
SRR17520069.13141163
SRR17520069.13239884
SRR17520069.13244605
SRR17520069.13244606
SRR17520069.13244607
SRR17520069.13244608
SRR17520069.13244609
SRR17520069.13244610
SRR17520069.13244611
SRR17520069.13244612
SRR17520069.13244613
SRR17520069.13244614
SRR17520069.13244615
SRR17520069.13244616
SRR17520069.13244617
SRR17520069.13244618
SRR17520069.13244619
SRR17520069.13244620
SRR17520069.13244621
SRR17520069.13244622

SRR17520069.13244623
SRR17520069.13244624
SRR17520069.13244625
SRR17520069.13244626
SRR17520069.13244627
SRR17520069.13244628
SRR17520069.13244629
SRR17520069.13244630
SRR17520069.13244631
SRR17520069.13244634
SRR17520069.13244635
SRR17520069.13244636
SRR17520069.13244639
SRR17520069.13244640
SRR17520069.13244644
SRR17520069.13244645
SRR17520069.13244646
SRR17520069.13247089
SRR17520069.13249206
SRR17520069.13249207
SRR17520069.13249208
SRR17520069.13249209
SRR17520069.13249210
SRR17520069.13249211
SRR17520069.13249212
SRR17520069.13249213
SRR17520069.13249214
SRR17520069.13249215
SRR17520069.13249216
SRR17520069.13249217
SRR17520069.13249218
SRR17520069.13249219
SRR17520069.13249220
SRR17520069.13249221
SRR17520069.13249222
SRR17520069.13249223
SRR17520069.13249224
SRR17520069.13249225
SRR17520069.13249226
SRR17520069.13249227
SRR17520069.13249228
SRR17520069.13249229
SRR17520069.13249230
SRR17520069.13249231
SRR17520069.13249232
SRR17520069.13249233
SRR17520069.13249234

SRR17520069.13249235
SRR17520069.13249236
SRR17520069.13249237
SRR17520069.13249238
SRR17520069.13249239
SRR17520069.13249240
SRR17520069.13249241
SRR17520069.13249242
SRR17520069.13249243
SRR17520069.13249244
SRR17520069.13249245
SRR17520069.13249246
SRR17520069.13251722
SRR17520069.13261867
SRR17520069.13262906
SRR17520069.13262907
SRR17520069.13262908
SRR17520069.13262909
SRR17520069.13262910
SRR17520069.13262911
SRR17520069.13262912
SRR17520069.13262913
SRR17520069.13262914
SRR17520069.13262915
SRR17520069.13262916
SRR17520069.13262917
SRR17520069.13262918
SRR17520069.13262919
SRR17520069.13262920
SRR17520069.13262921
SRR17520069.13262922
SRR17520069.13262923
SRR17520069.13262924
SRR17520069.13262925
SRR17520069.13262926
SRR17520069.13262927
SRR17520069.13262928
SRR17520069.13262929
SRR17520069.13262930
SRR17520069.13262931
SRR17520069.13262932
SRR17520069.13262933
SRR17520069.13262934
SRR17520069.13262935
SRR17520069.13262936
SRR17520069.13262937
SRR17520069.13262938

SRR17520069.13262939
SRR17520069.13262940
SRR17520069.13262941
SRR17520069.13262942
SRR17520069.13262943
SRR17520069.13262944
SRR17520069.13262945
SRR17520069.13262947
SRR17520069.13262948
SRR17520069.13295871
SRR17520069.13295872
SRR17520069.13295874
SRR17520069.13295875
SRR17520069.13295876
SRR17520069.13295877
SRR17520069.13295878
SRR17520069.13295879
SRR17520069.13295880
SRR17520069.13295881
SRR17520069.13295882
SRR17520069.13295883
SRR17520069.13295885
SRR17520069.13297391
SRR17520069.13355987
SRR17520069.13400923
SRR17520069.13400932
SRR17520069.13400946
SRR17520069.13400954
SRR17520069.13400970
SRR17520069.13401002
SRR17520069.13412033
SRR17520069.13431890
SRR17520069.13431891
SRR17520069.13431892
SRR17520069.13431893
SRR17520069.13431894
SRR17520069.13431895
SRR17520069.13431896
SRR17520069.13431897
SRR17520069.13431898
SRR17520069.13431900
SRR17520069.13431901
SRR17520069.13431902
SRR17520069.13431903
SRR17520069.13431904
SRR17520069.13431905
SRR17520069.13431906

SRR17520069.13431907
SRR17520069.13431908
SRR17520069.13431909
SRR17520069.13431910
SRR17520069.13431912
SRR17520069.13431913
SRR17520069.13431914
SRR17520069.13431915
SRR17520069.13431916
SRR17520069.13431917
SRR17520069.13431918
SRR17520069.13431919
SRR17520069.13431920
SRR17520069.13431921
SRR17520069.13431922
SRR17520069.13431923
SRR17520069.13431924
SRR17520069.13431925
SRR17520069.13431926
SRR17520069.13431927
SRR17520069.13431928
SRR17520069.13431929
SRR17520069.13431930
SRR17520069.13431931
SRR17520069.13431932
SRR17520069.13448852
SRR17520069.13459753
SRR17520069.13471202
SRR17520069.13473199
SRR17520069.13477118
SRR17520069.13477812
SRR17520069.13484709
SRR17520069.13490901
SRR17520069.13511388
SRR17520069.13521645
SRR17520069.13521646
SRR17520069.13521647
SRR17520069.13526843
SRR17520069.13526844
SRR17520069.13526845
SRR17520069.13526846
SRR17520069.13526847
SRR17520069.13526848
SRR17520069.13526849
SRR17520069.13526850
SRR17520069.13526851
SRR17520069.13526852

SRR17520069.13526853
SRR17520069.13526855
SRR17520069.13526856
SRR17520069.13526857
SRR17520069.13526858
SRR17520069.13526859
SRR17520069.13526860
SRR17520069.13526861
SRR17520069.13526862
SRR17520069.13526863
SRR17520069.13526864
SRR17520069.13526865
SRR17520069.13526866
SRR17520069.13526867
SRR17520069.13526868
SRR17520069.13526869
SRR17520069.13526870
SRR17520069.13526871
SRR17520069.13550754
SRR17520069.13554998
SRR17520069.13564223
SRR17520069.13564224
SRR17520069.13564229
SRR17520069.13564230
SRR17520069.13564231
SRR17520069.13582699
SRR17520069.13592149
SRR17520069.13605833
SRR17520069.13606612
SRR17520069.13606613
SRR17520069.13606614
SRR17520069.13606615
SRR17520069.13606616
SRR17520069.13606617
SRR17520069.13606618
SRR17520069.13606619
SRR17520069.13606620
SRR17520069.13606621
SRR17520069.13606622
SRR17520069.13606623
SRR17520069.13606624
SRR17520069.13606625
SRR17520069.13606626
SRR17520069.13606627
SRR17520069.13606628
SRR17520069.13606629
SRR17520069.13606630

SRR17520069.13606631
SRR17520069.13606632
SRR17520069.13606633
SRR17520069.13606634
SRR17520069.13606635
SRR17520069.13606636
SRR17520069.13606637
SRR17520069.13606638
SRR17520069.13606639
SRR17520069.13606640
SRR17520069.13606641
SRR17520069.13606642
SRR17520069.13606643
SRR17520069.13606644
SRR17520069.13606645
SRR17520069.13606646
SRR17520069.13606789
SRR17520069.13654902
SRR17520069.13654903
SRR17520069.13654904
SRR17520069.13654905
SRR17520069.13654906
SRR17520069.13654907
SRR17520069.13654908
SRR17520069.13654909
SRR17520069.13654910
SRR17520069.13654911
SRR17520069.13654912
SRR17520069.13654913
SRR17520069.13654914
SRR17520069.13654915
SRR17520069.13654916
SRR17520069.13654917
SRR17520069.13654918
SRR17520069.13654919
SRR17520069.13654920
SRR17520069.13654921
SRR17520069.13654922
SRR17520069.13654923
SRR17520069.13654924
SRR17520069.13654925
SRR17520069.13658970
SRR17520069.13667520
SRR17520069.13683134
SRR17520069.13683137
SRR17520069.13683142
SRR17520069.13683146

SRR17520069.13685952
SRR17520069.13691457
SRR17520069.13691923
SRR17520069.13695689
SRR17520069.13698159
SRR17520069.13698160
SRR17520069.13698161
SRR17520069.13698162
SRR17520069.13698163
SRR17520069.13698164
SRR17520069.13698165
SRR17520069.13698166
SRR17520069.13698167
SRR17520069.13698168
SRR17520069.13698169
SRR17520069.13698170
SRR17520069.13698171
SRR17520069.13698172
SRR17520069.13698173
SRR17520069.13698174
SRR17520069.13698175
SRR17520069.13698176
SRR17520069.13698177
SRR17520069.13698178
SRR17520069.13698179
SRR17520069.13698180
SRR17520069.13698182
SRR17520069.13698183
SRR17520069.13698184
SRR17520069.13698186
SRR17520069.13698187
SRR17520069.13698188
SRR17520069.13698189
SRR17520069.13698190
SRR17520069.13698191
SRR17520069.13715386
SRR17520069.13715418
SRR17520069.13715424
SRR17520069.13715428
SRR17520069.13715434
SRR17520069.13721277
SRR17520069.13721280
SRR17520069.13727708
SRR17520069.13757354
SRR17520069.13759959
SRR17520069.13759963
SRR17520069.13761530

SRR17520069.13764115
SRR17520069.13809109
SRR17520069.13880619
SRR17520069.13880620
SRR17520069.13880621
SRR17520069.13880622
SRR17520069.13880623
SRR17520069.13880624
SRR17520069.13880625
SRR17520069.13880626
SRR17520069.13880627
SRR17520069.13880628
SRR17520069.13880629
SRR17520069.13880630
SRR17520069.13880631
SRR17520069.13880632
SRR17520069.13880645
SRR17520069.13910751
SRR17520069.13917651
SRR17520069.13987916
SRR17520069.13987917
SRR17520069.13987918
SRR17520069.13987919
SRR17520069.13993141
SRR17520069.13997069
SRR17520069.13997070
SRR17520069.13997071
SRR17520069.13997072
SRR17520069.13997073
SRR17520069.13997074
SRR17520069.13997075
SRR17520069.13997076
SRR17520069.13997077
SRR17520069.13997078
SRR17520069.13997079
SRR17520069.14040061
SRR17520069.14040073
SRR17520069.14047336
SRR17520069.14057965
SRR17520069.14070701
SRR17520069.14093322
SRR17520069.14093323
SRR17520069.14093324
SRR17520069.14093325
SRR17520069.14093326
SRR17520069.14093327
SRR17520069.14093328

SRR17520069.14093329
SRR17520069.14093330
SRR17520069.14093331
SRR17520069.14093332
SRR17520069.14093333
SRR17520069.14100729
SRR17520069.14100730
SRR17520069.14100733
SRR17520069.14145732
SRR17520069.14156510
SRR17520069.14159713
SRR17520069.14174145
SRR17520069.14178439
SRR17520069.14182045
SRR17520069.14236810
SRR17520069.14284511
SRR17520069.14284576
SRR17520069.14326465
SRR17520069.14326466
SRR17520069.14326467
SRR17520069.14326468
SRR17520069.14326469
SRR17520069.14326470
SRR17520069.14326471
SRR17520069.14326472
SRR17520069.14326473
SRR17520069.14326474
SRR17520069.14326475
SRR17520069.14326476
SRR17520069.14326477
SRR17520069.14326478
SRR17520069.14326479
SRR17520069.14326480
SRR17520069.14326481
SRR17520069.14326482
SRR17520069.14326483
SRR17520069.14326484
SRR17520069.14326485
SRR17520069.14326486
SRR17520069.14326487
SRR17520069.14326488
SRR17520069.14326489
SRR17520069.14326490
SRR17520069.14326491
SRR17520069.14326492
SRR17520069.14326493
SRR17520069.14326494

SRR17520069.14326495
SRR17520069.14326496
SRR17520069.14326497
SRR17520069.14326498
SRR17520069.14326499
SRR17520069.14326500
SRR17520069.14326501
SRR17520069.14326502
SRR17520069.14326503
SRR17520069.14326504
SRR17520069.14326505
SRR17520069.14326506
SRR17520069.14326507
SRR17520069.14326508
SRR17520069.14326509
SRR17520069.14326510
SRR17520069.14326511
SRR17520069.14326512
SRR17520069.14326513
SRR17520069.14326515
SRR17520069.14326516
SRR17520069.14326517
SRR17520069.14339342
SRR17520069.14344206
SRR17520069.14397968
SRR17520069.14399908
SRR17520069.14426620
SRR17520069.14446388
SRR17520069.14446451
SRR17520069.14446452
SRR17520069.14447287
SRR17520069.14447288
SRR17520069.14447289
SRR17520069.14447290
SRR17520069.14447291
SRR17520069.14447292
SRR17520069.14447293
SRR17520069.14447294
SRR17520069.14447295
SRR17520069.14447296
SRR17520069.14447297
SRR17520069.14447298
SRR17520069.14447299
SRR17520069.14447300
SRR17520069.14447301
SRR17520069.14447302
SRR17520069.14447303

SRR17520069.14447304
SRR17520069.14447305
SRR17520069.14486089
SRR17520069.14544054
SRR17520069.14571767
SRR17520069.14572433
SRR17520069.14605647
SRR17520069.14609677
SRR17520069.14609678
SRR17520069.14609679
SRR17520069.14609680
SRR17520069.14609681
SRR17520069.14609682
SRR17520069.14609683
SRR17520069.14609684
SRR17520069.14609685
SRR17520069.14609686
SRR17520069.14609687
SRR17520069.14609688
SRR17520069.14609689
SRR17520069.14609690
SRR17520069.14609691
SRR17520069.14609692
SRR17520069.14609693
SRR17520069.14609694
SRR17520069.14609695
SRR17520069.14609696
SRR17520069.14609698
SRR17520069.14609699
SRR17520069.14609700
SRR17520069.14609701
SRR17520069.14609702
SRR17520069.14609703
SRR17520069.14609704
SRR17520069.14609705
SRR17520069.14609706
SRR17520069.14609707
SRR17520069.14609708
SRR17520069.14609709
SRR17520069.14609710
SRR17520069.14609711
SRR17520069.14622937
SRR17520069.14641142
SRR17520069.14663943
SRR17520069.14663947
SRR17520069.14663952
SRR17520069.14665435

SRR17520069.14683918
SRR17520069.14693822
SRR17520069.14710141
SRR17520069.14710142
SRR17520069.14710143
SRR17520069.14710144
SRR17520069.14710145
SRR17520069.14710146
SRR17520069.14710147
SRR17520069.14710148
SRR17520069.14710149
SRR17520069.14710150
SRR17520069.14710151
SRR17520069.14710152
SRR17520069.14710153
SRR17520069.14710154
SRR17520069.14710155
SRR17520069.14710156
SRR17520069.14710157
SRR17520069.14710159
SRR17520069.14710160
SRR17520069.14710161
SRR17520069.14710162
SRR17520069.14710165
SRR17520069.14710168
SRR17520069.14734162
SRR17520069.14752804
SRR17520069.14782177
SRR17520069.14783922
SRR17520069.14822245
SRR17520069.14843640
SRR17520069.14888431
SRR17520069.14888432
SRR17520069.14888433
SRR17520069.14888438
SRR17520069.14888439
SRR17520069.14892093
SRR17520069.14892571
SRR17520069.14918255
SRR17520069.14936063
SRR17520069.14973193
SRR17520069.14989534
SRR17520069.15005002
SRR17520069.15005003
SRR17520069.15005004
SRR17520069.15005005
SRR17520069.15005006

SRR17520069.15005007
SRR17520069.15005008
SRR17520069.15005009
SRR17520069.15005010
SRR17520069.15005012
SRR17520069.15005013
SRR17520069.15043861
SRR17520069.15063005
SRR17520069.15063045
SRR17520069.15063084
SRR17520069.15063107
SRR17520069.15063108
SRR17520069.15063109
SRR17520069.15063110
SRR17520069.15063111
SRR17520069.15063112
SRR17520069.15063113
SRR17520069.15063114
SRR17520069.15063115
SRR17520069.15063116
SRR17520069.15063117
SRR17520069.15063118
SRR17520069.15063119
SRR17520069.15063120
SRR17520069.15063121
SRR17520069.15063122
SRR17520069.15063123
SRR17520069.15063124
SRR17520069.15063125
SRR17520069.15063126
SRR17520069.15064712
SRR17520069.15064713
SRR17520069.15064714
SRR17520069.15064715
SRR17520069.15064716
SRR17520069.15064717
SRR17520069.15064718
SRR17520069.15064719
SRR17520069.15064720
SRR17520069.15064721
SRR17520069.15064722
SRR17520069.15064723
SRR17520069.15064724
SRR17520069.15064725
SRR17520069.15064726
SRR17520069.15064727
SRR17520069.15064728

SRR17520069.15064729
SRR17520069.15064730
SRR17520069.15064731
SRR17520069.15064732
SRR17520069.15064733
SRR17520069.15064734
SRR17520069.15064735
SRR17520069.15064736
SRR17520069.15064737
SRR17520069.15064738
SRR17520069.15064739
SRR17520069.15064740
SRR17520069.15064741
SRR17520069.15064742
SRR17520069.15064743
SRR17520069.15064744
SRR17520069.15064745
SRR17520069.15064746
SRR17520069.15072326
SRR17520069.15072327
SRR17520069.15072328
SRR17520069.15072329
SRR17520069.15072330
SRR17520069.15072331
SRR17520069.15072332
SRR17520069.15072333
SRR17520069.15072334
SRR17520069.15072335
SRR17520069.15072336
SRR17520069.15074094
SRR17520069.15074095
SRR17520069.15076374
SRR17520069.15076391
SRR17520069.15115156
SRR17520069.15117869
SRR17520069.15122309
SRR17520069.15157046
SRR17520069.15182321
SRR17520069.15182327
SRR17520069.15182725
SRR17520069.15182726
SRR17520069.15182727
SRR17520069.15182728
SRR17520069.15182729
SRR17520069.15182730
SRR17520069.15182731
SRR17520069.15182732

SRR17520069.15182733
SRR17520069.15182734
SRR17520069.15182735
SRR17520069.15182736
SRR17520069.15182737
SRR17520069.15182738
SRR17520069.15182739
SRR17520069.15182740
SRR17520069.15182741
SRR17520069.15182743
SRR17520069.15182744
SRR17520069.15182745
SRR17520069.15182746
SRR17520069.15182747
SRR17520069.15182748
SRR17520069.15182749
SRR17520069.15182750
SRR17520069.15182751
SRR17520069.15182752
SRR17520069.15182753
SRR17520069.15182754
SRR17520069.15182755
SRR17520069.15182757
SRR17520069.15182758
SRR17520069.15182760
SRR17520069.15182761
SRR17520069.15182762
SRR17520069.15182763
SRR17520069.15182764
SRR17520069.15182765
SRR17520069.15182766
SRR17520069.15182767
SRR17520069.15182768
SRR17520069.15182769
SRR17520069.15182770
SRR17520069.15182771
SRR17520069.15182772
SRR17520069.15182773
SRR17520069.15182774
SRR17520069.15214546
SRR17520069.15214547
SRR17520069.15214548
SRR17520069.15214549
SRR17520069.15214550
SRR17520069.15214551
SRR17520069.15214552
SRR17520069.15214553

SRR17520069.15214554
SRR17520069.15214555
SRR17520069.15214556
SRR17520069.15214557
SRR17520069.15214558
SRR17520069.15214559
SRR17520069.15214560
SRR17520069.15214561
SRR17520069.15214562
SRR17520069.15214563
SRR17520069.15233177
SRR17520069.15272066
SRR17520069.15285471
SRR17520069.15285694
SRR17520069.15296490
SRR17520069.15296514
SRR17520069.15305248
SRR17520069.15305249
SRR17520069.15305250
SRR17520069.15305251
SRR17520069.15305252
SRR17520069.15305253
SRR17520069.15305254
SRR17520069.15305255
SRR17520069.15305256
SRR17520069.15305257
SRR17520069.15305258
SRR17520069.15305259
SRR17520069.15323151
SRR17520069.15325693
SRR17520069.15340072
SRR17520069.15345775
SRR17520069.15352380
SRR17520069.15380493
SRR17520069.15414719
SRR17520069.15418007
SRR17520069.15491613
SRR17520069.15491614
SRR17520069.15491615
SRR17520069.15491616
SRR17520069.15491617
SRR17520069.15491618
SRR17520069.15491619
SRR17520069.15491620
SRR17520069.15491621
SRR17520069.15491622
SRR17520069.15491623

SRR17520069.15491624
SRR17520069.15491626
SRR17520069.15491627
SRR17520069.15494750
SRR17520069.15494751
SRR17520069.15494752
SRR17520069.15494753
SRR17520069.15494754
SRR17520069.15494755
SRR17520069.15494756
SRR17520069.15494757
SRR17520069.15494758
SRR17520069.15494759
SRR17520069.15494760
SRR17520069.15494761
SRR17520069.15494762
SRR17520069.15494763
SRR17520069.15494765
SRR17520069.15494766
SRR17520069.15494767
SRR17520069.15494768
SRR17520069.15494769
SRR17520069.15494772
SRR17520069.15494773
SRR17520069.15494777
SRR17520069.15494778
SRR17520069.15494779
SRR17520069.15494781
SRR17520069.15494782
SRR17520069.15494785
SRR17520069.15494787
SRR17520069.15514266
SRR17520069.15552589
SRR17520069.15564286
SRR17520069.15574857
SRR17520069.15574887
SRR17520069.15577673
SRR17520069.15577740
SRR17520069.15578418
SRR17520069.15581390
SRR17520069.15590215
SRR17520069.15596764
SRR17520069.15596772
SRR17520069.15596774
SRR17520069.15596782
SRR17520069.15596783
SRR17520069.15596788

SRR17520069.15596790
SRR17520069.15596792
SRR17520069.15596793
SRR17520069.15596794
SRR17520069.15596795
SRR17520069.15596796
SRR17520069.15596799
SRR17520069.15596800
SRR17520069.15596803
SRR17520069.15596804
SRR17520069.15596807
SRR17520069.15596810
SRR17520069.15616583
SRR17520069.15665101
SRR17520069.15671050
SRR17520069.15671084
SRR17520069.15691722
SRR17520069.15730032
SRR17520069.15748075
SRR17520069.15767774
SRR17520069.15767777
SRR17520069.15767778
SRR17520069.15767779
SRR17520069.15767780
SRR17520069.15767781
SRR17520069.15767782
SRR17520069.15767783
SRR17520069.15767785
SRR17520069.15767786
SRR17520069.15767787
SRR17520069.15767788
SRR17520069.15767789
SRR17520069.15767790
SRR17520069.15767791
SRR17520069.15767792
SRR17520069.15767793
SRR17520069.15767794
SRR17520069.15767795
SRR17520069.15767796
SRR17520069.15767797
SRR17520069.15767798
SRR17520069.15767799
SRR17520069.15767800
SRR17520069.15767801
SRR17520069.15814336
SRR17520069.15814337
SRR17520069.15814338

SRR17520069.15814339
SRR17520069.15814340
SRR17520069.15814341
SRR17520069.15814342
SRR17520069.15814343
SRR17520069.15814344
SRR17520069.15814345
SRR17520069.15814346
SRR17520069.15814347
SRR17520069.15814348
SRR17520069.15814349
SRR17520069.15814350
SRR17520069.15814351
SRR17520069.15814352
SRR17520069.15814353
SRR17520069.15814354
SRR17520069.15814355
SRR17520069.15814356
SRR17520069.15814357
SRR17520069.15814358
SRR17520069.15814359
SRR17520069.15814360
SRR17520069.15814361
SRR17520069.15814362
SRR17520069.15814363
SRR17520069.15814364
SRR17520069.15814365
SRR17520069.15814366
SRR17520069.15814367
SRR17520069.15814368
SRR17520069.15814369
SRR17520069.15814370
SRR17520069.15814371
SRR17520069.15814372
SRR17520069.15814373
SRR17520069.15814374
SRR17520069.15814375
SRR17520069.15814376
SRR17520069.15814377
SRR17520069.15814378
SRR17520069.15814379
SRR17520069.15814380
SRR17520069.15814381
SRR17520069.15814382
SRR17520069.15814383
SRR17520069.15814384
SRR17520069.15814385

SRR17520069.15814386
SRR17520069.15814387
SRR17520069.15814388
SRR17520069.15814389
SRR17520069.15814390
SRR17520069.15814391
SRR17520069.15814392
SRR17520069.15814393
SRR17520069.15814394
SRR17520069.15814395
SRR17520069.15814396
SRR17520069.15814397
SRR17520069.15814398
SRR17520069.15814399
SRR17520069.15814400
SRR17520069.15814401
SRR17520069.15814402
SRR17520069.15814403
SRR17520069.15814404
SRR17520069.15814405
SRR17520069.15814406
SRR17520069.15814407
SRR17520069.15824054
SRR17520069.15846352
SRR17520069.15853995
SRR17520069.15865129
SRR17520069.15879114
SRR17520069.15879174
SRR17520069.15885777
SRR17520069.15932666
SRR17520069.15948905
SRR17520069.15949883
SRR17520069.15969842
SRR17520069.15969843
SRR17520069.15969845
SRR17520069.15969846
SRR17520069.15969847
SRR17520069.15969848
SRR17520069.15969849
SRR17520069.15969850
SRR17520069.15969851
SRR17520069.15969853
SRR17520069.15969854
SRR17520069.15969855
SRR17520069.15969856
SRR17520069.15969857
SRR17520069.15969858

SRR17520069.15969860
SRR17520069.15969862
SRR17520069.15969863
SRR17520069.15969864
SRR17520069.15969866
SRR17520069.15969867
SRR17520069.15969870
SRR17520069.15969871
SRR17520069.15969872
SRR17520069.15969873
SRR17520069.15969874
SRR17520069.15969875
SRR17520069.15969876
SRR17520069.15969877
SRR17520069.15969878
SRR17520069.15969885
SRR17520069.15969886
SRR17520069.15969891
SRR17520069.15969894
SRR17520069.15969896
SRR17520069.15969899
SRR17520069.15969903
SRR17520069.15969904
SRR17520069.15969905
SRR17520069.15969906
SRR17520069.15969907
SRR17520069.15969908
SRR17520069.15969910
SRR17520069.15969911
SRR17520069.15969913
SRR17520069.15969914
SRR17520069.15969916
SRR17520069.15969918
SRR17520069.15969919
SRR17520069.15969923
SRR17520069.15969924
SRR17520069.15969925
SRR17520069.15969926
SRR17520069.15969928
SRR17520069.15969929
SRR17520069.15969931
SRR17520069.15970219
SRR17520069.15970390
SRR17520069.15981102
SRR17520069.15981107
SRR17520069.15981109
SRR17520069.15981110

SRR17520069.15981111
SRR17520069.15981112
SRR17520069.15981113
SRR17520069.15981114
SRR17520069.15981115
SRR17520069.15983084
SRR17520069.15983099
SRR17520069.15991811
SRR17520069.15991812
SRR17520069.15991813
SRR17520069.15991814
SRR17520069.15991815
SRR17520069.15991816
SRR17520069.15991817
SRR17520069.15991818
SRR17520069.15991819
SRR17520069.15991820
SRR17520069.15991821
SRR17520069.15991822
SRR17520069.15991823
SRR17520069.15991824
SRR17520069.15991825
SRR17520069.15991826
SRR17520069.15991827
SRR17520069.15991828
SRR17520069.15991829
SRR17520069.15991830
SRR17520069.15991831
SRR17520069.15991832
SRR17520069.15991833
SRR17520069.15991834
SRR17520069.15991835
SRR17520069.15991836
SRR17520069.15991837
SRR17520069.15991838
SRR17520069.15991839
SRR17520069.15991840
SRR17520069.15991841
SRR17520069.15991842
SRR17520069.15991843
SRR17520069.15991844
SRR17520069.15991845
SRR17520069.15991846
SRR17520069.15991847
SRR17520069.15991848
SRR17520069.15991849
SRR17520069.15991850

SRR17520069.15991851
SRR17520069.15991852
SRR17520069.15991853
SRR17520069.15991854
SRR17520069.15991855
SRR17520069.15991856
SRR17520069.15991857
SRR17520069.15991858
SRR17520069.15991859
SRR17520069.15991860
SRR17520069.15991861
SRR17520069.15991862
SRR17520069.15991864
SRR17520069.15991865
SRR17520069.15991866
SRR17520069.15991867
SRR17520069.15991868
SRR17520069.15991869
SRR17520069.15991870
SRR17520069.15991871
SRR17520069.15991872
SRR17520069.15991873
SRR17520069.15991874
SRR17520069.15991875
SRR17520069.15991876
SRR17520069.15991877
SRR17520069.15991878
SRR17520069.15991879
SRR17520069.15991880
SRR17520069.15991881
SRR17520069.15991882
SRR17520069.15991883
SRR17520069.15991884
SRR17520069.15991885
SRR17520069.15991887
SRR17520069.15991888
SRR17520069.15991889
SRR17520069.15991890
SRR17520069.15991891
SRR17520069.15991892
SRR17520069.15991893
SRR17520069.15991894
SRR17520069.15991895
SRR17520069.15991896
SRR17520069.15991897
SRR17520069.15991898
SRR17520069.15991899

SRR17520069.15991900
SRR17520069.15991901
SRR17520069.15991902
SRR17520069.15991903
SRR17520069.15991904
SRR17520069.15991905
SRR17520069.15991906
SRR17520069.15991907
SRR17520069.15991908
SRR17520069.15991909
SRR17520069.15991910
SRR17520069.15991911
SRR17520069.15991912
SRR17520069.15991913
SRR17520069.15991914
SRR17520069.16003890
SRR17520069.16016532
SRR17520069.16042507
SRR17520069.16048782
SRR17520069.16048783
SRR17520069.16048784
SRR17520069.16048785
SRR17520069.16048786
SRR17520069.16048787
SRR17520069.16048788
SRR17520069.16048789
SRR17520069.16048790
SRR17520069.16048792
SRR17520069.16048793
SRR17520069.16048794
SRR17520069.16048795
SRR17520069.16048796
SRR17520069.16048797
SRR17520069.16048798
SRR17520069.16048799
SRR17520069.16048800
SRR17520069.16048801
SRR17520069.16048802
SRR17520069.16048803
SRR17520069.16048804
SRR17520069.16048805
SRR17520069.16048806
SRR17520069.16048808
SRR17520069.16048809
SRR17520069.16048811
SRR17520069.16048812
SRR17520069.16048813

SRR17520069.16048819
SRR17520069.16048820
SRR17520069.16048821
SRR17520069.16048822
SRR17520069.16048823
SRR17520069.16048824
SRR17520069.16048825
SRR17520069.16048826
SRR17520069.16048827
SRR17520069.16048828
SRR17520069.16048829
SRR17520069.16048830
SRR17520069.16048831
SRR17520069.16048832
SRR17520069.16048833
SRR17520069.16048834
SRR17520069.16048835
SRR17520069.16048836
SRR17520069.16048837
SRR17520069.16048838
SRR17520069.16048839
SRR17520069.16048840
SRR17520069.16048841
SRR17520069.16048842
SRR17520069.16048843
SRR17520069.16048844
SRR17520069.16048845
SRR17520069.16048846
SRR17520069.16048847
SRR17520069.16048848
SRR17520069.16048849
SRR17520069.16048850
SRR17520069.16048851
SRR17520069.16048852
SRR17520069.16048853
SRR17520069.16048854
SRR17520069.16048855
SRR17520069.16048856
SRR17520069.16048857
SRR17520069.16048858
SRR17520069.16048859
SRR17520069.16048860
SRR17520069.16048861
SRR17520069.16048862
SRR17520069.16048863
SRR17520069.16048864
SRR17520069.16048865

SRR17520069.16048866
SRR17520069.16048867
SRR17520069.16048868
SRR17520069.16048869
SRR17520069.16048870
SRR17520069.16048871
SRR17520069.16048872
SRR17520069.16048873
SRR17520069.16048874
SRR17520069.16048875
SRR17520069.16048876
SRR17520069.16048877
SRR17520069.16048878
SRR17520069.16048879
SRR17520069.16048880
SRR17520069.16048881
SRR17520069.16048882
SRR17520069.16048883
SRR17520069.16048885
SRR17520069.16048886
SRR17520069.16048887
SRR17520069.16048888
SRR17520069.16048890
SRR17520069.16048891
SRR17520069.16048892
SRR17520069.16048893
SRR17520069.16048894
SRR17520069.16048895
SRR17520069.16048896
SRR17520069.16048897
SRR17520069.16048898
SRR17520069.16048899
SRR17520069.16048900
SRR17520069.16048901
SRR17520069.16048902
SRR17520069.16048903
SRR17520069.16048904
SRR17520069.16048905
SRR17520069.16048906
SRR17520069.16048907
SRR17520069.16048908
SRR17520069.16048909
SRR17520069.16048910
SRR17520069.16048911
SRR17520069.16048912
SRR17520069.16048913
SRR17520069.16048914

SRR17520069.16063510
SRR17520069.16065260
SRR17520069.16065261
SRR17520069.16065262
SRR17520069.16065263
SRR17520069.16091572
SRR17520069.16092342
SRR17520069.16112434
SRR17520069.16116945
SRR17520069.16117835
SRR17520069.16139467
SRR17520069.16139471
SRR17520069.16139473
SRR17520069.16139474
SRR17520069.16139475
SRR17520069.16139476
SRR17520069.16139477
SRR17520069.16139478
SRR17520069.16139479
SRR17520069.16139480
SRR17520069.16157721
SRR17520069.16157733
SRR17520069.16157760
SRR17520069.16217673
SRR17520069.16225719
SRR17520069.16239390
SRR17520069.16239392
SRR17520069.16239393
SRR17520069.16239394
SRR17520069.16239395
SRR17520069.16239397
SRR17520069.16239398
SRR17520069.16239399
SRR17520069.16239400
SRR17520069.16239401
SRR17520069.16239402
SRR17520069.16239404
SRR17520069.16239405
SRR17520069.16239406
SRR17520069.16239407
SRR17520069.16239408
SRR17520069.16239409
SRR17520069.16239410
SRR17520069.16239411
SRR17520069.16239412
SRR17520069.16239413
SRR17520069.16239414

SRR17520069.16239415
SRR17520069.16239417
SRR17520069.16239418
SRR17520069.16239419
SRR17520069.16239420
SRR17520069.16239421
SRR17520069.16239422
SRR17520069.16239423
SRR17520069.16239424
SRR17520069.16239425
SRR17520069.16239426
SRR17520069.16239427
SRR17520069.16239428
SRR17520069.16239429
SRR17520069.16239430
SRR17520069.16239431
SRR17520069.16239432
SRR17520069.16239433
SRR17520069.16239435
SRR17520069.16239436
SRR17520069.16239437
SRR17520069.16239438
SRR17520069.16239439
SRR17520069.16239440
SRR17520069.16239441
SRR17520069.16239442
SRR17520069.16239443
SRR17520069.16239444
SRR17520069.16239446
SRR17520069.16239447
SRR17520069.16239448
SRR17520069.16239449
SRR17520069.16239450
SRR17520069.16239451
SRR17520069.16239452
SRR17520069.16239453
SRR17520069.16239454
SRR17520069.16239455
SRR17520069.16239456
SRR17520069.16239457
SRR17520069.16239458
SRR17520069.16239459
SRR17520069.16239460
SRR17520069.16239461
SRR17520069.16239462
SRR17520069.16239463
SRR17520069.16239464

SRR17520069.16239465
SRR17520069.16239467
SRR17520069.16239468
SRR17520069.16239469
SRR17520069.16239470
SRR17520069.16239471
SRR17520069.16239472
SRR17520069.16239473
SRR17520069.16239474
SRR17520069.16239475
SRR17520069.16239477
SRR17520069.16239478
SRR17520069.16239479
SRR17520069.16239480
SRR17520069.16239481
SRR17520069.16239482
SRR17520069.16239483
SRR17520069.16239484
SRR17520069.16239485
SRR17520069.16239486
SRR17520069.16239487
SRR17520069.16239488
SRR17520069.16239489
SRR17520069.16239490
SRR17520069.16239491
SRR17520069.16239492
SRR17520069.16239493
SRR17520069.16239494
SRR17520069.16239495
SRR17520069.16239496
SRR17520069.16239497
SRR17520069.16239498
SRR17520069.16239499
SRR17520069.16239502
SRR17520069.16239503
SRR17520069.16239504
SRR17520069.16239505
SRR17520069.16239506
SRR17520069.16239507
SRR17520069.16239508
SRR17520069.16239509
SRR17520069.16239510
SRR17520069.16239511
SRR17520069.16239513
SRR17520069.16239514
SRR17520069.16239515
SRR17520069.16239516

SRR17520069.16239517
SRR17520069.16239518
SRR17520069.16239520
SRR17520069.16239521
SRR17520069.16239522
SRR17520069.16239524
SRR17520069.16239525
SRR17520069.16240764
SRR17520069.16242852
SRR17520069.16245837
SRR17520069.16259804
SRR17520069.16261834
SRR17520069.16303128
SRR17520069.16320234
SRR17520069.16320245
SRR17520069.16320265
SRR17520069.16327521
SRR17520069.16349217
SRR17520069.16352164
SRR17520069.16382832
SRR17520069.16400235
SRR17520069.16407361
SRR17520069.16427347
SRR17520069.16450307
SRR17520069.16450308
SRR17520069.16450309
SRR17520069.16450310
SRR17520069.16450311
SRR17520069.16450312
SRR17520069.16450313
SRR17520069.16450314
SRR17520069.16450315
SRR17520069.16450316
SRR17520069.16450317
SRR17520069.16450318
SRR17520069.16450319
SRR17520069.16450320
SRR17520069.16450321
SRR17520069.16450322
SRR17520069.16515552
SRR17520069.16516980
SRR17520069.16522377
SRR17520069.16531387
SRR17520069.16559419
SRR17520069.16575981
SRR17520069.16594954
SRR17520069.16594955

SRR17520069.16594956
SRR17520069.16594957
SRR17520069.16594958
SRR17520069.16594959
SRR17520069.16594960
SRR17520069.16596681
SRR17520069.16596682
SRR17520069.16596684
SRR17520069.16596685
SRR17520069.16596686
SRR17520069.16596687
SRR17520069.16596688
SRR17520069.16596689
SRR17520069.16596690
SRR17520069.16596691
SRR17520069.16596692
SRR17520069.16596693
SRR17520069.16596694
SRR17520069.16596695
SRR17520069.16596696
SRR17520069.16596697
SRR17520069.16596698
SRR17520069.16596699
SRR17520069.16596700
SRR17520069.16597319
SRR17520069.16597320
SRR17520069.16597321
SRR17520069.16597322
SRR17520069.16597323
SRR17520069.16597324
SRR17520069.16597325
SRR17520069.16597326
SRR17520069.16597329
SRR17520069.16597330
SRR17520069.16597331
SRR17520069.16597332
SRR17520069.16597333
SRR17520069.16597334
SRR17520069.16597335
SRR17520069.16597336
SRR17520069.16597337
SRR17520069.16597338
SRR17520069.16597339
SRR17520069.16597340
SRR17520069.16597341
SRR17520069.16597342
SRR17520069.16597343

SRR17520069.16597345
SRR17520069.16597346
SRR17520069.16597347
SRR17520069.16597348
SRR17520069.16597349
SRR17520069.16597350
SRR17520069.16597351
SRR17520069.16597352
SRR17520069.16597353
SRR17520069.16597354
SRR17520069.16597355
SRR17520069.16597356
SRR17520069.16597357
SRR17520069.16597358
SRR17520069.16597359
SRR17520069.16597360
SRR17520069.16597361
SRR17520069.16597362
SRR17520069.16597363
SRR17520069.16597364
SRR17520069.16597365
SRR17520069.16597366
SRR17520069.16597367
SRR17520069.16597368
SRR17520069.16597369
SRR17520069.16597370
SRR17520069.16597371
SRR17520069.16597372
SRR17520069.16597373
SRR17520069.16597374
SRR17520069.16597375
SRR17520069.16597376
SRR17520069.16597377
SRR17520069.16597378
SRR17520069.16597379
SRR17520069.16597381
SRR17520069.16597382
SRR17520069.16605684
SRR17520069.16656319
SRR17520069.16656320
SRR17520069.16656321
SRR17520069.16656322
SRR17520069.16656323
SRR17520069.16656324
SRR17520069.16656325
SRR17520069.16656327
SRR17520069.16656328

SRR17520069.16656329
SRR17520069.16656330
SRR17520069.16656331
SRR17520069.16656332
SRR17520069.16656333
SRR17520069.16656334
SRR17520069.16656335
SRR17520069.16656336
SRR17520069.16656337
SRR17520069.16656338
SRR17520069.16656339
SRR17520069.16656340
SRR17520069.16656341
SRR17520069.16656342
SRR17520069.16656343
SRR17520069.16656344
SRR17520069.16656345
SRR17520069.16656346
SRR17520069.16692453
SRR17520069.16720462
SRR17520069.16721534
SRR17520069.16748656
SRR17520069.16752858
SRR17520069.16790819
SRR17520069.16790820
SRR17520069.16790821
SRR17520069.16790822
SRR17520069.16790823
SRR17520069.16790824
SRR17520069.16790825
SRR17520069.16790826
SRR17520069.16798447
SRR17520069.16798479
SRR17520069.16823415
SRR17520069.16830356
SRR17520069.16852713
SRR17520069.16867559
SRR17520069.16867560
SRR17520069.16867561
SRR17520069.16867562
SRR17520069.16867563
SRR17520069.16867564
SRR17520069.16867565
SRR17520069.16867566
SRR17520069.16867567
SRR17520069.16867568
SRR17520069.16867569

SRR17520069.16867570
SRR17520069.16867571
SRR17520069.16867572
SRR17520069.16867573
SRR17520069.16867574
SRR17520069.16867575
SRR17520069.16867576
SRR17520069.16867577
SRR17520069.16867578
SRR17520069.16867579
SRR17520069.16867580
SRR17520069.16867581
SRR17520069.16867582
SRR17520069.16867583
SRR17520069.16867584
SRR17520069.16867585
SRR17520069.16867586
SRR17520069.16867587
SRR17520069.16867588
SRR17520069.16867589
SRR17520069.16867590
SRR17520069.16867591
SRR17520069.16867592
SRR17520069.16867593
SRR17520069.16867594
SRR17520069.16867595
SRR17520069.16867596
SRR17520069.16867597
SRR17520069.16867598
SRR17520069.16867599
SRR17520069.16889788
SRR17520069.16913302
SRR17520069.16925832
SRR17520069.16955209
SRR17520069.16955210
SRR17520069.16955211
SRR17520069.16955212
SRR17520069.16955213
SRR17520069.16955214
SRR17520069.16955215
SRR17520069.16955216
SRR17520069.16955217
SRR17520069.16955218
SRR17520069.16955219
SRR17520069.16955220
SRR17520069.16955221
SRR17520069.16960533

SRR17520069.16981715
SRR17520069.16981718
SRR17520069.16981723
SRR17520069.16981735
SRR17520069.16981738
SRR17520069.16981747
SRR17520069.16991635
SRR17520069.16992680
SRR17520069.16994827
SRR17520069.17004331
SRR17520069.17025316
SRR17520069.17025319
SRR17520069.17025321
SRR17520069.17025324
SRR17520069.17025329
SRR17520069.17025331
SRR17520069.17025336
SRR17520069.17025343
SRR17520069.17025347
SRR17520069.17025354
SRR17520069.17025368
SRR17520069.17025371
SRR17520069.17025373
SRR17520069.17025377
SRR17520069.17025380
SRR17520069.17025381
SRR17520069.17025388
SRR17520069.17025390
SRR17520069.17025396
SRR17520069.17025402
SRR17520069.17025409
SRR17520069.17025411
SRR17520069.17025412
SRR17520069.17025419
SRR17520069.17025420
SRR17520069.17025431
SRR17520069.17025438
SRR17520069.17025444
SRR17520069.17025448
SRR17520069.17025454
SRR17520069.17038393
SRR17520069.17040211
SRR17520069.17040212
SRR17520069.17040213
SRR17520069.17040214
SRR17520069.17040215
SRR17520069.17040216

SRR17520069.17040217
SRR17520069.17040218
SRR17520069.17040219
SRR17520069.17040220
SRR17520069.17040221
SRR17520069.17040222
SRR17520069.17040223
SRR17520069.17044306
SRR17520069.17070982
SRR17520069.17073338
SRR17520069.17073339
SRR17520069.17113470
SRR17520069.17119943
SRR17520069.17181884
SRR17520069.17194451
SRR17520069.17195299
SRR17520069.17215663
SRR17520069.17235650
SRR17520069.17235673
SRR17520069.17237377
SRR17520069.17237399
SRR17520069.17237401
SRR17520069.17241039
SRR17520069.17256276
SRR17520069.17278158
SRR17520069.17285271
SRR17520069.17295605
SRR17520069.17303100
SRR17520069.17303103
SRR17520069.17303104
SRR17520069.17303105
SRR17520069.17303106
SRR17520069.17303107
SRR17520069.17303109
SRR17520069.17303110
SRR17520069.17303111
SRR17520069.17303112
SRR17520069.17303113
SRR17520069.17303116
SRR17520069.17303117
SRR17520069.17303118
SRR17520069.17303119
SRR17520069.17303121
SRR17520069.17303122
SRR17520069.17303123
SRR17520069.17303124
SRR17520069.17303125

SRR17520069.17303126
SRR17520069.17303127
SRR17520069.17303128
SRR17520069.17303129
SRR17520069.17303130
SRR17520069.17303131
SRR17520069.17303132
SRR17520069.17303133
SRR17520069.17303134
SRR17520069.17303135
SRR17520069.17303136
SRR17520069.17303137
SRR17520069.17303138
SRR17520069.17303139
SRR17520069.17303140
SRR17520069.17303141
SRR17520069.17303142
SRR17520069.17303143
SRR17520069.17303144
SRR17520069.17303145
SRR17520069.17303146
SRR17520069.17303147
SRR17520069.17303148
SRR17520069.17303149
SRR17520069.17303150
SRR17520069.17303152
SRR17520069.17303153
SRR17520069.17303154
SRR17520069.17303155
SRR17520069.17303156
SRR17520069.17303157
SRR17520069.17303158
SRR17520069.17303159
SRR17520069.17303160
SRR17520069.17303161
SRR17520069.17303162
SRR17520069.17303163
SRR17520069.17303164
SRR17520069.17303165
SRR17520069.17303166
SRR17520069.17303167
SRR17520069.17303168
SRR17520069.17303169
SRR17520069.17303170
SRR17520069.17303171
SRR17520069.17303172
SRR17520069.17303174

SRR17520069.17303175
SRR17520069.17303176
SRR17520069.17303177
SRR17520069.17303178
SRR17520069.17303179
SRR17520069.17303180
SRR17520069.17303181
SRR17520069.17303182
SRR17520069.17303183
SRR17520069.17303184
SRR17520069.17303185
SRR17520069.17303186
SRR17520069.17303187
SRR17520069.17303188
SRR17520069.17303189
SRR17520069.17303190
SRR17520069.17303191
SRR17520069.17303192
SRR17520069.17303193
SRR17520069.17303194
SRR17520069.17304083
SRR17520069.17306440
SRR17520069.17322328
SRR17520069.17324352
SRR17520069.17333859
SRR17520069.17333913
SRR17520069.17339105
SRR17520069.17345581
SRR17520069.17357585
SRR17520069.17357676
SRR17520069.17357704
SRR17520069.17359064
SRR17520069.17362874
SRR17520069.17372408
SRR17520069.17374368
SRR17520069.17386578
SRR17520069.17387742
SRR17520069.17399325
SRR17520069.17403744
SRR17520069.17403753
SRR17520069.17430396
SRR17520069.17465851
SRR17520069.17465852
SRR17520069.17465853
SRR17520069.17465854
SRR17520069.17465855
SRR17520069.17465856

SRR17520069.17465857
SRR17520069.17465858
SRR17520069.17465859
SRR17520069.17477116
SRR17520069.17477117
SRR17520069.17477118
SRR17520069.17477119
SRR17520069.17477120
SRR17520069.17477121
SRR17520069.17477122
SRR17520069.17477123
SRR17520069.17477124
SRR17520069.17477125
SRR17520069.17477126
SRR17520069.17477127
SRR17520069.17477128
SRR17520069.17477129
SRR17520069.17477130
SRR17520069.17477131
SRR17520069.17477132
SRR17520069.17477133
SRR17520069.17477134
SRR17520069.17477135
SRR17520069.17477136
SRR17520069.17477137
SRR17520069.17477138
SRR17520069.17477139
SRR17520069.17477140
SRR17520069.17477141
SRR17520069.17477142
SRR17520069.17477143
SRR17520069.17477144
SRR17520069.17477145
SRR17520069.17477146
SRR17520069.17477147
SRR17520069.17477148
SRR17520069.17477149
SRR17520069.17477150
SRR17520069.17477151
SRR17520069.17477152
SRR17520069.17477153
SRR17520069.17477154
SRR17520069.17477155
SRR17520069.17477156
SRR17520069.17477157
SRR17520069.17477158
SRR17520069.17477159

SRR17520069.17477160
SRR17520069.17477161
SRR17520069.17477162
SRR17520069.17477163
SRR17520069.17477164
SRR17520069.17477165
SRR17520069.17477166
SRR17520069.17477167
SRR17520069.17477169
SRR17520069.17477170
SRR17520069.17477171
SRR17520069.17477172
SRR17520069.17477173
SRR17520069.17477174
SRR17520069.17477175
SRR17520069.17477176
SRR17520069.17477177
SRR17520069.17477178
SRR17520069.17477179
SRR17520069.17477180
SRR17520069.17477181
SRR17520069.17477182
SRR17520069.17477183
SRR17520069.17477184
SRR17520069.17477185
SRR17520069.17477186
SRR17520069.17477187
SRR17520069.17477188
SRR17520069.17477189
SRR17520069.17478273
SRR17520069.17487009
SRR17520069.17511631
SRR17520069.17511672
SRR17520069.17519976
SRR17520069.17519980
SRR17520069.17519990
SRR17520069.17519998
SRR17520069.17520140
SRR17520069.17530544
SRR17520069.17538582
SRR17520069.17540863
SRR17520069.17574031
SRR17520069.17574034
SRR17520069.17579066
SRR17520069.17585938
SRR17520069.17586290
SRR17520069.17588261

SRR17520069.17588448
SRR17520069.17647859
SRR17520069.17647862
SRR17520069.17647864
SRR17520069.17647868
SRR17520069.17647869
SRR17520069.17647876
SRR17520069.17647890
SRR17520069.17647892
SRR17520069.17647894
SRR17520069.17667514
SRR17520069.17687536
SRR17520069.17703998
SRR17520069.17710979
SRR17520069.17710996
SRR17520069.17710999
SRR17520069.17711000
SRR17520069.17711001
SRR17520069.17711002
SRR17520069.17711003
SRR17520069.17711004
SRR17520069.17711005
SRR17520069.17711006
SRR17520069.17715238
SRR17520069.17717314
SRR17520069.17717327
SRR17520069.17752286
SRR17520069.17752287
SRR17520069.17760780
SRR17520069.17766355
SRR17520069.17766356
SRR17520069.17766357
SRR17520069.17766358
SRR17520069.17766359
SRR17520069.17766360
SRR17520069.17766361
SRR17520069.17766362
SRR17520069.17766363
SRR17520069.17766364
SRR17520069.17766365
SRR17520069.17766366
SRR17520069.17766367
SRR17520069.17766368
SRR17520069.17766369
SRR17520069.17766370
SRR17520069.17766371
SRR17520069.17766372

SRR17520069.17766373
SRR17520069.17766374
SRR17520069.17766375
SRR17520069.17766376
SRR17520069.17766377
SRR17520069.17766379
SRR17520069.17766380
SRR17520069.17766381
SRR17520069.17766382
SRR17520069.17766384
SRR17520069.17766385
SRR17520069.17766386
SRR17520069.17766388
SRR17520069.17766389
SRR17520069.17766390
SRR17520069.17766391
SRR17520069.17766392
SRR17520069.17766393
SRR17520069.17766394
SRR17520069.17766395
SRR17520069.17766396
SRR17520069.17766397
SRR17520069.17766398
SRR17520069.17766399
SRR17520069.17766400
SRR17520069.17766401
SRR17520069.17766402
SRR17520069.17766403
SRR17520069.17766404
SRR17520069.17766405
SRR17520069.17766406
SRR17520069.17766407
SRR17520069.17766408
SRR17520069.17766409
SRR17520069.17766410
SRR17520069.17766411
SRR17520069.17766412
SRR17520069.17766413
SRR17520069.17766414
SRR17520069.17766415
SRR17520069.17766416
SRR17520069.17766417
SRR17520069.17766418
SRR17520069.17784571
SRR17520069.17791025
SRR17520069.17834883
SRR17520069.17834884

SRR17520069.17834885
SRR17520069.17834886
SRR17520069.17834887
SRR17520069.17834888
SRR17520069.17834889
SRR17520069.17834890
SRR17520069.17834891
SRR17520069.17834892
SRR17520069.17834893
SRR17520069.17834894
SRR17520069.17834895
SRR17520069.17834896
SRR17520069.17834897
SRR17520069.17834898
SRR17520069.17834899
SRR17520069.17834900
SRR17520069.17834901
SRR17520069.17834902
SRR17520069.17834903
SRR17520069.17834904
SRR17520069.17834905
SRR17520069.17834906
SRR17520069.17834907
SRR17520069.17834908
SRR17520069.17834909
SRR17520069.17834910
SRR17520069.17834911
SRR17520069.17834912
SRR17520069.17834913
SRR17520069.17834914
SRR17520069.17834915
SRR17520069.17846724
SRR17520069.17852439
SRR17520069.17852462
SRR17520069.17899494
SRR17520069.17899495
SRR17520069.17899496
SRR17520069.17899497
SRR17520069.17899498
SRR17520069.17899499
SRR17520069.17899500
SRR17520069.17899501
SRR17520069.17899502
SRR17520069.17899503
SRR17520069.17899504
SRR17520069.17899505
SRR17520069.17899506

SRR17520069.17899507
SRR17520069.17899508
SRR17520069.17899509
SRR17520069.17899510
SRR17520069.17908189
SRR17520069.17934934
SRR17520069.17941340
SRR17520069.17942992
SRR17520069.17944076
SRR17520069.17944090
SRR17520069.17957131
SRR17520069.17960381
SRR17520069.17993631
SRR17520069.18004875
SRR17520069.18006544
SRR17520069.18006545
SRR17520069.18006546
SRR17520069.18006547
SRR17520069.18006548
SRR17520069.18006549
SRR17520069.18006550
SRR17520069.18006551
SRR17520069.18006552
SRR17520069.18006553
SRR17520069.18006554
SRR17520069.18028833
SRR17520069.18028834
SRR17520069.18028835
SRR17520069.18028836
SRR17520069.18028837
SRR17520069.18034553
SRR17520069.18034662
SRR17520069.18035817
SRR17520069.18035855
SRR17520069.18035857
SRR17520069.18036451
SRR17520069.18058216
SRR17520069.18058222
SRR17520069.18064140
SRR17520069.18133085
SRR17520069.18151974
SRR17520069.18151975
SRR17520069.18192178
SRR17520069.18197594
SRR17520069.18197654
SRR17520069.18235206
SRR17520069.18235207

SRR17520069.18235208
SRR17520069.18235209
SRR17520069.18235210
SRR17520069.18235211
SRR17520069.18235212
SRR17520069.18235213
SRR17520069.18235214
SRR17520069.18235215
SRR17520069.18235216
SRR17520069.18235217
SRR17520069.18235219
SRR17520069.18235220
SRR17520069.18235221
SRR17520069.18235222
SRR17520069.18235223
SRR17520069.18235224
SRR17520069.18235225
SRR17520069.18235226
SRR17520069.18235227
SRR17520069.18235228
SRR17520069.18235229
SRR17520069.18235230
SRR17520069.18235231
SRR17520069.18235232
SRR17520069.18235233
SRR17520069.18235234
SRR17520069.18235235
SRR17520069.18235236
SRR17520069.18235237
SRR17520069.18235239
SRR17520069.18235240
SRR17520069.18235241
SRR17520069.18235242
SRR17520069.18235243
SRR17520069.18235244
SRR17520069.18235245
SRR17520069.18235246
SRR17520069.18235247
SRR17520069.18235248
SRR17520069.18235249
SRR17520069.18235250
SRR17520069.18235251
SRR17520069.18235252
SRR17520069.18235253
SRR17520069.18235254
SRR17520069.18235255
SRR17520069.18235256

SRR17520069.18235257
SRR17520069.18235258
SRR17520069.18235259
SRR17520069.18235260
SRR17520069.18235261
SRR17520069.18235262
SRR17520069.18235263
SRR17520069.18235264
SRR17520069.18235265
SRR17520069.18235266
SRR17520069.18235267
SRR17520069.18235268
SRR17520069.18235269
SRR17520069.18235270
SRR17520069.18235271
SRR17520069.18235272
SRR17520069.18235273
SRR17520069.18235274
SRR17520069.18235275
SRR17520069.18235276
SRR17520069.18235277
SRR17520069.18235278
SRR17520069.18235279
SRR17520069.18235280
SRR17520069.18235281
SRR17520069.18235282
SRR17520069.18235283
SRR17520069.18235284
SRR17520069.18235285
SRR17520069.18235286
SRR17520069.18235287
SRR17520069.18235288
SRR17520069.18235289
SRR17520069.18235291
SRR17520069.18235292
SRR17520069.18235293
SRR17520069.18235294
SRR17520069.18235295
SRR17520069.18235296
SRR17520069.18235297
SRR17520069.18235298
SRR17520069.18235300
SRR17520069.18235301
SRR17520069.18235302
SRR17520069.18235303
SRR17520069.18235304
SRR17520069.18238255

SRR17520069.18250970
SRR17520069.18272652
SRR17520069.18280457
SRR17520069.18281263
SRR17520069.18290813
SRR17520069.18301473
SRR17520069.18301529
SRR17520069.18304616
SRR17520069.18334012
SRR17520069.18334018
SRR17520069.18334957
SRR17520069.18356255
SRR17520069.18356266
SRR17520069.18356271
SRR17520069.18356274
SRR17520069.18373737
SRR17520069.18378960
SRR17520069.18379054
SRR17520069.18391405
SRR17520069.18393138
SRR17520069.18439448
SRR17520069.18444249
SRR17520069.18453685
SRR17520069.18453686
SRR17520069.18453687
SRR17520069.18453688
SRR17520069.18453689
SRR17520069.18453690
SRR17520069.18453691
SRR17520069.18453692
SRR17520069.18453693
SRR17520069.18453694
SRR17520069.18453695
SRR17520069.18453696
SRR17520069.18453697
SRR17520069.18453698
SRR17520069.18453699
SRR17520069.18453700
SRR17520069.18453701
SRR17520069.18453702
SRR17520069.18453703
SRR17520069.18453704
SRR17520069.18453705
SRR17520069.18453706
SRR17520069.18453707
SRR17520069.18453708
SRR17520069.18453709

SRR17520069.18453710
SRR17520069.18453711
SRR17520069.18453712
SRR17520069.18453713
SRR17520069.18453714
SRR17520069.18453715
SRR17520069.18453716
SRR17520069.18453717
SRR17520069.18453718
SRR17520069.18453719
SRR17520069.18453720
SRR17520069.18453721
SRR17520069.18464406
SRR17520069.18490415
SRR17520069.18490429
SRR17520069.18519979
SRR17520069.18520132
SRR17520069.18520134
SRR17520069.18523265
SRR17520069.18527210
SRR17520069.18588410
SRR17520069.18588411
SRR17520069.18588412
SRR17520069.18588413
SRR17520069.18588414
SRR17520069.18588415
SRR17520069.18588416
SRR17520069.18588417
SRR17520069.18588418
SRR17520069.18588419
SRR17520069.18588420
SRR17520069.18588421
SRR17520069.18588422
SRR17520069.18588423
SRR17520069.18588424
SRR17520069.18588425
SRR17520069.18592529
SRR17520069.18595983
SRR17520069.18623311
SRR17520069.18651772
SRR17520069.18673504
SRR17520069.18703428
SRR17520069.18703429
SRR17520069.18718245
SRR17520069.18720515
SRR17520069.18720521
SRR17520069.18720992

SRR17520069.18747027
SRR17520069.18790754
SRR17520069.18804777
SRR17520069.18804780
SRR17520069.18804781
SRR17520069.18804782
SRR17520069.18804783
SRR17520069.18804784
SRR17520069.18804786
SRR17520069.18810768
SRR17520069.18810771
SRR17520069.18810790
SRR17520069.18810800
SRR17520069.18817724
SRR17520069.18817729
SRR17520069.18823969
SRR17520069.18831556
SRR17520069.18879734
SRR17520069.18882351
SRR17520069.18882352
SRR17520069.18882353
SRR17520069.18882354
SRR17520069.18882355
SRR17520069.18882356
SRR17520069.18882357
SRR17520069.18882358
SRR17520069.18882359
SRR17520069.18882360
SRR17520069.18882361
SRR17520069.18882362
SRR17520069.18882363
SRR17520069.18882364
SRR17520069.18882365
SRR17520069.18882366
SRR17520069.18882367
SRR17520069.18882368
SRR17520069.18891278
SRR17520069.18935054
SRR17520069.18948527
SRR17520069.18949138
SRR17520069.18949144
SRR17520069.18949168
SRR17520069.18957656
SRR17520069.18957660
SRR17520069.18977984
SRR17520069.18977998
SRR17520069.18987910

SRR17520069.19006859
SRR17520069.19010372
SRR17520069.19048854
SRR17520069.19065964
SRR17520069.19066170
SRR17520069.19066186
SRR17520069.19076617
SRR17520069.19081920
SRR17520069.19082943
SRR17520069.19082944
SRR17520069.19082945
SRR17520069.19082947
SRR17520069.19082948
SRR17520069.19082949
SRR17520069.19082950
SRR17520069.19082951
SRR17520069.19082952
SRR17520069.19082953
SRR17520069.19082954
SRR17520069.19082955
SRR17520069.19082956
SRR17520069.19082957
SRR17520069.19082958
SRR17520069.19082959
SRR17520069.19082961
SRR17520069.19082962
SRR17520069.19104625
SRR17520069.19114596
SRR17520069.19114597
SRR17520069.19114598
SRR17520069.19114599
SRR17520069.19114600
SRR17520069.19114601
SRR17520069.19114602
SRR17520069.19114603
SRR17520069.19114604
SRR17520069.19114605
SRR17520069.19114606
SRR17520069.19114608
SRR17520069.19114609
SRR17520069.19114610
SRR17520069.19114611
SRR17520069.19114612
SRR17520069.19114613
SRR17520069.19114614
SRR17520069.19114615
SRR17520069.19114616

SRR17520069.19114617
SRR17520069.19114618
SRR17520069.19114619
SRR17520069.19114620
SRR17520069.19114621
SRR17520069.19114622
SRR17520069.19114623
SRR17520069.19114624
SRR17520069.19114625
SRR17520069.19114626
SRR17520069.19114627
SRR17520069.19114628
SRR17520069.19114629
SRR17520069.19114630
SRR17520069.19114631
SRR17520069.19114632
SRR17520069.19114633
SRR17520069.19114634
SRR17520069.19114635
SRR17520069.19114636
SRR17520069.19114637
SRR17520069.19114638
SRR17520069.19114639
SRR17520069.19114640
SRR17520069.19114641
SRR17520069.19114642
SRR17520069.19114643
SRR17520069.19114644
SRR17520069.19114645
SRR17520069.19114646
SRR17520069.19114647
SRR17520069.19114648
SRR17520069.19114649
SRR17520069.19114650
SRR17520069.19114651
SRR17520069.19114652
SRR17520069.19114653
SRR17520069.19114654
SRR17520069.19114655
SRR17520069.19114656
SRR17520069.19114657
SRR17520069.19114659
SRR17520069.19114660
SRR17520069.19114661
SRR17520069.19114662
SRR17520069.19114663
SRR17520069.19114664

SRR17520069.19114665
SRR17520069.19140198
SRR17520069.19200023
SRR17520069.19200024
SRR17520069.19200025
SRR17520069.19200026
SRR17520069.19200027
SRR17520069.19200028
SRR17520069.19200029
SRR17520069.19200030
SRR17520069.19200031
SRR17520069.19200032
SRR17520069.19200033
SRR17520069.19200034
SRR17520069.19200035
SRR17520069.19200036
SRR17520069.19200037
SRR17520069.19200038
SRR17520069.19200039
SRR17520069.19200040
SRR17520069.19201583
SRR17520069.19257472
SRR17520069.19257473
SRR17520069.19257474
SRR17520069.19257476
SRR17520069.19257477
SRR17520069.19257478
SRR17520069.19257479
SRR17520069.19257480
SRR17520069.19257481
SRR17520069.19257482
SRR17520069.19257484
SRR17520069.19257485
SRR17520069.19257486
SRR17520069.19257487
SRR17520069.19257488
SRR17520069.19257489
SRR17520069.19257490
SRR17520069.19257491
SRR17520069.19257492
SRR17520069.19257493
SRR17520069.19257494
SRR17520069.19257495
SRR17520069.19257496
SRR17520069.19257497
SRR17520069.19257498
SRR17520069.19257499

SRR17520069.19257500
SRR17520069.19257501
SRR17520069.19257502
SRR17520069.19257504
SRR17520069.19257505
SRR17520069.19257506
SRR17520069.19257507
SRR17520069.19257508
SRR17520069.19257509
SRR17520069.19257510
SRR17520069.19257511
SRR17520069.19257512
SRR17520069.19257513
SRR17520069.19257514
SRR17520069.19257516
SRR17520069.19257517
SRR17520069.19257518
SRR17520069.19257519
SRR17520069.19257520
SRR17520069.19257521
SRR17520069.19257522
SRR17520069.19257523
SRR17520069.19257524
SRR17520069.19257525
SRR17520069.19257526
SRR17520069.19257527
SRR17520069.19257528
SRR17520069.19257529
SRR17520069.19257530
SRR17520069.19257531
SRR17520069.19257532
SRR17520069.19257533
SRR17520069.19273438
SRR17520069.19273439
SRR17520069.19273440
SRR17520069.19273441
SRR17520069.19273442
SRR17520069.19273443
SRR17520069.19273444
SRR17520069.19273445
SRR17520069.19273446
SRR17520069.19273447
SRR17520069.19273448
SRR17520069.19273449
SRR17520069.19273450
SRR17520069.19273451
SRR17520069.19273453

SRR17520069.19273454
SRR17520069.19273455
SRR17520069.19273456
SRR17520069.19273457
SRR17520069.19273458
SRR17520069.19273459
SRR17520069.19273460
SRR17520069.19273461
SRR17520069.19273462
SRR17520069.19273463
SRR17520069.19273464
SRR17520069.19273465
SRR17520069.19273466
SRR17520069.19273467
SRR17520069.19273468
SRR17520069.19273469
SRR17520069.19273470
SRR17520069.19273471
SRR17520069.19273472
SRR17520069.19273473
SRR17520069.19273474
SRR17520069.19273475
SRR17520069.19273476
SRR17520069.19273477
SRR17520069.19273478
SRR17520069.19273479
SRR17520069.19273480
SRR17520069.19274822
SRR17520069.19279490
SRR17520069.19324213
SRR17520069.19341313
SRR17520069.19361007
SRR17520069.19361478
SRR17520069.19364638
SRR17520069.19368351
SRR17520069.19378123
SRR17520069.19378184
SRR17520069.19378196
SRR17520069.19443204
SRR17520069.19451434
SRR17520069.19451435
SRR17520069.19451436
SRR17520069.19451437
SRR17520069.19451438
SRR17520069.19451439
SRR17520069.19451440
SRR17520069.19451441

SRR17520069.19451442
SRR17520069.19451443
SRR17520069.19451444
SRR17520069.19451445
SRR17520069.19451446
SRR17520069.19451447
SRR17520069.19451448
SRR17520069.19451449
SRR17520069.19451450
SRR17520069.19451452
SRR17520069.19451453
SRR17520069.19451454
SRR17520069.19451455
SRR17520069.19451456
SRR17520069.19451457
SRR17520069.19451458
SRR17520069.19451459
SRR17520069.19451460
SRR17520069.19451461
SRR17520069.19451462
SRR17520069.19451463
SRR17520069.19451464
SRR17520069.19451465
SRR17520069.19451466
SRR17520069.19451467
SRR17520069.19451468
SRR17520069.19451469
SRR17520069.19451470
SRR17520069.19451471
SRR17520069.19451472
SRR17520069.19451473
SRR17520069.19451474
SRR17520069.19451476
SRR17520069.19451477
SRR17520069.19451478
SRR17520069.19451479
SRR17520069.19451480
SRR17520069.19451481
SRR17520069.19451483
SRR17520069.19451484
SRR17520069.19451485
SRR17520069.19451486
SRR17520069.19451487
SRR17520069.19451488
SRR17520069.19451489
SRR17520069.19451490
SRR17520069.19451491

SRR17520069.19451492
SRR17520069.19451493
SRR17520069.19451494
SRR17520069.19451495
SRR17520069.19451496
SRR17520069.19451497
SRR17520069.19451498
SRR17520069.19451499
SRR17520069.19451500
SRR17520069.19451501
SRR17520069.19451502
SRR17520069.19451503
SRR17520069.19451504
SRR17520069.19451505
SRR17520069.19451506
SRR17520069.19485566
SRR17520069.19491131
SRR17520069.19548672
SRR17520069.19553258
SRR17520069.29375
SRR17520069.29406
SRR17520069.72060
SRR17520069.114804
SRR17520069.169218
SRR17520069.186044
SRR17520069.187323
SRR17520069.192541
SRR17520069.212982
SRR17520069.234921
SRR17520069.243412
SRR17520069.317268
SRR17520069.317505
SRR17520069.319730
SRR17520069.341420
SRR17520069.383637
SRR17520069.416114
SRR17520069.478823
SRR17520069.521222
SRR17520069.573571
SRR17520069.578016
SRR17520069.578673
SRR17520069.592081
SRR17520069.623166
SRR17520069.623175
SRR17520069.630672
SRR17520069.630684
SRR17520069.643899

SRR17520069.690283
SRR17520069.700254
SRR17520069.700267
SRR17520069.700270
SRR17520069.715900
SRR17520069.731680
SRR17520069.731682
SRR17520069.745380
SRR17520069.763297
SRR17520069.764911
SRR17520069.774048
SRR17520069.867372
SRR17520069.868440
SRR17520069.873714
SRR17520069.883366
SRR17520069.883373
SRR17520069.900709
SRR17520069.902008
SRR17520069.902054
SRR17520069.902081
SRR17520069.906454
SRR17520069.911416
SRR17520069.919474
SRR17520069.922003
SRR17520069.931800
SRR17520069.941712
SRR17520069.981099
SRR17520069.1040333
SRR17520069.1110974
SRR17520069.1112020
SRR17520069.1128340
SRR17520069.1170823
SRR17520069.1178349
SRR17520069.1186309
SRR17520069.1196422
SRR17520069.1196423
SRR17520069.1206145
SRR17520069.1249542
SRR17520069.1265025
SRR17520069.1287111
SRR17520069.1351645
SRR17520069.1360246
SRR17520069.1435175
SRR17520069.1435178
SRR17520069.1435179
SRR17520069.1435185
SRR17520069.1448959

SRR17520069.1471325
SRR17520069.1496234
SRR17520069.1500516
SRR17520069.1503312
SRR17520069.1551276
SRR17520069.1552417
SRR17520069.1554068
SRR17520069.1554081
SRR17520069.1566134
SRR17520069.1570991
SRR17520069.1575704
SRR17520069.1575708
SRR17520069.1575769
SRR17520069.1628214
SRR17520069.1657148
SRR17520069.1658563
SRR17520069.1658570
SRR17520069.1691427
SRR17520069.1734622
SRR17520069.1734640
SRR17520069.1734643
SRR17520069.1734647
SRR17520069.1739630
SRR17520069.1752955
SRR17520069.1752964
SRR17520069.1752973
SRR17520069.1752978
SRR17520069.1752984
SRR17520069.1753003
SRR17520069.1753027
SRR17520069.1753058
SRR17520069.1753081
SRR17520069.1759671
SRR17520069.1767518
SRR17520069.1767550
SRR17520069.1786033
SRR17520069.1833328
SRR17520069.1833410
SRR17520069.1893940
SRR17520069.1895958
SRR17520069.1926812
SRR17520069.1949483
SRR17520069.1957418
SRR17520069.1990738
SRR17520069.1990759
SRR17520069.1990769
SRR17520069.2002298

SRR17520069.2017972
SRR17520069.2028434
SRR17520069.2028435
SRR17520069.2039197
SRR17520069.2107424
SRR17520069.2129779
SRR17520069.2134844
SRR17520069.2142903
SRR17520069.2142907
SRR17520069.2175308
SRR17520069.2250032
SRR17520069.2293388
SRR17520069.2299373
SRR17520069.2305245
SRR17520069.2316719
SRR17520069.2330387
SRR17520069.2340361
SRR17520069.2366203
SRR17520069.2366224
SRR17520069.2398443
SRR17520069.2402891
SRR17520069.2403674
SRR17520069.2432686
SRR17520069.2435242
SRR17520069.2435263
SRR17520069.2437203
SRR17520069.2440388
SRR17520069.2458063
SRR17520069.2458129
SRR17520069.2495067
SRR17520069.2495085
SRR17520069.2505990
SRR17520069.2524056
SRR17520069.2558858
SRR17520069.2573136
SRR17520069.2573207
SRR17520069.2687284
SRR17520069.2707160
SRR17520069.2737667
SRR17520069.2798782
SRR17520069.2820636
SRR17520069.2829185
SRR17520069.2841588
SRR17520069.2841603
SRR17520069.2841604
SRR17520069.2860203
SRR17520069.2860661

SRR17520069.2871356
SRR17520069.2891858
SRR17520069.2898296
SRR17520069.2981978
SRR17520069.2995396
SRR17520069.3006828
SRR17520069.3007335
SRR17520069.3070204
SRR17520069.3149205
SRR17520069.3183430
SRR17520069.3271406
SRR17520069.3274876
SRR17520069.3314022
SRR17520069.3331709
SRR17520069.3332731
SRR17520069.3332738
SRR17520069.3332739
SRR17520069.3345299
SRR17520069.3346081
SRR17520069.3346354
SRR17520069.3352869
SRR17520069.3369663
SRR17520069.3430867
SRR17520069.3482984
SRR17520069.3508285
SRR17520069.3508296
SRR17520069.3511646
SRR17520069.3514397
SRR17520069.3514403
SRR17520069.3544102
SRR17520069.3564899
SRR17520069.3576861
SRR17520069.3588622
SRR17520069.3608311
SRR17520069.3613712
SRR17520069.3630098
SRR17520069.3630976
SRR17520069.3643958
SRR17520069.3660027
SRR17520069.3665810
SRR17520069.3786588
SRR17520069.3795368
SRR17520069.3809446
SRR17520069.3864713
SRR17520069.3888320
SRR17520069.3946731
SRR17520069.3960612

SRR17520069.3993020
SRR17520069.4063083
SRR17520069.4064682
SRR17520069.4093487
SRR17520069.4107590
SRR17520069.4107629
SRR17520069.4111738
SRR17520069.4154686
SRR17520069.4158206
SRR17520069.4161730
SRR17520069.4161752
SRR17520069.4161829
SRR17520069.4181107
SRR17520069.4189979
SRR17520069.4196747
SRR17520069.4196754
SRR17520069.4233947
SRR17520069.4274590
SRR17520069.4307058
SRR17520069.4314298
SRR17520069.4319781
SRR17520069.4353844
SRR17520069.4356679
SRR17520069.4356867
SRR17520069.4360782
SRR17520069.4360802
SRR17520069.4367638
SRR17520069.4372563
SRR17520069.4373153
SRR17520069.4373154
SRR17520069.4396541
SRR17520069.4417053
SRR17520069.4424000
SRR17520069.4487444
SRR17520069.4492988
SRR17520069.4505294
SRR17520069.4519338
SRR17520069.4522975
SRR17520069.4532015
SRR17520069.4537195
SRR17520069.4537354
SRR17520069.4611853
SRR17520069.4639639
SRR17520069.4644898
SRR17520069.4680962
SRR17520069.4691623
SRR17520069.4693023

SRR17520069.4719665
SRR17520069.4720281
SRR17520069.4735369
SRR17520069.4764505
SRR17520069.4766796
SRR17520069.4813664
SRR17520069.4837227
SRR17520069.4931305
SRR17520069.4931314
SRR17520069.4988366
SRR17520069.4988387
SRR17520069.5016512
SRR17520069.5033157
SRR17520069.5033185
SRR17520069.5042441
SRR17520069.5042443
SRR17520069.5051038
SRR17520069.5056968
SRR17520069.5094253
SRR17520069.5114279
SRR17520069.5114797
SRR17520069.5118687
SRR17520069.5206567
SRR17520069.5224472
SRR17520069.5242349
SRR17520069.5303084
SRR17520069.5313441
SRR17520069.5313442
SRR17520069.5313443
SRR17520069.5313447
SRR17520069.5313468
SRR17520069.5360996
SRR17520069.5373449
SRR17520069.5375076
SRR17520069.5375077
SRR17520069.5375078
SRR17520069.5379294
SRR17520069.5385343
SRR17520069.5429534
SRR17520069.5430439
SRR17520069.5438812
SRR17520069.5496366
SRR17520069.5496368
SRR17520069.5496369
SRR17520069.5504308
SRR17520069.5513497
SRR17520069.5524366

SRR17520069.5558971
SRR17520069.5601834
SRR17520069.5601898
SRR17520069.5601948
SRR17520069.5601964
SRR17520069.5601971
SRR17520069.5610061
SRR17520069.5612408
SRR17520069.5618277
SRR17520069.5625182
SRR17520069.5625189
SRR17520069.5630868
SRR17520069.5649790
SRR17520069.5666575
SRR17520069.5694702
SRR17520069.5717706
SRR17520069.5717709
SRR17520069.5717710
SRR17520069.5717721
SRR17520069.5719047
SRR17520069.5738396
SRR17520069.5738399
SRR17520069.5738422
SRR17520069.5738449
SRR17520069.5738460
SRR17520069.5738462
SRR17520069.5738489
SRR17520069.5774772
SRR17520069.5811828
SRR17520069.5871276
SRR17520069.5871376
SRR17520069.5871386
SRR17520069.5871389
SRR17520069.5871411
SRR17520069.5873907
SRR17520069.5873917
SRR17520069.5885260
SRR17520069.5889642
SRR17520069.5922991
SRR17520069.5947172
SRR17520069.5994437
SRR17520069.6026662
SRR17520069.6040997
SRR17520069.6041159
SRR17520069.6054510
SRR17520069.6057002
SRR17520069.6057072

SRR17520069.6057075
SRR17520069.6057084
SRR17520069.6057110
SRR17520069.6058469
SRR17520069.6085844
SRR17520069.6093336
SRR17520069.6097157
SRR17520069.6100069
SRR17520069.6138949
SRR17520069.6148609
SRR17520069.6155197
SRR17520069.6172618
SRR17520069.6215179
SRR17520069.6215193
SRR17520069.6215196
SRR17520069.6224227
SRR17520069.6233356
SRR17520069.6250867
SRR17520069.6250874
SRR17520069.6264886
SRR17520069.6339573
SRR17520069.6342618
SRR17520069.6350222
SRR17520069.6419901
SRR17520069.6446181
SRR17520069.6451871
SRR17520069.6472847
SRR17520069.6560855
SRR17520069.6578707
SRR17520069.6578795
SRR17520069.6596583
SRR17520069.6597685
SRR17520069.6674652
SRR17520069.6715076
SRR17520069.6725650
SRR17520069.6740214
SRR17520069.6751487
SRR17520069.6781830
SRR17520069.6792171
SRR17520069.6792715
SRR17520069.6795054
SRR17520069.6807505
SRR17520069.6807538
SRR17520069.6810395
SRR17520069.6821167
SRR17520069.6837580
SRR17520069.6857976

SRR17520069.6907178
SRR17520069.6936665
SRR17520069.6938491
SRR17520069.6995151
SRR17520069.7052936
SRR17520069.7096690
SRR17520069.7150442
SRR17520069.7192126
SRR17520069.7220727
SRR17520069.7224747
SRR17520069.7251462
SRR17520069.7261578
SRR17520069.7261579
SRR17520069.7261581
SRR17520069.7261582
SRR17520069.7261583
SRR17520069.7261584
SRR17520069.7261585
SRR17520069.7261587
SRR17520069.7261588
SRR17520069.7261589
SRR17520069.7261591
SRR17520069.7261592
SRR17520069.7261593
SRR17520069.7261594
SRR17520069.7261595
SRR17520069.7261596
SRR17520069.7261597
SRR17520069.7261599
SRR17520069.7261600
SRR17520069.7261601
SRR17520069.7261603
SRR17520069.7261604
SRR17520069.7261605
SRR17520069.7261606
SRR17520069.7261607
SRR17520069.7261608
SRR17520069.7261609
SRR17520069.7261610
SRR17520069.7261611
SRR17520069.7261612
SRR17520069.7261613
SRR17520069.7261614
SRR17520069.7261615
SRR17520069.7261616
SRR17520069.7261617
SRR17520069.7261618

SRR17520069.7261619
SRR17520069.7261620
SRR17520069.7261621
SRR17520069.7261622
SRR17520069.7261623
SRR17520069.7261624
SRR17520069.7261625
SRR17520069.7261627
SRR17520069.7261629
SRR17520069.7261630
SRR17520069.7261631
SRR17520069.7261632
SRR17520069.7261633
SRR17520069.7261635
SRR17520069.7261636
SRR17520069.7261637
SRR17520069.7261638
SRR17520069.7261639
SRR17520069.7261640
SRR17520069.7261641
SRR17520069.7261642
SRR17520069.7261643
SRR17520069.7261644
SRR17520069.7261645
SRR17520069.7261646
SRR17520069.7261647
SRR17520069.7261648
SRR17520069.7261649
SRR17520069.7261650
SRR17520069.7261651
SRR17520069.7261652
SRR17520069.7261653
SRR17520069.7261654
SRR17520069.7261655
SRR17520069.7261656
SRR17520069.7261657
SRR17520069.7261658
SRR17520069.7261659
SRR17520069.7261660
SRR17520069.7261661
SRR17520069.7261663
SRR17520069.7261664
SRR17520069.7261665
SRR17520069.7261666
SRR17520069.7261667
SRR17520069.7261668
SRR17520069.7261669

SRR17520069.7261671
SRR17520069.7261672
SRR17520069.7261673
SRR17520069.7261674
SRR17520069.7261675
SRR17520069.7261676
SRR17520069.7261677
SRR17520069.7261678
SRR17520069.7261679
SRR17520069.7261680
SRR17520069.7261681
SRR17520069.7261682
SRR17520069.7261683
SRR17520069.7261684
SRR17520069.7261685
SRR17520069.7261689
SRR17520069.7261690
SRR17520069.7261691
SRR17520069.7261693
SRR17520069.7261694
SRR17520069.7261695
SRR17520069.7269326
SRR17520069.7307347
SRR17520069.7388090
SRR17520069.7401868
SRR17520069.7472354
SRR17520069.7472378
SRR17520069.7478258
SRR17520069.7478277
SRR17520069.7542724
SRR17520069.7542725
SRR17520069.7568888
SRR17520069.7579002
SRR17520069.7579020
SRR17520069.7623727
SRR17520069.7648500
SRR17520069.7648501
SRR17520069.7655646
SRR17520069.7688319
SRR17520069.7699241
SRR17520069.7710933
SRR17520069.7710934
SRR17520069.7743141
SRR17520069.7744020
SRR17520069.7746639
SRR17520069.7755070
SRR17520069.7817787

SRR17520069.7817807
SRR17520069.7835762
SRR17520069.7883765
SRR17520069.7898633
SRR17520069.7907826
SRR17520069.7927261
SRR17520069.7980153
SRR17520069.8026532
SRR17520069.8074447
SRR17520069.8080878
SRR17520069.8163575
SRR17520069.8193580
SRR17520069.8216583
SRR17520069.8225006
SRR17520069.8255026
SRR17520069.8255093
SRR17520069.8255101
SRR17520069.8260342
SRR17520069.8306442
SRR17520069.8315781
SRR17520069.8344110
SRR17520069.8344111
SRR17520069.8375300
SRR17520069.8378555
SRR17520069.8394714
SRR17520069.8441895
SRR17520069.8443366
SRR17520069.8483933
SRR17520069.8519975
SRR17520069.8524186
SRR17520069.8525830
SRR17520069.8556819
SRR17520069.8556828
SRR17520069.8567524
SRR17520069.8577170
SRR17520069.8585901
SRR17520069.8602279
SRR17520069.8607415
SRR17520069.8633576
SRR17520069.8640020
SRR17520069.8648479
SRR17520069.8656291
SRR17520069.8700373
SRR17520069.8702690
SRR17520069.8705163
SRR17520069.8723369
SRR17520069.8753540

SRR17520069.8853018
SRR17520069.8853060
SRR17520069.8861457
SRR17520069.8879354
SRR17520069.8879412
SRR17520069.8879467
SRR17520069.8879472
SRR17520069.8947171
SRR17520069.8947178
SRR17520069.8950477
SRR17520069.8971226
SRR17520069.8989790
SRR17520069.9011856
SRR17520069.9083594
SRR17520069.9104898
SRR17520069.9157253
SRR17520069.9187367
SRR17520069.9187386
SRR17520069.9187400
SRR17520069.9187453
SRR17520069.9195809
SRR17520069.9198269
SRR17520069.9202392
SRR17520069.9210557
SRR17520069.9213518
SRR17520069.9224425
SRR17520069.9238097
SRR17520069.9273457
SRR17520069.9275183
SRR17520069.9289706
SRR17520069.9298362
SRR17520069.9303351
SRR17520069.9308140
SRR17520069.9312276
SRR17520069.9328396
SRR17520069.9357931
SRR17520069.9357934
SRR17520069.9369653
SRR17520069.9371768
SRR17520069.9470832
SRR17520069.9470887
SRR17520069.9472226
SRR17520069.9481560
SRR17520069.9491402
SRR17520069.9500231
SRR17520069.9505283
SRR17520069.9588894

SRR17520069.9590264
SRR17520069.9643287
SRR17520069.9644310
SRR17520069.9710113
SRR17520069.9716572
SRR17520069.9738499
SRR17520069.9738520
SRR17520069.9741097
SRR17520069.9766596
SRR17520069.9772863
SRR17520069.9790303
SRR17520069.9790312
SRR17520069.9792847
SRR17520069.9874484
SRR17520069.9885547
SRR17520069.9901128
SRR17520069.9901157
SRR17520069.9901164
SRR17520069.9901174
SRR17520069.9901183
SRR17520069.9969341
SRR17520069.9982502
SRR17520069.9983983
SRR17520069.9983991
SRR17520069.9992011
SRR17520069.9997037
SRR17520069.10039962
SRR17520069.10119394
SRR17520069.10119411
SRR17520069.10119420
SRR17520069.10119421
SRR17520069.10119423
SRR17520069.10119424
SRR17520069.10119433
SRR17520069.10119435
SRR17520069.10119447
SRR17520069.10126587
SRR17520069.10126593
SRR17520069.10126597
SRR17520069.10126620
SRR17520069.10126627
SRR17520069.10130210
SRR17520069.10147597
SRR17520069.10147599
SRR17520069.10147638
SRR17520069.10154857
SRR17520069.10199043

SRR17520069.10203448
SRR17520069.10208716
SRR17520069.10273597
SRR17520069.10317350
SRR17520069.10340105
SRR17520069.10363676
SRR17520069.10429223
SRR17520069.10446427
SRR17520069.10448478
SRR17520069.10491689
SRR17520069.10520622
SRR17520069.10533808
SRR17520069.10576815
SRR17520069.10605763
SRR17520069.10605795
SRR17520069.10617479
SRR17520069.10639090
SRR17520069.10699983
SRR17520069.10699990
SRR17520069.10716003
SRR17520069.10716015
SRR17520069.10730819
SRR17520069.10746428
SRR17520069.10794546
SRR17520069.10794595
SRR17520069.10798100
SRR17520069.10824119
SRR17520069.10848950
SRR17520069.10912158
SRR17520069.10921467
SRR17520069.10933658
SRR17520069.10980449
SRR17520069.10980450
SRR17520069.11030816
SRR17520069.11030827
SRR17520069.11030828
SRR17520069.11046049
SRR17520069.11124635
SRR17520069.11175842
SRR17520069.11208199
SRR17520069.11231884
SRR17520069.11241636
SRR17520069.11296684
SRR17520069.11334103
SRR17520069.11336720
SRR17520069.11338647
SRR17520069.11378247

SRR17520069.11378252
SRR17520069.11378269
SRR17520069.11444091
SRR17520069.11444107
SRR17520069.11477998
SRR17520069.11530682
SRR17520069.11530688
SRR17520069.11530691
SRR17520069.11542368
SRR17520069.11615838
SRR17520069.11627935
SRR17520069.11627979
SRR17520069.11627984
SRR17520069.11627990
SRR17520069.11627998
SRR17520069.11644669
SRR17520069.11644670
SRR17520069.11644671
SRR17520069.11644672
SRR17520069.11736213
SRR17520069.11744260
SRR17520069.11777284
SRR17520069.11777286
SRR17520069.11780119
SRR17520069.11789973
SRR17520069.11818689
SRR17520069.11843598
SRR17520069.11905272
SRR17520069.11914007
SRR17520069.11938720
SRR17520069.11974894
SRR17520069.11989074
SRR17520069.12056183
SRR17520069.12098548
SRR17520069.12155226
SRR17520069.12155245
SRR17520069.12174121
SRR17520069.12188882
SRR17520069.12201264
SRR17520069.12201300
SRR17520069.12264332
SRR17520069.12297334
SRR17520069.12297415
SRR17520069.12317788
SRR17520069.12348074
SRR17520069.12371423
SRR17520069.12371428

SRR17520069.12383826
SRR17520069.12449841
SRR17520069.12529794
SRR17520069.12531020
SRR17520069.12531047
SRR17520069.12542861
SRR17520069.12599539
SRR17520069.12604288
SRR17520069.12604744
SRR17520069.12638448
SRR17520069.12645525
SRR17520069.12736852
SRR17520069.12749700
SRR17520069.12797184
SRR17520069.12839968
SRR17520069.12941041
SRR17520069.12973141
SRR17520069.12973149
SRR17520069.12973169
SRR17520069.12973182
SRR17520069.12973230
SRR17520069.13042791
SRR17520069.13042795
SRR17520069.13042859
SRR17520069.13043901
SRR17520069.13057523
SRR17520069.13080319
SRR17520069.13128643
SRR17520069.13141242
SRR17520069.13146689
SRR17520069.13158900
SRR17520069.13174528
SRR17520069.13177702
SRR17520069.13223747
SRR17520069.13245965
SRR17520069.13261871
SRR17520069.13263597
SRR17520069.13343531
SRR17520069.13343538
SRR17520069.13390525
SRR17520069.13397479
SRR17520069.13401480
SRR17520069.13429726
SRR17520069.13448302
SRR17520069.13471200
SRR17520069.13484771
SRR17520069.13529058

SRR17520069.13529061
SRR17520069.13529064
SRR17520069.13529092
SRR17520069.13529095
SRR17520069.13529110
SRR17520069.13529142
SRR17520069.13529147
SRR17520069.13529150
SRR17520069.13529636
SRR17520069.13543710
SRR17520069.13543717
SRR17520069.13543720
SRR17520069.13543725
SRR17520069.13543730
SRR17520069.13547133
SRR17520069.13554942
SRR17520069.13582698
SRR17520069.13584093
SRR17520069.13584095
SRR17520069.13584125
SRR17520069.13584145
SRR17520069.13584169
SRR17520069.13608190
SRR17520069.13608653
SRR17520069.13654263
SRR17520069.13672618
SRR17520069.13672629
SRR17520069.13715387
SRR17520069.13757498
SRR17520069.13757499
SRR17520069.13757500
SRR17520069.13757501
SRR17520069.13757502
SRR17520069.13757503
SRR17520069.13757504
SRR17520069.13757505
SRR17520069.13757506
SRR17520069.13757507
SRR17520069.13757508
SRR17520069.13757509
SRR17520069.13757510
SRR17520069.13757511
SRR17520069.13757512
SRR17520069.13757513
SRR17520069.13757514
SRR17520069.13757516
SRR17520069.13757517

SRR17520069.13757518
SRR17520069.13757519
SRR17520069.13757520
SRR17520069.13757521
SRR17520069.13757522
SRR17520069.13757524
SRR17520069.13757525
SRR17520069.13757526
SRR17520069.13757527
SRR17520069.13757528
SRR17520069.13757529
SRR17520069.13757530
SRR17520069.13757531
SRR17520069.13757533
SRR17520069.13757534
SRR17520069.13757536
SRR17520069.13757537
SRR17520069.13757538
SRR17520069.13757539
SRR17520069.13757540
SRR17520069.13757541
SRR17520069.13757542
SRR17520069.13757543
SRR17520069.13757544
SRR17520069.13757546
SRR17520069.13757547
SRR17520069.13757548
SRR17520069.13757549
SRR17520069.13757550
SRR17520069.13757551
SRR17520069.13757552
SRR17520069.13757553
SRR17520069.13757554
SRR17520069.13757555
SRR17520069.13757556
SRR17520069.13757557
SRR17520069.13757558
SRR17520069.13757559
SRR17520069.13757560
SRR17520069.13757561
SRR17520069.13757562
SRR17520069.13757563
SRR17520069.13757564
SRR17520069.13757565
SRR17520069.13757566
SRR17520069.13757567
SRR17520069.13757568

SRR17520069.13757569
SRR17520069.13757570
SRR17520069.13757571
SRR17520069.13757572
SRR17520069.13757573
SRR17520069.13757574
SRR17520069.13757575
SRR17520069.13757576
SRR17520069.13757577
SRR17520069.13757578
SRR17520069.13757579
SRR17520069.13757580
SRR17520069.13757581
SRR17520069.13757582
SRR17520069.13757583
SRR17520069.13757584
SRR17520069.13757585
SRR17520069.13757586
SRR17520069.13757587
SRR17520069.13757588
SRR17520069.13757589
SRR17520069.13757590
SRR17520069.13757591
SRR17520069.13757592
SRR17520069.13757593
SRR17520069.13757594
SRR17520069.13757595
SRR17520069.13757596
SRR17520069.13757597
SRR17520069.13757598
SRR17520069.13757599
SRR17520069.13757600
SRR17520069.13757601
SRR17520069.13757602
SRR17520069.13757603
SRR17520069.13757604
SRR17520069.13757605
SRR17520069.13757606
SRR17520069.13757607
SRR17520069.13757608
SRR17520069.13757609
SRR17520069.13757610
SRR17520069.13757611
SRR17520069.13757612
SRR17520069.13757614
SRR17520069.13757615
SRR17520069.13757616

SRR17520069.13757617
SRR17520069.13757618
SRR17520069.13757619
SRR17520069.13757620
SRR17520069.13757621
SRR17520069.13757622
SRR17520069.13757623
SRR17520069.13757624
SRR17520069.13767606
SRR17520069.13777123
SRR17520069.13802029
SRR17520069.13840882
SRR17520069.13840889
SRR17520069.13840890
SRR17520069.13840893
SRR17520069.13841744
SRR17520069.13860997
SRR17520069.13861978
SRR17520069.13884507
SRR17520069.13923584
SRR17520069.13936062
SRR17520069.13946239
SRR17520069.13987563
SRR17520069.14013063
SRR17520069.14034188
SRR17520069.14058870
SRR17520069.14058871
SRR17520069.14058878
SRR17520069.14058885
SRR17520069.14058891
SRR17520069.14145660
SRR17520069.14171321
SRR17520069.14171324
SRR17520069.14171329
SRR17520069.14171337
SRR17520069.14227434
SRR17520069.14264070
SRR17520069.14326824
SRR17520069.14361514
SRR17520069.14378128
SRR17520069.14378155
SRR17520069.14378228
SRR17520069.14378240
SRR17520069.14381707
SRR17520069.14399907
SRR17520069.14404030
SRR17520069.14404044

SRR17520069.14404062
SRR17520069.14451005
SRR17520069.14471262
SRR17520069.14481948
SRR17520069.14504791
SRR17520069.14508720
SRR17520069.14526040
SRR17520069.14547394
SRR17520069.14607639
SRR17520069.14629706
SRR17520069.14641533
SRR17520069.14641534
SRR17520069.14653712
SRR17520069.14672098
SRR17520069.14687085
SRR17520069.14693818
SRR17520069.14722874
SRR17520069.14782158
SRR17520069.14782162
SRR17520069.14782168
SRR17520069.14782173
SRR17520069.14782183
SRR17520069.14798081
SRR17520069.14805189
SRR17520069.14824840
SRR17520069.14873356
SRR17520069.14873369
SRR17520069.14873373
SRR17520069.14958564
SRR17520069.15015196
SRR17520069.15015197
SRR17520069.15015198
SRR17520069.15015199
SRR17520069.15015200
SRR17520069.15088458
SRR17520069.15123283
SRR17520069.15132378
SRR17520069.15160310
SRR17520069.15167752
SRR17520069.15194047
SRR17520069.15246374
SRR17520069.15272058
SRR17520069.15293871
SRR17520069.15300323
SRR17520069.15302702
SRR17520069.15304756
SRR17520069.15464458

SRR17520069.15477698
SRR17520069.15477737
SRR17520069.15534053
SRR17520069.15534057
SRR17520069.15566451
SRR17520069.15578420
SRR17520069.15614978
SRR17520069.15669319
SRR17520069.15685348
SRR17520069.15685349
SRR17520069.15685373
SRR17520069.15690260
SRR17520069.15786168
SRR17520069.15812876
SRR17520069.15838053
SRR17520069.15853983
SRR17520069.15894797
SRR17520069.15895539
SRR17520069.15915546
SRR17520069.15956970
SRR17520069.15970377
SRR17520069.15975372
SRR17520069.16001598
SRR17520069.16041890
SRR17520069.16045254
SRR17520069.16060278
SRR17520069.16075749
SRR17520069.16086557
SRR17520069.16090166
SRR17520069.16092244
SRR17520069.16092259
SRR17520069.16092260
SRR17520069.16092263
SRR17520069.16092355
SRR17520069.16130018
SRR17520069.16201789
SRR17520069.16223984
SRR17520069.16223989
SRR17520069.16223994
SRR17520069.16242856
SRR17520069.16245832
SRR17520069.16256589
SRR17520069.16298573
SRR17520069.16305596
SRR17520069.16306681
SRR17520069.16326965
SRR17520069.16326975

SRR17520069.16326981
SRR17520069.16342648
SRR17520069.16361950
SRR17520069.16382145
SRR17520069.16410206
SRR17520069.16422662
SRR17520069.16440017
SRR17520069.16440020
SRR17520069.16450393
SRR17520069.16517045
SRR17520069.16517047
SRR17520069.16517094
SRR17520069.16567311
SRR17520069.16567338
SRR17520069.16569793
SRR17520069.16612868
SRR17520069.16625822
SRR17520069.16697594
SRR17520069.16697617
SRR17520069.16697618
SRR17520069.16697627
SRR17520069.16697660
SRR17520069.16697671
SRR17520069.16697703
SRR17520069.16697721
SRR17520069.16697730
SRR17520069.16697741
SRR17520069.16701767
SRR17520069.16730356
SRR17520069.16743005
SRR17520069.16799381
SRR17520069.16822121
SRR17520069.16822178
SRR17520069.16899741
SRR17520069.16902215
SRR17520069.16903368
SRR17520069.16911470
SRR17520069.16981501
SRR17520069.16983860
SRR17520069.17048314
SRR17520069.17065901
SRR17520069.17077658
SRR17520069.17079793
SRR17520069.17079794
SRR17520069.17079800
SRR17520069.17079815
SRR17520069.17088684

SRR17520069.17116170
SRR17520069.17274977
SRR17520069.17306350
SRR17520069.17311921
SRR17520069.17337154
SRR17520069.17403798
SRR17520069.17411934
SRR17520069.17411939
SRR17520069.17411951
SRR17520069.17411952
SRR17520069.17411958
SRR17520069.17411965
SRR17520069.17411967
SRR17520069.17411968
SRR17520069.17411969
SRR17520069.17411971
SRR17520069.17427440
SRR17520069.17427491
SRR17520069.17439752
SRR17520069.17452381
SRR17520069.17473194
SRR17520069.17484077
SRR17520069.17526117
SRR17520069.17526128
SRR17520069.17557157
SRR17520069.17593661
SRR17520069.17608105
SRR17520069.17648773
SRR17520069.17651379
SRR17520069.17662819
SRR17520069.17671072
SRR17520069.17687534
SRR17520069.17865553
SRR17520069.17865570
SRR17520069.17883948
SRR17520069.17932041
SRR17520069.17933030
SRR17520069.17966147
SRR17520069.17966182
SRR17520069.17966187
SRR17520069.17966188
SRR17520069.17966193
SRR17520069.18001528
SRR17520069.18001531
SRR17520069.18008340
SRR17520069.18025337
SRR17520069.18035837

SRR17520069.18057654
SRR17520069.18057656
SRR17520069.18098457
SRR17520069.18126045
SRR17520069.18151973
SRR17520069.18152781
SRR17520069.18188392
SRR17520069.18188403
SRR17520069.18190143
SRR17520069.18190152
SRR17520069.18280449
SRR17520069.18324873
SRR17520069.18327285
SRR17520069.18329832
SRR17520069.18358750
SRR17520069.18358772
SRR17520069.18358801
SRR17520069.18358841
SRR17520069.18396426
SRR17520069.18410585
SRR17520069.18431203
SRR17520069.18439450
SRR17520069.18466305
SRR17520069.18528664
SRR17520069.18581575
SRR17520069.18588426
SRR17520069.18623903
SRR17520069.18744609
SRR17520069.18767379
SRR17520069.18817723
SRR17520069.18823348
SRR17520069.18826849
SRR17520069.18850644
SRR17520069.18850661
SRR17520069.18894653
SRR17520069.18918979
SRR17520069.18924529
SRR17520069.18986998
SRR17520069.19021155
SRR17520069.19021178
SRR17520069.19055104
SRR17520069.19064170
SRR17520069.19064227
SRR17520069.19068214
SRR17520069.19081893
SRR17520069.19081947
SRR17520069.19081951

SRR17520069.19099797
SRR17520069.19099798
SRR17520069.19103720
SRR17520069.19123801
SRR17520069.19129312
SRR17520069.19144136
SRR17520069.19175341
SRR17520069.19175344
SRR17520069.19201763
SRR17520069.19243002
SRR17520069.19280538
SRR17520069.19365916
SRR17520069.19368278
SRR17520069.19378164
SRR17520069.19443211
SRR17520069.19460325
SRR17520069.19460326
SRR17520069.19560281
SRR17520069.19560284
SRR17520069.19567122
SRR17520069.19567123
SRR17520069.1028
SRR17520069.1030
SRR17520069.1032
SRR17520069.1036
SRR17520069.2813
SRR17520069.2815
SRR17520069.2817
SRR17520069.2819
SRR17520069.2821
SRR17520069.2823
SRR17520069.2825
SRR17520069.2827
SRR17520069.2829
SRR17520069.2831
SRR17520069.2833
SRR17520069.2837
SRR17520069.2839
SRR17520069.2843
SRR17520069.2845
SRR17520069.2847
SRR17520069.2849
SRR17520069.2851
SRR17520069.2853
SRR17520069.2855
SRR17520069.2857
SRR17520069.2859

SRR17520069.2863
SRR17520069.2865
SRR17520069.2867
SRR17520069.2869
SRR17520069.2871
SRR17520069.2873
SRR17520069.2875
SRR17520069.2877
SRR17520069.5315
SRR17520069.5317
SRR17520069.5319
SRR17520069.5321
SRR17520069.5323
SRR17520069.5325
SRR17520069.5327
SRR17520069.7613
SRR17520069.7615
SRR17520069.7617
SRR17520069.7619
SRR17520069.7621
SRR17520069.7623
SRR17520069.7625
SRR17520069.7627
SRR17520069.7629
SRR17520069.7631
SRR17520069.7633
SRR17520069.7635
SRR17520069.7637
SRR17520069.7641
SRR17520069.7643
SRR17520069.7645
SRR17520069.7647
SRR17520069.7649
SRR17520069.7651
SRR17520069.7653
SRR17520069.7655
SRR17520069.7659
SRR17520069.7661
SRR17520069.7663
SRR17520069.7665
SRR17520069.7667
SRR17520069.7669
SRR17520069.7671
SRR17520069.7673
SRR17520069.7681
SRR17520069.7683
SRR17520069.7687

SRR17520069.9786
SRR17520069.9788
SRR17520069.9790
SRR17520069.9792
SRR17520069.9794
SRR17520069.9796
SRR17520069.9798
SRR17520069.11689
SRR17520069.11691
SRR17520069.11693
SRR17520069.13808
SRR17520069.13810
SRR17520069.13812
SRR17520069.23542
SRR17520069.23544
SRR17520069.23546
SRR17520069.23548
SRR17520069.23550
SRR17520069.23552
SRR17520069.23554
SRR17520069.23556
SRR17520069.23558
SRR17520069.23560
SRR17520069.23564
SRR17520069.23566
SRR17520069.23568
SRR17520069.23572
SRR17520069.23805
SRR17520069.23807
SRR17520069.23809
SRR17520069.23811
SRR17520069.23813
SRR17520069.23815
SRR17520069.23819
SRR17520069.23821
SRR17520069.23823
SRR17520069.23825
SRR17520069.23827
SRR17520069.23829
SRR17520069.23831
SRR17520069.23833
SRR17520069.23835
SRR17520069.23837
SRR17520069.23839
SRR17520069.23841
SRR17520069.23843
SRR17520069.23847

SRR17520069.23849
SRR17520069.23851
SRR17520069.25705
SRR17520069.30009
SRR17520069.30011
SRR17520069.30015
SRR17520069.30017
SRR17520069.30019
SRR17520069.30021
SRR17520069.30023
SRR17520069.30025
SRR17520069.30027
SRR17520069.30029
SRR17520069.30033
SRR17520069.30035
SRR17520069.30037
SRR17520069.30039
SRR17520069.30041
SRR17520069.30043
SRR17520069.30045
SRR17520069.30047
SRR17520069.30049
SRR17520069.30051
SRR17520069.30053
SRR17520069.30057
SRR17520069.30059
SRR17520069.30061
SRR17520069.30063
SRR17520069.30065
SRR17520069.30067
SRR17520069.30071
SRR17520069.30073
SRR17520069.30075
SRR17520069.32072
SRR17520069.32107
SRR17520069.34516
SRR17520069.36684
SRR17520069.36686
SRR17520069.36688
SRR17520069.36690
SRR17520069.36692
SRR17520069.36696
SRR17520069.36698
SRR17520069.36700
SRR17520069.36702
SRR17520069.36704
SRR17520069.53633

SRR17520069.53635
SRR17520069.53637
SRR17520069.53639
SRR17520069.53641
SRR17520069.53643
SRR17520069.53645
SRR17520069.53647
SRR17520069.53649
SRR17520069.53653
SRR17520069.53655
SRR17520069.53657
SRR17520069.53659
SRR17520069.53661
SRR17520069.53663
SRR17520069.53665
SRR17520069.53667
SRR17520069.53669
SRR17520069.53671
SRR17520069.53673
SRR17520069.53677
SRR17520069.53679
SRR17520069.53681
SRR17520069.53683
SRR17520069.53685
SRR17520069.53687
SRR17520069.53689
SRR17520069.53691
SRR17520069.53693
SRR17520069.53695
SRR17520069.53697
SRR17520069.53699
SRR17520069.53701
SRR17520069.53703
SRR17520069.53705
SRR17520069.53707
SRR17520069.53709
SRR17520069.53711
SRR17520069.53713
SRR17520069.53717
SRR17520069.53719
SRR17520069.53721
SRR17520069.53723
SRR17520069.53725
SRR17520069.53729
SRR17520069.53731
SRR17520069.53733
SRR17520069.53737

SRR17520069.53739
SRR17520069.53741
SRR17520069.53743
SRR17520069.53745
SRR17520069.53749
SRR17520069.53751
SRR17520069.53753
SRR17520069.53755
SRR17520069.53757
SRR17520069.53759
SRR17520069.53761
SRR17520069.53763
SRR17520069.53765
SRR17520069.53767
SRR17520069.53769
SRR17520069.53771
SRR17520069.53773
SRR17520069.53775
SRR17520069.53777
SRR17520069.53783
SRR17520069.53785
SRR17520069.53787
SRR17520069.53789
SRR17520069.53791
SRR17520069.53793
SRR17520069.53799
SRR17520069.53801
SRR17520069.53803
SRR17520069.53807
SRR17520069.53809
SRR17520069.53813
SRR17520069.53819
SRR17520069.53825
SRR17520069.56896
SRR17520069.56898
SRR17520069.56900
SRR17520069.56902
SRR17520069.56904
SRR17520069.56906
SRR17520069.56908
SRR17520069.56910
SRR17520069.56912
SRR17520069.56914
SRR17520069.56916
SRR17520069.56918
SRR17520069.56920
SRR17520069.56922

SRR17520069.56924
SRR17520069.56926
SRR17520069.57813
SRR17520069.60630
SRR17520069.60632
SRR17520069.60634
SRR17520069.60636
SRR17520069.60638
SRR17520069.60640
SRR17520069.60642
SRR17520069.60644
SRR17520069.60646
SRR17520069.60648
SRR17520069.60650
SRR17520069.60652
SRR17520069.60654
SRR17520069.60656
SRR17520069.60658
SRR17520069.60660
SRR17520069.60662
SRR17520069.62478
SRR17520069.62480
SRR17520069.62482
SRR17520069.62484
SRR17520069.62486
SRR17520069.62488
SRR17520069.62490
SRR17520069.62492
SRR17520069.62494
SRR17520069.62496
SRR17520069.62498
SRR17520069.62500
SRR17520069.62502
SRR17520069.62504
SRR17520069.62506
SRR17520069.62508
SRR17520069.64163
SRR17520069.64165
SRR17520069.64167
SRR17520069.64169
SRR17520069.64171
SRR17520069.64173
SRR17520069.64175
SRR17520069.64177
SRR17520069.64179
SRR17520069.64181
SRR17520069.64183

SRR17520069.64185
SRR17520069.64187
SRR17520069.64189
SRR17520069.65389
SRR17520069.65391
SRR17520069.65393
SRR17520069.65395
SRR17520069.65397
SRR17520069.65399
SRR17520069.65401
SRR17520069.65403
SRR17520069.65405
SRR17520069.65407
SRR17520069.65409
SRR17520069.65411
SRR17520069.65413
SRR17520069.79062
SRR17520069.79064
SRR17520069.79066
SRR17520069.79068
SRR17520069.79070
SRR17520069.79072
SRR17520069.79074
SRR17520069.79076
SRR17520069.79078
SRR17520069.79080
SRR17520069.79082
SRR17520069.79084
SRR17520069.79086
SRR17520069.103875
SRR17520069.103877
SRR17520069.103879
SRR17520069.103883
SRR17520069.103885
SRR17520069.103887
SRR17520069.103889
SRR17520069.103891
SRR17520069.106219
SRR17520069.106221
SRR17520069.106223
SRR17520069.106225
SRR17520069.106227
SRR17520069.106229
SRR17520069.108456
SRR17520069.108458
SRR17520069.108460
SRR17520069.108462

SRR17520069.108464
SRR17520069.108466
SRR17520069.108468
SRR17520069.108470
SRR17520069.108472
SRR17520069.114737
SRR17520069.114739
SRR17520069.114741
SRR17520069.114743
SRR17520069.114745
SRR17520069.114747
SRR17520069.114749
SRR17520069.114751
SRR17520069.114753
SRR17520069.114755
SRR17520069.114757
SRR17520069.114759
SRR17520069.114761
SRR17520069.114763
SRR17520069.114765
SRR17520069.114767
SRR17520069.114769
SRR17520069.114771
SRR17520069.114773
SRR17520069.114775
SRR17520069.114777
SRR17520069.114779
SRR17520069.114781
SRR17520069.114783
SRR17520069.114785
SRR17520069.114787
SRR17520069.114789
SRR17520069.114791
SRR17520069.114793
SRR17520069.114795
SRR17520069.114797
SRR17520069.114799
SRR17520069.114801
SRR17520069.114803
SRR17520069.123340
SRR17520069.123342
SRR17520069.123344
SRR17520069.123348
SRR17520069.123350
SRR17520069.123352
SRR17520069.123354
SRR17520069.123356

SRR17520069.124712
SRR17520069.124714
SRR17520069.124716
SRR17520069.124718
SRR17520069.124720
SRR17520069.124722
SRR17520069.124724
SRR17520069.124726
SRR17520069.124728
SRR17520069.124730
SRR17520069.124732
SRR17520069.124734
SRR17520069.124736
SRR17520069.124738
SRR17520069.124740
SRR17520069.124742
SRR17520069.124744
SRR17520069.124746
SRR17520069.124748
SRR17520069.124750
SRR17520069.124752
SRR17520069.124754
SRR17520069.124756
SRR17520069.124758
SRR17520069.124760
SRR17520069.124762
SRR17520069.124764
SRR17520069.125360
SRR17520069.125362
SRR17520069.125896
SRR17520069.126942
SRR17520069.126944
SRR17520069.126946
SRR17520069.126948
SRR17520069.126950
SRR17520069.126952
SRR17520069.126954
SRR17520069.126956
SRR17520069.131659
SRR17520069.131661
SRR17520069.131663
SRR17520069.131665
SRR17520069.131667
SRR17520069.131669
SRR17520069.131671
SRR17520069.131673
SRR17520069.131675

SRR17520069.131679
SRR17520069.131681
SRR17520069.131685
SRR17520069.131689
SRR17520069.131691
SRR17520069.131693
SRR17520069.131695
SRR17520069.131697
SRR17520069.145015
SRR17520069.145017
SRR17520069.145455
SRR17520069.145457
SRR17520069.145459
SRR17520069.145461
SRR17520069.145463
SRR17520069.145465
SRR17520069.145467
SRR17520069.145469
SRR17520069.145471
SRR17520069.145473
SRR17520069.145475
SRR17520069.145477
SRR17520069.145479
SRR17520069.145481
SRR17520069.145483
SRR17520069.145485
SRR17520069.145487
SRR17520069.145489
SRR17520069.145491
SRR17520069.145495
SRR17520069.145497
SRR17520069.145499
SRR17520069.145501
SRR17520069.145503
SRR17520069.145505
SRR17520069.145507
SRR17520069.145509
SRR17520069.145511
SRR17520069.145513
SRR17520069.145515
SRR17520069.145517
SRR17520069.145519
SRR17520069.145521
SRR17520069.145523
SRR17520069.145525
SRR17520069.145527
SRR17520069.145529

SRR17520069.145531
SRR17520069.145533
SRR17520069.145535
SRR17520069.145537
SRR17520069.145539
SRR17520069.145541
SRR17520069.145543
SRR17520069.145545
SRR17520069.145547
SRR17520069.145549
SRR17520069.145551
SRR17520069.145553
SRR17520069.145555
SRR17520069.145557
SRR17520069.145559
SRR17520069.145561
SRR17520069.145563
SRR17520069.145565
SRR17520069.145567
SRR17520069.145569
SRR17520069.145571
SRR17520069.145573
SRR17520069.145575
SRR17520069.145577
SRR17520069.145579
SRR17520069.145583
SRR17520069.145585
SRR17520069.145587
SRR17520069.145589
SRR17520069.145591
SRR17520069.145593
SRR17520069.145595
SRR17520069.145597
SRR17520069.145599
SRR17520069.145601
SRR17520069.145603
SRR17520069.145605
SRR17520069.145607
SRR17520069.145609
SRR17520069.145611
SRR17520069.148112
SRR17520069.148114
SRR17520069.148116
SRR17520069.148118
SRR17520069.148120
SRR17520069.148122
SRR17520069.148128

SRR17520069.148130
SRR17520069.148132
SRR17520069.148134
SRR17520069.148138
SRR17520069.148140
SRR17520069.148144
SRR17520069.148146
SRR17520069.148148
SRR17520069.148150
SRR17520069.148152
SRR17520069.148156
SRR17520069.148158
SRR17520069.148160
SRR17520069.148162
SRR17520069.148164
SRR17520069.148168
SRR17520069.148170
SRR17520069.148172
SRR17520069.148178
SRR17520069.148180
SRR17520069.148182
SRR17520069.148184
SRR17520069.148186
SRR17520069.148188
SRR17520069.148190
SRR17520069.148192
SRR17520069.148194
SRR17520069.151533
SRR17520069.151535
SRR17520069.151537
SRR17520069.151539
SRR17520069.151541
SRR17520069.151543
SRR17520069.151545
SRR17520069.151547
SRR17520069.151549
SRR17520069.151551
SRR17520069.151553
SRR17520069.151555
SRR17520069.151557
SRR17520069.151559
SRR17520069.151561
SRR17520069.151563
SRR17520069.151565
SRR17520069.151569
SRR17520069.151573
SRR17520069.151575

SRR17520069.151577
SRR17520069.151579
SRR17520069.151581
SRR17520069.151585
SRR17520069.151587
SRR17520069.151591
SRR17520069.154633
SRR17520069.154635
SRR17520069.154637
SRR17520069.154639
SRR17520069.154641
SRR17520069.154643
SRR17520069.154645
SRR17520069.154647
SRR17520069.154649
SRR17520069.154651
SRR17520069.154653
SRR17520069.154655
SRR17520069.154657
SRR17520069.154659
SRR17520069.154661
SRR17520069.154663
SRR17520069.154665
SRR17520069.154667
SRR17520069.154669
SRR17520069.154671
SRR17520069.154673
SRR17520069.154675
SRR17520069.154677
SRR17520069.154679
SRR17520069.154681
SRR17520069.154683
SRR17520069.154687
SRR17520069.154689
SRR17520069.154691
SRR17520069.154693
SRR17520069.154695
SRR17520069.154697
SRR17520069.154699
SRR17520069.154701
SRR17520069.154703
SRR17520069.154705
SRR17520069.154707
SRR17520069.154709
SRR17520069.154711
SRR17520069.154713
SRR17520069.154715

SRR17520069.154717
SRR17520069.154719
SRR17520069.155714
SRR17520069.155716
SRR17520069.155718
SRR17520069.155722
SRR17520069.155724
SRR17520069.155726
SRR17520069.155728
SRR17520069.155730
SRR17520069.155732
SRR17520069.155734
SRR17520069.155736
SRR17520069.155738
SRR17520069.155740
SRR17520069.155742
SRR17520069.155744
SRR17520069.155746
SRR17520069.155748
SRR17520069.155750
SRR17520069.155752
SRR17520069.155754
SRR17520069.155756
SRR17520069.155758
SRR17520069.155760
SRR17520069.155762
SRR17520069.155766
SRR17520069.155768
SRR17520069.155770
SRR17520069.155774
SRR17520069.155776
SRR17520069.155778
SRR17520069.155780
SRR17520069.155784
SRR17520069.155786
SRR17520069.155788
SRR17520069.155790
SRR17520069.155792
SRR17520069.155794
SRR17520069.155796
SRR17520069.155798
SRR17520069.155800
SRR17520069.155802
SRR17520069.155804
SRR17520069.155810
SRR17520069.155812
SRR17520069.155817

SRR17520069.155819
SRR17520069.155821
SRR17520069.160703
SRR17520069.160705
SRR17520069.160707
SRR17520069.160709
SRR17520069.160711
SRR17520069.160715
SRR17520069.160717
SRR17520069.160719
SRR17520069.160721
SRR17520069.160723
SRR17520069.160725
SRR17520069.160727
SRR17520069.160729
SRR17520069.160731
SRR17520069.160733
SRR17520069.160735
SRR17520069.160951
SRR17520069.160953
SRR17520069.160955
SRR17520069.160957
SRR17520069.160959
SRR17520069.160961
SRR17520069.160963
SRR17520069.160965
SRR17520069.160967
SRR17520069.160969
SRR17520069.160971
SRR17520069.160973
SRR17520069.160975
SRR17520069.160977
SRR17520069.160979
SRR17520069.160981
SRR17520069.160983
SRR17520069.160985
SRR17520069.160987
SRR17520069.160989
SRR17520069.160991
SRR17520069.160993
SRR17520069.160995
SRR17520069.160997
SRR17520069.160999
SRR17520069.161001
SRR17520069.161003
SRR17520069.161005
SRR17520069.161007

SRR17520069.161009
SRR17520069.161011
SRR17520069.162876
SRR17520069.162878
SRR17520069.162880
SRR17520069.162882
SRR17520069.162884
SRR17520069.162886
SRR17520069.162888
SRR17520069.162890
SRR17520069.162892
SRR17520069.162894
SRR17520069.162896
SRR17520069.162898
SRR17520069.162900
SRR17520069.162902
SRR17520069.162904
SRR17520069.162906
SRR17520069.162908
SRR17520069.162910
SRR17520069.162912
SRR17520069.162914
SRR17520069.162916
SRR17520069.162918
SRR17520069.162920
SRR17520069.165365
SRR17520069.165367
SRR17520069.165369
SRR17520069.165371
SRR17520069.165373
SRR17520069.165375
SRR17520069.165377
SRR17520069.165379
SRR17520069.165383
SRR17520069.167702
SRR17520069.167704
SRR17520069.167706
SRR17520069.167708
SRR17520069.167710
SRR17520069.169187
SRR17520069.176814
SRR17520069.176816
SRR17520069.176818
SRR17520069.176820
SRR17520069.176822
SRR17520069.176824
SRR17520069.176826

SRR17520069.176828
SRR17520069.176830
SRR17520069.176832
SRR17520069.176834
SRR17520069.176836
SRR17520069.176838
SRR17520069.176840
SRR17520069.176842
SRR17520069.176844
SRR17520069.176846
SRR17520069.176848
SRR17520069.176850
SRR17520069.176854
SRR17520069.176856
SRR17520069.176858
SRR17520069.176860
SRR17520069.176862
SRR17520069.176864
SRR17520069.176868
SRR17520069.176870
SRR17520069.176872
SRR17520069.176874
SRR17520069.176878
SRR17520069.176880
SRR17520069.179473
SRR17520069.179477
SRR17520069.179479
SRR17520069.179481
SRR17520069.179483
SRR17520069.179485
SRR17520069.179487
SRR17520069.179489
SRR17520069.179491
SRR17520069.179493
SRR17520069.179495
SRR17520069.179499
SRR17520069.179503
SRR17520069.179505
SRR17520069.179507
SRR17520069.179509
SRR17520069.179511
SRR17520069.179513
SRR17520069.179515
SRR17520069.186013
SRR17520069.186015
SRR17520069.186017
SRR17520069.186019

SRR17520069.186023
SRR17520069.186025
SRR17520069.186027
SRR17520069.186029
SRR17520069.186031
SRR17520069.186033
SRR17520069.186035
SRR17520069.186037
SRR17520069.187325
SRR17520069.187327
SRR17520069.187329
SRR17520069.187899
SRR17520069.187901
SRR17520069.187903
SRR17520069.187905
SRR17520069.187907
SRR17520069.187909
SRR17520069.187911
SRR17520069.187913
SRR17520069.187915
SRR17520069.187917
SRR17520069.187919
SRR17520069.187921
SRR17520069.187923
SRR17520069.187925
SRR17520069.187927
SRR17520069.187929
SRR17520069.187931
SRR17520069.187933
SRR17520069.187935
SRR17520069.187937
SRR17520069.187939
SRR17520069.187941
SRR17520069.189174
SRR17520069.189176
SRR17520069.189178
SRR17520069.193996
SRR17520069.193998
SRR17520069.194000
SRR17520069.194002
SRR17520069.194004
SRR17520069.194012
SRR17520069.194014
SRR17520069.194016
SRR17520069.194018
SRR17520069.194020
SRR17520069.194022

SRR17520069.194024
SRR17520069.194026
SRR17520069.194028
SRR17520069.194030
SRR17520069.194032
SRR17520069.194034
SRR17520069.194036
SRR17520069.194040
SRR17520069.194042
SRR17520069.194044
SRR17520069.194046
SRR17520069.194048
SRR17520069.194050
SRR17520069.196775
SRR17520069.196777
SRR17520069.196779
SRR17520069.196781
SRR17520069.196783
SRR17520069.197773
SRR17520069.197775
SRR17520069.198011
SRR17520069.198013
SRR17520069.198015
SRR17520069.198017
SRR17520069.198019
SRR17520069.198021
SRR17520069.198023
SRR17520069.198025
SRR17520069.198027
SRR17520069.198029
SRR17520069.198031
SRR17520069.198033
SRR17520069.198035
SRR17520069.200667
SRR17520069.200669
SRR17520069.202079
SRR17520069.202081
SRR17520069.202083
SRR17520069.202085
SRR17520069.202089
SRR17520069.204631
SRR17520069.204633
SRR17520069.204635
SRR17520069.204637
SRR17520069.207499
SRR17520069.207503
SRR17520069.207505

SRR17520069.207507
SRR17520069.207509
SRR17520069.207511
SRR17520069.207513
SRR17520069.216913
SRR17520069.217815
SRR17520069.217817
SRR17520069.217819
SRR17520069.217821
SRR17520069.217823
SRR17520069.217825
SRR17520069.218068
SRR17520069.218070
SRR17520069.218072
SRR17520069.218074
SRR17520069.218076
SRR17520069.218078
SRR17520069.218080
SRR17520069.218082
SRR17520069.218084
SRR17520069.218086
SRR17520069.218088
SRR17520069.218090
SRR17520069.218092
SRR17520069.218094
SRR17520069.218096
SRR17520069.218098
SRR17520069.218100
SRR17520069.218102
SRR17520069.218104
SRR17520069.218106
SRR17520069.218108
SRR17520069.218110
SRR17520069.218112
SRR17520069.218114
SRR17520069.218116
SRR17520069.218118
SRR17520069.218120
SRR17520069.218122
SRR17520069.218124
SRR17520069.218126
SRR17520069.218128
SRR17520069.218130
SRR17520069.218132
SRR17520069.218134
SRR17520069.218136
SRR17520069.218138

SRR17520069.218140
SRR17520069.218142
SRR17520069.218144
SRR17520069.218146
SRR17520069.218148
SRR17520069.218150
SRR17520069.218152
SRR17520069.218154
SRR17520069.218156
SRR17520069.218158
SRR17520069.218160
SRR17520069.218162
SRR17520069.218164
SRR17520069.218166
SRR17520069.218168
SRR17520069.218170
SRR17520069.218172
SRR17520069.218174
SRR17520069.218176
SRR17520069.218178
SRR17520069.218180
SRR17520069.218182
SRR17520069.218184
SRR17520069.218186
SRR17520069.218188
SRR17520069.218190
SRR17520069.224650
SRR17520069.224652
SRR17520069.224654
SRR17520069.224656
SRR17520069.224659
SRR17520069.224661
SRR17520069.224665
SRR17520069.224667
SRR17520069.224669
SRR17520069.224671
SRR17520069.224673
SRR17520069.224675
SRR17520069.224677
SRR17520069.224679
SRR17520069.224681
SRR17520069.224683
SRR17520069.224685
SRR17520069.224687
SRR17520069.224689
SRR17520069.225173
SRR17520069.225175

SRR17520069.225177
SRR17520069.225179
SRR17520069.225181
SRR17520069.225183
SRR17520069.225185
SRR17520069.225187
SRR17520069.225189
SRR17520069.225191
SRR17520069.225193
SRR17520069.225197
SRR17520069.225209
SRR17520069.227277
SRR17520069.227279
SRR17520069.227281
SRR17520069.227283
SRR17520069.227285
SRR17520069.227287
SRR17520069.227289
SRR17520069.227291
SRR17520069.227293
SRR17520069.227295
SRR17520069.227297
SRR17520069.234126
SRR17520069.234128
SRR17520069.234130
SRR17520069.234132
SRR17520069.234134
SRR17520069.234136
SRR17520069.234138
SRR17520069.234140
SRR17520069.234142
SRR17520069.234144
SRR17520069.234146
SRR17520069.234148
SRR17520069.234150
SRR17520069.234152
SRR17520069.234154
SRR17520069.234156
SRR17520069.234158
SRR17520069.234160
SRR17520069.234162
SRR17520069.234164
SRR17520069.234168
SRR17520069.234170
SRR17520069.234172
SRR17520069.234174
SRR17520069.234176

SRR17520069.234178
SRR17520069.234180
SRR17520069.234182
SRR17520069.234184
SRR17520069.234186
SRR17520069.234188
SRR17520069.234190
SRR17520069.234192
SRR17520069.234194
SRR17520069.234196
SRR17520069.234198
SRR17520069.234200
SRR17520069.234202
SRR17520069.234204
SRR17520069.234206
SRR17520069.234208
SRR17520069.234210
SRR17520069.234212
SRR17520069.234214
SRR17520069.234216
SRR17520069.234218
SRR17520069.234220
SRR17520069.234222
SRR17520069.234831
SRR17520069.234835
SRR17520069.234837
SRR17520069.234841
SRR17520069.234843
SRR17520069.234845
SRR17520069.234847
SRR17520069.234849
SRR17520069.234853
SRR17520069.234855
SRR17520069.234857
SRR17520069.234859
SRR17520069.234861
SRR17520069.234863
SRR17520069.234865
SRR17520069.234867
SRR17520069.234869
SRR17520069.234871
SRR17520069.234873
SRR17520069.234875
SRR17520069.234879
SRR17520069.234881
SRR17520069.234883
SRR17520069.234885

SRR17520069.234887
SRR17520069.234889
SRR17520069.234895
SRR17520069.234897
SRR17520069.234899
SRR17520069.234901
SRR17520069.234903
SRR17520069.234907
SRR17520069.234911
SRR17520069.234913
SRR17520069.234915
SRR17520069.234917
SRR17520069.234919
SRR17520069.238201
SRR17520069.238203
SRR17520069.238205
SRR17520069.238207
SRR17520069.238209
SRR17520069.238211
SRR17520069.238213
SRR17520069.238215
SRR17520069.238217
SRR17520069.238219
SRR17520069.238221
SRR17520069.238223
SRR17520069.238225
SRR17520069.238227
SRR17520069.238229
SRR17520069.238231
SRR17520069.238233
SRR17520069.238235
SRR17520069.239535
SRR17520069.245528
SRR17520069.245530
SRR17520069.245534
SRR17520069.245536
SRR17520069.245538
SRR17520069.245540
SRR17520069.245542
SRR17520069.245544
SRR17520069.245546
SRR17520069.245548
SRR17520069.245550
SRR17520069.245552
SRR17520069.245554
SRR17520069.245556
SRR17520069.245558

SRR17520069.245560
SRR17520069.245562
SRR17520069.245564
SRR17520069.245566
SRR17520069.245568
SRR17520069.245570
SRR17520069.245572
SRR17520069.245576
SRR17520069.245578
SRR17520069.245580
SRR17520069.245582
SRR17520069.245584
SRR17520069.245586
SRR17520069.245588
SRR17520069.245594
SRR17520069.245596
SRR17520069.245600
SRR17520069.245602
SRR17520069.245604
SRR17520069.245606
SRR17520069.245608
SRR17520069.245610
SRR17520069.245612
SRR17520069.245614
SRR17520069.245616
SRR17520069.245618
SRR17520069.245620
SRR17520069.245622
SRR17520069.245624
SRR17520069.245628
SRR17520069.245630
SRR17520069.245632
SRR17520069.245634
SRR17520069.245636
SRR17520069.245640
SRR17520069.245642
SRR17520069.245644
SRR17520069.252218
SRR17520069.252220
SRR17520069.252222
SRR17520069.252224
SRR17520069.252226
SRR17520069.252228
SRR17520069.252230
SRR17520069.252232
SRR17520069.252234
SRR17520069.252236

SRR17520069.252238
SRR17520069.252240
SRR17520069.252242
SRR17520069.252244
SRR17520069.252246
SRR17520069.253752
SRR17520069.253754
SRR17520069.253756
SRR17520069.257193
SRR17520069.257195
SRR17520069.257197
SRR17520069.257199
SRR17520069.257201
SRR17520069.257203
SRR17520069.257205
SRR17520069.257207
SRR17520069.257209
SRR17520069.257211
SRR17520069.257213
SRR17520069.257215
SRR17520069.257217
SRR17520069.257219
SRR17520069.257221
SRR17520069.257223
SRR17520069.257225
SRR17520069.257227
SRR17520069.261598
SRR17520069.261600
SRR17520069.261602
SRR17520069.261604
SRR17520069.261606
SRR17520069.261608
SRR17520069.261610
SRR17520069.262414
SRR17520069.262416
SRR17520069.262418
SRR17520069.262420
SRR17520069.262422
SRR17520069.262424
SRR17520069.262426
SRR17520069.262428
SRR17520069.262430
SRR17520069.263616
SRR17520069.263618
SRR17520069.263620
SRR17520069.263622
SRR17520069.263624

SRR17520069.263626
SRR17520069.263628
SRR17520069.263632
SRR17520069.265067
SRR17520069.265518
SRR17520069.265520
SRR17520069.265522
SRR17520069.265524
SRR17520069.265526
SRR17520069.265528
SRR17520069.265530
SRR17520069.265534
SRR17520069.265536
SRR17520069.266192
SRR17520069.266194
SRR17520069.266196
SRR17520069.266198
SRR17520069.266200
SRR17520069.266204
SRR17520069.266206
SRR17520069.266208
SRR17520069.266210
SRR17520069.266212
SRR17520069.266214
SRR17520069.266216
SRR17520069.266218
SRR17520069.266220
SRR17520069.266222
SRR17520069.266224
SRR17520069.266226
SRR17520069.266228
SRR17520069.266230
SRR17520069.266232
SRR17520069.274665
SRR17520069.274667
SRR17520069.274669
SRR17520069.274671
SRR17520069.274673
SRR17520069.274675
SRR17520069.274677
SRR17520069.274679
SRR17520069.274681
SRR17520069.274685
SRR17520069.274687
SRR17520069.274689
SRR17520069.274691
SRR17520069.274693

SRR17520069.274695
SRR17520069.274697
SRR17520069.274699
SRR17520069.274701
SRR17520069.274703
SRR17520069.274705
SRR17520069.274707
SRR17520069.274709
SRR17520069.274711
SRR17520069.274713
SRR17520069.274715
SRR17520069.274717
SRR17520069.274719
SRR17520069.274721
SRR17520069.274723
SRR17520069.279418
SRR17520069.279420
SRR17520069.279422
SRR17520069.279426
SRR17520069.279428
SRR17520069.279430
SRR17520069.279432
SRR17520069.279434
SRR17520069.279436
SRR17520069.279438
SRR17520069.279440
SRR17520069.279442
SRR17520069.279444
SRR17520069.279446
SRR17520069.279448
SRR17520069.279450
SRR17520069.279452
SRR17520069.279454
SRR17520069.279456
SRR17520069.279458
SRR17520069.279460
SRR17520069.279464
SRR17520069.279466
SRR17520069.279468
SRR17520069.279470
SRR17520069.279472
SRR17520069.279474
SRR17520069.279476
SRR17520069.279478
SRR17520069.279480
SRR17520069.279482
SRR17520069.279484

SRR17520069.279486
SRR17520069.279488
SRR17520069.279490
SRR17520069.279492
SRR17520069.279494
SRR17520069.279496
SRR17520069.279498
SRR17520069.279500
SRR17520069.279502
SRR17520069.279504
SRR17520069.279506
SRR17520069.279508
SRR17520069.279510
SRR17520069.279512
SRR17520069.279514
SRR17520069.279516
SRR17520069.279518
SRR17520069.279520
SRR17520069.279522
SRR17520069.288285
SRR17520069.288287
SRR17520069.288289
SRR17520069.288291
SRR17520069.288293
SRR17520069.288295
SRR17520069.288297
SRR17520069.288299
SRR17520069.288301
SRR17520069.288303
SRR17520069.288305
SRR17520069.288307
SRR17520069.288309
SRR17520069.288311
SRR17520069.288313
SRR17520069.288315
SRR17520069.288317
SRR17520069.288319
SRR17520069.288321
SRR17520069.288323
SRR17520069.288325
SRR17520069.288327
SRR17520069.288329
SRR17520069.288331
SRR17520069.288333
SRR17520069.288335
SRR17520069.288337
SRR17520069.288339

SRR17520069.288341
SRR17520069.288343
SRR17520069.288345
SRR17520069.288347
SRR17520069.288349
SRR17520069.288351
SRR17520069.293493
SRR17520069.293495
SRR17520069.293497
SRR17520069.293499
SRR17520069.293501
SRR17520069.293503
SRR17520069.293505
SRR17520069.293507
SRR17520069.293509
SRR17520069.293511
SRR17520069.293513
SRR17520069.293515
SRR17520069.293517
SRR17520069.293519
SRR17520069.293521
SRR17520069.293523
SRR17520069.293525
SRR17520069.293527
SRR17520069.293529
SRR17520069.293531
SRR17520069.293533
SRR17520069.293535
SRR17520069.293537
SRR17520069.293539
SRR17520069.293541
SRR17520069.297416
SRR17520069.297418
SRR17520069.297420
SRR17520069.297422
SRR17520069.297424
SRR17520069.297426
SRR17520069.297428
SRR17520069.297430
SRR17520069.297432
SRR17520069.297434
SRR17520069.297436
SRR17520069.297438
SRR17520069.297440
SRR17520069.297442
SRR17520069.297444
SRR17520069.298535

SRR17520069.298537
SRR17520069.298539
SRR17520069.298541
SRR17520069.298543
SRR17520069.298545
SRR17520069.298549
SRR17520069.298551
SRR17520069.298553
SRR17520069.298555
SRR17520069.298557
SRR17520069.298559
SRR17520069.298561
SRR17520069.299516
SRR17520069.300865
SRR17520069.300867
SRR17520069.300869
SRR17520069.300875
SRR17520069.300877
SRR17520069.300879
SRR17520069.300881
SRR17520069.300883
SRR17520069.300885
SRR17520069.300887
SRR17520069.300889
SRR17520069.300891
SRR17520069.300893
SRR17520069.300895
SRR17520069.301025
SRR17520069.301027
SRR17520069.301029
SRR17520069.301031
SRR17520069.301033
SRR17520069.301035
SRR17520069.301037
SRR17520069.301039
SRR17520069.301041
SRR17520069.301043
SRR17520069.301045
SRR17520069.301047
SRR17520069.301049
SRR17520069.301051
SRR17520069.305950
SRR17520069.305952
SRR17520069.305954
SRR17520069.305956
SRR17520069.305958
SRR17520069.305960

SRR17520069.305962
SRR17520069.311485
SRR17520069.311487
SRR17520069.311489
SRR17520069.311491
SRR17520069.311493
SRR17520069.311495
SRR17520069.311497
SRR17520069.311499
SRR17520069.311501
SRR17520069.311503
SRR17520069.311505
SRR17520069.311507
SRR17520069.311509
SRR17520069.311511
SRR17520069.311515
SRR17520069.311517
SRR17520069.311519
SRR17520069.311521
SRR17520069.311523
SRR17520069.311525
SRR17520069.311527
SRR17520069.311529
SRR17520069.311531
SRR17520069.311533
SRR17520069.311535
SRR17520069.311537
SRR17520069.311539
SRR17520069.311543
SRR17520069.311545
SRR17520069.311547
SRR17520069.311549
SRR17520069.311551
SRR17520069.311553
SRR17520069.311555
SRR17520069.311557
SRR17520069.311559
SRR17520069.311561
SRR17520069.311563
SRR17520069.311565
SRR17520069.311567
SRR17520069.311569
SRR17520069.311573
SRR17520069.311575
SRR17520069.311577
SRR17520069.311579
SRR17520069.311581

SRR17520069.311583
SRR17520069.311585
SRR17520069.311587
SRR17520069.311589
SRR17520069.311591
SRR17520069.311593
SRR17520069.311595
SRR17520069.311599
SRR17520069.311601
SRR17520069.311603
SRR17520069.311605
SRR17520069.311609
SRR17520069.311611
SRR17520069.311615
SRR17520069.311617
SRR17520069.311621
SRR17520069.311623
SRR17520069.311625
SRR17520069.311627
SRR17520069.311629
SRR17520069.311631
SRR17520069.312859
SRR17520069.312861
SRR17520069.312863
SRR17520069.312865
SRR17520069.312867
SRR17520069.312869
SRR17520069.312871
SRR17520069.312873
SRR17520069.312875
SRR17520069.312877
SRR17520069.312879
SRR17520069.312881
SRR17520069.312883
SRR17520069.312885
SRR17520069.312887
SRR17520069.312889
SRR17520069.312891
SRR17520069.312893
SRR17520069.312895
SRR17520069.312897
SRR17520069.312899
SRR17520069.312901
SRR17520069.312903
SRR17520069.312905
SRR17520069.312907
SRR17520069.312909

SRR17520069.312911
SRR17520069.312913
SRR17520069.312915
SRR17520069.312917
SRR17520069.312919
SRR17520069.312921
SRR17520069.312923
SRR17520069.312925
SRR17520069.312927
SRR17520069.312929
SRR17520069.312931
SRR17520069.312933
SRR17520069.312935
SRR17520069.312937
SRR17520069.312939
SRR17520069.312941
SRR17520069.312943
SRR17520069.312945
SRR17520069.312947
SRR17520069.312949
SRR17520069.312951
SRR17520069.312953
SRR17520069.312955
SRR17520069.312957
SRR17520069.312959
SRR17520069.312961
SRR17520069.312963
SRR17520069.312965
SRR17520069.312967
SRR17520069.312971
SRR17520069.312973
SRR17520069.312975
SRR17520069.312977
SRR17520069.312979
SRR17520069.312981
SRR17520069.313962
SRR17520069.313964
SRR17520069.313966
SRR17520069.313968
SRR17520069.313970
SRR17520069.313972
SRR17520069.313974
SRR17520069.313976
SRR17520069.317260
SRR17520069.317264
SRR17520069.321123
SRR17520069.321125

SRR17520069.321127
SRR17520069.321129
SRR17520069.321131
SRR17520069.321133
SRR17520069.321135
SRR17520069.321137
SRR17520069.321139
SRR17520069.321141
SRR17520069.321143
SRR17520069.321145
SRR17520069.321147
SRR17520069.321149
SRR17520069.321151
SRR17520069.321153
SRR17520069.321155
SRR17520069.321157
SRR17520069.321159
SRR17520069.321161
SRR17520069.325791
SRR17520069.325793
SRR17520069.325795
SRR17520069.325797
SRR17520069.325799
SRR17520069.325801
SRR17520069.325803
SRR17520069.325805
SRR17520069.325809
SRR17520069.325811
SRR17520069.328764
SRR17520069.328768
SRR17520069.328770
SRR17520069.328772
SRR17520069.328774
SRR17520069.328776
SRR17520069.328778
SRR17520069.328780
SRR17520069.332881
SRR17520069.332883
SRR17520069.332885
SRR17520069.332887
SRR17520069.332889
SRR17520069.332891
SRR17520069.332893
SRR17520069.332895
SRR17520069.332897
SRR17520069.332899
SRR17520069.332901

SRR17520069.332903
SRR17520069.332905
SRR17520069.332907
SRR17520069.332909
SRR17520069.332911
SRR17520069.332913
SRR17520069.341053
SRR17520069.341055
SRR17520069.341057
SRR17520069.341059
SRR17520069.341061
SRR17520069.341063
SRR17520069.341065
SRR17520069.341067
SRR17520069.341069
SRR17520069.341071
SRR17520069.341073
SRR17520069.341075
SRR17520069.341077
SRR17520069.341079
SRR17520069.341081
SRR17520069.341083
SRR17520069.341085
SRR17520069.341477
SRR17520069.341505
SRR17520069.343408
SRR17520069.343410
SRR17520069.344368
SRR17520069.344370
SRR17520069.347175
SRR17520069.347177
SRR17520069.347179
SRR17520069.348565
SRR17520069.348567
SRR17520069.348569
SRR17520069.348573
SRR17520069.348575
SRR17520069.348577
SRR17520069.348579
SRR17520069.348581
SRR17520069.348583
SRR17520069.348585
SRR17520069.348587
SRR17520069.348589
SRR17520069.348591
SRR17520069.348988
SRR17520069.348990

SRR17520069.348992
SRR17520069.354060
SRR17520069.354062
SRR17520069.354064
SRR17520069.354066
SRR17520069.354068
SRR17520069.354070
SRR17520069.354072
SRR17520069.354074
SRR17520069.359986
SRR17520069.359990
SRR17520069.359992
SRR17520069.359996
SRR17520069.359998
SRR17520069.360000
SRR17520069.360004
SRR17520069.360006
SRR17520069.360008
SRR17520069.360010
SRR17520069.360012
SRR17520069.360014
SRR17520069.360016
SRR17520069.360018
SRR17520069.360020
SRR17520069.360022
SRR17520069.360026
SRR17520069.360028
SRR17520069.360030
SRR17520069.360032
SRR17520069.360036
SRR17520069.360040
SRR17520069.360042
SRR17520069.360044
SRR17520069.363297
SRR17520069.363299
SRR17520069.366207
SRR17520069.366209
SRR17520069.366211
SRR17520069.366213
SRR17520069.366215
SRR17520069.366217
SRR17520069.366219
SRR17520069.366221
SRR17520069.366223
SRR17520069.366225
SRR17520069.366227
SRR17520069.366229

SRR17520069.366231
SRR17520069.366233
SRR17520069.366235
SRR17520069.366237
SRR17520069.366239
SRR17520069.366241
SRR17520069.366245
SRR17520069.366247
SRR17520069.366249
SRR17520069.366251
SRR17520069.366255
SRR17520069.366257
SRR17520069.366259
SRR17520069.366261
SRR17520069.366263
SRR17520069.366265
SRR17520069.366267
SRR17520069.366271
SRR17520069.366273
SRR17520069.366275
SRR17520069.366277
SRR17520069.366279
SRR17520069.366281
SRR17520069.366283
SRR17520069.366285
SRR17520069.366287
SRR17520069.366289
SRR17520069.366291
SRR17520069.366295
SRR17520069.366297
SRR17520069.366299
SRR17520069.366676
SRR17520069.366678
SRR17520069.366680
SRR17520069.366682
SRR17520069.366684
SRR17520069.366686
SRR17520069.366688
SRR17520069.366692
SRR17520069.366828
SRR17520069.366830
SRR17520069.366832
SRR17520069.366834
SRR17520069.366836
SRR17520069.366838
SRR17520069.366840
SRR17520069.366842

SRR17520069.366844
SRR17520069.366846
SRR17520069.366850
SRR17520069.366852
SRR17520069.366854
SRR17520069.366856
SRR17520069.366858
SRR17520069.366862
SRR17520069.366864
SRR17520069.366866
SRR17520069.366868
SRR17520069.366870
SRR17520069.366872
SRR17520069.366874
SRR17520069.366876
SRR17520069.366878
SRR17520069.366880
SRR17520069.366882
SRR17520069.366884
SRR17520069.366886
SRR17520069.366888
SRR17520069.366890
SRR17520069.366892
SRR17520069.366894
SRR17520069.366896
SRR17520069.366898
SRR17520069.366900
SRR17520069.366902
SRR17520069.366904
SRR17520069.366906
SRR17520069.366908
SRR17520069.366910
SRR17520069.366912
SRR17520069.366914
SRR17520069.366916
SRR17520069.366918
SRR17520069.366920
SRR17520069.366922
SRR17520069.366924
SRR17520069.366928
SRR17520069.366930
SRR17520069.366932
SRR17520069.366934
SRR17520069.366936
SRR17520069.366938
SRR17520069.366942
SRR17520069.366944

SRR17520069.366946
SRR17520069.366948
SRR17520069.366950
SRR17520069.366952
SRR17520069.366954
SRR17520069.366956
SRR17520069.366958
SRR17520069.366960
SRR17520069.366962
SRR17520069.366964
SRR17520069.366966
SRR17520069.366968
SRR17520069.366970
SRR17520069.366972
SRR17520069.366974
SRR17520069.366976
SRR17520069.366978
SRR17520069.366980
SRR17520069.366982
SRR17520069.366984
SRR17520069.366986
SRR17520069.366988
SRR17520069.366990
SRR17520069.366992
SRR17520069.366994
SRR17520069.366996
SRR17520069.366998
SRR17520069.367000
SRR17520069.367004
SRR17520069.367006
SRR17520069.367008
SRR17520069.367010
SRR17520069.367014
SRR17520069.367018
SRR17520069.367020
SRR17520069.367022
SRR17520069.367024
SRR17520069.367026
SRR17520069.367028
SRR17520069.367030
SRR17520069.367032
SRR17520069.367034
SRR17520069.367036
SRR17520069.367038
SRR17520069.367040
SRR17520069.368217
SRR17520069.368219

SRR17520069.368221
SRR17520069.368223
SRR17520069.368225
SRR17520069.368227
SRR17520069.368229
SRR17520069.368231
SRR17520069.368233
SRR17520069.368235
SRR17520069.368237
SRR17520069.368241
SRR17520069.368243
SRR17520069.368245
SRR17520069.368247
SRR17520069.368249
SRR17520069.368251
SRR17520069.368253
SRR17520069.368255
SRR17520069.368257
SRR17520069.368259
SRR17520069.368261
SRR17520069.368263
SRR17520069.368265
SRR17520069.368267
SRR17520069.368269
SRR17520069.368271
SRR17520069.368273
SRR17520069.368275
SRR17520069.368277
SRR17520069.368279
SRR17520069.368281
SRR17520069.368283
SRR17520069.368285
SRR17520069.368287
SRR17520069.368289
SRR17520069.368291
SRR17520069.368293
SRR17520069.368295
SRR17520069.368299
SRR17520069.368301
SRR17520069.368303
SRR17520069.368305
SRR17520069.368307
SRR17520069.368309
SRR17520069.368311
SRR17520069.368313
SRR17520069.368315
SRR17520069.368317

SRR17520069.368319
SRR17520069.368321
SRR17520069.368325
SRR17520069.368327
SRR17520069.368329
SRR17520069.368333
SRR17520069.368339
SRR17520069.368341
SRR17520069.368343
SRR17520069.368347
SRR17520069.371546
SRR17520069.371548
SRR17520069.371550
SRR17520069.371552
SRR17520069.371556
SRR17520069.371558
SRR17520069.371560
SRR17520069.371562
SRR17520069.372400
SRR17520069.372402
SRR17520069.372408
SRR17520069.372410
SRR17520069.372412
SRR17520069.372414
SRR17520069.372416
SRR17520069.372418
SRR17520069.372420
SRR17520069.372422
SRR17520069.372424
SRR17520069.373154
SRR17520069.373156
SRR17520069.373158
SRR17520069.373160
SRR17520069.373162
SRR17520069.373164
SRR17520069.373166
SRR17520069.373168
SRR17520069.373170
SRR17520069.373172
SRR17520069.373174
SRR17520069.373176
SRR17520069.373178
SRR17520069.373180
SRR17520069.373182
SRR17520069.373184
SRR17520069.373186
SRR17520069.373188

SRR17520069.373190
SRR17520069.373192
SRR17520069.373194
SRR17520069.373196
SRR17520069.373198
SRR17520069.373200
SRR17520069.373202
SRR17520069.373204
SRR17520069.373206
SRR17520069.373208
SRR17520069.373210
SRR17520069.373212
SRR17520069.373214
SRR17520069.373216
SRR17520069.373218
SRR17520069.373220
SRR17520069.373222
SRR17520069.373224
SRR17520069.373226
SRR17520069.373228
SRR17520069.373232
SRR17520069.373234
SRR17520069.373236
SRR17520069.373238
SRR17520069.373240
SRR17520069.373242
SRR17520069.373244
SRR17520069.373246
SRR17520069.373248
SRR17520069.381850
SRR17520069.381852
SRR17520069.381854
SRR17520069.381856
SRR17520069.381858
SRR17520069.381860
SRR17520069.381862
SRR17520069.381864
SRR17520069.381866
SRR17520069.381868
SRR17520069.381870
SRR17520069.381872
SRR17520069.381874
SRR17520069.381876
SRR17520069.381878
SRR17520069.381880
SRR17520069.381882
SRR17520069.381884

SRR17520069.381886
SRR17520069.381888
SRR17520069.381890
SRR17520069.381892
SRR17520069.381894
SRR17520069.381896
SRR17520069.381898
SRR17520069.381900
SRR17520069.381902
SRR17520069.381904
SRR17520069.381906
SRR17520069.381908
SRR17520069.381910
SRR17520069.381912
SRR17520069.381914
SRR17520069.381916
SRR17520069.381918
SRR17520069.381920
SRR17520069.383638
SRR17520069.383640
SRR17520069.383642
SRR17520069.383644
SRR17520069.383648
SRR17520069.383650
SRR17520069.383652
SRR17520069.383654
SRR17520069.383658
SRR17520069.383660
SRR17520069.383662
SRR17520069.383664
SRR17520069.383666
SRR17520069.383668
SRR17520069.383674
SRR17520069.383676
SRR17520069.383678
SRR17520069.383680
SRR17520069.383682
SRR17520069.383684
SRR17520069.383686
SRR17520069.383688
SRR17520069.383690
SRR17520069.383692
SRR17520069.383694
SRR17520069.383698
SRR17520069.383702
SRR17520069.383704
SRR17520069.383706

SRR17520069.383708
SRR17520069.383710
SRR17520069.383712
SRR17520069.383714
SRR17520069.383716
SRR17520069.383718
SRR17520069.383720
SRR17520069.390299
SRR17520069.390301
SRR17520069.390416
SRR17520069.390420
SRR17520069.390424
SRR17520069.390426
SRR17520069.390428
SRR17520069.390430
SRR17520069.390432
SRR17520069.390434
SRR17520069.390436
SRR17520069.390438
SRR17520069.390440
SRR17520069.390442
SRR17520069.390444
SRR17520069.390448
SRR17520069.390450
SRR17520069.392350
SRR17520069.392352
SRR17520069.392354
SRR17520069.392356
SRR17520069.392358
SRR17520069.392360
SRR17520069.392362
SRR17520069.392364
SRR17520069.392366
SRR17520069.392368
SRR17520069.392370
SRR17520069.392372
SRR17520069.392374
SRR17520069.392376
SRR17520069.392378
SRR17520069.392380
SRR17520069.392382
SRR17520069.392384
SRR17520069.392386
SRR17520069.392388
SRR17520069.392390
SRR17520069.392392
SRR17520069.392394

SRR17520069.392396
SRR17520069.392398
SRR17520069.392400
SRR17520069.392402
SRR17520069.392404
SRR17520069.392406
SRR17520069.392408
SRR17520069.392410
SRR17520069.392412
SRR17520069.392414
SRR17520069.392416
SRR17520069.392418
SRR17520069.392420
SRR17520069.392422
SRR17520069.395156
SRR17520069.395158
SRR17520069.395162
SRR17520069.395164
SRR17520069.395166
SRR17520069.395168
SRR17520069.395170
SRR17520069.395172
SRR17520069.395174
SRR17520069.395176
SRR17520069.395178
SRR17520069.395180
SRR17520069.395182
SRR17520069.395184
SRR17520069.395186
SRR17520069.395188
SRR17520069.395190
SRR17520069.395192
SRR17520069.395194
SRR17520069.395196
SRR17520069.395200
SRR17520069.396359
SRR17520069.396361
SRR17520069.396363
SRR17520069.397652
SRR17520069.397654
SRR17520069.397656
SRR17520069.397658
SRR17520069.397660
SRR17520069.397662
SRR17520069.397664
SRR17520069.403102
SRR17520069.403104

SRR17520069.403106
SRR17520069.415809
SRR17520069.415811
SRR17520069.415813
SRR17520069.416112
SRR17520069.416116
SRR17520069.416118
SRR17520069.416120
SRR17520069.420365
SRR17520069.420367
SRR17520069.420369
SRR17520069.420371
SRR17520069.420373
SRR17520069.420375
SRR17520069.420377
SRR17520069.428752
SRR17520069.428754
SRR17520069.429195
SRR17520069.429197
SRR17520069.429199
SRR17520069.429201
SRR17520069.429203
SRR17520069.429205
SRR17520069.429207
SRR17520069.429209
SRR17520069.429211
SRR17520069.429213
SRR17520069.429215
SRR17520069.429217
SRR17520069.429219
SRR17520069.429221
SRR17520069.429223
SRR17520069.429225
SRR17520069.429227
SRR17520069.429229
SRR17520069.429231
SRR17520069.429233
SRR17520069.429235
SRR17520069.429237
SRR17520069.429239
SRR17520069.429241
SRR17520069.429243
SRR17520069.429245
SRR17520069.429247
SRR17520069.429249
SRR17520069.429251
SRR17520069.429253

SRR17520069.429255
SRR17520069.429257
SRR17520069.429261
SRR17520069.429263
SRR17520069.429265
SRR17520069.429267
SRR17520069.432425
SRR17520069.432427
SRR17520069.432429
SRR17520069.432431
SRR17520069.433542
SRR17520069.433544
SRR17520069.433548
SRR17520069.433550
SRR17520069.433552
SRR17520069.433554
SRR17520069.433556
SRR17520069.433558
SRR17520069.433560
SRR17520069.433564
SRR17520069.433566
SRR17520069.433568
SRR17520069.433570
SRR17520069.433572
SRR17520069.433574
SRR17520069.433576
SRR17520069.433578
SRR17520069.433580
SRR17520069.433582
SRR17520069.433584
SRR17520069.433586
SRR17520069.433588
SRR17520069.433590
SRR17520069.433592
SRR17520069.433594
SRR17520069.433596
SRR17520069.433598
SRR17520069.433600
SRR17520069.433602
SRR17520069.433606
SRR17520069.433608
SRR17520069.433610
SRR17520069.433612
SRR17520069.433614
SRR17520069.433616
SRR17520069.433618
SRR17520069.433620

SRR17520069.433622
SRR17520069.433624
SRR17520069.442094
SRR17520069.442096
SRR17520069.442098
SRR17520069.442100
SRR17520069.442102
SRR17520069.442104
SRR17520069.442624
SRR17520069.442626
SRR17520069.442628
SRR17520069.442630
SRR17520069.442634
SRR17520069.442636
SRR17520069.442638
SRR17520069.442640
SRR17520069.442642
SRR17520069.442644
SRR17520069.442646
SRR17520069.442648
SRR17520069.442650
SRR17520069.442652
SRR17520069.442654
SRR17520069.442656
SRR17520069.442658
SRR17520069.442660
SRR17520069.442662
SRR17520069.442664
SRR17520069.442666
SRR17520069.442668
SRR17520069.442670
SRR17520069.442672
SRR17520069.442674
SRR17520069.442676
SRR17520069.442678
SRR17520069.442680
SRR17520069.445448
SRR17520069.445450
SRR17520069.445452
SRR17520069.445454
SRR17520069.445456
SRR17520069.445458
SRR17520069.445460
SRR17520069.445462
SRR17520069.445464
SRR17520069.445466
SRR17520069.445468

SRR17520069.450357
SRR17520069.450359
SRR17520069.450361
SRR17520069.450363
SRR17520069.450365
SRR17520069.450367
SRR17520069.450369
SRR17520069.450371
SRR17520069.450373
SRR17520069.450375
SRR17520069.450377
SRR17520069.450379
SRR17520069.450381
SRR17520069.450383
SRR17520069.450385
SRR17520069.450387
SRR17520069.450389
SRR17520069.450391
SRR17520069.450393
SRR17520069.450395
SRR17520069.450397
SRR17520069.450399
SRR17520069.450401
SRR17520069.450403
SRR17520069.450405
SRR17520069.450407
SRR17520069.450409
SRR17520069.450411
SRR17520069.450413
SRR17520069.450415
SRR17520069.450417
SRR17520069.450419
SRR17520069.450421
SRR17520069.450423
SRR17520069.450425
SRR17520069.450427
SRR17520069.450429
SRR17520069.450431
SRR17520069.450433
SRR17520069.450620
SRR17520069.450624
SRR17520069.450626
SRR17520069.450628
SRR17520069.450630
SRR17520069.450632
SRR17520069.450634
SRR17520069.450636

SRR17520069.452112
SRR17520069.452116
SRR17520069.452118
SRR17520069.452120
SRR17520069.452122
SRR17520069.452124
SRR17520069.452126
SRR17520069.452128
SRR17520069.452130
SRR17520069.452132
SRR17520069.452134
SRR17520069.452136
SRR17520069.452138
SRR17520069.452140
SRR17520069.452142
SRR17520069.452144
SRR17520069.452146
SRR17520069.452148
SRR17520069.452150
SRR17520069.452152
SRR17520069.452154
SRR17520069.452156
SRR17520069.452158
SRR17520069.452160
SRR17520069.452162
SRR17520069.452164
SRR17520069.453791
SRR17520069.453819
SRR17520069.458650
SRR17520069.458652
SRR17520069.458654
SRR17520069.458656
SRR17520069.458658
SRR17520069.471473
SRR17520069.471475
SRR17520069.471477
SRR17520069.471479
SRR17520069.471481
SRR17520069.471483
SRR17520069.471485
SRR17520069.471487
SRR17520069.471489
SRR17520069.471491
SRR17520069.471493
SRR17520069.471495
SRR17520069.471497
SRR17520069.471499

SRR17520069.471885
SRR17520069.471887
SRR17520069.472621
SRR17520069.472623
SRR17520069.472625
SRR17520069.472627
SRR17520069.472629
SRR17520069.472631
SRR17520069.472635
SRR17520069.472637
SRR17520069.472639
SRR17520069.472641
SRR17520069.472643
SRR17520069.472645
SRR17520069.472647
SRR17520069.472649
SRR17520069.472651
SRR17520069.472653
SRR17520069.472655
SRR17520069.472657
SRR17520069.472659
SRR17520069.472661
SRR17520069.472663
SRR17520069.472665
SRR17520069.472669
SRR17520069.472671
SRR17520069.472673
SRR17520069.472675
SRR17520069.472677
SRR17520069.472679
SRR17520069.472681
SRR17520069.472683
SRR17520069.472685
SRR17520069.472687
SRR17520069.472689
SRR17520069.472691
SRR17520069.472693
SRR17520069.472697
SRR17520069.472701
SRR17520069.472703
SRR17520069.472705
SRR17520069.472707
SRR17520069.472709
SRR17520069.472711
SRR17520069.472713
SRR17520069.472715
SRR17520069.472717

SRR17520069.472719
SRR17520069.472721
SRR17520069.472723
SRR17520069.472725
SRR17520069.472727
SRR17520069.472729
SRR17520069.472731
SRR17520069.475108
SRR17520069.475110
SRR17520069.475114
SRR17520069.475116
SRR17520069.475118
SRR17520069.475120
SRR17520069.475122
SRR17520069.475126
SRR17520069.475128
SRR17520069.475130
SRR17520069.475132
SRR17520069.475134
SRR17520069.475136
SRR17520069.475138
SRR17520069.475140
SRR17520069.475142
SRR17520069.476805
SRR17520069.476807
SRR17520069.476809
SRR17520069.476811
SRR17520069.476813
SRR17520069.478827
SRR17520069.491026
SRR17520069.491037
SRR17520069.491043
SRR17520069.491405
SRR17520069.491407
SRR17520069.491409
SRR17520069.491411
SRR17520069.491413
SRR17520069.491415
SRR17520069.491419
SRR17520069.491421
SRR17520069.491423
SRR17520069.491425
SRR17520069.491427
SRR17520069.491429
SRR17520069.491431
SRR17520069.491433
SRR17520069.491435

SRR17520069.491437
SRR17520069.491439
SRR17520069.491441
SRR17520069.491443
SRR17520069.491445
SRR17520069.491447
SRR17520069.491449
SRR17520069.491451
SRR17520069.498804
SRR17520069.500899
SRR17520069.501527
SRR17520069.501529
SRR17520069.501531
SRR17520069.501533
SRR17520069.501535
SRR17520069.501537
SRR17520069.501539
SRR17520069.501541
SRR17520069.501543
SRR17520069.501545
SRR17520069.501547
SRR17520069.501549
SRR17520069.501551
SRR17520069.501553
SRR17520069.501555
SRR17520069.504404
SRR17520069.504406
SRR17520069.504408
SRR17520069.504410
SRR17520069.508120
SRR17520069.508122
SRR17520069.508124
SRR17520069.508126
SRR17520069.508128
SRR17520069.508130
SRR17520069.508132
SRR17520069.508134
SRR17520069.508136
SRR17520069.514051
SRR17520069.514053
SRR17520069.514055
SRR17520069.514057
SRR17520069.514059
SRR17520069.514061
SRR17520069.514063
SRR17520069.514065
SRR17520069.514067

SRR17520069.514069
SRR17520069.514071
SRR17520069.514073
SRR17520069.514075
SRR17520069.514077
SRR17520069.514079
SRR17520069.514081
SRR17520069.514083
SRR17520069.514085
SRR17520069.514087
SRR17520069.514089
SRR17520069.514091
SRR17520069.514093
SRR17520069.514095
SRR17520069.514097
SRR17520069.514099
SRR17520069.514101
SRR17520069.516660
SRR17520069.521937
SRR17520069.521939
SRR17520069.521941
SRR17520069.521943
SRR17520069.521945
SRR17520069.521947
SRR17520069.521949
SRR17520069.521951
SRR17520069.521953
SRR17520069.521955
SRR17520069.521957
SRR17520069.533919
SRR17520069.533921
SRR17520069.533923
SRR17520069.533925
SRR17520069.533927
SRR17520069.533929
SRR17520069.533931
SRR17520069.533933
SRR17520069.533935
SRR17520069.533937
SRR17520069.533939
SRR17520069.533941
SRR17520069.533943
SRR17520069.533945
SRR17520069.533947
SRR17520069.533949
SRR17520069.533951
SRR17520069.533953

SRR17520069.537950
SRR17520069.537952
SRR17520069.537954
SRR17520069.537956
SRR17520069.537958
SRR17520069.537960
SRR17520069.537962
SRR17520069.538211
SRR17520069.538215
SRR17520069.538217
SRR17520069.538219
SRR17520069.538221
SRR17520069.538223
SRR17520069.538225
SRR17520069.538227
SRR17520069.538229
SRR17520069.544135
SRR17520069.544137
SRR17520069.544139
SRR17520069.544141
SRR17520069.544143
SRR17520069.544145
SRR17520069.544147
SRR17520069.544149
SRR17520069.544153
SRR17520069.544155
SRR17520069.544157
SRR17520069.544159
SRR17520069.544161
SRR17520069.544163
SRR17520069.544165
SRR17520069.544167
SRR17520069.544169
SRR17520069.544171
SRR17520069.544173
SRR17520069.544177
SRR17520069.544179
SRR17520069.544181
SRR17520069.544183
SRR17520069.544185
SRR17520069.544187
SRR17520069.544189
SRR17520069.544191
SRR17520069.544193
SRR17520069.544195
SRR17520069.544197
SRR17520069.544199

SRR17520069.544201
SRR17520069.544203
SRR17520069.544205
SRR17520069.544207
SRR17520069.544209
SRR17520069.544211
SRR17520069.544213
SRR17520069.544215
SRR17520069.544217
SRR17520069.544219
SRR17520069.544221
SRR17520069.544223
SRR17520069.544225
SRR17520069.544227
SRR17520069.544229
SRR17520069.544231
SRR17520069.544233
SRR17520069.544235
SRR17520069.544945
SRR17520069.544947
SRR17520069.544951
SRR17520069.544953
SRR17520069.544955
SRR17520069.545728
SRR17520069.545730
SRR17520069.545732
SRR17520069.545734
SRR17520069.545736
SRR17520069.545738
SRR17520069.545740
SRR17520069.545742
SRR17520069.545744
SRR17520069.545746
SRR17520069.545748
SRR17520069.545750
SRR17520069.545752
SRR17520069.545754
SRR17520069.545756
SRR17520069.545758
SRR17520069.545760
SRR17520069.545762
SRR17520069.545764
SRR17520069.545766
SRR17520069.545768
SRR17520069.545770
SRR17520069.545772
SRR17520069.545774

SRR17520069.545776
SRR17520069.545778
SRR17520069.545780
SRR17520069.545782
SRR17520069.545784
SRR17520069.545786
SRR17520069.545788
SRR17520069.545790
SRR17520069.545792
SRR17520069.545794
SRR17520069.545796
SRR17520069.545798
SRR17520069.545800
SRR17520069.545802
SRR17520069.545804
SRR17520069.545806
SRR17520069.545808
SRR17520069.545810
SRR17520069.545812
SRR17520069.545814
SRR17520069.545816
SRR17520069.545818
SRR17520069.545820
SRR17520069.545822
SRR17520069.545824
SRR17520069.545826
SRR17520069.552321
SRR17520069.552323
SRR17520069.552325
SRR17520069.552327
SRR17520069.552329
SRR17520069.552331
SRR17520069.552333
SRR17520069.552335
SRR17520069.552345
SRR17520069.552347
SRR17520069.552349
SRR17520069.552351
SRR17520069.556344
SRR17520069.556346
SRR17520069.556348
SRR17520069.556350
SRR17520069.556352
SRR17520069.556354
SRR17520069.561098
SRR17520069.561100
SRR17520069.561102

SRR17520069.561104
SRR17520069.561106
SRR17520069.561108
SRR17520069.561110
SRR17520069.561112
SRR17520069.561114
SRR17520069.561116
SRR17520069.561118
SRR17520069.561120
SRR17520069.561122
SRR17520069.561124
SRR17520069.561126
SRR17520069.561128
SRR17520069.561130
SRR17520069.569769
SRR17520069.569771
SRR17520069.569773
SRR17520069.569775
SRR17520069.569777
SRR17520069.569779
SRR17520069.569887
SRR17520069.569889
SRR17520069.569891
SRR17520069.569893
SRR17520069.569895
SRR17520069.569897
SRR17520069.569899
SRR17520069.569901
SRR17520069.569903
SRR17520069.569905
SRR17520069.569907
SRR17520069.569909
SRR17520069.569911
SRR17520069.569913
SRR17520069.569915
SRR17520069.569917
SRR17520069.569919
SRR17520069.569921
SRR17520069.569923
SRR17520069.569925
SRR17520069.569927
SRR17520069.569929
SRR17520069.569931
SRR17520069.569933
SRR17520069.569935
SRR17520069.569937
SRR17520069.569939

SRR17520069.569941
SRR17520069.569943
SRR17520069.569945
SRR17520069.569947
SRR17520069.569949
SRR17520069.569951
SRR17520069.569953
SRR17520069.569955
SRR17520069.569957
SRR17520069.569959
SRR17520069.569961
SRR17520069.572377
SRR17520069.572379
SRR17520069.572381
SRR17520069.572383
SRR17520069.572385
SRR17520069.572387
SRR17520069.572389
SRR17520069.572391
SRR17520069.572393
SRR17520069.572395
SRR17520069.572397
SRR17520069.572399
SRR17520069.572401
SRR17520069.572403
SRR17520069.572405
SRR17520069.572407
SRR17520069.572409
SRR17520069.572411
SRR17520069.572413
SRR17520069.572415
SRR17520069.572417
SRR17520069.572419
SRR17520069.572421
SRR17520069.572429
SRR17520069.572431
SRR17520069.572433
SRR17520069.573113
SRR17520069.573115
SRR17520069.573117
SRR17520069.578635
SRR17520069.578637
SRR17520069.578639
SRR17520069.578641
SRR17520069.578643
SRR17520069.578645
SRR17520069.578647

SRR17520069.578651
SRR17520069.578653
SRR17520069.578655
SRR17520069.578657
SRR17520069.578659
SRR17520069.578661
SRR17520069.578663
SRR17520069.578665
SRR17520069.578667
SRR17520069.578669
SRR17520069.578671
SRR17520069.578840
SRR17520069.578842
SRR17520069.584293
SRR17520069.584295
SRR17520069.584297
SRR17520069.584299
SRR17520069.584301
SRR17520069.584303
SRR17520069.584305
SRR17520069.584307
SRR17520069.585079
SRR17520069.585378
SRR17520069.585380
SRR17520069.585382
SRR17520069.585384
SRR17520069.589028
SRR17520069.589030
SRR17520069.589032
SRR17520069.589034
SRR17520069.589036
SRR17520069.589038
SRR17520069.589040
SRR17520069.589042
SRR17520069.589044
SRR17520069.589046
SRR17520069.589048
SRR17520069.592043
SRR17520069.592045
SRR17520069.592047
SRR17520069.592049
SRR17520069.592051
SRR17520069.592053
SRR17520069.592055
SRR17520069.592057
SRR17520069.592061
SRR17520069.592063

SRR17520069.592065
SRR17520069.592067
SRR17520069.592069
SRR17520069.592071
SRR17520069.592073
SRR17520069.592075
SRR17520069.592077
SRR17520069.592079
SRR17520069.592880
SRR17520069.592882
SRR17520069.592884
SRR17520069.592886
SRR17520069.592888
SRR17520069.592890
SRR17520069.592892
SRR17520069.592894
SRR17520069.592896
SRR17520069.592898
SRR17520069.592900
SRR17520069.592902
SRR17520069.592904
SRR17520069.592906
SRR17520069.592908
SRR17520069.592910
SRR17520069.592912
SRR17520069.592914
SRR17520069.592916
SRR17520069.592918
SRR17520069.592920
SRR17520069.592922
SRR17520069.592924
SRR17520069.592926
SRR17520069.592928
SRR17520069.592930
SRR17520069.592932
SRR17520069.592934
SRR17520069.592936
SRR17520069.592938
SRR17520069.592940
SRR17520069.592942
SRR17520069.592944
SRR17520069.592946
SRR17520069.592948
SRR17520069.592950
SRR17520069.592952
SRR17520069.592954
SRR17520069.592956

SRR17520069.592958
SRR17520069.592960
SRR17520069.592962
SRR17520069.592964
SRR17520069.592966
SRR17520069.592968
SRR17520069.592970
SRR17520069.592972
SRR17520069.592974
SRR17520069.592976
SRR17520069.592978
SRR17520069.592980
SRR17520069.592982
SRR17520069.592984
SRR17520069.592986
SRR17520069.592988
SRR17520069.592990
SRR17520069.592992
SRR17520069.592996
SRR17520069.592998
SRR17520069.593000
SRR17520069.593002
SRR17520069.593004
SRR17520069.593006
SRR17520069.593008
SRR17520069.593010
SRR17520069.593012
SRR17520069.593014
SRR17520069.593016
SRR17520069.593018
SRR17520069.593020
SRR17520069.593022
SRR17520069.593024
SRR17520069.593026
SRR17520069.593028
SRR17520069.593030
SRR17520069.593034
SRR17520069.593036
SRR17520069.593038
SRR17520069.593040
SRR17520069.593042
SRR17520069.593044
SRR17520069.593046
SRR17520069.593048
SRR17520069.593052
SRR17520069.593056
SRR17520069.593058

SRR17520069.593060
SRR17520069.593062
SRR17520069.593064
SRR17520069.593066
SRR17520069.593068
SRR17520069.593070
SRR17520069.593072
SRR17520069.593074
SRR17520069.593076
SRR17520069.594764
SRR17520069.594766
SRR17520069.594768
SRR17520069.594770
SRR17520069.594772
SRR17520069.594774
SRR17520069.594776
SRR17520069.594778
SRR17520069.594780
SRR17520069.594782
SRR17520069.594784
SRR17520069.594786
SRR17520069.594790
SRR17520069.594792
SRR17520069.594794
SRR17520069.594796
SRR17520069.594798
SRR17520069.594802
SRR17520069.594806
SRR17520069.594810
SRR17520069.594812
SRR17520069.594814
SRR17520069.594816
SRR17520069.607526
SRR17520069.607530
SRR17520069.607532
SRR17520069.607534
SRR17520069.607536
SRR17520069.607538
SRR17520069.607540
SRR17520069.610031
SRR17520069.610033
SRR17520069.610035
SRR17520069.610037
SRR17520069.610039
SRR17520069.610041
SRR17520069.610043
SRR17520069.610045

SRR17520069.610047
SRR17520069.610049
SRR17520069.610051
SRR17520069.610053
SRR17520069.610055
SRR17520069.610057
SRR17520069.610059
SRR17520069.610061
SRR17520069.610063
SRR17520069.610065
SRR17520069.610067
SRR17520069.610069
SRR17520069.610071
SRR17520069.610073
SRR17520069.610075
SRR17520069.610077
SRR17520069.610079
SRR17520069.610081
SRR17520069.610083
SRR17520069.610085
SRR17520069.610087
SRR17520069.610089
SRR17520069.610091
SRR17520069.610093
SRR17520069.610095
SRR17520069.610097
SRR17520069.610099
SRR17520069.610101
SRR17520069.610103
SRR17520069.610105
SRR17520069.610107
SRR17520069.610109
SRR17520069.610111
SRR17520069.610113
SRR17520069.610115
SRR17520069.610117
SRR17520069.610119
SRR17520069.610121
SRR17520069.610123
SRR17520069.610125
SRR17520069.610127
SRR17520069.610129
SRR17520069.610131
SRR17520069.610133
SRR17520069.610135
SRR17520069.610137
SRR17520069.610139

SRR17520069.610141
SRR17520069.610143
SRR17520069.610145
SRR17520069.610147
SRR17520069.610149
SRR17520069.610151
SRR17520069.610153
SRR17520069.610155
SRR17520069.610157
SRR17520069.610159
SRR17520069.610161
SRR17520069.610163
SRR17520069.610167
SRR17520069.610169
SRR17520069.610171
SRR17520069.610173
SRR17520069.615344
SRR17520069.615346
SRR17520069.615348
SRR17520069.615350
SRR17520069.615352
SRR17520069.615354
SRR17520069.615356
SRR17520069.615358
SRR17520069.615360
SRR17520069.615362
SRR17520069.615364
SRR17520069.615366
SRR17520069.615368
SRR17520069.615370
SRR17520069.615372
SRR17520069.615374
SRR17520069.615376
SRR17520069.615378
SRR17520069.623168
SRR17520069.624525
SRR17520069.624527
SRR17520069.624529
SRR17520069.624531
SRR17520069.624533
SRR17520069.624535
SRR17520069.624537
SRR17520069.624539
SRR17520069.624541
SRR17520069.624543
SRR17520069.624545
SRR17520069.624547

SRR17520069.624549
SRR17520069.624551
SRR17520069.624553
SRR17520069.624555
SRR17520069.624557
SRR17520069.624559
SRR17520069.624561
SRR17520069.624563
SRR17520069.624565
SRR17520069.624567
SRR17520069.624569
SRR17520069.624571
SRR17520069.624573
SRR17520069.624577
SRR17520069.624579
SRR17520069.624581
SRR17520069.624583
SRR17520069.624585
SRR17520069.624587
SRR17520069.624589
SRR17520069.624591
SRR17520069.624593
SRR17520069.624595
SRR17520069.624597
SRR17520069.624599
SRR17520069.624601
SRR17520069.624603
SRR17520069.624605
SRR17520069.624607
SRR17520069.624609
SRR17520069.624611
SRR17520069.624613
SRR17520069.624615
SRR17520069.624619
SRR17520069.624621
SRR17520069.624623
SRR17520069.624625
SRR17520069.624627
SRR17520069.624629
SRR17520069.624631
SRR17520069.624633
SRR17520069.624635
SRR17520069.624637
SRR17520069.624639
SRR17520069.624641
SRR17520069.624643
SRR17520069.624645

SRR17520069.624647
SRR17520069.624649
SRR17520069.624651
SRR17520069.624653
SRR17520069.624655
SRR17520069.624657
SRR17520069.624659
SRR17520069.624661
SRR17520069.624663
SRR17520069.624665
SRR17520069.624667
SRR17520069.624669
SRR17520069.624671
SRR17520069.624673
SRR17520069.624675
SRR17520069.624677
SRR17520069.624679
SRR17520069.624681
SRR17520069.624683
SRR17520069.624685
SRR17520069.624687
SRR17520069.624689
SRR17520069.624691
SRR17520069.624693
SRR17520069.624695
SRR17520069.630624
SRR17520069.630628
SRR17520069.630630
SRR17520069.630634
SRR17520069.630636
SRR17520069.630642
SRR17520069.630644
SRR17520069.630646
SRR17520069.630650
SRR17520069.630652
SRR17520069.630654
SRR17520069.630656
SRR17520069.630658
SRR17520069.630660
SRR17520069.630664
SRR17520069.630666
SRR17520069.630668
SRR17520069.630670
SRR17520069.630688
SRR17520069.630690
SRR17520069.630692
SRR17520069.630698

SRR17520069.630700
SRR17520069.630702
SRR17520069.630704
SRR17520069.630708
SRR17520069.630710
SRR17520069.630712
SRR17520069.630714
SRR17520069.630716
SRR17520069.630718
SRR17520069.630720
SRR17520069.630722
SRR17520069.630724
SRR17520069.630726
SRR17520069.630728
SRR17520069.630730
SRR17520069.630732
SRR17520069.630734
SRR17520069.630736
SRR17520069.630738
SRR17520069.630740
SRR17520069.630742
SRR17520069.630744
SRR17520069.630746
SRR17520069.630748
SRR17520069.630750
SRR17520069.630752
SRR17520069.634857
SRR17520069.634859
SRR17520069.634861
SRR17520069.634863
SRR17520069.634865
SRR17520069.634867
SRR17520069.634869
SRR17520069.634871
SRR17520069.634873
SRR17520069.634875
SRR17520069.634877
SRR17520069.634879
SRR17520069.634881
SRR17520069.634883
SRR17520069.634885
SRR17520069.634887
SRR17520069.634889
SRR17520069.634891
SRR17520069.634893
SRR17520069.634895
SRR17520069.634897

SRR17520069.634899
SRR17520069.634901
SRR17520069.634903
SRR17520069.634905
SRR17520069.634907
SRR17520069.634909
SRR17520069.634911
SRR17520069.634913
SRR17520069.634915
SRR17520069.634917
SRR17520069.634919
SRR17520069.634921
SRR17520069.635742
SRR17520069.635744
SRR17520069.635746
SRR17520069.635748
SRR17520069.635750
SRR17520069.635752
SRR17520069.635754
SRR17520069.635756
SRR17520069.635758
SRR17520069.635760
SRR17520069.635762
SRR17520069.635764
SRR17520069.635766
SRR17520069.635768
SRR17520069.635770
SRR17520069.635772
SRR17520069.635774
SRR17520069.635776
SRR17520069.635778
SRR17520069.635780
SRR17520069.635782
SRR17520069.635784
SRR17520069.635786
SRR17520069.635790
SRR17520069.635792
SRR17520069.635794
SRR17520069.635796
SRR17520069.635798
SRR17520069.635800
SRR17520069.640429
SRR17520069.640431
SRR17520069.640433
SRR17520069.640435
SRR17520069.640437
SRR17520069.640439

SRR17520069.640441
SRR17520069.640443
SRR17520069.641408
SRR17520069.641410
SRR17520069.641412
SRR17520069.641414
SRR17520069.641416
SRR17520069.641418
SRR17520069.641420
SRR17520069.641422
SRR17520069.641424
SRR17520069.641426
SRR17520069.641428
SRR17520069.641430
SRR17520069.641432
SRR17520069.641434
SRR17520069.641436
SRR17520069.641438
SRR17520069.641440
SRR17520069.641442
SRR17520069.641444
SRR17520069.641446
SRR17520069.641448
SRR17520069.641450
SRR17520069.641452
SRR17520069.641454
SRR17520069.641456
SRR17520069.641460
SRR17520069.641462
SRR17520069.641464
SRR17520069.641466
SRR17520069.641468
SRR17520069.641470
SRR17520069.641472
SRR17520069.641474
SRR17520069.641476
SRR17520069.641478
SRR17520069.641480
SRR17520069.641482
SRR17520069.641484
SRR17520069.641486
SRR17520069.641488
SRR17520069.641490
SRR17520069.641492
SRR17520069.641494
SRR17520069.641496
SRR17520069.641498

SRR17520069.641500
SRR17520069.641502
SRR17520069.641504
SRR17520069.641506
SRR17520069.641508
SRR17520069.641510
SRR17520069.641512
SRR17520069.641514
SRR17520069.644562
SRR17520069.644564
SRR17520069.644566
SRR17520069.644568
SRR17520069.644570
SRR17520069.644572
SRR17520069.644574
SRR17520069.644576
SRR17520069.644578
SRR17520069.644580
SRR17520069.654115
SRR17520069.654117
SRR17520069.654119
SRR17520069.654121
SRR17520069.654123
SRR17520069.654125
SRR17520069.654127
SRR17520069.654129
SRR17520069.654131
SRR17520069.654133
SRR17520069.654135
SRR17520069.654137
SRR17520069.654139
SRR17520069.654141
SRR17520069.654143
SRR17520069.654145
SRR17520069.654147
SRR17520069.654149
SRR17520069.654151
SRR17520069.654153
SRR17520069.654155
SRR17520069.654157
SRR17520069.654159
SRR17520069.654161
SRR17520069.654163
SRR17520069.654165
SRR17520069.654167
SRR17520069.654169
SRR17520069.654171

SRR17520069.654173
SRR17520069.654175
SRR17520069.654177
SRR17520069.654179
SRR17520069.654181
SRR17520069.654183
SRR17520069.654185
SRR17520069.654187
SRR17520069.654189
SRR17520069.654191
SRR17520069.654193
SRR17520069.654195
SRR17520069.654197
SRR17520069.654199
SRR17520069.654201
SRR17520069.654203
SRR17520069.654205
SRR17520069.654207
SRR17520069.654209
SRR17520069.654211
SRR17520069.654213
SRR17520069.654215
SRR17520069.655775
SRR17520069.662433
SRR17520069.663312
SRR17520069.663314
SRR17520069.663316
SRR17520069.663318
SRR17520069.663320
SRR17520069.663322
SRR17520069.663324
SRR17520069.663326
SRR17520069.663328
SRR17520069.663330
SRR17520069.663332
SRR17520069.663334
SRR17520069.663336
SRR17520069.663338
SRR17520069.663340
SRR17520069.663342
SRR17520069.663344
SRR17520069.663346
SRR17520069.663348
SRR17520069.663350
SRR17520069.663352
SRR17520069.663354
SRR17520069.663356

SRR17520069.663358
SRR17520069.663360
SRR17520069.663362
SRR17520069.663364
SRR17520069.663366
SRR17520069.663368
SRR17520069.663370
SRR17520069.663372
SRR17520069.663374
SRR17520069.663376
SRR17520069.663378
SRR17520069.663380
SRR17520069.663382
SRR17520069.663384
SRR17520069.663386
SRR17520069.663388
SRR17520069.663390
SRR17520069.663392
SRR17520069.663394
SRR17520069.663396
SRR17520069.663398
SRR17520069.663400
SRR17520069.663402
SRR17520069.663404
SRR17520069.663406
SRR17520069.663408
SRR17520069.663410
SRR17520069.663412
SRR17520069.663414
SRR17520069.663416
SRR17520069.663418
SRR17520069.663420
SRR17520069.663422
SRR17520069.663424
SRR17520069.663426
SRR17520069.663428
SRR17520069.663430
SRR17520069.663432
SRR17520069.663434
SRR17520069.663436
SRR17520069.663438
SRR17520069.663440
SRR17520069.663442
SRR17520069.663444
SRR17520069.663446
SRR17520069.663448
SRR17520069.663450

SRR17520069.663452
SRR17520069.663454
SRR17520069.663456
SRR17520069.663458
SRR17520069.663460
SRR17520069.663462
SRR17520069.663464
SRR17520069.663466
SRR17520069.663468
SRR17520069.663470
SRR17520069.664522
SRR17520069.664524
SRR17520069.664526
SRR17520069.664528
SRR17520069.664530
SRR17520069.664532
SRR17520069.664534
SRR17520069.664536
SRR17520069.664538
SRR17520069.664540
SRR17520069.664542
SRR17520069.664544
SRR17520069.664546
SRR17520069.664548
SRR17520069.664550
SRR17520069.664552
SRR17520069.664554
SRR17520069.664556
SRR17520069.664558
SRR17520069.664560
SRR17520069.664562
SRR17520069.664564
SRR17520069.664566
SRR17520069.664568
SRR17520069.664570
SRR17520069.664572
SRR17520069.664574
SRR17520069.664576
SRR17520069.664578
SRR17520069.664580
SRR17520069.664582
SRR17520069.664584
SRR17520069.664586
SRR17520069.664588
SRR17520069.664592
SRR17520069.664594
SRR17520069.664596

SRR17520069.664598
SRR17520069.664600
SRR17520069.664602
SRR17520069.664606
SRR17520069.664608
SRR17520069.666790
SRR17520069.672447
SRR17520069.672449
SRR17520069.679661
SRR17520069.679663
SRR17520069.680886
SRR17520069.680888
SRR17520069.680890
SRR17520069.680892
SRR17520069.680894
SRR17520069.680896
SRR17520069.680898
SRR17520069.680900
SRR17520069.680902
SRR17520069.680904
SRR17520069.680906
SRR17520069.680908
SRR17520069.680910
SRR17520069.680912
SRR17520069.680914
SRR17520069.680916
SRR17520069.680918
SRR17520069.680920
SRR17520069.680922
SRR17520069.680924
SRR17520069.680926
SRR17520069.686055
SRR17520069.686057
SRR17520069.686061
SRR17520069.686063
SRR17520069.686067
SRR17520069.686069
SRR17520069.686071
SRR17520069.686073
SRR17520069.686075
SRR17520069.686077
SRR17520069.686079
SRR17520069.686081
SRR17520069.690280
SRR17520069.690282
SRR17520069.695248
SRR17520069.695250

SRR17520069.695252
SRR17520069.695254
SRR17520069.695256
SRR17520069.695258
SRR17520069.695260
SRR17520069.695262
SRR17520069.695264
SRR17520069.695266
SRR17520069.695268
SRR17520069.695270
SRR17520069.695272
SRR17520069.695274
SRR17520069.695278
SRR17520069.695280
SRR17520069.695282
SRR17520069.695284
SRR17520069.695286
SRR17520069.695288
SRR17520069.695290
SRR17520069.695292
SRR17520069.695294
SRR17520069.695296
SRR17520069.695298
SRR17520069.695300
SRR17520069.695302
SRR17520069.695306
SRR17520069.695308
SRR17520069.695310
SRR17520069.695312
SRR17520069.695314
SRR17520069.695318
SRR17520069.695320
SRR17520069.695322
SRR17520069.695324
SRR17520069.696129
SRR17520069.696131
SRR17520069.696133
SRR17520069.696135
SRR17520069.696137
SRR17520069.696139
SRR17520069.696143
SRR17520069.696145
SRR17520069.696147
SRR17520069.696149
SRR17520069.696153
SRR17520069.696155
SRR17520069.696157

SRR17520069.696159
SRR17520069.696161
SRR17520069.696163
SRR17520069.696167
SRR17520069.700255
SRR17520069.701316
SRR17520069.701318
SRR17520069.701320
SRR17520069.701322
SRR17520069.701324
SRR17520069.701326
SRR17520069.701328
SRR17520069.701330
SRR17520069.701332
SRR17520069.702943
SRR17520069.702945
SRR17520069.708907
SRR17520069.708909
SRR17520069.708911
SRR17520069.708913
SRR17520069.708915
SRR17520069.708917
SRR17520069.708919
SRR17520069.711086
SRR17520069.711088
SRR17520069.711090
SRR17520069.711092
SRR17520069.711094
SRR17520069.711096
SRR17520069.715828
SRR17520069.715830
SRR17520069.715832
SRR17520069.715834
SRR17520069.715836
SRR17520069.715840
SRR17520069.715844
SRR17520069.715848
SRR17520069.715850
SRR17520069.715854
SRR17520069.715856
SRR17520069.715858
SRR17520069.715860
SRR17520069.715862
SRR17520069.715864
SRR17520069.715866
SRR17520069.715868
SRR17520069.715870

SRR17520069.715872
SRR17520069.715874
SRR17520069.715876
SRR17520069.715878
SRR17520069.715880
SRR17520069.715882
SRR17520069.715884
SRR17520069.715886
SRR17520069.715888
SRR17520069.715890
SRR17520069.715892
SRR17520069.715894
SRR17520069.715896
SRR17520069.715898
SRR17520069.717912
SRR17520069.721061
SRR17520069.721063
SRR17520069.721065
SRR17520069.721067
SRR17520069.721069
SRR17520069.721071
SRR17520069.721073
SRR17520069.721075
SRR17520069.721077
SRR17520069.721079
SRR17520069.721081
SRR17520069.721083
SRR17520069.725657
SRR17520069.725659
SRR17520069.725661
SRR17520069.725663
SRR17520069.725665
SRR17520069.725667
SRR17520069.725669
SRR17520069.732263
SRR17520069.732265
SRR17520069.732267
SRR17520069.732269
SRR17520069.732271
SRR17520069.732273
SRR17520069.732275
SRR17520069.732277
SRR17520069.732281
SRR17520069.732283
SRR17520069.732287
SRR17520069.732289
SRR17520069.732293

SRR17520069.732295
SRR17520069.732297
SRR17520069.732299
SRR17520069.732303
SRR17520069.732305
SRR17520069.732307
SRR17520069.732309
SRR17520069.732311
SRR17520069.732315
SRR17520069.732317
SRR17520069.733076
SRR17520069.733447
SRR17520069.733449
SRR17520069.733453
SRR17520069.733455
SRR17520069.733457
SRR17520069.733459
SRR17520069.733461
SRR17520069.733463
SRR17520069.733465
SRR17520069.733467
SRR17520069.733469
SRR17520069.733471
SRR17520069.733473
SRR17520069.733475
SRR17520069.733477
SRR17520069.733479
SRR17520069.733481
SRR17520069.733483
SRR17520069.733485
SRR17520069.733487
SRR17520069.733489
SRR17520069.733491
SRR17520069.733493
SRR17520069.733495
SRR17520069.733497
SRR17520069.737565
SRR17520069.739319
SRR17520069.739367
SRR17520069.739369
SRR17520069.739371
SRR17520069.739373
SRR17520069.739375
SRR17520069.739377
SRR17520069.739379
SRR17520069.739381
SRR17520069.739383

SRR17520069.739385
SRR17520069.739387
SRR17520069.739389
SRR17520069.739391
SRR17520069.739393
SRR17520069.739395
SRR17520069.739397
SRR17520069.739399
SRR17520069.739401
SRR17520069.739403
SRR17520069.739405
SRR17520069.739407
SRR17520069.739409
SRR17520069.739411
SRR17520069.739413
SRR17520069.739415
SRR17520069.739417
SRR17520069.739419
SRR17520069.739421
SRR17520069.739423
SRR17520069.739425
SRR17520069.739427
SRR17520069.739429
SRR17520069.750359
SRR17520069.751543
SRR17520069.751545
SRR17520069.751549
SRR17520069.751551
SRR17520069.751553
SRR17520069.751555
SRR17520069.751557
SRR17520069.751559
SRR17520069.751561
SRR17520069.751563
SRR17520069.751565
SRR17520069.751567
SRR17520069.751569
SRR17520069.751571
SRR17520069.751573
SRR17520069.751575
SRR17520069.751577
SRR17520069.751579
SRR17520069.751581
SRR17520069.751583
SRR17520069.751587
SRR17520069.751589
SRR17520069.751591

SRR17520069.751593
SRR17520069.751595
SRR17520069.751597
SRR17520069.751599
SRR17520069.751601
SRR17520069.751603
SRR17520069.751605
SRR17520069.751607
SRR17520069.751609
SRR17520069.751611
SRR17520069.751615
SRR17520069.751617
SRR17520069.752015
SRR17520069.752017
SRR17520069.752019
SRR17520069.752021
SRR17520069.752023
SRR17520069.752025
SRR17520069.752027
SRR17520069.752029
SRR17520069.752031
SRR17520069.752033
SRR17520069.752035
SRR17520069.752039
SRR17520069.752041
SRR17520069.752043
SRR17520069.752364
SRR17520069.752366
SRR17520069.752368
SRR17520069.753438
SRR17520069.753440
SRR17520069.753442
SRR17520069.753444
SRR17520069.753446
SRR17520069.753448
SRR17520069.753455
SRR17520069.753459
SRR17520069.755229
SRR17520069.755231
SRR17520069.755233
SRR17520069.755235
SRR17520069.755237
SRR17520069.755239
SRR17520069.755241
SRR17520069.755243
SRR17520069.755245
SRR17520069.755247

SRR17520069.755249
SRR17520069.755251
SRR17520069.755253
SRR17520069.755255
SRR17520069.755257
SRR17520069.755259
SRR17520069.755261
SRR17520069.755263
SRR17520069.755265
SRR17520069.755267
SRR17520069.755269
SRR17520069.755271
SRR17520069.755273
SRR17520069.755275
SRR17520069.755277
SRR17520069.755281
SRR17520069.755283
SRR17520069.755285
SRR17520069.755287
SRR17520069.755289
SRR17520069.755291
SRR17520069.755293
SRR17520069.755295
SRR17520069.755297
SRR17520069.755299
SRR17520069.755301
SRR17520069.764125
SRR17520069.764127
SRR17520069.764129
SRR17520069.764131
SRR17520069.764133
SRR17520069.764135
SRR17520069.764137
SRR17520069.764139
SRR17520069.764141
SRR17520069.764145
SRR17520069.764147
SRR17520069.764149
SRR17520069.764151
SRR17520069.764155
SRR17520069.764157
SRR17520069.764159
SRR17520069.764161
SRR17520069.764165
SRR17520069.764167
SRR17520069.764169
SRR17520069.764171

SRR17520069.764175
SRR17520069.764177
SRR17520069.764179
SRR17520069.764181
SRR17520069.764183
SRR17520069.764185
SRR17520069.764187
SRR17520069.764189
SRR17520069.766996
SRR17520069.766998
SRR17520069.767000
SRR17520069.767002
SRR17520069.767004
SRR17520069.767006
SRR17520069.767008
SRR17520069.767010
SRR17520069.767012
SRR17520069.767014
SRR17520069.769572
SRR17520069.769574
SRR17520069.769576
SRR17520069.769582
SRR17520069.769584
SRR17520069.769586
SRR17520069.769588
SRR17520069.769590
SRR17520069.769592
SRR17520069.769594
SRR17520069.769596
SRR17520069.769598
SRR17520069.769600
SRR17520069.771138
SRR17520069.771140
SRR17520069.771142
SRR17520069.771144
SRR17520069.771146
SRR17520069.771148
SRR17520069.771150
SRR17520069.771152
SRR17520069.771154
SRR17520069.771156
SRR17520069.771158
SRR17520069.771160
SRR17520069.771162
SRR17520069.771164
SRR17520069.771168
SRR17520069.771170

SRR17520069.771172
SRR17520069.771174
SRR17520069.771176
SRR17520069.771178
SRR17520069.772336
SRR17520069.773982
SRR17520069.773984
SRR17520069.773986
SRR17520069.773988
SRR17520069.773990
SRR17520069.773994
SRR17520069.773998
SRR17520069.774000
SRR17520069.774002
SRR17520069.774006
SRR17520069.774008
SRR17520069.774010
SRR17520069.774012
SRR17520069.774014
SRR17520069.774016
SRR17520069.774018
SRR17520069.774020
SRR17520069.774022
SRR17520069.774024
SRR17520069.774026
SRR17520069.774028
SRR17520069.774032
SRR17520069.774034
SRR17520069.774036
SRR17520069.774038
SRR17520069.774040
SRR17520069.774042
SRR17520069.774044
SRR17520069.774046
SRR17520069.777881
SRR17520069.777883
SRR17520069.777885
SRR17520069.777887
SRR17520069.777889
SRR17520069.777891
SRR17520069.777893
SRR17520069.777895
SRR17520069.777897
SRR17520069.777899
SRR17520069.777901
SRR17520069.777903
SRR17520069.777905

SRR17520069.777907
SRR17520069.777909
SRR17520069.777911
SRR17520069.777913
SRR17520069.777915
SRR17520069.785443
SRR17520069.785445
SRR17520069.785448
SRR17520069.785450
SRR17520069.785452
SRR17520069.785454
SRR17520069.785456
SRR17520069.785458
SRR17520069.785460
SRR17520069.789384
SRR17520069.789386
SRR17520069.789388
SRR17520069.789390
SRR17520069.789392
SRR17520069.789394
SRR17520069.789396
SRR17520069.789398
SRR17520069.789400
SRR17520069.789402
SRR17520069.789404
SRR17520069.789406
SRR17520069.789408
SRR17520069.789410
SRR17520069.789412
SRR17520069.789414
SRR17520069.789416
SRR17520069.789418
SRR17520069.789420
SRR17520069.791378
SRR17520069.791380
SRR17520069.791382
SRR17520069.791384
SRR17520069.791386
SRR17520069.791388
SRR17520069.791390
SRR17520069.791394
SRR17520069.791396
SRR17520069.791398
SRR17520069.791400
SRR17520069.791402
SRR17520069.791404
SRR17520069.791406

SRR17520069.791408
SRR17520069.791410
SRR17520069.791412
SRR17520069.791414
SRR17520069.791416
SRR17520069.793883
SRR17520069.793885
SRR17520069.793887
SRR17520069.793889
SRR17520069.793891
SRR17520069.793893
SRR17520069.793895
SRR17520069.793897
SRR17520069.793899
SRR17520069.798129
SRR17520069.798131
SRR17520069.805850
SRR17520069.805852
SRR17520069.805854
SRR17520069.805856
SRR17520069.805858
SRR17520069.805860
SRR17520069.805862
SRR17520069.805864
SRR17520069.805866
SRR17520069.805868
SRR17520069.805872
SRR17520069.805876
SRR17520069.805878
SRR17520069.805880
SRR17520069.805882
SRR17520069.805884
SRR17520069.805886
SRR17520069.805888
SRR17520069.805890
SRR17520069.805892
SRR17520069.805894
SRR17520069.805896
SRR17520069.805898
SRR17520069.813863
SRR17520069.813865
SRR17520069.813867
SRR17520069.813869
SRR17520069.813871
SRR17520069.813873
SRR17520069.813875
SRR17520069.813879

SRR17520069.813881
SRR17520069.813883
SRR17520069.813885
SRR17520069.813887
SRR17520069.816150
SRR17520069.816152
SRR17520069.816154
SRR17520069.816156
SRR17520069.816158
SRR17520069.816160
SRR17520069.816162
SRR17520069.816164
SRR17520069.816168
SRR17520069.816170
SRR17520069.816172
SRR17520069.816178
SRR17520069.818303
SRR17520069.821112
SRR17520069.821114
SRR17520069.821116
SRR17520069.821118
SRR17520069.821120
SRR17520069.821122
SRR17520069.821124
SRR17520069.821126
SRR17520069.821128
SRR17520069.821130
SRR17520069.821132
SRR17520069.821134
SRR17520069.821136
SRR17520069.821138
SRR17520069.821140
SRR17520069.826693
SRR17520069.826695
SRR17520069.826697
SRR17520069.826699
SRR17520069.826701
SRR17520069.839507
SRR17520069.839509
SRR17520069.839511
SRR17520069.839513
SRR17520069.839515
SRR17520069.839517
SRR17520069.839519
SRR17520069.847126
SRR17520069.847130
SRR17520069.847132

SRR17520069.847134
SRR17520069.847136
SRR17520069.847138
SRR17520069.847140
SRR17520069.847142
SRR17520069.847144
SRR17520069.847146
SRR17520069.847148
SRR17520069.847150
SRR17520069.847152
SRR17520069.847154
SRR17520069.847156
SRR17520069.847158
SRR17520069.847160
SRR17520069.847162
SRR17520069.847164
SRR17520069.847166
SRR17520069.847168
SRR17520069.847170
SRR17520069.847172
SRR17520069.847176
SRR17520069.847178
SRR17520069.847180
SRR17520069.847182
SRR17520069.847184
SRR17520069.847186
SRR17520069.847188
SRR17520069.847190
SRR17520069.849597
SRR17520069.849599
SRR17520069.849601
SRR17520069.855369
SRR17520069.855371
SRR17520069.855373
SRR17520069.855375
SRR17520069.855377
SRR17520069.855379
SRR17520069.855381
SRR17520069.855383
SRR17520069.855385
SRR17520069.855387
SRR17520069.855389
SRR17520069.855391
SRR17520069.855393
SRR17520069.855395
SRR17520069.855397
SRR17520069.855399

SRR17520069.855401
SRR17520069.855403
SRR17520069.855405
SRR17520069.855407
SRR17520069.855409
SRR17520069.855411
SRR17520069.855413
SRR17520069.855415
SRR17520069.855417
SRR17520069.855419
SRR17520069.855421
SRR17520069.855423
SRR17520069.855425
SRR17520069.855427
SRR17520069.855429
SRR17520069.855431
SRR17520069.855433
SRR17520069.855435
SRR17520069.855437
SRR17520069.855439
SRR17520069.855441
SRR17520069.855443
SRR17520069.863491
SRR17520069.863493
SRR17520069.863495
SRR17520069.863497
SRR17520069.863499
SRR17520069.863501
SRR17520069.863503
SRR17520069.863505
SRR17520069.863507
SRR17520069.863509
SRR17520069.863511
SRR17520069.863513
SRR17520069.863515
SRR17520069.863517
SRR17520069.863519
SRR17520069.863521
SRR17520069.863523
SRR17520069.863525
SRR17520069.865665
SRR17520069.865667
SRR17520069.865669
SRR17520069.865671
SRR17520069.865673
SRR17520069.865675
SRR17520069.865677

SRR17520069.865679
SRR17520069.865681
SRR17520069.865683
SRR17520069.865685
SRR17520069.865687
SRR17520069.865689
SRR17520069.865691
SRR17520069.865693
SRR17520069.865695
SRR17520069.865697
SRR17520069.865699
SRR17520069.865703
SRR17520069.865705
SRR17520069.865707
SRR17520069.865709
SRR17520069.865711
SRR17520069.865713
SRR17520069.865715
SRR17520069.865717
SRR17520069.865719
SRR17520069.865721
SRR17520069.865723
SRR17520069.865725
SRR17520069.865727
SRR17520069.865729
SRR17520069.865731
SRR17520069.865733
SRR17520069.865735
SRR17520069.865737
SRR17520069.865739
SRR17520069.865741
SRR17520069.865743
SRR17520069.865745
SRR17520069.865747
SRR17520069.865749
SRR17520069.865751
SRR17520069.865753
SRR17520069.865755
SRR17520069.865757
SRR17520069.865759
SRR17520069.865761
SRR17520069.865763
SRR17520069.865765
SRR17520069.865767
SRR17520069.865769
SRR17520069.865771
SRR17520069.865773

SRR17520069.865775
SRR17520069.865777
SRR17520069.865779
SRR17520069.865781
SRR17520069.865783
SRR17520069.865785
SRR17520069.865787
SRR17520069.867375
SRR17520069.867441
SRR17520069.867443
SRR17520069.867445
SRR17520069.868444
SRR17520069.868446
SRR17520069.868448
SRR17520069.868450
SRR17520069.868452
SRR17520069.868456
SRR17520069.868712
SRR17520069.868714
SRR17520069.868716
SRR17520069.868718
SRR17520069.868722
SRR17520069.868724
SRR17520069.868726
SRR17520069.868728
SRR17520069.868730
SRR17520069.868732
SRR17520069.868734
SRR17520069.868736
SRR17520069.868738
SRR17520069.868742
SRR17520069.868744
SRR17520069.868746
SRR17520069.868748
SRR17520069.871634
SRR17520069.871636
SRR17520069.871638
SRR17520069.871640
SRR17520069.871642
SRR17520069.871644
SRR17520069.871646
SRR17520069.871648
SRR17520069.871650
SRR17520069.871652
SRR17520069.871654
SRR17520069.871656
SRR17520069.873010

SRR17520069.873012
SRR17520069.873014
SRR17520069.873016
SRR17520069.873018
SRR17520069.873020
SRR17520069.873022
SRR17520069.873024
SRR17520069.873712
SRR17520069.873716
SRR17520069.876985
SRR17520069.876987
SRR17520069.876989
SRR17520069.876991
SRR17520069.876993
SRR17520069.876995
SRR17520069.876997
SRR17520069.876999
SRR17520069.877001
SRR17520069.877003
SRR17520069.877005
SRR17520069.877007
SRR17520069.877009
SRR17520069.877011
SRR17520069.877013
SRR17520069.877015
SRR17520069.877017
SRR17520069.877019
SRR17520069.877021
SRR17520069.877023
SRR17520069.877025
SRR17520069.877027
SRR17520069.877029
SRR17520069.877031
SRR17520069.877033
SRR17520069.877035
SRR17520069.877037
SRR17520069.877039
SRR17520069.877041
SRR17520069.877043
SRR17520069.877045
SRR17520069.877047
SRR17520069.877049
SRR17520069.877051
SRR17520069.877053
SRR17520069.877055
SRR17520069.877057
SRR17520069.877059

SRR17520069.877061
SRR17520069.877450
SRR17520069.877452
SRR17520069.877454
SRR17520069.877456
SRR17520069.877458
SRR17520069.877460
SRR17520069.882280
SRR17520069.882282
SRR17520069.882284
SRR17520069.882286
SRR17520069.882288
SRR17520069.882290
SRR17520069.882292
SRR17520069.882296
SRR17520069.882298
SRR17520069.882509
SRR17520069.882511
SRR17520069.882515
SRR17520069.882517
SRR17520069.882519
SRR17520069.882523
SRR17520069.882525
SRR17520069.882529
SRR17520069.882531
SRR17520069.882533
SRR17520069.882535
SRR17520069.882537
SRR17520069.882539
SRR17520069.882541
SRR17520069.882543
SRR17520069.882545
SRR17520069.882547
SRR17520069.882549
SRR17520069.882551
SRR17520069.882553
SRR17520069.882555
SRR17520069.882557
SRR17520069.882559
SRR17520069.882561
SRR17520069.882563
SRR17520069.882565
SRR17520069.882567
SRR17520069.882569
SRR17520069.882571
SRR17520069.882573
SRR17520069.882575

SRR17520069.882577
SRR17520069.882579
SRR17520069.882581
SRR17520069.882583
SRR17520069.882585
SRR17520069.882587
SRR17520069.882593
SRR17520069.882595
SRR17520069.882597
SRR17520069.882599
SRR17520069.882601
SRR17520069.882603
SRR17520069.882605
SRR17520069.882609
SRR17520069.882611
SRR17520069.882613
SRR17520069.883125
SRR17520069.883127
SRR17520069.883129
SRR17520069.883131
SRR17520069.885698
SRR17520069.885700
SRR17520069.885702
SRR17520069.885704
SRR17520069.885706
SRR17520069.885708
SRR17520069.885710
SRR17520069.885712
SRR17520069.885714
SRR17520069.885718
SRR17520069.885720
SRR17520069.885722
SRR17520069.885724
SRR17520069.885823
SRR17520069.885827
SRR17520069.885831
SRR17520069.885833
SRR17520069.885837
SRR17520069.885839
SRR17520069.885841
SRR17520069.885843
SRR17520069.885845
SRR17520069.885847
SRR17520069.885853
SRR17520069.885855
SRR17520069.885857
SRR17520069.885859

SRR17520069.885861
SRR17520069.885863
SRR17520069.885865
SRR17520069.885867
SRR17520069.885869
SRR17520069.885873
SRR17520069.885875
SRR17520069.885877
SRR17520069.885879
SRR17520069.885881
SRR17520069.885883
SRR17520069.885885
SRR17520069.885887
SRR17520069.885889
SRR17520069.885891
SRR17520069.885893
SRR17520069.887589
SRR17520069.887591
SRR17520069.887593
SRR17520069.887595
SRR17520069.887597
SRR17520069.887599
SRR17520069.887601
SRR17520069.887603
SRR17520069.887605
SRR17520069.887607
SRR17520069.887609
SRR17520069.887611
SRR17520069.887613
SRR17520069.887615
SRR17520069.887617
SRR17520069.887619
SRR17520069.887621
SRR17520069.887623
SRR17520069.887625
SRR17520069.887627
SRR17520069.887629
SRR17520069.887631
SRR17520069.887633
SRR17520069.887635
SRR17520069.888405
SRR17520069.888407
SRR17520069.888409
SRR17520069.888411
SRR17520069.888413
SRR17520069.896945
SRR17520069.896947

SRR17520069.896949
SRR17520069.896951
SRR17520069.896953
SRR17520069.896955
SRR17520069.896957
SRR17520069.896959
SRR17520069.896961
SRR17520069.896963
SRR17520069.896965
SRR17520069.896967
SRR17520069.896971
SRR17520069.896998
SRR17520069.897000
SRR17520069.897002
SRR17520069.909562
SRR17520069.909564
SRR17520069.909566
SRR17520069.909568
SRR17520069.909570
SRR17520069.909572
SRR17520069.909574
SRR17520069.909576
SRR17520069.909578
SRR17520069.909580
SRR17520069.909582
SRR17520069.909584
SRR17520069.909586
SRR17520069.909588
SRR17520069.909590
SRR17520069.909592
SRR17520069.911365
SRR17520069.911369
SRR17520069.912189
SRR17520069.912191
SRR17520069.912193
SRR17520069.912195
SRR17520069.912197
SRR17520069.912199
SRR17520069.912201
SRR17520069.912203
SRR17520069.913349
SRR17520069.913353
SRR17520069.913355
SRR17520069.913357
SRR17520069.929989
SRR17520069.929991
SRR17520069.929993

SRR17520069.930350
SRR17520069.930352
SRR17520069.930354
SRR17520069.930356
SRR17520069.930358
SRR17520069.930360
SRR17520069.930362
SRR17520069.930364
SRR17520069.930366
SRR17520069.930368
SRR17520069.930370
SRR17520069.930372
SRR17520069.930374
SRR17520069.930376
SRR17520069.930378
SRR17520069.930380
SRR17520069.930382
SRR17520069.930384
SRR17520069.930386
SRR17520069.930388
SRR17520069.930390
SRR17520069.930392
SRR17520069.930394
SRR17520069.930396
SRR17520069.930398
SRR17520069.930400
SRR17520069.930402
SRR17520069.930404
SRR17520069.930406
SRR17520069.930408
SRR17520069.930410
SRR17520069.930412
SRR17520069.930414
SRR17520069.930416
SRR17520069.930418
SRR17520069.930420
SRR17520069.930422
SRR17520069.930424
SRR17520069.930426
SRR17520069.930428
SRR17520069.930430
SRR17520069.930432
SRR17520069.930434
SRR17520069.930436
SRR17520069.930438
SRR17520069.930440
SRR17520069.930442

SRR17520069.930444
SRR17520069.930446
SRR17520069.930448
SRR17520069.930768
SRR17520069.930770
SRR17520069.930774
SRR17520069.930778
SRR17520069.930780
SRR17520069.930782
SRR17520069.930784
SRR17520069.930788
SRR17520069.930790
SRR17520069.930792
SRR17520069.930794
SRR17520069.930796
SRR17520069.931753
SRR17520069.931755
SRR17520069.931759
SRR17520069.931761
SRR17520069.931763
SRR17520069.931765
SRR17520069.931767
SRR17520069.931769
SRR17520069.931771
SRR17520069.931773
SRR17520069.931775
SRR17520069.931777
SRR17520069.931779
SRR17520069.931781
SRR17520069.931783
SRR17520069.931785
SRR17520069.931787
SRR17520069.931789
SRR17520069.931791
SRR17520069.931793
SRR17520069.931795
SRR17520069.931797
SRR17520069.931799
SRR17520069.937418
SRR17520069.937420
SRR17520069.937422
SRR17520069.937424
SRR17520069.937426
SRR17520069.937428
SRR17520069.937430
SRR17520069.937637
SRR17520069.937639

SRR17520069.937646
SRR17520069.937648
SRR17520069.937650
SRR17520069.937652
SRR17520069.937654
SRR17520069.937656
SRR17520069.937658
SRR17520069.937660
SRR17520069.937662
SRR17520069.937664
SRR17520069.937666
SRR17520069.937668
SRR17520069.937670
SRR17520069.937672
SRR17520069.948055
SRR17520069.948057
SRR17520069.948059
SRR17520069.948061
SRR17520069.948067
SRR17520069.948069
SRR17520069.948071
SRR17520069.948073
SRR17520069.948075
SRR17520069.948077
SRR17520069.948079
SRR17520069.949887
SRR17520069.949910
SRR17520069.949913
SRR17520069.949941
SRR17520069.949949
SRR17520069.951318
SRR17520069.951320
SRR17520069.951322
SRR17520069.951324
SRR17520069.957735
SRR17520069.957737
SRR17520069.957739
SRR17520069.957743
SRR17520069.957745
SRR17520069.958195
SRR17520069.958197
SRR17520069.958199
SRR17520069.958201
SRR17520069.961035
SRR17520069.961037
SRR17520069.961039
SRR17520069.961041

SRR17520069.961043
SRR17520069.961045
SRR17520069.961047
SRR17520069.961049
SRR17520069.961051
SRR17520069.961053
SRR17520069.961055
SRR17520069.961057
SRR17520069.961059
SRR17520069.961061
SRR17520069.961063
SRR17520069.961065
SRR17520069.961067
SRR17520069.961069
SRR17520069.961071
SRR17520069.961073
SRR17520069.961075
SRR17520069.961077
SRR17520069.961079
SRR17520069.961081
SRR17520069.961083
SRR17520069.961087
SRR17520069.961089
SRR17520069.961091
SRR17520069.961093
SRR17520069.961095
SRR17520069.961097
SRR17520069.961099
SRR17520069.961101
SRR17520069.961103
SRR17520069.961105
SRR17520069.963856
SRR17520069.963858
SRR17520069.963860
SRR17520069.963862
SRR17520069.963864
SRR17520069.963868
SRR17520069.963870
SRR17520069.963872
SRR17520069.963874
SRR17520069.963876
SRR17520069.963878
SRR17520069.963880
SRR17520069.963882
SRR17520069.963884
SRR17520069.963886
SRR17520069.963888

SRR17520069.963890
SRR17520069.963892
SRR17520069.963894
SRR17520069.963896
SRR17520069.963898
SRR17520069.963900
SRR17520069.963902
SRR17520069.963904
SRR17520069.963906
SRR17520069.963908
SRR17520069.963910
SRR17520069.963912
SRR17520069.963914
SRR17520069.963916
SRR17520069.963918
SRR17520069.963920
SRR17520069.963922
SRR17520069.963924
SRR17520069.963926
SRR17520069.963928
SRR17520069.963930
SRR17520069.963932
SRR17520069.963934
SRR17520069.963936
SRR17520069.963938
SRR17520069.963940
SRR17520069.963942
SRR17520069.963944
SRR17520069.963946
SRR17520069.963948
SRR17520069.964021
SRR17520069.964023
SRR17520069.965212
SRR17520069.965214
SRR17520069.965216
SRR17520069.965218
SRR17520069.965220
SRR17520069.965222
SRR17520069.965224
SRR17520069.965226
SRR17520069.971868
SRR17520069.971870
SRR17520069.971872
SRR17520069.971874
SRR17520069.971876
SRR17520069.971878
SRR17520069.971880

SRR17520069.971882
SRR17520069.971884
SRR17520069.971888
SRR17520069.978162
SRR17520069.981100
SRR17520069.981102
SRR17520069.981104
SRR17520069.981106
SRR17520069.981108
SRR17520069.981110
SRR17520069.981114
SRR17520069.981116
SRR17520069.981118
SRR17520069.981120
SRR17520069.981122
SRR17520069.988106
SRR17520069.988108
SRR17520069.988110
SRR17520069.988112
SRR17520069.988114
SRR17520069.988116
SRR17520069.988118
SRR17520069.988120
SRR17520069.988126
SRR17520069.988128
SRR17520069.988130
SRR17520069.998916
SRR17520069.998918
SRR17520069.998920
SRR17520069.998922
SRR17520069.998924
SRR17520069.998926
SRR17520069.998928
SRR17520069.998930
SRR17520069.998932
SRR17520069.998934
SRR17520069.998936
SRR17520069.998938
SRR17520069.998940
SRR17520069.998942
SRR17520069.998944
SRR17520069.1000219
SRR17520069.1000221
SRR17520069.1000223
SRR17520069.1000227
SRR17520069.1000229
SRR17520069.1000231

SRR17520069.1000233
SRR17520069.1000235
SRR17520069.1000239
SRR17520069.1000241
SRR17520069.1000243
SRR17520069.1000245
SRR17520069.1000247
SRR17520069.1000249
SRR17520069.1000251
SRR17520069.1000253
SRR17520069.1000255
SRR17520069.1000257
SRR17520069.1000259
SRR17520069.1000261
SRR17520069.1000263
SRR17520069.1001883
SRR17520069.1001891
SRR17520069.1004748
SRR17520069.1004750
SRR17520069.1004752
SRR17520069.1004754
SRR17520069.1004756
SRR17520069.1004758
SRR17520069.1017669
SRR17520069.1022722
SRR17520069.1022724
SRR17520069.1022726
SRR17520069.1022728
SRR17520069.1022730
SRR17520069.1022732
SRR17520069.1022734
SRR17520069.1022736
SRR17520069.1022738
SRR17520069.1022740
SRR17520069.1022742
SRR17520069.1022744
SRR17520069.1022746
SRR17520069.1022748
SRR17520069.1022750
SRR17520069.1022752
SRR17520069.1022754
SRR17520069.1022756
SRR17520069.1022758
SRR17520069.1022760
SRR17520069.1022762
SRR17520069.1022764
SRR17520069.1022766

SRR17520069.1022768
SRR17520069.1022770
SRR17520069.1022772
SRR17520069.1022774
SRR17520069.1022776
SRR17520069.1022778
SRR17520069.1022782
SRR17520069.1022784
SRR17520069.1022788
SRR17520069.1022790
SRR17520069.1022792
SRR17520069.1032915
SRR17520069.1032917
SRR17520069.1032919
SRR17520069.1032921
SRR17520069.1032923
SRR17520069.1032925
SRR17520069.1032929
SRR17520069.1032931
SRR17520069.1032933
SRR17520069.1032935
SRR17520069.1032937
SRR17520069.1032939
SRR17520069.1032943
SRR17520069.1032945
SRR17520069.1032947
SRR17520069.1033158
SRR17520069.1038513
SRR17520069.1038515
SRR17520069.1038517
SRR17520069.1038519
SRR17520069.1038521
SRR17520069.1038523
SRR17520069.1038525
SRR17520069.1039083
SRR17520069.1039085
SRR17520069.1039087
SRR17520069.1039089
SRR17520069.1039091
SRR17520069.1039093
SRR17520069.1039095
SRR17520069.1039097
SRR17520069.1039099
SRR17520069.1039101
SRR17520069.1039103
SRR17520069.1039105
SRR17520069.1039107

SRR17520069.1039109
SRR17520069.1039111
SRR17520069.1039113
SRR17520069.1039117
SRR17520069.1039119
SRR17520069.1039121
SRR17520069.1039123
SRR17520069.1039125
SRR17520069.1039127
SRR17520069.1039129
SRR17520069.1039131
SRR17520069.1039133
SRR17520069.1039139
SRR17520069.1039141
SRR17520069.1039143
SRR17520069.1039147
SRR17520069.1039149
SRR17520069.1039151
SRR17520069.1039155
SRR17520069.1039165
SRR17520069.1039171
SRR17520069.1039173
SRR17520069.1039175
SRR17520069.1039177
SRR17520069.1039179
SRR17520069.1039181
SRR17520069.1039183
SRR17520069.1039185
SRR17520069.1039187
SRR17520069.1039189
SRR17520069.1039191
SRR17520069.1039193
SRR17520069.1039195
SRR17520069.1039197
SRR17520069.1039199
SRR17520069.1039201
SRR17520069.1039203
SRR17520069.1039205
SRR17520069.1040321
SRR17520069.1040323
SRR17520069.1040325
SRR17520069.1040327
SRR17520069.1040331
SRR17520069.1043002
SRR17520069.1043004
SRR17520069.1043006
SRR17520069.1043008

SRR17520069.1043010
SRR17520069.1043012
SRR17520069.1043014
SRR17520069.1043016
SRR17520069.1043018
SRR17520069.1043020
SRR17520069.1043022
SRR17520069.1043024
SRR17520069.1043026
SRR17520069.1043028
SRR17520069.1043030
SRR17520069.1043032
SRR17520069.1043034
SRR17520069.1043036
SRR17520069.1043040
SRR17520069.1043042
SRR17520069.1043044
SRR17520069.1043046
SRR17520069.1043048
SRR17520069.1043050
SRR17520069.1043052
SRR17520069.1043054
SRR17520069.1043056
SRR17520069.1043058
SRR17520069.1043062
SRR17520069.1043064
SRR17520069.1043066
SRR17520069.1043070
SRR17520069.1043072
SRR17520069.1043074
SRR17520069.1043078
SRR17520069.1043080
SRR17520069.1043082
SRR17520069.1043084
SRR17520069.1043086
SRR17520069.1043088
SRR17520069.1043090
SRR17520069.1043092
SRR17520069.1043094
SRR17520069.1043096
SRR17520069.1043098
SRR17520069.1043100
SRR17520069.1043102
SRR17520069.1043104
SRR17520069.1043106
SRR17520069.1043108
SRR17520069.1043110

SRR17520069.1043112
SRR17520069.1043114
SRR17520069.1043118
SRR17520069.1044896
SRR17520069.1044898
SRR17520069.1044900
SRR17520069.1044902
SRR17520069.1044904
SRR17520069.1044906
SRR17520069.1044908
SRR17520069.1044910
SRR17520069.1044912
SRR17520069.1044914
SRR17520069.1044916
SRR17520069.1044918
SRR17520069.1044920
SRR17520069.1044922
SRR17520069.1044924
SRR17520069.1044926
SRR17520069.1044928
SRR17520069.1051458
SRR17520069.1051460
SRR17520069.1051462
SRR17520069.1056848
SRR17520069.1065394
SRR17520069.1065396
SRR17520069.1065398
SRR17520069.1065400
SRR17520069.1065402
SRR17520069.1065404
SRR17520069.1065406
SRR17520069.1065410
SRR17520069.1065414
SRR17520069.1065416
SRR17520069.1065418
SRR17520069.1065420
SRR17520069.1069271
SRR17520069.1071921
SRR17520069.1071925
SRR17520069.1071927
SRR17520069.1071929
SRR17520069.1071931
SRR17520069.1071933
SRR17520069.1071935
SRR17520069.1071937
SRR17520069.1071939
SRR17520069.1071941

SRR17520069.1071949
SRR17520069.1071951
SRR17520069.1071953
SRR17520069.1071955
SRR17520069.1071957
SRR17520069.1071959
SRR17520069.1071961
SRR17520069.1071963
SRR17520069.1071965
SRR17520069.1071967
SRR17520069.1071969
SRR17520069.1071971
SRR17520069.1071973
SRR17520069.1071977
SRR17520069.1071979
SRR17520069.1078080
SRR17520069.1078082
SRR17520069.1078084
SRR17520069.1078086
SRR17520069.1078088
SRR17520069.1078092
SRR17520069.1078094
SRR17520069.1078096
SRR17520069.1078098
SRR17520069.1078100
SRR17520069.1078102
SRR17520069.1078104
SRR17520069.1078106
SRR17520069.1078108
SRR17520069.1078110
SRR17520069.1078114
SRR17520069.1078116
SRR17520069.1078118
SRR17520069.1078120
SRR17520069.1078122
SRR17520069.1078124
SRR17520069.1078128
SRR17520069.1078132
SRR17520069.1078134
SRR17520069.1078136
SRR17520069.1081002
SRR17520069.1081004
SRR17520069.1081006
SRR17520069.1081008
SRR17520069.1081010
SRR17520069.1081012
SRR17520069.1081014

SRR17520069.1081016
SRR17520069.1081018
SRR17520069.1081020
SRR17520069.1081022
SRR17520069.1081024
SRR17520069.1090519
SRR17520069.1093379
SRR17520069.1093381
SRR17520069.1093383
SRR17520069.1093385
SRR17520069.1093387
SRR17520069.1093389
SRR17520069.1093391
SRR17520069.1093393
SRR17520069.1093395
SRR17520069.1093397
SRR17520069.1093399
SRR17520069.1093401
SRR17520069.1093403
SRR17520069.1093405
SRR17520069.1093407
SRR17520069.1100178
SRR17520069.1104028
SRR17520069.1104030
SRR17520069.1104032
SRR17520069.1104034
SRR17520069.1104036
SRR17520069.1104038
SRR17520069.1104040
SRR17520069.1104042
SRR17520069.1104044
SRR17520069.1104046
SRR17520069.1104048
SRR17520069.1104050
SRR17520069.1104052
SRR17520069.1104054
SRR17520069.1104056
SRR17520069.1104058
SRR17520069.1104060
SRR17520069.1104062
SRR17520069.1104064
SRR17520069.1104066
SRR17520069.1104068
SRR17520069.1104070
SRR17520069.1104072
SRR17520069.1104074
SRR17520069.1104620

SRR17520069.1104622
SRR17520069.1104624
SRR17520069.1104626
SRR17520069.1104628
SRR17520069.1104630
SRR17520069.1105631
SRR17520069.1105633
SRR17520069.1105635
SRR17520069.1105637
SRR17520069.1109450
SRR17520069.1109454
SRR17520069.1109456
SRR17520069.1109458
SRR17520069.1110976
SRR17520069.1110982
SRR17520069.1110984
SRR17520069.1110986
SRR17520069.1110988
SRR17520069.1110990
SRR17520069.1110992
SRR17520069.1110994
SRR17520069.1110996
SRR17520069.1110998
SRR17520069.1111000
SRR17520069.1111002
SRR17520069.1111004
SRR17520069.1111006
SRR17520069.1111008
SRR17520069.1111010
SRR17520069.1111012
SRR17520069.1111014
SRR17520069.1111016
SRR17520069.1111018
SRR17520069.1111020
SRR17520069.1111022
SRR17520069.1111024
SRR17520069.1111026
SRR17520069.1111028
SRR17520069.1111030
SRR17520069.1111032
SRR17520069.1111034
SRR17520069.1111036
SRR17520069.1111038
SRR17520069.1111040
SRR17520069.1111044
SRR17520069.1112024
SRR17520069.1112028

SRR17520069.1112030
SRR17520069.1112032
SRR17520069.1112038
SRR17520069.1112040
SRR17520069.1112044
SRR17520069.1112046
SRR17520069.1112048
SRR17520069.1115602
SRR17520069.1115604
SRR17520069.1115606
SRR17520069.1115608
SRR17520069.1115610
SRR17520069.1115612
SRR17520069.1115614
SRR17520069.1115961
SRR17520069.1115963
SRR17520069.1115965
SRR17520069.1115967
SRR17520069.1115969
SRR17520069.1115971
SRR17520069.1115973
SRR17520069.1116149
SRR17520069.1116151
SRR17520069.1116153
SRR17520069.1116155
SRR17520069.1116157
SRR17520069.1116159
SRR17520069.1116161
SRR17520069.1116163
SRR17520069.1116165
SRR17520069.1116167
SRR17520069.1116169
SRR17520069.1116171
SRR17520069.1116173
SRR17520069.1116175
SRR17520069.1116177
SRR17520069.1116179
SRR17520069.1116181
SRR17520069.1116183
SRR17520069.1116185
SRR17520069.1116187
SRR17520069.1116189
SRR17520069.1116191
SRR17520069.1116193
SRR17520069.1116197
SRR17520069.1116199
SRR17520069.1116201

SRR17520069.1116203
SRR17520069.1116205
SRR17520069.1116207
SRR17520069.1116209
SRR17520069.1116211
SRR17520069.1116213
SRR17520069.1116215
SRR17520069.1116217
SRR17520069.1116219
SRR17520069.1116221
SRR17520069.1116223
SRR17520069.1116225
SRR17520069.1116227
SRR17520069.1121237
SRR17520069.1121239
SRR17520069.1121241
SRR17520069.1121243
SRR17520069.1121245
SRR17520069.1121247
SRR17520069.1121384
SRR17520069.1124709
SRR17520069.1124714
SRR17520069.1125341
SRR17520069.1128342
SRR17520069.1128344
SRR17520069.1128346
SRR17520069.1128348
SRR17520069.1128350
SRR17520069.1128352
SRR17520069.1128354
SRR17520069.1128356
SRR17520069.1128358
SRR17520069.1136716
SRR17520069.1136718
SRR17520069.1136720
SRR17520069.1136722
SRR17520069.1136724
SRR17520069.1136726
SRR17520069.1136728
SRR17520069.1136730
SRR17520069.1136732
SRR17520069.1136734
SRR17520069.1136736
SRR17520069.1136738
SRR17520069.1136740
SRR17520069.1136742
SRR17520069.1136744

SRR17520069.1136746
SRR17520069.1136748
SRR17520069.1136750
SRR17520069.1136752
SRR17520069.1136754
SRR17520069.1136756
SRR17520069.1136758
SRR17520069.1136760
SRR17520069.1136762
SRR17520069.1141609
SRR17520069.1141611
SRR17520069.1141613
SRR17520069.1141615
SRR17520069.1141617
SRR17520069.1141619
SRR17520069.1141621
SRR17520069.1141623
SRR17520069.1141625
SRR17520069.1141627
SRR17520069.1141629
SRR17520069.1141631
SRR17520069.1141633
SRR17520069.1141635
SRR17520069.1141639
SRR17520069.1141641
SRR17520069.1141643
SRR17520069.1141645
SRR17520069.1141647
SRR17520069.1141649
SRR17520069.1141651
SRR17520069.1141653
SRR17520069.1141655
SRR17520069.1141657
SRR17520069.1141659
SRR17520069.1141661
SRR17520069.1141663
SRR17520069.1141665
SRR17520069.1141667
SRR17520069.1141669
SRR17520069.1141671
SRR17520069.1141673
SRR17520069.1141675
SRR17520069.1141677
SRR17520069.1141679
SRR17520069.1141681
SRR17520069.1141683
SRR17520069.1141685

SRR17520069.1141687
SRR17520069.1141689
SRR17520069.1141691
SRR17520069.1141693
SRR17520069.1141695
SRR17520069.1141697
SRR17520069.1141699
SRR17520069.1141701
SRR17520069.1141703
SRR17520069.1141705
SRR17520069.1149342
SRR17520069.1149344
SRR17520069.1149346
SRR17520069.1154408
SRR17520069.1154410
SRR17520069.1154412
SRR17520069.1154416
SRR17520069.1154418
SRR17520069.1155979
SRR17520069.1155981
SRR17520069.1155983
SRR17520069.1155985
SRR17520069.1155987
SRR17520069.1155989
SRR17520069.1155991
SRR17520069.1155993
SRR17520069.1155995
SRR17520069.1155997
SRR17520069.1155999
SRR17520069.1156001
SRR17520069.1156003
SRR17520069.1156005
SRR17520069.1156007
SRR17520069.1156009
SRR17520069.1156011
SRR17520069.1156013
SRR17520069.1156017
SRR17520069.1156019
SRR17520069.1170094
SRR17520069.1170096
SRR17520069.1170098
SRR17520069.1173491
SRR17520069.1173493
SRR17520069.1173495
SRR17520069.1173497
SRR17520069.1173499
SRR17520069.1173501

SRR17520069.1173503
SRR17520069.1173505
SRR17520069.1173507
SRR17520069.1173509
SRR17520069.1173511
SRR17520069.1173513
SRR17520069.1173515
SRR17520069.1173517
SRR17520069.1173519
SRR17520069.1173521
SRR17520069.1173523
SRR17520069.1173525
SRR17520069.1173527
SRR17520069.1173529
SRR17520069.1173531
SRR17520069.1173533
SRR17520069.1173535
SRR17520069.1173537
SRR17520069.1173539
SRR17520069.1173541
SRR17520069.1173543
SRR17520069.1178131
SRR17520069.1182973
SRR17520069.1182975
SRR17520069.1182977
SRR17520069.1182979
SRR17520069.1182981
SRR17520069.1182983
SRR17520069.1182985
SRR17520069.1182987
SRR17520069.1182989
SRR17520069.1182991
SRR17520069.1182993
SRR17520069.1182995
SRR17520069.1182997
SRR17520069.1182999
SRR17520069.1183001
SRR17520069.1183003
SRR17520069.1183005
SRR17520069.1183007
SRR17520069.1183009
SRR17520069.1183011
SRR17520069.1183013
SRR17520069.1183015
SRR17520069.1183017
SRR17520069.1183019
SRR17520069.1183021

SRR17520069.1183023
SRR17520069.1183025
SRR17520069.1183027
SRR17520069.1183029
SRR17520069.1183031
SRR17520069.1183033
SRR17520069.1183035
SRR17520069.1183037
SRR17520069.1183039
SRR17520069.1183041
SRR17520069.1183043
SRR17520069.1183045
SRR17520069.1183047
SRR17520069.1183049
SRR17520069.1183051
SRR17520069.1183053
SRR17520069.1183055
SRR17520069.1183057
SRR17520069.1183059
SRR17520069.1183061
SRR17520069.1183063
SRR17520069.1183065
SRR17520069.1183067
SRR17520069.1183069
SRR17520069.1183071
SRR17520069.1183073
SRR17520069.1183075
SRR17520069.1183077
SRR17520069.1183079
SRR17520069.1183081
SRR17520069.1183083
SRR17520069.1183085
SRR17520069.1185828
SRR17520069.1185830
SRR17520069.1185832
SRR17520069.1185834
SRR17520069.1185836
SRR17520069.1185838
SRR17520069.1185840
SRR17520069.1185842
SRR17520069.1187421
SRR17520069.1187423
SRR17520069.1187425
SRR17520069.1187427
SRR17520069.1187429
SRR17520069.1187431
SRR17520069.1187433

SRR17520069.1187437
SRR17520069.1187439
SRR17520069.1187441
SRR17520069.1187443
SRR17520069.1187445
SRR17520069.1187447
SRR17520069.1187449
SRR17520069.1187451
SRR17520069.1187453
SRR17520069.1187455
SRR17520069.1187457
SRR17520069.1187459
SRR17520069.1187461
SRR17520069.1187463
SRR17520069.1187465
SRR17520069.1187467
SRR17520069.1187471
SRR17520069.1187473
SRR17520069.1187475
SRR17520069.1187477
SRR17520069.1187479
SRR17520069.1187481
SRR17520069.1187483
SRR17520069.1187485
SRR17520069.1187487
SRR17520069.1187489
SRR17520069.1187491
SRR17520069.1187493
SRR17520069.1188074
SRR17520069.1188631
SRR17520069.1188633
SRR17520069.1188635
SRR17520069.1188637
SRR17520069.1188639
SRR17520069.1188641
SRR17520069.1188643
SRR17520069.1201709
SRR17520069.1202093
SRR17520069.1205151
SRR17520069.1205153
SRR17520069.1205155
SRR17520069.1205157
SRR17520069.1205159
SRR17520069.1205161
SRR17520069.1205163
SRR17520069.1205165
SRR17520069.1205167

SRR17520069.1205169
SRR17520069.1205171
SRR17520069.1205173
SRR17520069.1205175
SRR17520069.1205177
SRR17520069.1205179
SRR17520069.1205181
SRR17520069.1205183
SRR17520069.1205185
SRR17520069.1205187
SRR17520069.1208149
SRR17520069.1208151
SRR17520069.1208153
SRR17520069.1208155
SRR17520069.1208157
SRR17520069.1209634
SRR17520069.1209638
SRR17520069.1209640
SRR17520069.1209642
SRR17520069.1209644
SRR17520069.1214756
SRR17520069.1214760
SRR17520069.1214762
SRR17520069.1214764
SRR17520069.1214766
SRR17520069.1214768
SRR17520069.1214770
SRR17520069.1216339
SRR17520069.1216341
SRR17520069.1216343
SRR17520069.1217346
SRR17520069.1217348
SRR17520069.1225958
SRR17520069.1225960
SRR17520069.1225962
SRR17520069.1225964
SRR17520069.1225966
SRR17520069.1225968
SRR17520069.1225970
SRR17520069.1225972
SRR17520069.1225974
SRR17520069.1225976
SRR17520069.1225978
SRR17520069.1225980
SRR17520069.1225982
SRR17520069.1225984
SRR17520069.1228929

SRR17520069.1228931
SRR17520069.1228933
SRR17520069.1228935
SRR17520069.1228937
SRR17520069.1228939
SRR17520069.1228941
SRR17520069.1228943
SRR17520069.1228945
SRR17520069.1228947
SRR17520069.1228949
SRR17520069.1228951
SRR17520069.1228953
SRR17520069.1228957
SRR17520069.1228959
SRR17520069.1228961
SRR17520069.1228963
SRR17520069.1228965
SRR17520069.1228967
SRR17520069.1228969
SRR17520069.1235382
SRR17520069.1235384
SRR17520069.1235386
SRR17520069.1235388
SRR17520069.1235390
SRR17520069.1235392
SRR17520069.1235394
SRR17520069.1237662
SRR17520069.1237664
SRR17520069.1237666
SRR17520069.1237668
SRR17520069.1237670
SRR17520069.1237672
SRR17520069.1237674
SRR17520069.1237676
SRR17520069.1237678
SRR17520069.1238649
SRR17520069.1238651
SRR17520069.1238653
SRR17520069.1238655
SRR17520069.1238657
SRR17520069.1238659
SRR17520069.1238661
SRR17520069.1243686
SRR17520069.1243688
SRR17520069.1243690
SRR17520069.1243692
SRR17520069.1243694

SRR17520069.1243696
SRR17520069.1243698
SRR17520069.1243700
SRR17520069.1243702
SRR17520069.1243704
SRR17520069.1243706
SRR17520069.1243709
SRR17520069.1243711
SRR17520069.1243713
SRR17520069.1243715
SRR17520069.1243717
SRR17520069.1243719
SRR17520069.1243721
SRR17520069.1243723
SRR17520069.1243725
SRR17520069.1243727
SRR17520069.1243729
SRR17520069.1249508
SRR17520069.1249512
SRR17520069.1249516
SRR17520069.1249518
SRR17520069.1249520
SRR17520069.1249522
SRR17520069.1249524
SRR17520069.1249526
SRR17520069.1249530
SRR17520069.1249532
SRR17520069.1249534
SRR17520069.1249536
SRR17520069.1249538
SRR17520069.1249540
SRR17520069.1249544
SRR17520069.1249546
SRR17520069.1249550
SRR17520069.1249552
SRR17520069.1249556
SRR17520069.1249558
SRR17520069.1249560
SRR17520069.1249568
SRR17520069.1249572
SRR17520069.1249576
SRR17520069.1249578
SRR17520069.1249580
SRR17520069.1249582
SRR17520069.1249586
SRR17520069.1249588
SRR17520069.1249590

SRR17520069.1249594
SRR17520069.1249598
SRR17520069.1249600
SRR17520069.1256932
SRR17520069.1256938
SRR17520069.1256962
SRR17520069.1257631
SRR17520069.1257633
SRR17520069.1258183
SRR17520069.1258185
SRR17520069.1258187
SRR17520069.1258189
SRR17520069.1258191
SRR17520069.1258193
SRR17520069.1258195
SRR17520069.1258755
SRR17520069.1258757
SRR17520069.1258759
SRR17520069.1258761
SRR17520069.1258763
SRR17520069.1258765
SRR17520069.1258767
SRR17520069.1258769
SRR17520069.1258771
SRR17520069.1258773
SRR17520069.1258775
SRR17520069.1258777
SRR17520069.1258779
SRR17520069.1258781
SRR17520069.1258783
SRR17520069.1258785
SRR17520069.1258787
SRR17520069.1264773
SRR17520069.1267958
SRR17520069.1267960
SRR17520069.1267962
SRR17520069.1267964
SRR17520069.1267966
SRR17520069.1267970
SRR17520069.1267972
SRR17520069.1278768
SRR17520069.1278770
SRR17520069.1278772
SRR17520069.1278774
SRR17520069.1278776
SRR17520069.1278778
SRR17520069.1278780

SRR17520069.1278782
SRR17520069.1278784
SRR17520069.1278786
SRR17520069.1278788
SRR17520069.1278790
SRR17520069.1278792
SRR17520069.1278794
SRR17520069.1278796
SRR17520069.1278798
SRR17520069.1278800
SRR17520069.1278802
SRR17520069.1278804
SRR17520069.1278806
SRR17520069.1278808
SRR17520069.1278810
SRR17520069.1278814
SRR17520069.1278816
SRR17520069.1278818
SRR17520069.1278820
SRR17520069.1278822
SRR17520069.1278824
SRR17520069.1278826
SRR17520069.1278828
SRR17520069.1278830
SRR17520069.1278832
SRR17520069.1278834
SRR17520069.1278836
SRR17520069.1278838
SRR17520069.1278840
SRR17520069.1278842
SRR17520069.1278846
SRR17520069.1278848
SRR17520069.1278850
SRR17520069.1278852
SRR17520069.1278854
SRR17520069.1278856
SRR17520069.1278858
SRR17520069.1278860
SRR17520069.1278862
SRR17520069.1278864
SRR17520069.1278866
SRR17520069.1278868
SRR17520069.1278870
SRR17520069.1278872
SRR17520069.1278874
SRR17520069.1278876
SRR17520069.1278878

SRR17520069.1278880
SRR17520069.1278882
SRR17520069.1278884
SRR17520069.1278886
SRR17520069.1278888
SRR17520069.1278890
SRR17520069.1278903
SRR17520069.1278905
SRR17520069.1278907
SRR17520069.1278917
SRR17520069.1278927
SRR17520069.1278931
SRR17520069.1278939
SRR17520069.1282102
SRR17520069.1282104
SRR17520069.1288018
SRR17520069.1288020
SRR17520069.1288022
SRR17520069.1288024
SRR17520069.1288026
SRR17520069.1288028
SRR17520069.1288030
SRR17520069.1288032
SRR17520069.1288034
SRR17520069.1288036
SRR17520069.1288038
SRR17520069.1288040
SRR17520069.1288042
SRR17520069.1288044
SRR17520069.1288046
SRR17520069.1288048
SRR17520069.1288050
SRR17520069.1288052
SRR17520069.1288054
SRR17520069.1288056
SRR17520069.1288058
SRR17520069.1288062
SRR17520069.1288064
SRR17520069.1288066
SRR17520069.1288068
SRR17520069.1288070
SRR17520069.1288072
SRR17520069.1288074
SRR17520069.1288076
SRR17520069.1288078
SRR17520069.1288082
SRR17520069.1288084

SRR17520069.1288086
SRR17520069.1288088
SRR17520069.1288090
SRR17520069.1288092
SRR17520069.1288096
SRR17520069.1288098
SRR17520069.1288100
SRR17520069.1288102
SRR17520069.1288104
SRR17520069.1291972
SRR17520069.1291974
SRR17520069.1291976
SRR17520069.1291978
SRR17520069.1291980
SRR17520069.1291982
SRR17520069.1291984
SRR17520069.1291986
SRR17520069.1291988
SRR17520069.1291990
SRR17520069.1291992
SRR17520069.1291994
SRR17520069.1291996
SRR17520069.1291998
SRR17520069.1292000
SRR17520069.1292002
SRR17520069.1292004
SRR17520069.1292006
SRR17520069.1292008
SRR17520069.1292010
SRR17520069.1292012
SRR17520069.1292014
SRR17520069.1292016
SRR17520069.1292018
SRR17520069.1292020
SRR17520069.1292022
SRR17520069.1292024
SRR17520069.1292026
SRR17520069.1292028
SRR17520069.1292030
SRR17520069.1292032
SRR17520069.1292034
SRR17520069.1292036
SRR17520069.1292038
SRR17520069.1292040
SRR17520069.1292042
SRR17520069.1292044
SRR17520069.1292872

SRR17520069.1292874
SRR17520069.1292878
SRR17520069.1292880
SRR17520069.1292882
SRR17520069.1292884
SRR17520069.1292886
SRR17520069.1292890
SRR17520069.1292892
SRR17520069.1292894
SRR17520069.1293830
SRR17520069.1293832
SRR17520069.1293834
SRR17520069.1294886
SRR17520069.1295399
SRR17520069.1295401
SRR17520069.1295403
SRR17520069.1295407
SRR17520069.1295409
SRR17520069.1295411
SRR17520069.1295413
SRR17520069.1296435
SRR17520069.1296437
SRR17520069.1296439
SRR17520069.1296441
SRR17520069.1296443
SRR17520069.1296445
SRR17520069.1296447
SRR17520069.1296449
SRR17520069.1296451
SRR17520069.1298480
SRR17520069.1298482
SRR17520069.1298484
SRR17520069.1298486
SRR17520069.1298488
SRR17520069.1299051
SRR17520069.1299053
SRR17520069.1299055
SRR17520069.1299057
SRR17520069.1299059
SRR17520069.1299061
SRR17520069.1299063
SRR17520069.1299065
SRR17520069.1299067
SRR17520069.1299069
SRR17520069.1299071
SRR17520069.1299580
SRR17520069.1299582

SRR17520069.1299584
SRR17520069.1299586
SRR17520069.1299588
SRR17520069.1299590
SRR17520069.1299592
SRR17520069.1299594
SRR17520069.1299596
SRR17520069.1299598
SRR17520069.1302833
SRR17520069.1302835
SRR17520069.1302837
SRR17520069.1302839
SRR17520069.1302841
SRR17520069.1302843
SRR17520069.1302845
SRR17520069.1302847
SRR17520069.1302849
SRR17520069.1307456
SRR17520069.1307458
SRR17520069.1316685
SRR17520069.1316761
SRR17520069.1316773
SRR17520069.1319164
SRR17520069.1320279
SRR17520069.1320281
SRR17520069.1322946
SRR17520069.1322948
SRR17520069.1322950
SRR17520069.1322952
SRR17520069.1322954
SRR17520069.1322956
SRR17520069.1322958
SRR17520069.1322960
SRR17520069.1322962
SRR17520069.1322964
SRR17520069.1322966
SRR17520069.1322968
SRR17520069.1322970
SRR17520069.1322972
SRR17520069.1322974
SRR17520069.1322976
SRR17520069.1322978
SRR17520069.1322980
SRR17520069.1329758
SRR17520069.1329760
SRR17520069.1332796
SRR17520069.1332798

SRR17520069.1332800
SRR17520069.1332802
SRR17520069.1332804
SRR17520069.1332806
SRR17520069.1332808
SRR17520069.1332810
SRR17520069.1332812
SRR17520069.1332814
SRR17520069.1332816
SRR17520069.1332818
SRR17520069.1332820
SRR17520069.1332822
SRR17520069.1334564
SRR17520069.1334566
SRR17520069.1334568
SRR17520069.1334570
SRR17520069.1334574
SRR17520069.1334578
SRR17520069.1335975
SRR17520069.1335977
SRR17520069.1335979
SRR17520069.1335981
SRR17520069.1335983
SRR17520069.1335985
SRR17520069.1335987
SRR17520069.1335989
SRR17520069.1335991
SRR17520069.1335993
SRR17520069.1335995
SRR17520069.1335997
SRR17520069.1335999
SRR17520069.1336001
SRR17520069.1336003
SRR17520069.1336005
SRR17520069.1336007
SRR17520069.1336009
SRR17520069.1336011
SRR17520069.1336013
SRR17520069.1336015
SRR17520069.1336017
SRR17520069.1336019
SRR17520069.1336021
SRR17520069.1336023
SRR17520069.1336025
SRR17520069.1336027
SRR17520069.1336029
SRR17520069.1336031

SRR17520069.1336033
SRR17520069.1336035
SRR17520069.1336039
SRR17520069.1336041
SRR17520069.1336043
SRR17520069.1336045
SRR17520069.1336047
SRR17520069.1336049
SRR17520069.1336051
SRR17520069.1336053
SRR17520069.1336055
SRR17520069.1336059
SRR17520069.1336061
SRR17520069.1336063
SRR17520069.1336065
SRR17520069.1336067
SRR17520069.1340596
SRR17520069.1341610
SRR17520069.1341612
SRR17520069.1341614
SRR17520069.1342002
SRR17520069.1342004
SRR17520069.1342006
SRR17520069.1342008
SRR17520069.1342010
SRR17520069.1342012
SRR17520069.1342014
SRR17520069.1342016
SRR17520069.1342020
SRR17520069.1342022
SRR17520069.1342024
SRR17520069.1342026
SRR17520069.1342028
SRR17520069.1342030
SRR17520069.1342032
SRR17520069.1342034
SRR17520069.1342036
SRR17520069.1342038
SRR17520069.1342040
SRR17520069.1342042
SRR17520069.1342044
SRR17520069.1342046
SRR17520069.1342048
SRR17520069.1342050
SRR17520069.1342052
SRR17520069.1342054
SRR17520069.1342056

SRR17520069.1342058
SRR17520069.1342060
SRR17520069.1342062
SRR17520069.1342064
SRR17520069.1342066
SRR17520069.1342068
SRR17520069.1342070
SRR17520069.1342072
SRR17520069.1342074
SRR17520069.1342076
SRR17520069.1342078
SRR17520069.1342080
SRR17520069.1342082
SRR17520069.1342084
SRR17520069.1342086
SRR17520069.1347336
SRR17520069.1347411
SRR17520069.1351646
SRR17520069.1351648
SRR17520069.1351650
SRR17520069.1351652
SRR17520069.1351654
SRR17520069.1351658
SRR17520069.1351660
SRR17520069.1352024
SRR17520069.1352026
SRR17520069.1352028
SRR17520069.1352030
SRR17520069.1352032
SRR17520069.1352034
SRR17520069.1352038
SRR17520069.1352040
SRR17520069.1352042
SRR17520069.1352044
SRR17520069.1352046
SRR17520069.1352048
SRR17520069.1352050
SRR17520069.1352052
SRR17520069.1352054
SRR17520069.1352056
SRR17520069.1352058
SRR17520069.1352060
SRR17520069.1352064
SRR17520069.1352066
SRR17520069.1352068
SRR17520069.1352070
SRR17520069.1352072

SRR17520069.1352074
SRR17520069.1352076
SRR17520069.1352078
SRR17520069.1352080
SRR17520069.1352082
SRR17520069.1352084
SRR17520069.1352086
SRR17520069.1352088
SRR17520069.1352090
SRR17520069.1352092
SRR17520069.1352094
SRR17520069.1352096
SRR17520069.1352098
SRR17520069.1352100
SRR17520069.1357470
SRR17520069.1357472
SRR17520069.1357474
SRR17520069.1357476
SRR17520069.1357478
SRR17520069.1357480
SRR17520069.1357482
SRR17520069.1357484
SRR17520069.1357486
SRR17520069.1357488
SRR17520069.1357490
SRR17520069.1357492
SRR17520069.1357494
SRR17520069.1357496
SRR17520069.1357498
SRR17520069.1357500
SRR17520069.1357502
SRR17520069.1357504
SRR17520069.1357506
SRR17520069.1357508
SRR17520069.1357510
SRR17520069.1357512
SRR17520069.1357514
SRR17520069.1357516
SRR17520069.1357518
SRR17520069.1357520
SRR17520069.1357522
SRR17520069.1357524
SRR17520069.1357526
SRR17520069.1357528
SRR17520069.1357530
SRR17520069.1357532
SRR17520069.1357534

SRR17520069.1357536
SRR17520069.1357538
SRR17520069.1357540
SRR17520069.1357542
SRR17520069.1357544
SRR17520069.1357546
SRR17520069.1357548
SRR17520069.1357550
SRR17520069.1357552
SRR17520069.1357554
SRR17520069.1357556
SRR17520069.1357558
SRR17520069.1357560
SRR17520069.1357562
SRR17520069.1357564
SRR17520069.1357566
SRR17520069.1358094
SRR17520069.1358098
SRR17520069.1358100
SRR17520069.1358102
SRR17520069.1358104
SRR17520069.1358106
SRR17520069.1358108
SRR17520069.1358110
SRR17520069.1358112
SRR17520069.1358114
SRR17520069.1358116
SRR17520069.1358118
SRR17520069.1358120
SRR17520069.1358122
SRR17520069.1358126
SRR17520069.1358128
SRR17520069.1358130
SRR17520069.1358134
SRR17520069.1358136
SRR17520069.1358138
SRR17520069.1358140
SRR17520069.1362508
SRR17520069.1362510
SRR17520069.1362512
SRR17520069.1362514
SRR17520069.1362518
SRR17520069.1362520
SRR17520069.1362522
SRR17520069.1362524
SRR17520069.1362526
SRR17520069.1362528

SRR17520069.1362530
SRR17520069.1362532
SRR17520069.1364220
SRR17520069.1364222
SRR17520069.1364224
SRR17520069.1364226
SRR17520069.1364228
SRR17520069.1364230
SRR17520069.1364232
SRR17520069.1364234
SRR17520069.1364236
SRR17520069.1364238
SRR17520069.1364240
SRR17520069.1364242
SRR17520069.1364244
SRR17520069.1364246
SRR17520069.1364248
SRR17520069.1364250
SRR17520069.1364252
SRR17520069.1364254
SRR17520069.1364256
SRR17520069.1364258
SRR17520069.1364260
SRR17520069.1364262
SRR17520069.1364264
SRR17520069.1364266
SRR17520069.1364268
SRR17520069.1364270
SRR17520069.1364272
SRR17520069.1364274
SRR17520069.1364276
SRR17520069.1364278
SRR17520069.1364280
SRR17520069.1364282
SRR17520069.1364284
SRR17520069.1364286
SRR17520069.1364288
SRR17520069.1364290
SRR17520069.1364292
SRR17520069.1364296
SRR17520069.1364298
SRR17520069.1364300
SRR17520069.1364304
SRR17520069.1364308
SRR17520069.1364310
SRR17520069.1364312
SRR17520069.1364316

SRR17520069.1364318
SRR17520069.1364320
SRR17520069.1367223
SRR17520069.1367225
SRR17520069.1367227
SRR17520069.1367229
SRR17520069.1367231
SRR17520069.1367233
SRR17520069.1367237
SRR17520069.1367239
SRR17520069.1367241
SRR17520069.1368185
SRR17520069.1368189
SRR17520069.1368191
SRR17520069.1368193
SRR17520069.1368195
SRR17520069.1368199
SRR17520069.1368203
SRR17520069.1368205
SRR17520069.1368207
SRR17520069.1368209
SRR17520069.1368211
SRR17520069.1368215
SRR17520069.1368219
SRR17520069.1368221
SRR17520069.1368223
SRR17520069.1368225
SRR17520069.1368227
SRR17520069.1368229
SRR17520069.1368231
SRR17520069.1368233
SRR17520069.1368235
SRR17520069.1368237
SRR17520069.1381084
SRR17520069.1381086
SRR17520069.1381088
SRR17520069.1381090
SRR17520069.1381092
SRR17520069.1381094
SRR17520069.1382720
SRR17520069.1388992
SRR17520069.1388994
SRR17520069.1388996
SRR17520069.1388998
SRR17520069.1389000
SRR17520069.1389002
SRR17520069.1389004

SRR17520069.1389006
SRR17520069.1390013
SRR17520069.1390015
SRR17520069.1390017
SRR17520069.1390019
SRR17520069.1390023
SRR17520069.1390025
SRR17520069.1390029
SRR17520069.1390031
SRR17520069.1390033
SRR17520069.1390035
SRR17520069.1392522
SRR17520069.1394565
SRR17520069.1394567
SRR17520069.1394569
SRR17520069.1394571
SRR17520069.1394573
SRR17520069.1394575
SRR17520069.1394577
SRR17520069.1394579
SRR17520069.1394581
SRR17520069.1394960
SRR17520069.1394962
SRR17520069.1394966
SRR17520069.1394970
SRR17520069.1394972
SRR17520069.1394974
SRR17520069.1394976
SRR17520069.1396738
SRR17520069.1396740
SRR17520069.1396742
SRR17520069.1396744
SRR17520069.1396746
SRR17520069.1396748
SRR17520069.1396750
SRR17520069.1396752
SRR17520069.1396754
SRR17520069.1396756
SRR17520069.1396758
SRR17520069.1396760
SRR17520069.1396762
SRR17520069.1396764
SRR17520069.1396766
SRR17520069.1396768
SRR17520069.1396770
SRR17520069.1396772
SRR17520069.1396774

SRR17520069.1396776
SRR17520069.1396778
SRR17520069.1396780
SRR17520069.1396782
SRR17520069.1396784
SRR17520069.1396786
SRR17520069.1396788
SRR17520069.1396790
SRR17520069.1396792
SRR17520069.1396794
SRR17520069.1396796
SRR17520069.1396798
SRR17520069.1396800
SRR17520069.1396802
SRR17520069.1396804
SRR17520069.1396806
SRR17520069.1396808
SRR17520069.1396810
SRR17520069.1396812
SRR17520069.1396814
SRR17520069.1396816
SRR17520069.1396818
SRR17520069.1396820
SRR17520069.1396822
SRR17520069.1396826
SRR17520069.1396828
SRR17520069.1396830
SRR17520069.1396832
SRR17520069.1396834
SRR17520069.1396836
SRR17520069.1396838
SRR17520069.1396840
SRR17520069.1396842
SRR17520069.1396844
SRR17520069.1396846
SRR17520069.1396848
SRR17520069.1396850
SRR17520069.1396852
SRR17520069.1396854
SRR17520069.1396856
SRR17520069.1396858
SRR17520069.1396860
SRR17520069.1396862
SRR17520069.1396864
SRR17520069.1396866
SRR17520069.1396868
SRR17520069.1396870

SRR17520069.1396872
SRR17520069.1396874
SRR17520069.1396876
SRR17520069.1396878
SRR17520069.1396880
SRR17520069.1396882
SRR17520069.1396884
SRR17520069.1396886
SRR17520069.1396888
SRR17520069.1396890
SRR17520069.1396892
SRR17520069.1396894
SRR17520069.1396896
SRR17520069.1396898
SRR17520069.1396900
SRR17520069.1396902
SRR17520069.1396904
SRR17520069.1396906
SRR17520069.1396908
SRR17520069.1396910
SRR17520069.1396912
SRR17520069.1396914
SRR17520069.1396916
SRR17520069.1396918
SRR17520069.1400492
SRR17520069.1406290
SRR17520069.1406292
SRR17520069.1406391
SRR17520069.1409714
SRR17520069.1409716
SRR17520069.1409718
SRR17520069.1409720
SRR17520069.1409722
SRR17520069.1409724
SRR17520069.1409726
SRR17520069.1409728
SRR17520069.1409730
SRR17520069.1409732
SRR17520069.1409734
SRR17520069.1409736
SRR17520069.1409738
SRR17520069.1409740
SRR17520069.1409742
SRR17520069.1409744
SRR17520069.1412692
SRR17520069.1418511
SRR17520069.1418515

SRR17520069.1418517
SRR17520069.1418519
SRR17520069.1419543
SRR17520069.1419545
SRR17520069.1419547
SRR17520069.1419551
SRR17520069.1419553
SRR17520069.1419555
SRR17520069.1419559
SRR17520069.1433201
SRR17520069.1433205
SRR17520069.1433207
SRR17520069.1433209
SRR17520069.1433211
SRR17520069.1433215
SRR17520069.1433217
SRR17520069.1433219
SRR17520069.1433221
SRR17520069.1433223
SRR17520069.1436076
SRR17520069.1436078
SRR17520069.1436080
SRR17520069.1436082
SRR17520069.1436084
SRR17520069.1436088
SRR17520069.1436090
SRR17520069.1436092
SRR17520069.1436094
SRR17520069.1436096
SRR17520069.1436100
SRR17520069.1436102
SRR17520069.1436104
SRR17520069.1436106
SRR17520069.1436108
SRR17520069.1436110
SRR17520069.1436114
SRR17520069.1436116
SRR17520069.1436118
SRR17520069.1436120
SRR17520069.1436122
SRR17520069.1436124
SRR17520069.1436126
SRR17520069.1436162
SRR17520069.1442190
SRR17520069.1442192
SRR17520069.1442194
SRR17520069.1442196

SRR17520069.1442198
SRR17520069.1442200
SRR17520069.1442202
SRR17520069.1444608
SRR17520069.1444612
SRR17520069.1444614
SRR17520069.1444616
SRR17520069.1444618
SRR17520069.1444620
SRR17520069.1444622
SRR17520069.1445867
SRR17520069.1445869
SRR17520069.1445871
SRR17520069.1445873
SRR17520069.1445875
SRR17520069.1445877
SRR17520069.1445879
SRR17520069.1445881
SRR17520069.1445883
SRR17520069.1445885
SRR17520069.1445887
SRR17520069.1445889
SRR17520069.1446736
SRR17520069.1446738
SRR17520069.1446740
SRR17520069.1446742
SRR17520069.1446744
SRR17520069.1446746
SRR17520069.1446748
SRR17520069.1446750
SRR17520069.1446752
SRR17520069.1446756
SRR17520069.1446758
SRR17520069.1446760
SRR17520069.1446762
SRR17520069.1446764
SRR17520069.1446766
SRR17520069.1446768
SRR17520069.1446770
SRR17520069.1446772
SRR17520069.1446774
SRR17520069.1446776
SRR17520069.1446778
SRR17520069.1446780
SRR17520069.1446782
SRR17520069.1446784
SRR17520069.1446786

SRR17520069.1446788
SRR17520069.1446790
SRR17520069.1446792
SRR17520069.1446794
SRR17520069.1446796
SRR17520069.1446798
SRR17520069.1446800
SRR17520069.1446804
SRR17520069.1446806
SRR17520069.1446808
SRR17520069.1446810
SRR17520069.1446812
SRR17520069.1446814
SRR17520069.1446816
SRR17520069.1446818
SRR17520069.1446820
SRR17520069.1446822
SRR17520069.1446824
SRR17520069.1446826
SRR17520069.1446828
SRR17520069.1446830
SRR17520069.1446832
SRR17520069.1446836
SRR17520069.1446838
SRR17520069.1446840
SRR17520069.1446842
SRR17520069.1446844
SRR17520069.1446846
SRR17520069.1446848
SRR17520069.1446850
SRR17520069.1446852
SRR17520069.1446854
SRR17520069.1446856
SRR17520069.1446858
SRR17520069.1446860
SRR17520069.1446862
SRR17520069.1446864
SRR17520069.1452525
SRR17520069.1452527
SRR17520069.1452529
SRR17520069.1452531
SRR17520069.1453326
SRR17520069.1458488
SRR17520069.1458490
SRR17520069.1458492
SRR17520069.1458494
SRR17520069.1458498

SRR17520069.1458500
SRR17520069.1458502
SRR17520069.1458504
SRR17520069.1458506
SRR17520069.1458508
SRR17520069.1458510
SRR17520069.1458512
SRR17520069.1470937
SRR17520069.1478635
SRR17520069.1478637
SRR17520069.1478639
SRR17520069.1478641
SRR17520069.1478643
SRR17520069.1478645
SRR17520069.1478647
SRR17520069.1478649
SRR17520069.1478651
SRR17520069.1478653
SRR17520069.1478655
SRR17520069.1478657
SRR17520069.1478659
SRR17520069.1478661
SRR17520069.1478663
SRR17520069.1478665
SRR17520069.1478667
SRR17520069.1478669
SRR17520069.1478671
SRR17520069.1478673
SRR17520069.1478677
SRR17520069.1478681
SRR17520069.1478683
SRR17520069.1478685
SRR17520069.1478687
SRR17520069.1478689
SRR17520069.1478691
SRR17520069.1478695
SRR17520069.1478697
SRR17520069.1478699
SRR17520069.1478701
SRR17520069.1478703
SRR17520069.1478707
SRR17520069.1478709
SRR17520069.1478713
SRR17520069.1478715
SRR17520069.1478717
SRR17520069.1478719
SRR17520069.1478723

SRR17520069.1478725
SRR17520069.1478727
SRR17520069.1478729
SRR17520069.1478731
SRR17520069.1478733
SRR17520069.1478735
SRR17520069.1478737
SRR17520069.1478739
SRR17520069.1478741
SRR17520069.1478743
SRR17520069.1478745
SRR17520069.1478747
SRR17520069.1478749
SRR17520069.1478751
SRR17520069.1478753
SRR17520069.1478755
SRR17520069.1478757
SRR17520069.1478759
SRR17520069.1478761
SRR17520069.1478763
SRR17520069.1478765
SRR17520069.1478767
SRR17520069.1478769
SRR17520069.1478771
SRR17520069.1478773
SRR17520069.1478775
SRR17520069.1478779
SRR17520069.1478781
SRR17520069.1478783
SRR17520069.1478785
SRR17520069.1478787
SRR17520069.1478789
SRR17520069.1478791
SRR17520069.1478793
SRR17520069.1478795
SRR17520069.1478797
SRR17520069.1478799
SRR17520069.1478801
SRR17520069.1478803
SRR17520069.1478805
SRR17520069.1478807
SRR17520069.1478809
SRR17520069.1478811
SRR17520069.1478813
SRR17520069.1478815
SRR17520069.1478817
SRR17520069.1478819

SRR17520069.1478821
SRR17520069.1478823
SRR17520069.1478825
SRR17520069.1478829
SRR17520069.1478831
SRR17520069.1478833
SRR17520069.1478835
SRR17520069.1478841
SRR17520069.1478843
SRR17520069.1478845
SRR17520069.1478847
SRR17520069.1478851
SRR17520069.1478853
SRR17520069.1478855
SRR17520069.1478857
SRR17520069.1478859
SRR17520069.1478861
SRR17520069.1478863
SRR17520069.1478865
SRR17520069.1478867
SRR17520069.1478869
SRR17520069.1478871
SRR17520069.1478873
SRR17520069.1478875
SRR17520069.1478877
SRR17520069.1478879
SRR17520069.1478881
SRR17520069.1478883
SRR17520069.1478885
SRR17520069.1478887
SRR17520069.1478889
SRR17520069.1478891
SRR17520069.1478893
SRR17520069.1478895
SRR17520069.1478897
SRR17520069.1478899
SRR17520069.1478901
SRR17520069.1478903
SRR17520069.1478905
SRR17520069.1478907
SRR17520069.1478909
SRR17520069.1478911
SRR17520069.1478913
SRR17520069.1478915
SRR17520069.1478917
SRR17520069.1478919
SRR17520069.1478921

SRR17520069.1478923
SRR17520069.1478925
SRR17520069.1478927
SRR17520069.1478929
SRR17520069.1478931
SRR17520069.1478933
SRR17520069.1478935
SRR17520069.1478937
SRR17520069.1478939
SRR17520069.1478941
SRR17520069.1478943
SRR17520069.1478945
SRR17520069.1478949
SRR17520069.1478951
SRR17520069.1478953
SRR17520069.1478955
SRR17520069.1478957
SRR17520069.1478959
SRR17520069.1478961
SRR17520069.1478963
SRR17520069.1478965
SRR17520069.1478967
SRR17520069.1478969
SRR17520069.1478971
SRR17520069.1478973
SRR17520069.1478975
SRR17520069.1478977
SRR17520069.1478979
SRR17520069.1478981
SRR17520069.1486179
SRR17520069.1486181
SRR17520069.1486183
SRR17520069.1486185
SRR17520069.1486187
SRR17520069.1494140
SRR17520069.1494142
SRR17520069.1494144
SRR17520069.1494146
SRR17520069.1494148
SRR17520069.1494150
SRR17520069.1494152
SRR17520069.1494154
SRR17520069.1494156
SRR17520069.1494158
SRR17520069.1494160
SRR17520069.1494162
SRR17520069.1494164

SRR17520069.1494166
SRR17520069.1494168
SRR17520069.1494170
SRR17520069.1494172
SRR17520069.1494174
SRR17520069.1494176
SRR17520069.1494178
SRR17520069.1494180
SRR17520069.1494182
SRR17520069.1494184
SRR17520069.1494186
SRR17520069.1494190
SRR17520069.1494192
SRR17520069.1494194
SRR17520069.1494196
SRR17520069.1494198
SRR17520069.1494200
SRR17520069.1494202
SRR17520069.1494204
SRR17520069.1496185
SRR17520069.1496187
SRR17520069.1496189
SRR17520069.1496191
SRR17520069.1496195
SRR17520069.1496197
SRR17520069.1496199
SRR17520069.1496201
SRR17520069.1496203
SRR17520069.1496205
SRR17520069.1496207
SRR17520069.1496209
SRR17520069.1496211
SRR17520069.1496213
SRR17520069.1496215
SRR17520069.1496217
SRR17520069.1496219
SRR17520069.1496221
SRR17520069.1496223
SRR17520069.1496225
SRR17520069.1496227
SRR17520069.1496229
SRR17520069.1496231
SRR17520069.1496233
SRR17520069.1496235
SRR17520069.1496237
SRR17520069.1496239
SRR17520069.1496241

SRR17520069.1496243
SRR17520069.1496245
SRR17520069.1496247
SRR17520069.1496249
SRR17520069.1496255
SRR17520069.1496257
SRR17520069.1496259
SRR17520069.1496261
SRR17520069.1496263
SRR17520069.1496265
SRR17520069.1496267
SRR17520069.1496269
SRR17520069.1496271
SRR17520069.1496273
SRR17520069.1496275
SRR17520069.1496277
SRR17520069.1496279
SRR17520069.1496283
SRR17520069.1496285
SRR17520069.1496287
SRR17520069.1496289
SRR17520069.1496291
SRR17520069.1496293
SRR17520069.1496295
SRR17520069.1496297
SRR17520069.1496299
SRR17520069.1496301
SRR17520069.1496303
SRR17520069.1496305
SRR17520069.1496307
SRR17520069.1496309
SRR17520069.1496311
SRR17520069.1496313
SRR17520069.1496315
SRR17520069.1496319
SRR17520069.1496321
SRR17520069.1496323
SRR17520069.1496325
SRR17520069.1496327
SRR17520069.1496329
SRR17520069.1496331
SRR17520069.1496333
SRR17520069.1496335
SRR17520069.1496337
SRR17520069.1496339
SRR17520069.1496341
SRR17520069.1496343

SRR17520069.1496345
SRR17520069.1496347
SRR17520069.1496351
SRR17520069.1496353
SRR17520069.1496355
SRR17520069.1496357
SRR17520069.1496359
SRR17520069.1496361
SRR17520069.1496363
SRR17520069.1496365
SRR17520069.1496367
SRR17520069.1496371
SRR17520069.1496373
SRR17520069.1496377
SRR17520069.1496379
SRR17520069.1496381
SRR17520069.1496385
SRR17520069.1496387
SRR17520069.1496389
SRR17520069.1496391
SRR17520069.1496393
SRR17520069.1496395
SRR17520069.1496397
SRR17520069.1496399
SRR17520069.1496401
SRR17520069.1496403
SRR17520069.1496405
SRR17520069.1496407
SRR17520069.1496409
SRR17520069.1498734
SRR17520069.1500503
SRR17520069.1500505
SRR17520069.1500507
SRR17520069.1500509
SRR17520069.1500511
SRR17520069.1500513
SRR17520069.1500515
SRR17520069.1500519
SRR17520069.1503306
SRR17520069.1503308
SRR17520069.1503310
SRR17520069.1505158
SRR17520069.1505160
SRR17520069.1505162
SRR17520069.1505166
SRR17520069.1505168
SRR17520069.1505170

SRR17520069.1505172
SRR17520069.1505174
SRR17520069.1505176
SRR17520069.1505178
SRR17520069.1505180
SRR17520069.1505182
SRR17520069.1505184
SRR17520069.1505186
SRR17520069.1505188
SRR17520069.1505190
SRR17520069.1505192
SRR17520069.1505194
SRR17520069.1505196
SRR17520069.1505198
SRR17520069.1505200
SRR17520069.1505872
SRR17520069.1505874
SRR17520069.1505876
SRR17520069.1505878
SRR17520069.1505880
SRR17520069.1505882
SRR17520069.1505884
SRR17520069.1505886
SRR17520069.1505888
SRR17520069.1505890
SRR17520069.1505892
SRR17520069.1505894
SRR17520069.1505896
SRR17520069.1505898
SRR17520069.1505900
SRR17520069.1505902
SRR17520069.1505906
SRR17520069.1505908
SRR17520069.1505910
SRR17520069.1505912
SRR17520069.1505914
SRR17520069.1505916
SRR17520069.1505918
SRR17520069.1505920
SRR17520069.1505922
SRR17520069.1505924
SRR17520069.1505926
SRR17520069.1505928
SRR17520069.1505930
SRR17520069.1505932
SRR17520069.1505934
SRR17520069.1505936

SRR17520069.1505938
SRR17520069.1505940
SRR17520069.1505942
SRR17520069.1505944
SRR17520069.1505946
SRR17520069.1505948
SRR17520069.1505950
SRR17520069.1505952
SRR17520069.1505954
SRR17520069.1505956
SRR17520069.1505960
SRR17520069.1505962
SRR17520069.1505964
SRR17520069.1505966
SRR17520069.1505968
SRR17520069.1505970
SRR17520069.1505972
SRR17520069.1505974
SRR17520069.1505976
SRR17520069.1505978
SRR17520069.1505982
SRR17520069.1505984
SRR17520069.1505986
SRR17520069.1505990
SRR17520069.1505992
SRR17520069.1505994
SRR17520069.1505996
SRR17520069.1505998
SRR17520069.1506000
SRR17520069.1506002
SRR17520069.1506004
SRR17520069.1506006
SRR17520069.1506008
SRR17520069.1506010
SRR17520069.1506012
SRR17520069.1506014
SRR17520069.1506016
SRR17520069.1506022
SRR17520069.1506024
SRR17520069.1506026
SRR17520069.1506903
SRR17520069.1506905
SRR17520069.1506907
SRR17520069.1508022
SRR17520069.1508024
SRR17520069.1508026
SRR17520069.1508028

SRR17520069.1508030
SRR17520069.1508032
SRR17520069.1508034
SRR17520069.1508036
SRR17520069.1508038
SRR17520069.1508040
SRR17520069.1508042
SRR17520069.1508044
SRR17520069.1508046
SRR17520069.1508048
SRR17520069.1508050
SRR17520069.1508052
SRR17520069.1508054
SRR17520069.1508056
SRR17520069.1508058
SRR17520069.1508060
SRR17520069.1508062
SRR17520069.1508064
SRR17520069.1508066
SRR17520069.1508068
SRR17520069.1508070
SRR17520069.1508072
SRR17520069.1508074
SRR17520069.1516438
SRR17520069.1516440
SRR17520069.1516442
SRR17520069.1516444
SRR17520069.1516446
SRR17520069.1516448
SRR17520069.1516450
SRR17520069.1516452
SRR17520069.1516454
SRR17520069.1516456
SRR17520069.1516458
SRR17520069.1516460
SRR17520069.1516462
SRR17520069.1516464
SRR17520069.1516466
SRR17520069.1516468
SRR17520069.1516470
SRR17520069.1516472
SRR17520069.1516474
SRR17520069.1516476
SRR17520069.1516478
SRR17520069.1516480
SRR17520069.1516482
SRR17520069.1516484

SRR17520069.1516486
SRR17520069.1516488
SRR17520069.1516490
SRR17520069.1516492
SRR17520069.1516494
SRR17520069.1516496
SRR17520069.1516498
SRR17520069.1516500
SRR17520069.1516502
SRR17520069.1516504
SRR17520069.1516506
SRR17520069.1516508
SRR17520069.1516510
SRR17520069.1516512
SRR17520069.1516514
SRR17520069.1516516
SRR17520069.1517149
SRR17520069.1517151
SRR17520069.1517153
SRR17520069.1517155
SRR17520069.1517157
SRR17520069.1517159
SRR17520069.1517161
SRR17520069.1517163
SRR17520069.1517165
SRR17520069.1517167
SRR17520069.1517169
SRR17520069.1517171
SRR17520069.1517173
SRR17520069.1517177
SRR17520069.1517179
SRR17520069.1517181
SRR17520069.1517183
SRR17520069.1517185
SRR17520069.1518837
SRR17520069.1518841
SRR17520069.1518845
SRR17520069.1518847
SRR17520069.1518849
SRR17520069.1518851
SRR17520069.1518853
SRR17520069.1518855
SRR17520069.1518857
SRR17520069.1518859
SRR17520069.1518861
SRR17520069.1518863
SRR17520069.1518865

SRR17520069.1518867
SRR17520069.1518869
SRR17520069.1518871
SRR17520069.1518873
SRR17520069.1518875
SRR17520069.1518879
SRR17520069.1518881
SRR17520069.1518883
SRR17520069.1518885
SRR17520069.1518887
SRR17520069.1518889
SRR17520069.1518891
SRR17520069.1518893
SRR17520069.1518895
SRR17520069.1518897
SRR17520069.1518899
SRR17520069.1518901
SRR17520069.1518903
SRR17520069.1518905
SRR17520069.1518907
SRR17520069.1518909
SRR17520069.1518911
SRR17520069.1518913
SRR17520069.1518915
SRR17520069.1518917
SRR17520069.1518919
SRR17520069.1518921
SRR17520069.1518923
SRR17520069.1518925
SRR17520069.1518927
SRR17520069.1518929
SRR17520069.1518931
SRR17520069.1518933
SRR17520069.1518935
SRR17520069.1518937
SRR17520069.1518939
SRR17520069.1518941
SRR17520069.1518943
SRR17520069.1521109
SRR17520069.1521111
SRR17520069.1521113
SRR17520069.1521115
SRR17520069.1521117
SRR17520069.1521119
SRR17520069.1521121
SRR17520069.1521123
SRR17520069.1521125

SRR17520069.1521127
SRR17520069.1521129
SRR17520069.1521131
SRR17520069.1521133
SRR17520069.1521135
SRR17520069.1521137
SRR17520069.1521139
SRR17520069.1521141
SRR17520069.1521143
SRR17520069.1521145
SRR17520069.1521149
SRR17520069.1521151
SRR17520069.1521153
SRR17520069.1521155
SRR17520069.1521157
SRR17520069.1521159
SRR17520069.1521161
SRR17520069.1521163
SRR17520069.1521165
SRR17520069.1521167
SRR17520069.1521169
SRR17520069.1521171
SRR17520069.1522093
SRR17520069.1533082
SRR17520069.1533084
SRR17520069.1533086
SRR17520069.1533088
SRR17520069.1533090
SRR17520069.1533092
SRR17520069.1533094
SRR17520069.1533096
SRR17520069.1533098
SRR17520069.1535084
SRR17520069.1535772
SRR17520069.1535774
SRR17520069.1535776
SRR17520069.1535778
SRR17520069.1535780
SRR17520069.1535782
SRR17520069.1540096
SRR17520069.1540098
SRR17520069.1540100
SRR17520069.1542235
SRR17520069.1542237
SRR17520069.1542239
SRR17520069.1542241
SRR17520069.1542243

SRR17520069.1542245
SRR17520069.1542247
SRR17520069.1551277
SRR17520069.1554087
SRR17520069.1556179
SRR17520069.1556183
SRR17520069.1556185
SRR17520069.1556187
SRR17520069.1556189
SRR17520069.1556191
SRR17520069.1556193
SRR17520069.1556195
SRR17520069.1556199
SRR17520069.1556201
SRR17520069.1556203
SRR17520069.1556205
SRR17520069.1566135
SRR17520069.1566137
SRR17520069.1566139
SRR17520069.1566141
SRR17520069.1566143
SRR17520069.1566145
SRR17520069.1566149
SRR17520069.1566151
SRR17520069.1566153
SRR17520069.1566155
SRR17520069.1572749
SRR17520069.1572751
SRR17520069.1572753
SRR17520069.1572755
SRR17520069.1572757
SRR17520069.1572759
SRR17520069.1572763
SRR17520069.1572765
SRR17520069.1572769
SRR17520069.1572771
SRR17520069.1572773
SRR17520069.1572775
SRR17520069.1572777
SRR17520069.1572779
SRR17520069.1572783
SRR17520069.1572785
SRR17520069.1572787
SRR17520069.1572789
SRR17520069.1572791
SRR17520069.1572793
SRR17520069.1572795

SRR17520069.1572797
SRR17520069.1572799
SRR17520069.1572801
SRR17520069.1572803
SRR17520069.1572805
SRR17520069.1572807
SRR17520069.1572809
SRR17520069.1572813
SRR17520069.1575703
SRR17520069.1578059
SRR17520069.1578061
SRR17520069.1578063
SRR17520069.1578065
SRR17520069.1578067
SRR17520069.1578069
SRR17520069.1578071
SRR17520069.1578075
SRR17520069.1578077
SRR17520069.1578079
SRR17520069.1578083
SRR17520069.1578085
SRR17520069.1578087
SRR17520069.1578089
SRR17520069.1578091
SRR17520069.1578093
SRR17520069.1578095
SRR17520069.1578097
SRR17520069.1578099
SRR17520069.1578101
SRR17520069.1578103
SRR17520069.1578251
SRR17520069.1578253
SRR17520069.1578255
SRR17520069.1578257
SRR17520069.1578259
SRR17520069.1578261
SRR17520069.1578263
SRR17520069.1578265
SRR17520069.1578267
SRR17520069.1578269
SRR17520069.1578271
SRR17520069.1578273
SRR17520069.1578275
SRR17520069.1578277
SRR17520069.1578279
SRR17520069.1578281
SRR17520069.1578283

SRR17520069.1578285
SRR17520069.1578287
SRR17520069.1578289
SRR17520069.1585398
SRR17520069.1585402
SRR17520069.1589942
SRR17520069.1589944
SRR17520069.1589946
SRR17520069.1589948
SRR17520069.1589950
SRR17520069.1589952
SRR17520069.1589954
SRR17520069.1589956
SRR17520069.1589958
SRR17520069.1589960
SRR17520069.1589962
SRR17520069.1592648
SRR17520069.1592650
SRR17520069.1592652
SRR17520069.1592654
SRR17520069.1592656
SRR17520069.1592658
SRR17520069.1592660
SRR17520069.1592662
SRR17520069.1592664
SRR17520069.1592666
SRR17520069.1596619
SRR17520069.1596621
SRR17520069.1596623
SRR17520069.1596625
SRR17520069.1596627
SRR17520069.1596629
SRR17520069.1596631
SRR17520069.1596633
SRR17520069.1596635
SRR17520069.1596637
SRR17520069.1596639
SRR17520069.1596641
SRR17520069.1596643
SRR17520069.1596645
SRR17520069.1596647
SRR17520069.1596651
SRR17520069.1596653
SRR17520069.1596655
SRR17520069.1596657
SRR17520069.1596659
SRR17520069.1596667

SRR17520069.1596669
SRR17520069.1596671
SRR17520069.1596673
SRR17520069.1608006
SRR17520069.1608008
SRR17520069.1608010
SRR17520069.1608012
SRR17520069.1608014
SRR17520069.1608016
SRR17520069.1608018
SRR17520069.1608020
SRR17520069.1608022
SRR17520069.1608024
SRR17520069.1608026
SRR17520069.1608028
SRR17520069.1608030
SRR17520069.1608032
SRR17520069.1608034
SRR17520069.1608036
SRR17520069.1608038
SRR17520069.1608040
SRR17520069.1608042
SRR17520069.1608044
SRR17520069.1608046
SRR17520069.1608048
SRR17520069.1608050
SRR17520069.1608052
SRR17520069.1608054
SRR17520069.1608058
SRR17520069.1608060
SRR17520069.1608062
SRR17520069.1608064
SRR17520069.1608066
SRR17520069.1608068
SRR17520069.1608070
SRR17520069.1608072
SRR17520069.1608074
SRR17520069.1608076
SRR17520069.1608078
SRR17520069.1608080
SRR17520069.1608082
SRR17520069.1608084
SRR17520069.1608086
SRR17520069.1608088
SRR17520069.1608090
SRR17520069.1608092
SRR17520069.1608094

SRR17520069.1608096
SRR17520069.1608102
SRR17520069.1608110
SRR17520069.1608112
SRR17520069.1608114
SRR17520069.1608116
SRR17520069.1608118
SRR17520069.1608120
SRR17520069.1608122
SRR17520069.1608124
SRR17520069.1608130
SRR17520069.1608132
SRR17520069.1608136
SRR17520069.1608138
SRR17520069.1608142
SRR17520069.1608144
SRR17520069.1608146
SRR17520069.1608148
SRR17520069.1608150
SRR17520069.1608152
SRR17520069.1608154
SRR17520069.1608156
SRR17520069.1608158
SRR17520069.1608160
SRR17520069.1608162
SRR17520069.1608164
SRR17520069.1608166
SRR17520069.1608168
SRR17520069.1608172
SRR17520069.1608174
SRR17520069.1608176
SRR17520069.1608178
SRR17520069.1608180
SRR17520069.1608182
SRR17520069.1611249
SRR17520069.1614296
SRR17520069.1614298
SRR17520069.1623656
SRR17520069.1631635
SRR17520069.1631637
SRR17520069.1631639
SRR17520069.1631641
SRR17520069.1631643
SRR17520069.1631645
SRR17520069.1631647
SRR17520069.1631649
SRR17520069.1631651

SRR17520069.1631655
SRR17520069.1631657
SRR17520069.1631659
SRR17520069.1631661
SRR17520069.1631663
SRR17520069.1631665
SRR17520069.1631667
SRR17520069.1631669
SRR17520069.1631671
SRR17520069.1631673
SRR17520069.1631675
SRR17520069.1631677
SRR17520069.1631679
SRR17520069.1631681
SRR17520069.1631683
SRR17520069.1631685
SRR17520069.1631687
SRR17520069.1631689
SRR17520069.1631691
SRR17520069.1631693
SRR17520069.1631695
SRR17520069.1631697
SRR17520069.1631699
SRR17520069.1631701
SRR17520069.1631703
SRR17520069.1631705
SRR17520069.1635995
SRR17520069.1640966
SRR17520069.1640968
SRR17520069.1640970
SRR17520069.1640972
SRR17520069.1640974
SRR17520069.1640976
SRR17520069.1640978
SRR17520069.1640980
SRR17520069.1642993
SRR17520069.1642995
SRR17520069.1642997
SRR17520069.1642999
SRR17520069.1643001
SRR17520069.1643003
SRR17520069.1643005
SRR17520069.1643007
SRR17520069.1643009
SRR17520069.1643011
SRR17520069.1643013
SRR17520069.1643015

SRR17520069.1643017
SRR17520069.1643019
SRR17520069.1643021
SRR17520069.1643023
SRR17520069.1643025
SRR17520069.1643027
SRR17520069.1643029
SRR17520069.1643031
SRR17520069.1643033
SRR17520069.1643035
SRR17520069.1643037
SRR17520069.1643039
SRR17520069.1650478
SRR17520069.1650482
SRR17520069.1650484
SRR17520069.1650486
SRR17520069.1650488
SRR17520069.1650490
SRR17520069.1650492
SRR17520069.1650494
SRR17520069.1650496
SRR17520069.1650498
SRR17520069.1650500
SRR17520069.1650502
SRR17520069.1650504
SRR17520069.1650506
SRR17520069.1650508
SRR17520069.1650510
SRR17520069.1650512
SRR17520069.1650514
SRR17520069.1650516
SRR17520069.1650518
SRR17520069.1650520
SRR17520069.1650522
SRR17520069.1650524
SRR17520069.1650526
SRR17520069.1650528
SRR17520069.1650530
SRR17520069.1650532
SRR17520069.1650534
SRR17520069.1650536
SRR17520069.1650538
SRR17520069.1650540
SRR17520069.1650542
SRR17520069.1650544
SRR17520069.1650546
SRR17520069.1650550

SRR17520069.1650552
SRR17520069.1650554
SRR17520069.1650556
SRR17520069.1650558
SRR17520069.1650560
SRR17520069.1650562
SRR17520069.1650564
SRR17520069.1650566
SRR17520069.1650568
SRR17520069.1650570
SRR17520069.1650572
SRR17520069.1650574
SRR17520069.1650576
SRR17520069.1650578
SRR17520069.1650580
SRR17520069.1650582
SRR17520069.1650584
SRR17520069.1650586
SRR17520069.1650588
SRR17520069.1650590
SRR17520069.1650592
SRR17520069.1650594
SRR17520069.1650596
SRR17520069.1650598
SRR17520069.1650600
SRR17520069.1650602
SRR17520069.1650604
SRR17520069.1650606
SRR17520069.1656129
SRR17520069.1656131
SRR17520069.1656133
SRR17520069.1656135
SRR17520069.1656141
SRR17520069.1656145
SRR17520069.1656147
SRR17520069.1656149
SRR17520069.1656151
SRR17520069.1656153
SRR17520069.1656157
SRR17520069.1656159
SRR17520069.1656161
SRR17520069.1656163
SRR17520069.1656165
SRR17520069.1656167
SRR17520069.1656169
SRR17520069.1656171
SRR17520069.1656173

SRR17520069.1656175
SRR17520069.1659465
SRR17520069.1659467
SRR17520069.1660906
SRR17520069.1660908
SRR17520069.1660910
SRR17520069.1660912
SRR17520069.1660914
SRR17520069.1660916
SRR17520069.1660918
SRR17520069.1660920
SRR17520069.1660922
SRR17520069.1660924
SRR17520069.1660926
SRR17520069.1660928
SRR17520069.1660930
SRR17520069.1660932
SRR17520069.1660934
SRR17520069.1660936
SRR17520069.1660938
SRR17520069.1660940
SRR17520069.1660942
SRR17520069.1660944
SRR17520069.1660946
SRR17520069.1660948
SRR17520069.1660950
SRR17520069.1660952
SRR17520069.1660954
SRR17520069.1660956
SRR17520069.1660958
SRR17520069.1660960
SRR17520069.1663298
SRR17520069.1663911
SRR17520069.1663913
SRR17520069.1663915
SRR17520069.1663917
SRR17520069.1663919
SRR17520069.1663921
SRR17520069.1663923
SRR17520069.1663925
SRR17520069.1663927
SRR17520069.1663929
SRR17520069.1663931
SRR17520069.1663933
SRR17520069.1663935
SRR17520069.1663937
SRR17520069.1663939

SRR17520069.1663941
SRR17520069.1663943
SRR17520069.1663945
SRR17520069.1663947
SRR17520069.1663949
SRR17520069.1663951
SRR17520069.1663953
SRR17520069.1663955
SRR17520069.1663957
SRR17520069.1663959
SRR17520069.1663961
SRR17520069.1663963
SRR17520069.1663965
SRR17520069.1663967
SRR17520069.1663969
SRR17520069.1663971
SRR17520069.1663973
SRR17520069.1663975
SRR17520069.1663977
SRR17520069.1663979
SRR17520069.1663981
SRR17520069.1663983
SRR17520069.1663985
SRR17520069.1663987
SRR17520069.1663989
SRR17520069.1663991
SRR17520069.1663993
SRR17520069.1663995
SRR17520069.1663997
SRR17520069.1663999
SRR17520069.1664001
SRR17520069.1664003
SRR17520069.1664005
SRR17520069.1664007
SRR17520069.1664009
SRR17520069.1664011
SRR17520069.1664013
SRR17520069.1664015
SRR17520069.1664017
SRR17520069.1664019
SRR17520069.1664021
SRR17520069.1664023
SRR17520069.1664813
SRR17520069.1664815
SRR17520069.1664817
SRR17520069.1664819
SRR17520069.1664821

SRR17520069.1664823
SRR17520069.1664825
SRR17520069.1664827
SRR17520069.1664831
SRR17520069.1664833
SRR17520069.1664835
SRR17520069.1664837
SRR17520069.1664839
SRR17520069.1667930
SRR17520069.1667932
SRR17520069.1667934
SRR17520069.1667936
SRR17520069.1667938
SRR17520069.1667940
SRR17520069.1667942
SRR17520069.1667944
SRR17520069.1667946
SRR17520069.1667948
SRR17520069.1667950
SRR17520069.1667952
SRR17520069.1667954
SRR17520069.1667958
SRR17520069.1667960
SRR17520069.1667962
SRR17520069.1667964
SRR17520069.1667966
SRR17520069.1667968
SRR17520069.1667970
SRR17520069.1667974
SRR17520069.1667976
SRR17520069.1667978
SRR17520069.1667980
SRR17520069.1667982
SRR17520069.1667984
SRR17520069.1667986
SRR17520069.1667988
SRR17520069.1667990
SRR17520069.1667992
SRR17520069.1667994
SRR17520069.1667996
SRR17520069.1668000
SRR17520069.1668002
SRR17520069.1668004
SRR17520069.1668006
SRR17520069.1668008
SRR17520069.1668010
SRR17520069.1668012

SRR17520069.1668014
SRR17520069.1668016
SRR17520069.1671351
SRR17520069.1671353
SRR17520069.1671355
SRR17520069.1671357
SRR17520069.1671359
SRR17520069.1671361
SRR17520069.1671363
SRR17520069.1671365
SRR17520069.1671367
SRR17520069.1671369
SRR17520069.1671371
SRR17520069.1671373
SRR17520069.1671375
SRR17520069.1671377
SRR17520069.1671379
SRR17520069.1671381
SRR17520069.1671383
SRR17520069.1671385
SRR17520069.1671387
SRR17520069.1671389
SRR17520069.1671391
SRR17520069.1671393
SRR17520069.1671395
SRR17520069.1672190
SRR17520069.1672192
SRR17520069.1672194
SRR17520069.1672196
SRR17520069.1672941
SRR17520069.1672943
SRR17520069.1672945
SRR17520069.1672947
SRR17520069.1672949
SRR17520069.1672951
SRR17520069.1672953
SRR17520069.1672955
SRR17520069.1672957
SRR17520069.1674642
SRR17520069.1674644
SRR17520069.1674646
SRR17520069.1674648
SRR17520069.1674650
SRR17520069.1674652
SRR17520069.1674654
SRR17520069.1674656
SRR17520069.1674658

SRR17520069.1674660
SRR17520069.1674662
SRR17520069.1674664
SRR17520069.1674666
SRR17520069.1674668
SRR17520069.1674670
SRR17520069.1674672
SRR17520069.1674674
SRR17520069.1674676
SRR17520069.1674678
SRR17520069.1674680
SRR17520069.1674682
SRR17520069.1674684
SRR17520069.1674686
SRR17520069.1674688
SRR17520069.1674690
SRR17520069.1678502
SRR17520069.1678504
SRR17520069.1678506
SRR17520069.1678508
SRR17520069.1678510
SRR17520069.1678514
SRR17520069.1678518
SRR17520069.1678520
SRR17520069.1678522
SRR17520069.1678524
SRR17520069.1678997
SRR17520069.1678999
SRR17520069.1679001
SRR17520069.1679003
SRR17520069.1679005
SRR17520069.1679007
SRR17520069.1679009
SRR17520069.1679011
SRR17520069.1679013
SRR17520069.1679015
SRR17520069.1679017
SRR17520069.1679019
SRR17520069.1679021
SRR17520069.1679023
SRR17520069.1679025
SRR17520069.1679027
SRR17520069.1679029
SRR17520069.1679031
SRR17520069.1679033
SRR17520069.1679035
SRR17520069.1679037

SRR17520069.1679039
SRR17520069.1679043
SRR17520069.1679045
SRR17520069.1679047
SRR17520069.1679049
SRR17520069.1679051
SRR17520069.1679053
SRR17520069.1679055
SRR17520069.1679057
SRR17520069.1679059
SRR17520069.1679061
SRR17520069.1679063
SRR17520069.1679065
SRR17520069.1679067
SRR17520069.1679069
SRR17520069.1679071
SRR17520069.1679073
SRR17520069.1679075
SRR17520069.1679077
SRR17520069.1679079
SRR17520069.1679081
SRR17520069.1679083
SRR17520069.1679085
SRR17520069.1679087
SRR17520069.1679089
SRR17520069.1679091
SRR17520069.1679093
SRR17520069.1679095
SRR17520069.1680826
SRR17520069.1680828
SRR17520069.1680830
SRR17520069.1680832
SRR17520069.1680836
SRR17520069.1680838
SRR17520069.1680840
SRR17520069.1680842
SRR17520069.1680844
SRR17520069.1680846
SRR17520069.1680848
SRR17520069.1680850
SRR17520069.1680852
SRR17520069.1680854
SRR17520069.1680856
SRR17520069.1680858
SRR17520069.1680860
SRR17520069.1680862
SRR17520069.1680864

SRR17520069.1680866
SRR17520069.1680868
SRR17520069.1680870
SRR17520069.1680872
SRR17520069.1680874
SRR17520069.1680876
SRR17520069.1680878
SRR17520069.1680880
SRR17520069.1680882
SRR17520069.1680884
SRR17520069.1680886
SRR17520069.1680888
SRR17520069.1680890
SRR17520069.1680892
SRR17520069.1680894
SRR17520069.1686217
SRR17520069.1686219
SRR17520069.1686221
SRR17520069.1686225
SRR17520069.1686227
SRR17520069.1686573
SRR17520069.1686575
SRR17520069.1686577
SRR17520069.1690326
SRR17520069.1690328
SRR17520069.1690330
SRR17520069.1690332
SRR17520069.1690334
SRR17520069.1690336
SRR17520069.1690338
SRR17520069.1690340
SRR17520069.1690342
SRR17520069.1690344
SRR17520069.1690346
SRR17520069.1690348
SRR17520069.1690350
SRR17520069.1700483
SRR17520069.1700485
SRR17520069.1700487
SRR17520069.1700489
SRR17520069.1700491
SRR17520069.1700493
SRR17520069.1700495
SRR17520069.1700497
SRR17520069.1702400
SRR17520069.1702402
SRR17520069.1702404

SRR17520069.1702406
SRR17520069.1702408
SRR17520069.1702410
SRR17520069.1702412
SRR17520069.1702414
SRR17520069.1702416
SRR17520069.1702418
SRR17520069.1702420
SRR17520069.1704797
SRR17520069.1704799
SRR17520069.1704801
SRR17520069.1704803
SRR17520069.1704805
SRR17520069.1704807
SRR17520069.1704809
SRR17520069.1704811
SRR17520069.1704813
SRR17520069.1704815
SRR17520069.1704817
SRR17520069.1704819
SRR17520069.1704821
SRR17520069.1704823
SRR17520069.1704827
SRR17520069.1704829
SRR17520069.1704831
SRR17520069.1704833
SRR17520069.1705725
SRR17520069.1705727
SRR17520069.1705729
SRR17520069.1705731
SRR17520069.1705733
SRR17520069.1705735
SRR17520069.1705737
SRR17520069.1705739
SRR17520069.1705741
SRR17520069.1705743
SRR17520069.1709418
SRR17520069.1709420
SRR17520069.1709422
SRR17520069.1709424
SRR17520069.1709426
SRR17520069.1709428
SRR17520069.1709430
SRR17520069.1709432
SRR17520069.1709434
SRR17520069.1709436
SRR17520069.1709438

SRR17520069.1709440
SRR17520069.1709442
SRR17520069.1709444
SRR17520069.1709446
SRR17520069.1709448
SRR17520069.1709450
SRR17520069.1709452
SRR17520069.1709454
SRR17520069.1709456
SRR17520069.1709458
SRR17520069.1709460
SRR17520069.1709462
SRR17520069.1709464
SRR17520069.1716221
SRR17520069.1716223
SRR17520069.1716225
SRR17520069.1716227
SRR17520069.1716229
SRR17520069.1716231
SRR17520069.1716233
SRR17520069.1716235
SRR17520069.1716237
SRR17520069.1716239
SRR17520069.1716241
SRR17520069.1716243
SRR17520069.1716245
SRR17520069.1718504
SRR17520069.1718506
SRR17520069.1718508
SRR17520069.1718510
SRR17520069.1718512
SRR17520069.1718514
SRR17520069.1718516
SRR17520069.1718518
SRR17520069.1718520
SRR17520069.1718522
SRR17520069.1718524
SRR17520069.1718526
SRR17520069.1718528
SRR17520069.1718530
SRR17520069.1718532
SRR17520069.1718534
SRR17520069.1718536
SRR17520069.1718538
SRR17520069.1718540
SRR17520069.1718542
SRR17520069.1718544

SRR17520069.1718546
SRR17520069.1718548
SRR17520069.1718550
SRR17520069.1718552
SRR17520069.1718554
SRR17520069.1718556
SRR17520069.1726598
SRR17520069.1726600
SRR17520069.1726602
SRR17520069.1726604
SRR17520069.1726606
SRR17520069.1726608
SRR17520069.1726610
SRR17520069.1726612
SRR17520069.1726614
SRR17520069.1726616
SRR17520069.1726618
SRR17520069.1726620
SRR17520069.1726622
SRR17520069.1726624
SRR17520069.1726626
SRR17520069.1726628
SRR17520069.1726630
SRR17520069.1740901
SRR17520069.1740903
SRR17520069.1740905
SRR17520069.1740907
SRR17520069.1750484
SRR17520069.1750486
SRR17520069.1750488
SRR17520069.1750492
SRR17520069.1751719
SRR17520069.1751721
SRR17520069.1751723
SRR17520069.1751725
SRR17520069.1751727
SRR17520069.1751729
SRR17520069.1751731
SRR17520069.1751733
SRR17520069.1751735
SRR17520069.1751737
SRR17520069.1751739
SRR17520069.1751741
SRR17520069.1751743
SRR17520069.1751745
SRR17520069.1751747
SRR17520069.1751749

SRR17520069.1751751
SRR17520069.1751753
SRR17520069.1751755
SRR17520069.1751757
SRR17520069.1751759
SRR17520069.1751761
SRR17520069.1751763
SRR17520069.1751765
SRR17520069.1751767
SRR17520069.1751769
SRR17520069.1751771
SRR17520069.1751773
SRR17520069.1751775
SRR17520069.1752746
SRR17520069.1752748
SRR17520069.1752750
SRR17520069.1752752
SRR17520069.1752754
SRR17520069.1752756
SRR17520069.1752758
SRR17520069.1752760
SRR17520069.1752762
SRR17520069.1752764
SRR17520069.1752766
SRR17520069.1752768
SRR17520069.1753061
SRR17520069.1757738
SRR17520069.1757740
SRR17520069.1757742
SRR17520069.1757744
SRR17520069.1757746
SRR17520069.1759635
SRR17520069.1759639
SRR17520069.1759643
SRR17520069.1759645
SRR17520069.1759649
SRR17520069.1759651
SRR17520069.1759653
SRR17520069.1759655
SRR17520069.1759657
SRR17520069.1759659
SRR17520069.1759661
SRR17520069.1759663
SRR17520069.1759665
SRR17520069.1759669
SRR17520069.1765365
SRR17520069.1766100

SRR17520069.1766102
SRR17520069.1766104
SRR17520069.1766106
SRR17520069.1766108
SRR17520069.1766110
SRR17520069.1766112
SRR17520069.1766114
SRR17520069.1766116
SRR17520069.1766118
SRR17520069.1766120
SRR17520069.1766122
SRR17520069.1768585
SRR17520069.1769632
SRR17520069.1769634
SRR17520069.1769636
SRR17520069.1769638
SRR17520069.1769640
SRR17520069.1769642
SRR17520069.1769644
SRR17520069.1769646
SRR17520069.1769648
SRR17520069.1769650
SRR17520069.1769652
SRR17520069.1769654
SRR17520069.1769656
SRR17520069.1769658
SRR17520069.1769660
SRR17520069.1769662
SRR17520069.1769664
SRR17520069.1769666
SRR17520069.1769668
SRR17520069.1769670
SRR17520069.1769672
SRR17520069.1769674
SRR17520069.1769676
SRR17520069.1769678
SRR17520069.1769680
SRR17520069.1769682
SRR17520069.1769684
SRR17520069.1769686
SRR17520069.1769688
SRR17520069.1769690
SRR17520069.1769692
SRR17520069.1769694
SRR17520069.1769696
SRR17520069.1769698
SRR17520069.1769700

SRR17520069.1769702
SRR17520069.1769704
SRR17520069.1769706
SRR17520069.1769708
SRR17520069.1769710
SRR17520069.1769712
SRR17520069.1769714
SRR17520069.1769716
SRR17520069.1769718
SRR17520069.1769720
SRR17520069.1769722
SRR17520069.1769724
SRR17520069.1769726
SRR17520069.1769728
SRR17520069.1769730
SRR17520069.1769732
SRR17520069.1769734
SRR17520069.1769736
SRR17520069.1769738
SRR17520069.1769740
SRR17520069.1769742
SRR17520069.1769744
SRR17520069.1769746
SRR17520069.1769750
SRR17520069.1770385
SRR17520069.1770387
SRR17520069.1770389
SRR17520069.1770391
SRR17520069.1770393
SRR17520069.1770395
SRR17520069.1770397
SRR17520069.1770399
SRR17520069.1770401
SRR17520069.1770403
SRR17520069.1770405
SRR17520069.1774370
SRR17520069.1774375
SRR17520069.1774377
SRR17520069.1774379
SRR17520069.1774381
SRR17520069.1774383
SRR17520069.1774385
SRR17520069.1774387
SRR17520069.1774389
SRR17520069.1774391
SRR17520069.1774393
SRR17520069.1774397

SRR17520069.1774399
SRR17520069.1788146
SRR17520069.1788148
SRR17520069.1788150
SRR17520069.1788152
SRR17520069.1788154
SRR17520069.1796513
SRR17520069.1796515
SRR17520069.1796519
SRR17520069.1796521
SRR17520069.1796523
SRR17520069.1796527
SRR17520069.1796529
SRR17520069.1796531
SRR17520069.1796533
SRR17520069.1796535
SRR17520069.1796537
SRR17520069.1796539
SRR17520069.1796541
SRR17520069.1796543
SRR17520069.1796545
SRR17520069.1796547
SRR17520069.1796549
SRR17520069.1796551
SRR17520069.1796553
SRR17520069.1796555
SRR17520069.1796557
SRR17520069.1796559
SRR17520069.1796561
SRR17520069.1796563
SRR17520069.1796565
SRR17520069.1796569
SRR17520069.1796571
SRR17520069.1796573
SRR17520069.1796575
SRR17520069.1796577
SRR17520069.1796579
SRR17520069.1796581
SRR17520069.1796583
SRR17520069.1796585
SRR17520069.1796587
SRR17520069.1796589
SRR17520069.1796591
SRR17520069.1796593
SRR17520069.1796597
SRR17520069.1796603
SRR17520069.1796605

SRR17520069.1796607
SRR17520069.1796609
SRR17520069.1796611
SRR17520069.1796615
SRR17520069.1796617
SRR17520069.1796619
SRR17520069.1797812
SRR17520069.1797814
SRR17520069.1797816
SRR17520069.1797818
SRR17520069.1797820
SRR17520069.1797822
SRR17520069.1797824
SRR17520069.1797826
SRR17520069.1797828
SRR17520069.1797830
SRR17520069.1797832
SRR17520069.1797834
SRR17520069.1797836
SRR17520069.1797838
SRR17520069.1799143
SRR17520069.1799145
SRR17520069.1799147
SRR17520069.1799149
SRR17520069.1799151
SRR17520069.1799153
SRR17520069.1799155
SRR17520069.1799157
SRR17520069.1799161
SRR17520069.1799163
SRR17520069.1799165
SRR17520069.1799167
SRR17520069.1799169
SRR17520069.1799171
SRR17520069.1799173
SRR17520069.1799175
SRR17520069.1799177
SRR17520069.1799179
SRR17520069.1799181
SRR17520069.1799183
SRR17520069.1799185
SRR17520069.1799187
SRR17520069.1799189
SRR17520069.1799191
SRR17520069.1799193
SRR17520069.1799195
SRR17520069.1799197

SRR17520069.1799199
SRR17520069.1799201
SRR17520069.1799203
SRR17520069.1799205
SRR17520069.1799207
SRR17520069.1799209
SRR17520069.1799213
SRR17520069.1799215
SRR17520069.1799217
SRR17520069.1799219
SRR17520069.1799221
SRR17520069.1799223
SRR17520069.1799225
SRR17520069.1799227
SRR17520069.1799231
SRR17520069.1799233
SRR17520069.1799235
SRR17520069.1799241
SRR17520069.1799243
SRR17520069.1799245
SRR17520069.1799247
SRR17520069.1799249
SRR17520069.1799251
SRR17520069.1799253
SRR17520069.1799255
SRR17520069.1799257
SRR17520069.1799259
SRR17520069.1799261
SRR17520069.1799263
SRR17520069.1799265
SRR17520069.1799267
SRR17520069.1799271
SRR17520069.1799273
SRR17520069.1799275
SRR17520069.1799277
SRR17520069.1799281
SRR17520069.1799283
SRR17520069.1799285
SRR17520069.1799287
SRR17520069.1799289
SRR17520069.1799291
SRR17520069.1799293
SRR17520069.1799295
SRR17520069.1799297
SRR17520069.1799299
SRR17520069.1799301
SRR17520069.1799303

SRR17520069.1799305
SRR17520069.1799307
SRR17520069.1799309
SRR17520069.1799311
SRR17520069.1799313
SRR17520069.1799315
SRR17520069.1799317
SRR17520069.1799319
SRR17520069.1799321
SRR17520069.1799323
SRR17520069.1799325
SRR17520069.1799329
SRR17520069.1799331
SRR17520069.1799333
SRR17520069.1799335
SRR17520069.1799337
SRR17520069.1799339
SRR17520069.1799341
SRR17520069.1799343
SRR17520069.1799345
SRR17520069.1799347
SRR17520069.1799349
SRR17520069.1804859
SRR17520069.1804861
SRR17520069.1804863
SRR17520069.1804865
SRR17520069.1804867
SRR17520069.1804869
SRR17520069.1804871
SRR17520069.1804873
SRR17520069.1804875
SRR17520069.1804877
SRR17520069.1804879
SRR17520069.1805136
SRR17520069.1805323
SRR17520069.1805325
SRR17520069.1805327
SRR17520069.1805329
SRR17520069.1805331
SRR17520069.1805333
SRR17520069.1805335
SRR17520069.1805337
SRR17520069.1805339
SRR17520069.1807275
SRR17520069.1809680
SRR17520069.1809682
SRR17520069.1810018

SRR17520069.1810020
SRR17520069.1810022
SRR17520069.1810026
SRR17520069.1810028
SRR17520069.1810030
SRR17520069.1810032
SRR17520069.1810184
SRR17520069.1810186
SRR17520069.1810188
SRR17520069.1818449
SRR17520069.1831519
SRR17520069.1831521
SRR17520069.1831523
SRR17520069.1831525
SRR17520069.1831527
SRR17520069.1831529
SRR17520069.1831531
SRR17520069.1831533
SRR17520069.1831535
SRR17520069.1831537
SRR17520069.1831539
SRR17520069.1831541
SRR17520069.1831549
SRR17520069.1831551
SRR17520069.1831553
SRR17520069.1831555
SRR17520069.1831557
SRR17520069.1831559
SRR17520069.1831561
SRR17520069.1831563
SRR17520069.1831565
SRR17520069.1831567
SRR17520069.1831569
SRR17520069.1831571
SRR17520069.1831573
SRR17520069.1831575
SRR17520069.1831577
SRR17520069.1831579
SRR17520069.1831581
SRR17520069.1831583
SRR17520069.1831585
SRR17520069.1831587
SRR17520069.1831591
SRR17520069.1831593
SRR17520069.1831595
SRR17520069.1831670
SRR17520069.1831672

SRR17520069.1831674
SRR17520069.1831676
SRR17520069.1831678
SRR17520069.1831680
SRR17520069.1831684
SRR17520069.1831686
SRR17520069.1831688
SRR17520069.1831690
SRR17520069.1831692
SRR17520069.1831694
SRR17520069.1831696
SRR17520069.1831698
SRR17520069.1831700
SRR17520069.1831702
SRR17520069.1831704
SRR17520069.1831706
SRR17520069.1831708
SRR17520069.1831710
SRR17520069.1831712
SRR17520069.1831714
SRR17520069.1831716
SRR17520069.1831718
SRR17520069.1833296
SRR17520069.1833298
SRR17520069.1833302
SRR17520069.1833304
SRR17520069.1833306
SRR17520069.1833308
SRR17520069.1833310
SRR17520069.1833312
SRR17520069.1833314
SRR17520069.1833316
SRR17520069.1833318
SRR17520069.1833320
SRR17520069.1833324
SRR17520069.1833326
SRR17520069.1833330
SRR17520069.1833332
SRR17520069.1833334
SRR17520069.1833338
SRR17520069.1833340
SRR17520069.1833342
SRR17520069.1833346
SRR17520069.1833348
SRR17520069.1833350
SRR17520069.1833354
SRR17520069.1833358

SRR17520069.1833360
SRR17520069.1833362
SRR17520069.1833364
SRR17520069.1833366
SRR17520069.1833368
SRR17520069.1833370
SRR17520069.1833372
SRR17520069.1833374
SRR17520069.1833378
SRR17520069.1833380
SRR17520069.1833382
SRR17520069.1833384
SRR17520069.1833386
SRR17520069.1833390
SRR17520069.1833392
SRR17520069.1833394
SRR17520069.1833396
SRR17520069.1833402
SRR17520069.1833404
SRR17520069.1833406
SRR17520069.1833408
SRR17520069.1833412
SRR17520069.1833414
SRR17520069.1833416
SRR17520069.1833418
SRR17520069.1833420
SRR17520069.1833422
SRR17520069.1833424
SRR17520069.1833426
SRR17520069.1833428
SRR17520069.1833430
SRR17520069.1833432
SRR17520069.1833434
SRR17520069.1833436
SRR17520069.1833438
SRR17520069.1833440
SRR17520069.1833442
SRR17520069.1833444
SRR17520069.1833446
SRR17520069.1833450
SRR17520069.1833454
SRR17520069.1833457
SRR17520069.1833462
SRR17520069.1833464
SRR17520069.1833468
SRR17520069.1841480
SRR17520069.1841482

SRR17520069.1841486
SRR17520069.1841488
SRR17520069.1841490
SRR17520069.1841492
SRR17520069.1841494
SRR17520069.1841984
SRR17520069.1841986
SRR17520069.1841988
SRR17520069.1841990
SRR17520069.1841992
SRR17520069.1841994
SRR17520069.1841996
SRR17520069.1841998
SRR17520069.1842000
SRR17520069.1842002
SRR17520069.1845663
SRR17520069.1845665
SRR17520069.1845667
SRR17520069.1845669
SRR17520069.1845671
SRR17520069.1845673
SRR17520069.1845675
SRR17520069.1845677
SRR17520069.1845679
SRR17520069.1845681
SRR17520069.1845683
SRR17520069.1845685
SRR17520069.1845687
SRR17520069.1847354
SRR17520069.1847356
SRR17520069.1847358
SRR17520069.1847360
SRR17520069.1847362
SRR17520069.1847364
SRR17520069.1847366
SRR17520069.1847368
SRR17520069.1847370
SRR17520069.1847372
SRR17520069.1847374
SRR17520069.1847376
SRR17520069.1847378
SRR17520069.1847380
SRR17520069.1847382
SRR17520069.1847384
SRR17520069.1847388
SRR17520069.1847390
SRR17520069.1847392

SRR17520069.1847394
SRR17520069.1847396
SRR17520069.1847398
SRR17520069.1847400
SRR17520069.1847402
SRR17520069.1847404
SRR17520069.1847406
SRR17520069.1847408
SRR17520069.1847410
SRR17520069.1848461
SRR17520069.1848463
SRR17520069.1848465
SRR17520069.1848467
SRR17520069.1848469
SRR17520069.1848471
SRR17520069.1848473
SRR17520069.1848475
SRR17520069.1848477
SRR17520069.1848479
SRR17520069.1848481
SRR17520069.1848483
SRR17520069.1848485
SRR17520069.1848487
SRR17520069.1848489
SRR17520069.1848491
SRR17520069.1848493
SRR17520069.1848495
SRR17520069.1848497
SRR17520069.1848499
SRR17520069.1848501
SRR17520069.1848503
SRR17520069.1848505
SRR17520069.1848507
SRR17520069.1848509
SRR17520069.1848511
SRR17520069.1848513
SRR17520069.1848515
SRR17520069.1848517
SRR17520069.1848519
SRR17520069.1848521
SRR17520069.1848523
SRR17520069.1848525
SRR17520069.1848527
SRR17520069.1848529
SRR17520069.1848531
SRR17520069.1848533
SRR17520069.1848535

SRR17520069.1848537
SRR17520069.1848539
SRR17520069.1848541
SRR17520069.1848543
SRR17520069.1848545
SRR17520069.1848547
SRR17520069.1848549
SRR17520069.1848551
SRR17520069.1848553
SRR17520069.1848555
SRR17520069.1848557
SRR17520069.1848559
SRR17520069.1848561
SRR17520069.1848563
SRR17520069.1848565
SRR17520069.1848567
SRR17520069.1848569
SRR17520069.1848571
SRR17520069.1848573
SRR17520069.1848575
SRR17520069.1848577
SRR17520069.1848579
SRR17520069.1848581
SRR17520069.1848583
SRR17520069.1848585
SRR17520069.1848587
SRR17520069.1848590
SRR17520069.1848592
SRR17520069.1848594
SRR17520069.1848596
SRR17520069.1848598
SRR17520069.1848600
SRR17520069.1848602
SRR17520069.1848604
SRR17520069.1848606
SRR17520069.1848608
SRR17520069.1848610
SRR17520069.1848612
SRR17520069.1848614
SRR17520069.1848616
SRR17520069.1848618
SRR17520069.1848620
SRR17520069.1848622
SRR17520069.1848624
SRR17520069.1848626
SRR17520069.1848628
SRR17520069.1848630

SRR17520069.1849585
SRR17520069.1849587
SRR17520069.1849589
SRR17520069.1854318
SRR17520069.1854320
SRR17520069.1854322
SRR17520069.1854324
SRR17520069.1854326
SRR17520069.1854328
SRR17520069.1854330
SRR17520069.1854332
SRR17520069.1854334
SRR17520069.1854336
SRR17520069.1854338
SRR17520069.1854340
SRR17520069.1854342
SRR17520069.1854344
SRR17520069.1854346
SRR17520069.1854348
SRR17520069.1854350
SRR17520069.1854352
SRR17520069.1854354
SRR17520069.1854356
SRR17520069.1854358
SRR17520069.1854360
SRR17520069.1854362
SRR17520069.1854364
SRR17520069.1854366
SRR17520069.1854368
SRR17520069.1854370
SRR17520069.1854372
SRR17520069.1854374
SRR17520069.1854376
SRR17520069.1857592
SRR17520069.1857594
SRR17520069.1857596
SRR17520069.1857598
SRR17520069.1857600
SRR17520069.1857602
SRR17520069.1857604
SRR17520069.1857606
SRR17520069.1860743
SRR17520069.1860745
SRR17520069.1860747
SRR17520069.1860749
SRR17520069.1860751
SRR17520069.1860753

SRR17520069.1860755
SRR17520069.1860757
SRR17520069.1860759
SRR17520069.1860761
SRR17520069.1860763
SRR17520069.1860765
SRR17520069.1860767
SRR17520069.1860769
SRR17520069.1860771
SRR17520069.1860773
SRR17520069.1860775
SRR17520069.1860777
SRR17520069.1860779
SRR17520069.1860781
SRR17520069.1860783
SRR17520069.1860785
SRR17520069.1860787
SRR17520069.1860789
SRR17520069.1860791
SRR17520069.1860793
SRR17520069.1860795
SRR17520069.1860797
SRR17520069.1860799
SRR17520069.1860801
SRR17520069.1860803
SRR17520069.1860805
SRR17520069.1860807
SRR17520069.1860809
SRR17520069.1860811
SRR17520069.1860815
SRR17520069.1860817
SRR17520069.1860819
SRR17520069.1860821
SRR17520069.1860823
SRR17520069.1860825
SRR17520069.1860827
SRR17520069.1860829
SRR17520069.1866388
SRR17520069.1866390
SRR17520069.1866392
SRR17520069.1866394
SRR17520069.1866396
SRR17520069.1866398
SRR17520069.1866400
SRR17520069.1866402
SRR17520069.1866404
SRR17520069.1866406

SRR17520069.1866408
SRR17520069.1866917
SRR17520069.1870889
SRR17520069.1870891
SRR17520069.1870893
SRR17520069.1870895
SRR17520069.1870897
SRR17520069.1870899
SRR17520069.1870901
SRR17520069.1870903
SRR17520069.1870905
SRR17520069.1870907
SRR17520069.1870909
SRR17520069.1870911
SRR17520069.1870913
SRR17520069.1870915
SRR17520069.1870917
SRR17520069.1870919
SRR17520069.1870921
SRR17520069.1870923
SRR17520069.1870925
SRR17520069.1870927
SRR17520069.1870931
SRR17520069.1870933
SRR17520069.1870935
SRR17520069.1870939
SRR17520069.1878927
SRR17520069.1879966
SRR17520069.1879968
SRR17520069.1879970
SRR17520069.1879972
SRR17520069.1879974
SRR17520069.1879976
SRR17520069.1879978
SRR17520069.1879980
SRR17520069.1879982
SRR17520069.1879984
SRR17520069.1879986
SRR17520069.1879988
SRR17520069.1879990
SRR17520069.1879992
SRR17520069.1879994
SRR17520069.1879996
SRR17520069.1880000
SRR17520069.1880002
SRR17520069.1880004
SRR17520069.1881305

SRR17520069.1881307
SRR17520069.1881309
SRR17520069.1881311
SRR17520069.1881313
SRR17520069.1881315
SRR17520069.1881317
SRR17520069.1881319
SRR17520069.1881321
SRR17520069.1881323
SRR17520069.1881325
SRR17520069.1881327
SRR17520069.1881329
SRR17520069.1881331
SRR17520069.1881333
SRR17520069.1881335
SRR17520069.1881337
SRR17520069.1881339
SRR17520069.1881341
SRR17520069.1881343
SRR17520069.1881345
SRR17520069.1881347
SRR17520069.1881349
SRR17520069.1881351
SRR17520069.1881353
SRR17520069.1881355
SRR17520069.1881357
SRR17520069.1881359
SRR17520069.1881361
SRR17520069.1881363
SRR17520069.1881365
SRR17520069.1881367
SRR17520069.1881369
SRR17520069.1881371
SRR17520069.1881373
SRR17520069.1881375
SRR17520069.1881377
SRR17520069.1881379
SRR17520069.1881381
SRR17520069.1881383
SRR17520069.1881385
SRR17520069.1881387
SRR17520069.1881389
SRR17520069.1881391
SRR17520069.1881393
SRR17520069.1881395
SRR17520069.1881397
SRR17520069.1881399

SRR17520069.1881401
SRR17520069.1881403
SRR17520069.1881405
SRR17520069.1881407
SRR17520069.1881409
SRR17520069.1881411
SRR17520069.1881413
SRR17520069.1881415
SRR17520069.1881417
SRR17520069.1881419
SRR17520069.1881421
SRR17520069.1881423
SRR17520069.1881425
SRR17520069.1881427
SRR17520069.1881429
SRR17520069.1881431
SRR17520069.1881433
SRR17520069.1881435
SRR17520069.1881437
SRR17520069.1881439
SRR17520069.1881441
SRR17520069.1881443
SRR17520069.1881445
SRR17520069.1881447
SRR17520069.1881449
SRR17520069.1881451
SRR17520069.1881453
SRR17520069.1881455
SRR17520069.1881457
SRR17520069.1881459
SRR17520069.1881461
SRR17520069.1881463
SRR17520069.1881465
SRR17520069.1881467
SRR17520069.1881469
SRR17520069.1881471
SRR17520069.1881473
SRR17520069.1881475
SRR17520069.1881477
SRR17520069.1881479
SRR17520069.1881481
SRR17520069.1881483
SRR17520069.1881485
SRR17520069.1881487
SRR17520069.1881489
SRR17520069.1881491
SRR17520069.1881493

SRR17520069.1885096
SRR17520069.1885098
SRR17520069.1885100
SRR17520069.1885102
SRR17520069.1885104
SRR17520069.1885106
SRR17520069.1887369
SRR17520069.1887371
SRR17520069.1887373
SRR17520069.1887375
SRR17520069.1887379
SRR17520069.1887381
SRR17520069.1887383
SRR17520069.1887385
SRR17520069.1887387
SRR17520069.1887389
SRR17520069.1887391
SRR17520069.1887393
SRR17520069.1887395
SRR17520069.1887397
SRR17520069.1887399
SRR17520069.1887401
SRR17520069.1887403
SRR17520069.1887405
SRR17520069.1887407
SRR17520069.1887409
SRR17520069.1887411
SRR17520069.1887413
SRR17520069.1887415
SRR17520069.1887417
SRR17520069.1887419
SRR17520069.1887421
SRR17520069.1887423
SRR17520069.1887425
SRR17520069.1887427
SRR17520069.1887429
SRR17520069.1887431
SRR17520069.1890049
SRR17520069.1890992
SRR17520069.1890994
SRR17520069.1890996
SRR17520069.1890998
SRR17520069.1891000
SRR17520069.1891002
SRR17520069.1891006
SRR17520069.1891008
SRR17520069.1891010

SRR17520069.1891014
SRR17520069.1891018
SRR17520069.1891020
SRR17520069.1891022
SRR17520069.1891024
SRR17520069.1891026
SRR17520069.1891028
SRR17520069.1891030
SRR17520069.1891032
SRR17520069.1891034
SRR17520069.1891036
SRR17520069.1891038
SRR17520069.1891040
SRR17520069.1891042
SRR17520069.1891044
SRR17520069.1891046
SRR17520069.1891048
SRR17520069.1891050
SRR17520069.1891052
SRR17520069.1891054
SRR17520069.1891056
SRR17520069.1891058
SRR17520069.1891060
SRR17520069.1891062
SRR17520069.1891064
SRR17520069.1891066
SRR17520069.1891068
SRR17520069.1891070
SRR17520069.1891072
SRR17520069.1891076
SRR17520069.1891078
SRR17520069.1891080
SRR17520069.1891082
SRR17520069.1891084
SRR17520069.1891086
SRR17520069.1891088
SRR17520069.1891090
SRR17520069.1891092
SRR17520069.1891094
SRR17520069.1891096
SRR17520069.1891098
SRR17520069.1891100
SRR17520069.1891102
SRR17520069.1891104
SRR17520069.1891106
SRR17520069.1891108
SRR17520069.1892111

SRR17520069.1892113
SRR17520069.1892115
SRR17520069.1892117
SRR17520069.1892119
SRR17520069.1892121
SRR17520069.1892123
SRR17520069.1892125
SRR17520069.1892127
SRR17520069.1892129
SRR17520069.1892131
SRR17520069.1892133
SRR17520069.1892135
SRR17520069.1892137
SRR17520069.1892139
SRR17520069.1892141
SRR17520069.1892143
SRR17520069.1892145
SRR17520069.1892147
SRR17520069.1892149
SRR17520069.1892151
SRR17520069.1892153
SRR17520069.1892155
SRR17520069.1892157
SRR17520069.1892159
SRR17520069.1892161
SRR17520069.1892163
SRR17520069.1892165
SRR17520069.1892167
SRR17520069.1892169
SRR17520069.1892171
SRR17520069.1892173
SRR17520069.1892175
SRR17520069.1892177
SRR17520069.1895966
SRR17520069.1895968
SRR17520069.1895970
SRR17520069.1895972
SRR17520069.1895974
SRR17520069.1895976
SRR17520069.1895978
SRR17520069.1895980
SRR17520069.1895982
SRR17520069.1895984
SRR17520069.1895986
SRR17520069.1895990
SRR17520069.1895992
SRR17520069.1895994

SRR17520069.1895996
SRR17520069.1895998
SRR17520069.1896000
SRR17520069.1896002
SRR17520069.1896004
SRR17520069.1896006
SRR17520069.1896008
SRR17520069.1896010
SRR17520069.1896012
SRR17520069.1896014
SRR17520069.1896016
SRR17520069.1896018
SRR17520069.1896020
SRR17520069.1896022
SRR17520069.1896024
SRR17520069.1896026
SRR17520069.1896028
SRR17520069.1896030
SRR17520069.1896032
SRR17520069.1896034
SRR17520069.1896036
SRR17520069.1896038
SRR17520069.1896040
SRR17520069.1896042
SRR17520069.1896044
SRR17520069.1896046
SRR17520069.1896048
SRR17520069.1896050
SRR17520069.1896052
SRR17520069.1896054
SRR17520069.1896056
SRR17520069.1899900
SRR17520069.1899902
SRR17520069.1899904
SRR17520069.1899906
SRR17520069.1899908
SRR17520069.1899910
SRR17520069.1899912
SRR17520069.1903355
SRR17520069.1903361
SRR17520069.1903365
SRR17520069.1905386
SRR17520069.1905388
SRR17520069.1905390
SRR17520069.1905392
SRR17520069.1905394
SRR17520069.1905396

SRR17520069.1905398
SRR17520069.1905400
SRR17520069.1905402
SRR17520069.1905404
SRR17520069.1905408
SRR17520069.1905410
SRR17520069.1905412
SRR17520069.1905414
SRR17520069.1905416
SRR17520069.1905418
SRR17520069.1905420
SRR17520069.1910043
SRR17520069.1917897
SRR17520069.1917899
SRR17520069.1917901
SRR17520069.1917903
SRR17520069.1917905
SRR17520069.1917907
SRR17520069.1917909
SRR17520069.1917911
SRR17520069.1917913
SRR17520069.1917915
SRR17520069.1917917
SRR17520069.1917919
SRR17520069.1917921
SRR17520069.1917923
SRR17520069.1917925
SRR17520069.1917927
SRR17520069.1917929
SRR17520069.1917931
SRR17520069.1917933
SRR17520069.1917935
SRR17520069.1917937
SRR17520069.1917939
SRR17520069.1917941
SRR17520069.1917943
SRR17520069.1917945
SRR17520069.1917947
SRR17520069.1917949
SRR17520069.1917951
SRR17520069.1917953
SRR17520069.1917955
SRR17520069.1917958
SRR17520069.1917964
SRR17520069.1917966
SRR17520069.1917968
SRR17520069.1917970

SRR17520069.1917972
SRR17520069.1917974
SRR17520069.1917978
SRR17520069.1917984
SRR17520069.1917986
SRR17520069.1917988
SRR17520069.1917990
SRR17520069.1917992
SRR17520069.1917994
SRR17520069.1917996
SRR17520069.1917998
SRR17520069.1918000
SRR17520069.1918002
SRR17520069.1918004
SRR17520069.1918006
SRR17520069.1918008
SRR17520069.1918010
SRR17520069.1918014
SRR17520069.1918018
SRR17520069.1921243
SRR17520069.1921247
SRR17520069.1921249
SRR17520069.1921251
SRR17520069.1921253
SRR17520069.1921255
SRR17520069.1921662
SRR17520069.1921664
SRR17520069.1921666
SRR17520069.1921668
SRR17520069.1921670
SRR17520069.1921672
SRR17520069.1921674
SRR17520069.1921676
SRR17520069.1921678
SRR17520069.1921680
SRR17520069.1921682
SRR17520069.1921684
SRR17520069.1921686
SRR17520069.1921688
SRR17520069.1921690
SRR17520069.1921692
SRR17520069.1921694
SRR17520069.1921696
SRR17520069.1921698
SRR17520069.1921819
SRR17520069.1921821
SRR17520069.1921823

SRR17520069.1921825
SRR17520069.1921827
SRR17520069.1923432
SRR17520069.1923434
SRR17520069.1923436
SRR17520069.1923438
SRR17520069.1923440
SRR17520069.1923442
SRR17520069.1923444
SRR17520069.1923446
SRR17520069.1923684
SRR17520069.1923686
SRR17520069.1923688
SRR17520069.1923690
SRR17520069.1923692
SRR17520069.1923694
SRR17520069.1923696
SRR17520069.1923698
SRR17520069.1923700
SRR17520069.1923702
SRR17520069.1923704
SRR17520069.1923706
SRR17520069.1923708
SRR17520069.1923710
SRR17520069.1923712
SRR17520069.1923714
SRR17520069.1923716
SRR17520069.1923718
SRR17520069.1923720
SRR17520069.1923722
SRR17520069.1923724
SRR17520069.1926776
SRR17520069.1926778
SRR17520069.1926780
SRR17520069.1926782
SRR17520069.1926784
SRR17520069.1926786
SRR17520069.1926788
SRR17520069.1926790
SRR17520069.1926792
SRR17520069.1926794
SRR17520069.1926796
SRR17520069.1926798
SRR17520069.1926800
SRR17520069.1926802
SRR17520069.1926804
SRR17520069.1926806

SRR17520069.1926808
SRR17520069.1926810
SRR17520069.1926814
SRR17520069.1926816
SRR17520069.1926818
SRR17520069.1926820
SRR17520069.1926822
SRR17520069.1926824
SRR17520069.1926826
SRR17520069.1926830
SRR17520069.1926832
SRR17520069.1926834
SRR17520069.1926836
SRR17520069.1926838
SRR17520069.1926840
SRR17520069.1937398
SRR17520069.1937400
SRR17520069.1937402
SRR17520069.1937404
SRR17520069.1937406
SRR17520069.1938389
SRR17520069.1938393
SRR17520069.1938395
SRR17520069.1938397
SRR17520069.1938399
SRR17520069.1942012
SRR17520069.1942014
SRR17520069.1942016
SRR17520069.1942018
SRR17520069.1942020
SRR17520069.1942024
SRR17520069.1942026
SRR17520069.1949481
SRR17520069.1951531
SRR17520069.1951533
SRR17520069.1951535
SRR17520069.1951537
SRR17520069.1951539
SRR17520069.1951541
SRR17520069.1951543
SRR17520069.1951545
SRR17520069.1951547
SRR17520069.1951549
SRR17520069.1951551
SRR17520069.1951553
SRR17520069.1951555
SRR17520069.1951557

SRR17520069.1951559
SRR17520069.1951561
SRR17520069.1951563
SRR17520069.1951565
SRR17520069.1951567
SRR17520069.1951569
SRR17520069.1951571
SRR17520069.1951573
SRR17520069.1951575
SRR17520069.1951577
SRR17520069.1951579
SRR17520069.1951581
SRR17520069.1951585
SRR17520069.1951587
SRR17520069.1951589
SRR17520069.1951591
SRR17520069.1951593
SRR17520069.1951595
SRR17520069.1951597
SRR17520069.1951599
SRR17520069.1951601
SRR17520069.1951603
SRR17520069.1951605
SRR17520069.1951607
SRR17520069.1951609
SRR17520069.1951611
SRR17520069.1951613
SRR17520069.1951615
SRR17520069.1951617
SRR17520069.1951619
SRR17520069.1951621
SRR17520069.1951623
SRR17520069.1951625
SRR17520069.1951627
SRR17520069.1951629
SRR17520069.1957133
SRR17520069.1957135
SRR17520069.1957137
SRR17520069.1962143
SRR17520069.1962145
SRR17520069.1962147
SRR17520069.1962149
SRR17520069.1962151
SRR17520069.1968262
SRR17520069.1971247
SRR17520069.1971280
SRR17520069.1971699

SRR17520069.1971701
SRR17520069.1971703
SRR17520069.1971705
SRR17520069.1971707
SRR17520069.1971709
SRR17520069.1971711
SRR17520069.1971715
SRR17520069.1971717
SRR17520069.1971719
SRR17520069.1971721
SRR17520069.1977178
SRR17520069.1977180
SRR17520069.1977182
SRR17520069.1977184
SRR17520069.1977186
SRR17520069.1977188
SRR17520069.1977190
SRR17520069.1977192
SRR17520069.1977194
SRR17520069.1977196
SRR17520069.1977198
SRR17520069.1977200
SRR17520069.1977202
SRR17520069.1977204
SRR17520069.1977206
SRR17520069.1977208
SRR17520069.1977210
SRR17520069.1977212
SRR17520069.1977214
SRR17520069.1977216
SRR17520069.1977218
SRR17520069.1977220
SRR17520069.1977222
SRR17520069.1977224
SRR17520069.1977226
SRR17520069.1977228
SRR17520069.1977230
SRR17520069.1977232
SRR17520069.1977234
SRR17520069.1977236
SRR17520069.1977238
SRR17520069.1977240
SRR17520069.1977242
SRR17520069.1977244
SRR17520069.1977246
SRR17520069.1977248
SRR17520069.1977250

SRR17520069.1977252
SRR17520069.1977254
SRR17520069.1977256
SRR17520069.1977258
SRR17520069.1977260
SRR17520069.1977262
SRR17520069.1977264
SRR17520069.1977266
SRR17520069.1977268
SRR17520069.1978384
SRR17520069.1978386
SRR17520069.1978388
SRR17520069.1978390
SRR17520069.1978392
SRR17520069.1978394
SRR17520069.1978396
SRR17520069.1978399
SRR17520069.1978401
SRR17520069.1978403
SRR17520069.1978405
SRR17520069.1978407
SRR17520069.1978409
SRR17520069.1978411
SRR17520069.1978413
SRR17520069.1978415
SRR17520069.1978417
SRR17520069.1978419
SRR17520069.1978421
SRR17520069.1978423
SRR17520069.1978425
SRR17520069.1978427
SRR17520069.1978429
SRR17520069.1978431
SRR17520069.1978433
SRR17520069.1978435
SRR17520069.1978437
SRR17520069.1978439
SRR17520069.1978441
SRR17520069.1978443
SRR17520069.1978445
SRR17520069.1978447
SRR17520069.1978449
SRR17520069.1978451
SRR17520069.1978453
SRR17520069.1978455
SRR17520069.1978457
SRR17520069.1978459

SRR17520069.1978461
SRR17520069.1978463
SRR17520069.1978465
SRR17520069.1978467
SRR17520069.1978469
SRR17520069.1978637
SRR17520069.1978639
SRR17520069.1978641
SRR17520069.1978643
SRR17520069.1978645
SRR17520069.1978647
SRR17520069.1978649
SRR17520069.1978651
SRR17520069.1978653
SRR17520069.1978657
SRR17520069.1978659
SRR17520069.1978661
SRR17520069.1978663
SRR17520069.1978665
SRR17520069.1978667
SRR17520069.1978669
SRR17520069.1978673
SRR17520069.1978675
SRR17520069.1978677
SRR17520069.1978681
SRR17520069.1978683
SRR17520069.1978685
SRR17520069.1978687
SRR17520069.1980876
SRR17520069.1980878
SRR17520069.1980880
SRR17520069.1980882
SRR17520069.1980886
SRR17520069.1980888
SRR17520069.1980890
SRR17520069.1980892
SRR17520069.1980894
SRR17520069.1980896
SRR17520069.1980898
SRR17520069.1980900
SRR17520069.1980902
SRR17520069.1980904
SRR17520069.1980906
SRR17520069.1980908
SRR17520069.1980910
SRR17520069.1980912
SRR17520069.1980914

SRR17520069.1980916
SRR17520069.1980918
SRR17520069.1980920
SRR17520069.1980922
SRR17520069.1980924
SRR17520069.1980926
SRR17520069.1980928
SRR17520069.1980930
SRR17520069.1980932
SRR17520069.1980934
SRR17520069.1980936
SRR17520069.1980938
SRR17520069.1980940
SRR17520069.1980942
SRR17520069.1980944
SRR17520069.1980946
SRR17520069.1980948
SRR17520069.1980950
SRR17520069.1980952
SRR17520069.1980954
SRR17520069.1980956
SRR17520069.1980958
SRR17520069.1980960
SRR17520069.1980962
SRR17520069.1980964
SRR17520069.1980966
SRR17520069.1980968
SRR17520069.1980970
SRR17520069.1980972
SRR17520069.1980974
SRR17520069.1980976
SRR17520069.1980978
SRR17520069.1980982
SRR17520069.1980984
SRR17520069.1980986
SRR17520069.1980988
SRR17520069.1980992
SRR17520069.1980998
SRR17520069.1981000
SRR17520069.1981002
SRR17520069.1981004
SRR17520069.1981006
SRR17520069.1981008
SRR17520069.1981016
SRR17520069.1983069
SRR17520069.1983071
SRR17520069.1983073

SRR17520069.1983075
SRR17520069.1983077
SRR17520069.1983079
SRR17520069.1983081
SRR17520069.1983083
SRR17520069.1983085
SRR17520069.1983087
SRR17520069.1983089
SRR17520069.1983091
SRR17520069.1983093
SRR17520069.1983095
SRR17520069.1983097
SRR17520069.1983099
SRR17520069.1983101
SRR17520069.1983103
SRR17520069.1983105
SRR17520069.1983107
SRR17520069.1983109
SRR17520069.1983111
SRR17520069.1983113
SRR17520069.1983115
SRR17520069.1983117
SRR17520069.1983119
SRR17520069.1983121
SRR17520069.1983123
SRR17520069.1983125
SRR17520069.1983127
SRR17520069.1983129
SRR17520069.1983131
SRR17520069.1983133
SRR17520069.1983135
SRR17520069.1983137
SRR17520069.1983139
SRR17520069.1983141
SRR17520069.1983143
SRR17520069.1983145
SRR17520069.1983147
SRR17520069.1983149
SRR17520069.1983151
SRR17520069.1983153
SRR17520069.1983155
SRR17520069.1983157
SRR17520069.1983159
SRR17520069.1983161
SRR17520069.1983163
SRR17520069.1983165
SRR17520069.1983167

SRR17520069.1983169
SRR17520069.1983171
SRR17520069.1983790
SRR17520069.1983792
SRR17520069.1985079
SRR17520069.1985081
SRR17520069.1985083
SRR17520069.1985085
SRR17520069.1985087
SRR17520069.1985089
SRR17520069.1985091
SRR17520069.1985093
SRR17520069.1985095
SRR17520069.1985097
SRR17520069.1985099
SRR17520069.1993823
SRR17520069.1993955
SRR17520069.1993957
SRR17520069.1993959
SRR17520069.1993961
SRR17520069.1993963
SRR17520069.1993965
SRR17520069.1993967
SRR17520069.1995131
SRR17520069.1995133
SRR17520069.1995135
SRR17520069.1995137
SRR17520069.1995139
SRR17520069.1995141
SRR17520069.1995143
SRR17520069.1995145
SRR17520069.1995147
SRR17520069.1995149
SRR17520069.1995151
SRR17520069.1995153
SRR17520069.1998133
SRR17520069.1998135
SRR17520069.1998137
SRR17520069.1998139
SRR17520069.1998141
SRR17520069.1998145
SRR17520069.1998147
SRR17520069.1998149
SRR17520069.1998151
SRR17520069.1998153
SRR17520069.1998155
SRR17520069.1998157

SRR17520069.1998159
SRR17520069.1998161
SRR17520069.1998165
SRR17520069.1998167
SRR17520069.1998169
SRR17520069.1998171
SRR17520069.1998173
SRR17520069.1998175
SRR17520069.1998177
SRR17520069.1998179
SRR17520069.1998181
SRR17520069.1998183
SRR17520069.1998185
SRR17520069.1998187
SRR17520069.1998189
SRR17520069.1998191
SRR17520069.1998193
SRR17520069.1998197
SRR17520069.1998199
SRR17520069.1998201
SRR17520069.1998203
SRR17520069.1998205
SRR17520069.1998207
SRR17520069.1998209
SRR17520069.1998211
SRR17520069.1998213
SRR17520069.1998215
SRR17520069.1998217
SRR17520069.1998221
SRR17520069.1998223
SRR17520069.1998225
SRR17520069.1998227
SRR17520069.1998229
SRR17520069.1998231
SRR17520069.1998233
SRR17520069.1998235
SRR17520069.1998237
SRR17520069.1998239
SRR17520069.1998241
SRR17520069.1998243
SRR17520069.1998245
SRR17520069.1998247
SRR17520069.1998249
SRR17520069.1998251
SRR17520069.1998253
SRR17520069.2004398
SRR17520069.2004400

SRR17520069.2004402
SRR17520069.2004404
SRR17520069.2004406
SRR17520069.2004408
SRR17520069.2004410
SRR17520069.2004412
SRR17520069.2004414
SRR17520069.2004416
SRR17520069.2004418
SRR17520069.2006830
SRR17520069.2006832
SRR17520069.2006840
SRR17520069.2006842
SRR17520069.2006844
SRR17520069.2006846
SRR17520069.2006848
SRR17520069.2006850
SRR17520069.2006852
SRR17520069.2006854
SRR17520069.2006856
SRR17520069.2014514
SRR17520069.2014518
SRR17520069.2014520
SRR17520069.2014522
SRR17520069.2014524
SRR17520069.2014526
SRR17520069.2014528
SRR17520069.2014530
SRR17520069.2014532
SRR17520069.2014534
SRR17520069.2014536
SRR17520069.2014538
SRR17520069.2014540
SRR17520069.2014542
SRR17520069.2014544
SRR17520069.2014546
SRR17520069.2014548
SRR17520069.2014550
SRR17520069.2014552
SRR17520069.2014554
SRR17520069.2014558
SRR17520069.2014560
SRR17520069.2014562
SRR17520069.2014564
SRR17520069.2014566
SRR17520069.2014568
SRR17520069.2014570

SRR17520069.2014572
SRR17520069.2014574
SRR17520069.2014576
SRR17520069.2014578
SRR17520069.2014580
SRR17520069.2014582
SRR17520069.2014584
SRR17520069.2014586
SRR17520069.2014588
SRR17520069.2014590
SRR17520069.2014592
SRR17520069.2014594
SRR17520069.2014596
SRR17520069.2014598
SRR17520069.2014600
SRR17520069.2014602
SRR17520069.2014604
SRR17520069.2014606
SRR17520069.2014608
SRR17520069.2014610
SRR17520069.2014612
SRR17520069.2015900
SRR17520069.2015902
SRR17520069.2015904
SRR17520069.2015906
SRR17520069.2015908
SRR17520069.2015910
SRR17520069.2015912
SRR17520069.2015914
SRR17520069.2015916
SRR17520069.2015918
SRR17520069.2015920
SRR17520069.2015922
SRR17520069.2015924
SRR17520069.2015926
SRR17520069.2015928
SRR17520069.2015932
SRR17520069.2015934
SRR17520069.2015936
SRR17520069.2015938
SRR17520069.2015940
SRR17520069.2015942
SRR17520069.2015944
SRR17520069.2015946
SRR17520069.2015948
SRR17520069.2015950
SRR17520069.2015952

SRR17520069.2015954
SRR17520069.2015956
SRR17520069.2015958
SRR17520069.2015960
SRR17520069.2016952
SRR17520069.2016954
SRR17520069.2016956
SRR17520069.2016958
SRR17520069.2016960
SRR17520069.2016962
SRR17520069.2016964
SRR17520069.2016966
SRR17520069.2016968
SRR17520069.2016970
SRR17520069.2016972
SRR17520069.2016974
SRR17520069.2016976
SRR17520069.2016978
SRR17520069.2017508
SRR17520069.2017510
SRR17520069.2017512
SRR17520069.2017961
SRR17520069.2017963
SRR17520069.2017965
SRR17520069.2017967
SRR17520069.2017969
SRR17520069.2017973
SRR17520069.2017975
SRR17520069.2017977
SRR17520069.2017979
SRR17520069.2017981
SRR17520069.2017983
SRR17520069.2017985
SRR17520069.2017987
SRR17520069.2017989
SRR17520069.2017991
SRR17520069.2017993
SRR17520069.2017995
SRR17520069.2017997
SRR17520069.2017999
SRR17520069.2018001
SRR17520069.2018003
SRR17520069.2018005
SRR17520069.2018007
SRR17520069.2018009
SRR17520069.2018011
SRR17520069.2018013

SRR17520069.2018015
SRR17520069.2018017
SRR17520069.2023940
SRR17520069.2023942
SRR17520069.2023946
SRR17520069.2023948
SRR17520069.2023950
SRR17520069.2023952
SRR17520069.2023954
SRR17520069.2024223
SRR17520069.2024225
SRR17520069.2024227
SRR17520069.2024229
SRR17520069.2024231
SRR17520069.2024233
SRR17520069.2024235
SRR17520069.2024237
SRR17520069.2024239
SRR17520069.2024241
SRR17520069.2024243
SRR17520069.2024245
SRR17520069.2032874
SRR17520069.2032876
SRR17520069.2032878
SRR17520069.2032880
SRR17520069.2032882
SRR17520069.2032884
SRR17520069.2032886
SRR17520069.2032888
SRR17520069.2032890
SRR17520069.2032892
SRR17520069.2032894
SRR17520069.2032896
SRR17520069.2032898
SRR17520069.2032900
SRR17520069.2034169
SRR17520069.2034171
SRR17520069.2034173
SRR17520069.2034175
SRR17520069.2034177
SRR17520069.2034179
SRR17520069.2034181
SRR17520069.2034183
SRR17520069.2034185
SRR17520069.2034187
SRR17520069.2034189
SRR17520069.2034191

SRR17520069.2034193
SRR17520069.2034195
SRR17520069.2034197
SRR17520069.2034199
SRR17520069.2034201
SRR17520069.2034203
SRR17520069.2034205
SRR17520069.2034207
SRR17520069.2034209
SRR17520069.2034211
SRR17520069.2034213
SRR17520069.2034215
SRR17520069.2034217
SRR17520069.2034219
SRR17520069.2034221
SRR17520069.2034223
SRR17520069.2034225
SRR17520069.2034227
SRR17520069.2034229
SRR17520069.2034231
SRR17520069.2034233
SRR17520069.2034235
SRR17520069.2034237
SRR17520069.2034239
SRR17520069.2034241
SRR17520069.2034243
SRR17520069.2034245
SRR17520069.2034247
SRR17520069.2034249
SRR17520069.2034251
SRR17520069.2034253
SRR17520069.2034255
SRR17520069.2034257
SRR17520069.2035362
SRR17520069.2035364
SRR17520069.2035366
SRR17520069.2035370
SRR17520069.2035372
SRR17520069.2035374
SRR17520069.2035376
SRR17520069.2035378
SRR17520069.2035380
SRR17520069.2035382
SRR17520069.2035384
SRR17520069.2035386
SRR17520069.2035388
SRR17520069.2035390

SRR17520069.2035394
SRR17520069.2035396
SRR17520069.2035398
SRR17520069.2035400
SRR17520069.2035402
SRR17520069.2035404
SRR17520069.2035406
SRR17520069.2035408
SRR17520069.2035410
SRR17520069.2035412
SRR17520069.2035414
SRR17520069.2035418
SRR17520069.2035420
SRR17520069.2035422
SRR17520069.2038350
SRR17520069.2038352
SRR17520069.2038354
SRR17520069.2038356
SRR17520069.2038360
SRR17520069.2038362
SRR17520069.2038364
SRR17520069.2039083
SRR17520069.2039085
SRR17520069.2039129
SRR17520069.2041870
SRR17520069.2041872
SRR17520069.2041874
SRR17520069.2041876
SRR17520069.2041878
SRR17520069.2041880
SRR17520069.2041882
SRR17520069.2041884
SRR17520069.2041886
SRR17520069.2041888
SRR17520069.2041890
SRR17520069.2041892
SRR17520069.2041894
SRR17520069.2041896
SRR17520069.2041898
SRR17520069.2041900
SRR17520069.2044968
SRR17520069.2044970
SRR17520069.2044972
SRR17520069.2044974
SRR17520069.2044976
SRR17520069.2044978
SRR17520069.2044980

SRR17520069.2044982
SRR17520069.2044984
SRR17520069.2044986
SRR17520069.2044988
SRR17520069.2044990
SRR17520069.2044992
SRR17520069.2044994
SRR17520069.2044996
SRR17520069.2044998
SRR17520069.2045000
SRR17520069.2045002
SRR17520069.2045004
SRR17520069.2045006
SRR17520069.2045008
SRR17520069.2045010
SRR17520069.2045012
SRR17520069.2045014
SRR17520069.2045016
SRR17520069.2045018
SRR17520069.2045020
SRR17520069.2045022
SRR17520069.2045024
SRR17520069.2045026
SRR17520069.2045028
SRR17520069.2045030
SRR17520069.2045032
SRR17520069.2045034
SRR17520069.2045036
SRR17520069.2045038
SRR17520069.2045040
SRR17520069.2045042
SRR17520069.2045044
SRR17520069.2045046
SRR17520069.2045048
SRR17520069.2045050
SRR17520069.2045052
SRR17520069.2045054
SRR17520069.2045056
SRR17520069.2045569
SRR17520069.2045571
SRR17520069.2045573
SRR17520069.2045575
SRR17520069.2048048
SRR17520069.2048050
SRR17520069.2048052
SRR17520069.2052289
SRR17520069.2052291

SRR17520069.2052293
SRR17520069.2052295
SRR17520069.2052297
SRR17520069.2052299
SRR17520069.2052301
SRR17520069.2052303
SRR17520069.2052305
SRR17520069.2052307
SRR17520069.2052309
SRR17520069.2052311
SRR17520069.2052313
SRR17520069.2052315
SRR17520069.2052317
SRR17520069.2052319
SRR17520069.2052321
SRR17520069.2052323
SRR17520069.2052325
SRR17520069.2052327
SRR17520069.2052329
SRR17520069.2060681
SRR17520069.2060683
SRR17520069.2060685
SRR17520069.2060687
SRR17520069.2066657
SRR17520069.2066659
SRR17520069.2066661
SRR17520069.2066663
SRR17520069.2066665
SRR17520069.2066667
SRR17520069.2066669
SRR17520069.2066671
SRR17520069.2066673
SRR17520069.2066675
SRR17520069.2066677
SRR17520069.2066679
SRR17520069.2066681
SRR17520069.2066685
SRR17520069.2066687
SRR17520069.2066689
SRR17520069.2066691
SRR17520069.2073930
SRR17520069.2073932
SRR17520069.2073934
SRR17520069.2073936
SRR17520069.2073938
SRR17520069.2073940
SRR17520069.2073942

SRR17520069.2073944
SRR17520069.2073948
SRR17520069.2073952
SRR17520069.2073954
SRR17520069.2073956
SRR17520069.2073958
SRR17520069.2073960
SRR17520069.2073964
SRR17520069.2073966
SRR17520069.2073968
SRR17520069.2073970
SRR17520069.2073974
SRR17520069.2073976
SRR17520069.2073978
SRR17520069.2073982
SRR17520069.2073984
SRR17520069.2073986
SRR17520069.2073988
SRR17520069.2076538
SRR17520069.2076540
SRR17520069.2076542
SRR17520069.2076544
SRR17520069.2076546
SRR17520069.2076548
SRR17520069.2076550
SRR17520069.2076552
SRR17520069.2076554
SRR17520069.2076556
SRR17520069.2076558
SRR17520069.2076560
SRR17520069.2076568
SRR17520069.2076570
SRR17520069.2076574
SRR17520069.2076576
SRR17520069.2076578
SRR17520069.2076580
SRR17520069.2076582
SRR17520069.2076584
SRR17520069.2076586
SRR17520069.2076588
SRR17520069.2076590
SRR17520069.2076592
SRR17520069.2076594
SRR17520069.2076596
SRR17520069.2076598
SRR17520069.2076600
SRR17520069.2076602

SRR17520069.2076604
SRR17520069.2076606
SRR17520069.2076608
SRR17520069.2076610
SRR17520069.2076612
SRR17520069.2076614
SRR17520069.2076616
SRR17520069.2076618
SRR17520069.2076620
SRR17520069.2076622
SRR17520069.2076624
SRR17520069.2076626
SRR17520069.2076630
SRR17520069.2076632
SRR17520069.2076634
SRR17520069.2076636
SRR17520069.2076638
SRR17520069.2076640
SRR17520069.2076642
SRR17520069.2076644
SRR17520069.2076646
SRR17520069.2076648
SRR17520069.2076650
SRR17520069.2076652
SRR17520069.2076654
SRR17520069.2076656
SRR17520069.2076658
SRR17520069.2076660
SRR17520069.2076662
SRR17520069.2076664
SRR17520069.2076666
SRR17520069.2076668
SRR17520069.2076670
SRR17520069.2076672
SRR17520069.2076674
SRR17520069.2076676
SRR17520069.2076678
SRR17520069.2076680
SRR17520069.2076682
SRR17520069.2076684
SRR17520069.2076686
SRR17520069.2079030
SRR17520069.2079032
SRR17520069.2079034
SRR17520069.2079954
SRR17520069.2079956
SRR17520069.2079958

SRR17520069.2079960
SRR17520069.2079962
SRR17520069.2079964
SRR17520069.2079966
SRR17520069.2079968
SRR17520069.2079970
SRR17520069.2079972
SRR17520069.2079974
SRR17520069.2079976
SRR17520069.2079978
SRR17520069.2079980
SRR17520069.2079982
SRR17520069.2079984
SRR17520069.2079986
SRR17520069.2079988
SRR17520069.2079990
SRR17520069.2079992
SRR17520069.2079994
SRR17520069.2079996
SRR17520069.2079998
SRR17520069.2080000
SRR17520069.2080002
SRR17520069.2080004
SRR17520069.2080006
SRR17520069.2080008
SRR17520069.2080010
SRR17520069.2080012
SRR17520069.2080014
SRR17520069.2080016
SRR17520069.2080018
SRR17520069.2080020
SRR17520069.2080492
SRR17520069.2080494
SRR17520069.2080496
SRR17520069.2080498
SRR17520069.2080500
SRR17520069.2080502
SRR17520069.2080504
SRR17520069.2080506
SRR17520069.2080508
SRR17520069.2080510
SRR17520069.2080512
SRR17520069.2080514
SRR17520069.2080516
SRR17520069.2080518
SRR17520069.2080520
SRR17520069.2080522

SRR17520069.2080524
SRR17520069.2080526
SRR17520069.2080528
SRR17520069.2080530
SRR17520069.2080532
SRR17520069.2080534
SRR17520069.2080536
SRR17520069.2080538
SRR17520069.2080540
SRR17520069.2080542
SRR17520069.2087781
SRR17520069.2087783
SRR17520069.2087785
SRR17520069.2087787
SRR17520069.2087789
SRR17520069.2087791
SRR17520069.2087793
SRR17520069.2087795
SRR17520069.2087797
SRR17520069.2087799
SRR17520069.2087801
SRR17520069.2087803
SRR17520069.2087805
SRR17520069.2087807
SRR17520069.2087809
SRR17520069.2087811
SRR17520069.2087813
SRR17520069.2087815
SRR17520069.2087817
SRR17520069.2087819
SRR17520069.2087821
SRR17520069.2087823
SRR17520069.2087825
SRR17520069.2087827
SRR17520069.2087829
SRR17520069.2087831
SRR17520069.2087833
SRR17520069.2087835
SRR17520069.2087837
SRR17520069.2087839
SRR17520069.2087841
SRR17520069.2087843
SRR17520069.2087845
SRR17520069.2087847
SRR17520069.2087849
SRR17520069.2087851
SRR17520069.2087853

SRR17520069.2087855
SRR17520069.2087857
SRR17520069.2087859
SRR17520069.2087861
SRR17520069.2087863
SRR17520069.2087865
SRR17520069.2087867
SRR17520069.2087869
SRR17520069.2087871
SRR17520069.2087873
SRR17520069.2087875
SRR17520069.2097231
SRR17520069.2098220
SRR17520069.2098222
SRR17520069.2098226
SRR17520069.2098228
SRR17520069.2098230
SRR17520069.2101512
SRR17520069.2101514
SRR17520069.2101516
SRR17520069.2101518
SRR17520069.2101520
SRR17520069.2101522
SRR17520069.2101524
SRR17520069.2101526
SRR17520069.2101528
SRR17520069.2101530
SRR17520069.2101532
SRR17520069.2101536
SRR17520069.2101540
SRR17520069.2101542
SRR17520069.2101544
SRR17520069.2101546
SRR17520069.2101548
SRR17520069.2101550
SRR17520069.2101552
SRR17520069.2101554
SRR17520069.2101556
SRR17520069.2101558
SRR17520069.2101560
SRR17520069.2101564
SRR17520069.2101566
SRR17520069.2101568
SRR17520069.2101570
SRR17520069.2101572
SRR17520069.2101574
SRR17520069.2101576

SRR17520069.2101580
SRR17520069.2106129
SRR17520069.2106131
SRR17520069.2106133
SRR17520069.2106135
SRR17520069.2106137
SRR17520069.2106139
SRR17520069.2106141
SRR17520069.2106143
SRR17520069.2106584
SRR17520069.2106588
SRR17520069.2106590
SRR17520069.2106592
SRR17520069.2106594
SRR17520069.2106596
SRR17520069.2106598
SRR17520069.2106600
SRR17520069.2106602
SRR17520069.2106604
SRR17520069.2106606
SRR17520069.2106608
SRR17520069.2106610
SRR17520069.2106612
SRR17520069.2106616
SRR17520069.2106618
SRR17520069.2106620
SRR17520069.2106622
SRR17520069.2106624
SRR17520069.2106626
SRR17520069.2106628
SRR17520069.2106630
SRR17520069.2106632
SRR17520069.2106634
SRR17520069.2107402
SRR17520069.2107406
SRR17520069.2107408
SRR17520069.2107410
SRR17520069.2107412
SRR17520069.2107416
SRR17520069.2107418
SRR17520069.2107422
SRR17520069.2110842
SRR17520069.2110844
SRR17520069.2110846
SRR17520069.2110848
SRR17520069.2110850
SRR17520069.2110852

SRR17520069.2110854
SRR17520069.2110856
SRR17520069.2110858
SRR17520069.2110860
SRR17520069.2110862
SRR17520069.2110864
SRR17520069.2110868
SRR17520069.2110870
SRR17520069.2113411
SRR17520069.2113413
SRR17520069.2113415
SRR17520069.2113417
SRR17520069.2113421
SRR17520069.2113721
SRR17520069.2113723
SRR17520069.2113725
SRR17520069.2113727
SRR17520069.2113729
SRR17520069.2113731
SRR17520069.2125174
SRR17520069.2125176
SRR17520069.2125178
SRR17520069.2127071
SRR17520069.2127073
SRR17520069.2127075
SRR17520069.2128828
SRR17520069.2128830
SRR17520069.2134826
SRR17520069.2134828
SRR17520069.2134830
SRR17520069.2134832
SRR17520069.2134834
SRR17520069.2134836
SRR17520069.2134838
SRR17520069.2134840
SRR17520069.2134842
SRR17520069.2134846
SRR17520069.2134848
SRR17520069.2134850
SRR17520069.2136608
SRR17520069.2136610
SRR17520069.2136612
SRR17520069.2136614
SRR17520069.2136616
SRR17520069.2136618
SRR17520069.2136620
SRR17520069.2136622

SRR17520069.2136624
SRR17520069.2136626
SRR17520069.2136628
SRR17520069.2136630
SRR17520069.2136632
SRR17520069.2136634
SRR17520069.2136636
SRR17520069.2136638
SRR17520069.2136640
SRR17520069.2136642
SRR17520069.2136644
SRR17520069.2136646
SRR17520069.2136648
SRR17520069.2136650
SRR17520069.2136652
SRR17520069.2136654
SRR17520069.2136656
SRR17520069.2136658
SRR17520069.2136660
SRR17520069.2136662
SRR17520069.2137113
SRR17520069.2137115
SRR17520069.2137117
SRR17520069.2137119
SRR17520069.2137121
SRR17520069.2137123
SRR17520069.2138511
SRR17520069.2141496
SRR17520069.2141498
SRR17520069.2141500
SRR17520069.2142900
SRR17520069.2142902
SRR17520069.2142906
SRR17520069.2142912
SRR17520069.2142914
SRR17520069.2143718
SRR17520069.2143720
SRR17520069.2143722
SRR17520069.2143724
SRR17520069.2143726
SRR17520069.2143728
SRR17520069.2143730
SRR17520069.2143732
SRR17520069.2145037
SRR17520069.2145039
SRR17520069.2145043
SRR17520069.2145045

SRR17520069.2145047
SRR17520069.2145049
SRR17520069.2145051
SRR17520069.2145053
SRR17520069.2145055
SRR17520069.2145057
SRR17520069.2145059
SRR17520069.2145061
SRR17520069.2145065
SRR17520069.2145067
SRR17520069.2145069
SRR17520069.2145071
SRR17520069.2148916
SRR17520069.2148918
SRR17520069.2148920
SRR17520069.2148922
SRR17520069.2148924
SRR17520069.2148928
SRR17520069.2148932
SRR17520069.2148936
SRR17520069.2148938
SRR17520069.2148940
SRR17520069.2148942
SRR17520069.2148944
SRR17520069.2160474
SRR17520069.2160476
SRR17520069.2167598
SRR17520069.2170041
SRR17520069.2170043
SRR17520069.2170045
SRR17520069.2170047
SRR17520069.2170051
SRR17520069.2175264
SRR17520069.2175266
SRR17520069.2175268
SRR17520069.2175270
SRR17520069.2175272
SRR17520069.2175274
SRR17520069.2175276
SRR17520069.2175278
SRR17520069.2175280
SRR17520069.2175282
SRR17520069.2175284
SRR17520069.2175286
SRR17520069.2175290
SRR17520069.2175292
SRR17520069.2175294

SRR17520069.2175296
SRR17520069.2175298
SRR17520069.2175300
SRR17520069.2175302
SRR17520069.2175304
SRR17520069.2175306
SRR17520069.2175310
SRR17520069.2175312
SRR17520069.2175314
SRR17520069.2175318
SRR17520069.2175320
SRR17520069.2175322
SRR17520069.2175324
SRR17520069.2175326
SRR17520069.2175328
SRR17520069.2175330
SRR17520069.2176621
SRR17520069.2176623
SRR17520069.2176625
SRR17520069.2176627
SRR17520069.2176629
SRR17520069.2176631
SRR17520069.2176633
SRR17520069.2176635
SRR17520069.2176637
SRR17520069.2176639
SRR17520069.2176641
SRR17520069.2176643
SRR17520069.2176645
SRR17520069.2176647
SRR17520069.2181540
SRR17520069.2181542
SRR17520069.2181544
SRR17520069.2181546
SRR17520069.2181548
SRR17520069.2181550
SRR17520069.2181552
SRR17520069.2181554
SRR17520069.2181556
SRR17520069.2181558
SRR17520069.2181560
SRR17520069.2181562
SRR17520069.2188653
SRR17520069.2188655
SRR17520069.2188657
SRR17520069.2188659
SRR17520069.2188661

SRR17520069.2188663
SRR17520069.2188665
SRR17520069.2188669
SRR17520069.2188671
SRR17520069.2188673
SRR17520069.2188675
SRR17520069.2188677
SRR17520069.2188681
SRR17520069.2188683
SRR17520069.2188685
SRR17520069.2188687
SRR17520069.2188689
SRR17520069.2188691
SRR17520069.2188693
SRR17520069.2188695
SRR17520069.2188697
SRR17520069.2188699
SRR17520069.2188701
SRR17520069.2188703
SRR17520069.2188705
SRR17520069.2188707
SRR17520069.2188709
SRR17520069.2188711
SRR17520069.2188719
SRR17520069.2188721
SRR17520069.2190732
SRR17520069.2190734
SRR17520069.2190736
SRR17520069.2190738
SRR17520069.2190740
SRR17520069.2202764
SRR17520069.2202766
SRR17520069.2202768
SRR17520069.2209803
SRR17520069.2209805
SRR17520069.2209807
SRR17520069.2209809
SRR17520069.2209811
SRR17520069.2209813
SRR17520069.2209815
SRR17520069.2209817
SRR17520069.2209819
SRR17520069.2209821
SRR17520069.2209823
SRR17520069.2209825
SRR17520069.2209827
SRR17520069.2209829

SRR17520069.2209831
SRR17520069.2209833
SRR17520069.2209835
SRR17520069.2209837
SRR17520069.2209839
SRR17520069.2209841
SRR17520069.2209843
SRR17520069.2209845
SRR17520069.2209847
SRR17520069.2209849
SRR17520069.2209851
SRR17520069.2209853
SRR17520069.2209855
SRR17520069.2209857
SRR17520069.2209859
SRR17520069.2209861
SRR17520069.2209863
SRR17520069.2209865
SRR17520069.2209867
SRR17520069.2209869
SRR17520069.2209871
SRR17520069.2209873
SRR17520069.2209875
SRR17520069.2209877
SRR17520069.2209879
SRR17520069.2209881
SRR17520069.2209883
SRR17520069.2209885
SRR17520069.2209887
SRR17520069.2209889
SRR17520069.2209891
SRR17520069.2209893
SRR17520069.2209895
SRR17520069.2209897
SRR17520069.2209921
SRR17520069.2209923
SRR17520069.2209925
SRR17520069.2209927
SRR17520069.2209929
SRR17520069.2209931
SRR17520069.2209933
SRR17520069.2209935
SRR17520069.2209937
SRR17520069.2209939
SRR17520069.2209941
SRR17520069.2209943
SRR17520069.2223817

SRR17520069.2228854
SRR17520069.2228856
SRR17520069.2228858
SRR17520069.2230242
SRR17520069.2230244
SRR17520069.2230246
SRR17520069.2230252
SRR17520069.2230254
SRR17520069.2230256
SRR17520069.2230258
SRR17520069.2230260
SRR17520069.2230262
SRR17520069.2230264
SRR17520069.2230266
SRR17520069.2230270
SRR17520069.2230272
SRR17520069.2230274
SRR17520069.2230276
SRR17520069.2230278
SRR17520069.2230282
SRR17520069.2230284
SRR17520069.2230286
SRR17520069.2230288
SRR17520069.2230290
SRR17520069.2230292
SRR17520069.2230294
SRR17520069.2230296
SRR17520069.2230300
SRR17520069.2230306
SRR17520069.2230308
SRR17520069.2230310
SRR17520069.2230312
SRR17520069.2230314
SRR17520069.2230316
SRR17520069.2230318
SRR17520069.2230320
SRR17520069.2230322
SRR17520069.2230324
SRR17520069.2230326
SRR17520069.2230328
SRR17520069.2230330
SRR17520069.2230332
SRR17520069.2230334
SRR17520069.2230336
SRR17520069.2230338
SRR17520069.2230340
SRR17520069.2230342

SRR17520069.2235062
SRR17520069.2235064
SRR17520069.2235066
SRR17520069.2235068
SRR17520069.2235070
SRR17520069.2235072
SRR17520069.2235074
SRR17520069.2248857
SRR17520069.2248859
SRR17520069.2248861
SRR17520069.2248863
SRR17520069.2248865
SRR17520069.2248867
SRR17520069.2248869
SRR17520069.2248871
SRR17520069.2248875
SRR17520069.2248877
SRR17520069.2252032
SRR17520069.2252034
SRR17520069.2252036
SRR17520069.2252038
SRR17520069.2252040
SRR17520069.2252042
SRR17520069.2252044
SRR17520069.2252046
SRR17520069.2252048
SRR17520069.2252050
SRR17520069.2252052
SRR17520069.2252054
SRR17520069.2252056
SRR17520069.2252058
SRR17520069.2252060
SRR17520069.2252062
SRR17520069.2256993
SRR17520069.2256995
SRR17520069.2256997
SRR17520069.2256999
SRR17520069.2257001
SRR17520069.2257003
SRR17520069.2257005
SRR17520069.2257007
SRR17520069.2257009
SRR17520069.2257011
SRR17520069.2257013
SRR17520069.2257015
SRR17520069.2257017
SRR17520069.2257019

SRR17520069.2257021
SRR17520069.2261307
SRR17520069.2261309
SRR17520069.2262163
SRR17520069.2262165
SRR17520069.2262167
SRR17520069.2279524
SRR17520069.2279526
SRR17520069.2279528
SRR17520069.2279530
SRR17520069.2279532
SRR17520069.2279534
SRR17520069.2279536
SRR17520069.2279538
SRR17520069.2279540
SRR17520069.2279542
SRR17520069.2280984
SRR17520069.2280986
SRR17520069.2280988
SRR17520069.2280990
SRR17520069.2280992
SRR17520069.2280994
SRR17520069.2280996
SRR17520069.2280998
SRR17520069.2281000
SRR17520069.2281002
SRR17520069.2281004
SRR17520069.2281006
SRR17520069.2281008
SRR17520069.2281010
SRR17520069.2281012
SRR17520069.2281014
SRR17520069.2281016
SRR17520069.2281018
SRR17520069.2281020
SRR17520069.2282014
SRR17520069.2305246
SRR17520069.2305248
SRR17520069.2305250
SRR17520069.2305254
SRR17520069.2305256
SRR17520069.2307117
SRR17520069.2307119
SRR17520069.2310072
SRR17520069.2310074
SRR17520069.2310076
SRR17520069.2310078

SRR17520069.2310080
SRR17520069.2310082
SRR17520069.2310084
SRR17520069.2310086
SRR17520069.2310088
SRR17520069.2310090
SRR17520069.2310092
SRR17520069.2310094
SRR17520069.2310096
SRR17520069.2310098
SRR17520069.2310100
SRR17520069.2310102
SRR17520069.2310104
SRR17520069.2310106
SRR17520069.2310108
SRR17520069.2310110
SRR17520069.2314532
SRR17520069.2316720
SRR17520069.2321483
SRR17520069.2321485
SRR17520069.2321487
SRR17520069.2323262
SRR17520069.2323264
SRR17520069.2323266
SRR17520069.2323268
SRR17520069.2323270
SRR17520069.2323272
SRR17520069.2323274
SRR17520069.2323276
SRR17520069.2323278
SRR17520069.2323280
SRR17520069.2323282
SRR17520069.2323284
SRR17520069.2323286
SRR17520069.2323288
SRR17520069.2323290
SRR17520069.2328031
SRR17520069.2328033
SRR17520069.2328035
SRR17520069.2328037
SRR17520069.2328039
SRR17520069.2328041
SRR17520069.2328043
SRR17520069.2328045
SRR17520069.2328047
SRR17520069.2328049
SRR17520069.2328051

SRR17520069.2328053
SRR17520069.2328055
SRR17520069.2328057
SRR17520069.2328059
SRR17520069.2328061
SRR17520069.2328063
SRR17520069.2328065
SRR17520069.2328067
SRR17520069.2328069
SRR17520069.2328071
SRR17520069.2328075
SRR17520069.2328077
SRR17520069.2328079
SRR17520069.2328081
SRR17520069.2328083
SRR17520069.2328085
SRR17520069.2328087
SRR17520069.2328089
SRR17520069.2328094
SRR17520069.2328096
SRR17520069.2328098
SRR17520069.2328100
SRR17520069.2328102
SRR17520069.2328104
SRR17520069.2328106
SRR17520069.2328108
SRR17520069.2336080
SRR17520069.2336082
SRR17520069.2336084
SRR17520069.2336088
SRR17520069.2336090
SRR17520069.2336092
SRR17520069.2336094
SRR17520069.2336096
SRR17520069.2336098
SRR17520069.2336100
SRR17520069.2336102
SRR17520069.2336104
SRR17520069.2336106
SRR17520069.2336108
SRR17520069.2336110
SRR17520069.2336112
SRR17520069.2336114
SRR17520069.2336116
SRR17520069.2336118
SRR17520069.2336120
SRR17520069.2340344

SRR17520069.2340348
SRR17520069.2340350
SRR17520069.2340352
SRR17520069.2340354
SRR17520069.2340356
SRR17520069.2340358
SRR17520069.2340360
SRR17520069.2340362
SRR17520069.2340364
SRR17520069.2340366
SRR17520069.2340368
SRR17520069.2340370
SRR17520069.2340372
SRR17520069.2340374
SRR17520069.2340380
SRR17520069.2340382
SRR17520069.2340384
SRR17520069.2340388
SRR17520069.2340390
SRR17520069.2340392
SRR17520069.2340394
SRR17520069.2340396
SRR17520069.2340398
SRR17520069.2340400
SRR17520069.2340402
SRR17520069.2340404
SRR17520069.2340406
SRR17520069.2340408
SRR17520069.2340410
SRR17520069.2340412
SRR17520069.2340414
SRR17520069.2340416
SRR17520069.2340418
SRR17520069.2345404
SRR17520069.2345406
SRR17520069.2345408
SRR17520069.2345410
SRR17520069.2345412
SRR17520069.2345414
SRR17520069.2345416
SRR17520069.2345420
SRR17520069.2345422
SRR17520069.2345424
SRR17520069.2345426
SRR17520069.2345428
SRR17520069.2345430
SRR17520069.2345432

SRR17520069.2345434
SRR17520069.2345436
SRR17520069.2345438
SRR17520069.2345440
SRR17520069.2345442
SRR17520069.2345444
SRR17520069.2345446
SRR17520069.2345448
SRR17520069.2345450
SRR17520069.2345452
SRR17520069.2357982
SRR17520069.2357984
SRR17520069.2357986
SRR17520069.2357988
SRR17520069.2363264
SRR17520069.2363266
SRR17520069.2363268
SRR17520069.2363270
SRR17520069.2363272
SRR17520069.2363274
SRR17520069.2363276
SRR17520069.2363278
SRR17520069.2363280
SRR17520069.2363282
SRR17520069.2363284
SRR17520069.2363286
SRR17520069.2363288
SRR17520069.2363290
SRR17520069.2363292
SRR17520069.2363294
SRR17520069.2363296
SRR17520069.2363298
SRR17520069.2363300
SRR17520069.2363302
SRR17520069.2363304
SRR17520069.2363306
SRR17520069.2363308
SRR17520069.2363310
SRR17520069.2363312
SRR17520069.2363314
SRR17520069.2363316
SRR17520069.2363318
SRR17520069.2363320
SRR17520069.2363322
SRR17520069.2363324
SRR17520069.2363326
SRR17520069.2363328

SRR17520069.2363330
SRR17520069.2363332
SRR17520069.2363334
SRR17520069.2363336
SRR17520069.2363338
SRR17520069.2363340
SRR17520069.2363342
SRR17520069.2363344
SRR17520069.2363346
SRR17520069.2363348
SRR17520069.2363350
SRR17520069.2363352
SRR17520069.2363354
SRR17520069.2363356
SRR17520069.2363358
SRR17520069.2363360
SRR17520069.2363362
SRR17520069.2363364
SRR17520069.2363366
SRR17520069.2363368
SRR17520069.2363370
SRR17520069.2363372
SRR17520069.2363374
SRR17520069.2363376
SRR17520069.2363486
SRR17520069.2363488
SRR17520069.2363490
SRR17520069.2363492
SRR17520069.2363494
SRR17520069.2363496
SRR17520069.2363498
SRR17520069.2363500
SRR17520069.2363502
SRR17520069.2363504
SRR17520069.2363506
SRR17520069.2363508
SRR17520069.2363510
SRR17520069.2363512
SRR17520069.2363514
SRR17520069.2363516
SRR17520069.2363520
SRR17520069.2363524
SRR17520069.2363526
SRR17520069.2363528
SRR17520069.2363530
SRR17520069.2363532
SRR17520069.2363538

SRR17520069.2363542
SRR17520069.2363544
SRR17520069.2363546
SRR17520069.2364212
SRR17520069.2364214
SRR17520069.2364216
SRR17520069.2364218
SRR17520069.2364220
SRR17520069.2364222
SRR17520069.2364224
SRR17520069.2366357
SRR17520069.2366359
SRR17520069.2366361
SRR17520069.2366363
SRR17520069.2366365
SRR17520069.2366367
SRR17520069.2366369
SRR17520069.2366371
SRR17520069.2366373
SRR17520069.2366375
SRR17520069.2366377
SRR17520069.2366379
SRR17520069.2366381
SRR17520069.2366383
SRR17520069.2366387
SRR17520069.2366389
SRR17520069.2366391
SRR17520069.2377102
SRR17520069.2377104
SRR17520069.2377106
SRR17520069.2377108
SRR17520069.2377110
SRR17520069.2377112
SRR17520069.2377114
SRR17520069.2377116
SRR17520069.2377118
SRR17520069.2377120
SRR17520069.2377122
SRR17520069.2377124
SRR17520069.2377126
SRR17520069.2377128
SRR17520069.2377132
SRR17520069.2377134
SRR17520069.2377136
SRR17520069.2377138
SRR17520069.2377140
SRR17520069.2377142

SRR17520069.2377144
SRR17520069.2377146
SRR17520069.2377148
SRR17520069.2377150
SRR17520069.2377152
SRR17520069.2377154
SRR17520069.2377156
SRR17520069.2377158
SRR17520069.2377160
SRR17520069.2377162
SRR17520069.2377164
SRR17520069.2377166
SRR17520069.2377994
SRR17520069.2378038
SRR17520069.2378040
SRR17520069.2381320
SRR17520069.2381322
SRR17520069.2385598
SRR17520069.2385600
SRR17520069.2385602
SRR17520069.2385604
SRR17520069.2385606
SRR17520069.2385608
SRR17520069.2385610
SRR17520069.2385612
SRR17520069.2385614
SRR17520069.2385616
SRR17520069.2385618
SRR17520069.2385620
SRR17520069.2385622
SRR17520069.2385624
SRR17520069.2385626
SRR17520069.2385630
SRR17520069.2385632
SRR17520069.2385634
SRR17520069.2385636
SRR17520069.2385638
SRR17520069.2385640
SRR17520069.2385642
SRR17520069.2385644
SRR17520069.2385646
SRR17520069.2385648
SRR17520069.2385650
SRR17520069.2385652
SRR17520069.2385654
SRR17520069.2385658
SRR17520069.2385660

SRR17520069.2385662
SRR17520069.2385664
SRR17520069.2385666
SRR17520069.2385668
SRR17520069.2385670
SRR17520069.2385672
SRR17520069.2385674
SRR17520069.2385676
SRR17520069.2385678
SRR17520069.2385680
SRR17520069.2388459
SRR17520069.2388461
SRR17520069.2388463
SRR17520069.2388465
SRR17520069.2388467
SRR17520069.2388469
SRR17520069.2388471
SRR17520069.2388473
SRR17520069.2388475
SRR17520069.2388477
SRR17520069.2388479
SRR17520069.2388481
SRR17520069.2388483
SRR17520069.2388485
SRR17520069.2388487
SRR17520069.2388489
SRR17520069.2388491
SRR17520069.2388493
SRR17520069.2388495
SRR17520069.2388497
SRR17520069.2388499
SRR17520069.2388501
SRR17520069.2388503
SRR17520069.2388507
SRR17520069.2388509
SRR17520069.2388511
SRR17520069.2388515
SRR17520069.2388517
SRR17520069.2388519
SRR17520069.2388521
SRR17520069.2388523
SRR17520069.2388525
SRR17520069.2388527
SRR17520069.2388529
SRR17520069.2388531
SRR17520069.2388533
SRR17520069.2388535

SRR17520069.2388537
SRR17520069.2388539
SRR17520069.2388541
SRR17520069.2388543
SRR17520069.2388545
SRR17520069.2388547
SRR17520069.2388549
SRR17520069.2388551
SRR17520069.2388553
SRR17520069.2388555
SRR17520069.2388557
SRR17520069.2388559
SRR17520069.2388561
SRR17520069.2388563
SRR17520069.2388565
SRR17520069.2388567
SRR17520069.2388569
SRR17520069.2388571
SRR17520069.2388573
SRR17520069.2388575
SRR17520069.2388577
SRR17520069.2388579
SRR17520069.2388581
SRR17520069.2388583
SRR17520069.2388585
SRR17520069.2388587
SRR17520069.2388589
SRR17520069.2388597
SRR17520069.2388599
SRR17520069.2388607
SRR17520069.2388615
SRR17520069.2388617
SRR17520069.2388619
SRR17520069.2389558
SRR17520069.2391402
SRR17520069.2391404
SRR17520069.2391406
SRR17520069.2391408
SRR17520069.2391410
SRR17520069.2391412
SRR17520069.2391414
SRR17520069.2391416
SRR17520069.2394979
SRR17520069.2394981
SRR17520069.2394983
SRR17520069.2394985
SRR17520069.2394987

SRR17520069.2394989
SRR17520069.2394991
SRR17520069.2394995
SRR17520069.2394997
SRR17520069.2394999
SRR17520069.2395001
SRR17520069.2395003
SRR17520069.2395005
SRR17520069.2395007
SRR17520069.2395009
SRR17520069.2395011
SRR17520069.2395013
SRR17520069.2395015
SRR17520069.2395017
SRR17520069.2395019
SRR17520069.2395021
SRR17520069.2395023
SRR17520069.2395025
SRR17520069.2395027
SRR17520069.2395029
SRR17520069.2395031
SRR17520069.2395033
SRR17520069.2395035
SRR17520069.2395037
SRR17520069.2395041
SRR17520069.2395043
SRR17520069.2395047
SRR17520069.2395049
SRR17520069.2395051
SRR17520069.2395053
SRR17520069.2395055
SRR17520069.2395057
SRR17520069.2395059
SRR17520069.2395061
SRR17520069.2395063
SRR17520069.2395065
SRR17520069.2395067
SRR17520069.2395069
SRR17520069.2395071
SRR17520069.2395073
SRR17520069.2395075
SRR17520069.2395077
SRR17520069.2395079
SRR17520069.2395081
SRR17520069.2395083
SRR17520069.2395089
SRR17520069.2395091

SRR17520069.2395093
SRR17520069.2395095
SRR17520069.2395097
SRR17520069.2398352
SRR17520069.2398354
SRR17520069.2398356
SRR17520069.2398358
SRR17520069.2398360
SRR17520069.2398362
SRR17520069.2398364
SRR17520069.2398366
SRR17520069.2398368
SRR17520069.2398370
SRR17520069.2398374
SRR17520069.2398376
SRR17520069.2398378
SRR17520069.2398380
SRR17520069.2398384
SRR17520069.2398386
SRR17520069.2398388
SRR17520069.2398390
SRR17520069.2398392
SRR17520069.2398394
SRR17520069.2398396
SRR17520069.2398400
SRR17520069.2398402
SRR17520069.2398404
SRR17520069.2398406
SRR17520069.2398408
SRR17520069.2398410
SRR17520069.2398412
SRR17520069.2398414
SRR17520069.2398416
SRR17520069.2398418
SRR17520069.2398420
SRR17520069.2398424
SRR17520069.2398426
SRR17520069.2398428
SRR17520069.2398432
SRR17520069.2398434
SRR17520069.2398436
SRR17520069.2398438
SRR17520069.2398440
SRR17520069.2398442
SRR17520069.2398445
SRR17520069.2402399
SRR17520069.2402401

SRR17520069.2402403
SRR17520069.2402405
SRR17520069.2402407
SRR17520069.2402409
SRR17520069.2402411
SRR17520069.2402413
SRR17520069.2402415
SRR17520069.2402417
SRR17520069.2402419
SRR17520069.2402421
SRR17520069.2402423
SRR17520069.2402425
SRR17520069.2402429
SRR17520069.2402431
SRR17520069.2402433
SRR17520069.2402435
SRR17520069.2402437
SRR17520069.2402439
SRR17520069.2402441
SRR17520069.2402443
SRR17520069.2402445
SRR17520069.2402447
SRR17520069.2402449
SRR17520069.2402451
SRR17520069.2402453
SRR17520069.2402455
SRR17520069.2402457
SRR17520069.2402459
SRR17520069.2402461
SRR17520069.2402463
SRR17520069.2402465
SRR17520069.2402467
SRR17520069.2402469
SRR17520069.2402471
SRR17520069.2402473
SRR17520069.2402475
SRR17520069.2402477
SRR17520069.2402479
SRR17520069.2402481
SRR17520069.2402483
SRR17520069.2402485
SRR17520069.2402487
SRR17520069.2402489
SRR17520069.2402491
SRR17520069.2402493
SRR17520069.2402495
SRR17520069.2402497

SRR17520069.2402876
SRR17520069.2402878
SRR17520069.2402880
SRR17520069.2402882
SRR17520069.2402884
SRR17520069.2402886
SRR17520069.2402888
SRR17520069.2402890
SRR17520069.2402892
SRR17520069.2402894
SRR17520069.2408371
SRR17520069.2408373
SRR17520069.2408375
SRR17520069.2408377
SRR17520069.2408379
SRR17520069.2408381
SRR17520069.2408383
SRR17520069.2408385
SRR17520069.2408387
SRR17520069.2408389
SRR17520069.2408391
SRR17520069.2408393
SRR17520069.2408395
SRR17520069.2408397
SRR17520069.2408399
SRR17520069.2408401
SRR17520069.2408403
SRR17520069.2408405
SRR17520069.2408407
SRR17520069.2408409
SRR17520069.2408411
SRR17520069.2417732
SRR17520069.2417734
SRR17520069.2417736
SRR17520069.2417738
SRR17520069.2417740
SRR17520069.2417742
SRR17520069.2417744
SRR17520069.2417746
SRR17520069.2417748
SRR17520069.2417750
SRR17520069.2417752
SRR17520069.2417754
SRR17520069.2417758
SRR17520069.2417760
SRR17520069.2417762
SRR17520069.2417764

SRR17520069.2417766
SRR17520069.2417768
SRR17520069.2417770
SRR17520069.2424517
SRR17520069.2425180
SRR17520069.2425182
SRR17520069.2425184
SRR17520069.2425186
SRR17520069.2425188
SRR17520069.2431809
SRR17520069.2431811
SRR17520069.2431813
SRR17520069.2431815
SRR17520069.2431817
SRR17520069.2431819
SRR17520069.2431821
SRR17520069.2431823
SRR17520069.2431825
SRR17520069.2431827
SRR17520069.2431829
SRR17520069.2431831
SRR17520069.2431835
SRR17520069.2431837
SRR17520069.2431839
SRR17520069.2431841
SRR17520069.2431843
SRR17520069.2431845
SRR17520069.2431847
SRR17520069.2432688
SRR17520069.2432690
SRR17520069.2432692
SRR17520069.2435225
SRR17520069.2435227
SRR17520069.2435233
SRR17520069.2435237
SRR17520069.2435239
SRR17520069.2435251
SRR17520069.2435253
SRR17520069.2435255
SRR17520069.2435265
SRR17520069.2435267
SRR17520069.2435269
SRR17520069.2435271
SRR17520069.2435273
SRR17520069.2435277
SRR17520069.2435279
SRR17520069.2435281

SRR17520069.2435283
SRR17520069.2438285
SRR17520069.2438287
SRR17520069.2438289
SRR17520069.2438291
SRR17520069.2438293
SRR17520069.2438295
SRR17520069.2438297
SRR17520069.2438299
SRR17520069.2438301
SRR17520069.2438303
SRR17520069.2438305
SRR17520069.2438307
SRR17520069.2438309
SRR17520069.2438311
SRR17520069.2438313
SRR17520069.2438315
SRR17520069.2438317
SRR17520069.2438319
SRR17520069.2438321
SRR17520069.2438446
SRR17520069.2438448
SRR17520069.2438450
SRR17520069.2438452
SRR17520069.2438454
SRR17520069.2440389
SRR17520069.2440391
SRR17520069.2443705
SRR17520069.2443707
SRR17520069.2443709
SRR17520069.2448849
SRR17520069.2448851
SRR17520069.2448853
SRR17520069.2448855
SRR17520069.2448857
SRR17520069.2448859
SRR17520069.2448861
SRR17520069.2448863
SRR17520069.2448865
SRR17520069.2448867
SRR17520069.2448869
SRR17520069.2448871
SRR17520069.2448873
SRR17520069.2448875
SRR17520069.2448877
SRR17520069.2448879
SRR17520069.2448881

SRR17520069.2448883
SRR17520069.2448885
SRR17520069.2448887
SRR17520069.2448889
SRR17520069.2448891
SRR17520069.2448893
SRR17520069.2448895
SRR17520069.2448897
SRR17520069.2448899
SRR17520069.2448901
SRR17520069.2448903
SRR17520069.2448905
SRR17520069.2448907
SRR17520069.2448909
SRR17520069.2448911
SRR17520069.2448913
SRR17520069.2448915
SRR17520069.2448917
SRR17520069.2448919
SRR17520069.2448921
SRR17520069.2454527
SRR17520069.2454529
SRR17520069.2454531
SRR17520069.2454533
SRR17520069.2454535
SRR17520069.2454537
SRR17520069.2454539
SRR17520069.2454541
SRR17520069.2454543
SRR17520069.2454545
SRR17520069.2454547
SRR17520069.2454549
SRR17520069.2454551
SRR17520069.2454553
SRR17520069.2454555
SRR17520069.2454557
SRR17520069.2454559
SRR17520069.2454563
SRR17520069.2454565
SRR17520069.2454567
SRR17520069.2454569
SRR17520069.2454571
SRR17520069.2454573
SRR17520069.2454575
SRR17520069.2454579
SRR17520069.2454583
SRR17520069.2454585

SRR17520069.2454587
SRR17520069.2462057
SRR17520069.2462059
SRR17520069.2462065
SRR17520069.2462067
SRR17520069.2462069
SRR17520069.2462071
SRR17520069.2462073
SRR17520069.2462075
SRR17520069.2462077
SRR17520069.2462079
SRR17520069.2462081
SRR17520069.2462085
SRR17520069.2462087
SRR17520069.2462089
SRR17520069.2462548
SRR17520069.2462550
SRR17520069.2462552
SRR17520069.2462554
SRR17520069.2462556
SRR17520069.2462558
SRR17520069.2462560
SRR17520069.2462562
SRR17520069.2462564
SRR17520069.2463283
SRR17520069.2463285
SRR17520069.2463287
SRR17520069.2463289
SRR17520069.2463291
SRR17520069.2463293
SRR17520069.2463295
SRR17520069.2464937
SRR17520069.2464939
SRR17520069.2464941
SRR17520069.2464945
SRR17520069.2485113
SRR17520069.2485115
SRR17520069.2485117
SRR17520069.2485119
SRR17520069.2485121
SRR17520069.2489159
SRR17520069.2489161
SRR17520069.2489163
SRR17520069.2489165
SRR17520069.2489167
SRR17520069.2489169
SRR17520069.2489171

SRR17520069.2489173
SRR17520069.2489175
SRR17520069.2489177
SRR17520069.2489179
SRR17520069.2489181
SRR17520069.2489183
SRR17520069.2489185
SRR17520069.2489187
SRR17520069.2489189
SRR17520069.2489191
SRR17520069.2489193
SRR17520069.2489195
SRR17520069.2489197
SRR17520069.2489199
SRR17520069.2489201
SRR17520069.2489203
SRR17520069.2489205
SRR17520069.2489207
SRR17520069.2489209
SRR17520069.2489211
SRR17520069.2489213
SRR17520069.2489215
SRR17520069.2489217
SRR17520069.2489219
SRR17520069.2489221
SRR17520069.2489223
SRR17520069.2489225
SRR17520069.2489227
SRR17520069.2489229
SRR17520069.2489231
SRR17520069.2489233
SRR17520069.2489235
SRR17520069.2489239
SRR17520069.2489241
SRR17520069.2489243
SRR17520069.2489245
SRR17520069.2489247
SRR17520069.2489249
SRR17520069.2489251
SRR17520069.2489253
SRR17520069.2489255
SRR17520069.2489257
SRR17520069.2489259
SRR17520069.2489261
SRR17520069.2489263
SRR17520069.2489265
SRR17520069.2489267

SRR17520069.2489269
SRR17520069.2489271
SRR17520069.2489273
SRR17520069.2489275
SRR17520069.2489277
SRR17520069.2489279
SRR17520069.2489281
SRR17520069.2489283
SRR17520069.2489285
SRR17520069.2489287
SRR17520069.2489289
SRR17520069.2489291
SRR17520069.2489293
SRR17520069.2489295
SRR17520069.2489297
SRR17520069.2489299
SRR17520069.2489301
SRR17520069.2489303
SRR17520069.2489305
SRR17520069.2489307
SRR17520069.2489309
SRR17520069.2489311
SRR17520069.2489313
SRR17520069.2489315
SRR17520069.2490000
SRR17520069.2490002
SRR17520069.2490004
SRR17520069.2490006
SRR17520069.2490008
SRR17520069.2490010
SRR17520069.2490012
SRR17520069.2490014
SRR17520069.2490016
SRR17520069.2490018
SRR17520069.2490020
SRR17520069.2490022
SRR17520069.2490024
SRR17520069.2490026
SRR17520069.2490028
SRR17520069.2490030
SRR17520069.2490032
SRR17520069.2490034
SRR17520069.2494985
SRR17520069.2495028
SRR17520069.2495030
SRR17520069.2495032
SRR17520069.2495034

SRR17520069.2495036
SRR17520069.2495038
SRR17520069.2495040
SRR17520069.2495042
SRR17520069.2495044
SRR17520069.2495046
SRR17520069.2495048
SRR17520069.2495050
SRR17520069.2495052
SRR17520069.2495054
SRR17520069.2495056
SRR17520069.2495058
SRR17520069.2495060
SRR17520069.2495064
SRR17520069.2495066
SRR17520069.2495068
SRR17520069.2495072
SRR17520069.2495076
SRR17520069.2495080
SRR17520069.2495084
SRR17520069.2495088
SRR17520069.2495092
SRR17520069.2495096
SRR17520069.2497851
SRR17520069.2497853
SRR17520069.2497855
SRR17520069.2497857
SRR17520069.2497859
SRR17520069.2497861
SRR17520069.2497863
SRR17520069.2497865
SRR17520069.2497867
SRR17520069.2497869
SRR17520069.2497873
SRR17520069.2497875
SRR17520069.2497877
SRR17520069.2497879
SRR17520069.2497881
SRR17520069.2497883
SRR17520069.2497885
SRR17520069.2497887
SRR17520069.2497889
SRR17520069.2497891
SRR17520069.2497893
SRR17520069.2497895
SRR17520069.2497897
SRR17520069.2497899

SRR17520069.2497999
SRR17520069.2501454
SRR17520069.2501456
SRR17520069.2501458
SRR17520069.2501460
SRR17520069.2501462
SRR17520069.2501464
SRR17520069.2501466
SRR17520069.2501468
SRR17520069.2501470
SRR17520069.2501472
SRR17520069.2501474
SRR17520069.2501476
SRR17520069.2501478
SRR17520069.2501480
SRR17520069.2501482
SRR17520069.2501484
SRR17520069.2501486
SRR17520069.2505960
SRR17520069.2505964
SRR17520069.2505966
SRR17520069.2505972
SRR17520069.2505976
SRR17520069.2505978
SRR17520069.2505982
SRR17520069.2505984
SRR17520069.2505986
SRR17520069.2505988
SRR17520069.2505994
SRR17520069.2505996
SRR17520069.2505998
SRR17520069.2506000
SRR17520069.2506002
SRR17520069.2506004
SRR17520069.2506006
SRR17520069.2506008
SRR17520069.2506010
SRR17520069.2506012
SRR17520069.2506014
SRR17520069.2506016
SRR17520069.2506018
SRR17520069.2506020
SRR17520069.2506022
SRR17520069.2506026
SRR17520069.2506028
SRR17520069.2506030
SRR17520069.2506032

SRR17520069.2506036
SRR17520069.2506038
SRR17520069.2506040
SRR17520069.2506042
SRR17520069.2506044
SRR17520069.2506046
SRR17520069.2506048
SRR17520069.2506050
SRR17520069.2506052
SRR17520069.2506054
SRR17520069.2506056
SRR17520069.2514527
SRR17520069.2514529
SRR17520069.2514531
SRR17520069.2514533
SRR17520069.2514535
SRR17520069.2514537
SRR17520069.2514539
SRR17520069.2514541
SRR17520069.2514545
SRR17520069.2514547
SRR17520069.2514549
SRR17520069.2514551
SRR17520069.2514553
SRR17520069.2514555
SRR17520069.2514557
SRR17520069.2514559
SRR17520069.2514561
SRR17520069.2514563
SRR17520069.2514567
SRR17520069.2514569
SRR17520069.2514571
SRR17520069.2514573
SRR17520069.2514575
SRR17520069.2514577
SRR17520069.2514579
SRR17520069.2514581
SRR17520069.2514585
SRR17520069.2514587
SRR17520069.2514589
SRR17520069.2514591
SRR17520069.2514593
SRR17520069.2514595
SRR17520069.2514597
SRR17520069.2514599
SRR17520069.2514601
SRR17520069.2514603

SRR17520069.2514605
SRR17520069.2520965
SRR17520069.2520967
SRR17520069.2520969
SRR17520069.2520971
SRR17520069.2520973
SRR17520069.2520975
SRR17520069.2520977
SRR17520069.2520979
SRR17520069.2520981
SRR17520069.2520983
SRR17520069.2520985
SRR17520069.2520987
SRR17520069.2520991
SRR17520069.2520993
SRR17520069.2520995
SRR17520069.2520997
SRR17520069.2520999
SRR17520069.2521001
SRR17520069.2521003
SRR17520069.2521005
SRR17520069.2521007
SRR17520069.2524057
SRR17520069.2524059
SRR17520069.2524061
SRR17520069.2524063
SRR17520069.2524065
SRR17520069.2524067
SRR17520069.2524069
SRR17520069.2524071
SRR17520069.2524073
SRR17520069.2524075
SRR17520069.2524077
SRR17520069.2524079
SRR17520069.2524081
SRR17520069.2524083
SRR17520069.2524085
SRR17520069.2524087
SRR17520069.2524089
SRR17520069.2524091
SRR17520069.2524093
SRR17520069.2524095
SRR17520069.2524097
SRR17520069.2524099
SRR17520069.2524101
SRR17520069.2524103
SRR17520069.2524105

SRR17520069.2524107
SRR17520069.2524109
SRR17520069.2524111
SRR17520069.2524113
SRR17520069.2524115
SRR17520069.2524117
SRR17520069.2524119
SRR17520069.2524121
SRR17520069.2524123
SRR17520069.2524125
SRR17520069.2524127
SRR17520069.2524129
SRR17520069.2524131
SRR17520069.2524133
SRR17520069.2526004
SRR17520069.2526006
SRR17520069.2526008
SRR17520069.2526010
SRR17520069.2526012
SRR17520069.2526014
SRR17520069.2526016
SRR17520069.2526018
SRR17520069.2526020
SRR17520069.2526022
SRR17520069.2526024
SRR17520069.2526026
SRR17520069.2526028
SRR17520069.2526030
SRR17520069.2526032
SRR17520069.2526034
SRR17520069.2526036
SRR17520069.2526038
SRR17520069.2526040
SRR17520069.2526042
SRR17520069.2526044
SRR17520069.2526046
SRR17520069.2526048
SRR17520069.2526050
SRR17520069.2526052
SRR17520069.2526054
SRR17520069.2526056
SRR17520069.2526058
SRR17520069.2526060
SRR17520069.2526062
SRR17520069.2526064
SRR17520069.2526066
SRR17520069.2526068

SRR17520069.2526072
SRR17520069.2526074
SRR17520069.2526076
SRR17520069.2526078
SRR17520069.2526080
SRR17520069.2526082
SRR17520069.2526084
SRR17520069.2526086
SRR17520069.2526088
SRR17520069.2526090
SRR17520069.2526092
SRR17520069.2526094
SRR17520069.2526096
SRR17520069.2526098
SRR17520069.2526100
SRR17520069.2526102
SRR17520069.2526104
SRR17520069.2526106
SRR17520069.2526108
SRR17520069.2526110
SRR17520069.2526112
SRR17520069.2526114
SRR17520069.2526116
SRR17520069.2526118
SRR17520069.2526120
SRR17520069.2526122
SRR17520069.2526124
SRR17520069.2526126
SRR17520069.2532768
SRR17520069.2532770
SRR17520069.2532772
SRR17520069.2532774
SRR17520069.2532776
SRR17520069.2532778
SRR17520069.2532780
SRR17520069.2532782
SRR17520069.2532784
SRR17520069.2532786
SRR17520069.2532788
SRR17520069.2532790
SRR17520069.2532792
SRR17520069.2532794
SRR17520069.2532796
SRR17520069.2532798
SRR17520069.2532800
SRR17520069.2532802
SRR17520069.2532804

SRR17520069.2532806
SRR17520069.2532808
SRR17520069.2532810
SRR17520069.2532812
SRR17520069.2532814
SRR17520069.2532816
SRR17520069.2532818
SRR17520069.2532820
SRR17520069.2532822
SRR17520069.2532824
SRR17520069.2532826
SRR17520069.2532828
SRR17520069.2532830
SRR17520069.2532832
SRR17520069.2532834
SRR17520069.2532836
SRR17520069.2532838
SRR17520069.2532840
SRR17520069.2532842
SRR17520069.2532844
SRR17520069.2542071
SRR17520069.2542073
SRR17520069.2542075
SRR17520069.2542077
SRR17520069.2542079
SRR17520069.2542081
SRR17520069.2542083
SRR17520069.2542085
SRR17520069.2542087
SRR17520069.2542089
SRR17520069.2542091
SRR17520069.2542093
SRR17520069.2542095
SRR17520069.2542097
SRR17520069.2542099
SRR17520069.2542101
SRR17520069.2542103
SRR17520069.2542105
SRR17520069.2542107
SRR17520069.2542111
SRR17520069.2542113
SRR17520069.2542115
SRR17520069.2542117
SRR17520069.2542119
SRR17520069.2542121
SRR17520069.2542123
SRR17520069.2542125

SRR17520069.2542127
SRR17520069.2542129
SRR17520069.2543529
SRR17520069.2543531
SRR17520069.2543533
SRR17520069.2543537
SRR17520069.2543539
SRR17520069.2545275
SRR17520069.2545277
SRR17520069.2545279
SRR17520069.2545281
SRR17520069.2545283
SRR17520069.2545285
SRR17520069.2545287
SRR17520069.2545289
SRR17520069.2545291
SRR17520069.2545293
SRR17520069.2545295
SRR17520069.2545297
SRR17520069.2545299
SRR17520069.2545301
SRR17520069.2545303
SRR17520069.2545305
SRR17520069.2545307
SRR17520069.2545309
SRR17520069.2545311
SRR17520069.2545313
SRR17520069.2545315
SRR17520069.2545317
SRR17520069.2545319
SRR17520069.2545409
SRR17520069.2550476
SRR17520069.2550478
SRR17520069.2550480
SRR17520069.2550482
SRR17520069.2550484
SRR17520069.2550486
SRR17520069.2550488
SRR17520069.2550490
SRR17520069.2550492
SRR17520069.2550494
SRR17520069.2550496
SRR17520069.2550498
SRR17520069.2550500
SRR17520069.2550502
SRR17520069.2550504
SRR17520069.2550506

SRR17520069.2550510
SRR17520069.2550512
SRR17520069.2550514
SRR17520069.2550516
SRR17520069.2550518
SRR17520069.2550520
SRR17520069.2550522
SRR17520069.2550524
SRR17520069.2550526
SRR17520069.2550528
SRR17520069.2550530
SRR17520069.2550532
SRR17520069.2550534
SRR17520069.2550536
SRR17520069.2550538
SRR17520069.2550540
SRR17520069.2550542
SRR17520069.2550544
SRR17520069.2550546
SRR17520069.2550548
SRR17520069.2550550
SRR17520069.2550552
SRR17520069.2550554
SRR17520069.2550556
SRR17520069.2550558
SRR17520069.2550560
SRR17520069.2550562
SRR17520069.2550564
SRR17520069.2550566
SRR17520069.2550568
SRR17520069.2550570
SRR17520069.2550572
SRR17520069.2550574
SRR17520069.2550576
SRR17520069.2550578
SRR17520069.2550582
SRR17520069.2550584
SRR17520069.2550586
SRR17520069.2550588
SRR17520069.2550590
SRR17520069.2550592
SRR17520069.2555255
SRR17520069.2555257
SRR17520069.2555259
SRR17520069.2555261
SRR17520069.2555263
SRR17520069.2555265

SRR17520069.2555267
SRR17520069.2555269
SRR17520069.2555271
SRR17520069.2555273
SRR17520069.2555275
SRR17520069.2555277
SRR17520069.2555279
SRR17520069.2555281
SRR17520069.2555283
SRR17520069.2555285
SRR17520069.2555287
SRR17520069.2555289
SRR17520069.2555291
SRR17520069.2556255
SRR17520069.2556257
SRR17520069.2556259
SRR17520069.2556261
SRR17520069.2556263
SRR17520069.2563340
SRR17520069.2563342
SRR17520069.2563344
SRR17520069.2563346
SRR17520069.2563348
SRR17520069.2563350
SRR17520069.2563352
SRR17520069.2563354
SRR17520069.2563356
SRR17520069.2563358
SRR17520069.2564239
SRR17520069.2564243
SRR17520069.2564245
SRR17520069.2567029
SRR17520069.2567031
SRR17520069.2567033
SRR17520069.2567035
SRR17520069.2567037
SRR17520069.2567039
SRR17520069.2567041
SRR17520069.2567043
SRR17520069.2567045
SRR17520069.2567047
SRR17520069.2567049
SRR17520069.2567051
SRR17520069.2567053
SRR17520069.2567055
SRR17520069.2567057
SRR17520069.2567059

SRR17520069.2567061
SRR17520069.2567063
SRR17520069.2567065
SRR17520069.2567067
SRR17520069.2567069
SRR17520069.2567071
SRR17520069.2567073
SRR17520069.2567075
SRR17520069.2567077
SRR17520069.2567079
SRR17520069.2567081
SRR17520069.2567083
SRR17520069.2575943
SRR17520069.2575945
SRR17520069.2575949
SRR17520069.2577371
SRR17520069.2577856
SRR17520069.2577858
SRR17520069.2577860
SRR17520069.2577862
SRR17520069.2577864
SRR17520069.2597338
SRR17520069.2597340
SRR17520069.2597342
SRR17520069.2597344
SRR17520069.2597346
SRR17520069.2597348
SRR17520069.2597350
SRR17520069.2597352
SRR17520069.2597354
SRR17520069.2597356
SRR17520069.2597358
SRR17520069.2597360
SRR17520069.2597362
SRR17520069.2597364
SRR17520069.2597366
SRR17520069.2597368
SRR17520069.2597370
SRR17520069.2597372
SRR17520069.2597374
SRR17520069.2597378
SRR17520069.2597380
SRR17520069.2597382
SRR17520069.2597384
SRR17520069.2597386
SRR17520069.2597388
SRR17520069.2597390

SRR17520069.2597392
SRR17520069.2597394
SRR17520069.2597396
SRR17520069.2597398
SRR17520069.2597400
SRR17520069.2597402
SRR17520069.2597404
SRR17520069.2597406
SRR17520069.2597408
SRR17520069.2597410
SRR17520069.2600633
SRR17520069.2600635
SRR17520069.2600637
SRR17520069.2600639
SRR17520069.2600641
SRR17520069.2600643
SRR17520069.2600645
SRR17520069.2600647
SRR17520069.2600649
SRR17520069.2600651
SRR17520069.2600653
SRR17520069.2600655
SRR17520069.2604779
SRR17520069.2604781
SRR17520069.2604783
SRR17520069.2604785
SRR17520069.2604787
SRR17520069.2604789
SRR17520069.2604791
SRR17520069.2604793
SRR17520069.2604795
SRR17520069.2604797
SRR17520069.2604799
SRR17520069.2604801
SRR17520069.2604803
SRR17520069.2604805
SRR17520069.2604807
SRR17520069.2604809
SRR17520069.2604811
SRR17520069.2604813
SRR17520069.2604815
SRR17520069.2604817
SRR17520069.2604819
SRR17520069.2604821
SRR17520069.2604823
SRR17520069.2604825
SRR17520069.2604829

SRR17520069.2604831
SRR17520069.2605344
SRR17520069.2609906
SRR17520069.2609908
SRR17520069.2609910
SRR17520069.2611784
SRR17520069.2611786
SRR17520069.2611788
SRR17520069.2611790
SRR17520069.2611792
SRR17520069.2611794
SRR17520069.2611796
SRR17520069.2611798
SRR17520069.2611800
SRR17520069.2611802
SRR17520069.2611804
SRR17520069.2611806
SRR17520069.2611808
SRR17520069.2611810
SRR17520069.2611812
SRR17520069.2611816
SRR17520069.2611818
SRR17520069.2611820
SRR17520069.2611822
SRR17520069.2614047
SRR17520069.2614049
SRR17520069.2614051
SRR17520069.2614053
SRR17520069.2614055
SRR17520069.2614057
SRR17520069.2614059
SRR17520069.2614061
SRR17520069.2614063
SRR17520069.2614065
SRR17520069.2614067
SRR17520069.2614069
SRR17520069.2614071
SRR17520069.2614073
SRR17520069.2614075
SRR17520069.2614077
SRR17520069.2614079
SRR17520069.2614081
SRR17520069.2614083
SRR17520069.2614085
SRR17520069.2614087
SRR17520069.2614089
SRR17520069.2614091

SRR17520069.2614093
SRR17520069.2614095
SRR17520069.2614097
SRR17520069.2614101
SRR17520069.2614103
SRR17520069.2614105
SRR17520069.2614107
SRR17520069.2614109
SRR17520069.2614111
SRR17520069.2614113
SRR17520069.2614115
SRR17520069.2614117
SRR17520069.2614119
SRR17520069.2614121
SRR17520069.2614123
SRR17520069.2614125
SRR17520069.2614129
SRR17520069.2614131
SRR17520069.2614133
SRR17520069.2614135
SRR17520069.2614137
SRR17520069.2614139
SRR17520069.2614141
SRR17520069.2614143
SRR17520069.2614145
SRR17520069.2614147
SRR17520069.2614149
SRR17520069.2614151
SRR17520069.2614153
SRR17520069.2614155
SRR17520069.2614157
SRR17520069.2614159
SRR17520069.2614161
SRR17520069.2614163
SRR17520069.2614165
SRR17520069.2614167
SRR17520069.2614169
SRR17520069.2614171
SRR17520069.2614173
SRR17520069.2614288
SRR17520069.2614292
SRR17520069.2614294
SRR17520069.2614296
SRR17520069.2614302
SRR17520069.2614306
SRR17520069.2614308
SRR17520069.2614310

SRR17520069.2614312
SRR17520069.2614314
SRR17520069.2614316
SRR17520069.2614318
SRR17520069.2614320
SRR17520069.2614322
SRR17520069.2614324
SRR17520069.2614326
SRR17520069.2614328
SRR17520069.2614330
SRR17520069.2614332
SRR17520069.2625495
SRR17520069.2625497
SRR17520069.2625499
SRR17520069.2625503
SRR17520069.2626864
SRR17520069.2626866
SRR17520069.2626868
SRR17520069.2626870
SRR17520069.2626872
SRR17520069.2626874
SRR17520069.2626880
SRR17520069.2626882
SRR17520069.2629025
SRR17520069.2630244
SRR17520069.2630246
SRR17520069.2636933
SRR17520069.2636935
SRR17520069.2636937
SRR17520069.2636941
SRR17520069.2636943
SRR17520069.2636945
SRR17520069.2636947
SRR17520069.2636949
SRR17520069.2636951
SRR17520069.2636953
SRR17520069.2636955
SRR17520069.2636957
SRR17520069.2636959
SRR17520069.2636961
SRR17520069.2636963
SRR17520069.2636965
SRR17520069.2636967
SRR17520069.2636969
SRR17520069.2636971
SRR17520069.2636973
SRR17520069.2636975

SRR17520069.2636977
SRR17520069.2636979
SRR17520069.2636981
SRR17520069.2636983
SRR17520069.2636985
SRR17520069.2636987
SRR17520069.2636989
SRR17520069.2636991
SRR17520069.2636993
SRR17520069.2636995
SRR17520069.2638867
SRR17520069.2638869
SRR17520069.2638871
SRR17520069.2638873
SRR17520069.2638875
SRR17520069.2638877
SRR17520069.2638879
SRR17520069.2638881
SRR17520069.2638883
SRR17520069.2638885
SRR17520069.2638887
SRR17520069.2638889
SRR17520069.2638891
SRR17520069.2638893
SRR17520069.2638895
SRR17520069.2638897
SRR17520069.2638899
SRR17520069.2638901
SRR17520069.2638903
SRR17520069.2638905
SRR17520069.2638907
SRR17520069.2638909
SRR17520069.2638911
SRR17520069.2638913
SRR17520069.2638915
SRR17520069.2638917
SRR17520069.2638919
SRR17520069.2638921
SRR17520069.2638923
SRR17520069.2638925
SRR17520069.2638927
SRR17520069.2638929
SRR17520069.2638931
SRR17520069.2638933
SRR17520069.2638935
SRR17520069.2638937
SRR17520069.2638939

SRR17520069.2638941
SRR17520069.2638943
SRR17520069.2638945
SRR17520069.2638947
SRR17520069.2638949
SRR17520069.2638951
SRR17520069.2638953
SRR17520069.2638955
SRR17520069.2638957
SRR17520069.2638959
SRR17520069.2638963
SRR17520069.2638965
SRR17520069.2638967
SRR17520069.2638969
SRR17520069.2638971
SRR17520069.2638973
SRR17520069.2638975
SRR17520069.2638977
SRR17520069.2638979
SRR17520069.2638983
SRR17520069.2642410
SRR17520069.2642412
SRR17520069.2642414
SRR17520069.2642416
SRR17520069.2642418
SRR17520069.2642420
SRR17520069.2642422
SRR17520069.2642424
SRR17520069.2642426
SRR17520069.2642428
SRR17520069.2642430
SRR17520069.2642432
SRR17520069.2642434
SRR17520069.2642436
SRR17520069.2642440
SRR17520069.2642444
SRR17520069.2642446
SRR17520069.2642450
SRR17520069.2642452
SRR17520069.2642454
SRR17520069.2642456
SRR17520069.2642458
SRR17520069.2642460
SRR17520069.2642462
SRR17520069.2642464
SRR17520069.2642466
SRR17520069.2642468

SRR17520069.2642693
SRR17520069.2642695
SRR17520069.2642697
SRR17520069.2642699
SRR17520069.2642701
SRR17520069.2642703
SRR17520069.2645426
SRR17520069.2645428
SRR17520069.2645430
SRR17520069.2645432
SRR17520069.2645434
SRR17520069.2645436
SRR17520069.2645438
SRR17520069.2645440
SRR17520069.2645442
SRR17520069.2645444
SRR17520069.2645446
SRR17520069.2645448
SRR17520069.2645450
SRR17520069.2649972
SRR17520069.2649974
SRR17520069.2649976
SRR17520069.2649978
SRR17520069.2649980
SRR17520069.2649982
SRR17520069.2651267
SRR17520069.2651269
SRR17520069.2651271
SRR17520069.2651273
SRR17520069.2654313
SRR17520069.2654315
SRR17520069.2654317
SRR17520069.2654319
SRR17520069.2654321
SRR17520069.2654323
SRR17520069.2654325
SRR17520069.2654327
SRR17520069.2654329
SRR17520069.2654331
SRR17520069.2654333
SRR17520069.2654335
SRR17520069.2654339
SRR17520069.2654341
SRR17520069.2654343
SRR17520069.2654345
SRR17520069.2654347
SRR17520069.2654349

SRR17520069.2654351
SRR17520069.2654353
SRR17520069.2654355
SRR17520069.2654357
SRR17520069.2654359
SRR17520069.2654361
SRR17520069.2654363
SRR17520069.2654365
SRR17520069.2654367
SRR17520069.2654369
SRR17520069.2654371
SRR17520069.2654373
SRR17520069.2654377
SRR17520069.2654379
SRR17520069.2654381
SRR17520069.2654383
SRR17520069.2672073
SRR17520069.2672075
SRR17520069.2672077
SRR17520069.2672079
SRR17520069.2672081
SRR17520069.2672083
SRR17520069.2672085
SRR17520069.2687116
SRR17520069.2687118
SRR17520069.2687120
SRR17520069.2687122
SRR17520069.2687124
SRR17520069.2687126
SRR17520069.2687128
SRR17520069.2687130
SRR17520069.2687132
SRR17520069.2687134
SRR17520069.2687136
SRR17520069.2687138
SRR17520069.2687140
SRR17520069.2687142
SRR17520069.2687144
SRR17520069.2687146
SRR17520069.2687148
SRR17520069.2687150
SRR17520069.2687152
SRR17520069.2687273
SRR17520069.2687275
SRR17520069.2687277
SRR17520069.2687279
SRR17520069.2687281

SRR17520069.2687283
SRR17520069.2690624
SRR17520069.2690626
SRR17520069.2690628
SRR17520069.2690630
SRR17520069.2690632
SRR17520069.2690634
SRR17520069.2690636
SRR17520069.2690638
SRR17520069.2690640
SRR17520069.2690642
SRR17520069.2690644
SRR17520069.2690646
SRR17520069.2690648
SRR17520069.2690650
SRR17520069.2690652
SRR17520069.2690654
SRR17520069.2690658
SRR17520069.2690660
SRR17520069.2690662
SRR17520069.2690664
SRR17520069.2690666
SRR17520069.2690668
SRR17520069.2690670
SRR17520069.2690672
SRR17520069.2690674
SRR17520069.2690676
SRR17520069.2690678
SRR17520069.2690680
SRR17520069.2690682
SRR17520069.2690684
SRR17520069.2690686
SRR17520069.2690688
SRR17520069.2690690
SRR17520069.2690692
SRR17520069.2690694
SRR17520069.2690696
SRR17520069.2690698
SRR17520069.2690700
SRR17520069.2690702
SRR17520069.2690704
SRR17520069.2690706
SRR17520069.2690708
SRR17520069.2690710
SRR17520069.2690712
SRR17520069.2690714
SRR17520069.2690716

SRR17520069.2690718
SRR17520069.2690720
SRR17520069.2690722
SRR17520069.2690724
SRR17520069.2690726
SRR17520069.2690728
SRR17520069.2690730
SRR17520069.2690732
SRR17520069.2690734
SRR17520069.2690736
SRR17520069.2690738
SRR17520069.2690740
SRR17520069.2690742
SRR17520069.2690744
SRR17520069.2690746
SRR17520069.2690748
SRR17520069.2690750
SRR17520069.2690752
SRR17520069.2690754
SRR17520069.2690758
SRR17520069.2690760
SRR17520069.2690762
SRR17520069.2690766
SRR17520069.2690768
SRR17520069.2690770
SRR17520069.2693260
SRR17520069.2693262
SRR17520069.2693264
SRR17520069.2693266
SRR17520069.2693268
SRR17520069.2693270
SRR17520069.2693272
SRR17520069.2693274
SRR17520069.2693276
SRR17520069.2693708
SRR17520069.2693710
SRR17520069.2693712
SRR17520069.2693714
SRR17520069.2693718
SRR17520069.2693720
SRR17520069.2693722
SRR17520069.2693724
SRR17520069.2693726
SRR17520069.2693728
SRR17520069.2693730
SRR17520069.2693732
SRR17520069.2693734

SRR17520069.2693736
SRR17520069.2693738
SRR17520069.2693740
SRR17520069.2693742
SRR17520069.2693744
SRR17520069.2693746
SRR17520069.2693748
SRR17520069.2693750
SRR17520069.2693752
SRR17520069.2693754
SRR17520069.2693756
SRR17520069.2693758
SRR17520069.2693760
SRR17520069.2693762
SRR17520069.2693764
SRR17520069.2693766
SRR17520069.2693768
SRR17520069.2693770
SRR17520069.2693774
SRR17520069.2693776
SRR17520069.2693778
SRR17520069.2693780
SRR17520069.2693782
SRR17520069.2695724
SRR17520069.2695726
SRR17520069.2695728
SRR17520069.2695732
SRR17520069.2695734
SRR17520069.2695736
SRR17520069.2695738
SRR17520069.2695740
SRR17520069.2695742
SRR17520069.2695744
SRR17520069.2695746
SRR17520069.2695748
SRR17520069.2695750
SRR17520069.2695752
SRR17520069.2695754
SRR17520069.2695756
SRR17520069.2695758
SRR17520069.2695760
SRR17520069.2695762
SRR17520069.2695764
SRR17520069.2695766
SRR17520069.2695768
SRR17520069.2695770
SRR17520069.2700319

SRR17520069.2700321
SRR17520069.2702923
SRR17520069.2702925
SRR17520069.2702927
SRR17520069.2702929
SRR17520069.2702933
SRR17520069.2702935
SRR17520069.2702937
SRR17520069.2702939
SRR17520069.2702941
SRR17520069.2702943
SRR17520069.2702945
SRR17520069.2702947
SRR17520069.2702949
SRR17520069.2702951
SRR17520069.2702953
SRR17520069.2702955
SRR17520069.2702957
SRR17520069.2702959
SRR17520069.2702961
SRR17520069.2702965
SRR17520069.2702967
SRR17520069.2702969
SRR17520069.2702971
SRR17520069.2702973
SRR17520069.2705277
SRR17520069.2709441
SRR17520069.2709443
SRR17520069.2709445
SRR17520069.2709447
SRR17520069.2715464
SRR17520069.2715466
SRR17520069.2715468
SRR17520069.2715470
SRR17520069.2715472
SRR17520069.2715474
SRR17520069.2715478
SRR17520069.2715480
SRR17520069.2715482
SRR17520069.2715484
SRR17520069.2717369
SRR17520069.2717371
SRR17520069.2717373
SRR17520069.2717375
SRR17520069.2717377
SRR17520069.2717379
SRR17520069.2717381

SRR17520069.2717383
SRR17520069.2717385
SRR17520069.2717387
SRR17520069.2717389
SRR17520069.2717717
SRR17520069.2717719
SRR17520069.2717721
SRR17520069.2717723
SRR17520069.2717725
SRR17520069.2717727
SRR17520069.2717729
SRR17520069.2717731
SRR17520069.2717733
SRR17520069.2717735
SRR17520069.2717737
SRR17520069.2731045
SRR17520069.2731047
SRR17520069.2731049
SRR17520069.2733816
SRR17520069.2733818
SRR17520069.2733820
SRR17520069.2733822
SRR17520069.2733824
SRR17520069.2733826
SRR17520069.2733828
SRR17520069.2733830
SRR17520069.2733832
SRR17520069.2733834
SRR17520069.2733836
SRR17520069.2733838
SRR17520069.2733840
SRR17520069.2733842
SRR17520069.2733844
SRR17520069.2733846
SRR17520069.2733848
SRR17520069.2733850
SRR17520069.2733854
SRR17520069.2742437
SRR17520069.2742439
SRR17520069.2742441
SRR17520069.2742443
SRR17520069.2742445
SRR17520069.2742447
SRR17520069.2742449
SRR17520069.2742451
SRR17520069.2742455
SRR17520069.2742457

SRR17520069.2742459
SRR17520069.2742461
SRR17520069.2742463
SRR17520069.2742465
SRR17520069.2742467
SRR17520069.2742469
SRR17520069.2742471
SRR17520069.2742473
SRR17520069.2742477
SRR17520069.2743876
SRR17520069.2743878
SRR17520069.2743880
SRR17520069.2743882
SRR17520069.2743884
SRR17520069.2743886
SRR17520069.2743888
SRR17520069.2743890
SRR17520069.2743894
SRR17520069.2743896
SRR17520069.2743898
SRR17520069.2743900
SRR17520069.2743902
SRR17520069.2743904
SRR17520069.2743906
SRR17520069.2743908
SRR17520069.2743910
SRR17520069.2748844
SRR17520069.2748846
SRR17520069.2748848
SRR17520069.2748850
SRR17520069.2748852
SRR17520069.2748854
SRR17520069.2748856
SRR17520069.2748858
SRR17520069.2748860
SRR17520069.2748862
SRR17520069.2748864
SRR17520069.2748866
SRR17520069.2748868
SRR17520069.2748870
SRR17520069.2748872
SRR17520069.2748874
SRR17520069.2748876
SRR17520069.2748878
SRR17520069.2748880
SRR17520069.2748882
SRR17520069.2748884

SRR17520069.2748886
SRR17520069.2748888
SRR17520069.2748890
SRR17520069.2748892
SRR17520069.2748894
SRR17520069.2748896
SRR17520069.2748898
SRR17520069.2748900
SRR17520069.2748902
SRR17520069.2748904
SRR17520069.2748906
SRR17520069.2748908
SRR17520069.2748910
SRR17520069.2748912
SRR17520069.2748914
SRR17520069.2748916
SRR17520069.2748918
SRR17520069.2748920
SRR17520069.2748922
SRR17520069.2748924
SRR17520069.2748926
SRR17520069.2748928
SRR17520069.2748930
SRR17520069.2748932
SRR17520069.2748934
SRR17520069.2748936
SRR17520069.2752804
SRR17520069.2754324
SRR17520069.2754326
SRR17520069.2754328
SRR17520069.2754330
SRR17520069.2754332
SRR17520069.2754334
SRR17520069.2754336
SRR17520069.2754338
SRR17520069.2754340
SRR17520069.2754342
SRR17520069.2754344
SRR17520069.2754346
SRR17520069.2754348
SRR17520069.2754350
SRR17520069.2758388
SRR17520069.2758390
SRR17520069.2758392
SRR17520069.2758394
SRR17520069.2758396
SRR17520069.2758398

SRR17520069.2758400
SRR17520069.2758402
SRR17520069.2758404
SRR17520069.2758406
SRR17520069.2758408
SRR17520069.2758410
SRR17520069.2758412
SRR17520069.2758414
SRR17520069.2758416
SRR17520069.2758418
SRR17520069.2758420
SRR17520069.2758422
SRR17520069.2758424
SRR17520069.2758426
SRR17520069.2758428
SRR17520069.2758430
SRR17520069.2758434
SRR17520069.2758436
SRR17520069.2758438
SRR17520069.2758440
SRR17520069.2758442
SRR17520069.2758444
SRR17520069.2758446
SRR17520069.2758448
SRR17520069.2758450
SRR17520069.2758452
SRR17520069.2758454
SRR17520069.2758456
SRR17520069.2758458
SRR17520069.2758460
SRR17520069.2758462
SRR17520069.2758464
SRR17520069.2758466
SRR17520069.2758468
SRR17520069.2758470
SRR17520069.2760955
SRR17520069.2760957
SRR17520069.2760959
SRR17520069.2760961
SRR17520069.2773400
SRR17520069.2773402
SRR17520069.2773404
SRR17520069.2773432
SRR17520069.2773434
SRR17520069.2773436
SRR17520069.2773438
SRR17520069.2773440

SRR17520069.2773442
SRR17520069.2773444
SRR17520069.2773446
SRR17520069.2773448
SRR17520069.2773450
SRR17520069.2773452
SRR17520069.2773454
SRR17520069.2773456
SRR17520069.2773458
SRR17520069.2773460
SRR17520069.2773462
SRR17520069.2773464
SRR17520069.2773466
SRR17520069.2773468
SRR17520069.2773470
SRR17520069.2773472
SRR17520069.2773474
SRR17520069.2773476
SRR17520069.2777385
SRR17520069.2777387
SRR17520069.2777389
SRR17520069.2777393
SRR17520069.2777395
SRR17520069.2777397
SRR17520069.2777401
SRR17520069.2777403
SRR17520069.2777405
SRR17520069.2777407
SRR17520069.2777409
SRR17520069.2778825
SRR17520069.2778827
SRR17520069.2778829
SRR17520069.2778831
SRR17520069.2778833
SRR17520069.2778835
SRR17520069.2778837
SRR17520069.2778841
SRR17520069.2778843
SRR17520069.2778845
SRR17520069.2778847
SRR17520069.2778849
SRR17520069.2778851
SRR17520069.2778855
SRR17520069.2778857
SRR17520069.2778859
SRR17520069.2778861
SRR17520069.2778863

SRR17520069.2778865
SRR17520069.2778867
SRR17520069.2778869
SRR17520069.2778871
SRR17520069.2778873
SRR17520069.2778875
SRR17520069.2778877
SRR17520069.2778879
SRR17520069.2778881
SRR17520069.2778883
SRR17520069.2778885
SRR17520069.2778887
SRR17520069.2778889
SRR17520069.2778891
SRR17520069.2778893
SRR17520069.2778895
SRR17520069.2778897
SRR17520069.2778899
SRR17520069.2778901
SRR17520069.2778903
SRR17520069.2778905
SRR17520069.2778907
SRR17520069.2778909
SRR17520069.2778911
SRR17520069.2778913
SRR17520069.2778915
SRR17520069.2778917
SRR17520069.2780697
SRR17520069.2781766
SRR17520069.2781768
SRR17520069.2781770
SRR17520069.2781772
SRR17520069.2781774
SRR17520069.2781805
SRR17520069.2781807
SRR17520069.2781809
SRR17520069.2781811
SRR17520069.2781813
SRR17520069.2781815
SRR17520069.2781817
SRR17520069.2781819
SRR17520069.2781821
SRR17520069.2781823
SRR17520069.2781825
SRR17520069.2781827
SRR17520069.2781829
SRR17520069.2781831

SRR17520069.2781833
SRR17520069.2782895
SRR17520069.2782897
SRR17520069.2782899
SRR17520069.2785654
SRR17520069.2785656
SRR17520069.2785658
SRR17520069.2785660
SRR17520069.2785662
SRR17520069.2785664
SRR17520069.2785666
SRR17520069.2785668
SRR17520069.2785670
SRR17520069.2785672
SRR17520069.2785674
SRR17520069.2785676
SRR17520069.2785678
SRR17520069.2785680
SRR17520069.2785682
SRR17520069.2785684
SRR17520069.2785686
SRR17520069.2785688
SRR17520069.2785690
SRR17520069.2785692
SRR17520069.2785694
SRR17520069.2785696
SRR17520069.2785698
SRR17520069.2785700
SRR17520069.2785702
SRR17520069.2785704
SRR17520069.2785706
SRR17520069.2785708
SRR17520069.2785710
SRR17520069.2785712
SRR17520069.2785714
SRR17520069.2785716
SRR17520069.2785718
SRR17520069.2785720
SRR17520069.2785722
SRR17520069.2790822
SRR17520069.2790824
SRR17520069.2790828
SRR17520069.2790830
SRR17520069.2790832
SRR17520069.2790834
SRR17520069.2790836
SRR17520069.2790838

SRR17520069.2790840
SRR17520069.2790842
SRR17520069.2790844
SRR17520069.2790846
SRR17520069.2790848
SRR17520069.2790850
SRR17520069.2790852
SRR17520069.2790854
SRR17520069.2790856
SRR17520069.2790858
SRR17520069.2790860
SRR17520069.2790862
SRR17520069.2790864
SRR17520069.2790866
SRR17520069.2790868
SRR17520069.2790870
SRR17520069.2790872
SRR17520069.2804929
SRR17520069.2804944
SRR17520069.2814136
SRR17520069.2814138
SRR17520069.2814140
SRR17520069.2814142
SRR17520069.2814144
SRR17520069.2814146
SRR17520069.2814148
SRR17520069.2814150
SRR17520069.2814152
SRR17520069.2814154
SRR17520069.2814156
SRR17520069.2814158
SRR17520069.2814160
SRR17520069.2814162
SRR17520069.2814164
SRR17520069.2814166
SRR17520069.2814168
SRR17520069.2814170
SRR17520069.2814172
SRR17520069.2814174
SRR17520069.2814176
SRR17520069.2814178
SRR17520069.2814180
SRR17520069.2814182
SRR17520069.2814184
SRR17520069.2814186
SRR17520069.2814188
SRR17520069.2814190

SRR17520069.2814192
SRR17520069.2814194
SRR17520069.2814196
SRR17520069.2814198
SRR17520069.2814200
SRR17520069.2814202
SRR17520069.2814204
SRR17520069.2814206
SRR17520069.2816633
SRR17520069.2820625
SRR17520069.2823735
SRR17520069.2823737
SRR17520069.2823739
SRR17520069.2823741
SRR17520069.2823743
SRR17520069.2823745
SRR17520069.2836143
SRR17520069.2836145
SRR17520069.2836147
SRR17520069.2836149
SRR17520069.2836151
SRR17520069.2845384
SRR17520069.2845386
SRR17520069.2845388
SRR17520069.2845390
SRR17520069.2845392
SRR17520069.2845394
SRR17520069.2845396
SRR17520069.2845398
SRR17520069.2845400
SRR17520069.2845402
SRR17520069.2845404
SRR17520069.2845406
SRR17520069.2845408
SRR17520069.2845410
SRR17520069.2845412
SRR17520069.2845414
SRR17520069.2845416
SRR17520069.2845418
SRR17520069.2845420
SRR17520069.2845422
SRR17520069.2845426
SRR17520069.2845428
SRR17520069.2845430
SRR17520069.2845432
SRR17520069.2845434
SRR17520069.2845436

SRR17520069.2845438
SRR17520069.2845440
SRR17520069.2845442
SRR17520069.2845444
SRR17520069.2845446
SRR17520069.2845448
SRR17520069.2845450
SRR17520069.2845452
SRR17520069.2845454
SRR17520069.2845456
SRR17520069.2845458
SRR17520069.2845460
SRR17520069.2845462
SRR17520069.2845464
SRR17520069.2845466
SRR17520069.2845468
SRR17520069.2845470
SRR17520069.2845472
SRR17520069.2845474
SRR17520069.2845476
SRR17520069.2847034
SRR17520069.2847036
SRR17520069.2847038
SRR17520069.2847040
SRR17520069.2847042
SRR17520069.2847044
SRR17520069.2847046
SRR17520069.2847048
SRR17520069.2847050
SRR17520069.2847052
SRR17520069.2847054
SRR17520069.2847056
SRR17520069.2847060
SRR17520069.2847062
SRR17520069.2847064
SRR17520069.2847066
SRR17520069.2847068
SRR17520069.2847070
SRR17520069.2847072
SRR17520069.2847074
SRR17520069.2847076
SRR17520069.2847078
SRR17520069.2847080
SRR17520069.2855069
SRR17520069.2855071
SRR17520069.2856968
SRR17520069.2858004

SRR17520069.2858006
SRR17520069.2858008
SRR17520069.2858010
SRR17520069.2858012
SRR17520069.2858014
SRR17520069.2858016
SRR17520069.2858018
SRR17520069.2858020
SRR17520069.2858022
SRR17520069.2858024
SRR17520069.2858026
SRR17520069.2858028
SRR17520069.2858030
SRR17520069.2858032
SRR17520069.2858034
SRR17520069.2858036
SRR17520069.2858038
SRR17520069.2858040
SRR17520069.2858042
SRR17520069.2858044
SRR17520069.2858046
SRR17520069.2858048
SRR17520069.2858050
SRR17520069.2858052
SRR17520069.2858056
SRR17520069.2858058
SRR17520069.2858060
SRR17520069.2861749
SRR17520069.2861751
SRR17520069.2861753
SRR17520069.2861755
SRR17520069.2861757
SRR17520069.2861759
SRR17520069.2861761
SRR17520069.2861763
SRR17520069.2861765
SRR17520069.2861767
SRR17520069.2861769
SRR17520069.2861771
SRR17520069.2861773
SRR17520069.2861775
SRR17520069.2861777
SRR17520069.2861779
SRR17520069.2861781
SRR17520069.2861783
SRR17520069.2861785
SRR17520069.2861787

SRR17520069.2861789
SRR17520069.2861791
SRR17520069.2861793
SRR17520069.2861795
SRR17520069.2861797
SRR17520069.2861799
SRR17520069.2861801
SRR17520069.2861803
SRR17520069.2861805
SRR17520069.2861807
SRR17520069.2861809
SRR17520069.2861811
SRR17520069.2861813
SRR17520069.2861815
SRR17520069.2861817
SRR17520069.2861819
SRR17520069.2862869
SRR17520069.2862871
SRR17520069.2862873
SRR17520069.2862877
SRR17520069.2862879
SRR17520069.2862881
SRR17520069.2862883
SRR17520069.2862885
SRR17520069.2870755
SRR17520069.2871349
SRR17520069.2871351
SRR17520069.2871353
SRR17520069.2871355
SRR17520069.2871357
SRR17520069.2871359
SRR17520069.2871363
SRR17520069.2873140
SRR17520069.2873142
SRR17520069.2873144
SRR17520069.2873146
SRR17520069.2873148
SRR17520069.2873150
SRR17520069.2873154
SRR17520069.2873158
SRR17520069.2873160
SRR17520069.2873164
SRR17520069.2873166
SRR17520069.2873168
SRR17520069.2873170
SRR17520069.2873172
SRR17520069.2873174

SRR17520069.2873176
SRR17520069.2873178
SRR17520069.2873180
SRR17520069.2873182
SRR17520069.2873186
SRR17520069.2873188
SRR17520069.2873192
SRR17520069.2873194
SRR17520069.2873196
SRR17520069.2873198
SRR17520069.2873200
SRR17520069.2873202
SRR17520069.2873204
SRR17520069.2873206
SRR17520069.2875001
SRR17520069.2875003
SRR17520069.2875005
SRR17520069.2875007
SRR17520069.2875009
SRR17520069.2875011
SRR17520069.2875013
SRR17520069.2875015
SRR17520069.2875017
SRR17520069.2875019
SRR17520069.2875021
SRR17520069.2875023
SRR17520069.2875025
SRR17520069.2875029
SRR17520069.2875031
SRR17520069.2875033
SRR17520069.2875035
SRR17520069.2875037
SRR17520069.2875039
SRR17520069.2875041
SRR17520069.2875043
SRR17520069.2875045
SRR17520069.2875047
SRR17520069.2877842
SRR17520069.2877844
SRR17520069.2877846
SRR17520069.2877848
SRR17520069.2877850
SRR17520069.2877852
SRR17520069.2877854
SRR17520069.2877856
SRR17520069.2877858
SRR17520069.2877862

SRR17520069.2877864
SRR17520069.2877866
SRR17520069.2877868
SRR17520069.2877870
SRR17520069.2877872
SRR17520069.2877874
SRR17520069.2877876
SRR17520069.2877878
SRR17520069.2877880
SRR17520069.2877882
SRR17520069.2877884
SRR17520069.2877886
SRR17520069.2877888
SRR17520069.2877890
SRR17520069.2877892
SRR17520069.2877894
SRR17520069.2877896
SRR17520069.2877898
SRR17520069.2877900
SRR17520069.2877902
SRR17520069.2877904
SRR17520069.2878700
SRR17520069.2878702
SRR17520069.2880656
SRR17520069.2880658
SRR17520069.2880660
SRR17520069.2880662
SRR17520069.2880664
SRR17520069.2889372
SRR17520069.2889374
SRR17520069.2889378
SRR17520069.2889380
SRR17520069.2891109
SRR17520069.2891111
SRR17520069.2891113
SRR17520069.2891115
SRR17520069.2891117
SRR17520069.2891119
SRR17520069.2891121
SRR17520069.2891123
SRR17520069.2891125
SRR17520069.2891127
SRR17520069.2891129
SRR17520069.2891131
SRR17520069.2891133
SRR17520069.2891135
SRR17520069.2891137

SRR17520069.2891139
SRR17520069.2891141
SRR17520069.2891143
SRR17520069.2891145
SRR17520069.2891147
SRR17520069.2891149
SRR17520069.2891151
SRR17520069.2891153
SRR17520069.2891155
SRR17520069.2891157
SRR17520069.2891159
SRR17520069.2891161
SRR17520069.2891163
SRR17520069.2891165
SRR17520069.2891167
SRR17520069.2891169
SRR17520069.2891171
SRR17520069.2891173
SRR17520069.2891814
SRR17520069.2891816
SRR17520069.2891818
SRR17520069.2891820
SRR17520069.2891822
SRR17520069.2891824
SRR17520069.2891826
SRR17520069.2891828
SRR17520069.2891830
SRR17520069.2891832
SRR17520069.2891834
SRR17520069.2891836
SRR17520069.2891838
SRR17520069.2891840
SRR17520069.2891844
SRR17520069.2891846
SRR17520069.2891848
SRR17520069.2891850
SRR17520069.2891852
SRR17520069.2891854
SRR17520069.2891856
SRR17520069.2892498
SRR17520069.2892500
SRR17520069.2892502
SRR17520069.2894208
SRR17520069.2894210
SRR17520069.2895602
SRR17520069.2895604
SRR17520069.2895606

SRR17520069.2895608
SRR17520069.2895610
SRR17520069.2895612
SRR17520069.2895614
SRR17520069.2895616
SRR17520069.2895618
SRR17520069.2895620
SRR17520069.2895622
SRR17520069.2895626
SRR17520069.2895628
SRR17520069.2895630
SRR17520069.2895632
SRR17520069.2895634
SRR17520069.2895636
SRR17520069.2895638
SRR17520069.2895640
SRR17520069.2895642
SRR17520069.2895644
SRR17520069.2898562
SRR17520069.2898564
SRR17520069.2898566
SRR17520069.2898568
SRR17520069.2898572
SRR17520069.2898574
SRR17520069.2898576
SRR17520069.2898578
SRR17520069.2904835
SRR17520069.2908919
SRR17520069.2908921
SRR17520069.2908923
SRR17520069.2908925
SRR17520069.2908929
SRR17520069.2908931
SRR17520069.2908935
SRR17520069.2908937
SRR17520069.2908939
SRR17520069.2908943
SRR17520069.2908945
SRR17520069.2908947
SRR17520069.2908949
SRR17520069.2908951
SRR17520069.2908953
SRR17520069.2908955
SRR17520069.2908957
SRR17520069.2908959
SRR17520069.2908961
SRR17520069.2908963

SRR17520069.2908965
SRR17520069.2908969
SRR17520069.2908971
SRR17520069.2908973
SRR17520069.2908977
SRR17520069.2908979
SRR17520069.2908983
SRR17520069.2908985
SRR17520069.2908987
SRR17520069.2908989
SRR17520069.2908991
SRR17520069.2908993
SRR17520069.2908995
SRR17520069.2908997
SRR17520069.2908999
SRR17520069.2909001
SRR17520069.2909003
SRR17520069.2909005
SRR17520069.2909007
SRR17520069.2909009
SRR17520069.2909011
SRR17520069.2911489
SRR17520069.2911491
SRR17520069.2911493
SRR17520069.2911497
SRR17520069.2911499
SRR17520069.2911501
SRR17520069.2911503
SRR17520069.2911505
SRR17520069.2911507
SRR17520069.2911509
SRR17520069.2911511
SRR17520069.2911513
SRR17520069.2913975
SRR17520069.2913977
SRR17520069.2913979
SRR17520069.2913981
SRR17520069.2913983
SRR17520069.2913985
SRR17520069.2913987
SRR17520069.2913989
SRR17520069.2913991
SRR17520069.2913993
SRR17520069.2913995
SRR17520069.2913997
SRR17520069.2913999
SRR17520069.2914001

SRR17520069.2914003
SRR17520069.2914005
SRR17520069.2914007
SRR17520069.2914009
SRR17520069.2914011
SRR17520069.2914013
SRR17520069.2914015
SRR17520069.2914017
SRR17520069.2914019
SRR17520069.2914021
SRR17520069.2914023
SRR17520069.2914025
SRR17520069.2914027
SRR17520069.2914029
SRR17520069.2914031
SRR17520069.2914033
SRR17520069.2914035
SRR17520069.2914037
SRR17520069.2914039
SRR17520069.2914041
SRR17520069.2914043
SRR17520069.2914045
SRR17520069.2914047
SRR17520069.2914049
SRR17520069.2914051
SRR17520069.2914053
SRR17520069.2914055
SRR17520069.2914057
SRR17520069.2914059
SRR17520069.2914061
SRR17520069.2914063
SRR17520069.2914065
SRR17520069.2914067
SRR17520069.2914069
SRR17520069.2914071
SRR17520069.2914073
SRR17520069.2914075
SRR17520069.2914077
SRR17520069.2914079
SRR17520069.2915194
SRR17520069.2915196
SRR17520069.2915198
SRR17520069.2915200
SRR17520069.2915204
SRR17520069.2915208
SRR17520069.2915210
SRR17520069.2915212

SRR17520069.2915214
SRR17520069.2915216
SRR17520069.2915218
SRR17520069.2915222
SRR17520069.2915224
SRR17520069.2915230
SRR17520069.2915232
SRR17520069.2915234
SRR17520069.2915236
SRR17520069.2915238
SRR17520069.2915240
SRR17520069.2915242
SRR17520069.2915246
SRR17520069.2915248
SRR17520069.2915252
SRR17520069.2916773
SRR17520069.2916777
SRR17520069.2916779
SRR17520069.2916781
SRR17520069.2916783
SRR17520069.2916785
SRR17520069.2916787
SRR17520069.2916789
SRR17520069.2916791
SRR17520069.2916793
SRR17520069.2916795
SRR17520069.2916797
SRR17520069.2916799
SRR17520069.2916801
SRR17520069.2916803
SRR17520069.2916805
SRR17520069.2916807
SRR17520069.2916811
SRR17520069.2916815
SRR17520069.2916817
SRR17520069.2916819
SRR17520069.2916823
SRR17520069.2916825
SRR17520069.2916827
SRR17520069.2916829
SRR17520069.2916831
SRR17520069.2916833
SRR17520069.2916835
SRR17520069.2916839
SRR17520069.2916841
SRR17520069.2916845
SRR17520069.2916849

SRR17520069.2916851
SRR17520069.2916853
SRR17520069.2916855
SRR17520069.2916859
SRR17520069.2916861
SRR17520069.2916863
SRR17520069.2917995
SRR17520069.2917997
SRR17520069.2917999
SRR17520069.2918001
SRR17520069.2918003
SRR17520069.2918005
SRR17520069.2918007
SRR17520069.2918009
SRR17520069.2923254
SRR17520069.2925671
SRR17520069.2925673
SRR17520069.2925675
SRR17520069.2925677
SRR17520069.2925679
SRR17520069.2925681
SRR17520069.2925683
SRR17520069.2925685
SRR17520069.2925687
SRR17520069.2925689
SRR17520069.2925691
SRR17520069.2925693
SRR17520069.2925695
SRR17520069.2925697
SRR17520069.2925699
SRR17520069.2927333
SRR17520069.2927335
SRR17520069.2932278
SRR17520069.2932280
SRR17520069.2932282
SRR17520069.2932286
SRR17520069.2932288
SRR17520069.2932290
SRR17520069.2932292
SRR17520069.2932294
SRR17520069.2932296
SRR17520069.2932298
SRR17520069.2932300
SRR17520069.2932302
SRR17520069.2932304
SRR17520069.2932306
SRR17520069.2932308

SRR17520069.2932310
SRR17520069.2932312
SRR17520069.2932314
SRR17520069.2932318
SRR17520069.2932320
SRR17520069.2932322
SRR17520069.2932324
SRR17520069.2932326
SRR17520069.2932328
SRR17520069.2932330
SRR17520069.2932332
SRR17520069.2932334
SRR17520069.2932336
SRR17520069.2932338
SRR17520069.2932340
SRR17520069.2932342
SRR17520069.2932344
SRR17520069.2932346
SRR17520069.2935160
SRR17520069.2935162
SRR17520069.2935164
SRR17520069.2935166
SRR17520069.2935168
SRR17520069.2935170
SRR17520069.2935172
SRR17520069.2935174
SRR17520069.2935176
SRR17520069.2935178
SRR17520069.2935180
SRR17520069.2935182
SRR17520069.2935184
SRR17520069.2935186
SRR17520069.2935188
SRR17520069.2935192
SRR17520069.2935194
SRR17520069.2936115
SRR17520069.2936117
SRR17520069.2936119
SRR17520069.2936121
SRR17520069.2936123
SRR17520069.2936125
SRR17520069.2936127
SRR17520069.2936129
SRR17520069.2936131
SRR17520069.2936133
SRR17520069.2936135
SRR17520069.2936137

SRR17520069.2936139
SRR17520069.2936141
SRR17520069.2938057
SRR17520069.2938059
SRR17520069.2938061
SRR17520069.2938607
SRR17520069.2938609
SRR17520069.2938611
SRR17520069.2939602
SRR17520069.2943895
SRR17520069.2943897
SRR17520069.2943899
SRR17520069.2943901
SRR17520069.2943903
SRR17520069.2943905
SRR17520069.2943907
SRR17520069.2943909
SRR17520069.2943911
SRR17520069.2943913
SRR17520069.2943915
SRR17520069.2943917
SRR17520069.2943919
SRR17520069.2943921
SRR17520069.2943923
SRR17520069.2943925
SRR17520069.2943927
SRR17520069.2943929
SRR17520069.2943931
SRR17520069.2943933
SRR17520069.2943935
SRR17520069.2943937
SRR17520069.2943939
SRR17520069.2943941
SRR17520069.2943943
SRR17520069.2943945
SRR17520069.2943947
SRR17520069.2943949
SRR17520069.2943951
SRR17520069.2943953
SRR17520069.2943955
SRR17520069.2943957
SRR17520069.2943959
SRR17520069.2943961
SRR17520069.2943963
SRR17520069.2943965
SRR17520069.2943967
SRR17520069.2943969

SRR17520069.2943971
SRR17520069.2943973
SRR17520069.2943975
SRR17520069.2943977
SRR17520069.2943979
SRR17520069.2943981
SRR17520069.2943983
SRR17520069.2943985
SRR17520069.2943987
SRR17520069.2943989
SRR17520069.2943991
SRR17520069.2943993
SRR17520069.2943995
SRR17520069.2947977
SRR17520069.2947979
SRR17520069.2947981
SRR17520069.2953554
SRR17520069.2953556
SRR17520069.2953558
SRR17520069.2953560
SRR17520069.2953562
SRR17520069.2953566
SRR17520069.2953568
SRR17520069.2953570
SRR17520069.2953572
SRR17520069.2953574
SRR17520069.2953576
SRR17520069.2953590
SRR17520069.2953596
SRR17520069.2957105
SRR17520069.2957107
SRR17520069.2957109
SRR17520069.2957113
SRR17520069.2957115
SRR17520069.2957117
SRR17520069.2957119
SRR17520069.2957121
SRR17520069.2957123
SRR17520069.2957125
SRR17520069.2957127
SRR17520069.2957129
SRR17520069.2957131
SRR17520069.2957137
SRR17520069.2957139
SRR17520069.2957143
SRR17520069.2957145
SRR17520069.2957147

SRR17520069.2957149
SRR17520069.2957151
SRR17520069.2957153
SRR17520069.2957155
SRR17520069.2964951
SRR17520069.2964953
SRR17520069.2965833
SRR17520069.2965835
SRR17520069.2965837
SRR17520069.2965839
SRR17520069.2965841
SRR17520069.2965843
SRR17520069.2965845
SRR17520069.2965847
SRR17520069.2965849
SRR17520069.2965851
SRR17520069.2965853
SRR17520069.2965855
SRR17520069.2965857
SRR17520069.2965859
SRR17520069.2965861
SRR17520069.2965863
SRR17520069.2965865
SRR17520069.2965867
SRR17520069.2965869
SRR17520069.2965871
SRR17520069.2965873
SRR17520069.2965875
SRR17520069.2965877
SRR17520069.2965879
SRR17520069.2965881
SRR17520069.2965883
SRR17520069.2965885
SRR17520069.2965887
SRR17520069.2965889
SRR17520069.2965891
SRR17520069.2965893
SRR17520069.2965895
SRR17520069.2965897
SRR17520069.2965899
SRR17520069.2965901
SRR17520069.2965903
SRR17520069.2965905
SRR17520069.2965907
SRR17520069.2965909
SRR17520069.2965911
SRR17520069.2965913

SRR17520069.2965915
SRR17520069.2965917
SRR17520069.2965919
SRR17520069.2965921
SRR17520069.2965923
SRR17520069.2965925
SRR17520069.2965927
SRR17520069.2965929
SRR17520069.2965931
SRR17520069.2965933
SRR17520069.2965935
SRR17520069.2965937
SRR17520069.2965939
SRR17520069.2965941
SRR17520069.2965943
SRR17520069.2965945
SRR17520069.2965947
SRR17520069.2967915
SRR17520069.2967917
SRR17520069.2967921
SRR17520069.2967923
SRR17520069.2967925
SRR17520069.2967927
SRR17520069.2967929
SRR17520069.2967931
SRR17520069.2967933
SRR17520069.2967935
SRR17520069.2967937
SRR17520069.2967939
SRR17520069.2967941
SRR17520069.2967943
SRR17520069.2967945
SRR17520069.2967947
SRR17520069.2967949
SRR17520069.2967951
SRR17520069.2967953
SRR17520069.2967955
SRR17520069.2981336
SRR17520069.2981338
SRR17520069.2985837
SRR17520069.2985839
SRR17520069.2985841
SRR17520069.2985843
SRR17520069.2985847
SRR17520069.2985849
SRR17520069.2985851
SRR17520069.2986134

SRR17520069.2986136
SRR17520069.2986138
SRR17520069.2986140
SRR17520069.2986142
SRR17520069.2986144
SRR17520069.2986146
SRR17520069.2986148
SRR17520069.2986150
SRR17520069.2986152
SRR17520069.2986154
SRR17520069.2986156
SRR17520069.2986158
SRR17520069.2986160
SRR17520069.2986162
SRR17520069.2999864
SRR17520069.2999872
SRR17520069.2999874
SRR17520069.2999876
SRR17520069.2999878
SRR17520069.2999880
SRR17520069.2999882
SRR17520069.2999884
SRR17520069.2999886
SRR17520069.2999888
SRR17520069.2999890
SRR17520069.2999892
SRR17520069.2999896
SRR17520069.2999898
SRR17520069.2999900
SRR17520069.2999902
SRR17520069.2999904
SRR17520069.3006789
SRR17520069.3006791
SRR17520069.3006793
SRR17520069.3006795
SRR17520069.3006797
SRR17520069.3006799
SRR17520069.3006801
SRR17520069.3006803
SRR17520069.3006805
SRR17520069.3006807
SRR17520069.3006813
SRR17520069.3006825
SRR17520069.3006827
SRR17520069.3006829
SRR17520069.3007318
SRR17520069.3007320

SRR17520069.3007322
SRR17520069.3007324
SRR17520069.3007326
SRR17520069.3007328
SRR17520069.3007330
SRR17520069.3007332
SRR17520069.3007334
SRR17520069.3011190
SRR17520069.3011192
SRR17520069.3011194
SRR17520069.3011196
SRR17520069.3011198
SRR17520069.3011200
SRR17520069.3011202
SRR17520069.3011204
SRR17520069.3011206
SRR17520069.3011208
SRR17520069.3011210
SRR17520069.3011212
SRR17520069.3011214
SRR17520069.3011216
SRR17520069.3011218
SRR17520069.3011220
SRR17520069.3011222
SRR17520069.3011224
SRR17520069.3011226
SRR17520069.3011228
SRR17520069.3011230
SRR17520069.3011232
SRR17520069.3011234
SRR17520069.3011236
SRR17520069.3011238
SRR17520069.3011240
SRR17520069.3011242
SRR17520069.3011244
SRR17520069.3011246
SRR17520069.3011248
SRR17520069.3011250
SRR17520069.3011252
SRR17520069.3011254
SRR17520069.3011256
SRR17520069.3011258
SRR17520069.3011260
SRR17520069.3011262
SRR17520069.3011264
SRR17520069.3011266
SRR17520069.3011268

SRR17520069.3011270
SRR17520069.3011272
SRR17520069.3011274
SRR17520069.3011276
SRR17520069.3011992
SRR17520069.3011994
SRR17520069.3011996
SRR17520069.3011998
SRR17520069.3012000
SRR17520069.3012002
SRR17520069.3012004
SRR17520069.3012006
SRR17520069.3012008
SRR17520069.3012010
SRR17520069.3012012
SRR17520069.3012014
SRR17520069.3012016
SRR17520069.3012018
SRR17520069.3012020
SRR17520069.3012022
SRR17520069.3012024
SRR17520069.3012026
SRR17520069.3012028
SRR17520069.3012030
SRR17520069.3012032
SRR17520069.3012034
SRR17520069.3012036
SRR17520069.3012038
SRR17520069.3012040
SRR17520069.3012042
SRR17520069.3012044
SRR17520069.3012046
SRR17520069.3012048
SRR17520069.3012050
SRR17520069.3012052
SRR17520069.3012054
SRR17520069.3012056
SRR17520069.3012058
SRR17520069.3012060
SRR17520069.3012062
SRR17520069.3012064
SRR17520069.3012066
SRR17520069.3012068
SRR17520069.3012070
SRR17520069.3012072
SRR17520069.3012074
SRR17520069.3012076

SRR17520069.3012078
SRR17520069.3012080
SRR17520069.3012082
SRR17520069.3012084
SRR17520069.3012086
SRR17520069.3012088
SRR17520069.3012617
SRR17520069.3012619
SRR17520069.3017753
SRR17520069.3017755
SRR17520069.3017757
SRR17520069.3017759
SRR17520069.3017761
SRR17520069.3017763
SRR17520069.3017765
SRR17520069.3017767
SRR17520069.3017769
SRR17520069.3017771
SRR17520069.3017773
SRR17520069.3017775
SRR17520069.3017779
SRR17520069.3017781
SRR17520069.3017783
SRR17520069.3017785
SRR17520069.3017787
SRR17520069.3017789
SRR17520069.3017791
SRR17520069.3017793
SRR17520069.3017795
SRR17520069.3017797
SRR17520069.3017799
SRR17520069.3017801
SRR17520069.3017803
SRR17520069.3017805
SRR17520069.3017807
SRR17520069.3017809
SRR17520069.3017811
SRR17520069.3017813
SRR17520069.3017815
SRR17520069.3017817
SRR17520069.3017819
SRR17520069.3017821
SRR17520069.3017823
SRR17520069.3017825
SRR17520069.3017827
SRR17520069.3017829
SRR17520069.3017831

SRR17520069.3017833
SRR17520069.3017835
SRR17520069.3017837
SRR17520069.3017839
SRR17520069.3017841
SRR17520069.3017843
SRR17520069.3017845
SRR17520069.3017847
SRR17520069.3017849
SRR17520069.3017851
SRR17520069.3017853
SRR17520069.3017855
SRR17520069.3017857
SRR17520069.3017859
SRR17520069.3017861
SRR17520069.3017863
SRR17520069.3017865
SRR17520069.3017867
SRR17520069.3017869
SRR17520069.3017871
SRR17520069.3017873
SRR17520069.3017875
SRR17520069.3017877
SRR17520069.3017879
SRR17520069.3020134
SRR17520069.3020136
SRR17520069.3020138
SRR17520069.3020140
SRR17520069.3020142
SRR17520069.3020144
SRR17520069.3020146
SRR17520069.3020148
SRR17520069.3032050
SRR17520069.3032052
SRR17520069.3032054
SRR17520069.3032056
SRR17520069.3032779
SRR17520069.3032781
SRR17520069.3032783
SRR17520069.3032785
SRR17520069.3032789
SRR17520069.3032791
SRR17520069.3032793
SRR17520069.3032795
SRR17520069.3032797
SRR17520069.3032799
SRR17520069.3032801

SRR17520069.3032803
SRR17520069.3032805
SRR17520069.3032807
SRR17520069.3036622
SRR17520069.3042220
SRR17520069.3042222
SRR17520069.3042224
SRR17520069.3042226
SRR17520069.3042228
SRR17520069.3042230
SRR17520069.3044016
SRR17520069.3044018
SRR17520069.3044020
SRR17520069.3044022
SRR17520069.3044024
SRR17520069.3044026
SRR17520069.3044028
SRR17520069.3044030
SRR17520069.3044032
SRR17520069.3044034
SRR17520069.3044036
SRR17520069.3044038
SRR17520069.3044040
SRR17520069.3044042
SRR17520069.3044044
SRR17520069.3044046
SRR17520069.3044048
SRR17520069.3044054
SRR17520069.3044058
SRR17520069.3044060
SRR17520069.3044062
SRR17520069.3044064
SRR17520069.3044066
SRR17520069.3044068
SRR17520069.3044070
SRR17520069.3044072
SRR17520069.3044074
SRR17520069.3044076
SRR17520069.3044078
SRR17520069.3044082
SRR17520069.3044084
SRR17520069.3044086
SRR17520069.3044088
SRR17520069.3044090
SRR17520069.3044092
SRR17520069.3044094
SRR17520069.3044096

SRR17520069.3044098
SRR17520069.3044100
SRR17520069.3044102
SRR17520069.3044104
SRR17520069.3044106
SRR17520069.3044108
SRR17520069.3044110
SRR17520069.3044112
SRR17520069.3044114
SRR17520069.3044116
SRR17520069.3044118
SRR17520069.3044120
SRR17520069.3044124
SRR17520069.3044126
SRR17520069.3044128
SRR17520069.3044130
SRR17520069.3044132
SRR17520069.3044134
SRR17520069.3044136
SRR17520069.3044138
SRR17520069.3044140
SRR17520069.3044142
SRR17520069.3044144
SRR17520069.3044146
SRR17520069.3044148
SRR17520069.3044150
SRR17520069.3046820
SRR17520069.3046822
SRR17520069.3046824
SRR17520069.3046826
SRR17520069.3046828
SRR17520069.3046830
SRR17520069.3046832
SRR17520069.3046834
SRR17520069.3046836
SRR17520069.3046838
SRR17520069.3046840
SRR17520069.3046842
SRR17520069.3046844
SRR17520069.3046846
SRR17520069.3046848
SRR17520069.3046850
SRR17520069.3046852
SRR17520069.3046854
SRR17520069.3046856
SRR17520069.3046858
SRR17520069.3048497

SRR17520069.3053463
SRR17520069.3053465
SRR17520069.3053467
SRR17520069.3053469
SRR17520069.3053471
SRR17520069.3053473
SRR17520069.3053475
SRR17520069.3053477
SRR17520069.3053479
SRR17520069.3053481
SRR17520069.3053483
SRR17520069.3053485
SRR17520069.3053487
SRR17520069.3053489
SRR17520069.3055559
SRR17520069.3055561
SRR17520069.3055563
SRR17520069.3055565
SRR17520069.3055567
SRR17520069.3055569
SRR17520069.3055571
SRR17520069.3055575
SRR17520069.3059867
SRR17520069.3068890
SRR17520069.3068892
SRR17520069.3068894
SRR17520069.3068898
SRR17520069.3068900
SRR17520069.3069497
SRR17520069.3069499
SRR17520069.3069501
SRR17520069.3069503
SRR17520069.3069505
SRR17520069.3070209
SRR17520069.3070211
SRR17520069.3070215
SRR17520069.3070217
SRR17520069.3070219
SRR17520069.3070221
SRR17520069.3070223
SRR17520069.3070225
SRR17520069.3070227
SRR17520069.3070229
SRR17520069.3070231
SRR17520069.3070233
SRR17520069.3070235
SRR17520069.3070237

SRR17520069.3070239
SRR17520069.3070241
SRR17520069.3070243
SRR17520069.3070245
SRR17520069.3070247
SRR17520069.3070249
SRR17520069.3070251
SRR17520069.3070253
SRR17520069.3070255
SRR17520069.3070257
SRR17520069.3080078
SRR17520069.3080080
SRR17520069.3080082
SRR17520069.3080084
SRR17520069.3080086
SRR17520069.3080088
SRR17520069.3080090
SRR17520069.3080092
SRR17520069.3080094
SRR17520069.3080096
SRR17520069.3080098
SRR17520069.3082618
SRR17520069.3082620
SRR17520069.3091601
SRR17520069.3091667
SRR17520069.3093609
SRR17520069.3093611
SRR17520069.3093613
SRR17520069.3093615
SRR17520069.3093617
SRR17520069.3093619
SRR17520069.3093621
SRR17520069.3093623
SRR17520069.3093625
SRR17520069.3093627
SRR17520069.3093629
SRR17520069.3093631
SRR17520069.3093633
SRR17520069.3093635
SRR17520069.3093637
SRR17520069.3097183
SRR17520069.3097185
SRR17520069.3097187
SRR17520069.3097189
SRR17520069.3097191
SRR17520069.3097193
SRR17520069.3097195

SRR17520069.3097197
SRR17520069.3097199
SRR17520069.3097201
SRR17520069.3097203
SRR17520069.3097205
SRR17520069.3097207
SRR17520069.3097209
SRR17520069.3097211
SRR17520069.3097213
SRR17520069.3097215
SRR17520069.3097217
SRR17520069.3097219
SRR17520069.3097221
SRR17520069.3097223
SRR17520069.3097225
SRR17520069.3097227
SRR17520069.3097229
SRR17520069.3097231
SRR17520069.3097233
SRR17520069.3097235
SRR17520069.3097237
SRR17520069.3097239
SRR17520069.3097241
SRR17520069.3097243
SRR17520069.3097245
SRR17520069.3097247
SRR17520069.3097249
SRR17520069.3097251
SRR17520069.3098369
SRR17520069.3098371
SRR17520069.3098373
SRR17520069.3098375
SRR17520069.3098377
SRR17520069.3098379
SRR17520069.3098381
SRR17520069.3098383
SRR17520069.3098385
SRR17520069.3098387
SRR17520069.3098389
SRR17520069.3098391
SRR17520069.3098393
SRR17520069.3098395
SRR17520069.3098397
SRR17520069.3098399
SRR17520069.3098401
SRR17520069.3098403
SRR17520069.3098405

SRR17520069.3098407
SRR17520069.3098409
SRR17520069.3098411
SRR17520069.3107887
SRR17520069.3107889
SRR17520069.3107891
SRR17520069.3107893
SRR17520069.3107895
SRR17520069.3112262
SRR17520069.3112264
SRR17520069.3112266
SRR17520069.3112268
SRR17520069.3112270
SRR17520069.3112272
SRR17520069.3112274
SRR17520069.3112276
SRR17520069.3112278
SRR17520069.3112280
SRR17520069.3112282
SRR17520069.3112284
SRR17520069.3112286
SRR17520069.3112288
SRR17520069.3112290
SRR17520069.3112292
SRR17520069.3112294
SRR17520069.3112296
SRR17520069.3112298
SRR17520069.3112300
SRR17520069.3112302
SRR17520069.3112304
SRR17520069.3112306
SRR17520069.3112308
SRR17520069.3112310
SRR17520069.3112312
SRR17520069.3112314
SRR17520069.3112316
SRR17520069.3112318
SRR17520069.3112320
SRR17520069.3112322
SRR17520069.3112324
SRR17520069.3112326
SRR17520069.3112328
SRR17520069.3112330
SRR17520069.3112334
SRR17520069.3112336
SRR17520069.3112340
SRR17520069.3112342

SRR17520069.3112344
SRR17520069.3112346
SRR17520069.3112348
SRR17520069.3112350
SRR17520069.3112352
SRR17520069.3112354
SRR17520069.3112356
SRR17520069.3112358
SRR17520069.3112360
SRR17520069.3112362
SRR17520069.3112364
SRR17520069.3112366
SRR17520069.3112368
SRR17520069.3112370
SRR17520069.3112372
SRR17520069.3112374
SRR17520069.3112376
SRR17520069.3112380
SRR17520069.3112382
SRR17520069.3112384
SRR17520069.3112388
SRR17520069.3112390
SRR17520069.3112392
SRR17520069.3112394
SRR17520069.3112396
SRR17520069.3112398
SRR17520069.3112400
SRR17520069.3112402
SRR17520069.3112404
SRR17520069.3112406
SRR17520069.3112412
SRR17520069.3112414
SRR17520069.3115051
SRR17520069.3115055
SRR17520069.3115057
SRR17520069.3115059
SRR17520069.3115061
SRR17520069.3115063
SRR17520069.3118046
SRR17520069.3120445
SRR17520069.3120447
SRR17520069.3120449
SRR17520069.3120451
SRR17520069.3120453
SRR17520069.3120455
SRR17520069.3120457
SRR17520069.3120459

SRR17520069.3120461
SRR17520069.3120463
SRR17520069.3120465
SRR17520069.3120467
SRR17520069.3120469
SRR17520069.3120471
SRR17520069.3132233
SRR17520069.3132235
SRR17520069.3132237
SRR17520069.3132239
SRR17520069.3132241
SRR17520069.3132243
SRR17520069.3132245
SRR17520069.3132247
SRR17520069.3132249
SRR17520069.3137601
SRR17520069.3138075
SRR17520069.3138077
SRR17520069.3138079
SRR17520069.3138081
SRR17520069.3138083
SRR17520069.3138085
SRR17520069.3138087
SRR17520069.3138089
SRR17520069.3138091
SRR17520069.3138093
SRR17520069.3138095
SRR17520069.3138097
SRR17520069.3138099
SRR17520069.3138101
SRR17520069.3138103
SRR17520069.3138105
SRR17520069.3138107
SRR17520069.3138109
SRR17520069.3138111
SRR17520069.3138113
SRR17520069.3138115
SRR17520069.3138117
SRR17520069.3138119
SRR17520069.3138121
SRR17520069.3138123
SRR17520069.3138125
SRR17520069.3138127
SRR17520069.3138129
SRR17520069.3138131
SRR17520069.3138133
SRR17520069.3138135

SRR17520069.3138137
SRR17520069.3140525
SRR17520069.3146053
SRR17520069.3146055
SRR17520069.3146065
SRR17520069.3146071
SRR17520069.3146079
SRR17520069.3146081
SRR17520069.3152091
SRR17520069.3152100
SRR17520069.3152110
SRR17520069.3156633
SRR17520069.3156635
SRR17520069.3156637
SRR17520069.3156639
SRR17520069.3156641
SRR17520069.3156643
SRR17520069.3156645
SRR17520069.3156647
SRR17520069.3156649
SRR17520069.3156651
SRR17520069.3156653
SRR17520069.3156655
SRR17520069.3156657
SRR17520069.3156659
SRR17520069.3158726
SRR17520069.3158728
SRR17520069.3158730
SRR17520069.3158732
SRR17520069.3158734
SRR17520069.3158736
SRR17520069.3158738
SRR17520069.3158740
SRR17520069.3158742
SRR17520069.3158744
SRR17520069.3158746
SRR17520069.3158748
SRR17520069.3158750
SRR17520069.3158752
SRR17520069.3159984
SRR17520069.3159986
SRR17520069.3159988
SRR17520069.3159990
SRR17520069.3159992
SRR17520069.3161358
SRR17520069.3161360
SRR17520069.3167282

SRR17520069.3167284
SRR17520069.3167286
SRR17520069.3167288
SRR17520069.3167290
SRR17520069.3167291
SRR17520069.3167292
SRR17520069.3167294
SRR17520069.3167296
SRR17520069.3167298
SRR17520069.3167300
SRR17520069.3167302
SRR17520069.3167304
SRR17520069.3168712
SRR17520069.3168714
SRR17520069.3168716
SRR17520069.3168718
SRR17520069.3168720
SRR17520069.3169885
SRR17520069.3169887
SRR17520069.3169889
SRR17520069.3169891
SRR17520069.3169893
SRR17520069.3169895
SRR17520069.3169897
SRR17520069.3169899
SRR17520069.3169901
SRR17520069.3172835
SRR17520069.3172837
SRR17520069.3172839
SRR17520069.3172841
SRR17520069.3172843
SRR17520069.3172845
SRR17520069.3172847
SRR17520069.3172849
SRR17520069.3172851
SRR17520069.3172853
SRR17520069.3172855
SRR17520069.3172857
SRR17520069.3172859
SRR17520069.3172861
SRR17520069.3172863
SRR17520069.3172865
SRR17520069.3172867
SRR17520069.3172869
SRR17520069.3172871
SRR17520069.3172873
SRR17520069.3172875

SRR17520069.3172877
SRR17520069.3172879
SRR17520069.3172881
SRR17520069.3172883
SRR17520069.3172885
SRR17520069.3172887
SRR17520069.3172889
SRR17520069.3172891
SRR17520069.3172893
SRR17520069.3172895
SRR17520069.3172897
SRR17520069.3172899
SRR17520069.3172901
SRR17520069.3172903
SRR17520069.3172905
SRR17520069.3172907
SRR17520069.3172909
SRR17520069.3172911
SRR17520069.3172913
SRR17520069.3172915
SRR17520069.3172917
SRR17520069.3172919
SRR17520069.3172921
SRR17520069.3172923
SRR17520069.3172925
SRR17520069.3172931
SRR17520069.3172933
SRR17520069.3172935
SRR17520069.3172937
SRR17520069.3172941
SRR17520069.3172943
SRR17520069.3172945
SRR17520069.3172947
SRR17520069.3172949
SRR17520069.3172951
SRR17520069.3172953
SRR17520069.3172955
SRR17520069.3172957
SRR17520069.3172959
SRR17520069.3172961
SRR17520069.3172963
SRR17520069.3172967
SRR17520069.3172969
SRR17520069.3172971
SRR17520069.3172973
SRR17520069.3172977
SRR17520069.3172979

SRR17520069.3172983
SRR17520069.3172985
SRR17520069.3172987
SRR17520069.3172989
SRR17520069.3172991
SRR17520069.3172993
SRR17520069.3172995
SRR17520069.3172997
SRR17520069.3172999
SRR17520069.3173001
SRR17520069.3173003
SRR17520069.3173005
SRR17520069.3173007
SRR17520069.3173009
SRR17520069.3173013
SRR17520069.3173015
SRR17520069.3173017
SRR17520069.3181766
SRR17520069.3181768
SRR17520069.3181770
SRR17520069.3181772
SRR17520069.3181774
SRR17520069.3181778
SRR17520069.3181780
SRR17520069.3181782
SRR17520069.3181784
SRR17520069.3181786
SRR17520069.3181788
SRR17520069.3181792
SRR17520069.3181794
SRR17520069.3183431
SRR17520069.3183433
SRR17520069.3183435
SRR17520069.3187500
SRR17520069.3187502
SRR17520069.3187504
SRR17520069.3187506
SRR17520069.3187508
SRR17520069.3187510
SRR17520069.3187512
SRR17520069.3187514
SRR17520069.3187516
SRR17520069.3187518
SRR17520069.3187520
SRR17520069.3187522
SRR17520069.3187524
SRR17520069.3187526

SRR17520069.3187528
SRR17520069.3187530
SRR17520069.3187532
SRR17520069.3187534
SRR17520069.3187536
SRR17520069.3187538
SRR17520069.3187540
SRR17520069.3187542
SRR17520069.3187544
SRR17520069.3187546
SRR17520069.3187548
SRR17520069.3187550
SRR17520069.3187552
SRR17520069.3187554
SRR17520069.3187556
SRR17520069.3187558
SRR17520069.3187560
SRR17520069.3187562
SRR17520069.3187564
SRR17520069.3187566
SRR17520069.3187568
SRR17520069.3187570
SRR17520069.3187572
SRR17520069.3187574
SRR17520069.3187576
SRR17520069.3187578
SRR17520069.3187580
SRR17520069.3187582
SRR17520069.3187584
SRR17520069.3187586
SRR17520069.3187588
SRR17520069.3187590
SRR17520069.3187592
SRR17520069.3187594
SRR17520069.3187598
SRR17520069.3187600
SRR17520069.3187602
SRR17520069.3187604
SRR17520069.3187606
SRR17520069.3187608
SRR17520069.3187610
SRR17520069.3187612
SRR17520069.3187614
SRR17520069.3187616
SRR17520069.3187618
SRR17520069.3187620
SRR17520069.3187622

SRR17520069.3187624
SRR17520069.3187626
SRR17520069.3187630
SRR17520069.3187632
SRR17520069.3195221
SRR17520069.3195223
SRR17520069.3195225
SRR17520069.3195227
SRR17520069.3195229
SRR17520069.3195231
SRR17520069.3195233
SRR17520069.3195235
SRR17520069.3195237
SRR17520069.3195239
SRR17520069.3195241
SRR17520069.3195243
SRR17520069.3195245
SRR17520069.3195423
SRR17520069.3195425
SRR17520069.3195427
SRR17520069.3195429
SRR17520069.3195431
SRR17520069.3195433
SRR17520069.3195435
SRR17520069.3195437
SRR17520069.3195439
SRR17520069.3195441
SRR17520069.3195443
SRR17520069.3195445
SRR17520069.3195451
SRR17520069.3195453
SRR17520069.3195455
SRR17520069.3195461
SRR17520069.3195463
SRR17520069.3195465
SRR17520069.3195467
SRR17520069.3195469
SRR17520069.3195471
SRR17520069.3195473
SRR17520069.3195477
SRR17520069.3195479
SRR17520069.3195481
SRR17520069.3195483
SRR17520069.3195485
SRR17520069.3195489
SRR17520069.3195491
SRR17520069.3195493

SRR17520069.3195495
SRR17520069.3195497
SRR17520069.3195499
SRR17520069.3199449
SRR17520069.3199451
SRR17520069.3199453
SRR17520069.3199455
SRR17520069.3199457
SRR17520069.3199459
SRR17520069.3199461
SRR17520069.3199463
SRR17520069.3199465
SRR17520069.3199467
SRR17520069.3199469
SRR17520069.3199471
SRR17520069.3199473
SRR17520069.3199475
SRR17520069.3199477
SRR17520069.3199479
SRR17520069.3199481
SRR17520069.3199483
SRR17520069.3199485
SRR17520069.3199487
SRR17520069.3199489
SRR17520069.3199491
SRR17520069.3199493
SRR17520069.3199495
SRR17520069.3199497
SRR17520069.3199499
SRR17520069.3199501
SRR17520069.3199503
SRR17520069.3199505
SRR17520069.3199507
SRR17520069.3199509
SRR17520069.3199511
SRR17520069.3199513
SRR17520069.3199515
SRR17520069.3199517
SRR17520069.3199519
SRR17520069.3199521
SRR17520069.3199523
SRR17520069.3199525
SRR17520069.3199527
SRR17520069.3199529
SRR17520069.3199531
SRR17520069.3199533
SRR17520069.3199535

SRR17520069.3199804
SRR17520069.3199806
SRR17520069.3199808
SRR17520069.3199810
SRR17520069.3199812
SRR17520069.3199814
SRR17520069.3199816
SRR17520069.3199818
SRR17520069.3199820
SRR17520069.3199822
SRR17520069.3199824
SRR17520069.3201858
SRR17520069.3202752
SRR17520069.3202754
SRR17520069.3202756
SRR17520069.3202758
SRR17520069.3202760
SRR17520069.3202762
SRR17520069.3202764
SRR17520069.3202766
SRR17520069.3202768
SRR17520069.3202770
SRR17520069.3202772
SRR17520069.3202774
SRR17520069.3202776
SRR17520069.3202778
SRR17520069.3202780
SRR17520069.3202782
SRR17520069.3202784
SRR17520069.3204252
SRR17520069.3208940
SRR17520069.3208942
SRR17520069.3208944
SRR17520069.3208946
SRR17520069.3209957
SRR17520069.3219788
SRR17520069.3219790
SRR17520069.3219792
SRR17520069.3219794
SRR17520069.3219796
SRR17520069.3219800
SRR17520069.3219802
SRR17520069.3219804
SRR17520069.3219806
SRR17520069.3219808
SRR17520069.3219810
SRR17520069.3219812

SRR17520069.3219814
SRR17520069.3219816
SRR17520069.3219818
SRR17520069.3220395
SRR17520069.3220397
SRR17520069.3220399
SRR17520069.3220403
SRR17520069.3220405
SRR17520069.3220407
SRR17520069.3220409
SRR17520069.3220411
SRR17520069.3220413
SRR17520069.3220415
SRR17520069.3220417
SRR17520069.3220419
SRR17520069.3220421
SRR17520069.3220423
SRR17520069.3220425
SRR17520069.3220427
SRR17520069.3220429
SRR17520069.3221668
SRR17520069.3221670
SRR17520069.3221672
SRR17520069.3221674
SRR17520069.3221676
SRR17520069.3221678
SRR17520069.3225526
SRR17520069.3225528
SRR17520069.3225530
SRR17520069.3225532
SRR17520069.3225536
SRR17520069.3225538
SRR17520069.3225540
SRR17520069.3225542
SRR17520069.3225544
SRR17520069.3225546
SRR17520069.3225548
SRR17520069.3225550
SRR17520069.3225552
SRR17520069.3225554
SRR17520069.3225556
SRR17520069.3225558
SRR17520069.3225560
SRR17520069.3225562
SRR17520069.3225566
SRR17520069.3225568
SRR17520069.3225570

SRR17520069.3225572
SRR17520069.3225576
SRR17520069.3225578
SRR17520069.3225580
SRR17520069.3225582
SRR17520069.3225584
SRR17520069.3225586
SRR17520069.3225588
SRR17520069.3225590
SRR17520069.3225592
SRR17520069.3225594
SRR17520069.3225596
SRR17520069.3227968
SRR17520069.3227970
SRR17520069.3227972
SRR17520069.3227974
SRR17520069.3227978
SRR17520069.3227980
SRR17520069.3227982
SRR17520069.3227984
SRR17520069.3227986
SRR17520069.3227988
SRR17520069.3227990
SRR17520069.3227992
SRR17520069.3227994
SRR17520069.3227996
SRR17520069.3227998
SRR17520069.3228000
SRR17520069.3228002
SRR17520069.3228004
SRR17520069.3228006
SRR17520069.3228008
SRR17520069.3228010
SRR17520069.3228012
SRR17520069.3228014
SRR17520069.3228016
SRR17520069.3228020
SRR17520069.3228022
SRR17520069.3228024
SRR17520069.3228026
SRR17520069.3228028
SRR17520069.3228030
SRR17520069.3228032
SRR17520069.3228034
SRR17520069.3228036
SRR17520069.3228038
SRR17520069.3228040

SRR17520069.3228042
SRR17520069.3228044
SRR17520069.3228046
SRR17520069.3228048
SRR17520069.3228050
SRR17520069.3228052
SRR17520069.3228054
SRR17520069.3228056
SRR17520069.3228058
SRR17520069.3228060
SRR17520069.3228062
SRR17520069.3231610
SRR17520069.3231614
SRR17520069.3231616
SRR17520069.3231618
SRR17520069.3231620
SRR17520069.3235318
SRR17520069.3235321
SRR17520069.3237061
SRR17520069.3237063
SRR17520069.3237065
SRR17520069.3237067
SRR17520069.3237069
SRR17520069.3237071
SRR17520069.3237073
SRR17520069.3237075
SRR17520069.3237077
SRR17520069.3237079
SRR17520069.3237081
SRR17520069.3237083
SRR17520069.3237085
SRR17520069.3237087
SRR17520069.3237089
SRR17520069.3237091
SRR17520069.3237095
SRR17520069.3237097
SRR17520069.3237099
SRR17520069.3239616
SRR17520069.3239618
SRR17520069.3239620
SRR17520069.3239622
SRR17520069.3239624
SRR17520069.3239626
SRR17520069.3239628
SRR17520069.3239632
SRR17520069.3239634
SRR17520069.3239636

SRR17520069.3239638
SRR17520069.3239640
SRR17520069.3239642
SRR17520069.3239644
SRR17520069.3239646
SRR17520069.3239648
SRR17520069.3239650
SRR17520069.3239652
SRR17520069.3239654
SRR17520069.3239656
SRR17520069.3239658
SRR17520069.3239660
SRR17520069.3239662
SRR17520069.3239664
SRR17520069.3239666
SRR17520069.3239668
SRR17520069.3239670
SRR17520069.3239672
SRR17520069.3239674
SRR17520069.3239676
SRR17520069.3239678
SRR17520069.3239680
SRR17520069.3239682
SRR17520069.3239684
SRR17520069.3239686
SRR17520069.3239688
SRR17520069.3243604
SRR17520069.3243606
SRR17520069.3243608
SRR17520069.3245857
SRR17520069.3245859
SRR17520069.3245861
SRR17520069.3245865
SRR17520069.3245867
SRR17520069.3245869
SRR17520069.3245871
SRR17520069.3245883
SRR17520069.3245885
SRR17520069.3245887
SRR17520069.3245889
SRR17520069.3245891
SRR17520069.3245893
SRR17520069.3245895
SRR17520069.3245897
SRR17520069.3245899
SRR17520069.3245901
SRR17520069.3245903

SRR17520069.3245905
SRR17520069.3245907
SRR17520069.3245909
SRR17520069.3245911
SRR17520069.3245913
SRR17520069.3245915
SRR17520069.3245917
SRR17520069.3245919
SRR17520069.3245921
SRR17520069.3245923
SRR17520069.3245925
SRR17520069.3245927
SRR17520069.3245929
SRR17520069.3245931
SRR17520069.3245933
SRR17520069.3245935
SRR17520069.3245937
SRR17520069.3245939
SRR17520069.3245941
SRR17520069.3245943
SRR17520069.3245945
SRR17520069.3245947
SRR17520069.3245949
SRR17520069.3245951
SRR17520069.3245953
SRR17520069.3245955
SRR17520069.3245957
SRR17520069.3245959
SRR17520069.3245961
SRR17520069.3245963
SRR17520069.3245965
SRR17520069.3245967
SRR17520069.3245969
SRR17520069.3245971
SRR17520069.3245973
SRR17520069.3245975
SRR17520069.3245977
SRR17520069.3245979
SRR17520069.3245981
SRR17520069.3245983
SRR17520069.3245989
SRR17520069.3245991
SRR17520069.3245993
SRR17520069.3245995
SRR17520069.3245997
SRR17520069.3245999
SRR17520069.3246001

SRR17520069.3246003
SRR17520069.3246005
SRR17520069.3246007
SRR17520069.3246009
SRR17520069.3246011
SRR17520069.3246013
SRR17520069.3246015
SRR17520069.3246017
SRR17520069.3246019
SRR17520069.3246021
SRR17520069.3246023
SRR17520069.3246025
SRR17520069.3246027
SRR17520069.3246029
SRR17520069.3246031
SRR17520069.3246033
SRR17520069.3246035
SRR17520069.3246037
SRR17520069.3246039
SRR17520069.3246041
SRR17520069.3246043
SRR17520069.3246045
SRR17520069.3246047
SRR17520069.3246049
SRR17520069.3246051
SRR17520069.3246055
SRR17520069.3246057
SRR17520069.3246059
SRR17520069.3246061
SRR17520069.3246063
SRR17520069.3246065
SRR17520069.3246067
SRR17520069.3246069
SRR17520069.3246071
SRR17520069.3246073
SRR17520069.3246075
SRR17520069.3246077
SRR17520069.3246081
SRR17520069.3249812
SRR17520069.3249814
SRR17520069.3252780
SRR17520069.3252782
SRR17520069.3252784
SRR17520069.3252786
SRR17520069.3252792
SRR17520069.3252794
SRR17520069.3252796

SRR17520069.3252798
SRR17520069.3252800
SRR17520069.3252802
SRR17520069.3254976
SRR17520069.3254978
SRR17520069.3254980
SRR17520069.3254982
SRR17520069.3254984
SRR17520069.3254986
SRR17520069.3254988
SRR17520069.3254990
SRR17520069.3254994
SRR17520069.3254998
SRR17520069.3255000
SRR17520069.3255002
SRR17520069.3255006
SRR17520069.3255008
SRR17520069.3255010
SRR17520069.3255012
SRR17520069.3255014
SRR17520069.3255016
SRR17520069.3258007
SRR17520069.3258009
SRR17520069.3258011
SRR17520069.3258013
SRR17520069.3258015
SRR17520069.3258017
SRR17520069.3258019
SRR17520069.3258021
SRR17520069.3258023
SRR17520069.3258025
SRR17520069.3258027
SRR17520069.3258029
SRR17520069.3258031
SRR17520069.3258033
SRR17520069.3258035
SRR17520069.3258037
SRR17520069.3258039
SRR17520069.3258041
SRR17520069.3258043
SRR17520069.3258045
SRR17520069.3258047
SRR17520069.3258049
SRR17520069.3258051
SRR17520069.3258053
SRR17520069.3258055
SRR17520069.3258057

SRR17520069.3258059
SRR17520069.3258061
SRR17520069.3258063
SRR17520069.3258065
SRR17520069.3258071
SRR17520069.3258073
SRR17520069.3258077
SRR17520069.3258079
SRR17520069.3258081
SRR17520069.3258083
SRR17520069.3258085
SRR17520069.3258087
SRR17520069.3258089
SRR17520069.3258091
SRR17520069.3258093
SRR17520069.3269516
SRR17520069.3271388
SRR17520069.3271390
SRR17520069.3271392
SRR17520069.3271394
SRR17520069.3271396
SRR17520069.3271398
SRR17520069.3271400
SRR17520069.3271402
SRR17520069.3271404
SRR17520069.3273020
SRR17520069.3273886
SRR17520069.3273888
SRR17520069.3273890
SRR17520069.3273892
SRR17520069.3273894
SRR17520069.3273896
SRR17520069.3273898
SRR17520069.3273900
SRR17520069.3273902
SRR17520069.3274792
SRR17520069.3274794
SRR17520069.3274796
SRR17520069.3274798
SRR17520069.3274800
SRR17520069.3274802
SRR17520069.3274804
SRR17520069.3274806
SRR17520069.3274808
SRR17520069.3274810
SRR17520069.3274812
SRR17520069.3274814

SRR17520069.3274816
SRR17520069.3274818
SRR17520069.3274820
SRR17520069.3274822
SRR17520069.3274826
SRR17520069.3274830
SRR17520069.3274832
SRR17520069.3274834
SRR17520069.3274836
SRR17520069.3274838
SRR17520069.3274840
SRR17520069.3274842
SRR17520069.3274844
SRR17520069.3274846
SRR17520069.3274848
SRR17520069.3274850
SRR17520069.3274852
SRR17520069.3274854
SRR17520069.3274856
SRR17520069.3274858
SRR17520069.3274860
SRR17520069.3274862
SRR17520069.3274864
SRR17520069.3274868
SRR17520069.3274870
SRR17520069.3274872
SRR17520069.3274874
SRR17520069.3275604
SRR17520069.3275606
SRR17520069.3275608
SRR17520069.3279221
SRR17520069.3279223
SRR17520069.3279225
SRR17520069.3279227
SRR17520069.3279229
SRR17520069.3279233
SRR17520069.3279235
SRR17520069.3279237
SRR17520069.3279243
SRR17520069.3279245
SRR17520069.3279247
SRR17520069.3279249
SRR17520069.3279251
SRR17520069.3279253
SRR17520069.3279255
SRR17520069.3279257
SRR17520069.3282382

SRR17520069.3282384
SRR17520069.3282386
SRR17520069.3282388
SRR17520069.3282390
SRR17520069.3282392
SRR17520069.3282394
SRR17520069.3282396
SRR17520069.3282398
SRR17520069.3282400
SRR17520069.3282402
SRR17520069.3282404
SRR17520069.3282406
SRR17520069.3282408
SRR17520069.3282410
SRR17520069.3282412
SRR17520069.3282414
SRR17520069.3282416
SRR17520069.3282418
SRR17520069.3282420
SRR17520069.3286612
SRR17520069.3286614
SRR17520069.3286616
SRR17520069.3286618
SRR17520069.3286620
SRR17520069.3286622
SRR17520069.3286624
SRR17520069.3286626
SRR17520069.3286628
SRR17520069.3286630
SRR17520069.3286632
SRR17520069.3286634
SRR17520069.3286636
SRR17520069.3286638
SRR17520069.3286640
SRR17520069.3286642
SRR17520069.3286644
SRR17520069.3286646
SRR17520069.3286648
SRR17520069.3286650
SRR17520069.3286652
SRR17520069.3286654
SRR17520069.3286656
SRR17520069.3286658
SRR17520069.3286660
SRR17520069.3286662
SRR17520069.3286664
SRR17520069.3286666

SRR17520069.3286668
SRR17520069.3286670
SRR17520069.3286672
SRR17520069.3286674
SRR17520069.3292190
SRR17520069.3292192
SRR17520069.3292194
SRR17520069.3292196
SRR17520069.3292198
SRR17520069.3292200
SRR17520069.3292202
SRR17520069.3292204
SRR17520069.3292206
SRR17520069.3292208
SRR17520069.3292210
SRR17520069.3292212
SRR17520069.3292214
SRR17520069.3292216
SRR17520069.3292218
SRR17520069.3292221
SRR17520069.3292223
SRR17520069.3292225
SRR17520069.3292227
SRR17520069.3292229
SRR17520069.3310804
SRR17520069.3310806
SRR17520069.3310808
SRR17520069.3310810
SRR17520069.3310812
SRR17520069.3310814
SRR17520069.3314012
SRR17520069.3314014
SRR17520069.3314016
SRR17520069.3314018
SRR17520069.3314020
SRR17520069.3318920
SRR17520069.3318922
SRR17520069.3318924
SRR17520069.3318926
SRR17520069.3319923
SRR17520069.3319925
SRR17520069.3324969
SRR17520069.3324971
SRR17520069.3324973
SRR17520069.3324975
SRR17520069.3324977
SRR17520069.3324979

SRR17520069.3324983
SRR17520069.3324985
SRR17520069.3324987
SRR17520069.3324989
SRR17520069.3324991
SRR17520069.3324993
SRR17520069.3324995
SRR17520069.3324997
SRR17520069.3324999
SRR17520069.3325001
SRR17520069.3325003
SRR17520069.3325005
SRR17520069.3325007
SRR17520069.3325009
SRR17520069.3325011
SRR17520069.3325013
SRR17520069.3325015
SRR17520069.3325017
SRR17520069.3325019
SRR17520069.3325021
SRR17520069.3325023
SRR17520069.3325025
SRR17520069.3325027
SRR17520069.3325029
SRR17520069.3325031
SRR17520069.3325033
SRR17520069.3325035
SRR17520069.3325037
SRR17520069.3325039
SRR17520069.3325041
SRR17520069.3325043
SRR17520069.3325045
SRR17520069.3325047
SRR17520069.3325049
SRR17520069.3325051
SRR17520069.3325053
SRR17520069.3325055
SRR17520069.3325057
SRR17520069.3325059
SRR17520069.3325061
SRR17520069.3325063
SRR17520069.3325065
SRR17520069.3325067
SRR17520069.3325069
SRR17520069.3325071
SRR17520069.3325073
SRR17520069.3325075

SRR17520069.3325077
SRR17520069.3325079
SRR17520069.3325081
SRR17520069.3325083
SRR17520069.3325085
SRR17520069.3327933
SRR17520069.3327935
SRR17520069.3327937
SRR17520069.3327939
SRR17520069.3327941
SRR17520069.3327943
SRR17520069.3327945
SRR17520069.3327949
SRR17520069.3327953
SRR17520069.3327955
SRR17520069.3327957
SRR17520069.3327959
SRR17520069.3327961
SRR17520069.3327965
SRR17520069.3327967
SRR17520069.3327975
SRR17520069.3327977
SRR17520069.3327979
SRR17520069.3327981
SRR17520069.3327983
SRR17520069.3327985
SRR17520069.3327987
SRR17520069.3327989
SRR17520069.3327993
SRR17520069.3327999
SRR17520069.3328746
SRR17520069.3328748
SRR17520069.3328750
SRR17520069.3328752
SRR17520069.3328754
SRR17520069.3328756
SRR17520069.3328758
SRR17520069.3328760
SRR17520069.3328764
SRR17520069.3328766
SRR17520069.3328768
SRR17520069.3328770
SRR17520069.3328772
SRR17520069.3328774
SRR17520069.3328776
SRR17520069.3328778
SRR17520069.3328780

SRR17520069.3328782
SRR17520069.3328784
SRR17520069.3328786
SRR17520069.3328788
SRR17520069.3328790
SRR17520069.3328792
SRR17520069.3328794
SRR17520069.3328796
SRR17520069.3332712
SRR17520069.3332714
SRR17520069.3332716
SRR17520069.3332718
SRR17520069.3332720
SRR17520069.3332722
SRR17520069.3332724
SRR17520069.3332733
SRR17520069.3335354
SRR17520069.3335356
SRR17520069.3335358
SRR17520069.3335360
SRR17520069.3335362
SRR17520069.3335366
SRR17520069.3335368
SRR17520069.3335370
SRR17520069.3335372
SRR17520069.3335374
SRR17520069.3335376
SRR17520069.3335378
SRR17520069.3335382
SRR17520069.3335384
SRR17520069.3335386
SRR17520069.3335388
SRR17520069.3335390
SRR17520069.3335392
SRR17520069.3335394
SRR17520069.3335396
SRR17520069.3335398
SRR17520069.3335400
SRR17520069.3335402
SRR17520069.3335404
SRR17520069.3335406
SRR17520069.3335408
SRR17520069.3335410
SRR17520069.3335412
SRR17520069.3335414
SRR17520069.3335416
SRR17520069.3335418

SRR17520069.3335420
SRR17520069.3337657
SRR17520069.3338060
SRR17520069.3338062
SRR17520069.3338066
SRR17520069.3338068
SRR17520069.3338070
SRR17520069.3338072
SRR17520069.3338074
SRR17520069.3338076
SRR17520069.3338078
SRR17520069.3338080
SRR17520069.3338082
SRR17520069.3338084
SRR17520069.3338086
SRR17520069.3338088
SRR17520069.3338090
SRR17520069.3338092
SRR17520069.3338094
SRR17520069.3338096
SRR17520069.3338098
SRR17520069.3338100
SRR17520069.3339542
SRR17520069.3339544
SRR17520069.3339550
SRR17520069.3339552
SRR17520069.3339554
SRR17520069.3339556
SRR17520069.3339558
SRR17520069.3339560
SRR17520069.3339562
SRR17520069.3339564
SRR17520069.3339566
SRR17520069.3339568
SRR17520069.3339570
SRR17520069.3339572
SRR17520069.3339574
SRR17520069.3339576
SRR17520069.3339578
SRR17520069.3339580
SRR17520069.3339582
SRR17520069.3339584
SRR17520069.3339586
SRR17520069.3339588
SRR17520069.3339590
SRR17520069.3339592
SRR17520069.3339594

SRR17520069.3339596
SRR17520069.3339598
SRR17520069.3339600
SRR17520069.3339602
SRR17520069.3339604
SRR17520069.3339608
SRR17520069.3339610
SRR17520069.3339612
SRR17520069.3339614
SRR17520069.3339616
SRR17520069.3339618
SRR17520069.3339620
SRR17520069.3339622
SRR17520069.3339624
SRR17520069.3339626
SRR17520069.3339628
SRR17520069.3339630
SRR17520069.3339632
SRR17520069.3339634
SRR17520069.3339636
SRR17520069.3339640
SRR17520069.3339642
SRR17520069.3339644
SRR17520069.3339646
SRR17520069.3339648
SRR17520069.3339650
SRR17520069.3339652
SRR17520069.3339658
SRR17520069.3339660
SRR17520069.3339662
SRR17520069.3339664
SRR17520069.3339666
SRR17520069.3339668
SRR17520069.3339670
SRR17520069.3339672
SRR17520069.3339674
SRR17520069.3339676
SRR17520069.3339680
SRR17520069.3339682
SRR17520069.3339684
SRR17520069.3341527
SRR17520069.3341529
SRR17520069.3341531
SRR17520069.3341533
SRR17520069.3341535
SRR17520069.3341537
SRR17520069.3341539

SRR17520069.3341541
SRR17520069.3341543
SRR17520069.3341545
SRR17520069.3341547
SRR17520069.3341549
SRR17520069.3341551
SRR17520069.3341553
SRR17520069.3341555
SRR17520069.3341557
SRR17520069.3341559
SRR17520069.3341561
SRR17520069.3342621
SRR17520069.3342661
SRR17520069.3345267
SRR17520069.3345269
SRR17520069.3345271
SRR17520069.3345275
SRR17520069.3345277
SRR17520069.3345279
SRR17520069.3345281
SRR17520069.3345283
SRR17520069.3345285
SRR17520069.3345287
SRR17520069.3345289
SRR17520069.3345291
SRR17520069.3345293
SRR17520069.3345295
SRR17520069.3345297
SRR17520069.3345301
SRR17520069.3345303
SRR17520069.3345305
SRR17520069.3345307
SRR17520069.3345309
SRR17520069.3345311
SRR17520069.3345313
SRR17520069.3345317
SRR17520069.3345319
SRR17520069.3345321
SRR17520069.3345323
SRR17520069.3345325
SRR17520069.3345327
SRR17520069.3345329
SRR17520069.3345331
SRR17520069.3345333
SRR17520069.3345335
SRR17520069.3345337
SRR17520069.3345339

SRR17520069.3345341
SRR17520069.3345343
SRR17520069.3345345
SRR17520069.3345347
SRR17520069.3345349
SRR17520069.3345351
SRR17520069.3345353
SRR17520069.3345355
SRR17520069.3345357
SRR17520069.3345359
SRR17520069.3345363
SRR17520069.3345365
SRR17520069.3345367
SRR17520069.3345371
SRR17520069.3345373
SRR17520069.3345375
SRR17520069.3345377
SRR17520069.3345379
SRR17520069.3346075
SRR17520069.3346077
SRR17520069.3346079
SRR17520069.3346083
SRR17520069.3346085
SRR17520069.3346349
SRR17520069.3346351
SRR17520069.3346353
SRR17520069.3346357
SRR17520069.3346359
SRR17520069.3346361
SRR17520069.3346363
SRR17520069.3346365
SRR17520069.3346367
SRR17520069.3346371
SRR17520069.3346373
SRR17520069.3346375
SRR17520069.3352761
SRR17520069.3352763
SRR17520069.3352765
SRR17520069.3352767
SRR17520069.3352769
SRR17520069.3352771
SRR17520069.3352773
SRR17520069.3352775
SRR17520069.3352777
SRR17520069.3352779
SRR17520069.3352781
SRR17520069.3352783

SRR17520069.3352785
SRR17520069.3352787
SRR17520069.3352789
SRR17520069.3352791
SRR17520069.3352793
SRR17520069.3352795
SRR17520069.3352797
SRR17520069.3352799
SRR17520069.3352803
SRR17520069.3352805
SRR17520069.3352807
SRR17520069.3352809
SRR17520069.3352811
SRR17520069.3352813
SRR17520069.3352815
SRR17520069.3352817
SRR17520069.3352819
SRR17520069.3352821
SRR17520069.3352823
SRR17520069.3352825
SRR17520069.3352829
SRR17520069.3352831
SRR17520069.3352833
SRR17520069.3352835
SRR17520069.3352837
SRR17520069.3352839
SRR17520069.3352843
SRR17520069.3352845
SRR17520069.3352847
SRR17520069.3352849
SRR17520069.3352851
SRR17520069.3352853
SRR17520069.3352855
SRR17520069.3352857
SRR17520069.3352859
SRR17520069.3352861
SRR17520069.3352863
SRR17520069.3352865
SRR17520069.3352867
SRR17520069.3352870
SRR17520069.3352872
SRR17520069.3352874
SRR17520069.3362103
SRR17520069.3362105
SRR17520069.3362107
SRR17520069.3362109
SRR17520069.3362113

SRR17520069.3362115
SRR17520069.3366363
SRR17520069.3366365
SRR17520069.3366367
SRR17520069.3366369
SRR17520069.3367841
SRR17520069.3367843
SRR17520069.3375930
SRR17520069.3375936
SRR17520069.3375950
SRR17520069.3375976
SRR17520069.3376012
SRR17520069.3376020
SRR17520069.3376058
SRR17520069.3376120
SRR17520069.3376124
SRR17520069.3376136
SRR17520069.3376146
SRR17520069.3381329
SRR17520069.3381331
SRR17520069.3381333
SRR17520069.3386649
SRR17520069.3386651
SRR17520069.3386653
SRR17520069.3386655
SRR17520069.3386657
SRR17520069.3386659
SRR17520069.3386661
SRR17520069.3386665
SRR17520069.3386667
SRR17520069.3386669
SRR17520069.3391586
SRR17520069.3391588
SRR17520069.3391590
SRR17520069.3391592
SRR17520069.3391594
SRR17520069.3391596
SRR17520069.3391598
SRR17520069.3391600
SRR17520069.3391602
SRR17520069.3391604
SRR17520069.3391606
SRR17520069.3391608
SRR17520069.3391610
SRR17520069.3391612
SRR17520069.3391614
SRR17520069.3399142

SRR17520069.3399144
SRR17520069.3399146
SRR17520069.3399148
SRR17520069.3399150
SRR17520069.3399152
SRR17520069.3399154
SRR17520069.3399158
SRR17520069.3399587
SRR17520069.3399591
SRR17520069.3399593
SRR17520069.3399595
SRR17520069.3399597
SRR17520069.3399599
SRR17520069.3399601
SRR17520069.3399603
SRR17520069.3399605
SRR17520069.3399607
SRR17520069.3399609
SRR17520069.3399611
SRR17520069.3399613
SRR17520069.3399615
SRR17520069.3399617
SRR17520069.3399619
SRR17520069.3399621
SRR17520069.3399623
SRR17520069.3399625
SRR17520069.3399627
SRR17520069.3399629
SRR17520069.3407532
SRR17520069.3407552
SRR17520069.3407562
SRR17520069.3409435
SRR17520069.3409966
SRR17520069.3409968
SRR17520069.3409970
SRR17520069.3409972
SRR17520069.3409974
SRR17520069.3409976
SRR17520069.3409978
SRR17520069.3409980
SRR17520069.3409982
SRR17520069.3409984
SRR17520069.3409986
SRR17520069.3409988
SRR17520069.3409990
SRR17520069.3409992
SRR17520069.3409994

SRR17520069.3409996
SRR17520069.3409998
SRR17520069.3410000
SRR17520069.3410002
SRR17520069.3410004
SRR17520069.3410006
SRR17520069.3410008
SRR17520069.3410010
SRR17520069.3410012
SRR17520069.3410014
SRR17520069.3410016
SRR17520069.3410018
SRR17520069.3413802
SRR17520069.3413804
SRR17520069.3413806
SRR17520069.3413808
SRR17520069.3413810
SRR17520069.3413812
SRR17520069.3413814
SRR17520069.3413816
SRR17520069.3413818
SRR17520069.3413820
SRR17520069.3413822
SRR17520069.3413824
SRR17520069.3413826
SRR17520069.3413828
SRR17520069.3413830
SRR17520069.3413832
SRR17520069.3413834
SRR17520069.3413836
SRR17520069.3413838
SRR17520069.3413840
SRR17520069.3413842
SRR17520069.3413846
SRR17520069.3421003
SRR17520069.3421005
SRR17520069.3421007
SRR17520069.3421009
SRR17520069.3421011
SRR17520069.3421013
SRR17520069.3421015
SRR17520069.3421017
SRR17520069.3428106
SRR17520069.3428108
SRR17520069.3428110
SRR17520069.3430863
SRR17520069.3430865

SRR17520069.3430869
SRR17520069.3430871
SRR17520069.3430875
SRR17520069.3430877
SRR17520069.3430879
SRR17520069.3430883
SRR17520069.3430885
SRR17520069.3430887
SRR17520069.3430889
SRR17520069.3430891
SRR17520069.3430893
SRR17520069.3430895
SRR17520069.3430897
SRR17520069.3430901
SRR17520069.3434681
SRR17520069.3434683
SRR17520069.3434685
SRR17520069.3436395
SRR17520069.3437702
SRR17520069.3443265
SRR17520069.3446123
SRR17520069.3446125
SRR17520069.3446127
SRR17520069.3446129
SRR17520069.3446131
SRR17520069.3450611
SRR17520069.3450613
SRR17520069.3462332
SRR17520069.3462334
SRR17520069.3462336
SRR17520069.3462338
SRR17520069.3462340
SRR17520069.3462342
SRR17520069.3462344
SRR17520069.3462346
SRR17520069.3462348
SRR17520069.3462350
SRR17520069.3462352
SRR17520069.3462354
SRR17520069.3462356
SRR17520069.3462358
SRR17520069.3462360
SRR17520069.3462362
SRR17520069.3462364
SRR17520069.3462366
SRR17520069.3462368
SRR17520069.3462370

SRR17520069.3462372
SRR17520069.3462374
SRR17520069.3462376
SRR17520069.3462378
SRR17520069.3462380
SRR17520069.3463652
SRR17520069.3463654
SRR17520069.3463656
SRR17520069.3463658
SRR17520069.3467115
SRR17520069.3467117
SRR17520069.3467119
SRR17520069.3467121
SRR17520069.3467123
SRR17520069.3467125
SRR17520069.3467127
SRR17520069.3467129
SRR17520069.3467131
SRR17520069.3467133
SRR17520069.3467135
SRR17520069.3467137
SRR17520069.3467139
SRR17520069.3467141
SRR17520069.3467143
SRR17520069.3467145
SRR17520069.3467147
SRR17520069.3467149
SRR17520069.3467151
SRR17520069.3467153
SRR17520069.3467155
SRR17520069.3467157
SRR17520069.3467159
SRR17520069.3467163
SRR17520069.3467165
SRR17520069.3467167
SRR17520069.3467169
SRR17520069.3467171
SRR17520069.3467173
SRR17520069.3467175
SRR17520069.3467177
SRR17520069.3467181
SRR17520069.3467183
SRR17520069.3467185
SRR17520069.3467187
SRR17520069.3467191
SRR17520069.3467193
SRR17520069.3467195

SRR17520069.3467197
SRR17520069.3469647
SRR17520069.3469649
SRR17520069.3469651
SRR17520069.3469653
SRR17520069.3469655
SRR17520069.3469657
SRR17520069.3474124
SRR17520069.3474126
SRR17520069.3474128
SRR17520069.3474130
SRR17520069.3474132
SRR17520069.3474134
SRR17520069.3474136
SRR17520069.3474138
SRR17520069.3474140
SRR17520069.3474142
SRR17520069.3474144
SRR17520069.3474146
SRR17520069.3474148
SRR17520069.3474150
SRR17520069.3474152
SRR17520069.3474154
SRR17520069.3474156
SRR17520069.3474158
SRR17520069.3474162
SRR17520069.3474164
SRR17520069.3474166
SRR17520069.3474168
SRR17520069.3474170
SRR17520069.3474172
SRR17520069.3474174
SRR17520069.3474176
SRR17520069.3474178
SRR17520069.3474180
SRR17520069.3474182
SRR17520069.3474184
SRR17520069.3474186
SRR17520069.3474188
SRR17520069.3474190
SRR17520069.3476405
SRR17520069.3476407
SRR17520069.3476409
SRR17520069.3476411
SRR17520069.3476413
SRR17520069.3476415
SRR17520069.3476417

SRR17520069.3476419
SRR17520069.3476421
SRR17520069.3476423
SRR17520069.3476425
SRR17520069.3476427
SRR17520069.3476431
SRR17520069.3476433
SRR17520069.3476435
SRR17520069.3476437
SRR17520069.3476439
SRR17520069.3476441
SRR17520069.3476443
SRR17520069.3476445
SRR17520069.3476447
SRR17520069.3476449
SRR17520069.3476451
SRR17520069.3476453
SRR17520069.3476455
SRR17520069.3476457
SRR17520069.3478075
SRR17520069.3478077
SRR17520069.3478079
SRR17520069.3478081
SRR17520069.3478083
SRR17520069.3478085
SRR17520069.3478087
SRR17520069.3478089
SRR17520069.3478091
SRR17520069.3478093
SRR17520069.3478095
SRR17520069.3478097
SRR17520069.3478099
SRR17520069.3478101
SRR17520069.3478103
SRR17520069.3478105
SRR17520069.3478107
SRR17520069.3478109
SRR17520069.3478111
SRR17520069.3478113
SRR17520069.3478115
SRR17520069.3478117
SRR17520069.3478119
SRR17520069.3480289
SRR17520069.3480291
SRR17520069.3480293
SRR17520069.3480295
SRR17520069.3480297

SRR17520069.3480299
SRR17520069.3480301
SRR17520069.3480303
SRR17520069.3480305
SRR17520069.3480307
SRR17520069.3480309
SRR17520069.3485881
SRR17520069.3485883
SRR17520069.3485885
SRR17520069.3485887
SRR17520069.3485889
SRR17520069.3485891
SRR17520069.3485893
SRR17520069.3485895
SRR17520069.3485897
SRR17520069.3485899
SRR17520069.3485901
SRR17520069.3485903
SRR17520069.3487234
SRR17520069.3487236
SRR17520069.3487238
SRR17520069.3487240
SRR17520069.3487242
SRR17520069.3487244
SRR17520069.3487246
SRR17520069.3487248
SRR17520069.3487250
SRR17520069.3487252
SRR17520069.3487254
SRR17520069.3487256
SRR17520069.3487258
SRR17520069.3487260
SRR17520069.3487262
SRR17520069.3487264
SRR17520069.3487266
SRR17520069.3488491
SRR17520069.3489907
SRR17520069.3489909
SRR17520069.3489911
SRR17520069.3489913
SRR17520069.3489915
SRR17520069.3489917
SRR17520069.3489919
SRR17520069.3489921
SRR17520069.3489923
SRR17520069.3489925
SRR17520069.3489927

SRR17520069.3491548
SRR17520069.3491550
SRR17520069.3491552
SRR17520069.3491556
SRR17520069.3491558
SRR17520069.3491560
SRR17520069.3491562
SRR17520069.3491564
SRR17520069.3491566
SRR17520069.3491568
SRR17520069.3491570
SRR17520069.3491572
SRR17520069.3491574
SRR17520069.3491576
SRR17520069.3491578
SRR17520069.3491580
SRR17520069.3491582
SRR17520069.3491584
SRR17520069.3491586
SRR17520069.3491588
SRR17520069.3491590
SRR17520069.3491592
SRR17520069.3491594
SRR17520069.3491596
SRR17520069.3491598
SRR17520069.3491600
SRR17520069.3491602
SRR17520069.3491604
SRR17520069.3491606
SRR17520069.3491608
SRR17520069.3491610
SRR17520069.3491612
SRR17520069.3493531
SRR17520069.3493533
SRR17520069.3493535
SRR17520069.3501263
SRR17520069.3501265
SRR17520069.3501267
SRR17520069.3501269
SRR17520069.3501271
SRR17520069.3501273
SRR17520069.3501686
SRR17520069.3501688
SRR17520069.3501690
SRR17520069.3501692
SRR17520069.3501694
SRR17520069.3501696

SRR17520069.3501698
SRR17520069.3501700
SRR17520069.3501702
SRR17520069.3501704
SRR17520069.3501706
SRR17520069.3501708
SRR17520069.3501710
SRR17520069.3504714
SRR17520069.3508286
SRR17520069.3508288
SRR17520069.3508290
SRR17520069.3508292
SRR17520069.3508294
SRR17520069.3510559
SRR17520069.3510561
SRR17520069.3510563
SRR17520069.3510565
SRR17520069.3510567
SRR17520069.3510569
SRR17520069.3510575
SRR17520069.3510579
SRR17520069.3511631
SRR17520069.3511633
SRR17520069.3511635
SRR17520069.3511637
SRR17520069.3511639
SRR17520069.3511641
SRR17520069.3511643
SRR17520069.3511647
SRR17520069.3511649
SRR17520069.3511651
SRR17520069.3511653
SRR17520069.3511655
SRR17520069.3511659
SRR17520069.3511661
SRR17520069.3511663
SRR17520069.3511665
SRR17520069.3511667
SRR17520069.3511669
SRR17520069.3511671
SRR17520069.3511673
SRR17520069.3511675
SRR17520069.3511677
SRR17520069.3511679
SRR17520069.3511683
SRR17520069.3511687
SRR17520069.3511691

SRR17520069.3511693
SRR17520069.3511695
SRR17520069.3511697
SRR17520069.3512252
SRR17520069.3513986
SRR17520069.3513990
SRR17520069.3513992
SRR17520069.3513994
SRR17520069.3513996
SRR17520069.3513998
SRR17520069.3514000
SRR17520069.3514002
SRR17520069.3514004
SRR17520069.3514006
SRR17520069.3514010
SRR17520069.3514012
SRR17520069.3514014
SRR17520069.3514016
SRR17520069.3514018
SRR17520069.3514020
SRR17520069.3514022
SRR17520069.3514024
SRR17520069.3514026
SRR17520069.3514028
SRR17520069.3514030
SRR17520069.3514032
SRR17520069.3514034
SRR17520069.3514036
SRR17520069.3514038
SRR17520069.3514040
SRR17520069.3514042
SRR17520069.3514046
SRR17520069.3514050
SRR17520069.3514052
SRR17520069.3514054
SRR17520069.3514056
SRR17520069.3514058
SRR17520069.3514062
SRR17520069.3514064
SRR17520069.3514066
SRR17520069.3514068
SRR17520069.3514070
SRR17520069.3514072
SRR17520069.3514074
SRR17520069.3514076
SRR17520069.3514078
SRR17520069.3521620

SRR17520069.3521622
SRR17520069.3521624
SRR17520069.3521626
SRR17520069.3521628
SRR17520069.3522022
SRR17520069.3522024
SRR17520069.3522026
SRR17520069.3530380
SRR17520069.3530382
SRR17520069.3530384
SRR17520069.3530386
SRR17520069.3532375
SRR17520069.3532377
SRR17520069.3532379
SRR17520069.3532381
SRR17520069.3532383
SRR17520069.3532385
SRR17520069.3532387
SRR17520069.3532389
SRR17520069.3532391
SRR17520069.3532393
SRR17520069.3532395
SRR17520069.3532397
SRR17520069.3535951
SRR17520069.3535953
SRR17520069.3535955
SRR17520069.3535957
SRR17520069.3535959
SRR17520069.3535961
SRR17520069.3535963
SRR17520069.3535965
SRR17520069.3535967
SRR17520069.3535969
SRR17520069.3535971
SRR17520069.3535973
SRR17520069.3535975
SRR17520069.3535977
SRR17520069.3535979
SRR17520069.3535981
SRR17520069.3535983
SRR17520069.3535985
SRR17520069.3535987
SRR17520069.3535989
SRR17520069.3535991
SRR17520069.3535993
SRR17520069.3535995
SRR17520069.3535997

SRR17520069.3535999
SRR17520069.3536001
SRR17520069.3536003
SRR17520069.3536005
SRR17520069.3536007
SRR17520069.3536009
SRR17520069.3536011
SRR17520069.3536013
SRR17520069.3536015
SRR17520069.3536017
SRR17520069.3536019
SRR17520069.3536021
SRR17520069.3536023
SRR17520069.3536025
SRR17520069.3536027
SRR17520069.3536029
SRR17520069.3536031
SRR17520069.3536033
SRR17520069.3536035
SRR17520069.3536037
SRR17520069.3536039
SRR17520069.3536041
SRR17520069.3536055
SRR17520069.3536057
SRR17520069.3536059
SRR17520069.3536061
SRR17520069.3539844
SRR17520069.3539846
SRR17520069.3539848
SRR17520069.3539850
SRR17520069.3539852
SRR17520069.3539854
SRR17520069.3539856
SRR17520069.3539858
SRR17520069.3539860
SRR17520069.3539862
SRR17520069.3539864
SRR17520069.3539866
SRR17520069.3539868
SRR17520069.3539870
SRR17520069.3539872
SRR17520069.3539874
SRR17520069.3539876
SRR17520069.3539878
SRR17520069.3539880
SRR17520069.3539882
SRR17520069.3539884

SRR17520069.3539886
SRR17520069.3539888
SRR17520069.3539890
SRR17520069.3539892
SRR17520069.3539894
SRR17520069.3539896
SRR17520069.3539898
SRR17520069.3539900
SRR17520069.3539902
SRR17520069.3539906
SRR17520069.3539908
SRR17520069.3539912
SRR17520069.3539914
SRR17520069.3539916
SRR17520069.3539918
SRR17520069.3539920
SRR17520069.3539922
SRR17520069.3539924
SRR17520069.3539926
SRR17520069.3539928
SRR17520069.3539930
SRR17520069.3539932
SRR17520069.3539936
SRR17520069.3539938
SRR17520069.3539940
SRR17520069.3539942
SRR17520069.3539944
SRR17520069.3539946
SRR17520069.3539948
SRR17520069.3539950
SRR17520069.3539954
SRR17520069.3539956
SRR17520069.3540080
SRR17520069.3540087
SRR17520069.3542992
SRR17520069.3542994
SRR17520069.3542996
SRR17520069.3542998
SRR17520069.3543000
SRR17520069.3543002
SRR17520069.3543004
SRR17520069.3543006
SRR17520069.3543008
SRR17520069.3543010
SRR17520069.3543012
SRR17520069.3543014
SRR17520069.3543016

SRR17520069.3543018
SRR17520069.3543020
SRR17520069.3543022
SRR17520069.3543024
SRR17520069.3543026
SRR17520069.3543028
SRR17520069.3543030
SRR17520069.3543032
SRR17520069.3543034
SRR17520069.3543036
SRR17520069.3543038
SRR17520069.3543040
SRR17520069.3543042
SRR17520069.3543044
SRR17520069.3543046
SRR17520069.3543048
SRR17520069.3543050
SRR17520069.3544103
SRR17520069.3544105
SRR17520069.3544107
SRR17520069.3544109
SRR17520069.3544111
SRR17520069.3544119
SRR17520069.3544121
SRR17520069.3544123
SRR17520069.3544125
SRR17520069.3544127
SRR17520069.3544129
SRR17520069.3544131
SRR17520069.3544133
SRR17520069.3544135
SRR17520069.3544137
SRR17520069.3544139
SRR17520069.3544162
SRR17520069.3544631
SRR17520069.3544633
SRR17520069.3544635
SRR17520069.3544637
SRR17520069.3544639
SRR17520069.3544641
SRR17520069.3544643
SRR17520069.3544645
SRR17520069.3544647
SRR17520069.3544651
SRR17520069.3544653
SRR17520069.3544655
SRR17520069.3544657

SRR17520069.3544659
SRR17520069.3544661
SRR17520069.3544663
SRR17520069.3544667
SRR17520069.3544671
SRR17520069.3544673
SRR17520069.3544675
SRR17520069.3544677
SRR17520069.3544679
SRR17520069.3544681
SRR17520069.3544683
SRR17520069.3545265
SRR17520069.3545269
SRR17520069.3545273
SRR17520069.3545275
SRR17520069.3545277
SRR17520069.3545279
SRR17520069.3545281
SRR17520069.3545283
SRR17520069.3545287
SRR17520069.3545289
SRR17520069.3545293
SRR17520069.3545295
SRR17520069.3545297
SRR17520069.3545299
SRR17520069.3545301
SRR17520069.3545303
SRR17520069.3545305
SRR17520069.3545309
SRR17520069.3545311
SRR17520069.3545313
SRR17520069.3545315
SRR17520069.3545317
SRR17520069.3545319
SRR17520069.3545321
SRR17520069.3545323
SRR17520069.3545325
SRR17520069.3545327
SRR17520069.3545329
SRR17520069.3545331
SRR17520069.3545337
SRR17520069.3545339
SRR17520069.3545341
SRR17520069.3545343
SRR17520069.3545345
SRR17520069.3545349
SRR17520069.3545351

SRR17520069.3545353
SRR17520069.3552366
SRR17520069.3552368
SRR17520069.3552370
SRR17520069.3552372
SRR17520069.3552374
SRR17520069.3552378
SRR17520069.3552380
SRR17520069.3552382
SRR17520069.3552384
SRR17520069.3552386
SRR17520069.3555578
SRR17520069.3555580
SRR17520069.3555582
SRR17520069.3555584
SRR17520069.3555586
SRR17520069.3555588
SRR17520069.3555590
SRR17520069.3555592
SRR17520069.3555596
SRR17520069.3555598
SRR17520069.3555600
SRR17520069.3555602
SRR17520069.3555604
SRR17520069.3555606
SRR17520069.3555608
SRR17520069.3555610
SRR17520069.3555612
SRR17520069.3555614
SRR17520069.3555616
SRR17520069.3555618
SRR17520069.3555620
SRR17520069.3555624
SRR17520069.3555626
SRR17520069.3555628
SRR17520069.3555630
SRR17520069.3555632
SRR17520069.3555634
SRR17520069.3555636
SRR17520069.3555638
SRR17520069.3555640
SRR17520069.3555642
SRR17520069.3555644
SRR17520069.3555646
SRR17520069.3555648
SRR17520069.3555650
SRR17520069.3555652

SRR17520069.3555654
SRR17520069.3555656
SRR17520069.3555660
SRR17520069.3555662
SRR17520069.3555664
SRR17520069.3555666
SRR17520069.3555668
SRR17520069.3555670
SRR17520069.3563561
SRR17520069.3563563
SRR17520069.3563565
SRR17520069.3563567
SRR17520069.3563569
SRR17520069.3563571
SRR17520069.3563573
SRR17520069.3563575
SRR17520069.3563577
SRR17520069.3563579
SRR17520069.3563581
SRR17520069.3563583
SRR17520069.3563585
SRR17520069.3563587
SRR17520069.3563589
SRR17520069.3563591
SRR17520069.3563593
SRR17520069.3563595
SRR17520069.3563597
SRR17520069.3563599
SRR17520069.3563601
SRR17520069.3563603
SRR17520069.3563605
SRR17520069.3563607
SRR17520069.3563609
SRR17520069.3563611
SRR17520069.3563613
SRR17520069.3563615
SRR17520069.3563617
SRR17520069.3563619
SRR17520069.3563621
SRR17520069.3563623
SRR17520069.3563625
SRR17520069.3563627
SRR17520069.3563631
SRR17520069.3563633
SRR17520069.3563635
SRR17520069.3563637
SRR17520069.3563639

SRR17520069.3563641
SRR17520069.3563643
SRR17520069.3563645
SRR17520069.3563647
SRR17520069.3563649
SRR17520069.3563651
SRR17520069.3563653
SRR17520069.3563737
SRR17520069.3563739
SRR17520069.3563741
SRR17520069.3563743
SRR17520069.3563745
SRR17520069.3563747
SRR17520069.3563751
SRR17520069.3563753
SRR17520069.3563755
SRR17520069.3564895
SRR17520069.3564898
SRR17520069.3564900
SRR17520069.3564902
SRR17520069.3564904
SRR17520069.3564908
SRR17520069.3564910
SRR17520069.3564912
SRR17520069.3564914
SRR17520069.3564916
SRR17520069.3564920
SRR17520069.3564924
SRR17520069.3564926
SRR17520069.3568926
SRR17520069.3568930
SRR17520069.3568932
SRR17520069.3568934
SRR17520069.3568936
SRR17520069.3568938
SRR17520069.3568940
SRR17520069.3568942
SRR17520069.3568944
SRR17520069.3568946
SRR17520069.3568948
SRR17520069.3568950
SRR17520069.3568952
SRR17520069.3568954
SRR17520069.3568956
SRR17520069.3568958
SRR17520069.3568960
SRR17520069.3568962

SRR17520069.3568964
SRR17520069.3568966
SRR17520069.3568968
SRR17520069.3568970
SRR17520069.3568972
SRR17520069.3568974
SRR17520069.3568976
SRR17520069.3568978
SRR17520069.3568980
SRR17520069.3568982
SRR17520069.3568984
SRR17520069.3568986
SRR17520069.3568988
SRR17520069.3568990
SRR17520069.3568992
SRR17520069.3568994
SRR17520069.3568996
SRR17520069.3568998
SRR17520069.3569000
SRR17520069.3569002
SRR17520069.3569004
SRR17520069.3569006
SRR17520069.3569008
SRR17520069.3569010
SRR17520069.3569012
SRR17520069.3569014
SRR17520069.3569016
SRR17520069.3569018
SRR17520069.3569020
SRR17520069.3569022
SRR17520069.3573552
SRR17520069.3573554
SRR17520069.3573556
SRR17520069.3573558
SRR17520069.3573560
SRR17520069.3573562
SRR17520069.3573564
SRR17520069.3573566
SRR17520069.3573568
SRR17520069.3573570
SRR17520069.3573572
SRR17520069.3573574
SRR17520069.3573576
SRR17520069.3573578
SRR17520069.3573580
SRR17520069.3573582
SRR17520069.3573584

SRR17520069.3573586
SRR17520069.3573588
SRR17520069.3573590
SRR17520069.3573592
SRR17520069.3573594
SRR17520069.3573596
SRR17520069.3573598
SRR17520069.3573600
SRR17520069.3573602
SRR17520069.3573604
SRR17520069.3573606
SRR17520069.3573608
SRR17520069.3573610
SRR17520069.3573612
SRR17520069.3573614
SRR17520069.3573616
SRR17520069.3573618
SRR17520069.3573620
SRR17520069.3573622
SRR17520069.3573775
SRR17520069.3573779
SRR17520069.3573783
SRR17520069.3578283
SRR17520069.3578285
SRR17520069.3578287
SRR17520069.3578289
SRR17520069.3578293
SRR17520069.3578295
SRR17520069.3578297
SRR17520069.3578299
SRR17520069.3578301
SRR17520069.3578303
SRR17520069.3578305
SRR17520069.3578307
SRR17520069.3578309
SRR17520069.3578311
SRR17520069.3578313
SRR17520069.3578315
SRR17520069.3578317
SRR17520069.3578319
SRR17520069.3578321
SRR17520069.3578323
SRR17520069.3578325
SRR17520069.3578327
SRR17520069.3578329
SRR17520069.3578331
SRR17520069.3578333

SRR17520069.3578335
SRR17520069.3578337
SRR17520069.3578341
SRR17520069.3578343
SRR17520069.3578345
SRR17520069.3578347
SRR17520069.3578794
SRR17520069.3578832
SRR17520069.3580941
SRR17520069.3580943
SRR17520069.3584062
SRR17520069.3584064
SRR17520069.3584350
SRR17520069.3584352
SRR17520069.3584354
SRR17520069.3584356
SRR17520069.3584358
SRR17520069.3584360
SRR17520069.3584362
SRR17520069.3584364
SRR17520069.3584366
SRR17520069.3584368
SRR17520069.3584370
SRR17520069.3584372
SRR17520069.3584374
SRR17520069.3584376
SRR17520069.3594518
SRR17520069.3594520
SRR17520069.3594522
SRR17520069.3594524
SRR17520069.3602287
SRR17520069.3602289
SRR17520069.3602291
SRR17520069.3602293
SRR17520069.3602295
SRR17520069.3602297
SRR17520069.3602299
SRR17520069.3602301
SRR17520069.3602303
SRR17520069.3602305
SRR17520069.3602307
SRR17520069.3602309
SRR17520069.3602313
SRR17520069.3602315
SRR17520069.3602317
SRR17520069.3608221
SRR17520069.3608223

SRR17520069.3608225
SRR17520069.3608229
SRR17520069.3608231
SRR17520069.3608233
SRR17520069.3608235
SRR17520069.3608237
SRR17520069.3608239
SRR17520069.3608314
SRR17520069.3608316
SRR17520069.3608318
SRR17520069.3615895
SRR17520069.3615897
SRR17520069.3615899
SRR17520069.3615901
SRR17520069.3615903
SRR17520069.3615905
SRR17520069.3615907
SRR17520069.3615909
SRR17520069.3615911
SRR17520069.3615913
SRR17520069.3615915
SRR17520069.3615917
SRR17520069.3615919
SRR17520069.3615921
SRR17520069.3615923
SRR17520069.3615925
SRR17520069.3615929
SRR17520069.3615931
SRR17520069.3615933
SRR17520069.3615935
SRR17520069.3615937
SRR17520069.3615939
SRR17520069.3615941
SRR17520069.3615943
SRR17520069.3615945
SRR17520069.3615947
SRR17520069.3615949
SRR17520069.3615951
SRR17520069.3615953
SRR17520069.3615955
SRR17520069.3615957
SRR17520069.3615959
SRR17520069.3615961
SRR17520069.3615963
SRR17520069.3615965
SRR17520069.3615967
SRR17520069.3615969

SRR17520069.3615971
SRR17520069.3627996
SRR17520069.3627998
SRR17520069.3628002
SRR17520069.3628004
SRR17520069.3628006
SRR17520069.3628008
SRR17520069.3628010
SRR17520069.3628012
SRR17520069.3628014
SRR17520069.3628016
SRR17520069.3628018
SRR17520069.3628022
SRR17520069.3628024
SRR17520069.3628026
SRR17520069.3628030
SRR17520069.3628032
SRR17520069.3628034
SRR17520069.3628036
SRR17520069.3628038
SRR17520069.3628040
SRR17520069.3628042
SRR17520069.3628044
SRR17520069.3628046
SRR17520069.3628048
SRR17520069.3628050
SRR17520069.3628052
SRR17520069.3628056
SRR17520069.3630104
SRR17520069.3632219
SRR17520069.3632221
SRR17520069.3632223
SRR17520069.3632225
SRR17520069.3632227
SRR17520069.3632229
SRR17520069.3632231
SRR17520069.3632233
SRR17520069.3632235
SRR17520069.3632237
SRR17520069.3632239
SRR17520069.3632241
SRR17520069.3632243
SRR17520069.3632245
SRR17520069.3632247
SRR17520069.3632249
SRR17520069.3632251
SRR17520069.3632253

SRR17520069.3633907
SRR17520069.3633909
SRR17520069.3633911
SRR17520069.3633913
SRR17520069.3633917
SRR17520069.3633919
SRR17520069.3633921
SRR17520069.3633923
SRR17520069.3633925
SRR17520069.3633927
SRR17520069.3633929
SRR17520069.3633931
SRR17520069.3633933
SRR17520069.3633935
SRR17520069.3633937
SRR17520069.3633939
SRR17520069.3633941
SRR17520069.3633943
SRR17520069.3633945
SRR17520069.3636288
SRR17520069.3636290
SRR17520069.3636292
SRR17520069.3636294
SRR17520069.3636296
SRR17520069.3636298
SRR17520069.3636300
SRR17520069.3636302
SRR17520069.3636304
SRR17520069.3636306
SRR17520069.3636308
SRR17520069.3636310
SRR17520069.3636312
SRR17520069.3636314
SRR17520069.3636316
SRR17520069.3636318
SRR17520069.3636320
SRR17520069.3636821
SRR17520069.3641996
SRR17520069.3641998
SRR17520069.3642000
SRR17520069.3642002
SRR17520069.3642004
SRR17520069.3642006
SRR17520069.3642008
SRR17520069.3642010
SRR17520069.3642012
SRR17520069.3642014

SRR17520069.3642016
SRR17520069.3642018
SRR17520069.3642020
SRR17520069.3642022
SRR17520069.3642024
SRR17520069.3642026
SRR17520069.3642028
SRR17520069.3642030
SRR17520069.3642032
SRR17520069.3642034
SRR17520069.3642036
SRR17520069.3642038
SRR17520069.3642040
SRR17520069.3642042
SRR17520069.3642044
SRR17520069.3642046
SRR17520069.3642048
SRR17520069.3642050
SRR17520069.3642052
SRR17520069.3642054
SRR17520069.3642056
SRR17520069.3642058
SRR17520069.3642060
SRR17520069.3642062
SRR17520069.3642064
SRR17520069.3642066
SRR17520069.3642068
SRR17520069.3642070
SRR17520069.3642072
SRR17520069.3642074
SRR17520069.3642076
SRR17520069.3642078
SRR17520069.3642080
SRR17520069.3642082
SRR17520069.3642084
SRR17520069.3642086
SRR17520069.3642088
SRR17520069.3642090
SRR17520069.3643959
SRR17520069.3643961
SRR17520069.3644288
SRR17520069.3644663
SRR17520069.3644665
SRR17520069.3644667
SRR17520069.3644669
SRR17520069.3644671
SRR17520069.3644673

SRR17520069.3644675
SRR17520069.3644677
SRR17520069.3644679
SRR17520069.3644681
SRR17520069.3644683
SRR17520069.3644685
SRR17520069.3644687
SRR17520069.3644689
SRR17520069.3644691
SRR17520069.3644693
SRR17520069.3644695
SRR17520069.3644697
SRR17520069.3645367
SRR17520069.3645369
SRR17520069.3645371
SRR17520069.3645373
SRR17520069.3645375
SRR17520069.3645377
SRR17520069.3645379
SRR17520069.3645381
SRR17520069.3645383
SRR17520069.3645385
SRR17520069.3645389
SRR17520069.3645391
SRR17520069.3645393
SRR17520069.3645395
SRR17520069.3645397
SRR17520069.3645403
SRR17520069.3645407
SRR17520069.3645409
SRR17520069.3645411
SRR17520069.3645413
SRR17520069.3645415
SRR17520069.3645417
SRR17520069.3645421
SRR17520069.3645423
SRR17520069.3645427
SRR17520069.3645429
SRR17520069.3645437
SRR17520069.3645439
SRR17520069.3645441
SRR17520069.3645445
SRR17520069.3649559
SRR17520069.3649561
SRR17520069.3653659
SRR17520069.3653661
SRR17520069.3653663

SRR17520069.3653665
SRR17520069.3653667
SRR17520069.3653669
SRR17520069.3653671
SRR17520069.3653673
SRR17520069.3653675
SRR17520069.3653677
SRR17520069.3653679
SRR17520069.3653681
SRR17520069.3653683
SRR17520069.3653685
SRR17520069.3657808
SRR17520069.3657810
SRR17520069.3657812
SRR17520069.3657814
SRR17520069.3657816
SRR17520069.3657818
SRR17520069.3657820
SRR17520069.3657822
SRR17520069.3657824
SRR17520069.3657826
SRR17520069.3657828
SRR17520069.3657830
SRR17520069.3657832
SRR17520069.3657834
SRR17520069.3657836
SRR17520069.3657838
SRR17520069.3657840
SRR17520069.3657842
SRR17520069.3657844
SRR17520069.3657846
SRR17520069.3657848
SRR17520069.3657850
SRR17520069.3657852
SRR17520069.3657854
SRR17520069.3657856
SRR17520069.3657858
SRR17520069.3657860
SRR17520069.3657862
SRR17520069.3657864
SRR17520069.3657866
SRR17520069.3657868
SRR17520069.3657870
SRR17520069.3657872
SRR17520069.3657874
SRR17520069.3657876
SRR17520069.3657878

SRR17520069.3657880
SRR17520069.3657882
SRR17520069.3657884
SRR17520069.3657886
SRR17520069.3657888
SRR17520069.3657890
SRR17520069.3657892
SRR17520069.3662689
SRR17520069.3662691
SRR17520069.3662693
SRR17520069.3662695
SRR17520069.3662697
SRR17520069.3662699
SRR17520069.3662701
SRR17520069.3662703
SRR17520069.3662705
SRR17520069.3662707
SRR17520069.3662709
SRR17520069.3662711
SRR17520069.3662713
SRR17520069.3662715
SRR17520069.3662717
SRR17520069.3662719
SRR17520069.3662724
SRR17520069.3662726
SRR17520069.3662728
SRR17520069.3662730
SRR17520069.3662732
SRR17520069.3662734
SRR17520069.3662736
SRR17520069.3662738
SRR17520069.3662740
SRR17520069.3662742
SRR17520069.3662744
SRR17520069.3662746
SRR17520069.3662748
SRR17520069.3662750
SRR17520069.3662752
SRR17520069.3662754
SRR17520069.3662756
SRR17520069.3662758
SRR17520069.3662760
SRR17520069.3662762
SRR17520069.3662764
SRR17520069.3662766
SRR17520069.3662768
SRR17520069.3662770

SRR17520069.3662772
SRR17520069.3662774
SRR17520069.3662776
SRR17520069.3662778
SRR17520069.3662780
SRR17520069.3662782
SRR17520069.3662784
SRR17520069.3662786
SRR17520069.3662788
SRR17520069.3662790
SRR17520069.3662792
SRR17520069.3662794
SRR17520069.3662796
SRR17520069.3662800
SRR17520069.3662802
SRR17520069.3662804
SRR17520069.3662806
SRR17520069.3662808
SRR17520069.3662810
SRR17520069.3662812
SRR17520069.3662814
SRR17520069.3662816
SRR17520069.3662818
SRR17520069.3662820
SRR17520069.3662822
SRR17520069.3662824
SRR17520069.3662826
SRR17520069.3662828
SRR17520069.3662830
SRR17520069.3662832
SRR17520069.3662834
SRR17520069.3662836
SRR17520069.3662838
SRR17520069.3662840
SRR17520069.3662844
SRR17520069.3662846
SRR17520069.3662848
SRR17520069.3662850
SRR17520069.3662852
SRR17520069.3662854
SRR17520069.3662856
SRR17520069.3662860
SRR17520069.3662862
SRR17520069.3662864
SRR17520069.3662866
SRR17520069.3662868
SRR17520069.3662870

SRR17520069.3662872
SRR17520069.3662874
SRR17520069.3662876
SRR17520069.3662882
SRR17520069.3662884
SRR17520069.3662886
SRR17520069.3662888
SRR17520069.3662890
SRR17520069.3662892
SRR17520069.3662894
SRR17520069.3662896
SRR17520069.3663180
SRR17520069.3663182
SRR17520069.3663184
SRR17520069.3663186
SRR17520069.3663188
SRR17520069.3663190
SRR17520069.3663192
SRR17520069.3663194
SRR17520069.3663196
SRR17520069.3663198
SRR17520069.3663200
SRR17520069.3663202
SRR17520069.3663204
SRR17520069.3665813
SRR17520069.3665815
SRR17520069.3665817
SRR17520069.3665819
SRR17520069.3665821
SRR17520069.3665823
SRR17520069.3665825
SRR17520069.3665827
SRR17520069.3665829
SRR17520069.3665831
SRR17520069.3665833
SRR17520069.3665835
SRR17520069.3665837
SRR17520069.3665839
SRR17520069.3665841
SRR17520069.3665843
SRR17520069.3665845
SRR17520069.3665847
SRR17520069.3665849
SRR17520069.3665851
SRR17520069.3665853
SRR17520069.3665855
SRR17520069.3665857

SRR17520069.3665859
SRR17520069.3665861
SRR17520069.3665863
SRR17520069.3665865
SRR17520069.3665867
SRR17520069.3665869
SRR17520069.3665871
SRR17520069.3665873
SRR17520069.3666409
SRR17520069.3666411
SRR17520069.3666413
SRR17520069.3666415
SRR17520069.3666417
SRR17520069.3666419
SRR17520069.3666421
SRR17520069.3666423
SRR17520069.3666425
SRR17520069.3666427
SRR17520069.3666429
SRR17520069.3666431
SRR17520069.3666433
SRR17520069.3666435
SRR17520069.3666437
SRR17520069.3666439
SRR17520069.3666441
SRR17520069.3666443
SRR17520069.3666445
SRR17520069.3666447
SRR17520069.3666449
SRR17520069.3666451
SRR17520069.3666453
SRR17520069.3666455
SRR17520069.3666457
SRR17520069.3666459
SRR17520069.3666461
SRR17520069.3666463
SRR17520069.3666465
SRR17520069.3666467
SRR17520069.3666469
SRR17520069.3666471
SRR17520069.3667953
SRR17520069.3667955
SRR17520069.3667957
SRR17520069.3667959
SRR17520069.3667961
SRR17520069.3667963
SRR17520069.3667965

SRR17520069.3667967
SRR17520069.3667969
SRR17520069.3667971
SRR17520069.3667975
SRR17520069.3667977
SRR17520069.3667979
SRR17520069.3667981
SRR17520069.3667983
SRR17520069.3667985
SRR17520069.3667987
SRR17520069.3667989
SRR17520069.3667991
SRR17520069.3667993
SRR17520069.3667995
SRR17520069.3667997
SRR17520069.3667999
SRR17520069.3668001
SRR17520069.3668003
SRR17520069.3668005
SRR17520069.3668007
SRR17520069.3668009
SRR17520069.3668011
SRR17520069.3668013
SRR17520069.3668015
SRR17520069.3668017
SRR17520069.3668019
SRR17520069.3668021
SRR17520069.3668023
SRR17520069.3668025
SRR17520069.3668027
SRR17520069.3668029
SRR17520069.3668031
SRR17520069.3668033
SRR17520069.3668035
SRR17520069.3668037
SRR17520069.3668472
SRR17520069.3668474
SRR17520069.3668476
SRR17520069.3668478
SRR17520069.3668480
SRR17520069.3668482
SRR17520069.3668484
SRR17520069.3668486
SRR17520069.3668488
SRR17520069.3668490
SRR17520069.3668492
SRR17520069.3668496

SRR17520069.3668498
SRR17520069.3668500
SRR17520069.3668502
SRR17520069.3668504
SRR17520069.3668506
SRR17520069.3668508
SRR17520069.3668510
SRR17520069.3668512
SRR17520069.3668514
SRR17520069.3668516
SRR17520069.3668518
SRR17520069.3668520
SRR17520069.3668524
SRR17520069.3668526
SRR17520069.3668528
SRR17520069.3668530
SRR17520069.3668532
SRR17520069.3668534
SRR17520069.3668536
SRR17520069.3668538
SRR17520069.3668540
SRR17520069.3668542
SRR17520069.3668544
SRR17520069.3668546
SRR17520069.3668548
SRR17520069.3668550
SRR17520069.3668552
SRR17520069.3668554
SRR17520069.3668556
SRR17520069.3668558
SRR17520069.3668560
SRR17520069.3668562
SRR17520069.3668564
SRR17520069.3668566
SRR17520069.3668568
SRR17520069.3668570
SRR17520069.3668572
SRR17520069.3668574
SRR17520069.3668576
SRR17520069.3668578
SRR17520069.3668580
SRR17520069.3668584
SRR17520069.3668586
SRR17520069.3668588
SRR17520069.3668590
SRR17520069.3668592
SRR17520069.3668594

SRR17520069.3668596
SRR17520069.3668598
SRR17520069.3668600
SRR17520069.3668602
SRR17520069.3668604
SRR17520069.3668606
SRR17520069.3670952
SRR17520069.3670954
SRR17520069.3672722
SRR17520069.3672724
SRR17520069.3672726
SRR17520069.3672728
SRR17520069.3672730
SRR17520069.3672732
SRR17520069.3672734
SRR17520069.3672736
SRR17520069.3672738
SRR17520069.3672740
SRR17520069.3672742
SRR17520069.3672744
SRR17520069.3672746
SRR17520069.3672748
SRR17520069.3672750
SRR17520069.3672752
SRR17520069.3672754
SRR17520069.3672756
SRR17520069.3672758
SRR17520069.3674697
SRR17520069.3674699
SRR17520069.3674701
SRR17520069.3674703
SRR17520069.3674705
SRR17520069.3674707
SRR17520069.3674709
SRR17520069.3674711
SRR17520069.3675019
SRR17520069.3675021
SRR17520069.3675025
SRR17520069.3679689
SRR17520069.3679691
SRR17520069.3679693
SRR17520069.3679695
SRR17520069.3679697
SRR17520069.3679699
SRR17520069.3679701
SRR17520069.3679703
SRR17520069.3679705

SRR17520069.3679707
SRR17520069.3679709
SRR17520069.3679711
SRR17520069.3679713
SRR17520069.3679715
SRR17520069.3679717
SRR17520069.3679719
SRR17520069.3679721
SRR17520069.3679725
SRR17520069.3679727
SRR17520069.3680774
SRR17520069.3680776
SRR17520069.3680778
SRR17520069.3680780
SRR17520069.3680782
SRR17520069.3680784
SRR17520069.3680786
SRR17520069.3680792
SRR17520069.3680794
SRR17520069.3680796
SRR17520069.3680798
SRR17520069.3680800
SRR17520069.3680802
SRR17520069.3686566
SRR17520069.3686568
SRR17520069.3686570
SRR17520069.3686572
SRR17520069.3686574
SRR17520069.3686576
SRR17520069.3686578
SRR17520069.3686580
SRR17520069.3686582
SRR17520069.3686584
SRR17520069.3686586
SRR17520069.3686588
SRR17520069.3686590
SRR17520069.3686592
SRR17520069.3688351
SRR17520069.3688353
SRR17520069.3688355
SRR17520069.3688357
SRR17520069.3688359
SRR17520069.3688363
SRR17520069.3688365
SRR17520069.3702736
SRR17520069.3702738
SRR17520069.3702740

SRR17520069.3702742
SRR17520069.3702744
SRR17520069.3702746
SRR17520069.3702748
SRR17520069.3702750
SRR17520069.3702752
SRR17520069.3702754
SRR17520069.3702756
SRR17520069.3702758
SRR17520069.3702760
SRR17520069.3702762
SRR17520069.3702764
SRR17520069.3702766
SRR17520069.3702768
SRR17520069.3702770
SRR17520069.3702772
SRR17520069.3702774
SRR17520069.3702776
SRR17520069.3702778
SRR17520069.3702780
SRR17520069.3702782
SRR17520069.3702784
SRR17520069.3702786
SRR17520069.3702788
SRR17520069.3702790
SRR17520069.3702792
SRR17520069.3702794
SRR17520069.3702796
SRR17520069.3702798
SRR17520069.3702800
SRR17520069.3702802
SRR17520069.3702804
SRR17520069.3702806
SRR17520069.3702808
SRR17520069.3710416
SRR17520069.3710418
SRR17520069.3710420
SRR17520069.3710422
SRR17520069.3710424
SRR17520069.3710426
SRR17520069.3710428
SRR17520069.3710430
SRR17520069.3710432
SRR17520069.3710434
SRR17520069.3710436
SRR17520069.3710438
SRR17520069.3710440

SRR17520069.3710442
SRR17520069.3710444
SRR17520069.3710446
SRR17520069.3710448
SRR17520069.3710450
SRR17520069.3710452
SRR17520069.3710454
SRR17520069.3710456
SRR17520069.3710460
SRR17520069.3710462
SRR17520069.3710464
SRR17520069.3710466
SRR17520069.3710468
SRR17520069.3710470
SRR17520069.3710474
SRR17520069.3710476
SRR17520069.3710478
SRR17520069.3710480
SRR17520069.3710482
SRR17520069.3710484
SRR17520069.3710486
SRR17520069.3710488
SRR17520069.3710490
SRR17520069.3710492
SRR17520069.3712068
SRR17520069.3712070
SRR17520069.3712072
SRR17520069.3712074
SRR17520069.3712076
SRR17520069.3712078
SRR17520069.3712080
SRR17520069.3712082
SRR17520069.3712084
SRR17520069.3712086
SRR17520069.3712088
SRR17520069.3712090
SRR17520069.3712092
SRR17520069.3712094
SRR17520069.3712096
SRR17520069.3712098
SRR17520069.3712100
SRR17520069.3712102
SRR17520069.3712104
SRR17520069.3712106
SRR17520069.3712108
SRR17520069.3712110
SRR17520069.3712112

SRR17520069.3712114
SRR17520069.3712116
SRR17520069.3712118
SRR17520069.3712122
SRR17520069.3712124
SRR17520069.3712126
SRR17520069.3712128
SRR17520069.3712130
SRR17520069.3712132
SRR17520069.3712134
SRR17520069.3715768
SRR17520069.3715770
SRR17520069.3715772
SRR17520069.3715774
SRR17520069.3715776
SRR17520069.3715778
SRR17520069.3715780
SRR17520069.3715782
SRR17520069.3715786
SRR17520069.3715788
SRR17520069.3716038
SRR17520069.3722045
SRR17520069.3722047
SRR17520069.3722049
SRR17520069.3722051
SRR17520069.3722053
SRR17520069.3722055
SRR17520069.3722059
SRR17520069.3722061
SRR17520069.3722063
SRR17520069.3722065
SRR17520069.3722067
SRR17520069.3722069
SRR17520069.3722071
SRR17520069.3722073
SRR17520069.3722075
SRR17520069.3722077
SRR17520069.3726283
SRR17520069.3726285
SRR17520069.3726287
SRR17520069.3726289
SRR17520069.3726291
SRR17520069.3726293
SRR17520069.3726295
SRR17520069.3726297
SRR17520069.3726299
SRR17520069.3726301

SRR17520069.3726303
SRR17520069.3726305
SRR17520069.3726307
SRR17520069.3726309
SRR17520069.3726311
SRR17520069.3726313
SRR17520069.3726315
SRR17520069.3726317
SRR17520069.3726319
SRR17520069.3726321
SRR17520069.3726323
SRR17520069.3726325
SRR17520069.3726327
SRR17520069.3726329
SRR17520069.3726331
SRR17520069.3726333
SRR17520069.3726335
SRR17520069.3726337
SRR17520069.3726339
SRR17520069.3726341
SRR17520069.3726343
SRR17520069.3726345
SRR17520069.3726347
SRR17520069.3726349
SRR17520069.3726351
SRR17520069.3726353
SRR17520069.3726355
SRR17520069.3726357
SRR17520069.3726359
SRR17520069.3726361
SRR17520069.3726363
SRR17520069.3726365
SRR17520069.3726370
SRR17520069.3726372
SRR17520069.3726374
SRR17520069.3726376
SRR17520069.3726378
SRR17520069.3729227
SRR17520069.3729229
SRR17520069.3729231
SRR17520069.3729233
SRR17520069.3729235
SRR17520069.3729237
SRR17520069.3729239
SRR17520069.3729241
SRR17520069.3729243
SRR17520069.3729247

SRR17520069.3730119
SRR17520069.3730121
SRR17520069.3730123
SRR17520069.3730125
SRR17520069.3730127
SRR17520069.3730129
SRR17520069.3730131
SRR17520069.3730133
SRR17520069.3730135
SRR17520069.3730137
SRR17520069.3730139
SRR17520069.3730141
SRR17520069.3730143
SRR17520069.3730145
SRR17520069.3730147
SRR17520069.3730149
SRR17520069.3730151
SRR17520069.3730153
SRR17520069.3730157
SRR17520069.3730159
SRR17520069.3730161
SRR17520069.3730163
SRR17520069.3730165
SRR17520069.3730167
SRR17520069.3730169
SRR17520069.3730171
SRR17520069.3730173
SRR17520069.3730175
SRR17520069.3730177
SRR17520069.3730179
SRR17520069.3730181
SRR17520069.3730183
SRR17520069.3730185
SRR17520069.3730187
SRR17520069.3730189
SRR17520069.3730191
SRR17520069.3730193
SRR17520069.3730195
SRR17520069.3730197
SRR17520069.3730199
SRR17520069.3730201
SRR17520069.3730203
SRR17520069.3730205
SRR17520069.3730207
SRR17520069.3730209
SRR17520069.3730211
SRR17520069.3730213

SRR17520069.3732787
SRR17520069.3732789
SRR17520069.3732791
SRR17520069.3732793
SRR17520069.3732795
SRR17520069.3732797
SRR17520069.3732799
SRR17520069.3732801
SRR17520069.3732803
SRR17520069.3732805
SRR17520069.3732807
SRR17520069.3732809
SRR17520069.3732811
SRR17520069.3732813
SRR17520069.3732815
SRR17520069.3732817
SRR17520069.3732819
SRR17520069.3732821
SRR17520069.3732823
SRR17520069.3732825
SRR17520069.3732827
SRR17520069.3732829
SRR17520069.3732831
SRR17520069.3732833
SRR17520069.3732835
SRR17520069.3732837
SRR17520069.3732839
SRR17520069.3732841
SRR17520069.3732843
SRR17520069.3732847
SRR17520069.3732849
SRR17520069.3732851
SRR17520069.3732853
SRR17520069.3732855
SRR17520069.3732857
SRR17520069.3732859
SRR17520069.3732861
SRR17520069.3732863
SRR17520069.3732865
SRR17520069.3732867
SRR17520069.3732869
SRR17520069.3732871
SRR17520069.3732873
SRR17520069.3732875
SRR17520069.3732877
SRR17520069.3732879
SRR17520069.3732883

SRR17520069.3732885
SRR17520069.3732887
SRR17520069.3732889
SRR17520069.3732891
SRR17520069.3732893
SRR17520069.3732895
SRR17520069.3732897
SRR17520069.3732899
SRR17520069.3732901
SRR17520069.3732905
SRR17520069.3732907
SRR17520069.3732911
SRR17520069.3732913
SRR17520069.3732915
SRR17520069.3732919
SRR17520069.3732921
SRR17520069.3732923
SRR17520069.3732927
SRR17520069.3732929
SRR17520069.3732931
SRR17520069.3732933
SRR17520069.3732935
SRR17520069.3732937
SRR17520069.3732939
SRR17520069.3732945
SRR17520069.3737367
SRR17520069.3737369
SRR17520069.3737371
SRR17520069.3737373
SRR17520069.3737375
SRR17520069.3737377
SRR17520069.3737379
SRR17520069.3737381
SRR17520069.3737383
SRR17520069.3737385
SRR17520069.3737387
SRR17520069.3738198
SRR17520069.3738200
SRR17520069.3738202
SRR17520069.3738206
SRR17520069.3738208
SRR17520069.3738210
SRR17520069.3738212
SRR17520069.3744988
SRR17520069.3744990
SRR17520069.3744994
SRR17520069.3744996

SRR17520069.3744998
SRR17520069.3745000
SRR17520069.3745733
SRR17520069.3745735
SRR17520069.3745737
SRR17520069.3745739
SRR17520069.3745741
SRR17520069.3745743
SRR17520069.3745745
SRR17520069.3748483
SRR17520069.3748485
SRR17520069.3751880
SRR17520069.3751882
SRR17520069.3751884
SRR17520069.3751886
SRR17520069.3751888
SRR17520069.3751890
SRR17520069.3751892
SRR17520069.3751896
SRR17520069.3751898
SRR17520069.3751900
SRR17520069.3751902
SRR17520069.3751904
SRR17520069.3751906
SRR17520069.3751908
SRR17520069.3751910
SRR17520069.3751912
SRR17520069.3751914
SRR17520069.3751916
SRR17520069.3751918
SRR17520069.3762377
SRR17520069.3762379
SRR17520069.3762381
SRR17520069.3762383
SRR17520069.3762385
SRR17520069.3762387
SRR17520069.3762389
SRR17520069.3762391
SRR17520069.3762393
SRR17520069.3762395
SRR17520069.3762397
SRR17520069.3762399
SRR17520069.3762401
SRR17520069.3762403
SRR17520069.3762405
SRR17520069.3762407
SRR17520069.3762409

SRR17520069.3766735
SRR17520069.3766737
SRR17520069.3766745
SRR17520069.3766747
SRR17520069.3766749
SRR17520069.3766751
SRR17520069.3766753
SRR17520069.3766755
SRR17520069.3766757
SRR17520069.3766759
SRR17520069.3766761
SRR17520069.3766763
SRR17520069.3766765
SRR17520069.3766767
SRR17520069.3766769
SRR17520069.3766771
SRR17520069.3766773
SRR17520069.3766775
SRR17520069.3766777
SRR17520069.3766779
SRR17520069.3766781
SRR17520069.3766783
SRR17520069.3766785
SRR17520069.3766787
SRR17520069.3766789
SRR17520069.3766791
SRR17520069.3766793
SRR17520069.3766795
SRR17520069.3766797
SRR17520069.3766799
SRR17520069.3766801
SRR17520069.3766803
SRR17520069.3766805
SRR17520069.3766807
SRR17520069.3766809
SRR17520069.3766811
SRR17520069.3766813
SRR17520069.3766815
SRR17520069.3766817
SRR17520069.3770144
SRR17520069.3770146
SRR17520069.3770148
SRR17520069.3770150
SRR17520069.3770152
SRR17520069.3770154
SRR17520069.3770158
SRR17520069.3770160

SRR17520069.3770162
SRR17520069.3770164
SRR17520069.3770166
SRR17520069.3770168
SRR17520069.3770170
SRR17520069.3770172
SRR17520069.3770174
SRR17520069.3772233
SRR17520069.3772235
SRR17520069.3772237
SRR17520069.3772239
SRR17520069.3772241
SRR17520069.3772243
SRR17520069.3772245
SRR17520069.3772432
SRR17520069.3772434
SRR17520069.3772436
SRR17520069.3772438
SRR17520069.3772440
SRR17520069.3772442
SRR17520069.3772444
SRR17520069.3772446
SRR17520069.3772448
SRR17520069.3772450
SRR17520069.3772452
SRR17520069.3772454
SRR17520069.3772456
SRR17520069.3772458
SRR17520069.3772460
SRR17520069.3772462
SRR17520069.3772464
SRR17520069.3772468
SRR17520069.3772470
SRR17520069.3772472
SRR17520069.3772474
SRR17520069.3776258
SRR17520069.3776260
SRR17520069.3776262
SRR17520069.3776264
SRR17520069.3776266
SRR17520069.3776268
SRR17520069.3776270
SRR17520069.3776272
SRR17520069.3776274
SRR17520069.3776276
SRR17520069.3776278
SRR17520069.3776280

SRR17520069.3776282
SRR17520069.3776284
SRR17520069.3776286
SRR17520069.3776288
SRR17520069.3776290
SRR17520069.3776292
SRR17520069.3776294
SRR17520069.3776684
SRR17520069.3776686
SRR17520069.3776688
SRR17520069.3776690
SRR17520069.3776692
SRR17520069.3776694
SRR17520069.3776696
SRR17520069.3776698
SRR17520069.3776918
SRR17520069.3776920
SRR17520069.3776922
SRR17520069.3776924
SRR17520069.3776926
SRR17520069.3776930
SRR17520069.3776932
SRR17520069.3776934
SRR17520069.3776936
SRR17520069.3776938
SRR17520069.3776940
SRR17520069.3776942
SRR17520069.3776944
SRR17520069.3776946
SRR17520069.3776948
SRR17520069.3776950
SRR17520069.3776952
SRR17520069.3776954
SRR17520069.3776956
SRR17520069.3776958
SRR17520069.3776960
SRR17520069.3776962
SRR17520069.3776964
SRR17520069.3776966
SRR17520069.3776968
SRR17520069.3776970
SRR17520069.3790661
SRR17520069.3790663
SRR17520069.3790667
SRR17520069.3790669
SRR17520069.3790671
SRR17520069.3790673

SRR17520069.3790675
SRR17520069.3790677
SRR17520069.3790679
SRR17520069.3790681
SRR17520069.3790683
SRR17520069.3790685
SRR17520069.3790687
SRR17520069.3790689
SRR17520069.3790691
SRR17520069.3793884
SRR17520069.3795845
SRR17520069.3795847
SRR17520069.3795849
SRR17520069.3795853
SRR17520069.3802103
SRR17520069.3802105
SRR17520069.3802107
SRR17520069.3802109
SRR17520069.3802111
SRR17520069.3802113
SRR17520069.3802115
SRR17520069.3802117
SRR17520069.3802119
SRR17520069.3802121
SRR17520069.3802123
SRR17520069.3802125
SRR17520069.3802127
SRR17520069.3802129
SRR17520069.3802133
SRR17520069.3802135
SRR17520069.3802137
SRR17520069.3802139
SRR17520069.3802141
SRR17520069.3802143
SRR17520069.3802145
SRR17520069.3802147
SRR17520069.3802149
SRR17520069.3802151
SRR17520069.3802319
SRR17520069.3802321
SRR17520069.3802323
SRR17520069.3802325
SRR17520069.3802327
SRR17520069.3802329
SRR17520069.3802331
SRR17520069.3802333
SRR17520069.3802335

SRR17520069.3802337
SRR17520069.3802339
SRR17520069.3802341
SRR17520069.3802343
SRR17520069.3802345
SRR17520069.3802347
SRR17520069.3802349
SRR17520069.3802351
SRR17520069.3802353
SRR17520069.3802355
SRR17520069.3802357
SRR17520069.3802359
SRR17520069.3802361
SRR17520069.3803682
SRR17520069.3803684
SRR17520069.3803686
SRR17520069.3803688
SRR17520069.3803690
SRR17520069.3803692
SRR17520069.3803694
SRR17520069.3803696
SRR17520069.3803698
SRR17520069.3803700
SRR17520069.3803702
SRR17520069.3803704
SRR17520069.3803706
SRR17520069.3803708
SRR17520069.3803710
SRR17520069.3803712
SRR17520069.3803714
SRR17520069.3803716
SRR17520069.3803734
SRR17520069.3803736
SRR17520069.3803738
SRR17520069.3803740
SRR17520069.3803742
SRR17520069.3803744
SRR17520069.3803746
SRR17520069.3803748
SRR17520069.3803750
SRR17520069.3803752
SRR17520069.3803754
SRR17520069.3803756
SRR17520069.3803758
SRR17520069.3803760
SRR17520069.3803762
SRR17520069.3803764

SRR17520069.3803766
SRR17520069.3803768
SRR17520069.3803770
SRR17520069.3803772
SRR17520069.3803774
SRR17520069.3803776
SRR17520069.3803778
SRR17520069.3803780
SRR17520069.3803782
SRR17520069.3803784
SRR17520069.3803786
SRR17520069.3803788
SRR17520069.3803790
SRR17520069.3803792
SRR17520069.3803794
SRR17520069.3803796
SRR17520069.3803798
SRR17520069.3803800
SRR17520069.3803802
SRR17520069.3803804
SRR17520069.3803806
SRR17520069.3803808
SRR17520069.3803810
SRR17520069.3803812
SRR17520069.3803814
SRR17520069.3803818
SRR17520069.3803820
SRR17520069.3803824
SRR17520069.3803826
SRR17520069.3809382
SRR17520069.3809384
SRR17520069.3809386
SRR17520069.3809388
SRR17520069.3809390
SRR17520069.3809392
SRR17520069.3809394
SRR17520069.3809398
SRR17520069.3809400
SRR17520069.3809402
SRR17520069.3809406
SRR17520069.3809408
SRR17520069.3809410
SRR17520069.3809412
SRR17520069.3809414
SRR17520069.3809418
SRR17520069.3809420
SRR17520069.3809422

SRR17520069.3809424
SRR17520069.3809426
SRR17520069.3809428
SRR17520069.3809432
SRR17520069.3809434
SRR17520069.3809438
SRR17520069.3809440
SRR17520069.3809442
SRR17520069.3809444
SRR17520069.3809448
SRR17520069.3816267
SRR17520069.3816269
SRR17520069.3825966
SRR17520069.3825968
SRR17520069.3825970
SRR17520069.3825972
SRR17520069.3825976
SRR17520069.3825978
SRR17520069.3825980
SRR17520069.3825984
SRR17520069.3825986
SRR17520069.3825988
SRR17520069.3825990
SRR17520069.3825992
SRR17520069.3825994
SRR17520069.3825996
SRR17520069.3825998
SRR17520069.3826000
SRR17520069.3826002
SRR17520069.3826004
SRR17520069.3826006
SRR17520069.3826008
SRR17520069.3826012
SRR17520069.3826014
SRR17520069.3826016
SRR17520069.3826018
SRR17520069.3826020
SRR17520069.3826022
SRR17520069.3826024
SRR17520069.3826028
SRR17520069.3826030
SRR17520069.3826032
SRR17520069.3826034
SRR17520069.3826036
SRR17520069.3826038
SRR17520069.3826040
SRR17520069.3826042

SRR17520069.3826044
SRR17520069.3826046
SRR17520069.3826048
SRR17520069.3826050
SRR17520069.3826052
SRR17520069.3826054
SRR17520069.3826056
SRR17520069.3826058
SRR17520069.3826060
SRR17520069.3826064
SRR17520069.3826066
SRR17520069.3826068
SRR17520069.3826070
SRR17520069.3826072
SRR17520069.3826074
SRR17520069.3826078
SRR17520069.3826080
SRR17520069.3826082
SRR17520069.3826084
SRR17520069.3826088
SRR17520069.3826090
SRR17520069.3826092
SRR17520069.3826094
SRR17520069.3826096
SRR17520069.3826100
SRR17520069.3826102
SRR17520069.3826104
SRR17520069.3826108
SRR17520069.3826110
SRR17520069.3826112
SRR17520069.3826114
SRR17520069.3826116
SRR17520069.3826118
SRR17520069.3828076
SRR17520069.3828078
SRR17520069.3828080
SRR17520069.3828082
SRR17520069.3828084
SRR17520069.3828086
SRR17520069.3828088
SRR17520069.3828090
SRR17520069.3828092
SRR17520069.3828096
SRR17520069.3828098
SRR17520069.3828100
SRR17520069.3828102
SRR17520069.3828106

SRR17520069.3828108
SRR17520069.3828110
SRR17520069.3828112
SRR17520069.3828114
SRR17520069.3830413
SRR17520069.3830415
SRR17520069.3830657
SRR17520069.3830659
SRR17520069.3830661
SRR17520069.3830663
SRR17520069.3830665
SRR17520069.3830667
SRR17520069.3830669
SRR17520069.3830671
SRR17520069.3830673
SRR17520069.3830675
SRR17520069.3830677
SRR17520069.3830679
SRR17520069.3830681
SRR17520069.3830683
SRR17520069.3830685
SRR17520069.3836608
SRR17520069.3836610
SRR17520069.3836612
SRR17520069.3836614
SRR17520069.3836616
SRR17520069.3836618
SRR17520069.3836620
SRR17520069.3836622
SRR17520069.3836624
SRR17520069.3836626
SRR17520069.3836628
SRR17520069.3836630
SRR17520069.3836632
SRR17520069.3836634
SRR17520069.3839136
SRR17520069.3839138
SRR17520069.3839140
SRR17520069.3839142
SRR17520069.3839144
SRR17520069.3839148
SRR17520069.3839150
SRR17520069.3839152
SRR17520069.3839154
SRR17520069.3839156
SRR17520069.3839158
SRR17520069.3839160

SRR17520069.3839162
SRR17520069.3839164
SRR17520069.3839166
SRR17520069.3839168
SRR17520069.3839170
SRR17520069.3839172
SRR17520069.3839174
SRR17520069.3839176
SRR17520069.3839178
SRR17520069.3839180
SRR17520069.3839182
SRR17520069.3839184
SRR17520069.3839186
SRR17520069.3839188
SRR17520069.3839190
SRR17520069.3839192
SRR17520069.3839194
SRR17520069.3839196
SRR17520069.3839198
SRR17520069.3843013
SRR17520069.3843015
SRR17520069.3843017
SRR17520069.3843019
SRR17520069.3843021
SRR17520069.3843023
SRR17520069.3843025
SRR17520069.3843027
SRR17520069.3843029
SRR17520069.3843031
SRR17520069.3843033
SRR17520069.3843035
SRR17520069.3843037
SRR17520069.3843039
SRR17520069.3843041
SRR17520069.3843043
SRR17520069.3843045
SRR17520069.3843047
SRR17520069.3843049
SRR17520069.3843051
SRR17520069.3843053
SRR17520069.3843055
SRR17520069.3843057
SRR17520069.3843059
SRR17520069.3849554
SRR17520069.3849556
SRR17520069.3849558
SRR17520069.3849560

SRR17520069.3849562
SRR17520069.3849564
SRR17520069.3849566
SRR17520069.3849568
SRR17520069.3849570
SRR17520069.3849572
SRR17520069.3849574
SRR17520069.3849576
SRR17520069.3849578
SRR17520069.3849580
SRR17520069.3849582
SRR17520069.3849584
SRR17520069.3849586
SRR17520069.3849588
SRR17520069.3849590
SRR17520069.3849592
SRR17520069.3849594
SRR17520069.3849596
SRR17520069.3849598
SRR17520069.3849600
SRR17520069.3849602
SRR17520069.3849604
SRR17520069.3849606
SRR17520069.3849608
SRR17520069.3849610
SRR17520069.3849612
SRR17520069.3849614
SRR17520069.3858193
SRR17520069.3861278
SRR17520069.3861280
SRR17520069.3861282
SRR17520069.3861284
SRR17520069.3861286
SRR17520069.3861288
SRR17520069.3861290
SRR17520069.3861292
SRR17520069.3861294
SRR17520069.3861296
SRR17520069.3861298
SRR17520069.3861302
SRR17520069.3861304
SRR17520069.3861306
SRR17520069.3861308
SRR17520069.3861310
SRR17520069.3861312
SRR17520069.3861314
SRR17520069.3861316

SRR17520069.3861318
SRR17520069.3861320
SRR17520069.3861322
SRR17520069.3862575
SRR17520069.3862577
SRR17520069.3862579
SRR17520069.3862581
SRR17520069.3862583
SRR17520069.3862585
SRR17520069.3862587
SRR17520069.3863449
SRR17520069.3863451
SRR17520069.3863453
SRR17520069.3863457
SRR17520069.3863459
SRR17520069.3863461
SRR17520069.3863463
SRR17520069.3863465
SRR17520069.3863467
SRR17520069.3863469
SRR17520069.3863471
SRR17520069.3863473
SRR17520069.3863475
SRR17520069.3863477
SRR17520069.3863479
SRR17520069.3863481
SRR17520069.3863483
SRR17520069.3863485
SRR17520069.3863487
SRR17520069.3863489
SRR17520069.3863491
SRR17520069.3863493
SRR17520069.3863495
SRR17520069.3863497
SRR17520069.3863499
SRR17520069.3863501
SRR17520069.3863503
SRR17520069.3863505
SRR17520069.3863507
SRR17520069.3863509
SRR17520069.3863511
SRR17520069.3863513
SRR17520069.3863515
SRR17520069.3863517
SRR17520069.3863519
SRR17520069.3863521
SRR17520069.3864817

SRR17520069.3864819
SRR17520069.3864821
SRR17520069.3864823
SRR17520069.3864825
SRR17520069.3864827
SRR17520069.3864829
SRR17520069.3864831
SRR17520069.3864833
SRR17520069.3864835
SRR17520069.3865268
SRR17520069.3865270
SRR17520069.3865272
SRR17520069.3865274
SRR17520069.3865276
SRR17520069.3865278
SRR17520069.3865280
SRR17520069.3873821
SRR17520069.3877131
SRR17520069.3877133
SRR17520069.3877135
SRR17520069.3877137
SRR17520069.3877139
SRR17520069.3877141
SRR17520069.3877143
SRR17520069.3877145
SRR17520069.3877147
SRR17520069.3877149
SRR17520069.3877151
SRR17520069.3881548
SRR17520069.3881550
SRR17520069.3881552
SRR17520069.3881554
SRR17520069.3881556
SRR17520069.3884386
SRR17520069.3884388
SRR17520069.3884390
SRR17520069.3884392
SRR17520069.3884394
SRR17520069.3884396
SRR17520069.3884398
SRR17520069.3884400
SRR17520069.3884402
SRR17520069.3884404
SRR17520069.3884406
SRR17520069.3884408
SRR17520069.3887065
SRR17520069.3887067

SRR17520069.3887069
SRR17520069.3888308
SRR17520069.3891837
SRR17520069.3891839
SRR17520069.3891841
SRR17520069.3891843
SRR17520069.3891845
SRR17520069.3891847
SRR17520069.3891849
SRR17520069.3891851
SRR17520069.3891855
SRR17520069.3891857
SRR17520069.3891859
SRR17520069.3891861
SRR17520069.3891863
SRR17520069.3891865
SRR17520069.3891867
SRR17520069.3891869
SRR17520069.3891871
SRR17520069.3891873
SRR17520069.3891875
SRR17520069.3891877
SRR17520069.3891879
SRR17520069.3896496
SRR17520069.3896498
SRR17520069.3902608
SRR17520069.3902610
SRR17520069.3902612
SRR17520069.3902614
SRR17520069.3902616
SRR17520069.3902618
SRR17520069.3902620
SRR17520069.3902622
SRR17520069.3902624
SRR17520069.3902626
SRR17520069.3902628
SRR17520069.3902630
SRR17520069.3902632
SRR17520069.3902634
SRR17520069.3902638
SRR17520069.3902640
SRR17520069.3902642
SRR17520069.3902644
SRR17520069.3902646
SRR17520069.3902648
SRR17520069.3902650
SRR17520069.3902652

SRR17520069.3902654
SRR17520069.3902656
SRR17520069.3902658
SRR17520069.3902660
SRR17520069.3903088
SRR17520069.3903090
SRR17520069.3903092
SRR17520069.3903094
SRR17520069.3903096
SRR17520069.3903098
SRR17520069.3903100
SRR17520069.3903102
SRR17520069.3903104
SRR17520069.3903106
SRR17520069.3903108
SRR17520069.3903110
SRR17520069.3903112
SRR17520069.3903114
SRR17520069.3903116
SRR17520069.3903118
SRR17520069.3903120
SRR17520069.3903122
SRR17520069.3904400
SRR17520069.3904402
SRR17520069.3904404
SRR17520069.3907460
SRR17520069.3907462
SRR17520069.3907464
SRR17520069.3907466
SRR17520069.3907468
SRR17520069.3908529
SRR17520069.3908531
SRR17520069.3908535
SRR17520069.3908537
SRR17520069.3908539
SRR17520069.3908541
SRR17520069.3908543
SRR17520069.3908545
SRR17520069.3908547
SRR17520069.3908549
SRR17520069.3908551
SRR17520069.3908553
SRR17520069.3908555
SRR17520069.3908557
SRR17520069.3908559
SRR17520069.3908561
SRR17520069.3908563

SRR17520069.3911381
SRR17520069.3911383
SRR17520069.3911385
SRR17520069.3911387
SRR17520069.3911389
SRR17520069.3911391
SRR17520069.3911395
SRR17520069.3911397
SRR17520069.3911399
SRR17520069.3911401
SRR17520069.3911403
SRR17520069.3911405
SRR17520069.3911407
SRR17520069.3911409
SRR17520069.3911411
SRR17520069.3911413
SRR17520069.3911415
SRR17520069.3911417
SRR17520069.3911419
SRR17520069.3911421
SRR17520069.3911423
SRR17520069.3913872
SRR17520069.3913874
SRR17520069.3913876
SRR17520069.3913878
SRR17520069.3913880
SRR17520069.3913882
SRR17520069.3913884
SRR17520069.3913886
SRR17520069.3913888
SRR17520069.3913890
SRR17520069.3913892
SRR17520069.3913894
SRR17520069.3913898
SRR17520069.3913900
SRR17520069.3913902
SRR17520069.3913904
SRR17520069.3913906
SRR17520069.3913908
SRR17520069.3913910
SRR17520069.3913912
SRR17520069.3913914
SRR17520069.3913916
SRR17520069.3913918
SRR17520069.3913920
SRR17520069.3913922
SRR17520069.3913924

SRR17520069.3913926
SRR17520069.3913928
SRR17520069.3913930
SRR17520069.3913932
SRR17520069.3913934
SRR17520069.3913936
SRR17520069.3913938
SRR17520069.3913940
SRR17520069.3913942
SRR17520069.3913944
SRR17520069.3913946
SRR17520069.3913948
SRR17520069.3914530
SRR17520069.3914532
SRR17520069.3914534
SRR17520069.3914536
SRR17520069.3914538
SRR17520069.3914540
SRR17520069.3914542
SRR17520069.3914544
SRR17520069.3914546
SRR17520069.3914548
SRR17520069.3914550
SRR17520069.3914552
SRR17520069.3914554
SRR17520069.3914556
SRR17520069.3914558
SRR17520069.3918979
SRR17520069.3918981
SRR17520069.3918985
SRR17520069.3918987
SRR17520069.3918989
SRR17520069.3918991
SRR17520069.3918993
SRR17520069.3918995
SRR17520069.3918997
SRR17520069.3918999
SRR17520069.3919001
SRR17520069.3919003
SRR17520069.3919005
SRR17520069.3919007
SRR17520069.3919009
SRR17520069.3919011
SRR17520069.3919013
SRR17520069.3924718
SRR17520069.3924720
SRR17520069.3924722

SRR17520069.3924724
SRR17520069.3924726
SRR17520069.3924728
SRR17520069.3924730
SRR17520069.3924732
SRR17520069.3924734
SRR17520069.3924736
SRR17520069.3924738
SRR17520069.3924740
SRR17520069.3924742
SRR17520069.3924744
SRR17520069.3924748
SRR17520069.3924750
SRR17520069.3924752
SRR17520069.3924754
SRR17520069.3924756
SRR17520069.3924758
SRR17520069.3924760
SRR17520069.3924762
SRR17520069.3924764
SRR17520069.3924766
SRR17520069.3924768
SRR17520069.3924770
SRR17520069.3924772
SRR17520069.3924774
SRR17520069.3924776
SRR17520069.3924778
SRR17520069.3924780
SRR17520069.3924782
SRR17520069.3924784
SRR17520069.3924786
SRR17520069.3924788
SRR17520069.3924790
SRR17520069.3924792
SRR17520069.3924794
SRR17520069.3924796
SRR17520069.3924798
SRR17520069.3924802
SRR17520069.3924804
SRR17520069.3924806
SRR17520069.3924808
SRR17520069.3924810
SRR17520069.3924816
SRR17520069.3924818
SRR17520069.3924820
SRR17520069.3924822
SRR17520069.3924824

SRR17520069.3930273
SRR17520069.3930275
SRR17520069.3930277
SRR17520069.3930279
SRR17520069.3930281
SRR17520069.3930283
SRR17520069.3930285
SRR17520069.3930287
SRR17520069.3930289
SRR17520069.3930291
SRR17520069.3930293
SRR17520069.3930295
SRR17520069.3930297
SRR17520069.3930299
SRR17520069.3930303
SRR17520069.3930305
SRR17520069.3930307
SRR17520069.3930309
SRR17520069.3930311
SRR17520069.3930313
SRR17520069.3930315
SRR17520069.3930317
SRR17520069.3930319
SRR17520069.3930321
SRR17520069.3930323
SRR17520069.3930325
SRR17520069.3930327
SRR17520069.3930329
SRR17520069.3930331
SRR17520069.3930333
SRR17520069.3942422
SRR17520069.3942424
SRR17520069.3942426
SRR17520069.3942428
SRR17520069.3942430
SRR17520069.3946733
SRR17520069.3946735
SRR17520069.3946737
SRR17520069.3946739
SRR17520069.3949834
SRR17520069.3949836
SRR17520069.3949838
SRR17520069.3949840
SRR17520069.3949842
SRR17520069.3949844
SRR17520069.3949846
SRR17520069.3949848

SRR17520069.3949850
SRR17520069.3953033
SRR17520069.3953035
SRR17520069.3953037
SRR17520069.3953039
SRR17520069.3953041
SRR17520069.3953043
SRR17520069.3953045
SRR17520069.3953047
SRR17520069.3953049
SRR17520069.3953051
SRR17520069.3953053
SRR17520069.3953055
SRR17520069.3953057
SRR17520069.3953059
SRR17520069.3953061
SRR17520069.3954774
SRR17520069.3954776
SRR17520069.3954780
SRR17520069.3954782
SRR17520069.3954784
SRR17520069.3954788
SRR17520069.3954790
SRR17520069.3954794
SRR17520069.3954798
SRR17520069.3954800
SRR17520069.3954802
SRR17520069.3954804
SRR17520069.3954806
SRR17520069.3954810
SRR17520069.3954812
SRR17520069.3954814
SRR17520069.3954816
SRR17520069.3954818
SRR17520069.3954820
SRR17520069.3954822
SRR17520069.3954824
SRR17520069.3954826
SRR17520069.3954828
SRR17520069.3954832
SRR17520069.3954834
SRR17520069.3954836
SRR17520069.3954838
SRR17520069.3954840
SRR17520069.3954842
SRR17520069.3954844
SRR17520069.3954850

SRR17520069.3954852
SRR17520069.3954854
SRR17520069.3954856
SRR17520069.3954858
SRR17520069.3960570
SRR17520069.3960572
SRR17520069.3960574
SRR17520069.3960576
SRR17520069.3960580
SRR17520069.3960582
SRR17520069.3960584
SRR17520069.3960590
SRR17520069.3960592
SRR17520069.3960594
SRR17520069.3960596
SRR17520069.3960598
SRR17520069.3960600
SRR17520069.3960604
SRR17520069.3960606
SRR17520069.3960608
SRR17520069.3960614
SRR17520069.3960618
SRR17520069.3960620
SRR17520069.3960622
SRR17520069.3960624
SRR17520069.3960626
SRR17520069.3960628
SRR17520069.3960630
SRR17520069.3962077
SRR17520069.3962079
SRR17520069.3962081
SRR17520069.3962083
SRR17520069.3962085
SRR17520069.3962087
SRR17520069.3962089
SRR17520069.3962091
SRR17520069.3962093
SRR17520069.3962095
SRR17520069.3962097
SRR17520069.3962099
SRR17520069.3962101
SRR17520069.3962103
SRR17520069.3962105
SRR17520069.3962107
SRR17520069.3962109
SRR17520069.3962111
SRR17520069.3962113

SRR17520069.3962115
SRR17520069.3962117
SRR17520069.3962119
SRR17520069.3962121
SRR17520069.3962123
SRR17520069.3962125
SRR17520069.3962127
SRR17520069.3962129
SRR17520069.3962131
SRR17520069.3962133
SRR17520069.3962135
SRR17520069.3962137
SRR17520069.3962139
SRR17520069.3962141
SRR17520069.3962143
SRR17520069.3962145
SRR17520069.3962147
SRR17520069.3962149
SRR17520069.3962151
SRR17520069.3962153
SRR17520069.3962155
SRR17520069.3962157
SRR17520069.3962159
SRR17520069.3962161
SRR17520069.3962163
SRR17520069.3962165
SRR17520069.3962167
SRR17520069.3962169
SRR17520069.3962171
SRR17520069.3962173
SRR17520069.3962175
SRR17520069.3962177
SRR17520069.3962179
SRR17520069.3962181
SRR17520069.3962183
SRR17520069.3966527
SRR17520069.3966529
SRR17520069.3966531
SRR17520069.3966533
SRR17520069.3966535
SRR17520069.3966537
SRR17520069.3966539
SRR17520069.3967024
SRR17520069.3968287
SRR17520069.3968289
SRR17520069.3968291
SRR17520069.3968293

SRR17520069.3968295
SRR17520069.3968297
SRR17520069.3968299
SRR17520069.3968301
SRR17520069.3968303
SRR17520069.3968305
SRR17520069.3968307
SRR17520069.3968310
SRR17520069.3968312
SRR17520069.3968314
SRR17520069.3971240
SRR17520069.3971242
SRR17520069.3973165
SRR17520069.3973167
SRR17520069.3973169
SRR17520069.3973171
SRR17520069.3973173
SRR17520069.3973175
SRR17520069.3973177
SRR17520069.3973179
SRR17520069.3973181
SRR17520069.3973183
SRR17520069.3973185
SRR17520069.3973187
SRR17520069.3973189
SRR17520069.3973191
SRR17520069.3973193
SRR17520069.3973195
SRR17520069.3973197
SRR17520069.3973199
SRR17520069.3973201
SRR17520069.3973203
SRR17520069.3973205
SRR17520069.3973207
SRR17520069.3973209
SRR17520069.3973211
SRR17520069.3973213
SRR17520069.3973215
SRR17520069.3973217
SRR17520069.3975042
SRR17520069.3975044
SRR17520069.3981274
SRR17520069.3981276
SRR17520069.3981278
SRR17520069.3981280
SRR17520069.3981282
SRR17520069.3981284

SRR17520069.3984359
SRR17520069.3984361
SRR17520069.3984363
SRR17520069.3984365
SRR17520069.3984367
SRR17520069.3984369
SRR17520069.3984371
SRR17520069.3987302
SRR17520069.3987308
SRR17520069.3992998
SRR17520069.3993000
SRR17520069.3993002
SRR17520069.3993004
SRR17520069.3993006
SRR17520069.3993008
SRR17520069.3993010
SRR17520069.3993012
SRR17520069.3993014
SRR17520069.3993016
SRR17520069.3993018
SRR17520069.3996179
SRR17520069.3996183
SRR17520069.3999780
SRR17520069.3999784
SRR17520069.3999786
SRR17520069.4001607
SRR17520069.4001618
SRR17520069.4004010
SRR17520069.4004012
SRR17520069.4004016
SRR17520069.4004018
SRR17520069.4004020
SRR17520069.4004022
SRR17520069.4004024
SRR17520069.4004026
SRR17520069.4004028
SRR17520069.4004030
SRR17520069.4004032
SRR17520069.4004034
SRR17520069.4004038
SRR17520069.4004342
SRR17520069.4004344
SRR17520069.4004346
SRR17520069.4019662
SRR17520069.4019664
SRR17520069.4019666
SRR17520069.4019668

SRR17520069.4019670
SRR17520069.4019672
SRR17520069.4021063
SRR17520069.4021065
SRR17520069.4021067
SRR17520069.4021069
SRR17520069.4021071
SRR17520069.4021073
SRR17520069.4021075
SRR17520069.4021077
SRR17520069.4021079
SRR17520069.4021081
SRR17520069.4021083
SRR17520069.4021085
SRR17520069.4021087
SRR17520069.4021089
SRR17520069.4021091
SRR17520069.4021093
SRR17520069.4021095
SRR17520069.4021097
SRR17520069.4021099
SRR17520069.4021101
SRR17520069.4021103
SRR17520069.4021105
SRR17520069.4021107
SRR17520069.4021109
SRR17520069.4021111
SRR17520069.4021113
SRR17520069.4021115
SRR17520069.4021117
SRR17520069.4021119
SRR17520069.4021121
SRR17520069.4021123
SRR17520069.4021125
SRR17520069.4021127
SRR17520069.4021129
SRR17520069.4021131
SRR17520069.4021133
SRR17520069.4021135
SRR17520069.4021137
SRR17520069.4027790
SRR17520069.4027792
SRR17520069.4028670
SRR17520069.4032397
SRR17520069.4032399
SRR17520069.4032401
SRR17520069.4033120

SRR17520069.4039260
SRR17520069.4039262
SRR17520069.4039264
SRR17520069.4039266
SRR17520069.4039268
SRR17520069.4039270
SRR17520069.4039272
SRR17520069.4039274
SRR17520069.4039276
SRR17520069.4039278
SRR17520069.4039280
SRR17520069.4039282
SRR17520069.4039284
SRR17520069.4039286
SRR17520069.4039288
SRR17520069.4039290
SRR17520069.4039292
SRR17520069.4039294
SRR17520069.4039296
SRR17520069.4039298
SRR17520069.4039300
SRR17520069.4039302
SRR17520069.4039304
SRR17520069.4039306
SRR17520069.4039308
SRR17520069.4039310
SRR17520069.4039312
SRR17520069.4039314
SRR17520069.4039316
SRR17520069.4039320
SRR17520069.4039322
SRR17520069.4039324
SRR17520069.4039326
SRR17520069.4039328
SRR17520069.4039330
SRR17520069.4039332
SRR17520069.4039334
SRR17520069.4039336
SRR17520069.4039338
SRR17520069.4039340
SRR17520069.4039342
SRR17520069.4039344
SRR17520069.4039346
SRR17520069.4039348
SRR17520069.4039350
SRR17520069.4039352
SRR17520069.4039354

SRR17520069.4039356
SRR17520069.4039358
SRR17520069.4043585
SRR17520069.4049572
SRR17520069.4049574
SRR17520069.4049576
SRR17520069.4049578
SRR17520069.4049580
SRR17520069.4049582
SRR17520069.4049586
SRR17520069.4049588
SRR17520069.4049590
SRR17520069.4049592
SRR17520069.4049594
SRR17520069.4049598
SRR17520069.4049600
SRR17520069.4049602
SRR17520069.4049604
SRR17520069.4049606
SRR17520069.4049608
SRR17520069.4049610
SRR17520069.4049612
SRR17520069.4049614
SRR17520069.4049616
SRR17520069.4049618
SRR17520069.4049620
SRR17520069.4049622
SRR17520069.4049624
SRR17520069.4049626
SRR17520069.4049628
SRR17520069.4049630
SRR17520069.4049632
SRR17520069.4049634
SRR17520069.4049636
SRR17520069.4049638
SRR17520069.4049640
SRR17520069.4049642
SRR17520069.4049646
SRR17520069.4049648
SRR17520069.4049650
SRR17520069.4049652
SRR17520069.4049654
SRR17520069.4049656
SRR17520069.4049658
SRR17520069.4049660
SRR17520069.4049662
SRR17520069.4049664

SRR17520069.4049666
SRR17520069.4049668
SRR17520069.4049670
SRR17520069.4049672
SRR17520069.4049674
SRR17520069.4049676
SRR17520069.4049783
SRR17520069.4049785
SRR17520069.4049787
SRR17520069.4049789
SRR17520069.4049791
SRR17520069.4049793
SRR17520069.4049795
SRR17520069.4049797
SRR17520069.4049799
SRR17520069.4049801
SRR17520069.4049803
SRR17520069.4049805
SRR17520069.4049807
SRR17520069.4049809
SRR17520069.4049811
SRR17520069.4049813
SRR17520069.4049815
SRR17520069.4049817
SRR17520069.4049819
SRR17520069.4049821
SRR17520069.4049823
SRR17520069.4049825
SRR17520069.4049827
SRR17520069.4049829
SRR17520069.4049831
SRR17520069.4049833
SRR17520069.4049835
SRR17520069.4049837
SRR17520069.4049839
SRR17520069.4049841
SRR17520069.4049843
SRR17520069.4049845
SRR17520069.4049847
SRR17520069.4049849
SRR17520069.4049851
SRR17520069.4049853
SRR17520069.4049855
SRR17520069.4049857
SRR17520069.4049859
SRR17520069.4049861
SRR17520069.4049863

SRR17520069.4049865
SRR17520069.4049867
SRR17520069.4049871
SRR17520069.4049873
SRR17520069.4049875
SRR17520069.4049877
SRR17520069.4049879
SRR17520069.4049881
SRR17520069.4049883
SRR17520069.4049887
SRR17520069.4049889
SRR17520069.4049891
SRR17520069.4049895
SRR17520069.4049897
SRR17520069.4049899
SRR17520069.4049901
SRR17520069.4049905
SRR17520069.4049907
SRR17520069.4049909
SRR17520069.4049911
SRR17520069.4049913
SRR17520069.4051104
SRR17520069.4051106
SRR17520069.4051108
SRR17520069.4051110
SRR17520069.4051112
SRR17520069.4051114
SRR17520069.4051118
SRR17520069.4051120
SRR17520069.4051122
SRR17520069.4051124
SRR17520069.4051126
SRR17520069.4051128
SRR17520069.4051130
SRR17520069.4051134
SRR17520069.4053268
SRR17520069.4053270
SRR17520069.4053272
SRR17520069.4053274
SRR17520069.4053276
SRR17520069.4053278
SRR17520069.4053280
SRR17520069.4053282
SRR17520069.4053288
SRR17520069.4053290
SRR17520069.4053292
SRR17520069.4053294

SRR17520069.4053296
SRR17520069.4053298
SRR17520069.4053300
SRR17520069.4053520
SRR17520069.4053522
SRR17520069.4053524
SRR17520069.4053526
SRR17520069.4053528
SRR17520069.4055528
SRR17520069.4055530
SRR17520069.4055532
SRR17520069.4055534
SRR17520069.4055536
SRR17520069.4055538
SRR17520069.4055540
SRR17520069.4055542
SRR17520069.4055544
SRR17520069.4055546
SRR17520069.4055548
SRR17520069.4055550
SRR17520069.4063072
SRR17520069.4063074
SRR17520069.4063076
SRR17520069.4063078
SRR17520069.4063082
SRR17520069.4063084
SRR17520069.4063086
SRR17520069.4065724
SRR17520069.4065726
SRR17520069.4065728
SRR17520069.4065730
SRR17520069.4065732
SRR17520069.4065734
SRR17520069.4065736
SRR17520069.4065738
SRR17520069.4065740
SRR17520069.4065742
SRR17520069.4065744
SRR17520069.4069851
SRR17520069.4069853
SRR17520069.4069855
SRR17520069.4069857
SRR17520069.4069885
SRR17520069.4069887
SRR17520069.4069889
SRR17520069.4069891
SRR17520069.4072205

SRR17520069.4091959
SRR17520069.4091961
SRR17520069.4091963
SRR17520069.4091965
SRR17520069.4091967
SRR17520069.4091969
SRR17520069.4091971
SRR17520069.4091973
SRR17520069.4091975
SRR17520069.4091977
SRR17520069.4091979
SRR17520069.4091981
SRR17520069.4091983
SRR17520069.4091985
SRR17520069.4091987
SRR17520069.4091989
SRR17520069.4091991
SRR17520069.4091993
SRR17520069.4091995
SRR17520069.4091997
SRR17520069.4091999
SRR17520069.4092001
SRR17520069.4092003
SRR17520069.4092005
SRR17520069.4092007
SRR17520069.4092009
SRR17520069.4092011
SRR17520069.4092013
SRR17520069.4092015
SRR17520069.4092017
SRR17520069.4092019
SRR17520069.4092021
SRR17520069.4092023
SRR17520069.4092025
SRR17520069.4092027
SRR17520069.4092029
SRR17520069.4092031
SRR17520069.4092033
SRR17520069.4092035
SRR17520069.4092037
SRR17520069.4092039
SRR17520069.4092041
SRR17520069.4092043
SRR17520069.4092045
SRR17520069.4092047
SRR17520069.4092049
SRR17520069.4092051

SRR17520069.4092053
SRR17520069.4092055
SRR17520069.4092057
SRR17520069.4092059
SRR17520069.4092061
SRR17520069.4092063
SRR17520069.4092065
SRR17520069.4092067
SRR17520069.4092069
SRR17520069.4092071
SRR17520069.4092073
SRR17520069.4096184
SRR17520069.4096186
SRR17520069.4096188
SRR17520069.4096190
SRR17520069.4096192
SRR17520069.4096194
SRR17520069.4096196
SRR17520069.4096198
SRR17520069.4096200
SRR17520069.4096202
SRR17520069.4096204
SRR17520069.4096206
SRR17520069.4096208
SRR17520069.4096210
SRR17520069.4096212
SRR17520069.4096214
SRR17520069.4096216
SRR17520069.4096218
SRR17520069.4096220
SRR17520069.4096222
SRR17520069.4096224
SRR17520069.4096226
SRR17520069.4096230
SRR17520069.4098127
SRR17520069.4098129
SRR17520069.4098131
SRR17520069.4098133
SRR17520069.4098135
SRR17520069.4098137
SRR17520069.4098139
SRR17520069.4098141
SRR17520069.4098143
SRR17520069.4098145
SRR17520069.4098147
SRR17520069.4098149
SRR17520069.4098151

SRR17520069.4098155
SRR17520069.4098663
SRR17520069.4098665
SRR17520069.4098667
SRR17520069.4098669
SRR17520069.4098671
SRR17520069.4098673
SRR17520069.4098675
SRR17520069.4098677
SRR17520069.4098679
SRR17520069.4098681
SRR17520069.4103216
SRR17520069.4103218
SRR17520069.4103220
SRR17520069.4103222
SRR17520069.4103224
SRR17520069.4103226
SRR17520069.4103228
SRR17520069.4103230
SRR17520069.4103232
SRR17520069.4103234
SRR17520069.4103236
SRR17520069.4103238
SRR17520069.4103240
SRR17520069.4103242
SRR17520069.4103244
SRR17520069.4103246
SRR17520069.4103248
SRR17520069.4103250
SRR17520069.4103252
SRR17520069.4103254
SRR17520069.4103256
SRR17520069.4103258
SRR17520069.4103260
SRR17520069.4103262
SRR17520069.4103264
SRR17520069.4103266
SRR17520069.4103268
SRR17520069.4103270
SRR17520069.4103272
SRR17520069.4103274
SRR17520069.4103276
SRR17520069.4103280
SRR17520069.4103282
SRR17520069.4103284
SRR17520069.4103286
SRR17520069.4103288

SRR17520069.4103290
SRR17520069.4103292
SRR17520069.4103294
SRR17520069.4103296
SRR17520069.4103298
SRR17520069.4103300
SRR17520069.4103302
SRR17520069.4103304
SRR17520069.4103306
SRR17520069.4103308
SRR17520069.4103310
SRR17520069.4103312
SRR17520069.4103314
SRR17520069.4103316
SRR17520069.4103318
SRR17520069.4103320
SRR17520069.4103322
SRR17520069.4103324
SRR17520069.4103328
SRR17520069.4103330
SRR17520069.4103332
SRR17520069.4103334
SRR17520069.4103336
SRR17520069.4103338
SRR17520069.4103345
SRR17520069.4105951
SRR17520069.4105953
SRR17520069.4105955
SRR17520069.4105959
SRR17520069.4105961
SRR17520069.4105963
SRR17520069.4105965
SRR17520069.4105967
SRR17520069.4105969
SRR17520069.4105971
SRR17520069.4105973
SRR17520069.4105975
SRR17520069.4105977
SRR17520069.4105979
SRR17520069.4105981
SRR17520069.4105983
SRR17520069.4105985
SRR17520069.4105987
SRR17520069.4105989
SRR17520069.4105991
SRR17520069.4105993
SRR17520069.4105995

SRR17520069.4105997
SRR17520069.4105999
SRR17520069.4106001
SRR17520069.4106003
SRR17520069.4106005
SRR17520069.4106007
SRR17520069.4106009
SRR17520069.4106013
SRR17520069.4106015
SRR17520069.4106017
SRR17520069.4106019
SRR17520069.4106021
SRR17520069.4106023
SRR17520069.4106025
SRR17520069.4106027
SRR17520069.4106031
SRR17520069.4106033
SRR17520069.4107636
SRR17520069.4108319
SRR17520069.4108321
SRR17520069.4108323
SRR17520069.4108325
SRR17520069.4108327
SRR17520069.4108333
SRR17520069.4108335
SRR17520069.4110524
SRR17520069.4111692
SRR17520069.4111694
SRR17520069.4111698
SRR17520069.4111700
SRR17520069.4111704
SRR17520069.4111706
SRR17520069.4111708
SRR17520069.4111716
SRR17520069.4111718
SRR17520069.4111720
SRR17520069.4111732
SRR17520069.4111734
SRR17520069.4111740
SRR17520069.4111742
SRR17520069.4111748
SRR17520069.4111752
SRR17520069.4111756
SRR17520069.4112489
SRR17520069.4112491
SRR17520069.4112493
SRR17520069.4113184

SRR17520069.4113186
SRR17520069.4113188
SRR17520069.4113190
SRR17520069.4114809
SRR17520069.4114811
SRR17520069.4114813
SRR17520069.4114815
SRR17520069.4114817
SRR17520069.4114819
SRR17520069.4114821
SRR17520069.4114827
SRR17520069.4120018
SRR17520069.4120020
SRR17520069.4120022
SRR17520069.4120029
SRR17520069.4120031
SRR17520069.4120033
SRR17520069.4128533
SRR17520069.4128535
SRR17520069.4128537
SRR17520069.4128539
SRR17520069.4128541
SRR17520069.4128543
SRR17520069.4128547
SRR17520069.4128549
SRR17520069.4128551
SRR17520069.4128553
SRR17520069.4128555
SRR17520069.4128557
SRR17520069.4128559
SRR17520069.4128561
SRR17520069.4128563
SRR17520069.4128565
SRR17520069.4128567
SRR17520069.4128569
SRR17520069.4128571
SRR17520069.4138501
SRR17520069.4138503
SRR17520069.4138505
SRR17520069.4138507
SRR17520069.4138509
SRR17520069.4138511
SRR17520069.4138513
SRR17520069.4138515
SRR17520069.4144726
SRR17520069.4144728
SRR17520069.4144730

SRR17520069.4144732
SRR17520069.4144734
SRR17520069.4144736
SRR17520069.4151388
SRR17520069.4151390
SRR17520069.4151392
SRR17520069.4151394
SRR17520069.4151396
SRR17520069.4151398
SRR17520069.4151400
SRR17520069.4151402
SRR17520069.4151404
SRR17520069.4155076
SRR17520069.4155078
SRR17520069.4155080
SRR17520069.4155082
SRR17520069.4155084
SRR17520069.4155088
SRR17520069.4155090
SRR17520069.4155092
SRR17520069.4155094
SRR17520069.4155096
SRR17520069.4155098
SRR17520069.4155100
SRR17520069.4155102
SRR17520069.4155104
SRR17520069.4155106
SRR17520069.4155108
SRR17520069.4155110
SRR17520069.4155112
SRR17520069.4155114
SRR17520069.4155116
SRR17520069.4155118
SRR17520069.4155120
SRR17520069.4155122
SRR17520069.4157357
SRR17520069.4157359
SRR17520069.4157361
SRR17520069.4157363
SRR17520069.4157365
SRR17520069.4157367
SRR17520069.4157369
SRR17520069.4157371
SRR17520069.4157373
SRR17520069.4157375
SRR17520069.4157377
SRR17520069.4158208

SRR17520069.4158210
SRR17520069.4158212
SRR17520069.4158218
SRR17520069.4158220
SRR17520069.4158222
SRR17520069.4158224
SRR17520069.4158226
SRR17520069.4158228
SRR17520069.4158230
SRR17520069.4158232
SRR17520069.4158234
SRR17520069.4158236
SRR17520069.4158238
SRR17520069.4158240
SRR17520069.4158242
SRR17520069.4158244
SRR17520069.4158246
SRR17520069.4158248
SRR17520069.4158250
SRR17520069.4158252
SRR17520069.4158254
SRR17520069.4158256
SRR17520069.4158258
SRR17520069.4158260
SRR17520069.4158262
SRR17520069.4158264
SRR17520069.4158268
SRR17520069.4158270
SRR17520069.4158272
SRR17520069.4158274
SRR17520069.4158276
SRR17520069.4158278
SRR17520069.4158280
SRR17520069.4158282
SRR17520069.4158284
SRR17520069.4158286
SRR17520069.4158288
SRR17520069.4158290
SRR17520069.4158292
SRR17520069.4158294
SRR17520069.4158296
SRR17520069.4158298
SRR17520069.4158300
SRR17520069.4158302
SRR17520069.4158304
SRR17520069.4158306
SRR17520069.4158308

SRR17520069.4158310
SRR17520069.4158312
SRR17520069.4158314
SRR17520069.4158316
SRR17520069.4158318
SRR17520069.4158320
SRR17520069.4160319
SRR17520069.4160321
SRR17520069.4160323
SRR17520069.4160325
SRR17520069.4160327
SRR17520069.4162533
SRR17520069.4162535
SRR17520069.4164824
SRR17520069.4164826
SRR17520069.4164828
SRR17520069.4164830
SRR17520069.4164832
SRR17520069.4164834
SRR17520069.4164836
SRR17520069.4164838
SRR17520069.4164840
SRR17520069.4164842
SRR17520069.4164844
SRR17520069.4164846
SRR17520069.4164848
SRR17520069.4164850
SRR17520069.4164852
SRR17520069.4164854
SRR17520069.4164856
SRR17520069.4164858
SRR17520069.4164860
SRR17520069.4164862
SRR17520069.4164864
SRR17520069.4164866
SRR17520069.4164868
SRR17520069.4164870
SRR17520069.4164872
SRR17520069.4164874
SRR17520069.4164876
SRR17520069.4164878
SRR17520069.4164880
SRR17520069.4164882
SRR17520069.4164886
SRR17520069.4164888
SRR17520069.4164890
SRR17520069.4164892

SRR17520069.4164894
SRR17520069.4164896
SRR17520069.4164898
SRR17520069.4170344
SRR17520069.4170346
SRR17520069.4170348
SRR17520069.4170350
SRR17520069.4170352
SRR17520069.4170354
SRR17520069.4170356
SRR17520069.4170358
SRR17520069.4170362
SRR17520069.4170364
SRR17520069.4170366
SRR17520069.4170368
SRR17520069.4170370
SRR17520069.4170372
SRR17520069.4170374
SRR17520069.4170376
SRR17520069.4170378
SRR17520069.4170380
SRR17520069.4170384
SRR17520069.4170386
SRR17520069.4170388
SRR17520069.4170390
SRR17520069.4170392
SRR17520069.4170394
SRR17520069.4170396
SRR17520069.4170398
SRR17520069.4170400
SRR17520069.4170402
SRR17520069.4170404
SRR17520069.4170406
SRR17520069.4173064
SRR17520069.4173066
SRR17520069.4173068
SRR17520069.4173070
SRR17520069.4173072
SRR17520069.4173074
SRR17520069.4173076
SRR17520069.4173078
SRR17520069.4173080
SRR17520069.4173082
SRR17520069.4173084
SRR17520069.4173086
SRR17520069.4173088
SRR17520069.4173090

SRR17520069.4173092
SRR17520069.4173094
SRR17520069.4173096
SRR17520069.4173098
SRR17520069.4173100
SRR17520069.4173102
SRR17520069.4173104
SRR17520069.4173106
SRR17520069.4173108
SRR17520069.4173110
SRR17520069.4173112
SRR17520069.4173114
SRR17520069.4173116
SRR17520069.4173118
SRR17520069.4178804
SRR17520069.4178806
SRR17520069.4178808
SRR17520069.4178810
SRR17520069.4178812
SRR17520069.4178814
SRR17520069.4183816
SRR17520069.4183818
SRR17520069.4183820
SRR17520069.4183822
SRR17520069.4183824
SRR17520069.4183826
SRR17520069.4183828
SRR17520069.4189748
SRR17520069.4189750
SRR17520069.4189752
SRR17520069.4189754
SRR17520069.4189980
SRR17520069.4189982
SRR17520069.4189986
SRR17520069.4189988
SRR17520069.4189994
SRR17520069.4189996
SRR17520069.4190000
SRR17520069.4190002
SRR17520069.4190004
SRR17520069.4190006
SRR17520069.4190299
SRR17520069.4190301
SRR17520069.4190303
SRR17520069.4190305
SRR17520069.4190307
SRR17520069.4190309

SRR17520069.4190311
SRR17520069.4190313
SRR17520069.4190315
SRR17520069.4190317
SRR17520069.4190319
SRR17520069.4190321
SRR17520069.4190323
SRR17520069.4190325
SRR17520069.4190327
SRR17520069.4190329
SRR17520069.4190331
SRR17520069.4190333
SRR17520069.4190337
SRR17520069.4190339
SRR17520069.4190341
SRR17520069.4190343
SRR17520069.4190345
SRR17520069.4190347
SRR17520069.4190349
SRR17520069.4190351
SRR17520069.4190353
SRR17520069.4190355
SRR17520069.4190357
SRR17520069.4190359
SRR17520069.4190361
SRR17520069.4190363
SRR17520069.4190365
SRR17520069.4190367
SRR17520069.4190369
SRR17520069.4190371
SRR17520069.4190373
SRR17520069.4190375
SRR17520069.4190377
SRR17520069.4190379
SRR17520069.4190381
SRR17520069.4190383
SRR17520069.4190385
SRR17520069.4190387
SRR17520069.4190389
SRR17520069.4190391
SRR17520069.4190393
SRR17520069.4190395
SRR17520069.4191667
SRR17520069.4191669
SRR17520069.4191671
SRR17520069.4191673
SRR17520069.4191675

SRR17520069.4191677
SRR17520069.4191681
SRR17520069.4191683
SRR17520069.4191685
SRR17520069.4191687
SRR17520069.4191689
SRR17520069.4191691
SRR17520069.4191693
SRR17520069.4191695
SRR17520069.4191697
SRR17520069.4191699
SRR17520069.4191701
SRR17520069.4191703
SRR17520069.4191705
SRR17520069.4191707
SRR17520069.4191709
SRR17520069.4191711
SRR17520069.4191713
SRR17520069.4191715
SRR17520069.4191717
SRR17520069.4191719
SRR17520069.4191721
SRR17520069.4204579
SRR17520069.4205996
SRR17520069.4205998
SRR17520069.4206000
SRR17520069.4206002
SRR17520069.4206004
SRR17520069.4206006
SRR17520069.4206008
SRR17520069.4206010
SRR17520069.4206012
SRR17520069.4206014
SRR17520069.4206016
SRR17520069.4206018
SRR17520069.4206020
SRR17520069.4206573
SRR17520069.4214549
SRR17520069.4214551
SRR17520069.4214557
SRR17520069.4214563
SRR17520069.4214565
SRR17520069.4214567
SRR17520069.4214569
SRR17520069.4214571
SRR17520069.4214573
SRR17520069.4214575

SRR17520069.4214577
SRR17520069.4214579
SRR17520069.4214583
SRR17520069.4214585
SRR17520069.4215624
SRR17520069.4215626
SRR17520069.4215628
SRR17520069.4215630
SRR17520069.4215632
SRR17520069.4215634
SRR17520069.4215636
SRR17520069.4215638
SRR17520069.4215640
SRR17520069.4215642
SRR17520069.4215644
SRR17520069.4215646
SRR17520069.4215648
SRR17520069.4217484
SRR17520069.4217486
SRR17520069.4217488
SRR17520069.4217490
SRR17520069.4217492
SRR17520069.4217494
SRR17520069.4217496
SRR17520069.4217498
SRR17520069.4217500
SRR17520069.4217502
SRR17520069.4217504
SRR17520069.4217506
SRR17520069.4217508
SRR17520069.4217510
SRR17520069.4217512
SRR17520069.4217516
SRR17520069.4217518
SRR17520069.4217520
SRR17520069.4217522
SRR17520069.4217524
SRR17520069.4217526
SRR17520069.4217528
SRR17520069.4217530
SRR17520069.4217532
SRR17520069.4217534
SRR17520069.4217536
SRR17520069.4217540
SRR17520069.4217542
SRR17520069.4217544
SRR17520069.4217546

SRR17520069.4217548
SRR17520069.4217550
SRR17520069.4217552
SRR17520069.4217554
SRR17520069.4217556
SRR17520069.4217558
SRR17520069.4217560
SRR17520069.4217562
SRR17520069.4217564
SRR17520069.4217566
SRR17520069.4217568
SRR17520069.4221315
SRR17520069.4221317
SRR17520069.4223171
SRR17520069.4223173
SRR17520069.4223175
SRR17520069.4223177
SRR17520069.4223179
SRR17520069.4223181
SRR17520069.4223183
SRR17520069.4223185
SRR17520069.4223187
SRR17520069.4223189
SRR17520069.4223191
SRR17520069.4223193
SRR17520069.4223195
SRR17520069.4223197
SRR17520069.4223199
SRR17520069.4223201
SRR17520069.4223203
SRR17520069.4223205
SRR17520069.4223207
SRR17520069.4223209
SRR17520069.4223211
SRR17520069.4223213
SRR17520069.4223215
SRR17520069.4223217
SRR17520069.4223219
SRR17520069.4223221
SRR17520069.4223223
SRR17520069.4223225
SRR17520069.4223229
SRR17520069.4223231
SRR17520069.4223233
SRR17520069.4223235
SRR17520069.4223237
SRR17520069.4223239

SRR17520069.4223241
SRR17520069.4223243
SRR17520069.4223245
SRR17520069.4223247
SRR17520069.4225248
SRR17520069.4225250
SRR17520069.4225252
SRR17520069.4231936
SRR17520069.4231938
SRR17520069.4231940
SRR17520069.4231942
SRR17520069.4231944
SRR17520069.4231946
SRR17520069.4231948
SRR17520069.4231950
SRR17520069.4231952
SRR17520069.4231954
SRR17520069.4231956
SRR17520069.4231958
SRR17520069.4231960
SRR17520069.4231962
SRR17520069.4231964
SRR17520069.4231966
SRR17520069.4231968
SRR17520069.4231970
SRR17520069.4231972
SRR17520069.4231974
SRR17520069.4231976
SRR17520069.4231978
SRR17520069.4231980
SRR17520069.4231982
SRR17520069.4231984
SRR17520069.4231986
SRR17520069.4231988
SRR17520069.4231990
SRR17520069.4231992
SRR17520069.4231994
SRR17520069.4231996
SRR17520069.4231998
SRR17520069.4232000
SRR17520069.4232002
SRR17520069.4232004
SRR17520069.4232006
SRR17520069.4232008
SRR17520069.4232010
SRR17520069.4232012
SRR17520069.4232014

SRR17520069.4232016
SRR17520069.4232018
SRR17520069.4232020
SRR17520069.4232022
SRR17520069.4232024
SRR17520069.4232026
SRR17520069.4232030
SRR17520069.4232032
SRR17520069.4232034
SRR17520069.4232036
SRR17520069.4232038
SRR17520069.4232040
SRR17520069.4232042
SRR17520069.4232044
SRR17520069.4232046
SRR17520069.4232048
SRR17520069.4232050
SRR17520069.4232052
SRR17520069.4232054
SRR17520069.4232056
SRR17520069.4232058
SRR17520069.4232060
SRR17520069.4232062
SRR17520069.4232064
SRR17520069.4232066
SRR17520069.4232068
SRR17520069.4232070
SRR17520069.4232072
SRR17520069.4232074
SRR17520069.4232076
SRR17520069.4232078
SRR17520069.4232080
SRR17520069.4232082
SRR17520069.4232084
SRR17520069.4232086
SRR17520069.4232088
SRR17520069.4232090
SRR17520069.4232092
SRR17520069.4232094
SRR17520069.4232098
SRR17520069.4232100
SRR17520069.4233936
SRR17520069.4233938
SRR17520069.4233940
SRR17520069.4233942
SRR17520069.4233944
SRR17520069.4233946

SRR17520069.4234688
SRR17520069.4234690
SRR17520069.4234692
SRR17520069.4234694
SRR17520069.4234696
SRR17520069.4234698
SRR17520069.4234700
SRR17520069.4234702
SRR17520069.4234704
SRR17520069.4234706
SRR17520069.4234708
SRR17520069.4234710
SRR17520069.4234712
SRR17520069.4234714
SRR17520069.4234752
SRR17520069.4234754
SRR17520069.4234756
SRR17520069.4234758
SRR17520069.4234760
SRR17520069.4234762
SRR17520069.4234764
SRR17520069.4234766
SRR17520069.4234768
SRR17520069.4234770
SRR17520069.4234772
SRR17520069.4234774
SRR17520069.4234776
SRR17520069.4234778
SRR17520069.4234780
SRR17520069.4234782
SRR17520069.4234784
SRR17520069.4234786
SRR17520069.4234788
SRR17520069.4234790
SRR17520069.4234792
SRR17520069.4234794
SRR17520069.4234796
SRR17520069.4234966
SRR17520069.4234968
SRR17520069.4234970
SRR17520069.4234972
SRR17520069.4234974
SRR17520069.4234976
SRR17520069.4234978
SRR17520069.4234980
SRR17520069.4234982
SRR17520069.4234984

SRR17520069.4234986
SRR17520069.4234988
SRR17520069.4234990
SRR17520069.4234992
SRR17520069.4234994
SRR17520069.4234996
SRR17520069.4234998
SRR17520069.4235419
SRR17520069.4235421
SRR17520069.4235423
SRR17520069.4235425
SRR17520069.4235427
SRR17520069.4235429
SRR17520069.4235431
SRR17520069.4235433
SRR17520069.4235435
SRR17520069.4235437
SRR17520069.4235439
SRR17520069.4235441
SRR17520069.4235445
SRR17520069.4235447
SRR17520069.4240553
SRR17520069.4240555
SRR17520069.4240557
SRR17520069.4240559
SRR17520069.4240561
SRR17520069.4240563
SRR17520069.4240565
SRR17520069.4240567
SRR17520069.4240569
SRR17520069.4240571
SRR17520069.4240573
SRR17520069.4240575
SRR17520069.4240577
SRR17520069.4240579
SRR17520069.4240581
SRR17520069.4240583
SRR17520069.4240766
SRR17520069.4240768
SRR17520069.4240770
SRR17520069.4240772
SRR17520069.4240774
SRR17520069.4240776
SRR17520069.4240778
SRR17520069.4240780
SRR17520069.4240782
SRR17520069.4240784

SRR17520069.4240786
SRR17520069.4240788
SRR17520069.4240792
SRR17520069.4240794
SRR17520069.4240796
SRR17520069.4240800
SRR17520069.4240802
SRR17520069.4240804
SRR17520069.4240806
SRR17520069.4240808
SRR17520069.4240810
SRR17520069.4240812
SRR17520069.4240814
SRR17520069.4240816
SRR17520069.4240818
SRR17520069.4240820
SRR17520069.4240822
SRR17520069.4240824
SRR17520069.4240830
SRR17520069.4240832
SRR17520069.4240834
SRR17520069.4240836
SRR17520069.4240840
SRR17520069.4240842
SRR17520069.4240844
SRR17520069.4240846
SRR17520069.4240850
SRR17520069.4240852
SRR17520069.4240854
SRR17520069.4240856
SRR17520069.4240858
SRR17520069.4240862
SRR17520069.4240866
SRR17520069.4240868
SRR17520069.4242378
SRR17520069.4242380
SRR17520069.4242384
SRR17520069.4242699
SRR17520069.4242701
SRR17520069.4242703
SRR17520069.4242705
SRR17520069.4245569
SRR17520069.4245571
SRR17520069.4247738
SRR17520069.4247740
SRR17520069.4247742
SRR17520069.4247744

SRR17520069.4247746
SRR17520069.4247748
SRR17520069.4247750
SRR17520069.4247752
SRR17520069.4247754
SRR17520069.4247756
SRR17520069.4247758
SRR17520069.4247760
SRR17520069.4247762
SRR17520069.4247764
SRR17520069.4247766
SRR17520069.4247768
SRR17520069.4247770
SRR17520069.4247772
SRR17520069.4247774
SRR17520069.4247776
SRR17520069.4247778
SRR17520069.4247780
SRR17520069.4247782
SRR17520069.4247784
SRR17520069.4248572
SRR17520069.4248576
SRR17520069.4248580
SRR17520069.4248582
SRR17520069.4248588
SRR17520069.4248596
SRR17520069.4248598
SRR17520069.4248600
SRR17520069.4248602
SRR17520069.4248604
SRR17520069.4248606
SRR17520069.4248608
SRR17520069.4256825
SRR17520069.4256827
SRR17520069.4256829
SRR17520069.4256831
SRR17520069.4256833
SRR17520069.4256835
SRR17520069.4256837
SRR17520069.4256839
SRR17520069.4256841
SRR17520069.4256843
SRR17520069.4256845
SRR17520069.4256847
SRR17520069.4259355
SRR17520069.4259357
SRR17520069.4259359

SRR17520069.4259361
SRR17520069.4259363
SRR17520069.4259365
SRR17520069.4259367
SRR17520069.4259369
SRR17520069.4259371
SRR17520069.4259373
SRR17520069.4259375
SRR17520069.4259377
SRR17520069.4259379
SRR17520069.4259381
SRR17520069.4259383
SRR17520069.4259385
SRR17520069.4259387
SRR17520069.4259389
SRR17520069.4259391
SRR17520069.4259393
SRR17520069.4259395
SRR17520069.4259397
SRR17520069.4259399
SRR17520069.4259401
SRR17520069.4259403
SRR17520069.4259405
SRR17520069.4259407
SRR17520069.4259409
SRR17520069.4259411
SRR17520069.4259413
SRR17520069.4259415
SRR17520069.4259417
SRR17520069.4259419
SRR17520069.4259421
SRR17520069.4259423
SRR17520069.4259425
SRR17520069.4259427
SRR17520069.4259429
SRR17520069.4259431
SRR17520069.4259433
SRR17520069.4259435
SRR17520069.4259437
SRR17520069.4259439
SRR17520069.4259441
SRR17520069.4259443
SRR17520069.4259445
SRR17520069.4259447
SRR17520069.4259449
SRR17520069.4259451
SRR17520069.4259453

SRR17520069.4259455
SRR17520069.4259457
SRR17520069.4259459
SRR17520069.4259461
SRR17520069.4259463
SRR17520069.4259465
SRR17520069.4259469
SRR17520069.4259471
SRR17520069.4259473
SRR17520069.4259475
SRR17520069.4259477
SRR17520069.4259479
SRR17520069.4259481
SRR17520069.4259483
SRR17520069.4259485
SRR17520069.4259487
SRR17520069.4259489
SRR17520069.4259491
SRR17520069.4259493
SRR17520069.4259495
SRR17520069.4259497
SRR17520069.4259499
SRR17520069.4259501
SRR17520069.4259503
SRR17520069.4262986
SRR17520069.4264347
SRR17520069.4264351
SRR17520069.4264353
SRR17520069.4264355
SRR17520069.4264357
SRR17520069.4264361
SRR17520069.4264363
SRR17520069.4264365
SRR17520069.4264367
SRR17520069.4264371
SRR17520069.4264373
SRR17520069.4264375
SRR17520069.4264377
SRR17520069.4264379
SRR17520069.4264381
SRR17520069.4264383
SRR17520069.4264387
SRR17520069.4264389
SRR17520069.4264731
SRR17520069.4264733
SRR17520069.4264735
SRR17520069.4264737

SRR17520069.4264739
SRR17520069.4264741
SRR17520069.4264743
SRR17520069.4264745
SRR17520069.4264747
SRR17520069.4264749
SRR17520069.4264751
SRR17520069.4264753
SRR17520069.4272757
SRR17520069.4272759
SRR17520069.4272761
SRR17520069.4272763
SRR17520069.4272767
SRR17520069.4272771
SRR17520069.4274523
SRR17520069.4277967
SRR17520069.4277969
SRR17520069.4277971
SRR17520069.4289971
SRR17520069.4289973
SRR17520069.4289975
SRR17520069.4289977
SRR17520069.4289979
SRR17520069.4289981
SRR17520069.4289983
SRR17520069.4289985
SRR17520069.4289987
SRR17520069.4289989
SRR17520069.4289991
SRR17520069.4289993
SRR17520069.4289995
SRR17520069.4289997
SRR17520069.4289999
SRR17520069.4290001
SRR17520069.4290003
SRR17520069.4290005
SRR17520069.4290007
SRR17520069.4290009
SRR17520069.4290011
SRR17520069.4290013
SRR17520069.4290015
SRR17520069.4290017
SRR17520069.4290019
SRR17520069.4290021
SRR17520069.4290023
SRR17520069.4290025
SRR17520069.4290027

SRR17520069.4290029
SRR17520069.4290031
SRR17520069.4290033
SRR17520069.4292474
SRR17520069.4292476
SRR17520069.4292480
SRR17520069.4292484
SRR17520069.4306276
SRR17520069.4306280
SRR17520069.4306282
SRR17520069.4306286
SRR17520069.4306288
SRR17520069.4306290
SRR17520069.4306294
SRR17520069.4306298
SRR17520069.4306300
SRR17520069.4306302
SRR17520069.4306304
SRR17520069.4306306
SRR17520069.4306308
SRR17520069.4306310
SRR17520069.4306312
SRR17520069.4306314
SRR17520069.4306316
SRR17520069.4306318
SRR17520069.4306320
SRR17520069.4306322
SRR17520069.4306324
SRR17520069.4306326
SRR17520069.4306328
SRR17520069.4306330
SRR17520069.4306332
SRR17520069.4313852
SRR17520069.4313854
SRR17520069.4313858
SRR17520069.4313860
SRR17520069.4318140
SRR17520069.4318142
SRR17520069.4319782
SRR17520069.4319784
SRR17520069.4319786
SRR17520069.4319788
SRR17520069.4319790
SRR17520069.4320020
SRR17520069.4320022
SRR17520069.4320026
SRR17520069.4320028

SRR17520069.4320030
SRR17520069.4320032
SRR17520069.4320034
SRR17520069.4320038
SRR17520069.4320040
SRR17520069.4320042
SRR17520069.4320044
SRR17520069.4320046
SRR17520069.4320048
SRR17520069.4320050
SRR17520069.4320052
SRR17520069.4320054
SRR17520069.4320056
SRR17520069.4320058
SRR17520069.4320060
SRR17520069.4320062
SRR17520069.4324353
SRR17520069.4324355
SRR17520069.4324357
SRR17520069.4324359
SRR17520069.4324363
SRR17520069.4324365
SRR17520069.4324367
SRR17520069.4329440
SRR17520069.4329442
SRR17520069.4329446
SRR17520069.4329448
SRR17520069.4329450
SRR17520069.4329452
SRR17520069.4329454
SRR17520069.4329456
SRR17520069.4329458
SRR17520069.4329460
SRR17520069.4329462
SRR17520069.4329465
SRR17520069.4329467
SRR17520069.4329471
SRR17520069.4329473
SRR17520069.4329475
SRR17520069.4329477
SRR17520069.4329479
SRR17520069.4329481
SRR17520069.4329483
SRR17520069.4329485
SRR17520069.4329487
SRR17520069.4329489
SRR17520069.4329491

SRR17520069.4329493
SRR17520069.4329495
SRR17520069.4329497
SRR17520069.4329499
SRR17520069.4329503
SRR17520069.4329505
SRR17520069.4329507
SRR17520069.4329509
SRR17520069.4329511
SRR17520069.4329513
SRR17520069.4329515
SRR17520069.4329517
SRR17520069.4329519
SRR17520069.4329521
SRR17520069.4329523
SRR17520069.4329525
SRR17520069.4329527
SRR17520069.4329529
SRR17520069.4329531
SRR17520069.4329533
SRR17520069.4329535
SRR17520069.4329537
SRR17520069.4329539
SRR17520069.4329541
SRR17520069.4329543
SRR17520069.4329545
SRR17520069.4329547
SRR17520069.4329549
SRR17520069.4329551
SRR17520069.4329553
SRR17520069.4329555
SRR17520069.4329557
SRR17520069.4329559
SRR17520069.4329561
SRR17520069.4329563
SRR17520069.4329565
SRR17520069.4329569
SRR17520069.4329571
SRR17520069.4329573
SRR17520069.4329575
SRR17520069.4329577
SRR17520069.4329579
SRR17520069.4329581
SRR17520069.4329583
SRR17520069.4329587
SRR17520069.4329589
SRR17520069.4329593

SRR17520069.4329595
SRR17520069.4329597
SRR17520069.4329599
SRR17520069.4329601
SRR17520069.4329603
SRR17520069.4333505
SRR17520069.4333507
SRR17520069.4333509
SRR17520069.4333511
SRR17520069.4333513
SRR17520069.4333515
SRR17520069.4333517
SRR17520069.4333519
SRR17520069.4333521
SRR17520069.4333523
SRR17520069.4333525
SRR17520069.4333527
SRR17520069.4333529
SRR17520069.4333531
SRR17520069.4333533
SRR17520069.4333535
SRR17520069.4333537
SRR17520069.4333539
SRR17520069.4333541
SRR17520069.4333543
SRR17520069.4333545
SRR17520069.4333547
SRR17520069.4333549
SRR17520069.4333553
SRR17520069.4333555
SRR17520069.4333557
SRR17520069.4333559
SRR17520069.4333970
SRR17520069.4333972
SRR17520069.4333974
SRR17520069.4333976
SRR17520069.4333978
SRR17520069.4333980
SRR17520069.4333982
SRR17520069.4333984
SRR17520069.4333986
SRR17520069.4333988
SRR17520069.4333990
SRR17520069.4333992
SRR17520069.4333994
SRR17520069.4333996
SRR17520069.4333998

SRR17520069.4334000
SRR17520069.4334002
SRR17520069.4334004
SRR17520069.4334006
SRR17520069.4334008
SRR17520069.4334010
SRR17520069.4334012
SRR17520069.4334014
SRR17520069.4334016
SRR17520069.4334018
SRR17520069.4334020
SRR17520069.4334022
SRR17520069.4336313
SRR17520069.4340049
SRR17520069.4340051
SRR17520069.4340053
SRR17520069.4341287
SRR17520069.4341289
SRR17520069.4341291
SRR17520069.4341293
SRR17520069.4341295
SRR17520069.4342273
SRR17520069.4342275
SRR17520069.4342277
SRR17520069.4342279
SRR17520069.4342281
SRR17520069.4342285
SRR17520069.4342287
SRR17520069.4342289
SRR17520069.4342291
SRR17520069.4342293
SRR17520069.4342295
SRR17520069.4342297
SRR17520069.4342299
SRR17520069.4342301
SRR17520069.4342303
SRR17520069.4342305
SRR17520069.4342307
SRR17520069.4342309
SRR17520069.4342311
SRR17520069.4342313
SRR17520069.4342315
SRR17520069.4342317
SRR17520069.4342319
SRR17520069.4342321
SRR17520069.4342323
SRR17520069.4342325

SRR17520069.4342327
SRR17520069.4342329
SRR17520069.4342331
SRR17520069.4342333
SRR17520069.4342335
SRR17520069.4342337
SRR17520069.4342339
SRR17520069.4342341
SRR17520069.4342343
SRR17520069.4342345
SRR17520069.4342347
SRR17520069.4342349
SRR17520069.4342351
SRR17520069.4342355
SRR17520069.4342357
SRR17520069.4342359
SRR17520069.4355748
SRR17520069.4355750
SRR17520069.4355752
SRR17520069.4355754
SRR17520069.4355756
SRR17520069.4355758
SRR17520069.4355760
SRR17520069.4355762
SRR17520069.4355764
SRR17520069.4355766
SRR17520069.4355768
SRR17520069.4355770
SRR17520069.4355772
SRR17520069.4355774
SRR17520069.4355776
SRR17520069.4355778
SRR17520069.4355780
SRR17520069.4355782
SRR17520069.4355784
SRR17520069.4355786
SRR17520069.4355788
SRR17520069.4355790
SRR17520069.4355792
SRR17520069.4355794
SRR17520069.4355796
SRR17520069.4355798
SRR17520069.4355800
SRR17520069.4355802
SRR17520069.4355804
SRR17520069.4355806
SRR17520069.4355808

SRR17520069.4355810
SRR17520069.4355812
SRR17520069.4355814
SRR17520069.4355816
SRR17520069.4355818
SRR17520069.4355820
SRR17520069.4355822
SRR17520069.4355826
SRR17520069.4355828
SRR17520069.4355830
SRR17520069.4355832
SRR17520069.4355834
SRR17520069.4355836
SRR17520069.4355838
SRR17520069.4355840
SRR17520069.4355842
SRR17520069.4355844
SRR17520069.4355846
SRR17520069.4355848
SRR17520069.4355850
SRR17520069.4355852
SRR17520069.4355854
SRR17520069.4355856
SRR17520069.4355858
SRR17520069.4355860
SRR17520069.4355862
SRR17520069.4355864
SRR17520069.4355866
SRR17520069.4355868
SRR17520069.4355870
SRR17520069.4355872
SRR17520069.4355874
SRR17520069.4355876
SRR17520069.4355878
SRR17520069.4356682
SRR17520069.4356684
SRR17520069.4356868
SRR17520069.4356870
SRR17520069.4356872
SRR17520069.4356876
SRR17520069.4356878
SRR17520069.4356880
SRR17520069.4356882
SRR17520069.4356888
SRR17520069.4356890
SRR17520069.4356892
SRR17520069.4356894

SRR17520069.4357442
SRR17520069.4357446
SRR17520069.4357450
SRR17520069.4357452
SRR17520069.4357454
SRR17520069.4357456
SRR17520069.4357458
SRR17520069.4357460
SRR17520069.4357462
SRR17520069.4357464
SRR17520069.4357466
SRR17520069.4357468
SRR17520069.4357470
SRR17520069.4357472
SRR17520069.4357474
SRR17520069.4357476
SRR17520069.4365563
SRR17520069.4365565
SRR17520069.4365567
SRR17520069.4365569
SRR17520069.4365571
SRR17520069.4365573
SRR17520069.4365575
SRR17520069.4365577
SRR17520069.4365579
SRR17520069.4365581
SRR17520069.4365583
SRR17520069.4365585
SRR17520069.4365587
SRR17520069.4365589
SRR17520069.4365591
SRR17520069.4365593
SRR17520069.4365595
SRR17520069.4365597
SRR17520069.4365599
SRR17520069.4365601
SRR17520069.4365603
SRR17520069.4365605
SRR17520069.4365607
SRR17520069.4367640
SRR17520069.4367642
SRR17520069.4367644
SRR17520069.4367646
SRR17520069.4367648
SRR17520069.4367650
SRR17520069.4373887
SRR17520069.4373889

SRR17520069.4373891
SRR17520069.4373893
SRR17520069.4373895
SRR17520069.4373897
SRR17520069.4373899
SRR17520069.4373901
SRR17520069.4373903
SRR17520069.4373905
SRR17520069.4373907
SRR17520069.4373909
SRR17520069.4373911
SRR17520069.4383235
SRR17520069.4383237
SRR17520069.4383239
SRR17520069.4383241
SRR17520069.4385294
SRR17520069.4385296
SRR17520069.4385300
SRR17520069.4385302
SRR17520069.4385304
SRR17520069.4385306
SRR17520069.4385308
SRR17520069.4385310
SRR17520069.4385312
SRR17520069.4385314
SRR17520069.4385316
SRR17520069.4385318
SRR17520069.4385320
SRR17520069.4385322
SRR17520069.4385324
SRR17520069.4385326
SRR17520069.4385328
SRR17520069.4414652
SRR17520069.4414654
SRR17520069.4414656
SRR17520069.4416749
SRR17520069.4416751
SRR17520069.4416753
SRR17520069.4416755
SRR17520069.4416757
SRR17520069.4416759
SRR17520069.4416911
SRR17520069.4416913
SRR17520069.4416915
SRR17520069.4416917
SRR17520069.4416919
SRR17520069.4416921

SRR17520069.4416923
SRR17520069.4416925
SRR17520069.4416927
SRR17520069.4416929
SRR17520069.4416931
SRR17520069.4416933
SRR17520069.4416935
SRR17520069.4416937
SRR17520069.4416939
SRR17520069.4416941
SRR17520069.4416943
SRR17520069.4416945
SRR17520069.4416947
SRR17520069.4416949
SRR17520069.4416951
SRR17520069.4416953
SRR17520069.4416955
SRR17520069.4416957
SRR17520069.4416959
SRR17520069.4416961
SRR17520069.4416963
SRR17520069.4417073
SRR17520069.4417081
SRR17520069.4417083
SRR17520069.4417085
SRR17520069.4417087
SRR17520069.4417089
SRR17520069.4417091
SRR17520069.4417141
SRR17520069.4429372
SRR17520069.4429374
SRR17520069.4429376
SRR17520069.4429378
SRR17520069.4429380
SRR17520069.4429382
SRR17520069.4429384
SRR17520069.4429386
SRR17520069.4429388
SRR17520069.4429390
SRR17520069.4429392
SRR17520069.4429394
SRR17520069.4429396
SRR17520069.4429398
SRR17520069.4429400
SRR17520069.4435811
SRR17520069.4435813
SRR17520069.4435815

SRR17520069.4435817
SRR17520069.4435819
SRR17520069.4435823
SRR17520069.4435828
SRR17520069.4435830
SRR17520069.4435832
SRR17520069.4435834
SRR17520069.4435836
SRR17520069.4438186
SRR17520069.4438188
SRR17520069.4438190
SRR17520069.4438192
SRR17520069.4438194
SRR17520069.4439767
SRR17520069.4439769
SRR17520069.4439771
SRR17520069.4439773
SRR17520069.4439775
SRR17520069.4439777
SRR17520069.4439779
SRR17520069.4439781
SRR17520069.4439783
SRR17520069.4439785
SRR17520069.4439787
SRR17520069.4441081
SRR17520069.4441087
SRR17520069.4441089
SRR17520069.4441091
SRR17520069.4441093
SRR17520069.4442479
SRR17520069.4442504
SRR17520069.4442506
SRR17520069.4442508
SRR17520069.4442510
SRR17520069.4442537
SRR17520069.4443945
SRR17520069.4443947
SRR17520069.4443949
SRR17520069.4456274
SRR17520069.4456485
SRR17520069.4456487
SRR17520069.4456489
SRR17520069.4456491
SRR17520069.4456493
SRR17520069.4456495
SRR17520069.4456497
SRR17520069.4456499

SRR17520069.4456501
SRR17520069.4456503
SRR17520069.4456505
SRR17520069.4462777
SRR17520069.4462779
SRR17520069.4462781
SRR17520069.4462783
SRR17520069.4462785
SRR17520069.4462787
SRR17520069.4462789
SRR17520069.4462791
SRR17520069.4468521
SRR17520069.4471429
SRR17520069.4471431
SRR17520069.4471437
SRR17520069.4471439
SRR17520069.4474412
SRR17520069.4474414
SRR17520069.4474416
SRR17520069.4474418
SRR17520069.4474420
SRR17520069.4474422
SRR17520069.4474424
SRR17520069.4474426
SRR17520069.4474428
SRR17520069.4474430
SRR17520069.4474432
SRR17520069.4474434
SRR17520069.4474436
SRR17520069.4474438
SRR17520069.4474440
SRR17520069.4474444
SRR17520069.4474446
SRR17520069.4474448
SRR17520069.4474450
SRR17520069.4474452
SRR17520069.4475503
SRR17520069.4475507
SRR17520069.4475509
SRR17520069.4475511
SRR17520069.4475513
SRR17520069.4475515
SRR17520069.4475517
SRR17520069.4475519
SRR17520069.4475521
SRR17520069.4475523
SRR17520069.4475525

SRR17520069.4475527
SRR17520069.4475529
SRR17520069.4475531
SRR17520069.4475533
SRR17520069.4475535
SRR17520069.4475537
SRR17520069.4475539
SRR17520069.4475541
SRR17520069.4475543
SRR17520069.4475545
SRR17520069.4475547
SRR17520069.4475549
SRR17520069.4475551
SRR17520069.4475553
SRR17520069.4475555
SRR17520069.4475557
SRR17520069.4475559
SRR17520069.4475561
SRR17520069.4475563
SRR17520069.4475565
SRR17520069.4475567
SRR17520069.4475569
SRR17520069.4475571
SRR17520069.4475573
SRR17520069.4475575
SRR17520069.4475577
SRR17520069.4475579
SRR17520069.4475581
SRR17520069.4475583
SRR17520069.4475585
SRR17520069.4475587
SRR17520069.4475589
SRR17520069.4475591
SRR17520069.4475593
SRR17520069.4475595
SRR17520069.4475597
SRR17520069.4475599
SRR17520069.4475601
SRR17520069.4478681
SRR17520069.4478685
SRR17520069.4478687
SRR17520069.4478689
SRR17520069.4478691
SRR17520069.4478693
SRR17520069.4478695
SRR17520069.4478697
SRR17520069.4478699

SRR17520069.4478701
SRR17520069.4478703
SRR17520069.4478705
SRR17520069.4478707
SRR17520069.4478709
SRR17520069.4478711
SRR17520069.4478713
SRR17520069.4478715
SRR17520069.4478719
SRR17520069.4478721
SRR17520069.4478723
SRR17520069.4478725
SRR17520069.4478727
SRR17520069.4478729
SRR17520069.4478731
SRR17520069.4478733
SRR17520069.4478735
SRR17520069.4478737
SRR17520069.4478739
SRR17520069.4478741
SRR17520069.4478743
SRR17520069.4478745
SRR17520069.4478747
SRR17520069.4480726
SRR17520069.4480728
SRR17520069.4480730
SRR17520069.4480732
SRR17520069.4480734
SRR17520069.4480736
SRR17520069.4480738
SRR17520069.4480740
SRR17520069.4480742
SRR17520069.4480744
SRR17520069.4480746
SRR17520069.4480748
SRR17520069.4480750
SRR17520069.4480752
SRR17520069.4480754
SRR17520069.4480756
SRR17520069.4480758
SRR17520069.4480760
SRR17520069.4480762
SRR17520069.4480764
SRR17520069.4480768
SRR17520069.4480770
SRR17520069.4480772
SRR17520069.4480774

SRR17520069.4480776
SRR17520069.4480778
SRR17520069.4480780
SRR17520069.4480782
SRR17520069.4480784
SRR17520069.4480786
SRR17520069.4480788
SRR17520069.4480790
SRR17520069.4480792
SRR17520069.4480794
SRR17520069.4480796
SRR17520069.4480798
SRR17520069.4480800
SRR17520069.4480804
SRR17520069.4480806
SRR17520069.4480808
SRR17520069.4480810
SRR17520069.4480812
SRR17520069.4481216
SRR17520069.4481218
SRR17520069.4481220
SRR17520069.4481222
SRR17520069.4481224
SRR17520069.4481226
SRR17520069.4481230
SRR17520069.4481232
SRR17520069.4481234
SRR17520069.4481236
SRR17520069.4481238
SRR17520069.4481240
SRR17520069.4481242
SRR17520069.4481244
SRR17520069.4481246
SRR17520069.4481248
SRR17520069.4481250
SRR17520069.4481252
SRR17520069.4481254
SRR17520069.4481256
SRR17520069.4481258
SRR17520069.4481260
SRR17520069.4481262
SRR17520069.4481266
SRR17520069.4481268
SRR17520069.4481270
SRR17520069.4481272
SRR17520069.4481274
SRR17520069.4481276

SRR17520069.4481278
SRR17520069.4481280
SRR17520069.4481282
SRR17520069.4481284
SRR17520069.4481286
SRR17520069.4481288
SRR17520069.4481290
SRR17520069.4481292
SRR17520069.4481294
SRR17520069.4481296
SRR17520069.4481298
SRR17520069.4481300
SRR17520069.4481302
SRR17520069.4481304
SRR17520069.4481306
SRR17520069.4481308
SRR17520069.4481310
SRR17520069.4481312
SRR17520069.4481314
SRR17520069.4481316
SRR17520069.4481318
SRR17520069.4481320
SRR17520069.4481322
SRR17520069.4481324
SRR17520069.4481326
SRR17520069.4481328
SRR17520069.4481330
SRR17520069.4481332
SRR17520069.4487446
SRR17520069.4487448
SRR17520069.4487450
SRR17520069.4488849
SRR17520069.4488851
SRR17520069.4488853
SRR17520069.4488855
SRR17520069.4488857
SRR17520069.4488859
SRR17520069.4488861
SRR17520069.4488863
SRR17520069.4488865
SRR17520069.4488867
SRR17520069.4488869
SRR17520069.4488871
SRR17520069.4488873
SRR17520069.4488875
SRR17520069.4488877
SRR17520069.4488879

SRR17520069.4488881
SRR17520069.4488883
SRR17520069.4488885
SRR17520069.4488887
SRR17520069.4488889
SRR17520069.4488891
SRR17520069.4488895
SRR17520069.4488897
SRR17520069.4488899
SRR17520069.4488901
SRR17520069.4488903
SRR17520069.4488905
SRR17520069.4488907
SRR17520069.4488909
SRR17520069.4488911
SRR17520069.4492991
SRR17520069.4492993
SRR17520069.4492997
SRR17520069.4492999
SRR17520069.4493001
SRR17520069.4493003
SRR17520069.4493005
SRR17520069.4493007
SRR17520069.4493009
SRR17520069.4493011
SRR17520069.4493013
SRR17520069.4493015
SRR17520069.4493017
SRR17520069.4493019
SRR17520069.4493021
SRR17520069.4493023
SRR17520069.4493025
SRR17520069.4493027
SRR17520069.4493029
SRR17520069.4493031
SRR17520069.4493033
SRR17520069.4493035
SRR17520069.4493037
SRR17520069.4493039
SRR17520069.4493041
SRR17520069.4493043
SRR17520069.4493045
SRR17520069.4493047
SRR17520069.4493049
SRR17520069.4493051
SRR17520069.4493053
SRR17520069.4493055

SRR17520069.4493057
SRR17520069.4493059
SRR17520069.4493061
SRR17520069.4493063
SRR17520069.4493065
SRR17520069.4493067
SRR17520069.4493069
SRR17520069.4503602
SRR17520069.4503604
SRR17520069.4503606
SRR17520069.4503608
SRR17520069.4503610
SRR17520069.4503612
SRR17520069.4503614
SRR17520069.4503616
SRR17520069.4503620
SRR17520069.4503622
SRR17520069.4503624
SRR17520069.4503626
SRR17520069.4503628
SRR17520069.4503632
SRR17520069.4503634
SRR17520069.4503636
SRR17520069.4503638
SRR17520069.4503640
SRR17520069.4503642
SRR17520069.4503644
SRR17520069.4503646
SRR17520069.4503648
SRR17520069.4503650
SRR17520069.4503652
SRR17520069.4503654
SRR17520069.4503656
SRR17520069.4503658
SRR17520069.4503660
SRR17520069.4503662
SRR17520069.4503666
SRR17520069.4503668
SRR17520069.4503670
SRR17520069.4503672
SRR17520069.4503674
SRR17520069.4503676
SRR17520069.4505295
SRR17520069.4505297
SRR17520069.4506250
SRR17520069.4506252
SRR17520069.4506254

SRR17520069.4506256
SRR17520069.4506258
SRR17520069.4506260
SRR17520069.4506262
SRR17520069.4506264
SRR17520069.4506681
SRR17520069.4506683
SRR17520069.4506685
SRR17520069.4506687
SRR17520069.4506689
SRR17520069.4519141
SRR17520069.4519143
SRR17520069.4519145
SRR17520069.4519147
SRR17520069.4519363
SRR17520069.4520523
SRR17520069.4520525
SRR17520069.4520527
SRR17520069.4520529
SRR17520069.4520531
SRR17520069.4520539
SRR17520069.4520541
SRR17520069.4520543
SRR17520069.4520545
SRR17520069.4520547
SRR17520069.4520549
SRR17520069.4520555
SRR17520069.4520557
SRR17520069.4520559
SRR17520069.4520561
SRR17520069.4520563
SRR17520069.4520565
SRR17520069.4520567
SRR17520069.4520569
SRR17520069.4520571
SRR17520069.4521322
SRR17520069.4521324
SRR17520069.4521326
SRR17520069.4521328
SRR17520069.4522959
SRR17520069.4522961
SRR17520069.4522963
SRR17520069.4522965
SRR17520069.4522971
SRR17520069.4525735
SRR17520069.4525737
SRR17520069.4525739

SRR17520069.4525741
SRR17520069.4525743
SRR17520069.4525745
SRR17520069.4525747
SRR17520069.4525749
SRR17520069.4525751
SRR17520069.4525753
SRR17520069.4525755
SRR17520069.4525759
SRR17520069.4525761
SRR17520069.4525763
SRR17520069.4525765
SRR17520069.4525769
SRR17520069.4525771
SRR17520069.4525773
SRR17520069.4525775
SRR17520069.4525777
SRR17520069.4525779
SRR17520069.4525781
SRR17520069.4525785
SRR17520069.4525787
SRR17520069.4526876
SRR17520069.4526880
SRR17520069.4526882
SRR17520069.4526884
SRR17520069.4526890
SRR17520069.4526892
SRR17520069.4526894
SRR17520069.4526896
SRR17520069.4526900
SRR17520069.4526902
SRR17520069.4526904
SRR17520069.4526908
SRR17520069.4526910
SRR17520069.4526912
SRR17520069.4527499
SRR17520069.4527501
SRR17520069.4527505
SRR17520069.4528454
SRR17520069.4528456
SRR17520069.4528458
SRR17520069.4528460
SRR17520069.4528462
SRR17520069.4528464
SRR17520069.4528466
SRR17520069.4528468
SRR17520069.4528470

SRR17520069.4528472
SRR17520069.4528474
SRR17520069.4528478
SRR17520069.4528480
SRR17520069.4528482
SRR17520069.4528484
SRR17520069.4528486
SRR17520069.4528488
SRR17520069.4528492
SRR17520069.4528496
SRR17520069.4528500
SRR17520069.4528504
SRR17520069.4528506
SRR17520069.4528508
SRR17520069.4528665
SRR17520069.4528667
SRR17520069.4528669
SRR17520069.4528671
SRR17520069.4528673
SRR17520069.4528675
SRR17520069.4528677
SRR17520069.4528679
SRR17520069.4528681
SRR17520069.4528683
SRR17520069.4528685
SRR17520069.4528687
SRR17520069.4528689
SRR17520069.4528691
SRR17520069.4528693
SRR17520069.4528695
SRR17520069.4528697
SRR17520069.4528699
SRR17520069.4528701
SRR17520069.4528703
SRR17520069.4528705
SRR17520069.4529998
SRR17520069.4530000
SRR17520069.4530002
SRR17520069.4530004
SRR17520069.4530006
SRR17520069.4530008
SRR17520069.4530010
SRR17520069.4530012
SRR17520069.4530016
SRR17520069.4530018
SRR17520069.4530020
SRR17520069.4530022

SRR17520069.4530024
SRR17520069.4530026
SRR17520069.4530028
SRR17520069.4530030
SRR17520069.4530032
SRR17520069.4530034
SRR17520069.4530038
SRR17520069.4530040
SRR17520069.4530042
SRR17520069.4530044
SRR17520069.4530046
SRR17520069.4530048
SRR17520069.4530050
SRR17520069.4530052
SRR17520069.4530054
SRR17520069.4530056
SRR17520069.4530058
SRR17520069.4530060
SRR17520069.4530062
SRR17520069.4530064
SRR17520069.4530066
SRR17520069.4530068
SRR17520069.4530070
SRR17520069.4530072
SRR17520069.4530074
SRR17520069.4530076
SRR17520069.4530078
SRR17520069.4530080
SRR17520069.4530082
SRR17520069.4530084
SRR17520069.4530086
SRR17520069.4530088
SRR17520069.4530090
SRR17520069.4530092
SRR17520069.4530094
SRR17520069.4530096
SRR17520069.4530098
SRR17520069.4530100
SRR17520069.4530102
SRR17520069.4530104
SRR17520069.4530106
SRR17520069.4530108
SRR17520069.4530110
SRR17520069.4530112
SRR17520069.4530114
SRR17520069.4530116
SRR17520069.4530118

SRR17520069.4530120
SRR17520069.4530122
SRR17520069.4530124
SRR17520069.4530126
SRR17520069.4530128
SRR17520069.4530130
SRR17520069.4530132
SRR17520069.4530134
SRR17520069.4530136
SRR17520069.4530138
SRR17520069.4530140
SRR17520069.4530142
SRR17520069.4530144
SRR17520069.4530146
SRR17520069.4530148
SRR17520069.4530150
SRR17520069.4530152
SRR17520069.4530154
SRR17520069.4530156
SRR17520069.4530158
SRR17520069.4530160
SRR17520069.4530162
SRR17520069.4530164
SRR17520069.4530166
SRR17520069.4530168
SRR17520069.4530170
SRR17520069.4530172
SRR17520069.4530174
SRR17520069.4530176
SRR17520069.4530178
SRR17520069.4530180
SRR17520069.4530182
SRR17520069.4530184
SRR17520069.4530186
SRR17520069.4530188
SRR17520069.4530190
SRR17520069.4530192
SRR17520069.4530194
SRR17520069.4530196
SRR17520069.4530198
SRR17520069.4530200
SRR17520069.4530202
SRR17520069.4530206
SRR17520069.4530208
SRR17520069.4530210
SRR17520069.4530212
SRR17520069.4530214

SRR17520069.4530216
SRR17520069.4530218
SRR17520069.4530220
SRR17520069.4530222
SRR17520069.4530224
SRR17520069.4530226
SRR17520069.4530228
SRR17520069.4530230
SRR17520069.4530232
SRR17520069.4530234
SRR17520069.4530236
SRR17520069.4530238
SRR17520069.4530240
SRR17520069.4530242
SRR17520069.4530244
SRR17520069.4530246
SRR17520069.4530248
SRR17520069.4530250
SRR17520069.4530252
SRR17520069.4531069
SRR17520069.4531071
SRR17520069.4531073
SRR17520069.4531075
SRR17520069.4531079
SRR17520069.4531081
SRR17520069.4531083
SRR17520069.4531085
SRR17520069.4544236
SRR17520069.4544238
SRR17520069.4544242
SRR17520069.4544244
SRR17520069.4544246
SRR17520069.4544248
SRR17520069.4544250
SRR17520069.4544252
SRR17520069.4544254
SRR17520069.4544256
SRR17520069.4544258
SRR17520069.4544260
SRR17520069.4544262
SRR17520069.4544264
SRR17520069.4544266
SRR17520069.4544268
SRR17520069.4544270
SRR17520069.4544272
SRR17520069.4544274
SRR17520069.4544276

SRR17520069.4544278
SRR17520069.4544280
SRR17520069.4544282
SRR17520069.4544284
SRR17520069.4544286
SRR17520069.4544288
SRR17520069.4544292
SRR17520069.4544294
SRR17520069.4545204
SRR17520069.4545206
SRR17520069.4545208
SRR17520069.4545210
SRR17520069.4545212
SRR17520069.4545214
SRR17520069.4545216
SRR17520069.4545218
SRR17520069.4545220
SRR17520069.4545222
SRR17520069.4545224
SRR17520069.4545226
SRR17520069.4545228
SRR17520069.4545230
SRR17520069.4545232
SRR17520069.4545234
SRR17520069.4545236
SRR17520069.4545238
SRR17520069.4545240
SRR17520069.4545244
SRR17520069.4545248
SRR17520069.4545250
SRR17520069.4545252
SRR17520069.4545254
SRR17520069.4545258
SRR17520069.4545260
SRR17520069.4545262
SRR17520069.4545264
SRR17520069.4545266
SRR17520069.4545268
SRR17520069.4545270
SRR17520069.4545272
SRR17520069.4545274
SRR17520069.4545276
SRR17520069.4545278
SRR17520069.4545280
SRR17520069.4545282
SRR17520069.4545284
SRR17520069.4545286

SRR17520069.4545288
SRR17520069.4545290
SRR17520069.4545292
SRR17520069.4545294
SRR17520069.4545296
SRR17520069.4545298
SRR17520069.4545300
SRR17520069.4549477
SRR17520069.4549479
SRR17520069.4549481
SRR17520069.4549483
SRR17520069.4549485
SRR17520069.4549487
SRR17520069.4549489
SRR17520069.4549491
SRR17520069.4549493
SRR17520069.4549495
SRR17520069.4549497
SRR17520069.4549499
SRR17520069.4549501
SRR17520069.4549503
SRR17520069.4549505
SRR17520069.4549507
SRR17520069.4549509
SRR17520069.4549511
SRR17520069.4549513
SRR17520069.4549515
SRR17520069.4549517
SRR17520069.4549519
SRR17520069.4549521
SRR17520069.4549523
SRR17520069.4549525
SRR17520069.4549527
SRR17520069.4549529
SRR17520069.4549531
SRR17520069.4549533
SRR17520069.4549535
SRR17520069.4549537
SRR17520069.4549539
SRR17520069.4549541
SRR17520069.4549543
SRR17520069.4549545
SRR17520069.4549547
SRR17520069.4549549
SRR17520069.4549551
SRR17520069.4551128
SRR17520069.4551130

SRR17520069.4551132
SRR17520069.4551134
SRR17520069.4551136
SRR17520069.4551138
SRR17520069.4551140
SRR17520069.4551142
SRR17520069.4551144
SRR17520069.4551146
SRR17520069.4551148
SRR17520069.4551150
SRR17520069.4551152
SRR17520069.4551154
SRR17520069.4551156
SRR17520069.4551160
SRR17520069.4551162
SRR17520069.4551164
SRR17520069.4551166
SRR17520069.4551168
SRR17520069.4551170
SRR17520069.4551172
SRR17520069.4551176
SRR17520069.4551178
SRR17520069.4551180
SRR17520069.4551182
SRR17520069.4551184
SRR17520069.4551186
SRR17520069.4551188
SRR17520069.4551190
SRR17520069.4551192
SRR17520069.4551194
SRR17520069.4551196
SRR17520069.4551198
SRR17520069.4551200
SRR17520069.4551202
SRR17520069.4551204
SRR17520069.4551206
SRR17520069.4551208
SRR17520069.4551210
SRR17520069.4551212
SRR17520069.4551214
SRR17520069.4551216
SRR17520069.4551218
SRR17520069.4551220
SRR17520069.4551222
SRR17520069.4551224
SRR17520069.4551226
SRR17520069.4551228

SRR17520069.4551230
SRR17520069.4551232
SRR17520069.4551234
SRR17520069.4551236
SRR17520069.4551238
SRR17520069.4551240
SRR17520069.4551242
SRR17520069.4551244
SRR17520069.4551246
SRR17520069.4551248
SRR17520069.4551250
SRR17520069.4551252
SRR17520069.4551254
SRR17520069.4551256
SRR17520069.4551260
SRR17520069.4551262
SRR17520069.4551264
SRR17520069.4551266
SRR17520069.4551268
SRR17520069.4551270
SRR17520069.4551933
SRR17520069.4551935
SRR17520069.4551937
SRR17520069.4551939
SRR17520069.4551941
SRR17520069.4551943
SRR17520069.4551945
SRR17520069.4551947
SRR17520069.4551949
SRR17520069.4554413
SRR17520069.4554415
SRR17520069.4554417
SRR17520069.4554419
SRR17520069.4554421
SRR17520069.4554423
SRR17520069.4554425
SRR17520069.4554427
SRR17520069.4554429
SRR17520069.4554431
SRR17520069.4554433
SRR17520069.4561521
SRR17520069.4561523
SRR17520069.4561525
SRR17520069.4561527
SRR17520069.4561529
SRR17520069.4561531
SRR17520069.4561533

SRR17520069.4561535
SRR17520069.4561537
SRR17520069.4561539
SRR17520069.4561541
SRR17520069.4561543
SRR17520069.4561545
SRR17520069.4561547
SRR17520069.4561549
SRR17520069.4561551
SRR17520069.4561553
SRR17520069.4561555
SRR17520069.4561557
SRR17520069.4561561
SRR17520069.4561563
SRR17520069.4561565
SRR17520069.4561567
SRR17520069.4561569
SRR17520069.4561571
SRR17520069.4561573
SRR17520069.4561575
SRR17520069.4561577
SRR17520069.4562455
SRR17520069.4562457
SRR17520069.4562567
SRR17520069.4562569
SRR17520069.4562571
SRR17520069.4562573
SRR17520069.4562577
SRR17520069.4562785
SRR17520069.4562787
SRR17520069.4562789
SRR17520069.4562791
SRR17520069.4562793
SRR17520069.4562795
SRR17520069.4562797
SRR17520069.4562799
SRR17520069.4562801
SRR17520069.4562803
SRR17520069.4562805
SRR17520069.4562807
SRR17520069.4562809
SRR17520069.4562811
SRR17520069.4562815
SRR17520069.4562819
SRR17520069.4562821
SRR17520069.4562823
SRR17520069.4562825

SRR17520069.4562827
SRR17520069.4562829
SRR17520069.4562831
SRR17520069.4562833
SRR17520069.4562835
SRR17520069.4562839
SRR17520069.4562843
SRR17520069.4562845
SRR17520069.4562847
SRR17520069.4562853
SRR17520069.4563042
SRR17520069.4563044
SRR17520069.4563046
SRR17520069.4563048
SRR17520069.4563050
SRR17520069.4563052
SRR17520069.4565141
SRR17520069.4565143
SRR17520069.4565145
SRR17520069.4565147
SRR17520069.4565149
SRR17520069.4565153
SRR17520069.4565155
SRR17520069.4565157
SRR17520069.4565159
SRR17520069.4565161
SRR17520069.4565163
SRR17520069.4565165
SRR17520069.4565167
SRR17520069.4565169
SRR17520069.4565171
SRR17520069.4565173
SRR17520069.4565175
SRR17520069.4565177
SRR17520069.4565179
SRR17520069.4565181
SRR17520069.4565183
SRR17520069.4565185
SRR17520069.4565187
SRR17520069.4565189
SRR17520069.4565191
SRR17520069.4565193
SRR17520069.4565195
SRR17520069.4565197
SRR17520069.4565199
SRR17520069.4565201
SRR17520069.4565203

SRR17520069.4565205
SRR17520069.4565207
SRR17520069.4565649
SRR17520069.4565651
SRR17520069.4565653
SRR17520069.4565655
SRR17520069.4565657
SRR17520069.4565661
SRR17520069.4567281
SRR17520069.4567283
SRR17520069.4567285
SRR17520069.4567287
SRR17520069.4567289
SRR17520069.4567291
SRR17520069.4567293
SRR17520069.4567295
SRR17520069.4567297
SRR17520069.4567299
SRR17520069.4567301
SRR17520069.4567303
SRR17520069.4567305
SRR17520069.4569013
SRR17520069.4569015
SRR17520069.4569017
SRR17520069.4569019
SRR17520069.4569021
SRR17520069.4569023
SRR17520069.4569025
SRR17520069.4569027
SRR17520069.4569029
SRR17520069.4579196
SRR17520069.4579198
SRR17520069.4579200
SRR17520069.4579202
SRR17520069.4579204
SRR17520069.4579206
SRR17520069.4592967
SRR17520069.4592969
SRR17520069.4592971
SRR17520069.4592973
SRR17520069.4592975
SRR17520069.4592977
SRR17520069.4592979
SRR17520069.4592981
SRR17520069.4592985
SRR17520069.4592987
SRR17520069.4592989

SRR17520069.4592991
SRR17520069.4594999
SRR17520069.4604089
SRR17520069.4604091
SRR17520069.4604093
SRR17520069.4604095
SRR17520069.4604097
SRR17520069.4604099
SRR17520069.4604101
SRR17520069.4607647
SRR17520069.4607649
SRR17520069.4611521
SRR17520069.4616262
SRR17520069.4616594
SRR17520069.4616596
SRR17520069.4617118
SRR17520069.4617120
SRR17520069.4617122
SRR17520069.4617124
SRR17520069.4617126
SRR17520069.4617128
SRR17520069.4617130
SRR17520069.4617132
SRR17520069.4617134
SRR17520069.4617136
SRR17520069.4617138
SRR17520069.4617140
SRR17520069.4617142
SRR17520069.4617144
SRR17520069.4622792
SRR17520069.4622794
SRR17520069.4622796
SRR17520069.4622798
SRR17520069.4622800
SRR17520069.4622802
SRR17520069.4622804
SRR17520069.4622806
SRR17520069.4622808
SRR17520069.4622810
SRR17520069.4622812
SRR17520069.4622814
SRR17520069.4622816
SRR17520069.4622818
SRR17520069.4622820
SRR17520069.4622822
SRR17520069.4622824
SRR17520069.4622826

SRR17520069.4622828
SRR17520069.4622830
SRR17520069.4622832
SRR17520069.4622834
SRR17520069.4622836
SRR17520069.4622838
SRR17520069.4622840
SRR17520069.4622842
SRR17520069.4622844
SRR17520069.4622846
SRR17520069.4622848
SRR17520069.4622850
SRR17520069.4626135
SRR17520069.4626137
SRR17520069.4626139
SRR17520069.4626141
SRR17520069.4626143
SRR17520069.4626145
SRR17520069.4626147
SRR17520069.4626149
SRR17520069.4626151
SRR17520069.4626153
SRR17520069.4626155
SRR17520069.4626157
SRR17520069.4626159
SRR17520069.4626161
SRR17520069.4626163
SRR17520069.4626165
SRR17520069.4626167
SRR17520069.4626169
SRR17520069.4626171
SRR17520069.4626173
SRR17520069.4626175
SRR17520069.4626177
SRR17520069.4626179
SRR17520069.4626181
SRR17520069.4626183
SRR17520069.4626185
SRR17520069.4626187
SRR17520069.4626189
SRR17520069.4629222
SRR17520069.4629224
SRR17520069.4629226
SRR17520069.4629228
SRR17520069.4629230
SRR17520069.4629232
SRR17520069.4629234

SRR17520069.4633157
SRR17520069.4639505
SRR17520069.4639507
SRR17520069.4639509
SRR17520069.4639511
SRR17520069.4639513
SRR17520069.4639517
SRR17520069.4639519
SRR17520069.4639521
SRR17520069.4639523
SRR17520069.4639525
SRR17520069.4639527
SRR17520069.4639529
SRR17520069.4639531
SRR17520069.4639533
SRR17520069.4639535
SRR17520069.4639537
SRR17520069.4639539
SRR17520069.4639541
SRR17520069.4639543
SRR17520069.4639545
SRR17520069.4639547
SRR17520069.4639549
SRR17520069.4639553
SRR17520069.4639555
SRR17520069.4639557
SRR17520069.4639563
SRR17520069.4639565
SRR17520069.4639567
SRR17520069.4639569
SRR17520069.4639571
SRR17520069.4639573
SRR17520069.4639575
SRR17520069.4639577
SRR17520069.4639579
SRR17520069.4639581
SRR17520069.4639583
SRR17520069.4639585
SRR17520069.4639587
SRR17520069.4639589
SRR17520069.4639591
SRR17520069.4639593
SRR17520069.4639595
SRR17520069.4639597
SRR17520069.4639599
SRR17520069.4639601
SRR17520069.4639603

SRR17520069.4639605
SRR17520069.4639607
SRR17520069.4639611
SRR17520069.4639613
SRR17520069.4639615
SRR17520069.4639617
SRR17520069.4639619
SRR17520069.4639621
SRR17520069.4639623
SRR17520069.4639627
SRR17520069.4639629
SRR17520069.4639631
SRR17520069.4639633
SRR17520069.4639635
SRR17520069.4639637
SRR17520069.4639641
SRR17520069.4639643
SRR17520069.4639645
SRR17520069.4639647
SRR17520069.4639649
SRR17520069.4639651
SRR17520069.4639653
SRR17520069.4639655
SRR17520069.4639657
SRR17520069.4639659
SRR17520069.4639661
SRR17520069.4639663
SRR17520069.4639669
SRR17520069.4639671
SRR17520069.4639673
SRR17520069.4639675
SRR17520069.4639677
SRR17520069.4639679
SRR17520069.4639681
SRR17520069.4639685
SRR17520069.4639687
SRR17520069.4639689
SRR17520069.4640025
SRR17520069.4640027
SRR17520069.4640029
SRR17520069.4641685
SRR17520069.4641687
SRR17520069.4641689
SRR17520069.4641693
SRR17520069.4642511
SRR17520069.4642515
SRR17520069.4642517

SRR17520069.4642519
SRR17520069.4642521
SRR17520069.4642523
SRR17520069.4642529
SRR17520069.4642531
SRR17520069.4642533
SRR17520069.4642535
SRR17520069.4642537
SRR17520069.4642541
SRR17520069.4642543
SRR17520069.4642545
SRR17520069.4642547
SRR17520069.4642549
SRR17520069.4642551
SRR17520069.4642553
SRR17520069.4642555
SRR17520069.4642557
SRR17520069.4642559
SRR17520069.4642561
SRR17520069.4642563
SRR17520069.4642565
SRR17520069.4642567
SRR17520069.4642569
SRR17520069.4642573
SRR17520069.4642577
SRR17520069.4642579
SRR17520069.4642581
SRR17520069.4642583
SRR17520069.4642585
SRR17520069.4642587
SRR17520069.4642589
SRR17520069.4642591
SRR17520069.4642593
SRR17520069.4642595
SRR17520069.4642597
SRR17520069.4642599
SRR17520069.4642601
SRR17520069.4642603
SRR17520069.4642605
SRR17520069.4642607
SRR17520069.4642609
SRR17520069.4642613
SRR17520069.4642615
SRR17520069.4645903
SRR17520069.4655934
SRR17520069.4655936
SRR17520069.4655938

SRR17520069.4655940
SRR17520069.4655942
SRR17520069.4655944
SRR17520069.4655946
SRR17520069.4655948
SRR17520069.4655950
SRR17520069.4655952
SRR17520069.4655954
SRR17520069.4655956
SRR17520069.4655958
SRR17520069.4655960
SRR17520069.4655962
SRR17520069.4655964
SRR17520069.4655966
SRR17520069.4655968
SRR17520069.4655970
SRR17520069.4655972
SRR17520069.4655974
SRR17520069.4655976
SRR17520069.4655978
SRR17520069.4655980
SRR17520069.4655982
SRR17520069.4655984
SRR17520069.4656915
SRR17520069.4656917
SRR17520069.4656919
SRR17520069.4656921
SRR17520069.4656923
SRR17520069.4656925
SRR17520069.4656927
SRR17520069.4656929
SRR17520069.4656931
SRR17520069.4674856
SRR17520069.4674858
SRR17520069.4674860
SRR17520069.4674862
SRR17520069.4674864
SRR17520069.4674866
SRR17520069.4674868
SRR17520069.4674870
SRR17520069.4674872
SRR17520069.4674874
SRR17520069.4674876
SRR17520069.4674878
SRR17520069.4674880
SRR17520069.4674882
SRR17520069.4674884

SRR17520069.4674886
SRR17520069.4674888
SRR17520069.4674890
SRR17520069.4674892
SRR17520069.4674894
SRR17520069.4674896
SRR17520069.4674898
SRR17520069.4674900
SRR17520069.4674902
SRR17520069.4674904
SRR17520069.4674906
SRR17520069.4674908
SRR17520069.4674910
SRR17520069.4674912
SRR17520069.4674914
SRR17520069.4674916
SRR17520069.4677559
SRR17520069.4677561
SRR17520069.4677563
SRR17520069.4677565
SRR17520069.4677567
SRR17520069.4677569
SRR17520069.4677571
SRR17520069.4677573
SRR17520069.4677575
SRR17520069.4677577
SRR17520069.4677579
SRR17520069.4677581
SRR17520069.4677583
SRR17520069.4677585
SRR17520069.4677587
SRR17520069.4677589
SRR17520069.4677591
SRR17520069.4677593
SRR17520069.4677595
SRR17520069.4677597
SRR17520069.4677599
SRR17520069.4677601
SRR17520069.4677603
SRR17520069.4677605
SRR17520069.4677607
SRR17520069.4677609
SRR17520069.4677611
SRR17520069.4677613
SRR17520069.4677615
SRR17520069.4677617
SRR17520069.4677621

SRR17520069.4677623
SRR17520069.4677625
SRR17520069.4677627
SRR17520069.4677629
SRR17520069.4677631
SRR17520069.4677633
SRR17520069.4677635
SRR17520069.4677637
SRR17520069.4677639
SRR17520069.4679263
SRR17520069.4679265
SRR17520069.4679267
SRR17520069.4679269
SRR17520069.4679271
SRR17520069.4679273
SRR17520069.4679275
SRR17520069.4679277
SRR17520069.4679279
SRR17520069.4679281
SRR17520069.4679283
SRR17520069.4679285
SRR17520069.4679287
SRR17520069.4679289
SRR17520069.4679291
SRR17520069.4679293
SRR17520069.4679295
SRR17520069.4679297
SRR17520069.4679299
SRR17520069.4679301
SRR17520069.4679303
SRR17520069.4679305
SRR17520069.4679307
SRR17520069.4679311
SRR17520069.4679313
SRR17520069.4679315
SRR17520069.4679317
SRR17520069.4684474
SRR17520069.4684476
SRR17520069.4684478
SRR17520069.4684480
SRR17520069.4684482
SRR17520069.4684484
SRR17520069.4684486
SRR17520069.4684488
SRR17520069.4684490
SRR17520069.4684492
SRR17520069.4684494

SRR17520069.4684496
SRR17520069.4684500
SRR17520069.4684502
SRR17520069.4685138
SRR17520069.4685140
SRR17520069.4685142
SRR17520069.4685144
SRR17520069.4685146
SRR17520069.4685950
SRR17520069.4685952
SRR17520069.4685954
SRR17520069.4685956
SRR17520069.4685958
SRR17520069.4686714
SRR17520069.4687170
SRR17520069.4687172
SRR17520069.4687174
SRR17520069.4687176
SRR17520069.4687178
SRR17520069.4687180
SRR17520069.4687182
SRR17520069.4687184
SRR17520069.4687186
SRR17520069.4687188
SRR17520069.4687190
SRR17520069.4687192
SRR17520069.4687194
SRR17520069.4687196
SRR17520069.4687198
SRR17520069.4689789
SRR17520069.4689791
SRR17520069.4689793
SRR17520069.4689795
SRR17520069.4689797
SRR17520069.4689799
SRR17520069.4689801
SRR17520069.4689803
SRR17520069.4689805
SRR17520069.4689807
SRR17520069.4689809
SRR17520069.4689811
SRR17520069.4689813
SRR17520069.4689815
SRR17520069.4689817
SRR17520069.4689819
SRR17520069.4689821
SRR17520069.4689823

SRR17520069.4689825
SRR17520069.4689827
SRR17520069.4689829
SRR17520069.4689831
SRR17520069.4689833
SRR17520069.4689835
SRR17520069.4689837
SRR17520069.4689839
SRR17520069.4689841
SRR17520069.4691672
SRR17520069.4691674
SRR17520069.4691676
SRR17520069.4691678
SRR17520069.4691680
SRR17520069.4691682
SRR17520069.4691684
SRR17520069.4699833
SRR17520069.4699835
SRR17520069.4699837
SRR17520069.4699841
SRR17520069.4699843
SRR17520069.4699845
SRR17520069.4699847
SRR17520069.4699851
SRR17520069.4699853
SRR17520069.4699855
SRR17520069.4699859
SRR17520069.4699861
SRR17520069.4699863
SRR17520069.4699865
SRR17520069.4699867
SRR17520069.4699869
SRR17520069.4699871
SRR17520069.4699873
SRR17520069.4699875
SRR17520069.4699877
SRR17520069.4699879
SRR17520069.4699881
SRR17520069.4699883
SRR17520069.4699885
SRR17520069.4699887
SRR17520069.4699889
SRR17520069.4699891
SRR17520069.4699893
SRR17520069.4699895
SRR17520069.4699897
SRR17520069.4699899

SRR17520069.4699901
SRR17520069.4699903
SRR17520069.4699905
SRR17520069.4699907
SRR17520069.4699909
SRR17520069.4699911
SRR17520069.4699913
SRR17520069.4699915
SRR17520069.4699917
SRR17520069.4699919
SRR17520069.4699921
SRR17520069.4703107
SRR17520069.4703110
SRR17520069.4703293
SRR17520069.4703295
SRR17520069.4703297
SRR17520069.4703299
SRR17520069.4703301
SRR17520069.4703303
SRR17520069.4703305
SRR17520069.4703307
SRR17520069.4703309
SRR17520069.4703311
SRR17520069.4703313
SRR17520069.4703315
SRR17520069.4703317
SRR17520069.4703319
SRR17520069.4703321
SRR17520069.4703323
SRR17520069.4703325
SRR17520069.4703327
SRR17520069.4703329
SRR17520069.4703331
SRR17520069.4703333
SRR17520069.4705766
SRR17520069.4705768
SRR17520069.4705770
SRR17520069.4705772
SRR17520069.4705774
SRR17520069.4705776
SRR17520069.4705778
SRR17520069.4705780
SRR17520069.4705782
SRR17520069.4705784
SRR17520069.4705786
SRR17520069.4705788
SRR17520069.4705790

SRR17520069.4705792
SRR17520069.4705794
SRR17520069.4705796
SRR17520069.4705798
SRR17520069.4705800
SRR17520069.4705802
SRR17520069.4705804
SRR17520069.4705806
SRR17520069.4705808
SRR17520069.4705810
SRR17520069.4705812
SRR17520069.4705814
SRR17520069.4705816
SRR17520069.4705818
SRR17520069.4705822
SRR17520069.4705824
SRR17520069.4705826
SRR17520069.4705828
SRR17520069.4705830
SRR17520069.4705832
SRR17520069.4705834
SRR17520069.4705838
SRR17520069.4705844
SRR17520069.4705846
SRR17520069.4708683
SRR17520069.4708685
SRR17520069.4708687
SRR17520069.4708689
SRR17520069.4708691
SRR17520069.4708693
SRR17520069.4710329
SRR17520069.4710331
SRR17520069.4710333
SRR17520069.4710335
SRR17520069.4710337
SRR17520069.4710339
SRR17520069.4710341
SRR17520069.4711507
SRR17520069.4711522
SRR17520069.4711524
SRR17520069.4711526
SRR17520069.4711528
SRR17520069.4711530
SRR17520069.4712583
SRR17520069.4712585
SRR17520069.4712587
SRR17520069.4712589

SRR17520069.4712591
SRR17520069.4712595
SRR17520069.4712601
SRR17520069.4712603
SRR17520069.4712605
SRR17520069.4712609
SRR17520069.4712611
SRR17520069.4712613
SRR17520069.4719388
SRR17520069.4719390
SRR17520069.4719392
SRR17520069.4719394
SRR17520069.4719396
SRR17520069.4719400
SRR17520069.4719402
SRR17520069.4719404
SRR17520069.4719406
SRR17520069.4719408
SRR17520069.4719605
SRR17520069.4719607
SRR17520069.4719609
SRR17520069.4719613
SRR17520069.4719629
SRR17520069.4719631
SRR17520069.4719633
SRR17520069.4719635
SRR17520069.4719637
SRR17520069.4719639
SRR17520069.4719643
SRR17520069.4719645
SRR17520069.4719647
SRR17520069.4719649
SRR17520069.4719651
SRR17520069.4719653
SRR17520069.4719655
SRR17520069.4719657
SRR17520069.4719659
SRR17520069.4719661
SRR17520069.4719667
SRR17520069.4720477
SRR17520069.4730894
SRR17520069.4730896
SRR17520069.4730898
SRR17520069.4730900
SRR17520069.4730902
SRR17520069.4730904
SRR17520069.4730906

SRR17520069.4730908
SRR17520069.4730910
SRR17520069.4730912
SRR17520069.4730914
SRR17520069.4738443
SRR17520069.4738445
SRR17520069.4738449
SRR17520069.4738451
SRR17520069.4738453
SRR17520069.4738455
SRR17520069.4738457
SRR17520069.4738459
SRR17520069.4738461
SRR17520069.4738463
SRR17520069.4738465
SRR17520069.4738467
SRR17520069.4738469
SRR17520069.4738471
SRR17520069.4738473
SRR17520069.4741427
SRR17520069.4741429
SRR17520069.4741431
SRR17520069.4741433
SRR17520069.4741435
SRR17520069.4741437
SRR17520069.4741439
SRR17520069.4741441
SRR17520069.4741443
SRR17520069.4741445
SRR17520069.4741447
SRR17520069.4741449
SRR17520069.4741451
SRR17520069.4741453
SRR17520069.4741455
SRR17520069.4741457
SRR17520069.4741459
SRR17520069.4741461
SRR17520069.4741463
SRR17520069.4741465
SRR17520069.4741467
SRR17520069.4741469
SRR17520069.4741473
SRR17520069.4741475
SRR17520069.4741477
SRR17520069.4741479
SRR17520069.4741481
SRR17520069.4741483

SRR17520069.4741485
SRR17520069.4741487
SRR17520069.4741489
SRR17520069.4741493
SRR17520069.4741495
SRR17520069.4741497
SRR17520069.4745742
SRR17520069.4747942
SRR17520069.4747944
SRR17520069.4747946
SRR17520069.4747948
SRR17520069.4747950
SRR17520069.4747952
SRR17520069.4749459
SRR17520069.4749461
SRR17520069.4749463
SRR17520069.4749465
SRR17520069.4749467
SRR17520069.4749469
SRR17520069.4749471
SRR17520069.4749473
SRR17520069.4749475
SRR17520069.4749477
SRR17520069.4749479
SRR17520069.4749481
SRR17520069.4749483
SRR17520069.4749488
SRR17520069.4749490
SRR17520069.4749492
SRR17520069.4749494
SRR17520069.4749496
SRR17520069.4756758
SRR17520069.4763367
SRR17520069.4763369
SRR17520069.4763371
SRR17520069.4763832
SRR17520069.4763834
SRR17520069.4763836
SRR17520069.4763840
SRR17520069.4765225
SRR17520069.4768317
SRR17520069.4768319
SRR17520069.4768321
SRR17520069.4768323
SRR17520069.4768325
SRR17520069.4770516
SRR17520069.4770518

SRR17520069.4770520
SRR17520069.4770522
SRR17520069.4770524
SRR17520069.4770526
SRR17520069.4770528
SRR17520069.4770530
SRR17520069.4770532
SRR17520069.4770534
SRR17520069.4771114
SRR17520069.4771118
SRR17520069.4771120
SRR17520069.4771122
SRR17520069.4771124
SRR17520069.4771130
SRR17520069.4771136
SRR17520069.4771138
SRR17520069.4771466
SRR17520069.4771468
SRR17520069.4771470
SRR17520069.4771472
SRR17520069.4771474
SRR17520069.4771476
SRR17520069.4771478
SRR17520069.4771482
SRR17520069.4771484
SRR17520069.4771486
SRR17520069.4771490
SRR17520069.4771492
SRR17520069.4771500
SRR17520069.4771502
SRR17520069.4771506
SRR17520069.4771508
SRR17520069.4771510
SRR17520069.4771514
SRR17520069.4771516
SRR17520069.4771518
SRR17520069.4771520
SRR17520069.4785450
SRR17520069.4785452
SRR17520069.4785454
SRR17520069.4785456
SRR17520069.4785458
SRR17520069.4785460
SRR17520069.4785462
SRR17520069.4785464
SRR17520069.4785466
SRR17520069.4785468

SRR17520069.4785470
SRR17520069.4785474
SRR17520069.4785476
SRR17520069.4785478
SRR17520069.4785480
SRR17520069.4785482
SRR17520069.4785484
SRR17520069.4785486
SRR17520069.4785488
SRR17520069.4785490
SRR17520069.4785492
SRR17520069.4785494
SRR17520069.4785496
SRR17520069.4785498
SRR17520069.4785500
SRR17520069.4785504
SRR17520069.4785506
SRR17520069.4785508
SRR17520069.4785510
SRR17520069.4785512
SRR17520069.4785514
SRR17520069.4785516
SRR17520069.4785518
SRR17520069.4785520
SRR17520069.4785522
SRR17520069.4785524
SRR17520069.4785526
SRR17520069.4785528
SRR17520069.4785530
SRR17520069.4785532
SRR17520069.4785534
SRR17520069.4785536
SRR17520069.4785538
SRR17520069.4785542
SRR17520069.4785544
SRR17520069.4785548
SRR17520069.4785550
SRR17520069.4785552
SRR17520069.4785554
SRR17520069.4785556
SRR17520069.4785558
SRR17520069.4785560
SRR17520069.4785564
SRR17520069.4785566
SRR17520069.4785568
SRR17520069.4785570
SRR17520069.4785572

SRR17520069.4785574
SRR17520069.4785576
SRR17520069.4791378
SRR17520069.4792216
SRR17520069.4793279
SRR17520069.4793281
SRR17520069.4793283
SRR17520069.4793285
SRR17520069.4793287
SRR17520069.4793289
SRR17520069.4793291
SRR17520069.4798391
SRR17520069.4798393
SRR17520069.4798395
SRR17520069.4798397
SRR17520069.4798399
SRR17520069.4798401
SRR17520069.4798403
SRR17520069.4798405
SRR17520069.4798407
SRR17520069.4798409
SRR17520069.4798411
SRR17520069.4798413
SRR17520069.4798415
SRR17520069.4798417
SRR17520069.4798419
SRR17520069.4798421
SRR17520069.4798423
SRR17520069.4798425
SRR17520069.4798427
SRR17520069.4798429
SRR17520069.4798431
SRR17520069.4798433
SRR17520069.4798435
SRR17520069.4798437
SRR17520069.4798439
SRR17520069.4798441
SRR17520069.4798443
SRR17520069.4798445
SRR17520069.4798447
SRR17520069.4798449
SRR17520069.4798451
SRR17520069.4798453
SRR17520069.4798455
SRR17520069.4798457
SRR17520069.4798459
SRR17520069.4798461

SRR17520069.4798463
SRR17520069.4798465
SRR17520069.4803868
SRR17520069.4803870
SRR17520069.4803872
SRR17520069.4803874
SRR17520069.4803876
SRR17520069.4803878
SRR17520069.4803880
SRR17520069.4803882
SRR17520069.4803884
SRR17520069.4803888
SRR17520069.4803890
SRR17520069.4803892
SRR17520069.4803894
SRR17520069.4803896
SRR17520069.4803898
SRR17520069.4803900
SRR17520069.4803902
SRR17520069.4803904
SRR17520069.4803906
SRR17520069.4803908
SRR17520069.4803910
SRR17520069.4820719
SRR17520069.4820725
SRR17520069.4820727
SRR17520069.4820729
SRR17520069.4820733
SRR17520069.4820735
SRR17520069.4820737
SRR17520069.4820739
SRR17520069.4820741
SRR17520069.4820743
SRR17520069.4820745
SRR17520069.4820747
SRR17520069.4820751
SRR17520069.4820753
SRR17520069.4820755
SRR17520069.4820757
SRR17520069.4820759
SRR17520069.4820761
SRR17520069.4820763
SRR17520069.4820765
SRR17520069.4820767
SRR17520069.4820769
SRR17520069.4820771
SRR17520069.4821501

SRR17520069.4826967
SRR17520069.4826969
SRR17520069.4826971
SRR17520069.4826973
SRR17520069.4826975
SRR17520069.4826977
SRR17520069.4826979
SRR17520069.4826981
SRR17520069.4826983
SRR17520069.4826985
SRR17520069.4826987
SRR17520069.4826989
SRR17520069.4826991
SRR17520069.4826993
SRR17520069.4826995
SRR17520069.4826997
SRR17520069.4826999
SRR17520069.4827001
SRR17520069.4827003
SRR17520069.4827005
SRR17520069.4827007
SRR17520069.4827009
SRR17520069.4827011
SRR17520069.4827013
SRR17520069.4827015
SRR17520069.4827017
SRR17520069.4827019
SRR17520069.4827021
SRR17520069.4827023
SRR17520069.4829148
SRR17520069.4829150
SRR17520069.4829152
SRR17520069.4829154
SRR17520069.4829156
SRR17520069.4829158
SRR17520069.4829160
SRR17520069.4829162
SRR17520069.4829164
SRR17520069.4829166
SRR17520069.4829168
SRR17520069.4829170
SRR17520069.4829172
SRR17520069.4829174
SRR17520069.4829176
SRR17520069.4829182
SRR17520069.4829184
SRR17520069.4829186

SRR17520069.4829188
SRR17520069.4829190
SRR17520069.4829192
SRR17520069.4832396
SRR17520069.4837739
SRR17520069.4837741
SRR17520069.4837743
SRR17520069.4837745
SRR17520069.4837747
SRR17520069.4837749
SRR17520069.4837751
SRR17520069.4837753
SRR17520069.4837755
SRR17520069.4837757
SRR17520069.4837759
SRR17520069.4837761
SRR17520069.4837763
SRR17520069.4837765
SRR17520069.4837767
SRR17520069.4837769
SRR17520069.4837771
SRR17520069.4837773
SRR17520069.4837775
SRR17520069.4837777
SRR17520069.4837779
SRR17520069.4837781
SRR17520069.4837783
SRR17520069.4837785
SRR17520069.4837787
SRR17520069.4837789
SRR17520069.4837791
SRR17520069.4837793
SRR17520069.4837795
SRR17520069.4837797
SRR17520069.4837799
SRR17520069.4837801
SRR17520069.4837803
SRR17520069.4837805
SRR17520069.4837807
SRR17520069.4837809
SRR17520069.4837811
SRR17520069.4837813
SRR17520069.4837815
SRR17520069.4837817
SRR17520069.4837819
SRR17520069.4837821
SRR17520069.4837823

SRR17520069.4837825
SRR17520069.4837827
SRR17520069.4838836
SRR17520069.4838838
SRR17520069.4838840
SRR17520069.4838842
SRR17520069.4838844
SRR17520069.4838846
SRR17520069.4838848
SRR17520069.4838850
SRR17520069.4838852
SRR17520069.4838854
SRR17520069.4838856
SRR17520069.4838858
SRR17520069.4838862
SRR17520069.4838864
SRR17520069.4838868
SRR17520069.4838870
SRR17520069.4838872
SRR17520069.4838874
SRR17520069.4838876
SRR17520069.4838878
SRR17520069.4838880
SRR17520069.4838882
SRR17520069.4838884
SRR17520069.4838886
SRR17520069.4838888
SRR17520069.4838890
SRR17520069.4838892
SRR17520069.4838894
SRR17520069.4838896
SRR17520069.4838898
SRR17520069.4838900
SRR17520069.4838902
SRR17520069.4838904
SRR17520069.4838906
SRR17520069.4838908
SRR17520069.4838910
SRR17520069.4838912
SRR17520069.4838914
SRR17520069.4838916
SRR17520069.4838920
SRR17520069.4838922
SRR17520069.4838924
SRR17520069.4838926
SRR17520069.4838928
SRR17520069.4841940

SRR17520069.4841942
SRR17520069.4841944
SRR17520069.4841946
SRR17520069.4841948
SRR17520069.4841950
SRR17520069.4841952
SRR17520069.4841954
SRR17520069.4841956
SRR17520069.4841958
SRR17520069.4841960
SRR17520069.4842533
SRR17520069.4842535
SRR17520069.4842537
SRR17520069.4842539
SRR17520069.4842541
SRR17520069.4842543
SRR17520069.4842545
SRR17520069.4842547
SRR17520069.4842549
SRR17520069.4842551
SRR17520069.4842553
SRR17520069.4842555
SRR17520069.4842557
SRR17520069.4842559
SRR17520069.4842561
SRR17520069.4842563
SRR17520069.4842565
SRR17520069.4842567
SRR17520069.4842569
SRR17520069.4842571
SRR17520069.4842573
SRR17520069.4842575
SRR17520069.4842577
SRR17520069.4842579
SRR17520069.4842581
SRR17520069.4842583
SRR17520069.4842585
SRR17520069.4842587
SRR17520069.4842589
SRR17520069.4842591
SRR17520069.4842593
SRR17520069.4842595
SRR17520069.4842597
SRR17520069.4842599
SRR17520069.4842601
SRR17520069.4842603
SRR17520069.4842605

SRR17520069.4842607
SRR17520069.4842609
SRR17520069.4842611
SRR17520069.4842613
SRR17520069.4842615
SRR17520069.4842617
SRR17520069.4842619
SRR17520069.4842621
SRR17520069.4842623
SRR17520069.4842625
SRR17520069.4842627
SRR17520069.4842629
SRR17520069.4842631
SRR17520069.4842633
SRR17520069.4842635
SRR17520069.4842637
SRR17520069.4842639
SRR17520069.4842641
SRR17520069.4842643
SRR17520069.4842645
SRR17520069.4842647
SRR17520069.4842649
SRR17520069.4842651
SRR17520069.4842653
SRR17520069.4842655
SRR17520069.4842657
SRR17520069.4842659
SRR17520069.4842661
SRR17520069.4842663
SRR17520069.4842665
SRR17520069.4842667
SRR17520069.4842669
SRR17520069.4842671
SRR17520069.4842673
SRR17520069.4842675
SRR17520069.4842677
SRR17520069.4842679
SRR17520069.4842681
SRR17520069.4842683
SRR17520069.4842687
SRR17520069.4842689
SRR17520069.4842691
SRR17520069.4842693
SRR17520069.4842695
SRR17520069.4842699
SRR17520069.4842701
SRR17520069.4842703

SRR17520069.4842705
SRR17520069.4842707
SRR17520069.4842709
SRR17520069.4842711
SRR17520069.4842713
SRR17520069.4842715
SRR17520069.4842717
SRR17520069.4842719
SRR17520069.4842721
SRR17520069.4842723
SRR17520069.4842725
SRR17520069.4842727
SRR17520069.4842729
SRR17520069.4842731
SRR17520069.4842733
SRR17520069.4842735
SRR17520069.4843939
SRR17520069.4846474
SRR17520069.4846476
SRR17520069.4846478
SRR17520069.4846480
SRR17520069.4846482
SRR17520069.4846484
SRR17520069.4846486
SRR17520069.4846488
SRR17520069.4846490
SRR17520069.4846492
SRR17520069.4846494
SRR17520069.4846496
SRR17520069.4846498
SRR17520069.4846500
SRR17520069.4846502
SRR17520069.4846504
SRR17520069.4846506
SRR17520069.4846508
SRR17520069.4846510
SRR17520069.4846887
SRR17520069.4846889
SRR17520069.4846891
SRR17520069.4846893
SRR17520069.4846897
SRR17520069.4846899
SRR17520069.4846901
SRR17520069.4846903
SRR17520069.4846905
SRR17520069.4846907
SRR17520069.4846909

SRR17520069.4846911
SRR17520069.4846913
SRR17520069.4846915
SRR17520069.4846917
SRR17520069.4846919
SRR17520069.4848344
SRR17520069.4848346
SRR17520069.4848348
SRR17520069.4848350
SRR17520069.4848352
SRR17520069.4848354
SRR17520069.4848356
SRR17520069.4848358
SRR17520069.4848360
SRR17520069.4848362
SRR17520069.4848364
SRR17520069.4848366
SRR17520069.4848368
SRR17520069.4848370
SRR17520069.4848372
SRR17520069.4848374
SRR17520069.4848378
SRR17520069.4848380
SRR17520069.4849319
SRR17520069.4849321
SRR17520069.4849323
SRR17520069.4849325
SRR17520069.4849327
SRR17520069.4849329
SRR17520069.4849331
SRR17520069.4849333
SRR17520069.4849335
SRR17520069.4849337
SRR17520069.4849339
SRR17520069.4849341
SRR17520069.4849343
SRR17520069.4849347
SRR17520069.4849349
SRR17520069.4849351
SRR17520069.4853890
SRR17520069.4853892
SRR17520069.4853894
SRR17520069.4853896
SRR17520069.4853898
SRR17520069.4853900
SRR17520069.4853902
SRR17520069.4853904

SRR17520069.4853906
SRR17520069.4853908
SRR17520069.4853910
SRR17520069.4853912
SRR17520069.4853914
SRR17520069.4853918
SRR17520069.4853920
SRR17520069.4853922
SRR17520069.4853924
SRR17520069.4853926
SRR17520069.4866760
SRR17520069.4866762
SRR17520069.4866764
SRR17520069.4866766
SRR17520069.4866768
SRR17520069.4866770
SRR17520069.4866772
SRR17520069.4866774
SRR17520069.4866776
SRR17520069.4866778
SRR17520069.4866780
SRR17520069.4866782
SRR17520069.4866784
SRR17520069.4866786
SRR17520069.4866788
SRR17520069.4866790
SRR17520069.4866792
SRR17520069.4866794
SRR17520069.4866796
SRR17520069.4866798
SRR17520069.4866800
SRR17520069.4866802
SRR17520069.4866804
SRR17520069.4866806
SRR17520069.4866808
SRR17520069.4866810
SRR17520069.4866812
SRR17520069.4866814
SRR17520069.4866816
SRR17520069.4866818
SRR17520069.4866820
SRR17520069.4866822
SRR17520069.4866824
SRR17520069.4866826
SRR17520069.4866828
SRR17520069.4866830
SRR17520069.4866832

SRR17520069.4866834
SRR17520069.4866836
SRR17520069.4866838
SRR17520069.4866840
SRR17520069.4866842
SRR17520069.4866844
SRR17520069.4866846
SRR17520069.4866848
SRR17520069.4866850
SRR17520069.4876693
SRR17520069.4876695
SRR17520069.4876697
SRR17520069.4876699
SRR17520069.4876701
SRR17520069.4876703
SRR17520069.4876705
SRR17520069.4876707
SRR17520069.4876709
SRR17520069.4876711
SRR17520069.4876713
SRR17520069.4876715
SRR17520069.4876717
SRR17520069.4876723
SRR17520069.4876725
SRR17520069.4876727
SRR17520069.4876729
SRR17520069.4876731
SRR17520069.4876735
SRR17520069.4876737
SRR17520069.4876739
SRR17520069.4876741
SRR17520069.4876743
SRR17520069.4876745
SRR17520069.4876747
SRR17520069.4876749
SRR17520069.4876751
SRR17520069.4876753
SRR17520069.4876755
SRR17520069.4876757
SRR17520069.4876759
SRR17520069.4876761
SRR17520069.4876763
SRR17520069.4876765
SRR17520069.4876767
SRR17520069.4876769
SRR17520069.4876771
SRR17520069.4876773

SRR17520069.4876775
SRR17520069.4876779
SRR17520069.4876818
SRR17520069.4876820
SRR17520069.4879437
SRR17520069.4879439
SRR17520069.4879441
SRR17520069.4879443
SRR17520069.4879445
SRR17520069.4879447
SRR17520069.4879449
SRR17520069.4879451
SRR17520069.4879453
SRR17520069.4879455
SRR17520069.4879457
SRR17520069.4879459
SRR17520069.4879461
SRR17520069.4879463
SRR17520069.4879465
SRR17520069.4879469
SRR17520069.4879471
SRR17520069.4879473
SRR17520069.4879475
SRR17520069.4879477
SRR17520069.4879706
SRR17520069.4879710
SRR17520069.4879712
SRR17520069.4879714
SRR17520069.4879716
SRR17520069.4879718
SRR17520069.4879720
SRR17520069.4879722
SRR17520069.4879724
SRR17520069.4879726
SRR17520069.4879728
SRR17520069.4879730
SRR17520069.4879732
SRR17520069.4879734
SRR17520069.4879736
SRR17520069.4879738
SRR17520069.4879740
SRR17520069.4879742
SRR17520069.4879744
SRR17520069.4879746
SRR17520069.4879748
SRR17520069.4879750
SRR17520069.4879752

SRR17520069.4879754
SRR17520069.4879756
SRR17520069.4879758
SRR17520069.4879760
SRR17520069.4879762
SRR17520069.4879764
SRR17520069.4879766
SRR17520069.4879770
SRR17520069.4879772
SRR17520069.4879774
SRR17520069.4879776
SRR17520069.4879778
SRR17520069.4879780
SRR17520069.4879782
SRR17520069.4879784
SRR17520069.4879786
SRR17520069.4879788
SRR17520069.4879790
SRR17520069.4879794
SRR17520069.4879796
SRR17520069.4879798
SRR17520069.4886076
SRR17520069.4886080
SRR17520069.4886082
SRR17520069.4886084
SRR17520069.4886086
SRR17520069.4886088
SRR17520069.4886090
SRR17520069.4886092
SRR17520069.4886094
SRR17520069.4886096
SRR17520069.4886098
SRR17520069.4886100
SRR17520069.4886102
SRR17520069.4903274
SRR17520069.4903276
SRR17520069.4903278
SRR17520069.4903280
SRR17520069.4903282
SRR17520069.4903284
SRR17520069.4903286
SRR17520069.4903288
SRR17520069.4904565
SRR17520069.4904567
SRR17520069.4904569
SRR17520069.4904571
SRR17520069.4904573

SRR17520069.4904575
SRR17520069.4904577
SRR17520069.4904579
SRR17520069.4904581
SRR17520069.4904583
SRR17520069.4904585
SRR17520069.4904587
SRR17520069.4904591
SRR17520069.4904593
SRR17520069.4904595
SRR17520069.4904597
SRR17520069.4904599
SRR17520069.4904601
SRR17520069.4904603
SRR17520069.4904605
SRR17520069.4904607
SRR17520069.4904609
SRR17520069.4904611
SRR17520069.4904613
SRR17520069.4904615
SRR17520069.4904617
SRR17520069.4904619
SRR17520069.4904621
SRR17520069.4904623
SRR17520069.4904625
SRR17520069.4904627
SRR17520069.4904629
SRR17520069.4904631
SRR17520069.4904633
SRR17520069.4904635
SRR17520069.4904637
SRR17520069.4904639
SRR17520069.4904643
SRR17520069.4904645
SRR17520069.4904647
SRR17520069.4904649
SRR17520069.4904651
SRR17520069.4904653
SRR17520069.4904657
SRR17520069.4904659
SRR17520069.4904661
SRR17520069.4904663
SRR17520069.4904665
SRR17520069.4904667
SRR17520069.4904669
SRR17520069.4904671
SRR17520069.4904673

SRR17520069.4904675
SRR17520069.4904677
SRR17520069.4904679
SRR17520069.4904681
SRR17520069.4904683
SRR17520069.4904685
SRR17520069.4904687
SRR17520069.4904689
SRR17520069.4904691
SRR17520069.4904693
SRR17520069.4904695
SRR17520069.4904697
SRR17520069.4904699
SRR17520069.4904701
SRR17520069.4904703
SRR17520069.4904705
SRR17520069.4904707
SRR17520069.4904709
SRR17520069.4904711
SRR17520069.4904713
SRR17520069.4904715
SRR17520069.4904717
SRR17520069.4904719
SRR17520069.4904721
SRR17520069.4904723
SRR17520069.4904725
SRR17520069.4904727
SRR17520069.4904729
SRR17520069.4904731
SRR17520069.4904733
SRR17520069.4904735
SRR17520069.4904737
SRR17520069.4904739
SRR17520069.4904741
SRR17520069.4904743
SRR17520069.4905678
SRR17520069.4905680
SRR17520069.4905682
SRR17520069.4905684
SRR17520069.4905686
SRR17520069.4913978
SRR17520069.4921604
SRR17520069.4921606
SRR17520069.4921608
SRR17520069.4921610
SRR17520069.4921612
SRR17520069.4921614

SRR17520069.4921618
SRR17520069.4921620
SRR17520069.4921624
SRR17520069.4921626
SRR17520069.4921628
SRR17520069.4921630
SRR17520069.4924383
SRR17520069.4924385
SRR17520069.4926848
SRR17520069.4926853
SRR17520069.4926855
SRR17520069.4930332
SRR17520069.4931232
SRR17520069.4931234
SRR17520069.4931236
SRR17520069.4931238
SRR17520069.4931242
SRR17520069.4931244
SRR17520069.4931246
SRR17520069.4931248
SRR17520069.4931250
SRR17520069.4931252
SRR17520069.4931256
SRR17520069.4931258
SRR17520069.4931260
SRR17520069.4931264
SRR17520069.4931268
SRR17520069.4931270
SRR17520069.4931272
SRR17520069.4931274
SRR17520069.4931276
SRR17520069.4931278
SRR17520069.4931280
SRR17520069.4931282
SRR17520069.4931284
SRR17520069.4931286
SRR17520069.4931288
SRR17520069.4931290
SRR17520069.4931292
SRR17520069.4931294
SRR17520069.4931296
SRR17520069.4931298
SRR17520069.4931300
SRR17520069.4931302
SRR17520069.4931304
SRR17520069.4931306
SRR17520069.4931313

SRR17520069.4931315
SRR17520069.4931317
SRR17520069.4935123
SRR17520069.4935125
SRR17520069.4935127
SRR17520069.4935129
SRR17520069.4935133
SRR17520069.4935135
SRR17520069.4935137
SRR17520069.4935139
SRR17520069.4935141
SRR17520069.4935143
SRR17520069.4935145
SRR17520069.4935147
SRR17520069.4935149
SRR17520069.4935151
SRR17520069.4935153
SRR17520069.4935155
SRR17520069.4935159
SRR17520069.4935161
SRR17520069.4935163
SRR17520069.4935165
SRR17520069.4935167
SRR17520069.4935169
SRR17520069.4935171
SRR17520069.4935173
SRR17520069.4935175
SRR17520069.4935177
SRR17520069.4935179
SRR17520069.4935181
SRR17520069.4935183
SRR17520069.4935185
SRR17520069.4935187
SRR17520069.4935189
SRR17520069.4935191
SRR17520069.4935193
SRR17520069.4935195
SRR17520069.4935197
SRR17520069.4935201
SRR17520069.4935203
SRR17520069.4935205
SRR17520069.4935207
SRR17520069.4935209
SRR17520069.4935211
SRR17520069.4935213
SRR17520069.4935217
SRR17520069.4935219

SRR17520069.4935221
SRR17520069.4943331
SRR17520069.4943333
SRR17520069.4943335
SRR17520069.4943337
SRR17520069.4943339
SRR17520069.4943341
SRR17520069.4943343
SRR17520069.4943345
SRR17520069.4943347
SRR17520069.4943349
SRR17520069.4943351
SRR17520069.4943353
SRR17520069.4943355
SRR17520069.4943357
SRR17520069.4943359
SRR17520069.4943361
SRR17520069.4943363
SRR17520069.4943365
SRR17520069.4943367
SRR17520069.4943369
SRR17520069.4943371
SRR17520069.4943373
SRR17520069.4943375
SRR17520069.4943377
SRR17520069.4943379
SRR17520069.4943381
SRR17520069.4943383
SRR17520069.4943385
SRR17520069.4943387
SRR17520069.4943389
SRR17520069.4943391
SRR17520069.4943393
SRR17520069.4949971
SRR17520069.4949979
SRR17520069.4952339
SRR17520069.4952341
SRR17520069.4952343
SRR17520069.4952345
SRR17520069.4952347
SRR17520069.4952349
SRR17520069.4952351
SRR17520069.4952353
SRR17520069.4952355
SRR17520069.4952357
SRR17520069.4952359
SRR17520069.4952361

SRR17520069.4952363
SRR17520069.4957858
SRR17520069.4957860
SRR17520069.4957862
SRR17520069.4957864
SRR17520069.4957866
SRR17520069.4957870
SRR17520069.4957872
SRR17520069.4957874
SRR17520069.4957876
SRR17520069.4957878
SRR17520069.4962922
SRR17520069.4962924
SRR17520069.4962926
SRR17520069.4962928
SRR17520069.4962930
SRR17520069.4962932
SRR17520069.4962934
SRR17520069.4967554
SRR17520069.4967558
SRR17520069.4971372
SRR17520069.4971374
SRR17520069.4971376
SRR17520069.4971378
SRR17520069.4971380
SRR17520069.4971382
SRR17520069.4971384
SRR17520069.4971386
SRR17520069.4971388
SRR17520069.4971390
SRR17520069.4971392
SRR17520069.4971394
SRR17520069.4971396
SRR17520069.4971398
SRR17520069.4971402
SRR17520069.4971404
SRR17520069.4971406
SRR17520069.4971408
SRR17520069.4971410
SRR17520069.4971412
SRR17520069.4971414
SRR17520069.4971416
SRR17520069.4971418
SRR17520069.4971420
SRR17520069.4971422
SRR17520069.4971424
SRR17520069.4971426

SRR17520069.4984287
SRR17520069.4986396
SRR17520069.4986400
SRR17520069.4986402
SRR17520069.4987376
SRR17520069.4988391
SRR17520069.4988393
SRR17520069.4988395
SRR17520069.4988397
SRR17520069.4988399
SRR17520069.4988401
SRR17520069.4988405
SRR17520069.4988407
SRR17520069.4988409
SRR17520069.4988411
SRR17520069.4988413
SRR17520069.4988415
SRR17520069.4988417
SRR17520069.4988419
SRR17520069.4988421
SRR17520069.4988425
SRR17520069.4988427
SRR17520069.4988429
SRR17520069.4988431
SRR17520069.4988435
SRR17520069.4988437
SRR17520069.4988439
SRR17520069.4988441
SRR17520069.4988443
SRR17520069.4988445
SRR17520069.4988449
SRR17520069.4988451
SRR17520069.4988453
SRR17520069.4988457
SRR17520069.4988459
SRR17520069.4988461
SRR17520069.4988463
SRR17520069.4988467
SRR17520069.4988469
SRR17520069.4988471
SRR17520069.4988473
SRR17520069.4988475
SRR17520069.4988477
SRR17520069.4988479
SRR17520069.4988481
SRR17520069.4988483
SRR17520069.4988485

SRR17520069.4988489
SRR17520069.4988491
SRR17520069.4988493
SRR17520069.4988497
SRR17520069.4988501
SRR17520069.4988503
SRR17520069.4988505
SRR17520069.4988507
SRR17520069.4993981
SRR17520069.4993983
SRR17520069.4993985
SRR17520069.4993987
SRR17520069.4993989
SRR17520069.4993991
SRR17520069.4993993
SRR17520069.4993995
SRR17520069.4993997
SRR17520069.4993999
SRR17520069.4994001
SRR17520069.4994003
SRR17520069.4994005
SRR17520069.4994007
SRR17520069.4994009
SRR17520069.4994011
SRR17520069.4994013
SRR17520069.4994015
SRR17520069.4994017
SRR17520069.4996506
SRR17520069.5000422
SRR17520069.5000466
SRR17520069.5004402
SRR17520069.5004404
SRR17520069.5004406
SRR17520069.5004410
SRR17520069.5004412
SRR17520069.5004414
SRR17520069.5004416
SRR17520069.5006644
SRR17520069.5011924
SRR17520069.5011926
SRR17520069.5011928
SRR17520069.5011930
SRR17520069.5011932
SRR17520069.5011934
SRR17520069.5011936
SRR17520069.5011938
SRR17520069.5011940

SRR17520069.5011942
SRR17520069.5011944
SRR17520069.5011946
SRR17520069.5011948
SRR17520069.5011950
SRR17520069.5011952
SRR17520069.5011954
SRR17520069.5011956
SRR17520069.5011958
SRR17520069.5011960
SRR17520069.5011962
SRR17520069.5011964
SRR17520069.5011966
SRR17520069.5011968
SRR17520069.5011970
SRR17520069.5011972
SRR17520069.5011974
SRR17520069.5011976
SRR17520069.5011978
SRR17520069.5014728
SRR17520069.5014730
SRR17520069.5014732
SRR17520069.5014734
SRR17520069.5014736
SRR17520069.5014738
SRR17520069.5014740
SRR17520069.5014742
SRR17520069.5014744
SRR17520069.5014746
SRR17520069.5014750
SRR17520069.5014752
SRR17520069.5014754
SRR17520069.5014756
SRR17520069.5014758
SRR17520069.5014760
SRR17520069.5014762
SRR17520069.5014854
SRR17520069.5014856
SRR17520069.5014858
SRR17520069.5014860
SRR17520069.5016473
SRR17520069.5016475
SRR17520069.5016477
SRR17520069.5016479
SRR17520069.5016481
SRR17520069.5016483
SRR17520069.5016485

SRR17520069.5016487
SRR17520069.5016489
SRR17520069.5016491
SRR17520069.5016493
SRR17520069.5016495
SRR17520069.5016497
SRR17520069.5016499
SRR17520069.5016501
SRR17520069.5016503
SRR17520069.5016505
SRR17520069.5016507
SRR17520069.5016509
SRR17520069.5016511
SRR17520069.5016513
SRR17520069.5016515
SRR17520069.5016517
SRR17520069.5016519
SRR17520069.5016521
SRR17520069.5016523
SRR17520069.5016525
SRR17520069.5016527
SRR17520069.5016529
SRR17520069.5016531
SRR17520069.5016533
SRR17520069.5016535
SRR17520069.5016537
SRR17520069.5016539
SRR17520069.5016541
SRR17520069.5016543
SRR17520069.5016545
SRR17520069.5016547
SRR17520069.5016549
SRR17520069.5016551
SRR17520069.5016555
SRR17520069.5016561
SRR17520069.5016563
SRR17520069.5016565
SRR17520069.5016567
SRR17520069.5016569
SRR17520069.5016571
SRR17520069.5016573
SRR17520069.5016575
SRR17520069.5016577
SRR17520069.5020487
SRR17520069.5020489
SRR17520069.5020491
SRR17520069.5020493

SRR17520069.5020495
SRR17520069.5020497
SRR17520069.5020501
SRR17520069.5020503
SRR17520069.5020505
SRR17520069.5020507
SRR17520069.5026890
SRR17520069.5026892
SRR17520069.5026894
SRR17520069.5026896
SRR17520069.5026898
SRR17520069.5031757
SRR17520069.5031759
SRR17520069.5031761
SRR17520069.5031763
SRR17520069.5031765
SRR17520069.5032188
SRR17520069.5032190
SRR17520069.5039438
SRR17520069.5045072
SRR17520069.5045074
SRR17520069.5045076
SRR17520069.5045078
SRR17520069.5045080
SRR17520069.5045084
SRR17520069.5045086
SRR17520069.5045088
SRR17520069.5045090
SRR17520069.5045092
SRR17520069.5045096
SRR17520069.5045098
SRR17520069.5045100
SRR17520069.5045102
SRR17520069.5045108
SRR17520069.5045110
SRR17520069.5045112
SRR17520069.5045118
SRR17520069.5045120
SRR17520069.5045122
SRR17520069.5045124
SRR17520069.5045126
SRR17520069.5049699
SRR17520069.5051040
SRR17520069.5052439
SRR17520069.5052441
SRR17520069.5052443
SRR17520069.5052445

SRR17520069.5052447
SRR17520069.5052449
SRR17520069.5052453
SRR17520069.5052455
SRR17520069.5052457
SRR17520069.5052459
SRR17520069.5052461
SRR17520069.5052463
SRR17520069.5052465
SRR17520069.5052467
SRR17520069.5052469
SRR17520069.5052471
SRR17520069.5071510
SRR17520069.5071512
SRR17520069.5071514
SRR17520069.5071518
SRR17520069.5071520
SRR17520069.5072838
SRR17520069.5072842
SRR17520069.5072844
SRR17520069.5072846
SRR17520069.5072848
SRR17520069.5072850
SRR17520069.5072852
SRR17520069.5072854
SRR17520069.5072856
SRR17520069.5072860
SRR17520069.5072862
SRR17520069.5072864
SRR17520069.5072866
SRR17520069.5072868
SRR17520069.5072872
SRR17520069.5072874
SRR17520069.5072876
SRR17520069.5072878
SRR17520069.5072884
SRR17520069.5072886
SRR17520069.5072888
SRR17520069.5072890
SRR17520069.5072898
SRR17520069.5072900
SRR17520069.5072904
SRR17520069.5074987
SRR17520069.5074989
SRR17520069.5074991
SRR17520069.5074993
SRR17520069.5074995

SRR17520069.5074997
SRR17520069.5074999
SRR17520069.5075001
SRR17520069.5075003
SRR17520069.5075005
SRR17520069.5075007
SRR17520069.5075009
SRR17520069.5075011
SRR17520069.5075013
SRR17520069.5075015
SRR17520069.5075017
SRR17520069.5075019
SRR17520069.5075021
SRR17520069.5075023
SRR17520069.5075025
SRR17520069.5075027
SRR17520069.5075029
SRR17520069.5075031
SRR17520069.5075033
SRR17520069.5075037
SRR17520069.5075039
SRR17520069.5075041
SRR17520069.5075043
SRR17520069.5075045
SRR17520069.5075047
SRR17520069.5075049
SRR17520069.5075051
SRR17520069.5075053
SRR17520069.5075055
SRR17520069.5075057
SRR17520069.5075059
SRR17520069.5075061
SRR17520069.5075065
SRR17520069.5075067
SRR17520069.5075069
SRR17520069.5075071
SRR17520069.5075073
SRR17520069.5075075
SRR17520069.5075077
SRR17520069.5075079
SRR17520069.5075081
SRR17520069.5075085
SRR17520069.5075087
SRR17520069.5075089
SRR17520069.5075091
SRR17520069.5075093
SRR17520069.5075095

SRR17520069.5075097
SRR17520069.5080428
SRR17520069.5080430
SRR17520069.5080432
SRR17520069.5080434
SRR17520069.5080436
SRR17520069.5080438
SRR17520069.5080440
SRR17520069.5080442
SRR17520069.5083324
SRR17520069.5083326
SRR17520069.5083332
SRR17520069.5083336
SRR17520069.5083460
SRR17520069.5083462
SRR17520069.5083464
SRR17520069.5083466
SRR17520069.5083468
SRR17520069.5083470
SRR17520069.5083472
SRR17520069.5083474
SRR17520069.5083476
SRR17520069.5083478
SRR17520069.5083480
SRR17520069.5083482
SRR17520069.5083484
SRR17520069.5083486
SRR17520069.5083488
SRR17520069.5083490
SRR17520069.5083492
SRR17520069.5083494
SRR17520069.5083496
SRR17520069.5085420
SRR17520069.5085424
SRR17520069.5085426
SRR17520069.5085428
SRR17520069.5085430
SRR17520069.5085432
SRR17520069.5085434
SRR17520069.5085436
SRR17520069.5085438
SRR17520069.5085440
SRR17520069.5085442
SRR17520069.5085444
SRR17520069.5085446
SRR17520069.5085448
SRR17520069.5085450

SRR17520069.5085452
SRR17520069.5085454
SRR17520069.5085456
SRR17520069.5085458
SRR17520069.5085460
SRR17520069.5085462
SRR17520069.5085464
SRR17520069.5085466
SRR17520069.5085468
SRR17520069.5085470
SRR17520069.5085472
SRR17520069.5085474
SRR17520069.5085476
SRR17520069.5085478
SRR17520069.5085480
SRR17520069.5085482
SRR17520069.5085484
SRR17520069.5085486
SRR17520069.5085488
SRR17520069.5085490
SRR17520069.5085492
SRR17520069.5087786
SRR17520069.5087789
SRR17520069.5094854
SRR17520069.5094856
SRR17520069.5094858
SRR17520069.5094860
SRR17520069.5094862
SRR17520069.5094864
SRR17520069.5094866
SRR17520069.5094868
SRR17520069.5094870
SRR17520069.5094872
SRR17520069.5094874
SRR17520069.5094876
SRR17520069.5094878
SRR17520069.5094880
SRR17520069.5094882
SRR17520069.5094884
SRR17520069.5094886
SRR17520069.5094888
SRR17520069.5094890
SRR17520069.5094892
SRR17520069.5094894
SRR17520069.5094896
SRR17520069.5094898
SRR17520069.5094900

SRR17520069.5094902
SRR17520069.5094904
SRR17520069.5094906
SRR17520069.5094908
SRR17520069.5094910
SRR17520069.5094912
SRR17520069.5094914
SRR17520069.5094916
SRR17520069.5094918
SRR17520069.5094920
SRR17520069.5094922
SRR17520069.5094924
SRR17520069.5094926
SRR17520069.5094928
SRR17520069.5094930
SRR17520069.5094932
SRR17520069.5094934
SRR17520069.5094936
SRR17520069.5094938
SRR17520069.5094940
SRR17520069.5094942
SRR17520069.5094944
SRR17520069.5094946
SRR17520069.5095382
SRR17520069.5095384
SRR17520069.5095386
SRR17520069.5095388
SRR17520069.5098113
SRR17520069.5098115
SRR17520069.5098117
SRR17520069.5098119
SRR17520069.5098123
SRR17520069.5098125
SRR17520069.5098127
SRR17520069.5098129
SRR17520069.5098131
SRR17520069.5098133
SRR17520069.5098135
SRR17520069.5100602
SRR17520069.5100604
SRR17520069.5100606
SRR17520069.5100608
SRR17520069.5100610
SRR17520069.5100612
SRR17520069.5100614
SRR17520069.5100616
SRR17520069.5100618

SRR17520069.5100622
SRR17520069.5100624
SRR17520069.5100626
SRR17520069.5100628
SRR17520069.5100630
SRR17520069.5100632
SRR17520069.5100634
SRR17520069.5100636
SRR17520069.5100642
SRR17520069.5100644
SRR17520069.5100646
SRR17520069.5100648
SRR17520069.5100652
SRR17520069.5100654
SRR17520069.5100656
SRR17520069.5100658
SRR17520069.5100660
SRR17520069.5100662
SRR17520069.5100664
SRR17520069.5100666
SRR17520069.5100668
SRR17520069.5100670
SRR17520069.5100672
SRR17520069.5100674
SRR17520069.5100676
SRR17520069.5100678
SRR17520069.5100680
SRR17520069.5100682
SRR17520069.5100684
SRR17520069.5100686
SRR17520069.5100688
SRR17520069.5100690
SRR17520069.5100692
SRR17520069.5100694
SRR17520069.5100696
SRR17520069.5100698
SRR17520069.5100700
SRR17520069.5100706
SRR17520069.5101471
SRR17520069.5101473
SRR17520069.5101475
SRR17520069.5101477
SRR17520069.5101479
SRR17520069.5101483
SRR17520069.5101485
SRR17520069.5101487
SRR17520069.5101490

SRR17520069.5101492
SRR17520069.5101494
SRR17520069.5101496
SRR17520069.5101498
SRR17520069.5101500
SRR17520069.5101502
SRR17520069.5101504
SRR17520069.5101508
SRR17520069.5104971
SRR17520069.5104973
SRR17520069.5104975
SRR17520069.5104979
SRR17520069.5104981
SRR17520069.5104983
SRR17520069.5104985
SRR17520069.5104987
SRR17520069.5104989
SRR17520069.5104991
SRR17520069.5104993
SRR17520069.5104995
SRR17520069.5104997
SRR17520069.5104999
SRR17520069.5105001
SRR17520069.5112814
SRR17520069.5112816
SRR17520069.5112820
SRR17520069.5112822
SRR17520069.5112824
SRR17520069.5112826
SRR17520069.5112828
SRR17520069.5112830
SRR17520069.5112832
SRR17520069.5112834
SRR17520069.5112836
SRR17520069.5112838
SRR17520069.5112840
SRR17520069.5112842
SRR17520069.5112844
SRR17520069.5112846
SRR17520069.5112848
SRR17520069.5112850
SRR17520069.5112852
SRR17520069.5112854
SRR17520069.5112856
SRR17520069.5112858
SRR17520069.5112860
SRR17520069.5112862

SRR17520069.5112864
SRR17520069.5112868
SRR17520069.5112870
SRR17520069.5112872
SRR17520069.5112874
SRR17520069.5112876
SRR17520069.5112878
SRR17520069.5112880
SRR17520069.5112882
SRR17520069.5112884
SRR17520069.5112886
SRR17520069.5112888
SRR17520069.5112894
SRR17520069.5112896
SRR17520069.5112898
SRR17520069.5112900
SRR17520069.5112902
SRR17520069.5112904
SRR17520069.5112906
SRR17520069.5112908
SRR17520069.5112910
SRR17520069.5112912
SRR17520069.5112916
SRR17520069.5112918
SRR17520069.5112920
SRR17520069.5112922
SRR17520069.5112924
SRR17520069.5112926
SRR17520069.5112928
SRR17520069.5112930
SRR17520069.5112932
SRR17520069.5112934
SRR17520069.5112936
SRR17520069.5112938
SRR17520069.5112940
SRR17520069.5112942
SRR17520069.5112946
SRR17520069.5112948
SRR17520069.5112950
SRR17520069.5112952
SRR17520069.5112954
SRR17520069.5112956
SRR17520069.5112958
SRR17520069.5112960
SRR17520069.5112962
SRR17520069.5112964
SRR17520069.5112966

SRR17520069.5112968
SRR17520069.5112970
SRR17520069.5112974
SRR17520069.5112976
SRR17520069.5112978
SRR17520069.5112980
SRR17520069.5112982
SRR17520069.5114237
SRR17520069.5114239
SRR17520069.5114241
SRR17520069.5114243
SRR17520069.5114245
SRR17520069.5114247
SRR17520069.5114249
SRR17520069.5114251
SRR17520069.5114253
SRR17520069.5114255
SRR17520069.5114257
SRR17520069.5114259
SRR17520069.5114261
SRR17520069.5114263
SRR17520069.5114265
SRR17520069.5114267
SRR17520069.5114272
SRR17520069.5114274
SRR17520069.5114276
SRR17520069.5115169
SRR17520069.5115171
SRR17520069.5115173
SRR17520069.5115175
SRR17520069.5116358
SRR17520069.5116362
SRR17520069.5116364
SRR17520069.5116366
SRR17520069.5116368
SRR17520069.5116370
SRR17520069.5116372
SRR17520069.5116374
SRR17520069.5116376
SRR17520069.5116378
SRR17520069.5116380
SRR17520069.5116382
SRR17520069.5116384
SRR17520069.5118613
SRR17520069.5118615
SRR17520069.5118617
SRR17520069.5118619

SRR17520069.5118623
SRR17520069.5118625
SRR17520069.5118627
SRR17520069.5118629
SRR17520069.5118631
SRR17520069.5118633
SRR17520069.5118635
SRR17520069.5118637
SRR17520069.5118639
SRR17520069.5118641
SRR17520069.5118643
SRR17520069.5118645
SRR17520069.5118647
SRR17520069.5118649
SRR17520069.5118651
SRR17520069.5118655
SRR17520069.5118657
SRR17520069.5118659
SRR17520069.5118661
SRR17520069.5118663
SRR17520069.5118665
SRR17520069.5118667
SRR17520069.5118669
SRR17520069.5118671
SRR17520069.5118673
SRR17520069.5118675
SRR17520069.5118679
SRR17520069.5119095
SRR17520069.5119097
SRR17520069.5119099
SRR17520069.5119101
SRR17520069.5119103
SRR17520069.5119105
SRR17520069.5119107
SRR17520069.5119109
SRR17520069.5119111
SRR17520069.5119113
SRR17520069.5125826
SRR17520069.5125828
SRR17520069.5125830
SRR17520069.5125832
SRR17520069.5126124
SRR17520069.5126130
SRR17520069.5126694
SRR17520069.5126696
SRR17520069.5126698
SRR17520069.5126700

SRR17520069.5126702
SRR17520069.5126704
SRR17520069.5126706
SRR17520069.5126708
SRR17520069.5126710
SRR17520069.5126712
SRR17520069.5126714
SRR17520069.5127380
SRR17520069.5132287
SRR17520069.5132289
SRR17520069.5132291
SRR17520069.5132293
SRR17520069.5132297
SRR17520069.5132301
SRR17520069.5132303
SRR17520069.5132305
SRR17520069.5132307
SRR17520069.5132309
SRR17520069.5132311
SRR17520069.5132315
SRR17520069.5132317
SRR17520069.5132323
SRR17520069.5132325
SRR17520069.5132327
SRR17520069.5132329
SRR17520069.5132331
SRR17520069.5132333
SRR17520069.5132337
SRR17520069.5132341
SRR17520069.5133738
SRR17520069.5133740
SRR17520069.5133742
SRR17520069.5133744
SRR17520069.5133746
SRR17520069.5133748
SRR17520069.5154498
SRR17520069.5154500
SRR17520069.5154502
SRR17520069.5154504
SRR17520069.5154506
SRR17520069.5154508
SRR17520069.5154510
SRR17520069.5154512
SRR17520069.5154514
SRR17520069.5154516
SRR17520069.5154518
SRR17520069.5154520

SRR17520069.5154522
SRR17520069.5154524
SRR17520069.5154526
SRR17520069.5154528
SRR17520069.5154530
SRR17520069.5154532
SRR17520069.5154534
SRR17520069.5154536
SRR17520069.5154538
SRR17520069.5154540
SRR17520069.5154542
SRR17520069.5154544
SRR17520069.5154548
SRR17520069.5154550
SRR17520069.5156729
SRR17520069.5156731
SRR17520069.5156733
SRR17520069.5156735
SRR17520069.5156737
SRR17520069.5156739
SRR17520069.5156741
SRR17520069.5156743
SRR17520069.5156745
SRR17520069.5156747
SRR17520069.5156749
SRR17520069.5156751
SRR17520069.5156753
SRR17520069.5156755
SRR17520069.5156757
SRR17520069.5156759
SRR17520069.5156761
SRR17520069.5156763
SRR17520069.5156765
SRR17520069.5156767
SRR17520069.5156769
SRR17520069.5156771
SRR17520069.5156773
SRR17520069.5156775
SRR17520069.5156777
SRR17520069.5156779
SRR17520069.5156781
SRR17520069.5156783
SRR17520069.5156785
SRR17520069.5156787
SRR17520069.5156789
SRR17520069.5156791
SRR17520069.5156793

SRR17520069.5156795
SRR17520069.5156797
SRR17520069.5156799
SRR17520069.5156801
SRR17520069.5156803
SRR17520069.5156805
SRR17520069.5156807
SRR17520069.5156809
SRR17520069.5156811
SRR17520069.5156813
SRR17520069.5156815
SRR17520069.5156817
SRR17520069.5156819
SRR17520069.5156821
SRR17520069.5156823
SRR17520069.5156825
SRR17520069.5156827
SRR17520069.5156829
SRR17520069.5156831
SRR17520069.5160794
SRR17520069.5160796
SRR17520069.5160798
SRR17520069.5160800
SRR17520069.5160802
SRR17520069.5160804
SRR17520069.5160806
SRR17520069.5160808
SRR17520069.5160810
SRR17520069.5160812
SRR17520069.5160814
SRR17520069.5160816
SRR17520069.5160818
SRR17520069.5160820
SRR17520069.5160822
SRR17520069.5160824
SRR17520069.5160826
SRR17520069.5160828
SRR17520069.5160830
SRR17520069.5160832
SRR17520069.5160834
SRR17520069.5160836
SRR17520069.5160838
SRR17520069.5160840
SRR17520069.5160842
SRR17520069.5160844
SRR17520069.5160846
SRR17520069.5160848

SRR17520069.5160850
SRR17520069.5160852
SRR17520069.5160854
SRR17520069.5160856
SRR17520069.5173104
SRR17520069.5173106
SRR17520069.5173108
SRR17520069.5173110
SRR17520069.5173112
SRR17520069.5173114
SRR17520069.5173116
SRR17520069.5173118
SRR17520069.5173120
SRR17520069.5175680
SRR17520069.5175682
SRR17520069.5175684
SRR17520069.5175686
SRR17520069.5175688
SRR17520069.5175690
SRR17520069.5175692
SRR17520069.5175694
SRR17520069.5175696
SRR17520069.5175698
SRR17520069.5175700
SRR17520069.5175704
SRR17520069.5175706
SRR17520069.5175708
SRR17520069.5175710
SRR17520069.5175712
SRR17520069.5175714
SRR17520069.5175716
SRR17520069.5175718
SRR17520069.5175720
SRR17520069.5175722
SRR17520069.5175724
SRR17520069.5175726
SRR17520069.5175728
SRR17520069.5175730
SRR17520069.5175732
SRR17520069.5175734
SRR17520069.5175736
SRR17520069.5175738
SRR17520069.5175740
SRR17520069.5175742
SRR17520069.5175744
SRR17520069.5175746
SRR17520069.5175748

SRR17520069.5175750
SRR17520069.5175752
SRR17520069.5175754
SRR17520069.5175756
SRR17520069.5175758
SRR17520069.5175760
SRR17520069.5175762
SRR17520069.5175764
SRR17520069.5175766
SRR17520069.5175768
SRR17520069.5175770
SRR17520069.5175772
SRR17520069.5175774
SRR17520069.5175776
SRR17520069.5175778
SRR17520069.5175780
SRR17520069.5175782
SRR17520069.5175784
SRR17520069.5175786
SRR17520069.5175788
SRR17520069.5175790
SRR17520069.5175792
SRR17520069.5175794
SRR17520069.5175796
SRR17520069.5175798
SRR17520069.5175800
SRR17520069.5175802
SRR17520069.5175804
SRR17520069.5175806
SRR17520069.5175808
SRR17520069.5175810
SRR17520069.5175812
SRR17520069.5175814
SRR17520069.5176083
SRR17520069.5176111
SRR17520069.5176135
SRR17520069.5176456
SRR17520069.5177582
SRR17520069.5177584
SRR17520069.5177586
SRR17520069.5177588
SRR17520069.5177590
SRR17520069.5177592
SRR17520069.5177594
SRR17520069.5177596
SRR17520069.5177598
SRR17520069.5177600

SRR17520069.5177602
SRR17520069.5177604
SRR17520069.5177606
SRR17520069.5177608
SRR17520069.5177610
SRR17520069.5177612
SRR17520069.5177614
SRR17520069.5177616
SRR17520069.5177618
SRR17520069.5177620
SRR17520069.5177622
SRR17520069.5177624
SRR17520069.5177626
SRR17520069.5177628
SRR17520069.5177630
SRR17520069.5177632
SRR17520069.5177634
SRR17520069.5177636
SRR17520069.5177638
SRR17520069.5177640
SRR17520069.5177642
SRR17520069.5177644
SRR17520069.5177646
SRR17520069.5177648
SRR17520069.5177650
SRR17520069.5184228
SRR17520069.5184230
SRR17520069.5184232
SRR17520069.5184234
SRR17520069.5184236
SRR17520069.5184238
SRR17520069.5184240
SRR17520069.5184242
SRR17520069.5184244
SRR17520069.5184246
SRR17520069.5184248
SRR17520069.5184250
SRR17520069.5184252
SRR17520069.5184254
SRR17520069.5184256
SRR17520069.5184258
SRR17520069.5184260
SRR17520069.5184262
SRR17520069.5184264
SRR17520069.5184266
SRR17520069.5184268
SRR17520069.5184270

SRR17520069.5184272
SRR17520069.5184274
SRR17520069.5184276
SRR17520069.5184278
SRR17520069.5184280
SRR17520069.5184282
SRR17520069.5184284
SRR17520069.5184286
SRR17520069.5184288
SRR17520069.5184290
SRR17520069.5184292
SRR17520069.5184294
SRR17520069.5184296
SRR17520069.5184298
SRR17520069.5184300
SRR17520069.5184302
SRR17520069.5184304
SRR17520069.5184306
SRR17520069.5184308
SRR17520069.5184310
SRR17520069.5184312
SRR17520069.5184314
SRR17520069.5184316
SRR17520069.5184318
SRR17520069.5184320
SRR17520069.5184322
SRR17520069.5184324
SRR17520069.5184326
SRR17520069.5184328
SRR17520069.5184330
SRR17520069.5184332
SRR17520069.5194687
SRR17520069.5194689
SRR17520069.5194691
SRR17520069.5194693
SRR17520069.5194695
SRR17520069.5194697
SRR17520069.5194699
SRR17520069.5194701
SRR17520069.5194703
SRR17520069.5194705
SRR17520069.5194707
SRR17520069.5194709
SRR17520069.5199391
SRR17520069.5199393
SRR17520069.5199395
SRR17520069.5199397

SRR17520069.5199399
SRR17520069.5199401
SRR17520069.5199403
SRR17520069.5199405
SRR17520069.5199407
SRR17520069.5199409
SRR17520069.5199411
SRR17520069.5199413
SRR17520069.5199415
SRR17520069.5199417
SRR17520069.5199419
SRR17520069.5199421
SRR17520069.5199423
SRR17520069.5199425
SRR17520069.5199427
SRR17520069.5199429
SRR17520069.5199431
SRR17520069.5199433
SRR17520069.5199435
SRR17520069.5199437
SRR17520069.5199439
SRR17520069.5199441
SRR17520069.5199443
SRR17520069.5199445
SRR17520069.5199447
SRR17520069.5199449
SRR17520069.5199451
SRR17520069.5200091
SRR17520069.5200139
SRR17520069.5204911
SRR17520069.5204913
SRR17520069.5204915
SRR17520069.5204917
SRR17520069.5204919
SRR17520069.5204921
SRR17520069.5204923
SRR17520069.5204925
SRR17520069.5204927
SRR17520069.5204929
SRR17520069.5204931
SRR17520069.5204933
SRR17520069.5204935
SRR17520069.5204937
SRR17520069.5204939
SRR17520069.5204941
SRR17520069.5204943
SRR17520069.5204945

SRR17520069.5204947
SRR17520069.5204949
SRR17520069.5204951
SRR17520069.5204953
SRR17520069.5204955
SRR17520069.5204957
SRR17520069.5204959
SRR17520069.5204961
SRR17520069.5204963
SRR17520069.5204965
SRR17520069.5204967
SRR17520069.5204969
SRR17520069.5204971
SRR17520069.5204973
SRR17520069.5204975
SRR17520069.5204977
SRR17520069.5204979
SRR17520069.5204981
SRR17520069.5204983
SRR17520069.5204985
SRR17520069.5204987
SRR17520069.5207344
SRR17520069.5207346
SRR17520069.5207348
SRR17520069.5207350
SRR17520069.5207352
SRR17520069.5207354
SRR17520069.5207356
SRR17520069.5207358
SRR17520069.5207360
SRR17520069.5207362
SRR17520069.5207364
SRR17520069.5207366
SRR17520069.5207368
SRR17520069.5207370
SRR17520069.5207372
SRR17520069.5207374
SRR17520069.5207376
SRR17520069.5207378
SRR17520069.5207380
SRR17520069.5207382
SRR17520069.5207384
SRR17520069.5207386
SRR17520069.5207388
SRR17520069.5207390
SRR17520069.5207392
SRR17520069.5207394

SRR17520069.5207396
SRR17520069.5207398
SRR17520069.5207400
SRR17520069.5207402
SRR17520069.5207404
SRR17520069.5207406
SRR17520069.5207408
SRR17520069.5207410
SRR17520069.5207412
SRR17520069.5207414
SRR17520069.5214044
SRR17520069.5219526
SRR17520069.5219528
SRR17520069.5219530
SRR17520069.5219532
SRR17520069.5219534
SRR17520069.5219538
SRR17520069.5219540
SRR17520069.5219542
SRR17520069.5219544
SRR17520069.5219546
SRR17520069.5219548
SRR17520069.5219550
SRR17520069.5219552
SRR17520069.5219554
SRR17520069.5219556
SRR17520069.5219560
SRR17520069.5219562
SRR17520069.5219564
SRR17520069.5219566
SRR17520069.5219568
SRR17520069.5219570
SRR17520069.5219572
SRR17520069.5219574
SRR17520069.5219578
SRR17520069.5219580
SRR17520069.5219582
SRR17520069.5219584
SRR17520069.5219586
SRR17520069.5219590
SRR17520069.5219592
SRR17520069.5219594
SRR17520069.5219596
SRR17520069.5219598
SRR17520069.5219600
SRR17520069.5219602
SRR17520069.5219604

SRR17520069.5219606
SRR17520069.5219608
SRR17520069.5219610
SRR17520069.5219612
SRR17520069.5219614
SRR17520069.5219616
SRR17520069.5219618
SRR17520069.5219620
SRR17520069.5219622
SRR17520069.5219624
SRR17520069.5219626
SRR17520069.5219628
SRR17520069.5219630
SRR17520069.5219632
SRR17520069.5219634
SRR17520069.5219636
SRR17520069.5219638
SRR17520069.5219640
SRR17520069.5219642
SRR17520069.5219644
SRR17520069.5219646
SRR17520069.5219648
SRR17520069.5219650
SRR17520069.5219652
SRR17520069.5219654
SRR17520069.5219658
SRR17520069.5219660
SRR17520069.5219662
SRR17520069.5219664
SRR17520069.5219666
SRR17520069.5219668
SRR17520069.5219672
SRR17520069.5219676
SRR17520069.5219678
SRR17520069.5219680
SRR17520069.5219682
SRR17520069.5219684
SRR17520069.5219686
SRR17520069.5219688
SRR17520069.5219690
SRR17520069.5219692
SRR17520069.5219694
SRR17520069.5219897
SRR17520069.5219899
SRR17520069.5219901
SRR17520069.5219903
SRR17520069.5219905

SRR17520069.5219907
SRR17520069.5219909
SRR17520069.5219911
SRR17520069.5219913
SRR17520069.5219917
SRR17520069.5219921
SRR17520069.5219923
SRR17520069.5219925
SRR17520069.5219927
SRR17520069.5219929
SRR17520069.5219931
SRR17520069.5219933
SRR17520069.5219935
SRR17520069.5219937
SRR17520069.5219939
SRR17520069.5219941
SRR17520069.5219943
SRR17520069.5219945
SRR17520069.5219947
SRR17520069.5219949
SRR17520069.5219951
SRR17520069.5219953
SRR17520069.5219955
SRR17520069.5219957
SRR17520069.5219959
SRR17520069.5219961
SRR17520069.5219963
SRR17520069.5219965
SRR17520069.5223097
SRR17520069.5223099
SRR17520069.5223101
SRR17520069.5223103
SRR17520069.5223105
SRR17520069.5223107
SRR17520069.5223109
SRR17520069.5223111
SRR17520069.5223113
SRR17520069.5223115
SRR17520069.5223117
SRR17520069.5223119
SRR17520069.5223121
SRR17520069.5223123
SRR17520069.5223125
SRR17520069.5223127
SRR17520069.5223129
SRR17520069.5223131
SRR17520069.5223133

SRR17520069.5223135
SRR17520069.5223137
SRR17520069.5223139
SRR17520069.5223141
SRR17520069.5223278
SRR17520069.5223280
SRR17520069.5223282
SRR17520069.5223286
SRR17520069.5223288
SRR17520069.5223290
SRR17520069.5223292
SRR17520069.5223294
SRR17520069.5223296
SRR17520069.5223298
SRR17520069.5223300
SRR17520069.5223302
SRR17520069.5223304
SRR17520069.5223306
SRR17520069.5223308
SRR17520069.5223310
SRR17520069.5223312
SRR17520069.5223314
SRR17520069.5223316
SRR17520069.5223318
SRR17520069.5223320
SRR17520069.5223322
SRR17520069.5225577
SRR17520069.5225581
SRR17520069.5225583
SRR17520069.5225585
SRR17520069.5225587
SRR17520069.5225589
SRR17520069.5225591
SRR17520069.5225593
SRR17520069.5225595
SRR17520069.5225597
SRR17520069.5233287
SRR17520069.5233289
SRR17520069.5233291
SRR17520069.5233293
SRR17520069.5233295
SRR17520069.5233297
SRR17520069.5233299
SRR17520069.5233301
SRR17520069.5233303
SRR17520069.5233305
SRR17520069.5233307

SRR17520069.5233309
SRR17520069.5233311
SRR17520069.5233313
SRR17520069.5233315
SRR17520069.5233317
SRR17520069.5233319
SRR17520069.5233916
SRR17520069.5233918
SRR17520069.5233920
SRR17520069.5233922
SRR17520069.5233924
SRR17520069.5233926
SRR17520069.5233928
SRR17520069.5233930
SRR17520069.5233932
SRR17520069.5233934
SRR17520069.5233936
SRR17520069.5233938
SRR17520069.5233940
SRR17520069.5233942
SRR17520069.5233944
SRR17520069.5233946
SRR17520069.5233948
SRR17520069.5233950
SRR17520069.5233952
SRR17520069.5233954
SRR17520069.5233956
SRR17520069.5233958
SRR17520069.5233960
SRR17520069.5233962
SRR17520069.5233964
SRR17520069.5233966
SRR17520069.5233968
SRR17520069.5233970
SRR17520069.5233972
SRR17520069.5238818
SRR17520069.5238822
SRR17520069.5238824
SRR17520069.5238826
SRR17520069.5238828
SRR17520069.5238830
SRR17520069.5240545
SRR17520069.5240547
SRR17520069.5240549
SRR17520069.5240551
SRR17520069.5240553
SRR17520069.5240555

SRR17520069.5240557
SRR17520069.5240559
SRR17520069.5240561
SRR17520069.5240567
SRR17520069.5240569
SRR17520069.5240571
SRR17520069.5240573
SRR17520069.5240577
SRR17520069.5240579
SRR17520069.5240581
SRR17520069.5240583
SRR17520069.5240585
SRR17520069.5240587
SRR17520069.5240591
SRR17520069.5240593
SRR17520069.5240595
SRR17520069.5242350
SRR17520069.5242352
SRR17520069.5242354
SRR17520069.5243410
SRR17520069.5243412
SRR17520069.5243414
SRR17520069.5243416
SRR17520069.5243418
SRR17520069.5243420
SRR17520069.5243422
SRR17520069.5243424
SRR17520069.5243426
SRR17520069.5243428
SRR17520069.5243430
SRR17520069.5243434
SRR17520069.5243438
SRR17520069.5243440
SRR17520069.5243442
SRR17520069.5243444
SRR17520069.5243446
SRR17520069.5243448
SRR17520069.5243450
SRR17520069.5243454
SRR17520069.5243456
SRR17520069.5243458
SRR17520069.5243460
SRR17520069.5243462
SRR17520069.5243464
SRR17520069.5243466
SRR17520069.5243470
SRR17520069.5243472

SRR17520069.5243474
SRR17520069.5243476
SRR17520069.5243478
SRR17520069.5243480
SRR17520069.5243482
SRR17520069.5243484
SRR17520069.5243488
SRR17520069.5243490
SRR17520069.5243492
SRR17520069.5243494
SRR17520069.5243498
SRR17520069.5243500
SRR17520069.5243502
SRR17520069.5243506
SRR17520069.5243508
SRR17520069.5243510
SRR17520069.5243512
SRR17520069.5243514
SRR17520069.5243516
SRR17520069.5243518
SRR17520069.5243520
SRR17520069.5243522
SRR17520069.5243524
SRR17520069.5243526
SRR17520069.5243528
SRR17520069.5243530
SRR17520069.5243532
SRR17520069.5243534
SRR17520069.5243536
SRR17520069.5243538
SRR17520069.5243540
SRR17520069.5248803
SRR17520069.5249672
SRR17520069.5249674
SRR17520069.5249676
SRR17520069.5249678
SRR17520069.5249680
SRR17520069.5249682
SRR17520069.5249684
SRR17520069.5249686
SRR17520069.5249688
SRR17520069.5249690
SRR17520069.5249692
SRR17520069.5249694
SRR17520069.5249696
SRR17520069.5249698
SRR17520069.5249700

SRR17520069.5249702
SRR17520069.5249704
SRR17520069.5249708
SRR17520069.5249710
SRR17520069.5249712
SRR17520069.5249714
SRR17520069.5249716
SRR17520069.5249718
SRR17520069.5249720
SRR17520069.5249722
SRR17520069.5249724
SRR17520069.5249726
SRR17520069.5249728
SRR17520069.5249730
SRR17520069.5249732
SRR17520069.5249734
SRR17520069.5249736
SRR17520069.5249738
SRR17520069.5249740
SRR17520069.5249742
SRR17520069.5249744
SRR17520069.5249746
SRR17520069.5249748
SRR17520069.5249750
SRR17520069.5249752
SRR17520069.5249754
SRR17520069.5249756
SRR17520069.5249758
SRR17520069.5249760
SRR17520069.5249762
SRR17520069.5249764
SRR17520069.5249766
SRR17520069.5249768
SRR17520069.5249770
SRR17520069.5249772
SRR17520069.5249774
SRR17520069.5249778
SRR17520069.5249780
SRR17520069.5249782
SRR17520069.5249784
SRR17520069.5249786
SRR17520069.5249788
SRR17520069.5249790
SRR17520069.5249792
SRR17520069.5249794
SRR17520069.5249796
SRR17520069.5249798

SRR17520069.5255626
SRR17520069.5255628
SRR17520069.5255630
SRR17520069.5255634
SRR17520069.5255636
SRR17520069.5255638
SRR17520069.5255640
SRR17520069.5255642
SRR17520069.5255644
SRR17520069.5255646
SRR17520069.5255648
SRR17520069.5255650
SRR17520069.5255652
SRR17520069.5255654
SRR17520069.5255656
SRR17520069.5255658
SRR17520069.5255660
SRR17520069.5255662
SRR17520069.5255664
SRR17520069.5270985
SRR17520069.5270987
SRR17520069.5270989
SRR17520069.5270991
SRR17520069.5270993
SRR17520069.5270997
SRR17520069.5270999
SRR17520069.5271001
SRR17520069.5271003
SRR17520069.5271005
SRR17520069.5271007
SRR17520069.5271009
SRR17520069.5271011
SRR17520069.5271013
SRR17520069.5271015
SRR17520069.5271017
SRR17520069.5271021
SRR17520069.5271023
SRR17520069.5271025
SRR17520069.5271027
SRR17520069.5271029
SRR17520069.5271031
SRR17520069.5271033
SRR17520069.5271035
SRR17520069.5271037
SRR17520069.5271039
SRR17520069.5271043
SRR17520069.5271045

SRR17520069.5271049
SRR17520069.5271051
SRR17520069.5271053
SRR17520069.5271057
SRR17520069.5271061
SRR17520069.5271063
SRR17520069.5276314
SRR17520069.5276326
SRR17520069.5276342
SRR17520069.5276344
SRR17520069.5276348
SRR17520069.5276352
SRR17520069.5276354
SRR17520069.5276356
SRR17520069.5276360
SRR17520069.5276362
SRR17520069.5276374
SRR17520069.5281086
SRR17520069.5281088
SRR17520069.5281090
SRR17520069.5281092
SRR17520069.5286175
SRR17520069.5286179
SRR17520069.5286181
SRR17520069.5286183
SRR17520069.5286185
SRR17520069.5286187
SRR17520069.5286189
SRR17520069.5286191
SRR17520069.5286193
SRR17520069.5286195
SRR17520069.5286197
SRR17520069.5286199
SRR17520069.5297902
SRR17520069.5297904
SRR17520069.5297908
SRR17520069.5303080
SRR17520069.5303082
SRR17520069.5304724
SRR17520069.5304726
SRR17520069.5304730
SRR17520069.5304732
SRR17520069.5304734
SRR17520069.5304736
SRR17520069.5304738
SRR17520069.5304740
SRR17520069.5304742

SRR17520069.5304744
SRR17520069.5304746
SRR17520069.5305199
SRR17520069.5313090
SRR17520069.5313092
SRR17520069.5313094
SRR17520069.5313425
SRR17520069.5313429
SRR17520069.5313467
SRR17520069.5316912
SRR17520069.5316914
SRR17520069.5316916
SRR17520069.5316922
SRR17520069.5316924
SRR17520069.5316930
SRR17520069.5316932
SRR17520069.5316936
SRR17520069.5316938
SRR17520069.5316940
SRR17520069.5316942
SRR17520069.5316944
SRR17520069.5316946
SRR17520069.5316948
SRR17520069.5316952
SRR17520069.5316954
SRR17520069.5321506
SRR17520069.5321508
SRR17520069.5321510
SRR17520069.5321512
SRR17520069.5321514
SRR17520069.5321516
SRR17520069.5321518
SRR17520069.5321520
SRR17520069.5321522
SRR17520069.5321524
SRR17520069.5321526
SRR17520069.5321528
SRR17520069.5321530
SRR17520069.5321532
SRR17520069.5321534
SRR17520069.5321536
SRR17520069.5321540
SRR17520069.5321542
SRR17520069.5321546
SRR17520069.5321548
SRR17520069.5321550
SRR17520069.5321554

SRR17520069.5323003
SRR17520069.5323005
SRR17520069.5323007
SRR17520069.5323009
SRR17520069.5323011
SRR17520069.5323013
SRR17520069.5323015
SRR17520069.5323017
SRR17520069.5323019
SRR17520069.5323023
SRR17520069.5323025
SRR17520069.5323027
SRR17520069.5323029
SRR17520069.5323031
SRR17520069.5323033
SRR17520069.5323035
SRR17520069.5323037
SRR17520069.5323039
SRR17520069.5323043
SRR17520069.5323045
SRR17520069.5323047
SRR17520069.5323049
SRR17520069.5323051
SRR17520069.5323053
SRR17520069.5323057
SRR17520069.5323059
SRR17520069.5323061
SRR17520069.5323063
SRR17520069.5323065
SRR17520069.5323067
SRR17520069.5323069
SRR17520069.5323071
SRR17520069.5323073
SRR17520069.5323075
SRR17520069.5323077
SRR17520069.5323079
SRR17520069.5323081
SRR17520069.5323083
SRR17520069.5324142
SRR17520069.5324144
SRR17520069.5324146
SRR17520069.5324148
SRR17520069.5324150
SRR17520069.5324152
SRR17520069.5324154
SRR17520069.5324228
SRR17520069.5324230

SRR17520069.5324232
SRR17520069.5324234
SRR17520069.5324236
SRR17520069.5324238
SRR17520069.5324240
SRR17520069.5324242
SRR17520069.5324244
SRR17520069.5324246
SRR17520069.5324248
SRR17520069.5324250
SRR17520069.5324252
SRR17520069.5324254
SRR17520069.5324256
SRR17520069.5324258
SRR17520069.5324260
SRR17520069.5324262
SRR17520069.5324264
SRR17520069.5324266
SRR17520069.5324268
SRR17520069.5324270
SRR17520069.5324272
SRR17520069.5324274
SRR17520069.5324276
SRR17520069.5324278
SRR17520069.5324280
SRR17520069.5324282
SRR17520069.5324284
SRR17520069.5324286
SRR17520069.5324288
SRR17520069.5324292
SRR17520069.5324294
SRR17520069.5324296
SRR17520069.5324298
SRR17520069.5324300
SRR17520069.5324302
SRR17520069.5324304
SRR17520069.5324306
SRR17520069.5324308
SRR17520069.5331039
SRR17520069.5331041
SRR17520069.5331043
SRR17520069.5331045
SRR17520069.5331047
SRR17520069.5331049
SRR17520069.5331051
SRR17520069.5331053
SRR17520069.5331055

SRR17520069.5331057
SRR17520069.5331059
SRR17520069.5331061
SRR17520069.5331063
SRR17520069.5331065
SRR17520069.5331067
SRR17520069.5331069
SRR17520069.5331071
SRR17520069.5331209
SRR17520069.5331211
SRR17520069.5331213
SRR17520069.5331215
SRR17520069.5331217
SRR17520069.5331219
SRR17520069.5331221
SRR17520069.5331223
SRR17520069.5331225
SRR17520069.5331227
SRR17520069.5331229
SRR17520069.5331231
SRR17520069.5331233
SRR17520069.5331235
SRR17520069.5331237
SRR17520069.5331239
SRR17520069.5331241
SRR17520069.5331243
SRR17520069.5331245
SRR17520069.5331247
SRR17520069.5331249
SRR17520069.5331251
SRR17520069.5331253
SRR17520069.5331255
SRR17520069.5331257
SRR17520069.5331259
SRR17520069.5331261
SRR17520069.5331263
SRR17520069.5331265
SRR17520069.5331267
SRR17520069.5331269
SRR17520069.5331271
SRR17520069.5331273
SRR17520069.5331275
SRR17520069.5331277
SRR17520069.5331279
SRR17520069.5331281
SRR17520069.5331283
SRR17520069.5331285

SRR17520069.5331287
SRR17520069.5331289
SRR17520069.5331291
SRR17520069.5331293
SRR17520069.5331295
SRR17520069.5331297
SRR17520069.5331301
SRR17520069.5331303
SRR17520069.5331305
SRR17520069.5331307
SRR17520069.5331309
SRR17520069.5331311
SRR17520069.5331313
SRR17520069.5331315
SRR17520069.5331317
SRR17520069.5331319
SRR17520069.5340382
SRR17520069.5340384
SRR17520069.5349152
SRR17520069.5349154
SRR17520069.5351231
SRR17520069.5351233
SRR17520069.5351235
SRR17520069.5351237
SRR17520069.5351239
SRR17520069.5351243
SRR17520069.5351245
SRR17520069.5351247
SRR17520069.5351249
SRR17520069.5351251
SRR17520069.5351253
SRR17520069.5351336
SRR17520069.5351338
SRR17520069.5351340
SRR17520069.5351342
SRR17520069.5351344
SRR17520069.5351346
SRR17520069.5351348
SRR17520069.5351350
SRR17520069.5351352
SRR17520069.5351354
SRR17520069.5351356
SRR17520069.5351358
SRR17520069.5351360
SRR17520069.5351364
SRR17520069.5351366
SRR17520069.5351368

SRR17520069.5351370
SRR17520069.5351372
SRR17520069.5351374
SRR17520069.5351376
SRR17520069.5351378
SRR17520069.5351380
SRR17520069.5351382
SRR17520069.5351384
SRR17520069.5351386
SRR17520069.5351388
SRR17520069.5351390
SRR17520069.5351392
SRR17520069.5351394
SRR17520069.5351396
SRR17520069.5351398
SRR17520069.5351400
SRR17520069.5351402
SRR17520069.5351404
SRR17520069.5351406
SRR17520069.5351408
SRR17520069.5351410
SRR17520069.5351412
SRR17520069.5351414
SRR17520069.5351416
SRR17520069.5351418
SRR17520069.5351420
SRR17520069.5351422
SRR17520069.5351424
SRR17520069.5351426
SRR17520069.5351428
SRR17520069.5351430
SRR17520069.5351432
SRR17520069.5351434
SRR17520069.5351436
SRR17520069.5351438
SRR17520069.5351440
SRR17520069.5351442
SRR17520069.5351444
SRR17520069.5351446
SRR17520069.5351448
SRR17520069.5351450
SRR17520069.5351452
SRR17520069.5351454
SRR17520069.5351456
SRR17520069.5351458
SRR17520069.5351518
SRR17520069.5351520

SRR17520069.5351522
SRR17520069.5351526
SRR17520069.5351528
SRR17520069.5351530
SRR17520069.5351532
SRR17520069.5351534
SRR17520069.5351536
SRR17520069.5351538
SRR17520069.5351540
SRR17520069.5351542
SRR17520069.5351544
SRR17520069.5351546
SRR17520069.5351548
SRR17520069.5351550
SRR17520069.5351552
SRR17520069.5351554
SRR17520069.5351556
SRR17520069.5351558
SRR17520069.5351560
SRR17520069.5351562
SRR17520069.5351564
SRR17520069.5351566
SRR17520069.5351568
SRR17520069.5351570
SRR17520069.5351572
SRR17520069.5351574
SRR17520069.5351576
SRR17520069.5351578
SRR17520069.5351580
SRR17520069.5351582
SRR17520069.5351584
SRR17520069.5351586
SRR17520069.5351588
SRR17520069.5351590
SRR17520069.5351592
SRR17520069.5351594
SRR17520069.5351596
SRR17520069.5351598
SRR17520069.5351600
SRR17520069.5351602
SRR17520069.5351604
SRR17520069.5351606
SRR17520069.5351608
SRR17520069.5351610
SRR17520069.5351612
SRR17520069.5351614
SRR17520069.5351616

SRR17520069.5351618
SRR17520069.5353161
SRR17520069.5353163
SRR17520069.5353165
SRR17520069.5353169
SRR17520069.5353171
SRR17520069.5353173
SRR17520069.5353175
SRR17520069.5353177
SRR17520069.5353179
SRR17520069.5353181
SRR17520069.5353183
SRR17520069.5353185
SRR17520069.5353187
SRR17520069.5353189
SRR17520069.5353193
SRR17520069.5353195
SRR17520069.5353197
SRR17520069.5353199
SRR17520069.5353201
SRR17520069.5353203
SRR17520069.5353207
SRR17520069.5353209
SRR17520069.5356255
SRR17520069.5356257
SRR17520069.5356259
SRR17520069.5356261
SRR17520069.5356263
SRR17520069.5356265
SRR17520069.5356267
SRR17520069.5356269
SRR17520069.5356271
SRR17520069.5356273
SRR17520069.5356275
SRR17520069.5356277
SRR17520069.5356279
SRR17520069.5356281
SRR17520069.5356283
SRR17520069.5356959
SRR17520069.5356963
SRR17520069.5356965
SRR17520069.5356967
SRR17520069.5356969
SRR17520069.5356971
SRR17520069.5356975
SRR17520069.5356977
SRR17520069.5356979

SRR17520069.5356981
SRR17520069.5356983
SRR17520069.5356985
SRR17520069.5356987
SRR17520069.5356995
SRR17520069.5356997
SRR17520069.5356999
SRR17520069.5357001
SRR17520069.5357003
SRR17520069.5357005
SRR17520069.5357007
SRR17520069.5357009
SRR17520069.5357011
SRR17520069.5357013
SRR17520069.5357015
SRR17520069.5358396
SRR17520069.5358398
SRR17520069.5358400
SRR17520069.5358402
SRR17520069.5358404
SRR17520069.5358406
SRR17520069.5358408
SRR17520069.5358410
SRR17520069.5358412
SRR17520069.5358414
SRR17520069.5358416
SRR17520069.5358418
SRR17520069.5358420
SRR17520069.5358422
SRR17520069.5358424
SRR17520069.5358426
SRR17520069.5358428
SRR17520069.5358430
SRR17520069.5358432
SRR17520069.5358434
SRR17520069.5358436
SRR17520069.5358438
SRR17520069.5358440
SRR17520069.5358442
SRR17520069.5358444
SRR17520069.5358446
SRR17520069.5358448
SRR17520069.5358450
SRR17520069.5358452
SRR17520069.5358454
SRR17520069.5358456
SRR17520069.5358458

SRR17520069.5358460
SRR17520069.5358462
SRR17520069.5358464
SRR17520069.5358466
SRR17520069.5358468
SRR17520069.5358470
SRR17520069.5358472
SRR17520069.5358474
SRR17520069.5358476
SRR17520069.5358478
SRR17520069.5358480
SRR17520069.5358484
SRR17520069.5358486
SRR17520069.5358488
SRR17520069.5358490
SRR17520069.5358492
SRR17520069.5358494
SRR17520069.5358496
SRR17520069.5358498
SRR17520069.5358500
SRR17520069.5358502
SRR17520069.5358504
SRR17520069.5358506
SRR17520069.5358508
SRR17520069.5358510
SRR17520069.5358512
SRR17520069.5358514
SRR17520069.5366300
SRR17520069.5366302
SRR17520069.5366304
SRR17520069.5366306
SRR17520069.5366308
SRR17520069.5366310
SRR17520069.5366312
SRR17520069.5366314
SRR17520069.5366316
SRR17520069.5366318
SRR17520069.5366320
SRR17520069.5366322
SRR17520069.5366324
SRR17520069.5366326
SRR17520069.5366328
SRR17520069.5366330
SRR17520069.5366332
SRR17520069.5379282
SRR17520069.5379284
SRR17520069.5379286

SRR17520069.5379288
SRR17520069.5379295
SRR17520069.5379297
SRR17520069.5379299
SRR17520069.5379303
SRR17520069.5379305
SRR17520069.5379307
SRR17520069.5379309
SRR17520069.5379311
SRR17520069.5379313
SRR17520069.5379315
SRR17520069.5379317
SRR17520069.5379319
SRR17520069.5379321
SRR17520069.5379552
SRR17520069.5379556
SRR17520069.5379558
SRR17520069.5379560
SRR17520069.5379562
SRR17520069.5379564
SRR17520069.5379566
SRR17520069.5379568
SRR17520069.5379570
SRR17520069.5379572
SRR17520069.5379574
SRR17520069.5379576
SRR17520069.5379578
SRR17520069.5379580
SRR17520069.5379582
SRR17520069.5379584
SRR17520069.5379586
SRR17520069.5379588
SRR17520069.5379590
SRR17520069.5379592
SRR17520069.5379594
SRR17520069.5385214
SRR17520069.5398541
SRR17520069.5398543
SRR17520069.5398545
SRR17520069.5398547
SRR17520069.5398549
SRR17520069.5398551
SRR17520069.5398553
SRR17520069.5398555
SRR17520069.5398557
SRR17520069.5398559
SRR17520069.5398561

SRR17520069.5398563
SRR17520069.5398565
SRR17520069.5398567
SRR17520069.5398569
SRR17520069.5398573
SRR17520069.5398575
SRR17520069.5398577
SRR17520069.5398579
SRR17520069.5398581
SRR17520069.5398583
SRR17520069.5398585
SRR17520069.5398587
SRR17520069.5402873
SRR17520069.5402875
SRR17520069.5402877
SRR17520069.5402879
SRR17520069.5402881
SRR17520069.5402883
SRR17520069.5402885
SRR17520069.5402887
SRR17520069.5402889
SRR17520069.5402891
SRR17520069.5402893
SRR17520069.5402895
SRR17520069.5402897
SRR17520069.5402899
SRR17520069.5402901
SRR17520069.5402903
SRR17520069.5402905
SRR17520069.5402907
SRR17520069.5402909
SRR17520069.5402911
SRR17520069.5402913
SRR17520069.5402915
SRR17520069.5402917
SRR17520069.5402919
SRR17520069.5402921
SRR17520069.5402923
SRR17520069.5402925
SRR17520069.5402927
SRR17520069.5402931
SRR17520069.5402933
SRR17520069.5402935
SRR17520069.5402937
SRR17520069.5402939
SRR17520069.5402941
SRR17520069.5402943

SRR17520069.5402945
SRR17520069.5402947
SRR17520069.5402949
SRR17520069.5402951
SRR17520069.5402953
SRR17520069.5414001
SRR17520069.5414005
SRR17520069.5414007
SRR17520069.5414009
SRR17520069.5414949
SRR17520069.5414951
SRR17520069.5414953
SRR17520069.5424103
SRR17520069.5424115
SRR17520069.5424117
SRR17520069.5424119
SRR17520069.5424121
SRR17520069.5424123
SRR17520069.5424125
SRR17520069.5424127
SRR17520069.5424129
SRR17520069.5424131
SRR17520069.5424133
SRR17520069.5424135
SRR17520069.5424137
SRR17520069.5424139
SRR17520069.5424141
SRR17520069.5424143
SRR17520069.5424145
SRR17520069.5424147
SRR17520069.5424149
SRR17520069.5424151
SRR17520069.5424153
SRR17520069.5424155
SRR17520069.5424157
SRR17520069.5424159
SRR17520069.5424161
SRR17520069.5424163
SRR17520069.5424165
SRR17520069.5424167
SRR17520069.5424169
SRR17520069.5424171
SRR17520069.5424173
SRR17520069.5424175
SRR17520069.5424177
SRR17520069.5424189
SRR17520069.5424191

SRR17520069.5424193
SRR17520069.5424195
SRR17520069.5424197
SRR17520069.5424199
SRR17520069.5424201
SRR17520069.5424203
SRR17520069.5424205
SRR17520069.5424207
SRR17520069.5424209
SRR17520069.5424211
SRR17520069.5424215
SRR17520069.5424217
SRR17520069.5424219
SRR17520069.5424221
SRR17520069.5424223
SRR17520069.5424225
SRR17520069.5424227
SRR17520069.5424229
SRR17520069.5424231
SRR17520069.5424233
SRR17520069.5424235
SRR17520069.5424237
SRR17520069.5424239
SRR17520069.5424241
SRR17520069.5424243
SRR17520069.5424245
SRR17520069.5424247
SRR17520069.5424249
SRR17520069.5424251
SRR17520069.5424253
SRR17520069.5424255
SRR17520069.5424257
SRR17520069.5424259
SRR17520069.5424261
SRR17520069.5424263
SRR17520069.5424265
SRR17520069.5424267
SRR17520069.5424269
SRR17520069.5424271
SRR17520069.5424273
SRR17520069.5424275
SRR17520069.5424277
SRR17520069.5424279
SRR17520069.5424281
SRR17520069.5424283
SRR17520069.5424285
SRR17520069.5424289

SRR17520069.5424291
SRR17520069.5424293
SRR17520069.5424295
SRR17520069.5424297
SRR17520069.5424299
SRR17520069.5424301
SRR17520069.5424303
SRR17520069.5424305
SRR17520069.5424307
SRR17520069.5424309
SRR17520069.5424311
SRR17520069.5424313
SRR17520069.5424315
SRR17520069.5424317
SRR17520069.5424319
SRR17520069.5424321
SRR17520069.5424323
SRR17520069.5424325
SRR17520069.5424327
SRR17520069.5424329
SRR17520069.5424331
SRR17520069.5424333
SRR17520069.5424335
SRR17520069.5424337
SRR17520069.5424339
SRR17520069.5424341
SRR17520069.5424343
SRR17520069.5424345
SRR17520069.5424347
SRR17520069.5424349
SRR17520069.5424351
SRR17520069.5424353
SRR17520069.5424355
SRR17520069.5433630
SRR17520069.5433632
SRR17520069.5433634
SRR17520069.5433636
SRR17520069.5438814
SRR17520069.5438816
SRR17520069.5438818
SRR17520069.5438820
SRR17520069.5438822
SRR17520069.5438824
SRR17520069.5438826
SRR17520069.5438828
SRR17520069.5438830
SRR17520069.5438832

SRR17520069.5438834
SRR17520069.5438836
SRR17520069.5438838
SRR17520069.5438840
SRR17520069.5442533
SRR17520069.5442686
SRR17520069.5442688
SRR17520069.5442690
SRR17520069.5442692
SRR17520069.5442694
SRR17520069.5442696
SRR17520069.5442698
SRR17520069.5442700
SRR17520069.5442702
SRR17520069.5442704
SRR17520069.5442706
SRR17520069.5442708
SRR17520069.5442710
SRR17520069.5442712
SRR17520069.5442716
SRR17520069.5442718
SRR17520069.5442720
SRR17520069.5442724
SRR17520069.5442728
SRR17520069.5442730
SRR17520069.5442732
SRR17520069.5446836
SRR17520069.5446838
SRR17520069.5452794
SRR17520069.5457682
SRR17520069.5457712
SRR17520069.5460121
SRR17520069.5460143
SRR17520069.5460188
SRR17520069.5460190
SRR17520069.5460192
SRR17520069.5460194
SRR17520069.5460196
SRR17520069.5460200
SRR17520069.5460204
SRR17520069.5460206
SRR17520069.5460208
SRR17520069.5460210
SRR17520069.5460212
SRR17520069.5460214
SRR17520069.5460218
SRR17520069.5460220

SRR17520069.5460222
SRR17520069.5460224
SRR17520069.5460226
SRR17520069.5460228
SRR17520069.5460230
SRR17520069.5460232
SRR17520069.5460234
SRR17520069.5460236
SRR17520069.5460238
SRR17520069.5460240
SRR17520069.5460242
SRR17520069.5460244
SRR17520069.5460246
SRR17520069.5460248
SRR17520069.5460250
SRR17520069.5460252
SRR17520069.5460254
SRR17520069.5460256
SRR17520069.5460258
SRR17520069.5460260
SRR17520069.5460262
SRR17520069.5460264
SRR17520069.5460266
SRR17520069.5460268
SRR17520069.5460270
SRR17520069.5460272
SRR17520069.5460274
SRR17520069.5460276
SRR17520069.5460278
SRR17520069.5460280
SRR17520069.5460282
SRR17520069.5460284
SRR17520069.5460286
SRR17520069.5460288
SRR17520069.5460290
SRR17520069.5460292
SRR17520069.5460294
SRR17520069.5460296
SRR17520069.5460298
SRR17520069.5460300
SRR17520069.5460302
SRR17520069.5460304
SRR17520069.5460306
SRR17520069.5460308
SRR17520069.5460310
SRR17520069.5460312
SRR17520069.5460314

SRR17520069.5460316
SRR17520069.5460318
SRR17520069.5460320
SRR17520069.5460322
SRR17520069.5460324
SRR17520069.5460326
SRR17520069.5466018
SRR17520069.5466020
SRR17520069.5466022
SRR17520069.5466024
SRR17520069.5466026
SRR17520069.5466028
SRR17520069.5466030
SRR17520069.5466032
SRR17520069.5466034
SRR17520069.5466038
SRR17520069.5466040
SRR17520069.5466042
SRR17520069.5466044
SRR17520069.5466046
SRR17520069.5466048
SRR17520069.5466050
SRR17520069.5466052
SRR17520069.5469884
SRR17520069.5469886
SRR17520069.5469888
SRR17520069.5469890
SRR17520069.5469892
SRR17520069.5469894
SRR17520069.5469896
SRR17520069.5469898
SRR17520069.5469900
SRR17520069.5469902
SRR17520069.5469904
SRR17520069.5469906
SRR17520069.5469908
SRR17520069.5469912
SRR17520069.5469914
SRR17520069.5469916
SRR17520069.5469918
SRR17520069.5469920
SRR17520069.5469924
SRR17520069.5469926
SRR17520069.5469928
SRR17520069.5469930
SRR17520069.5469932
SRR17520069.5469934

SRR17520069.5469936
SRR17520069.5469938
SRR17520069.5469940
SRR17520069.5469944
SRR17520069.5469946
SRR17520069.5469948
SRR17520069.5469950
SRR17520069.5469954
SRR17520069.5469956
SRR17520069.5469958
SRR17520069.5469960
SRR17520069.5469962
SRR17520069.5469964
SRR17520069.5469966
SRR17520069.5469968
SRR17520069.5469970
SRR17520069.5469974
SRR17520069.5469976
SRR17520069.5469978
SRR17520069.5469980
SRR17520069.5469982
SRR17520069.5474054
SRR17520069.5489476
SRR17520069.5489480
SRR17520069.5489482
SRR17520069.5489484
SRR17520069.5489486
SRR17520069.5489488
SRR17520069.5489490
SRR17520069.5489492
SRR17520069.5489494
SRR17520069.5489496
SRR17520069.5489498
SRR17520069.5489500
SRR17520069.5489502
SRR17520069.5489504
SRR17520069.5489506
SRR17520069.5489508
SRR17520069.5489510
SRR17520069.5489512
SRR17520069.5489516
SRR17520069.5489518
SRR17520069.5489520
SRR17520069.5490821
SRR17520069.5490823
SRR17520069.5490829
SRR17520069.5490831

SRR17520069.5490833
SRR17520069.5490835
SRR17520069.5490837
SRR17520069.5490839
SRR17520069.5490843
SRR17520069.5490847
SRR17520069.5490849
SRR17520069.5490851
SRR17520069.5490853
SRR17520069.5490855
SRR17520069.5490857
SRR17520069.5490859
SRR17520069.5490861
SRR17520069.5490863
SRR17520069.5490865
SRR17520069.5490867
SRR17520069.5490869
SRR17520069.5490871
SRR17520069.5490873
SRR17520069.5490875
SRR17520069.5490877
SRR17520069.5490879
SRR17520069.5490881
SRR17520069.5490885
SRR17520069.5490891
SRR17520069.5496365
SRR17520069.5496367
SRR17520069.5496375
SRR17520069.5496377
SRR17520069.5496381
SRR17520069.5496383
SRR17520069.5496385
SRR17520069.5496387
SRR17520069.5496389
SRR17520069.5496391
SRR17520069.5496393
SRR17520069.5496395
SRR17520069.5496397
SRR17520069.5496399
SRR17520069.5496401
SRR17520069.5496403
SRR17520069.5496405
SRR17520069.5496407
SRR17520069.5496409
SRR17520069.5496411
SRR17520069.5496413
SRR17520069.5496415

SRR17520069.5496417
SRR17520069.5496419
SRR17520069.5496421
SRR17520069.5496423
SRR17520069.5496427
SRR17520069.5496429
SRR17520069.5496431
SRR17520069.5496433
SRR17520069.5496435
SRR17520069.5496437
SRR17520069.5496439
SRR17520069.5496441
SRR17520069.5496443
SRR17520069.5496447
SRR17520069.5496449
SRR17520069.5496451
SRR17520069.5496453
SRR17520069.5496455
SRR17520069.5496457
SRR17520069.5496459
SRR17520069.5496461
SRR17520069.5496463
SRR17520069.5496465
SRR17520069.5496467
SRR17520069.5496469
SRR17520069.5496471
SRR17520069.5496473
SRR17520069.5496475
SRR17520069.5496477
SRR17520069.5496479
SRR17520069.5496481
SRR17520069.5496483
SRR17520069.5496487
SRR17520069.5496489
SRR17520069.5496491
SRR17520069.5496493
SRR17520069.5496497
SRR17520069.5496499
SRR17520069.5504265
SRR17520069.5504267
SRR17520069.5504269
SRR17520069.5504271
SRR17520069.5504273
SRR17520069.5504275
SRR17520069.5504277
SRR17520069.5504279
SRR17520069.5504281

SRR17520069.5504283
SRR17520069.5504285
SRR17520069.5504287
SRR17520069.5504289
SRR17520069.5504291
SRR17520069.5504295
SRR17520069.5504299
SRR17520069.5504301
SRR17520069.5504305
SRR17520069.5506190
SRR17520069.5506192
SRR17520069.5506196
SRR17520069.5506198
SRR17520069.5506200
SRR17520069.5506202
SRR17520069.5509865
SRR17520069.5509867
SRR17520069.5509869
SRR17520069.5509871
SRR17520069.5509873
SRR17520069.5509875
SRR17520069.5509877
SRR17520069.5509879
SRR17520069.5509881
SRR17520069.5509883
SRR17520069.5509885
SRR17520069.5509887
SRR17520069.5509889
SRR17520069.5509891
SRR17520069.5509893
SRR17520069.5509895
SRR17520069.5509899
SRR17520069.5509903
SRR17520069.5509905
SRR17520069.5509907
SRR17520069.5509909
SRR17520069.5509911
SRR17520069.5509913
SRR17520069.5509915
SRR17520069.5509917
SRR17520069.5509919
SRR17520069.5509921
SRR17520069.5509923
SRR17520069.5509925
SRR17520069.5509927
SRR17520069.5509929
SRR17520069.5509931

SRR17520069.5509933
SRR17520069.5509935
SRR17520069.5509937
SRR17520069.5509939
SRR17520069.5509941
SRR17520069.5509943
SRR17520069.5509945
SRR17520069.5509947
SRR17520069.5509949
SRR17520069.5509951
SRR17520069.5509953
SRR17520069.5509955
SRR17520069.5509957
SRR17520069.5509959
SRR17520069.5509961
SRR17520069.5509963
SRR17520069.5517484
SRR17520069.5517488
SRR17520069.5517490
SRR17520069.5517492
SRR17520069.5517494
SRR17520069.5517496
SRR17520069.5517498
SRR17520069.5517500
SRR17520069.5520671
SRR17520069.5520693
SRR17520069.5521411
SRR17520069.5521413
SRR17520069.5521415
SRR17520069.5521417
SRR17520069.5521419
SRR17520069.5521421
SRR17520069.5521423
SRR17520069.5521971
SRR17520069.5521973
SRR17520069.5521975
SRR17520069.5521977
SRR17520069.5521979
SRR17520069.5521981
SRR17520069.5521983
SRR17520069.5521985
SRR17520069.5521987
SRR17520069.5521989
SRR17520069.5521991
SRR17520069.5521993
SRR17520069.5521995
SRR17520069.5521997

SRR17520069.5524342
SRR17520069.5524344
SRR17520069.5524346
SRR17520069.5524348
SRR17520069.5524350
SRR17520069.5524352
SRR17520069.5524354
SRR17520069.5524356
SRR17520069.5524358
SRR17520069.5524360
SRR17520069.5524362
SRR17520069.5524535
SRR17520069.5524537
SRR17520069.5524539
SRR17520069.5524541
SRR17520069.5524543
SRR17520069.5524545
SRR17520069.5524547
SRR17520069.5524549
SRR17520069.5528434
SRR17520069.5530152
SRR17520069.5530154
SRR17520069.5530156
SRR17520069.5530158
SRR17520069.5530160
SRR17520069.5530162
SRR17520069.5530164
SRR17520069.5530166
SRR17520069.5530168
SRR17520069.5530170
SRR17520069.5530172
SRR17520069.5530174
SRR17520069.5530178
SRR17520069.5530180
SRR17520069.5530182
SRR17520069.5530184
SRR17520069.5530188
SRR17520069.5530190
SRR17520069.5530192
SRR17520069.5530194
SRR17520069.5530198
SRR17520069.5530200
SRR17520069.5530202
SRR17520069.5530208
SRR17520069.5530212
SRR17520069.5530214
SRR17520069.5530216

SRR17520069.5530218
SRR17520069.5530220
SRR17520069.5530222
SRR17520069.5530224
SRR17520069.5530226
SRR17520069.5530228
SRR17520069.5530230
SRR17520069.5531812
SRR17520069.5531814
SRR17520069.5531816
SRR17520069.5531818
SRR17520069.5531820
SRR17520069.5531822
SRR17520069.5531824
SRR17520069.5531826
SRR17520069.5531828
SRR17520069.5531830
SRR17520069.5531832
SRR17520069.5531836
SRR17520069.5531838
SRR17520069.5531840
SRR17520069.5531842
SRR17520069.5531844
SRR17520069.5531846
SRR17520069.5531848
SRR17520069.5531852
SRR17520069.5531854
SRR17520069.5531858
SRR17520069.5532215
SRR17520069.5532217
SRR17520069.5534454
SRR17520069.5538394
SRR17520069.5539548
SRR17520069.5548605
SRR17520069.5548607
SRR17520069.5548609
SRR17520069.5548613
SRR17520069.5548615
SRR17520069.5548617
SRR17520069.5548619
SRR17520069.5548621
SRR17520069.5548623
SRR17520069.5548625
SRR17520069.5548627
SRR17520069.5548629
SRR17520069.5548631
SRR17520069.5548633

SRR17520069.5551210
SRR17520069.5554105
SRR17520069.5561778
SRR17520069.5561780
SRR17520069.5561782
SRR17520069.5561784
SRR17520069.5561786
SRR17520069.5561788
SRR17520069.5561790
SRR17520069.5561792
SRR17520069.5561794
SRR17520069.5561796
SRR17520069.5561798
SRR17520069.5561800
SRR17520069.5561802
SRR17520069.5561804
SRR17520069.5561806
SRR17520069.5561808
SRR17520069.5561810
SRR17520069.5561812
SRR17520069.5561814
SRR17520069.5562824
SRR17520069.5562826
SRR17520069.5563194
SRR17520069.5563196
SRR17520069.5563198
SRR17520069.5563200
SRR17520069.5563202
SRR17520069.5563204
SRR17520069.5563206
SRR17520069.5563208
SRR17520069.5563210
SRR17520069.5565572
SRR17520069.5565574
SRR17520069.5565576
SRR17520069.5565578
SRR17520069.5565580
SRR17520069.5565582
SRR17520069.5565584
SRR17520069.5565586
SRR17520069.5565588
SRR17520069.5565590
SRR17520069.5565592
SRR17520069.5565594
SRR17520069.5570402
SRR17520069.5570404
SRR17520069.5570406

SRR17520069.5570408
SRR17520069.5573015
SRR17520069.5573017
SRR17520069.5573019
SRR17520069.5573021
SRR17520069.5573023
SRR17520069.5573025
SRR17520069.5573027
SRR17520069.5573031
SRR17520069.5573033
SRR17520069.5573035
SRR17520069.5573037
SRR17520069.5573039
SRR17520069.5573041
SRR17520069.5573043
SRR17520069.5573045
SRR17520069.5573047
SRR17520069.5573049
SRR17520069.5573051
SRR17520069.5573053
SRR17520069.5573055
SRR17520069.5573057
SRR17520069.5573059
SRR17520069.5573061
SRR17520069.5573063
SRR17520069.5573065
SRR17520069.5573067
SRR17520069.5573069
SRR17520069.5573071
SRR17520069.5573073
SRR17520069.5573075
SRR17520069.5573077
SRR17520069.5573079
SRR17520069.5573081
SRR17520069.5573083
SRR17520069.5573085
SRR17520069.5573087
SRR17520069.5573089
SRR17520069.5573091
SRR17520069.5573093
SRR17520069.5573095
SRR17520069.5573099
SRR17520069.5573101
SRR17520069.5573103
SRR17520069.5573105
SRR17520069.5573107
SRR17520069.5573109

SRR17520069.5573111
SRR17520069.5573115
SRR17520069.5573117
SRR17520069.5573119
SRR17520069.5573121
SRR17520069.5573123
SRR17520069.5573125
SRR17520069.5573129
SRR17520069.5573131
SRR17520069.5573133
SRR17520069.5573135
SRR17520069.5573137
SRR17520069.5573139
SRR17520069.5573141
SRR17520069.5573145
SRR17520069.5573149
SRR17520069.5573151
SRR17520069.5573155
SRR17520069.5573157
SRR17520069.5573159
SRR17520069.5573161
SRR17520069.5573163
SRR17520069.5573165
SRR17520069.5573167
SRR17520069.5573169
SRR17520069.5573171
SRR17520069.5573173
SRR17520069.5573175
SRR17520069.5573177
SRR17520069.5573179
SRR17520069.5573181
SRR17520069.5573183
SRR17520069.5573185
SRR17520069.5573187
SRR17520069.5573189
SRR17520069.5573191
SRR17520069.5573193
SRR17520069.5573195
SRR17520069.5573197
SRR17520069.5573199
SRR17520069.5573201
SRR17520069.5573203
SRR17520069.5573205
SRR17520069.5573207
SRR17520069.5573209
SRR17520069.5573211
SRR17520069.5573213

SRR17520069.5573215
SRR17520069.5573217
SRR17520069.5573219
SRR17520069.5573221
SRR17520069.5573225
SRR17520069.5573227
SRR17520069.5573229
SRR17520069.5573231
SRR17520069.5573233
SRR17520069.5573235
SRR17520069.5573237
SRR17520069.5573239
SRR17520069.5573241
SRR17520069.5573243
SRR17520069.5573247
SRR17520069.5573249
SRR17520069.5573251
SRR17520069.5573253
SRR17520069.5573255
SRR17520069.5573257
SRR17520069.5573259
SRR17520069.5573261
SRR17520069.5573263
SRR17520069.5573880
SRR17520069.5573882
SRR17520069.5573884
SRR17520069.5573886
SRR17520069.5573888
SRR17520069.5573890
SRR17520069.5573892
SRR17520069.5573894
SRR17520069.5573896
SRR17520069.5573898
SRR17520069.5573900
SRR17520069.5573902
SRR17520069.5573904
SRR17520069.5573906
SRR17520069.5573908
SRR17520069.5573910
SRR17520069.5573912
SRR17520069.5573914
SRR17520069.5573916
SRR17520069.5573918
SRR17520069.5573920
SRR17520069.5573922
SRR17520069.5573924
SRR17520069.5573926

SRR17520069.5573928
SRR17520069.5573930
SRR17520069.5573932
SRR17520069.5573934
SRR17520069.5573936
SRR17520069.5573938
SRR17520069.5573940
SRR17520069.5573942
SRR17520069.5573944
SRR17520069.5573946
SRR17520069.5573948
SRR17520069.5573950
SRR17520069.5573952
SRR17520069.5573954
SRR17520069.5573956
SRR17520069.5573958
SRR17520069.5573960
SRR17520069.5573962
SRR17520069.5573964
SRR17520069.5573966
SRR17520069.5573968
SRR17520069.5573970
SRR17520069.5573972
SRR17520069.5573974
SRR17520069.5573976
SRR17520069.5573978
SRR17520069.5573980
SRR17520069.5573982
SRR17520069.5573984
SRR17520069.5573986
SRR17520069.5573988
SRR17520069.5573990
SRR17520069.5573992
SRR17520069.5573994
SRR17520069.5573996
SRR17520069.5573998
SRR17520069.5574000
SRR17520069.5574002
SRR17520069.5574004
SRR17520069.5576251
SRR17520069.5576253
SRR17520069.5576255
SRR17520069.5576257
SRR17520069.5576259
SRR17520069.5576261
SRR17520069.5576263
SRR17520069.5576265

SRR17520069.5576267
SRR17520069.5576269
SRR17520069.5576271
SRR17520069.5576273
SRR17520069.5576275
SRR17520069.5576279
SRR17520069.5576281
SRR17520069.5576283
SRR17520069.5587192
SRR17520069.5587194
SRR17520069.5587196
SRR17520069.5587198
SRR17520069.5587200
SRR17520069.5587203
SRR17520069.5587205
SRR17520069.5587207
SRR17520069.5587209
SRR17520069.5587213
SRR17520069.5587219
SRR17520069.5587221
SRR17520069.5587223
SRR17520069.5587225
SRR17520069.5587227
SRR17520069.5587229
SRR17520069.5587231
SRR17520069.5587233
SRR17520069.5587235
SRR17520069.5587237
SRR17520069.5587239
SRR17520069.5587241
SRR17520069.5587243
SRR17520069.5587247
SRR17520069.5587249
SRR17520069.5587251
SRR17520069.5587253
SRR17520069.5587255
SRR17520069.5587257
SRR17520069.5587259
SRR17520069.5587261
SRR17520069.5587263
SRR17520069.5587265
SRR17520069.5590509
SRR17520069.5590511
SRR17520069.5590513
SRR17520069.5590515
SRR17520069.5590517
SRR17520069.5609231

SRR17520069.5609233
SRR17520069.5609235
SRR17520069.5609237
SRR17520069.5609239
SRR17520069.5609241
SRR17520069.5615713
SRR17520069.5615715
SRR17520069.5615717
SRR17520069.5615719
SRR17520069.5615721
SRR17520069.5615723
SRR17520069.5615725
SRR17520069.5615727
SRR17520069.5615729
SRR17520069.5615731
SRR17520069.5615735
SRR17520069.5615737
SRR17520069.5615739
SRR17520069.5615741
SRR17520069.5615743
SRR17520069.5615747
SRR17520069.5615751
SRR17520069.5615753
SRR17520069.5615755
SRR17520069.5615757
SRR17520069.5615759
SRR17520069.5615761
SRR17520069.5615763
SRR17520069.5615765
SRR17520069.5615767
SRR17520069.5615771
SRR17520069.5615773
SRR17520069.5615775
SRR17520069.5615777
SRR17520069.5615779
SRR17520069.5615781
SRR17520069.5615785
SRR17520069.5615787
SRR17520069.5615789
SRR17520069.5615791
SRR17520069.5615793
SRR17520069.5615795
SRR17520069.5615797
SRR17520069.5615799
SRR17520069.5615801
SRR17520069.5615803
SRR17520069.5615805

SRR17520069.5615807
SRR17520069.5615809
SRR17520069.5615811
SRR17520069.5615813
SRR17520069.5615815
SRR17520069.5615817
SRR17520069.5615819
SRR17520069.5615823
SRR17520069.5615825
SRR17520069.5615827
SRR17520069.5616599
SRR17520069.5616601
SRR17520069.5616604
SRR17520069.5616606
SRR17520069.5616608
SRR17520069.5616610
SRR17520069.5616612
SRR17520069.5616614
SRR17520069.5616616
SRR17520069.5616618
SRR17520069.5618257
SRR17520069.5618259
SRR17520069.5618261
SRR17520069.5618263
SRR17520069.5618265
SRR17520069.5618267
SRR17520069.5618269
SRR17520069.5618271
SRR17520069.5618273
SRR17520069.5618275
SRR17520069.5625173
SRR17520069.5625177
SRR17520069.5625188
SRR17520069.5625190
SRR17520069.5625192
SRR17520069.5625194
SRR17520069.5625196
SRR17520069.5625198
SRR17520069.5625200
SRR17520069.5625202
SRR17520069.5625204
SRR17520069.5625208
SRR17520069.5625210
SRR17520069.5631649
SRR17520069.5631651
SRR17520069.5631653
SRR17520069.5631655

SRR17520069.5631657
SRR17520069.5631659
SRR17520069.5631661
SRR17520069.5631663
SRR17520069.5631665
SRR17520069.5631667
SRR17520069.5631669
SRR17520069.5631671
SRR17520069.5631673
SRR17520069.5631675
SRR17520069.5631677
SRR17520069.5631679
SRR17520069.5631681
SRR17520069.5631683
SRR17520069.5631685
SRR17520069.5631687
SRR17520069.5631689
SRR17520069.5631691
SRR17520069.5631693
SRR17520069.5631695
SRR17520069.5631697
SRR17520069.5631699
SRR17520069.5631701
SRR17520069.5631703
SRR17520069.5631705
SRR17520069.5631707
SRR17520069.5631709
SRR17520069.5631711
SRR17520069.5631713
SRR17520069.5631717
SRR17520069.5631719
SRR17520069.5631721
SRR17520069.5631723
SRR17520069.5631725
SRR17520069.5631727
SRR17520069.5631729
SRR17520069.5631731
SRR17520069.5631733
SRR17520069.5631735
SRR17520069.5631737
SRR17520069.5631739
SRR17520069.5631741
SRR17520069.5631743
SRR17520069.5631745
SRR17520069.5631747
SRR17520069.5631749
SRR17520069.5631751

SRR17520069.5631753
SRR17520069.5631755
SRR17520069.5631757
SRR17520069.5631759
SRR17520069.5631761
SRR17520069.5631763
SRR17520069.5631765
SRR17520069.5631767
SRR17520069.5631769
SRR17520069.5631771
SRR17520069.5631773
SRR17520069.5631775
SRR17520069.5631777
SRR17520069.5631779
SRR17520069.5631781
SRR17520069.5631783
SRR17520069.5631785
SRR17520069.5631787
SRR17520069.5631789
SRR17520069.5631791
SRR17520069.5631793
SRR17520069.5631795
SRR17520069.5631797
SRR17520069.5631799
SRR17520069.5631801
SRR17520069.5631803
SRR17520069.5631805
SRR17520069.5631807
SRR17520069.5631809
SRR17520069.5631811
SRR17520069.5631813
SRR17520069.5631815
SRR17520069.5631817
SRR17520069.5631819
SRR17520069.5631821
SRR17520069.5631823
SRR17520069.5631825
SRR17520069.5631827
SRR17520069.5631829
SRR17520069.5631831
SRR17520069.5631833
SRR17520069.5631835
SRR17520069.5631837
SRR17520069.5637236
SRR17520069.5637238
SRR17520069.5637240
SRR17520069.5637242

SRR17520069.5637246
SRR17520069.5637248
SRR17520069.5637250
SRR17520069.5637252
SRR17520069.5637254
SRR17520069.5637256
SRR17520069.5637258
SRR17520069.5637262
SRR17520069.5637266
SRR17520069.5637268
SRR17520069.5637270
SRR17520069.5637272
SRR17520069.5637274
SRR17520069.5637278
SRR17520069.5637280
SRR17520069.5637284
SRR17520069.5637286
SRR17520069.5637288
SRR17520069.5637290
SRR17520069.5637294
SRR17520069.5637298
SRR17520069.5637302
SRR17520069.5637304
SRR17520069.5637306
SRR17520069.5637308
SRR17520069.5637310
SRR17520069.5637312
SRR17520069.5637314
SRR17520069.5639396
SRR17520069.5639402
SRR17520069.5639404
SRR17520069.5639406
SRR17520069.5639408
SRR17520069.5639410
SRR17520069.5639412
SRR17520069.5639414
SRR17520069.5639416
SRR17520069.5639418
SRR17520069.5639420
SRR17520069.5639422
SRR17520069.5639424
SRR17520069.5639428
SRR17520069.5639430
SRR17520069.5639432
SRR17520069.5639434
SRR17520069.5639436
SRR17520069.5639438

SRR17520069.5639440
SRR17520069.5641457
SRR17520069.5643510
SRR17520069.5643512
SRR17520069.5643514
SRR17520069.5643516
SRR17520069.5643518
SRR17520069.5643520
SRR17520069.5643522
SRR17520069.5643524
SRR17520069.5643526
SRR17520069.5643528
SRR17520069.5643530
SRR17520069.5643532
SRR17520069.5643534
SRR17520069.5643536
SRR17520069.5643538
SRR17520069.5643540
SRR17520069.5643546
SRR17520069.5643548
SRR17520069.5643550
SRR17520069.5643552
SRR17520069.5643554
SRR17520069.5643556
SRR17520069.5643568
SRR17520069.5643570
SRR17520069.5644726
SRR17520069.5644728
SRR17520069.5644730
SRR17520069.5644732
SRR17520069.5644734
SRR17520069.5644736
SRR17520069.5644738
SRR17520069.5644740
SRR17520069.5644742
SRR17520069.5644744
SRR17520069.5644746
SRR17520069.5644748
SRR17520069.5647341
SRR17520069.5647343
SRR17520069.5647345
SRR17520069.5647347
SRR17520069.5647349
SRR17520069.5647351
SRR17520069.5647353
SRR17520069.5647355
SRR17520069.5647357

SRR17520069.5647359
SRR17520069.5647361
SRR17520069.5647363
SRR17520069.5647880
SRR17520069.5647884
SRR17520069.5647886
SRR17520069.5647888
SRR17520069.5652705
SRR17520069.5652707
SRR17520069.5652713
SRR17520069.5652715
SRR17520069.5652717
SRR17520069.5652719
SRR17520069.5652721
SRR17520069.5652723
SRR17520069.5652725
SRR17520069.5652727
SRR17520069.5652729
SRR17520069.5663436
SRR17520069.5663438
SRR17520069.5663442
SRR17520069.5676454
SRR17520069.5676456
SRR17520069.5676458
SRR17520069.5676460
SRR17520069.5676462
SRR17520069.5676464
SRR17520069.5676466
SRR17520069.5676468
SRR17520069.5676470
SRR17520069.5678549
SRR17520069.5678551
SRR17520069.5678555
SRR17520069.5678557
SRR17520069.5678559
SRR17520069.5678561
SRR17520069.5678563
SRR17520069.5678565
SRR17520069.5678567
SRR17520069.5678569
SRR17520069.5678571
SRR17520069.5678573
SRR17520069.5678575
SRR17520069.5678577
SRR17520069.5678579
SRR17520069.5678581
SRR17520069.5678583

SRR17520069.5678585
SRR17520069.5678587
SRR17520069.5678589
SRR17520069.5678591
SRR17520069.5678593
SRR17520069.5678595
SRR17520069.5678597
SRR17520069.5678599
SRR17520069.5678601
SRR17520069.5678603
SRR17520069.5678605
SRR17520069.5678607
SRR17520069.5678609
SRR17520069.5678611
SRR17520069.5678878
SRR17520069.5679011
SRR17520069.5686856
SRR17520069.5691389
SRR17520069.5693917
SRR17520069.5693919
SRR17520069.5695415
SRR17520069.5695417
SRR17520069.5695419
SRR17520069.5695421
SRR17520069.5695423
SRR17520069.5695425
SRR17520069.5695427
SRR17520069.5695429
SRR17520069.5695431
SRR17520069.5695433
SRR17520069.5695435
SRR17520069.5695523
SRR17520069.5695525
SRR17520069.5695527
SRR17520069.5695529
SRR17520069.5695531
SRR17520069.5695533
SRR17520069.5701803
SRR17520069.5701807
SRR17520069.5701809
SRR17520069.5701811
SRR17520069.5701815
SRR17520069.5701819
SRR17520069.5701821
SRR17520069.5701825
SRR17520069.5703701
SRR17520069.5703705

SRR17520069.5703707
SRR17520069.5703709
SRR17520069.5703711
SRR17520069.5703713
SRR17520069.5703715
SRR17520069.5703717
SRR17520069.5703719
SRR17520069.5704275
SRR17520069.5704277
SRR17520069.5704279
SRR17520069.5704281
SRR17520069.5704283
SRR17520069.5704285
SRR17520069.5704287
SRR17520069.5704321
SRR17520069.5704323
SRR17520069.5704325
SRR17520069.5704327
SRR17520069.5704329
SRR17520069.5704331
SRR17520069.5704333
SRR17520069.5704335
SRR17520069.5704337
SRR17520069.5704339
SRR17520069.5704341
SRR17520069.5704343
SRR17520069.5704345
SRR17520069.5704347
SRR17520069.5704349
SRR17520069.5704351
SRR17520069.5704353
SRR17520069.5704355
SRR17520069.5704357
SRR17520069.5704359
SRR17520069.5704361
SRR17520069.5704363
SRR17520069.5704365
SRR17520069.5704367
SRR17520069.5704369
SRR17520069.5704371
SRR17520069.5704373
SRR17520069.5704375
SRR17520069.5704377
SRR17520069.5704379
SRR17520069.5704816
SRR17520069.5704818
SRR17520069.5704820

SRR17520069.5704822
SRR17520069.5704824
SRR17520069.5704826
SRR17520069.5704830
SRR17520069.5704832
SRR17520069.5704834
SRR17520069.5704836
SRR17520069.5704840
SRR17520069.5704846
SRR17520069.5704848
SRR17520069.5711166
SRR17520069.5711168
SRR17520069.5711170
SRR17520069.5714215
SRR17520069.5714217
SRR17520069.5714219
SRR17520069.5714221
SRR17520069.5715503
SRR17520069.5715507
SRR17520069.5715938
SRR17520069.5715940
SRR17520069.5715942
SRR17520069.5715944
SRR17520069.5715946
SRR17520069.5715948
SRR17520069.5715950
SRR17520069.5715952
SRR17520069.5715954
SRR17520069.5715956
SRR17520069.5715958
SRR17520069.5715960
SRR17520069.5715962
SRR17520069.5715964
SRR17520069.5715966
SRR17520069.5715968
SRR17520069.5715972
SRR17520069.5715974
SRR17520069.5715976
SRR17520069.5715978
SRR17520069.5715990
SRR17520069.5715994
SRR17520069.5715996
SRR17520069.5715998
SRR17520069.5716000
SRR17520069.5721929
SRR17520069.5721931
SRR17520069.5721933

SRR17520069.5721935
SRR17520069.5721937
SRR17520069.5721939
SRR17520069.5721941
SRR17520069.5721943
SRR17520069.5728998
SRR17520069.5734190
SRR17520069.5734192
SRR17520069.5734194
SRR17520069.5734196
SRR17520069.5734198
SRR17520069.5734202
SRR17520069.5734204
SRR17520069.5734206
SRR17520069.5734208
SRR17520069.5734210
SRR17520069.5734212
SRR17520069.5734214
SRR17520069.5734216
SRR17520069.5734218
SRR17520069.5734220
SRR17520069.5734222
SRR17520069.5734224
SRR17520069.5734226
SRR17520069.5734228
SRR17520069.5734230
SRR17520069.5734232
SRR17520069.5734234
SRR17520069.5734236
SRR17520069.5734238
SRR17520069.5734240
SRR17520069.5734242
SRR17520069.5734244
SRR17520069.5734246
SRR17520069.5734248
SRR17520069.5734250
SRR17520069.5734252
SRR17520069.5734254
SRR17520069.5741101
SRR17520069.5741103
SRR17520069.5741105
SRR17520069.5741107
SRR17520069.5741109
SRR17520069.5741111
SRR17520069.5741113
SRR17520069.5741115
SRR17520069.5741117

SRR17520069.5741119
SRR17520069.5741121
SRR17520069.5741123
SRR17520069.5741125
SRR17520069.5741127
SRR17520069.5741129
SRR17520069.5741131
SRR17520069.5741133
SRR17520069.5741135
SRR17520069.5741137
SRR17520069.5741139
SRR17520069.5741141
SRR17520069.5742185
SRR17520069.5742187
SRR17520069.5742189
SRR17520069.5742191
SRR17520069.5742193
SRR17520069.5744099
SRR17520069.5744101
SRR17520069.5744103
SRR17520069.5744105
SRR17520069.5744107
SRR17520069.5744109
SRR17520069.5744111
SRR17520069.5744113
SRR17520069.5744115
SRR17520069.5744117
SRR17520069.5744899
SRR17520069.5744901
SRR17520069.5744903
SRR17520069.5744905
SRR17520069.5744907
SRR17520069.5744909
SRR17520069.5744911
SRR17520069.5744913
SRR17520069.5744915
SRR17520069.5744917
SRR17520069.5744919
SRR17520069.5744921
SRR17520069.5744923
SRR17520069.5744925
SRR17520069.5744927
SRR17520069.5744929
SRR17520069.5744931
SRR17520069.5744933
SRR17520069.5744935
SRR17520069.5750685

SRR17520069.5750687
SRR17520069.5750689
SRR17520069.5750693
SRR17520069.5753746
SRR17520069.5753748
SRR17520069.5753750
SRR17520069.5753752
SRR17520069.5753754
SRR17520069.5753756
SRR17520069.5753758
SRR17520069.5753760
SRR17520069.5753762
SRR17520069.5753764
SRR17520069.5753766
SRR17520069.5753768
SRR17520069.5753770
SRR17520069.5753772
SRR17520069.5753774
SRR17520069.5753776
SRR17520069.5753778
SRR17520069.5753780
SRR17520069.5753782
SRR17520069.5753784
SRR17520069.5753786
SRR17520069.5753788
SRR17520069.5753790
SRR17520069.5753792
SRR17520069.5753794
SRR17520069.5753796
SRR17520069.5753798
SRR17520069.5753800
SRR17520069.5753802
SRR17520069.5753804
SRR17520069.5753806
SRR17520069.5753808
SRR17520069.5753810
SRR17520069.5760593
SRR17520069.5760682
SRR17520069.5760684
SRR17520069.5760686
SRR17520069.5760688
SRR17520069.5760690
SRR17520069.5762141
SRR17520069.5762143
SRR17520069.5762145
SRR17520069.5762147
SRR17520069.5762149

SRR17520069.5762151
SRR17520069.5762153
SRR17520069.5762155
SRR17520069.5762157
SRR17520069.5762159
SRR17520069.5762161
SRR17520069.5762163
SRR17520069.5762165
SRR17520069.5762167
SRR17520069.5762169
SRR17520069.5762171
SRR17520069.5762173
SRR17520069.5762175
SRR17520069.5762177
SRR17520069.5762179
SRR17520069.5762181
SRR17520069.5762183
SRR17520069.5762185
SRR17520069.5762189
SRR17520069.5762191
SRR17520069.5762195
SRR17520069.5762197
SRR17520069.5762199
SRR17520069.5762201
SRR17520069.5762203
SRR17520069.5762205
SRR17520069.5762207
SRR17520069.5762209
SRR17520069.5762211
SRR17520069.5762213
SRR17520069.5762215
SRR17520069.5762217
SRR17520069.5762219
SRR17520069.5762221
SRR17520069.5762223
SRR17520069.5762225
SRR17520069.5762227
SRR17520069.5762229
SRR17520069.5762231
SRR17520069.5762233
SRR17520069.5762237
SRR17520069.5762239
SRR17520069.5762241
SRR17520069.5762243
SRR17520069.5762245
SRR17520069.5780188
SRR17520069.5780190

SRR17520069.5780192
SRR17520069.5780194
SRR17520069.5780196
SRR17520069.5780198
SRR17520069.5780200
SRR17520069.5780202
SRR17520069.5780204
SRR17520069.5780206
SRR17520069.5780208
SRR17520069.5780210
SRR17520069.5780212
SRR17520069.5783924
SRR17520069.5783926
SRR17520069.5783930
SRR17520069.5783932
SRR17520069.5783934
SRR17520069.5783936
SRR17520069.5783938
SRR17520069.5783940
SRR17520069.5783942
SRR17520069.5783944
SRR17520069.5783946
SRR17520069.5783948
SRR17520069.5783950
SRR17520069.5783952
SRR17520069.5783956
SRR17520069.5783958
SRR17520069.5783962
SRR17520069.5783966
SRR17520069.5783968
SRR17520069.5783970
SRR17520069.5783972
SRR17520069.5783974
SRR17520069.5783976
SRR17520069.5783978
SRR17520069.5783980
SRR17520069.5783982
SRR17520069.5783984
SRR17520069.5783986
SRR17520069.5783988
SRR17520069.5783990
SRR17520069.5783992
SRR17520069.5783994
SRR17520069.5783996
SRR17520069.5783998
SRR17520069.5784000
SRR17520069.5784002

SRR17520069.5784004
SRR17520069.5784006
SRR17520069.5784008
SRR17520069.5784010
SRR17520069.5784012
SRR17520069.5784014
SRR17520069.5784016
SRR17520069.5784018
SRR17520069.5784020
SRR17520069.5784022
SRR17520069.5784024
SRR17520069.5784026
SRR17520069.5784028
SRR17520069.5784032
SRR17520069.5784034
SRR17520069.5784036
SRR17520069.5784038
SRR17520069.5784040
SRR17520069.5784044
SRR17520069.5784046
SRR17520069.5784048
SRR17520069.5784050
SRR17520069.5784052
SRR17520069.5784054
SRR17520069.5784056
SRR17520069.5784058
SRR17520069.5784060
SRR17520069.5784068
SRR17520069.5784070
SRR17520069.5784072
SRR17520069.5784074
SRR17520069.5784076
SRR17520069.5784078
SRR17520069.5784080
SRR17520069.5784082
SRR17520069.5784357
SRR17520069.5784359
SRR17520069.5784361
SRR17520069.5784363
SRR17520069.5784365
SRR17520069.5784367
SRR17520069.5784369
SRR17520069.5784371
SRR17520069.5784373
SRR17520069.5784375
SRR17520069.5784377
SRR17520069.5784379

SRR17520069.5784381
SRR17520069.5784383
SRR17520069.5784385
SRR17520069.5784387
SRR17520069.5784389
SRR17520069.5784391
SRR17520069.5784393
SRR17520069.5785802
SRR17520069.5785804
SRR17520069.5785806
SRR17520069.5785808
SRR17520069.5785810
SRR17520069.5785812
SRR17520069.5785814
SRR17520069.5785816
SRR17520069.5785818
SRR17520069.5785820
SRR17520069.5785822
SRR17520069.5785824
SRR17520069.5785826
SRR17520069.5785828
SRR17520069.5785830
SRR17520069.5785832
SRR17520069.5786208
SRR17520069.5786210
SRR17520069.5786212
SRR17520069.5786214
SRR17520069.5786216
SRR17520069.5786218
SRR17520069.5786220
SRR17520069.5786222
SRR17520069.5786224
SRR17520069.5795072
SRR17520069.5797450
SRR17520069.5800202
SRR17520069.5800204
SRR17520069.5800206
SRR17520069.5800208
SRR17520069.5800210
SRR17520069.5800212
SRR17520069.5800214
SRR17520069.5800216
SRR17520069.5800280
SRR17520069.5800607
SRR17520069.5800609
SRR17520069.5800611
SRR17520069.5800613

SRR17520069.5800615
SRR17520069.5800617
SRR17520069.5800619
SRR17520069.5800621
SRR17520069.5800623
SRR17520069.5800625
SRR17520069.5800627
SRR17520069.5800629
SRR17520069.5800631
SRR17520069.5800633
SRR17520069.5800635
SRR17520069.5800637
SRR17520069.5800639
SRR17520069.5800641
SRR17520069.5800643
SRR17520069.5800645
SRR17520069.5800647
SRR17520069.5800649
SRR17520069.5800653
SRR17520069.5800655
SRR17520069.5800657
SRR17520069.5800659
SRR17520069.5800661
SRR17520069.5800663
SRR17520069.5800665
SRR17520069.5800667
SRR17520069.5800669
SRR17520069.5800671
SRR17520069.5800673
SRR17520069.5800675
SRR17520069.5807937
SRR17520069.5807939
SRR17520069.5807941
SRR17520069.5807943
SRR17520069.5807945
SRR17520069.5807949
SRR17520069.5807951
SRR17520069.5807953
SRR17520069.5807955
SRR17520069.5807957
SRR17520069.5807959
SRR17520069.5807961
SRR17520069.5807963
SRR17520069.5807965
SRR17520069.5807967
SRR17520069.5807969
SRR17520069.5807971

SRR17520069.5807973
SRR17520069.5807975
SRR17520069.5807977
SRR17520069.5810032
SRR17520069.5811780
SRR17520069.5811782
SRR17520069.5811784
SRR17520069.5811786
SRR17520069.5811788
SRR17520069.5811790
SRR17520069.5811792
SRR17520069.5811794
SRR17520069.5811796
SRR17520069.5811798
SRR17520069.5811800
SRR17520069.5811804
SRR17520069.5811806
SRR17520069.5811808
SRR17520069.5811810
SRR17520069.5811812
SRR17520069.5811814
SRR17520069.5811816
SRR17520069.5811818
SRR17520069.5811820
SRR17520069.5811822
SRR17520069.5811826
SRR17520069.5818949
SRR17520069.5818951
SRR17520069.5818953
SRR17520069.5818955
SRR17520069.5818957
SRR17520069.5818959
SRR17520069.5818961
SRR17520069.5818963
SRR17520069.5818975
SRR17520069.5818977
SRR17520069.5818979
SRR17520069.5818983
SRR17520069.5818985
SRR17520069.5818987
SRR17520069.5818991
SRR17520069.5818997
SRR17520069.5818999
SRR17520069.5819001
SRR17520069.5819003
SRR17520069.5819005
SRR17520069.5819007

SRR17520069.5819009
SRR17520069.5819011
SRR17520069.5819013
SRR17520069.5819015
SRR17520069.5819017
SRR17520069.5819019
SRR17520069.5819021
SRR17520069.5819025
SRR17520069.5819027
SRR17520069.5819029
SRR17520069.5819031
SRR17520069.5819033
SRR17520069.5819035
SRR17520069.5819037
SRR17520069.5819041
SRR17520069.5819043
SRR17520069.5819045
SRR17520069.5819047
SRR17520069.5819049
SRR17520069.5819051
SRR17520069.5819053
SRR17520069.5819055
SRR17520069.5819057
SRR17520069.5819059
SRR17520069.5819061
SRR17520069.5819065
SRR17520069.5822088
SRR17520069.5822090
SRR17520069.5822100
SRR17520069.5822102
SRR17520069.5822104
SRR17520069.5822108
SRR17520069.5822110
SRR17520069.5822114
SRR17520069.5822118
SRR17520069.5822124
SRR17520069.5832390
SRR17520069.5832392
SRR17520069.5832394
SRR17520069.5832396
SRR17520069.5832398
SRR17520069.5832400
SRR17520069.5832402
SRR17520069.5832404
SRR17520069.5832406
SRR17520069.5832408
SRR17520069.5834149

SRR17520069.5834151
SRR17520069.5834153
SRR17520069.5834155
SRR17520069.5834157
SRR17520069.5834163
SRR17520069.5834165
SRR17520069.5834167
SRR17520069.5834173
SRR17520069.5838243
SRR17520069.5838247
SRR17520069.5838249
SRR17520069.5838919
SRR17520069.5838921
SRR17520069.5838923
SRR17520069.5838925
SRR17520069.5838927
SRR17520069.5838929
SRR17520069.5838931
SRR17520069.5838933
SRR17520069.5838935
SRR17520069.5838937
SRR17520069.5838939
SRR17520069.5838941
SRR17520069.5838943
SRR17520069.5838945
SRR17520069.5838947
SRR17520069.5839944
SRR17520069.5839946
SRR17520069.5839948
SRR17520069.5839950
SRR17520069.5839952
SRR17520069.5839954
SRR17520069.5839956
SRR17520069.5839960
SRR17520069.5839962
SRR17520069.5839964
SRR17520069.5843626
SRR17520069.5843628
SRR17520069.5843630
SRR17520069.5843632
SRR17520069.5843634
SRR17520069.5843636
SRR17520069.5843638
SRR17520069.5843640
SRR17520069.5843642
SRR17520069.5843644
SRR17520069.5843646

SRR17520069.5843648
SRR17520069.5843650
SRR17520069.5843652
SRR17520069.5843654
SRR17520069.5843656
SRR17520069.5843658
SRR17520069.5843660
SRR17520069.5843664
SRR17520069.5843666
SRR17520069.5843668
SRR17520069.5843670
SRR17520069.5843672
SRR17520069.5843674
SRR17520069.5843676
SRR17520069.5843678
SRR17520069.5843682
SRR17520069.5843684
SRR17520069.5846888
SRR17520069.5846890
SRR17520069.5846892
SRR17520069.5846894
SRR17520069.5846896
SRR17520069.5846898
SRR17520069.5846900
SRR17520069.5846902
SRR17520069.5846906
SRR17520069.5846908
SRR17520069.5846912
SRR17520069.5846914
SRR17520069.5846916
SRR17520069.5847272
SRR17520069.5847274
SRR17520069.5847276
SRR17520069.5847278
SRR17520069.5848483
SRR17520069.5848485
SRR17520069.5848487
SRR17520069.5848489
SRR17520069.5848491
SRR17520069.5848493
SRR17520069.5848495
SRR17520069.5848497
SRR17520069.5848499
SRR17520069.5848501
SRR17520069.5848503
SRR17520069.5848505
SRR17520069.5848507

SRR17520069.5848509
SRR17520069.5848511
SRR17520069.5848513
SRR17520069.5848515
SRR17520069.5848517
SRR17520069.5848519
SRR17520069.5848521
SRR17520069.5848523
SRR17520069.5848525
SRR17520069.5848527
SRR17520069.5848529
SRR17520069.5848531
SRR17520069.5848533
SRR17520069.5848535
SRR17520069.5848537
SRR17520069.5848539
SRR17520069.5848541
SRR17520069.5848543
SRR17520069.5848545
SRR17520069.5848547
SRR17520069.5848549
SRR17520069.5848551
SRR17520069.5848553
SRR17520069.5848555
SRR17520069.5848557
SRR17520069.5848559
SRR17520069.5848561
SRR17520069.5848563
SRR17520069.5848565
SRR17520069.5848567
SRR17520069.5848569
SRR17520069.5848571
SRR17520069.5848573
SRR17520069.5848575
SRR17520069.5848577
SRR17520069.5848579
SRR17520069.5848581
SRR17520069.5848583
SRR17520069.5848585
SRR17520069.5848587
SRR17520069.5848589
SRR17520069.5848591
SRR17520069.5848593
SRR17520069.5848595
SRR17520069.5848597
SRR17520069.5848599
SRR17520069.5848601

SRR17520069.5848603
SRR17520069.5848605
SRR17520069.5848607
SRR17520069.5848948
SRR17520069.5848950
SRR17520069.5848952
SRR17520069.5848954
SRR17520069.5848956
SRR17520069.5848958
SRR17520069.5848960
SRR17520069.5848962
SRR17520069.5848964
SRR17520069.5848966
SRR17520069.5848968
SRR17520069.5848972
SRR17520069.5848974
SRR17520069.5848976
SRR17520069.5848978
SRR17520069.5848980
SRR17520069.5848982
SRR17520069.5848984
SRR17520069.5848988
SRR17520069.5849691
SRR17520069.5849693
SRR17520069.5849695
SRR17520069.5849697
SRR17520069.5849699
SRR17520069.5849701
SRR17520069.5849703
SRR17520069.5849707
SRR17520069.5849709
SRR17520069.5849711
SRR17520069.5849713
SRR17520069.5849715
SRR17520069.5849717
SRR17520069.5849719
SRR17520069.5849721
SRR17520069.5849723
SRR17520069.5849725
SRR17520069.5849727
SRR17520069.5849729
SRR17520069.5849731
SRR17520069.5849733
SRR17520069.5849735
SRR17520069.5849737
SRR17520069.5849739
SRR17520069.5849741

SRR17520069.5849743
SRR17520069.5849745
SRR17520069.5849747
SRR17520069.5849749
SRR17520069.5849751
SRR17520069.5849753
SRR17520069.5849755
SRR17520069.5849757
SRR17520069.5849759
SRR17520069.5849761
SRR17520069.5849763
SRR17520069.5849765
SRR17520069.5849767
SRR17520069.5849769
SRR17520069.5849771
SRR17520069.5849773
SRR17520069.5849775
SRR17520069.5849777
SRR17520069.5849779
SRR17520069.5849781
SRR17520069.5849783
SRR17520069.5849785
SRR17520069.5849787
SRR17520069.5849789
SRR17520069.5849791
SRR17520069.5849793
SRR17520069.5849795
SRR17520069.5849797
SRR17520069.5849799
SRR17520069.5849801
SRR17520069.5849803
SRR17520069.5849805
SRR17520069.5849807
SRR17520069.5849809
SRR17520069.5849811
SRR17520069.5849813
SRR17520069.5849818
SRR17520069.5849820
SRR17520069.5849822
SRR17520069.5849824
SRR17520069.5849826
SRR17520069.5849828
SRR17520069.5862424
SRR17520069.5862426
SRR17520069.5862428
SRR17520069.5862430
SRR17520069.5862432

SRR17520069.5862434
SRR17520069.5862436
SRR17520069.5864102
SRR17520069.5869858
SRR17520069.5869860
SRR17520069.5869862
SRR17520069.5869864
SRR17520069.5869866
SRR17520069.5869868
SRR17520069.5869870
SRR17520069.5869872
SRR17520069.5869874
SRR17520069.5869876
SRR17520069.5869878
SRR17520069.5869880
SRR17520069.5869882
SRR17520069.5869884
SRR17520069.5869886
SRR17520069.5869888
SRR17520069.5869890
SRR17520069.5869892
SRR17520069.5869894
SRR17520069.5869896
SRR17520069.5869898
SRR17520069.5869900
SRR17520069.5869902
SRR17520069.5869904
SRR17520069.5869906
SRR17520069.5869908
SRR17520069.5869910
SRR17520069.5869912
SRR17520069.5869914
SRR17520069.5869916
SRR17520069.5869926
SRR17520069.5869928
SRR17520069.5871296
SRR17520069.5871300
SRR17520069.5871326
SRR17520069.5871356
SRR17520069.5871368
SRR17520069.5871374
SRR17520069.5871397
SRR17520069.5873806
SRR17520069.5876805
SRR17520069.5876807
SRR17520069.5876809
SRR17520069.5876811

SRR17520069.5879983
SRR17520069.5888593
SRR17520069.5888595
SRR17520069.5888597
SRR17520069.5888599
SRR17520069.5888601
SRR17520069.5888603
SRR17520069.5888605
SRR17520069.5888607
SRR17520069.5888609
SRR17520069.5888611
SRR17520069.5888613
SRR17520069.5888615
SRR17520069.5888617
SRR17520069.5888619
SRR17520069.5888621
SRR17520069.5888623
SRR17520069.5888625
SRR17520069.5888627
SRR17520069.5888629
SRR17520069.5888631
SRR17520069.5888633
SRR17520069.5888635
SRR17520069.5888637
SRR17520069.5888639
SRR17520069.5888641
SRR17520069.5888643
SRR17520069.5888645
SRR17520069.5888647
SRR17520069.5888649
SRR17520069.5888651
SRR17520069.5888653
SRR17520069.5888655
SRR17520069.5888657
SRR17520069.5888659
SRR17520069.5888661
SRR17520069.5888663
SRR17520069.5888665
SRR17520069.5888667
SRR17520069.5888669
SRR17520069.5888671
SRR17520069.5888673
SRR17520069.5888675
SRR17520069.5888677
SRR17520069.5889112
SRR17520069.5889114
SRR17520069.5889122

SRR17520069.5889124
SRR17520069.5889126
SRR17520069.5889128
SRR17520069.5889130
SRR17520069.5889132
SRR17520069.5889134
SRR17520069.5889136
SRR17520069.5889138
SRR17520069.5889140
SRR17520069.5889142
SRR17520069.5889144
SRR17520069.5889146
SRR17520069.5889148
SRR17520069.5889150
SRR17520069.5889152
SRR17520069.5889154
SRR17520069.5889156
SRR17520069.5889607
SRR17520069.5889609
SRR17520069.5889611
SRR17520069.5889613
SRR17520069.5889615
SRR17520069.5889617
SRR17520069.5889621
SRR17520069.5889623
SRR17520069.5889627
SRR17520069.5889629
SRR17520069.5889631
SRR17520069.5889633
SRR17520069.5889635
SRR17520069.5889637
SRR17520069.5889639
SRR17520069.5889641
SRR17520069.5889643
SRR17520069.5889645
SRR17520069.5889647
SRR17520069.5889649
SRR17520069.5889651
SRR17520069.5889653
SRR17520069.5889828
SRR17520069.5889830
SRR17520069.5889832
SRR17520069.5889834
SRR17520069.5889836
SRR17520069.5889838
SRR17520069.5889840
SRR17520069.5889842

SRR17520069.5889844
SRR17520069.5889848
SRR17520069.5889850
SRR17520069.5889852
SRR17520069.5889854
SRR17520069.5889856
SRR17520069.5889858
SRR17520069.5889860
SRR17520069.5889862
SRR17520069.5889864
SRR17520069.5889866
SRR17520069.5889868
SRR17520069.5889870
SRR17520069.5889872
SRR17520069.5889874
SRR17520069.5889876
SRR17520069.5889878
SRR17520069.5889880
SRR17520069.5889884
SRR17520069.5889886
SRR17520069.5889888
SRR17520069.5889890
SRR17520069.5889892
SRR17520069.5889894
SRR17520069.5889898
SRR17520069.5889900
SRR17520069.5889904
SRR17520069.5889906
SRR17520069.5889908
SRR17520069.5889910
SRR17520069.5889912
SRR17520069.5889914
SRR17520069.5889916
SRR17520069.5889918
SRR17520069.5889920
SRR17520069.5889922
SRR17520069.5889924
SRR17520069.5891748
SRR17520069.5891750
SRR17520069.5891752
SRR17520069.5891754
SRR17520069.5891756
SRR17520069.5891758
SRR17520069.5891760
SRR17520069.5891762
SRR17520069.5891764
SRR17520069.5891766

SRR17520069.5891768
SRR17520069.5891770
SRR17520069.5891772
SRR17520069.5891774
SRR17520069.5891776
SRR17520069.5891783
SRR17520069.5891785
SRR17520069.5891787
SRR17520069.5891789
SRR17520069.5891791
SRR17520069.5891793
SRR17520069.5891795
SRR17520069.5891797
SRR17520069.5891799
SRR17520069.5891801
SRR17520069.5892331
SRR17520069.5892333
SRR17520069.5892335
SRR17520069.5892337
SRR17520069.5892339
SRR17520069.5892343
SRR17520069.5892349
SRR17520069.5892351
SRR17520069.5892353
SRR17520069.5892355
SRR17520069.5892359
SRR17520069.5894140
SRR17520069.5894142
SRR17520069.5894144
SRR17520069.5894146
SRR17520069.5894150
SRR17520069.5894152
SRR17520069.5894154
SRR17520069.5894156
SRR17520069.5894158
SRR17520069.5894160
SRR17520069.5894162
SRR17520069.5894164
SRR17520069.5894166
SRR17520069.5894170
SRR17520069.5894172
SRR17520069.5894174
SRR17520069.5894176
SRR17520069.5894178
SRR17520069.5894184
SRR17520069.5894186
SRR17520069.5894188

SRR17520069.5894192
SRR17520069.5894198
SRR17520069.5894916
SRR17520069.5894918
SRR17520069.5894920
SRR17520069.5894922
SRR17520069.5894924
SRR17520069.5894926
SRR17520069.5894928
SRR17520069.5894930
SRR17520069.5894932
SRR17520069.5894934
SRR17520069.5894936
SRR17520069.5894938
SRR17520069.5894940
SRR17520069.5894942
SRR17520069.5894944
SRR17520069.5894946
SRR17520069.5894948
SRR17520069.5894950
SRR17520069.5894952
SRR17520069.5894954
SRR17520069.5894956
SRR17520069.5894958
SRR17520069.5894960
SRR17520069.5894962
SRR17520069.5894964
SRR17520069.5894966
SRR17520069.5894968
SRR17520069.5894970
SRR17520069.5894972
SRR17520069.5894974
SRR17520069.5894976
SRR17520069.5894978
SRR17520069.5894980
SRR17520069.5894982
SRR17520069.5894984
SRR17520069.5894986
SRR17520069.5894988
SRR17520069.5900739
SRR17520069.5900741
SRR17520069.5901805
SRR17520069.5901807
SRR17520069.5901809
SRR17520069.5901811
SRR17520069.5901813
SRR17520069.5901815

SRR17520069.5901817
SRR17520069.5901819
SRR17520069.5901821
SRR17520069.5901823
SRR17520069.5901825
SRR17520069.5901827
SRR17520069.5901829
SRR17520069.5901831
SRR17520069.5901833
SRR17520069.5901835
SRR17520069.5901837
SRR17520069.5901839
SRR17520069.5901841
SRR17520069.5901843
SRR17520069.5901845
SRR17520069.5901847
SRR17520069.5901849
SRR17520069.5901851
SRR17520069.5901853
SRR17520069.5901855
SRR17520069.5901857
SRR17520069.5901859
SRR17520069.5901861
SRR17520069.5901863
SRR17520069.5901865
SRR17520069.5901867
SRR17520069.5901869
SRR17520069.5901871
SRR17520069.5901873
SRR17520069.5901875
SRR17520069.5901877
SRR17520069.5901879
SRR17520069.5901881
SRR17520069.5901883
SRR17520069.5901885
SRR17520069.5901887
SRR17520069.5901889
SRR17520069.5901891
SRR17520069.5901893
SRR17520069.5901895
SRR17520069.5901897
SRR17520069.5917750
SRR17520069.5917754
SRR17520069.5917756
SRR17520069.5917758
SRR17520069.5917760
SRR17520069.5917764

SRR17520069.5917766
SRR17520069.5917768
SRR17520069.5917770
SRR17520069.5917772
SRR17520069.5917774
SRR17520069.5917776
SRR17520069.5917778
SRR17520069.5917780
SRR17520069.5917782
SRR17520069.5917784
SRR17520069.5917786
SRR17520069.5922949
SRR17520069.5922951
SRR17520069.5922953
SRR17520069.5922955
SRR17520069.5922957
SRR17520069.5922959
SRR17520069.5922961
SRR17520069.5922963
SRR17520069.5922967
SRR17520069.5922969
SRR17520069.5922971
SRR17520069.5922973
SRR17520069.5922977
SRR17520069.5922979
SRR17520069.5922983
SRR17520069.5922987
SRR17520069.5938635
SRR17520069.5938637
SRR17520069.5938639
SRR17520069.5938641
SRR17520069.5938643
SRR17520069.5938645
SRR17520069.5938647
SRR17520069.5938649
SRR17520069.5938651
SRR17520069.5938653
SRR17520069.5938655
SRR17520069.5938657
SRR17520069.5938659
SRR17520069.5938661
SRR17520069.5938663
SRR17520069.5938665
SRR17520069.5938667
SRR17520069.5938669
SRR17520069.5938671
SRR17520069.5938675

SRR17520069.5938677
SRR17520069.5938679
SRR17520069.5938683
SRR17520069.5938685
SRR17520069.5938687
SRR17520069.5938691
SRR17520069.5938693
SRR17520069.5938695
SRR17520069.5938697
SRR17520069.5938701
SRR17520069.5938703
SRR17520069.5938705
SRR17520069.5938707
SRR17520069.5938711
SRR17520069.5938715
SRR17520069.5947176
SRR17520069.5947178
SRR17520069.5947180
SRR17520069.5947182
SRR17520069.5947184
SRR17520069.5947186
SRR17520069.5947190
SRR17520069.5947192
SRR17520069.5947194
SRR17520069.5947196
SRR17520069.5947198
SRR17520069.5947200
SRR17520069.5947202
SRR17520069.5947204
SRR17520069.5947208
SRR17520069.5952813
SRR17520069.5952815
SRR17520069.5952817
SRR17520069.5952819
SRR17520069.5952821
SRR17520069.5952823
SRR17520069.5952825
SRR17520069.5952827
SRR17520069.5952829
SRR17520069.5952831
SRR17520069.5952833
SRR17520069.5952835
SRR17520069.5952837
SRR17520069.5952839
SRR17520069.5952841
SRR17520069.5952843
SRR17520069.5952845

SRR17520069.5952847
SRR17520069.5952849
SRR17520069.5952851
SRR17520069.5952853
SRR17520069.5952855
SRR17520069.5952857
SRR17520069.5952859
SRR17520069.5952861
SRR17520069.5952863
SRR17520069.5952865
SRR17520069.5952867
SRR17520069.5952869
SRR17520069.5952871
SRR17520069.5952873
SRR17520069.5952875
SRR17520069.5952877
SRR17520069.5952879
SRR17520069.5952881
SRR17520069.5952883
SRR17520069.5952885
SRR17520069.5952887
SRR17520069.5952889
SRR17520069.5952891
SRR17520069.5952893
SRR17520069.5952895
SRR17520069.5952897
SRR17520069.5952899
SRR17520069.5952901
SRR17520069.5952903
SRR17520069.5952905
SRR17520069.5952907
SRR17520069.5952909
SRR17520069.5952911
SRR17520069.5952913
SRR17520069.5952915
SRR17520069.5952917
SRR17520069.5952919
SRR17520069.5956280
SRR17520069.5956282
SRR17520069.5956284
SRR17520069.5956288
SRR17520069.5956290
SRR17520069.5956292
SRR17520069.5956294
SRR17520069.5956296
SRR17520069.5956298
SRR17520069.5956300

SRR17520069.5956302
SRR17520069.5956304
SRR17520069.5956306
SRR17520069.5956308
SRR17520069.5956310
SRR17520069.5956312
SRR17520069.5956314
SRR17520069.5956316
SRR17520069.5956318
SRR17520069.5956320
SRR17520069.5956322
SRR17520069.5956324
SRR17520069.5956326
SRR17520069.5960217
SRR17520069.5960219
SRR17520069.5960221
SRR17520069.5960223
SRR17520069.5960225
SRR17520069.5960227
SRR17520069.5960229
SRR17520069.5960231
SRR17520069.5960233
SRR17520069.5960235
SRR17520069.5960237
SRR17520069.5960239
SRR17520069.5960241
SRR17520069.5960243
SRR17520069.5960245
SRR17520069.5960247
SRR17520069.5960249
SRR17520069.5960251
SRR17520069.5960253
SRR17520069.5960255
SRR17520069.5960257
SRR17520069.5960259
SRR17520069.5960261
SRR17520069.5960263
SRR17520069.5960265
SRR17520069.5960267
SRR17520069.5960269
SRR17520069.5960271
SRR17520069.5960273
SRR17520069.5960275
SRR17520069.5960277
SRR17520069.5960279
SRR17520069.5960281
SRR17520069.5960283

SRR17520069.5960285
SRR17520069.5971826
SRR17520069.5971828
SRR17520069.5971830
SRR17520069.5971832
SRR17520069.5971834
SRR17520069.5971836
SRR17520069.5971838
SRR17520069.5971840
SRR17520069.5971842
SRR17520069.5971844
SRR17520069.5982849
SRR17520069.5982851
SRR17520069.5982853
SRR17520069.5982855
SRR17520069.5982857
SRR17520069.5982859
SRR17520069.5982861
SRR17520069.5982863
SRR17520069.5982865
SRR17520069.5982867
SRR17520069.5982869
SRR17520069.5982871
SRR17520069.5982873
SRR17520069.5982875
SRR17520069.5982877
SRR17520069.5982879
SRR17520069.5982881
SRR17520069.5982883
SRR17520069.5982885
SRR17520069.5982887
SRR17520069.5982889
SRR17520069.5982891
SRR17520069.5982893
SRR17520069.5982895
SRR17520069.5982897
SRR17520069.5982899
SRR17520069.5982901
SRR17520069.5982903
SRR17520069.5982905
SRR17520069.5982907
SRR17520069.5982909
SRR17520069.5982911
SRR17520069.5982913
SRR17520069.5982915
SRR17520069.5982917
SRR17520069.5982919

SRR17520069.5982921
SRR17520069.5982923
SRR17520069.5982925
SRR17520069.5983583
SRR17520069.5983585
SRR17520069.5983587
SRR17520069.5983589
SRR17520069.5983591
SRR17520069.5983593
SRR17520069.5983595
SRR17520069.5983597
SRR17520069.5983599
SRR17520069.5983601
SRR17520069.5983603
SRR17520069.5983605
SRR17520069.5983607
SRR17520069.5983609
SRR17520069.5983611
SRR17520069.5983613
SRR17520069.5983615
SRR17520069.5983617
SRR17520069.5983619
SRR17520069.5983621
SRR17520069.5983623
SRR17520069.5983625
SRR17520069.5983627
SRR17520069.5983629
SRR17520069.5983631
SRR17520069.5983633
SRR17520069.5983635
SRR17520069.5983637
SRR17520069.5983639
SRR17520069.5983641
SRR17520069.5983643
SRR17520069.5983645
SRR17520069.5984444
SRR17520069.5984446
SRR17520069.5984448
SRR17520069.5984450
SRR17520069.5984452
SRR17520069.5984454
SRR17520069.5984456
SRR17520069.5984458
SRR17520069.5984460
SRR17520069.5984462
SRR17520069.5984464
SRR17520069.5984466

SRR17520069.5984468
SRR17520069.5984470
SRR17520069.5984472
SRR17520069.5984474
SRR17520069.5984476
SRR17520069.5984478
SRR17520069.5984480
SRR17520069.5984482
SRR17520069.5984484
SRR17520069.5984486
SRR17520069.5984488
SRR17520069.5984490
SRR17520069.5984492
SRR17520069.5984494
SRR17520069.5984496
SRR17520069.5984498
SRR17520069.5984500
SRR17520069.5984502
SRR17520069.5984504
SRR17520069.5984506
SRR17520069.5984508
SRR17520069.5984510
SRR17520069.5984512
SRR17520069.5984514
SRR17520069.5984516
SRR17520069.5985359
SRR17520069.5985361
SRR17520069.5985363
SRR17520069.5985365
SRR17520069.5985367
SRR17520069.5985369
SRR17520069.5985371
SRR17520069.5985373
SRR17520069.5985375
SRR17520069.5985377
SRR17520069.5987748
SRR17520069.5987750
SRR17520069.5987752
SRR17520069.5987754
SRR17520069.5987756
SRR17520069.5987758
SRR17520069.5987760
SRR17520069.5987762
SRR17520069.5987764
SRR17520069.5987766
SRR17520069.5987768
SRR17520069.5987770

SRR17520069.5987772
SRR17520069.5987774
SRR17520069.5987776
SRR17520069.5987778
SRR17520069.5987780
SRR17520069.5987782
SRR17520069.5987784
SRR17520069.5987786
SRR17520069.5987788
SRR17520069.5987790
SRR17520069.5987794
SRR17520069.5987796
SRR17520069.5987798
SRR17520069.5987800
SRR17520069.5987802
SRR17520069.5987804
SRR17520069.5987806
SRR17520069.5987808
SRR17520069.5987810
SRR17520069.5987812
SRR17520069.5987814
SRR17520069.5987816
SRR17520069.5987818
SRR17520069.5987820
SRR17520069.5987822
SRR17520069.5987824
SRR17520069.5987826
SRR17520069.5987828
SRR17520069.5987830
SRR17520069.5987832
SRR17520069.5987834
SRR17520069.5987836
SRR17520069.5987838
SRR17520069.5987840
SRR17520069.5987842
SRR17520069.5987844
SRR17520069.5987846
SRR17520069.5987848
SRR17520069.5988720
SRR17520069.5988725
SRR17520069.5988727
SRR17520069.5988729
SRR17520069.5988731
SRR17520069.5988733
SRR17520069.5988735
SRR17520069.5988737
SRR17520069.5988739

SRR17520069.5988741
SRR17520069.5988743
SRR17520069.5988745
SRR17520069.5988747
SRR17520069.5989209
SRR17520069.5997662
SRR17520069.5997664
SRR17520069.5997666
SRR17520069.5997668
SRR17520069.5997672
SRR17520069.5997674
SRR17520069.5997676
SRR17520069.5997678
SRR17520069.5997680
SRR17520069.5997682
SRR17520069.5997684
SRR17520069.5997686
SRR17520069.5997688
SRR17520069.5997690
SRR17520069.5997692
SRR17520069.5997694
SRR17520069.5997696
SRR17520069.5997698
SRR17520069.5997700
SRR17520069.5997702
SRR17520069.5997704
SRR17520069.5997706
SRR17520069.5997708
SRR17520069.5997825
SRR17520069.5997827
SRR17520069.5997829
SRR17520069.5997831
SRR17520069.5997833
SRR17520069.5997835
SRR17520069.5997837
SRR17520069.5997839
SRR17520069.5997841
SRR17520069.6005078
SRR17520069.6005080
SRR17520069.6005082
SRR17520069.6005084
SRR17520069.6005086
SRR17520069.6005088
SRR17520069.6005090
SRR17520069.6005092
SRR17520069.6005094
SRR17520069.6011302

SRR17520069.6011304
SRR17520069.6011306
SRR17520069.6011308
SRR17520069.6011310
SRR17520069.6011312
SRR17520069.6011314
SRR17520069.6011316
SRR17520069.6011318
SRR17520069.6011320
SRR17520069.6015057
SRR17520069.6015059
SRR17520069.6015061
SRR17520069.6015063
SRR17520069.6015065
SRR17520069.6015067
SRR17520069.6015069
SRR17520069.6015071
SRR17520069.6015073
SRR17520069.6015075
SRR17520069.6015077
SRR17520069.6015079
SRR17520069.6015081
SRR17520069.6015083
SRR17520069.6015085
SRR17520069.6015087
SRR17520069.6015089
SRR17520069.6015091
SRR17520069.6015542
SRR17520069.6015545
SRR17520069.6016876
SRR17520069.6021277
SRR17520069.6021279
SRR17520069.6021281
SRR17520069.6027230
SRR17520069.6027232
SRR17520069.6027234
SRR17520069.6027236
SRR17520069.6027238
SRR17520069.6027240
SRR17520069.6027242
SRR17520069.6027244
SRR17520069.6027246
SRR17520069.6027248
SRR17520069.6027250
SRR17520069.6027252
SRR17520069.6027254
SRR17520069.6027256

SRR17520069.6027258
SRR17520069.6027260
SRR17520069.6027262
SRR17520069.6027264
SRR17520069.6027266
SRR17520069.6027268
SRR17520069.6027270
SRR17520069.6027272
SRR17520069.6027274
SRR17520069.6027276
SRR17520069.6027278
SRR17520069.6027280
SRR17520069.6027282
SRR17520069.6027284
SRR17520069.6027286
SRR17520069.6027288
SRR17520069.6027290
SRR17520069.6028450
SRR17520069.6028452
SRR17520069.6028454
SRR17520069.6028456
SRR17520069.6028458
SRR17520069.6028460
SRR17520069.6028462
SRR17520069.6030376
SRR17520069.6030378
SRR17520069.6030380
SRR17520069.6030384
SRR17520069.6030386
SRR17520069.6030388
SRR17520069.6030390
SRR17520069.6030392
SRR17520069.6030394
SRR17520069.6030396
SRR17520069.6031857
SRR17520069.6031859
SRR17520069.6031861
SRR17520069.6031863
SRR17520069.6031865
SRR17520069.6031867
SRR17520069.6031869
SRR17520069.6031871
SRR17520069.6031873
SRR17520069.6031886
SRR17520069.6031888
SRR17520069.6031892
SRR17520069.6031894

SRR17520069.6031896
SRR17520069.6031898
SRR17520069.6031900
SRR17520069.6031902
SRR17520069.6031904
SRR17520069.6031906
SRR17520069.6031908
SRR17520069.6031910
SRR17520069.6031912
SRR17520069.6031914
SRR17520069.6031916
SRR17520069.6031918
SRR17520069.6031920
SRR17520069.6031922
SRR17520069.6031924
SRR17520069.6031926
SRR17520069.6031928
SRR17520069.6031930
SRR17520069.6031932
SRR17520069.6031934
SRR17520069.6031936
SRR17520069.6031940
SRR17520069.6031942
SRR17520069.6031944
SRR17520069.6031946
SRR17520069.6031948
SRR17520069.6031952
SRR17520069.6031954
SRR17520069.6031956
SRR17520069.6031958
SRR17520069.6031960
SRR17520069.6032563
SRR17520069.6034064
SRR17520069.6034066
SRR17520069.6034068
SRR17520069.6034070
SRR17520069.6034072
SRR17520069.6034074
SRR17520069.6034076
SRR17520069.6034078
SRR17520069.6034080
SRR17520069.6034082
SRR17520069.6034084
SRR17520069.6034086
SRR17520069.6034088
SRR17520069.6034090
SRR17520069.6034092

SRR17520069.6034094
SRR17520069.6041366
SRR17520069.6041368
SRR17520069.6041370
SRR17520069.6041372
SRR17520069.6041374
SRR17520069.6041376
SRR17520069.6041378
SRR17520069.6041380
SRR17520069.6041382
SRR17520069.6041384
SRR17520069.6041386
SRR17520069.6041388
SRR17520069.6041390
SRR17520069.6041392
SRR17520069.6041523
SRR17520069.6041527
SRR17520069.6041531
SRR17520069.6041533
SRR17520069.6041535
SRR17520069.6041537
SRR17520069.6041539
SRR17520069.6041541
SRR17520069.6041543
SRR17520069.6041545
SRR17520069.6041547
SRR17520069.6041549
SRR17520069.6041551
SRR17520069.6041553
SRR17520069.6041557
SRR17520069.6041559
SRR17520069.6041561
SRR17520069.6041563
SRR17520069.6041565
SRR17520069.6041569
SRR17520069.6041571
SRR17520069.6041573
SRR17520069.6041575
SRR17520069.6045230
SRR17520069.6045765
SRR17520069.6045767
SRR17520069.6045769
SRR17520069.6045771
SRR17520069.6045773
SRR17520069.6045775
SRR17520069.6045777
SRR17520069.6048685

SRR17520069.6048774
SRR17520069.6048776
SRR17520069.6048778
SRR17520069.6048780
SRR17520069.6048782
SRR17520069.6048784
SRR17520069.6048786
SRR17520069.6048788
SRR17520069.6048790
SRR17520069.6048792
SRR17520069.6048794
SRR17520069.6048796
SRR17520069.6048798
SRR17520069.6048800
SRR17520069.6048804
SRR17520069.6048806
SRR17520069.6048808
SRR17520069.6048810
SRR17520069.6048812
SRR17520069.6048814
SRR17520069.6048816
SRR17520069.6048818
SRR17520069.6048820
SRR17520069.6048822
SRR17520069.6048824
SRR17520069.6048826
SRR17520069.6048828
SRR17520069.6048830
SRR17520069.6048832
SRR17520069.6048834
SRR17520069.6048836
SRR17520069.6049135
SRR17520069.6049137
SRR17520069.6049141
SRR17520069.6049143
SRR17520069.6049145
SRR17520069.6049147
SRR17520069.6049149
SRR17520069.6049151
SRR17520069.6049153
SRR17520069.6049155
SRR17520069.6049157
SRR17520069.6049159
SRR17520069.6049161
SRR17520069.6049163
SRR17520069.6051366
SRR17520069.6051368

SRR17520069.6051370
SRR17520069.6051372
SRR17520069.6051374
SRR17520069.6051376
SRR17520069.6051378
SRR17520069.6054829
SRR17520069.6056894
SRR17520069.6056896
SRR17520069.6056898
SRR17520069.6056902
SRR17520069.6056904
SRR17520069.6056906
SRR17520069.6056908
SRR17520069.6056912
SRR17520069.6057112
SRR17520069.6058470
SRR17520069.6058472
SRR17520069.6058476
SRR17520069.6058484
SRR17520069.6058486
SRR17520069.6058492
SRR17520069.6058494
SRR17520069.6058496
SRR17520069.6058498
SRR17520069.6058500
SRR17520069.6058504
SRR17520069.6058506
SRR17520069.6058508
SRR17520069.6067346
SRR17520069.6072421
SRR17520069.6072423
SRR17520069.6072427
SRR17520069.6072429
SRR17520069.6072431
SRR17520069.6072433
SRR17520069.6072435
SRR17520069.6072437
SRR17520069.6072439
SRR17520069.6072441
SRR17520069.6072443
SRR17520069.6072445
SRR17520069.6072447
SRR17520069.6072449
SRR17520069.6072453
SRR17520069.6072455
SRR17520069.6072457
SRR17520069.6072459

SRR17520069.6072463
SRR17520069.6072465
SRR17520069.6072467
SRR17520069.6072469
SRR17520069.6072475
SRR17520069.6072477
SRR17520069.6072479
SRR17520069.6072483
SRR17520069.6072485
SRR17520069.6072489
SRR17520069.6072491
SRR17520069.6072495
SRR17520069.6072497
SRR17520069.6072499
SRR17520069.6072501
SRR17520069.6072503
SRR17520069.6072505
SRR17520069.6072507
SRR17520069.6072509
SRR17520069.6072511
SRR17520069.6072513
SRR17520069.6072515
SRR17520069.6072521
SRR17520069.6072523
SRR17520069.6072525
SRR17520069.6072529
SRR17520069.6074406
SRR17520069.6074408
SRR17520069.6074410
SRR17520069.6074414
SRR17520069.6074416
SRR17520069.6074420
SRR17520069.6074422
SRR17520069.6074424
SRR17520069.6076848
SRR17520069.6080165
SRR17520069.6080167
SRR17520069.6080171
SRR17520069.6085056
SRR17520069.6085058
SRR17520069.6085060
SRR17520069.6085062
SRR17520069.6085064
SRR17520069.6085066
SRR17520069.6085068
SRR17520069.6085074
SRR17520069.6085076

SRR17520069.6085078
SRR17520069.6085080
SRR17520069.6085082
SRR17520069.6093547
SRR17520069.6093549
SRR17520069.6093551
SRR17520069.6093553
SRR17520069.6093555
SRR17520069.6093557
SRR17520069.6093559
SRR17520069.6093561
SRR17520069.6093563
SRR17520069.6093565
SRR17520069.6093567
SRR17520069.6093569
SRR17520069.6093571
SRR17520069.6093573
SRR17520069.6093575
SRR17520069.6093577
SRR17520069.6093579
SRR17520069.6093581
SRR17520069.6093583
SRR17520069.6093585
SRR17520069.6093587
SRR17520069.6093589
SRR17520069.6093591
SRR17520069.6093593
SRR17520069.6093597
SRR17520069.6093599
SRR17520069.6093601
SRR17520069.6093603
SRR17520069.6093605
SRR17520069.6093607
SRR17520069.6096914
SRR17520069.6096916
SRR17520069.6096918
SRR17520069.6096920
SRR17520069.6096922
SRR17520069.6096924
SRR17520069.6096926
SRR17520069.6096928
SRR17520069.6096930
SRR17520069.6096932
SRR17520069.6096934
SRR17520069.6096936
SRR17520069.6097160
SRR17520069.6097165

SRR17520069.6097171
SRR17520069.6097173
SRR17520069.6097177
SRR17520069.6097179
SRR17520069.6097181
SRR17520069.6097183
SRR17520069.6097185
SRR17520069.6097187
SRR17520069.6097189
SRR17520069.6097191
SRR17520069.6097193
SRR17520069.6097195
SRR17520069.6097197
SRR17520069.6097199
SRR17520069.6097858
SRR17520069.6097860
SRR17520069.6097862
SRR17520069.6097864
SRR17520069.6097866
SRR17520069.6097868
SRR17520069.6097870
SRR17520069.6097872
SRR17520069.6097874
SRR17520069.6099965
SRR17520069.6099967
SRR17520069.6099969
SRR17520069.6099971
SRR17520069.6099973
SRR17520069.6099975
SRR17520069.6099977
SRR17520069.6099979
SRR17520069.6099981
SRR17520069.6099983
SRR17520069.6099985
SRR17520069.6099987
SRR17520069.6099989
SRR17520069.6099991
SRR17520069.6099993
SRR17520069.6099995
SRR17520069.6099997
SRR17520069.6099999
SRR17520069.6100001
SRR17520069.6100003
SRR17520069.6100005
SRR17520069.6100007
SRR17520069.6100009
SRR17520069.6100011

SRR17520069.6100013
SRR17520069.6100015
SRR17520069.6100019
SRR17520069.6100021
SRR17520069.6100023
SRR17520069.6100025
SRR17520069.6100027
SRR17520069.6100029
SRR17520069.6100031
SRR17520069.6100033
SRR17520069.6100035
SRR17520069.6100039
SRR17520069.6100041
SRR17520069.6100043
SRR17520069.6100045
SRR17520069.6100047
SRR17520069.6100049
SRR17520069.6100051
SRR17520069.6100053
SRR17520069.6100055
SRR17520069.6100057
SRR17520069.6100059
SRR17520069.6100061
SRR17520069.6100063
SRR17520069.6100065
SRR17520069.6100067
SRR17520069.6104390
SRR17520069.6104392
SRR17520069.6104394
SRR17520069.6104396
SRR17520069.6104398
SRR17520069.6104400
SRR17520069.6104402
SRR17520069.6106359
SRR17520069.6106361
SRR17520069.6106363
SRR17520069.6106365
SRR17520069.6106367
SRR17520069.6106369
SRR17520069.6106371
SRR17520069.6106373
SRR17520069.6106375
SRR17520069.6106377
SRR17520069.6106379
SRR17520069.6106381
SRR17520069.6106383
SRR17520069.6106385

SRR17520069.6106387
SRR17520069.6106389
SRR17520069.6106391
SRR17520069.6106393
SRR17520069.6106395
SRR17520069.6106397
SRR17520069.6106399
SRR17520069.6106401
SRR17520069.6106403
SRR17520069.6106405
SRR17520069.6106407
SRR17520069.6106409
SRR17520069.6106411
SRR17520069.6106413
SRR17520069.6106415
SRR17520069.6106417
SRR17520069.6106419
SRR17520069.6106421
SRR17520069.6106423
SRR17520069.6106425
SRR17520069.6106427
SRR17520069.6106429
SRR17520069.6106431
SRR17520069.6106433
SRR17520069.6106435
SRR17520069.6106437
SRR17520069.6106439
SRR17520069.6106441
SRR17520069.6106443
SRR17520069.6106445
SRR17520069.6106447
SRR17520069.6106449
SRR17520069.6106451
SRR17520069.6106453
SRR17520069.6106455
SRR17520069.6106457
SRR17520069.6106459
SRR17520069.6106461
SRR17520069.6106463
SRR17520069.6106465
SRR17520069.6106467
SRR17520069.6106469
SRR17520069.6106471
SRR17520069.6106473
SRR17520069.6106475
SRR17520069.6106477
SRR17520069.6106479

SRR17520069.6106481
SRR17520069.6106483
SRR17520069.6106485
SRR17520069.6106487
SRR17520069.6106489
SRR17520069.6106491
SRR17520069.6106493
SRR17520069.6106495
SRR17520069.6106497
SRR17520069.6106499
SRR17520069.6106501
SRR17520069.6106503
SRR17520069.6106505
SRR17520069.6106507
SRR17520069.6106509
SRR17520069.6106511
SRR17520069.6106513
SRR17520069.6106515
SRR17520069.6106517
SRR17520069.6106519
SRR17520069.6106521
SRR17520069.6106525
SRR17520069.6106527
SRR17520069.6106529
SRR17520069.6106531
SRR17520069.6106533
SRR17520069.6106535
SRR17520069.6108740
SRR17520069.6108744
SRR17520069.6108746
SRR17520069.6108748
SRR17520069.6108752
SRR17520069.6108756
SRR17520069.6108760
SRR17520069.6108764
SRR17520069.6111304
SRR17520069.6111308
SRR17520069.6111310
SRR17520069.6111312
SRR17520069.6112671
SRR17520069.6113947
SRR17520069.6113949
SRR17520069.6113951
SRR17520069.6113953
SRR17520069.6117525
SRR17520069.6117527
SRR17520069.6117529

SRR17520069.6117531
SRR17520069.6117533
SRR17520069.6117535
SRR17520069.6117537
SRR17520069.6117539
SRR17520069.6117541
SRR17520069.6117543
SRR17520069.6117545
SRR17520069.6117547
SRR17520069.6117549
SRR17520069.6117551
SRR17520069.6117553
SRR17520069.6117555
SRR17520069.6117557
SRR17520069.6117559
SRR17520069.6117561
SRR17520069.6117563
SRR17520069.6117565
SRR17520069.6117571
SRR17520069.6117573
SRR17520069.6117575
SRR17520069.6117577
SRR17520069.6117579
SRR17520069.6117581
SRR17520069.6117804
SRR17520069.6117806
SRR17520069.6117808
SRR17520069.6117810
SRR17520069.6117812
SRR17520069.6117814
SRR17520069.6117816
SRR17520069.6117818
SRR17520069.6117820
SRR17520069.6117822
SRR17520069.6117824
SRR17520069.6117826
SRR17520069.6118788
SRR17520069.6124141
SRR17520069.6124143
SRR17520069.6124147
SRR17520069.6124149
SRR17520069.6124151
SRR17520069.6124153
SRR17520069.6124155
SRR17520069.6124157
SRR17520069.6124159
SRR17520069.6124161

SRR17520069.6124163
SRR17520069.6124165
SRR17520069.6124167
SRR17520069.6124169
SRR17520069.6124171
SRR17520069.6124173
SRR17520069.6124175
SRR17520069.6124177
SRR17520069.6124179
SRR17520069.6124181
SRR17520069.6124185
SRR17520069.6124187
SRR17520069.6124189
SRR17520069.6124191
SRR17520069.6124193
SRR17520069.6124199
SRR17520069.6124201
SRR17520069.6124203
SRR17520069.6124205
SRR17520069.6124207
SRR17520069.6124209
SRR17520069.6124211
SRR17520069.6124213
SRR17520069.6124215
SRR17520069.6124217
SRR17520069.6124219
SRR17520069.6124221
SRR17520069.6124223
SRR17520069.6124225
SRR17520069.6124227
SRR17520069.6124229
SRR17520069.6124231
SRR17520069.6124233
SRR17520069.6124235
SRR17520069.6127098
SRR17520069.6127100
SRR17520069.6127102
SRR17520069.6127104
SRR17520069.6127106
SRR17520069.6127108
SRR17520069.6127110
SRR17520069.6127112
SRR17520069.6127114
SRR17520069.6127116
SRR17520069.6127119
SRR17520069.6127121
SRR17520069.6127123

SRR17520069.6127125
SRR17520069.6127127
SRR17520069.6127129
SRR17520069.6127417
SRR17520069.6127419
SRR17520069.6135011
SRR17520069.6135013
SRR17520069.6135015
SRR17520069.6135017
SRR17520069.6135019
SRR17520069.6135021
SRR17520069.6135023
SRR17520069.6135025
SRR17520069.6135027
SRR17520069.6135029
SRR17520069.6135031
SRR17520069.6135035
SRR17520069.6135037
SRR17520069.6135039
SRR17520069.6135041
SRR17520069.6136275
SRR17520069.6136297
SRR17520069.6138140
SRR17520069.6138142
SRR17520069.6138144
SRR17520069.6138146
SRR17520069.6138148
SRR17520069.6138150
SRR17520069.6138152
SRR17520069.6138154
SRR17520069.6138156
SRR17520069.6138158
SRR17520069.6138950
SRR17520069.6138952
SRR17520069.6138954
SRR17520069.6138956
SRR17520069.6138958
SRR17520069.6138960
SRR17520069.6138962
SRR17520069.6144126
SRR17520069.6144128
SRR17520069.6144132
SRR17520069.6146889
SRR17520069.6146891
SRR17520069.6146893
SRR17520069.6146895
SRR17520069.6146897

SRR17520069.6146899
SRR17520069.6146901
SRR17520069.6146905
SRR17520069.6146907
SRR17520069.6146909
SRR17520069.6146911
SRR17520069.6146913
SRR17520069.6147687
SRR17520069.6147689
SRR17520069.6147691
SRR17520069.6147693
SRR17520069.6147695
SRR17520069.6147697
SRR17520069.6147699
SRR17520069.6147701
SRR17520069.6147703
SRR17520069.6147705
SRR17520069.6147707
SRR17520069.6147709
SRR17520069.6147711
SRR17520069.6147713
SRR17520069.6147715
SRR17520069.6147717
SRR17520069.6147719
SRR17520069.6147723
SRR17520069.6147725
SRR17520069.6147727
SRR17520069.6147729
SRR17520069.6147731
SRR17520069.6147733
SRR17520069.6147735
SRR17520069.6147737
SRR17520069.6147739
SRR17520069.6147741
SRR17520069.6147743
SRR17520069.6148566
SRR17520069.6148568
SRR17520069.6148570
SRR17520069.6149732
SRR17520069.6149736
SRR17520069.6149738
SRR17520069.6155945
SRR17520069.6155947
SRR17520069.6155949
SRR17520069.6155951
SRR17520069.6155953
SRR17520069.6155955

SRR17520069.6155957
SRR17520069.6155959
SRR17520069.6155961
SRR17520069.6155963
SRR17520069.6155965
SRR17520069.6155967
SRR17520069.6155969
SRR17520069.6155971
SRR17520069.6155973
SRR17520069.6155975
SRR17520069.6155977
SRR17520069.6155979
SRR17520069.6155981
SRR17520069.6155983
SRR17520069.6155987
SRR17520069.6155989
SRR17520069.6155991
SRR17520069.6155993
SRR17520069.6155995
SRR17520069.6155997
SRR17520069.6155999
SRR17520069.6156001
SRR17520069.6156003
SRR17520069.6156005
SRR17520069.6156007
SRR17520069.6156009
SRR17520069.6156011
SRR17520069.6156013
SRR17520069.6156015
SRR17520069.6156017
SRR17520069.6156019
SRR17520069.6162828
SRR17520069.6162830
SRR17520069.6162832
SRR17520069.6162836
SRR17520069.6162838
SRR17520069.6162842
SRR17520069.6162844
SRR17520069.6164151
SRR17520069.6164153
SRR17520069.6164155
SRR17520069.6164157
SRR17520069.6164161
SRR17520069.6164163
SRR17520069.6164165
SRR17520069.6164167
SRR17520069.6164169

SRR17520069.6164171
SRR17520069.6164173
SRR17520069.6169733
SRR17520069.6170523
SRR17520069.6170592
SRR17520069.6170594
SRR17520069.6170596
SRR17520069.6170598
SRR17520069.6170600
SRR17520069.6170602
SRR17520069.6170604
SRR17520069.6170606
SRR17520069.6170608
SRR17520069.6170610
SRR17520069.6170612
SRR17520069.6170614
SRR17520069.6170616
SRR17520069.6170618
SRR17520069.6170620
SRR17520069.6170622
SRR17520069.6170624
SRR17520069.6170626
SRR17520069.6170628
SRR17520069.6170630
SRR17520069.6170632
SRR17520069.6170634
SRR17520069.6170636
SRR17520069.6170638
SRR17520069.6170640
SRR17520069.6170642
SRR17520069.6170644
SRR17520069.6170646
SRR17520069.6170648
SRR17520069.6170650
SRR17520069.6170652
SRR17520069.6171244
SRR17520069.6171246
SRR17520069.6171248
SRR17520069.6171250
SRR17520069.6171252
SRR17520069.6171254
SRR17520069.6171256
SRR17520069.6171258
SRR17520069.6171260
SRR17520069.6171262
SRR17520069.6171264
SRR17520069.6171266

SRR17520069.6171268
SRR17520069.6171270
SRR17520069.6171272
SRR17520069.6171274
SRR17520069.6171276
SRR17520069.6171278
SRR17520069.6171280
SRR17520069.6171282
SRR17520069.6171284
SRR17520069.6171286
SRR17520069.6171288
SRR17520069.6171290
SRR17520069.6171292
SRR17520069.6171294
SRR17520069.6171296
SRR17520069.6171298
SRR17520069.6171300
SRR17520069.6171302
SRR17520069.6171304
SRR17520069.6171306
SRR17520069.6171308
SRR17520069.6171310
SRR17520069.6171312
SRR17520069.6171314
SRR17520069.6171316
SRR17520069.6171318
SRR17520069.6171320
SRR17520069.6171322
SRR17520069.6171324
SRR17520069.6171326
SRR17520069.6171328
SRR17520069.6171330
SRR17520069.6171332
SRR17520069.6171334
SRR17520069.6171336
SRR17520069.6171338
SRR17520069.6171340
SRR17520069.6171342
SRR17520069.6171344
SRR17520069.6171346
SRR17520069.6173809
SRR17520069.6173811
SRR17520069.6173813
SRR17520069.6173815
SRR17520069.6173817
SRR17520069.6173819
SRR17520069.6173821

SRR17520069.6173823
SRR17520069.6173825
SRR17520069.6173827
SRR17520069.6173829
SRR17520069.6173831
SRR17520069.6173833
SRR17520069.6173835
SRR17520069.6182522
SRR17520069.6185095
SRR17520069.6185097
SRR17520069.6185099
SRR17520069.6185107
SRR17520069.6185109
SRR17520069.6188032
SRR17520069.6188034
SRR17520069.6188036
SRR17520069.6188038
SRR17520069.6188040
SRR17520069.6188042
SRR17520069.6188044
SRR17520069.6188046
SRR17520069.6188048
SRR17520069.6188050
SRR17520069.6188052
SRR17520069.6188054
SRR17520069.6188056
SRR17520069.6188058
SRR17520069.6188062
SRR17520069.6188064
SRR17520069.6188066
SRR17520069.6188068
SRR17520069.6188070
SRR17520069.6188384
SRR17520069.6188386
SRR17520069.6188388
SRR17520069.6188390
SRR17520069.6188392
SRR17520069.6188394
SRR17520069.6188396
SRR17520069.6188398
SRR17520069.6188400
SRR17520069.6188402
SRR17520069.6188404
SRR17520069.6188406
SRR17520069.6188410
SRR17520069.6188412
SRR17520069.6188414

SRR17520069.6188416
SRR17520069.6188418
SRR17520069.6188420
SRR17520069.6188422
SRR17520069.6188424
SRR17520069.6188426
SRR17520069.6188428
SRR17520069.6188430
SRR17520069.6188432
SRR17520069.6188434
SRR17520069.6188436
SRR17520069.6188438
SRR17520069.6188440
SRR17520069.6188442
SRR17520069.6188444
SRR17520069.6188446
SRR17520069.6188448
SRR17520069.6188450
SRR17520069.6188452
SRR17520069.6188454
SRR17520069.6188456
SRR17520069.6188458
SRR17520069.6188460
SRR17520069.6189059
SRR17520069.6189061
SRR17520069.6190215
SRR17520069.6190217
SRR17520069.6190219
SRR17520069.6190221
SRR17520069.6190223
SRR17520069.6190225
SRR17520069.6190227
SRR17520069.6190229
SRR17520069.6191612
SRR17520069.6191614
SRR17520069.6191616
SRR17520069.6191618
SRR17520069.6191620
SRR17520069.6191622
SRR17520069.6191624
SRR17520069.6191626
SRR17520069.6191628
SRR17520069.6191630
SRR17520069.6191634
SRR17520069.6192439
SRR17520069.6194924
SRR17520069.6194926

SRR17520069.6194928
SRR17520069.6194932
SRR17520069.6194934
SRR17520069.6194936
SRR17520069.6194938
SRR17520069.6194940
SRR17520069.6194942
SRR17520069.6194944
SRR17520069.6198354
SRR17520069.6198356
SRR17520069.6198358
SRR17520069.6198362
SRR17520069.6198364
SRR17520069.6198366
SRR17520069.6198368
SRR17520069.6198370
SRR17520069.6198372
SRR17520069.6198374
SRR17520069.6198378
SRR17520069.6198380
SRR17520069.6198382
SRR17520069.6198384
SRR17520069.6198386
SRR17520069.6198388
SRR17520069.6198390
SRR17520069.6198392
SRR17520069.6198394
SRR17520069.6198396
SRR17520069.6198398
SRR17520069.6198400
SRR17520069.6198402
SRR17520069.6198404
SRR17520069.6198406
SRR17520069.6198410
SRR17520069.6198412
SRR17520069.6198414
SRR17520069.6198416
SRR17520069.6198422
SRR17520069.6198424
SRR17520069.6198426
SRR17520069.6198430
SRR17520069.6198432
SRR17520069.6198436
SRR17520069.6198438
SRR17520069.6198440
SRR17520069.6198442
SRR17520069.6198444

SRR17520069.6198446
SRR17520069.6198448
SRR17520069.6198450
SRR17520069.6198452
SRR17520069.6198454
SRR17520069.6198456
SRR17520069.6198458
SRR17520069.6198460
SRR17520069.6198462
SRR17520069.6198464
SRR17520069.6198466
SRR17520069.6198468
SRR17520069.6198470
SRR17520069.6198472
SRR17520069.6198476
SRR17520069.6198478
SRR17520069.6198480
SRR17520069.6198482
SRR17520069.6198484
SRR17520069.6198486
SRR17520069.6198488
SRR17520069.6198490
SRR17520069.6198492
SRR17520069.6198494
SRR17520069.6198496
SRR17520069.6200287
SRR17520069.6200289
SRR17520069.6200291
SRR17520069.6200293
SRR17520069.6200295
SRR17520069.6200297
SRR17520069.6200299
SRR17520069.6200301
SRR17520069.6200303
SRR17520069.6200305
SRR17520069.6200307
SRR17520069.6200309
SRR17520069.6200311
SRR17520069.6200313
SRR17520069.6200315
SRR17520069.6200317
SRR17520069.6200319
SRR17520069.6200321
SRR17520069.6200323
SRR17520069.6200325
SRR17520069.6200327
SRR17520069.6200329

SRR17520069.6200331
SRR17520069.6200333
SRR17520069.6200335
SRR17520069.6200337
SRR17520069.6200339
SRR17520069.6200343
SRR17520069.6200345
SRR17520069.6200347
SRR17520069.6200349
SRR17520069.6200351
SRR17520069.6200353
SRR17520069.6200355
SRR17520069.6200357
SRR17520069.6200359
SRR17520069.6200361
SRR17520069.6200363
SRR17520069.6200365
SRR17520069.6200367
SRR17520069.6200369
SRR17520069.6200371
SRR17520069.6200373
SRR17520069.6200375
SRR17520069.6200377
SRR17520069.6200379
SRR17520069.6200381
SRR17520069.6200383
SRR17520069.6200385
SRR17520069.6200387
SRR17520069.6200389
SRR17520069.6200391
SRR17520069.6200393
SRR17520069.6200397
SRR17520069.6200399
SRR17520069.6200401
SRR17520069.6200403
SRR17520069.6200405
SRR17520069.6200407
SRR17520069.6200409
SRR17520069.6200411
SRR17520069.6200413
SRR17520069.6200415
SRR17520069.6200417
SRR17520069.6200421
SRR17520069.6200425
SRR17520069.6200427
SRR17520069.6200429
SRR17520069.6200431

SRR17520069.6200433
SRR17520069.6200435
SRR17520069.6200437
SRR17520069.6200439
SRR17520069.6200441
SRR17520069.6200443
SRR17520069.6200445
SRR17520069.6200447
SRR17520069.6200449
SRR17520069.6200451
SRR17520069.6200453
SRR17520069.6200455
SRR17520069.6200459
SRR17520069.6200461
SRR17520069.6200463
SRR17520069.6203225
SRR17520069.6203227
SRR17520069.6203229
SRR17520069.6203231
SRR17520069.6203233
SRR17520069.6203235
SRR17520069.6203237
SRR17520069.6203239
SRR17520069.6203241
SRR17520069.6203243
SRR17520069.6203245
SRR17520069.6203247
SRR17520069.6203249
SRR17520069.6203251
SRR17520069.6203253
SRR17520069.6203255
SRR17520069.6203257
SRR17520069.6203259
SRR17520069.6203261
SRR17520069.6203263
SRR17520069.6203265
SRR17520069.6203267
SRR17520069.6208866
SRR17520069.6208868
SRR17520069.6208870
SRR17520069.6208872
SRR17520069.6208874
SRR17520069.6208876
SRR17520069.6208878
SRR17520069.6208880
SRR17520069.6208882
SRR17520069.6208884

SRR17520069.6208886
SRR17520069.6208888
SRR17520069.6208890
SRR17520069.6208892
SRR17520069.6208894
SRR17520069.6208896
SRR17520069.6208898
SRR17520069.6208900
SRR17520069.6208902
SRR17520069.6208904
SRR17520069.6208906
SRR17520069.6208908
SRR17520069.6208910
SRR17520069.6208912
SRR17520069.6208914
SRR17520069.6208916
SRR17520069.6208918
SRR17520069.6209462
SRR17520069.6209464
SRR17520069.6209466
SRR17520069.6209468
SRR17520069.6210957
SRR17520069.6210959
SRR17520069.6210961
SRR17520069.6210963
SRR17520069.6210965
SRR17520069.6210967
SRR17520069.6210969
SRR17520069.6210971
SRR17520069.6210973
SRR17520069.6210975
SRR17520069.6210977
SRR17520069.6210979
SRR17520069.6210981
SRR17520069.6210983
SRR17520069.6210985
SRR17520069.6210987
SRR17520069.6210989
SRR17520069.6210991
SRR17520069.6210993
SRR17520069.6210995
SRR17520069.6210997
SRR17520069.6210999
SRR17520069.6211001
SRR17520069.6219186
SRR17520069.6220644
SRR17520069.6220646

SRR17520069.6222462
SRR17520069.6222464
SRR17520069.6222466
SRR17520069.6222468
SRR17520069.6222470
SRR17520069.6222472
SRR17520069.6222474
SRR17520069.6222476
SRR17520069.6222478
SRR17520069.6222480
SRR17520069.6222482
SRR17520069.6222484
SRR17520069.6222486
SRR17520069.6222488
SRR17520069.6222490
SRR17520069.6222492
SRR17520069.6222494
SRR17520069.6222496
SRR17520069.6222498
SRR17520069.6222500
SRR17520069.6222502
SRR17520069.6222504
SRR17520069.6222506
SRR17520069.6222508
SRR17520069.6222510
SRR17520069.6222512
SRR17520069.6222514
SRR17520069.6222516
SRR17520069.6222518
SRR17520069.6222520
SRR17520069.6222522
SRR17520069.6222524
SRR17520069.6222526
SRR17520069.6222528
SRR17520069.6222530
SRR17520069.6222532
SRR17520069.6222534
SRR17520069.6222536
SRR17520069.6222538
SRR17520069.6222540
SRR17520069.6222542
SRR17520069.6222544
SRR17520069.6222548
SRR17520069.6222550
SRR17520069.6222552
SRR17520069.6222554
SRR17520069.6222556

SRR17520069.6222558
SRR17520069.6222560
SRR17520069.6222562
SRR17520069.6222564
SRR17520069.6222566
SRR17520069.6222568
SRR17520069.6222572
SRR17520069.6222574
SRR17520069.6222576
SRR17520069.6222578
SRR17520069.6222580
SRR17520069.6222582
SRR17520069.6222584
SRR17520069.6222586
SRR17520069.6222588
SRR17520069.6222590
SRR17520069.6222592
SRR17520069.6222594
SRR17520069.6222596
SRR17520069.6222598
SRR17520069.6222600
SRR17520069.6222602
SRR17520069.6222604
SRR17520069.6222608
SRR17520069.6222610
SRR17520069.6222612
SRR17520069.6222614
SRR17520069.6222616
SRR17520069.6222618
SRR17520069.6225472
SRR17520069.6225749
SRR17520069.6225751
SRR17520069.6225753
SRR17520069.6225755
SRR17520069.6225757
SRR17520069.6225759
SRR17520069.6225761
SRR17520069.6225763
SRR17520069.6225765
SRR17520069.6225767
SRR17520069.6225769
SRR17520069.6225771
SRR17520069.6225773
SRR17520069.6225775
SRR17520069.6225777
SRR17520069.6225779
SRR17520069.6225781

SRR17520069.6225783
SRR17520069.6225785
SRR17520069.6225787
SRR17520069.6225789
SRR17520069.6225791
SRR17520069.6225793
SRR17520069.6225795
SRR17520069.6225797
SRR17520069.6225799
SRR17520069.6225801
SRR17520069.6225803
SRR17520069.6225805
SRR17520069.6225807
SRR17520069.6226997
SRR17520069.6226999
SRR17520069.6228486
SRR17520069.6228488
SRR17520069.6228490
SRR17520069.6228492
SRR17520069.6228494
SRR17520069.6228496
SRR17520069.6228498
SRR17520069.6228500
SRR17520069.6228502
SRR17520069.6228504
SRR17520069.6228506
SRR17520069.6228508
SRR17520069.6228510
SRR17520069.6228512
SRR17520069.6228946
SRR17520069.6228948
SRR17520069.6228950
SRR17520069.6228952
SRR17520069.6228954
SRR17520069.6228956
SRR17520069.6228958
SRR17520069.6228960
SRR17520069.6228962
SRR17520069.6228964
SRR17520069.6228966
SRR17520069.6228968
SRR17520069.6228970
SRR17520069.6228972
SRR17520069.6228974
SRR17520069.6228976
SRR17520069.6228978
SRR17520069.6228980

SRR17520069.6228982
SRR17520069.6228984
SRR17520069.6228986
SRR17520069.6228988
SRR17520069.6228990
SRR17520069.6228992
SRR17520069.6228994
SRR17520069.6228996
SRR17520069.6228998
SRR17520069.6229000
SRR17520069.6229002
SRR17520069.6232477
SRR17520069.6232481
SRR17520069.6232483
SRR17520069.6232487
SRR17520069.6232489
SRR17520069.6232491
SRR17520069.6232493
SRR17520069.6232495
SRR17520069.6232499
SRR17520069.6232501
SRR17520069.6232505
SRR17520069.6232507
SRR17520069.6232509
SRR17520069.6232511
SRR17520069.6232513
SRR17520069.6232515
SRR17520069.6232517
SRR17520069.6232519
SRR17520069.6232521
SRR17520069.6232523
SRR17520069.6232525
SRR17520069.6232527
SRR17520069.6232529
SRR17520069.6232531
SRR17520069.6232533
SRR17520069.6232535
SRR17520069.6232537
SRR17520069.6232539
SRR17520069.6232543
SRR17520069.6232545
SRR17520069.6232547
SRR17520069.6237034
SRR17520069.6237036
SRR17520069.6237038
SRR17520069.6237040
SRR17520069.6237042

SRR17520069.6237048
SRR17520069.6237052
SRR17520069.6237054
SRR17520069.6237056
SRR17520069.6237058
SRR17520069.6237060
SRR17520069.6237062
SRR17520069.6237066
SRR17520069.6237068
SRR17520069.6237070
SRR17520069.6237072
SRR17520069.6237074
SRR17520069.6237076
SRR17520069.6237078
SRR17520069.6237080
SRR17520069.6237082
SRR17520069.6237086
SRR17520069.6237088
SRR17520069.6237090
SRR17520069.6237092
SRR17520069.6246965
SRR17520069.6248935
SRR17520069.6248937
SRR17520069.6248939
SRR17520069.6248941
SRR17520069.6248943
SRR17520069.6248945
SRR17520069.6248947
SRR17520069.6248949
SRR17520069.6248951
SRR17520069.6248953
SRR17520069.6248955
SRR17520069.6248957
SRR17520069.6248959
SRR17520069.6248961
SRR17520069.6248963
SRR17520069.6248965
SRR17520069.6248967
SRR17520069.6248969
SRR17520069.6252364
SRR17520069.6252366
SRR17520069.6252368
SRR17520069.6252370
SRR17520069.6252372
SRR17520069.6252374
SRR17520069.6252376
SRR17520069.6252378

SRR17520069.6252380
SRR17520069.6252382
SRR17520069.6252384
SRR17520069.6252386
SRR17520069.6252388
SRR17520069.6252390
SRR17520069.6252392
SRR17520069.6252394
SRR17520069.6252396
SRR17520069.6252398
SRR17520069.6252400
SRR17520069.6252402
SRR17520069.6252404
SRR17520069.6252406
SRR17520069.6252408
SRR17520069.6252410
SRR17520069.6252412
SRR17520069.6252414
SRR17520069.6252416
SRR17520069.6252420
SRR17520069.6252422
SRR17520069.6252424
SRR17520069.6252426
SRR17520069.6252428
SRR17520069.6252430
SRR17520069.6252432
SRR17520069.6252434
SRR17520069.6252436
SRR17520069.6252438
SRR17520069.6252440
SRR17520069.6252442
SRR17520069.6252444
SRR17520069.6252446
SRR17520069.6252448
SRR17520069.6252450
SRR17520069.6252454
SRR17520069.6252456
SRR17520069.6252458
SRR17520069.6252460
SRR17520069.6252462
SRR17520069.6252464
SRR17520069.6262466
SRR17520069.6262468
SRR17520069.6262470
SRR17520069.6262472
SRR17520069.6262474
SRR17520069.6262476

SRR17520069.6262478
SRR17520069.6271868
SRR17520069.6271870
SRR17520069.6271872
SRR17520069.6271874
SRR17520069.6271876
SRR17520069.6271878
SRR17520069.6271880
SRR17520069.6271885
SRR17520069.6271889
SRR17520069.6271891
SRR17520069.6271899
SRR17520069.6271903
SRR17520069.6278851
SRR17520069.6278853
SRR17520069.6278855
SRR17520069.6283356
SRR17520069.6283358
SRR17520069.6283360
SRR17520069.6283362
SRR17520069.6283364
SRR17520069.6283366
SRR17520069.6283372
SRR17520069.6283374
SRR17520069.6283380
SRR17520069.6283382
SRR17520069.6283384
SRR17520069.6283388
SRR17520069.6283392
SRR17520069.6283394
SRR17520069.6283396
SRR17520069.6283398
SRR17520069.6283400
SRR17520069.6283402
SRR17520069.6283404
SRR17520069.6283406
SRR17520069.6283410
SRR17520069.6283412
SRR17520069.6283414
SRR17520069.6283416
SRR17520069.6283420
SRR17520069.6283422
SRR17520069.6286889
SRR17520069.6286891
SRR17520069.6286893
SRR17520069.6286895
SRR17520069.6286897

SRR17520069.6286899
SRR17520069.6286901
SRR17520069.6286903
SRR17520069.6286905
SRR17520069.6286907
SRR17520069.6286909
SRR17520069.6286911
SRR17520069.6288632
SRR17520069.6289449
SRR17520069.6289451
SRR17520069.6289453
SRR17520069.6289455
SRR17520069.6289457
SRR17520069.6289459
SRR17520069.6289461
SRR17520069.6289463
SRR17520069.6289465
SRR17520069.6289467
SRR17520069.6289469
SRR17520069.6289471
SRR17520069.6289473
SRR17520069.6289475
SRR17520069.6289477
SRR17520069.6289479
SRR17520069.6289481
SRR17520069.6289483
SRR17520069.6289485
SRR17520069.6289487
SRR17520069.6289489
SRR17520069.6289491
SRR17520069.6289493
SRR17520069.6289495
SRR17520069.6289497
SRR17520069.6289499
SRR17520069.6289501
SRR17520069.6289503
SRR17520069.6289505
SRR17520069.6289507
SRR17520069.6289509
SRR17520069.6289511
SRR17520069.6289513
SRR17520069.6289941
SRR17520069.6291724
SRR17520069.6291726
SRR17520069.6291728
SRR17520069.6291730
SRR17520069.6291732

SRR17520069.6291734
SRR17520069.6291736
SRR17520069.6291738
SRR17520069.6291740
SRR17520069.6291742
SRR17520069.6291744
SRR17520069.6291746
SRR17520069.6291748
SRR17520069.6291750
SRR17520069.6291754
SRR17520069.6291756
SRR17520069.6291758
SRR17520069.6291760
SRR17520069.6291762
SRR17520069.6291764
SRR17520069.6291766
SRR17520069.6291774
SRR17520069.6291776
SRR17520069.6294103
SRR17520069.6296567
SRR17520069.6296569
SRR17520069.6296571
SRR17520069.6296575
SRR17520069.6296577
SRR17520069.6296579
SRR17520069.6296581
SRR17520069.6296583
SRR17520069.6296585
SRR17520069.6296587
SRR17520069.6296593
SRR17520069.6296597
SRR17520069.6296599
SRR17520069.6298939
SRR17520069.6298941
SRR17520069.6298943
SRR17520069.6298945
SRR17520069.6298947
SRR17520069.6298949
SRR17520069.6298951
SRR17520069.6298953
SRR17520069.6298955
SRR17520069.6298957
SRR17520069.6298959
SRR17520069.6298961
SRR17520069.6298963
SRR17520069.6298965
SRR17520069.6298967

SRR17520069.6298969
SRR17520069.6302991
SRR17520069.6302993
SRR17520069.6302995
SRR17520069.6302999
SRR17520069.6303001
SRR17520069.6303003
SRR17520069.6303005
SRR17520069.6303007
SRR17520069.6303009
SRR17520069.6303011
SRR17520069.6303013
SRR17520069.6303015
SRR17520069.6303017
SRR17520069.6316687
SRR17520069.6316689
SRR17520069.6323520
SRR17520069.6323522
SRR17520069.6323524
SRR17520069.6323526
SRR17520069.6323528
SRR17520069.6323530
SRR17520069.6323532
SRR17520069.6323534
SRR17520069.6323536
SRR17520069.6323538
SRR17520069.6323540
SRR17520069.6323542
SRR17520069.6323544
SRR17520069.6323546
SRR17520069.6323548
SRR17520069.6323550
SRR17520069.6323552
SRR17520069.6323554
SRR17520069.6323556
SRR17520069.6323558
SRR17520069.6323560
SRR17520069.6323562
SRR17520069.6323564
SRR17520069.6323566
SRR17520069.6323568
SRR17520069.6323570
SRR17520069.6323572
SRR17520069.6323574
SRR17520069.6323576
SRR17520069.6323578
SRR17520069.6323580

SRR17520069.6323582
SRR17520069.6323584
SRR17520069.6323586
SRR17520069.6323588
SRR17520069.6323590
SRR17520069.6323592
SRR17520069.6323594
SRR17520069.6323596
SRR17520069.6323598
SRR17520069.6323602
SRR17520069.6323604
SRR17520069.6323606
SRR17520069.6323608
SRR17520069.6323610
SRR17520069.6323612
SRR17520069.6323614
SRR17520069.6323616
SRR17520069.6323618
SRR17520069.6339566
SRR17520069.6339572
SRR17520069.6341485
SRR17520069.6341487
SRR17520069.6341491
SRR17520069.6341493
SRR17520069.6341495
SRR17520069.6341497
SRR17520069.6341499
SRR17520069.6341501
SRR17520069.6341503
SRR17520069.6341505
SRR17520069.6341507
SRR17520069.6341509
SRR17520069.6341511
SRR17520069.6341513
SRR17520069.6341515
SRR17520069.6341517
SRR17520069.6341519
SRR17520069.6341521
SRR17520069.6341523
SRR17520069.6341525
SRR17520069.6341527
SRR17520069.6341529
SRR17520069.6341531
SRR17520069.6341533
SRR17520069.6341535
SRR17520069.6341537
SRR17520069.6341541

SRR17520069.6341543
SRR17520069.6341545
SRR17520069.6341547
SRR17520069.6341549
SRR17520069.6341551
SRR17520069.6341553
SRR17520069.6341555
SRR17520069.6341557
SRR17520069.6341559
SRR17520069.6342825
SRR17520069.6342829
SRR17520069.6342831
SRR17520069.6342833
SRR17520069.6342835
SRR17520069.6343663
SRR17520069.6343665
SRR17520069.6343667
SRR17520069.6343669
SRR17520069.6343671
SRR17520069.6343673
SRR17520069.6343675
SRR17520069.6343677
SRR17520069.6343679
SRR17520069.6343681
SRR17520069.6343683
SRR17520069.6343687
SRR17520069.6343689
SRR17520069.6343691
SRR17520069.6343693
SRR17520069.6343695
SRR17520069.6343697
SRR17520069.6343699
SRR17520069.6343701
SRR17520069.6343703
SRR17520069.6343707
SRR17520069.6343709
SRR17520069.6343711
SRR17520069.6343713
SRR17520069.6343715
SRR17520069.6343717
SRR17520069.6343719
SRR17520069.6343721
SRR17520069.6343725
SRR17520069.6343727
SRR17520069.6349865
SRR17520069.6350190
SRR17520069.6350196

SRR17520069.6350198
SRR17520069.6350200
SRR17520069.6350202
SRR17520069.6350204
SRR17520069.6350206
SRR17520069.6350208
SRR17520069.6350210
SRR17520069.6350212
SRR17520069.6350214
SRR17520069.6350216
SRR17520069.6353903
SRR17520069.6353907
SRR17520069.6353909
SRR17520069.6353915
SRR17520069.6353917
SRR17520069.6353923
SRR17520069.6353925
SRR17520069.6353927
SRR17520069.6353929
SRR17520069.6353931
SRR17520069.6353933
SRR17520069.6353937
SRR17520069.6353941
SRR17520069.6353943
SRR17520069.6353947
SRR17520069.6354698
SRR17520069.6354700
SRR17520069.6354702
SRR17520069.6354706
SRR17520069.6354708
SRR17520069.6354710
SRR17520069.6354712
SRR17520069.6354714
SRR17520069.6359090
SRR17520069.6359096
SRR17520069.6359098
SRR17520069.6359100
SRR17520069.6359102
SRR17520069.6359104
SRR17520069.6359106
SRR17520069.6359108
SRR17520069.6359110
SRR17520069.6359114
SRR17520069.6359116
SRR17520069.6359118
SRR17520069.6359120
SRR17520069.6359122

SRR17520069.6359124
SRR17520069.6359126
SRR17520069.6359128
SRR17520069.6359130
SRR17520069.6359132
SRR17520069.6359134
SRR17520069.6359136
SRR17520069.6359138
SRR17520069.6359140
SRR17520069.6359142
SRR17520069.6359146
SRR17520069.6359148
SRR17520069.6359150
SRR17520069.6359154
SRR17520069.6359156
SRR17520069.6359158
SRR17520069.6359160
SRR17520069.6359162
SRR17520069.6359164
SRR17520069.6359166
SRR17520069.6359168
SRR17520069.6360061
SRR17520069.6360063
SRR17520069.6360065
SRR17520069.6360067
SRR17520069.6360069
SRR17520069.6360071
SRR17520069.6360073
SRR17520069.6360075
SRR17520069.6360077
SRR17520069.6360079
SRR17520069.6368215
SRR17520069.6368217
SRR17520069.6368219
SRR17520069.6368221
SRR17520069.6368227
SRR17520069.6368229
SRR17520069.6368231
SRR17520069.6368233
SRR17520069.6368235
SRR17520069.6368237
SRR17520069.6368239
SRR17520069.6368241
SRR17520069.6368243
SRR17520069.6368245
SRR17520069.6368247
SRR17520069.6368249

SRR17520069.6368251
SRR17520069.6368253
SRR17520069.6368255
SRR17520069.6368257
SRR17520069.6368259
SRR17520069.6368261
SRR17520069.6368263
SRR17520069.6368265
SRR17520069.6368267
SRR17520069.6368269
SRR17520069.6368271
SRR17520069.6368273
SRR17520069.6368275
SRR17520069.6368277
SRR17520069.6368281
SRR17520069.6368283
SRR17520069.6368285
SRR17520069.6368287
SRR17520069.6368289
SRR17520069.6368291
SRR17520069.6368293
SRR17520069.6368295
SRR17520069.6368297
SRR17520069.6368299
SRR17520069.6368301
SRR17520069.6375000
SRR17520069.6375002
SRR17520069.6378039
SRR17520069.6378041
SRR17520069.6378043
SRR17520069.6378045
SRR17520069.6378047
SRR17520069.6378049
SRR17520069.6378051
SRR17520069.6378053
SRR17520069.6378055
SRR17520069.6378059
SRR17520069.6378061
SRR17520069.6378063
SRR17520069.6378065
SRR17520069.6378067
SRR17520069.6378069
SRR17520069.6378071
SRR17520069.6378075
SRR17520069.6378077
SRR17520069.6378079
SRR17520069.6379756

SRR17520069.6379758
SRR17520069.6379760
SRR17520069.6379762
SRR17520069.6379764
SRR17520069.6379766
SRR17520069.6379768
SRR17520069.6379770
SRR17520069.6379772
SRR17520069.6379774
SRR17520069.6379776
SRR17520069.6379778
SRR17520069.6379780
SRR17520069.6379782
SRR17520069.6379784
SRR17520069.6379786
SRR17520069.6379790
SRR17520069.6379792
SRR17520069.6379794
SRR17520069.6379796
SRR17520069.6379798
SRR17520069.6379800
SRR17520069.6382159
SRR17520069.6382161
SRR17520069.6382163
SRR17520069.6382165
SRR17520069.6382167
SRR17520069.6382169
SRR17520069.6382171
SRR17520069.6382173
SRR17520069.6382175
SRR17520069.6382177
SRR17520069.6382179
SRR17520069.6382181
SRR17520069.6382183
SRR17520069.6382185
SRR17520069.6382187
SRR17520069.6382189
SRR17520069.6382191
SRR17520069.6382193
SRR17520069.6382195
SRR17520069.6382197
SRR17520069.6382199
SRR17520069.6382201
SRR17520069.6382203
SRR17520069.6382205
SRR17520069.6382207
SRR17520069.6382209

SRR17520069.6382211
SRR17520069.6382213
SRR17520069.6382215
SRR17520069.6382217
SRR17520069.6382219
SRR17520069.6382221
SRR17520069.6382223
SRR17520069.6382225
SRR17520069.6382227
SRR17520069.6382229
SRR17520069.6382231
SRR17520069.6382233
SRR17520069.6382235
SRR17520069.6382237
SRR17520069.6382239
SRR17520069.6383705
SRR17520069.6383707
SRR17520069.6383709
SRR17520069.6383711
SRR17520069.6383713
SRR17520069.6383715
SRR17520069.6383717
SRR17520069.6383719
SRR17520069.6383721
SRR17520069.6383723
SRR17520069.6383725
SRR17520069.6383727
SRR17520069.6387517
SRR17520069.6387740
SRR17520069.6387742
SRR17520069.6387744
SRR17520069.6388289
SRR17520069.6388291
SRR17520069.6388293
SRR17520069.6388295
SRR17520069.6389060
SRR17520069.6389062
SRR17520069.6389068
SRR17520069.6389070
SRR17520069.6389072
SRR17520069.6389074
SRR17520069.6389076
SRR17520069.6389078
SRR17520069.6389080
SRR17520069.6392325
SRR17520069.6392327
SRR17520069.6392329

SRR17520069.6392331
SRR17520069.6392333
SRR17520069.6392335
SRR17520069.6392337
SRR17520069.6399263
SRR17520069.6399265
SRR17520069.6399267
SRR17520069.6399916
SRR17520069.6399918
SRR17520069.6399920
SRR17520069.6399922
SRR17520069.6399924
SRR17520069.6399926
SRR17520069.6401956
SRR17520069.6401958
SRR17520069.6401960
SRR17520069.6401962
SRR17520069.6401964
SRR17520069.6401966
SRR17520069.6401968
SRR17520069.6401970
SRR17520069.6401972
SRR17520069.6401974
SRR17520069.6401976
SRR17520069.6401978
SRR17520069.6401982
SRR17520069.6401984
SRR17520069.6401986
SRR17520069.6401988
SRR17520069.6401990
SRR17520069.6401992
SRR17520069.6401994
SRR17520069.6407888
SRR17520069.6407890
SRR17520069.6408019
SRR17520069.6408109
SRR17520069.6408111
SRR17520069.6408113
SRR17520069.6408115
SRR17520069.6408494
SRR17520069.6408496
SRR17520069.6408498
SRR17520069.6408500
SRR17520069.6408502
SRR17520069.6408504
SRR17520069.6408506
SRR17520069.6408508

SRR17520069.6408510
SRR17520069.6408512
SRR17520069.6408514
SRR17520069.6408516
SRR17520069.6408518
SRR17520069.6408520
SRR17520069.6408522
SRR17520069.6408524
SRR17520069.6408526
SRR17520069.6408528
SRR17520069.6408530
SRR17520069.6408746
SRR17520069.6408752
SRR17520069.6408755
SRR17520069.6414953
SRR17520069.6414955
SRR17520069.6414958
SRR17520069.6414962
SRR17520069.6418303
SRR17520069.6419701
SRR17520069.6419703
SRR17520069.6421043
SRR17520069.6421171
SRR17520069.6421173
SRR17520069.6421175
SRR17520069.6421177
SRR17520069.6421179
SRR17520069.6421181
SRR17520069.6421183
SRR17520069.6421185
SRR17520069.6421187
SRR17520069.6421189
SRR17520069.6421191
SRR17520069.6421193
SRR17520069.6421195
SRR17520069.6421197
SRR17520069.6421199
SRR17520069.6421201
SRR17520069.6421205
SRR17520069.6428909
SRR17520069.6428911
SRR17520069.6428913
SRR17520069.6428915
SRR17520069.6428917
SRR17520069.6429286
SRR17520069.6429288
SRR17520069.6438087

SRR17520069.6438089
SRR17520069.6438093
SRR17520069.6443863
SRR17520069.6443865
SRR17520069.6443867
SRR17520069.6446021
SRR17520069.6448984
SRR17520069.6449005
SRR17520069.6457927
SRR17520069.6457929
SRR17520069.6463927
SRR17520069.6463929
SRR17520069.6463931
SRR17520069.6463933
SRR17520069.6463935
SRR17520069.6463937
SRR17520069.6463939
SRR17520069.6463941
SRR17520069.6463943
SRR17520069.6463945
SRR17520069.6466422
SRR17520069.6466424
SRR17520069.6466426
SRR17520069.6466428
SRR17520069.6466430
SRR17520069.6466432
SRR17520069.6466434
SRR17520069.6466436
SRR17520069.6466438
SRR17520069.6466440
SRR17520069.6466442
SRR17520069.6466444
SRR17520069.6466446
SRR17520069.6466448
SRR17520069.6466450
SRR17520069.6466452
SRR17520069.6466454
SRR17520069.6466456
SRR17520069.6466458
SRR17520069.6466460
SRR17520069.6466462
SRR17520069.6466464
SRR17520069.6466466
SRR17520069.6466468
SRR17520069.6466470
SRR17520069.6466472
SRR17520069.6466474

SRR17520069.6466476
SRR17520069.6466478
SRR17520069.6466480
SRR17520069.6466482
SRR17520069.6466484
SRR17520069.6466486
SRR17520069.6466488
SRR17520069.6466490
SRR17520069.6466492
SRR17520069.6466494
SRR17520069.6466496
SRR17520069.6466498
SRR17520069.6466500
SRR17520069.6466502
SRR17520069.6466504
SRR17520069.6466506
SRR17520069.6466508
SRR17520069.6466510
SRR17520069.6466512
SRR17520069.6466514
SRR17520069.6466516
SRR17520069.6466518
SRR17520069.6466520
SRR17520069.6466522
SRR17520069.6466524
SRR17520069.6466526
SRR17520069.6466528
SRR17520069.6466530
SRR17520069.6466532
SRR17520069.6466534
SRR17520069.6466536
SRR17520069.6466538
SRR17520069.6466540
SRR17520069.6466542
SRR17520069.6466544
SRR17520069.6466546
SRR17520069.6466548
SRR17520069.6466550
SRR17520069.6466552
SRR17520069.6466554
SRR17520069.6466556
SRR17520069.6466558
SRR17520069.6466560
SRR17520069.6466562
SRR17520069.6466564
SRR17520069.6472815
SRR17520069.6472817

SRR17520069.6472821
SRR17520069.6472823
SRR17520069.6472825
SRR17520069.6472827
SRR17520069.6472829
SRR17520069.6472831
SRR17520069.6472833
SRR17520069.6472835
SRR17520069.6472837
SRR17520069.6472839
SRR17520069.6472841
SRR17520069.6472843
SRR17520069.6472845
SRR17520069.6472849
SRR17520069.6472851
SRR17520069.6472853
SRR17520069.6472855
SRR17520069.6472857
SRR17520069.6472859
SRR17520069.6472861
SRR17520069.6472863
SRR17520069.6472865
SRR17520069.6472867
SRR17520069.6472869
SRR17520069.6472871
SRR17520069.6472873
SRR17520069.6472875
SRR17520069.6472877
SRR17520069.6472879
SRR17520069.6472881
SRR17520069.6472883
SRR17520069.6479570
SRR17520069.6479572
SRR17520069.6479574
SRR17520069.6480943
SRR17520069.6480945
SRR17520069.6480947
SRR17520069.6480949
SRR17520069.6480951
SRR17520069.6480953
SRR17520069.6480955
SRR17520069.6480957
SRR17520069.6482641
SRR17520069.6482643
SRR17520069.6482645
SRR17520069.6482647
SRR17520069.6482649

SRR17520069.6482651
SRR17520069.6482653
SRR17520069.6482655
SRR17520069.6482657
SRR17520069.6482659
SRR17520069.6482661
SRR17520069.6482663
SRR17520069.6482665
SRR17520069.6482667
SRR17520069.6482669
SRR17520069.6482671
SRR17520069.6482673
SRR17520069.6482675
SRR17520069.6482677
SRR17520069.6482679
SRR17520069.6482681
SRR17520069.6482683
SRR17520069.6482685
SRR17520069.6482687
SRR17520069.6482689
SRR17520069.6482691
SRR17520069.6482693
SRR17520069.6482695
SRR17520069.6482697
SRR17520069.6482699
SRR17520069.6482701
SRR17520069.6482703
SRR17520069.6482705
SRR17520069.6482707
SRR17520069.6482709
SRR17520069.6482711
SRR17520069.6482713
SRR17520069.6482715
SRR17520069.6482717
SRR17520069.6482719
SRR17520069.6482721
SRR17520069.6482723
SRR17520069.6482725
SRR17520069.6482727
SRR17520069.6482729
SRR17520069.6482731
SRR17520069.6482733
SRR17520069.6482735
SRR17520069.6482737
SRR17520069.6482739
SRR17520069.6482741
SRR17520069.6482743

SRR17520069.6482745
SRR17520069.6482747
SRR17520069.6482749
SRR17520069.6482751
SRR17520069.6482753
SRR17520069.6482755
SRR17520069.6482757
SRR17520069.6482759
SRR17520069.6482761
SRR17520069.6482763
SRR17520069.6482765
SRR17520069.6482767
SRR17520069.6482769
SRR17520069.6482771
SRR17520069.6485736
SRR17520069.6485738
SRR17520069.6485740
SRR17520069.6485742
SRR17520069.6485744
SRR17520069.6485746
SRR17520069.6485748
SRR17520069.6485750
SRR17520069.6485752
SRR17520069.6485754
SRR17520069.6485756
SRR17520069.6485758
SRR17520069.6485760
SRR17520069.6485762
SRR17520069.6485764
SRR17520069.6485766
SRR17520069.6485768
SRR17520069.6485770
SRR17520069.6485772
SRR17520069.6485774
SRR17520069.6485778
SRR17520069.6485780
SRR17520069.6485782
SRR17520069.6485784
SRR17520069.6485786
SRR17520069.6485788
SRR17520069.6485790
SRR17520069.6485792
SRR17520069.6485794
SRR17520069.6485796
SRR17520069.6485800
SRR17520069.6485802
SRR17520069.6485804

SRR17520069.6485808
SRR17520069.6485810
SRR17520069.6485812
SRR17520069.6499575
SRR17520069.6499577
SRR17520069.6499579
SRR17520069.6499581
SRR17520069.6499583
SRR17520069.6499585
SRR17520069.6505286
SRR17520069.6505288
SRR17520069.6505290
SRR17520069.6505292
SRR17520069.6505649
SRR17520069.6508835
SRR17520069.6508837
SRR17520069.6508839
SRR17520069.6508841
SRR17520069.6508843
SRR17520069.6510820
SRR17520069.6510822
SRR17520069.6510824
SRR17520069.6510826
SRR17520069.6510828
SRR17520069.6511912
SRR17520069.6517398
SRR17520069.6517400
SRR17520069.6517402
SRR17520069.6517404
SRR17520069.6517406
SRR17520069.6517408
SRR17520069.6517410
SRR17520069.6517412
SRR17520069.6517414
SRR17520069.6517416
SRR17520069.6517418
SRR17520069.6517420
SRR17520069.6517422
SRR17520069.6518368
SRR17520069.6518370
SRR17520069.6525759
SRR17520069.6527528
SRR17520069.6527530
SRR17520069.6527532
SRR17520069.6527534
SRR17520069.6527536
SRR17520069.6527538

SRR17520069.6527540
SRR17520069.6527542
SRR17520069.6527544
SRR17520069.6528705
SRR17520069.6535190
SRR17520069.6538599
SRR17520069.6538601
SRR17520069.6538603
SRR17520069.6538605
SRR17520069.6538607
SRR17520069.6538609
SRR17520069.6538611
SRR17520069.6538613
SRR17520069.6538615
SRR17520069.6538617
SRR17520069.6538621
SRR17520069.6538623
SRR17520069.6538627
SRR17520069.6538631
SRR17520069.6538633
SRR17520069.6538635
SRR17520069.6538637
SRR17520069.6538639
SRR17520069.6538641
SRR17520069.6538643
SRR17520069.6538645
SRR17520069.6538647
SRR17520069.6538649
SRR17520069.6538651
SRR17520069.6540333
SRR17520069.6540335
SRR17520069.6540337
SRR17520069.6540339
SRR17520069.6540341
SRR17520069.6540343
SRR17520069.6540345
SRR17520069.6540347
SRR17520069.6540349
SRR17520069.6540351
SRR17520069.6540353
SRR17520069.6540355
SRR17520069.6545515
SRR17520069.6545517
SRR17520069.6551424
SRR17520069.6554108
SRR17520069.6554142
SRR17520069.6557153

SRR17520069.6557155
SRR17520069.6557157
SRR17520069.6557159
SRR17520069.6557161
SRR17520069.6557163
SRR17520069.6557165
SRR17520069.6557167
SRR17520069.6557169
SRR17520069.6557171
SRR17520069.6557173
SRR17520069.6557175
SRR17520069.6557177
SRR17520069.6557179
SRR17520069.6557181
SRR17520069.6557183
SRR17520069.6557185
SRR17520069.6557187
SRR17520069.6557189
SRR17520069.6557191
SRR17520069.6557193
SRR17520069.6557195
SRR17520069.6557197
SRR17520069.6557199
SRR17520069.6557201
SRR17520069.6557203
SRR17520069.6557205
SRR17520069.6557207
SRR17520069.6557209
SRR17520069.6557211
SRR17520069.6557213
SRR17520069.6557215
SRR17520069.6557217
SRR17520069.6557219
SRR17520069.6557221
SRR17520069.6557223
SRR17520069.6557225
SRR17520069.6557227
SRR17520069.6557229
SRR17520069.6557231
SRR17520069.6557233
SRR17520069.6557235
SRR17520069.6557237
SRR17520069.6557239
SRR17520069.6557241
SRR17520069.6557243
SRR17520069.6557245
SRR17520069.6557247

SRR17520069.6557249
SRR17520069.6557251
SRR17520069.6557253
SRR17520069.6557255
SRR17520069.6557257
SRR17520069.6557259
SRR17520069.6557261
SRR17520069.6557263
SRR17520069.6557265
SRR17520069.6557267
SRR17520069.6557269
SRR17520069.6557271
SRR17520069.6557273
SRR17520069.6557275
SRR17520069.6557277
SRR17520069.6558939
SRR17520069.6558941
SRR17520069.6558943
SRR17520069.6558945
SRR17520069.6561788
SRR17520069.6561790
SRR17520069.6561792
SRR17520069.6561794
SRR17520069.6561796
SRR17520069.6561798
SRR17520069.6561800
SRR17520069.6564881
SRR17520069.6564883
SRR17520069.6564885
SRR17520069.6564887
SRR17520069.6564889
SRR17520069.6564891
SRR17520069.6564893
SRR17520069.6564895
SRR17520069.6564897
SRR17520069.6564899
SRR17520069.6564901
SRR17520069.6564903
SRR17520069.6564905
SRR17520069.6565297
SRR17520069.6565299
SRR17520069.6565301
SRR17520069.6565303
SRR17520069.6565305
SRR17520069.6565307
SRR17520069.6565309
SRR17520069.6565311

SRR17520069.6565313
SRR17520069.6565315
SRR17520069.6565317
SRR17520069.6565319
SRR17520069.6565321
SRR17520069.6565323
SRR17520069.6565325
SRR17520069.6565327
SRR17520069.6565329
SRR17520069.6565331
SRR17520069.6565333
SRR17520069.6565335
SRR17520069.6565337
SRR17520069.6565339
SRR17520069.6565341
SRR17520069.6565343
SRR17520069.6565345
SRR17520069.6565347
SRR17520069.6565349
SRR17520069.6565351
SRR17520069.6565353
SRR17520069.6565355
SRR17520069.6565357
SRR17520069.6565359
SRR17520069.6565361
SRR17520069.6565363
SRR17520069.6565365
SRR17520069.6565367
SRR17520069.6565369
SRR17520069.6565371
SRR17520069.6565373
SRR17520069.6565375
SRR17520069.6565377
SRR17520069.6565379
SRR17520069.6565381
SRR17520069.6565383
SRR17520069.6565385
SRR17520069.6565387
SRR17520069.6565389
SRR17520069.6565391
SRR17520069.6565393
SRR17520069.6565395
SRR17520069.6565397
SRR17520069.6565399
SRR17520069.6565401
SRR17520069.6565403
SRR17520069.6565405

SRR17520069.6565407
SRR17520069.6565409
SRR17520069.6565411
SRR17520069.6565413
SRR17520069.6565415
SRR17520069.6565417
SRR17520069.6565419
SRR17520069.6565421
SRR17520069.6565423
SRR17520069.6565425
SRR17520069.6565427
SRR17520069.6565429
SRR17520069.6565431
SRR17520069.6573950
SRR17520069.6573952
SRR17520069.6573954
SRR17520069.6573956
SRR17520069.6573958
SRR17520069.6573960
SRR17520069.6573962
SRR17520069.6573964
SRR17520069.6573966
SRR17520069.6573968
SRR17520069.6573970
SRR17520069.6573972
SRR17520069.6573974
SRR17520069.6573976
SRR17520069.6573978
SRR17520069.6573980
SRR17520069.6573982
SRR17520069.6573984
SRR17520069.6573986
SRR17520069.6573988
SRR17520069.6573990
SRR17520069.6573992
SRR17520069.6573994
SRR17520069.6573996
SRR17520069.6573998
SRR17520069.6574000
SRR17520069.6574002
SRR17520069.6574004
SRR17520069.6574006
SRR17520069.6574008
SRR17520069.6574010
SRR17520069.6574012
SRR17520069.6574014
SRR17520069.6574016

SRR17520069.6574020
SRR17520069.6574022
SRR17520069.6574024
SRR17520069.6574026
SRR17520069.6574028
SRR17520069.6574030
SRR17520069.6574032
SRR17520069.6574034
SRR17520069.6574036
SRR17520069.6574038
SRR17520069.6574040
SRR17520069.6574042
SRR17520069.6574044
SRR17520069.6574046
SRR17520069.6574050
SRR17520069.6574052
SRR17520069.6574054
SRR17520069.6574056
SRR17520069.6574058
SRR17520069.6574060
SRR17520069.6574062
SRR17520069.6574066
SRR17520069.6574068
SRR17520069.6574070
SRR17520069.6574072
SRR17520069.6574074
SRR17520069.6574076
SRR17520069.6574078
SRR17520069.6574080
SRR17520069.6574082
SRR17520069.6574084
SRR17520069.6574086
SRR17520069.6574088
SRR17520069.6574090
SRR17520069.6574092
SRR17520069.6574094
SRR17520069.6574096
SRR17520069.6574098
SRR17520069.6574100
SRR17520069.6574102
SRR17520069.6574104
SRR17520069.6574106
SRR17520069.6574108
SRR17520069.6574110
SRR17520069.6574112
SRR17520069.6574114
SRR17520069.6574118

SRR17520069.6574120
SRR17520069.6577900
SRR17520069.6577902
SRR17520069.6577904
SRR17520069.6577906
SRR17520069.6577908
SRR17520069.6577910
SRR17520069.6577912
SRR17520069.6577914
SRR17520069.6577916
SRR17520069.6577918
SRR17520069.6577920
SRR17520069.6578641
SRR17520069.6578643
SRR17520069.6578645
SRR17520069.6578647
SRR17520069.6578651
SRR17520069.6578653
SRR17520069.6578655
SRR17520069.6578657
SRR17520069.6578659
SRR17520069.6578661
SRR17520069.6578663
SRR17520069.6578667
SRR17520069.6578669
SRR17520069.6578671
SRR17520069.6578673
SRR17520069.6578675
SRR17520069.6578677
SRR17520069.6578679
SRR17520069.6578686
SRR17520069.6578692
SRR17520069.6578694
SRR17520069.6578696
SRR17520069.6578698
SRR17520069.6578700
SRR17520069.6578702
SRR17520069.6578704
SRR17520069.6578706
SRR17520069.6578708
SRR17520069.6578710
SRR17520069.6578712
SRR17520069.6578714
SRR17520069.6578716
SRR17520069.6578718
SRR17520069.6578722
SRR17520069.6578724

SRR17520069.6578726
SRR17520069.6578728
SRR17520069.6578730
SRR17520069.6578732
SRR17520069.6578736
SRR17520069.6578738
SRR17520069.6578740
SRR17520069.6578742
SRR17520069.6578746
SRR17520069.6578748
SRR17520069.6578750
SRR17520069.6578752
SRR17520069.6578756
SRR17520069.6578758
SRR17520069.6578760
SRR17520069.6578762
SRR17520069.6578764
SRR17520069.6578766
SRR17520069.6578768
SRR17520069.6578770
SRR17520069.6578774
SRR17520069.6578776
SRR17520069.6578778
SRR17520069.6578780
SRR17520069.6578784
SRR17520069.6578790
SRR17520069.6578792
SRR17520069.6578794
SRR17520069.6578796
SRR17520069.6578800
SRR17520069.6578802
SRR17520069.6579269
SRR17520069.6579271
SRR17520069.6579273
SRR17520069.6579275
SRR17520069.6579277
SRR17520069.6579279
SRR17520069.6588605
SRR17520069.6588607
SRR17520069.6589313
SRR17520069.6589315
SRR17520069.6589317
SRR17520069.6589319
SRR17520069.6589321
SRR17520069.6589323
SRR17520069.6589325
SRR17520069.6589327

SRR17520069.6589329
SRR17520069.6589331
SRR17520069.6589333
SRR17520069.6589335
SRR17520069.6594626
SRR17520069.6597155
SRR17520069.6597157
SRR17520069.6598054
SRR17520069.6598057
SRR17520069.6598059
SRR17520069.6598061
SRR17520069.6598063
SRR17520069.6598067
SRR17520069.6598069
SRR17520069.6598071
SRR17520069.6598073
SRR17520069.6598075
SRR17520069.6598077
SRR17520069.6598079
SRR17520069.6598081
SRR17520069.6598083
SRR17520069.6598085
SRR17520069.6598087
SRR17520069.6598089
SRR17520069.6598091
SRR17520069.6598093
SRR17520069.6598095
SRR17520069.6598101
SRR17520069.6599743
SRR17520069.6599745
SRR17520069.6599747
SRR17520069.6599749
SRR17520069.6613090
SRR17520069.6613092
SRR17520069.6613094
SRR17520069.6613096
SRR17520069.6613098
SRR17520069.6613100
SRR17520069.6615100
SRR17520069.6615102
SRR17520069.6615104
SRR17520069.6615106
SRR17520069.6615108
SRR17520069.6615110
SRR17520069.6615112
SRR17520069.6615114
SRR17520069.6615116

SRR17520069.6615118
SRR17520069.6615120
SRR17520069.6615122
SRR17520069.6615126
SRR17520069.6615128
SRR17520069.6615130
SRR17520069.6615132
SRR17520069.6615134
SRR17520069.6622699
SRR17520069.6622701
SRR17520069.6632163
SRR17520069.6632167
SRR17520069.6632169
SRR17520069.6632171
SRR17520069.6632173
SRR17520069.6632175
SRR17520069.6632177
SRR17520069.6632179
SRR17520069.6632181
SRR17520069.6632183
SRR17520069.6632185
SRR17520069.6632187
SRR17520069.6632189
SRR17520069.6632191
SRR17520069.6632317
SRR17520069.6632319
SRR17520069.6632321
SRR17520069.6632323
SRR17520069.6632325
SRR17520069.6632327
SRR17520069.6632329
SRR17520069.6632331
SRR17520069.6632333
SRR17520069.6632335
SRR17520069.6632337
SRR17520069.6632339
SRR17520069.6632341
SRR17520069.6632345
SRR17520069.6632347
SRR17520069.6632349
SRR17520069.6632351
SRR17520069.6632353
SRR17520069.6632355
SRR17520069.6632357
SRR17520069.6632359
SRR17520069.6632361
SRR17520069.6632363

SRR17520069.6632367
SRR17520069.6632369
SRR17520069.6632371
SRR17520069.6632373
SRR17520069.6632375
SRR17520069.6632377
SRR17520069.6632379
SRR17520069.6632381
SRR17520069.6632383
SRR17520069.6632385
SRR17520069.6632387
SRR17520069.6632389
SRR17520069.6632391
SRR17520069.6632393
SRR17520069.6632397
SRR17520069.6632399
SRR17520069.6634451
SRR17520069.6634453
SRR17520069.6634455
SRR17520069.6634457
SRR17520069.6634459
SRR17520069.6634461
SRR17520069.6634463
SRR17520069.6634465
SRR17520069.6634467
SRR17520069.6634469
SRR17520069.6634471
SRR17520069.6634473
SRR17520069.6634475
SRR17520069.6634477
SRR17520069.6634479
SRR17520069.6634481
SRR17520069.6634483
SRR17520069.6634485
SRR17520069.6636042
SRR17520069.6636044
SRR17520069.6636046
SRR17520069.6636048
SRR17520069.6636050
SRR17520069.6636052
SRR17520069.6636054
SRR17520069.6636056
SRR17520069.6636058
SRR17520069.6636060
SRR17520069.6636062
SRR17520069.6636064
SRR17520069.6636066

SRR17520069.6636068
SRR17520069.6636070
SRR17520069.6636072
SRR17520069.6636074
SRR17520069.6636076
SRR17520069.6636078
SRR17520069.6636080
SRR17520069.6636082
SRR17520069.6636084
SRR17520069.6636086
SRR17520069.6636088
SRR17520069.6636090
SRR17520069.6636092
SRR17520069.6636094
SRR17520069.6636096
SRR17520069.6636098
SRR17520069.6636100
SRR17520069.6636102
SRR17520069.6636104
SRR17520069.6636106
SRR17520069.6636108
SRR17520069.6636110
SRR17520069.6636112
SRR17520069.6636114
SRR17520069.6636116
SRR17520069.6636118
SRR17520069.6636120
SRR17520069.6636122
SRR17520069.6636124
SRR17520069.6636126
SRR17520069.6636128
SRR17520069.6636130
SRR17520069.6636132
SRR17520069.6636134
SRR17520069.6636136
SRR17520069.6636138
SRR17520069.6636140
SRR17520069.6636142
SRR17520069.6638329
SRR17520069.6638331
SRR17520069.6640581
SRR17520069.6640583
SRR17520069.6640585
SRR17520069.6640587
SRR17520069.6640589
SRR17520069.6640591
SRR17520069.6640593

SRR17520069.6640595
SRR17520069.6640597
SRR17520069.6640599
SRR17520069.6640601
SRR17520069.6640603
SRR17520069.6640605
SRR17520069.6640607
SRR17520069.6640609
SRR17520069.6640611
SRR17520069.6640613
SRR17520069.6640615
SRR17520069.6640617
SRR17520069.6640619
SRR17520069.6640621
SRR17520069.6640623
SRR17520069.6640625
SRR17520069.6640627
SRR17520069.6640629
SRR17520069.6640631
SRR17520069.6640633
SRR17520069.6640635
SRR17520069.6640637
SRR17520069.6640639
SRR17520069.6643610
SRR17520069.6643612
SRR17520069.6643614
SRR17520069.6643616
SRR17520069.6643618
SRR17520069.6643620
SRR17520069.6643622
SRR17520069.6643624
SRR17520069.6643626
SRR17520069.6643628
SRR17520069.6643630
SRR17520069.6643632
SRR17520069.6643634
SRR17520069.6643638
SRR17520069.6643640
SRR17520069.6643642
SRR17520069.6643644
SRR17520069.6643646
SRR17520069.6643648
SRR17520069.6643650
SRR17520069.6643654
SRR17520069.6643656
SRR17520069.6643658
SRR17520069.6643660

SRR17520069.6643662
SRR17520069.6643664
SRR17520069.6643666
SRR17520069.6643668
SRR17520069.6643670
SRR17520069.6643672
SRR17520069.6643674
SRR17520069.6643676
SRR17520069.6644947
SRR17520069.6644949
SRR17520069.6644953
SRR17520069.6644957
SRR17520069.6644959
SRR17520069.6644961
SRR17520069.6644963
SRR17520069.6644965
SRR17520069.6644967
SRR17520069.6644969
SRR17520069.6644971
SRR17520069.6644973
SRR17520069.6644977
SRR17520069.6644979
SRR17520069.6644981
SRR17520069.6644983
SRR17520069.6644985
SRR17520069.6644987
SRR17520069.6644989
SRR17520069.6644991
SRR17520069.6646805
SRR17520069.6646807
SRR17520069.6646809
SRR17520069.6646811
SRR17520069.6648819
SRR17520069.6648821
SRR17520069.6648823
SRR17520069.6648825
SRR17520069.6648827
SRR17520069.6648829
SRR17520069.6648831
SRR17520069.6648833
SRR17520069.6648835
SRR17520069.6648837
SRR17520069.6648839
SRR17520069.6648841
SRR17520069.6648843
SRR17520069.6648845
SRR17520069.6648847

SRR17520069.6648849
SRR17520069.6648851
SRR17520069.6648853
SRR17520069.6648855
SRR17520069.6648857
SRR17520069.6648859
SRR17520069.6648861
SRR17520069.6648863
SRR17520069.6648865
SRR17520069.6648867
SRR17520069.6648869
SRR17520069.6648871
SRR17520069.6648873
SRR17520069.6648875
SRR17520069.6648877
SRR17520069.6648879
SRR17520069.6648881
SRR17520069.6648883
SRR17520069.6648887
SRR17520069.6648889
SRR17520069.6648891
SRR17520069.6648893
SRR17520069.6648895
SRR17520069.6648897
SRR17520069.6657669
SRR17520069.6657671
SRR17520069.6657673
SRR17520069.6657675
SRR17520069.6657677
SRR17520069.6657679
SRR17520069.6657681
SRR17520069.6657683
SRR17520069.6657685
SRR17520069.6657687
SRR17520069.6657689
SRR17520069.6657691
SRR17520069.6657693
SRR17520069.6657695
SRR17520069.6657697
SRR17520069.6657699
SRR17520069.6657701
SRR17520069.6657703
SRR17520069.6657707
SRR17520069.6657709
SRR17520069.6657711
SRR17520069.6657713
SRR17520069.6657715

SRR17520069.6657717
SRR17520069.6657719
SRR17520069.6657721
SRR17520069.6657723
SRR17520069.6657725
SRR17520069.6657727
SRR17520069.6657731
SRR17520069.6657733
SRR17520069.6657735
SRR17520069.6657737
SRR17520069.6657739
SRR17520069.6657741
SRR17520069.6657743
SRR17520069.6657745
SRR17520069.6657747
SRR17520069.6657749
SRR17520069.6657751
SRR17520069.6657753
SRR17520069.6657755
SRR17520069.6657757
SRR17520069.6657759
SRR17520069.6657761
SRR17520069.6657763
SRR17520069.6657765
SRR17520069.6657767
SRR17520069.6657769
SRR17520069.6657771
SRR17520069.6659488
SRR17520069.6660074
SRR17520069.6660076
SRR17520069.6660078
SRR17520069.6660080
SRR17520069.6660082
SRR17520069.6660084
SRR17520069.6665943
SRR17520069.6665945
SRR17520069.6665947
SRR17520069.6665949
SRR17520069.6665951
SRR17520069.6665953
SRR17520069.6665955
SRR17520069.6665957
SRR17520069.6665959
SRR17520069.6665961
SRR17520069.6665963
SRR17520069.6665965
SRR17520069.6665967

SRR17520069.6665969
SRR17520069.6665971
SRR17520069.6665973
SRR17520069.6665975
SRR17520069.6665977
SRR17520069.6665979
SRR17520069.6665981
SRR17520069.6665983
SRR17520069.6665985
SRR17520069.6665987
SRR17520069.6665989
SRR17520069.6665991
SRR17520069.6665993
SRR17520069.6665995
SRR17520069.6665997
SRR17520069.6665999
SRR17520069.6666001
SRR17520069.6666003
SRR17520069.6666005
SRR17520069.6666007
SRR17520069.6666009
SRR17520069.6666011
SRR17520069.6666013
SRR17520069.6666019
SRR17520069.6666025
SRR17520069.6666033
SRR17520069.6666039
SRR17520069.6666041
SRR17520069.6666043
SRR17520069.6666049
SRR17520069.6666067
SRR17520069.6666069
SRR17520069.6666071
SRR17520069.6666077
SRR17520069.6666079
SRR17520069.6666083
SRR17520069.6668158
SRR17520069.6668164
SRR17520069.6668186
SRR17520069.6668196
SRR17520069.6668208
SRR17520069.6672888
SRR17520069.6672890
SRR17520069.6672892
SRR17520069.6672894
SRR17520069.6672896
SRR17520069.6672900

SRR17520069.6672902
SRR17520069.6672904
SRR17520069.6672906
SRR17520069.6672908
SRR17520069.6672910
SRR17520069.6672912
SRR17520069.6672914
SRR17520069.6672916
SRR17520069.6672920
SRR17520069.6672922
SRR17520069.6677503
SRR17520069.6677505
SRR17520069.6677507
SRR17520069.6678574
SRR17520069.6679342
SRR17520069.6679346
SRR17520069.6685401
SRR17520069.6685421
SRR17520069.6685423
SRR17520069.6685425
SRR17520069.6685427
SRR17520069.6685830
SRR17520069.6685832
SRR17520069.6685834
SRR17520069.6685836
SRR17520069.6685838
SRR17520069.6685840
SRR17520069.6685842
SRR17520069.6685844
SRR17520069.6685846
SRR17520069.6685850
SRR17520069.6685852
SRR17520069.6685854
SRR17520069.6685856
SRR17520069.6685858
SRR17520069.6685860
SRR17520069.6685862
SRR17520069.6685864
SRR17520069.6685866
SRR17520069.6685868
SRR17520069.6685870
SRR17520069.6685872
SRR17520069.6685874
SRR17520069.6685876
SRR17520069.6685878
SRR17520069.6686536
SRR17520069.6689404

SRR17520069.6689757
SRR17520069.6689759
SRR17520069.6689761
SRR17520069.6689763
SRR17520069.6689765
SRR17520069.6691489
SRR17520069.6691491
SRR17520069.6691493
SRR17520069.6691495
SRR17520069.6691497
SRR17520069.6691499
SRR17520069.6691501
SRR17520069.6691503
SRR17520069.6691505
SRR17520069.6691507
SRR17520069.6691509
SRR17520069.6691511
SRR17520069.6700651
SRR17520069.6700653
SRR17520069.6700655
SRR17520069.6700657
SRR17520069.6700659
SRR17520069.6701430
SRR17520069.6701432
SRR17520069.6701434
SRR17520069.6701438
SRR17520069.6701440
SRR17520069.6701442
SRR17520069.6701444
SRR17520069.6701446
SRR17520069.6701448
SRR17520069.6701450
SRR17520069.6701454
SRR17520069.6701456
SRR17520069.6701458
SRR17520069.6701460
SRR17520069.6701462
SRR17520069.6701464
SRR17520069.6701466
SRR17520069.6701468
SRR17520069.6701470
SRR17520069.6701472
SRR17520069.6701474
SRR17520069.6701476
SRR17520069.6701478
SRR17520069.6701480
SRR17520069.6701482

SRR17520069.6701484
SRR17520069.6701486
SRR17520069.6701490
SRR17520069.6701492
SRR17520069.6701494
SRR17520069.6701498
SRR17520069.6701500
SRR17520069.6701502
SRR17520069.6701504
SRR17520069.6704009
SRR17520069.6710152
SRR17520069.6710154
SRR17520069.6710156
SRR17520069.6710158
SRR17520069.6710160
SRR17520069.6711902
SRR17520069.6711904
SRR17520069.6712702
SRR17520069.6712704
SRR17520069.6712706
SRR17520069.6712708
SRR17520069.6712710
SRR17520069.6712714
SRR17520069.6712716
SRR17520069.6712718
SRR17520069.6712720
SRR17520069.6712722
SRR17520069.6719209
SRR17520069.6721590
SRR17520069.6721592
SRR17520069.6721594
SRR17520069.6721596
SRR17520069.6721598
SRR17520069.6721600
SRR17520069.6721602
SRR17520069.6721604
SRR17520069.6721606
SRR17520069.6721608
SRR17520069.6721610
SRR17520069.6721612
SRR17520069.6730185
SRR17520069.6730187
SRR17520069.6730191
SRR17520069.6730193
SRR17520069.6730195
SRR17520069.6744181
SRR17520069.6744510

SRR17520069.6744512
SRR17520069.6744514
SRR17520069.6744516
SRR17520069.6744518
SRR17520069.6744522
SRR17520069.6744524
SRR17520069.6744528
SRR17520069.6744530
SRR17520069.6744532
SRR17520069.6744534
SRR17520069.6744536
SRR17520069.6744538
SRR17520069.6744540
SRR17520069.6744542
SRR17520069.6744544
SRR17520069.6744546
SRR17520069.6744548
SRR17520069.6744550
SRR17520069.6744552
SRR17520069.6744554
SRR17520069.6744556
SRR17520069.6744558
SRR17520069.6744560
SRR17520069.6744562
SRR17520069.6744564
SRR17520069.6744566
SRR17520069.6744568
SRR17520069.6744570
SRR17520069.6744572
SRR17520069.6744574
SRR17520069.6744576
SRR17520069.6744578
SRR17520069.6744580
SRR17520069.6744582
SRR17520069.6744584
SRR17520069.6744586
SRR17520069.6752370
SRR17520069.6752372
SRR17520069.6752374
SRR17520069.6752376
SRR17520069.6752378
SRR17520069.6752380
SRR17520069.6762428
SRR17520069.6762430
SRR17520069.6766301
SRR17520069.6768526
SRR17520069.6768528

SRR17520069.6768530
SRR17520069.6768532
SRR17520069.6768534
SRR17520069.6768536
SRR17520069.6768538
SRR17520069.6768540
SRR17520069.6768542
SRR17520069.6768544
SRR17520069.6768546
SRR17520069.6768548
SRR17520069.6768550
SRR17520069.6768552
SRR17520069.6768554
SRR17520069.6768556
SRR17520069.6768558
SRR17520069.6768560
SRR17520069.6768562
SRR17520069.6768564
SRR17520069.6768566
SRR17520069.6768568
SRR17520069.6768570
SRR17520069.6768572
SRR17520069.6768574
SRR17520069.6768576
SRR17520069.6768578
SRR17520069.6768580
SRR17520069.6768582
SRR17520069.6768584
SRR17520069.6768586
SRR17520069.6768588
SRR17520069.6768590
SRR17520069.6768592
SRR17520069.6768594
SRR17520069.6768596
SRR17520069.6768598
SRR17520069.6768600
SRR17520069.6768602
SRR17520069.6768604
SRR17520069.6768606
SRR17520069.6768608
SRR17520069.6768610
SRR17520069.6768612
SRR17520069.6768614
SRR17520069.6768616
SRR17520069.6768618
SRR17520069.6768620
SRR17520069.6768622

SRR17520069.6768624
SRR17520069.6768626
SRR17520069.6768628
SRR17520069.6768630
SRR17520069.6768632
SRR17520069.6768634
SRR17520069.6768636
SRR17520069.6768638
SRR17520069.6768640
SRR17520069.6768642
SRR17520069.6768644
SRR17520069.6768646
SRR17520069.6768648
SRR17520069.6768650
SRR17520069.6768652
SRR17520069.6768654
SRR17520069.6768656
SRR17520069.6768658
SRR17520069.6768660
SRR17520069.6768662
SRR17520069.6768664
SRR17520069.6768666
SRR17520069.6768668
SRR17520069.6768670
SRR17520069.6768672
SRR17520069.6768674
SRR17520069.6768676
SRR17520069.6768678
SRR17520069.6768680
SRR17520069.6768682
SRR17520069.6768684
SRR17520069.6768686
SRR17520069.6768688
SRR17520069.6768690
SRR17520069.6768692
SRR17520069.6768694
SRR17520069.6768696
SRR17520069.6768698
SRR17520069.6768700
SRR17520069.6768702
SRR17520069.6768704
SRR17520069.6768706
SRR17520069.6768708
SRR17520069.6768710
SRR17520069.6768712
SRR17520069.6768714
SRR17520069.6768716

SRR17520069.6768718
SRR17520069.6768720
SRR17520069.6768722
SRR17520069.6768724
SRR17520069.6768726
SRR17520069.6768728
SRR17520069.6768730
SRR17520069.6768732
SRR17520069.6768734
SRR17520069.6768736
SRR17520069.6768738
SRR17520069.6768742
SRR17520069.6768744
SRR17520069.6768746
SRR17520069.6768748
SRR17520069.6768750
SRR17520069.6768752
SRR17520069.6768754
SRR17520069.6768756
SRR17520069.6768758
SRR17520069.6768760
SRR17520069.6768762
SRR17520069.6768764
SRR17520069.6768766
SRR17520069.6768768
SRR17520069.6768770
SRR17520069.6768772
SRR17520069.6768774
SRR17520069.6768776
SRR17520069.6768778
SRR17520069.6768780
SRR17520069.6780208
SRR17520069.6780212
SRR17520069.6780322
SRR17520069.6780324
SRR17520069.6780326
SRR17520069.6780328
SRR17520069.6780330
SRR17520069.6780332
SRR17520069.6780334
SRR17520069.6780336
SRR17520069.6780338
SRR17520069.6780340
SRR17520069.6780342
SRR17520069.6780344
SRR17520069.6780346
SRR17520069.6780348

SRR17520069.6780350
SRR17520069.6780352
SRR17520069.6780354
SRR17520069.6780356
SRR17520069.6780358
SRR17520069.6780360
SRR17520069.6780362
SRR17520069.6780364
SRR17520069.6780366
SRR17520069.6780368
SRR17520069.6780370
SRR17520069.6780372
SRR17520069.6780374
SRR17520069.6780376
SRR17520069.6780378
SRR17520069.6780380
SRR17520069.6780382
SRR17520069.6780384
SRR17520069.6780386
SRR17520069.6780388
SRR17520069.6780390
SRR17520069.6780392
SRR17520069.6780394
SRR17520069.6780396
SRR17520069.6780398
SRR17520069.6780400
SRR17520069.6781818
SRR17520069.6783036
SRR17520069.6783038
SRR17520069.6783040
SRR17520069.6783042
SRR17520069.6784325
SRR17520069.6784329
SRR17520069.6784331
SRR17520069.6784333
SRR17520069.6784335
SRR17520069.6784337
SRR17520069.6784339
SRR17520069.6784341
SRR17520069.6784343
SRR17520069.6784345
SRR17520069.6785051
SRR17520069.6785053
SRR17520069.6785055
SRR17520069.6785057
SRR17520069.6785059
SRR17520069.6787708

SRR17520069.6787710
SRR17520069.6787712
SRR17520069.6787714
SRR17520069.6787716
SRR17520069.6787718
SRR17520069.6787720
SRR17520069.6787722
SRR17520069.6787724
SRR17520069.6787726
SRR17520069.6787728
SRR17520069.6787730
SRR17520069.6787732
SRR17520069.6787734
SRR17520069.6787736
SRR17520069.6787738
SRR17520069.6787740
SRR17520069.6787742
SRR17520069.6787744
SRR17520069.6787746
SRR17520069.6787748
SRR17520069.6787750
SRR17520069.6792165
SRR17520069.6792167
SRR17520069.6792169
SRR17520069.6792877
SRR17520069.6792879
SRR17520069.6792881
SRR17520069.6792883
SRR17520069.6792885
SRR17520069.6792887
SRR17520069.6792889
SRR17520069.6792891
SRR17520069.6792893
SRR17520069.6792895
SRR17520069.6795694
SRR17520069.6795696
SRR17520069.6795698
SRR17520069.6795700
SRR17520069.6795702
SRR17520069.6795704
SRR17520069.6795706
SRR17520069.6795710
SRR17520069.6795712
SRR17520069.6795714
SRR17520069.6795716
SRR17520069.6795718
SRR17520069.6795720

SRR17520069.6795722
SRR17520069.6795724
SRR17520069.6795726
SRR17520069.6795728
SRR17520069.6795730
SRR17520069.6795732
SRR17520069.6795734
SRR17520069.6795736
SRR17520069.6795738
SRR17520069.6795740
SRR17520069.6795742
SRR17520069.6795744
SRR17520069.6795746
SRR17520069.6795748
SRR17520069.6795750
SRR17520069.6795752
SRR17520069.6795754
SRR17520069.6795756
SRR17520069.6795758
SRR17520069.6795760
SRR17520069.6795762
SRR17520069.6795764
SRR17520069.6795766
SRR17520069.6801172
SRR17520069.6801174
SRR17520069.6801176
SRR17520069.6801178
SRR17520069.6801180
SRR17520069.6801182
SRR17520069.6801184
SRR17520069.6801186
SRR17520069.6801188
SRR17520069.6801190
SRR17520069.6801192
SRR17520069.6801194
SRR17520069.6801196
SRR17520069.6801198
SRR17520069.6801200
SRR17520069.6801202
SRR17520069.6801204
SRR17520069.6801206
SRR17520069.6801208
SRR17520069.6801210
SRR17520069.6801212
SRR17520069.6801214
SRR17520069.6801216
SRR17520069.6801218

SRR17520069.6801220
SRR17520069.6801222
SRR17520069.6801224
SRR17520069.6801226
SRR17520069.6801228
SRR17520069.6801230
SRR17520069.6801232
SRR17520069.6801234
SRR17520069.6801236
SRR17520069.6801238
SRR17520069.6801240
SRR17520069.6801242
SRR17520069.6801244
SRR17520069.6801246
SRR17520069.6801248
SRR17520069.6801250
SRR17520069.6801252
SRR17520069.6801254
SRR17520069.6801256
SRR17520069.6801258
SRR17520069.6801260
SRR17520069.6801262
SRR17520069.6801264
SRR17520069.6810397
SRR17520069.6810399
SRR17520069.6810401
SRR17520069.6810403
SRR17520069.6810405
SRR17520069.6810407
SRR17520069.6810409
SRR17520069.6810411
SRR17520069.6810413
SRR17520069.6810415
SRR17520069.6810417
SRR17520069.6810419
SRR17520069.6810667
SRR17520069.6810669
SRR17520069.6810671
SRR17520069.6810673
SRR17520069.6810675
SRR17520069.6810677
SRR17520069.6810679
SRR17520069.6810681
SRR17520069.6810683
SRR17520069.6810685
SRR17520069.6810687
SRR17520069.6810689

SRR17520069.6810691
SRR17520069.6810693
SRR17520069.6810695
SRR17520069.6810697
SRR17520069.6810699
SRR17520069.6810701
SRR17520069.6810703
SRR17520069.6810705
SRR17520069.6810707
SRR17520069.6810709
SRR17520069.6810711
SRR17520069.6810713
SRR17520069.6810715
SRR17520069.6810717
SRR17520069.6810719
SRR17520069.6810721
SRR17520069.6810723
SRR17520069.6810725
SRR17520069.6810727
SRR17520069.6810729
SRR17520069.6810731
SRR17520069.6810733
SRR17520069.6810735
SRR17520069.6810739
SRR17520069.6810741
SRR17520069.6810743
SRR17520069.6810745
SRR17520069.6810747
SRR17520069.6810749
SRR17520069.6810751
SRR17520069.6810753
SRR17520069.6810755
SRR17520069.6812318
SRR17520069.6812320
SRR17520069.6812322
SRR17520069.6812324
SRR17520069.6812326
SRR17520069.6812328
SRR17520069.6812332
SRR17520069.6812334
SRR17520069.6812336
SRR17520069.6812338
SRR17520069.6812340
SRR17520069.6812342
SRR17520069.6812344
SRR17520069.6812346
SRR17520069.6812348

SRR17520069.6812350
SRR17520069.6812352
SRR17520069.6812354
SRR17520069.6812356
SRR17520069.6812358
SRR17520069.6812360
SRR17520069.6812362
SRR17520069.6812364
SRR17520069.6812366
SRR17520069.6812368
SRR17520069.6812370
SRR17520069.6812372
SRR17520069.6812374
SRR17520069.6812376
SRR17520069.6812378
SRR17520069.6812380
SRR17520069.6812382
SRR17520069.6812384
SRR17520069.6812386
SRR17520069.6812388
SRR17520069.6812390
SRR17520069.6812392
SRR17520069.6812394
SRR17520069.6812396
SRR17520069.6812398
SRR17520069.6812400
SRR17520069.6812402
SRR17520069.6812404
SRR17520069.6812406
SRR17520069.6812408
SRR17520069.6812410
SRR17520069.6812412
SRR17520069.6812414
SRR17520069.6812416
SRR17520069.6812418
SRR17520069.6812420
SRR17520069.6812422
SRR17520069.6812424
SRR17520069.6812426
SRR17520069.6812428
SRR17520069.6812430
SRR17520069.6812432
SRR17520069.6812434
SRR17520069.6812436
SRR17520069.6812438
SRR17520069.6812440
SRR17520069.6812442

SRR17520069.6812444
SRR17520069.6812446
SRR17520069.6812448
SRR17520069.6812450
SRR17520069.6812452
SRR17520069.6812454
SRR17520069.6812456
SRR17520069.6820170
SRR17520069.6820172
SRR17520069.6820597
SRR17520069.6820599
SRR17520069.6820601
SRR17520069.6820603
SRR17520069.6820605
SRR17520069.6821139
SRR17520069.6821141
SRR17520069.6821143
SRR17520069.6821145
SRR17520069.6821149
SRR17520069.6821151
SRR17520069.6821153
SRR17520069.6821155
SRR17520069.6821157
SRR17520069.6821159
SRR17520069.6821161
SRR17520069.6821165
SRR17520069.6821169
SRR17520069.6821171
SRR17520069.6821173
SRR17520069.6821175
SRR17520069.6821177
SRR17520069.6821179
SRR17520069.6821181
SRR17520069.6821183
SRR17520069.6821185
SRR17520069.6821187
SRR17520069.6821189
SRR17520069.6821191
SRR17520069.6821193
SRR17520069.6821195
SRR17520069.6821197
SRR17520069.6821199
SRR17520069.6821201
SRR17520069.6821203
SRR17520069.6821205
SRR17520069.6821207
SRR17520069.6821209

SRR17520069.6821211
SRR17520069.6821213
SRR17520069.6821215
SRR17520069.6821217
SRR17520069.6821219
SRR17520069.6821221
SRR17520069.6821349
SRR17520069.6821351
SRR17520069.6821353
SRR17520069.6821355
SRR17520069.6821357
SRR17520069.6821359
SRR17520069.6821361
SRR17520069.6824867
SRR17520069.6824869
SRR17520069.6824871
SRR17520069.6824873
SRR17520069.6824875
SRR17520069.6824877
SRR17520069.6824879
SRR17520069.6824881
SRR17520069.6824883
SRR17520069.6824885
SRR17520069.6824887
SRR17520069.6824889
SRR17520069.6824891
SRR17520069.6824893
SRR17520069.6824895
SRR17520069.6824897
SRR17520069.6824899
SRR17520069.6824901
SRR17520069.6824903
SRR17520069.6824905
SRR17520069.6824907
SRR17520069.6826078
SRR17520069.6835682
SRR17520069.6838797
SRR17520069.6838799
SRR17520069.6844561
SRR17520069.6844563
SRR17520069.6844565
SRR17520069.6844567
SRR17520069.6844569
SRR17520069.6844571
SRR17520069.6844573
SRR17520069.6844575
SRR17520069.6844577

SRR17520069.6844579
SRR17520069.6844581
SRR17520069.6844585
SRR17520069.6844587
SRR17520069.6844589
SRR17520069.6844591
SRR17520069.6844593
SRR17520069.6844595
SRR17520069.6844597
SRR17520069.6844599
SRR17520069.6844601
SRR17520069.6844603
SRR17520069.6844605
SRR17520069.6854663
SRR17520069.6857962
SRR17520069.6857964
SRR17520069.6857966
SRR17520069.6857970
SRR17520069.6857973
SRR17520069.6857975
SRR17520069.6857977
SRR17520069.6857979
SRR17520069.6857981
SRR17520069.6857983
SRR17520069.6857985
SRR17520069.6857989
SRR17520069.6857991
SRR17520069.6857993
SRR17520069.6857995
SRR17520069.6857997
SRR17520069.6857999
SRR17520069.6858001
SRR17520069.6858003
SRR17520069.6858005
SRR17520069.6858007
SRR17520069.6858009
SRR17520069.6858011
SRR17520069.6858013
SRR17520069.6858015
SRR17520069.6858017
SRR17520069.6858021
SRR17520069.6858023
SRR17520069.6858025
SRR17520069.6858027
SRR17520069.6858029
SRR17520069.6858031
SRR17520069.6858033

SRR17520069.6858037
SRR17520069.6858039
SRR17520069.6858041
SRR17520069.6858043
SRR17520069.6858045
SRR17520069.6858047
SRR17520069.6858049
SRR17520069.6858051
SRR17520069.6858053
SRR17520069.6858055
SRR17520069.6858057
SRR17520069.6858059
SRR17520069.6858061
SRR17520069.6858063
SRR17520069.6858065
SRR17520069.6858067
SRR17520069.6858069
SRR17520069.6858071
SRR17520069.6858073
SRR17520069.6858075
SRR17520069.6858077
SRR17520069.6858079
SRR17520069.6858081
SRR17520069.6858083
SRR17520069.6858085
SRR17520069.6858087
SRR17520069.6858089
SRR17520069.6858091
SRR17520069.6858093
SRR17520069.6858097
SRR17520069.6858099
SRR17520069.6858101
SRR17520069.6858103
SRR17520069.6858109
SRR17520069.6858111
SRR17520069.6858113
SRR17520069.6858115
SRR17520069.6858117
SRR17520069.6858119
SRR17520069.6858121
SRR17520069.6858123
SRR17520069.6862876
SRR17520069.6862878
SRR17520069.6862880
SRR17520069.6862882
SRR17520069.6862884
SRR17520069.6862886

SRR17520069.6862888
SRR17520069.6862890
SRR17520069.6862892
SRR17520069.6862894
SRR17520069.6862896
SRR17520069.6862898
SRR17520069.6862900
SRR17520069.6862902
SRR17520069.6862904
SRR17520069.6862906
SRR17520069.6862908
SRR17520069.6862910
SRR17520069.6862912
SRR17520069.6862914
SRR17520069.6862916
SRR17520069.6862918
SRR17520069.6862920
SRR17520069.6862922
SRR17520069.6862924
SRR17520069.6862926
SRR17520069.6862928
SRR17520069.6862930
SRR17520069.6862932
SRR17520069.6862934
SRR17520069.6862936
SRR17520069.6862938
SRR17520069.6862940
SRR17520069.6862942
SRR17520069.6862944
SRR17520069.6862946
SRR17520069.6862948
SRR17520069.6862950
SRR17520069.6862952
SRR17520069.6862954
SRR17520069.6863157
SRR17520069.6867034
SRR17520069.6867036
SRR17520069.6867038
SRR17520069.6867040
SRR17520069.6867042
SRR17520069.6867044
SRR17520069.6867046
SRR17520069.6867048
SRR17520069.6867050
SRR17520069.6867052
SRR17520069.6867054
SRR17520069.6867056

SRR17520069.6867058
SRR17520069.6867060
SRR17520069.6867062
SRR17520069.6867064
SRR17520069.6867066
SRR17520069.6867068
SRR17520069.6867070
SRR17520069.6867072
SRR17520069.6867074
SRR17520069.6867076
SRR17520069.6867078
SRR17520069.6876332
SRR17520069.6880147
SRR17520069.6880149
SRR17520069.6880151
SRR17520069.6880153
SRR17520069.6903062
SRR17520069.6903064
SRR17520069.6903066
SRR17520069.6903068
SRR17520069.6903072
SRR17520069.6903074
SRR17520069.6903076
SRR17520069.6903078
SRR17520069.6903080
SRR17520069.6903082
SRR17520069.6903594
SRR17520069.6903596
SRR17520069.6903598
SRR17520069.6903600
SRR17520069.6903602
SRR17520069.6903604
SRR17520069.6903606
SRR17520069.6903608
SRR17520069.6903610
SRR17520069.6903614
SRR17520069.6903616
SRR17520069.6903622
SRR17520069.6903626
SRR17520069.6905196
SRR17520069.6905198
SRR17520069.6905200
SRR17520069.6905202
SRR17520069.6905204
SRR17520069.6905206
SRR17520069.6905208
SRR17520069.6905210

SRR17520069.6905212
SRR17520069.6905214
SRR17520069.6905216
SRR17520069.6905218
SRR17520069.6905220
SRR17520069.6905222
SRR17520069.6905224
SRR17520069.6905226
SRR17520069.6905228
SRR17520069.6905230
SRR17520069.6907160
SRR17520069.6907162
SRR17520069.6907164
SRR17520069.6907166
SRR17520069.6907168
SRR17520069.6907170
SRR17520069.6907172
SRR17520069.6907174
SRR17520069.6907176
SRR17520069.6907180
SRR17520069.6907182
SRR17520069.6907184
SRR17520069.6907186
SRR17520069.6907188
SRR17520069.6907190
SRR17520069.6907192
SRR17520069.6907194
SRR17520069.6907196
SRR17520069.6907198
SRR17520069.6907200
SRR17520069.6911690
SRR17520069.6911692
SRR17520069.6911696
SRR17520069.6911698
SRR17520069.6911702
SRR17520069.6911704
SRR17520069.6911706
SRR17520069.6911708
SRR17520069.6911710
SRR17520069.6911712
SRR17520069.6911714
SRR17520069.6911716
SRR17520069.6911718
SRR17520069.6911720
SRR17520069.6911722
SRR17520069.6911724
SRR17520069.6911726

SRR17520069.6911728
SRR17520069.6911730
SRR17520069.6911732
SRR17520069.6911734
SRR17520069.6911738
SRR17520069.6911740
SRR17520069.6911744
SRR17520069.6911746
SRR17520069.6911748
SRR17520069.6911750
SRR17520069.6911752
SRR17520069.6911754
SRR17520069.6911756
SRR17520069.6911758
SRR17520069.6911760
SRR17520069.6921186
SRR17520069.6921188
SRR17520069.6921190
SRR17520069.6925396
SRR17520069.6927888
SRR17520069.6927890
SRR17520069.6927892
SRR17520069.6927894
SRR17520069.6927896
SRR17520069.6927898
SRR17520069.6927900
SRR17520069.6927902
SRR17520069.6927904
SRR17520069.6927906
SRR17520069.6927908
SRR17520069.6931694
SRR17520069.6931696
SRR17520069.6931698
SRR17520069.6931700
SRR17520069.6931702
SRR17520069.6931704
SRR17520069.6931706
SRR17520069.6931708
SRR17520069.6931710
SRR17520069.6931712
SRR17520069.6931714
SRR17520069.6931716
SRR17520069.6931718
SRR17520069.6931720
SRR17520069.6931722
SRR17520069.6931724
SRR17520069.6931726

SRR17520069.6931728
SRR17520069.6931730
SRR17520069.6931732
SRR17520069.6931734
SRR17520069.6931736
SRR17520069.6931738
SRR17520069.6933570
SRR17520069.6933572
SRR17520069.6933574
SRR17520069.6933576
SRR17520069.6933578
SRR17520069.6933580
SRR17520069.6933582
SRR17520069.6933584
SRR17520069.6933586
SRR17520069.6933588
SRR17520069.6933590
SRR17520069.6933592
SRR17520069.6935422
SRR17520069.6935424
SRR17520069.6935426
SRR17520069.6936107
SRR17520069.6936109
SRR17520069.6936111
SRR17520069.6936113
SRR17520069.6936115
SRR17520069.6936117
SRR17520069.6936119
SRR17520069.6936121
SRR17520069.6936123
SRR17520069.6936125
SRR17520069.6936127
SRR17520069.6936129
SRR17520069.6936131
SRR17520069.6936133
SRR17520069.6936135
SRR17520069.6936137
SRR17520069.6936139
SRR17520069.6936143
SRR17520069.6936145
SRR17520069.6936147
SRR17520069.6936149
SRR17520069.6936151
SRR17520069.6936153
SRR17520069.6936155
SRR17520069.6936157
SRR17520069.6936159

SRR17520069.6936163
SRR17520069.6936165
SRR17520069.6936167
SRR17520069.6936169
SRR17520069.6936171
SRR17520069.6936173
SRR17520069.6936175
SRR17520069.6936177
SRR17520069.6936179
SRR17520069.6936181
SRR17520069.6936183
SRR17520069.6936185
SRR17520069.6936187
SRR17520069.6936189
SRR17520069.6936587
SRR17520069.6936589
SRR17520069.6936591
SRR17520069.6936593
SRR17520069.6936595
SRR17520069.6936597
SRR17520069.6936599
SRR17520069.6936601
SRR17520069.6936603
SRR17520069.6936605
SRR17520069.6936610
SRR17520069.6936612
SRR17520069.6936614
SRR17520069.6936616
SRR17520069.6936618
SRR17520069.6936620
SRR17520069.6936622
SRR17520069.6936624
SRR17520069.6936628
SRR17520069.6936630
SRR17520069.6936632
SRR17520069.6936634
SRR17520069.6936636
SRR17520069.6936638
SRR17520069.6936640
SRR17520069.6936644
SRR17520069.6936646
SRR17520069.6936648
SRR17520069.6936650
SRR17520069.6936654
SRR17520069.6936656
SRR17520069.6936658
SRR17520069.6936662

SRR17520069.6936664
SRR17520069.6943937
SRR17520069.6943939
SRR17520069.6943941
SRR17520069.6943943
SRR17520069.6943945
SRR17520069.6943947
SRR17520069.6943949
SRR17520069.6943951
SRR17520069.6943953
SRR17520069.6943955
SRR17520069.6943957
SRR17520069.6943959
SRR17520069.6943961
SRR17520069.6943963
SRR17520069.6943965
SRR17520069.6943967
SRR17520069.6943969
SRR17520069.6943971
SRR17520069.6943973
SRR17520069.6943975
SRR17520069.6943977
SRR17520069.6943979
SRR17520069.6943981
SRR17520069.6943983
SRR17520069.6943985
SRR17520069.6943987
SRR17520069.6943989
SRR17520069.6943991
SRR17520069.6943993
SRR17520069.6943997
SRR17520069.6943999
SRR17520069.6944001
SRR17520069.6944003
SRR17520069.6944005
SRR17520069.6944007
SRR17520069.6944011
SRR17520069.6944013
SRR17520069.6944015
SRR17520069.6944017
SRR17520069.6944019
SRR17520069.6944021
SRR17520069.6944023
SRR17520069.6944024
SRR17520069.6944025
SRR17520069.6944027
SRR17520069.6944029

SRR17520069.6944031
SRR17520069.6944712
SRR17520069.6944714
SRR17520069.6944716
SRR17520069.6944718
SRR17520069.6947613
SRR17520069.6947617
SRR17520069.6947619
SRR17520069.6947621
SRR17520069.6947623
SRR17520069.6947625
SRR17520069.6947627
SRR17520069.6947629
SRR17520069.6947631
SRR17520069.6947633
SRR17520069.6947635
SRR17520069.6947637
SRR17520069.6947639
SRR17520069.6947641
SRR17520069.6947643
SRR17520069.6947645
SRR17520069.6947647
SRR17520069.6947649
SRR17520069.6947651
SRR17520069.6947653
SRR17520069.6947655
SRR17520069.6947657
SRR17520069.6947661
SRR17520069.6947663
SRR17520069.6947665
SRR17520069.6947667
SRR17520069.6947669
SRR17520069.6947671
SRR17520069.6947673
SRR17520069.6947675
SRR17520069.6947677
SRR17520069.6947679
SRR17520069.6947681
SRR17520069.6947687
SRR17520069.6947691
SRR17520069.6953145
SRR17520069.6953147
SRR17520069.6953149
SRR17520069.6953151
SRR17520069.6953153
SRR17520069.6953155
SRR17520069.6953157

SRR17520069.6953163
SRR17520069.6953165
SRR17520069.6953167
SRR17520069.6953171
SRR17520069.6953177
SRR17520069.6953179
SRR17520069.6953187
SRR17520069.6964098
SRR17520069.6964100
SRR17520069.6964102
SRR17520069.6964351
SRR17520069.6964353
SRR17520069.6965143
SRR17520069.6965145
SRR17520069.6965149
SRR17520069.6965151
SRR17520069.6965153
SRR17520069.6965155
SRR17520069.6965161
SRR17520069.6965163
SRR17520069.6965165
SRR17520069.6965167
SRR17520069.6965169
SRR17520069.6965171
SRR17520069.6965173
SRR17520069.6965175
SRR17520069.6965177
SRR17520069.6965179
SRR17520069.6965181
SRR17520069.6965183
SRR17520069.6965185
SRR17520069.6965187
SRR17520069.6965189
SRR17520069.6965191
SRR17520069.6965193
SRR17520069.6965197
SRR17520069.6965199
SRR17520069.6965203
SRR17520069.6965205
SRR17520069.6965207
SRR17520069.6965209
SRR17520069.6967624
SRR17520069.6967626
SRR17520069.6967628
SRR17520069.6967630
SRR17520069.6967632
SRR17520069.6967634

SRR17520069.6974714
SRR17520069.6980805
SRR17520069.6980807
SRR17520069.6980809
SRR17520069.6980811
SRR17520069.6980813
SRR17520069.6981925
SRR17520069.6984248
SRR17520069.6984252
SRR17520069.6984254
SRR17520069.6984256
SRR17520069.6984258
SRR17520069.6984264
SRR17520069.6984268
SRR17520069.6984270
SRR17520069.6984272
SRR17520069.6984274
SRR17520069.6984276
SRR17520069.6984280
SRR17520069.6984284
SRR17520069.6984288
SRR17520069.6984290
SRR17520069.6984292
SRR17520069.6984294
SRR17520069.6984296
SRR17520069.6984774
SRR17520069.6984788
SRR17520069.6984790
SRR17520069.6984792
SRR17520069.6990774
SRR17520069.6990776
SRR17520069.6990778
SRR17520069.6990780
SRR17520069.6990782
SRR17520069.6990784
SRR17520069.6990786
SRR17520069.6990788
SRR17520069.6990790
SRR17520069.6990792
SRR17520069.6990794
SRR17520069.6990796
SRR17520069.6990798
SRR17520069.6990800
SRR17520069.6990802
SRR17520069.6990804
SRR17520069.6990806
SRR17520069.6990810

SRR17520069.6990812
SRR17520069.6990814
SRR17520069.6990816
SRR17520069.6990818
SRR17520069.6990820
SRR17520069.6990822
SRR17520069.6990824
SRR17520069.6990826
SRR17520069.6990828
SRR17520069.6990830
SRR17520069.6990832
SRR17520069.6990834
SRR17520069.6990836
SRR17520069.6990838
SRR17520069.6990840
SRR17520069.6990842
SRR17520069.6990844
SRR17520069.6990846
SRR17520069.6990848
SRR17520069.6990850
SRR17520069.6990852
SRR17520069.6990854
SRR17520069.6990856
SRR17520069.6990858
SRR17520069.6990860
SRR17520069.6990862
SRR17520069.6990864
SRR17520069.6990866
SRR17520069.6990868
SRR17520069.6990870
SRR17520069.6990872
SRR17520069.6990874
SRR17520069.6990876
SRR17520069.6990878
SRR17520069.6990880
SRR17520069.6990882
SRR17520069.6990884
SRR17520069.6990886
SRR17520069.6990888
SRR17520069.6991883
SRR17520069.6991885
SRR17520069.6991887
SRR17520069.6991889
SRR17520069.6991891
SRR17520069.6991893
SRR17520069.6991895
SRR17520069.6991897

SRR17520069.6991899
SRR17520069.6991901
SRR17520069.6991903
SRR17520069.6991905
SRR17520069.6991907
SRR17520069.6991909
SRR17520069.6991911
SRR17520069.6991913
SRR17520069.6991915
SRR17520069.6992700
SRR17520069.6992702
SRR17520069.6992704
SRR17520069.6992706
SRR17520069.6992708
SRR17520069.6995111
SRR17520069.6995965
SRR17520069.6995967
SRR17520069.6995969
SRR17520069.6995971
SRR17520069.6995973
SRR17520069.6995975
SRR17520069.6995977
SRR17520069.6995979
SRR17520069.6995981
SRR17520069.6995983
SRR17520069.6995985
SRR17520069.6995987
SRR17520069.6995989
SRR17520069.6995991
SRR17520069.6995993
SRR17520069.6995995
SRR17520069.6995997
SRR17520069.6995999
SRR17520069.6996001
SRR17520069.6996003
SRR17520069.6996005
SRR17520069.6996007
SRR17520069.6996009
SRR17520069.6996011
SRR17520069.6996013
SRR17520069.6996015
SRR17520069.7000493
SRR17520069.7000495
SRR17520069.7000497
SRR17520069.7000499
SRR17520069.7000501
SRR17520069.7009864

SRR17520069.7012710
SRR17520069.7013859
SRR17520069.7013861
SRR17520069.7013863
SRR17520069.7013865
SRR17520069.7013867
SRR17520069.7013869
SRR17520069.7013871
SRR17520069.7013873
SRR17520069.7013875
SRR17520069.7013877
SRR17520069.7013879
SRR17520069.7013881
SRR17520069.7013883
SRR17520069.7013885
SRR17520069.7013887
SRR17520069.7013889
SRR17520069.7013891
SRR17520069.7016066
SRR17520069.7016068
SRR17520069.7016070
SRR17520069.7016072
SRR17520069.7016074
SRR17520069.7016078
SRR17520069.7016080
SRR17520069.7016082
SRR17520069.7016084
SRR17520069.7016086
SRR17520069.7016781
SRR17520069.7016783
SRR17520069.7020950
SRR17520069.7020952
SRR17520069.7020954
SRR17520069.7020956
SRR17520069.7020958
SRR17520069.7020960
SRR17520069.7020962
SRR17520069.7020964
SRR17520069.7020966
SRR17520069.7020968
SRR17520069.7020970
SRR17520069.7020972
SRR17520069.7020974
SRR17520069.7020976
SRR17520069.7020978
SRR17520069.7020980
SRR17520069.7020982

SRR17520069.7020984
SRR17520069.7020986
SRR17520069.7020988
SRR17520069.7020990
SRR17520069.7020992
SRR17520069.7020994
SRR17520069.7020996
SRR17520069.7020998
SRR17520069.7021000
SRR17520069.7021002
SRR17520069.7021004
SRR17520069.7021006
SRR17520069.7021008
SRR17520069.7021010
SRR17520069.7021012
SRR17520069.7021014
SRR17520069.7021016
SRR17520069.7021018
SRR17520069.7021020
SRR17520069.7021022
SRR17520069.7021024
SRR17520069.7021444
SRR17520069.7021446
SRR17520069.7021448
SRR17520069.7021452
SRR17520069.7021454
SRR17520069.7021456
SRR17520069.7021458
SRR17520069.7021460
SRR17520069.7021462
SRR17520069.7021464
SRR17520069.7021466
SRR17520069.7021468
SRR17520069.7021470
SRR17520069.7021472
SRR17520069.7021474
SRR17520069.7021476
SRR17520069.7021478
SRR17520069.7021480
SRR17520069.7021482
SRR17520069.7021484
SRR17520069.7021486
SRR17520069.7021488
SRR17520069.7021490
SRR17520069.7033394
SRR17520069.7033396
SRR17520069.7033398

SRR17520069.7033400
SRR17520069.7033402
SRR17520069.7033404
SRR17520069.7033406
SRR17520069.7033408
SRR17520069.7033410
SRR17520069.7033412
SRR17520069.7033414
SRR17520069.7033416
SRR17520069.7033418
SRR17520069.7033420
SRR17520069.7040885
SRR17520069.7040887
SRR17520069.7040889
SRR17520069.7040891
SRR17520069.7042213
SRR17520069.7042215
SRR17520069.7042217
SRR17520069.7042219
SRR17520069.7042221
SRR17520069.7042223
SRR17520069.7050686
SRR17520069.7050688
SRR17520069.7052885
SRR17520069.7052887
SRR17520069.7052889
SRR17520069.7052891
SRR17520069.7052893
SRR17520069.7052895
SRR17520069.7052897
SRR17520069.7052899
SRR17520069.7052901
SRR17520069.7052903
SRR17520069.7052905
SRR17520069.7052907
SRR17520069.7052909
SRR17520069.7052911
SRR17520069.7052915
SRR17520069.7052917
SRR17520069.7052919
SRR17520069.7052921
SRR17520069.7052925
SRR17520069.7052927
SRR17520069.7052937
SRR17520069.7062497
SRR17520069.7062501
SRR17520069.7062503

SRR17520069.7062507
SRR17520069.7062511
SRR17520069.7062513
SRR17520069.7062515
SRR17520069.7062517
SRR17520069.7062519
SRR17520069.7062521
SRR17520069.7062523
SRR17520069.7062525
SRR17520069.7062527
SRR17520069.7062529
SRR17520069.7062531
SRR17520069.7062533
SRR17520069.7062535
SRR17520069.7062537
SRR17520069.7062539
SRR17520069.7062541
SRR17520069.7062543
SRR17520069.7062545
SRR17520069.7062547
SRR17520069.7062549
SRR17520069.7062551
SRR17520069.7062553
SRR17520069.7062555
SRR17520069.7062557
SRR17520069.7062559
SRR17520069.7062561
SRR17520069.7062563
SRR17520069.7062565
SRR17520069.7062567
SRR17520069.7062569
SRR17520069.7062571
SRR17520069.7062573
SRR17520069.7062575
SRR17520069.7062577
SRR17520069.7062579
SRR17520069.7062581
SRR17520069.7062583
SRR17520069.7062585
SRR17520069.7062587
SRR17520069.7062589
SRR17520069.7062591
SRR17520069.7062593
SRR17520069.7062595
SRR17520069.7062597
SRR17520069.7062599
SRR17520069.7062601

SRR17520069.7062603
SRR17520069.7063738
SRR17520069.7063740
SRR17520069.7063742
SRR17520069.7063746
SRR17520069.7063748
SRR17520069.7063750
SRR17520069.7063752
SRR17520069.7063754
SRR17520069.7063756
SRR17520069.7063758
SRR17520069.7063760
SRR17520069.7063762
SRR17520069.7063764
SRR17520069.7063766
SRR17520069.7063768
SRR17520069.7063770
SRR17520069.7063772
SRR17520069.7063774
SRR17520069.7063776
SRR17520069.7063778
SRR17520069.7063780
SRR17520069.7063782
SRR17520069.7063784
SRR17520069.7063786
SRR17520069.7063788
SRR17520069.7063790
SRR17520069.7063792
SRR17520069.7063794
SRR17520069.7063796
SRR17520069.7064116
SRR17520069.7064118
SRR17520069.7064120
SRR17520069.7064122
SRR17520069.7064124
SRR17520069.7064126
SRR17520069.7064128
SRR17520069.7064130
SRR17520069.7064132
SRR17520069.7064134
SRR17520069.7064136
SRR17520069.7064138
SRR17520069.7064140
SRR17520069.7064142
SRR17520069.7064144
SRR17520069.7064146
SRR17520069.7064148

SRR17520069.7064150
SRR17520069.7064152
SRR17520069.7064154
SRR17520069.7064156
SRR17520069.7064158
SRR17520069.7064160
SRR17520069.7064164
SRR17520069.7064166
SRR17520069.7064168
SRR17520069.7064170
SRR17520069.7064172
SRR17520069.7064174
SRR17520069.7064176
SRR17520069.7064178
SRR17520069.7064180
SRR17520069.7073382
SRR17520069.7073384
SRR17520069.7074365
SRR17520069.7074367
SRR17520069.7074369
SRR17520069.7074371
SRR17520069.7074373
SRR17520069.7080584
SRR17520069.7080586
SRR17520069.7080588
SRR17520069.7080590
SRR17520069.7080592
SRR17520069.7080594
SRR17520069.7080596
SRR17520069.7080598
SRR17520069.7082056
SRR17520069.7084500
SRR17520069.7084502
SRR17520069.7084504
SRR17520069.7089414
SRR17520069.7101941
SRR17520069.7101943
SRR17520069.7101945
SRR17520069.7101947
SRR17520069.7101949
SRR17520069.7101951
SRR17520069.7101953
SRR17520069.7101955
SRR17520069.7101957
SRR17520069.7101959
SRR17520069.7101961
SRR17520069.7101963

SRR17520069.7101965
SRR17520069.7102346
SRR17520069.7102348
SRR17520069.7102350
SRR17520069.7102352
SRR17520069.7102354
SRR17520069.7102356
SRR17520069.7102358
SRR17520069.7102360
SRR17520069.7102362
SRR17520069.7102364
SRR17520069.7102366
SRR17520069.7102368
SRR17520069.7102370
SRR17520069.7102372
SRR17520069.7102374
SRR17520069.7103569
SRR17520069.7103571
SRR17520069.7107263
SRR17520069.7120361
SRR17520069.7120363
SRR17520069.7120365
SRR17520069.7120367
SRR17520069.7120369
SRR17520069.7120371
SRR17520069.7120373
SRR17520069.7120375
SRR17520069.7120377
SRR17520069.7134333
SRR17520069.7134335
SRR17520069.7134872
SRR17520069.7134875
SRR17520069.7146874
SRR17520069.7146876
SRR17520069.7146878
SRR17520069.7146882
SRR17520069.7146884
SRR17520069.7146886
SRR17520069.7146888
SRR17520069.7149141
SRR17520069.7149145
SRR17520069.7149147
SRR17520069.7149151
SRR17520069.7149153
SRR17520069.7149159
SRR17520069.7149161
SRR17520069.7149163

SRR17520069.7149165
SRR17520069.7149167
SRR17520069.7149169
SRR17520069.7149171
SRR17520069.7149173
SRR17520069.7149175
SRR17520069.7149177
SRR17520069.7149179
SRR17520069.7149181
SRR17520069.7149183
SRR17520069.7149185
SRR17520069.7149187
SRR17520069.7149189
SRR17520069.7149191
SRR17520069.7149193
SRR17520069.7149195
SRR17520069.7155331
SRR17520069.7155333
SRR17520069.7155335
SRR17520069.7165723
SRR17520069.7165725
SRR17520069.7165727
SRR17520069.7165729
SRR17520069.7165731
SRR17520069.7165733
SRR17520069.7165735
SRR17520069.7165737
SRR17520069.7165739
SRR17520069.7165741
SRR17520069.7165745
SRR17520069.7165747
SRR17520069.7165749
SRR17520069.7165751
SRR17520069.7165753
SRR17520069.7165755
SRR17520069.7165757
SRR17520069.7165759
SRR17520069.7165761
SRR17520069.7165763
SRR17520069.7165765
SRR17520069.7165767
SRR17520069.7165769
SRR17520069.7165771
SRR17520069.7165773
SRR17520069.7165775
SRR17520069.7165777
SRR17520069.7168383

SRR17520069.7168385
SRR17520069.7168387
SRR17520069.7168389
SRR17520069.7168391
SRR17520069.7168393
SRR17520069.7168395
SRR17520069.7169075
SRR17520069.7173584
SRR17520069.7173586
SRR17520069.7173588
SRR17520069.7173590
SRR17520069.7173592
SRR17520069.7173594
SRR17520069.7173596
SRR17520069.7173598
SRR17520069.7173600
SRR17520069.7173602
SRR17520069.7173604
SRR17520069.7173606
SRR17520069.7173608
SRR17520069.7173610
SRR17520069.7178850
SRR17520069.7178852
SRR17520069.7178854
SRR17520069.7178856
SRR17520069.7178858
SRR17520069.7178860
SRR17520069.7178862
SRR17520069.7178864
SRR17520069.7178866
SRR17520069.7178868
SRR17520069.7178870
SRR17520069.7178872
SRR17520069.7178874
SRR17520069.7178876
SRR17520069.7178878
SRR17520069.7178880
SRR17520069.7178882
SRR17520069.7178884
SRR17520069.7178886
SRR17520069.7178888
SRR17520069.7178890
SRR17520069.7178892
SRR17520069.7178894
SRR17520069.7178896
SRR17520069.7178898
SRR17520069.7178900

SRR17520069.7178902
SRR17520069.7178904
SRR17520069.7178906
SRR17520069.7178908
SRR17520069.7178910
SRR17520069.7178912
SRR17520069.7178914
SRR17520069.7178916
SRR17520069.7178918
SRR17520069.7178920
SRR17520069.7178922
SRR17520069.7178924
SRR17520069.7180780
SRR17520069.7180782
SRR17520069.7180784
SRR17520069.7180786
SRR17520069.7180788
SRR17520069.7180790
SRR17520069.7180792
SRR17520069.7180794
SRR17520069.7180796
SRR17520069.7180798
SRR17520069.7180800
SRR17520069.7180802
SRR17520069.7192070
SRR17520069.7192072
SRR17520069.7192074
SRR17520069.7192076
SRR17520069.7192078
SRR17520069.7192082
SRR17520069.7192086
SRR17520069.7192090
SRR17520069.7192094
SRR17520069.7192096
SRR17520069.7192098
SRR17520069.7192102
SRR17520069.7192104
SRR17520069.7192108
SRR17520069.7192110
SRR17520069.7192112
SRR17520069.7192114
SRR17520069.7192116
SRR17520069.7192118
SRR17520069.7192120
SRR17520069.7192122
SRR17520069.7197557
SRR17520069.7197559

SRR17520069.7197563
SRR17520069.7197565
SRR17520069.7197567
SRR17520069.7198595
SRR17520069.7202364
SRR17520069.7202366
SRR17520069.7202368
SRR17520069.7202370
SRR17520069.7202372
SRR17520069.7202374
SRR17520069.7202376
SRR17520069.7202378
SRR17520069.7202380
SRR17520069.7202382
SRR17520069.7202384
SRR17520069.7202386
SRR17520069.7202388
SRR17520069.7202390
SRR17520069.7202392
SRR17520069.7202394
SRR17520069.7202396
SRR17520069.7202398
SRR17520069.7202400
SRR17520069.7202402
SRR17520069.7202404
SRR17520069.7202406
SRR17520069.7205301
SRR17520069.7205303
SRR17520069.7205305
SRR17520069.7205307
SRR17520069.7205309
SRR17520069.7205311
SRR17520069.7205313
SRR17520069.7205315
SRR17520069.7205317
SRR17520069.7205319
SRR17520069.7205321
SRR17520069.7205323
SRR17520069.7205325
SRR17520069.7205327
SRR17520069.7205329
SRR17520069.7205331
SRR17520069.7205333
SRR17520069.7205335
SRR17520069.7205337
SRR17520069.7205339
SRR17520069.7206915

SRR17520069.7206917
SRR17520069.7211634
SRR17520069.7211636
SRR17520069.7211638
SRR17520069.7211640
SRR17520069.7211642
SRR17520069.7211644
SRR17520069.7211646
SRR17520069.7211648
SRR17520069.7211650
SRR17520069.7211652
SRR17520069.7211654
SRR17520069.7211656
SRR17520069.7211658
SRR17520069.7211660
SRR17520069.7211662
SRR17520069.7211664
SRR17520069.7211666
SRR17520069.7211668
SRR17520069.7211670
SRR17520069.7211672
SRR17520069.7211674
SRR17520069.7211676
SRR17520069.7215788
SRR17520069.7215790
SRR17520069.7215792
SRR17520069.7215794
SRR17520069.7215796
SRR17520069.7216881
SRR17520069.7216883
SRR17520069.7216885
SRR17520069.7216887
SRR17520069.7216889
SRR17520069.7216893
SRR17520069.7216895
SRR17520069.7220385
SRR17520069.7220387
SRR17520069.7220389
SRR17520069.7220391
SRR17520069.7220393
SRR17520069.7220395
SRR17520069.7220397
SRR17520069.7220399
SRR17520069.7220401
SRR17520069.7220403
SRR17520069.7220405
SRR17520069.7220407

SRR17520069.7220409
SRR17520069.7220411
SRR17520069.7220413
SRR17520069.7220415
SRR17520069.7220417
SRR17520069.7220419
SRR17520069.7220421
SRR17520069.7220423
SRR17520069.7220425
SRR17520069.7220427
SRR17520069.7220429
SRR17520069.7220431
SRR17520069.7220433
SRR17520069.7220435
SRR17520069.7220437
SRR17520069.7220439
SRR17520069.7220441
SRR17520069.7220443
SRR17520069.7220445
SRR17520069.7220447
SRR17520069.7220449
SRR17520069.7220451
SRR17520069.7220453
SRR17520069.7220457
SRR17520069.7220459
SRR17520069.7220461
SRR17520069.7220463
SRR17520069.7220465
SRR17520069.7220467
SRR17520069.7220715
SRR17520069.7220717
SRR17520069.7220719
SRR17520069.7220721
SRR17520069.7220723
SRR17520069.7220725
SRR17520069.7221187
SRR17520069.7221191
SRR17520069.7221193
SRR17520069.7221195
SRR17520069.7221197
SRR17520069.7221199
SRR17520069.7221201
SRR17520069.7221203
SRR17520069.7221205
SRR17520069.7221207
SRR17520069.7221209
SRR17520069.7221211

SRR17520069.7221213
SRR17520069.7221215
SRR17520069.7221217
SRR17520069.7221219
SRR17520069.7221221
SRR17520069.7228017
SRR17520069.7228987
SRR17520069.7228989
SRR17520069.7228993
SRR17520069.7228995
SRR17520069.7228997
SRR17520069.7228999
SRR17520069.7229001
SRR17520069.7229005
SRR17520069.7229007
SRR17520069.7229009
SRR17520069.7229013
SRR17520069.7231850
SRR17520069.7231852
SRR17520069.7231858
SRR17520069.7231860
SRR17520069.7231862
SRR17520069.7231864
SRR17520069.7231868
SRR17520069.7231870
SRR17520069.7231872
SRR17520069.7231874
SRR17520069.7231876
SRR17520069.7231878
SRR17520069.7231880
SRR17520069.7231882
SRR17520069.7231884
SRR17520069.7231886
SRR17520069.7231888
SRR17520069.7231890
SRR17520069.7231894
SRR17520069.7231896
SRR17520069.7231898
SRR17520069.7231900
SRR17520069.7231902
SRR17520069.7231904
SRR17520069.7231908
SRR17520069.7231912
SRR17520069.7231914
SRR17520069.7231916
SRR17520069.7231918
SRR17520069.7231920

SRR17520069.7231924
SRR17520069.7231926
SRR17520069.7231928
SRR17520069.7231930
SRR17520069.7231932
SRR17520069.7231934
SRR17520069.7231936
SRR17520069.7231938
SRR17520069.7231940
SRR17520069.7231942
SRR17520069.7231944
SRR17520069.7231946
SRR17520069.7231948
SRR17520069.7231950
SRR17520069.7231952
SRR17520069.7231954
SRR17520069.7231956
SRR17520069.7231958
SRR17520069.7231960
SRR17520069.7231962
SRR17520069.7231964
SRR17520069.7231966
SRR17520069.7231968
SRR17520069.7231970
SRR17520069.7231972
SRR17520069.7231974
SRR17520069.7231976
SRR17520069.7231982
SRR17520069.7231984
SRR17520069.7231986
SRR17520069.7231990
SRR17520069.7231992
SRR17520069.7231994
SRR17520069.7231996
SRR17520069.7231998
SRR17520069.7232000
SRR17520069.7232002
SRR17520069.7232004
SRR17520069.7232006
SRR17520069.7232010
SRR17520069.7232012
SRR17520069.7232014
SRR17520069.7232016
SRR17520069.7232018
SRR17520069.7232020
SRR17520069.7232022
SRR17520069.7232024

SRR17520069.7232026
SRR17520069.7232028
SRR17520069.7232030
SRR17520069.7232032
SRR17520069.7232034
SRR17520069.7232036
SRR17520069.7232038
SRR17520069.7232040
SRR17520069.7232042
SRR17520069.7232044
SRR17520069.7232046
SRR17520069.7232048
SRR17520069.7232050
SRR17520069.7232052
SRR17520069.7232054
SRR17520069.7232056
SRR17520069.7232058
SRR17520069.7232060
SRR17520069.7232062
SRR17520069.7232064
SRR17520069.7236068
SRR17520069.7236070
SRR17520069.7236072
SRR17520069.7236074
SRR17520069.7236078
SRR17520069.7236080
SRR17520069.7236082
SRR17520069.7236084
SRR17520069.7236090
SRR17520069.7236092
SRR17520069.7236094
SRR17520069.7236096
SRR17520069.7236098
SRR17520069.7236100
SRR17520069.7236102
SRR17520069.7236104
SRR17520069.7236106
SRR17520069.7236108
SRR17520069.7236110
SRR17520069.7236112
SRR17520069.7236114
SRR17520069.7236116
SRR17520069.7236118
SRR17520069.7236120
SRR17520069.7236122
SRR17520069.7237133
SRR17520069.7237135

SRR17520069.7237137
SRR17520069.7237139
SRR17520069.7237141
SRR17520069.7237143
SRR17520069.7237145
SRR17520069.7237147
SRR17520069.7237149
SRR17520069.7239677
SRR17520069.7251141
SRR17520069.7251143
SRR17520069.7251145
SRR17520069.7251147
SRR17520069.7251149
SRR17520069.7251151
SRR17520069.7251153
SRR17520069.7251467
SRR17520069.7251469
SRR17520069.7251471
SRR17520069.7251473
SRR17520069.7251475
SRR17520069.7251477
SRR17520069.7251479
SRR17520069.7251481
SRR17520069.7251483
SRR17520069.7251485
SRR17520069.7251487
SRR17520069.7251489
SRR17520069.7251491
SRR17520069.7251493
SRR17520069.7251495
SRR17520069.7253734
SRR17520069.7253736
SRR17520069.7253738
SRR17520069.7253740
SRR17520069.7253742
SRR17520069.7253744
SRR17520069.7253746
SRR17520069.7253748
SRR17520069.7253750
SRR17520069.7253752
SRR17520069.7253754
SRR17520069.7253756
SRR17520069.7253758
SRR17520069.7253760
SRR17520069.7253762
SRR17520069.7253764
SRR17520069.7253766

SRR17520069.7253768
SRR17520069.7253770
SRR17520069.7253772
SRR17520069.7253774
SRR17520069.7253776
SRR17520069.7253778
SRR17520069.7253780
SRR17520069.7253782
SRR17520069.7253784
SRR17520069.7253786
SRR17520069.7253788
SRR17520069.7253790
SRR17520069.7253792
SRR17520069.7253794
SRR17520069.7253796
SRR17520069.7253798
SRR17520069.7253800
SRR17520069.7253802
SRR17520069.7253804
SRR17520069.7253806
SRR17520069.7253808
SRR17520069.7253810
SRR17520069.7253812
SRR17520069.7253814
SRR17520069.7253816
SRR17520069.7253818
SRR17520069.7253820
SRR17520069.7253822
SRR17520069.7254119
SRR17520069.7254123
SRR17520069.7254125
SRR17520069.7254127
SRR17520069.7254129
SRR17520069.7258258
SRR17520069.7258260
SRR17520069.7258262
SRR17520069.7258264
SRR17520069.7258266
SRR17520069.7258268
SRR17520069.7258270
SRR17520069.7258272
SRR17520069.7258274
SRR17520069.7258276
SRR17520069.7258278
SRR17520069.7258280
SRR17520069.7258282
SRR17520069.7258284

SRR17520069.7258286
SRR17520069.7258288
SRR17520069.7258290
SRR17520069.7258292
SRR17520069.7258294
SRR17520069.7258296
SRR17520069.7258298
SRR17520069.7258300
SRR17520069.7258302
SRR17520069.7258304
SRR17520069.7258306
SRR17520069.7258308
SRR17520069.7258312
SRR17520069.7258316
SRR17520069.7258318
SRR17520069.7258320
SRR17520069.7258322
SRR17520069.7258326
SRR17520069.7258330
SRR17520069.7258332
SRR17520069.7258336
SRR17520069.7258338
SRR17520069.7258342
SRR17520069.7261948
SRR17520069.7261950
SRR17520069.7267095
SRR17520069.7267097
SRR17520069.7267099
SRR17520069.7267101
SRR17520069.7270462
SRR17520069.7270464
SRR17520069.7270466
SRR17520069.7270468
SRR17520069.7270470
SRR17520069.7270472
SRR17520069.7270474
SRR17520069.7270476
SRR17520069.7270478
SRR17520069.7270480
SRR17520069.7270482
SRR17520069.7274567
SRR17520069.7274569
SRR17520069.7274571
SRR17520069.7274573
SRR17520069.7274575
SRR17520069.7274577
SRR17520069.7274579

SRR17520069.7274581
SRR17520069.7274583
SRR17520069.7274585
SRR17520069.7274587
SRR17520069.7274589
SRR17520069.7274591
SRR17520069.7274593
SRR17520069.7274595
SRR17520069.7274597
SRR17520069.7274599
SRR17520069.7274601
SRR17520069.7274603
SRR17520069.7274605
SRR17520069.7274607
SRR17520069.7274609
SRR17520069.7274611
SRR17520069.7284356
SRR17520069.7284358
SRR17520069.7284360
SRR17520069.7284362
SRR17520069.7284364
SRR17520069.7284368
SRR17520069.7284370
SRR17520069.7284372
SRR17520069.7284374
SRR17520069.7284376
SRR17520069.7284378
SRR17520069.7284380
SRR17520069.7284382
SRR17520069.7284384
SRR17520069.7284386
SRR17520069.7284388
SRR17520069.7284390
SRR17520069.7284392
SRR17520069.7284394
SRR17520069.7284396
SRR17520069.7284398
SRR17520069.7284400
SRR17520069.7284402
SRR17520069.7284404
SRR17520069.7284406
SRR17520069.7284408
SRR17520069.7284410
SRR17520069.7284412
SRR17520069.7284414
SRR17520069.7284416
SRR17520069.7284418

SRR17520069.7284420
SRR17520069.7284422
SRR17520069.7284424
SRR17520069.7284426
SRR17520069.7284428
SRR17520069.7284430
SRR17520069.7284432
SRR17520069.7284434
SRR17520069.7284436
SRR17520069.7284438
SRR17520069.7284440
SRR17520069.7284442
SRR17520069.7284446
SRR17520069.7284448
SRR17520069.7284450
SRR17520069.7285559
SRR17520069.7285561
SRR17520069.7285563
SRR17520069.7285565
SRR17520069.7285567
SRR17520069.7285569
SRR17520069.7285571
SRR17520069.7285573
SRR17520069.7285575
SRR17520069.7285577
SRR17520069.7285579
SRR17520069.7285581
SRR17520069.7285583
SRR17520069.7285585
SRR17520069.7285589
SRR17520069.7285591
SRR17520069.7285593
SRR17520069.7285595
SRR17520069.7285597
SRR17520069.7285599
SRR17520069.7285601
SRR17520069.7285603
SRR17520069.7285605
SRR17520069.7285804
SRR17520069.7285806
SRR17520069.7285808
SRR17520069.7285810
SRR17520069.7285812
SRR17520069.7285814
SRR17520069.7285816
SRR17520069.7285818
SRR17520069.7299625

SRR17520069.7299627
SRR17520069.7299629
SRR17520069.7299633
SRR17520069.7299635
SRR17520069.7299637
SRR17520069.7299639
SRR17520069.7299641
SRR17520069.7299643
SRR17520069.7299645
SRR17520069.7299647
SRR17520069.7299649
SRR17520069.7299651
SRR17520069.7299653
SRR17520069.7299655
SRR17520069.7299657
SRR17520069.7299659
SRR17520069.7299661
SRR17520069.7299663
SRR17520069.7299665
SRR17520069.7299667
SRR17520069.7299669
SRR17520069.7299671
SRR17520069.7299673
SRR17520069.7299675
SRR17520069.7299677
SRR17520069.7299679
SRR17520069.7299681
SRR17520069.7299683
SRR17520069.7299685
SRR17520069.7299687
SRR17520069.7299689
SRR17520069.7299691
SRR17520069.7299693
SRR17520069.7299695
SRR17520069.7299697
SRR17520069.7299699
SRR17520069.7299701
SRR17520069.7299703
SRR17520069.7299705
SRR17520069.7300560
SRR17520069.7307302
SRR17520069.7308712
SRR17520069.7308714
SRR17520069.7311052
SRR17520069.7311054
SRR17520069.7311056
SRR17520069.7311058

SRR17520069.7311060
SRR17520069.7311062
SRR17520069.7311064
SRR17520069.7311066
SRR17520069.7311068
SRR17520069.7311070
SRR17520069.7311072
SRR17520069.7311074
SRR17520069.7311076
SRR17520069.7311078
SRR17520069.7311080
SRR17520069.7311082
SRR17520069.7311084
SRR17520069.7311086
SRR17520069.7311092
SRR17520069.7311094
SRR17520069.7312225
SRR17520069.7318818
SRR17520069.7318820
SRR17520069.7318824
SRR17520069.7318828
SRR17520069.7318830
SRR17520069.7318832
SRR17520069.7318834
SRR17520069.7318836
SRR17520069.7318838
SRR17520069.7318840
SRR17520069.7318842
SRR17520069.7318844
SRR17520069.7318846
SRR17520069.7318848
SRR17520069.7318850
SRR17520069.7318854
SRR17520069.7318856
SRR17520069.7318858
SRR17520069.7318860
SRR17520069.7318862
SRR17520069.7318864
SRR17520069.7318871
SRR17520069.7318873
SRR17520069.7318875
SRR17520069.7318877
SRR17520069.7318879
SRR17520069.7318881
SRR17520069.7318883
SRR17520069.7318885
SRR17520069.7318887

SRR17520069.7318889
SRR17520069.7318891
SRR17520069.7318893
SRR17520069.7318895
SRR17520069.7318897
SRR17520069.7318899
SRR17520069.7318901
SRR17520069.7318903
SRR17520069.7318905
SRR17520069.7318907
SRR17520069.7318909
SRR17520069.7325655
SRR17520069.7325657
SRR17520069.7325659
SRR17520069.7325661
SRR17520069.7325663
SRR17520069.7325665
SRR17520069.7325667
SRR17520069.7325669
SRR17520069.7325671
SRR17520069.7325673
SRR17520069.7325675
SRR17520069.7325677
SRR17520069.7325679
SRR17520069.7325681
SRR17520069.7325683
SRR17520069.7325685
SRR17520069.7325687
SRR17520069.7325689
SRR17520069.7325691
SRR17520069.7325693
SRR17520069.7325695
SRR17520069.7325697
SRR17520069.7325699
SRR17520069.7325701
SRR17520069.7325703
SRR17520069.7325705
SRR17520069.7325707
SRR17520069.7325709
SRR17520069.7325711
SRR17520069.7325713
SRR17520069.7325715
SRR17520069.7325717
SRR17520069.7325719
SRR17520069.7325721
SRR17520069.7325723
SRR17520069.7325725

SRR17520069.7325727
SRR17520069.7325729
SRR17520069.7325731
SRR17520069.7325733
SRR17520069.7325735
SRR17520069.7325737
SRR17520069.7325739
SRR17520069.7325741
SRR17520069.7325743
SRR17520069.7325745
SRR17520069.7325747
SRR17520069.7325749
SRR17520069.7325751
SRR17520069.7325753
SRR17520069.7325755
SRR17520069.7325757
SRR17520069.7325759
SRR17520069.7325761
SRR17520069.7325763
SRR17520069.7325765
SRR17520069.7325767
SRR17520069.7325769
SRR17520069.7325771
SRR17520069.7325773
SRR17520069.7325775
SRR17520069.7325777
SRR17520069.7325779
SRR17520069.7325781
SRR17520069.7325783
SRR17520069.7325785
SRR17520069.7325787
SRR17520069.7327694
SRR17520069.7327696
SRR17520069.7327698
SRR17520069.7327700
SRR17520069.7327702
SRR17520069.7327704
SRR17520069.7327706
SRR17520069.7327710
SRR17520069.7327712
SRR17520069.7327714
SRR17520069.7327716
SRR17520069.7327718
SRR17520069.7327720
SRR17520069.7327722
SRR17520069.7327724
SRR17520069.7327726

SRR17520069.7327730
SRR17520069.7327732
SRR17520069.7327734
SRR17520069.7327736
SRR17520069.7327738
SRR17520069.7327740
SRR17520069.7327742
SRR17520069.7327744
SRR17520069.7327746
SRR17520069.7327748
SRR17520069.7327750
SRR17520069.7327752
SRR17520069.7327754
SRR17520069.7327756
SRR17520069.7327758
SRR17520069.7327760
SRR17520069.7327762
SRR17520069.7327764
SRR17520069.7327766
SRR17520069.7327768
SRR17520069.7327770
SRR17520069.7327772
SRR17520069.7327791
SRR17520069.7327793
SRR17520069.7327799
SRR17520069.7327801
SRR17520069.7327805
SRR17520069.7327807
SRR17520069.7327809
SRR17520069.7327811
SRR17520069.7327813
SRR17520069.7327815
SRR17520069.7327817
SRR17520069.7327819
SRR17520069.7327821
SRR17520069.7327823
SRR17520069.7327825
SRR17520069.7327829
SRR17520069.7327831
SRR17520069.7327833
SRR17520069.7327835
SRR17520069.7327837
SRR17520069.7327839
SRR17520069.7327841
SRR17520069.7327843
SRR17520069.7327845
SRR17520069.7327847

SRR17520069.7327849
SRR17520069.7327853
SRR17520069.7327855
SRR17520069.7327857
SRR17520069.7327859
SRR17520069.7327861
SRR17520069.7327863
[truncated: 322,931 more chars]
